# Supplementary material for: Rise, and pronounced regional variation, in methylphenidate, amphetamine, and lisdexamfetamine distribution in the United States
Source: PeerJ. 2021 Dec 21;9:e12619. doi: 10.7717/peerj.12619 (PMC8706327; doi:10.7717/peerj.12619)
Supplement: Supplemental Information 1 [file peerj-09-12619-s001.pdf]

DRUG CODE: 1100 DRUG NAME: AMPHETAMINE

STATE: ALABAMA

ZIP CODE	QUARTER 1	QUARTER 2	QUARTER 3	QUARTER 4	TOTAL GRAMS
350	17,317.77	17,592.62	17,601.30	18,026.63	70,538.32
351	8,853.04	8,730.36	8,871.69	8,885.75	35,340.84
352	19,011.67	18,775.54	19,012	19,869.23	76,668.44
354	8,415.59	8,008.47	7,903.15	8,576.03	32,903.24
355	3,437.61	3,592.54	3,505.06	3,423.91	13,959.12
356	9,419.78	9,504.50	9,896.92	10,055.83	38,877.03
357	4,092.23	4,275.89	4,362.04	4,447.32	17,177.48
358	5,351.36	5,555.98	5,650.23	5,681.07	22,238.64
359	4,819.79	4,836.55	4,619.88	5,088.01	19,364.23
360	6,516.37	6,441.69	6,372.90	6,768.46	26,099.42
361	5,920.66	5,825.58	5,225.22	5,243.17	22,214.63
362	2,442.19	2,350.13	2,347.92	2,345.96	9,486.20
363	5,219.18	5,291.86	5,210.23	5,424.80	21,146.07
364	1,273.24	1,315.80	1,144.04	1,299.77	5,032.85
365	11,488.92	11,634.86	12,049.97	12,420.28	47,594.03
366	7,815.82	7,687.68	7,886.41	7,953.48	31,343.39
367	1,557.50	1,463.38	1,478.39	1,572.70	6,071.97
368	6,415.23	6,190.82	6,134.74	6,677.12	25,417.91
369	154.58	123.62	127.6	143.08	548.88
TOTAL	129,522.53	129,197.87	129,399.69	133,902.60	522,022.69

STATE: ALASKA

ZIP CODE	QUARTER 1	QUARTER 2	QUARTER 3	QUARTER 4	TOTAL GRAMS
995	4,873.14	4,889.14	4,867.94	5,002.53	19,632.75
996	1,456.63	1,348.71	1,465.13	1,383.28	5,653.75
997	1,142.20	1,151.50	1,215.34	1,125.86	4,634.90
998	380.28	454.81	459.8	425.87	1,720.76
999	165.38	191.37	181.93	154.42	693.1
TOTAL	8,017.63	8,035.53	8,190.14	8,091.96	32,335.26

STATE: AMERICAN SAMOA

ZIP CODE	QUARTER 1	QUARTER 2	QUARTER 3	QUARTER 4	TOTAL GRAMS

	967	1.25	1.25	0	0	2.5
TOTAL		1.25	1.25	0	0	2.5

STATE:	ARIZONA					
ZIP CODE		QUARTER 1	QUARTER 2	QUARTER 3	QUARTER 4	TOTAL GRAMS
	850	17,085.55	17,122.42	16,792.26	17,695.67	68,695.90
	851	2,949.78	3,116.14	3,116.29	3,382.39	12,564.60
	852	32,696.05	32,496.97	33,207.28	36,130.70	134,531
	853	11,110.41	10,528	10,559.23	10,991.41	43,189.05
	855	495.76	409.32	472.1	477.97	1,855.15
	856	1,667.39	1,636.98	1,527.61	1,696	6,527.98
	857	10,892.17	10,595.68	10,465.69	10,987.87	42,941.41
	859	529.34	520.01	497.23	472.8	2,019.38
	860	1,319.04	1,228.49	1,210.32	1,333.18	5,091.03
	863	1,569.48	1,587.04	1,464.17	1,718.05	6,338.74
	864	942.56	892.58	859.54	923.26	3,617.94
	865	54.23	51.09	27.5	64.94	197.76
TOTAL		81,311.76	80,184.72	80,199.22	85,874.24	327,569.94

STATE:	ARKANSAS					
ZIP CODE		QUARTER 1	QUARTER 2	QUARTER 3	QUARTER 4	TOTAL GRAMS
	716	2,788.22	2,809.06	2,886.64	2,919.10	11,403.02
	717	965.54	961.04	883.31	971.54	3,781.43
	718	959.19	832.46	896.19	951.83	3,639.67
	719	3,491.96	3,332.89	3,360.64	3,380.55	13,566.04
	720	7,605.38	7,648.33	7,746.66	8,031.25	31,031.62
	721	6,064.07	6,106.61	6,095.45	6,280.22	24,546.35
	722	7,089.99	7,013.36	7,301.47	7,287.35	28,692.17
	723	1,517.55	1,539.92	1,482.32	1,548.20	6,087.99
	724	2,812.94	2,734.62	2,673.57	2,854.60	11,075.73
	725	770.69	726.82	750.23	754.58	3,002.32
	726	1,540.53	1,469.13	1,554.03	1,537	6,100.69
	727	12,037.52	12,369.29	12,280.49	12,836.22	49,523.52
	728	986.65	986.5	934.46	944.66	3,852.27
	729	1,974.59	2,021.71	2,058.32	2,014.25	8,068.87

TOTAL	50,604.82	50,551.74	50,903.78	52,311.35	204,371.69

STATE: CALIFORNIA

ZIP CODE	QUARTER 1	QUARTER 2	QUARTER 3	QUARTER 4	TOTAL GRAMS
900	21,190.33	22,283.22	21,463.94	22,760.37	87,697.86
902	12,294.30	12,770.35	12,367.35	12,893.80	50,325.80
903	284.12	308.32	300.17	268.1	1,160.71
904	3,922.68	4,176.69	4,174.37	4,013.15	16,286.89
905	1,735.29	1,845.88	1,699.89	1,773.27	7,054.33
906	2,281.68	2,339.11	2,313.36	2,520.19	9,454.34
907	3,240.07	3,310.64	3,332.77	3,319.06	13,202.54
908	2,910.12	3,110.81	2,869.36	3,035.64	11,925.93
910	1,581.76	1,684.27	1,612.79	1,533.60	6,412.42
911	2,567.45	2,549.78	2,645.98	2,681.86	10,445.07
912	1,515.12	1,620.92	1,521.64	1,551.68	6,209.36
913	14,340.39	14,686.23	14,323.01	14,801.06	58,150.69
914	4,225.08	4,490.77	4,553.27	4,423.62	17,692.74
915	1,745.92	1,786.70	1,849.83	1,784.17	7,166.62
916	1,635.52	1,720.58	1,679.19	1,750.31	6,785.60
917	6,716.13	6,933.52	6,657.49	6,996.58	27,303.72
918	370.21	402.93	394.83	377.61	1,545.58
919	2,659.25	2,863.81	2,620.39	2,672.26	10,815.71
920	26,382.62	26,689.43	26,373.24	27,140.41	106,585.70
921	17,531.85	17,972.54	17,362.58	18,097.34	70,964.31
922	3,662.11	3,531.42	3,351.59	3,520.91	14,066.03
923	4,086.95	4,025.14	4,055.80	4,255.37	16,423.26
924	314.71	363.32	311.13	345.45	1,334.61
925	6,303.63	6,478.64	6,260.11	6,536.16	25,578.54
926	21,129.93	21,430.31	21,464.66	21,524.94	85,549.84
927	4,741.72	4,589.58	4,533.97	4,533.72	18,398.99
928	7,901.55	8,001.24	7,967.18	7,972.67	31,842.64
930	5,658	5,870.35	5,803.10	5,636.99	22,968.44
931	3,418.50	3,490.90	3,200.40	3,150.94	13,260.74
932	1,826.73	1,878.82	1,766.65	1,860.53	7,332.73
933	1,830.32	1,980.89	1,917.32	1,991.08	7,719.61
934	3,243.50	3,464.45	3,384.48	3,554.32	13,646.75

	935	2,266.78	2,331.36	2,216.47	2,324.32	9,138.93
	936	2,191.61	2,238.72	2,220.73	2,267.07	8,918.13
	937	3,220.95	3,489.91	3,420.07	3,443.30	13,574.23
	939	1,782.44	1,517.15	1,647.58	1,661.16	6,608.33
	940	6,015.56	5,875.57	5,830.74	6,075.81	23,797.68
	941	11,324.71	11,891.46	11,453.66	12,133.52	46,803.35
	943	1,042.64	1,049.59	1,020.94	1,029.72	4,142.89
	944	1,300.21	1,214.39	1,265.94	1,289.23	5,069.77
	945	15,812.97	16,272.27	16,129.27	16,479.85	64,694.36
	946	3,512.79	3,749.70	3,607.58	3,806.18	14,676.25
	947	1,390.10	1,431.27	1,322.38	1,537.79	5,681.54
	948	728.19	668.91	659.4	804.53	2,861.03
	949	4,749.65	4,929.17	4,658.09	5,030.87	19,367.78
	950	7,343.05	7,224.69	7,112.19	7,503	29,182.93
	951	3,836.41	4,018.19	3,870.05	3,868.50	15,593.15
	952	1,954.64	1,911.29	1,927.89	2,044.15	7,837.97
	953	4,988.01	4,896.66	4,795.65	4,973.85	19,654.17
	954	3,788.85	3,787.57	3,910.37	3,706.14	15,192.93
	955	812.13	917.55	872.84	937.29	3,539.81
	956	10,543.11	9,969.77	10,392.98	10,298.08	41,203.94
	957	2,330.69	2,259.98	2,288.37	2,483.16	9,362.20
	958	4,801.01	4,939.51	4,870.09	5,178.32	19,788.93
	959	4,385.25	4,154.12	4,210.39	4,367.89	17,117.65
	960	1,650.51	1,617.02	1,718.10	1,743.77	6,729.40
	961	528.79	534	603.12	622.15	2,288.06
TOTAL		295,548.59	301,541.38	296,156.73	304,886.81	1,198,133.51

STATE: COLORADO

ZIP CODE	QUARTER 1	QUARTER 2	QUARTER 3	QUARTER 4	TOTAL GRAMS
800	11,696.71	11,873.03	12,101.73	12,318.67	47,990.14
801	14,129.25	14,264.28	14,925.21	15,316.41	58,635.15
802	19,488.96	19,977.75	20,030.15	20,805.07	80,301.93
803	3,425.91	3,432.56	3,287.27	3,594.31	13,740.05
804	2,992.20	2,898.28	3,075.77	3,195.80	12,162.05
805	7,533.98	7,640.99	7,691.15	7,985.39	30,851.51
806	2,383.33	2,337.20	2,339.16	2,445.47	9,505.16

	807	311.91	304.22	351.84	357.16	1,325.13
	808	1,157.90	1,131.96	1,196.88	1,111.36	4,598.10
	809	7,517.63	7,595.61	7,720.62	7,692.14	30,526
	810	1,344.58	1,397.99	1,443.49	1,503.88	5,689.94
	811	221.92	209.73	271.26	245.93	948.84
	812	541.97	549.05	584.12	599.06	2,274.20
	813	659.07	580.92	707.89	670.75	2,618.63
	814	541.33	554.16	592	590.08	2,277.57
	815	1,676.64	1,700.33	1,645.41	1,712.63	6,735.01
	816	2,338.52	2,314.90	2,366.51	2,254.82	9,274.75
TOTAL		77,961.81	78,762.96	80,330.46	82,398.93	319,454.16

STATE:	CONNECTICUT				
ZIP CODE	QUARTER 1	QUARTER 2	QUARTER 3	QUARTER 4	TOTAL GRAMS
60	8,933.06	9,171.42	9,042.11	9,352.24	36,498.83
61	3,307	3,319.39	3,222.41	3,337.42	13,186.22
62	2,287.48	2,162.52	2,143.04	2,278.32	8,871.36
63	4,877.18	5,125.23	5,472.97	5,471.01	20,946.39
64	12,158.63	12,459.19	12,371.59	12,745.33	49,734.74
65	3,984.48	4,079.48	4,003.77	4,186.85	16,254.58
66	1,363.78	1,439.68	1,463.26	1,523.12	5,789.84
67	3,421.25	3,383.24	3,466.52	3,534.35	13,805.36
68	9,316.71	9,788.80	9,843.18	10,230.35	39,179.04
69	1,642.23	1,833.29	1,777.89	1,752.73	7,006.14
TOTAL	51,291.80	52,762.24	52,806.74	54,411.72	211,272.50

STATE:	DELAWARE				
ZIP CODE	QUARTER 1	QUARTER 2	QUARTER 3	QUARTER 4	TOTAL GRAMS
197	9,287.84	9,074.47	8,805.55	9,323.69	36,491.55
198	6,799.40	6,770.01	6,609.31	7,015.87	27,194.59
199	5,547.29	5,657.26	5,785.12	5,526.09	22,515.76
TOTAL	21,634.53	21,501.74	21,199.98	21,865.65	86,201.90

RETAIL DRUG DISTRIBUTION BY ZIP CODE WITHIN STATE BY GRAMS WT

STATE: DISTRICT OF COLUMBIA

ZIP CODE	QUARTER 1	QUARTER 2	QUARTER 3	QUARTER 4	TOTAL GRAMS
200	10,996.52	11,145.90	10,861.12	11,405.14	44,408.68
203	125.13	106.36	114.8	184.37	530.66
204	444.22	388.26	527.48	366.3	1,726.26
205	0	0	1.25	0	1.25
TOTAL	11,565.87	11,640.52	11,504.65	11,955.81	46,666.85

STATE: FLORIDA

ZIP CODE	QUARTER 1	QUARTER 2	QUARTER 3	QUARTER 4	TOTAL GRAMS
320	9,146.12	9,207.99	9,041.66	9,609.20	37,004.97
321	7,373.40	7,526.75	7,433.44	7,992.75	30,326.34
322	21,971.65	22,084.34	21,925.61	22,612.64	88,594.24
323	9,889.21	9,661.28	9,289.17	9,882.67	38,722.33
324	8,460.43	8,427.05	8,049.03	8,703.87	33,640.38
325	18,579.24	18,584.37	19,630.60	18,646.41	75,440.62
326	5,964	5,814.84	5,722.68	6,086.29	23,587.81
327	16,761.71	17,214.41	16,724.06	18,667.88	69,368.06
328	13,391.49	13,285.12	13,026	14,154.71	53,857.32
329	12,842.22	12,697.29	12,777.45	13,707.01	52,023.97
330	14,727.70	14,941.64	13,669.64	15,286.96	58,625.94
331	16,637.04	16,744.48	15,447.49	17,890.81	66,719.82
333	11,919.82	12,383.42	11,858.74	13,286.79	49,448.77
334	24,406.03	23,924.55	23,260.12	25,221.13	96,811.83
335	10,112.16	10,176.29	10,422.99	10,955.02	41,666.46
336	13,939.13	14,414.81	14,191.35	15,338.86	57,884.15
337	13,469.21	13,587.98	13,954.16	14,728.35	55,739.70
338	7,175.21	6,998.49	6,800.10	7,472.60	28,446.40
339	12,465.27	12,580.42	11,567.97	13,482.35	50,096.01
341	6,902.51	6,763.77	6,346.86	7,617.42	27,630.56
342	13,439.02	13,363.75	13,146.62	14,212.58	54,161.97
344	3,968.04	3,990.32	3,632.20	4,283.13	15,873.69
346	8,663.74	8,873.38	8,915.93	9,606.74	36,059.79
347	7,964.67	7,923.20	7,507.01	8,313.70	31,708.58
349	6,725.69	6,835.63	6,931.43	7,272.84	27,765.59

TOTAL		296,894.71	298,005.57	291,272.31	315,032.71	1,201,205.30

STATE:	GEORGIA					
ZIP CODE		QUARTER 1	QUARTER 2	QUARTER 3	QUARTER 4	TOTAL GRAMS
	300	37,676.98	37,377.16	37,809.55	38,916.56	151,780.25
	301	21,547.12	21,826.22	21,991.20	22,618.70	87,983.24
	302	13,337.13	13,286.42	13,554.99	14,429.06	54,607.60
	303	22,373.26	22,698.53	22,705.62	23,290.28	91,067.69
	304	6,930.35	6,837.09	6,915.72	7,049.87	27,733.03
	305	11,686.40	11,679.45	11,764.08	12,444.59	47,574.52
	306	8,229.83	7,813.74	7,927.69	8,397.37	32,368.63
	307	6,685.50	6,600.43	6,483.94	6,687.35	26,457.22
	308	3,526.40	3,380.23	3,524.14	3,604.96	14,035.73
	309	5,474.83	5,373.12	5,549.52	5,579.15	21,976.62
	310	7,300.20	6,997.06	6,842.57	7,564.48	28,704.31
	312	2,805.14	2,791.83	2,692.69	2,849.14	11,138.80
	313	6,238.15	5,973.83	6,225.68	6,040.99	24,478.65
	314	6,046.71	6,233.43	5,904.11	6,292.20	24,476.45
	315	7,885.51	7,701.29	7,967.72	8,233.54	31,788.06
	316	3,508.77	3,226.40	3,135.50	3,352.62	13,223.29
	317	6,614.76	6,332.81	6,380.13	6,657.74	25,985.44
	318	474.43	427.07	455.52	496.2	1,853.22
	319	6,616.44	6,793.61	6,993.49	6,727.59	27,131.13
	398	1,526.83	1,363	1,503.87	1,506.14	5,899.84
TOTAL		186,484.74	184,712.72	186,327.73	192,738.53	750,263.72

STATE:	GUAM					
ZIP CODE		QUARTER 1	QUARTER 2	QUARTER 3	QUARTER 4	TOTAL GRAMS
	969	411.77	559.5	574.21	358.63	1,904.11
TOTAL		411.77	559.5	574.21	358.63	1,904.11

STATE:	HAWAII					
ZIP CODE		QUARTER 1	QUARTER 2	QUARTER 3	QUARTER 4	TOTAL GRAMS
	967	4,677.70	4,633.36	4,693.58	4,785.69	18,790.33

	968	4,891.50	5,201.06	5,246.38	4,790.81	20,129.75
TOTAL		9,569.20	9,834.42	9,939.96	9,576.50	38,920.08

STATE:	IDAHO					
ZIP CODE		QUARTER 1	QUARTER 2	QUARTER 3	QUARTER 4	TOTAL GRAMS
	832	1,936.65	2,020.93	2,030.11	2,205.96	8,193.65
	833	1,770.90	1,849.49	1,790.76	1,917.01	7,328.16
	834	4,511.05	4,455.38	4,542.72	4,865.26	18,374.41
	835	363.39	334.26	410.27	414.16	1,522.08
	836	7,113.85	7,341.79	7,319.31	7,549.85	29,324.80
	837	6,912.39	7,182.50	7,174.81	7,482.23	28,751.93
	838	2,000.15	2,056.63	2,067.54	2,145.21	8,269.53
TOTAL		24,608.38	25,240.98	25,335.52	26,579.68	101,764.56

STATE:	ILLINOIS					
ZIP CODE		QUARTER 1	QUARTER 2	QUARTER 3	QUARTER 4	TOTAL GRAMS
	600	32,258.21	32,583.18	32,973.54	33,987.59	131,802.52
	601	22,148.68	22,334.46	22,703.45	23,420.12	90,606.71
	602	1,470.60	1,483.08	1,394.87	1,495.24	5,843.79
	603	1,262.07	1,200.83	1,194.54	1,371.88	5,029.32
	604	18,245.11	17,922.09	18,210.04	18,869.25	73,246.49
	605	17,328.76	17,421.46	17,470.52	18,237.56	70,458.30
	606	36,207.78	36,728.69	36,788.13	38,577.71	148,302.31
	607	2,114.51	2,201.15	2,182.65	2,338.65	8,836.96
	608	618.01	648.01	707.04	687.3	2,660.36
	609	1,987.42	1,969.36	2,027.29	2,158.88	8,142.95
	610	3,460.11	3,426.85	3,553.35	3,525.47	13,965.78
	611	3,749.15	3,811.54	3,928.17	3,952.67	15,441.53
	612	3,160.71	3,104.18	3,048.40	3,088.29	12,401.58
	613	2,377.45	2,454.66	2,422.23	2,556.25	9,810.59
	614	1,968.80	1,927.23	1,925.05	1,954.03	7,775.11
	615	3,190.98	3,162.57	3,184.67	3,310.95	12,849.17
	616	4,008.57	3,971.24	4,095.63	4,225.31	16,300.75
	617	3,810.21	3,469.35	3,572.56	3,748.50	14,600.62
	618	3,926.67	3,904.98	3,668.10	3,922.61	15,422.36

	619	1,314.31	1,222.32	1,127.18	1,304.52	4,968.33
	620	3,596.67	3,534.07	3,495.65	3,728.19	14,354.58
	622	5,212.19	5,019.72	5,295.60	5,495.74	21,023.25
	623	1,931.39	1,934.56	1,867.40	1,993.46	7,726.81
	624	1,256.72	1,183.47	1,176.22	1,180.58	4,796.99
	625	1,802.72	1,684.66	1,605.38	1,721.83	6,814.59
	626	1,533.48	1,512.41	1,490.88	1,636.76	6,173.53
	627	2,433.51	2,394.49	2,326.37	2,470.36	9,624.73
	628	2,382.42	2,337.55	2,368.47	2,402.20	9,490.64
	629	3,971.66	3,830.07	3,855.80	3,838.99	15,496.52
TOTAL		188,728.87	188,378.23	189,659.18	197,200.89	763,967.17

STATE: INDIANA

ZIP CODE		QUARTER 1	QUARTER 2	QUARTER 3	QUARTER 4	TOTAL GRAMS
	460	14,082.04	14,427.79	14,621.18	15,147.16	58,278.17
	461	8,537.15	8,637.74	8,835.51	9,152.99	35,163.39
	462	15,913.92	16,007.20	16,383.52	16,792.26	65,096.90
	463	11,699.54	11,559.31	12,066.72	12,367.67	47,693.24
	464	1,287.23	1,217.21	1,179.85	1,182.23	4,866.52
	465	16,953.33	17,451.93	17,211.78	17,726.66	69,343.70
	466	4,512.19	4,660	4,598.08	4,851.14	18,621.41
	467	3,454.65	3,314.09	3,289.37	3,471.43	13,529.54
	468	5,698.03	5,598.25	5,614.15	5,962.56	22,872.99
	469	5,346.98	5,294.25	5,416.86	5,570.08	21,628.17
	470	1,565.39	1,532.66	1,500.86	1,660.66	6,259.57
	471	7,675.23	8,608.07	8,248.72	7,710.49	32,242.51
	472	2,185.13	2,225.92	2,365.60	2,441.40	9,218.05
	473	5,005.73	4,862.14	5,062.47	5,002.41	19,932.75
	474	4,052.96	3,972.20	3,838.75	4,136.40	16,000.31
	475	2,231.19	2,174.47	2,262.22	2,371.55	9,039.43
	476	2,550.80	2,548	2,520.70	2,739.84	10,359.34
	477	5,789.77	5,555.45	5,876.81	5,816.12	23,038.15
	478	2,258.54	2,249.28	2,290.69	2,432.91	9,231.42
	479	6,918.42	6,840.65	6,770.16	7,045.86	27,575.09
TOTAL		127,718.22	128,736.61	129,954	133,581.82	519,990.65

STATE:	IOWA				
ZIP CODE	QUARTER 1	QUARTER 2	QUARTER 3	QUARTER 4	TOTAL GRAMS
500	4,007.25	3,989.62	4,087.29	4,321.12	16,405.28
501	1,758.57	1,643.92	1,837.44	1,917.16	7,157.09
502	2,882.65	2,972.32	2,942.58	3,054.74	11,852.29
503	5,567.10	5,762.03	5,860.45	6,020.78	23,210.36
504	816.87	850.76	811.09	837.64	3,316.36
505	1,125.31	1,149.99	1,138.33	1,218.90	4,632.53
506	1,471.68	1,466.43	1,379.27	1,636.21	5,953.59
507	1,124.64	1,208.32	1,183.53	1,233.66	4,750.15
508	168.56	173.34	171.79	148.28	661.97
510	923.41	950.13	947.23	961.13	3,781.90
511	2,530.50	2,644.26	2,688.89	2,698.10	10,561.75
512	433.75	385.45	406.02	472.05	1,697.27
513	681.41	713.7	726.62	720.64	2,842.37
514	410.67	381.67	393.64	412.03	1,598.01
515	1,992.99	2,066.72	2,092.30	2,098.33	8,250.34
516	190.95	184.42	187.6	192.62	755.59
520	3,062.20	3,144.39	3,217.16	3,377.22	12,800.97
521	482.58	426.14	471.72	522.79	1,903.23
522	4,806.13	4,885.96	4,786.67	4,995.88	19,474.64
523	2,248.97	2,263.87	2,342.46	2,567.90	9,423.20
524	3,762.67	3,876.31	3,941.52	4,263.08	15,843.58
525	928.9	904.04	925.1	976.7	3,734.74
526	1,574.98	1,660.19	1,609.89	1,595.20	6,440.26
527	3,153.56	3,122.25	3,195.28	3,273.03	12,744.12
528	2,329.13	2,568.24	2,473.54	2,607.58	9,978.49
TOTAL	48,435.43	49,394.47	49,817.41	52,122.77	199,770.08

STATE:	KANSAS				
ZIP CODE	QUARTER 1	QUARTER 2	QUARTER 3	QUARTER 4	TOTAL GRAMS
660	13,760.24	14,306.76	14,433.43	14,969.69	57,470.12
661	1,682.82	1,874.74	1,751.35	1,872.93	7,181.84
662	17,745.17	18,500.59	18,647.34	19,424.40	74,317.50
664	966.74	924.22	1,103.54	785.16	3,779.66

	665	1,535.75	1,433.16	1,461.22	1,609.66	6,039.79
	666	4,714.97	4,994.61	5,006.25	5,122.81	19,838.64
	667	1,709.61	1,672.62	1,703.08	1,704.10	6,789.41
	668	645.09	618.96	619.26	607.98	2,491.29
	669	104.15	108.91	89.56	116.87	419.49
	670	2,389.58	2,400.78	2,470.36	2,478.99	9,739.71
	671	910.24	929.37	976.74	1,086.33	3,902.68
	672	7,395.99	7,313.87	7,454.12	7,592.29	29,756.27
	673	813.9	761.67	794.99	782.72	3,153.28
	674	1,313.93	1,235.99	1,250.87	1,257.59	5,058.38
	675	1,094.34	1,010	1,035.38	1,182.57	4,322.29
	676	608.71	602.23	665.44	730.47	2,606.85
	677	351.49	337.56	333.83	356.52	1,379.40
	678	684.29	644.57	662.79	686.34	2,677.99
	679	213.79	216.18	200.61	203.54	834.12
TOTAL		58,640.80	59,886.79	60,660.16	62,570.96	241,758.71

STATE:	KENTUCKY					
ZIP CODE		QUARTER 1	QUARTER 2	QUARTER 3	QUARTER 4	TOTAL GRAMS
	400	3,799.62	3,561.18	3,803.46	3,845.21	15,009.47
	401	1,626.35	1,684.28	1,861.63	1,381.23	6,553.49
	402	16,526.98	16,030.78	16,159.74	16,825.22	65,542.72
	403	4,014.41	3,751.53	3,913.64	4,033.78	15,713.36
	404	1,626.28	1,650.46	1,606.80	1,828.94	6,712.48
	405	6,901.37	6,739.77	6,768.40	7,247.03	27,656.57
	406	733.18	750.61	712.82	765.58	2,962.19
	407	548.77	519.13	515.76	582.06	2,165.72
	408	150.05	185.11	160.31	173.37	668.84
	409	174.23	175.91	202.89	226.33	779.36
	410	9,460.87	9,428.55	9,257.37	10,049.20	38,195.99
	411	1,351.43	1,344.22	1,358.49	1,396.30	5,450.44
	412	383.97	327.08	303	350.08	1,364.13
	413	143.76	114.73	106.38	128.02	492.89
	414	146.43	138.74	119.86	140.52	545.55
	415	458.58	511.43	520.73	504.19	1,994.93
	416	333.06	309.02	333.96	337.28	1,313.32

	417	368.09	319.9	349.19	369.16	1,406.34
	418	120.15	88.01	109.48	89.96	407.6
	420	4,204.70	4,332.25	4,347.94	4,491.43	17,376.32
	421	2,877.44	2,795.05	2,861.53	2,993.76	11,527.78
	422	2,169.96	2,073.88	2,316.23	2,102.13	8,662.20
	423	3,558.06	3,451.01	3,361.22	3,513.17	13,883.46
	424	3,457.80	3,433.88	3,319.88	3,471.92	13,683.48
	425	399.28	398.05	349.51	432.53	1,579.37
	426	222.87	191.4	209.92	211.68	835.87
	427	2,357.79	2,249.94	2,352.46	2,503.57	9,463.76
TOTAL		68,115.48	66,555.90	67,282.60	69,993.65	271,947.63

STATE: LOUISIANA

ZIP CODE		QUARTER 1	QUARTER 2	QUARTER 3	QUARTER 4	TOTAL GRAMS
	700	19,234.03	18,825.21	19,172.10	20,164.14	77,395.48
	701	9,828.23	9,744.46	9,810.42	10,601.66	39,984.77
	703	6,074.21	5,728.41	5,752.10	6,197.80	23,752.52
	704	18,262.36	18,193.06	18,838.30	19,606.50	74,900.22
	705	31,529.78	30,602.94	31,156.61	33,096.45	126,385.78
	706	10,912.52	10,783.84	10,932.92	11,738.63	44,367.91
	707	18,031.06	18,298.23	18,444.97	19,670.39	74,444.65
	708	15,974.03	15,780.14	15,668.68	16,706.46	64,129.31
	710	1,865.68	1,822.96	1,949.23	1,989.83	7,627.70
	711	7,227.87	7,146.95	7,343.48	7,418.68	29,136.98
	712	6,671.04	6,456.30	6,577.05	6,743.51	26,447.90
	713	5,312.33	5,305.65	5,246.79	5,668.09	21,532.86
	714	1,916.92	1,714.14	1,927.70	1,943.64	7,502.40
TOTAL		152,840.06	150,402.29	152,820.35	161,545.78	617,608.48

STATE: MAINE

ZIP CODE		QUARTER 1	QUARTER 2	QUARTER 3	QUARTER 4	TOTAL GRAMS
	39	811.63	834.75	912.81	901.4	3,460.59
	40	7,349.93	7,760.05	8,147.28	7,889.66	31,146.92
	41	3,797.64	4,161.47	4,221.02	4,061.28	16,241.41
	42	1,817.12	1,950.28	1,916.86	2,007.25	7,691.51

	43	1,365.26	1,391.25	1,505.57	1,437.36	5,699.44
	44	2,155.53	2,146.54	2,163.66	2,264	8,729.73
	45	656.27	771.75	790.47	758.08	2,976.57
	46	679.18	744.43	820.01	746.88	2,990.50
	47	504.94	553.17	501.82	459.47	2,019.40
	48	508.01	601.72	581.36	568.4	2,259.49
	49	1,774.41	1,861.93	1,893.68	1,926.70	7,456.72
TOTAL		21,419.92	22,777.34	23,454.54	23,020.48	90,672.28

STATE: MARYLAND

ZIP CODE		QUARTER 1	QUARTER 2	QUARTER 3	QUARTER 4	TOTAL GRAMS
	206	4,305.63	4,261.36	4,411.87	4,322.56	17,301.42
	207	6,198.69	6,038.62	5,998.51	6,036.84	24,272.66
	208	14,360.66	14,503.05	14,634.05	14,455.43	57,953.19
	209	2,611.66	2,646.45	2,568.07	2,753.44	10,579.62
	210	16,700.18	17,201.82	17,225.21	17,953.79	69,081
	211	7,843.55	8,070.78	7,672.37	8,160.60	31,747.30
	212	16,051.43	16,095.77	16,134.42	16,944.78	65,226.40
	214	2,555.94	2,641.90	2,619.49	2,708.32	10,525.65
	215	1,748.91	1,843.08	1,862.29	1,933.06	7,387.34
	216	2,973.73	3,027.90	3,066.30	3,137.70	12,205.63
	217	10,277.06	10,213.21	10,200.74	10,770.95	41,461.96
	218	2,013.85	2,042.68	2,023.88	2,076.93	8,157.34
	219	2,539.02	2,521.03	2,623.55	2,680.27	10,363.87
TOTAL		90,180.31	91,107.65	91,040.75	93,934.67	366,263.38

STATE: MASSACHUSETTS

ZIP CODE		QUARTER 1	QUARTER 2	QUARTER 3	QUARTER 4	TOTAL GRAMS
	10	8,821.20	9,201.54	8,646.89	9,207	35,876.63
	11	2,263.63	2,164.77	2,071.93	2,264.63	8,764.96
	12	2,319.16	2,295.96	2,212.25	2,417.26	9,244.63
	13	2,465.56	2,520.33	2,559.08	2,668.17	10,213.14
	14	5,077.38	5,268.81	5,145.08	5,385.99	20,877.26
	15	7,214.75	7,316.36	6,879.90	7,332.50	28,743.51
	16	5,800.43	5,978.76	5,856.56	5,675.37	23,311.12

	17	11,031.49	11,536.75	11,243.66	11,617	45,428.90
	18	19,618.17	20,333.38	20,003.70	20,565.59	80,520.84
	19	17,907.80	17,710.95	17,926.62	18,192.87	71,738.24
	20	13,411.10	13,807.91	13,524.27	14,111.91	54,855.19
	21	33,206.60	34,157.36	32,873.18	34,291.66	134,528.80
	22	1,714.26	1,685.24	1,492.41	1,582.23	6,474.14
	23	13,004.34	13,302.17	13,264.71	13,563.67	53,134.89
	24	11,168.54	11,327.45	10,511.87	11,251.79	44,259.65
	25	3,843.66	4,161.42	4,162.77	4,027.41	16,195.26
	26	4,546.35	4,979.82	5,299.21	4,916.52	19,741.90
	27	11,580.96	11,969.58	12,017.31	12,382.07	47,949.92
TOTAL		174,995.38	179,718.56	175,691.40	181,453.64	711,858.98

STATE:	MICHIGAN					
ZIP CODE		QUARTER 1	QUARTER 2	QUARTER 3	QUARTER 4	TOTAL GRAMS
	480	38,656.60	38,648.05	39,024.60	39,455.22	155,784.47
	481	39,332.73	39,905.80	39,748.89	41,263.84	160,251.26
	482	6,850.44	7,019.11	6,703.56	7,003.62	27,576.73
	483	32,245.21	32,071.50	32,229.85	32,989.82	129,536.38
	484	8,367.48	8,316.75	8,423.08	8,726.81	33,834.12
	485	2,865.71	2,810.55	2,800.97	2,753.72	11,230.95
	486	5,092.86	5,052.84	4,979.66	5,071.62	20,196.98
	487	2,974.80	3,087.54	2,912.99	2,944.34	11,919.67
	488	14,864.89	14,396.30	14,521.59	15,414.39	59,197.17
	489	4,268.94	4,142.66	4,247.53	4,255.99	16,915.12
	490	18,047.81	17,831.34	17,620.62	17,845.43	71,345.20
	491	2,198.71	2,237.25	2,193.20	2,214.41	8,843.57
	492	4,228.85	4,093.76	4,238.92	4,228.24	16,789.77
	493	7,137.22	6,984.17	6,939.20	7,169.91	28,230.50
	494	19,061.59	19,115.64	19,176.82	19,731.66	77,085.71
	495	19,011.11	19,421.45	19,183.13	19,649.02	77,264.71
	496	5,556.97	5,516.70	5,745.99	5,672.27	22,491.93
	497	3,247.40	3,415.96	3,606.06	3,490.72	13,760.14
	498	3,145.86	3,282.97	3,339.05	3,341.59	13,109.47
	499	1,732.95	1,668.29	1,584.75	1,591.47	6,577.46
TOTAL		238,888.13	239,018.63	239,220.46	244,814.09	961,941.31

STATE: MINNESOTA

ZIP CODE	QUARTER 1	QUARTER 2	QUARTER 3	QUARTER 4	TOTAL GRAMS
550	12,269.97	12,406.39	12,449.60	12,896	50,021.96
551	17,582.18	17,522.84	17,539.11	18,256.55	70,900.68
553	22,333.84	22,337.21	22,514.02	23,228.86	90,413.93
554	25,523.27	26,036.67	25,618.70	26,664.52	103,843.16
556	115.42	102.78	121.52	107.42	447.14
557	3,059.16	3,086.96	3,034.53	3,101.94	12,282.59
558	2,063.74	2,014.90	1,931.05	2,140.05	8,149.74
559	5,234.76	5,273.28	5,150.75	5,327.15	20,985.94
560	5,190.94	5,166.22	5,069.61	5,297.46	20,724.23
561	776.7	835.56	735.28	832.42	3,179.96
562	1,672.96	1,590.17	1,553.49	1,715.47	6,532.09
563	4,197.23	4,174.58	4,154.96	4,346.07	16,872.84
564	2,198.72	2,168.91	2,287.62	2,255.63	8,910.88
565	2,553.22	2,587.72	2,638.54	2,808.72	10,588.20
566	1,209.33	1,237.78	1,242.86	1,299.88	4,989.85
567	658.95	677.45	701.69	770.25	2,808.34
TOTAL	106,640.39	107,219.42	106,743.33	111,048.39	431,651.53

STATE: MISSISSIPPI

ZIP CODE	QUARTER 1	QUARTER 2	QUARTER 3	QUARTER 4	TOTAL GRAMS
386	9,325.98	8,954.60	9,062.62	9,487.48	36,830.68
387	1,566.22	1,447.84	1,525.47	1,520.57	6,060.10
388	5,068.11	4,916.24	4,730.20	4,970.83	19,685.38
389	1,515.18	1,500.01	1,466.44	1,491.24	5,972.87
390	5,218.56	4,994.31	5,019.33	5,063.92	20,296.12
391	6,290.53	6,140.98	6,128.04	6,366.66	24,926.21
392	5,283.71	5,074.05	5,079.07	5,266.11	20,702.94
393	4,176.49	4,104.13	3,859.95	4,045.21	16,185.78
394	8,916.65	8,647.63	8,665.39	8,957.79	35,187.46
395	8,850.27	8,723.88	8,930.98	9,452.34	35,957.47
396	2,505.74	2,494.17	2,487.21	2,424.41	9,911.53
397	3,886.42	3,448.36	3,482.63	3,503.70	14,321.11

TOTAL		62,603.86	60,446.20	60,437.33	62,550.26	246,037.65

STATE:	MISSOURI					
ZIP CODE		QUARTER 1	QUARTER 2	QUARTER 3	QUARTER 4	TOTAL GRAMS
	630	19,181.59	19,284.93	19,296.99	20,172.99	77,936.50
	631	38,390.63	37,857.57	39,594.90	40,418.54	156,261.64
	633	13,801.98	13,789.18	13,775.98	14,406.98	55,774.12
	634	1,140.37	1,115.66	1,143.29	1,248.91	4,648.23
	635	1,183.08	1,256.13	1,169.78	1,301.98	4,910.97
	636	1,997.66	2,048.85	1,956.21	2,068.95	8,071.67
	637	2,217.43	2,242.36	2,250.24	2,278.04	8,988.07
	638	1,612.84	1,507.82	1,556.13	1,645.27	6,322.06
	639	565.93	522.82	492.89	506.96	2,088.60
	640	13,719.85	14,107.80	14,160.35	14,531.50	56,519.50
	641	16,730.58	17,453.02	17,306.09	18,154.32	69,644.01
	644	689.6	646.84	626.9	679.84	2,643.18
	645	1,134	1,090.84	1,159.53	1,174.74	4,559.11
	646	426.64	443.96	422.91	456.25	1,749.76
	647	944.67	984.64	1,007.46	1,035.43	3,972.20
	648	1,555.05	1,453.38	1,499.07	1,466.53	5,974.03
	650	1,018.86	1,075.39	1,121.85	1,139.17	4,355.27
	651	1,176.32	1,158.61	1,151.90	1,144.43	4,631.26
	652	5,358.44	5,191.98	5,038.46	5,447.29	21,036.17
	653	998.82	974.58	961.19	984.39	3,918.98
	654	832.27	855.1	934.73	913.25	3,535.35
	655	684.21	637.07	691.81	634	2,647.09
	656	1,357.64	1,263.55	1,350.67	1,384.11	5,355.97
	657	1,725.62	1,775.44	1,776.91	1,893.37	7,171.34
	658	3,429.02	3,424.56	3,459.75	3,568.61	13,881.94
TOTAL		131,873.10	132,162.08	133,905.99	138,655.85	536,597.02

STATE:	MONTANA					
ZIP CODE		QUARTER 1	QUARTER 2	QUARTER 3	QUARTER 4	TOTAL GRAMS
	590	567.46	594.35	640.11	597.27	2,399.19
	591	2,343.69	2,356.78	2,317.45	2,419.18	9,437.10

	592	366.39	350.56	347.69	356.48	1,421.12
	593	309.3	328.65	316.07	312.53	1,266.55
	594	1,105.99	1,142.66	1,163.40	1,117.01	4,529.06
	595	199.58	193.12	220.31	219.75	832.76
	596	1,078.58	1,069.82	1,080.63	1,137.37	4,366.40
	597	2,979.15	3,154.06	3,258.84	3,376.49	12,768.54
	598	3,476.33	3,486.02	3,562.76	3,602.68	14,127.79
	599	1,179.06	1,202.38	1,252.61	1,214.09	4,848.14
TOTAL		13,605.53	13,878.40	14,159.87	14,352.85	55,996.65

STATE: NEBRASKA

ZIP CODE		QUARTER 1	QUARTER 2	QUARTER 3	QUARTER 4	TOTAL GRAMS
	680	2,240.59	2,316.89	2,295.69	2,347.71	9,200.88
	681	11,977.48	12,024.90	12,377.83	12,739.13	49,119.34
	683	520.51	503.38	531.87	506.4	2,062.16
	684	450.57	424.11	470.27	460.84	1,805.79
	685	5,322.50	5,188.87	5,377.41	5,620.76	21,509.54
	686	445.86	490.58	466.69	474.83	1,877.96
	687	1,024.39	1,023.16	1,046.33	1,131.23	4,225.11
	688	1,448.08	1,404.51	1,447.51	1,620.31	5,920.41
	689	476.83	467.89	510.8	484.47	1,939.99
	690	181.53	176.25	197.56	168.88	724.22
	691	678.64	637.9	696.39	672.44	2,685.37
	692	28.83	26.73	41.47	39.16	136.19
	693	527.25	540.12	557.75	484.2	2,109.32
TOTAL		25,323.06	25,225.29	26,017.57	26,750.36	103,316.28

STATE: NEVADA

ZIP CODE		QUARTER 1	QUARTER 2	QUARTER 3	QUARTER 4	TOTAL GRAMS
	890	5,755.79	5,737.56	5,701.19	5,840.85	23,035.39
	891	14,646.21	15,020.56	14,888.37	15,247.04	59,802.18
	893	60.72	55.73	52.7	45.63	214.78
	894	1,537.89	1,547.57	1,569.84	1,726.76	6,382.06
	895	2,604.83	2,713.18	2,823.42	2,851.37	10,992.80
	897	495.33	484.57	504.33	533.57	2,017.80

	898	394.42	425.21	430.54	452.37	1,702.54
TOTAL		25,495.19	25,984.38	25,970.39	26,697.59	104,147.55

STATE: NEW HAMPSHIRE

ZIP CODE		QUARTER 1	QUARTER 2	QUARTER 3	QUARTER 4	TOTAL GRAMS
	30	11,366.07	11,964.81	11,848.87	12,047.21	47,226.96
	31	5,938.12	6,306.54	6,145.86	6,467.55	24,858.07
	32	2,642.38	2,809.01	2,831.69	2,834.78	11,117.86
	33	1,963.79	1,977.96	2,061.07	2,058.70	8,061.52
	34	1,473.37	1,537.42	1,496.38	1,598.25	6,105.42
	35	524.8	536.52	508.71	543.84	2,113.87
	36	95.36	91.15	94.54	96.88	377.93
	37	1,754.28	1,855.55	1,734.25	1,772.81	7,116.89
	38	11,510.33	11,662.69	11,754.65	11,962.92	46,890.59
TOTAL		37,268.50	38,741.65	38,476.02	39,382.94	153,869.11

STATE: NEW JERSEY

ZIP CODE		QUARTER 1	QUARTER 2	QUARTER 3	QUARTER 4	TOTAL GRAMS
	70	15,822.01	16,225.65	16,176.53	17,069.03	65,293.22
	71	1,362.78	1,351.99	1,209.23	1,389.54	5,313.54
	72	577.89	593.78	551.94	615.44	2,339.05
	73	1,418.64	1,502.29	1,486.48	1,586.56	5,993.97
	74	5,893.38	5,951.77	5,977.31	6,428.95	24,251.41
	75	601.72	641.84	614.53	568.53	2,426.62
	76	5,189.34	5,197.87	5,345.28	5,628.45	21,360.94
	77	7,943.89	8,216.46	8,314.21	8,677.09	33,151.65
	78	5,307.88	5,315.30	5,343.38	5,521.29	21,487.85
	79	4,524.63	4,595.77	4,762.45	4,712.85	18,595.70
	80	23,402.18	23,353.42	23,142.33	23,732.08	93,630.01
	81	1,613.60	1,684.18	1,700.54	1,741.29	6,739.61
	82	3,754.25	3,953.29	4,056.56	3,952.98	15,717.08
	83	2,608.24	2,490.07	2,481.96	2,590.39	10,170.66
	84	380.06	390.25	390.37	334.11	1,494.79
	85	3,460.34	3,549.86	3,487.46	3,588.23	14,085.89
	86	2,698.23	2,721.11	2,689.84	2,709.88	10,819.06

	87	5,016.36	5,413.95	5,738.26	6,068.13	22,236.70
	88	9,402.33	9,540.22	9,105.88	9,549.85	37,598.28
	89	991.79	975.82	916.5	1,088.59	3,972.70
TOTAL		101,969.54	103,664.89	103,491.04	107,553.26	416,678.73

STATE: NEW MEXICO

ZIP CODE		QUARTER 1	QUARTER 2	QUARTER 3	QUARTER 4	TOTAL GRAMS
	870	752.21	657.43	659.95	616.09	2,685.68
	871	8,068.49	7,692.69	7,111.24	6,479.99	29,352.41
	873	129.82	123.92	126.73	126.8	507.27
	874	631.23	632.8	633.83	581.07	2,478.93
	875	1,944.24	1,958.64	1,952.37	1,919.33	7,774.58
	877	186.94	176.19	183.3	168.11	714.54
	878	62.71	48.82	40.93	45.49	197.95
	879	83.55	79.04	76.79	68.51	307.89
	880	1,963.32	1,922.91	1,753.11	2,000.27	7,639.61
	881	327.22	235.34	394.08	340.12	1,296.76
	882	1,741.97	1,797.58	1,785.25	1,772.94	7,097.74
	883	847.47	837.96	847.84	839.84	3,373.11
	884	62.88	63.04	57.24	50.7	233.86
TOTAL		16,802.05	16,226.36	15,622.66	15,009.26	63,660.33

STATE: NEW YORK

ZIP CODE		QUARTER 1	QUARTER 2	QUARTER 3	QUARTER 4	TOTAL GRAMS
	100	47,685.50	50,501.81	49,015.61	51,570.23	198,773.15
	101	2,854.39	2,719.06	2,563.27	2,918.06	11,054.78
	102	478.26	477.81	471.29	486.35	1,913.71
	103	3,111.01	3,307.45	3,231.45	3,407.75	13,057.66
	104	3,929	3,887.25	3,650.77	3,862.95	15,329.97
	105	8,901.30	9,158.57	9,351.09	9,614.99	37,025.95
	106	1,313.83	1,296.55	1,191.40	1,247.61	5,049.39
	107	1,627.88	1,781.05	1,772.28	1,852.64	7,033.85
	108	768.11	765.46	706.68	762.88	3,003.13
	109	6,258.25	6,351.17	6,242.36	6,741.89	25,593.67
	110	2,363.33	2,464.57	2,550.93	2,527.27	9,906.10

111	2,606.59	2,690.64	2,703.04	2,888.19	10,888.46
112	15,912.46	16,678.13	16,141.09	17,061.77	65,793.45
113	5,195.38	5,472.84	5,269.04	5,644.96	21,582.22
114	1,259.58	1,316.61	1,230.88	1,330.71	5,137.78
115	7,035.65	7,334.60	7,506.12	7,684.90	29,561.27
116	523.49	520.42	503.4	543.59	2,090.90
117	19,754.28	20,088.74	20,570.61	21,076.11	81,489.74
118	974.16	1,100.85	1,059.99	1,099.63	4,234.63
119	2,702.73	2,976.62	3,237.74	2,968.02	11,885.11
120	5,089.25	5,108.10	5,303.27	5,347.21	20,847.83
121	3,571.85	3,591.02	3,631.99	3,798.22	14,593.08
122	2,757.39	2,800.59	2,800.66	2,947.66	11,306.30
123	2,195.85	2,196.15	2,126.06	2,329.26	8,847.32
124	1,742.70	1,828.83	1,919.16	1,928.61	7,419.30
125	5,145.02	5,313.37	5,236.16	5,667.06	21,361.61
126	1,262.35	1,300.02	1,275.32	1,359.24	5,196.93
127	1,113.24	1,127.64	1,178.42	1,172.83	4,592.13
128	3,205.85	3,213.98	3,409.34	3,343.34	13,172.51
129	2,159.74	2,244.31	2,290.65	2,351.49	9,046.19
130	5,602.05	5,570.61	5,672.83	6,091.26	22,936.75
131	1,745.53	1,713.55	1,701.69	1,881.86	7,042.63
132	3,058.22	3,122.78	3,106.88	3,272.72	12,560.60
133	1,000.73	986.42	904.02	1,053.83	3,945
134	1,868.75	1,994.33	1,797.23	1,932.55	7,592.86
135	937.37	941.53	970	954.62	3,803.52
136	2,531.80	2,638.83	2,726.73	2,674.07	10,571.43
137	1,248.33	1,252.52	1,185.77	1,253.74	4,940.36
138	1,518.94	1,630.03	1,504.45	1,656.66	6,310.08
139	1,081.32	1,021.85	959.78	1,037.87	4,100.82
140	6,193.95	6,213.22	6,284.54	6,494.76	25,186.47
141	2,806.79	2,892.28	2,887.81	2,862.07	11,448.95
142	8,823.66	8,918.68	8,857.45	9,367.87	35,967.66
143	1,005.50	1,048.34	989.63	1,156.62	4,200.09
144	5,382.73	5,393.73	5,397.44	5,816.16	21,990.06
145	4,863.37	5,150.55	5,006.84	5,250.25	20,271.01
146	9,129.97	9,516.47	9,316.67	9,859.76	37,822.87
147	1,598.31	1,598.96	1,508.75	1,511.42	6,217.44

	148	3,576.33	3,605.26	3,627.32	3,676.92	14,485.83
	149	906.85	874.03	880.18	890.33	3,551.39
TOTAL		228,378.92	235,698.18	233,426.08	244,230.76	941,733.94

STATE: NORTH CAROLINA

ZIP CODE		QUARTER 1	QUARTER 2	QUARTER 3	QUARTER 4	TOTAL GRAMS
	270	4,002.76	3,918.63	3,793.51	4,133.40	15,848.30
	271	5,610.48	5,417.73	5,595.30	5,774.14	22,397.65
	272	10,948.88	10,964.31	10,830.60	11,508.69	44,252.48
	273	5,801.83	5,874.17	5,856.59	6,040.14	23,572.73
	274	7,700.25	7,835.55	7,711.70	8,003.83	31,251.33
	275	23,093.80	23,304.62	23,170.67	24,480.88	94,049.97
	276	13,962.66	14,177.32	14,111.56	15,149.33	57,400.87
	277	5,653.65	5,879.62	5,765.86	5,889.22	23,188.35
	278	9,201.28	8,794.35	8,798.11	9,371.84	36,165.58
	279	2,981.77	3,179.85	3,149.41	3,313.22	12,624.25
	280	17,724.67	17,513.17	17,411.99	18,153.37	70,803.20
	281	11,947.85	11,931.06	12,097.88	12,292.27	48,269.06
	282	19,401.42	19,824.62	19,539.60	20,385.49	79,151.13
	283	14,806.90	14,563.26	14,686.99	14,952.25	59,009.40
	284	16,914.69	17,390.68	17,302.66	17,740.92	69,348.95
	285	11,357.36	11,417.50	11,732.43	10,965.96	45,473.25
	286	7,914.07	7,908	7,857.71	8,171.47	31,851.25
	287	7,119.54	7,173.23	7,063.36	7,359.54	28,715.67
	288	4,305.50	4,330.87	4,381.53	4,490.35	17,508.25
	289	385.46	406.43	436.57	421.36	1,649.82
TOTAL		200,834.82	201,804.97	201,294.03	208,597.67	812,531.49

STATE: NORTH DAKOTA

ZIP CODE		QUARTER 1	QUARTER 2	QUARTER 3	QUARTER 4	TOTAL GRAMS
	580	712.58	669.74	695.3	691.53	2,769.15
	581	2,147.83	2,115.42	2,219.63	2,236.90	8,719.78
	582	1,486.56	1,487.49	1,548.45	1,524.90	6,047.40
	583	582.59	512.39	529.29	494.65	2,118.92
	584	275.03	302.77	276.72	267.32	1,121.84

	585	1,435.23	1,509.72	1,452.50	1,528.08	5,925.53
	586	350.79	374.91	383.07	377.83	1,486.60
	587	1,305.35	1,383.08	1,380.44	1,426.98	5,495.85
	588	512.95	531.38	585.45	649.46	2,279.24
TOTAL		8,808.91	8,886.90	9,070.85	9,197.65	35,964.31

STATE:	OHIO					
ZIP CODE		QUARTER 1	QUARTER 2	QUARTER 3	QUARTER 4	TOTAL GRAMS
	430	15,577.69	15,691.25	15,377.30	16,021.50	62,667.74
	431	5,802.53	5,925.26	5,910.77	6,090.54	23,729.10
	432	31,407.16	31,151.36	32,995.32	36,156.42	131,710.26
	433	2,368.10	2,299.59	2,279.74	2,399.05	9,346.48
	434	2,061.63	2,044.61	2,024.24	2,168.74	8,299.22
	435	5,118.27	5,192.30	5,074.72	5,421.81	20,807.10
	436	7,949.73	8,157.50	8,246.90	8,406.35	32,760.48
	437	1,537.62	1,601.49	1,587.40	1,705.34	6,431.85
	438	215.21	223.07	234.29	258.23	930.8
	439	1,926.51	1,999.75	1,987.25	2,039.04	7,952.55
	440	14,161.18	14,420.70	14,591.11	15,394.64	58,567.63
	441	19,830.89	20,202.49	20,483.60	21,321.46	81,838.44
	442	8,214	8,192.14	8,318.92	8,443.48	33,168.54
	443	3,957.60	4,033.68	3,833.38	3,935.89	15,760.55
	444	4,410.40	4,349.92	4,489.45	4,658.23	17,908
	445	4,264.52	4,327.17	4,567.68	4,766.48	17,925.85
	446	5,779.88	5,891.81	5,859.68	5,934.44	23,465.81
	447	5,141.28	5,207.49	5,223.87	5,293.16	20,865.80
	448	3,352.58	3,241.79	3,298.12	3,441.26	13,333.75
	449	881.48	891.29	825.07	879.31	3,477.15
	450	14,744.50	14,931.86	14,868.74	15,574.40	60,119.50
	451	5,284.21	5,197.16	5,168.43	5,630.70	21,280.50
	452	21,077.55	20,853.22	20,818.05	21,932.22	84,681.04
	453	5,013.22	5,000.12	4,918.38	5,165.61	20,097.33
	454	6,024.62	5,994.59	5,846	6,186.27	24,051.48
	455	1,610.94	1,440.29	1,391.31	1,445.84	5,888.38
	456	3,719.97	3,529.66	3,463.64	3,702.07	14,415.34
	457	3,170.28	2,949.72	2,885.08	3,070.89	12,075.97

	458	3,338.24	3,259.81	3,344.34	3,520.82	13,463.21
TOTAL		207,941.79	208,201.09	209,912.78	220,964.19	847,019.85

STATE: OKLAHOMA

ZIP CODE		QUARTER 1	QUARTER 2	QUARTER 3	QUARTER 4	TOTAL GRAMS
	730	10,397.12	10,269.08	10,391.43	10,839.02	41,896.65
	731	9,047.19	9,215.82	8,922.56	9,401.65	36,587.22
	734	910.77	878.53	929.02	969.52	3,687.84
	735	2,452.25	2,497.80	2,713.73	2,285.70	9,949.48
	736	240.67	265.19	258.97	277.51	1,042.34
	737	909.53	995.94	986.48	1,052.38	3,944.33
	738	163.88	159.96	173.77	172.13	669.74
	739	73.34	75.8	70.78	58.19	278.11
	740	11,938.26	11,867.98	11,871.44	12,446.58	48,124.26
	741	9,406.92	9,695.19	9,520.66	9,940.79	38,563.56
	743	1,008.76	998.96	982.4	992.04	3,982.16
	744	2,599.40	2,651.18	2,682.03	2,620.17	10,552.78
	745	486.54	478.65	450.64	474.46	1,890.29
	746	265.01	259.01	274.66	263.01	1,061.69
	747	581.73	552.82	629.19	657.41	2,421.15
	748	1,364.83	1,301.56	1,256.71	1,366.84	5,289.94
	749	438.53	407.47	401.65	415.54	1,663.19
TOTAL		52,284.73	52,570.94	52,516.12	54,232.94	211,604.73

STATE: OREGON

ZIP CODE		QUARTER 1	QUARTER 2	QUARTER 3	QUARTER 4	TOTAL GRAMS
	970	12,457.94	12,606.28	12,605.77	12,828.90	50,498.89
	971	4,749.31	4,985.34	4,884.17	4,914.79	19,533.61
	972	17,358.93	18,610.34	18,357.45	18,514.58	72,841.30
	973	8,537.23	8,739.51	8,465.55	8,721.94	34,464.23
	974	6,683.65	6,741.94	6,648.55	6,618.30	26,692.44
	975	2,114.95	2,181.56	2,224.05	2,427.32	8,947.88
	976	486.81	420.59	437.81	457.61	1,802.82
	977	3,610.31	3,737.36	3,744.50	3,718.32	14,810.49
	978	826.36	939.42	854.07	933.33	3,553.18

	979	360.11	310.05	347.04	402.15	1,419.35
TOTAL		57,185.60	59,272.39	58,568.96	59,537.24	234,564.19

STATE: PENNSYLVANIA

ZIP CODE		QUARTER 1	QUARTER 2	QUARTER 3	QUARTER 4	TOTAL GRAMS
	150	6,286.33	6,292.89	6,342.71	6,392.33	25,314.26
	151	5,093.39	4,999.04	5,115.10	5,217.30	20,424.83
	152	10,722.99	10,592.92	10,789.04	11,054.52	43,159.47
	153	2,383.27	2,429.01	2,455.01	2,470.31	9,737.60
	154	1,981.13	1,879.02	1,785.30	1,925.75	7,571.20
	155	894.99	862.51	892.49	972.73	3,622.72
	156	4,535.84	4,380.69	4,407.35	4,442.83	17,766.71
	157	1,479.39	1,269.26	1,326.14	1,442.14	5,516.93
	158	1,272.77	1,273.05	1,277.49	1,247.72	5,071.03
	159	1,395.40	1,373.64	1,399.31	1,445.92	5,614.27
	160	3,090.29	3,108.98	3,099.60	3,112.39	12,411.26
	161	2,468.82	2,376.40	2,529.17	2,728.85	10,103.24
	162	964.99	916.22	880.24	955.62	3,717.07
	163	2,055.05	2,017.23	1,881.06	1,978.75	7,932.09
	164	964.97	943.9	980.54	1,036.47	3,925.88
	165	4,014.10	4,090.38	4,092.97	4,047.28	16,244.73
	166	2,734.21	2,702.93	2,715.19	2,786.90	10,939.23
	167	609.27	574.59	589.98	589.46	2,363.30
	168	2,883.91	2,773.32	2,750.02	2,815.66	11,222.91
	169	467.32	467.82	469.61	480.79	1,885.54
	170	11,695.29	11,829.93	11,864.55	11,808.30	47,198.07
	171	2,868.87	2,955.66	3,030.41	3,090.65	11,945.59
	172	3,106.65	3,102.76	3,227.46	3,228.57	12,665.44
	173	5,274.53	5,339.07	5,189.58	5,404.58	21,207.76
	174	4,170.01	4,205.22	4,132.61	4,291.17	16,799.01
	175	3,720.63	3,610.40	3,782.78	3,698.35	14,812.16
	176	3,964.30	3,960.05	4,040.38	4,139.95	16,104.68
	177	2,099.91	2,029.04	2,006.73	2,162.58	8,298.26
	178	2,596.46	2,668.49	2,617.31	2,743.48	10,625.74
	179	962.74	945.43	905.74	955.84	3,769.75
	180	5,581.76	5,639.63	5,613.68	5,811.15	22,646.22

181	2,981.36	3,027.62	2,866.73	3,015.86	11,891.57
182	932.5	1,008.12	938.18	1,024.51	3,903.31
183	1,324.87	1,298.86	1,422.59	1,501.55	5,547.87
184	2,091.83	2,352.68	2,274.44	2,423.51	9,142.46
185	3,665.84	3,633.99	3,800	4,013.76	15,113.59
186	1,757.25	1,777.49	1,761.16	1,893.87	7,189.77
187	16,263.25	18,137.16	15,896.68	17,945.60	68,242.69
188	657.78	630.93	574.36	599.41	2,462.48
189	7,281.32	7,384.59	7,629.80	7,678.36	29,974.07
190	22,526.70	23,093.92	23,116.35	23,352.81	92,089.78
191	20,498.52	20,729.84	19,846.22	20,842.10	81,916.68
193	10,193.07	10,256.68	10,220.75	10,266.77	40,937.27
194	11,137.20	11,400.16	11,433.18	11,631.63	45,602.17
195	1,907.18	1,802.08	1,866.09	1,887.42	7,462.77
196	2,945.43	3,103.97	3,003.17	3,189	12,241.57
TOTAL	208,503.68	211,247.57	208,839.25	215,744.50	844,335

STATE: PUERTO RICO

ZIP CODE	QUARTER 1	QUARTER 2	QUARTER 3	QUARTER 4	TOTAL GRAMS
6	2,036.97	1,883.50	1,884.21	1,821.97	7,626.65
7	2,215.78	2,035.05	1,758.58	1,764.10	7,773.51
9	4,823.87	4,523.81	4,202.79	4,164.60	17,715.07
TOTAL	9,076.62	8,442.36	7,845.58	7,750.67	33,115.23

STATE: RHODE ISLAND

ZIP CODE	QUARTER 1	QUARTER 2	QUARTER 3	QUARTER 4	TOTAL GRAMS
28	22,511.48	23,256.34	23,363.81	23,812.45	92,944.08
29	12,873.29	13,224.85	13,490.61	14,006.29	53,595.04
TOTAL	35,384.77	36,481.19	36,854.42	37,818.74	146,539.12

STATE: SOUTH CAROLINA

ZIP CODE	QUARTER 1	QUARTER 2	QUARTER 3	QUARTER 4	TOTAL GRAMS
290	12,157.26	11,987.72	11,757.93	12,317.68	48,220.59
291	9,839.22	9,812.74	9,574.48	9,939.63	39,166.07

	292	10,443.79	10,172.93	10,163.75	10,346.59	41,127.06
	293	8,530.70	8,451.77	8,245.80	8,683.32	33,911.59
	294	33,171.24	32,863.04	32,244.56	34,065.76	132,344.60
	295	18,135.98	17,838.95	18,024.01	18,110.94	72,109.88
	296	28,183.57	28,062.82	27,985.60	29,606.06	113,838.05
	297	7,620.71	7,439.27	7,809.08	8,085.47	30,954.53
	298	4,826.42	4,508.63	4,191.87	4,577.69	18,104.61
	299	3,752.47	3,816.27	3,939.56	3,907.65	15,415.95
TOTAL		136,661.36	134,954.14	133,936.64	139,640.79	545,192.93

STATE: SOUTH DAKOTA

ZIP CODE		QUARTER 1	QUARTER 2	QUARTER 3	QUARTER 4	TOTAL GRAMS
	570	2,154.92	2,065.66	2,033.34	2,121.19	8,375.11
	571	9,124.64	8,230.04	7,772.28	8,240.94	33,367.90
	572	641.09	605.57	643.44	648.48	2,538.58
	573	604.76	637.5	656.83	609.2	2,508.29
	574	659.33	657.28	727.14	707.86	2,751.61
	575	244.23	255.81	252.02	282.3	1,034.36
	576	53.14	53.1	42.28	55.12	203.64
	577	2,501.35	2,410.86	2,528.44	2,592.09	10,032.74
TOTAL		15,983.46	14,915.82	14,655.77	15,257.18	60,812.23

STATE: TENNESSEE

ZIP CODE		QUARTER 1	QUARTER 2	QUARTER 3	QUARTER 4	TOTAL GRAMS
	370	19,914.15	20,446.43	20,113.64	21,212.23	81,686.45
	371	12,690.80	12,666.69	12,678.29	12,972.62	51,008.40
	372	13,795.91	14,113.90	14,207.19	14,692.42	56,809.42
	373	11,994.58	12,025.25	11,804.24	12,194.45	48,018.52
	374	8,955.75	8,882.51	8,749.13	9,356.93	35,944.32
	376	4,034.02	3,948.45	4,020.91	4,234.17	16,237.55
	377	4,367.01	4,456.34	4,381.72	4,506.26	17,711.33
	378	6,661.64	6,643.52	6,808.64	7,077.53	27,191.33
	379	11,142.59	10,967.09	11,197.71	11,645.90	44,953.29
	380	9,864.75	9,387.08	9,541.94	10,031.19	38,824.96
	381	11,785.36	11,673.54	11,744.76	12,407.81	47,611.47

	382	3,312.02	3,217.44	3,223.76	3,205.07	12,958.29
	383	4,753.89	4,460.62	4,615.68	4,934.45	18,764.64
	384	3,479.16	3,450.80	3,370.36	3,484.59	13,784.91
	385	2,269.08	2,145.98	2,261.77	2,257.04	8,933.87
TOTAL		129,020.71	128,485.64	128,719.74	134,212.66	520,438.75

STATE: TEXAS

ZIP CODE	QUARTER 1	QUARTER 2	QUARTER 3	QUARTER 4	TOTAL GRAMS
750	52,977.30	53,821.17	52,663.51	52,865.68	212,327.66
751	8,796.92	8,947.83	8,878.34	9,416.20	36,039.29
752	25,195.07	25,973.74	25,521.70	26,973.85	103,664.36
753	16.46	23.47	31.01	34.92	105.86
754	3,944.85	4,002.35	4,046.93	4,393.16	16,387.29
755	1,189.32	1,219.66	1,167.56	1,261.41	4,837.95
756	3,681.08	3,728.43	3,579.84	3,796.61	14,785.96
757	4,673.25	4,869.17	4,698.22	4,980.23	19,220.87
758	848.54	775.87	766.17	894	3,284.58
759	2,483.80	2,423.55	2,507.10	2,477.30	9,891.75
760	22,617.33	22,706.25	23,081.17	23,967.62	92,372.37
761	13,578.94	13,772.70	13,737.87	14,827.14	55,916.65
762	11,303.11	11,333.85	11,402.01	11,950.33	45,989.30
763	3,172.93	3,311.83	3,379.12	3,449.63	13,313.51
764	1,388.39	1,310.96	1,365.69	1,415.35	5,480.39
765	5,730.29	5,722.54	6,196.89	5,541.52	23,191.24
766	1,121.36	1,109.95	1,080.50	1,192.41	4,504.22
767	3,475.59	3,373.16	3,275.31	3,610.29	13,734.35
768	778.49	800.96	804.68	820	3,204.13
769	1,629.92	1,654.43	1,622.73	1,832.75	6,739.83
770	38,848.59	39,521.68	38,044.50	41,628.73	158,043.50
772	38.16	49.11	32.22	49.89	169.38
773	28,335.85	28,543.69	28,415.73	30,315.97	115,611.24
774	20,162.21	20,297.03	19,828.38	21,359.97	81,647.59
775	22,757.20	22,986.66	22,438.01	23,847.21	92,029.08
776	4,828.73	4,819.71	4,778.33	5,054.03	19,480.80
777	2,512.50	2,517.69	2,426.06	2,621.17	10,077.42
778	6,977.41	6,735	6,715.97	7,131.18	27,559.56

	779	2,177.86	2,153.85	2,004.27	2,213.75	8,549.73
	780	4,581.36	4,756.95	4,649.12	5,136.69	19,124.12
	781	5,936.02	5,925.33	6,221.20	6,441.24	24,523.79
	782	26,025.30	25,617.27	25,924.24	27,682.14	105,248.95
	783	2,060.57	2,015.38	1,982.18	1,890.07	7,948.20
	784	4,859.77	4,635.57	4,660.40	4,900.94	19,056.68
	785	3,078.25	2,792.13	2,838.76	3,174.33	11,883.47
	786	16,825.95	16,889.88	17,093.83	18,197.40	69,007.06
	787	28,082.76	28,823.78	28,213.16	30,580.73	115,700.43
	788	629.59	627.81	706.57	615.04	2,579.01
	789	690.71	656.82	694.38	730.93	2,772.84
	790	903.12	888.58	858.18	899.4	3,549.28
	791	1,688.17	1,812.12	1,755.97	1,921.51	7,177.77
	792	70.29	71.69	83.07	81.15	306.2
	793	466.52	455.64	466.39	481.19	1,869.74
	794	4,592.37	4,558.65	4,591.74	4,817.78	18,560.54
	795	464.01	478.06	516.82	522.06	1,980.95
	796	2,159.51	2,162.05	2,092.64	2,220.52	8,634.72
	797	5,707.66	6,055.47	6,110.70	6,384.55	24,258.38
	798	248	217.44	201.4	215.05	881.89
	799	3,159.62	3,078.47	3,566.71	2,831.02	12,635.82
TOTAL		407,471	411,025.38	407,717.28	429,646.04	1,655,859.70

STATE: UTAH

ZIP CODE		QUARTER 1	QUARTER 2	QUARTER 3	QUARTER 4	TOTAL GRAMS
	840	40,080.76	41,363.65	41,678.84	43,562.33	166,685.58
	841	15,987.90	16,400.75	16,333.30	16,999.48	65,721.43
	843	4,110.92	4,286.18	4,329.52	4,590.22	17,316.84
	844	3,671.30	3,827.05	3,777.10	4,159.28	15,434.73
	845	726.09	593.64	637.05	672.19	2,628.97
	846	8,307.69	8,328.13	8,538.70	8,860.93	34,035.45
	847	6,328.21	6,229.66	6,454.22	6,851.31	25,863.40
TOTAL		79,212.87	81,029.06	81,748.73	85,695.74	327,686.40

STATE: VERMONT

ZIP CODE	QUARTER 1	QUARTER 2	QUARTER 3	QUARTER 4	TOTAL GRAMS
50	635.55	578.1	497.19	590.97	2,301.81
51	542.27	490.95	570.95	524.63	2,128.80
52	712.57	709.25	692.82	723.12	2,837.76
53	1,614.01	1,609.38	1,644.18	1,731.01	6,598.58
54	3,253.72	3,338.83	3,274.56	3,401.11	13,268.22
56	1,636.76	1,638.93	1,650.35	1,659.53	6,585.57
57	840.5	919.94	880	916.78	3,557.22
58	564.64	623.97	570.99	651.86	2,411.46
TOTAL	9,800.02	9,909.35	9,781.04	10,199.01	39,689.42

STATE: VIRGIN ISLANDS

ZIP CODE	QUARTER 1	QUARTER 2	QUARTER 3	QUARTER 4	TOTAL GRAMS
8	368.82	364.19	251.15	233.08	1,217.24
TOTAL	368.82	364.19	251.15	233.08	1,217.24

STATE: VIRGINIA

ZIP CODE	QUARTER 1	QUARTER 2	QUARTER 3	QUARTER 4	TOTAL GRAMS
201	18,061.29	17,971.69	17,923.90	18,716.86	72,673.74
220	10,528.57	10,214.45	10,448.90	10,454.96	41,646.88
221	6,912.54	7,062.72	6,868.53	7,331.99	28,175.78
222	4,791.73	5,029.40	4,714.86	4,629.21	19,165.20
223	3,933.01	4,032.93	4,011.68	4,275.69	16,253.31
224	4,542.77	4,681.80	4,481.74	4,700.12	18,406.43
225	2,059.65	2,106.04	1,978.13	2,128.30	8,272.12
226	3,036.79	3,128.81	3,094.84	3,327.86	12,588.30
227	1,083.35	1,052.26	1,080.33	1,065.64	4,281.58
228	3,183.47	3,097.28	2,897.85	3,154.29	12,332.89
229	6,345.28	6,249.85	6,318.83	6,503.63	25,417.59
230	4,907.31	4,799.23	4,802.70	5,076	19,585.24
231	11,468.86	11,681.76	11,567.48	12,200.32	46,918.42
232	15,970.95	16,035.99	16,040.45	16,221.24	64,268.63
233	9,643.61	9,850.78	9,844.15	9,987.53	39,326.07
234	24,154.23	24,390.20	24,181.45	24,883.07	97,608.95

	235	6,124.39	6,373.08	6,014.14	6,313.24	24,824.85
	236	9,406.51	9,584.18	10,035.86	9,236.87	38,263.42
	237	4,110.91	4,169.09	3,971.07	4,184.49	16,435.56
	238	6,597.99	6,583.20	6,295.86	6,774.44	26,251.49
	239	1,029.59	1,027.78	1,050.80	1,032.35	4,140.52
	240	5,321.29	5,428.17	5,202.75	5,498.57	21,450.78
	241	2,753.62	2,723.67	2,620.40	2,783.92	10,881.61
	242	1,437.57	1,304.17	1,385.50	1,389.63	5,516.87
	243	906.76	886.96	830.7	849.8	3,474.22
	244	3,251.13	3,309.54	3,211.31	3,448.53	13,220.51
	245	5,239.40	5,229.88	5,329.16	5,409.06	21,207.50
	246	356.39	357.62	392.01	388.71	1,494.73
TOTAL		177,158.96	178,362.53	176,595.38	181,966.32	714,083.19

STATE: WASHINGTON

ZIP CODE		QUARTER 1	QUARTER 2	QUARTER 3	QUARTER 4	TOTAL GRAMS
	980	26,199.11	26,917.52	26,847.86	26,763.73	106,728.22
	981	17,081.65	17,494.90	17,264.76	17,933.80	69,775.11
	982	15,963.42	16,382.22	16,092.72	16,609.58	65,047.94
	983	10,153.15	10,528.89	10,394.54	10,587.39	41,663.97
	984	7,189.03	7,340.15	7,140.58	6,990.34	28,660.10
	985	5,105.72	5,292.46	5,248.30	5,368.19	21,014.67
	986	8,020.81	8,270.09	8,090.06	8,099.95	32,480.91
	988	1,818.96	1,749.07	1,800.57	1,894.11	7,262.71
	989	2,520.28	2,516.04	2,357.39	2,426.01	9,819.72
	990	897.67	929.36	979.18	1,000.32	3,806.53
	991	1,174.44	1,021.36	1,079.61	1,092.36	4,367.77
	992	5,401.87	5,452.08	5,430.31	5,717.49	22,001.75
	993	5,483.30	5,676.46	5,339.66	5,530.12	22,029.54
	994	186.99	176.27	182.49	196.41	742.16
TOTAL		107,196.40	109,746.87	108,248.03	110,209.80	435,401.10

STATE: WEST VIRGINIA

ZIP CODE		QUARTER 1	QUARTER 2	QUARTER 3	QUARTER 4	TOTAL GRAMS
	247	283.55	272.89	309.84	292	1,158.28

	248	221.17	187.41	219.48	201.31	829.37
	249	411.88	436.06	426.43	394.08	1,668.45
	250	907.09	845.37	805.15	832.62	3,390.23
	251	676.9	670.38	702.5	713.54	2,763.32
	252	540.04	557.15	503.51	470.89	2,071.59
	253	3,663.21	3,640.36	3,707.73	3,485.15	14,496.45
	254	2,425.50	2,449.94	2,474.69	2,579.58	9,929.71
	255	2,397.40	2,230.80	2,227.81	2,453.65	9,309.66
	256	266.95	273.59	246.71	261.15	1,048.40
	257	1,749.10	1,661.37	1,769.47	1,784.52	6,964.46
	258	1,225.69	1,243.03	1,129.56	1,264.32	4,862.60
	259	329.28	265.6	276.74	260.2	1,131.82
	260	1,452.94	1,427.92	1,395.42	1,397.80	5,674.08
	261	2,315.91	2,288.21	2,206.75	2,217.07	9,027.94
	262	295.14	278.38	313.09	329.37	1,215.98
	263	954.76	960.65	996.3	1,004.76	3,916.47
	264	268.85	239.79	215.18	271.04	994.86
	265	2,842.17	2,705.95	2,660.63	2,752.79	10,961.54
	266	137.27	130.71	131.52	123.78	523.28
	267	256.58	276.47	281.04	290.63	1,104.72
	268	233.96	225.05	224.04	246.54	929.59
TOTAL		23,855.34	23,267.08	23,223.59	23,626.79	93,972.80

STATE:	WISCONSIN					
ZIP CODE		QUARTER 1	QUARTER 2	QUARTER 3	QUARTER 4	TOTAL GRAMS
	530	14,494.54	14,076.99	14,146.19	14,728.04	57,445.76
	531	15,111.66	14,688.09	14,682.48	15,265.22	59,747.45
	532	13,660.07	13,381.61	13,086.07	13,621.37	53,749.12
	534	4,307.56	4,086.75	3,988.51	4,108.37	16,491.19
	535	10,047.03	9,848.42	9,735.87	10,208.38	39,839.70
	537	8,599.88	8,268.59	8,065.53	8,300.65	33,234.65
	538	1,210.99	1,127.64	1,070.99	1,145.22	4,554.84
	539	3,680.43	3,490.13	3,510.22	3,600.40	14,281.18
	540	2,619.15	2,568.49	2,571.74	2,498.43	10,257.81
	541	6,814.01	6,845.83	6,814.58	6,861.29	27,335.71
	542	2,748.42	2,688.36	2,745.60	2,718.71	10,901.09

	543	8,151.85	7,953.90	8,163.90	8,352.14	32,621.79
	544	8,807.30	8,498.18	8,326.06	8,338.27	33,969.81
	545	2,367.05	2,264.78	2,280.49	2,185.23	9,097.55
	546	4,561.95	4,500.34	4,274.53	4,469.03	17,805.85
	547	4,486.56	4,533.37	4,197.07	4,503.49	17,720.49
	548	2,525.77	2,396.82	2,396.23	2,355.12	9,673.94
	549	15,792.30	15,395.98	15,411.44	15,505.34	62,105.06
TOTAL		129,986.52	126,614.27	125,467.50	128,764.70	510,832.99

STATE: WYOMING

ZIP CODE		QUARTER 1	QUARTER 2	QUARTER 3	QUARTER 4	TOTAL GRAMS
	820	1,404.46	1,354.91	1,313.54	1,417.19	5,490.10
	822	87.49	80.74	79.08	79.35	326.66
	823	122.86	116.28	131.73	83.68	454.55
	824	899.32	845.86	803.52	809.94	3,358.64
	825	306.28	310.44	306.1	294.89	1,217.71
	826	1,063.62	970.14	937.79	999.83	3,971.38
	827	490.5	456.51	529.46	489.21	1,965.68
	828	609.67	564.28	670.01	598.46	2,442.42
	829	1,024.68	987.22	992.08	1,071.36	4,075.34
	830	469.62	436.05	533.86	511.34	1,950.87
	831	131.36	109.68	113.77	127.79	482.6
TOTAL		6,609.86	6,232.11	6,410.94	6,483.04	25,735.95

DRUG CODE: 1105B DRUG NAME: DL-METHAMPHETAMINE RACEMIC BASE
STATE: ALABAMA

ZIP CODE		QUARTER 1	QUARTER 2	QUARTER 3	QUARTER 4	TOTAL GRAMS
	350	1.61	2.41	0.8	1.21	6.03
	351	4.42	5.22	3.62	4.82	18.08
	352	5.22	4.42	4.42	3.62	17.68
	354	0	1.61	1.21	1.21	4.03
	355	3.62	4.02	8.04	4.42	20.1
	356	0	0.8	2.81	0	3.61
	358	0.4	0	0	0	0.4
	359	0	0	0.4	0.4	0.8

	361	1.21	0.8	0.8	0	2.81
	362	4.42	4.02	4.42	4.42	17.28
	365	0.4	0	0	0	0.4
	366	1.21	1.21	1.21	1.21	4.84
TOTAL		22.51	24.51	27.73	21.31	96.06

STATE: ALASKA

ZIP CODE		QUARTER 1	QUARTER 2	QUARTER 3	QUARTER 4	TOTAL GRAMS
	995	0.8	3.21	2.41	0	6.42
	997	0	1.61	1.61	1.21	4.43
TOTAL		0.8	4.82	4.02	1.21	10.85

STATE: ARIZONA

ZIP CODE		QUARTER 1	QUARTER 2	QUARTER 3	QUARTER 4	TOTAL GRAMS
	850	2.81	2.01	2.01	2.01	8.84
	852	21.7	25.32	22.1	30.54	99.66
	853	5.22	2.81	2.81	2.41	13.25
	855	2.01	1.21	1.21	1.21	5.64
	856	2.41	0.8	0.4	2.41	6.02
	857	0	0	0	0.8	0.8
TOTAL		34.15	32.15	28.53	39.38	134.21

STATE: ARKANSAS

ZIP CODE		QUARTER 1	QUARTER 2	QUARTER 3	QUARTER 4	TOTAL GRAMS
	720	0.8	4.42	3.62	2.81	11.65
	721	2.01	1.61	0.4	0.4	4.42
	722	0.8	0.8	0.8	1.21	3.61
	727	1.61	0.4	2.01	1.61	5.63
TOTAL		5.22	7.23	6.83	6.03	25.31

STATE: CALIFORNIA

ZIP CODE		QUARTER 1	QUARTER 2	QUARTER 3	QUARTER 4	TOTAL GRAMS
	900	34.16	22.5	10.85	8.04	75.55

902	8.84	2.81	4.82	4.02	20.49
904	0	0.8	0	0	0.8
905	0.8	0	0	0	0.8
906	0	0.8	0	0	0.8
907	0	0	0	0.8	0.8
908	1.21	0.4	0.8	0.4	2.81
910	10.05	0.4	4.82	4.82	20.09
911	0.8	0	2.81	4.02	7.63
912	1.61	8.44	5.22	4.42	19.69
913	30.94	39.78	34.56	30.54	135.82
914	6.83	4.42	3.62	2.81	17.68
917	20.09	11.25	1.61	2.41	35.36
919	2.01	2.01	2.81	2.01	8.84
920	32.15	38.58	36.97	26.92	134.62
921	9.64	6.03	9.64	18.49	43.8
922	6.43	5.22	4.82	3.21	19.68
923	0	0	0	5.63	5.63
925	4.42	5.22	6.43	5.63	21.7
926	20.9	23.71	25.72	12.86	83.19
927	7.23	10.85	6.03	2.41	26.52
928	5.63	11.65	8.84	8.04	34.16
930	6.43	2.81	3.62	3.21	16.07
931	7.23	3.62	3.21	0.4	14.46
933	0	0	0	0.4	0.4
934	0	0.8	1.21	0.8	2.81
935	2.81	0.8	0.4	1.61	5.62
936	0.4	0	0	0	0.4
937	6.43	4.42	6.03	5.63	22.51
939	1.61	1.21	1.21	0.8	4.83
940	9.24	9.24	10.05	14.87	43.4
941	13.26	16.88	16.88	12.06	59.08
943	0	0.4	0	0	0.4
945	10.05	10.45	7.64	8.84	36.98
946	3.62	2.41	2.41	2.01	10.45
947	0.8	2.01	1.21	0	4.02
949	10.05	8.44	2.41	1.61	22.51
950	10.45	11.65	6.43	10.85	39.38

	951	12.46	12.46	14.47	15.27	54.66
	952	0	0.4	0	0	0.4
	953	3.21	3.21	2.81	2.81	12.04
	954	0	0.8	0.8	0.4	2
	955	1.21	0.8	1.21	1.21	4.43
	956	2.01	4.82	4.42	5.63	16.88
	957	2.01	0.8	2.41	2.41	7.63
	958	7.23	6.03	4.82	4.02	22.1
	959	6.03	10.85	6.43	6.83	30.14
	960	0.4	0.8	0.8	0.8	2.8
TOTAL		320.68	310.98	271.25	249.95	1,152.86

STATE: COLORADO

ZIP CODE		QUARTER 1	QUARTER 2	QUARTER 3	QUARTER 4	TOTAL GRAMS
	800	9.64	9.64	11.65	8.04	38.97
	801	15.67	20.49	13.26	21.3	70.72
	802	8.44	5.22	10.45	12.46	36.57
	803	0.4	0	0	0	0.4
	804	1.21	1.21	0.4	3.62	6.44
	805	4.42	6.03	6.03	2.41	18.89
	806	0	0.4	0	0.4	0.8
	809	3.21	6.83	2.81	2.81	15.66
	810	1.61	0.8	2.81	0.8	6.02
	811	0	0	0.4	0.4	0.8
	812	2.01	1.61	0.4	0.8	4.82
	813	0	0	0	1.21	1.21
	815	1.21	1.21	0.4	0	2.82
	816	1.21	0.8	1.21	3.21	6.43
TOTAL		49.03	54.24	49.82	57.46	210.55

STATE: CONNECTICUT

ZIP CODE		QUARTER 1	QUARTER 2	QUARTER 3	QUARTER 4	TOTAL GRAMS
	60	4.42	6.03	5.22	6.03	21.7
	61	2.81	2.81	4.02	2.41	12.05
	64	0.8	1.61	0	0	2.41

	65	4.42	6.03	1.21	3.21	14.87
	66	0	0	0.4	0	0.4
	67	1.21	0.4	2.41	4.82	8.84
	68	6.43	5.63	5.63	6.03	23.72
	69	0.4	2.01	1.21	2.01	5.63
TOTAL		20.49	24.52	20.1	24.51	89.62

STATE: DELAWARE

ZIP CODE	QUARTER 1	QUARTER 2	QUARTER 3	QUARTER 4	TOTAL GRAMS
197	0	0	0.4	0.8	1.2
198	1.21	2.01	3.21	2.01	8.44
199	1.21	1.61	1.21	1.61	5.64
TOTAL	2.42	3.62	4.82	4.42	15.28

STATE: DISTRICT OF COLUMBIA

ZIP CODE	QUARTER 1	QUARTER 2	QUARTER 3	QUARTER 4	TOTAL GRAMS
200	4.02	2.01	0	0.8	6.83
TOTAL	4.02	2.01	0	0.8	6.83

STATE: FLORIDA

ZIP CODE	QUARTER 1	QUARTER 2	QUARTER 3	QUARTER 4	TOTAL GRAMS
321	0	0	0.4	1.61	2.01
322	3.62	6.03	4.42	2.01	16.08
323	0.8	0.4	0	0	1.2
324	0	0.4	1.61	0	2.01
325	2.81	2.41	3.62	1.61	10.45
326	2.81	2.81	1.61	1.21	8.44
327	1.61	2.01	2.81	2.41	8.84
328	0	0	3.21	2.01	5.22
329	1.61	2.41	1.61	0.8	6.43
330	0.8	0	2.41	3.21	6.42
331	1.61	3.21	0	2.01	6.83
333	1.21	3.21	4.42	2.41	11.25
334	1.61	2.81	0.4	0.8	5.62

	335	1.21	0.4	0.4	0.4	2.41
	336	0.8	0	0	0	0.8
	337	0.8	0.8	0	0.4	2
	338	0	4.42	1.61	3.21	9.24
	339	2.01	4.02	3.62	2.41	12.06
	341	0.4	0	0.4	1.21	2.01
	342	2.81	4.02	2.41	2.41	11.65
	346	0.4	0	1.61	1.61	3.62
	347	1.21	0	0	0	1.21
TOTAL		28.13	39.36	36.57	31.74	135.8

STATE: GEORGIA

ZIP CODE		QUARTER 1	QUARTER 2	QUARTER 3	QUARTER 4	TOTAL GRAMS
	300	5.22	4.42	5.22	6.83	21.69
	301	6.03	4.42	4.82	6.83	22.1
	303	6.43	5.22	2.81	2.41	16.87
	304	2.01	2.01	1.61	1.61	7.24
	305	1.61	0	0	0	1.61
	306	0.8	0.4	0.8	0	2
	309	3.21	2.81	0	0	6.02
	310	2.81	1.21	0	0	4.02
	313	1.21	1.21	0.8	2.41	5.63
	315	2.01	2.81	0	0	4.82
	316	0.4	0	0.4	0	0.8
	317	0.4	0	0.4	0.4	1.2
	398	0	0	0.8	0	0.8
TOTAL		32.14	24.51	17.66	20.49	94.8

STATE: HAWAII

ZIP CODE		QUARTER 1	QUARTER 2	QUARTER 3	QUARTER 4	TOTAL GRAMS
	967	3.21	5.63	0.8	0.4	10.04
	968	2.01	1.21	3.21	4.02	10.45
TOTAL		5.22	6.84	4.01	4.42	20.49

STATE: IDAHO

ZIP CODE	QUARTER 1	QUARTER 2	QUARTER 3	QUARTER 4	TOTAL GRAMS
832	0	0	0.8	0	0.8
833	0.8	0	0	0	0.8
834	0.4	0	0.8	0.4	1.6
836	0.4	2.41	5.63	7.64	16.08
837	1.61	0.8	2.81	1.61	6.83
838	4.42	3.62	0.4	0.4	8.84
TOTAL	7.63	6.83	10.44	10.05	34.95

STATE: ILLINOIS

ZIP CODE	QUARTER 1	QUARTER 2	QUARTER 3	QUARTER 4	TOTAL GRAMS
600	20.49	21.3	16.48	20.9	79.17
601	4.42	4.42	4.82	4.82	18.48
602	0	0	0.4	0	0.4
603	0	0	1.21	0	1.21
604	1.61	2.41	2.41	4.42	10.85
605	19.29	16.07	17.68	18.49	71.53
606	11.25	6.83	10.05	6.43	34.56
607	0.4	0.4	0	0	0.8
611	1.21	0	1.21	0.8	3.22
612	0	0.8	2.41	0	3.21
614	1.21	2.01	1.21	1.21	5.64
615	0	0	0	0.8	0.8
617	0	0	0	0.4	0.4
618	0	0.4	0	0	0.4
620	0.4	0.4	1.61	0	2.41
623	0	0.4	0	0	0.4
624	0	0	0.8	0.4	1.2
627	3.62	2.81	3.21	3.62	13.26
629	0	0	0	0.4	0.4
TOTAL	63.9	58.25	63.5	62.69	248.34

STATE: INDIANA

ZIP CODE	QUARTER 1	QUARTER 2	QUARTER 3	QUARTER 4	TOTAL GRAMS

	460	9.24	6.43	5.22	7.23	28.12
	462	2.41	1.21	0.4	0	4.02
	463	0	0	0.8	1.21	2.01
	469	7.23	9.64	14.47	6.43	37.77
	471	2.81	2.41	2.81	1.21	9.24
	474	6.43	6.03	4.82	1.61	18.89
	477	0.8	0.4	1.21	0.4	2.81
	478	0.8	0	0	0	0.8
TOTAL		29.72	26.12	29.73	18.09	103.66

STATE: IOWA

ZIP CODE		QUARTER 1	QUARTER 2	QUARTER 3	QUARTER 4	TOTAL GRAMS
	500	0	0	1.21	1.61	2.82
	501	0	0.4	0	0	0.4
	502	4.02	6.43	7.64	8.04	26.13
	503	2.01	2.81	1.61	2.41	8.84
	504	3.21	4.02	3.62	5.22	16.07
	505	0	0	0.4	0.4	0.8
	506	0.4	0.4	0	0	0.8
	515	1.61	1.61	2.41	0.8	6.43
	516	1.61	1.21	0	0.8	3.62
	520	0.4	0	0	0	0.4
	528	1.61	2.01	2.01	2.01	7.64
TOTAL		14.87	18.89	18.9	21.29	73.95

STATE: KANSAS

ZIP CODE		QUARTER 1	QUARTER 2	QUARTER 3	QUARTER 4	TOTAL GRAMS
	660	3.21	4.42	3.62	3.62	14.87
	661	0	0.8	0	0	0.8
	665	1.61	1.61	1.21	1.61	6.04
	666	0	0	0	1.21	1.21
	670	0	0	0	0.4	0.4
	672	6.03	5.63	3.21	3.62	18.49
TOTAL		10.85	12.46	8.04	10.46	41.81

STATE: KENTUCKY

ZIP CODE	QUARTER 1	QUARTER 2	QUARTER 3	QUARTER 4	TOTAL GRAMS
400	0.4	0	0	0	0.4
402	2.01	0.4	0.4	0	2.81
403	2.81	2.01	3.21	1.61	9.64
405	0	2.41	2.81	5.22	10.44
406	0	0	0.8	0.4	1.2
408	2.41	1.21	0	0	3.62
410	0.4	0.4	0	0.4	1.2
417	0.4	0.4	0	0	0.8
420	0.8	0.8	0	1.21	2.81
424	1.61	1.61	2.01	0	5.23
425	0	0.8	0.4	0.4	1.6
TOTAL	10.84	10.04	9.63	9.24	39.75

STATE: LOUISIANA

ZIP CODE	QUARTER 1	QUARTER 2	QUARTER 3	QUARTER 4	TOTAL GRAMS
700	0	0.4	0	0	0.4
701	0.4	0	0	0	0.4
703	1.61	2.41	2.81	3.21	10.04
704	2.01	1.61	2.41	1.21	7.24
705	0.4	0	0	0	0.4
706	1.21	0.4	1.61	0.8	4.02
707	1.61	2.41	0.8	2.01	6.83
708	2.41	2.81	0.8	0	6.02
710	0.8	0	0	0	0.8
711	0.8	2.41	2.41	2.81	8.43
712	0	0.4	0.4	0	0.8
713	2.81	2.01	2.81	2.01	9.64
TOTAL	14.06	14.86	14.05	12.05	55.02

STATE: MAINE

ZIP CODE	QUARTER 1	QUARTER 2	QUARTER 3	QUARTER 4	TOTAL GRAMS
39	0	0	0.4	0	0.4

	40	3.21	3.62	1.61	6.03	14.47
	41	0	0	0	2.01	2.01
	42	0	2.01	8.04	3.62	13.67
	48	0	1.21	0.4	1.21	2.82
TOTAL		3.21	6.84	10.45	12.87	33.37

STATE:	MARYLAND				
ZIP CODE	QUARTER 1	QUARTER 2	QUARTER 3	QUARTER 4	TOTAL GRAMS
206	0	0.4	0	0	0.4
207	0	0	0.4	0.8	1.2
208	11.25	7.23	8.44	8.84	35.76
210	10.45	7.23	7.23	7.64	32.55
211	8.84	8.84	11.65	6.03	35.36
212	9.64	18.89	8.04	15.67	52.24
214	0.4	0	0.8	0	1.2
217	0.8	2.01	1.61	1.21	5.63
TOTAL	41.38	44.6	38.17	40.19	164.34

STATE:	MASSACHUSETTS				
ZIP CODE	QUARTER 1	QUARTER 2	QUARTER 3	QUARTER 4	TOTAL GRAMS
10	19.29	18.08	17.68	16.48	71.53
12	2.41	2.01	2.41	1.61	8.44
14	3.21	2.81	2.01	0.8	8.83
16	0	0	0	1.21	1.21
17	3.62	3.21	3.21	4.02	14.06
18	2.81	2.81	4.42	6.43	16.47
19	2.81	4.42	4.02	2.81	14.06
20	0	0.8	0	0	0.8
21	5.63	4.02	4.42	2.81	16.88
23	0.8	0.4	0.8	0.4	2.4
24	2.81	2.01	1.61	1.21	7.64
27	1.21	0.8	2.41	1.61	6.03
TOTAL	44.6	41.37	42.99	39.39	168.35

RETAIL DRUG DISTRIBUTION BY ZIP CODE WITHIN STATE BY GRAMS WT

STATE: MICHIGAN

ZIP CODE	QUARTER 1	QUARTER 2	QUARTER 3	QUARTER 4	TOTAL GRAMS
480	1.61	0.8	0.4	1.21	4.02
481	4.82	4.42	4.42	2.41	16.07
483	0.8	2.01	3.21	2.81	8.83
484	0	0.4	0	0	0.4
486	2.41	0	0	0	2.41
488	2.01	1.61	1.61	0.8	6.03
489	0	0	0	0.4	0.4
490	9.64	8.84	10.85	12.46	41.79
492	0	0	0	0.8	0.8
493	1.61	3.62	0	2.01	7.24
495	0	0.4	0.4	0.8	1.6
498	0	0.4	0.8	0.8	2
TOTAL	22.9	22.5	21.69	24.5	91.59

STATE: MINNESOTA

ZIP CODE	QUARTER 1	QUARTER 2	QUARTER 3	QUARTER 4	TOTAL GRAMS
550	0.8	0.4	5.22	3.62	10.04
551	13.26	12.06	5.63	8.04	38.99
553	3.62	5.22	7.64	5.63	22.11
554	27.73	34.96	18.08	14.47	95.24
557	1.21	4.02	2.41	4.42	12.06
559	21.7	24.91	19.69	19.29	85.59
560	2.41	2.41	1.21	1.61	7.64
562	2.81	0.4	1.61	0.8	5.62
563	1.21	0.8	0	0	2.01
565	2.41	2.01	2.41	1.21	8.04
566	1.21	0	0	0.8	2.01
TOTAL	78.37	87.19	63.9	59.89	289.35

STATE: MISSISSIPPI

ZIP CODE	QUARTER 1	QUARTER 2	QUARTER 3	QUARTER 4	TOTAL GRAMS
386	0	0	0	0.4	0.4

	391	0.4	0	0	0	0.4
	392	6.43	6.43	7.64	5.63	26.13
	394	0	0	0	0.4	0.4
	396	1.21	0.8	1.21	1.21	4.43
	397	0.4	0.8	0	0	1.2
TOTAL		8.44	8.03	8.85	7.64	32.96

STATE: MISSOURI

ZIP CODE		QUARTER 1	QUARTER 2	QUARTER 3	QUARTER 4	TOTAL GRAMS
	630	3.62	2.41	2.41	3.21	11.65
	631	34.16	49.43	6.43	48.62	138.64
	634	0.8	1.21	0	2.01	4.02
	636	2.41	0	0	1.21	3.62
	639	1.61	1.21	1.61	1.61	6.04
	640	0.8	0.4	0.8	2.81	4.81
	641	1.61	1.61	2.01	1.21	6.44
	645	1.21	2.01	2.81	2.01	8.04
	652	1.21	2.41	0	0	3.62
	653	0.4	0.8	0	0	1.2
	655	0.4	0	0	0	0.4
	657	0.8	0	0	0	0.8
	658	0	0.8	0	0	0.8
TOTAL		49.03	62.29	16.07	62.69	190.08

STATE: MONTANA

ZIP CODE		QUARTER 1	QUARTER 2	QUARTER 3	QUARTER 4	TOTAL GRAMS
	593	0.4	0.4	0.4	0.4	1.6
	594	0.4	0	0	0	0.4
	597	0	0.4	0	0	0.4
	598	0.4	0	0	0	0.4
TOTAL		1.2	0.8	0.4	0.4	2.8

STATE: NEBRASKA

ZIP CODE		QUARTER 1	QUARTER 2	QUARTER 3	QUARTER 4	TOTAL GRAMS

	680	0.4	1.61	0	0	2.01
	681	4.82	6.03	5.63	5.63	22.11
	684	0	0	1.21	2.01	3.22
	685	0	0.8	1.21	0.8	2.81
	687	0.8	0.4	0.8	0.8	2.8
TOTAL		6.02	8.84	8.85	9.24	32.95

STATE: NEVADA

ZIP CODE	QUARTER 1	QUARTER 2	QUARTER 3	QUARTER 4	TOTAL GRAMS
890	1.21	0.8	0	0	2.01
891	20.09	25.72	17.68	18.08	81.57
893	0.4	0.8	0.4	1.61	3.21
894	0	0.4	1.61	0	2.01
895	2.01	1.21	2.41	2.41	8.04
897	0	0	0	0.4	0.4
898	0	0.4	0	0	0.4
TOTAL	23.71	29.33	22.1	22.5	97.64

STATE: NEW HAMPSHIRE

ZIP CODE	QUARTER 1	QUARTER 2	QUARTER 3	QUARTER 4	TOTAL GRAMS
31	2.41	2.41	3.62	1.61	10.05
37	3.21	2.41	3.21	1.21	10.04
38	2.41	2.81	0	0.8	6.02
TOTAL	8.03	7.63	6.83	3.62	26.11

STATE: NEW JERSEY

ZIP CODE	QUARTER 1	QUARTER 2	QUARTER 3	QUARTER 4	TOTAL GRAMS
70	4.42	1.21	2.81	2.01	10.45
71	0	0	0	0.8	0.8
73	0.4	0	0	0	0.4
74	2.41	3.21	2.41	3.21	11.24
76	9.24	7.23	6.83	7.64	30.94
77	0	0	0	1.21	1.21

78	4.82	1.21	3.62	4.82	14.47
79	1.21	1.21	1.61	0.8	4.83
80	7.64	8.44	8.04	11.25	35.37
81	1.21	1.21	2.01	0.8	5.23
85	1.21	1.61	0.4	0.4	3.62
86	0	0	0.4	1.21	1.61
87	0.8	0	0	0	0.8
88	4.82	5.22	3.62	3.62	17.28
TOTAL	38.18	30.55	31.75	37.77	138.25

STATE: NEW MEXICO

ZIP CODE	QUARTER 1	QUARTER 2	QUARTER 3	QUARTER 4	TOTAL GRAMS
871	20.9	15.67	12.06	4.02	52.65
880	2.01	2.41	0	0.8	5.22
882	3.21	0.4	0.4	0	4.01
883	1.21	2.41	0.4	0	4.02
TOTAL	27.33	20.89	12.86	4.82	65.9

STATE: NEW YORK

ZIP CODE	QUARTER 1	QUARTER 2	QUARTER 3	QUARTER 4	TOTAL GRAMS
100	20.49	19.29	16.48	16.88	73.14
101	0.4	0.8	0	0.8	2
104	0.8	0.8	0.4	0.8	2.8
105	2.01	1.21	1.61	5.63	10.46
106	8.84	8.84	10.05	10.85	38.58
108	0	0	1.21	0	1.21
109	6.03	6.83	4.82	2.81	20.49
111	0	0.8	0	0	0.8
112	0.4	0.4	1.21	2.41	4.42
113	2.81	2.81	1.61	2.41	9.64
114	1.61	2.01	1.61	2.41	7.64
115	2.41	5.22	4.82	4.42	16.87
117	6.03	8.04	8.04	16.07	38.18
118	4.02	3.21	0	0	7.23
119	0.4	0	0	0	0.4

	120	2.01	1.21	1.61	2.01	6.84
	122	0	0.8	1.61	0	2.41
	123	3.62	3.62	3.21	2.01	12.46
	125	0.8	0.4	0	1.21	2.41
	127	0	0	0.4	1.61	2.01
	128	0.8	1.61	1.21	0.8	4.42
	130	0.8	1.21	2.41	2.81	7.23
	131	0	0.4	0	0	0.4
	134	0.8	0	0.4	0.4	1.6
	136	0	0	0	2.01	2.01
	139	0	0.8	0.8	0.8	2.4
	140	0.8	1.21	3.62	6.03	11.66
	141	1.61	3.21	2.81	3.21	10.84
	142	2.81	3.62	2.41	1.61	10.45
	144	2.41	0	0	0	2.41
	148	1.61	2.41	4.42	3.62	12.06
TOTAL		74.32	80.76	76.77	93.62	325.47

STATE:	NORTH CAROLINA					
ZIP CODE		QUARTER 1	QUARTER 2	QUARTER 3	QUARTER 4	TOTAL GRAMS
	270	0	0	0	0.4	0.4
	271	0	0	0.8	0.8	1.6
	272	7.64	6.43	1.61	2.41	18.09
	273	0	0.8	0.8	0	1.6
	274	0	0.4	0	0.8	1.2
	275	11.25	22.5	10.85	10.85	55.45
	276	6.83	6.43	10.05	8.44	31.75
	277	2.81	4.02	4.02	3.21	14.06
	278	0.8	1.21	0	0	2.01
	280	2.01	0.8	0.4	2.41	5.62
	281	7.23	4.42	6.03	6.43	24.11
	282	2.01	2.01	2.41	2.01	8.44
	283	3.21	4.02	2.81	2.41	12.45
	284	0	0	0	1.61	1.61
	285	0.4	0	0	0	0.4
	286	2.01	1.21	0	0	3.22

	287	0.4	1.21	1.21	0.8	3.62
	288	1.21	1.21	1.21	0	3.63
TOTAL		47.81	56.67	42.2	42.58	189.26

STATE: NORTH DAKOTA

ZIP CODE		QUARTER 1	QUARTER 2	QUARTER 3	QUARTER 4	TOTAL GRAMS
	581	1.21	0	0	0	1.21
	584	1.61	4.02	4.02	5.63	15.28
	587	0	3.62	2.01	1.61	7.24
TOTAL		2.82	7.64	6.03	7.24	23.73

STATE: OHIO

ZIP CODE		QUARTER 1	QUARTER 2	QUARTER 3	QUARTER 4	TOTAL GRAMS
	430	0	0	0.4	0.4	0.8
	431	0.4	1.21	1.61	1.21	4.43
	432	47.02	49.83	21.3	68.72	186.87
	434	0	0.8	0.8	0	1.6
	439	0	0.4	0	0	0.4
	440	0	0	0	0.4	0.4
	441	4.02	4.42	4.02	2.01	14.47
	442	0	0.4	0	0	0.4
	444	0	0.8	0	0.4	1.2
	445	2.01	3.21	2.41	2.01	9.64
	446	2.01	0.4	1.21	2.41	6.03
	447	1.21	1.61	1.61	1.21	5.64
	448	0	1.21	1.61	2.01	4.83
	450	9.64	9.24	8.84	6.83	34.55
	451	0.8	0	0	0	0.8
	452	2.01	0.8	2.01	1.61	6.43
	453	1.21	0.8	0.8	1.21	4.02
	454	5.22	4.42	2.01	2.81	14.46
	457	2.01	1.21	0	0	3.22
TOTAL		77.56	80.76	48.63	93.24	300.19

STATE: OKLAHOMA

ZIP CODE	QUARTER 1	QUARTER 2	QUARTER 3	QUARTER 4	TOTAL GRAMS
730	4.02	3.62	3.21	1.61	12.46
731	3.21	4.02	5.22	1.61	14.06
735	2.01	1.61	2.81	2.81	9.24
737	0	0	0	1.21	1.21
740	7.23	3.21	6.83	4.02	21.29
741	5.63	3.62	0.8	2.41	12.46
745	0	0.4	0.8	1.61	2.81
747	1.21	0	0.8	0.4	2.41
748	0.4	0	0	0.8	1.2
TOTAL	23.71	16.48	20.47	16.48	77.14

STATE: OREGON

ZIP CODE	QUARTER 1	QUARTER 2	QUARTER 3	QUARTER 4	TOTAL GRAMS
970	5.22	4.82	2.81	6.83	19.68
971	8.04	0.4	0	0.4	8.84
972	10.05	7.64	6.43	9.64	33.76
973	6.03	2.81	6.03	9.64	24.51
974	0.8	0.4	1.21	2.81	5.22
975	5.63	7.23	1.61	0	14.47
977	0.4	1.61	1.21	2.01	5.23
978	8.04	8.04	0	0	16.08
TOTAL	44.21	32.95	19.3	31.33	127.79

STATE: PENNSYLVANIA

ZIP CODE	QUARTER 1	QUARTER 2	QUARTER 3	QUARTER 4	TOTAL GRAMS
150	6.83	2.81	6.43	5.63	21.7
151	3.62	2.81	3.21	2.41	12.05
152	16.07	20.9	22.1	18.49	77.56
156	0.8	1.21	1.21	0.4	3.62
160	2.41	3.21	1.21	3.21	10.04
168	2.81	5.22	3.62	4.82	16.47
170	4.02	2.81	3.21	2.41	12.45

	171	0	1.21	1.21	4.02	6.44
	173	1.61	2.01	0.8	4.82	9.24
	174	0	0.4	1.61	0.8	2.81
	175	1.21	0.4	0	0	1.61
	176	0.8	0.8	0.4	0	2
	178	2.81	4.02	6.03	6.03	18.89
	180	1.61	0.4	0.4	3.21	5.62
	181	2.81	2.81	1.21	3.21	10.04
	183	1.61	0	0	0	1.61
	184	3.62	5.22	3.62	2.01	14.47
	185	4.42	4.02	2.81	0	11.25
	186	1.61	2.01	1.61	2.01	7.24
	187	32.95	45.81	32.55	30.54	141.85
	189	4.42	4.42	5.22	5.63	19.69
	190	8.84	7.64	5.22	5.22	26.92
	191	9.64	7.23	4.02	8.04	28.93
	193	1.21	2.81	4.02	3.62	11.66
	194	1.61	3.21	2.41	3.62	10.85
	195	0.4	0.8	1.21	0	2.41
	196	0.8	0	0.8	0	1.6
TOTAL		118.54	134.19	116.14	120.15	489.02

STATE: RHODE ISLAND

ZIP CODE		QUARTER 1	QUARTER 2	QUARTER 3	QUARTER 4	TOTAL GRAMS
	28	4.42	2.41	1.61	2.01	10.45
	29	0.4	3.21	1.21	1.61	6.43
TOTAL		4.82	5.62	2.82	3.62	16.88

STATE: SOUTH CAROLINA

ZIP CODE		QUARTER 1	QUARTER 2	QUARTER 3	QUARTER 4	TOTAL GRAMS
	290	0	0.4	1.61	0.8	2.81
	292	4.02	2.81	2.01	0.8	9.64
	293	5.22	0.8	0	0	6.02
	294	0.8	0.8	3.21	2.41	7.22

	295	2.41	2.81	5.63	2.81	13.66
	296	6.03	4.02	4.02	5.63	19.7
	297	0	0	0	0.8	0.8
	298	0	0.4	0	0	0.4
TOTAL		18.48	12.04	16.48	13.25	60.25

STATE:	SOUTH DAKOTA				
ZIP CODE	QUARTER 1	QUARTER 2	QUARTER 3	QUARTER 4	TOTAL GRAMS
571	11.25	18.89	16.07	13.66	59.87
573	0	0.8	0	0	0.8
574	0	0.4	0	0	0.4
577	4.42	2.01	1.21	0.8	8.44
TOTAL	15.67	22.1	17.28	14.46	69.51

STATE:	TENNESSEE				
ZIP CODE	QUARTER 1	QUARTER 2	QUARTER 3	QUARTER 4	TOTAL GRAMS
372	0	0.4	0	3.21	3.61
373	0	0	1.21	1.61	2.82
374	0.4	1.21	0.4	0.8	2.81
378	1.21	1.61	1.21	2.01	6.04
379	0.4	1.21	1.61	1.21	4.43
380	2.01	0.8	1.21	1.61	5.63
381	6.43	7.23	6.83	4.82	25.31
TOTAL	10.45	12.46	12.47	15.27	50.65

STATE:	TEXAS				
ZIP CODE	QUARTER 1	QUARTER 2	QUARTER 3	QUARTER 4	TOTAL GRAMS
750	24.91	27.73	24.11	24.51	101.26
751	0	0	0	0.8	0.8
752	17.68	18.49	13.66	15.27	65.1
754	2.01	2.41	3.21	2.81	10.44
755	0	0.8	0	0	0.8
756	1.21	2.81	0	0	4.02
757	2.01	0.8	0.8	0	3.61

	759	0	0.4	0	0	0.4
	760	11.25	6.43	5.63	11.25	34.56
	761	0	0.4	1.21	0.8	2.41
	762	4.42	1.21	0.4	3.21	9.24
	763	0	0.8	0	0	0.8
	764	0	1.61	0.8	0.8	3.21
	765	0	0	0	0.8	0.8
	766	0	0	0	1.21	1.21
	769	0	0.8	0.8	0.8	2.4
	770	9.24	6.43	2.41	5.63	23.71
	773	3.21	1.21	0.8	1.61	6.83
	774	3.62	6.03	3.62	4.42	17.69
	775	3.62	3.62	3.21	0.8	11.25
	778	0	0	0	1.21	1.21
	780	0.4	0	0.4	0	0.8
	781	1.61	4.02	2.41	0	8.04
	782	4.02	3.21	2.01	2.41	11.65
	783	0	0.4	0	0	0.4
	784	2.41	2.41	1.21	1.21	7.24
	785	4.42	2.81	6.83	3.62	17.68
	786	3.21	2.41	4.02	4.82	14.46
	787	8.04	10.45	10.05	9.64	38.18
	788	2.01	3.62	0	0	5.63
	789	2.01	0.8	0.8	1.61	5.22
	790	1.61	1.61	1.61	1.21	6.04
	797	3.62	2.41	1.21	2.01	9.25
	799	0	0	0	0.4	0.4
TOTAL		116.54	116.13	91.21	102.86	426.74

STATE:	UTAH				
ZIP CODE		QUARTER 1	QUARTER 2	QUARTER 3	QUARTER 4	TOTAL GRAMS
	840	6.83	6.83	7.23	4.82	25.71
	841	4.42	2.41	9.24	9.24	25.31
	846	6.43	5.63	4.42	5.63	22.11
	847	7.64	8.44	5.63	9.24	30.95
TOTAL		25.32	23.31	26.52	28.93	104.08

STATE:	VERMONT				
ZIP CODE	QUARTER 1	QUARTER 2	QUARTER 3	QUARTER 4	TOTAL GRAMS
56	0	1.21	1.21	2.01	4.43
57	0.8	0.8	0.8	0.4	2.8
TOTAL	0.8	2.01	2.01	2.41	7.23

STATE:	VIRGINIA				
ZIP CODE	QUARTER 1	QUARTER 2	QUARTER 3	QUARTER 4	TOTAL GRAMS
201	8.04	7.23	4.02	7.64	26.93
220	4.42	3.21	3.62	4.02	15.27
221	0	0.4	0	1.21	1.61
222	1.61	2.01	1.21	3.21	8.04
223	1.61	1.21	0.8	2.01	5.63
224	1.61	0	0.8	1.61	4.02
228	0	0.8	0	0	0.8
229	0.4	0	0	1.61	2.01
231	4.02	2.81	3.21	0.8	10.84
232	7.23	5.22	5.22	4.82	22.49
233	1.61	0	0	0	1.61
234	7.64	5.22	4.82	4.42	22.1
235	3.21	2.81	2.41	1.61	10.04
237	0	0.4	1.21	1.21	2.82
238	4.02	3.21	4.02	4.42	15.67
240	3.21	3.21	5.63	3.21	15.26
241	0	1.61	0.8	2.01	4.42
245	2.41	1.21	2.01	0	5.63
TOTAL	51.04	40.56	39.78	43.81	175.19

STATE:	WASHINGTON				
ZIP CODE	QUARTER 1	QUARTER 2	QUARTER 3	QUARTER 4	TOTAL GRAMS
980	28.13	33.76	36.57	37.37	135.83
981	10.05	15.67	8.84	12.86	47.42
982	15.27	13.26	18.49	15.27	62.29

	983	6.03	4.42	1.21	3.21	14.87
	984	2.81	2.01	2.01	2.41	9.24
	985	4.42	3.21	2.01	1.61	11.25
	986	16.07	14.06	14.06	20.49	64.68
	988	1.21	0.4	0	0	1.61
	989	1.61	1.61	0.8	0.8	4.82
	992	5.22	6.83	5.22	8.04	25.31
	993	0	0.4	2.01	1.61	4.02
TOTAL		90.82	95.63	91.22	103.67	381.34

STATE: WEST VIRGINIA

ZIP CODE	QUARTER 1	QUARTER 2	QUARTER 3	QUARTER 4	TOTAL GRAMS
254	0	0	1.21	0	1.21
258	0.8	3.21	0.4	2.01	6.42
260	2.41	1.21	1.61	0	5.23
265	0	0	2.41	1.21	3.62
TOTAL	3.21	4.42	5.63	3.22	16.48

STATE: WISCONSIN

ZIP CODE	QUARTER 1	QUARTER 2	QUARTER 3	QUARTER 4	TOTAL GRAMS
530	3.21	1.61	0	0	4.82
531	2.01	4.02	2.81	4.42	13.26
532	9.64	15.67	9.24	3.62	38.17
535	2.41	3.62	0	0.4	6.43
537	16.88	14.06	12.86	7.64	51.44
539	2.41	0.4	1.21	1.61	5.63
540	1.61	2.01	2.81	1.21	7.64
541	0	0.4	2.01	1.61	4.02
542	7.23	6.03	8.44	8.04	29.74
543	4.82	1.21	1.21	2.81	10.05
544	0	0	1.61	0	1.61
549	11.25	13.26	0	0	24.51
TOTAL	61.47	62.29	42.2	31.36	197.32

STATE: WYOMING

ZIP CODE	QUARTER 1	QUARTER 2	QUARTER 3	QUARTER 4	TOTAL GRAMS
820	2.81	4.82	3.21	2.41	13.25
826	1.61	1.61	1.61	0.8	5.63
829	1.61	0.8	0	0	2.41
TOTAL	6.03	7.23	4.82	3.21	21.29

DRUG CODE: 1105D DRUG NAME: D-METHAMPHETAMINE

STATE: ALABAMA

ZIP CODE	QUARTER 1	QUARTER 2	QUARTER 3	QUARTER 4	TOTAL GRAMS
350	0	0	2.01	0.8	2.81
351	0	0.8	2.01	0.8	3.61
356	0	0	2.81	5.62	8.43
TOTAL	0	0.8	6.83	7.22	14.85

STATE: ALASKA

ZIP CODE	QUARTER 1	QUARTER 2	QUARTER 3	QUARTER 4	TOTAL GRAMS
996	0	0	0.8	0	0.8
TOTAL	0	0	0.8	0	0.8

STATE: ARIZONA

ZIP CODE	QUARTER 1	QUARTER 2	QUARTER 3	QUARTER 4	TOTAL GRAMS
852	0	4.42	0.8	9.64	14.86
853	0	0	3.21	1.61	4.82
TOTAL	0	4.42	4.01	11.25	19.68

STATE: CALIFORNIA

ZIP CODE	QUARTER 1	QUARTER 2	QUARTER 3	QUARTER 4	TOTAL GRAMS
900	0	8.84	16.87	17.27	42.98
902	0	0	0.8	0.4	1.2
914	0	1.21	0.8	0.8	2.81
917	0	8.03	22.09	21.69	51.81
920	0	1.21	6.83	4.82	12.86

921	0	0.8	4.02	1.21	6.03
923	0	0	1.21	2.81	4.02
926	0	0	1.61	2.01	3.62
927	0	0.8	1.61	0.8	3.21
928	0	0.8	2.41	2.41	5.62
931	0	0.8	0.8	2.01	3.61
940	0	0.4	2.01	2.41	4.82
943	0	0.16	0.08	0	0.24
TOTAL	0	23.05	61.14	58.64	142.83

STATE: CONNECTICUT

ZIP CODE	QUARTER 1	QUARTER 2	QUARTER 3	QUARTER 4	TOTAL GRAMS
65	0	0	3.62	4.02	7.64
66	0	0	0	0.4	0.4
TOTAL	0	0	3.62	4.42	8.04

STATE: DISTRICT OF COLUMBIA

ZIP CODE	QUARTER 1	QUARTER 2	QUARTER 3	QUARTER 4	TOTAL GRAMS
200	0	0.8	2.81	1.61	5.22
TOTAL	0	0.8	2.81	1.61	5.22

STATE: FLORIDA

ZIP CODE	QUARTER 1	QUARTER 2	QUARTER 3	QUARTER 4	TOTAL GRAMS
326	0	0.8	2.01	1.21	4.02
329	0	0	0	0.8	0.8
334	0	4.02	0	0	4.02
337	0	0	2.41	1.21	3.62
TOTAL	0	4.82	4.42	3.22	12.46

STATE: GEORGIA

ZIP CODE	QUARTER 1	QUARTER 2	QUARTER 3	QUARTER 4	TOTAL GRAMS
310	0	1.61	2.41	2.41	6.43
315	0	0	2.41	2.41	4.82

TOTAL	0	1.61	4.82	4.82	11.25

STATE: HAWAII

ZIP CODE	QUARTER 1	QUARTER 2	QUARTER 3	QUARTER 4	TOTAL GRAMS
967	0	0	1.61	1.61	3.22
TOTAL	0	0	1.61	1.61	3.22

STATE: IDAHO

ZIP CODE	QUARTER 1	QUARTER 2	QUARTER 3	QUARTER 4	TOTAL GRAMS
838	0	1.21	5.22	4.02	10.45
TOTAL	0	1.21	5.22	4.02	10.45

STATE: ILLINOIS

ZIP CODE	QUARTER 1	QUARTER 2	QUARTER 3	QUARTER 4	TOTAL GRAMS
606	0	0	2.41	1.61	4.02
607	0	0	1.21	0.8	2.01
TOTAL	0	0	3.62	2.41	6.03

STATE: KANSAS

ZIP CODE	QUARTER 1	QUARTER 2	QUARTER 3	QUARTER 4	TOTAL GRAMS
660	0	0	0	0.8	0.8
666	0	0	0	0.8	0.8
672	0	0.8	0.8	1.21	2.81
TOTAL	0	0.8	0.8	2.81	4.41

STATE: KENTUCKY

ZIP CODE	QUARTER 1	QUARTER 2	QUARTER 3	QUARTER 4	TOTAL GRAMS
408	0	0.4	1.61	1.61	3.62
TOTAL	0	0.4	1.61	1.61	3.62

STATE: LOUISIANA

ZIP CODE	QUARTER 1	QUARTER 2	QUARTER 3	QUARTER 4	TOTAL GRAMS
708	0	0	2.01	2.41	4.42
TOTAL	0	0	2.01	2.41	4.42

STATE: MARYLAND

ZIP CODE	QUARTER 1	QUARTER 2	QUARTER 3	QUARTER 4	TOTAL GRAMS
208	0	0	0.8	1.21	2.01
210	0	0	0.4	2.41	2.81
TOTAL	0	0	1.2	3.62	4.82

STATE: MASSACHUSETTS

ZIP CODE	QUARTER 1	QUARTER 2	QUARTER 3	QUARTER 4	TOTAL GRAMS
10	0	0.4	2.01	1.61	4.02
18	0	0	0.4	0	0.4
21	0	0.4	1.21	0.4	2.01
24	0	0	0	0.4	0.4
TOTAL	0	0.8	3.62	2.41	6.83

STATE: MINNESOTA

ZIP CODE	QUARTER 1	QUARTER 2	QUARTER 3	QUARTER 4	TOTAL GRAMS
551	0	0.8	4.42	3.62	8.84
554	0	2.81	20.89	15.26	38.96
559	0	0	0	4.02	4.02
563	0	0.8	0.4	0	1.2
TOTAL	0	4.41	25.71	22.9	53.02

STATE: MISSISSIPPI

ZIP CODE	QUARTER 1	QUARTER 2	QUARTER 3	QUARTER 4	TOTAL GRAMS
397	0	0	1.61	0.8	2.41
TOTAL	0	0	1.61	0.8	2.41

STATE: MISSOURI

ZIP CODE	QUARTER 1	QUARTER 2	QUARTER 3	QUARTER 4	TOTAL GRAMS
631	0	2.81	10.85	15.67	29.33
641	0	0	0	0.8	0.8
655	0	0.4	1.21	0.4	2.01
TOTAL	0	3.21	12.06	16.87	32.14

STATE: NEBRASKA

ZIP CODE	QUARTER 1	QUARTER 2	QUARTER 3	QUARTER 4	TOTAL GRAMS
684	0	0	0	0.8	0.8
TOTAL	0	0	0	0.8	0.8

STATE: NEVADA

ZIP CODE	QUARTER 1	QUARTER 2	QUARTER 3	QUARTER 4	TOTAL GRAMS
891	0	0	0.4	0	0.4
TOTAL	0	0	0.4	0	0.4

STATE: NEW JERSEY

ZIP CODE	QUARTER 1	QUARTER 2	QUARTER 3	QUARTER 4	TOTAL GRAMS
70	0	0.8	0.8	0	1.6
79	0	0	0.4	0.8	1.2
85	0	0	1.61	1.21	2.82
TOTAL	0	0.8	2.81	2.01	5.62

STATE: NEW YORK

ZIP CODE	QUARTER 1	QUARTER 2	QUARTER 3	QUARTER 4	TOTAL GRAMS
101	0	0	0	0.8	0.8
104	0	0	4.02	4.1	8.12
106	0	0	1.21	1.21	2.42
113	0	0	1.61	0.8	2.41
128	4.02	0	0	0	4.02
TOTAL	4.02	0	6.84	6.91	17.77

STATE: NORTH CAROLINA

ZIP CODE	QUARTER 1	QUARTER 2	QUARTER 3	QUARTER 4	TOTAL GRAMS
272	0	0	0.8	0	0.8
275	0	0	4.02	4.02	8.04
278	0	0	1.21	1.61	2.82
280	0	0	0.8	0.8	1.6
281	0	0.8	0.4	0	1.2
286	0	0.8	1.61	2.01	4.42
288	0	0.4	0.8	1.61	2.81
TOTAL	0	2	9.64	10.05	21.69

STATE: NORTH DAKOTA

ZIP CODE	QUARTER 1	QUARTER 2	QUARTER 3	QUARTER 4	TOTAL GRAMS
581	0	0	1.61	0.4	2.01
582	0	0	4.02	0	4.02
TOTAL	0	0	5.63	0.4	6.03

STATE: OHIO

ZIP CODE	QUARTER 1	QUARTER 2	QUARTER 3	QUARTER 4	TOTAL GRAMS
432	0	0	3.62	7.63	11.25
446	0	0	0.4	0	0.4
450	0	0	2.01	2.41	4.42
452	0	0	0.4	0	0.4
454	0	0	0	0.8	0.8
457	0	0.4	2.01	1.61	4.02
TOTAL	0	0.4	8.44	12.45	21.29

STATE: OKLAHOMA

ZIP CODE	QUARTER 1	QUARTER 2	QUARTER 3	QUARTER 4	TOTAL GRAMS
741	0	0.4	4.02	2.81	7.23
TOTAL	0	0.4	4.02	2.81	7.23

STATE: OREGON

ZIP CODE	QUARTER 1	QUARTER 2	QUARTER 3	QUARTER 4	TOTAL GRAMS
972	0	0.4	2.01	2.01	4.42
973	0	4.82	0	0	4.82
974	0	0	0	0.4	0.4
978	0	4.02	12.05	12.05	28.12
TOTAL	0	9.24	14.06	14.46	37.76

STATE: PENNSYLVANIA

ZIP CODE	QUARTER 1	QUARTER 2	QUARTER 3	QUARTER 4	TOTAL GRAMS
151	0	0	2.01	2.01	4.02
187	0	3.21	6.43	6.03	15.67
190	0	0	3.21	3.21	6.42
TOTAL	0	3.21	11.65	11.25	26.11

STATE: RHODE ISLAND

ZIP CODE	QUARTER 1	QUARTER 2	QUARTER 3	QUARTER 4	TOTAL GRAMS
28	0	0	1.61	2.01	3.62
TOTAL	0	0	1.61	2.01	3.62

STATE: SOUTH CAROLINA

ZIP CODE	QUARTER 1	QUARTER 2	QUARTER 3	QUARTER 4	TOTAL GRAMS
292	0	2.01	4.02	2.81	8.84
TOTAL	0	2.01	4.02	2.81	8.84

STATE: TENNESSEE

ZIP CODE	QUARTER 1	QUARTER 2	QUARTER 3	QUARTER 4	TOTAL GRAMS
370	0	0	0	0.4	0.4
TOTAL	0	0	0	0.4	0.4

STATE: TEXAS

ZIP CODE	QUARTER 1	QUARTER 2	QUARTER 3	QUARTER 4	TOTAL GRAMS
750	0	0	1.21	0	1.21

	751	0	0	0	0.4	0.4
	752	0	0.8	4.02	1.61	6.43
	754	0	0	0.4	0	0.4
	756	0	0.4	2.01	2.01	4.42
	757	0	1.61	1.21	2.81	5.63
	759	0	0.8	0.8	1.61	3.21
	760	0	0	1.61	1.21	2.82
	761	0	0	0	0.4	0.4
	770	0	0.8	2.41	2.41	5.62
	782	0	0	0	1.21	1.21
	787	0	0	0.8	0	0.8
	788	0	0	3.62	3.21	6.83
	799	0	0	0	0.4	0.4
TOTAL		0	4.41	18.09	17.28	39.78

STATE: WASHINGTON

ZIP CODE	QUARTER 1	QUARTER 2	QUARTER 3	QUARTER 4	TOTAL GRAMS
981	0	0	0.8	0.8	1.6
986	0	0	0	0.8	0.8
TOTAL	0	0	0.8	1.6	2.4

STATE: WEST VIRGINIA

ZIP CODE	QUARTER 1	QUARTER 2	QUARTER 3	QUARTER 4	TOTAL GRAMS
260	0	0	2.41	1.21	3.62
TOTAL	0	0	2.41	1.21	3.62

STATE: WISCONSIN

ZIP CODE	QUARTER 1	QUARTER 2	QUARTER 3	QUARTER 4	TOTAL GRAMS
530	0	0.8	2.41	1.61	4.82
531	0	0	0	1.21	1.21
532	0	0	2.41	1.61	4.02
535	0	0	2.81	2.01	4.82
537	0	0.8	2.41	2.81	6.02
549	0	0	3.62	3.62	7.24

TOTAL		0	1.6	13.66	12.87	28.13

DRUG CODE: 1205 DRUG NAME: LISDEXAMFETAMINE

STATE: ALABAMA

ZIP CODE		QUARTER 1	QUARTER 2	QUARTER 3	QUARTER 4	TOTAL GRAMS
	350	6,887.48	6,568.95	6,546.98	6,970.15	26,973.56
	351	4,517.85	4,297.02	4,361.19	4,520.74	17,696.80
	352	9,847.93	9,235.73	9,262.90	9,830.01	38,176.57
	354	4,604.57	4,298.75	4,232.85	4,773.95	17,910.12
	355	1,366.05	1,297.26	1,296.68	1,356.22	5,316.21
	356	3,860.55	3,854.19	3,853.04	4,056.53	15,624.31
	357	2,342.46	2,232.04	2,347.09	2,297.95	9,219.54
	358	2,611.86	2,548.26	2,550.58	2,700.31	10,411.01
	359	2,580.64	2,403.74	2,330.32	2,428.60	9,743.30
	360	4,000.45	3,571.50	3,784.24	3,859.40	15,215.59
	361	2,972.59	2,653.48	2,614.17	2,881.83	11,122.07
	362	1,970.74	1,784.59	1,801.36	1,932.59	7,489.28
	363	4,388.94	4,171.57	4,181.51	4,271.19	17,013.21
	364	987.39	1,018.03	1,027.86	1,064.28	4,097.56
	365	7,968.53	7,737.87	7,954.08	8,354.70	32,015.18
	366	6,723.88	6,346.38	6,400.72	6,445.24	25,916.22
	367	816.86	637.07	719.73	790.84	2,964.50
	368	4,518.43	4,223.02	4,349.05	4,723.08	17,813.58
	369	157.24	128.92	156.09	164.18	606.43
TOTAL		73,124.44	69,008.37	69,770.44	73,421.79	285,325.04

STATE: ALASKA

ZIP CODE		QUARTER 1	QUARTER 2	QUARTER 3	QUARTER 4	TOTAL GRAMS
	995	1,127.87	1,201.87	1,235.40	1,258.52	4,823.66
	996	535.32	474.04	559.02	585.04	2,153.42
	997	352.06	373.45	367.67	343.97	1,437.15
	998	216.79	216.21	216.79	211.58	861.37
	999	134.7	147.99	155.51	152.04	590.24
TOTAL		2,366.74	2,413.56	2,534.39	2,551.15	9,865.84

STATE: ARIZONA

ZIP CODE	QUARTER 1	QUARTER 2	QUARTER 3	QUARTER 4	TOTAL GRAMS
850	6,502.47	6,472.99	6,322.10	6,675.90	25,973.46
851	1,233.67	1,223.84	1,298.41	1,367.78	5,123.70
852	16,863.18	16,603.61	17,038.92	18,626.96	69,132.67
853	4,206.26	3,956.52	3,975.59	4,406.86	16,545.23
855	132.38	118.51	95.96	145.1	491.95
856	682.74	483.87	543.99	593.13	2,303.73
857	4,683.19	4,330.55	4,413.22	4,771.06	18,198.02
859	131.23	145.1	184.99	196.55	657.87
860	347.44	335.88	299.46	350.91	1,333.69
863	633.02	586.19	644.58	720.31	2,584.10
864	237.02	258.41	264.19	276.33	1,035.95
TOTAL	35,652.60	34,515.47	35,081.41	38,130.89	143,380.37

STATE: ARKANSAS

ZIP CODE	QUARTER 1	QUARTER 2	QUARTER 3	QUARTER 4	TOTAL GRAMS
716	1,601.34	1,442.94	1,438.31	1,527.92	6,010.51
717	805.87	624.93	607.01	783.9	2,821.71
718	764.25	709.33	662.5	789.68	2,925.76
719	2,129.14	1,882.29	1,967.85	2,043.01	8,022.29
720	4,554.27	4,096.99	4,297.60	4,655.44	17,604.30
721	3,426.98	3,054.10	3,112.49	3,387.67	12,981.24
722	3,597.52	3,545.49	3,517.16	3,646.39	14,306.56
723	1,250.43	1,045.78	1,030.17	1,175.28	4,501.66
724	3,029.82	2,538.44	2,658.68	2,813.61	11,040.55
725	711.64	689.1	608.16	659.61	2,668.51
726	812.81	752.11	771.76	797.78	3,134.46
727	5,346.85	4,933.51	5,017.33	5,497.73	20,795.42
728	1,204.18	1,005.32	1,035.96	1,186.26	4,431.72
729	2,699.73	2,412.41	2,282.34	2,564.45	9,958.93
TOTAL	31,934.83	28,732.74	29,007.32	31,528.73	121,203.62

STATE: CALIFORNIA

DATE RANGE: 01/01/2017 TO 12/31/2017 ARCOS 3 - REPORT 1 Run Date: 07/03/2018

RETAIL DRUG DISTRIBUTION BY ZIP CODE WITHIN STATE BY GRAMS WT

ZIP CODE	QUARTER 1	QUARTER 2	QUARTER 3	QUARTER 4	TOTAL GRAMS
900	6,115.14	6,404.77	6,296.67	6,443.50	25,260.08
902	4,816.73	4,902.87	4,795.34	4,883.79	19,398.73
903	146.84	142.21	134.12	168.81	591.98
904	964.85	1,097.81	1,092.61	1,098.39	4,253.66
905	838.25	785.64	768.29	755	3,147.18
906	1,094.92	1,040	1,141.17	1,146.95	4,423.04
907	1,501.90	1,468.95	1,397.27	1,600.18	5,968.30
908	918.6	844.6	765.98	756.91	3,286.09
910	775.81	697.19	718	741.12	2,932.12
911	1,399	1,432.53	1,482.83	1,507.68	5,822.04
912	476.93	455.54	508.73	508.15	1,949.35
913	5,970.62	5,902.40	5,859.04	5,842.28	23,574.34
914	1,105.91	1,297.83	1,131.92	1,296.68	4,832.34
915	522.6	507.57	570.01	585.62	2,185.80
916	456.7	481.56	503.53	536.48	1,978.27
917	2,899.75	2,907.26	2,755.22	2,977.22	11,539.45
918	119.67	134.12	97.12	160.13	511.04
919	1,039.42	1,046.36	901.84	1,007.05	3,994.67
920	13,825.26	13,622.35	13,816.59	14,444.41	55,708.61
921	4,943.91	4,876.85	4,618.33	4,978.02	19,417.11
922	1,291.48	1,316.33	1,252.74	1,343.97	5,204.52
923	1,927.96	1,935.48	1,974.21	2,014.56	7,852.21
924	144.53	153.2	148.57	141.06	587.36
925	2,287.54	2,214.12	1,997.80	2,225.73	8,725.19
926	8,367.42	8,373.20	8,136.18	8,311.34	33,188.14
927	1,363.74	1,357.38	1,261.41	1,376.46	5,358.99
928	2,910.16	2,999.76	2,908.42	3,005.54	11,823.88
930	2,872	2,922.30	2,648.85	2,913.62	11,356.77
931	1,007.05	1,051.56	922.07	930.16	3,910.84
932	1,155.04	1,118.62	1,097.23	1,116.31	4,487.20
933	850.96	826.1	763.09	852.7	3,292.85
934	2,038.38	2,023.35	1,912.35	1,997.91	7,971.99
935	1,023.24	1,117.47	1,003.58	1,114	4,258.29
936	1,464.33	1,475.31	1,371.25	1,444.09	5,754.98
937	2,038.38	2,130.88	1,819.28	2,035.49	8,024.03
939	752.69	794.89	749.8	838.25	3,135.63

	940	2,119.31	2,146.49	2,129.14	2,273.67	8,668.61
	941	3,562.25	3,602.14	3,848.99	4,002.19	15,015.57
	943	424.9	431.26	404.67	457.28	1,718.11
	944	476.93	471.15	501.21	466.53	1,915.82
	945	4,812.68	4,860.09	4,757.18	4,936.97	19,366.92
	946	855.01	1,040.58	965.43	1,035.38	3,896.40
	947	501.21	459.01	497.17	551.51	2,008.90
	948	68.79	75.15	97.7	104.06	345.7
	949	1,312.87	1,444.67	1,325.58	1,494.39	5,577.51
	950	2,199.67	2,379.46	2,085.78	2,447.68	9,112.59
	951	1,235.40	1,241.18	1,212.85	1,284.54	4,973.97
	952	938.83	916.87	926.69	993.18	3,775.57
	953	2,352.87	2,304.88	2,325.12	2,348.24	9,331.11
	954	661.35	634.75	628.97	685.05	2,610.12
	955	129.49	96.54	115.62	144.53	486.18
	956	3,328.70	3,114.22	2,881.25	3,078.96	12,403.13
	957	993.75	1,003	1,057.34	1,069.49	4,123.58
	958	1,405.36	1,470.69	1,352.75	1,615.79	5,844.59
	959	1,501.90	1,347.55	1,434.27	1,438.31	5,722.03
	960	831.31	744.59	787.37	811.65	3,174.92
	961	121.4	132.38	137.59	120.24	511.61
TOTAL		111,261.69	111,775.01	108,794.11	114,459.20	446,290.01

STATE:		COLORADO				
ZIP CODE		QUARTER 1	QUARTER 2	QUARTER 3	QUARTER 4	TOTAL GRAMS
	800	4,064.62	4,066.36	3,838.58	4,238.63	16,208.19
	801	5,368.24	5,329.50	5,487.90	5,560.17	21,745.81
	802	5,706.43	5,782.16	5,815.11	6,117.45	23,421.15
	803	1,530.23	1,681.11	1,581.68	1,703.08	6,496.10
	804	924.96	861.37	829	931.9	3,547.23
	805	2,823.44	2,891.66	2,902.06	3,180.71	11,797.87
	806	1,261.41	1,145.22	1,155.62	1,190.31	4,752.56
	807	116.2	142.79	114.46	149.73	523.18
	808	431.84	446.29	456.12	436.47	1,770.72
	809	2,758.12	2,822.86	2,923.45	2,989.36	11,493.79
	810	580.99	546.88	520.87	609.32	2,258.06

	811	86.72	83.25	102.9	95.39	368.26
	812	116.78	108.1	127.76	135.28	487.92
	813	169.38	195.98	216.79	180.95	763.1
	814	127.76	115.62	129.49	145.1	517.97
	815	491.96	419.7	437.04	417.39	1,766.09
	816	760.2	756.73	856.74	741.12	3,114.79
TOTAL		27,319.28	27,395.58	27,495.57	28,822.36	111,032.79

STATE: CONNECTICUT

ZIP CODE		QUARTER 1	QUARTER 2	QUARTER 3	QUARTER 4	TOTAL GRAMS
	60	5,839.97	6,112.25	5,872.92	6,278.17	24,103.31
	61	2,263.84	2,181.75	1,933.17	2,218.75	8,597.51
	62	1,691.52	1,708.86	1,589.78	1,799.05	6,789.21
	63	3,620.64	3,561.10	3,574.39	3,711.40	14,467.53
	64	7,263.25	7,562.13	7,042.41	7,616.47	29,484.26
	65	1,826.22	1,837.78	1,666.08	1,973.06	7,303.14
	66	1,385.71	1,465.48	1,382.82	1,592.67	5,826.68
	67	3,033.87	3,005.54	2,804.94	3,046.59	11,890.94
	68	7,982.98	8,079.53	8,050.04	8,086.46	32,199.01
	69	1,038.27	1,013.41	986.82	1,051.56	4,090.06
TOTAL		35,946.27	36,527.83	34,903.37	37,374.18	144,751.65

STATE: DELAWARE

ZIP CODE		QUARTER 1	QUARTER 2	QUARTER 3	QUARTER 4	TOTAL GRAMS
	197	5,331.24	5,245.10	5,080.34	5,646.88	21,303.56
	198	3,632.20	3,540.28	3,472.07	3,728.75	14,373.30
	199	5,426.62	5,445.70	5,190.76	5,476.92	21,540
TOTAL		14,390.06	14,231.08	13,743.17	14,852.55	57,216.86

STATE: DISTRICT OF COLUMBIA

ZIP CODE		QUARTER 1	QUARTER 2	QUARTER 3	QUARTER 4	TOTAL GRAMS
	200	5,067.62	5,111.56	5,134.11	5,428.94	20,742.23
	203	5.2	17.34	71.11	0	93.65
	204	54.34	37.58	56.08	26.01	174.01

TOTAL		5,127.16	5,166.48	5,261.30	5,454.95	21,009.89

STATE:	FLORIDA					
ZIP CODE		QUARTER 1	QUARTER 2	QUARTER 3	QUARTER 4	TOTAL GRAMS
	320	4,714.41	4,438.65	4,289.50	4,597.63	18,040.19
	321	3,183.02	2,947.73	2,809.57	3,101.51	12,041.83
	322	8,924.13	8,328.69	8,162.66	8,677.43	34,092.91
	323	5,196.54	4,936.40	4,728.86	5,321.99	20,183.79
	324	3,543.17	3,189.38	3,231	3,143.13	13,106.68
	325	7,840.19	7,552.30	7,503.16	7,640.17	30,535.82
	326	3,334.48	3,280.72	3,038.67	3,324.08	12,977.95
	327	9,707.46	9,268.10	8,708.27	9,508.47	37,192.30
	328	8,386.50	7,861.58	7,389.62	8,239.34	31,877.04
	329	7,046.46	6,532.53	6,304.18	6,934.31	26,817.48
	330	7,102.54	6,563.75	5,700.64	6,858.58	26,225.51
	331	8,654.74	7,758.10	7,364.42	8,572.64	32,349.90
	333	3,713.71	3,679.61	3,257.02	3,695.79	14,346.13
	334	9,582.01	9,068.08	8,299.20	9,274.46	36,223.75
	335	7,008.31	6,646.99	6,686.88	7,015.24	27,357.42
	336	6,242.32	5,952.12	5,708.74	6,374.13	24,277.31
	337	5,224.29	4,665.27	4,723.08	4,933.30	19,545.94
	338	6,238.86	5,828.98	5,356.67	6,055.60	23,480.11
	339	5,076.30	4,714.98	4,472.18	4,919.63	19,183.09
	341	2,322.23	2,219.90	1,941.84	2,085.78	8,569.75
	342	5,899.51	5,728.97	5,482.12	6,053.86	23,164.46
	344	2,662.15	2,458.08	2,525.14	2,763.90	10,409.27
	346	4,411.48	4,025.31	3,871.54	4,031.67	16,340
	347	5,349.16	4,982.07	4,778	5,261.29	20,370.52
	349	2,610.12	2,455.77	2,334.37	2,691.06	10,091.32
TOTAL		143,974.09	135,084.06	128,667.33	141,074.99	548,800.47

STATE:	GEORGIA					
ZIP CODE		QUARTER 1	QUARTER 2	QUARTER 3	QUARTER 4	TOTAL GRAMS
	300	25,595.38	24,502.19	24,767.54	25,312.08	100,177.19
	301	12,216.99	11,183.34	11,857.99	12,042.40	47,300.72

	302	8,004.95	7,567.33	7,695.09	7,929.80	31,197.17
	303	12,388.68	12,419.32	12,179.41	12,658.08	49,645.49
	304	3,139.08	2,983.57	2,968.54	3,152.38	12,243.57
	305	6,744.11	5,964.84	6,381.65	6,642.95	25,733.55
	306	5,142.78	4,717.87	4,962.99	5,014.44	19,838.08
	307	2,650.59	2,475.42	2,498.55	2,593.36	10,217.92
	308	2,288.70	2,038.38	2,052.83	2,067.86	8,447.77
	309	3,040.81	2,618.21	2,830.96	3,113.65	11,603.63
	310	5,160.70	4,705.73	4,770.48	4,970.50	19,607.41
	312	2,192.16	2,054.57	2,043.01	2,213.54	8,503.28
	313	2,608.39	2,388.71	2,375.41	2,549.42	9,921.93
	314	3,428.13	3,151.80	3,061.62	3,226.38	12,867.93
	315	3,354.14	2,949.47	3,142.55	3,056.99	12,503.15
	316	1,680.54	1,393.22	1,625.62	1,566.07	6,265.45
	317	3,070.87	2,896.86	2,922.30	3,024.04	11,914.07
	318	232.97	235.86	234.71	235.86	939.4
	319	2,228.58	2,198.51	2,287.54	2,158.63	8,873.26
	398	442.82	461.9	450.34	417.97	1,773.03
TOTAL		105,611.37	98,907.10	101,109.13	103,946.40	409,574

STATE:	GUAM				
ZIP CODE	QUARTER 1	QUARTER 2	QUARTER 3	QUARTER 4	TOTAL GRAMS
969	37.58	27.17	12.14	13.87	90.76
TOTAL	37.58	27.17	12.14	13.87	90.76

STATE:	HAWAII				
ZIP CODE	QUARTER 1	QUARTER 2	QUARTER 3	QUARTER 4	TOTAL GRAMS
967	760.78	686.78	705.28	663.08	2,815.92
968	587.93	617.99	626.66	709.91	2,542.49
TOTAL	1,348.71	1,304.77	1,331.94	1,372.99	5,358.41

STATE:	IDAHO				
ZIP CODE	QUARTER 1	QUARTER 2	QUARTER 3	QUARTER 4	TOTAL GRAMS
832	450.34	497.17	502.37	562.49	2,012.37

	833	721.47	774.65	810.5	815.12	3,121.74
	834	1,438.31	1,387.44	1,389.75	1,481.67	5,697.17
	835	157.82	156.67	184.41	174.01	672.91
	836	2,739.62	2,900.91	2,872.58	3,217.13	11,730.24
	837	2,004.27	2,086.36	1,977.10	2,147.64	8,215.37
	838	980.46	945.77	1,045.78	975.25	3,947.26
TOTAL		8,492.29	8,748.97	8,782.49	9,373.31	35,397.06

STATE:	ILLINOIS					
ZIP CODE		QUARTER 1	QUARTER 2	QUARTER 3	QUARTER 4	TOTAL GRAMS
	600	16,113.96	15,945.15	15,856.70	16,303.58	64,219.39
	601	9,868.17	9,607.44	9,731.74	9,908.06	39,115.41
	602	741.7	770.61	669.44	733.03	2,914.78
	603	633.02	647.47	614.52	617.41	2,512.42
	604	7,208.33	7,077.10	7,076.52	7,409.51	28,771.46
	605	8,826.43	8,983.10	8,632.77	9,198.15	35,640.45
	606	10,593.10	10,353.19	10,498.87	11,028.99	42,474.15
	607	702.97	716.27	633.02	685.63	2,737.89
	608	165.34	184.99	141.63	184.41	676.37
	609	1,088.56	1,016.88	1,029.02	1,101.86	4,236.32
	610	931.32	849.81	874.67	912.24	3,568.04
	611	1,061.97	952.13	924.38	1,052.14	3,990.62
	612	679.27	596.02	624.35	649.21	2,548.85
	613	557.29	570.58	560.76	615.1	2,303.73
	614	675.22	649.78	638.8	727.83	2,691.63
	615	682.16	716.27	646.89	728.98	2,774.30
	616	1,122.67	1,072.95	1,090.30	1,207.07	4,492.99
	617	1,412.30	1,352.18	1,397.85	1,517.51	5,679.84
	618	1,352.75	1,253.32	1,207.07	1,517.51	5,330.65
	619	244.54	201.76	240.49	249.16	935.95
	620	937.68	848.65	946.35	1,004.16	3,736.84
	622	1,611.74	1,462.59	1,596.71	1,799.05	6,470.09
	623	768.29	778.7	809.92	898.95	3,255.86
	624	260.72	253.79	277.49	250.9	1,042.90
	625	486.18	424.9	456.7	530.12	1,897.90
	626	378.66	345.7	342.24	391.37	1,457.97

	627	645.74	564.23	593.13	624.93	2,428.03
	628	413.92	360.16	383.28	422.01	1,579.37
	629	1,020.35	927.27	973.52	1,034.80	3,955.94
TOTAL		71,184.35	69,482.99	69,469.13	73,303.67	283,440.14

STATE: INDIANA

ZIP CODE		QUARTER 1	QUARTER 2	QUARTER 3	QUARTER 4	TOTAL GRAMS
	460	10,186.70	10,034.66	10,127.16	10,645.71	40,994.23
	461	6,612.89	6,253.89	6,496.11	6,775.33	26,138.22
	462	9,568.71	9,423.03	9,494.14	9,862.96	38,348.84
	463	8,215.96	7,985.87	7,985.30	8,460.49	32,647.62
	464	805.87	704.7	579.26	712.22	2,802.05
	465	8,154.10	8,155.26	7,595.08	7,955.81	31,860.25
	466	2,615.90	2,568.50	2,434.96	2,670.82	10,290.18
	467	2,678.34	2,443.05	2,637.29	2,574.86	10,333.54
	468	3,424.66	3,425.82	3,437.96	3,685.39	13,973.83
	469	6,778.80	6,544.67	6,485.70	6,817.53	26,626.70
	470	653.83	670.6	620.3	692.56	2,637.29
	471	4,964.72	5,537.62	5,510.45	5,182.09	21,194.88
	472	1,390.91	1,314.02	1,330.21	1,530.23	5,565.37
	473	3,468.02	3,026.93	3,181.28	3,378.42	13,054.65
	474	3,046.01	2,684.70	2,809.57	3,011.32	11,551.60
	475	1,741.82	1,607.12	1,657.41	1,690.94	6,697.29
	476	2,147.06	2,054.57	2,114.69	2,288.12	8,604.44
	477	5,104.62	4,836.96	5,092.48	5,043.34	20,077.40
	478	1,327.90	1,167.18	1,164.29	1,218.63	4,878
	479	3,472.65	3,040.23	3,212.50	3,367.43	13,092.81
TOTAL		86,359.47	83,479.38	83,966.14	87,564.20	341,369.19

STATE: IOWA

ZIP CODE		QUARTER 1	QUARTER 2	QUARTER 3	QUARTER 4	TOTAL GRAMS
	500	2,621.68	2,391.60	2,456.35	2,544.22	10,013.85
	501	1,408.83	1,379.92	1,320.96	1,403.63	5,513.34
	502	1,560.29	1,499.01	1,468.95	1,613.48	6,141.73
	503	2,839.05	2,858.70	2,759.85	2,889.34	11,346.94

	504	912.82	883.34	857.9	832.46	3,486.52
	505	1,510.58	1,502.48	1,497.28	1,515.78	6,026.12
	506	1,550.46	1,411.14	1,351.60	1,431.38	5,744.58
	507	1,182.79	1,156.78	1,094.34	1,142.90	4,576.81
	508	167.65	192.51	160.71	187.3	708.17
	510	1,194.35	1,152.15	1,057.34	1,145.22	4,549.06
	511	2,483.52	2,565.03	2,545.37	2,726.90	10,320.82
	512	445.14	386.75	437.04	476.93	1,745.86
	513	749.22	616.83	611.63	627.24	2,604.92
	514	365.36	323.74	308.13	327.2	1,324.43
	515	1,699.04	1,656.83	1,636.60	1,726.21	6,718.68
	516	191.93	201.76	174.01	186.15	753.85
	520	1,733.72	1,713.49	1,640.65	1,856.86	6,944.72
	521	208.69	189.62	238.76	242.8	879.87
	522	2,460.39	2,582.95	2,494.50	2,465.02	10,002.86
	523	1,798.47	1,678.22	1,649.90	1,782.28	6,908.87
	524	2,510.69	2,501.44	2,383.51	2,460.97	9,856.61
	525	1,126.72	1,139.44	1,067.17	1,195.51	4,528.84
	526	1,253.32	1,077	1,105.33	1,147.53	4,583.18
	527	1,688.63	1,546.42	1,581.68	1,731.41	6,548.14
	528	1,523.87	1,418.66	1,375.30	1,493.23	5,811.06
TOTAL		35,187.21	34,025.81	33,274.86	35,151.95	137,639.83

STATE: KANSAS

ZIP CODE		QUARTER 1	QUARTER 2	QUARTER 3	QUARTER 4	TOTAL GRAMS
	660	7,128.55	7,028.54	7,060.91	7,502.58	28,720.58
	661	885.65	864.84	805.29	961.96	3,517.74
	662	7,574.84	7,430.32	7,595.66	8,048.31	30,649.13
	664	509.31	433.58	472.31	490.81	1,906.01
	665	849.23	812.81	808.18	955.02	3,425.24
	666	3,448.37	3,455.30	3,489.99	3,646.08	14,039.74
	667	1,246.96	1,155.62	1,157.93	1,180.48	4,740.99
	668	487.34	495.43	470.57	539.95	1,993.29
	669	110.42	107.53	98.28	104.64	420.87
	670	1,505.95	1,447.56	1,502.48	1,629.09	6,085.08
	671	617.99	607.58	598.33	627.24	2,451.14

	672	3,770.95	4,003.34	3,757.07	4,072.14	15,603.50
	673	545.15	550.35	560.76	631.29	2,287.55
	674	920.91	918.02	819.75	940.57	3,599.25
	675	853.85	778.12	744.59	879.87	3,256.43
	676	589.66	564.8	550.93	585.62	2,291.01
	677	119.67	109.26	137.01	133.54	499.48
	678	346.86	304.66	288.47	332.41	1,272.40
	679	92.5	82.09	95.96	122.56	393.11
TOTAL		31,604.16	31,149.75	31,014.47	33,384.16	127,152.54

STATE: KENTUCKY

ZIP CODE		QUARTER 1	QUARTER 2	QUARTER 3	QUARTER 4	TOTAL GRAMS
	400	3,260.48	3,109.60	3,059.31	3,136.19	12,565.58
	401	990.86	920.34	884.49	886.23	3,681.92
	402	10,683.87	10,215.61	9,907.48	10,216.76	41,023.72
	403	2,515.89	2,276.56	2,389.29	2,523.41	9,705.15
	404	1,027.86	962.54	1,020.92	1,071.22	4,082.54
	405	3,834.53	3,618.90	3,757.62	4,072.14	15,283.19
	406	464.79	492.54	451.5	408.14	1,816.97
	407	299.46	275.75	332.99	353.22	1,261.42
	408	117.35	94.23	101.17	91.92	404.67
	409	143.95	147.42	130.65	150.88	572.9
	410	4,657.75	4,442.12	4,352.51	4,464.09	17,916.47
	411	698.92	620.88	659.03	696.61	2,675.44
	412	280.96	237.6	196.55	231.82	946.93
	413	51.45	42.78	42.78	52.61	189.62
	414	101.75	91.92	88.45	91.92	374.04
	415	391.37	368.25	455.54	448.61	1,663.77
	416	279.22	258.99	265.93	259.57	1,063.71
	417	205.8	165.34	163.6	187.88	722.62
	418	47.4	33.53	34.69	40.47	156.09
	420	4,264.64	4,066.36	4,087.75	4,229.96	16,648.71
	421	1,708.29	1,630.24	1,710.02	1,665.51	6,714.06
	422	988.55	865.42	1,077	824.37	3,755.34
	423	2,134.92	1,943.57	1,879.40	1,890.39	7,848.28

	424	2,896.86	2,652.90	2,534.39	2,827.49	10,911.64
	425	348.02	295.41	338.77	358.42	1,340.62
	426	105.21	90.76	106.95	84.4	387.32
	427	1,477.62	1,413.45	1,457.97	1,569.54	5,918.58
TOTAL		43,977.77	41,333.01	41,486.75	42,833.77	169,631.30

STATE:	LOUISIANA				
ZIP CODE	QUARTER 1	QUARTER 2	QUARTER 3	QUARTER 4	TOTAL GRAMS
700	9,042.06	8,172.60	8,340.25	8,679.02	34,233.93
701	4,853.73	4,475.65	4,389.51	4,582.60	18,301.49
703	5,498.89	4,848.52	5,126.59	5,414.48	20,888.48
704	9,648.49	8,777.29	9,028.19	9,412.62	36,866.59
705	19,229.92	17,358.61	17,413.53	18,243.10	72,245.16
706	9,675.08	8,771.51	8,823.54	9,339.09	36,609.22
707	12,591.02	11,593.22	11,686.87	12,362.09	48,233.20
708	10,027.72	8,866.90	8,954.19	9,740.99	37,589.80
710	1,926.81	1,803.09	1,835.47	2,037.80	7,603.17
711	6,286.26	5,799.50	5,967.73	6,007.62	24,061.11
712	3,975.02	3,557.63	3,509.07	3,940.33	14,982.05
713	3,566.30	3,228.69	3,203.25	3,507.91	13,506.15
714	1,689.79	1,442.36	1,472.42	1,634.87	6,239.44
TOTAL	98,011.09	88,695.57	89,750.61	94,902.52	371,359.79

STATE:	MAINE				
ZIP CODE	QUARTER 1	QUARTER 2	QUARTER 3	QUARTER 4	TOTAL GRAMS
39	600.07	623.77	645.16	643.43	2,512.43
40	5,366.50	5,681.57	5,759.61	5,724.35	22,532.03
41	2,079.43	2,155.73	2,203.14	2,075.38	8,513.68
42	1,589.78	1,748.75	1,644.12	1,839.51	6,822.16
43	708.17	776.39	808.76	824.95	3,118.27
44	2,043.01	1,899.06	1,841.83	1,989.82	7,773.72
45	402.36	414.5	400.62	402.94	1,620.42
46	842.87	822.64	812.81	803.56	3,281.88
47	922.07	848.07	919.76	911.09	3,600.99
48	308.13	320.85	340.5	315.64	1,285.12

	49	1,423.86	1,528.50	1,604.23	1,693.25	6,249.84
TOTAL		16,286.25	16,819.83	16,980.54	17,223.92	67,310.54

STATE:	MARYLAND					
ZIP CODE		QUARTER 1	QUARTER 2	QUARTER 3	QUARTER 4	TOTAL GRAMS
	206	3,302.69	3,062.77	2,959.29	3,128.68	12,453.43
	207	4,086.01	3,868.07	3,770.37	4,111.45	15,835.90
	208	7,765.04	7,907.25	7,516.46	7,969.69	31,158.44
	209	1,446.41	1,459.12	1,355.64	1,459.70	5,720.87
	210	10,254.92	10,191.32	9,768.73	10,474.59	40,689.56
	211	5,183.82	5,044.50	4,806.90	5,087.86	20,123.08
	212	9,538.65	9,251.91	8,841.46	9,650.86	37,282.88
	214	1,460.28	1,496.70	1,444.09	1,444.09	5,845.16
	215	2,062.08	1,956.87	1,923.34	1,934.32	7,876.61
	216	2,400.85	2,418.19	2,220.48	2,515.89	9,555.41
	217	5,680.41	5,709.89	5,440.50	6,036.52	22,867.32
	218	2,317.60	2,204.87	2,140.70	2,199.67	8,862.84
	219	1,271.82	1,222.68	1,257.95	1,432.53	5,184.98
TOTAL		56,770.58	55,794.14	53,445.91	57,445.85	223,456.48

STATE:	MASSACHUSETTS					
ZIP CODE		QUARTER 1	QUARTER 2	QUARTER 3	QUARTER 4	TOTAL GRAMS
	10	1,834.89	1,759.16	1,669.55	1,847.03	7,110.63
	11	797.2	832.46	626.66	692.56	2,948.88
	12	497.17	456.12	483.87	591.97	2,029.13
	13	287.32	339.34	301.19	395.42	1,323.27
	14	1,341.77	1,367.21	1,216.32	1,344.66	5,269.96
	15	1,515.20	1,512.31	1,364.89	1,556.25	5,948.65
	16	962.54	942.88	871.2	935.37	3,711.99
	17	2,519.94	2,631.51	2,379.46	2,665.62	10,196.53
	18	4,408.01	4,466.40	4,302.80	4,308.58	17,485.79
	19	3,907.96	3,848.99	3,782.51	4,075.03	15,614.49
	20	3,022.88	3,019.99	2,895.70	3,093.41	12,031.98
	21	5,389.63	5,624.91	5,167.64	5,965.41	22,147.59
	22	292.52	323.16	250.32	387.33	1,253.33

	23	2,516.47	2,535.55	2,378.88	2,433.22	9,864.12
	24	2,601.45	2,767.94	2,619.37	2,726.32	10,715.08
	25	623.77	697.77	744.59	664.24	2,730.37
	26	757.89	855.01	1,000.69	901.26	3,514.85
	27	2,291.01	2,240.14	2,167.30	2,302.57	9,001.02
TOTAL		35,567.62	36,220.85	34,222.94	36,886.25	142,897.66

STATE: MICHIGAN

ZIP CODE	QUARTER 1	QUARTER 2	QUARTER 3	QUARTER 4	TOTAL GRAMS
480	7,469.05	7,510.10	7,097.33	7,806.66	29,883.14
481	7,124.45	6,782.85	6,649.31	7,405.46	27,962.07
482	1,644.69	1,608.27	1,437.73	1,652.21	6,342.90
483	6,638.32	6,420.38	6,383.84	6,852.10	26,294.64
484	2,098.50	1,927.39	1,855.70	2,034.91	7,916.50
485	814.54	822.06	744.59	811.07	3,192.26
486	2,552.31	2,387.55	2,360.38	2,528.61	9,828.85
487	1,012.25	1,097.81	956.76	1,029.02	4,095.84
488	4,685.50	4,432.87	4,524.21	4,666.42	18,309
489	1,121.51	1,113.42	1,188.57	1,104.75	4,528.25
490	6,018.60	5,516.23	5,459.58	5,863.09	22,857.50
491	869.46	893.74	848.65	912.82	3,524.67
492	1,610.59	1,450.45	1,448.72	1,627.93	6,137.69
493	1,921.60	1,900.21	1,766.67	2,021.04	7,609.52
494	5,005.77	5,046.81	5,040.45	5,376.33	20,469.36
495	4,228.80	4,140.35	4,158.27	4,247.30	16,774.72
496	2,494.50	2,641.92	2,490.45	2,620.53	10,247.40
497	1,345.82	1,431.95	1,312.87	1,425.59	5,516.23
498	1,230.77	1,182.21	1,151.58	1,263.73	4,828.29
499	386.75	371.14	362.47	413.34	1,533.70
TOTAL	60,273.78	58,677.71	57,238.13	61,662.91	237,852.53

STATE: MINNESOTA

ZIP CODE	QUARTER 1	QUARTER 2	QUARTER 3	QUARTER 4	TOTAL GRAMS
550	2,770.83	2,751.76	2,805.52	2,768.52	11,096.63
551	4,009.12	4,058.26	4,020.11	4,125.90	16,213.39
553	5,861.93	5,845.17	5,789.67	6,052.71	23,549.48

	554	5,685.61	6,018.60	5,937.67	6,053.29	23,695.17
	556	17.92	10.98	19.08	13.3	61.28
	557	1,099.55	1,134.81	1,115.15	1,036.53	4,386.04
	558	372.3	433	383.28	408.72	1,597.30
	559	1,218.06	1,344.08	1,233.67	1,211.70	5,007.51
	560	3,013.06	2,981.26	3,141.97	2,877.78	12,014.07
	561	684.47	721.47	671.75	615.68	2,693.37
	562	853.28	909.93	870.62	620.88	3,254.71
	563	1,520.98	1,420.97	1,459.70	1,330.79	5,732.44
	564	1,106.48	1,087.98	1,027.28	890.85	4,112.59
	565	1,919.29	1,837.20	1,708.86	1,392.64	6,857.99
	566	896.63	878.13	905.88	894.9	3,575.54
	567	774.65	815.12	717.42	524.91	2,832.10
TOTAL		31,804.16	32,248.72	31,807.63	30,819.10	126,679.61

STATE: MISSISSIPPI

ZIP CODE		QUARTER 1	QUARTER 2	QUARTER 3	QUARTER 4	TOTAL GRAMS
	386	7,082.88	6,477.61	6,705.96	7,035.48	27,301.93
	387	630.71	508.15	550.93	628.39	2,318.18
	388	2,974.32	2,758.69	2,707.24	2,887.61	11,327.86
	389	905.88	867.73	806.45	848.65	3,428.71
	390	2,983.57	2,727.48	2,762.16	2,911.31	11,384.52
	391	3,481.32	3,259.33	3,354.71	3,431.02	13,526.38
	392	3,144.86	2,804.36	2,751.18	3,098.04	11,798.44
	393	2,283.50	2,150.53	2,296.79	2,352.87	9,083.69
	394	6,845.86	6,275.85	6,289.15	6,736.60	26,147.46
	395	6,278.74	5,785.05	5,926.68	6,296.09	24,286.56
	396	2,027.40	1,705.97	1,826.22	1,964.38	7,523.97
	397	2,101.97	1,794.42	1,884.61	2,012.37	7,793.37
TOTAL		40,741.01	37,115.17	37,862.08	40,202.81	155,921.07

STATE: MISSOURI

ZIP CODE		QUARTER 1	QUARTER 2	QUARTER 3	QUARTER 4	TOTAL GRAMS
	630	7,741.34	7,387.54	7,437.26	7,758.10	30,324.24
	631	21,788.01	21,029.54	20,719.10	21,471.79	85,008.44

	633	7,036.63	6,884.01	7,040.10	7,345.34	28,306.08
	634	532.43	520.87	545.15	587.93	2,186.38
	635	613.36	530.7	497.17	557.29	2,198.52
	636	860.79	786.79	778.7	858.48	3,284.76
	637	572.9	524.34	531.27	528.96	2,157.47
	638	1,031.91	920.91	942.3	1,227.31	4,122.43
	639	426.06	354.95	346.86	367.67	1,495.54
	640	5,790.25	5,648.62	5,611.04	5,805.86	22,855.77
	641	9,015.47	8,793.48	8,739.14	9,095.25	35,643.34
	644	404.09	360.73	368.25	419.7	1,552.77
	645	1,209.39	1,143.48	1,216.32	1,241.18	4,810.37
	646	191.35	174.01	191.35	220.26	776.97
	647	794.89	640.53	692.56	697.19	2,825.17
	648	1,147.53	1,042.31	1,074.11	1,145.22	4,409.17
	650	493.12	467.1	538.21	518.56	2,016.99
	651	694.3	622.61	645.74	746.91	2,709.56
	652	2,259.21	2,074.22	2,020.46	2,381.77	8,735.66
	653	517.98	420.86	418.54	454.39	1,811.77
	654	382.7	335.3	428.95	471.15	1,618.10
	655	554.4	583.88	561.91	600.07	2,300.26
	656	794.89	634.75	656.14	746.91	2,832.69
	657	1,168.34	1,087.41	1,145.79	1,168.92	4,570.46
	658	1,888.07	1,851.08	1,918.71	2,001.96	7,659.82
TOTAL		67,909.41	64,820.02	65,065.13	68,418.17	266,212.73

STATE:	MONTANA					
ZIP CODE		QUARTER 1	QUARTER 2	QUARTER 3	QUARTER 4	TOTAL GRAMS
	590	282.11	192.51	216.21	200.02	890.85
	591	1,147.53	1,066.59	1,050.99	1,159.09	4,424.20
	592	116.2	102.32	105.79	115.04	439.35
	593	200.02	201.76	195.98	236.44	834.2
	594	746.91	657.88	637.64	674.06	2,716.49
	595	129.49	127.76	127.76	125.45	510.46
	596	304.66	323.74	300.03	396	1,324.43
	597	1,119.20	1,022.66	1,080.47	1,142.33	4,364.66
	598	1,389.75	1,360.27	1,363.16	1,428.49	5,541.67

	599	408.72	479.82	490.81	484.45	1,863.80
TOTAL		5,844.59	5,535.31	5,568.84	5,961.37	22,910.11

STATE: NEBRASKA

ZIP CODE	QUARTER 1	QUARTER 2	QUARTER 3	QUARTER 4	TOTAL GRAMS
680	1,511.15	1,419.24	1,551.04	1,642.96	6,124.39
681	6,354.48	6,159.08	6,303.02	6,722.72	25,539.30
683	519.13	413.34	437.04	452.07	1,821.58
684	413.92	357.27	345.7	413.34	1,530.23
685	3,816.62	3,854.77	3,851.42	4,265.71	15,788.52
686	431.26	455.54	395.42	493.7	1,775.92
687	583.88	564.23	526.07	590.24	2,264.42
688	1,326.74	1,243.49	1,211.70	1,296.10	5,078.03
689	486.18	428.37	423.17	446.29	1,784.01
690	139.9	155.51	192.51	174.01	661.93
691	382.12	383.28	367.67	387.33	1,520.40
692	20.81	13.3	13.3	16.19	63.6
693	430.68	471.15	457.86	453.81	1,813.50
TOTAL	16,416.87	15,918.57	16,075.92	17,354.47	65,765.83

STATE: NEVADA

ZIP CODE	QUARTER 1	QUARTER 2	QUARTER 3	QUARTER 4	TOTAL GRAMS
890	1,891.54	1,881.14	1,723.89	1,878.25	7,374.82
891	3,993.51	3,779.62	3,708.97	3,832.22	15,314.32
893	22.55	6.36	15.03	7.52	51.46
894	666.55	644.58	626.66	690.25	2,628.04
895	952.13	939.41	900.68	1,001.27	3,793.49
897	221.99	245.69	171.7	242.22	881.6
898	51.45	54.92	60.12	68.22	234.71
TOTAL	7,799.72	7,551.72	7,207.05	7,719.95	30,278.44

STATE: NEW HAMPSHIRE

ZIP CODE	QUARTER 1	QUARTER 2	QUARTER 3	QUARTER 4	TOTAL GRAMS
30	4,035.14	4,136.31	3,943.80	3,962.88	16,078.13

31	2,226.84	2,322.23	2,161.52	2,202.56	8,913.15
32	1,127.30	1,119.78	1,042.31	1,153.31	4,442.70
33	889.12	809.34	851.54	883.91	3,433.91
34	425.48	431.26	368.25	411.61	1,636.60
35	310.44	289.63	302.35	304.66	1,207.08
36	29.48	28.33	25.44	43.94	127.19
37	756.15	794.89	722.05	752.11	3,025.20
38	4,151.34	4,206.83	4,147.29	4,268.69	16,774.15
TOTAL	13,951.29	14,138.60	13,564.55	13,983.67	55,638.11

STATE: NEW JERSEY

ZIP CODE	QUARTER 1	QUARTER 2	QUARTER 3	QUARTER 4	TOTAL GRAMS
70	8,994.08	8,970.96	8,282.44	8,923.55	35,171.03
71	501.79	501.79	526.07	603.54	2,133.19
72	386.17	354.38	303.5	377.5	1,421.55
73	504.1	578.68	530.7	579.26	2,192.74
74	3,320.03	3,465.71	3,297.48	3,399.23	13,482.45
75	396	387.91	363.62	356.11	1,503.64
76	3,296.90	3,292.28	3,130.41	3,431.60	13,151.19
77	3,092.26	3,281.30	3,228.69	3,297.48	12,899.73
78	3,009.59	2,909.58	2,829.22	2,932.70	11,681.09
79	3,140.24	3,187.07	3,076.65	3,263.37	12,667.33
80	9,025.30	9,121.84	8,727.58	8,733.36	35,608.08
81	623.19	624.93	590.82	659.61	2,498.55
82	1,115.73	1,309.40	1,296.10	1,190.31	4,911.54
83	994.33	1,029.02	846.34	897.21	3,766.90
84	90.18	126.6	149.73	115.04	481.55
85	2,161.52	2,307.78	2,088.10	2,306.04	8,863.44
86	1,415.19	1,521.56	1,414.03	1,492.08	5,842.86
87	2,302.57	2,233.78	2,318.76	2,252.86	9,107.97
88	4,222.44	4,163.48	4,001.03	4,291.24	16,678.19
89	471.73	456.7	454.96	514.51	1,897.90
TOTAL	49,063.34	49,824.75	47,456.23	49,616.60	195,960.92

STATE: NEW MEXICO

ZIP CODE		QUARTER 1	QUARTER 2	QUARTER 3	QUARTER 4	TOTAL GRAMS
	870	273.44	239.33	254.36	263.61	1,030.74
	871	2,382.93	2,151.69	2,018.15	1,737.19	8,289.96
	873	30.06	24.28	17.92	32.37	104.63
	874	128.34	125.45	132.38	143.37	529.54
	875	402.94	412.76	396.58	386.17	1,598.45
	877	79.2	94.23	70.53	91.34	335.3
	878	40.47	45.67	39.89	42.2	168.23
	879	33.53	17.34	17.92	13.87	82.66
	880	437.04	401.78	366.52	433	1,638.34
	881	133.54	94.81	90.76	93.65	412.76
	882	556.71	536.48	497.17	580.41	2,170.77
	883	198.87	193.66	169.38	235.86	797.77
	884	14.45	31.22	30.06	24.86	100.59
TOTAL		4,711.52	4,368.70	4,101.62	4,077.90	17,259.74

STATE: NEW YORK

ZIP CODE		QUARTER 1	QUARTER 2	QUARTER 3	QUARTER 4	TOTAL GRAMS
	100	15,013.26	16,397.81	15,924.34	17,150.45	64,485.86
	101	1,001.85	1,003	902.99	933.05	3,840.89
	102	174.59	156.67	139.9	157.82	628.98
	103	1,139.44	1,146.37	1,018.61	1,164.87	4,469.29
	104	1,279.34	1,285.12	994.33	1,116.89	4,675.68
	105	5,071.67	5,125.43	5,021.95	5,334.71	20,553.76
	106	556.13	511.04	522.6	529.54	2,119.31
	107	679.27	692.56	672.33	681	2,725.16
	108	474.04	403.51	412.76	410.45	1,700.76
	109	2,903.80	2,614.17	2,565.03	2,607.23	10,690.23
	110	1,291.48	1,338.30	1,248.70	1,378.77	5,257.25
	111	737.66	815.12	764.83	814.54	3,132.15
	112	5,737.64	5,683.88	5,239.90	5,890.84	22,552.26
	113	1,627.35	1,641.80	1,514.62	1,714.07	6,497.84
	114	560.18	530.7	575.21	534.16	2,200.25
	115	4,342.69	4,370.44	4,141.51	4,382.58	17,237.22
	116	348.02	332.41	312.75	340.5	1,333.68
	117	8,655.31	8,550.10	8,174.33	8,763.42	34,143.16

	118	503.53	552.66	522.02	626.66	2,204.87
	119	1,002.43	1,092.03	1,179.32	1,050.99	4,324.77
	120	1,895.59	1,804.25	1,899.64	1,934.90	7,534.38
	121	1,052.72	945.19	1,034.80	1,110.53	4,143.24
	122	718.58	746.91	675.8	800.09	2,941.38
	123	767.14	723.2	720.89	714.53	2,925.76
	124	456.12	437.62	485.03	474.62	1,853.39
	125	2,218.75	2,370.79	2,316.45	2,417.04	9,323.03
	126	502.95	490.81	437.62	487.92	1,919.30
	127	449.18	542.26	470	458.43	1,919.87
	128	1,385.68	1,403.60	1,346.97	1,446.98	5,583.23
	129	453.81	499.48	429.53	457.86	1,840.68
	130	1,834.89	1,839.51	1,821.02	1,915.25	7,410.67
	131	727.83	725.52	627.24	737.66	2,818.25
	132	913.98	867.15	931.32	980.46	3,692.91
	133	579.26	553.82	500.63	491.39	2,125.10
	134	659.61	698.34	675.22	701.24	2,734.41
	135	232.97	242.22	246.85	238.76	960.8
	136	1,170.65	1,095.50	1,167.18	1,282.80	4,716.13
	137	559.02	562.49	573.48	568.85	2,263.84
	138	799.51	768.29	753.26	848.07	3,169.13
	139	573.48	599.49	509.31	605.85	2,288.13
	140	1,589.20	1,468.37	1,454.50	1,533.70	6,045.77
	141	593.13	591.4	576.37	563.07	2,323.97
	142	1,936.64	2,030.87	1,865.53	2,122.21	7,955.25
	143	137.59	151.46	164.18	137.01	590.24
	144	2,037.22	1,978.26	1,955.71	2,278.29	8,249.48
	145	1,699.61	1,833.16	1,714.07	1,847.61	7,094.45
	146	2,645.39	2,617.64	2,684.12	2,837.89	10,785.04
	147	707.02	709.91	732.45	690.25	2,839.63
	148	1,139.44	1,066.02	1,071.22	1,100.12	4,376.80
	149	324.31	282.11	255.52	359	1,220.94
TOTAL		83,860.95	84,888.76	81,943.94	87,724.92	338,418.57

STATE:	NORTH CAROLINA				
ZIP CODE	QUARTER 1	QUARTER 2	QUARTER 3	QUARTER 4	TOTAL GRAMS

	270	2,374.26	2,327.43	2,168.45	2,529.77	9,399.91
	271	3,333.90	3,076.65	3,211.35	3,335.06	12,956.96
	272	5,997.21	5,915.70	5,637.63	6,329.04	23,879.58
	273	3,554.16	3,438.54	3,220.02	3,530.46	13,743.18
	274	3,828.18	3,720.65	3,447.79	3,872.11	14,868.73
	275	11,765.49	11,805.38	11,125.53	12,384.06	47,080.46
	276	5,605.84	5,476.34	5,374.02	5,849.79	22,305.99
	277	2,215.89	2,186.37	2,190.42	2,209.04	8,801.72
	278	5,287.88	4,710.36	4,757.76	5,164.75	19,920.75
	279	1,271.82	1,286.27	1,182.21	1,261.99	5,002.29
	280	9,887.24	9,336.89	9,132.82	9,801.69	38,158.64
	281	7,577.16	7,320.48	6,774.18	7,589.30	29,261.12
	282	8,201.50	8,211.33	7,762.15	8,526.98	32,701.96
	283	8,161.62	7,671.97	7,293.31	8,018.25	31,145.15
	284	6,533.69	6,281.63	6,176.42	6,520.39	25,512.13
	285	5,372.86	5,316.79	5,422	5,496.57	21,608.22
	286	7,763.88	7,685.84	7,508.36	8,584.21	31,542.29
	287	3,830.49	3,703.31	3,783.66	3,922.41	15,239.87
	288	1,575.32	1,619.84	1,491.50	1,719.27	6,405.93
	289	262.46	297.72	237.02	239.91	1,037.11
TOTAL		104,400.85	101,389.49	97,896.60	106,885.05	410,571.99

STATE:		NORTH DAKOTA				
ZIP CODE		QUARTER 1	QUARTER 2	QUARTER 3	QUARTER 4	TOTAL GRAMS
	580	647.47	557.87	612.79	572.9	2,391.03
	581	1,770.72	1,614.63	1,624.46	1,668.97	6,678.78
	582	1,231.35	1,116.89	1,162.56	1,241.76	4,752.56
	583	313.91	314.49	266.5	294.25	1,189.15
	584	208.12	161.87	165.34	185.57	720.9
	585	550.93	455.54	496.59	527.81	2,030.87
	586	115.04	131.81	120.24	121.4	488.49
	587	873.51	959.07	941.72	987.97	3,762.27
	588	220.83	204.07	202.34	250.9	878.14
TOTAL		5,931.88	5,516.24	5,592.54	5,851.53	22,892.19

RETAIL DRUG DISTRIBUTION BY ZIP CODE WITHIN STATE BY GRAMS WT

STATE: OHIO

ZIP CODE	QUARTER 1	QUARTER 2	QUARTER 3	QUARTER 4	TOTAL GRAMS
430	9,625.94	9,287.75	9,142.65	9,550.21	37,606.55
431	3,734.53	3,619.48	3,610.81	3,836.27	14,801.09
432	22,553.99	19,659.45	20,684.42	20,626.03	83,523.89
433	900.68	926.69	861.95	904.73	3,594.05
434	1,119.20	1,053.88	972.36	1,118.62	4,264.06
435	2,432.64	2,286.39	2,199.09	2,352.29	9,270.41
436	3,063.35	3,003.23	2,807.25	2,797.43	11,671.26
437	1,084.52	1,051.56	909.93	991.44	4,037.45
438	182.1	212.16	181.52	189.62	765.4
439	1,106.48	1,140.59	1,083.94	1,163.14	4,494.15
440	9,544.43	9,318.39	9,278.51	9,517.26	37,658.59
441	10,734.97	10,393.66	10,153.29	10,925.86	42,207.78
442	4,800.54	4,778	4,752.56	4,984.38	19,315.48
443	1,799.63	1,716.38	1,725.63	1,795.58	7,037.22
444	2,199.09	2,184.06	2,189.84	2,263.84	8,836.83
445	2,023.93	1,927.96	1,899.64	2,000.23	7,851.76
446	3,395.76	3,320.03	3,235.63	3,341.42	13,292.84
447	2,806.10	2,661.57	2,557.51	2,719.96	10,745.14
448	1,842.40	1,655.10	1,704.24	1,712.33	6,914.07
449	766.56	737.08	656.14	745.17	2,904.95
450	6,795.57	6,327.88	6,314.01	7,048.02	26,485.48
451	2,386.97	2,306.04	2,200.25	2,432.64	9,325.90
452	7,726.88	7,364.42	7,287.53	7,759.26	30,138.09
453	3,432.76	3,297.48	3,348.93	3,355.87	13,435.04
454	3,682.50	3,718.92	3,533.93	3,843.79	14,779.14
455	837.67	744.01	776.39	824.37	3,182.44
456	1,827.95	1,702.50	1,673.60	1,760.89	6,964.94
457	1,694.99	1,559.71	1,496.70	1,669.55	6,420.95
458	1,238.87	1,256.79	1,138.86	1,354.49	4,989.01
TOTAL	115,341	109,211.16	108,377.11	113,584.69	446,513.96

STATE: OKLAHOMA

ZIP CODE	QUARTER 1	QUARTER 2	QUARTER 3	QUARTER 4	TOTAL GRAMS
730	8,606.17	8,441.42	8,430.43	8,830.48	34,308.50

	731	7,066.05	6,684.57	6,756.13	7,066.15	27,572.90
	734	781.59	670.6	702.39	716.27	2,870.85
	735	1,215.74	1,221.53	1,312.29	1,309.40	5,058.96
	736	327.78	307.55	321.42	335.88	1,292.63
	737	831.31	846.34	805.87	882.76	3,366.28
	738	122.56	99.43	64.75	98.86	385.6
	739	70.53	60.7	37	36.42	204.65
	740	6,848.17	6,536.58	6,756.83	7,523.39	27,664.97
	741	4,449.06	4,211.46	4,343.27	4,523.05	17,526.84
	743	1,134.23	1,097.81	1,006.47	1,211.12	4,449.63
	744	1,816.97	1,639.49	1,732.57	1,864.37	7,053.40
	745	622.04	621.46	729.56	671.17	2,644.23
	746	428.95	359.58	394.26	426.64	1,609.43
	747	627.82	545.15	648.05	612.79	2,433.81
	748	1,553.93	1,471.84	1,318.65	1,644.69	5,989.11
	749	612.21	489.65	506.42	637.07	2,245.35
TOTAL		37,115.11	35,305.16	35,866.36	38,390.51	146,677.14

STATE: OREGON

ZIP CODE		QUARTER 1	QUARTER 2	QUARTER 3	QUARTER 4	TOTAL GRAMS
	970	3,696.95	3,765.17	3,661.11	3,802.13	14,925.36
	971	1,018.03	1,085.09	1,094.92	1,091.45	4,289.49
	972	4,454.26	4,639.83	4,473.34	4,714.98	18,282.41
	973	1,715.22	1,647.01	1,579.95	1,738.92	6,681.10
	974	1,479.36	1,433.69	1,396.69	1,565.49	5,875.23
	975	608.74	631.29	603.54	601.22	2,444.79
	976	102.32	91.92	67.64	119.67	381.55
	977	1,315.18	1,286.85	1,328.47	1,325.01	5,255.51
	978	397.15	422.59	406.98	432.42	1,659.14
	979	73.42	82.67	79.2	87.87	323.16
TOTAL		14,860.63	15,086.11	14,691.84	15,479.16	60,117.74

STATE: PENNSYLVANIA

ZIP CODE		QUARTER 1	QUARTER 2	QUARTER 3	QUARTER 4	TOTAL GRAMS
	150	2,642.50	2,462.13	2,489.30	2,728.05	10,321.98

151	1,852.23	1,866.11	1,822.75	1,967.27	7,508.36
152	4,263.49	3,936.28	3,947.84	4,344.42	16,492.03
153	939.99	920.91	982.77	970.63	3,814.30
154	730.72	757.89	744.01	821.48	3,054.10
155	286.16	278.64	270.55	308.13	1,143.48
156	1,659.73	1,690.94	1,673.60	1,827.95	6,852.22
157	1,289.16	1,244.65	1,189.15	1,313.44	5,036.40
158	1,096.08	1,079.31	1,129.03	1,152.73	4,457.15
159	757.89	789.68	760.2	781.59	3,089.36
160	1,330.21	1,349.86	1,384.55	1,463.17	5,527.79
161	1,608.27	1,531.97	1,515.20	1,677.65	6,333.09
162	571.16	637.07	596.6	641.11	2,445.94
163	1,205.34	1,080.47	1,013.41	1,128.45	4,427.67
164	639.38	609.32	659.61	686.78	2,595.09
165	2,848.88	2,840.78	2,806.10	2,826.33	11,322.09
166	1,324.43	1,363.16	1,314.02	1,438.89	5,440.50
167	479.82	449.76	501.79	470	1,901.37
168	1,513.47	1,334.83	1,483.40	1,493.81	5,825.51
169	417.97	381.55	352.06	417.97	1,569.55
170	4,490.68	4,461.78	4,439.23	4,573.93	17,965.62
171	1,296.10	1,235.40	1,274.13	1,356.80	5,162.43
172	1,150.42	1,115.73	1,199.56	1,301.30	4,767.01
173	2,170.19	2,100.82	1,934.90	2,052.26	8,258.17
174	1,186.84	1,274.13	1,127.87	1,326.16	4,915
175	1,367.21	1,365.47	1,349.29	1,431.95	5,513.92
176	1,500.17	1,631.98	1,486.30	1,629.66	6,248.11
177	739.39	702.39	707.59	770.61	2,919.98
178	636.49	664.24	671.17	855.59	2,827.49
179	256.1	300.61	289.05	280.38	1,126.14
180	2,293.32	2,193.31	2,113.53	2,151.11	8,751.27
181	902.99	825.53	844.6	975.25	3,548.37
182	339.92	309.86	272.29	301.19	1,223.26
183	517.4	511.62	513.35	551.51	2,093.88
184	592.55	586.19	548.62	637.64	2,365
185	398.89	418.54	463.06	427.22	1,707.71
186	454.96	426.06	359.58	386.75	1,627.35
187	10,886.78	10,912.79	10,891.98	11,118.02	43,809.57

	188	200.02	231.82	176.32	180.37	788.53
	189	2,348.82	2,304.88	2,410.10	2,374.83	9,438.63
	190	8,091.67	8,115.95	7,782.38	8,362.79	32,352.79
	191	5,821.47	5,726.08	5,551.49	6,157.34	23,256.38
	193	4,832.92	4,594.16	4,469.29	4,748.51	18,644.88
	194	3,574.39	3,737.42	3,560.52	3,660.53	14,532.86
	195	965.43	935.94	832.46	937.68	3,671.51
	196	1,721.58	1,749.91	1,667.82	1,788.06	6,927.37
TOTAL		86,193.58	85,037.92	83,572.42	88,797.29	343,601.21

STATE: PUERTO RICO

ZIP CODE		QUARTER 1	QUARTER 2	QUARTER 3	QUARTER 4	TOTAL GRAMS
	6	304.08	285.58	286.74	317.96	1,194.36
	7	313.33	219.68	221.41	223.15	977.57
	9	1,503.06	1,360.27	1,222.10	1,348.13	5,433.56
TOTAL		2,120.47	1,865.53	1,730.25	1,889.24	7,605.49

STATE: RHODE ISLAND

ZIP CODE		QUARTER 1	QUARTER 2	QUARTER 3	QUARTER 4	TOTAL GRAMS
	28	5,021.95	5,241.63	4,904.02	5,330.08	20,497.68
	29	2,417.04	2,433.80	2,267.89	2,495.08	9,613.81
TOTAL		7,438.99	7,675.43	7,171.91	7,825.16	30,111.49

STATE: SOUTH CAROLINA

ZIP CODE		QUARTER 1	QUARTER 2	QUARTER 3	QUARTER 4	TOTAL GRAMS
	290	5,370.55	4,957.79	5,063.58	5,654.97	21,046.89
	291	3,658.79	3,538.55	3,169.72	3,646.08	14,013.14
	292	4,750.25	4,165.21	4,334.59	4,929.46	18,179.51
	293	4,939.29	4,854.31	4,455.99	5,212.15	19,461.74
	294	14,073.84	13,381.86	13,131.43	14,348.76	54,935.89
	295	7,828.63	7,261.51	7,385.81	7,682.95	30,158.90
	296	14,389.49	13,774.97	13,626.40	15,154.89	56,945.75
	297	3,287.65	3,104.40	3,008.43	3,474.96	12,875.44

	298	2,540.75	2,211.23	2,127.41	2,411.83	9,291.22
	299	1,975.37	1,857.44	1,758	1,932.01	7,522.82
TOTAL		62,814.61	59,107.27	58,061.36	64,448.06	244,431.30

STATE: SOUTH DAKOTA

ZIP CODE	QUARTER 1	QUARTER 2	QUARTER 3	QUARTER 4	TOTAL GRAMS
570	1,643.54	1,536.59	1,564.34	1,666.08	6,410.55
571	6,508.83	6,343.49	5,917.43	6,098.96	24,868.71
572	467.1	379.81	389.06	411.03	1,647
573	950.97	851.54	861.37	905.3	3,569.18
574	601.8	494.28	481.56	522.02	2,099.66
575	330.67	261.3	299.46	283.85	1,175.28
576	7.52	12.72	23.12	14.45	57.81
577	944.62	1,019.77	978.72	1,064.28	4,007.39
TOTAL	11,455.05	10,899.50	10,515.06	10,965.97	43,835.58

STATE: TENNESSEE

ZIP CODE	QUARTER 1	QUARTER 2	QUARTER 3	QUARTER 4	TOTAL GRAMS
370	12,078.82	11,279.31	11,620.39	11,893.83	46,872.35
371	7,747.70	7,414.71	7,182.31	7,544.21	29,888.93
372	7,357.48	7,256.31	7,077.68	7,317.01	29,008.48
373	5,731.28	5,057.22	5,291.35	5,364.19	21,444.04
374	3,478.43	3,401.54	3,198.63	3,322.92	13,401.52
376	2,428.02	2,291.59	2,185.22	2,419.93	9,324.76
377	2,804.94	2,664.46	2,752.91	2,870.27	11,092.58
378	4,287.77	4,158.27	4,302.80	4,413.79	17,162.63
379	5,912.23	5,758.45	5,821.47	6,108.20	23,600.35
380	5,972.93	5,508.14	5,511.61	5,722.03	22,714.71
381	7,825.16	7,374.82	7,397.48	7,741.05	30,338.51
382	913.4	673.49	796.04	864.26	3,247.19
383	2,474.85	2,143.02	2,264.42	2,441.32	9,323.61
384	1,734.30	1,684.58	1,731.99	1,807.72	6,958.59
385	1,667.82	1,426.17	1,507.11	1,619.84	6,220.94
TOTAL	72,415.13	68,092.08	68,641.41	71,450.57	280,599.19

STATE: TEXAS

ZIP CODE	QUARTER 1	QUARTER 2	QUARTER 3	QUARTER 4	TOTAL GRAMS
750	34,490.60	32,915.86	31,851	31,782.78	131,040.24
751	8,307.88	7,787.59	7,426.85	7,870.83	31,393.15
752	15,162.41	15,187.27	14,685.47	15,337.57	60,372.72
753	16.19	10.98	10.41	20.81	58.39
754	2,737.30	2,375.41	2,393.91	2,703.77	10,210.39
755	939.99	771.76	856.74	902.41	3,470.90
756	3,690.59	3,440.85	3,411.95	3,669.78	14,213.17
757	3,737.42	3,592.31	3,582.49	3,874.43	14,786.65
758	750.37	686.2	694.3	801.82	2,932.69
759	2,306.04	2,189.84	1,974.79	2,356.91	8,827.58
760	14,021.24	13,860.53	13,611.36	14,301.62	55,794.75
761	7,353.43	7,073.63	6,756.83	7,175.96	28,359.85
762	6,072.94	6,026.11	5,881.59	6,342.34	24,322.98
763	1,888.07	1,853.39	1,870.73	1,889.23	7,501.42
764	605.85	548.62	579.83	593.71	2,328.01
765	3,162.79	2,629.78	2,674.29	2,641.34	11,108.20
766	825.53	700.66	693.72	757.89	2,977.80
767	2,995.71	2,720.54	2,650.59	2,756.38	11,123.22
768	401.2	385.01	391.37	399.47	1,577.05
769	1,696.15	1,532.54	1,451.61	1,549.89	6,230.19
770	28,521.72	28,156.94	25,092.20	27,585.20	109,356.06
772	11.56	16.19	24.28	10.98	63.01
773	20,837.61	20,003.42	19,122.39	20,841.08	80,804.50
774	17,066.67	16,825.60	14,930.01	16,550.42	65,372.70
775	13,783.64	12,850.01	11,964.94	12,523.38	51,121.97
776	3,392.29	3,343.15	3,187.64	3,666.89	13,589.97
777	1,775.35	1,796.73	1,615.79	1,815.81	7,003.68
778	6,240.01	5,759.03	5,626.65	6,394.36	24,020.05
779	2,244.18	1,985.20	1,796.16	1,978.84	8,004.38
780	5,341.64	4,904.60	4,729.44	5,397.14	20,372.82
781	3,970.39	3,543.17	3,543.17	4,041.50	15,098.23
782	16,416.31	16,111.65	15,574.94	16,656.85	64,759.75
783	2,316.45	2,047.05	2,032.60	2,128.56	8,524.66
784	4,151.34	3,670.36	3,458.19	3,864.60	15,144.49

	785	7,868.52	6,755.10	6,770.71	7,408.35	28,802.68
	786	9,138.03	8,802.73	8,695.20	9,405.69	36,041.65
	787	13,569.74	13,800.40	13,377.23	14,704.38	55,451.75
	788	722.05	617.99	649.21	687.94	2,677.19
	789	411.61	418.54	376.92	464.21	1,671.28
	790	700.66	552.66	554.98	623.19	2,431.49
	791	1,178.17	1,198.98	1,194.93	1,368.94	4,941.02
	792	78.62	57.81	84.4	81.51	302.34
	793	411.61	383.86	317.96	365.36	1,478.79
	794	3,316.56	3,037.34	2,961.61	3,484.21	12,799.72
	795	414.5	380.39	353.8	459.59	1,608.28
	796	1,485.14	1,333.10	1,267.20	1,585.15	5,670.59
	797	2,864.49	2,805.52	2,792.22	2,939.06	11,401.29
	798	83.82	87.87	60.7	67.06	299.45
	799	3,095.73	2,782.97	2,719.38	2,933.86	11,531.94
TOTAL		282,570.11	270,317.24	258,324.68	277,763.05	1,088,975.08

STATE: UTAH

ZIP CODE		QUARTER 1	QUARTER 2	QUARTER 3	QUARTER 4	TOTAL GRAMS
	840	12,689.87	12,550.55	12,461.52	13,668.60	51,370.54
	841	3,898.71	3,920.67	3,903.33	4,133.99	15,856.70
	843	795.47	760.78	834.78	970.05	3,361.08
	844	1,083.94	1,005.89	988.55	1,005.89	4,084.27
	845	126.03	112.73	97.12	143.37	479.25
	846	1,952.82	1,955.71	2,016.41	2,115.27	8,040.21
	847	851.54	805.29	811.65	945.19	3,413.67
TOTAL		21,398.38	21,111.62	21,113.36	22,982.36	86,605.72

STATE: VERMONT

ZIP CODE		QUARTER 1	QUARTER 2	QUARTER 3	QUARTER 4	TOTAL GRAMS
	50	275.75	308.71	258.99	327.2	1,170.65

DRUG CODE:1205DRUG NAME:LISDEXAMFETAMINE
STATE:VERMONT

ZIP CODE	QUARTER 1	QUARTER 2	QUARTER 3	QUARTER 4	TOTAL GRAMS
51	298.3	276.33	253.79	261.88	1,090.30
52	563.07	631.86	619.15	694.88	2,508.96
53	696.61	686.78	678.69	696.03	2,758.11
54	2,720.54	2,583.53	2,584.11	2,809.57	10,697.75
56	649.21	648.63	668.28	698.34	2,664.46
57	890.85	884.49	914.55	926.12	3,616.01
58	781.01	772.34	809.34	835.35	3,198.04
TOTAL	6,875.34	6,792.67	6,786.90	7,249.37	27,704.28

STATE: VIRGIN ISLANDS

ZIP CODE	QUARTER 1	QUARTER 2	QUARTER 3	QUARTER 4	TOTAL GRAMS
8	78.62	68.22	47.98	72.84	267.66
TOTAL	78.62	68.22	47.98	72.84	267.66

STATE: VIRGINIA

ZIP CODE	QUARTER 1	QUARTER 2	QUARTER 3	QUARTER 4	TOTAL GRAMS
201	6,943.56	7,169.02	6,920.44	7,476.57	28,509.59
220	3,616.59	3,726.43	3,355.29	3,389.98	14,088.29
221	2,911.31	2,813.61	2,869.11	2,899.17	11,493.20
222	1,976.52	2,223.37	2,030.29	2,063.24	8,293.42
223	1,744.13	1,879.40	1,890.97	1,892.12	7,406.62
224	2,108.91	2,033.18	2,012.94	2,162.67	8,317.70
225	1,005.32	1,020.35	976.99	1,063.70	4,066.36
226	1,497.86	1,429.06	1,325.01	1,483.40	5,735.33
227	496.01	506.99	519.71	541.68	2,064.39
228	1,219.79	1,002.43	1,023.24	1,123.25	4,368.71
229	2,284.65	2,220.48	2,147.06	2,353.45	9,005.64
230	1,668.97	1,444.09	1,495.54	1,596.13	6,204.73
231	4,690.70	4,757.18	4,227.65	4,605.72	18,281.25
232	5,434.14	5,330.66	5,102.89	5,473.45	21,341.14
233	3,262.22	3,210.77	2,947.15	3,027.51	12,447.65
234	7,249.37	7,145.89	6,751.05	6,800.77	27,947.08
235	1,628.51	1,733.72	1,696.15	1,656.26	6,714.64
236	3,102.08	2,972.01	3,029.24	3,043.12	12,146.45

	237	1,241.18	1,307.66	1,138.28	1,301.88	4,989
	238	2,201.40	2,180.02	1,982.88	2,068.21	8,432.51
	239	645.16	501.21	566.54	537.05	2,249.96
	240	3,248.92	3,130.99	2,872	3,412.52	12,664.43
	241	1,949.35	1,875.93	1,728.52	1,915.25	7,469.05
	242	970.05	865.42	896.63	918.6	3,650.70
	243	944.62	922.65	885.07	946.93	3,699.27
	244	1,425.02	1,355.07	1,345.82	1,335.99	5,461.90
	245	2,892.23	2,863.33	2,805.52	3,050.63	11,611.71
	246	679.27	542.84	535.32	563.65	2,321.08
TOTAL		69,037.84	68,163.76	65,077.30	68,702.90	270,981.80

STATE: WASHINGTON

ZIP CODE		QUARTER 1	QUARTER 2	QUARTER 3	QUARTER 4	TOTAL GRAMS
	980	9,739.25	9,952.57	9,669.18	9,888.31	39,249.31
	981	5,493.11	5,927.84	5,846.33	5,873.50	23,140.78
	982	3,336.79	3,399.23	3,603.88	3,526.99	13,866.89
	983	3,399.81	3,478.43	3,385.35	3,308.47	13,572.06
	984	2,076.54	2,073.07	1,987.51	1,962.07	8,099.19
	985	1,777.08	1,815.81	1,741.82	1,892.70	7,227.41
	986	2,270.20	2,322.23	2,201.40	2,336.68	9,130.51
	988	254.94	216.21	238.18	289.05	998.38
	989	488.49	569.43	539.37	647.47	2,244.76
	990	423.17	420.28	376.92	419.7	1,640.07
	991	265.93	234.71	301.19	337.03	1,138.86
	992	2,212.97	2,246.50	2,073.07	2,415.88	8,948.42
	993	1,659.15	1,681.69	1,666.66	1,745.28	6,752.78
	994	81.51	63.01	76.89	81.51	302.92
TOTAL		33,478.94	34,401.01	33,707.75	34,724.64	136,312.34

STATE: WEST VIRGINIA

ZIP CODE		QUARTER 1	QUARTER 2	QUARTER 3	QUARTER 4	TOTAL GRAMS
	247	378.66	346.86	367.09	375.19	1,467.80
	248	268.82	262.46	258.41	283.85	1,073.54
	249	379.81	373.45	351.48	389.06	1,493.80

	250	821.48	697.19	634.75	693.14	2,846.56
	251	577.52	506.99	540.52	535.9	2,160.93
	252	406.4	380.39	415.08	389.06	1,590.93
	253	2,006.59	1,788.64	1,779.39	1,784.59	7,359.21
	254	1,333.68	1,375.30	1,291.48	1,376.46	5,376.92
	255	1,784.59	1,749.91	1,625.62	1,885.18	7,045.30
	256	235.29	239.33	218.52	232.97	926.11
	257	1,638.34	1,516.36	1,488.03	1,553.93	6,196.66
	258	1,023.82	1,026.71	997.22	1,058.50	4,106.25
	259	360.73	299.46	294.25	311.02	1,265.46
	260	1,125.56	1,080.47	1,055.61	1,075.84	4,337.48
	261	2,132.61	2,018.73	1,942.42	2,002.54	8,096.30
	262	607.01	604.11	559.02	653.25	2,423.39
	263	1,371.25	1,383.39	1,319.80	1,360.27	5,434.71
	264	376.92	341.66	330.67	359.58	1,408.83
	265	2,277.71	1,966.12	2,012.94	2,138.97	8,395.74
	266	285	224.88	231.82	250.32	992.02
	267	683.31	741.7	715.69	729.56	2,870.26
	268	445.72	440.51	433.58	399.47	1,719.28
TOTAL		20,520.82	19,364.62	18,863.39	19,838.65	78,587.48

STATE:	WISCONSIN					
ZIP CODE		QUARTER 1	QUARTER 2	QUARTER 3	QUARTER 4	TOTAL GRAMS
	530	6,955.12	7,067.27	7,077.10	7,714.74	28,814.23
	531	7,632.08	7,828.05	7,852.33	8,776.14	32,088.60
	532	8,535.65	8,441.99	8,433.90	9,302.79	34,714.33
	534	2,296.79	2,263.26	2,162.67	2,494.50	9,217.22
	535	4,726.55	5,050.28	4,825.40	5,315.63	19,917.86
	537	2,826.91	2,836.74	2,958.14	3,159.32	11,781.11
	538	808.76	804.14	834.78	954.44	3,402.12
	539	1,858.59	1,914.67	1,888.65	1,996.18	7,658.09
	540	721.47	670.02	649.78	767.14	2,808.41
	541	3,609.66	3,584.22	3,644.92	3,940.33	14,779.13
	542	1,641.23	1,683.43	1,653.94	1,645.85	6,624.45
	543	5,074.56	5,378.64	5,283.83	5,551.49	21,288.52
	544	5,360.72	5,341.07	5,144.51	5,479.81	21,326.11

	545	1,238.29	1,173.54	1,303.04	1,215.17	4,930.04
	546	1,743.55	1,778.81	1,703.08	1,919.87	7,145.31
	547	1,367.21	1,321.54	1,286.27	1,471.84	5,446.86
	548	1,388.60	1,457.97	1,404.20	1,580.53	5,831.30
	549	7,261.51	7,218.73	7,318.17	7,979.51	29,777.92
TOTAL		65,047.25	65,814.37	65,424.71	71,265.28	267,551.61

STATE: WYOMING

ZIP CODE		QUARTER 1	QUARTER 2	QUARTER 3	QUARTER 4	TOTAL GRAMS
	820	580.99	603.54	633.6	628.97	2,447.10
	822	104.06	83.82	77.47	72.84	338.19
	823	35.26	31.8	24.86	27.75	119.67
	824	459.01	443.98	477.51	536.48	1,916.98
	825	111	60.12	84.4	61.86	317.38
	826	357.84	335.88	295.41	345.7	1,334.83
	827	291.94	276.91	279.22	256.1	1,104.17
	828	220.26	195.98	190.77	171.12	778.13
	829	391.37	382.12	335.3	412.19	1,520.98
	830	137.59	107.53	142.21	94.81	482.14
	831	12.14	37	27.75	13.87	90.76
TOTAL		2,701.46	2,558.68	2,568.50	2,621.69	10,450.33

DRUG CODE: 1724 DRUG NAME: METHYLPHENIDATE (DL;D;L;ISOMERS)

STATE: ALABAMA

ZIP CODE		QUARTER 1	QUARTER 2	QUARTER 3	QUARTER 4	TOTAL GRAMS
	350	9,161.26	8,435.17	8,416.40	9,317.56	35,330.39
	351	5,060.77	4,583.27	4,594.38	5,076.71	19,315.13
	352	9,560.83	8,226.96	8,098.49	8,932.20	34,818.48
	354	2,827.34	2,494.94	2,468.52	2,801.73	10,592.53
	355	2,057.54	2,119.26	1,967.89	2,251.21	8,395.90
	356	4,403.64	4,087.63	4,078.18	4,319.75	16,889.20
	357	2,672.42	2,482.29	2,540.19	2,608.52	10,303.42
	358	3,027.85	2,981.22	2,935.96	2,894.13	11,839.16
	359	4,164.49	3,679.80	3,707.55	4,106.04	15,657.88
	360	3,914.23	3,436.26	3,635.07	3,810.50	14,796.06

	361	3,058.49	2,971.62	2,890.99	2,882.54	11,803.64
	362	2,082.46	2,015.89	1,885.83	2,084.16	8,068.34
	363	6,071.56	5,600.51	5,391.28	5,856.94	22,920.29
	364	1,490.17	1,262.12	1,282.66	1,472.50	5,507.45
	365	6,891.06	6,275.41	6,260.52	6,850.85	26,277.84
	366	5,343	5,146.06	4,940.50	5,170.31	20,599.87
	367	671.41	540.53	588.27	665.01	2,465.22
	368	3,785.78	3,425.56	3,413.17	3,927.82	14,552.33
	369	197.55	174.73	177.29	186.58	736.15
TOTAL		76,441.85	69,939.23	69,273.14	75,215.06	290,869.28

STATE: ALASKA

ZIP CODE		QUARTER 1	QUARTER 2	QUARTER 3	QUARTER 4	TOTAL GRAMS
	995	4,385.48	4,337.54	4,266.74	4,395.24	17,385
	996	1,533.45	1,411.27	1,400.01	1,431.88	5,776.61
	997	1,415.27	1,203.83	1,269.01	1,305.07	5,193.18
	998	524.28	528.32	480.22	524.61	2,057.43
	999	449.77	457.7	396.97	422.31	1,726.75
TOTAL		8,308.25	7,938.66	7,812.95	8,079.11	32,138.97

STATE: AMERICAN SAMOA

ZIP CODE		QUARTER 1	QUARTER 2	QUARTER 3	QUARTER 4	TOTAL GRAMS
	967	2.59	6.05	4.32	4.76	17.72
TOTAL		2.59	6.05	4.32	4.76	17.72

STATE: ARIZONA

ZIP CODE		QUARTER 1	QUARTER 2	QUARTER 3	QUARTER 4	TOTAL GRAMS
	850	12,674.64	11,752.78	11,633.75	12,168.87	48,230.04
	851	3,733.77	3,606.55	4,046.76	4,052.18	15,439.26
	852	25,509.39	24,715.80	24,777.61	28,175.65	103,178.45
	853	12,650.88	11,348.09	11,221.77	11,877.47	47,098.21
	855	819.52	731.32	713.93	789.63	3,054.40
	856	3,098.53	2,694.97	2,557.20	2,746.76	11,097.46
	857	12,366.76	11,394.05	11,433.69	12,123.75	47,318.25

	859	828.97	761.45	798.21	739.94	3,128.57
	860	1,574.67	1,414.74	1,486.06	1,388.10	5,863.57
	863	2,115.81	2,044.57	1,879.15	2,205.66	8,245.19
	864	1,158.48	976.03	955.28	1,027.41	4,117.20
	865	311.98	300.74	349.51	387.47	1,349.70
TOTAL		76,843.40	71,741.09	71,852.92	77,682.89	298,120.30

STATE:	ARKANSAS					
ZIP CODE		QUARTER 1	QUARTER 2	QUARTER 3	QUARTER 4	TOTAL GRAMS
	716	1,691.17	1,425.22	1,517.44	1,641.14	6,274.97
	717	1,075.10	968.32	912.15	1,046.47	4,002.04
	718	1,091.60	985.17	950.16	955.58	3,982.51
	719	2,267.64	2,061.43	2,081.54	2,151.78	8,562.39
	720	6,507.87	6,102.11	5,955.99	6,418.92	24,984.89
	721	5,053.99	4,572.57	4,489.51	4,811.28	18,927.35
	722	4,476.63	4,052.98	4,095.46	4,238.70	16,863.77
	723	1,570.28	1,365.16	1,312.38	1,445.89	5,693.71
	724	4,157.76	3,379.63	3,351.49	3,668.83	14,557.71
	725	1,251.81	1,136.46	1,088.54	1,226.98	4,703.79
	726	1,535.92	1,317.23	1,375.66	1,353.38	5,582.19
	727	7,303.05	7,181.34	6,716.43	7,068.60	28,269.42
	728	1,540.58	1,394.98	1,290.67	1,436.42	5,662.65
	729	3,214.88	2,942.88	2,848.39	3,027.36	12,033.51
TOTAL		42,738.28	38,885.48	37,985.81	40,491.33	160,100.90

STATE:	CALIFORNIA					
ZIP CODE		QUARTER 1	QUARTER 2	QUARTER 3	QUARTER 4	TOTAL GRAMS
	900	10,209.38	10,222.70	10,051.88	10,540.13	41,024.09
	902	7,452.04	7,389.82	7,078.97	7,544.09	29,464.92
	903	396.6	482.04	345.21	415.31	1,639.16
	904	1,383.88	1,432.59	1,130.18	1,536.26	5,482.91
	905	1,374.39	1,361.06	1,127.23	1,157.36	5,020.04
	906	3,965.02	3,172.14	3,224.59	3,367	13,728.75
	907	3,489.71	3,526.11	3,162.86	3,498.31	13,676.99
	908	2,232.36	2,335.84	2,148.82	2,197.29	8,914.31
	910	1,463.52	1,561.19	1,397.90	1,500.26	5,922.87

911	2,011.74	2,014.24	2,154.49	2,325.17	8,505.64
912	956.45	1,029.31	963.56	983.76	3,933.08
913	12,460.26	12,134.13	11,856.17	12,167.19	48,617.75
914	2,798.67	2,819.43	2,672.01	2,796.15	11,086.26
915	840.56	834.67	774.99	849.01	3,299.23
916	825.31	773.85	693.73	814.7	3,107.59
917	8,532.29	8,437.93	7,830.01	8,466.61	33,266.84
918	239.68	223.74	235.31	266.62	965.35
919	2,657.02	2,779.33	2,475.68	2,791.50	10,703.53
920	28,871.62	27,565.48	26,836.29	29,386.35	112,659.74
921	11,815.40	11,520.47	11,053.14	11,823.78	46,212.79
922	4,642.07	4,257.95	3,826.10	4,432.21	17,158.33
923	7,783.63	7,616.34	7,174.54	7,810.92	30,385.43
924	844.85	830.64	798.58	841.04	3,315.11
925	9,237.73	9,276.17	7,867.55	9,149.07	35,530.52
926	12,364.70	11,923.46	11,485.25	12,024.84	47,798.25
927	3,105.90	2,866.14	2,938.86	3,125.76	12,036.66
928	7,931.58	7,886.57	7,648.69	8,078.30	31,545.14
930	6,125.24	6,006.09	5,996.03	5,788.31	23,915.67
931	1,592.40	1,648.08	1,513.51	1,552.16	6,306.15
932	3,441.34	3,118.61	3,078.55	3,375.15	13,013.65
933	3,083.14	2,891.72	2,798.86	2,895.44	11,669.16
934	3,619.09	3,388.01	3,172.83	3,281.67	13,461.60
935	3,424.96	3,361.87	3,387.89	3,385.04	13,559.76
936	3,864.47	3,727.37	3,487.68	3,931.27	15,010.79
937	5,438.25	5,452.37	4,707.94	5,097.53	20,696.09
939	2,289.70	2,107.07	2,014.63	2,124.17	8,535.57
940	5,424.77	5,465.80	5,229.20	5,577.43	21,697.20
941	6,099.54	6,327.67	5,818.78	6,463.91	24,709.90
943	1,187.03	1,230.51	1,038.51	1,154.88	4,610.93
944	1,191.84	1,126.99	1,135.94	1,068.82	4,523.59
945	19,319.78	18,557.26	17,727.88	18,720.63	74,325.55
946	3,101.03	3,087.88	2,873.16	2,994.28	12,056.35
947	770.92	854.04	797.82	987.96	3,410.74
948	693.28	586.8	640.64	676.17	2,596.89
949	3,887.74	3,943.73	3,607.36	4,075.49	15,514.32
950	7,080.76	6,577.84	6,374.56	6,947.83	26,980.99

	951	4,098.17	4,144.28	3,744.79	4,236.89	16,224.13
	952	3,296.68	3,015.26	2,783.26	2,833.30	11,928.50
	953	6,040.33	5,697.10	5,638.32	5,988.12	23,363.87
	954	3,757.84	3,675.01	3,653.13	3,268.56	14,354.54
	955	978.96	1,007.67	946.17	1,126.42	4,059.22
	956	13,866.13	12,664.13	13,070.65	12,962.23	52,563.14
	957	3,361.14	3,016.93	3,038.52	3,311.47	12,728.06
	958	5,941.85	5,903.21	5,524.66	5,700.79	23,070.51
	959	3,772.06	3,709.17	3,610.31	3,978.47	15,070.01
	960	2,956.02	2,743.86	2,639.55	2,993.49	11,332.92
	961	426.79	481.12	365.02	419.29	1,692.22
TOTAL		280,017.61	271,790.79	259,368.74	276,806.16	1,087,983.30

STATE: COLORADO

ZIP CODE		QUARTER 1	QUARTER 2	QUARTER 3	QUARTER 4	TOTAL GRAMS
	800	10,250.66	9,652.78	9,466.71	10,162.74	39,532.89
	801	11,279.12	10,999.97	10,710.12	11,293.66	44,282.87
	802	10,796.10	10,462.18	10,340.02	11,267.79	42,866.09
	803	1,973.84	1,951.01	1,784.69	2,008.84	7,718.38
	804	1,781.16	1,574.34	1,695.62	1,808.15	6,859.27
	805	6,674.71	6,544.62	6,488.51	7,059.72	26,767.56
	806	2,376.41	2,223.50	2,180.97	2,497.51	9,278.39
	807	408.04	400.1	445.86	490.76	1,744.76
	808	1,447.62	1,418.35	1,587.05	1,314.79	5,767.81
	809	8,199.44	7,907.60	7,743.07	7,713.09	31,563.20
	810	2,275.29	2,079.49	2,031.18	2,275.41	8,661.37
	811	333.24	280.4	272.12	283.9	1,169.66
	812	660.61	650.1	569.74	649.83	2,530.28
	813	514.94	527.22	545.9	520.8	2,108.86
	814	751.17	718.59	612.08	793.48	2,875.32
	815	2,066.14	2,002.86	1,961.49	2,148.07	8,178.56
	816	1,173.24	1,017.97	1,125.70	1,173.29	4,490.20
TOTAL		62,961.73	60,411.08	59,560.83	63,461.83	246,395.47

STATE: CONNECTICUT

ZIP CODE	QUARTER 1	QUARTER 2	QUARTER 3	QUARTER 4	TOTAL GRAMS
60	11,671.18	11,452.17	10,765.50	11,861.07	45,749.92
61	4,257.82	4,304.71	3,825.09	4,157.78	16,545.40
62	2,658.90	2,402.13	2,290.16	2,440.39	9,791.58
63	4,658.81	4,827.29	4,662.90	4,721.58	18,870.58
64	12,777.82	12,202.26	11,055.18	12,161.56	48,196.82
65	3,601.31	3,570.67	3,099.11	3,769.30	14,040.39
66	1,579.27	1,519.61	1,463.34	1,566.66	6,128.88
67	4,665.87	4,559.68	4,286.49	4,351.32	17,863.36
68	8,721.27	8,967.98	8,019.48	8,897.50	34,606.23
69	1,062.03	1,201.49	1,064.99	1,194.25	4,522.76
TOTAL	55,654.28	55,007.99	50,532.24	55,121.41	216,315.92

STATE: DELAWARE

ZIP CODE	QUARTER 1	QUARTER 2	QUARTER 3	QUARTER 4	TOTAL GRAMS
197	7,188.95	7,109.30	6,546.90	7,190.23	28,035.38
198	5,660.29	5,431.11	4,903.65	5,401.81	21,396.86
199	6,573.53	6,368.55	6,202.81	6,518.63	25,663.52
TOTAL	19,422.77	18,908.96	17,653.36	19,110.67	75,095.76

STATE: DISTRICT OF COLUMBIA

ZIP CODE	QUARTER 1	QUARTER 2	QUARTER 3	QUARTER 4	TOTAL GRAMS
200	7,364.24	7,699.14	6,667.48	7,396.72	29,127.58
203	308.75	126.94	331.19	378.56	1,145.44
204	476.45	450.08	548.31	452.15	1,926.99
TOTAL	8,149.44	8,276.16	7,546.98	8,227.43	32,200.01

STATE: FLORIDA

ZIP CODE	QUARTER 1	QUARTER 2	QUARTER 3	QUARTER 4	TOTAL GRAMS
320	8,790.46	8,289.69	7,480.66	8,455.25	33,016.06
321	5,479.50	5,095.65	4,620.43	5,019.06	20,214.64
322	14,034.63	12,371.23	11,660.22	12,823.21	50,889.29
323	5,454.22	4,862.46	4,675.48	5,155.33	20,147.49
324	6,714	6,192.87	5,833.93	6,343.86	25,084.66

	325	14,120.03	12,968.02	12,990.17	12,603.16	52,681.38
	326	6,215.67	5,602.05	5,225.13	5,944.78	22,987.63
	327	12,829.80	12,286.62	10,870.66	12,346.74	48,333.82
	328	8,690.02	8,233.54	7,354	8,139.28	32,416.84
	329	10,532.68	9,297.86	8,789.62	10,026.71	38,646.87
	330	10,848.15	10,203.93	7,987.05	9,962.47	39,001.60
	331	12,026.96	10,966.96	8,922.27	11,140.70	43,056.89
	333	5,064.82	4,725.16	4,026.88	4,922.07	18,738.93
	334	14,209.52	13,411.36	11,403.27	13,464.87	52,489.02
	335	10,195.09	9,758.19	8,894.22	9,701.85	38,549.35
	336	10,038.92	9,307.28	8,364.88	9,830.23	37,541.31
	337	7,735.35	7,107.83	6,517.37	7,531.50	28,892.05
	338	9,032.23	8,332.74	7,545.04	8,531.81	33,441.82
	339	9,495.59	8,811.85	7,761.85	9,300.20	35,369.49
	341	3,737.43	3,300.67	2,519.19	3,219.06	12,776.35
	342	10,019.59	9,512.45	8,441.46	9,385.20	37,358.70
	344	6,000.37	5,591.14	5,111.28	5,473.52	22,176.31
	346	8,799.20	7,928.68	7,544.39	7,888.98	32,161.25
	347	7,397.04	6,800.24	6,116.50	6,899.48	27,213.26
	349	5,284.84	4,822.06	4,240.80	5,240.95	19,588.65
TOTAL		222,746.11	205,780.53	184,896.75	209,350.27	822,773.66

STATE:	GEORGIA					
ZIP CODE		QUARTER 1	QUARTER 2	QUARTER 3	QUARTER 4	TOTAL GRAMS
	300	32,780.44	29,362.49	29,800	31,634.78	123,577.71
	301	20,112.09	17,914.74	18,238.81	19,762.47	76,028.11
	302	15,750.58	13,406.16	13,547.95	14,751.67	57,456.36
	303	13,045.71	12,007.49	11,951.14	12,900.86	49,905.20
	304	4,586.83	4,377.83	4,197.83	4,203.94	17,366.43
	305	10,681.46	9,397.92	9,174.30	9,844.60	39,098.28
	306	6,314.23	5,548.90	5,731.62	6,106.45	23,701.20
	307	6,878.02	6,123.02	6,042.93	6,686.07	25,730.04
	308	3,584.09	2,765.33	3,213.02	3,308	12,870.44
	309	5,318.44	4,714.61	4,876.84	5,062.80	19,972.69
	310	12,287.26	10,688.73	10,625.51	11,570.59	45,172.09
	312	4,099.79	3,678.90	3,913.03	4,147.74	15,839.46

313	5,584.61	4,743.66	4,928.94	5,240.90	20,498.11
314	4,180.07	3,763.71	3,697.64	4,065.73	15,707.15
315	9,268.35	7,916.26	8,028.97	8,518.32	33,731.90
316	4,307.34	3,510.49	3,541.69	4,097.07	15,456.59
317	9,232.57	7,751.06	7,935.79	8,520.12	33,439.54
318	622.89	501.36	492.07	649.63	2,265.95
319	5,260.62	4,729.13	5,036.25	4,886.96	19,912.96
398	1,613.69	1,406.23	1,433.09	1,522.09	5,975.10
TOTAL	175,509.08	154,308.02	156,407.42	167,480.79	653,705.31

STATE: GUAM

ZIP CODE	QUARTER 1	QUARTER 2	QUARTER 3	QUARTER 4	TOTAL GRAMS
969	562.07	394.32	633.74	324.62	1,914.75
TOTAL	562.07	394.32	633.74	324.62	1,914.75

STATE: HAWAII

ZIP CODE	QUARTER 1	QUARTER 2	QUARTER 3	QUARTER 4	TOTAL GRAMS
967	5,932.28	5,642.34	5,182.28	5,809.31	22,566.21
968	4,961.27	5,290.64	5,600.54	5,217.64	21,070.09
TOTAL	10,893.55	10,932.98	10,782.82	11,026.95	43,636.30

STATE: IDAHO

ZIP CODE	QUARTER 1	QUARTER 2	QUARTER 3	QUARTER 4	TOTAL GRAMS
832	2,814.52	2,670.72	2,514.78	2,775.20	10,775.22
833	2,721.89	2,691.20	2,471.35	2,697.27	10,581.71
834	3,475.36	3,452.25	3,208.10	3,405.98	13,541.69
835	821.99	794.34	731	816.83	3,164.16
836	9,011.63	8,771.92	8,270.97	8,820.35	34,874.87
837	6,470.51	6,627.44	6,096.62	6,876.04	26,070.61
838	2,666.14	2,764.18	2,504.17	2,644.36	10,578.85
TOTAL	27,982.04	27,772.05	25,796.99	28,036.03	109,587.11

STATE: ILLINOIS

ZIP CODE	QUARTER 1	QUARTER 2	QUARTER 3	QUARTER 4	TOTAL GRAMS
600	27,702.37	26,558.32	25,087.75	27,264.35	106,612.79
601	19,455.07	18,852.84	17,840.15	19,338.96	75,487.02
602	1,348.23	1,399.17	1,215.32	1,347.51	5,310.23
603	1,364.29	1,282.89	1,175.78	1,277.31	5,100.27
604	14,190.91	12,930.42	12,629.21	13,519.58	53,270.12
605	16,594.64	15,887.38	14,773.61	15,842.07	63,097.70
606	16,991.14	16,843.74	14,869.40	17,106.37	65,810.65
607	1,356.06	1,317.43	1,110.60	1,244.14	5,028.23
608	680.19	646.44	550.53	690.07	2,567.23
609	2,354.96	2,149.79	2,179.07	2,388.45	9,072.27
610	3,950.59	3,681.85	3,651.37	3,860.92	15,144.73
611	4,272.64	4,061.16	3,789.22	4,138.66	16,261.68
612	3,321.01	3,101.73	2,981.19	3,130.88	12,534.81
613	2,586.04	2,335.25	2,284.12	2,379.92	9,585.33
614	2,325.54	2,217.50	1,994.54	2,175.63	8,713.21
615	4,314.42	3,932.42	3,997.96	4,170.24	16,415.04
616	5,367.43	5,025.45	4,960.73	5,219.05	20,572.66
617	6,387.51	5,860.14	5,998.44	6,338.34	24,584.43
618	6,788.93	6,384.30	5,888.13	6,595.76	25,657.12
619	2,387.90	2,148.11	2,042.04	2,478.07	9,056.12
620	5,831.98	5,091.08	5,207.25	5,761.98	21,892.29
622	7,838.04	6,945.33	7,077.19	7,490.16	29,350.72
623	2,283.02	2,094.15	2,127.43	2,176.36	8,680.96
624	2,271.81	2,038.44	2,171.39	2,270.75	8,752.39
625	3,158.43	2,604.47	2,609.39	2,704.02	11,076.31
626	4,218.11	3,773.58	3,881.66	3,885.27	15,758.62
627	5,064.49	4,539.57	4,494.50	5,007.90	19,106.46
628	3,910.89	3,423.38	3,550.21	3,676.86	14,561.34
629	4,745.40	4,010.40	4,196.46	4,292.46	17,244.72
TOTAL	183,062.04	171,136.73	164,334.64	177,772.04	696,305.45

STATE: INDIANA

ZIP CODE	QUARTER 1	QUARTER 2	QUARTER 3	QUARTER 4	TOTAL GRAMS
460	14,089.61	12,897.94	12,931.52	13,888.78	53,807.85
461	11,559.76	10,774.40	10,448.41	11,226.63	44,009.20

	462	17,733.17	16,260.15	16,303.11	16,538.83	66,835.26
	463	9,991.41	9,536.88	9,182.13	9,795.27	38,505.69
	464	923.86	799.57	708.55	858.05	3,290.03
	465	9,134.39	8,397.91	7,878.03	8,279.19	33,689.52
	466	2,565.06	2,548.31	2,395.18	2,643.79	10,152.34
	467	5,243.64	4,726.91	4,573.06	4,851.75	19,395.36
	468	7,486.39	6,925.02	6,676.43	7,043.26	28,131.10
	469	5,286.85	4,969.14	4,735.14	5,166.53	20,157.66
	470	1,647.10	1,460.80	1,520.15	1,664.70	6,292.75
	471	7,881.05	8,823.19	7,789.15	6,740.26	31,233.65
	472	3,944.17	3,551.86	3,608.62	3,594.78	14,699.43
	473	5,826.24	5,110.13	5,196.34	5,140.20	21,272.91
	474	3,771.76	3,439.47	3,643.10	3,659.79	14,514.12
	475	3,416.83	2,730.67	2,823	2,892.48	11,862.98
	476	2,705.06	2,432.15	2,482.61	2,858.41	10,478.23
	477	5,853.05	5,153.65	5,296.97	5,504.49	21,808.16
	478	3,384.90	3,056.57	2,993.15	3,185.86	12,620.48
	479	6,060.76	5,425.83	5,276.90	5,778.93	22,542.42
TOTAL		128,505.06	119,020.55	116,461.55	121,311.98	485,299.14

STATE: IOWA

ZIP CODE	QUARTER 1	QUARTER 2	QUARTER 3	QUARTER 4	TOTAL GRAMS
500	6,914.61	6,373.60	6,131.84	6,799.48	26,219.53
501	3,862.64	3,801.39	3,704.67	3,841.20	15,209.90
502	4,840.53	4,778.18	4,508.95	4,699.13	18,826.79
503	8,294.83	7,942.46	7,316.76	7,634.38	31,188.43
504	2,111.32	2,011.19	1,914.38	2,017.85	8,054.74
505	2,333.37	2,248.23	2,146	2,276.56	9,004.16
506	3,301.48	3,194.30	3,018.75	3,189.48	12,704.01
507	2,056.92	2,042.68	1,823.75	1,895.52	7,818.87
508	638.53	524.71	551.3	628.53	2,343.07
510	2,159.49	2,089.79	1,981.82	2,216.22	8,447.32
511	4,031.32	4,005.95	3,523.10	3,852.04	15,412.41
512	1,174.37	938.68	963.89	1,077.43	4,154.37
513	1,070.86	1,066.58	1,012.98	1,028.74	4,179.16
514	1,006.79	945.73	964.13	993.61	3,910.26

	515	3,591.30	3,420.85	3,225.68	3,435.69	13,673.52
	516	528.61	494.58	466.33	510.43	1,999.95
	520	4,129.64	3,808.24	3,768.31	3,951.66	15,657.85
	521	1,164.14	1,037.37	1,138.13	1,107.32	4,446.96
	522	7,465.49	7,208.33	6,640.64	7,026.89	28,341.35
	523	4,053.20	3,929.50	3,705.80	4,187.41	15,875.91
	524	6,665	6,518.80	6,216.02	6,642.64	26,042.46
	525	2,431.97	2,222.97	2,079.12	2,282.52	9,016.58
	526	3,181.20	2,943.75	2,914.04	2,957.72	11,996.71
	527	4,767.76	4,441.09	4,229.68	4,572.23	18,010.76
	528	3,526.06	3,325.13	3,169.79	3,520.34	13,541.32
TOTAL		85,301.43	81,314.08	77,115.86	82,345.02	326,076.39

STATE:	KANSAS					
ZIP CODE		QUARTER 1	QUARTER 2	QUARTER 3	QUARTER 4	TOTAL GRAMS
	660	10,341.32	9,715.61	9,537.33	10,190.73	39,784.99
	661	1,344.29	1,240.22	1,077.36	1,254.88	4,916.75
	662	9,060.52	8,677.60	8,157.39	8,845.41	34,740.92
	664	1,703.39	1,510.85	1,791.67	1,498.25	6,504.16
	665	1,851.79	1,755.45	1,568.09	1,820.34	6,995.67
	666	5,750.58	5,641.77	5,335.29	5,404.63	22,132.27
	667	1,926.90	1,819.40	1,747.49	1,846.60	7,340.39
	668	807.55	750.56	694.56	720.71	2,973.38
	669	276.85	267.34	280.6	274.42	1,099.21
	670	2,868.64	2,968.55	2,843.22	2,780.68	11,461.09
	671	1,540.86	1,433.88	1,513.80	1,584.87	6,073.41
	672	6,939.78	6,406.41	6,153.12	6,525.37	26,024.68
	673	1,548.06	1,512.42	1,476.63	1,551.44	6,088.55
	674	2,114.07	1,866.14	1,819.18	1,976.15	7,775.54
	675	1,858.26	1,742.18	1,582.57	1,741.96	6,924.97
	676	1,069.50	968.36	943.33	1,053.38	4,034.57
	677	323.29	347.86	322.39	347.29	1,340.83
	678	884.5	883.68	833.99	902.74	3,504.91
	679	119.33	114.87	136.4	166.39	536.99
TOTAL		52,329.48	49,623.15	47,814.41	50,486.24	200,253.28

STATE: KENTUCKY

ZIP CODE	QUARTER 1	QUARTER 2	QUARTER 3	QUARTER 4	TOTAL GRAMS
400	4,553.38	4,193.77	4,123.80	4,136.79	17,007.74
401	1,943.50	1,893.48	2,041.85	1,818.31	7,697.14
402	15,014.92	13,169.87	13,160.57	13,849.07	55,194.43
403	6,302.88	5,910.80	5,904.27	6,086.81	24,204.76
404	2,380.81	2,190.26	2,014.71	2,228.09	8,813.87
405	5,751.75	4,956.51	5,281.95	5,651.27	21,641.48
406	1,084.53	1,009.76	944.98	1,020.29	4,059.56
407	1,808.40	1,607.42	1,534.94	1,691.14	6,641.90
408	406.69	365.15	345.82	329.69	1,447.35
409	633.22	566.94	576.07	604.58	2,380.81
410	10,725.63	9,823.63	9,412.40	10,556.60	40,518.26
411	2,262.41	2,032.11	1,965.03	2,177.50	8,437.05
412	808.02	703.77	682.31	697.69	2,891.79
413	398.75	352.3	387.77	393.06	1,531.88
414	320.03	305.89	282.52	309.65	1,218.09
415	1,166.56	1,085.71	1,159.66	1,225.98	4,637.91
416	651.77	538.48	602.36	619.14	2,411.75
417	1,025.37	891.63	848.6	899.04	3,664.64
418	235.35	273.68	283.77	288.51	1,081.31
420	4,888.27	4,255.24	4,424.36	4,636.70	18,204.57
421	3,214.28	2,818.25	3,017.34	3,167.17	12,217.04
422	3,086.43	2,329.28	3,225.93	2,500.47	11,142.11
423	3,029.72	2,762.02	2,590.65	2,796.22	11,178.61
424	3,736.19	3,448.18	3,375.02	3,800.68	14,360.07
425	1,005.02	887.84	836.67	1,145.43	3,874.96
426	637.76	558.94	625.29	729.3	2,551.29
427	2,885.74	2,633.04	2,776.41	2,862.31	11,157.50
TOTAL	79,957.38	71,563.95	72,425.05	76,221.49	300,167.87

STATE: LOUISIANA

ZIP CODE	QUARTER 1	QUARTER 2	QUARTER 3	QUARTER 4	TOTAL GRAMS
700	10,721.37	9,198.57	8,870.38	9,867.30	38,657.62
701	4,809.75	4,300.68	4,108.35	4,791.81	18,010.59

	703	5,247.83	4,485.31	4,358.12	4,623.12	18,714.38
	704	10,669.09	9,413.51	9,145.08	10,037.74	39,265.42
	705	14,506.78	11,539.41	12,188.45	13,519.16	51,753.80
	706	9,139.90	7,582.95	7,474.18	8,155.89	32,352.92
	707	10,712.30	9,164.39	9,438.20	10,438.40	39,753.29
	708	7,989.68	6,667.66	6,749.88	7,466.19	28,873.41
	710	1,976.36	1,666.23	1,763.02	2,064.32	7,469.93
	711	5,357.42	4,733	4,466.94	4,891.96	19,449.32
	712	5,323.33	4,519.17	4,606.46	5,077.61	19,526.57
	713	3,941.09	3,310.57	3,370.04	3,830.31	14,452.01
	714	2,817.04	2,290.34	2,695.61	2,638.17	10,441.16
TOTAL		93,211.94	78,871.79	79,234.71	87,401.98	338,720.42

STATE: MAINE

ZIP CODE		QUARTER 1	QUARTER 2	QUARTER 3	QUARTER 4	TOTAL GRAMS
	39	743.55	742.18	790.65	699.5	2,975.88
	40	7,783.99	7,807.19	7,733.55	7,627.75	30,952.48
	41	2,887.36	2,961.21	2,914.33	2,977.35	11,740.25
	42	3,236.98	3,120.45	2,907.68	3,207.46	12,472.57
	43	2,652.56	2,849.68	2,787.66	2,715.59	11,005.49
	44	6,744.14	6,455.98	6,164.10	6,465.26	25,829.48
	45	1,328.13	1,314.51	1,273.07	1,307.49	5,223.20
	46	2,020.29	1,956.39	1,982.23	2,048.10	8,007.01
	47	2,186.48	2,095.50	1,810.11	2,030.92	8,123.01
	48	1,147.72	1,252.25	1,242.19	1,150.67	4,792.83
	49	4,977.73	5,169.78	5,005.06	4,950.03	20,102.60
TOTAL		35,708.93	35,725.12	34,610.63	35,180.12	141,224.80

STATE: MARYLAND

ZIP CODE		QUARTER 1	QUARTER 2	QUARTER 3	QUARTER 4	TOTAL GRAMS
	206	4,394.87	3,845.12	3,706.63	3,890.24	15,836.86
	207	7,077.91	7,090.35	6,293.47	6,451.98	26,913.71
	208	15,914.19	15,509.18	14,473	15,800.60	61,696.97
	209	2,779.33	2,725.99	2,668.17	2,653.85	10,827.34
	210	17,456.93	17,128.60	15,573.82	17,318.05	67,477.40

211	8,814.42	8,398.21	7,681.10	8,627.15	33,520.88
212	20,715.10	20,047.12	18,214.30	20,263.68	79,240.20
214	2,053.58	1,930.34	1,728.49	1,898.44	7,610.85
215	3,614.88	3,748.20	3,444.20	3,582.97	14,390.25
216	4,128.04	4,025.62	3,664.30	3,840.95	15,658.91
217	12,157.43	11,520	10,864.46	11,622.10	46,163.99
218	3,700.59	3,287.41	3,052.48	3,358.68	13,399.16
219	1,889.01	1,795.86	1,750	2,026.94	7,461.81
TOTAL	104,696.28	101,052	93,114.42	101,335.63	400,198.33

STATE: MASSACHUSETTS

ZIP CODE	QUARTER 1	QUARTER 2	QUARTER 3	QUARTER 4	TOTAL GRAMS
10	10,696.65	10,812.48	10,048.63	10,574.22	42,131.98
11	4,630.97	4,540.66	3,784.24	4,490.52	17,446.39
12	4,428.59	4,286.89	3,992.21	4,396.11	17,103.80
13	2,166.20	2,081.19	2,113.34	2,089.39	8,450.12
14	6,176.46	6,193.40	5,767.51	5,890.35	24,027.72
15	8,351.34	7,967.56	7,777.96	8,192.24	32,289.10
16	4,678.74	4,442.30	4,041.47	4,469.38	17,631.89
17	10,737.19	11,049.44	9,752.55	10,783.77	42,322.95
18	16,054.06	16,104.72	14,322.75	15,340.88	61,822.41
19	11,945.15	11,738.55	10,622.94	11,755.43	46,062.07
20	10,240.17	10,113.76	9,083.04	10,132.71	39,569.68
21	20,996.92	21,486.97	19,808.94	21,908.75	84,201.58
22	1,216.59	1,158.78	782.09	1,017.86	4,175.32
23	9,473.26	8,970.71	8,450.54	9,138.49	36,033
24	11,568.60	11,820.92	10,667.24	11,942.44	45,999.20
25	3,043.92	3,061.48	2,968.55	2,975.96	12,049.91
26	3,898.22	3,959.74	4,158.11	4,062.84	16,078.91
27	9,420.18	9,691.30	8,689.68	9,453.08	37,254.24
TOTAL	149,723.21	149,480.85	136,831.79	148,614.42	584,650.27

STATE: MICHIGAN

ZIP CODE	QUARTER 1	QUARTER 2	QUARTER 3	QUARTER 4	TOTAL GRAMS
480	25,111.36	24,199.28	22,158.43	24,259.70	95,728.77

	481	28,053.25	26,412.76	24,636.99	27,948.24	107,051.24
	482	5,624.20	5,426.41	4,579.84	5,387.08	21,017.53
	483	19,612.91	19,325.40	18,162.22	19,785.52	76,886.05
	484	6,722.05	6,610.79	6,020.22	6,906.12	26,259.18
	485	2,665.24	2,577.78	2,269.66	2,386	9,898.68
	486	7,634.85	7,454.12	7,009.19	7,315.87	29,414.03
	487	4,524.13	4,318.12	3,868.12	4,349.18	17,059.55
	488	12,981.63	12,354.69	11,760.95	12,970.57	50,067.84
	489	3,104.34	3,031.06	2,879.86	3,029.44	12,044.70
	490	14,042.78	13,395.76	12,976.79	13,899.57	54,314.90
	491	1,230.47	1,150.32	1,056.07	1,025.01	4,461.87
	492	6,004.54	5,507.74	5,506.89	6,022.77	23,041.94
	493	6,366.75	5,977.68	5,945.37	6,091.85	24,381.65
	494	18,020.11	17,049.84	15,998.55	17,553.27	68,621.77
	495	13,843.45	13,191.30	12,242.55	13,203.97	52,481.27
	496	7,405.14	7,113.64	7,021.67	7,231.88	28,772.33
	497	5,594.58	5,456.06	5,383.87	5,305.16	21,739.67
	498	6,576.51	6,385.74	6,353.84	6,559.10	25,875.19
	499	1,511.69	1,390.17	1,401.84	1,453.83	5,757.53
TOTAL		196,629.98	188,328.66	177,232.92	192,684.13	754,875.69

STATE: MINNESOTA

ZIP CODE		QUARTER 1	QUARTER 2	QUARTER 3	QUARTER 4	TOTAL GRAMS
	550	11,192.38	10,961.66	10,422.48	11,432.66	44,009.18
	551	15,086.57	14,592.36	13,771.80	15,475.54	58,926.27
	553	21,128.21	20,204.76	18,859.36	21,092.47	81,284.80
	554	20,216	20,102.71	18,539.43	20,787.54	79,645.68
	556	216.85	167.5	184.53	175.83	744.71
	557	2,306.59	2,145.72	2,125.12	2,434.79	9,012.22
	558	2,606.19	2,434.30	2,331.63	2,606.64	9,978.76
	559	8,028.16	8,053.74	7,633.88	8,390.54	32,106.32
	560	4,778.17	4,659.90	4,458.46	4,618.03	18,514.56
	561	1,448.76	1,385.71	1,337.86	1,447.65	5,619.98
	562	2,305.19	2,233.60	2,089.41	2,276.69	8,904.89
	563	6,182.01	5,963.94	5,479.34	6,064.84	23,690.13
	564	2,152.94	2,078.75	2,004.56	2,200.06	8,436.31

	565	3,721.45	3,555.36	3,456.10	3,878.39	14,611.30
	566	1,022.61	1,128.05	943.24	1,000.60	4,094.50
	567	989.41	895.8	864.35	1,011.61	3,761.17
TOTAL		103,381.49	100,563.86	94,501.55	104,893.88	403,340.78

STATE: MISSISSIPPI

ZIP CODE		QUARTER 1	QUARTER 2	QUARTER 3	QUARTER 4	TOTAL GRAMS
	386	6,538.18	5,558.31	5,920.57	6,272.18	24,289.24
	387	631.97	506.44	605.83	571.65	2,315.89
	388	3,225.61	2,849.45	2,823.37	3,135.53	12,033.96
	389	1,155.63	1,042.58	1,001.66	1,132.75	4,332.62
	390	3,954.67	3,545.09	3,537.98	3,705.87	14,743.61
	391	4,101.81	3,486.17	3,535.60	3,555.50	14,679.08
	392	3,519.37	3,125.52	3,209.05	3,464.69	13,318.63
	393	2,822.32	2,565.60	2,501.34	2,749.05	10,638.31
	394	7,190.23	6,125.16	6,290.16	6,730.88	26,336.43
	395	9,636.51	8,213.61	8,477.45	9,077.15	35,404.72
	396	2,035.58	1,720.01	1,720.74	1,819.34	7,295.67
	397	1,652.68	1,355.88	1,492.44	1,430.56	5,931.56
TOTAL		46,464.56	40,093.82	41,116.19	43,645.15	171,319.72

STATE: MISSOURI

ZIP CODE		QUARTER 1	QUARTER 2	QUARTER 3	QUARTER 4	TOTAL GRAMS
	630	17,140.75	15,786.42	15,589.33	17,353.11	65,869.61
	631	45,187.48	42,161.05	45,910.07	48,018.76	181,277.36
	633	11,682.21	10,474.82	10,493.13	11,617.86	44,268.02
	634	1,395.50	1,383.65	1,281.37	1,343.49	5,404.01
	635	749.88	692.07	659.52	716.37	2,817.84
	636	2,252.05	2,209.74	2,064.06	2,240.11	8,765.96
	637	2,153.21	1,936.61	1,940.84	2,112.32	8,142.98
	638	2,409.83	2,054.49	2,085.47	2,430.76	8,980.55
	639	965.82	850.76	784.12	773.69	3,374.39
	640	10,054.42	9,618.55	9,284.59	10,068.10	39,025.66
	641	12,135.52	11,756.34	11,358.29	12,089.84	47,339.99
	644	1,077.13	968.88	919.15	1,048.22	4,013.38

	645	2,036.98	1,902.20	1,924.44	1,933.26	7,796.88
	646	596.73	543.95	529.93	572.87	2,243.48
	647	1,006.12	927.83	981.84	1,053.03	3,968.82
	648	3,153.84	2,953.08	2,897.11	3,091.51	12,095.54
	650	1,410.91	1,288.43	1,304.83	1,425.80	5,429.97
	651	1,763.56	1,590.51	1,566.11	1,618.79	6,538.97
	652	4,013.86	3,605.60	3,587.63	3,894.97	15,102.06
	653	1,280.99	1,208.53	1,219.52	1,123.51	4,832.55
	654	1,576.43	1,428.33	1,574.27	1,466.21	6,045.24
	655	1,549.08	1,463.20	1,391.70	1,384.88	5,788.86
	656	1,662.12	1,580.08	1,656.45	1,680.38	6,579.03
	657	3,609.27	3,445.95	3,419.88	3,447.27	13,922.37
	658	4,320.40	4,312.97	4,066.67	4,051.40	16,751.44
TOTAL		135,184.09	126,144.04	128,490.32	136,556.51	526,374.96

STATE:	MONTANA				
ZIP CODE	QUARTER 1	QUARTER 2	QUARTER 3	QUARTER 4	TOTAL GRAMS
590	1,056.93	807.39	946.4	946.61	3,757.33
591	4,457.82	4,194.62	3,892.82	4,128.81	16,674.07
592	658.65	545.99	585.97	626.06	2,416.67
593	806.22	731.88	706.4	741.61	2,986.11
594	2,599.61	2,453.46	2,324.19	2,439.07	9,816.33
595	373.59	351.83	347.9	335.51	1,408.83
596	1,330.35	1,219.30	1,196.20	1,286.68	5,032.53
597	2,551.15	2,555	2,508.39	2,580.21	10,194.75
598	3,185.29	3,071.54	3,092.23	3,002.27	12,351.33
599	1,328.25	1,244.61	1,358.27	1,406.78	5,337.91
TOTAL	18,347.86	17,175.62	16,958.77	17,493.61	69,975.86

STATE:	NEBRASKA				
ZIP CODE	QUARTER 1	QUARTER 2	QUARTER 3	QUARTER 4	TOTAL GRAMS
680	3,418.53	3,273.88	3,142.56	3,242.18	13,077.15
681	12,992.39	12,181.43	11,631.17	12,841.65	49,646.64
683	936.58	934.47	885.16	888.03	3,644.24
684	760.14	754.32	735.4	668.47	2,918.33

685	6,079.33	5,641.24	5,623.48	5,836.69	23,180.74
686	784.52	686.61	677.12	805.67	2,953.92
687	1,638.50	1,411.70	1,464.06	1,560.89	6,075.15
688	2,541.93	2,364.49	2,299.66	2,452.08	9,658.16
689	1,214.93	1,082.34	1,047.70	1,156.18	4,501.15
690	314.4	282.68	252.16	278.42	1,127.66
691	1,320.32	1,112.53	1,145.91	1,130.32	4,709.08
692	49.25	72.06	22.86	40.68	184.85
693	975.72	817.59	815.47	854.5	3,463.28
TOTAL	33,026.54	30,615.34	29,742.71	31,755.76	125,140.35

STATE: NEVADA

ZIP CODE	QUARTER 1	QUARTER 2	QUARTER 3	QUARTER 4	TOTAL GRAMS
890	3,271.20	3,154.43	3,135.03	3,373.52	12,934.18
891	7,245.65	6,873.89	6,287.29	6,830.92	27,237.75
893	28.79	27.41	28.45	25.34	109.99
894	1,600.46	1,677.76	1,510.88	1,667.71	6,456.81
895	2,355.30	2,182.47	2,176.33	2,243.50	8,957.60
897	474.68	460.72	485.52	495.84	1,916.76
898	430.89	422.14	353.97	421.19	1,628.19
TOTAL	15,406.97	14,798.82	13,977.47	15,058.02	59,241.28

STATE: NEW HAMPSHIRE

ZIP CODE	QUARTER 1	QUARTER 2	QUARTER 3	QUARTER 4	TOTAL GRAMS
30	8,586.96	8,615.94	8,059.43	8,698.29	33,960.62
31	5,283.71	5,391.13	4,938.43	5,011.45	20,624.72
32	3,246.35	3,220.45	3,183.71	3,199.49	12,850
33	2,306.91	2,169.36	2,193.93	2,221.47	8,891.67
34	1,992.72	2,232.18	1,974.29	2,056.86	8,256.05
35	1,750.93	1,961.06	1,706.04	1,778.44	7,196.47
36	108.35	126.29	130.22	111.37	476.23
37	3,278.82	3,322.71	3,071.33	3,403.98	13,076.84
38	7,855.36	7,697.51	7,324.38	7,430.10	30,307.35
TOTAL	34,410.11	34,736.63	32,581.76	33,911.45	135,639.95

STATE: NEW JERSEY

ZIP CODE	QUARTER 1	QUARTER 2	QUARTER 3	QUARTER 4	TOTAL GRAMS
70	16,536.10	16,440.24	14,547.20	16,476.76	64,000.30
71	1,987.73	1,780.36	1,590.30	1,861.64	7,220.03
72	1,091.77	1,007.62	899.08	958.32	3,956.79
73	1,120.78	1,102.32	953.88	977.99	4,154.97
74	5,508.84	5,111.87	4,500.91	5,246.84	20,368.46
75	1,052.81	955.1	842.07	889.87	3,739.85
76	4,927.11	4,877.05	3,968.49	4,798.82	18,571.47
77	6,581.40	6,213.08	5,795.21	6,417.06	25,006.75
78	4,976	4,960.23	4,451.94	4,667.55	19,055.72
79	4,938.93	4,718.32	4,501.52	4,911.93	19,070.70
80	18,279.24	17,353.24	16,116.39	17,379.24	69,128.11
81	1,672	1,637.20	1,316.27	1,556.50	6,181.97
82	2,789.98	2,681.01	2,790.22	2,771.31	11,032.52
83	2,499.24	2,436.85	2,045.85	2,435.23	9,417.17
84	217.88	198.26	214.83	212.9	843.87
85	4,166.33	4,156.27	3,833.62	4,094.62	16,250.84
86	3,088.52	2,923.02	2,788.08	3,012.34	11,811.96
87	4,908.75	4,913.10	4,529.38	4,899.13	19,250.36
88	9,040.92	8,902.67	7,684.57	8,704.96	34,333.12
89	878.11	849.04	770.51	887.72	3,385.38
TOTAL	96,262.44	93,216.85	84,140.32	93,160.73	366,780.34

STATE: NEW MEXICO

ZIP CODE	QUARTER 1	QUARTER 2	QUARTER 3	QUARTER 4	TOTAL GRAMS
870	1,540.19	1,495.40	1,411.24	1,450.44	5,897.27
871	9,261.90	8,581.27	8,683.35	8,352.82	34,879.34
873	580.83	486.9	540.96	579.23	2,187.92
874	1,616.84	1,526.76	1,383.11	1,406.68	5,933.39
875	1,919.46	1,899.94	1,877.54	1,899.17	7,596.11
877	269.53	305.69	290.95	299.88	1,166.05
878	122.1	126.09	118.12	135.2	501.51
879	84.05	78.79	119.92	86.76	369.52
880	1,804.04	1,703.67	1,704.25	1,691.27	6,903.23

	881	532.68	408.65	354.56	418.27	1,714.16
	882	1,667.39	1,586.96	1,627.62	1,740.35	6,622.32
	883	527.2	552.49	463.92	566.85	2,110.46
	884	94.12	108.48	97.75	88.76	389.11
TOTAL		20,020.33	18,861.09	18,673.29	18,715.68	76,270.39

STATE: NEW YORK

ZIP CODE		QUARTER 1	QUARTER 2	QUARTER 3	QUARTER 4	TOTAL GRAMS
	100	21,492.48	22,215.87	19,529.45	21,921.89	85,159.69
	101	1,413.86	1,258.19	1,073.32	1,318.34	5,063.71
	102	117.7	162.69	141.56	128.19	550.14
	103	2,704.18	2,610.14	2,266.59	2,721.93	10,302.84
	104	8,180.02	8,250.37	6,942.63	7,847.02	31,220.04
	105	10,713.65	10,326.44	9,274.78	10,182.34	40,497.21
	106	1,192.57	1,227.06	1,051.85	1,199.93	4,671.41
	107	1,959.55	1,940.26	1,646.08	1,984.71	7,530.60
	108	999.51	977.61	912.11	960.91	3,850.14
	109	7,157.53	6,897.03	6,228.60	6,724.51	27,007.67
	110	2,929.86	2,918.88	2,554.90	2,806.08	11,209.72
	111	1,115.95	1,189.72	996.58	1,205.51	4,507.76
	112	12,541.73	12,561.70	10,429.42	11,866.55	47,399.40
	113	4,889.04	5,011.21	4,045.01	5,033.13	18,978.39
	114	2,627.97	2,481.26	1,919.45	2,382	9,410.68
	115	8,662.71	8,549.54	7,656.59	8,495.36	33,364.20
	116	865.48	833.45	686.85	831.66	3,217.44
	117	21,082.74	20,253.17	18,160.80	20,379.77	79,876.48
	118	1,386.54	1,351.45	1,206.56	1,352.87	5,297.42
	119	2,749.16	2,889.63	2,818.64	2,741.54	11,198.97
	120	7,789.82	7,511.86	6,964.89	7,728.51	29,995.08
	121	4,778.41	4,849.29	4,294.06	4,834.74	18,756.50
	122	2,879.35	2,665.11	2,591.22	2,907.72	11,043.40
	123	3,250.53	3,306.83	2,950.86	3,174.89	12,683.11
	124	1,781.58	1,857.07	1,807.56	1,918.34	7,364.55
	125	5,395.25	5,397.91	4,629.63	5,256.87	20,679.66
	126	1,100.39	1,105.85	926.08	1,231.28	4,363.60
	127	1,059.53	1,246.39	1,031.59	1,026.75	4,364.26

	128	5,016.45	5,021.28	4,609.41	5,034.40	19,681.54
	129	4,024.96	3,945.93	3,758.58	4,180.72	15,910.19
	130	6,330.80	6,415.33	5,927.36	6,506.02	25,179.51
	131	2,333.46	2,339.73	1,986.41	2,314.08	8,973.68
	132	3,552.69	3,540.55	3,239.84	3,636.59	13,969.67
	133	2,001.54	1,986.60	1,804.64	2,066.61	7,859.39
	134	2,601.64	2,730.55	2,511.09	2,579.02	10,422.30
	135	1,043.62	1,082.11	958.12	1,020.15	4,104
	136	5,740.21	5,721.72	5,699.25	5,572.65	22,733.83
	137	1,400.38	1,372.61	1,138.09	1,366.13	5,277.21
	138	2,545.18	2,464.76	2,312.85	2,575.21	9,898
	139	1,271.89	1,130.74	973.63	1,172.55	4,548.81
	140	5,840.44	5,735.78	5,437.63	5,968.72	22,982.57
	141	2,833.62	2,887.75	2,636.20	2,739.21	11,096.78
	142	7,477.91	7,404.61	7,085.50	7,756.33	29,724.35
	143	628.39	604.59	607.52	669.09	2,509.59
	144	7,762.32	7,600.52	7,129.23	7,704.70	30,196.77
	145	8,090.46	8,096.18	7,595.74	7,771.65	31,554.03
	146	12,713.74	12,280.29	11,537.99	12,554.63	49,086.65
	147	2,214.19	2,218.18	1,995.66	2,213.90	8,641.93
	148	5,396.06	5,275.46	4,922.28	5,496.74	21,090.54
	149	1,320.82	1,311.19	1,195.38	1,247.70	5,075.09
TOTAL		234,957.86	233,012.44	209,800.06	232,310.14	910,080.50

STATE: NORTH CAROLINA

ZIP CODE		QUARTER 1	QUARTER 2	QUARTER 3	QUARTER 4	TOTAL GRAMS
	270	5,347.53	4,729.80	4,373.23	4,930.23	19,380.79
	271	5,801.83	5,109.41	4,868.43	5,521.21	21,300.88
	272	11,940.38	11,242.68	10,305.83	11,315.21	44,804.10
	273	7,595.65	6,882.43	6,128.73	6,867.53	27,474.34
	274	6,156.92	5,611.18	5,449.20	5,486.10	22,703.40
	275	25,913.54	24,308.45	22,580.13	24,637.95	97,440.07
	276	9,765.52	9,014.17	8,489.32	9,064.09	36,333.10
	277	6,095.73	6,022.85	5,659.28	6,038.51	23,816.37
	278	10,033.17	8,748.20	8,421.84	9,318.31	36,521.52
	279	2,573.30	2,550.91	2,164.54	2,630.78	9,919.53

	ZIP CODE	QUARTER 1	QUARTER 2	QUARTER 3	QUARTER 4	TOTAL GRAMS
	280	19,201.70	17,638.76	16,003.66	17,903.19	70,747.31
	281	14,830.84	13,388.42	12,348.56	13,806.58	54,374.40
	282	14,011.10	13,173.14	11,974.27	14,002.90	53,161.41
	283	17,635.59	15,936.71	14,817.50	15,904	64,293.80
	284	9,552.92	8,954.21	7,734.92	8,687.68	34,929.73
	285	12,630.15	11,988.27	11,266.90	10,813.42	46,698.74
	286	12,064.47	11,152.39	10,348.70	11,321.38	44,886.94
	287	9,245.32	8,533.79	8,162.40	8,831.31	34,772.82
	288	4,170.53	3,951.81	3,687.15	3,916.05	15,725.54
	289	750.41	806.64	698.2	639.64	2,894.89
TOTAL		205,316.60	189,744.22	175,482.79	191,636.07	762,179.68

STATE: NORTH DAKOTA

ZIP CODE		QUARTER 1	QUARTER 2	QUARTER 3	QUARTER 4	TOTAL GRAMS
	580	1,450.21	1,458.94	1,365.79	1,533.25	5,808.19
	581	3,674.74	3,556.80	3,298.05	3,520.11	14,049.70
	582	2,437.98	2,325.69	2,238.03	2,317.92	9,319.62
	583	1,143.84	972.02	970.93	972.19	4,058.98
	584	719.88	663.34	615.92	663.49	2,662.63
	585	3,023.03	2,714.86	2,716.90	3,141.48	11,596.27
	586	734.6	729.22	678.16	713.27	2,855.25
	587	1,802.17	1,751.75	1,653.63	1,792.39	6,999.94
	588	707.01	698.35	663.02	709.63	2,778.01
TOTAL		15,693.46	14,870.97	14,200.43	15,363.73	60,128.59

STATE: OHIO

ZIP CODE		QUARTER 1	QUARTER 2	QUARTER 3	QUARTER 4	TOTAL GRAMS
	430	17,332.65	16,053.31	15,442.04	16,775.34	65,603.34
	431	8,352.38	8,065.09	7,596.62	8,276.50	32,290.59
	432	40,131.49	37,467.16	42,200.52	46,052.29	165,851.46
	433	2,848.10	2,849.32	2,697.17	2,836.56	11,231.15
	434	2,862.05	2,700.28	2,619.66	2,841.30	11,023.29
	435	7,048.03	6,202.97	6,115.83	6,443.44	25,810.27
	436	8,101.27	7,577.81	7,226.65	7,358.55	30,264.28
	437	2,718	2,494.03	2,406.14	2,543.28	10,161.45

	438	428.48	563.17	558.54	475.68	2,025.87
	439	2,312.62	2,207.20	2,026.23	2,106.12	8,652.17
	440	14,280.30	13,460.02	12,523.41	14,058.56	54,322.29
	441	16,749.30	15,826.44	15,043.63	16,195.84	63,815.21
	442	8,537.39	7,901.79	7,928.47	8,163.59	32,531.24
	443	4,256.22	3,864.25	3,725.59	3,866.40	15,712.46
	444	3,818.23	3,687.54	3,425.86	3,727.82	14,659.45
	445	3,051.83	2,889.61	2,704.47	2,940.84	11,586.75
	446	6,410.24	5,817.78	5,813	6,241.29	24,282.31
	447	5,003.73	4,475.86	4,434.39	4,709.05	18,623.03
	448	4,793.69	4,197.03	4,252.97	4,484.37	17,728.06
	449	1,676.70	1,533.46	1,347.58	1,488.75	6,046.49
	450	16,851.57	15,417.48	15,103.85	16,417.80	63,790.70
	451	6,878.56	6,148.64	6,184.71	6,610.11	25,822.02
	452	20,998.37	19,307.71	18,377.16	20,758.17	79,441.41
	453	8,014.14	7,586.48	7,235.89	8,176.20	31,012.71
	454	9,972.49	9,466.86	8,898.73	9,441.29	37,779.37
	455	2,777	2,415.70	2,436.50	2,533.96	10,163.16
	456	6,877.73	6,326.05	6,361.71	6,779.51	26,345
	457	2,628.01	2,236.94	2,318.12	2,566.04	9,749.11
	458	5,358.81	5,091.06	4,722.91	5,397.68	20,570.46
TOTAL		241,069.38	223,831.04	221,728.35	240,266.33	926,895.10

STATE:	OKLAHOMA					
ZIP CODE		QUARTER 1	QUARTER 2	QUARTER 3	QUARTER 4	TOTAL GRAMS
	730	9,707.97	9,098.98	8,987.46	9,618.02	37,412.43
	731	7,160.20	6,628.87	6,654.63	7,058.33	27,502.03
	734	650.08	611.8	682.13	635.67	2,579.68
	735	3,626.02	3,401.31	3,475.03	2,999.04	13,501.40
	736	759.87	690.89	693.25	817.29	2,961.30
	737	1,326.94	1,110.31	1,126.22	1,242.17	4,805.64
	738	190.45	162.12	207.85	188.06	748.48
	739	103.67	48.39	55.61	55.21	262.88
	740	11,279.12	9,967.10	9,916.91	10,889.57	42,052.70
	741	6,502.51	5,885.16	5,810.75	5,926.96	24,125.38
	743	1,878.95	1,638.12	1,735.97	1,879.81	7,132.85

	744	3,093.25	2,682.67	3,039.49	3,042.11	11,857.52
	745	945.61	827.47	726.11	767.01	3,266.20
	746	729.35	633.19	660.62	763.7	2,786.86
	747	791.46	689.67	671.33	795.76	2,948.22
	748	1,706.14	1,552.14	1,531.90	1,621.11	6,411.29
	749	936.2	763.62	712.6	745.3	3,157.72
TOTAL		51,387.79	46,391.81	46,687.86	49,045.12	193,512.58

STATE: OREGON

ZIP CODE		QUARTER 1	QUARTER 2	QUARTER 3	QUARTER 4	TOTAL GRAMS
	970	12,889.34	12,893.73	11,599.11	12,565.74	49,947.92
	971	5,241.58	5,507.11	5,103.33	5,569.05	21,421.07
	972	15,798.87	16,616.18	15,549.64	16,192.79	64,157.48
	973	11,533.67	11,573.52	10,364.94	10,980.14	44,452.27
	974	7,381.19	7,326.44	6,808.83	7,130.31	28,646.77
	975	3,201.75	3,141.60	2,958.02	3,228.84	12,530.21
	976	665.48	641.08	606.27	653.17	2,566
	977	3,255.04	3,388.50	3,093.79	3,274.52	13,011.85
	978	1,798.69	1,751.80	1,569.54	1,905.90	7,025.93
	979	562.37	510.22	523.74	548.4	2,144.73
TOTAL		62,327.98	63,350.18	58,177.21	62,048.86	245,904.23

STATE: PENNSYLVANIA

ZIP CODE		QUARTER 1	QUARTER 2	QUARTER 3	QUARTER 4	TOTAL GRAMS
	150	7,925.16	7,547.51	6,972.05	7,586.98	30,031.70
	151	6,674.83	6,407.39	6,037.05	6,670.25	25,789.52
	152	12,436.52	11,748.21	10,863.74	11,916.89	46,965.36
	153	3,655.46	3,446.85	3,276.07	3,315.26	13,693.64
	154	1,610.95	1,505.60	1,418.48	1,530.41	6,065.44
	155	1,087.15	1,048.28	1,119.43	1,133.56	4,388.42
	156	5,392.92	5,161.79	4,948.83	5,259.73	20,763.27
	157	2,404.06	2,367.21	2,253.68	2,287.89	9,312.84
	158	2,219.69	2,127.84	2,145.79	2,156.05	8,649.37
	159	2,225.53	2,247.33	2,016.08	2,221.61	8,710.55
	160	3,991.18	3,656.04	3,729.83	3,787.36	15,164.41

	161	4,074.78	3,905.03		3,716.42	4,030.86	15,727.09
	162	1,752.41	1,547.09		1,469.27	1,577.21	6,345.98
	163	2,518.81	2,353.54		2,100.79	2,280.44	9,253.58
	164	1,198.15	1,078.94		1,161.43	1,183.85	4,622.37
	165	4,797.41	4,621.58		4,339.09	4,477.37	18,235.45
	166	3,873.90	3,646.45		3,358.61	3,731.46	14,610.42
	167	771.4	754.1		749.56	829.08	3,104.14
	168	3,337.31	3,126.39		3,288.88	3,150.79	12,903.37
	169	790.34	749.84		700.04	718.22	2,958.44
	170	15,911.07	14,764.73		14,565.48	15,403.46	60,644.74
	171	3,127.57	3,059.25		2,794.75	3,130.26	12,111.83
	172	4,035.27	3,732.71		3,623.45	3,767.48	15,158.91
	173	6,825.74	6,499.15		5,877.39	6,383.93	25,586.21
	174	4,804.17	4,913.56		4,396.63	4,847.50	18,961.86
	175	5,199.17	5,000.91		4,779.95	4,870.68	19,850.71
	176	4,639.80	4,560.60		4,259.44	4,384.13	17,843.97
	177	3,006.43	2,947.96		2,738.72	3,026.62	11,719.73
	178	3,512.76	3,429.54		3,173.57	3,581.26	13,697.13
	179	1,439.11	1,392.87		1,265.37	1,265.05	5,362.40
	180	6,047.87	5,975.95		5,671.98	5,822.20	23,518
	181	3,094.71	2,926.70		2,830.71	2,912.53	11,764.65
	182	1,400.64	1,313.18		1,156.81	1,397.34	5,267.97
	183	1,504.55	1,477.29		1,425.50	1,615.06	6,022.40
	184	2,223.63	2,122.78		2,061.21	2,238.57	8,646.19
	185	2,598.78	2,521.21		2,453.07	2,630.23	10,203.29
	186	2,388.58	2,252.64		1,965.19	2,302.33	8,908.74
	187	21,679.25	25,213.04		25,890.09	28,674.36	101,456.74
	188	1,034.65	998.19		1,023.31	1,053.55	4,109.70
	189	6,221.09	5,803.88		5,447.47	6,039.74	23,512.18
	190	18,505.54	17,926.45		17,052.87	17,891.22	71,376.08
	191	13,693.46	13,342.14		11,929.72	13,785.29	52,750.61
	193	9,191.35	8,783.61		8,399.69	9,111.26	35,485.91
	194	8,657.94	8,431.33		8,118.89	8,643.23	33,851.39
	195	2,527.26	2,430.46		2,356.19	2,567.79	9,881.70
	196	3,790.63	3,758.84		3,473.21	3,803.67	14,826.35
TOTAL		229,798.98	224,625.98		214,395.78	230,994.01	899,814.75

STATE: PUERTO RICO

ZIP CODE	QUARTER 1	QUARTER 2	QUARTER 3	QUARTER 4	TOTAL GRAMS
6	5,504.94	4,176.44	3,558.11	3,489.28	16,728.77
7	7,153.45	5,488.30	4,081.09	3,636.42	20,359.26
9	9,873.94	8,198.92	6,410.97	6,292.94	30,776.77
TOTAL	22,532.33	17,863.66	14,050.17	13,418.64	67,864.80

STATE: RHODE ISLAND

ZIP CODE	QUARTER 1	QUARTER 2	QUARTER 3	QUARTER 4	TOTAL GRAMS
28	12,735.71	13,165.48	11,892.83	12,809.09	50,603.11
29	6,304.49	6,384.24	5,770.50	6,236.93	24,696.16
TOTAL	19,040.20	19,549.72	17,663.33	19,046.02	75,299.27

STATE: SOUTH CAROLINA

ZIP CODE	QUARTER 1	QUARTER 2	QUARTER 3	QUARTER 4	TOTAL GRAMS
290	7,502.84	6,642.26	6,351.30	7,067.53	27,563.93
291	6,225.30	5,607.08	5,218.87	5,749.86	22,801.11
292	6,906.67	6,042.33	6,277.32	6,162.62	25,388.94
293	7,948.24	7,131.80	7,019.13	7,852.10	29,951.27
294	19,877.30	17,810.55	17,669.92	19,312.20	74,669.97
295	11,965.68	10,673.47	10,312.30	11,198.43	44,149.88
296	20,608.20	18,544.67	17,910.25	19,764.99	76,828.11
297	7,698.83	6,800.71	6,602.60	7,366.84	28,468.98
298	3,348.03	2,937.34	2,697.97	3,104.92	12,088.26
299	3,160.56	2,754.48	2,986.74	2,959.95	11,861.73
TOTAL	95,241.65	84,944.69	83,046.40	90,539.44	353,772.18

STATE: SOUTH DAKOTA

ZIP CODE	QUARTER 1	QUARTER 2	QUARTER 3	QUARTER 4	TOTAL GRAMS
570	2,748.79	2,503.21	2,462.63	2,618.44	10,333.07
571	13,967.95	10,191.14	12,911.46	14,211.90	51,282.45
572	1,091.78	1,006.29	1,003.09	1,118.04	4,219.20
573	1,821.48	1,513.61	1,653.98	1,723.56	6,712.63

	574	1,178.47	1,158.80	1,125.22	1,142.37	4,604.86
	575	948.5	841.9	891.01	942.9	3,624.31
	576	209.08	167.2	173.8	177.26	727.34
	577	3,019.16	2,797.93	2,747.61	2,916.60	11,481.30
TOTAL		24,985.21	20,180.08	22,968.80	24,851.07	92,985.16

STATE:	TENNESSEE					
ZIP CODE		QUARTER 1	QUARTER 2	QUARTER 3	QUARTER 4	TOTAL GRAMS
	370	13,202.28	12,023.41	12,222.70	12,591.06	50,039.45
	371	8,851.90	8,458.74	8,038.71	8,773.20	34,122.55
	372	6,234.62	5,664.03	5,511.56	5,873.17	23,283.38
	373	8,202.75	7,604.27	7,553	8,239.40	31,599.42
	374	4,014.57	3,374.73	3,439.03	3,423.71	14,252.04
	376	5,151.61	4,720.58	4,731.06	4,993.29	19,596.54
	377	4,905.12	4,522.40	4,552.80	4,721.48	18,701.80
	378	7,049.75	6,562.54	6,707.80	7,109.45	27,429.54
	379	6,631.83	6,149.37	5,935.75	6,523.70	25,240.65
	380	6,694.60	5,730.54	5,935.58	6,061.46	24,422.18
	381	7,931.67	7,261.14	7,307.20	7,684.88	30,184.89
	382	1,667.54	1,444.87	1,542.15	1,492.76	6,147.32
	383	3,782.91	3,266.70	3,515.50	3,628.19	14,193.30
	384	2,149.66	2,018.96	1,878.81	1,945.78	7,993.21
	385	2,224.31	2,103.86	2,023.21	2,085.18	8,436.56
TOTAL		88,695.12	80,906.14	80,894.86	85,146.71	335,642.83

STATE:	TEXAS					
ZIP CODE		QUARTER 1	QUARTER 2	QUARTER 3	QUARTER 4	TOTAL GRAMS
	750	37,816.45	34,580.49	30,803.86	32,122	135,322.80
	751	9,702.23	8,922.55	8,308.14	8,893.24	35,826.16
	752	12,081.40	11,712.06	10,308.33	11,846.52	45,948.31
	753	15.91	12.88	20.58	33.72	83.09
	754	5,641.70	5,080.66	4,882.98	5,515.57	21,120.91
	755	1,593.80	1,375.52	1,273.69	1,426.04	5,669.05
	756	4,316.40	4,102.66	3,894.61	4,349.17	16,662.84
	757	5,373.97	5,117.54	4,858.70	5,207	20,557.21

758	1,291.96	1,140.41	1,111.14	1,367.29	4,910.80
759	4,585.90	4,187.07	3,812.75	4,405.62	16,991.34
760	19,234.20	17,608.14	16,780.17	17,924.23	71,546.74
761	11,029.85	10,602.12	9,320.83	10,754.86	41,707.66
762	8,149.83	7,431.80	7,393.14	8,151.84	31,126.61
763	3,977.42	3,684.11	3,844.27	3,583.97	15,089.77
764	1,418.37	1,293.93	1,243.60	1,421.31	5,377.21
765	9,760.11	9,573.44	9,532.33	9,274.30	38,140.18
766	1,446.56	1,283.63	1,278.30	1,428.66	5,437.15
767	4,267.94	4,014.31	3,680.95	4,291.75	16,254.95
768	1,494.99	1,341.51	1,231.52	1,425.78	5,493.80
769	2,739.17	2,638.69	2,305.61	2,808.51	10,491.98
770	25,685.28	25,106.46	20,876.30	24,793.55	96,461.59
772	3.46	1.73	4.32	0.86	10.37
773	18,968.01	17,356.57	15,315.36	17,959.91	69,599.85
774	18,345.33	17,135.67	14,319.58	17,252.26	67,052.84
775	18,177.38	15,885.32	14,103.94	16,430.64	64,597.28
776	4,153.96	3,823.84	3,012.65	3,684.57	14,675.02
777	1,874.32	1,555.08	1,352.94	1,603.87	6,386.21
778	4,963.45	4,694.31	4,396.45	4,706.60	18,760.81
779	2,367.19	2,227.64	1,971.72	2,337.55	8,904.10
780	6,092.74	5,709.12	5,206.09	5,912.44	22,920.39
781	6,484.17	5,803.91	5,632.65	6,000.11	23,920.84
782	26,487.69	24,668.86	23,781.95	25,294.86	100,233.36
783	3,412.07	3,033.60	2,698.83	3,080.82	12,225.32
784	6,041.18	5,547.24	5,015.67	5,390.85	21,994.94
785	12,439.35	11,093.07	10,448.98	11,815.33	45,796.73
786	13,252.23	12,212.78	11,540.92	13,531.98	50,537.91
787	13,339.23	12,625.03	11,788.17	13,097.86	50,850.29
788	1,573.15	1,405.96	1,313.43	1,424.73	5,717.27
789	648.12	551.97	668.24	645.44	2,513.77
790	1,494.67	1,307.70	1,199.92	1,456.97	5,459.26
791	2,425.38	2,405.36	2,226.95	2,447.54	9,505.23
792	313.95	284.66	297.48	340.04	1,236.13
793	1,161.79	1,070	1,024.52	1,156.48	4,412.79
794	5,310.22	5,237.55	4,842.01	5,231.99	20,621.77
795	1,104.54	1,049.97	1,084.80	1,146.53	4,385.84

	796	3,641.10	3,449.05	2,966.62	3,273.03	13,329.80
	797	4,578.67	4,266.79	4,012.20	4,442.87	17,300.53
	798	276.58	245.47	201.56	196.61	920.22
	799	8,857.38	8,203.81	8,007.53	7,617.61	32,686.33
TOTAL		359,410.75	333,662.04	305,197.28	338,505.28	1,336,775.35

STATE: UTAH

ZIP CODE		QUARTER 1	QUARTER 2	QUARTER 3	QUARTER 4	TOTAL GRAMS
	840	24,645.26	23,494.80	22,752.54	24,528.92	95,421.52
	841	9,216.31	8,839.17	8,595.55	9,049.94	35,700.97
	843	2,220.30	2,104.38	2,005.49	2,314.82	8,644.99
	844	2,811.59	2,713.48	2,582.80	2,853.78	10,961.65
	845	563.06	509.15	512.41	514.15	2,098.77
	846	4,938.24	4,597.42	4,360.63	4,696.20	18,592.49
	847	3,356.15	3,009.04	3,007.10	3,368.28	12,740.57
TOTAL		47,750.91	45,267.44	43,816.52	47,326.09	184,160.96

STATE: VERMONT

ZIP CODE		QUARTER 1	QUARTER 2	QUARTER 3	QUARTER 4	TOTAL GRAMS
	50	1,555.46	1,636.53	1,315.32	1,445.10	5,952.41
	51	790	880.22	798.03	852.02	3,320.27
	52	1,257.68	1,238.29	1,301.11	1,315.21	5,112.29
	53	1,164.39	1,200.30	1,194.69	1,281.78	4,841.16
	54	6,413.43	6,308.38	6,172.16	6,679.94	25,573.91
	56	3,521.61	3,513.80	3,400.69	3,443.07	13,879.17
	57	2,525.43	2,488.16	2,540.25	2,401.13	9,954.97
	58	2,023.54	2,109.98	1,939.70	2,219.95	8,293.17
TOTAL		19,251.54	19,375.66	18,661.95	19,638.20	76,927.35

STATE: VIRGIN ISLANDS

ZIP CODE		QUARTER 1	QUARTER 2	QUARTER 3	QUARTER 4	TOTAL GRAMS
	8	210	180.55	99.13	107.51	597.19
TOTAL		210	180.55	99.13	107.51	597.19

DATE RANGE: 01/01/2017 TO 12/31/2017 | ARCOS 3 - REPORT 1 | Run Date: 07/03/2018

RETAIL DRUG DISTRIBUTION BY ZIP CODE WITHIN STATE BY GRAMS WT

STATE: VIRGINIA

ZIP CODE	QUARTER 1	QUARTER 2	QUARTER 3	QUARTER 4	TOTAL GRAMS
201	12,950.80	11,934.74	11,613.73	12,562.60	49,061.87
220	9,669.13	9,161.77	8,906.60	8,538.82	36,276.32
221	5,909.88	5,692.87	5,328.43	6,084.89	23,016.07
222	2,808.94	2,943.80	2,638.54	2,711.88	11,103.16
223	3,410.84	3,216.69	3,097.55	3,321.97	13,047.05
224	4,726.86	4,616.67	4,163.32	4,624.52	18,131.37
225	2,311.14	2,399.63	2,189.37	2,275.76	9,175.90
226	3,066.63	2,861.91	2,803.78	3,002.06	11,734.38
227	927.03	888.33	837.39	993.06	3,645.81
228	2,157.23	1,906.88	1,835.64	2,127.43	8,027.18
229	6,264.18	5,923.02	5,495.29	6,061.54	23,744.03
230	3,864.80	3,709.85	3,458.53	3,840.51	14,873.69
231	8,456.95	8,625.66	7,301.92	8,492.20	32,876.73
232	10,030.21	9,723.57	8,793.09	9,793.95	38,340.82
233	6,292.79	6,448.77	5,377.03	5,750.43	23,869.02
234	12,423.86	12,267.52	10,738.09	11,982.98	47,412.45
235	3,265.54	3,495.31	2,916.35	3,326.55	13,003.75
236	8,265.88	7,686	7,252.76	7,214.03	30,418.67
237	3,306.26	3,110.94	3,116.68	2,941.05	12,474.93
238	5,284.91	5,001.99	4,298.07	5,016.48	19,601.45
239	1,231.24	1,117.31	1,246.57	1,360.40	4,955.52
240	5,963.96	5,457.88	5,360.06	5,801.32	22,583.22
241	3,504.27	3,341.05	3,161.99	3,686.84	13,694.15
242	2,625.17	2,356.96	2,277.76	2,426.81	9,686.70
243	1,910.49	1,827.06	1,689.94	1,900.32	7,327.81
244	2,592.99	2,538.56	2,444.89	2,692.50	10,268.94
245	6,168.59	5,748.66	5,775.86	5,922.65	23,615.76
246	992.71	902.53	886	1,035.98	3,817.22
TOTAL	140,383.28	134,905.93	125,005.23	135,489.53	535,783.97

STATE: WASHINGTON

ZIP CODE	QUARTER 1	QUARTER 2	QUARTER 3	QUARTER 4	TOTAL GRAMS
980	24,450.66	24,686.77	22,586.62	24,583.06	96,307.11

	981	12,850.13	13,229.23	12,331.70	13,434.65	51,845.71
	982	15,646.24	15,821.80	14,441.39	15,633.49	61,542.92
	983	12,544.26	12,514.69	11,552.82	12,226.06	48,837.83
	984	8,218.95	8,133.18	7,661.01	7,378.51	31,391.65
	985	7,361.37	7,245.98	6,703.06	7,404.30	28,714.71
	986	9,271.48	9,457.73	8,797.92	9,076.93	36,604.06
	988	2,546.93	2,626.66	2,288.91	2,570.10	10,032.60
	989	3,531.78	3,489.60	2,991.30	3,499.63	13,512.31
	990	1,811.15	1,735.95	1,732.45	1,942.43	7,221.98
	991	1,286.42	1,225.84	1,203.96	1,272.52	4,988.74
	992	8,251.36	8,213.21	7,525.95	8,188.63	32,179.15
	993	5,500.34	5,695.43	4,816.07	5,495.04	21,506.88
	994	515.18	562.41	512.01	581.27	2,170.87
TOTAL		113,786.25	114,638.48	105,145.17	113,286.62	446,856.52

STATE: WEST VIRGINIA

ZIP CODE		QUARTER 1	QUARTER 2	QUARTER 3	QUARTER 4	TOTAL GRAMS
	247	1,066.97	1,001.32	929.46	1,055.32	4,053.07
	248	452.45	407.79	431.35	408.42	1,700.01
	249	793.58	725.05	679.93	674.92	2,873.48
	250	1,191.92	1,059.08	1,019.90	1,114.21	4,385.11
	251	974.91	895.4	892.08	988.95	3,751.34
	252	823.85	763.56	667.6	754.5	3,009.51
	253	3,124.01	2,933.73	2,703.09	2,769.05	11,529.88
	254	3,232.72	2,965.08	2,962.49	3,008.45	12,168.74
	255	3,354.40	2,948.77	2,934.76	3,062.98	12,300.91
	256	762.63	670.96	699.95	735.36	2,868.90
	257	2,529.03	2,355.92	2,448.76	2,435.17	9,768.88
	258	2,294.74	2,218.79	2,055.66	2,093.42	8,662.61
	259	828.18	760.4	782.28	792.75	3,163.61
	260	2,213.89	1,903.48	1,973.91	1,990.34	8,081.62
	261	2,027.45	2,062.72	1,877.11	1,953.42	7,920.70
	262	894.27	873.52	829.45	979.76	3,577
	263	1,474.33	1,353.63	1,393	1,381.21	5,602.17
	264	479.51	482.05	450.37	498.15	1,910.08
	265	3,110.85	2,767.61	2,892.80	3,148.25	11,919.51

266	415.01	391.71	426.44	405.26	1,638.42
267	890.18	814.56	748.96	800.07	3,253.77
268	621.62	544.13	541.17	530.51	2,237.43
TOTAL	33,556.50	30,899.26	30,340.52	31,580.47	126,376.76

STATE: WISCONSIN

ZIP CODE	QUARTER 1	QUARTER 2	QUARTER 3	QUARTER 4	TOTAL GRAMS
530	12,503.68	12,184.28	11,911.43	13,005.27	49,604.66
531	11,274.08	10,940.33	10,161.70	11,282.45	43,658.56
532	12,172.92	11,434.21	10,772.87	11,559.31	45,939.31
534	2,381.45	2,223.07	2,099.56	2,219.18	8,923.26
535	10,728.68	10,553.77	9,507.98	10,595.26	41,385.69
537	6,767.53	6,397.71	6,119.76	6,466.38	25,751.38
538	930.72	903.7	813.06	1,050.59	3,698.07
539	3,714.14	3,484.39	3,208.81	3,664.07	14,071.41
540	2,467.63	2,480.45	2,320.40	2,381.29	9,649.77
541	3,947.60	3,920.34	3,848.70	3,988.37	15,705.01
542	2,136.21	2,134.02	2,067.68	2,137.20	8,475.11
543	3,801.18	3,601.28	3,516.05	3,717.73	14,636.24
544	7,788.01	7,422.14	7,037.13	7,605.08	29,852.36
545	1,587.44	1,575.06	1,567.32	1,580.85	6,310.67
546	5,524.63	5,108.76	4,993.63	5,409.04	21,036.06
547	5,755.74	5,442.96	5,061.03	5,602.30	21,862.03
548	2,785.99	2,837.29	2,557.94	2,820.77	11,001.99
549	10,842.89	10,398.16	9,871.44	10,990.63	42,103.12
TOTAL	107,110.52	103,041.92	97,436.49	106,075.77	413,664.70

STATE: WYOMING

ZIP CODE	QUARTER 1	QUARTER 2	QUARTER 3	QUARTER 4	TOTAL GRAMS
820	2,345.31	2,184.91	2,208.88	2,270.66	9,009.76
822	308.45	260.47	244.43	255.45	1,068.80
823	131.41	111.72	95.15	101.21	439.49
824	878.35	747.08	877.12	794.82	3,297.37
825	548.5	506.04	399.74	476.29	1,930.57
826	1,710.32	1,586.53	1,497	1,539.41	6,333.26

	827	712.95	664.17	609.94	716.72	2,703.78
	828	653.68	615.53	588.83	613.52	2,471.56
	829	833.83	783.54	777.52	834.78	3,229.67
	830	362.48	325.16	294.86	311.61	1,294.11
	831	257.42	162.13	201.37	176.68	797.6
TOTAL		8,742.70	7,947.28	7,794.84	8,091.15	32,575.97

DRUG CODE: 2012 DRUG NAME: GAMMA HYDROXYBUTYRIC ACID(FDA APPROV

STATE: CALIFORNIA

ZIP CODE	QUARTER 1	QUARTER 2	QUARTER 3	QUARTER 4	TOTAL GRAMS
908	594.43	520.13	520.13	445.82	2,080.51
928	817.34	817.34	817.34	817.34	3,269.36
940	148.61	0	0	2,526.34	2,674.95
TOTAL	1,560.38	1,337.47	1,337.47	3,789.50	8,024.82

STATE: ILLINOIS

ZIP CODE	QUARTER 1	QUARTER 2	QUARTER 3	QUARTER 4	TOTAL GRAMS
606	520.13	743.04	668.74	743.04	2,674.95
TOTAL	520.13	743.04	668.74	743.04	2,674.95

STATE: LOUISIANA

ZIP CODE	QUARTER 1	QUARTER 2	QUARTER 3	QUARTER 4	TOTAL GRAMS
700	297.22	148.61	0	0	445.83
TOTAL	297.22	148.61	0	0	445.83

STATE: MASSACHUSETTS

ZIP CODE	QUARTER 1	QUARTER 2	QUARTER 3	QUARTER 4	TOTAL GRAMS
21	0	1,560.38	297.22	817.34	2,674.94
TOTAL	0	1,560.38	297.22	817.34	2,674.94

STATE: MICHIGAN

ZIP CODE	QUARTER 1	QUARTER 2	QUARTER 3	QUARTER 4	TOTAL GRAMS

	481	594.43	0	297.22	445.82	1,337.47
TOTAL		594.43	0	297.22	445.82	1,337.47

STATE:		MISSOURI				
ZIP CODE		QUARTER 1	QUARTER 2	QUARTER 3	QUARTER 4	TOTAL GRAMS
	631	9,221,200.70	5,490,842.69	8,990,338.18	6,709,576.90	30,411,958.47
TOTAL		9,221,200.70	5,490,842.69	8,990,338.18	6,709,576.90	30,411,958.47

STATE:		NEW YORK				
ZIP CODE		QUARTER 1	QUARTER 2	QUARTER 3	QUARTER 4	TOTAL GRAMS
	104	0	0	2,972.16	4,978.37	7,950.53
TOTAL		0	0	2,972.16	4,978.37	7,950.53

STATE:		NORTH CAROLINA				
ZIP CODE		QUARTER 1	QUARTER 2	QUARTER 3	QUARTER 4	TOTAL GRAMS
	277	222.91	817.34	594.43	668.74	2,303.42
	280	0	594.43	1,411.78	668.74	2,674.95
TOTAL		222.91	1,411.77	2,006.21	1,337.48	4,978.37

STATE:		OHIO				
ZIP CODE		QUARTER 1	QUARTER 2	QUARTER 3	QUARTER 4	TOTAL GRAMS
	441	0	817.34	817.34	817.34	2,452.02
	452	1,188.86	1,263.17	1,114.56	1,411.78	4,978.37
TOTAL		1,188.86	2,080.51	1,931.90	2,229.12	7,430.39

STATE:		SOUTH CAROLINA				
ZIP CODE		QUARTER 1	QUARTER 2	QUARTER 3	QUARTER 4	TOTAL GRAMS
	292	0	1,337.47	371.52	743.04	2,452.03
	296	965.95	1,411.78	0	2,749.25	5,126.98
TOTAL		965.95	2,749.25	371.52	3,492.29	7,579.01

DATE RANGE: 01/01/2017 TO 12/31/2017 ARCOS 3 - REPORT 1 Run Date: 07/03/2018

RETAIL DRUG DISTRIBUTION BY ZIP CODE WITHIN STATE BY GRAMS WT

STATE: TENNESSEE

ZIP CODE	QUARTER 1	QUARTER 2	QUARTER 3	QUARTER 4	TOTAL GRAMS
381	594.43	594.43	594.43	594.43	2,377.72
TOTAL	594.43	594.43	594.43	594.43	2,377.72

STATE: TEXAS

ZIP CODE	QUARTER 1	QUARTER 2	QUARTER 3	QUARTER 4	TOTAL GRAMS
752	1,486.08	1,263.17	520.13	0	3,269.38
770	817.34	222.91	520.13	668.74	2,229.12
782	1,040.26	594.43	1,188.86	743.04	3,566.59
787	817.34	891.65	0	371.52	2,080.51
TOTAL	4,161.02	2,972.16	2,229.12	1,783.30	11,145.60

STATE: VIRGINIA

ZIP CODE	QUARTER 1	QUARTER 2	QUARTER 3	QUARTER 4	TOTAL GRAMS
235	0	2,229.12	1,634.69	2,229.12	6,092.93
TOTAL	0	2,229.12	1,634.69	2,229.12	6,092.93

STATE: WASHINGTON

ZIP CODE	QUARTER 1	QUARTER 2	QUARTER 3	QUARTER 4	TOTAL GRAMS
981	371.52	817.34	594.43	445.82	2,229.11
TOTAL	371.52	817.34	594.43	445.82	2,229.11

DRUG CODE: 2100 DRUG NAME: BARBITURIC ACID DERIVIATIVE OR SALT [PER

STATE: ALABAMA

ZIP CODE	QUARTER 1	QUARTER 2	QUARTER 3	QUARTER 4	TOTAL GRAMS
350	250	180	205	150	785
351	75	80	60	50	265
352	195	135	100	170	600
354	60	30	55	70	215
355	15	35	50	45	145
356	170	85	95	80	430

357	80	75	5	35	195
358	100	75	80	55	310
359	175	135	130	110	550
360	80	35	10	65	190
361	30	20	20	15	85
362	95	65	60	80	300
363	100	60	75	45	280
364	75	70	80	50	275
365	130	125	160	230	645
366	70	65	70	55	260
367	15	20	20	20	75
368	150	125	145	120	540
TOTAL	1,865	1,415	1,420	1,445	6,145

STATE: ALASKA

ZIP CODE	QUARTER 1	QUARTER 2	QUARTER 3	QUARTER 4	TOTAL GRAMS
995	60	65	60	55	240
996	95	60	55	60	270
998	50	40	40	30	160
999	20	10	10	5	45
TOTAL	225	175	165	150	715

STATE: ARIZONA

ZIP CODE	QUARTER 1	QUARTER 2	QUARTER 3	QUARTER 4	TOTAL GRAMS
850	790	1,085	865	750	3,490
851	405	270	300	405	1,380
852	1,665	1,495	1,295	1,380	5,835
853	1,150	900	780	695	3,525
855	95	90	35	10	230
856	165	130	75	125	495
857	910	740	700	645	2,995
859	85	100	105	25	315
860	25	5	5	20	55
863	195	205	155	175	730
864	335	360	215	290	1,200

TOTAL	5,820	5,380	4,530	4,520	20,250

STATE: ARKANSAS

ZIP CODE	QUARTER 1	QUARTER 2	QUARTER 3	QUARTER 4	TOTAL GRAMS
716	120	100	160	100	480
717	70	40	55	55	220
718	40	45	45	30	160
719	275	190	95	80	640
720	255	120	170	180	725
721	140	180	155	190	665
722	165	130	90	55	440
723	55	80	50	95	280
724	130	110	90	80	410
725	80	75	75	70	300
726	40	15	10	5	70
727	110	95	130	75	410
728	25	0	10	10	45
729	135	155	130	60	480
TOTAL	1,640	1,335	1,265	1,085	5,325

STATE: CALIFORNIA

ZIP CODE	QUARTER 1	QUARTER 2	QUARTER 3	QUARTER 4	TOTAL GRAMS
900	365	305	360	350	1,380
902	585	585	630	620	2,420
903	40	20	35	35	130
904	25	5	10	45	85
905	145	125	60	110	440
906	305	360	385	330	1,380
907	355	395	370	330	1,450
908	140	235	205	145	725
910	70	100	155	50	375
911	65	30	90	25	210
912	30	20	0	45	95
913	695	735	835	755	3,020
914	105	175	130	105	515

915	130	65	140	85	420
916	15	80	55	40	190
917	505	485	740	435	2,165
918	15	0	10	5	30
919	40	45	105	55	245
920	720	545	525	915	2,705
921	315	325	450	290	1,380
922	325	330	405	370	1,430
923	585	430	690	405	2,110
924	45	20	45	25	135
925	645	500	630	454	2,229
926	905	995	980	944.5	3,824.50
927	250	220	165	215	850
928	450	420	660	465	1,995
930	725	740	600	520	2,585
931	80	75	65	50	270
932	280	275	360	270	1,185
933	275	260	170	150	855
934	190	195	225	240	850
935	295	160	250	220	925
936	260	145	220	235	860
937	215	170	170	170	725
939	160	220	290	165	835
940	170	240	295	210	915
941	215	115	215	165	710
943	55	70	95	35	255
944	25	30	20	15	90
945	760	650	815	735	2,960
946	105	65	85	45	300
947	30	40	30	20	120
948	25	10	20	10	65
949	160	175	175	135	645
950	415	350	420	380	1,565
951	270	270	355	300	1,195
952	170	175	140	240	725
953	570	525	545	500	2,140
954	80	65	165	75	385

955	75	60	40	50	225
956	625	530	615	530	2,300
957	160	170	175	165	670
958	230	230	170	145	775
959	435	370	350	385	1,540
960	100	155	160	105	520
961	20	15	10	15	60
TOTAL	15,045	14,100	16,110	13,928.50	59,183.50

STATE: COLORADO

ZIP CODE	QUARTER 1	QUARTER 2	QUARTER 3	QUARTER 4	TOTAL GRAMS
800	250	185	95	125	655
801	295	305	220	225	1,045
802	230	250	220	235	935
803	35	40	10	25	110
804	75	105	60	60	300
805	220	170	175	170	735
806	100	105	60	40	305
807	70	70	30	30	200
808	85	60	60	80	285
809	320	230	230	135	915
810	110	155	145	105	515
811	5	20	30	20	75
812	50	40	20	15	125
813	30	10	5	10	55
814	20	45	15	30	110
815	20	20	35	5	80
816	60	30	30	20	140
TOTAL	1,975	1,840	1,440	1,330	6,585

STATE: CONNECTICUT

ZIP CODE	QUARTER 1	QUARTER 2	QUARTER 3	QUARTER 4	TOTAL GRAMS
60	495	475	580	395	1,945
61	80	75	175	80	410
62	90	90	85	120	385

	63	145	130	105	155	535
	64	580	515	610	560	2,265
	65	50	95	65	65	275
	66	85	90	100	95	370
	67	220	195	205	165	785
	68	395	390	480	320	1,585
	69	20	30	45	60	155
TOTAL		2,160	2,085	2,450	2,015	8,710

STATE: DELAWARE

ZIP CODE		QUARTER 1	QUARTER 2	QUARTER 3	QUARTER 4	TOTAL GRAMS
	197	130	90	100	110	430
	198	170	175	190	200	735
	199	180	115	200	180	675
TOTAL		480	380	490	490	1,840

STATE: DISTRICT OF COLUMBIA

ZIP CODE		QUARTER 1	QUARTER 2	QUARTER 3	QUARTER 4	TOTAL GRAMS
	200	280	180	285	225	970
TOTAL		280	180	285	225	970

STATE: FLORIDA

ZIP CODE		QUARTER 1	QUARTER 2	QUARTER 3	QUARTER 4	TOTAL GRAMS
	320	710	575	580	595	2,460
	321	545	505	435	400	1,885
	322	855	725	810	720	3,110
	323	515	440	450	470	1,875
	324	320	285	205	180	990
	325	550	465	375	395	1,785
	326	310	330	255	250	1,145
	327	965	715	970	825	3,475
	328	635	485	620	515	2,255
	329	860	740	850	710	3,160
	330	1,650	1,175	1,090	1,050	4,965

	331	785	805	855	775	3,220
	333	850	680	710	560	2,800
	334	1,980	1,620	1,755	1,680	7,035
	335	965	690	820	830	3,305
	336	845	735	835	715	3,130
	337	950	860	1,025	955	3,790
	338	770	495	445	500	2,210
	339	1,255	645	715	785	3,400
	341	390	305	395	295	1,385
	342	785	660	725	670	2,840
	344	720	435	695	495	2,345
	346	875	945	850	875	3,545
	347	450	410	445	435	1,740
	349	600	415	535	445	1,995
TOTAL		20,135	16,140	17,445	16,125	69,845

STATE: GEORGIA

ZIP CODE		QUARTER 1	QUARTER 2	QUARTER 3	QUARTER 4	TOTAL GRAMS
	300	1,495	1,400	1,065	945	4,905
	301	785	760	575	625	2,745
	302	580	550	550	410	2,090
	303	435	410	255	255	1,355
	304	240	280	185	85	790
	305	260	330	140	160	890
	306	215	255	200	155	825
	307	180	145	130	50	505
	308	140	135	150	95	520
	309	195	210	205	115	725
	310	310	235	240	160	945
	312	130	65	60	65	320
	313	105	100	100	60	365
	314	165	145	65	85	460
	315	590	605	405	325	1,925
	316	110	100	80	70	360
	317	230	165	255	230	880
	318	0	5	0	5	10

319	85	45	65	55	250
398	35	35	50	40	160
TOTAL	6,285	5,975	4,775	3,990	21,025

STATE: GUAM

ZIP CODE	QUARTER 1	QUARTER 2	QUARTER 3	QUARTER 4	TOTAL GRAMS
969	0	10	0	0	10
TOTAL	0	10	0	0	10

STATE: HAWAII

ZIP CODE	QUARTER 1	QUARTER 2	QUARTER 3	QUARTER 4	TOTAL GRAMS
967	450	550	610	425	2,035
968	320	380	330	315	1,345
TOTAL	770	930	940	740	3,380

STATE: IDAHO

ZIP CODE	QUARTER 1	QUARTER 2	QUARTER 3	QUARTER 4	TOTAL GRAMS
832	60	40	25	30	155
833	115	140	40	25	320
834	135	130	120	105	490
835	65	90	40	55	250
836	125	125	90	65	405
837	85	85	110	130	410
838	55	35	20	25	135
TOTAL	640	645	445	435	2,165

STATE: ILLINOIS

ZIP CODE	QUARTER 1	QUARTER 2	QUARTER 3	QUARTER 4	TOTAL GRAMS
600	1,675	1,655	1,815	1,675	6,820
601	950	1,140	1,180	1,180	4,450
602	40	20	55	40	155
603	20	20	10	30	80
604	1,135	1,040	1,095	1,100	4,370

605	755	650	910	825	3,140
606	730	665	760	735	2,890
607	55	65	85	55	260
608	15	0	5	35	55
609	100	65	70	115	350
610	100	115	95	70	380
611	125	95	135	160	515
612	15	30	40	40	125
613	45	40	40	30	155
614	65	60	75	80	280
615	105	90	110	120	425
616	100	105	155	115	475
617	140	125	135	125	525
618	135	215	295	240	885
619	10	10	5	5	30
620	190	225	220	170	805
622	190	175	170	145	680
623	25	30	35	65	155
624	15	20	30	10	75
625	55	110	70	55	290
626	65	45	80	75	265
627	170	185	155	165	675
628	80	110	55	55	300
629	250	185	90	100	625
TOTAL	7,355	7,290	7,975	7,615	30,235

STATE: INDIANA

ZIP CODE	QUARTER 1	QUARTER 2	QUARTER 3	QUARTER 4	TOTAL GRAMS
460	270	195	185	160	810
461	385	315	300	285	1,285
462	440	435	395	295	1,565
463	600	595	635	580	2,410
464	100	75	95	105	375
465	120	165	155	155	595
466	15	30	30	60	135
467	65	100	75	75	315

468	55	55	100	75	285
469	120	110	80	70	380
470	80	65	55	80	280
471	180	225	225	205	835
472	95	50	80	80	305
473	90	150	85	105	430
474	90	85	115	75	365
475	160	165	205	175	705
476	95	125	85	115	420
477	310	190	230	260	990
478	50	75	50	60	235
479	105	95	135	105	440
TOTAL	3,425	3,300	3,315	3,120	13,160

STATE: IOWA

ZIP CODE	QUARTER 1	QUARTER 2	QUARTER 3	QUARTER 4	TOTAL GRAMS
500	50	50	50	60	210
501	30	25	25	5	85
502	45	35	25	20	125
503	50	70	90	100	310
504	5	15	5	0	25
505	5	5	15	15	40
506	10	20	10	15	55
507	0	10	15	5	30
508	15	30	25	20	90
510	5	0	5	0	10
511	60	80	35	60	235
512	5	0	0	0	5
513	5	0	0	5	10
514	20	5	5	0	30
515	80	65	80	80	305
520	105	80	80	130	395
522	25	10	0	10	45
523	10	10	25	15	60
524	20	15	15	20	70
525	0	5	5	5	15

	526	45	25	30	15	115
	527	40	10	10	15	75
	528	90	110	100	140	440
TOTAL		720	675	650	735	2,780

STATE: KANSAS

ZIP CODE		QUARTER 1	QUARTER 2	QUARTER 3	QUARTER 4	TOTAL GRAMS
	660	140	160	110	70	480
	661	140	80	95	95	410
	662	355	515	320	315	1,505
	664	0	0	0	5	5
	665	25	20	20	10	75
	666	50	50	95	90	285
	667	35	55	60	60	210
	668	45	30	15	10	100
	670	105	115	105	95	420
	671	25	25	15	5	70
	672	250	285	140	155	830
	673	15	20	0	0	35
	674	70	55	25	35	185
	675	10	25	10	5	50
	676	0	0	0	5	5
	677	5	5	0	0	10
	678	35	40	20	20	115
	679	0	10	0	20	30
TOTAL		1,305	1,490	1,030	995	4,820

STATE: KENTUCKY

ZIP CODE		QUARTER 1	QUARTER 2	QUARTER 3	QUARTER 4	TOTAL GRAMS
	400	135	170	85	75	465
	401	75	35	40	60	210
	402	640	535	505	410	2,090
	403	100	105	60	55	320
	404	40	30	25	30	125
	405	85	20	35	40	180

	406	10	0	10	0	20
	407	65	60	55	65	245
	409	35	50	40	30	155
	410	325	325	255	225	1,130
	411	20	15	0	5	40
	412	10	5	0	5	20
	415	10	5	0	0	15
	417	5	0	0	0	5
	420	130	100	135	125	490
	421	105	120	120	70	415
	422	55	90	70	85	300
	423	40	50	60	40	190
	424	25	65	85	15	190
	425	10	35	25	30	100
	426	30	15	30	30	105
	427	110	165	104	70	449
TOTAL		2,060	1,995	1,739	1,465	7,259

STATE: LOUISIANA

ZIP CODE		QUARTER 1	QUARTER 2	QUARTER 3	QUARTER 4	TOTAL GRAMS
	700	745	845	780	610	2,980
	701	270	185	255	180	890
	703	95	70	75	70	310
	704	590	540	620	435	2,185
	705	410	360	420	430	1,620
	706	330	270	310	335	1,245
	707	150	185	210	145	690
	708	140	160	180	160	640
	710	30	60	95	15	200
	711	65	55	165	105	390
	712	205	175	195	110	685
	713	245	310	340	265	1,160
	714	95	105	140	90	430
TOTAL		3,370	3,320	3,785	2,950	13,425

STATE: MAINE

ZIP CODE	QUARTER 1	QUARTER 2	QUARTER 3	QUARTER 4	TOTAL GRAMS
39	5	0	0	0	5
40	175	150	200	210	735
41	65	75	60	80	280
42	35	15	20	10	80
43	25	20	25	15	85
44	60	15	5	35	115
45	10	20	10	0	40
46	10	0	0	5	15
47	30	15	10	25	80
49	65	65	75	50	255
TOTAL	480	375	405	430	1,690

STATE: MARYLAND

ZIP CODE	QUARTER 1	QUARTER 2	QUARTER 3	QUARTER 4	TOTAL GRAMS
206	340	275	320	300	1,235
207	280	265	220	175	940
208	260	220	350	245	1,075
209	165	45	95	105	410
210	325	375	340	265	1,305
211	110	115	85	155	465
212	420	315	370	285	1,390
214	80	110	100	70	360
215	90	90	60	80	320
216	90	50	75	100	315
217	315	345	400	275	1,335
218	95	35	65	30	225
219	40	60	40	25	165
TOTAL	2,610	2,300	2,520	2,110	9,540

STATE: MASSACHUSETTS

ZIP CODE	QUARTER 1	QUARTER 2	QUARTER 3	QUARTER 4	TOTAL GRAMS
10	240	195	255	145	835
11	35	15	30	20	100

12	10	30	45	15	100
13	10	0	35	15	60
14	180	140	225	75	620
15	200	155	180	155	690
16	190	185	295	120	790
17	190	185	215	225	815
18	250	260	255	255	1,020
19	225	275	275	300	1,075
20	135	190	155	165	645
21	365	355	465	340	1,525
22	10	5	10	5	30
23	130	145	200	180	655
24	155	165	195	225	740
25	35	25	10	25	95
26	85	65	70	65	285
27	180	165	270	125	740
TOTAL	2,625	2,555	3,185	2,455	10,820

STATE: MICHIGAN

ZIP CODE	QUARTER 1	QUARTER 2	QUARTER 3	QUARTER 4	TOTAL GRAMS
480	1,590	1,685	1,615	1,400	6,290
481	1,270	1,475	1,470	1,490	5,705
482	225	325	350	285	1,185
483	980	1,020	1,080	920	4,000
484	300	435	200	210	1,145
485	45	60	135	85	325
486	130	145	150	145	570
487	100	90	70	70	330
488	205	400	385	405	1,395
489	165	140	125	130	560
490	260	310	385	300	1,255
491	15	15	10	15	55
492	220	180	205	210	815
493	55	50	35	70	210
494	275	385	400	455	1,515
495	130	205	220	215	770

496	30	60	50	60	200
497	45	65	145	25	280
498	95	65	50	35	245
499	5	15	10	30	60
TOTAL	6,140	7,125	7,090	6,555	26,910

STATE: MINNESOTA

ZIP CODE	QUARTER 1	QUARTER 2	QUARTER 3	QUARTER 4	TOTAL GRAMS
550	170	130	165	125	590
551	260	280	280	235	1,055
553	310	265	280	265	1,120
554	195	220	210	225	850
556	0	0	5	0	5
557	95	100	85	100	380
558	25	30	25	50	130
559	0	5	0	5	10
560	70	70	40	45	225
561	15	15	15	25	70
562	60	20	70	55	205
563	85	55	45	55	240
564	50	80	60	15	205
565	30	25	25	35	115
566	25	20	35	25	105
567	0	5	5	0	10
TOTAL	1,390	1,320	1,345	1,260	5,315

STATE: MISSISSIPPI

ZIP CODE	QUARTER 1	QUARTER 2	QUARTER 3	QUARTER 4	TOTAL GRAMS
386	155	130	135	65	485
387	10	5	5	15	35
388	125	95	60	75	355
389	50	30	55	40	175
390	55	80	35	10	180
391	130	25	90	50	295
392	45	45	35	45	170

	393	35	25	15	40	115
	394	215	195	250	105	765
	395	275	320	310	200	1,105
	396	65	50	45	30	190
	397	40	50	45	15	150
TOTAL		1,200	1,050	1,080	690	4,020

STATE: MISSOURI

ZIP CODE		QUARTER 1	QUARTER 2	QUARTER 3	QUARTER 4	TOTAL GRAMS
	630	660	590	725	570	2,545
	631	1,480	1,850	755	570	4,655
	633	625	520	615	400	2,160
	634	5	0	0	5	10
	635	20	10	15	15	60
	636	60	20	45	35	160
	637	75	65	75	75	290
	638	40	65	65	40	210
	639	85	95	65	80	325
	640	240	245	315	320	1,120
	641	385	465	460	580	1,890
	644	10	15	15	5	45
	645	10	30	60	45	145
	647	5	5	15	10	35
	648	65	100	95	75	335
	650	70	45	65	35	215
	651	55	55	75	60	245
	652	10	15	5	10	40
	653	0	0	15	10	25
	654	10	5	0	5	20
	655	0	15	5	10	30
	656	55	50	75	35	215
	657	70	115	155	110	450
	658	145	140	195	115	595
TOTAL		4,180	4,515	3,910	3,215	15,820

STATE: MONTANA

ZIP CODE	QUARTER 1	QUARTER 2	QUARTER 3	QUARTER 4	TOTAL GRAMS
590	25	25	20	10	80
591	50	75	75	60	260
592	5	10	0	10	25
593	40	45	30	5	120
594	30	30	95	50	205
595	5	10	0	0	15
596	15	40	25	20	100
597	105	100	95	60	360
598	115	125	185	140	565
599	100	80	65	20	265
TOTAL	490	540	590	375	1,995

STATE: NEBRASKA

ZIP CODE	QUARTER 1	QUARTER 2	QUARTER 3	QUARTER 4	TOTAL GRAMS
680	100	75	75	110	360
681	305	285	340	310	1,240
684	15	15	25	20	75
685	120	160	155	140	575
686	20	40	40	20	120
687	25	20	50	45	140
688	35	25	20	20	100
689	10	5	15	10	40
691	10	0	5	15	30
692	0	0	15	0	15
693	75	80	60	60	275
TOTAL	715	705	800	750	2,970

STATE: NEVADA

ZIP CODE	QUARTER 1	QUARTER 2	QUARTER 3	QUARTER 4	TOTAL GRAMS
890	1,410	1,490	1,175	1,415	5,490
891	2,260	2,115	1,795	1,925	8,095
893	15	70	35	35	155

	894	475	405	375	315	1,570
	895	535	470	510	315	1,830
	897	85	90	110	75	360
	898	240	195	195	145	775
TOTAL		5,020	4,835	4,195	4,225	18,275
STATE:	NEW HAMPSHIRE					
ZIP CODE	QUARTER 1	QUARTER 2	QUARTER 3		QUARTER 4	TOTAL GRAMS
	30	120	180	175	210	685
	31	145	150	110	125	530
	32	40	30	25	30	125
	33	0	5	5	5	15
	34	10	5	20	10	45
	36	10	10	10	10	40
	37	0	0	5	0	5
	38	160	130	125	135	550
TOTAL		485	510	475	525	1,995
STATE:	NEW JERSEY					
ZIP CODE	QUARTER 1	QUARTER 2	QUARTER 3		QUARTER 4	TOTAL GRAMS
	70	640	690	855	730	2,915
	71	30	60	35	35	160
	72	105	30	60	45	240
	73	65	50	55	35	205
	74	645	590	745	560	2,540
	75	40	35	40	50	165
	76	325	260	345	365	1,295
	77	515	385	535	410	1,845
	78	215	205	230	185	835
	79	215	105	120	160	600
	80	645	650	620	620	2,535
	81	45	40	10	35	130
	82	140	180	320	170	810
	83	130	100	110	90	430
	84	10	10	10	5	35

85	70	45	75	40	230
86	60	50	55	70	235
87	210	300	385	215	1,110
88	425	400	435	400	1,660
89	50	20	25	40	135
TOTAL	4,580	4,205	5,065	4,260	18,110

STATE: NEW MEXICO

ZIP CODE	QUARTER 1	QUARTER 2	QUARTER 3	QUARTER 4	TOTAL GRAMS
870	100	105	80	90	375
871	320	225	325	265	1,135
873	0	0	0	5	5
874	35	0	10	0	45
875	90	115	115	95	415
877	15	5	20	25	65
879	5	5	15	35	60
880	130	185	130	130	575
881	10	10	15	0	35
882	95	145	90	95	425
883	35	40	55	45	175
884	5	0	5	5	15
TOTAL	840	835	860	790	3,325

STATE: NEW YORK

ZIP CODE	QUARTER 1	QUARTER 2	QUARTER 3	QUARTER 4	TOTAL GRAMS
100	450	300	385	315	1,450
101	60	45	30	20	155
103	200	130	185	130	645
104	210	180	180	205	775
105	210	190	130	175	705
106	40	50	30	65	185
107	110	110	60	40	320
108	75	60	50	20	205
109	315	175	255	265	1,010
110	140	90	70	55	355

111	35	60	40	0	135
112	365	450	360	340	1,515
113	255	295	170	180	900
114	110	100	155	105	470
115	300	355	315	285	1,255
116	25	25	30	10	90
117	615	635	545	430	2,225
118	35	50	60	40	185
119	70	60	65	125	320
120	35	80	100	40	255
121	25	10	40	30	105
122	20	15	15	60	110
123	115	95	130	95	435
124	85	75	85	80	325
125	65	50	75	125	315
126	40	35	65	30	170
127	15	0	35	30	80
128	85	65	40	65	255
130	5	5	5	15	30
131	35	35	30	30	130
132	5	0	5	0	10
133	45	35	25	40	145
134	15	5	15	0	35
135	0	5	0	0	5
136	0	10	20	15	45
137	35	35	20	30	120
138	70	25	60	70	225
139	45	35	20	30	130
140	5	15	15	80	115
141	30	30	45	30	135
142	120	90	120	130	460
143	0	10	5	10	25
144	75	85	70	30	260
145	10	60	105	50	225
146	80	85	80	70	315
147	20	25	50	50	145
148	10	10	10	15	45

	149	10	5	10	10	35
TOTAL		4,720	4,390	4,410	4,065	17,585

STATE: NORTH CAROLINA

ZIP CODE		QUARTER 1	QUARTER 2	QUARTER 3	QUARTER 4	TOTAL GRAMS
	270	75	110	90	85	360
	271	90	100	115	105	410
	272	300	220	325	225	1,070
	273	90	90	110	120	410
	274	45	20	45	95	205
	275	305	315	450	325	1,395
	276	185	205	200	205	795
	277	130	80	60	40	310
	278	185	165	200	130	680
	279	90	75	130	90	385
	280	240	340	285	195	1,060
	281	310	190	325	210	1,035
	282	265	285	260	265	1,075
	283	405	355	310	340	1,410
	284	320	330	350	305	1,305
	285	230	240	240	245	955
	286	245	250	205	310	1,010
	287	200	205	240	160	805
	288	80	100	105	105	390
	289	85	40	40	35	200
TOTAL		3,875	3,715	4,085	3,590	15,265

STATE: NORTH DAKOTA

ZIP CODE		QUARTER 1	QUARTER 2	QUARTER 3	QUARTER 4	TOTAL GRAMS
	580	0	5	0	0	5
	581	20	0	5	5	30
	582	25	25	5	15	70
	584	0	5	0	0	5
	585	35	50	55	40	180
	586	15	10	10	5	40

	587	0	10	5	10	25
	588	75	65	45	25	210
TOTAL		170	170	125	100	565

STATE: OHIO

ZIP CODE		QUARTER 1	QUARTER 2	QUARTER 3	QUARTER 4	TOTAL GRAMS
	430	375	330	220	200	1,125
	431	295	260	175	150	880
	432	1,285	1,455	285	270	3,295
	433	30	35	25	5	95
	434	75	65	50	55	245
	435	195	210	45	30	480
	436	270	255	140	170	835
	437	30	20	10	5	65
	438	15	0	5	0	20
	439	25	20	45	30	120
	440	245	275	320	200	1,040
	441	460	400	410	235	1,505
	442	175	110	180	120	585
	443	65	70	45	45	225
	444	40	55	40	30	165
	445	25	35	85	35	180
	446	120	190	145	110	565
	447	145	5	85	95	330
	448	50	15	15	30	110
	449	40	35	55	20	150
	450	275	270	265	175	985
	451	140	75	80	60	355
	452	545	510	330	295	1,680
	453	185	155	100	95	535
	454	90	145	85	90	410
	455	40	45	35	70	190
	456	125	110	30	50	315
	457	45	40	60	40	185
	458	15	30	60	20	125
TOTAL		5,420	5,220	3,425	2,730	16,795

DATE RANGE: 01/01/2017 TO 12/31/2017 | ARCOS 3 - REPORT 1 | Run Date: 07/03/2018

RETAIL DRUG DISTRIBUTION BY ZIP CODE WITHIN STATE BY GRAMS WT

STATE: OKLAHOMA

ZIP CODE	QUARTER 1	QUARTER 2	QUARTER 3	QUARTER 4	TOTAL GRAMS
730	520	530	555	550	2,155
731	670	610	760	615	2,655
734	80	70	105	65	320
735	80	90	135	145	450
736	25	10	5	15	55
737	35	40	20	55	150
738	20	60	35	25	140
740	560	590	575	610	2,335
741	215	320	280	315	1,130
743	75	20	25	25	145
744	185	135	265	250	835
745	50	40	45	40	175
746	30	30	45	30	135
747	50	50	35	30	165
748	50	85	155	65	355
749	20	20	15	0	55
TOTAL	2,665	2,700	3,055	2,835	11,255

STATE: OREGON

ZIP CODE	QUARTER 1	QUARTER 2	QUARTER 3	QUARTER 4	TOTAL GRAMS
970	240	235	100	100	675
971	115	105	55	30	305
972	180	230	175	130	715
973	255	210	190	220	875
974	240	190	210	135	775
975	100	95	45	55	295
977	65	40	25	40	170
978	10	10	10	5	35
979	0	0	20	0	20
TOTAL	1,205	1,115	830	715	3,865

DATE RANGE: 01/01/2017 TO 12/31/2017 | ARCOS 3 - REPORT 1 | Run Date: 07/03/2018

RETAIL DRUG DISTRIBUTION BY ZIP CODE WITHIN STATE BY GRAMS WT

STATE: PENNSYLVANIA

ZIP CODE	QUARTER 1	QUARTER 2	QUARTER 3	QUARTER 4	TOTAL GRAMS
150	245	180	200	160	785
151	105	95	150	30	380
152	245	165	185	100	695
153	60	35	55	35	185
154	85	75	45	25	230
155	35	15	25	15	90
156	75	105	110	20	310
157	75	60	55	30	220
158	15	20	10	0	45
159	95	100	60	65	320
160	50	70	25	35	180
161	60	35	15	20	130
162	30	40	30	20	120
163	15	15	20	15	65
164	25	20	5	5	55
165	25	45	40	25	135
166	35	25	35	5	100
167	15	10	15	10	50
168	15	15	10	10	50
169	5	5	5	5	20
170	265	360	310	285	1,220
171	55	50	50	60	215
172	75	100	60	55	290
173	130	105	90	130	455
174	75	95	130	145	445
175	180	155	180	125	640
176	95	80	95	65	335
177	115	105	90	55	365
178	50	75	90	60	275
179	20	60	25	35	140
180	110	140	140	145	535
181	105	85	165	90	445
182	25	35	30	40	130
183	80	70	145	35	330
184	55	50	45	15	165

	185	155	115	160	140	570
	186	90	100	65	80	335
	187	575	780	610	430	2,395
	188	0	0	0	5	5
	189	195	145	190	170	700
	190	715	715	580	540	2,550
	191	695	590	630	550	2,465
	193	115	110	145	155	525
	194	270	360	270	330	1,230
	195	55	70	50	50	225
	196	130	165	120	110	525
TOTAL		5,740	5,845	5,560	4,530	21,675

STATE: PUERTO RICO

ZIP CODE		QUARTER 1	QUARTER 2	QUARTER 3	QUARTER 4	TOTAL GRAMS
	6	60	60	60	45	225
	7	15	10	30	25	80
	9	60	80	75	85	300
TOTAL		135	150	165	155	605

STATE: RHODE ISLAND

ZIP CODE		QUARTER 1	QUARTER 2	QUARTER 3	QUARTER 4	TOTAL GRAMS
	28	345	345	400	335	1,425
	29	290	255	290	260	1,095
TOTAL		635	600	690	595	2,520

STATE: SOUTH CAROLINA

ZIP CODE		QUARTER 1	QUARTER 2	QUARTER 3	QUARTER 4	TOTAL GRAMS
	290	200	200	115	130	645
	291	130	75	95	110	410
	292	105	140	165	85	495
	293	240	140	160	135	675
	294	545	500	660	515	2,220
	295	470	390	410	500	1,770

	296	640	565	570	515	2,290
	297	110	70	55	70	305
	298	50	45	60	35	190
	299	130	80	85	100	395
TOTAL		2,620	2,205	2,375	2,195	9,395

STATE: SOUTH DAKOTA

ZIP CODE		QUARTER 1	QUARTER 2	QUARTER 3	QUARTER 4	TOTAL GRAMS
	570	35	15	5	10	65
	571	135	110	85	70	400
	572	5	0	0	15	20
	573	25	15	25	25	90
	575	30	25	25	25	105
	577	40	55	85	45	225
TOTAL		270	220	225	190	905

STATE: TENNESSEE

ZIP CODE		QUARTER 1	QUARTER 2	QUARTER 3	QUARTER 4	TOTAL GRAMS
	370	385	325	285	265	1,260
	371	260	225	315	195	995
	372	235	245	200	150	830
	373	195	280	305	285	1,065
	374	120	185	130	115	550
	376	275	345	290	280	1,190
	377	130	165	170	205	670
	378	285	275	250	240	1,050
	379	265	215	220	305	1,005
	380	110	145	180	110	545
	381	175	150	185	180	690
	382	35	20	35	30	120
	383	225	165	165	120	675
	384	50	30	50	30	160
	385	115	145	100	140	500
TOTAL		2,860	2,915	2,880	2,650	11,305

STATE: TEXAS

ZIP CODE	QUARTER 1	QUARTER 2	QUARTER 3	QUARTER 4	TOTAL GRAMS
750	1,315	1,225	1,105	1,165	4,810
751	415	430	430	330	1,605
752	400	510	470	395	1,775
753	0	0	5	0	5
754	165	165	215	180	725
755	30	10	10	10	60
756	110	135	105	105	455
757	115	135	205	135	590
758	65	55	35	25	180
759	265	135	120	190	710
760	755	790	800	775	3,120
761	500	525	570	445	2,040
762	425	450	390	230	1,495
763	75	65	65	60	265
764	0	10	5	5	20
765	70	70	70	30	240
766	5	10	0	20	35
767	25	35	45	30	135
768	10	15	20	25	70
769	10	10	25	20	65
770	800	940	1,134.90	860	3,734.90
773	1,060	960	845	680	3,545
774	600	620	610	575	2,405
775	915	850	910	745	3,420
776	230	200	315	225	970
777	85	105	135	140	465
778	25	55	45	35	160
779	25	15	40	20	100
780	350	410	455	255	1,470
781	260	215	340	205	1,020
782	750	900	1,000	645	3,295
783	120	210	175	115	620
784	560	455	535	450	2,000
785	280	350	335	240	1,205

786	510	555	465	400	1,930
787	480	385	570	385	1,820
788	10	25	15	0	50
789	5	0	10	0	15
790	15	10	20	35	80
791	130	120	80	70	400
792	0	0	5	5	10
793	10	20	10	5	45
794	150	75	150	160	535
795	0	0	0	5	5
796	15	20	40	15	90
797	180	180	170	155	685
798	0	0	0	5	5
799	235	185	195	210	825
TOTAL	12,555	12,640	13,294.90	10,815	49,304.90

STATE: UTAH

ZIP CODE	QUARTER 1	QUARTER 2	QUARTER 3	QUARTER 4	TOTAL GRAMS
840	745	915	575	635	2,870
841	160	175	140	160	635
843	70	50	15	5	140
844	155	150	65	65	435
845	15	10	5	10	40
846	120	100	95	115	430
847	365	310	230	230	1,135
TOTAL	1,630	1,710	1,125	1,220	5,685

STATE: VERMONT

ZIP CODE	QUARTER 1	QUARTER 2	QUARTER 3	QUARTER 4	TOTAL GRAMS
51	5	0	0	0	5
52	10	0	5	0	15
53	5	0	0	0	5
54	15	0	0	5	20
56	5	0	10	0	15
57	55	60	70	35	220

58	5	0	0	0	5
TOTAL	100	60	85	40	285

STATE: VIRGIN ISLANDS

ZIP CODE	QUARTER 1	QUARTER 2	QUARTER 3	QUARTER 4	TOTAL GRAMS
8	20	45	25	15	105
TOTAL	20	45	25	15	105

STATE: VIRGINIA

ZIP CODE	QUARTER 1	QUARTER 2	QUARTER 3	QUARTER 4	TOTAL GRAMS
201	495	490	365	415	1,765
220	85	145	100	95	425
221	70	90	80	50	290
222	85	75	90	55	305
223	80	95	60	95	330
224	90	105	115	145	455
225	35	40	60	45	180
226	40	20	25	20	105
227	25	20	35	15	95
228	35	20	10	25	90
229	50	85	85	100	320
230	150	130	150	200	630
231	255	340	200	255	1,050
232	295	220	175	235	925
233	90	150	65	75	380
234	330	390	245	255	1,220
235	170	185	105	145	605
236	135	170	205	195	705
237	10	15	60	0	85
238	185	135	130	140	590
239	25	35	20	15	95
240	220	200	145	130	695
241	105	85	70	80	340
242	80	125	100	95	400
243	110	120	60	50	340

	244	135	150	65	20	370
	245	170	170	220	105	665
	246	115	160	105	65	445
TOTAL		3,670	3,965	3,145	3,120	13,900

STATE: WASHINGTON

ZIP CODE		QUARTER 1	QUARTER 2	QUARTER 3	QUARTER 4	TOTAL GRAMS
	980	455	460	405	260	1,580
	981	350	330	215	150	1,045
	982	350	315	330	185	1,180
	983	320	370	220	185	1,095
	984	280	300	225	240	1,045
	985	135	90	60	115	400
	986	410	320	205	255	1,190
	988	5	15	25	10	55
	989	90	95	110	60	355
	990	10	25	20	25	80
	991	25	40	30	15	110
	992	95	155	125	65	440
	993	145	160	140	130	575
	994	40	25	20	5	90
TOTAL		2,710	2,700	2,130	1,700	9,240

STATE: WEST VIRGINIA

ZIP CODE		QUARTER 1	QUARTER 2	QUARTER 3	QUARTER 4	TOTAL GRAMS
	247	5	10	10	5	30
	248	5	0	5	0	10
	249	25	20	25	0	70
	250	35	20	10	15	80
	251	25	5	20	25	75
	252	0	0	0	5	5
	253	55	55	55	40	205
	254	90	75	15	65	245
	255	70	30	55	55	210
	257	35	40	30	10	115

	258	45	35	60	25	165
	259	5	0	0	0	5
	260	90	85	30	40	245
	261	5	0	10	0	15
	262	20	20	20	20	80
	263	60	40	75	35	210
	265	45	65	50	55	215
	266	20	15	10	5	50
	267	30	35	20	5	90
TOTAL		665	550	500	405	2,120

STATE: WISCONSIN

ZIP CODE		QUARTER 1	QUARTER 2	QUARTER 3	QUARTER 4	TOTAL GRAMS
	530	210	205	265	245	925
	531	405	455	435	360	1,655
	532	280	210	250	285	1,025
	534	55	20	45	10	130
	535	205	150	265	200	820
	537	25	25	20	25	95
	538	5	0	0	0	5
	539	20	5	30	25	80
	540	25	35	55	35	150
	541	25	15	15	40	95
	542	10	10	30	0	50
	543	20	35	30	20	105
	544	55	20	30	25	130
	545	40	20	25	10	95
	546	30	20	20	15	85
	547	45	45	50	70	210
	548	50	70	25	40	185
	549	115	110	160	135	520
TOTAL		1,620	1,450	1,750	1,540	6,360

STATE: WYOMING

ZIP CODE		QUARTER 1	QUARTER 2	QUARTER 3	QUARTER 4	TOTAL GRAMS

	820	15	25	25	15	80
	822	0	0	0	10	10
	823	5	0	0	0	5
	824	45	55	95	70	265
	825	25	35	20	20	100
	826	65	50	30	30	175
	827	0	5	0	5	10
	828	180	150	170	180	680
	829	95	75	5	20	195
	830	0	0	5	0	5
TOTAL		430	395	350	350	1,525

DRUG CODE: 2125 DRUG NAME: AMOBARBITAL (SCHEDULE 2)

STATE: ARIZONA

ZIP CODE		QUARTER 1	QUARTER 2	QUARTER 3	QUARTER 4	TOTAL GRAMS
	850	11.85	11.39	12.76	8.2	44.2
	857	0.91	0	0	0	0.91
TOTAL		12.76	11.39	12.76	8.2	45.11

STATE: ARKANSAS

ZIP CODE		QUARTER 1	QUARTER 2	QUARTER 3	QUARTER 4	TOTAL GRAMS
	722	0	0.46	0.46	0	0.92
TOTAL		0	0.46	0.46	0	0.92

STATE: CALIFORNIA

ZIP CODE		QUARTER 1	QUARTER 2	QUARTER 3	QUARTER 4	TOTAL GRAMS
	900	13.67	13.67	9.57	7.75	44.66
	910	0.46	0	0	0.46	0.92
	911	0	0	0.91	0	0.91
	915	1.82	0	0.91	2.28	5.01
	920	1.82	1.82	2.73	0.91	7.28
	921	0	2.73	1.82	0	4.55
	923	1.82	0.91	0.46	0.91	4.1
	926	0	0	0.46	0.91	1.37

	928	3.19	1.82	2.73	0	7.74
	941	0	0	4.56	7.29	11.85
	958	4.56	0	5.92	1.82	12.3
TOTAL		27.34	20.95	30.07	22.33	100.69

STATE: COLORADO

ZIP CODE		QUARTER 1	QUARTER 2	QUARTER 3	QUARTER 4	TOTAL GRAMS
	800	15.04	7.75	10.48	10.03	43.3
	801	6.84	4.56	0	0	11.4
	802	5.92	3.65	4.1	0	13.67
TOTAL		27.8	15.96	14.58	10.03	68.37

STATE: CONNECTICUT

ZIP CODE		QUARTER 1	QUARTER 2	QUARTER 3	QUARTER 4	TOTAL GRAMS
	61	0	0.46	0	1.37	1.83
	65	0	1.82	0	0	1.82
TOTAL		0	2.28	0	1.37	3.65

STATE: DISTRICT OF COLUMBIA

ZIP CODE		QUARTER 1	QUARTER 2	QUARTER 3	QUARTER 4	TOTAL GRAMS
	200	0.46	0	1.82	4.56	6.84
TOTAL		0.46	0	1.82	4.56	6.84

STATE: FLORIDA

ZIP CODE		QUARTER 1	QUARTER 2	QUARTER 3	QUARTER 4	TOTAL GRAMS
	322	0	0.91	5.92	0.46	7.29
	325	0.91	0	0	0	0.91
	328	3.19	1.37	1.37	5.47	11.4
	330	0	0	1.37	2.28	3.65
TOTAL		4.1	2.28	8.66	8.21	23.25

STATE: GEORGIA

ZIP CODE	QUARTER 1	QUARTER 2	QUARTER 3	QUARTER 4	TOTAL GRAMS
301	0	0	0.46	0	0.46
303	1.82	0.91	7.75	0	10.48
309	0	0	2.28	13.22	15.5
TOTAL	1.82	0.91	10.49	13.22	26.44

STATE: ILLINOIS

ZIP CODE	QUARTER 1	QUARTER 2	QUARTER 3	QUARTER 4	TOTAL GRAMS
601	0.46	0.91	0.46	1.37	3.2
602	0.91	0	0.46	0	1.37
604	2.73	0	0	0	2.73
606	0	0	3.19	1.82	5.01
617	0	0	0.46	0	0.46
618	0.46	0	0	0	0.46
627	0	0	2.28	0.46	2.74
TOTAL	4.56	0.91	6.85	3.65	15.97

STATE: INDIANA

ZIP CODE	QUARTER 1	QUARTER 2	QUARTER 3	QUARTER 4	TOTAL GRAMS
462	0	0	2.28	1.82	4.1
TOTAL	0	0	2.28	1.82	4.1

STATE: IOWA

ZIP CODE	QUARTER 1	QUARTER 2	QUARTER 3	QUARTER 4	TOTAL GRAMS
522	0	0	0.46	0	0.46
TOTAL	0	0	0.46	0	0.46

STATE: KANSAS

ZIP CODE	QUARTER 1	QUARTER 2	QUARTER 3	QUARTER 4	TOTAL GRAMS
661	1.82	5.01	0	5.47	12.3
672	0	0	0.46	0.91	1.37
TOTAL	1.82	5.01	0.46	6.38	13.67

STATE: KENTUCKY

ZIP CODE	QUARTER 1	QUARTER 2	QUARTER 3	QUARTER 4	TOTAL GRAMS
402	4.56	2.73	2.73	5.47	15.49
405	0	0.91	0.91	1.82	3.64
TOTAL	4.56	3.64	3.64	7.29	19.13

STATE: MAINE

ZIP CODE	QUARTER 1	QUARTER 2	QUARTER 3	QUARTER 4	TOTAL GRAMS
41	0	0	0.46	2.28	2.74
TOTAL	0	0	0.46	2.28	2.74

STATE: MARYLAND

ZIP CODE	QUARTER 1	QUARTER 2	QUARTER 3	QUARTER 4	TOTAL GRAMS
208	0.91	1.82	3.19	0	5.92
209	0	0	0	1.82	1.82
212	6.38	1.37	3.19	8.66	19.6
TOTAL	7.29	3.19	6.38	10.48	27.34

STATE: MASSACHUSETTS

ZIP CODE	QUARTER 1	QUARTER 2	QUARTER 3	QUARTER 4	TOTAL GRAMS
16	0	0	0	1.82	1.82
21	7.29	1.82	0.46	2.73	12.3
22	2.28	0	0.46	3.19	5.93
TOTAL	9.57	1.82	0.92	7.74	20.05

STATE: MICHIGAN

ZIP CODE	QUARTER 1	QUARTER 2	QUARTER 3	QUARTER 4	TOTAL GRAMS
480	0	0	0.91	0	0.91
481	0	0	0.46	0	0.46
482	1.37	0.91	3.19	0.46	5.93
483	0	0	0	1.37	1.37

	489	0	0.91	0	0	0.91
	490	1.82	0	0	0	1.82
	495	2.73	2.28	1.37	0.91	7.29
TOTAL		5.92	4.1	5.93	2.74	18.69

STATE:	MINNESOTA					
ZIP CODE		QUARTER 1	QUARTER 2	QUARTER 3	QUARTER 4	TOTAL GRAMS
	551	2.28	0	0.91	1.37	4.56
	554	2.28	2.73	5.92	3.65	14.58
	559	0.46	0.46	0.46	2.73	4.11
TOTAL		5.02	3.19	7.29	7.75	23.25

STATE:	MISSISSIPPI					
ZIP CODE		QUARTER 1	QUARTER 2	QUARTER 3	QUARTER 4	TOTAL GRAMS
	392	1.82	0	1.37	1.37	4.56
TOTAL		1.82	0	1.37	1.37	4.56

STATE:	MISSOURI					
ZIP CODE		QUARTER 1	QUARTER 2	QUARTER 3	QUARTER 4	TOTAL GRAMS
	631	0	0	2.73	0	2.73
	641	0.46	0.91	0	0.91	2.28
	652	0	0	0	1.82	1.82
TOTAL		0.46	0.91	2.73	2.73	6.83

STATE:	NEBRASKA					
ZIP CODE		QUARTER 1	QUARTER 2	QUARTER 3	QUARTER 4	TOTAL GRAMS
	681	2.73	0	0	0.91	3.64
TOTAL		2.73	0	0	0.91	3.64

STATE:	NEW HAMPSHIRE					
ZIP CODE		QUARTER 1	QUARTER 2	QUARTER 3	QUARTER 4	TOTAL GRAMS
	37	4.1	1.82	2.73	3.65	12.3

TOTAL	4.1	1.82	2.73	3.65	12.3

STATE: NEW JERSEY

ZIP CODE	QUARTER 1	QUARTER 2	QUARTER 3	QUARTER 4	TOTAL GRAMS
70	0	0.91	0	0	0.91
71	0	0	3.65	6.38	10.03
74	0	0	0.91	0	0.91
76	1.37	0	0	7.29	8.66
77	0	0	0	0.46	0.46
79	1.82	2.28	2.28	0.46	6.84
80	0	0	0.46	0	0.46
81	0.46	0	0	1.82	2.28
TOTAL	3.65	3.19	7.3	16.41	30.55

STATE: NEW YORK

ZIP CODE	QUARTER 1	QUARTER 2	QUARTER 3	QUARTER 4	TOTAL GRAMS
100	27.34	12.3	9.57	15.49	64.7
103	0	0	0.91	0	0.91
104	3.65	0	0	1.37	5.02
105	0	0	0	2.28	2.28
110	11.85	0.91	2.73	7.29	22.78
112	3.65	1.82	0.46	2.73	8.66
113	0	0	0	1.37	1.37
117	3.65	1.37	0	5.01	10.03
122	0	0.46	1.82	4.56	6.84
126	0	0	0.46	1.82	2.28
142	4.56	2.28	9.11	7.29	23.24
146	0	2.73	1.82	0	4.55
TOTAL	54.7	21.87	26.88	49.21	152.66

STATE: NORTH CAROLINA

ZIP CODE	QUARTER 1	QUARTER 2	QUARTER 3	QUARTER 4	TOTAL GRAMS
271	1.37	2.73	2.28	1.82	8.2
275	3.65	1.37	2.28	0.91	8.21

	277	4.1	0	0.91	0	5.01
	280	3.65	0.91	0	4.1	8.66
	282	3.65	0	0	4.56	8.21
TOTAL		16.42	5.01	5.47	11.39	38.29

STATE:	OHIO				
ZIP CODE	QUARTER 1	QUARTER 2	QUARTER 3	QUARTER 4	TOTAL GRAMS
432	0	0.46	0.46	0.46	1.38
443	0	0	1.82	0	1.82
452	2.28	1.37	3.19	4.1	10.94
454	0.91	1.82	0	0.91	3.64
TOTAL	3.19	3.65	5.47	5.47	17.78

STATE:	OREGON				
ZIP CODE	QUARTER 1	QUARTER 2	QUARTER 3	QUARTER 4	TOTAL GRAMS
972	1.37	1.37	1.37	2.73	6.84
TOTAL	1.37	1.37	1.37	2.73	6.84

STATE:	PENNSYLVANIA				
ZIP CODE	QUARTER 1	QUARTER 2	QUARTER 3	QUARTER 4	TOTAL GRAMS
152	0	0	0	4.56	4.56
170	0.46	0	0	0	0.46
174	0.46	0.91	0.91	0	2.28
178	5.47	0	0	0	5.47
180	0	0	0	0.46	0.46
187	0	0	0.91	0	0.91
191	7.29	1.82	2.28	3.65	15.04
TOTAL	13.68	2.73	4.1	8.67	29.18

STATE:	RHODE ISLAND				
ZIP CODE	QUARTER 1	QUARTER 2	QUARTER 3	QUARTER 4	TOTAL GRAMS
29	3.65	0	0	0	3.65

TOTAL		3.65	0	0	0	3.65

STATE:	SOUTH CAROLINA					
ZIP CODE		QUARTER 1	QUARTER 2	QUARTER 3	QUARTER 4	TOTAL GRAMS
	292	0	0	2.73	0	2.73
	294	1.37	4.1	0.91	4.1	10.48
	296	0	0	1.82	0	1.82
TOTAL		1.37	4.1	5.46	4.1	15.03

STATE:	TENNESSEE					
ZIP CODE		QUARTER 1	QUARTER 2	QUARTER 3	QUARTER 4	TOTAL GRAMS
	372	4.1	3.19	4.56	1.37	13.22
	378	0	0.46	0	0	0.46
	381	0	1.37	0.46	0.46	2.29
TOTAL		4.1	5.02	5.02	1.83	15.97

STATE:	TEXAS					
ZIP CODE		QUARTER 1	QUARTER 2	QUARTER 3	QUARTER 4	TOTAL GRAMS
	750	0	0	0	0.46	0.46
	752	4.56	0	0	0	4.56
	753	0.46	0	0	0.46	0.92
	761	0	0	0	0.46	0.46
	765	0	0.46	4.1	1.82	6.38
	770	8.66	4.56	6.38	4.1	23.7
	782	4.56	4.56	8.66	4.56	22.34
	787	7.29	4.1	8.2	3.65	23.24
	794	0	0	0.46	0.91	1.37
TOTAL		25.53	13.68	27.8	16.42	83.43

STATE:	UTAH					
ZIP CODE		QUARTER 1	QUARTER 2	QUARTER 3	QUARTER 4	TOTAL GRAMS
	841	1.82	13.67	14.58	4.1	34.17
TOTAL		1.82	13.67	14.58	4.1	34.17

STATE: VERMONT

ZIP CODE	QUARTER 1	QUARTER 2	QUARTER 3	QUARTER 4	TOTAL GRAMS
54	0	0	0.91	0	0.91
TOTAL	0	0	0.91	0	0.91

STATE: VIRGINIA

ZIP CODE	QUARTER 1	QUARTER 2	QUARTER 3	QUARTER 4	TOTAL GRAMS
229	0.46	0.46	1.37	1.37	3.66
232	3.19	1.82	0.91	0.91	6.83
240	0	0.46	0.46	0	0.92
TOTAL	3.65	2.74	2.74	2.28	11.41

STATE: WASHINGTON

ZIP CODE	QUARTER 1	QUARTER 2	QUARTER 3	QUARTER 4	TOTAL GRAMS
980	0.46	0	0.46	1.37	2.29
981	2.28	0	10.94	15.95	29.17
992	1.82	0	0	3.65	5.47
TOTAL	4.56	0	11.4	20.97	36.93

STATE: WEST VIRGINIA

ZIP CODE	QUARTER 1	QUARTER 2	QUARTER 3	QUARTER 4	TOTAL GRAMS
265	0	0	0	1.82	1.82
TOTAL	0	0	0	1.82	1.82

STATE: WISCONSIN

ZIP CODE	QUARTER 1	QUARTER 2	QUARTER 3	QUARTER 4	TOTAL GRAMS
532	5.01	2.28	2.73	6.38	16.4
537	1.37	0.91	4.1	3.65	10.03
543	0	0	0.46	0	0.46
TOTAL	6.38	3.19	7.29	10.03	26.89

DRUG CODE: 2165 DRUG NAME: BUTALBITAL

STATE: ALABAMA

ZIP CODE	QUARTER 1	QUARTER 2	QUARTER 3	QUARTER 4	TOTAL GRAMS
350	625	625	605	525	2,380
351	330	445	280	270	1,325
352	270	325	300	300	1,195
354	255	305	420	280	1,260
355	175	165	150	120	610
356	315	370	385	280	1,350
357	140	165	140	160	605
358	160	200	155	125	640
359	245	285	305	270	1,105
360	170	175	140	195	680
361	90	100	100	75	365
362	125	145	150	165	585
363	325	255	285	320	1,185
364	100	85	105	95	385
365	420	445	410	255	1,530
366	275	275	290	255	1,095
367	190	150	125	105	570
368	195	210	255	225	885
369	10	20	15	5	50
TOTAL	4,415	4,745	4,615	4,025	17,800

STATE: ALASKA

ZIP CODE	QUARTER 1	QUARTER 2	QUARTER 3	QUARTER 4	TOTAL GRAMS
995	85	130	105	60	380
996	80	75	110	100	365
997	0	15	30	25	70
998	15	45	30	10	100
999	0	0	0	10	10
TOTAL	180	265	275	205	925

STATE: AMERICAN SAMOA

ZIP CODE	QUARTER 1	QUARTER 2	QUARTER 3	QUARTER 4	TOTAL GRAMS
967	10	0	0	0	10
TOTAL	10	0	0	0	10

STATE: ARIZONA

ZIP CODE	QUARTER 1	QUARTER 2	QUARTER 3	QUARTER 4	TOTAL GRAMS
850	720	715	785	875	3,095
851	130	235	275	270	910
852	1,675	1,665	1,895	1,855	7,090
853	785	665	820	950	3,220
855	195	220	165	140	720
856	250	225	245	275	995
857	500	410	505	570	1,985
859	55	60	90	85	290
860	30	45	35	70	180
863	145	200	255	200	800
864	225	265	425	335	1,250
TOTAL	4,710	4,705	5,495	5,625	20,535

STATE: ARKANSAS

ZIP CODE	QUARTER 1	QUARTER 2	QUARTER 3	QUARTER 4	TOTAL GRAMS
716	100	105	120	120	445
717	115	70	95	85	365
718	120	170	140	105	535
719	290	235	360	325	1,210
720	365	470	465	425	1,725
721	365	310	275	270	1,220
722	295	265	305	325	1,190
723	145	165	195	205	710
724	305	300	355	340	1,300
725	120	155	180	195	650
726	160	235	185	185	765
727	605	705	565	635	2,510
728	90	85	130	75	380
729	220	165	150	225	760

TOTAL	3,295	3,435	3,520	3,515	13,765

STATE: CALIFORNIA

ZIP CODE	QUARTER 1	QUARTER 2	QUARTER 3	QUARTER 4	TOTAL GRAMS
900	910	790	1,005	820	3,525
902	935	1,080	1,030	885	3,930
903	45	30	20	20	115
904	140	120	110	105	475
905	220	175	215	215	825
906	410	495	485	415	1,805
907	265	260	290	310	1,125
908	180	180	180	150	690
910	275	230	285	250	1,040
911	95	140	105	140	480
912	155	110	85	80	430
913	1,275	1,195	1,310	1,145	4,925
914	275	275	295	365	1,210
915	30	40	70	110	250
916	90	125	135	135	485
917	1,165	1,085	1,060	865	4,175
918	45	60	60	55	220
919	115	155	110	95	475
920	1,195	1,245	1,005	1,205	4,650
921	365	325	375	420	1,485
922	470	295	330	390	1,485
923	785	895	800	780	3,260
924	50	60	20	15	145
925	1,025	1,160	1,095	870	4,150
926	1,350	1,270	1,320	1,145	5,085
927	195	230	240	300	965
928	890	1,020	995	910	3,815
930	795	690	710	690	2,885
931	115	170	145	190	620
932	515	480	420	465	1,880
933	415	495	395	445	1,750
934	510	710	590	520	2,330

	935	495	485	470	370	1,820
	936	505	420	405	460	1,790
	937	245	255	295	265	1,060
	939	370	415	465	420	1,670
	940	220	280	335	300	1,135
	941	110	115	130	120	475
	943	20	15	10	5	50
	944	30	15	25	20	90
	945	1,725	1,720	1,495	1,425	6,365
	946	120	75	95	60	350
	947	50	35	60	55	200
	948	35	35	5	20	95
	949	345	340	455	425	1,565
	950	400	435	650	460	1,945
	951	250	305	310	280	1,145
	952	485	385	495	440	1,805
	953	895	970	1,155	1,120	4,140
	954	260	310	235	280	1,085
	955	95	85	110	125	415
	956	1,110	1,185	1,165	1,165	4,625
	957	255	200	225	125	805
	958	370	325	325	330	1,350
	959	665	665	585	670	2,585
	960	215	220	185	185	805
	961	60	60	85	50	255
TOTAL		24,630	24,940	25,060	23,680	98,310

STATE:	COLORADO				
ZIP CODE	QUARTER 1	QUARTER 2	QUARTER 3	QUARTER 4	TOTAL GRAMS
800	270	245	255	340	1,110
801	270	325	280	410	1,285
802	200	180	230	265	875
803	30	5	25	40	100
804	25	40	65	55	185
805	210	240	235	260	945
806	40	110	55	80	285

807	30	5	0	0	35
808	25	45	25	35	130
809	215	195	270	245	925
810	125	105	195	150	575
811	30	45	40	20	135
812	80	40	80	100	300
813	5	5	0	15	25
814	45	65	75	105	290
815	80	115	100	100	395
816	35	70	55	55	215
TOTAL	1,715	1,835	1,985	2,275	7,810

STATE: CONNECTICUT

ZIP CODE	QUARTER 1	QUARTER 2	QUARTER 3	QUARTER 4	TOTAL GRAMS
60	920	810	880	625	3,235
61	80	145	205	75	505
62	135	65	105	65	370
63	310	280	240	245	1,075
64	695	685	865	695	2,940
65	70	90	85	85	330
66	120	65	90	80	355
67	375	375	365	350	1,465
68	460	410	360	370	1,600
69	90	115	155	65	425
TOTAL	3,255	3,040	3,350	2,655	12,300

STATE: DELAWARE

ZIP CODE	QUARTER 1	QUARTER 2	QUARTER 3	QUARTER 4	TOTAL GRAMS
197	255	215	220	230	920
198	170	175	185	185	715
199	135	160	90	90	475
TOTAL	560	550	495	505	2,110

STATE: DISTRICT OF COLUMBIA

ZIP CODE	QUARTER 1	QUARTER 2	QUARTER 3	QUARTER 4	TOTAL GRAMS
200	315	350	395	340	1,400
TOTAL	315	350	395	340	1,400

STATE: FLORIDA

ZIP CODE	QUARTER 1	QUARTER 2	QUARTER 3	QUARTER 4	TOTAL GRAMS
320	480	635	855	730	2,700
321	350	530	605	630	2,115
322	850	895	920	750	3,415
323	330	345	355	475	1,505
324	640	595	575	645	2,455
325	525	370	440	405	1,740
326	290	345	400	385	1,420
327	785	770	680	735	2,970
328	475	525	580	640	2,220
329	725	815	945	810	3,295
330	1,000	1,240	1,255	1,065	4,560
331	350	350	375	340	1,415
333	530	605	735	780	2,650
334	1,480	1,615	1,530	1,790	6,415
335	900	990	1,120	1,185	4,195
336	525	575	660	565	2,325
337	955	890	710	730	3,285
338	295	410	465	435	1,605
339	715	955	1,080	990	3,740
341	320	310	265	265	1,160
342	590	780	660	670	2,700
344	350	560	575	485	1,970
346	750	775	600	720	2,845
347	310	425	415	465	1,615
349	315	320	415	355	1,405
TOTAL	14,835	16,625	17,215	17,045	65,720

STATE: GEORGIA

ZIP CODE	QUARTER 1	QUARTER 2	QUARTER 3	QUARTER 4	TOTAL GRAMS

300	1,285	1,325	1,620	1,570	5,800
301	1,325	1,500	1,700	1,850	6,375
302	1,065	1,150	1,150	1,205	4,570
303	480	530	690	675	2,375
304	285	315	335	305	1,240
305	825	860	885	835	3,405
306	330	390	435	475	1,630
307	430	375	470	455	1,730
308	155	200	205	165	725
309	235	180	255	210	880
310	360	325	375	365	1,425
312	185	135	225	175	720
313	205	275	270	160	910
314	75	85	115	105	380
315	760	770	685	685	2,900
316	335	320	315	355	1,325
317	335	445	415	355	1,550
318	30	35	15	30	110
319	365	380	355	315	1,415
398	105	65	90	75	335
TOTAL	9,170	9,660	10,605	10,365	39,800

STATE: GUAM

ZIP CODE	QUARTER 1	QUARTER 2	QUARTER 3	QUARTER 4	TOTAL GRAMS
969	25	5	5	5	40
TOTAL	25	5	5	5	40

STATE: HAWAII

ZIP CODE	QUARTER 1	QUARTER 2	QUARTER 3	QUARTER 4	TOTAL GRAMS
967	810	820	740	670	3,040
968	300	255	305	260	1,120
TOTAL	1,110	1,075	1,045	930	4,160

STATE: IDAHO

ZIP CODE	QUARTER 1	QUARTER 2	QUARTER 3	QUARTER 4	TOTAL GRAMS
832	175	215	190	290	870
833	120	65	200	165	550
834	115	80	145	150	490
835	45	60	70	85	260
836	345	335	365	295	1,340
837	185	170	190	190	735
838	195	315	270	220	1,000
TOTAL	1,180	1,240	1,430	1,395	5,245

STATE: ILLINOIS

ZIP CODE	QUARTER 1	QUARTER 2	QUARTER 3	QUARTER 4	TOTAL GRAMS
600	1,925	1,755	1,620	1,670	6,970
601	1,010	1,190	1,045	985	4,230
602	35	30	30	45	140
603	0	35	40	30	105
604	930	1,025	955	920	3,830
605	580	640	595	480	2,295
606	475	485	420	490	1,870
607	30	20	15	40	105
608	20	0	25	15	60
609	50	115	160	130	455
610	105	50	90	95	340
611	175	220	180	195	770
612	50	30	20	20	120
613	95	65	85	70	315
614	70	95	80	105	350
615	125	135	245	150	655
616	90	105	85	80	360
617	150	150	150	95	545
618	220	175	175	135	705
619	25	40	30	30	125
620	220	265	250	240	975
622	185	220	255	195	855
623	60	80	80	70	290
624	110	150	145	85	490

625	135	175	100	125	535
626	25	30	35	40	130
627	135	120	125	150	530
628	105	155	100	205	565
629	165	185	315	280	945
TOTAL	7,300	7,740	7,450	7,170	29,660

STATE: INDIANA

ZIP CODE	QUARTER 1	QUARTER 2	QUARTER 3	QUARTER 4	TOTAL GRAMS
460	310	340	290	275	1,215
461	365	285	420	325	1,395
462	435	470	575	570	2,050
463	455	395	350	370	1,570
464	85	90	150	130	455
465	250	200	160	185	795
466	65	65	30	15	175
467	180	165	255	220	820
468	95	65	105	130	395
469	60	115	50	105	330
470	85	110	105	110	410
471	370	300	435	335	1,440
472	35	70	100	90	295
473	155	150	175	175	655
474	155	195	225	220	795
475	185	165	110	100	560
476	130	160	150	165	605
477	205	175	235	240	855
478	80	60	85	45	270
479	110	105	185	110	510
TOTAL	3,810	3,680	4,190	3,915	15,595

STATE: IOWA

ZIP CODE	QUARTER 1	QUARTER 2	QUARTER 3	QUARTER 4	TOTAL GRAMS
500	80	80	75	55	290
501	65	65	50	30	210

	502	95	85	80	105	365
	503	85	140	95	105	425
	504	45	45	40	30	160
	505	70	35	80	85	270
	506	55	30	55	70	210
	507	25	60	35	35	155
	508	5	0	0	20	25
	510	80	100	85	60	325
	511	25	60	55	35	175
	512	0	0	5	0	5
	513	5	15	20	0	40
	514	100	95	105	70	370
	515	35	15	15	25	90
	516	45	20	50	40	155
	520	65	105	105	90	365
	521	40	40	45	45	170
	522	20	60	10	30	120
	523	20	15	30	30	95
	524	20	15	30	20	85
	525	30	20	20	30	100
	526	65	45	100	60	270
	527	105	95	90	105	395
	528	50	50	60	70	230
TOTAL		1,230	1,290	1,335	1,245	5,100

STATE: KANSAS

ZIP CODE		QUARTER 1	QUARTER 2	QUARTER 3	QUARTER 4	TOTAL GRAMS
	660	390	250	230	320	1,190
	661	85	70	145	35	335
	662	340	345	595	325	1,605
	664	70	55	75	75	275
	665	65	15	50	15	145
	666	110	55	50	30	245
	667	130	145	135	115	525
	668	55	80	105	105	345
	669	0	0	5	0	5

	670	70	85	110	140	405
	671	70	80	75	80	305
	672	170	140	280	295	885
	673	160	110	140	140	550
	674	285	205	245	220	955
	675	65	45	110	65	285
	676	10	15	30	10	65
	677	10	35	35	25	105
	678	50	35	60	25	170
	679	85	110	105	110	410
TOTAL		2,220	1,875	2,580	2,130	8,805

STATE: KENTUCKY

ZIP CODE		QUARTER 1	QUARTER 2	QUARTER 3	QUARTER 4	TOTAL GRAMS
	400	80	155	190	195	620
	401	20	15	30	20	85
	402	355	300	440	375	1,470
	403	175	165	215	240	795
	404	20	20	50	45	135
	405	75	90	120	105	390
	406	5	10	30	5	50
	407	100	75	95	85	355
	408	75	45	25	50	195
	409	25	40	35	65	165
	410	330	250	385	410	1,375
	411	50	45	80	50	225
	412	15	10	10	10	45
	413	0	35	0	0	35
	414	0	10	5	5	20
	415	55	65	30	20	170
	416	30	35	30	40	135
	417	35	30	45	0	110
	418	45	20	40	55	160
	420	245	245	275	255	1,020
	421	110	95	130	80	415
	422	30	90	70	70	260

423	205	190	195	195	785
424	75	125	115	110	425
425	30	35	30	35	130
426	25	35	25	30	115
427	175	180	135	190	680
TOTAL	2,385	2,410	2,830	2,740	10,365

STATE: LOUISIANA

ZIP CODE	QUARTER 1	QUARTER 2	QUARTER 3	QUARTER 4	TOTAL GRAMS
700	1,225	1,160	990	980	4,355
701	200	290	325	265	1,080
703	60	105	70	95	330
704	610	630	635	525	2,400
705	345	295	280	265	1,185
706	220	205	220	215	860
707	245	280	280	250	1,055
708	120	175	115	135	545
710	55	20	40	105	220
711	155	165	170	155	645
712	315	275	310	370	1,270
713	200	170	115	130	615
714	400	280	285	225	1,190
TOTAL	4,150	4,050	3,835	3,715	15,750

STATE: MAINE

ZIP CODE	QUARTER 1	QUARTER 2	QUARTER 3	QUARTER 4	TOTAL GRAMS
39	15	15	15	5	50
40	90	130	130	120	470
41	45	35	30	30	140
42	75	50	60	25	210
43	100	60	55	65	280
44	25	30	35	10	100
45	5	10	20	0	35
46	40	10	25	55	130
47	170	185	115	125	595

	48	0	10	0	10	20
	49	185	100	125	80	490
TOTAL		750	635	610	525	2,520

STATE: MARYLAND

ZIP CODE		QUARTER 1	QUARTER 2	QUARTER 3	QUARTER 4	TOTAL GRAMS
	206	420	445	445	560	1,870
	207	360	345	565	375	1,645
	208	585	555	715	575	2,430
	209	130	100	115	85	430
	210	825	785	790	730	3,130
	211	235	290	345	250	1,120
	212	645	605	610	765	2,625
	214	115	85	105	90	395
	215	195	210	200	145	750
	216	140	110	100	155	505
	217	945	765	680	790	3,180
	218	180	110	130	115	535
	219	75	85	95	120	375
TOTAL		4,850	4,490	4,895	4,755	18,990

STATE: MASSACHUSETTS

ZIP CODE		QUARTER 1	QUARTER 2	QUARTER 3	QUARTER 4	TOTAL GRAMS
	10	325	310	275	345	1,255
	11	60	40	35	20	155
	12	90	50	65	55	260
	13	50	25	70	10	155
	14	85	85	50	50	270
	15	180	165	180	150	675
	16	50	20	30	10	110
	17	95	85	115	115	410
	18	280	280	235	260	1,055
	19	135	150	150	65	500
	20	110	105	105	110	430
	21	465	380	455	295	1,595

	22	0	10	10	0	20
	23	50	120	70	90	330
	24	75	70	80	60	285
	25	120	120	115	155	510
	26	150	215	125	115	605
	27	210	175	185	110	680
TOTAL		2,530	2,405	2,350	2,015	9,300

STATE: MICHIGAN

ZIP CODE		QUARTER 1	QUARTER 2	QUARTER 3	QUARTER 4	TOTAL GRAMS
	480	1,585	1,420	1,505	1,480	5,990
	481	1,595	1,430	1,630	1,650	6,305
	482	475	380	390	310	1,555
	483	1,060	1,115	1,170	1,205	4,550
	484	625	660	635	790	2,710
	485	280	225	340	175	1,020
	486	215	240	290	355	1,100
	487	140	155	180	100	575
	488	810	640	610	740	2,800
	489	275	280	280	235	1,070
	490	585	565	470	520	2,140
	491	20	0	10	40	70
	492	295	250	285	220	1,050
	493	130	120	160	90	500
	494	385	330	315	240	1,270
	495	205	185	120	130	640
	496	245	185	190	155	775
	497	185	185	200	140	710
	498	135	95	140	145	515
	499	20	35	20	100	175
TOTAL		9,265	8,495	8,940	8,820	35,520

STATE: MINNESOTA

ZIP CODE		QUARTER 1	QUARTER 2	QUARTER 3	QUARTER 4	TOTAL GRAMS
	550	145	140	160	200	645

	551	235	270	280	355	1,140
	553	310	405	320	395	1,430
	554	240	135	205	195	775
	556	5	0	0	5	10
	557	95	110	115	190	510
	558	15	15	40	20	90
	559	35	40	20	20	115
	560	75	110	50	85	320
	561	0	10	0	5	15
	562	45	70	80	35	230
	563	105	110	60	125	400
	564	45	55	70	80	250
	565	110	90	95	125	420
	566	30	25	40	40	135
	567	45	55	60	60	220
TOTAL		1,535	1,640	1,595	1,935	6,705

STATE: MISSISSIPPI

ZIP CODE		QUARTER 1	QUARTER 2	QUARTER 3	QUARTER 4	TOTAL GRAMS
	386	240	215	230	275	960
	387	40	30	70	20	160
	388	185	265	215	170	835
	389	15	0	10	10	35
	390	150	155	160	175	640
	391	335	340	305	295	1,275
	392	70	55	150	65	340
	393	195	195	180	145	715
	394	275	300	270	200	1,045
	395	425	385	400	560	1,770
	396	85	45	35	5	170
	397	115	145	160	180	600
TOTAL		2,130	2,130	2,185	2,100	8,545

STATE: MISSOURI

ZIP CODE		QUARTER 1	QUARTER 2	QUARTER 3	QUARTER 4	TOTAL GRAMS

	630	630	645	665	835	2,775
	631	635	640	1,510	1,605	4,390
	633	420	450	480	630	1,980
	634	80	110	80	75	345
	635	10	40	35	10	95
	636	220	285	295	220	1,020
	637	340	295	295	250	1,180
	638	175	125	100	90	490
	639	60	65	60	50	235
	640	470	550	500	515	2,035
	641	775	775	645	790	2,985
	644	60	105	50	90	305
	645	75	100	95	70	340
	646	115	140	90	80	425
	647	135	140	150	95	520
	648	205	165	80	150	600
	650	95	75	90	55	315
	651	15	20	35	40	110
	652	75	150	110	60	395
	653	100	80	100	75	355
	654	100	110	110	90	410
	655	150	180	155	150	635
	656	110	90	120	120	440
	657	145	210	145	150	650
	658	210	180	110	220	720
TOTAL		5,405	5,725	6,105	6,515	23,750

STATE:	MONTANA				
ZIP CODE	QUARTER 1	QUARTER 2	QUARTER 3	QUARTER 4	TOTAL GRAMS
590	80	115	65	35	295
591	185	140	245	115	685
592	15	15	10	10	50
593	55	65	35	105	260
594	30	15	30	25	100
595	10	20	5	10	45
596	40	55	30	40	165

	597	150	145	220	135	650
	598	115	125	155	100	495
	599	55	75	85	120	335
TOTAL		735	770	880	695	3,080

STATE:	NEBRASKA					
ZIP CODE		QUARTER 1	QUARTER 2	QUARTER 3	QUARTER 4	TOTAL GRAMS
	680	45	30	25	35	135
	681	180	205	250	260	895
	683	45	80	75	85	285
	684	35	25	25	25	110
	685	240	250	230	225	945
	686	80	85	80	95	340
	687	45	45	25	50	165
	688	150	130	130	155	565
	689	10	5	10	25	50
	691	75	105	135	140	455
	692	0	0	10	5	15
	693	145	120	110	100	475
TOTAL		1,050	1,080	1,105	1,200	4,435

STATE:	NEVADA					
ZIP CODE		QUARTER 1	QUARTER 2	QUARTER 3	QUARTER 4	TOTAL GRAMS
	890	670	620	855	825	2,970
	891	1,425	1,465	1,620	1,690	6,200
	893	15	40	90	75	220
	894	530	460	565	650	2,205
	895	620	675	680	550	2,525
	897	140	155	175	120	590
	898	35	5	40	25	105
TOTAL		3,435	3,420	4,025	3,935	14,815

STATE:	NEW HAMPSHIRE					
ZIP CODE		QUARTER 1	QUARTER 2	QUARTER 3	QUARTER 4	TOTAL GRAMS

	30	165	145	185	170	665
	31	130	160	110	100	500
	32	120	125	115	135	495
	33	35	10	40	50	135
	34	45	15	60	25	145
	35	30	25	40	65	160
	37	20	10	15	45	90
	38	80	125	145	125	475
TOTAL		625	615	710	715	2,665

STATE: NEW JERSEY

ZIP CODE		QUARTER 1	QUARTER 2	QUARTER 3	QUARTER 4	TOTAL GRAMS
	70	940	845	950	910	3,645
	71	50	25	55	70	200
	72	5	5	0	5	15
	73	5	25	20	0	50
	74	550	620	565	515	2,250
	75	95	110	85	80	370
	76	290	190	220	260	960
	77	895	750	560	490	2,695
	78	330	355	295	220	1,200
	79	155	155	165	85	560
	80	1,200	1,350	1,410	1,320	5,280
	81	35	40	45	15	135
	82	300	265	380	250	1,195
	83	110	135	95	85	425
	84	5	15	5	5	30
	85	235	195	170	165	765
	86	260	240	210	215	925
	87	310	240	305	230	1,085
	88	745	595	700	625	2,665
	89	55	35	70	60	220
TOTAL		6,570	6,190	6,305	5,605	24,670

STATE: NEW MEXICO

RETAIL DRUG DISTRIBUTION BY ZIP CODE WITHIN STATE BY GRAMS WT

ZIP CODE	QUARTER 1	QUARTER 2	QUARTER 3	QUARTER 4	TOTAL GRAMS
870	95	90	105	125	415
871	280	320	365	295	1,260
873	0	0	5	0	5
874	15	60	40	40	155
875	50	85	105	90	330
877	25	20	15	20	80
878	0	5	0	0	5
879	30	55	65	50	200
880	150	155	110	60	475
881	15	5	30	5	55
882	150	185	130	155	620
883	5	10	20	20	55
884	5	20	5	15	45
TOTAL	820	1,010	995	875	3,700

STATE: NEW YORK

ZIP CODE	QUARTER 1	QUARTER 2	QUARTER 3	QUARTER 4	TOTAL GRAMS
100	295	310	330	355	1,290
101	45	45	55	35	180
103	260	160	245	145	810
104	125	125	85	70	405
105	210	260	190	125	785
106	25	30	55	75	185
107	65	35	90	75	265
108	10	10	10	0	30
109	270	315	320	280	1,185
110	40	15	45	25	125
111	15	5	15	0	35
112	240	265	220	135	860
113	90	65	65	45	265
114	110	105	105	130	450
115	265	255	330	260	1,110
116	15	0	0	10	25
117	855	900	710	775	3,240
118	5	10	5	0	20

	119	120	110	130	70	430
	120	275	215	230	195	915
	121	25	65	45	45	180
	122	130	105	155	55	445
	123	55	100	60	50	265
	124	80	60	45	30	215
	125	95	165	190	90	540
	126	60	75	45	10	190
	127	90	165	70	80	405
	128	165	140	120	145	570
	129	60	70	40	80	250
	130	190	180	140	185	695
	131	70	90	65	45	270
	132	70	95	75	40	280
	133	80	75	40	50	245
	134	25	40	20	35	120
	135	25	25	30	35	115
	136	145	120	150	95	510
	137	0	20	20	15	55
	138	40	50	70	45	205
	139	45	50	45	35	175
	140	195	235	165	180	775
	141	125	105	95	65	390
	142	225	280	220	195	920
	143	0	5	15	5	25
	144	175	120	160	155	610
	145	100	125	120	110	455
	146	205	115	155	140	615
	147	60	65	75	55	255
	148	75	90	80	75	320
	149	20	10	20	20	70
TOTAL		5,965	6,075	5,765	4,970	22,775

STATE: NORTH CAROLINA

ZIP CODE	QUARTER 1	QUARTER 2	QUARTER 3	QUARTER 4	TOTAL GRAMS
270	220	185	185	185	775

271	145	95	140	105	485
272	200	130	180	215	725
273	90	110	95	75	370
274	120	140	100	60	420
275	430	350	325	455	1,560
276	185	170	300	215	870
277	10	30	50	35	125
278	155	155	165	260	735
279	230	225	255	210	920
280	480	500	410	495	1,885
281	225	295	285	305	1,110
282	205	225	300	185	915
283	535	375	430	490	1,830
284	340	345	480	425	1,590
285	390	380	290	265	1,325
286	420	525	425	390	1,760
287	560	610	570	600	2,340
288	160	120	75	100	455
289	40	65	45	45	195
TOTAL	5,140	5,030	5,105	5,115	20,390

STATE: NORTH DAKOTA

ZIP CODE	QUARTER 1	QUARTER 2	QUARTER 3	QUARTER 4	TOTAL GRAMS
580	50	55	50	50	205
581	40	35	20	25	120
582	35	30	30	10	105
583	0	25	15	0	40
584	15	25	0	5	45
585	35	35	25	45	140
586	15	0	0	5	20
587	25	25	35	35	120
588	95	105	130	160	490
TOTAL	310	335	305	335	1,285

STATE: OHIO

ZIP CODE	QUARTER 1	QUARTER 2	QUARTER 3	QUARTER 4	TOTAL GRAMS
430	310	315	445	370	1,440
431	225	135	295	290	945
432	445	300	1,260	1,570	3,575
433	85	60	95	80	320
434	85	85	105	60	335
435	345	365	520	400	1,630
436	430	290	375	455	1,550
437	175	145	150	105	575
438	5	10	15	15	45
439	40	60	100	65	265
440	500	450	535	570	2,055
441	450	345	375	465	1,635
442	270	245	190	285	990
443	90	95	65	65	315
444	230	250	200	135	815
445	35	70	40	70	215
446	200	255	235	245	935
447	230	210	190	255	885
448	80	125	120	140	465
449	40	75	100	40	255
450	160	205	350	390	1,105
451	105	85	95	135	420
452	310	225	440	400	1,375
453	290	255	380	300	1,225
454	305	285	335	325	1,250
455	35	55	50	55	195
456	190	150	175	165	680
457	70	55	80	70	275
458	180	255	180	245	860
TOTAL	5,915	5,455	7,495	7,765	26,630

STATE: OKLAHOMA

ZIP CODE	QUARTER 1	QUARTER 2	QUARTER 3	QUARTER 4	TOTAL GRAMS
730	1,050	1,070	1,020	870	4,010
731	950	955	910	765	3,580

	734	125	105	115	110	455
	735	345	325	310	240	1,220
	736	100	60	60	55	275
	737	245	275	275	255	1,050
	738	95	45	25	25	190
	739	20	10	15	5	50
	740	995	915	700	705	3,315
	741	385	365	325	325	1,400
	743	195	205	245	235	880
	744	235	295	250	250	1,030
	745	35	40	25	35	135
	746	15	20	20	20	75
	747	265	240	180	180	865
	748	240	175	200	205	820
	749	150	175	150	165	640
TOTAL		5,445	5,275	4,825	4,445	19,990

STATE: OREGON

ZIP CODE		QUARTER 1	QUARTER 2	QUARTER 3	QUARTER 4	TOTAL GRAMS
	970	365	340	450	480	1,635
	971	235	160	220	270	885
	972	250	235	255	250	990
	973	385	265	320	380	1,350
	974	460	470	490	485	1,905
	975	275	245	275	330	1,125
	976	45	35	60	50	190
	977	170	125	160	135	590
	978	160	170	145	165	640
	979	25	30	15	5	75
TOTAL		2,370	2,075	2,390	2,550	9,385

STATE: PENNSYLVANIA

ZIP CODE		QUARTER 1	QUARTER 2	QUARTER 3	QUARTER 4	TOTAL GRAMS
	150	450	285	335	355	1,425
	151	205	120	190	250	765

152	435	430	445	600	1,910
153	145	95	130	200	570
154	150	145	215	155	665
155	45	25	35	30	135
156	330	205	325	390	1,250
157	105	80	160	135	480
158	95	100	125	110	430
159	145	220	265	190	820
160	90	95	125	80	390
161	55	70	70	80	275
162	35	45	50	85	215
163	15	55	10	20	100
164	125	125	160	130	540
165	230	185	165	125	705
166	75	50	70	95	290
167	30	50	40	25	145
168	45	25	45	35	150
169	45	50	55	40	190
170	420	460	420	425	1,725
171	120	80	170	170	540
172	180	185	190	145	700
173	350	415	395	285	1,445
174	150	140	185	105	580
175	115	50	60	80	305
176	210	260	220	185	875
177	165	120	130	135	550
178	155	180	110	120	565
179	130	125	190	125	570
180	295	280	330	250	1,155
181	120	45	90	30	285
182	175	235	180	140	730
183	170	155	215	160	700
184	380	345	410	330	1,465
185	150	140	105	140	535
186	215	220	250	220	905
187	690	590	485	505	2,270
188	15	65	55	60	195

189	475	445	400	445	1,765
190	1,705	1,745	1,895	1,460	6,805
191	1,130	1,185	1,075	1,105	4,495
193	370	325	360	430	1,485
194	515	540	525	360	1,940
195	340	265	235	305	1,145
196	430	395	410	385	1,620
TOTAL	12,020	11,445	12,105	11,230	46,800

STATE: RHODE ISLAND

ZIP CODE	QUARTER 1	QUARTER 2	QUARTER 3	QUARTER 4	TOTAL GRAMS
28	290	310	260	250	1,110
29	85	105	100	70	360
TOTAL	375	415	360	320	1,470

STATE: SOUTH CAROLINA

ZIP CODE	QUARTER 1	QUARTER 2	QUARTER 3	QUARTER 4	TOTAL GRAMS
290	285	285	245	315	1,130
291	265	305	230	230	1,030
292	270	280	245	235	1,030
293	200	115	285	245	845
294	500	575	425	515	2,015
295	545	615	625	680	2,465
296	910	905	1,040	790	3,645
297	175	145	125	170	615
298	60	75	80	50	265
299	85	105	130	120	440
TOTAL	3,295	3,405	3,430	3,350	13,480

STATE: SOUTH DAKOTA

ZIP CODE	QUARTER 1	QUARTER 2	QUARTER 3	QUARTER 4	TOTAL GRAMS
570	35	85	60	85	265
571	40	60	90	100	290
572	75	80	55	30	240

	573	40	60	50	75	225
	574	140	90	130	140	500
	575	50	45	35	30	160
	576	105	75	65	90	335
	577	175	140	165	100	580
TOTAL		660	635	650	650	2,595

STATE: TENNESSEE

ZIP CODE	QUARTER 1	QUARTER 2	QUARTER 3	QUARTER 4	TOTAL GRAMS
370	370	475	510	510	1,865
371	590	650	565	630	2,435
372	215	235	300	165	915
373	395	445	465	410	1,715
374	225	160	160	150	695
376	135	180	225	250	790
377	140	130	170	215	655
378	330	275	295	345	1,245
379	150	190	245	250	835
380	185	160	185	270	800
381	95	115	180	140	530
382	130	145	170	160	605
383	315	240	310	265	1,130
384	90	60	80	155	385
385	165	165	165	170	665
TOTAL	3,530	3,625	4,025	4,085	15,265

STATE: TEXAS

ZIP CODE	QUARTER 1	QUARTER 2	QUARTER 3	QUARTER 4	TOTAL GRAMS
750	1,785	1,580	1,690	1,825	6,880
751	560	520	545	570	2,195
752	460	420	390	390	1,660
753	0	5	0	0	5
754	595	720	425	495	2,235
755	145	135	110	130	520
756	455	425	360	460	1,700

757	585	555	435	505	2,080
758	225	245	265	200	935
759	345	450	345	525	1,665
760	995	1,015	1,080	955	4,045
761	710	760	780	765	3,015
762	450	400	530	435	1,815
763	250	275	255	305	1,085
764	275	250	200	160	885
765	135	200	140	95	570
766	270	260	245	225	1,000
767	200	170	145	170	685
768	160	190	160	120	630
769	110	80	100	130	420
770	1,045	975	1,075	1,140	4,235
773	1,240	1,015	1,345	1,230	4,830
774	630	575	610	680	2,495
775	1,035	1,010	1,115	1,205	4,365
776	295	280	335	230	1,140
777	155	85	150	180	570
778	140	175	205	200	720
779	145	115	95	140	495
780	245	255	295	345	1,140
781	380	305	350	380	1,415
782	880	930	935	1,120	3,865
783	215	220	185	180	800
784	330	285	275	320	1,210
785	345	230	205	360	1,140
786	615	740	580	730	2,665
787	500	430	440	585	1,955
788	30	40	70	45	185
789	135	155	135	125	550
790	250	185	225	210	870
791	425	355	455	380	1,615
792	40	20	15	0	75
793	155	200	175	170	700
794	180	205	195	170	750
795	50	80	55	75	260

	796	140	190	180	165	675
	797	315	255	390	410	1,370
	798	10	15	30	30	85
	799	150	170	125	150	595
TOTAL		18,785	18,150	18,445	19,415	74,795

STATE: UTAH

ZIP CODE	QUARTER 1	QUARTER 2	QUARTER 3	QUARTER 4	TOTAL GRAMS
840	1,265	1,080	1,495	1,645	5,485
841	385	420	375	410	1,590
843	210	175	200	240	825
844	270	295	440	335	1,340
845	155	60	125	150	490
846	390	415	485	390	1,680
847	380	290	310	330	1,310
TOTAL	3,055	2,735	3,430	3,500	12,720

STATE: VERMONT

ZIP CODE	QUARTER 1	QUARTER 2	QUARTER 3	QUARTER 4	TOTAL GRAMS
50	0	0	5	0	5
51	5	5	5	5	20
52	25	5	5	5	40
53	5	0	0	5	10
54	40	20	40	50	150
56	0	15	10	20	45
57	45	35	15	25	120
58	30	5	15	30	80
TOTAL	150	85	95	140	470

STATE: VIRGIN ISLANDS

ZIP CODE	QUARTER 1	QUARTER 2	QUARTER 3	QUARTER 4	TOTAL GRAMS
8	0	0	20	25	45
TOTAL	0	0	20	25	45

STATE: VIRGINIA

ZIP CODE	QUARTER 1	QUARTER 2	QUARTER 3	QUARTER 4	TOTAL GRAMS
201	615	565	515	430	2,125
220	180	185	160	175	700
221	370	300	315	325	1,310
222	45	40	70	35	190
223	195	115	160	220	690
224	230	225	135	145	735
225	110	125	95	75	405
226	180	245	185	110	720
227	30	35	20	25	110
228	90	70	130	105	395
229	255	200	240	220	915
230	185	195	145	185	710
231	240	220	260	265	985
232	260	305	310	240	1,115
233	240	170	140	160	710
234	295	285	295	205	1,080
235	80	55	75	90	300
236	285	220	235	225	965
237	35	55	5	10	105
238	250	180	285	125	840
239	35	15	20	5	75
240	190	195	250	290	925
241	105	145	130	140	520
242	310	280	295	355	1,240
243	150	105	120	115	490
244	45	80	70	125	320
245	220	185	200	195	800
246	320	360	305	410	1,395
TOTAL	5,545	5,155	5,165	5,005	20,870

STATE: WASHINGTON

ZIP CODE	QUARTER 1	QUARTER 2	QUARTER 3	QUARTER 4	TOTAL GRAMS
980	700	730	785	955	3,170

	981	200	320	285	395	1,200
	982	540	630	665	745	2,580
	983	390	420	530	520	1,860
	984	175	155	235	250	815
	985	410	500	375	405	1,690
	986	155	250	300	330	1,035
	988	215	195	215	165	790
	989	175	165	185	185	710
	990	100	70	95	140	405
	991	75	120	125	135	455
	992	235	285	295	380	1,195
	993	370	360	300	315	1,345
	994	25	30	55	45	155
TOTAL		3,765	4,230	4,445	4,965	17,405

STATE: WEST VIRGINIA

ZIP CODE	QUARTER 1	QUARTER 2	QUARTER 3	QUARTER 4	TOTAL GRAMS
247	60	70	45	55	230
248	10	0	0	5	15
249	35	40	25	70	170
250	25	30	30	15	100
251	0	20	15	30	65
252	120	160	165	160	605
253	85	80	75	65	305
254	145	105	125	100	475
255	50	75	95	50	270
256	50	55	55	65	225
257	80	80	75	55	290
258	140	125	130	100	495
259	0	0	0	5	5
260	60	80	65	130	335
261	45	45	80	60	230
262	55	50	60	75	240
263	55	60	75	60	250
264	15	15	35	15	80
265	95	90	105	125	415

	266	5	0	0	0	5
	267	75	40	75	0	190
	268	10	20	25	20	75
TOTAL		1,215	1,240	1,355	1,260	5,070

STATE: WISCONSIN

ZIP CODE		QUARTER 1	QUARTER 2	QUARTER 3	QUARTER 4	TOTAL GRAMS
	530	345	275	340	180	1,140
	531	325	355	405	365	1,450
	532	130	145	230	190	695
	534	15	30	50	20	115
	535	125	145	130	140	540
	537	20	10	20	20	70
	538	10	10	10	5	35
	539	25	60	40	35	160
	540	40	20	0	20	80
	541	35	75	65	65	240
	542	55	30	55	25	165
	543	40	75	70	55	240
	544	50	80	125	85	340
	545	70	90	80	75	315
	546	35	40	25	35	135
	547	115	130	100	140	485
	548	60	40	50	30	180
	549	175	135	145	155	610
TOTAL		1,670	1,745	1,940	1,640	6,995

STATE: WYOMING

ZIP CODE		QUARTER 1	QUARTER 2	QUARTER 3	QUARTER 4	TOTAL GRAMS
	820	25	15	35	40	115
	822	55	70	60	50	235
	823	0	5	0	5	10
	824	140	155	130	130	555
	825	40	15	35	35	125
	826	30	45	100	80	255

	827	5	0	10	5	20
	828	5	15	5	10	35
	829	35	55	95	90	275
	831	5	0	0	0	5
TOTAL		340	375	470	445	1,630

DRUG CODE: 2270 DRUG NAME: PENTOBARBITAL (SCHEDULE 2)

STATE: ALABAMA

ZIP CODE	QUARTER 1	QUARTER 2	QUARTER 3	QUARTER 4	TOTAL GRAMS
350	1,510.65	1,599.51	3,287.88	2,399.26	8,797.30
351	710.89	1,688.37	888.62	887.24	4,175.12
352	394.54	2,772.10	2,464.87	926.8	6,558.31
354	1,066.34	0	1,155.20	189.12	2,410.66
355	0	533.17	533.17	533.17	1,599.51
356	355.45	0	710.89	88.86	1,155.20
357	177.72	710.89	2,310.40	888.62	4,087.63
358	266.58	0	177.72	205.07	649.37
359	888.62	977.48	1,777.23	1,154.37	4,797.70
360	1,598.41	1,331.82	976.38	3,821.04	7,727.65
361	88.86	0	2,754.71	88.86	2,932.43
362	533.17	0	710.89	1,066.34	2,310.40
363	1,066.34	88.86	1,244.06	355.45	2,754.71
364	177.17	622.03	621.48	532.62	1,953.30
365	1,244.06	2,574.78	266.58	3,554.46	7,639.88
366	0	890.42	1,421.78	0	2,312.20
367	533.17	0	177.72	355.45	1,066.34
368	533.17	710.89	888.62	533.17	2,665.85
TOTAL	11,145.14	14,500.32	22,368.20	17,579.90	65,593.56

STATE: ALASKA

ZIP CODE	QUARTER 1	QUARTER 2	QUARTER 3	QUARTER 4	TOTAL GRAMS
995	2,843.57	1,886.14	3,376.74	1,711.15	9,817.60
996	622.03	1,421.78	977.48	2,488.12	5,509.41
997	1,599.51	1,332.92	1,421.78	1,332.92	5,687.13
998	622.03	355.45	444.31	355.45	1,777.24

TOTAL		5,687.14	4,996.29	6,220.31	5,887.64	22,791.38

STATE:	ARIZONA					
ZIP CODE		QUARTER 1	QUARTER 2	QUARTER 3	QUARTER 4	TOTAL GRAMS
	850	6,799.04	3,520.74	9,301.38	10,704.39	30,325.55
	851	533.17	3,462.30	2,043.81	3,998.77	10,038.05
	852	12,253.22	10,837.80	11,285.77	16,172.24	50,549.03
	853	9,063.87	15,106.46	13,684.67	15,373.04	53,228.04
	855	1,155.20	0	0	1,421.78	2,576.98
	856	444.31	2,043.81	1,510.65	3,021.29	7,020.06
	857	8,944.94	5,493.46	9,201.49	10,510.26	34,150.15
	859	0	444.31	1,066.34	0	1,510.65
	860	537.73	401.02	1,952.11	2,071.16	4,962.02
	863	4,176.49	2,399.26	5,776	5,332.60	17,684.35
	864	977.48	888.62	622.03	533.17	3,021.30
TOTAL		44,885.45	44,597.78	56,444.25	69,138.70	215,066.18

STATE:	ARKANSAS					
ZIP CODE		QUARTER 1	QUARTER 2	QUARTER 3	QUARTER 4	TOTAL GRAMS
	717	0	533.17	0	444.31	977.48
	718	0	622.03	0	177.72	799.75
	719	799.48	1,066.34	799.75	710.89	3,376.46
	720	710.89	266.58	1,777.23	888.62	3,643.32
	721	888.62	2,754.71	2,756.53	1.82	6,401.68
	722	533.17	4,470.42	1,094.76	732.22	6,830.57
	723	0	177.72	888.62	533.17	1,599.51
	724	2,221.54	799.75	799.75	622.03	4,443.07
	726	533.17	1,155.20	266.58	890.44	2,845.39
	727	977.2	978.57	533.17	355.17	2,844.11
	728	0	1,066.34	0	0	1,066.34
	729	888.62	0	0	710.89	1,599.51
TOTAL		7,552.69	13,890.83	8,916.39	6,067.28	36,427.19

STATE: CALIFORNIA

RETAIL DRUG DISTRIBUTION BY ZIP CODE WITHIN STATE BY GRAMS WT

ZIP CODE	QUARTER 1	QUARTER 2	QUARTER 3	QUARTER 4	TOTAL GRAMS
900	4,580.24	1,399.45	1,985.03	2,230.20	10,194.92
902	355.45	8,897.54	9,597.04	11.39	18,861.42
905	0	68.36	0	39.19	107.55
906	0	2.28	1,066.34	1,244.06	2,312.68
907	0	533.17	533.17	355.45	1,421.79
908	29.62	1,066.34	0	13.67	1,109.63
910	3,050.91	1,296.47	1,254.09	1,584.92	7,186.39
911	2,132.68	0	0	2,223.82	4,356.50
912	1,332.92	0	177.72	1,510.65	3,021.29
913	3,998.77	4,989	6,402.59	4,889.66	20,280.02
914	266.58	710.89	447.04	0	1,424.51
915	20.51	0	0	16.86	37.37
916	266.58	88.86	444.31	622.03	1,421.78
917	1,338.39	22,308.79	2,262.64	3,199.01	29,108.83
918	0	710.89	0	0	710.89
919	1,822.80	1,244.06	2,665.85	3,203.57	8,936.28
920	8,175.26	4,976.24	10,245.05	9,311.32	32,707.87
921	2,615.72	1,471	2,115.82	2,016.02	8,218.56
922	4,354.21	9,152.73	7,376.42	7,819.81	28,703.17
923	3,533.67	8,245.80	5,670.64	9,227.36	26,677.47
924	0	2,156.37	2,132.68	9.11	4,298.16
925	6,137.37	15,204.89	12,575.95	12,225.06	46,143.27
926	991.15	2,226.09	2,231.11	1,697.11	7,145.46
927	303.04	339.5	9.11	4,701.46	5,353.11
928	3,921.30	2,021.03	8,568.98	2,422.05	16,933.36
930	2,134.50	1,421.78	1,956.78	1,690.19	7,203.25
931	355.45	355.45	444.31	355.45	1,510.66
932	9,599.78	5,164.90	5,159.44	5,600.10	25,524.22
933	8,712.98	5,420.55	6,603.09	8,175.26	28,911.88
934	2,852.68	3,998.77	3,465.60	710.89	11,027.94
935	6,589.42	2,665.85	8,708.43	6,220.31	24,184.01
936	3,821.04	1,435.46	3,113.34	2,493.59	10,863.43
937	9,064.78	96.15	9,809.40	196.86	19,167.19
939	10,125.65	3,111.06	2,223.36	2,182.61	17,642.68
940	1,332.92	622.03	1,688.37	977.48	4,620.80
941	1,025.78	219.19	31.44	1,190.74	2,467.15

	943	533.17	0.91	897.73	100.25	1,532.06
	944	977.48	177.72	888.62	355.45	2,399.27
	945	10,406.27	5,343.08	8,274.95	6,333.68	30,357.98
	946	1,102.79	1,510.65	534.99	1,252.63	4,401.06
	947	622.03	533.17	1,066.34	1,421.78	3,643.32
	948	0	0	0	266.58	266.58
	949	5,864.58	1,244.06	2,332	8,001.18	17,441.82
	950	1,874.93	1,244.06	5,603.74	3,375.91	12,098.64
	951	532.89	177.45	314.34	538.09	1,562.77
	952	3,021.29	2,754.71	1,777.23	3,554.46	11,107.69
	953	18,926.85	8,976.83	10,143.06	16,349.21	54,395.95
	954	1,954.95	4,268.53	3,554.46	988.59	10,766.53
	955	3,021.29	710.89	1,332.92	2,488.12	7,553.22
	956	5,254.22	10,762.08	9,921.96	13,523.62	39,461.88
	957	177.72	0	534.99	88.86	801.57
	958	2,274.40	8,781.34	988.87	2,335.46	14,380.07
	959	6,574.93	4,265.35	9,681.78	3,375.91	23,897.97
	960	5,101.56	7,642.09	9,152.73	6,590.33	28,486.71
	961	355.45	1,244.06	1,510.65	1,332.92	4,443.08
TOTAL		173,418.95	173,257.92	189,476.50	172,640.29	708,793.66

STATE: COLORADO

ZIP CODE		QUARTER 1	QUARTER 2	QUARTER 3	QUARTER 4	TOTAL GRAMS
	800	1,772.67	2,470.07	3,380.84	3,886.21	11,509.79
	801	4,891.39	3,287.88	5,470.68	9,931.98	23,581.93
	802	8,086.40	1,877.48	3,287.88	7,022.79	20,274.55
	803	1,688.37	355.45	177.72	1,521.58	3,743.12
	804	6,486.89	2,754.71	2,576.98	7,552.95	19,371.53
	805	5,776	7,309.43	7,123.05	7,197.78	27,406.26
	806	2,754.71	1,332.92	1,688.37	1,692.93	7,468.93
	807	266.58	533.17	266.58	0	1,066.33
	808	888.62	2,132.68	710.89	355.45	4,087.64
	809	2,932.43	3,021.29	383.7	3,554.46	9,891.88
	810	2,665.85	3,910.82	3,376.74	2,843.57	12,796.98
	811	177.72	710.89	0	710.07	1,598.68
	812	888.62	531.52	88.86	1,423.61	2,932.61

	813	177.72	1,510.65	1,066.34	2,932.43	5,687.14
	814	1,244.06	4,533.21	266.58	4,354.21	10,398.06
	815	4,265.35	1,332.92	2,000.52	3,110.15	10,708.94
	816	2,310.40	977.48	1,155.20	2,488.12	6,931.20
TOTAL		47,273.78	38,582.57	33,020.93	60,578.29	179,455.57

STATE: CONNECTICUT

ZIP CODE	QUARTER 1	QUARTER 2	QUARTER 3	QUARTER 4	TOTAL GRAMS
60	2,570.39	4,172.09	3,814.45	5,067.27	15,624.20
61	287.09	489.88	401.02	1,489.68	2,667.67
62	710.89	1,155.20	2,043.81	3,465.60	7,375.50
63	1,510.65	266.58	622.03	1,599.51	3,998.77
64	7,107.27	5,242.83	7,286.64	6,930.92	26,567.66
65	739.15	0	888.62	797.02	2,424.79
66	186.84	271.14	177.72	266.58	902.28
67	1,243.51	2,843.02	4,531.66	1,954.40	10,572.59
68	533.17	0	630.23	0	1,163.40
69	0	88.86	1,066.34	0	1,155.20
TOTAL	14,888.96	14,529.60	21,462.52	21,570.98	72,452.06

STATE: DELAWARE

ZIP CODE	QUARTER 1	QUARTER 2	QUARTER 3	QUARTER 4	TOTAL GRAMS
197	0	91.14	0	307.68	398.82
198	76.56	109.37	111.65	61.06	358.64
199	710.89	1,775.58	1,597.86	2,307.10	6,391.43
TOTAL	787.45	1,976.09	1,709.51	2,675.84	7,148.89

STATE: DISTRICT OF COLUMBIA

ZIP CODE	QUARTER 1	QUARTER 2	QUARTER 3	QUARTER 4	TOTAL GRAMS
200	195.95	9.11	871.3	1,476.47	2,552.83
TOTAL	195.95	9.11	871.3	1,476.47	2,552.83

STATE: FLORIDA

ZIP CODE	QUARTER 1	QUARTER 2	QUARTER 3	QUARTER 4	TOTAL GRAMS
320	2,842.37	1,422.24	2,935.62	2,399.26	9,599.49
321	716.36	4,909.71	1,156.11	3,909.91	10,692.09
322	1,244.69	426.08	4,096.92	2,441.81	8,209.50
323	1,244.06	1,421.78	6,131.44	2,406.55	11,203.83
324	1,421.78	3,999.22	2,135.41	3,110.61	10,667.02
325	5,152.59	9,963.42	621.21	1,244.06	16,981.28
326	1,453.68	1,066.34	1,424.06	2,091.66	6,035.74
327	3,648.79	4,195.44	5,874.23	4,479.15	18,197.61
328	430.64	710.34	5,142.76	2,328.71	8,612.45
329	5,804.79	3,465.60	4,175.67	3,287.33	16,733.39
330	629.78	991.15	1,531.78	1,642.34	4,795.05
331	116.2	474.56	401.02	861.27	1,853.05
333	3,998.77	5,243.28	4,443.08	3,547.62	17,232.75
334	318.72	7,247.63	4,022.83	713.44	12,302.62
335	2,579.34	2,221.54	2,314.04	1,687.82	8,802.74
336	5,566.38	588.58	1,211.71	5,826.58	13,193.25
337	1,371.66	4,958.02	3,519.73	3,065.95	12,915.36
338	3,529.85	8,120.30	1,690.83	9,125.39	22,466.37
339	2,487.30	2,276.22	3,749.59	1,692.01	10,205.12
341	653.93	266.58	444.31	3,380.38	4,745.20
342	622.03	822.54	1,512.47	2,595.21	5,552.25
344	12,350.10	3,819.94	5,330.32	3,496.67	24,997.03
346	2,399.26	2,488.12	2,487.30	2,842.74	10,217.42
347	2,310.40	2,398.71	3,554.19	266.31	8,529.61
349	2,221.54	2,665.85	535.45	4,443.08	9,865.92
TOTAL	65,115.01	76,163.19	70,442.08	72,885.86	284,606.14

STATE: GEORGIA

ZIP CODE	QUARTER 1	QUARTER 2	QUARTER 3	QUARTER 4	TOTAL GRAMS
300	4,192.95	7,280.59	5,595.13	5,819.18	22,887.85
301	4,892.58	3,466.51	5,336.06	4,533.48	18,228.63
302	1,596.76	1,332.92	1,154.92	3,195.44	7,280.04
303	857.89	2,591.57	457.98	1,476.47	5,383.91
304	0	0	444.31	2,843.57	3,287.88
305	1,656.47	1,332.92	1,786.07	3,296.71	8,072.17

	306	2,487.57	3,376.74	2,664.75	5,153.97	13,683.03
	307	1,510.65	2,397.06	4,354.21	888.62	9,150.54
	308	4,265.35	177.72	0	0	4,443.07
	309	2,221.54	4.56	4,525.10	539.55	7,290.75
	310	177.72	710.89	443.21	977.48	2,309.30
	312	365.47	2,150.90	1,958.60	280.26	4,755.23
	313	1,155.20	1,510.65	355.45	5,864.86	8,886.16
	314	177.45	92.23	632.51	266.31	1,168.50
	315	3,375.09	1,509	3,021.29	2,238.12	10,143.50
	316	1,071.81	0	1,071.81	0	2,143.62
	317	6,309.17	4,176.49	3,998.77	5,776	20,260.43
	318	0	531.52	0	531.52	1,063.04
	319	622.03	1,155.20	799.75	533.17	3,110.15
	398	88.86	88.86	88.86	0	266.58
TOTAL		37,024.56	33,886.33	38,688.78	44,214.71	153,814.38

STATE: GUAM

ZIP CODE		QUARTER 1	QUARTER 2	QUARTER 3	QUARTER 4	TOTAL GRAMS
	969	0	3,215.42	0	3,215.42	6,430.84
TOTAL		0	3,215.42	0	3,215.42	6,430.84

DRUG CODE:2270DRUG NAME:PENTOBARBITAL (SCHEDULE 2)

STATE: HAWAII

ZIP CODE		QUARTER 1	QUARTER 2	QUARTER 3	QUARTER 4	TOTAL GRAMS
	967	4,287.23	888.62	8,204.42	4,545.61	17,925.88
	968	344.05	341.78	9,020.13	312.15	10,018.11
TOTAL		4,631.28	1,230.40	17,224.55	4,857.76	27,943.99

STATE: IDAHO

ZIP CODE		QUARTER 1	QUARTER 2	QUARTER 3	QUARTER 4	TOTAL GRAMS
	832	622.03	91.6	2,491.77	1,689.28	4,894.68
	833	355.45	5,687.14	3,199.01	1,510.65	10,752.25

	834	1,510.65	3,397.24	4,354.21	4,087.63	13,349.73
	835	533.17	1,244.06	1,510.65	1,244.06	4,531.94
	836	9,073.90	4,535.13	8,624.01	12,447.90	34,680.94
	837	544.11	3,188.99	4,464.95	943.3	9,141.35
	838	3,502.05	1,954.95	3,820.49	3,821.04	13,098.53
TOTAL		16,141.36	20,099.11	28,465.09	25,743.86	90,449.42

STATE:	ILLINOIS					
ZIP CODE		QUARTER 1	QUARTER 2	QUARTER 3	QUARTER 4	TOTAL GRAMS
	600	10,347.57	12,501.90	10,929.96	14,190.77	47,970.20
	601	8,896.54	3,960.39	7,907.30	7,853.35	28,617.58
	602	265.76	88.86	532.34	93.42	980.38
	603	0	977.48	0	622.03	1,599.51
	604	5,174.02	5,927.37	1,600.78	10,685.89	23,388.06
	605	4,176.49	2,771.93	5,598.27	3,578.97	16,125.66
	606	3,534.86	3,015.37	5,026.83	23,400.64	34,977.70
	607	533.17	444.31	0	622.03	1,599.51
	608	0	88.86	0	0	88.86
	609	0	2,219.34	565.07	1,244.06	4,028.47
	610	2,665.85	4,709.66	2,043.81	5,420.55	14,839.87
	611	621.76	2,074.35	1,068.80	1,510.65	5,275.56
	612	0	0	977.48	533.17	1,510.65
	613	2,129.38	1,594.56	1,595.11	3,016.89	8,335.94
	614	1,954.95	2,576.98	2,851.77	1,244.06	8,627.76
	615	710.89	710.89	355.45	2,043.81	3,821.04
	616	2,497.24	2,791.16	0	3,558.11	8,846.51
	617	977.48	533.17	533.17	710.89	2,754.71
	618	2,052.93	4,623.53	3,471.07	3,914.19	14,061.72
	619	444.31	977.48	710.89	1,775.58	3,908.26
	620	888.62	177.72	2,754.71	1,155.20	4,976.25
	622	1,155.20	2,043.81	1,421.78	1,331.55	5,952.34
	623	88.86	1,155.20	1,244.06	355.45	2,843.57
	624	3,199.01	0	888.07	3,643.32	7,730.40
	625	710.89	533.17	622.03	888.62	2,754.71
	626	1,244.06	1,510.10	1,332.92	3,109.60	7,196.68
	627	199.96	461.99	2,132.68	371.3	3,165.93

	628	533.17	710.89	355.45	1,421.23	3,020.74
	629	1,155.20	888.62	3,021.29	3,198.19	8,263.30
TOTAL		56,158.17	60,069.09	59,541.09	101,493.52	277,261.87
STATE:	INDIANA					
ZIP CODE		QUARTER 1	QUARTER 2	QUARTER 3	QUARTER 4	TOTAL GRAMS
	460	3,199.01	3,552.81	4,174.84	4,363.33	15,289.99
	461	1,155.20	2,930.51	4,178.22	3,287.88	11,551.81
	462	5,184.40	717.73	1,615.72	2,671.95	10,189.80
	463	3,109.60	4,798.52	2,220.71	8,527.95	18,656.78
	464	0	355.45	533.17	4.56	893.18
	465	4,176.49	3,466.51	4,089.45	4,444.90	16,177.35
	466	355.45	1,510.65	266.58	534.99	2,667.67
	467	5,598.27	3,021.29	3,110.15	5,425.11	17,154.82
	468	3,021.29	4,361.50	3,943.17	4,450.82	15,776.78
	469	2,399.26	1,332.92	3,373.99	4,210.67	11,316.84
	470	0	0	0	177.72	177.72
	471	1,510.65	1,957.23	2,315.87	2,045.82	7,829.57
	472	799.75	1,599.51	2,398.44	1,865.27	6,662.97
	473	3,293.34	977.48	1,331.27	2,400.17	8,002.26
	474	2,319.59	1,598.41	3,310.66	3,107.68	10,336.34
	475	710.89	0	1,510.65	1,243.79	3,465.33
	476	2,394.04	533.17	976.1	2,216.59	6,119.90
	477	9.11	2,942.17	1,070.44	1,601.79	5,623.51
	478	1,420.13	1,330.72	1,066.34	2,751.41	6,568.60
	479	621.21	1,063.86	1,953.30	1,685.07	5,323.44
TOTAL		41,277.68	38,050.44	43,439.07	57,017.47	179,784.66
STATE:	IOWA					
ZIP CODE		QUARTER 1	QUARTER 2	QUARTER 3	QUARTER 4	TOTAL GRAMS
	500	888.62	5,242.83	1,244.06	7,286.64	14,662.15
	501	88.86	624.76	977.48	891.35	2,582.45
	502	355.45	444.31	977.48	622.94	2,400.18
	503	1,519.76	1,337.48	2,132.68	892.26	5,882.18
	504	2.73	1.82	177.17	270.59	452.31
	505	1,066.34	0	355.45	888.62	2,310.41

	506	266.58	0	266.58	1,782.70	2,315.86
	507	0	0	888.62	0	888.62
	508	0	0	355.45	355.45	710.9
	510	0	710.89	355.45	0	1,066.34
	511	533.17	710.89	266.58	888.62	2,399.26
	513	533.17	266.58	0	1,866.09	2,665.84
	515	533.17	0	177.72	2,049.28	2,760.17
	516	535.9	535.9	0	0	1,071.80
	520	0	177.72	1,155.20	444.31	1,777.23
	521	0	177.72	0	0	177.72
	522	872.67	36	66.08	1,220.36	2,195.11
	523	1,155.20	444.31	533.17	622.03	2,754.71
	524	444.31	1,599.23	2,310.40	1,903.18	6,257.12
	525	0	1,421.78	266.58	710.89	2,399.25
	526	1,599.51	1,777.23	799.75	4,620.80	8,797.29
	527	177.72	0	355.45	1,421.78	1,954.95
	528	710.89	1,066.34	533.17	710.89	3,021.29
TOTAL		11,284.05	16,575.79	14,194.52	29,448.78	71,503.14

STATE:	KANSAS				
ZIP CODE	QUARTER 1	QUARTER 2	QUARTER 3	QUARTER 4	TOTAL GRAMS
660	1,777.69	2,132.68	3,643.32	5,153.97	12,707.66
661	341.78	0	266.58	355.45	963.81
662	453.42	444.31	355.45	6,494.18	7,747.36
664	88.86	266.58	710.89	2,043.81	3,110.14
665	7,108.92	177.72	4,620.80	888.62	12,796.06
666	1,672.42	622.03	1,599.51	710.89	4,604.85
667	0	0	710.89	2,488.12	3,199.01
668	0	0	0	177.72	177.72
669	0	177.72	0	0	177.72
670	1,155.20	1,688.37	2,399.26	1,510.65	6,753.48
671	1,066.34	1,244.06	1,866.09	1,954.95	6,131.44
672	3,080.53	2,540.07	2,313.59	4,809.91	12,744.10
673	533.17	622.03	0	888.62	2,043.82
674	799.75	1,421.78	356.81	88.86	2,667.20
675	1,954.95	533.17	533.17	3,998.77	7,020.06

	676	177.72	533.17	267.5	888.62	1,867.01
	677	0	355.45	357.27	0	712.72
	678	1,510.65	1,688.37	1,332.92	533.17	5,065.11
	679	0	0	0.91	0	0.91
TOTAL		21,721.40	14,447.51	21,334.96	32,986.31	90,490.18

STATE: KENTUCKY

ZIP CODE		QUARTER 1	QUARTER 2	QUARTER 3	QUARTER 4	TOTAL GRAMS
	400	1,421.78	977.48	3,020.74	532.62	5,952.62
	401	0	886.97	0	177.72	1,064.69
	402	2,339	2,894.95	1,955.02	7,310.42	14,499.39
	403	1,332.92	4,172.35	5,597.71	1,155.20	12,258.18
	404	2,220.71	2,488.12	1,598.41	2,398.99	8,706.23
	405	1,083.19	0	4,228.74	2,962.19	8,274.12
	406	888.62	267.04	177.72	88.59	1,421.97
	407	177.72	1,066.34	1,066.34	1,244.06	3,554.46
	408	0	0	0	444.31	444.31
	409	88.86	444.31	444.31	177.72	1,155.20
	410	886.69	1,604.43	2,129.93	1,862.79	6,483.84
	411	0	0	0	0.91	0.91
	412	0	0	0	265.76	265.76
	417	0	0	0	355.45	355.45
	420	2,843.57	2,668.58	3,110.15	3,643.32	12,265.62
	421	2,576.98	1,510.65	4,354.21	1,065.24	9,507.08
	422	1,510.65	888.62	1,066.34	2,488.12	5,953.73
	423	1,331.82	443.76	1,154.92	2,660.62	5,591.12
	424	533.17	1,507.90	710.89	1,155.20	3,907.16
	425	354.35	0	531.52	88.86	974.73
	426	0	177.17	88.86	533.17	799.2
	427	886.97	888.62	3,999.40	2,132.68	7,907.67
TOTAL		20,477	22,887.29	35,235.21	32,743.94	111,343.44

STATE: LOUISIANA

ZIP CODE	QUARTER 1	QUARTER 2	QUARTER 3	QUARTER 4	TOTAL GRAMS

700	2,491.03	3,109.88	2,501.79	3,921.75	12,024.45
701	476.21	1,421.78	2,079.81	808.87	4,786.67
703	888.62	3,200.65	1,332.92	2,131.85	7,554.04
704	4,706.91	4,354.21	5,965.11	5,864.86	20,891.09
705	3,590.68	4,411.57	1,785.14	3,707.99	13,495.38
706	531.52	0	5,154.14	1,336.57	7,022.23
707	177.17	710.89	266.58	444.31	1,598.95
708	2,178.25	0	1,421.78	1,648.81	5,248.84
710	1,063.04	0	265.76	1,420.96	2,749.76
711	5,952.07	354.35	2,754.71	1,474.54	10,535.67
712	1,433.99	989.69	1,426.98	1,163.03	5,013.69
713	0	2,131.85	1,510.10	1,332.92	4,974.87
714	1,331.27	799.75	799.75	1,509	4,439.77
TOTAL	24,820.76	21,484.62	27,264.57	26,765.46	100,335.41

STATE: MAINE

ZIP CODE	QUARTER 1	QUARTER 2	QUARTER 3	QUARTER 4	TOTAL GRAMS
39	1,154.92	0	1,332.92	0	2,487.84
40	4,087.63	4,621.25	4,354.21	5,242.83	18,305.92
41	1,421.78	856.26	1,545.28	2,043.81	5,867.13
42	5,065.11	1,332.92	3,732.18	3,109.60	13,239.81
43	1,599.51	533.17	888.62	533.17	3,554.47
44	4,354.21	4,354.21	3,287.88	8,015.76	20,012.06
45	266.58	88.59	88.59	976.65	1,420.41
46	1,155.20	2,576.98	177.72	1,244.06	5,153.96
47	355.45	355.45	533.17	266.58	1,510.65
48	266.31	1,154.10	1,332.92	621.21	3,374.54
49	1,599.51	1,244.06	2,132.68	977.48	5,953.73
TOTAL	21,326.21	17,116.99	19,406.17	23,031.15	80,880.52

STATE: MARYLAND

ZIP CODE	QUARTER 1	QUARTER 2	QUARTER 3	QUARTER 4	TOTAL GRAMS
206	2,232.93	443.48	2,410.65	1,687.54	6,774.60
207	2,499.06	1,155.20	4,128.64	799.75	8,582.65
208	904.56	445.86	1,515.66	1,155.20	4,021.28

	209	0	0	0	355.45	355.45
	210	2,754.16	3,287.88	3,641.02	4,620.52	14,303.58
	211	1,510.65	888.62	2,132.68	7,211.45	11,743.40
	212	5,575.49	180	858.08	2,335.92	8,949.49
	214	533.17	0	1,066.34	0	1,599.51
	215	533.17	0	977.48	533.17	2,043.82
	216	1,953.30	531.52	88.86	531.52	3,105.20
	217	2,487.02	6,753.47	6,130.62	7,194.48	22,565.59
	218	620.66	533.17	444.31	444.31	2,042.45
	219	1,421.78	444.31	444.31	533.17	2,843.57
TOTAL		23,025.95	14,663.51	23,838.65	27,402.48	88,930.59

STATE: MASSACHUSETTS

ZIP CODE		QUARTER 1	QUARTER 2	QUARTER 3	QUARTER 4	TOTAL GRAMS
	010	1,776.96	4,353.66	2,043.26	1,598.13	9,772.01
	011	888.62	0	0	1,165.22	2,053.84
	012	710.89	1,332.92	444.31	1,155.20	3,643.32
	013	177.72	1,777.23	444.31	1,688.37	4,087.63
	014	977.48	444.31	3,643.32	1,599.51	6,664.62
	015	1,954.40	1,421.78	2,932.43	5,680.54	11,989.15
	016	22.79	13.67	931.07	633.78	1,601.31
	017	1,155.20	1,954.68	3,287.88	799.75	7,197.51
	018	444.31	4,265.35	1,155.20	2,576.98	8,441.84
	019	1,510.65	1,332.92	1,066.34	2,575.61	6,485.52
	020	5,172.20	1,297.38	2,521.84	1,534.80	10,526.22
	021	0	0	0	15.95	15.95
	022	1,599.51	444.31	2,310.40	2,221.54	6,575.76
	023	622.03	533.17	799.75	1,421.78	3,376.73
	024	533.17	1,332.92	1,066.34	1,332.92	4,265.35
	025	1,066.34	1,244.06	2,665.85	2,399.26	7,375.51
	026	710.89	2,132.68	2,043.81	1,599.51	6,486.89
TOTAL		21,722.42	26,302.17	31,710.32	33,023.51	112,758.42

STATE: MICHIGAN

ZIP CODE		QUARTER 1	QUARTER 2	QUARTER 3	QUARTER 4	TOTAL GRAMS
	480	9,441.83	8,642.35	13,620.14	9,670.41	41,374.73
	481	14,898.45	11,543.23	14,289.01	17,603.68	58,334.37
	482	3,618.26	4,536.04	3,271.93	8,144.73	19,570.96
	483	5,889.92	4,271.91	7,914.41	7,642.54	25,718.78
	484	5,418.63	5,507.49	9,237.28	7,466.64	27,630.04
	485	2,132.68	1,244.06	3,465.60	183.19	7,025.53
	486	2,142.49	2,579.44	1,872.02	1,272.76	7,866.71
	487	533.17	533.17	888.62	3,998.77	5,953.73
	488	11,465.32	6,219.76	16,083.11	10,573.42	44,341.61
	489	4,553.81	799.75	8.2	1,435.46	6,797.22
	490	4,870.88	14,304.42	13,642.56	17,229.47	50,047.33
	491	1,244.06	622.03	2,221.54	533.17	4,620.80
	492	2,310.40	810.32	3,020.19	1,333.83	7,474.74
	493	1,954.95	3,199.01	5,599.19	3,554.46	14,307.61
	494	4,621.71	3,825.60	3,379.47	5,871.24	17,698.02
	495	6,266.79	3,340.74	4,198.36	3,919.02	17,724.91
	496	2,132.68	2,403.82	3,020.74	1,887.23	9,444.47
	497	3,199.01	622.03	1,866.09	1,421.78	7,108.91
	498	2,840.27	1,421.78	2,132.68	1,954.95	8,349.68
	499	0	1,686.72	354.9	1,509.55	3,551.17
TOTAL		89,535.31	78,113.67	110,086.04	107,206.30	384,941.32

STATE: MINNESOTA

ZIP CODE		QUARTER 1	QUARTER 2	QUARTER 3	QUARTER 4	TOTAL GRAMS
	550	2,759.26	5,153.97	3,909.91	4,887.38	16,710.52
	551	3,023.57	4,903.33	3,988.29	10,745.41	22,660.60
	553	4,798.52	7,642.09	7,730.95	8,975.01	29,146.57
	554	4,010.88	4,074.04	3,122.54	15,108.08	26,315.54
	556	0	0	355.45	0	355.45
	557	1,151.90	1,421.78	1,599.51	1,954.95	6,128.14
	558	177.72	1,166.59	444.31	538.64	2,327.26
	559	2,665.48	2,494.50	1,333.01	2,490.40	8,983.39
	560	1,420.68	3,021.29	2,043.81	533.17	7,018.95
	561	266.58	177.72	88.86	0	533.16
	562	977.48	799.75	533.17	1,155.20	3,465.60

	563	1,510.10	2,221.54	3,731.63	2,754.71	10,217.98
	564	355.45	266.58	1,777.23	0	2,399.26
	565	2,665.85	1,688.37	0	1,421.78	5,776
	566	444.31	0	0	444.31	888.62
	567	0	1,066.34	531.52	354.9	1,952.76
TOTAL		26,227.78	36,097.89	31,190.19	51,363.94	144,879.80

STATE:	MISSISSIPPI					
ZIP CODE		QUARTER 1	QUARTER 2	QUARTER 3	QUARTER 4	TOTAL GRAMS
	386	3,376.74	1,866.09	0	1,777.23	7,020.06
	387	0	0	355.45	0	355.45
	388	0	355.45	728.21	0	1,083.66
	389	0	533.17	0	0	533.17
	390	0	177.72	0	177.72	355.44
	391	355.45	265.76	0	355.45	976.66
	392	3,557.65	0	2,535.51	2,843.57	8,936.73
	393	974.73	0	177.72	1,063.04	2,215.49
	394	462.54	906.84	533.17	1,066.34	2,968.89
	395	2,310.40	888.62	1,420.13	799.75	5,418.90
	396	88.59	531.52	0	0	620.11
	397	0	0	531.52	1,507.35	2,038.87
TOTAL		11,126.10	5,525.17	6,281.71	9,590.45	32,523.43

STATE:	MISSOURI					
ZIP CODE		QUARTER 1	QUARTER 2	QUARTER 3	QUARTER 4	TOTAL GRAMS
	630	5,506.11	2,219.89	5,774.35	7,021.88	20,522.23
	631	3,101.49	2,084.83	5,736.81	3,764.44	14,687.57
	633	3,020.74	3,558.55	5,962.83	3,907.98	16,450.10
	634	0	0	1,421.78	1,066.34	2,488.12
	635	0	178.63	979.3	356.36	1,514.29
	636	1,155.20	2,488.12	1,510.65	2,399.26	7,553.23
	637	1,155.20	1,782.70	801.58	1,345.68	5,085.16
	638	88.86	177.72	177.72	355.45	799.75
	639	0	355.45	177.72	0	533.17
	640	6,664.61	4,620.80	7,127.15	11,374.27	29,786.83
	641	901.37	747.07	1,440.92	2,819.14	5,908.50

	644	2,132.68	977.48	1,510.65	3,021.29	7,642.10
	646	355.45	0	0	0	355.45
	647	533.17	977.48	444.31	1,244.06	3,199.02
	648	977.48	1,599.51	622.03	2,310.40	5,509.42
	650	1,424.52	0	0	1,066.34	2,490.86
	651	1,599.51	88.86	1,332.92	88.86	3,110.15
	652	5,493.46	1,214.44	5,202.27	232.86	12,143.03
	653	0	0	888.62	0	888.62
	654	355.45	0	533.17	0	888.62
	655	620.38	886.97	1,333.38	886.97	3,727.70
	656	1,242.41	1,688.37	2,576.98	5,153.97	10,661.73
	657	533.17	266.58	1,332.92	1,155.20	3,287.87
	658	91.6	192.31	266.58	1,243.79	1,794.28
TOTAL		36,952.86	26,105.76	47,154.64	50,814.54	161,027.80

STATE: MONTANA

ZIP CODE		QUARTER 1	QUARTER 2	QUARTER 3	QUARTER 4	TOTAL GRAMS
	590	1,510.10	1,155.20	889.53	5,598.27	9,153.10
	591	1,246.80	800.66	1,868.83	1,156.11	5,072.40
	592	0	533.17	177.72	710.89	1,421.78
	593	0	0	622.03	3,465.60	4,087.63
	594	1,244.06	799.75	2,310.40	1,510.65	5,864.86
	595	533.17	0	533.17	0	1,066.34
	596	1,688.37	177.72	355.45	2,221.54	4,443.08
	597	1,421.78	3,643.32	3,554.46	8,441.84	17,061.40
	598	3,821.04	2,315.87	5,509.41	5,598.27	17,244.59
	599	977.48	1,066.34	5,688.96	977.48	8,710.26
TOTAL		12,442.80	10,492.03	21,509.96	29,680.65	74,125.44

STATE: NEBRASKA

ZIP CODE		QUARTER 1	QUARTER 2	QUARTER 3	QUARTER 4	TOTAL GRAMS
	680	1,066.34	1,777.23	2,310.40	4,265.35	9,419.32
	681	2,306.30	2,807.11	4,499.58	6,284.01	15,897
	683	1,422.70	799.75	1,692.01	1,421.78	5,336.24
	684	0	799.75	1,155.20	1,066.34	3,021.29

	685	3,643.32	3,110.15	4,000.59	4,620.80	15,374.86
	686	266.58	266.58	444.31	888.62	1,866.09
	687	0	1,332.92	2,488.12	1,421.78	5,242.82
	688	1,599.51	2,576.98	1,599.51	2,046.55	7,822.55
	689	533.17	710.89	799.75	2,843.57	4,887.38
	690	0	0	0	533.17	533.17
	691	801.12	2,310.40	1,245.43	2,311.77	6,668.72
	693	888.62	2,576.98	267.04	2,665.85	6,398.49
TOTAL		12,527.66	19,068.74	20,501.94	30,369.59	82,467.93

STATE: NEVADA

ZIP CODE		QUARTER 1	QUARTER 2	QUARTER 3	QUARTER 4	TOTAL GRAMS
	890	1,778.14	977.93	1,422.70	1,599.51	5,778.28
	891	3,355.41	6,113.67	9,279.87	9,916.12	28,665.07
	893	266.58	0	0	0	266.58
	894	4,354.67	2,932.43	4,443.08	4,443.08	16,173.26
	895	959.25	911.86	2,688.63	2,400.63	6,960.37
	897	710.89	622.03	180	799.75	2,312.67
	898	799.75	0	266.58	533.17	1,599.50
TOTAL		12,224.69	11,557.92	18,280.86	19,692.26	61,755.73

DRUG CODE:2270DRUG NAME:PENTOBARBITAL (SCHEDULE 2)

STATE: NEW HAMPSHIRE

ZIP CODE		QUARTER 1	QUARTER 2	QUARTER 3	QUARTER 4	TOTAL GRAMS
	030	2,310.40	5,239.53	4,264.25	4,620.42	16,434.60
	031	18.23	177.72	88.86	355.45	640.26
	032	2,665.85	4,976.24	5,418.90	5,242.83	18,303.82
	033	975.83	177.72	266.58	1,064.69	2,484.82
	034	1,421.78	799.75	1,599.51	2,665.85	6,486.89
	035	0	88.86	177.72	0	266.58
	036	88.86	266.58	0	355.45	710.89
	037	1,546.65	2,343.56	2,488.12	710.89	7,089.22
	038	2,843.57	2,576.98	2,488.12	1,599.51	9,508.18
TOTAL		11,871.17	16,646.94	16,792.06	16,615.09	61,925.26

STATE: NEW JERSEY

ZIP CODE	QUARTER 1	QUARTER 2	QUARTER 3	QUARTER 4	TOTAL GRAMS
070	1,332.92	1,599.51	1,332.92	1,906.55	6,171.90
071	86.58	9.11	2,349.59	43.29	2,488.57
072	0	0	0.91	0	0.91
074	532.62	1,065.79	1,067.06	1,157.02	3,822.49
075	0	0	122.13	0	122.13
076	1,954.95	1,911.66	1,877.48	2,087.56	7,831.65
077	1,421.78	799.75	2,756.53	3,465.60	8,443.66
078	4,176.49	1,599.51	6,220.31	1,599.51	13,595.82
079	1,102.79	532.89	977.48	199.6	2,812.76
080	2,323.61	1,784.52	3,472.89	4,370.62	11,951.64
081	0	0	0	9.11	9.11
082	0	977.48	266.58	799.48	2,043.54
083	1,332.92	1,866.09	1,954.95	1,244.06	6,398.02
085	2,749.76	1,064.69	3,016.07	2,752.51	9,583.03
086	27.34	0	177.72	88.86	293.92
087	177.72	533.17	444.31	266.58	1,421.78
088	1,166.20	1,512.01	266.58	1,010.83	3,955.62
089	444.31	458.89	23.7	44.66	971.56
TOTAL	18,829.99	15,715.07	26,327.21	21,045.84	81,918.11

STATE: NEW MEXICO

ZIP CODE	QUARTER 1	QUARTER 2	QUARTER 3	QUARTER 4	TOTAL GRAMS
870	3,910.82	4,266.26	2,844.48	4,799.43	15,820.99
871	2,064.32	2,400.17	2,665.85	5,513.42	12,643.76
874	1,782.70	2,221.54	3,026.76	3,826.51	10,857.51
875	1,332.92	3,110.15	6,677.37	977.48	12,097.92
877	799.75	0	622.03	88.86	1,510.64
878	0	0	177.72	355.45	533.17
879	354.35	0	177.17	177.17	708.69
880	2,310.40	5,143.88	5,335.06	3,294.26	16,083.60
881	355.45	533.17	888.62	0	1,777.24
882	710.89	267.95	3,469.70	2,399.26	6,847.80

	883	266.58	177.72	622.03	355.45	1,421.78
TOTAL		13,888.18	18,120.84	26,506.79	21,787.29	80,303.10

STATE: NEW YORK

ZIP CODE		QUARTER 1	QUARTER 2	QUARTER 3	QUARTER 4	TOTAL GRAMS
	100	91.14	2,148.17	682.64	477.12	3,399.07
	101	0	88.86	177.72	0	266.58
	103	0	0	355.45	355.45	710.9
	104	0	93.14	191.39	19.14	303.67
	105	975.2	1,778.14	982.03	1,599.51	5,334.88
	109	976.1	977.48	1,065.24	444.31	3,463.13
	110	0	0	88.86	407.12	495.98
	112	23.7	2.73	1,070.90	10.94	1,108.27
	113	2.28	8.2	0	5.47	15.95
	114	0	0	533.17	0	533.17
	115	799.75	442.93	444.31	799.75	2,486.74
	117	710.89	1,242.96	3,483.83	1,619.37	7,057.05
	118	0	0	88.59	0	88.59
	119	444.31	533.17	1,066.34	711.8	2,755.62
	120	444.31	1,420.13	1,065.79	1,331.55	4,261.78
	121	5,152.32	5,065.11	5,420.55	7,107.27	22,745.25
	122	1,197.47	88.86	88.86	444.31	1,819.50
	123	88.86	200.51	88.86	177.72	555.95
	124	0	1,688.37	0	533.17	2,221.54

DRUG CODE:2270DRUG NAME:PENTOBARBITAL (SCHEDULE 2)
STATE:NEW YORK

ZIP CODE		QUARTER 1	QUARTER 2	QUARTER 3	QUARTER 4	TOTAL GRAMS
	125	7,552.49	5,772.70	9,062.22	7,015.11	29,402.52
	126	622.03	266.58	533.17	379.14	1,800.92
	127	0	0	0	88.86	88.86
	128	1,777.23	2,664.20	4,443.08	3,730.53	12,615.04
	129	1,599.51	1,688.37	1,599.51	3,732.18	8,619.57
	130	3,549.79	4,615.30	5,149.02	5,503.64	18,817.75

	131	266.58	1,332.92	977.48	4,443.08	7,020.06
	132	747.35	736.58	1,082.46	1,273.68	3,840.07
	133	1,777.23	3,287.88	888.62	1,777.23	7,730.96
	134	2,310.40	3,373.44	1,510.65	2,043.81	9,238.30
	136	0	265.76	1,778.31	977.48	3,021.55
	137	442.93	708.69	531.52	531.52	2,214.66
	138	1,509.55	1,951.65	2,396.24	3,195.17	9,052.61
	139	0	177.72	177.72	88.86	444.3
	140	4,265.35	4,972.67	5,152.59	8,086.40	22,477.01
	141	4,627.63	3,287.88	3,110.15	3,465.32	14,490.98
	142	2,485.92	3,838.71	5,901.21	6,710.36	18,936.20
	143	177.17	266.31	266.31	1,154.92	1,864.71
	144	2,575.88	1,686.44	5,507.76	5,516.23	15,286.31
	145	4,876.94	11,444.44	10,829.83	5,232.38	32,383.59
	146	263.58	400.74	845.96	337.67	1,847.95
	147	1,155.20	975.83	1,597.86	977.48	4,706.37
	148	2,664.75	7,194.76	3,639.75	7,372.21	20,871.47
TOTAL		56,153.84	76,688.33	83,875.95	89,677.26	306,395.38

STATE: NORTH CAROLINA

ZIP CODE		QUARTER 1	QUARTER 2	QUARTER 3	QUARTER 4	TOTAL GRAMS
	270	1,954.95	2,488.12	2,219.89	1,774.48	8,437.44
	271	5,523.08	1,646.44	1,442.29	1,433.18	10,044.99
	272	1,332.92	2,843.57	3,913.10	3,998.77	12,088.36
	273	1,421.78	2,043.54	4,975.97	3,288.51	11,729.80
	274	1,884.32	1,442.29	2,148.17	2,155.01	7,629.79
	275	4,351	3,284.03	7,328.17	4,047.87	19,011.07
	276	97.98	4,798.52	533.17	5,509.41	10,939.08
	277	480.76	2,645.34	534.08	3,346.21	7,006.39
	278	2,266.28	2,780.95	2,689.54	3,448.82	11,185.59
	279	177.72	799.75	1,777.23	710.89	3,465.59
	280	2,220.99	1,687.82	2,843.02	5,686.95	12,438.78
	281	7,010.16	9,232.25	4,882.98	9,500.48	30,625.87
	282	3,643.32	1,089.12	3,839.27	3,009.90	11,581.61
	283	8,264.12	6,309.17	5,862.11	9,686.81	30,122.21
	284	1,102.79	635.43	1,626.85	1,525.59	4,890.66

	285	2,129.38	1,866.09	1,155.20	3,462.30	8,612.97
	286	2,221.54	5,776	6,398.03	2,843.57	17,239.14
	287	3,110.15	1,954.95	3,821.04	6,931.20	15,817.34
	288	1,929.89	2,132.68	2,576.98	2,317.23	8,956.78
	289	88.86	177.72	177.72	266.58	710.88
TOTAL		51,211.99	55,633.78	60,744.81	74,943.76	242,534.34

STATE:	NORTH DAKOTA					
ZIP CODE		QUARTER 1	QUARTER 2	QUARTER 3	QUARTER 4	TOTAL GRAMS
	580	1,244.06	2,310.40	799.75	2,576.98	6,931.19
	581	906.84	2,232.47	862.64	1,954.95	5,956.90
	582	2,665.85	888.62	177.72	355.45	4,087.64
	584	2,132.68	533.17	2,132.68	1,066.34	5,864.87
	585	533.17	0	284.81	533.17	1,351.15
	586	0	0	0	888.62	888.62
	587	1,244.06	533.17	533.17	977.48	3,287.88
TOTAL		8,726.66	6,497.83	4,790.77	8,352.99	28,368.25

STATE:	OHIO					
	430	3,462.84	3,467.51	3,643.86	6,398.37	16,972.58
	431	797.55	533.17	1,331.27	1,952.75	4,614.74
	432	791.27	4,148.50	2,836.45	908.84	8,685.06
	433	355.45	4,999.03	4,465.86	4,821.31	14,641.65
	434	177.17	977.48	443.76	354.9	1,953.31
	435	2,405.64	3,733.55	2,851.77	2,403.82	11,394.78
	436	1,691.10	669.88	1,717.99	1,066.34	5,145.31
	437	3,643.32	1,688.37	3,643.32	3,554.46	12,529.47
	438	0	1,599.51	888.62	1,066.34	3,554.47
	439	444.31	710.89	1,155.20	177.72	2,488.12
	440	3,999.86	5,508.86	3,998.22	10,928.59	24,435.53
	441	6,905.86	8,366.10	3,098.30	6,475.95	24,846.21
	442	6,134.44	1,954.95	5,153.97	6,139.65	19,383.01
	443	710.89	2,132.68	5,360.40	1,510.65	9,714.62
	444	444.31	799.75	4,887.38	4,709.66	10,841.10
	445	800.39	1,333.56	2,122.38	888.07	5,144.40

	446	2,754.71	1,516.11	5,690.68	4,626.27	14,587.77
	447	3,733.27	975.83	1,775.58	1,420.68	7,905.36
	448	3,465.60	2,399.26	5,419.45	4,265.35	15,549.66
	449	0	532.07	9.11	444.31	985.49
	450	5,868.50	1,958.60	3,552.81	9,333.37	20,713.28
	451	622.03	2,132.68	1,688.09	3,109.88	7,552.68
	452	1,934.45	1,054.95	924.16	2,103.51	6,017.07
	453	3,819.40	4,263.98	6,307.24	2,665.85	17,056.47
	454	970.64	2,866.35	1,594.95	5,621.06	11,053
	455	710.89	799.75	1,510.65	355.45	3,376.74
	456	1,066.34	977.48	444.31	533.17	3,021.30
	457	1,155.20	1,777.23	1,155.20	444.31	4,531.94
	458	979.3	1,866.09	555.95	3,025.39	6,426.73
TOTAL		59,844.73	65,744.17	78,226.93	91,306.02	295,121.85

STATE:	OKLAHOMA					
ZIP CODE		QUARTER 1	QUARTER 2	QUARTER 3	QUARTER 4	TOTAL GRAMS
	730	4,176.49	2,844.48	4,093.10	7,551.58	18,665.65
	731	2,426.60	5,780.55	2,762	9,824.89	20,794.04
	734	1,332.92	1,777.23	0	1,421.78	4,531.93
	735	533.17	177.72	0	0	710.89
	736	0	355.45	0	0	355.45
	740	1,599.51	1,599.51	1,688.37	799.75	5,687.14
	741	2,371.92	7,559.78	3,514.81	5,795.14	19,241.65
	743	177.17	0	177.17	88.86	443.2
	744	533.17	0	977.48	533.17	2,043.82
	745	88.59	1,063.04	265.76	0	1,417.39
	748	444.31	355.45	622.03	799.75	2,221.54
TOTAL		13,683.85	21,513.21	14,100.72	26,814.92	76,112.70

STATE:	OREGON					
ZIP CODE		QUARTER 1	QUARTER 2	QUARTER 3	QUARTER 4	TOTAL GRAMS
	970	5,864.86	7,907.02	5,953.72	10,041.35	29,766.95
	971	4,709.66	533.17	4,087.63	1,688.37	11,018.83
	972	2,310.40	2,533.69	4,799.43	4,187.43	13,830.95

	973	4,976.24	5,153.97	7,464.37	5,864.86	23,459.44
	974	4,531.94	7,464.37	5,864.86	6,753.47	24,614.64
	975	2,132.68	4,797.97	4,531.94	5,331.42	16,794.01
	976	888.62	710.89	2,843.57	1,421.78	5,864.86
	977	2,137.23	3,021.29	6,758.94	3,201.75	15,119.21
	978	1,066.34	2,576.98	1,332.92	6,931.20	11,907.44
	979	355.45	622.03	355.45	977.48	2,310.41
TOTAL		28,973.42	35,321.38	43,992.83	46,399.11	154,686.74

STATE: PENNSYLVANIA

ZIP CODE		QUARTER 1	QUARTER 2	QUARTER 3	QUARTER 4	TOTAL GRAMS
	150	1,421.78	2,488.12	266.58	2,754.71	6,931.19
	151	2,396.24	1,537.99	2,753.33	1,599.51	8,287.07
	152	623.85	4,137.30	4,625.17	3,462.85	12,849.17
	153	1,332.92	0	5,590.03	0	6,922.95
	154	0	88.86	1,687.27	177.72	1,953.85
	155	355.45	710.89	710.89	444.31	2,221.54
	156	3,554.19	1,066.34	1,599.51	4,265.08	10,485.12
	159	288.54	446.59	43.29	378.23	1,156.65
	160	266.58	0	1,597.86	533.17	2,397.61
	161	1,066.34	2,132.68	0	1,688.37	4,887.39
	162	0	0	266.58	177.72	444.3
	163	1,244.06	1,066.34	2,132.68	533.62	4,976.70
	164	2,043.81	1,954.95	2,399.26	1,244.06	7,642.08
	165	2,111.60	2,932.43	290.28	1,622.10	6,956.41
	166	533.17	1,155.20	710.89	0	2,399.26
	168	2,932.43	3,110.15	710.89	1,244.06	7,997.53
	169	355.45	1,421.78	2,043.81	533.17	4,354.21
	170	3,109.60	2,842.74	4,620.25	4,043.51	14,616.10
	171	798.65	977.48	533.17	821.44	3,130.74
	172	2,398.16	2,132.68	3,020.74	2,043.54	9,595.12
	173	888.62	177.72	710.89	888.62	2,665.85
	174	3,155.72	2,064.32	988.87	2,399.26	8,608.17
	175	1,066.34	1,244.06	3,554.46	799.75	6,664.61
	176	1,827.36	1,245.88	0	894.08	3,967.32
	177	533.17	622.03	1,599.51	1,599.51	4,354.22

	178	2,157.74	2,932.43	3,998.77	1,954.95	11,043.89
	179	266.58	177.72	88.86	266.58	799.74
	180	888.62	2,498.15	2,310.40	3,749.67	9,446.84
	181	1,182.54	690.39	36.46	710.89	2,620.28
	182	710.89	0	888.62	355.45	1,954.96
	183	710.89	977.48	1,777.23	444.31	3,909.91
	184	622.03	1,599.51	1,954.95	1,599.51	5,776
	185	0	0	888.62	355.45	1,244.07
	186	0	177.72	888.62	1,244.06	2,310.40
	187	266.58	0	5,598.27	634.33	6,499.18
	188	88.86	88.86	266.58	0.91	445.21
	189	2,842.47	5,153.97	5,151.22	6,753.47	19,901.13
	190	977.48	1,797.74	1,970.81	1,538.90	6,284.93
	191	487.6	1,328.35	746.44	761.93	3,324.32
	193	3,909.63	2,222.45	3,998.77	7,553.23	17,684.08
	194	822.99	5,244.65	8,372.58	1,260.92	15,701.14
	195	1,332.92	355.45	2,754.71	1,155.20	5,598.28
	196	1,952.75	2,399.89	3,559.65	269.04	8,181.33
TOTAL		53,524.60	63,201.29	87,707.77	64,757.19	269,190.85

STATE:	PUERTO RICO				
ZIP CODE	QUARTER 1	QUARTER 2	QUARTER 3	QUARTER 4	TOTAL GRAMS
009	0	0	0.2	0	0.2
TOTAL	0	0	0.2	0	0.2

STATE:	RHODE ISLAND				
ZIP CODE	QUARTER 1	QUARTER 2	QUARTER 3	QUARTER 4	TOTAL GRAMS
028	3,021.29	5,953.72	7,108.65	6,575.75	22,659.41
029	186.38	992.15	181.82	542.64	1,902.99
TOTAL	3,207.67	6,945.87	7,290.47	7,118.39	24,562.40

STATE:	SOUTH CAROLINA				
ZIP CODE	QUARTER 1	QUARTER 2	QUARTER 3	QUARTER 4	TOTAL GRAMS
290	3,014.69	799.75	2,132.40	1,688.37	7,635.21

	291	88.86	1,066.34	1,244.06	2,043.81	4,443.07
	292	1,191.66	3,235.47	1,266.39	3,502.05	9,195.57
	293	2,665.85	2,136.32	1,777.23	1,510.65	8,090.05
	294	1,510.65	1,332.92	2,221.54	2,537.34	7,602.45
	295	2,155.46	1,066.34	1,547.10	1,954.95	6,723.85
	296	885.87	995.7	355.45	1,256.82	3,493.84
	297	0	355.45	355.45	177.72	888.62
	298	533.17	1,599.51	3,021.29	1,155.20	6,309.17
	299	533.17	266.58	0	533.17	1,332.92
TOTAL		12,579.38	12,854.38	13,920.91	16,360.08	55,714.75

STATE:	SOUTH DAKOTA				
ZIP CODE	QUARTER 1	QUARTER 2	QUARTER 3	QUARTER 4	TOTAL GRAMS
570	533.17	1,599.51	1,777.23	1,777.23	5,687.14
571	711.35	1,244.97	533.17	1,600.42	4,089.91
573	0	0	0	177.72	177.72
575	533.17	0	0	1,066.34	1,599.51
577	4,003.32	2,581.54	4,365.61	2,488.12	13,438.59
TOTAL	5,781.01	5,426.02	6,676.01	7,109.83	24,992.87

STATE:	TENNESSEE				
ZIP CODE	QUARTER 1	QUARTER 2	QUARTER 3	QUARTER 4	TOTAL GRAMS
370	3,550.06	3,550.34	4,262.05	5,145.17	16,507.62
371	2,485.92	3,992.72	3,107.40	2,487.85	12,073.89
372	269.32	367.29	444.31	2,952.94	4,033.86
373	2,665.02	2,575.61	4,441.43	6,662.96	16,345.02
374	266.58	2,433.44	897.73	631.14	4,228.89
376	3,474.24	5,193.70	4,797.97	2,494.68	15,960.59
377	1,955.59	3,642.58	2,580.80	1,687.54	9,866.51
378	3,198.54	2,668.56	3,110.23	4,714.82	13,692.15
379	3,283.48	6,061.24	4,631.34	1,950.28	15,926.34
380	1,953.58	1,066.34	799.75	1,688.37	5,508.04
381	779.7	5,611.67	6.84	2,065.04	8,463.25
382	355.45	266.58	1,599.51	1,155.20	3,376.74
383	1,598.96	531.52	1,510.37	1,510.10	5,150.95

	384	801.58	2,310.12	88.86	2,756.25	5,956.81
	385	0	1,066.34	2,925.83	1,510.65	5,502.82
TOTAL		26,638.02	41,338.05	35,204.42	39,412.99	142,593.48

STATE: TEXAS

ZIP CODE	QUARTER 1	QUARTER 2	QUARTER 3	QUARTER 4	TOTAL GRAMS
750	5,466.12	6,058.08	9,508.35	14,851.07	35,883.62
751	2,843.57	4,620.80	2,929.13	4,620.80	15,014.30
752	3,866.61	1,637.33	2,135.41	4,070.04	11,709.39
753	0	0	32.81	106.63	139.44
754	2,399.26	1,689.28	2,042.17	3,021.65	9,152.36
755	444.31	355.45	355.45	888.62	2,043.83
756	2,049.74	1,420.68	2,486.47	4,537.40	10,494.29
757	3,019.72	2,798.63	1,662.12	2,501.52	9,981.99
758	2,857.24	533.17	0	1,421.78	4,812.19
759	2,308.20	5,059.61	3,017.99	1,064.96	11,450.76
760	3,021.29	3,998.77	9,508.18	7,730.95	24,259.19
761	14,221.49	9,158.20	1,055.86	11,713.77	36,149.32
762	2,401.54	1,777.23	4,443.99	3,379.47	12,002.23
763	888.62	4,443.08	977.48	2,043.81	8,352.99
764	177.72	177.72	1,599.51	1,244.06	3,199.01
765	1,041.27	2,390.60	3,126.56	1,335.47	7,893.90
766	88.86	177.72	177.72	177.72	622.02
767	2,754.71	266.58	1,331.82	799.75	5,152.86
768	888.62	177.72	0	710.89	1,777.23
769	0	0	2,321.34	0	2,321.34
770	1,229.02	20,176.12	19,353.58	4,133.65	44,892.37
773	3,021.75	1,264.47	4,483.99	4,458.11	13,228.32
774	2,310.40	710.89	2,932.43	977.48	6,931.20
775	1,066.34	3,475.06	7,287.10	6,875.49	18,703.99
776	354.35	444.31	2,040.79	620.66	3,460.11
777	2,043.81	0	0	1,598.68	3,642.49
778	2,222.07	3,727.78	2,841.90	3,999.30	12,791.05
779	177.72	444.31	622.03	622.03	1,866.09
780	4,887.38	88.86	1,155.20	4,442.53	10,573.97
781	888.62	622.03	2,043.81	3,376.74	6,931.20

	782	1,723	398.74	3,021.02	2,780.23	7,922.99
	783	1,332.92	533.17	2,576.98	622.03	5,065.10
	784	544.11	0	0	11.85	555.96
	785	9,685.90	9,512.74	11,023.38	10,060.21	40,282.23
	786	8,708.43	3,021.29	3,821.04	7,997.54	23,548.30
	787	266.58	1,818.24	1,421.78	2,518.20	6,024.80
	788	1,866.09	0	710.89	1,599.51	4,176.49
	789	0	1,244.06	0	0	1,244.06
	790	1,066.34	3,554.46	1,777.23	2,932.43	9,330.46
	791	988.87	626.59	9,961.60	1,866.09	13,443.15
	793	710.89	1,157.02	1,954.95	266.58	4,089.44
	794	2,365.08	3,224.08	2,319.97	3,583.62	11,492.75
	795	355.45	355.45	533.17	531.52	1,775.59
	796	2,488.12	1,954.40	1,954.95	1,066.34	7,463.81
	797	3,820.22	2,221.54	6,432.83	5,248.01	17,722.60
	798	0	0	533.17	0	533.17
	799	551.4	640.26	2,861.80	2,435.72	6,489.18
TOTAL		105,413.75	107,956.52	142,377.95	140,844.91	496,593.13

STATE: UTAH

ZIP CODE		QUARTER 1	QUARTER 2	QUARTER 3	QUARTER 4	TOTAL GRAMS
	840	622.03	3,381.29	4,880.78	3,199.01	12,083.11
	841	1,736.22	1,491.96	4,158.26	5,987.90	13,374.34
	843	0	0	355.45	88.86	444.31
	844	1,777.23	444.31	444.31	1,066.34	3,732.19
	845	1,244.06	533.17	177.72	3,287.88	5,242.83
	846	2,399.26	3,465.60	1,066.34	3,732.18	10,663.38
	847	1,866.09	1,955.41	2,221.54	5,331.69	11,374.73
TOTAL		9,644.89	11,271.74	13,304.40	22,693.86	56,914.89

STATE: VERMONT

ZIP CODE		QUARTER 1	QUARTER 2	QUARTER 3	QUARTER 4	TOTAL GRAMS
	050	1,421.78	977.2	2,665.85	710.62	5,775.45
	051	355.45	532.07	0	444.31	1,331.83
	052	533.17	622.03	1,688.37	799.75	3,643.32

	053	88.86	355.45	88.86	355.45	888.62
	054	2,064.68	4,389.65	7,749.11	6,929.55	21,132.99
	056	3,199.01	2,043.81	1,066.34	5,509.41	11,818.57
	057	2,043.81	444.31	2,399.26	3,021.29	7,908.67
	058	88.86	0	1,773.93	0	1,862.79
TOTAL		9,795.62	9,364.52	17,431.72	17,770.38	54,362.24

STATE: VIRGINIA

ZIP CODE	QUARTER 1	QUARTER 2	QUARTER 3	QUARTER 4	TOTAL GRAMS
201	3,198.74	1,243.79	2,044.73	5,776	12,263.26
220	3,732.18	2,665.85	2,399.26	3,305.19	12,102.48
221	891.35	799.75	1,599.51	622.03	3,912.64
222	0	0	88.86	0	88.86
223	446.59	893.17	444.31	977.48	2,761.55
224	88.86	976.1	1,155.20	976.1	3,196.26
225	1,242.41	977.48	1,686.72	1,777.23	5,683.84
226	1,155.20	2,223.36	1,874.29	3,379.47	8,632.32
227	620.38	0	2,042.17	0	2,662.55
228	1,510.65	710.89	2,665.85	1,066.34	5,953.73
229	5,513.06	6,053.97	6,073.57	4,537.40	22,178
230	2,132.68	3,287.88	3,465.32	2,843.57	11,729.45
231	622.03	888.62	533.17	1,243.24	3,287.06
232	196.41	332.21	90.68	983.86	1,603.16
233	710.89	710.89	1,244.06	1,155.20	3,821.04
234	8,620.43	5,363.37	5,065.11	4,272.19	23,321.10
235	2,132.68	1,157.93	2,138.14	21.87	5,450.62
236	1,066.34	1,954.95	2,754.71	2,843.57	8,619.57
237	266.58	0	271.14	4.56	542.28
238	1,688.37	3,732.18	1,777.23	1,066.34	8,264.12
239	0	444.31	977.48	1,155.20	2,576.99
240	0	3,652.16	2,852.68	3,310.39	9,815.23
241	710.89	799.75	533.17	533.17	2,576.98
242	0	0	0	2,665.85	2,665.85
243	2,132.68	888.62	355.45	2,308.20	5,684.95
244	1,155.20	0	888.62	2,310.40	4,354.22
245	3,021.29	6,844.16	1,067.25	3,998.77	14,931.47

	246	0	622.03	1,066.34	622.03	2,310.40
TOTAL		42,855.89	47,223.42	47,155.02	53,755.65	190,989.98

STATE:	WASHINGTON					
ZIP CODE		QUARTER 1	QUARTER 2	QUARTER 3	QUARTER 4	TOTAL GRAMS
	980	4,265.35	5,949.60	6,309.17	4,620.80	21,144.92
	981	1,344.32	713.17	2,271.66	1,472.82	5,801.97
	982	14,132.17	11,467.69	11,114.07	19,343.55	56,057.48
	983	4,265.35	3,909.91	2,309.57	12,351.75	22,836.58
	984	1,927.61	2,932.43	2,316.78	1,688.37	8,865.19
	985	4,887.38	8,797.29	4,709.66	8,264.12	26,658.45
	986	7,380.85	4,709.66	8,877.90	6,839.04	27,807.45
	988	1,332.92	2,579.72	1,421.78	2,044.27	7,378.69
	989	3,554.46	2,132.68	2,402.91	3,021.29	11,111.34
	990	7,819.81	2,310.40	6,309.17	8,441.84	24,881.22
	991	0	2,046.55	1,066.34	1,866.09	4,978.98
	992	915.96	4,907.89	3,110.15	2,892.33	11,826.33
	993	3,554.46	888.62	4,443.08	1,074.54	9,960.70
	994	177.72	266.58	355.45	710.89	1,510.64
TOTAL		55,558.36	53,612.19	57,017.69	74,631.70	240,819.94

STATE:	WEST VIRGINIA					
ZIP CODE		QUARTER 1	QUARTER 2	QUARTER 3	QUARTER 4	TOTAL GRAMS
	247	0	1,066.34	0	0	1,066.34
	250	533.17	88.86	266.58	266.58	1,155.19
	251	0	266.58	0	0	266.58
	252	88.86	355.45	444.31	177.72	1,066.34
	253	266.58	2,665.30	922.79	1,599.51	5,454.18
	254	1,332.92	2,576.98	1,332.92	533.17	5,775.99
	255	355.45	710.89	1,066.34	710.89	2,843.57
	256	0	355.45	0	0	355.45
	257	929.35	0	1,787.26	24.15	2,740.76
	258	0	710.89	0	0	710.89
	259	177.72	355.45	888.62	266.58	1,688.37
	260	1,066.34	622.03	3,199.01	1,954.95	6,842.33

	261	266.58	977.48	1,332.92	533.17	3,110.15
	262	177.72	266.58	0	177.72	622.02
	263	0	88.86	266.58	0	355.44
	264	0	444.31	0	88.59	532.9
	265	532.07	666.23	355.45	1,609.80	3,163.55
	266	1,066.34	0	885.87	0	1,952.21
	267	0	355.45	1,244.06	355.45	1,954.96
	268	177.72	0	266.58	0	444.3
TOTAL		6,970.82	12,573.13	14,259.29	8,298.28	42,101.52

STATE: WISCONSIN

ZIP CODE		QUARTER 1	QUARTER 2	QUARTER 3	QUARTER 4	TOTAL GRAMS
	530	2,129.93	3,463.38	3,997.12	5,943.82	15,534.25
	531	6,581.22	6,756.21	5,157.51	11,200.56	29,695.50
	532	2,141.79	6,777.05	4,846.37	5,718.67	19,483.88
	534	0	1,155.20	266.58	888.62	2,310.40
	535	7,374.40	8,620.93	6,309.17	14,838.22	37,142.72
	537	4,268.09	891.8	5,116.14	6,142.84	16,418.87
	538	1,063.04	88.86	1,329.62	444.31	2,925.83
	539	710.89	888.62	1,335.66	3,731.63	6,666.80
	540	177.72	1,421.78	1,866.09	1,155.20	4,620.79
	541	1,599.23	533.17	889.07	266.31	3,287.78
	542	531.52	1,332.10	1,420.13	443.21	3,726.96
	543	1,159.57	710.89	1,257.54	355.45	3,483.45
	544	1,775.58	1,330.72	3,199.72	4,572.92	10,878.94
	545	355.45	710.89	1,422.24	1,866.09	4,354.67
	546	710.89	799.75	1,164.31	733.68	3,408.63
	547	2,308.20	3,104.10	2,930.23	3,198.46	11,540.99
	548	2,488.12	1,066.34	3,909.91	2,843.57	10,307.94
	549	5,595.53	3,909.34	5,702.61	8,424.61	23,632.09
TOTAL		40,971.17	43,561.13	52,120.02	72,768.17	209,420.49

STATE: WYOMING

ZIP CODE		QUARTER 1	QUARTER 2	QUARTER 3	QUARTER 4	TOTAL GRAMS
	820	1,066.34	2,043.81	2,156.37	6,131.44	11,397.96

	822	0	0	444.31	0	444.31
	823	0	0	1,066.34	0	1,066.34
	824	177.72	5,065.11	266.58	1,599.51	7,108.92
	826	1,066.34	2,404.73	9.11	2,843.57	6,323.75
	827	710.89	799.75	1,155.20	355.45	3,021.29
	828	1,510.65	1,066.34	2,754.71	1,688.37	7,020.07
	829	0	710.89	1,244.06	1,421.78	3,376.73
	830	0	0	0	177.72	177.72
	831	0	533.17	0	0	533.17
TOTAL		4,531.94	12,623.80	9,096.68	14,217.84	40,470.26

DRUG CODE: 2315 DRUG NAME: SECOBARBITAL (SCHEDULE 2)

STATE: ARIZONA

ZIP CODE		QUARTER 1	QUARTER 2	QUARTER 3	QUARTER 4	TOTAL GRAMS
	852	0	0	0	9.16	9.16
	853	9.16	0	0	0	9.16
TOTAL		9.16	0	0	9.16	18.32

STATE: CALIFORNIA

ZIP CODE		QUARTER 1	QUARTER 2	QUARTER 3	QUARTER 4	TOTAL GRAMS
	900	18.31	9.16	9.16	18.31	54.94
	902	0	9.16	9.16	0	18.32
	906	27.47	18.31	27.47	18.31	91.56
	910	27.47	9.16	9.16	27.47	73.26
	912	0	0	9.16	9.16	18.32
	913	9.16	0	9.16	0	18.32
	914	9.16	9.16	9.16	0	27.48
	920	119.02	73.24	201.41	265.5	659.17
	921	9.16	0	9.16	18.31	36.63
	926	0	36.62	18.31	18.31	73.24
	928	137.33	164.79	146.48	210.57	659.17
	930	9.16	0	18.31	18.31	45.78
	931	0	0	18.31	18.31	36.62
	934	0	9.16	0	0	9.16

	935	0	9.16	0	0	9.16
	939	0	9.16	9.16	9.16	27.48
	941	421.13	311.27	347.89	320.43	1,400.72
	943	36.62	9.16	9.16	27.47	82.41
	944	9.16	45.78	45.78	54.93	155.65
	945	9.16	9.16	9.16	0	27.48
	949	18.31	0	9.16	0	27.47
	950	0	18.31	9.16	9.16	36.63
	956	9.16	183.1	173.95	283.81	650.02
	958	27.47	45.78	45.78	0	119.03
	961	0	9.16	9.16	0	18.32
TOTAL		897.25	988.8	1,162.77	1,327.52	4,376.34

STATE: COLORADO

ZIP CODE		QUARTER 1	QUARTER 2	QUARTER 3	QUARTER 4	TOTAL GRAMS
	800	45.78	18.31	64.09	27.47	155.65
	801	9.16	18.31	0	9.16	36.63
	802	0	0	18.31	0	18.31
	803	0	9.16	0	9.16	18.32
	804	0	0	0	9.16	9.16
	805	9.16	0	0	0	9.16
	809	36.62	36.62	9.16	0	82.4
	810	9.16	0	0	0	9.16
	812	0	9.16	0	0	9.16
	814	0	0	0	9.16	9.16
	815	0	0	9.16	0	9.16
	816	0	0	9.16	0	9.16
TOTAL		109.88	91.56	109.88	64.11	375.43

STATE: CONNECTICUT

ZIP CODE		QUARTER 1	QUARTER 2	QUARTER 3	QUARTER 4	TOTAL GRAMS
	63	9.16	0	0	0	9.16
	64	9.16	9.16	9.16	9.16	36.64
	65	0	0	0	9.16	9.16
	67	9.16	18.31	0	0	27.47

	68	9.16	0	0	0	9.16
TOTAL		36.64	27.47	9.16	18.32	91.59

STATE:	DELAWARE				
ZIP CODE	QUARTER 1	QUARTER 2	QUARTER 3	QUARTER 4	TOTAL GRAMS
197	0	9.16	9.16	9.16	27.48
TOTAL	0	9.16	9.16	9.16	27.48

STATE:	FLORIDA				
ZIP CODE	QUARTER 1	QUARTER 2	QUARTER 3	QUARTER 4	TOTAL GRAMS
330	45.78	9.16	18.31	9.16	82.41
331	18.31	18.31	9.16	18.31	64.09
333	0	0	0	9.16	9.16
334	27.47	36.62	54.93	36.62	155.64
337	0	9.16	9.16	9.16	27.48
341	9.16	0	0	0	9.16
342	0	0	0	9.16	9.16
349	0	0	9.16	0	9.16
TOTAL	100.72	73.25	100.72	91.57	366.26

STATE:	GEORGIA				
ZIP CODE	QUARTER 1	QUARTER 2	QUARTER 3	QUARTER 4	TOTAL GRAMS
300	9.16	0	0	0	9.16
302	27.47	9.16	0	0	36.63
TOTAL	36.63	9.16	0	0	45.79

STATE:	HAWAII				
ZIP CODE	QUARTER 1	QUARTER 2	QUARTER 3	QUARTER 4	TOTAL GRAMS
968	9.16	0	0	0	9.16
TOTAL	9.16	0	0	0	9.16

STATE: ILLINOIS

ZIP CODE	QUARTER 1	QUARTER 2	QUARTER 3	QUARTER 4	TOTAL GRAMS
600	27.47	36.62	0	9.16	73.25
606	18.31	18.31	27.47	18.31	82.4
610	0	0	9.16	0	9.16
TOTAL	45.78	54.93	36.63	27.47	164.81

STATE: KANSAS

ZIP CODE	QUARTER 1	QUARTER 2	QUARTER 3	QUARTER 4	TOTAL GRAMS
662	0	0	0	9.16	9.16
667	27.47	18.31	18.31	18.31	82.4
672	9.16	0	0	0	9.16
TOTAL	36.63	18.31	18.31	27.47	100.72

STATE: MARYLAND

ZIP CODE	QUARTER 1	QUARTER 2	QUARTER 3	QUARTER 4	TOTAL GRAMS
208	0	9.16	0	0	9.16
209	18.31	18.31	18.31	18.31	73.24
210	0	9.16	0	0	9.16
217	45.78	27.47	27.47	27.47	128.19
TOTAL	64.09	64.1	45.78	45.78	219.75

STATE: MASSACHUSETTS

ZIP CODE	QUARTER 1	QUARTER 2	QUARTER 3	QUARTER 4	TOTAL GRAMS
10	9.16	9.16	0	0	18.32
16	18.31	9.16	18.31	18.31	64.09
17	0	18.31	9.16	9.16	36.63
18	9.16	0	0	0	9.16
24	18.31	9.16	9.16	9.16	45.79
26	0	0	0	18.31	18.31
TOTAL	54.94	45.79	36.63	54.94	192.3

STATE: MICHIGAN

ZIP CODE	QUARTER 1	QUARTER 2	QUARTER 3	QUARTER 4	TOTAL GRAMS

	480	0	0	0	18.31	18.31
TOTAL		0	0	0	18.31	18.31

STATE:	MINNESOTA					
ZIP CODE		QUARTER 1	QUARTER 2	QUARTER 3	QUARTER 4	TOTAL GRAMS
	554	9.16	0	0	9.16	18.32
TOTAL		9.16	0	0	9.16	18.32

STATE:	MISSOURI					
ZIP CODE		QUARTER 1	QUARTER 2	QUARTER 3	QUARTER 4	TOTAL GRAMS
	637	18.31	9.16	27.47	18.31	73.25
	654	9.16	9.16	9.16	0	27.48
TOTAL		27.47	18.32	36.63	18.31	100.73

STATE:	NEVADA					
ZIP CODE		QUARTER 1	QUARTER 2	QUARTER 3	QUARTER 4	TOTAL GRAMS
	891	9.16	0	0	0	9.16
TOTAL		9.16	0	0	0	9.16

STATE:	NEW HAMPSHIRE					
ZIP CODE		QUARTER 1	QUARTER 2	QUARTER 3	QUARTER 4	TOTAL GRAMS
	30	9.16	0	0	0	9.16
	33	9.16	0	0	0	9.16
	37	0	9.16	0	0	9.16
TOTAL		18.32	9.16	0	0	27.48

STATE:	NEW JERSEY					
ZIP CODE		QUARTER 1	QUARTER 2	QUARTER 3	QUARTER 4	TOTAL GRAMS
	70	27.47	18.31	27.47	18.31	91.56
	74	9.16	0	0	0	9.16
	77	9.16	0	0	9.16	18.32
	78	0	9.16	0	0	9.16

	80	9.16	9.16	9.16	9.16	36.64
	85	9.16	0	0	0	9.16
	88	9.16	18.31	18.31	9.16	54.94
TOTAL		73.27	54.94	54.94	45.79	228.94

DRUG CODE:2315DRUG NAME:SECOBARBITAL (SCHEDULE 2)

STATE: NEW YORK

ZIP CODE	QUARTER 1	QUARTER 2	QUARTER 3	QUARTER 4	TOTAL GRAMS
100	100.71	119.02	64.09	54.93	338.75
101	9.16	0	0	0	9.16
103	36.62	27.47	27.47	27.47	119.03
105	27.47	9.16	9.16	9.16	54.95
106	9.16	9.16	0	0	18.32
108	9.16	9.16	0	9.16	27.48
110	9.16	0	9.16	9.16	27.48
112	173.95	119.02	109.86	109.86	512.69
113	0	9.16	0	0	9.16
114	18.31	27.47	18.31	18.31	82.4
115	0	9.16	0	0	9.16
116	45.78	36.62	45.78	45.78	173.96
117	18.31	0	0	9.16	27.47
119	9.16	18.31	0	27.47	54.94
142	0	9.16	9.16	9.16	27.48
144	0	0	0	9.16	9.16
TOTAL	466.95	402.87	292.99	338.78	1,501.59

STATE: NORTH CAROLINA

ZIP CODE	QUARTER 1	QUARTER 2	QUARTER 3	QUARTER 4	TOTAL GRAMS
275	0	9.16	0	0	9.16
TOTAL	0	9.16	0	0	9.16

STATE: OHIO

ZIP CODE	QUARTER 1	QUARTER 2	QUARTER 3	QUARTER 4	TOTAL GRAMS
430	9.16	0	9.16	9.16	27.48

	448	36.62	18.31	18.31	27.47	100.71
	452	9.16	0	0	0	9.16
TOTAL		54.94	18.31	27.47	36.63	137.35

STATE: OKLAHOMA

ZIP CODE		QUARTER 1	QUARTER 2	QUARTER 3	QUARTER 4	TOTAL GRAMS
	740	0	0	27.47	9.16	36.63
TOTAL		0	0	27.47	9.16	36.63

STATE: OREGON

ZIP CODE		QUARTER 1	QUARTER 2	QUARTER 3	QUARTER 4	TOTAL GRAMS
	970	9.16	0	9.16	9.16	27.48
	971	0	9.16	9.16	9.16	27.48
	972	119.02	91.55	128.17	128.17	466.91
	973	54.93	36.62	36.62	91.55	219.72
	974	36.62	0	18.31	27.47	82.4
	975	27.47	36.62	9.16	36.62	109.87
	977	0	0	9.16	0	9.16
	978	9.16	0	0	0	9.16
TOTAL		256.36	173.95	219.74	302.13	952.18

STATE: PENNSYLVANIA

ZIP CODE		QUARTER 1	QUARTER 2	QUARTER 3	QUARTER 4	TOTAL GRAMS
	170	0	0	9.16	0	9.16
	173	9.16	27.47	18.31	36.62	91.56
	187	9.16	27.47	18.31	18.31	73.25
	190	9.16	18.31	27.47	18.31	73.25
	191	9.16	0	0	0	9.16
	193	0	0	9.16	0	9.16
TOTAL		36.64	73.25	82.41	73.24	265.54

STATE: RHODE ISLAND

ZIP CODE		QUARTER 1	QUARTER 2	QUARTER 3	QUARTER 4	TOTAL GRAMS

	28	9.16	9.16	0	18.31	36.63
TOTAL		9.16	9.16	0	18.31	36.63
STATE:	SOUTH CAROLINA					
ZIP CODE	QUARTER 1	QUARTER 2	QUARTER 3	QUARTER 4	TOTAL GRAMS	
	296	0	0	18.31	18.31	36.62
TOTAL		0	0	18.31	18.31	36.62
STATE:	SOUTH DAKOTA					
ZIP CODE	QUARTER 1	QUARTER 2	QUARTER 3	QUARTER 4	TOTAL GRAMS	
	571	0	0	0	9.16	9.16
TOTAL		0	0	0	9.16	9.16
STATE:	TENNESSEE					
ZIP CODE	QUARTER 1	QUARTER 2	QUARTER 3	QUARTER 4	TOTAL GRAMS	
	381	0	9.16	0	0	9.16
ZIP CODE	QUARTER 1	QUARTER 2	QUARTER 3	QUARTER 4	TOTAL GRAMS	
TOTAL		0	9.16	0	0	9.16
STATE:	TEXAS					
ZIP CODE	QUARTER 1	QUARTER 2	QUARTER 3	QUARTER 4	TOTAL GRAMS	
	750	9.16	0	9.16	18.31	36.63
	786	0	0	0	9.16	9.16
TOTAL		9.16	0	9.16	27.47	45.79
STATE:	VERMONT					
ZIP CODE	QUARTER 1	QUARTER 2	QUARTER 3	QUARTER 4	TOTAL GRAMS	
	57	54.93	27.47	0	54.93	137.33
TOTAL		54.93	27.47	0	54.93	137.33

STATE: VIRGINIA

ZIP CODE	QUARTER 1	QUARTER 2	QUARTER 3	QUARTER 4	TOTAL GRAMS
220	9.16	0	0	0	9.16
222	0	9.16	0	0	9.16
235	18.31	18.31	27.47	27.47	91.56
TOTAL	27.47	27.47	27.47	27.47	109.88

STATE: WASHINGTON

ZIP CODE	QUARTER 1	QUARTER 2	QUARTER 3	QUARTER 4	TOTAL GRAMS
980	0	0	18.31	9.16	27.47
981	128.17	128.17	109.86	128.17	494.37
982	9.16	9.16	0	0	18.32
983	45.78	0	9.16	0	54.94
984	0	0	0	9.16	9.16
985	9.16	9.16	0	0	18.32
986	18.31	18.31	0	9.16	45.78
988	9.16	9.16	9.16	9.16	36.64
992	9.16	0	0	0	9.16
993	0	0	9.16	0	9.16
TOTAL	228.9	173.96	155.65	164.81	723.32

STATE: WISCONSIN

ZIP CODE	QUARTER 1	QUARTER 2	QUARTER 3	QUARTER 4	TOTAL GRAMS
535	9.16	0	0	0	9.16
TOTAL	9.16	0	0	0	9.16

DRUG CODE: 7365 DRUG NAME: DRONABINOL IN AN ORAL SOLUTION IN FDA AP

STATE: ALABAMA

ZIP CODE	QUARTER 1	QUARTER 2	QUARTER 3	QUARTER 4	TOTAL GRAMS
352	0	0	0.75	0.15	0.9
354	0	0	0	0.9	0.9
356	0	0	0	0.3	0.3
358	0	0	0	0.3	0.3
363	0	0	0.75	0	0.75

	366	0	0	0	1.05	1.05
TOTAL		0	0	1.5	2.7	4.2

STATE:	ARIZONA				
ZIP CODE	QUARTER 1	QUARTER 2	QUARTER 3	QUARTER 4	TOTAL GRAMS
850	0	0	12	7.2	19.2
852	0	0	2.4	0	2.4
TOTAL	0	0	14.4	7.2	21.6

STATE:	ARKANSAS				
ZIP CODE	QUARTER 1	QUARTER 2	QUARTER 3	QUARTER 4	TOTAL GRAMS
720	0	0	0	0.6	0.6
727	0	0	0.6	0	0.6
TOTAL	0	0	0.6	0.6	1.2

STATE:	CALIFORNIA				
ZIP CODE	QUARTER 1	QUARTER 2	QUARTER 3	QUARTER 4	TOTAL GRAMS
900	0	0	5.55	1.95	7.5
904	0	0	0.9	1.35	2.25
906	0	0	0	0.15	0.15
911	0	0	0	0.15	0.15
913	0	0	1.5	3	4.5
920	0	0	0	6.15	6.15
921	0	0	0.9	6.45	7.35
922	0	0	0	0.6	0.6
924	0	0	0.3	2.7	3
925	0	0	0	0.9	0.9
928	0	0	0	0.15	0.15
933	0	0	0.15	1.2	1.35
937	0	0	0	0.6	0.6
941	0	0	0.6	0	0.6
945	0	0	1.8	0.75	2.55
946	0	0	0.9	0	0.9

	952	0	0	0	0.15	0.15
	956	0	0	0.15	0	0.15
	958	0	0	2.4	2.4	4.8
TOTAL		0	0	15.15	28.65	43.8

STATE: COLORADO

ZIP CODE		QUARTER 1	QUARTER 2	QUARTER 3	QUARTER 4	TOTAL GRAMS
	801	0	0	1.2	0	1.2
	802	0	0	2.4	0	2.4
	815	0	0	0	0.15	0.15
TOTAL		0	0	3.6	0.15	3.75

STATE: CONNECTICUT

ZIP CODE		QUARTER 1	QUARTER 2	QUARTER 3	QUARTER 4	TOTAL GRAMS
	60	0	0	0	0.15	0.15
	64	0	0	0	0.15	0.15
	65	0	0	0	0.15	0.15
	68	0	0	1.2	0	1.2
TOTAL		0	0	1.2	0.45	1.65

STATE: DELAWARE

ZIP CODE		QUARTER 1	QUARTER 2	QUARTER 3	QUARTER 4	TOTAL GRAMS
	198	0	0	0.15	0	0.15
TOTAL		0	0	0.15	0	0.15

STATE: DISTRICT OF COLUMBIA

ZIP CODE		QUARTER 1	QUARTER 2	QUARTER 3	QUARTER 4	TOTAL GRAMS
	200	0	0	0	0.75	0.75
TOTAL		0	0	0	0.75	0.75

STATE: FLORIDA

ZIP CODE		QUARTER 1	QUARTER 2	QUARTER 3	QUARTER 4	TOTAL GRAMS

	323	0	0	0.3	0	0.3
	325	0	0	0.15	0	0.15
	327	0	0	2.4	2.4	4.8
	328	0	0	0.9	1.5	2.4
	330	0	0	0.15	0	0.15
	331	0	0	0.3	0.15	0.45
	333	0	0	0.3	0.3	0.6
	334	0	0	0	0.3	0.3
	335	0	0	4.8	4.8	9.6
	336	0	0	0	1.2	1.2
	337	0	0	0	1.8	1.8
	338	0	0	0	0.45	0.45
	342	0	0	4.65	9.75	14.4
TOTAL		0	0	13.95	22.65	36.6

STATE: GEORGIA

ZIP CODE		QUARTER 1	QUARTER 2	QUARTER 3	QUARTER 4	TOTAL GRAMS
	300	0	0	0.3	1.05	1.35
	301	0	0	0.75	0.15	0.9
	303	0	0	0	1.95	1.95
	314	0	0	0.3	0	0.3
	319	0	0	0	0.15	0.15
TOTAL		0	0	1.35	3.3	4.65

STATE: ILLINOIS

ZIP CODE		QUARTER 1	QUARTER 2	QUARTER 3	QUARTER 4	TOTAL GRAMS
	600	0	0	0.3	0.75	1.05
TOTAL		0	0	0.3	0.75	1.05

STATE: INDIANA

ZIP CODE		QUARTER 1	QUARTER 2	QUARTER 3	QUARTER 4	TOTAL GRAMS
	460	0	0	0.9	0	0.9
	467	0	0	0.45	0	0.45
	473	0	0	0.3	0	0.3

TOTAL	0	0	1.65	0	1.65

STATE: KANSAS

ZIP CODE	QUARTER 1	QUARTER 2	QUARTER 3	QUARTER 4	TOTAL GRAMS
662	0	0	0	0.6	0.6
TOTAL	0	0	0	0.6	0.6

STATE: KENTUCKY

ZIP CODE	QUARTER 1	QUARTER 2	QUARTER 3	QUARTER 4	TOTAL GRAMS
402	0	0	0	0.15	0.15
403	0	0	0.15	0	0.15
405	0	0	0.6	0.6	1.2
TOTAL	0	0	0.75	0.75	1.5

STATE: LOUISIANA

ZIP CODE	QUARTER 1	QUARTER 2	QUARTER 3	QUARTER 4	TOTAL GRAMS
700	0	0	0.15	0.15	0.3
TOTAL	0	0	0.15	0.15	0.3

STATE: MAINE

ZIP CODE	QUARTER 1	QUARTER 2	QUARTER 3	QUARTER 4	TOTAL GRAMS
42	0	0	0.3	0	0.3
TOTAL	0	0	0.3	0	0.3

STATE: MARYLAND

ZIP CODE	QUARTER 1	QUARTER 2	QUARTER 3	QUARTER 4	TOTAL GRAMS
208	0	0	0	0.9	0.9
210	0	0	0	0.3	0.3
212	0	0	0.15	0.6	0.75
TOTAL	0	0	0.15	1.8	1.95

STATE: MASSACHUSETTS

ZIP CODE	QUARTER 1	QUARTER 2	QUARTER 3	QUARTER 4	TOTAL GRAMS
17	0	0	0	0.9	0.9
21	0	0	0.15	0	0.15
22	0	0	0.6	1.95	2.55
23	0	0	0	0.15	0.15
TOTAL	0	0	0.75	3	3.75

STATE: MICHIGAN

ZIP CODE	QUARTER 1	QUARTER 2	QUARTER 3	QUARTER 4	TOTAL GRAMS
480	0	0	0	0.15	0.15
481	0	0	0.3	0.45	0.75
482	0	0	0.3	0.15	0.45
483	0	0	0	0.6	0.6
484	0	0	0.15	0	0.15
488	0	0	0.15	0	0.15
494	0	0	0.15	0	0.15
TOTAL	0	0	1.05	1.35	2.4

STATE: MINNESOTA

ZIP CODE	QUARTER 1	QUARTER 2	QUARTER 3	QUARTER 4	TOTAL GRAMS
551	0	0	0	0.45	0.45
553	0	0	0	2.4	2.4
TOTAL	0	0	0	2.85	2.85

STATE: MISSISSIPPI

ZIP CODE	QUARTER 1	QUARTER 2	QUARTER 3	QUARTER 4	TOTAL GRAMS
392	0	0	0.6	0.15	0.75
TOTAL	0	0	0.6	0.15	0.75

STATE: MISSOURI

ZIP CODE	QUARTER 1	QUARTER 2	QUARTER 3	QUARTER 4	TOTAL GRAMS
630	0	0	0	0.15	0.15

	631	0	0	4.2	2.55	6.75
	640	0	0	0	0.3	0.3
	641	0	0	0	0.6	0.6
	648	0	0	0.15	0	0.15
	657	0	0	0	0.6	0.6
TOTAL		0	0	4.35	4.2	8.55

STATE: MONTANA

ZIP CODE		QUARTER 1	QUARTER 2	QUARTER 3	QUARTER 4	TOTAL GRAMS
	591	0	0	0	0.3	0.3
TOTAL		0	0	0	0.3	0.3

STATE: NEVADA

ZIP CODE		QUARTER 1	QUARTER 2	QUARTER 3	QUARTER 4	TOTAL GRAMS
	891	0	0	4.8	6.6	11.4
	895	0	0	0.15	0	0.15
TOTAL		0	0	4.95	6.6	11.55

DRUG CODE:7365DRUG NAME:DRONABINOL IN AN ORAL SOLUTION IN FDA AP

STATE: NEW HAMPSHIRE

ZIP CODE		QUARTER 1	QUARTER 2	QUARTER 3	QUARTER 4	TOTAL GRAMS
	030	0	0	0.3	0.6	0.9
	031	0	0	0.45	0.45	0.9
TOTAL		0	0	0.75	1.05	1.8

STATE: NEW JERSEY

ZIP CODE		QUARTER 1	QUARTER 2	QUARTER 3	QUARTER 4	TOTAL GRAMS
	070	0	0	70.8	69.9	140.7
	073	0	0	0	0.15	0.15
	076	0	0	0	0.45	0.45
	080	0	0	0	0.9	0.9
	082	0	0	0	0.9	0.9
	088	0	0	0	0.45	0.45

TOTAL	0	0	70.8	72.75	143.55

STATE: NEW MEXICO

ZIP CODE	QUARTER 1	QUARTER 2	QUARTER 3	QUARTER 4	TOTAL GRAMS
871	0	0	0.15	0	0.15
TOTAL	0	0	0.15	0	0.15

STATE: NEW YORK

ZIP CODE	QUARTER 1	QUARTER 2	QUARTER 3	QUARTER 4	TOTAL GRAMS
100	0	0	0	0.15	0.15
103	0	0	0.15	0.45	0.6
112	0	0	0.45	0	0.45
115	0	0	0	0.3	0.3
120	0	0	0.15	0	0.15
140	0	0	0	0.15	0.15
142	0	0	0	0.15	0.15
TOTAL	0	0	0.75	1.2	1.95

STATE: NORTH CAROLINA

ZIP CODE	QUARTER 1	QUARTER 2	QUARTER 3	QUARTER 4	TOTAL GRAMS
271	0	0	0.15	0	0.15
273	0	0	0.3	0	0.3
274	0	0	0	0.3	0.3
276	0	0	0	0.3	0.3
TOTAL	0	0	0.45	0.6	1.05

STATE: OHIO

ZIP CODE	QUARTER 1	QUARTER 2	QUARTER 3	QUARTER 4	TOTAL GRAMS
432	0	0	0	5.1	5.1
436	0	0	0.3	1.2	1.5
441	0	0	0	1.8	1.8
444	0	0	0	0.15	0.15
448	0	0	0.15	0	0.15

TOTAL	0	0	0.45	8.25	8.7

STATE: PENNSYLVANIA

ZIP CODE	QUARTER 1	QUARTER 2	QUARTER 3	QUARTER 4	TOTAL GRAMS
151	0	0	0	0.3	0.3
152	0	0	0	0.3	0.3
157	0	0	0	0.15	0.15
176	0	0	0	0.15	0.15
179	0	0	0	0.15	0.15
186	0	0	0	0.45	0.45
190	0	0	14.1	0.3	14.4
191	0	0	0.3	0	0.3
193	0	0	0	0.15	0.15
TOTAL	0	0	14.4	1.95	16.35

STATE: SOUTH CAROLINA

ZIP CODE	QUARTER 1	QUARTER 2	QUARTER 3	QUARTER 4	TOTAL GRAMS
294	0	0	0.3	0	0.3
TOTAL	0	0	0.3	0	0.3

STATE: TENNESSEE

ZIP CODE	QUARTER 1	QUARTER 2	QUARTER 3	QUARTER 4	TOTAL GRAMS
372	0	0	0	0.6	0.6
376	0	0	0	0.75	0.75
378	0	0	0	0.15	0.15
381	0	0	1.5	0.6	2.1
TOTAL	0	0	1.5	2.1	3.6

STATE: TEXAS

ZIP CODE	QUARTER 1	QUARTER 2	QUARTER 3	QUARTER 4	TOTAL GRAMS
750	0	0	0	0.3	0.3
753	0	0	0.6	1.5	2.1
761	0	0	2.7	0	2.7

	765	0	0	0	0.6	0.6
	770	0	0	0.9	0.9	1.8
	773	0	0	0	0.15	0.15
	775	0	0	0	1.35	1.35
	782	0	0	0	0.3	0.3
	787	0	0	2.4	0	2.4
TOTAL		0	0	6.6	5.1	11.7

STATE: UTAH

ZIP CODE		QUARTER 1	QUARTER 2	QUARTER 3	QUARTER 4	TOTAL GRAMS
	841	0	0	0.45	0.15	0.6
TOTAL		0	0	0.45	0.15	0.6

STATE: VIRGINIA

ZIP CODE		QUARTER 1	QUARTER 2	QUARTER 3	QUARTER 4	TOTAL GRAMS
	201	0	0	0	0.45	0.45
	220	0	0	0	0.15	0.15
	235	0	0	0	0.15	0.15
TOTAL		0	0	0	0.75	0.75

STATE: WISCONSIN

ZIP CODE		QUARTER 1	QUARTER 2	QUARTER 3	QUARTER 4	TOTAL GRAMS
	531	0	0	0.15	0	0.15
TOTAL		0	0	0.15	0	0.15

DRUG CODE: 7379 DRUG NAME: NABILONE

STATE: ARIZONA

ZIP CODE		QUARTER 1	QUARTER 2	QUARTER 3	QUARTER 4	TOTAL GRAMS
	852	0	0	0.15	0.05	0.2
TOTAL		0	0	0.15	0.05	0.2

RETAIL DRUG DISTRIBUTION BY ZIP CODE WITHIN STATE BY GRAMS WT

STATE: CALIFORNIA

ZIP CODE	QUARTER 1	QUARTER 2	QUARTER 3	QUARTER 4	TOTAL GRAMS
945	0	0.25	0.05	0.35	0.65
TOTAL	0	0.25	0.05	0.35	0.65

STATE: COLORADO

ZIP CODE	QUARTER 1	QUARTER 2	QUARTER 3	QUARTER 4	TOTAL GRAMS
801	0.1	0.05	0	0.15	0.3
815	0.05	0	0	0	0.05
TOTAL	0.15	0.05	0	0.15	0.35

STATE: CONNECTICUT

ZIP CODE	QUARTER 1	QUARTER 2	QUARTER 3	QUARTER 4	TOTAL GRAMS
60	0.1	0.1	0.1	0	0.3
64	0	0.05	0	0	0.05
TOTAL	0.1	0.15	0.1	0	0.35

STATE: FLORIDA

ZIP CODE	QUARTER 1	QUARTER 2	QUARTER 3	QUARTER 4	TOTAL GRAMS
322	0.05	0	0	0	0.05
330	0.25	0.2	0.05	0	0.5
331	0	0	0	0.15	0.15
335	0.4	0.15	0.2	0	0.75
336	0.15	0	0	0	0.15
337	0.05	0.1	0	0	0.15
338	0.15	0	0.1	0.05	0.3
346	0	0	0.1	0	0.1
TOTAL	1.05	0.45	0.45	0.2	2.15

STATE: HAWAII

ZIP CODE	QUARTER 1	QUARTER 2	QUARTER 3	QUARTER 4	TOTAL GRAMS
967	0	0	0.15	0	0.15
968	0.45	0	0	0	0.45

TOTAL	0.45	0	0.15	0	0.6

STATE: ILLINOIS

ZIP CODE	QUARTER 1	QUARTER 2	QUARTER 3	QUARTER 4	TOTAL GRAMS
605	0.1	0	0	0	0.1
606	0.1	0.05	0.1	0.1	0.35
619	0.05	0	0	0	0.05
TOTAL	0.25	0.05	0.1	0.1	0.5

STATE: IOWA

ZIP CODE	QUARTER 1	QUARTER 2	QUARTER 3	QUARTER 4	TOTAL GRAMS
500	0.05	0.05	0	0	0.1
TOTAL	0.05	0.05	0	0	0.1

STATE: KENTUCKY

ZIP CODE	QUARTER 1	QUARTER 2	QUARTER 3	QUARTER 4	TOTAL GRAMS
410	0.15	0.2	0	0	0.35
TOTAL	0.15	0.2	0	0	0.35

STATE: MAINE

ZIP CODE	QUARTER 1	QUARTER 2	QUARTER 3	QUARTER 4	TOTAL GRAMS
44	0.1	0.1	0.1	0	0.3
TOTAL	0.1	0.1	0.1	0	0.3

STATE: MARYLAND

ZIP CODE	QUARTER 1	QUARTER 2	QUARTER 3	QUARTER 4	TOTAL GRAMS
208	0.7	0.9	1	0.8	3.4
212	0.1	0	0	0	0.1
216	0.25	0.2	0.05	0.1	0.6
TOTAL	1.05	1.1	1.05	0.9	4.1

STATE:	MINNESOTA				
ZIP CODE	QUARTER 1	QUARTER 2	QUARTER 3	QUARTER 4	TOTAL GRAMS
551	0.05	0	0	0	0.05
558	0.05	0	0	0	0.05
TOTAL	0.1	0	0	0	0.1

STATE:	MISSOURI				
ZIP CODE	QUARTER 1	QUARTER 2	QUARTER 3	QUARTER 4	TOTAL GRAMS
631	0.2	0	0	0.05	0.25
641	0	0.1	0.1	0.1	0.3
TOTAL	0.2	0.1	0.1	0.15	0.55

STATE:	NEBRASKA				
ZIP CODE	QUARTER 1	QUARTER 2	QUARTER 3	QUARTER 4	TOTAL GRAMS
681	0.15	0	0	0	0.15
684	0	0	0	0.15	0.15
TOTAL	0.15	0	0	0.15	0.3

STATE:	NEW HAMPSHIRE				
ZIP CODE	QUARTER 1	QUARTER 2	QUARTER 3	QUARTER 4	TOTAL GRAMS
37	0	0	0	0.05	0.05
TOTAL	0	0	0	0.05	0.05

STATE:	NEW JERSEY				
ZIP CODE	QUARTER 1	QUARTER 2	QUARTER 3	QUARTER 4	TOTAL GRAMS
80	0	0	0.1	0	0.1
83	0.1	0	0	0	0.1
87	0	0.1	0	0	0.1
88	0	0.05	0	0	0.05
TOTAL	0.1	0.15	0.1	0	0.35

DATE RANGE: 01/01/2017 TO 12/31/2017 ARCOS 3 - REPORT 1 Run Date: 07/03/2018

RETAIL DRUG DISTRIBUTION BY ZIP CODE WITHIN STATE BY GRAMS WT

STATE: NEW MEXICO

ZIP CODE	QUARTER 1	QUARTER 2	QUARTER 3	QUARTER 4	TOTAL GRAMS
877	0	0	0.1	0	0.1
TOTAL	0	0	0.1	0	0.1

STATE: NEW YORK

ZIP CODE	QUARTER 1	QUARTER 2	QUARTER 3	QUARTER 4	TOTAL GRAMS
100	0.15	0	0	0	0.15
110	0	0.05	0.05	0	0.1
117	0	0.05	0	0	0.05
132	0.05	0.05	0	0	0.1
TOTAL	0.2	0.15	0.05	0	0.4

STATE: NORTH CAROLINA

ZIP CODE	QUARTER 1	QUARTER 2	QUARTER 3	QUARTER 4	TOTAL GRAMS
277	0.2	0.15	0.15	0.25	0.75
TOTAL	0.2	0.15	0.15	0.25	0.75

STATE: OHIO

ZIP CODE	QUARTER 1	QUARTER 2	QUARTER 3	QUARTER 4	TOTAL GRAMS
430	0.1	0.1	0	0	0.2
432	0	0	0.1	0.7	0.8
TOTAL	0.1	0.1	0.1	0.7	1

STATE: OREGON

ZIP CODE	QUARTER 1	QUARTER 2	QUARTER 3	QUARTER 4	TOTAL GRAMS
972	0	0	0	0.05	0.05
TOTAL	0	0	0	0.05	0.05

DRUG CODE:7379DRUG NAME:NABILONE

STATE: PENNSYLVANIA

ZIP CODE	QUARTER 1	QUARTER 2	QUARTER 3	QUARTER 4	TOTAL GRAMS
184	0	0	0.15	0	0.15
187	0.05	0.05	0	0.1	0.2
TOTAL	0.05	0.05	0.15	0.1	0.35

STATE: VIRGINIA

ZIP CODE	QUARTER 1	QUARTER 2	QUARTER 3	QUARTER 4	TOTAL GRAMS
220	0.15	0.1	0.05	0	0.3
222	0	0.2	0.1	0	0.3
231	0	0.05	0.05	0	0.1
232	0.05	0	0	0	0.05
TOTAL	0.2	0.35	0.2	0	0.75

STATE: WASHINGTON

ZIP CODE	QUARTER 1	QUARTER 2	QUARTER 3	QUARTER 4	TOTAL GRAMS
980	0	0	0	0.05	0.05
TOTAL	0	0	0	0.05	0.05

DRUG CODE: 9041L DRUG NAME: COCAINE

STATE: ALABAMA

ZIP CODE	QUARTER 1	QUARTER 2	QUARTER 3	QUARTER 4	TOTAL GRAMS
350	0.71	0	0	0.29	1
351	1.29	2.29	1.29	1.29	6.16
352	14.28	13.28	11.28	7.86	46.7
354	0.43	1.14	1	0.71	3.28
356	2.57	3.71	2.29	4.14	12.71
357	1.14	0.57	0.86	0.86	3.43
358	4.86	3.57	3.71	1	13.14
359	0.43	2.29	1.86	1.43	6.01
360	0.29	1.71	0	1.57	3.57
361	20.28	22.71	15.71	16.43	75.13

	362	2.29	0	0.71	1.14	4.14
	364	1.57	1.57	1.57	0.29	5
	365	6.86	8	6	8.29	29.15
	366	3.86	3	3	2.86	12.72
	367	0	0	0.29	0	0.29
	368	1.57	2	0.29	2.43	6.29
TOTAL		62.43	65.84	49.86	50.59	228.72

STATE: ALASKA

ZIP CODE		QUARTER 1	QUARTER 2	QUARTER 3	QUARTER 4	TOTAL GRAMS
	995	4.43	4.57	3.43	6	18.43
	996	0.29	1	1.29	0.29	2.87
	997	3.57	14.07	4.29	3.43	25.36
	998	0.29	0	0.14	0.29	0.72
	999	0	0.14	0.43	0	0.57
TOTAL		8.58	19.78	9.58	10.01	47.95

STATE: ARIZONA

ZIP CODE		QUARTER 1	QUARTER 2	QUARTER 3	QUARTER 4	TOTAL GRAMS
	850	42.71	42.46	39.71	39.14	164.02
	851	2.43	4.57	0.71	3.14	10.85
	852	36.35	27.78	29.57	25.43	119.13
	853	5.14	8.71	9.14	8.86	31.85
	855	0	0	0.29	0	0.29
	856	2.29	1.29	1.14	1.29	6.01
	857	9.71	13.71	11.43	16.14	50.99
	859	0.57	0	0.29	0.14	1
	860	7.43	5.71	3	3.29	19.43
	863	0.29	0	1	0.71	2
	864	0.71	2.57	3.14	1.71	8.13
	865	2.86	2.86	0.14	0	5.86
TOTAL		110.49	109.66	99.56	99.85	419.56

STATE: ARKANSAS

RETAIL DRUG DISTRIBUTION BY ZIP CODE WITHIN STATE BY GRAMS WT

ZIP CODE	QUARTER 1	QUARTER 2	QUARTER 3	QUARTER 4	TOTAL GRAMS
716	0	0	0.29	0	0.29
719	0.86	1.71	0.43	1.14	4.14
720	10	6	9	9.43	34.43
721	1.14	0.57	2.29	2.14	6.14
722	1.86	1.71	4.57	0.57	8.71
723	0	4.61	0.14	0	4.75
724	16.18	15.75	12.03	16.18	60.14
725	1.29	1.57	1	0.14	4
726	2.29	1.86	2.57	3.57	10.29
727	19.86	2.86	2.71	2.71	28.14
728	0	0.29	0.29	0.29	0.87
729	2.86	4	2.86	4	13.72
TOTAL	56.34	40.93	38.18	40.17	175.62

STATE: CALIFORNIA

ZIP CODE	QUARTER 1	QUARTER 2	QUARTER 3	QUARTER 4	TOTAL GRAMS
900	53.78	53.32	56.92	52.6	216.62
902	20.71	21	18	22.93	82.64
903	0.57	0	0.57	0.29	1.43
904	20.57	18.28	17.32	17.86	74.03
905	13.14	10.43	18.71	18.28	60.56
906	3.86	1.71	2.43	4.29	12.29
907	12.28	12.71	11.43	7.86	44.28
908	16.86	17.57	17.71	14	66.14
910	7.71	7.29	6.71	6.29	28
911	5	4	3.71	4.29	17
912	10.14	11.14	10.29	9	40.57
913	28.57	33.57	27.86	24	114
914	9.71	14.93	6.43	4.71	35.78
915	0	0.29	0	0	0.29
917	22.14	18.43	16.14	14	70.71
918	0.71	0.86	0	1.57	3.14
919	9.29	8.29	10.29	7.14	35.01
920	35.5	34.32	43.03	28.39	141.24
921	59	59.42	60.14	56.57	235.13

	922	16.57	15.14	12.86	14.14	58.71
	923	13.14	12.14	13	9.71	47.99
	925	11.71	7.14	10	9.29	38.14
	926	19.14	20.14	24.14	24.86	88.28
	927	9.86	6.86	8.14	12.71	37.57
	928	13.14	19.86	13.86	13	59.86
	930	3.71	4.71	7.14	7	22.56
	931	6.43	13.86	8.71	10.14	39.14
	932	23.14	18.21	24.43	26.14	91.92
	933	17.71	11.28	14.14	12.14	55.27
	934	7.14	5.57	7.86	7.43	28
	935	4.57	1.86	5.71	5.14	17.28
	936	3.57	1.86	2.86	2.57	10.86
	937	11.43	7.14	8.86	12.28	39.71
	939	17.57	12.28	14.71	16.78	61.34
	940	25.82	27.61	19	21.14	93.57
	941	83.28	69.78	48.96	45.6	247.62
	943	24.18	14.71	14.28	24	77.17
	944	0	2.14	2.29	5.32	9.75
	945	37.14	44.93	36.35	35	153.42
	946	21.43	21.57	23	11.86	77.86
	947	3.43	1.29	1.14	2.29	8.15
	948	9.57	10.29	9	6.86	35.72
	949	10.29	12.43	11.57	14.86	49.15
	950	16.86	10.57	16.14	17.14	60.71
	951	24.86	16.28	25.28	15.14	81.56
	952	14.39	17.39	13.07	19.71	64.56
	953	35.35	26.57	24.71	25.21	111.84
	954	5.36	8.07	8.14	8	29.57
	955	2.43	0.71	1.86	5.29	10.29
	956	38.57	39.57	30	38.93	147.07
	957	0.29	1.29	0.86	0.57	3.01
	958	35.57	35.43	27	31.57	129.57
	959	14.28	16	10.86	14	55.14
	960	13.43	12.43	5.71	5.79	37.36
	961	2.71	4.43	2.43	2.43	12
TOTAL		927.61	879.1	835.76	828.11	3,470.58

STATE: COLORADO

ZIP CODE	QUARTER 1	QUARTER 2	QUARTER 3	QUARTER 4	TOTAL GRAMS
800	8.29	11.71	6.14	8.29	34.43
801	16.43	28.18	17.78	22	84.39
802	19.25	13.86	9.86	12.14	55.11
803	0.86	1.86	1	1.86	5.58
804	0.57	0.71	0.29	2.29	3.86
805	24.57	40.28	20.57	24.86	110.28
806	0.71	1.14	3.29	1.57	6.71
807	0.71	1	1.43	1.29	4.43
809	1.57	0.86	1.71	1.86	6
810	6	7.14	5.43	1	19.57
813	0.29	0.29	0.57	0.43	1.58
814	1.71	1.14	1.71	0.86	5.42
816	1.14	1	1.43	2.14	5.71
TOTAL	82.1	109.17	71.21	80.59	343.07

STATE: CONNECTICUT

ZIP CODE	QUARTER 1	QUARTER 2	QUARTER 3	QUARTER 4	TOTAL GRAMS
060	0.14	0.43	0.57	0.57	1.71
061	1.07	0.71	1.71	0	3.49
062	2.57	1.43	2.57	0.86	7.43
063	6.57	4.86	1.43	3.14	16
064	4.86	1.43	3	1.43	10.72
065	8.14	9.29	10.14	8.29	35.86
066	1.43	1.43	1.43	1.43	5.72
067	0.86	2.29	2.57	1.29	7.01
068	13.43	10.43	26.71	12.43	63
TOTAL	39.07	32.3	50.13	29.44	150.94

STATE: DELAWARE

ZIP CODE	QUARTER 1	QUARTER 2	QUARTER 3	QUARTER 4	TOTAL GRAMS
197	0.57	0.57	1.71	0.57	3.42

	198	0.43	0.86	1.14	0.71	3.14
	199	5.57	17.43	12	10.29	45.29
TOTAL		6.57	18.86	14.85	11.57	51.85

STATE:	DISTRICT OF COLUMBIA					
ZIP CODE		QUARTER 1	QUARTER 2	QUARTER 3	QUARTER 4	TOTAL GRAMS
	200	22.25	21.46	28.14	20.75	92.6
	204	0.29	0	0	0	0.29
TOTAL		22.54	21.46	28.14	20.75	92.89

STATE:	FLORIDA					
ZIP CODE		QUARTER 1	QUARTER 2	QUARTER 3	QUARTER 4	TOTAL GRAMS
	320	4.14	5.43	3.29	4.29	17.15
	321	1.57	0.14	1	1.57	4.28
	322	28.43	32.86	36.28	35.28	132.85
	323	3.86	4	2.86	3	13.72
	324	0.29	0.43	0.29	0.14	1.15
	325	3.14	5	4.43	0.71	13.28
	326	4.57	5.86	5.14	5.57	21.14
	327	3.79	8.5	6.86	2.43	21.58
	328	2.71	4.14	7	4	17.85
	329	5.86	6.43	10	10.57	32.86
	330	16.28	19	18.86	16.93	71.07
	331	4.57	7.71	9.43	18.89	40.6
	333	15.14	13.28	12	18.43	58.85
	334	28.39	31.61	29.43	26.14	115.57
	335	20.71	9.61	9.04	3.29	42.65
	336	74.57	85.78	67.5	68.78	296.63
	337	8.43	7.57	11.43	6.86	34.29
	338	6.86	2.14	4.14	3.43	16.57
	339	10.14	15.71	7	4.86	37.71
	341	13.71	6.57	18	6.29	44.57
	342	18.28	19.28	17.14	11	65.7
	344	1.29	1.57	1.14	0.57	4.57

	346	3	5.14	4	7.43	19.57
	347	1	1.29	0.86	1.07	4.22
	349	8.43	7.71	7.57	10	33.71
TOTAL		289.16	306.76	294.69	271.53	1,162.14

STATE: GEORGIA

DRUG CODE:9041LDRUG NAME:COCAINE
STATE:GEORGIA

ZIP CODE		QUARTER 1	QUARTER 2	QUARTER 3	QUARTER 4	TOTAL GRAMS
	300	55.75	66	55.28	71.71	248.74
	301	24.43	22	18.86	23.86	89.15
	302	12.43	7.57	10.71	10	40.71
	303	37.28	40.85	36.57	48.28	162.98
	304	3.43	2.29	2.14	2	9.86
	305	1.29	3.43	2.86	5.29	12.87
	306	3.14	3.71	3.14	2.43	12.42
	307	1.29	1.29	0.43	0	3.01
	309	2.57	2.04	0.14	1.57	6.32
	310	0.43	0.43	1.29	1	3.15
	312	0.57	1.14	1.14	0.71	3.56
	314	1.14	4.14	2.14	2	9.42
	315	0.57	0.71	1.29	2.14	4.71
	316	2.57	3.43	1.14	0.29	7.43
	317	6.29	9.57	8.43	9.86	34.15
	319	1.29	2.57	1.57	3	8.43
TOTAL		154.47	171.17	147.13	184.14	656.91

STATE: GUAM

ZIP CODE		QUARTER 1	QUARTER 2	QUARTER 3	QUARTER 4	TOTAL GRAMS
	969	0	0	0.36	0	0.36
TOTAL		0	0	0.36	0	0.36

STATE: HAWAII

ZIP CODE	QUARTER 1	QUARTER 2	QUARTER 3	QUARTER 4	TOTAL GRAMS
967	13.71	9.14	10.57	10.71	44.13
968	14.57	14.71	14.28	9.71	53.27
TOTAL	28.28	23.85	24.85	20.42	97.4

STATE: IDAHO

ZIP CODE	QUARTER 1	QUARTER 2	QUARTER 3	QUARTER 4	TOTAL GRAMS
832	10	1.43	6.86	6.57	24.86
833	10.64	10.21	10.5	15.11	46.46
834	30.03	6.71	8.43	29.57	74.74
835	0	2	0.57	0	2.57
836	9.43	11.28	8.71	11.28	40.7
837	4.07	5.79	10.36	5	25.22
838	0.43	0	1.43	0.14	2
TOTAL	64.6	37.42	46.86	67.67	216.55

STATE: ILLINOIS

ZIP CODE	QUARTER 1	QUARTER 2	QUARTER 3	QUARTER 4	TOTAL GRAMS
600	38.64	24.28	20.86	31	114.78
601	58.85	56.6	53.43	54	222.88
602	12.43	13.43	2.71	14.21	42.78
603	1.14	2.14	1.14	1.57	5.99
604	14.57	22.28	17.28	18.14	72.27
605	40.57	46.28	29.14	39.85	155.84
606	61.28	77.6	44.85	75.42	259.15
607	0.14	0	0	0	0.14
608	0	0	0.29	0	0.29
609	19.14	30.5	29.93	32.5	112.07
610	16	14.71	6.29	3.57	40.57
611	6.71	5.29	6.71	4.29	23
612	5	3.86	2.71	3	14.57
613	0.43	1.14	0.71	1	3.28
614	1	12.5	9.93	10.21	33.64

	615	2.29	2.29	3.86	2.86	11.3
	616	5.43	5.29	4.71	4.14	19.57
	617	7.14	5.14	2.57	6.86	21.71
	618	1.57	2.71	1.57	2.86	8.71
	619	0.43	0	0.14	0	0.57
	620	11	5.57	9.14	1.14	26.85
	622	7.14	5.14	5.57	5.14	22.99
	623	1.43	1.14	1.14	5	8.71
	624	0	0.43	0.43	0.14	1
	625	5.43	7.86	6.43	6.86	26.58
	626	0.57	0.43	0.14	0.57	1.71
	627	1.71	3.71	2.14	1.71	9.27
	628	6.14	5.93	7.86	7.93	27.86
	629	0.43	0.57	1	0	2
TOTAL		326.61	356.82	272.68	333.97	1,290.08

STATE:	INDIANA					
ZIP CODE		QUARTER 1	QUARTER 2	QUARTER 3	QUARTER 4	TOTAL GRAMS
	460	24.64	25.5	24.78	21.07	95.99
	461	11.43	5.14	9.57	11	37.14
	462	93.99	53.85	42.14	56.57	246.55
	463	29.43	22.86	21.14	16.86	90.29
	464	4.43	4.14	3.57	4.71	16.85
	465	7.18	7.61	0.29	2.29	17.37
	466	14.89	17.5	8.57	20.36	61.32
	467	3	5.29	3.93	4.29	16.51
	468	1	1	3	1.14	6.14
	469	18.28	9.29	14.28	16.86	58.71
	471	9.29	5.86	6.57	16	37.72
	472	10.29	8.14	5.14	6.86	30.43
	473	8.71	14.14	2	4.43	29.28
	474	0	0	0.57	0	0.57
	475	1.29	1.14	0.86	1.14	4.43
	476	1.14	1	0.86	1	4
	477	2	3.29	0.29	1.71	7.29
	478	1	0.71	0.29	0.71	2.71

	479	9.14	9.29	5.29	13.93	37.65
TOTAL		251.13	195.75	153.14	200.93	800.95

STATE: IOWA

ZIP CODE		QUARTER 1	QUARTER 2	QUARTER 3	QUARTER 4	TOTAL GRAMS
	500	0.29	0.29	0.29	0.57	1.44
	501	0.14	0.29	0.71	0	1.14
	502	1.71	2.57	1.14	2.14	7.56
	503	14.28	16.14	11.43	11.14	52.99
	504	0.29	0	0.14	0.14	0.57
	505	2.43	2	2	0	6.43
	506	1.57	0.86	2.43	1.43	6.29
	507	23.43	26	19.86	30.28	99.57
	508	0	0	0.29	0.29	0.58
	510	0.86	0	0.14	1.43	2.43
	511	4.71	5.43	6.14	6.57	22.85
	512	0.14	0	0.29	0	0.43
	513	0.14	0	0.29	0.14	0.57
	514	0.57	0	0.29	0	0.86
	515	1.57	1.14	1.29	1	5
	516	0	0	0.43	0.43	0.86
	520	3.14	2.29	0.71	2.86	9
	521	0.71	0.71	0.14	0.43	1.99
	522	15.28	5.43	7.32	6.57	34.6
	523	0	0	0.29	0.29	0.58
	524	9.57	12.28	9.29	11.71	42.85
	525	5.29	6.29	5.71	6.14	23.43
	526	1.57	1.14	1.57	1.71	5.99
	527	5.29	3.29	6.57	1.86	17.01
	528	12.43	16.71	7.36	11.5	48
TOTAL		105.41	102.86	86.12	98.63	393.02

STATE: KANSAS

ZIP CODE		QUARTER 1	QUARTER 2	QUARTER 3	QUARTER 4	TOTAL GRAMS
	660	7.57	7.14	9.29	7.43	31.43

	661	2.43	4	2.57	3.57	12.57
	662	35.68	45.75	47.39	56.53	185.35
	664	0	0.14	0.29	0	0.43
	665	4.71	4.71	5.14	6.86	21.42
	666	3.71	9.71	9.79	9.21	32.42
	667	1.86	3	1.86	1.86	8.58
	670	1	1.14	0.71	0.86	3.71
	671	0	0.71	0.29	0	1
	672	4.57	5.43	3.14	8.86	22
	673	0.57	1.14	0	0.29	2
	674	0.86	0	0.71	1.57	3.14
	676	0	0.29	0.57	0.29	1.15
	677	0.29	0.57	0.29	0	1.15
	678	0	0.71	0.29	0	1
	679	0.29	0.43	0	0.57	1.29
TOTAL		63.54	84.87	82.33	97.9	328.64

STATE:	KENTUCKY				
ZIP CODE	QUARTER 1	QUARTER 2	QUARTER 3	QUARTER 4	TOTAL GRAMS
400	1.71	1	1.57	1.29	5.57
402	26.43	25.14	24	23.14	98.71
403	4.86	4.14	2	2.57	13.57
404	0.57	0.57	0	1.71	2.85
405	18.53	20.96	11.89	12.75	64.13
406	0	0	0.86	0	0.86
407	2.71	1	1.57	1.43	6.71
409	0	0.57	0.86	0.29	1.72
410	11.86	14.86	6.29	10.46	43.47
411	9.29	11.14	7.29	9.86	37.58
412	0.86	0.14	0.14	0.29	1.43
415	0.43	0	0.14	0.43	1
416	2.43	2.86	2.29	4.14	11.72
420	14.36	14.93	10.75	21.21	61.25
421	4.57	8.71	7.57	11.43	32.28
422	0	0.29	1	1.14	2.43
423	4.29	5.14	2.29	6.29	18.01

	424	0.86	0	0.29	1.71	2.86
	426	0	0	0.57	0.43	1
	427	6.14	6.43	7.14	7.57	27.28
TOTAL		109.9	117.88	88.51	118.14	434.43

STATE: LOUISIANA

ZIP CODE		QUARTER 1	QUARTER 2	QUARTER 3	QUARTER 4	TOTAL GRAMS
	700	5.61	1.71	2	1.29	10.61
	701	15.71	15.14	11.28	24	66.13
	703	1.43	3.86	3	5.29	13.58
	704	2	1.71	1.86	1.86	7.43
	705	15.86	18.71	17.57	20.71	72.85
	706	7.86	9	6.57	7.71	31.14
	707	0	0	0.43	0	0.43
	708	3	2.57	3.71	2.43	11.71
	710	0.43	0.14	0.29	0.29	1.15
	711	31	32.86	27.57	36.85	128.28
	712	0.86	1.71	0.57	0.57	3.71
	713	5.14	6.29	8	10.71	30.14
	714	3.43	3	3.86	2	12.29
TOTAL		92.33	96.7	86.71	113.71	389.45

STATE: MAINE

ZIP CODE		QUARTER 1	QUARTER 2	QUARTER 3	QUARTER 4	TOTAL GRAMS
	039	0.14	0.43	1.29	0.57	2.43
	040	0.71	0.43	0.57	0.29	2
	041	4.71	3.14	4	4.43	16.28
	042	0.29	0.71	0.29	0.14	1.43
	043	1.86	1.57	2	2.57	8
	044	8.71	13.36	6.29	3.07	31.43
	045	0	0	0.14	0.57	0.71
	046	1.14	0.86	0.71	1	3.71
	047	2.14	1.71	1.86	1	6.71
	048	2	2.14	3.14	2.43	9.71
	049	0.71	0.57	2.57	1.14	4.99

TOTAL	22.41	24.92	22.86	17.21	87.4

STATE: MARYLAND

ZIP CODE	QUARTER 1	QUARTER 2	QUARTER 3	QUARTER 4	TOTAL GRAMS
206	3.29	0.43	2.86	0.71	7.29
207	3.71	5.43	6.43	4.29	19.86
208	19.07	12	23.14	14.71	68.92
209	6.29	7.86	0	2	16.15
210	11.04	10.71	5.46	9.18	36.39
211	7.43	8.43	6.43	7	29.29
212	33.57	49.18	35.14	28.86	146.75
214	37.25	40.57	29.07	31.36	138.25
215	1.14	1.14	0.86	0.71	3.85
216	1.29	0.71	0.57	2.29	4.86
217	10.57	7.14	6.14	5.14	28.99
218	3.43	0.57	1	0.86	5.86
219	2.71	2.43	2.43	3.57	11.14
TOTAL	140.79	146.6	119.53	110.68	517.6

STATE: MASSACHUSETTS

ZIP CODE	QUARTER 1	QUARTER 2	QUARTER 3	QUARTER 4	TOTAL GRAMS
10	8.43	5.71	5.57	7.57	27.28
11	16.57	17	16.57	16.28	66.42
12	7.86	6.71	4	5.86	24.43
13	0.57	0.71	1	0.86	3.14
14	13	8.29	10.43	8.57	40.29
15	3.86	4.29	3.57	2.43	14.15
16	27.14	23.86	16.57	20.28	87.85
17	10.43	8.71	5	10.86	35
18	20.28	12.71	12.86	18.43	64.28
19	9.71	10.86	8.29	9.14	38
20	0	0.29	0	0.14	0.43
21	84.42	78.28	73.71	88.64	325.05
22	7.29	9.57	7.43	3.86	28.15
23	2.14	2.43	2.29	2.86	9.72

	24	9.14	5.71	6.29	2.86	24
	25	3.86	4	3.43	2.71	14
	26	2.14	1.14	1.14	1	5.42
	27	1.57	2.43	2.43	1.43	7.86
TOTAL		228.41	202.7	180.58	203.78	815.47

STATE: MICHIGAN

ZIP CODE		QUARTER 1	QUARTER 2	QUARTER 3	QUARTER 4	TOTAL GRAMS
	480	43.35	56.71	56.35	59.5	215.91
	481	46.85	37.6	39.78	30	154.23
	482	17.57	18.57	19.43	16.86	72.43
	483	24.43	30.86	20.86	23.28	99.43
	484	6.29	7.14	3.29	4.57	21.29
	485	12	13.86	8.86	11.14	45.86
	486	4.14	3.86	4.57	8.29	20.86
	487	5.71	7.57	4.71	3.71	21.7
	488	8.86	7.71	12	10.14	38.71
	489	3.57	9.29	4.57	4.14	21.57
	490	38.57	43.6	50.39	53.64	186.2
	491	11.78	38.57	4.57	5.61	60.53
	492	5.43	2.71	2.43	3.71	14.28
	493	0.29	0.86	1.43	0.86	3.44
	494	63.14	74.35	52.35	63.71	253.55
	495	33.43	23.71	24.57	30	111.71
	496	16.86	16.14	13.71	14.43	61.14
	497	20	21.18	10.43	30	81.61
	498	4.71	7.14	1.71	7.14	20.7
	499	0.57	0.71	0.57	0.86	2.71
TOTAL		367.55	422.14	336.58	381.59	1,507.86

STATE: MINNESOTA

ZIP CODE		QUARTER 1	QUARTER 2	QUARTER 3	QUARTER 4	TOTAL GRAMS
	550	8	12.57	10.71	11.43	42.71
	551	15.43	11.57	16.14	18.43	61.57
	553	26.43	25.28	25.14	33.93	110.78

	554	81.14	90.24	65.14	73.71	310.23
	557	5.71	6.57	7.29	6.43	26
	558	19.71	22.14	14.14	12.43	68.42
	559	38.96	46.78	41.39	34.96	162.09
	560	7.14	7.43	9.71	5.71	29.99
	561	0.14	0.43	0	0.86	1.43
	562	4.71	3.29	4.86	4.71	17.57
	563	0.57	1.71	0.29	0.57	3.14
	564	1	2.14	1.57	1.43	6.14
	565	0.86	0.43	0.57	0	1.86
	566	1.29	2.86	1	5	10.15
	567	0.43	0.14	1.29	1.57	3.43
TOTAL		211.52	233.58	199.24	211.17	855.51

STATE: MISSISSIPPI

ZIP CODE		QUARTER 1	QUARTER 2	QUARTER 3	QUARTER 4	TOTAL GRAMS
	386	0.29	0	4	0.29	4.58
	388	0.86	1	0.71	0.43	3
	390	1.57	0	0.14	0.43	2.14
	391	0	0.14	0	0.14	0.28
	392	10.57	14.86	15.71	9.29	50.43
	393	0	0	0.14	0	0.14
	394	19.71	20.46	23.89	21.14	85.2
	395	12.43	44	7.43	30.75	94.61
	396	13.39	8.93	8.93	8.93	40.18
	397	20.86	12.14	14.28	11.14	58.42
TOTAL		79.68	101.53	75.23	82.54	338.98

STATE: MISSOURI

ZIP CODE		QUARTER 1	QUARTER 2	QUARTER 3	QUARTER 4	TOTAL GRAMS
	630	5.43	6.71	4.29	2.29	18.72

	631	17	26.14	20.71	25.57	89.42
	633	6.29	5.29	6.57	6.14	24.29
	634	0.29	2.14	1.86	0.57	4.86
	635	0.57	0.29	0.86	0.29	2.01
	636	6.43	4.14	7.14	7.86	25.57
	637	12.5	21	12.21	22	67.71
	638	0.14	0	0.14	0	0.28
	639	0.14	0	0.57	0	0.71
	640	27.75	33.07	38.53	19.46	118.81
	641	16.64	17.28	7.57	6.86	48.35
	644	0.57	1.86	0.29	1.07	3.79
	645	5.71	4.29	5.71	7.57	23.28
	647	2	2.14	2.43	0.71	7.28
	648	16.71	16.57	11.43	12.71	57.42
	650	0	0	0.14	0	0.14
	651	2.71	0.57	0.71	2.43	6.42
	652	14.18	12.18	10.32	11.82	48.5
	653	9	7.57	7	9.43	33
	654	0.71	0.71	1.29	1.93	4.64
	655	0	0.36	0	0	0.36
	656	8.43	4.57	4.86	4.57	22.43
	657	0	0.14	0.43	0	0.57
	658	11	13.86	14.86	11.28	51
TOTAL		164.2	180.88	159.92	154.56	659.56

STATE:	MONTANA				
ZIP CODE	QUARTER 1	QUARTER 2	QUARTER 3	QUARTER 4	TOTAL GRAMS
590	0	0	0.57	0.57	1.14
591	8.29	9.43	10	15.86	43.58
592	3.43	6.29	2.86	4.43	17.01
593	0.57	0	0.86	0	1.43
594	4.14	5.71	4.43	5.29	19.57
596	7.57	9.14	7.14	12.43	36.28
597	3.86	3.57	5.14	4.14	16.71
598	5.32	5.32	0.79	0.71	12.14
599	15.68	15.53	9.93	22.61	63.75

TOTAL		48.86	54.99	41.72	66.04	211.61

STATE:	NEBRASKA					
ZIP CODE		QUARTER 1	QUARTER 2	QUARTER 3	QUARTER 4	TOTAL GRAMS
	680	0.29	1.71	2	1.29	5.29
	681	9.86	12.43	10.71	10.57	43.57
	683	0.14	0	0.14	0	0.28
	684	0.14	0	0.43	0	0.57
	685	0.29	2.86	1.86	1	6.01
	686	0.29	0.14	4.61	0	5.04
	687	0	0.71	2	0	2.71
	688	3.71	3.57	4.71	4.86	16.85
	689	1.29	0.86	2	3.14	7.29
	691	4	4.57	6.29	5.43	20.29
	693	1.57	4.14	2.29	3.29	11.29
TOTAL		21.58	30.99	37.04	29.58	119.19

STATE:	NEVADA					
ZIP CODE		QUARTER 1	QUARTER 2	QUARTER 3	QUARTER 4	TOTAL GRAMS
	890	0.86	1.14	0.29	2.14	4.43
	891	14.57	12	8.57	9.75	44.89
	894	1.29	1.71	1.57	0.71	5.28
	895	21.18	30.39	29.21	19.28	100.06
	897	0.57	2	0.86	0	3.43
	898	6.14	4.86	3.43	7.43	21.86
TOTAL		44.61	52.1	43.93	39.31	179.95

STATE:	NEW HAMPSHIRE					
ZIP CODE		QUARTER 1	QUARTER 2	QUARTER 3	QUARTER 4	TOTAL GRAMS
	30	7	11.04	7.29	13.43	38.76
	31	10.86	15.43	8.29	18	52.58
	32	1	2.43	1.43	1.71	6.57

33	0.29	0	0.57	0.29	1.15
34	0.29	1.86	0	1	3.15
35	1.43	0.43	0.71	1	3.57
37	5	9.14	5.71	5.57	25.42
38	12.86	14.57	16.57	15.14	59.14
TOTAL	38.73	54.9	40.57	56.14	190.34

STATE: NEW JERSEY

ZIP CODE	QUARTER 1	QUARTER 2	QUARTER 3	QUARTER 4	TOTAL GRAMS
070	19	24.43	22.43	14.43	80.29
071	10.43	12.71	8.71	9.43	41.28
072	2.57	0.43	0.86	0.57	4.43
073	1.71	1.29	1	0.43	4.43
074	36.57	42.57	31.28	35.71	146.13
075	4.57	1.71	1	1	8.28
076	11.86	11.57	14.57	12	50
077	6.43	8.86	11.43	7	33.72
078	8.14	6.86	5.14	9.43	29.57
079	3.43	3.71	4.14	5.29	16.57
080	17.86	14	14.14	12	58
081	2.86	1.43	2.86	1.43	8.58
082	9.18	2.71	4.14	3.14	19.17
083	2.43	1.14	1.29	1.43	6.29
084	0.71	2.86	1.71	2.29	7.57
085	2.86	2.29	2.29	2.57	10.01
086	4.14	2.14	1.18	4.14	11.6
087	6	8	2.57	8.43	25
088	14.57	12.57	8.14	12.86	48.14
089	10.71	7.43	10	8.57	36.71
TOTAL	176.03	168.71	148.88	152.15	645.77

STATE: NEW MEXICO

ZIP CODE	QUARTER 1	QUARTER 2	QUARTER 3	QUARTER 4	TOTAL GRAMS

	871	65.92	61.57	65.92	59.71	253.12
	873	0	0.43	0.86	0	1.29
	874	0	0.43	2.71	0.71	3.85
	875	4.57	3.57	2.43	4.14	14.71
	880	1.43	6.29	0	0.57	8.29
	882	4.43	1.29	1.71	1.43	8.86
	883	0	0.14	0.14	0	0.28
TOTAL		76.35	73.72	73.77	66.56	290.4

STATE:	NEW YORK					
ZIP CODE		QUARTER 1	QUARTER 2	QUARTER 3	QUARTER 4	TOTAL GRAMS
	100	138.1	124.92	76.14	125.99	465.15
	101	0.29	1.86	0.43	0.43	3.01
	103	1	0.86	0.57	1	3.43
	104	6.29	9.14	4.43	4.57	24.43
	105	2.86	3.14	7.29	4.57	17.86
	106	0.71	2.14	0	0	2.85
	107	1.43	3.14	1.43	1.14	7.14
	108	0.57	1.14	0	0	1.71
	109	5.71	6.86	6.43	7	26
	110	15.43	8.29	16.5	11.71	51.93
	111	0.43	1	0	0.29	1.72
	112	14.57	21.78	29.71	8.61	74.67
	113	2.86	1.86	5.14	2	11.86
	115	6.43	6.14	17.57	8.43	38.57
	116	1.71	2.14	1.14	1.71	6.7
	117	5.29	4.43	7.43	5.71	22.86
	118	2.29	3.14	3.14	1.43	10
	119	1.57	3.5	1.71	1.14	7.92
	121	0.14	0	0.86	0.14	1.14
	122	2.57	3.71	2.57	1	9.85
	123	0.86	1.43	0.14	1	3.43
	125	0.64	0.36	1.14	3	5.14
	126	9.86	7.71	6.14	6.14	29.85
	127	2.57	0	2.14	0.57	5.28
	128	10.14	11	8.43	9	38.57

	129	0.57	0.86	0.57	0	2
	130	3.29	2.07	1.57	2	8.93
	131	4.29	3.14	1.71	2.29	11.43
	132	6.79	9.64	11.21	7.79	35.43
	133	0	1	0	0.29	1.29
	134	3.71	1.29	2.29	2.14	9.43
	135	2	0.29	1.43	1.71	5.43
	136	2.29	1.57	3.57	4.57	12
	137	0.71	0.57	0.71	1	2.99
	138	0.57	0.57	1.14	1	3.28
	139	2.86	2.29	3.29	1.57	10.01
	140	2	2.43	1.86	2.14	8.43
	141	1.86	4.14	2.43	0	8.43
	142	48.75	60.75	44.71	46.85	201.06
	144	4.71	6.29	3.86	4.71	19.57
	145	0	1.14	1.43	0.43	3
	146	36.68	32.14	23	30.57	122.39
	148	4.43	7.29	11	9.43	32.15
	149	2	3.86	3.29	2.43	11.58
TOTAL		361.83	371.02	319.55	327.5	1,379.90

STATE:	NORTH CAROLINA					
ZIP CODE		QUARTER 1	QUARTER 2	QUARTER 3	QUARTER 4	TOTAL GRAMS
	270	0.43	0.43	1.43	1	3.29
	271	13.14	15.71	18.28	16.28	63.41
	272	2.29	1.43	1.71	3.14	8.57
	273	0.71	0.71	0.14	0.29	1.85
	274	0.29	1.43	0.71	0.57	3
	275	20.93	7.43	15.71	8.14	52.21
	276	2.14	0.86	2.29	2.29	7.58
	277	5.29	2.04	2.43	3.04	12.8
	278	15.71	18.14	11	11.57	56.42
	279	1.86	1	2	2.86	7.72
	280	18.14	14.43	17.43	16.28	66.28
	281	1.71	1	1.57	0.93	5.21
	282	4	8.82	8.29	6.14	27.25

	283	1.43	2.14	0.86	1.43	5.86
	284	11.32	7.86	11.14	10.71	41.03
	285	0.86	5	2.14	1.29	9.29
	286	25.43	37.68	39.1	43.57	145.78
	287	8.14	12.71	12.57	12	45.42
	288	1.71	2.86	3.14	1	8.71
TOTAL		135.53	141.68	151.94	142.53	571.68

STATE: NORTH DAKOTA

ZIP CODE		QUARTER 1	QUARTER 2	QUARTER 3	QUARTER 4	TOTAL GRAMS
	581	1.86	3.29	1.14	2.29	8.58
	582	7.29	6.29	9	5.29	27.87
	583	0	0	0.29	0	0.29
	584	0	1.71	1.57	1.43	4.71
	585	16.25	50.82	18.11	33.82	119
	586	1.14	0	2.86	0.86	4.86
	587	6.43	4.29	4.57	0.57	15.86
	588	0.29	0	0.29	0.29	0.87
TOTAL		33.26	66.4	37.83	44.55	182.04

STATE: OHIO

ZIP CODE		QUARTER 1	QUARTER 2	QUARTER 3	QUARTER 4	TOTAL GRAMS
	430	3.43	5.43	5.14	3.29	17.29
	431	1	0.86	1.29	0.29	3.44
	432	9.86	11.57	13.18	23.93	58.54
	433	3.71	5.86	2.29	4.86	16.72
	435	4.43	5	6.43	7.57	23.43
	436	4.29	2.43	2	3.14	11.86
	437	1.71	2.14	1.43	1.14	6.42
	439	4	6.29	1.43	2.57	14.29
	440	18.57	20	20.71	17	76.28
	441	21.14	29.57	24.71	17.86	93.28
	442	11.86	18.14	13.14	11.86	55
	443	4.14	2.57	4.71	3.57	14.99
	444	1.14	3	1.86	3	9

445	13.57	14.57	11.86	13.43	53.43
446	1	0.43	1.57	1.71	4.71
447	1.14	2.29	0.86	1.71	6
448	4.14	10	4.57	4.57	23.28
449	0	0	1.71	0	1.71
450	11.71	10.86	13	12.57	48.14
451	0.29	0.57	0.14	0.29	1.29
452	14.14	20.32	17.86	21.32	73.64
453	7	6	4.57	3.43	21
454	17.64	7.14	6.57	9.29	40.64
455	2.86	6.29	0.57	6.14	15.86
456	8.14	8.14	5.14	6	27.42
457	0	0	0.71	0.14	0.85
458	4.57	2.57	2.57	2.57	12.28
TOTAL	175.48	202.04	170.02	183.25	730.79

STATE: OKLAHOMA

ZIP CODE	QUARTER 1	QUARTER 2	QUARTER 3	QUARTER 4	TOTAL GRAMS
730	2.86	2.57	2.86	2.43	10.72
731	9.86	7.14	9.14	9.43	35.57
735	0.57	2.29	0.71	0.43	4
737	0.29	0.71	0	0	1
740	3.57	1	1	3.86	9.43
741	13.43	19.43	13.71	21.71	68.28
743	0	0	0	0.29	0.29
744	0.71	0.86	0.71	1.43	3.71
TOTAL	31.29	34	28.13	39.58	133

STATE: OREGON

ZIP CODE	QUARTER 1	QUARTER 2	QUARTER 3	QUARTER 4	TOTAL GRAMS
970	10.86	7.43	8.29	7.86	34.44
971	5.14	5.14	4.29	5.43	20
972	35.32	34.03	33.28	38.75	141.38
973	7.43	11.04	6.71	6.71	31.89

	974	8.57	9.14	7.86	7.57	33.14
	975	27.57	24.57	35.64	15.36	103.14
	977	3.14	0.86	0.29	0.29	4.58
	978	3.29	2.14	3.14	2.29	10.86
	979	2.14	1.57	0.86	0.57	5.14
TOTAL		103.46	95.92	100.36	84.83	384.57

STATE: PENNSYLVANIA

ZIP CODE	QUARTER 1	QUARTER 2	QUARTER 3	QUARTER 4	TOTAL GRAMS
150	4.57	6	5.43	6.86	22.86
151	1.29	1.71	1.14	2.71	6.85
152	10.75	10.43	8	11	40.18
154	2	0.43	1.57	0.57	4.57
155	0.71	0	0.86	1.43	3
156	3.57	7.14	2.14	5	17.85
157	0.86	4.29	3	1.57	9.72
162	1.71	2.43	1.14	1.86	7.14
163	0	0.14	0.14	0	0.28
165	2.29	1.71	2	2.71	8.71
166	0.14	0.29	0	0	0.43
167	0.71	0	0.57	0	1.28
168	8.93	10.64	10.64	5.61	35.82
169	1.43	1.71	2	2.14	7.28
170	6.14	11.43	4.14	2.57	24.28
171	5.61	2.86	1.43	4.71	14.61
172	2.14	1.14	1	2.14	6.42
173	0.29	0	0	0.14	0.43
174	9.86	5.57	13.89	16.18	45.5
175	2.43	3.14	3.43	1.43	10.43
176	17	13.57	16.71	17.14	64.42
177	14.28	19.28	8.86	14.71	57.13
178	10.57	18.18	7.29	7.86	43.9
179	9.29	12.43	6.29	8.57	36.58
180	6	14.14	8.14	11.71	39.99
181	10.57	14.57	9.79	12.71	47.64
182	0.29	0.57	0.57	0.29	1.72

	183	3.43	3.57	4.86	7.29	19.15
	184	0.43	0	0.14	0.29	0.86
	185	3.04	2.29	3.14	2.18	10.65
	186	0.86	4.57	2.43	1.57	9.43
	187	5.36	5.14	5.11	2.43	18.04
	188	3.71	1.86	1	1.14	7.71
	189	13.71	16.71	14	11.18	55.6
	190	70.71	56	61.71	63.85	252.27
	191	118.89	99.64	90.85	97.49	406.87
	193	29.14	26.43	19.28	31	105.85
	194	28.43	28.71	30.43	15.43	103
	196	1.57	1.43	3.29	2	8.29
TOTAL		412.71	410.15	356.41	377.47	1,556.74

STATE: RHODE ISLAND

ZIP CODE		QUARTER 1	QUARTER 2	QUARTER 3	QUARTER 4	TOTAL GRAMS
	28	14.43	12.14	7.71	12.86	47.14
	29	10.86	4.29	9.71	4	28.86
TOTAL		25.29	16.43	17.42	16.86	76

STATE: SOUTH CAROLINA

ZIP CODE		QUARTER 1	QUARTER 2	QUARTER 3	QUARTER 4	TOTAL GRAMS
	290	0	2.86	0.71	0	3.57
	291	3.71	2.29	3.86	3.57	13.43
	292	2.32	2.29	3.57	3.14	11.32
	293	0.71	0.71	0.57	0.57	2.56
	294	4.86	12	2.86	1.71	21.43
	295	7.14	4.71	9.14	8.71	29.7
	296	10.14	5.86	6.57	9	31.57
	297	1.71	0.57	0.71	1.43	4.42
	298	0	0.29	0	0	0.29
TOTAL		30.59	31.58	27.99	28.13	118.29

STATE: SOUTH DAKOTA

ZIP CODE	QUARTER 1	QUARTER 2	QUARTER 3	QUARTER 4	TOTAL GRAMS
570	6.79	7.79	11.25	5.68	31.51
571	24.46	6.57	7.64	33.11	71.78
572	1	0.29	0.14	1.43	2.86
573	0.29	0.57	0.43	0.43	1.72
574	0.14	0.14	0.29	0.29	0.86
575	0	0	0.86	0.86	1.72
577	11.57	9.43	6.86	10.57	38.43
TOTAL	44.25	24.79	27.47	52.37	148.88

STATE: TENNESSEE

ZIP CODE	QUARTER 1	QUARTER 2	QUARTER 3	QUARTER 4	TOTAL GRAMS
370	21	17.43	23.14	22.14	83.71
371	14.93	10.71	11.14	14.43	51.21
372	21.87	20.71	15.43	13.71	71.72
373	4.29	5.14	5.86	5.57	20.86
374	9.43	15.78	6.14	9	40.35
376	17.86	15.57	24	22.14	79.57
377	3	2.86	3.86	3.29	13.01
378	7.71	8.14	5.57	6.71	28.13
379	35	31.14	32.71	31.14	129.99
380	2.71	1.29	1.86	1.43	7.29
381	31.25	19.14	19.64	24.86	94.89
382	0.57	0	0.14	0.43	1.14
383	9.29	10.71	7.86	11.57	39.43
384	1.29	0.57	0.86	1.14	3.86
385	0.86	0.93	1.5	1.57	4.86
TOTAL	181.06	160.12	159.71	169.13	670.02

STATE: TEXAS

ZIP CODE	QUARTER 1	QUARTER 2	QUARTER 3	QUARTER 4	TOTAL GRAMS
750	9.04	12.71	10.57	17.57	49.89
751	1	2.43	0.43	0.71	4.57
752	14.18	15	21.57	16.86	67.61
753	1.29	2.29	2.14	1.14	6.86

754	0	0	0.71	0.43	1.14
756	1.43	1.86	1.86	2.43	7.58
757	1.14	1.14	0.86	1.86	5
758	0.14	0.29	0	0	0.43
759	1	2	3.43	3	9.43
760	33.57	28.86	26	35.71	124.14
761	48.43	45.28	43.43	54.35	191.49
762	8.46	7.18	1.29	8.46	25.39
763	0	0.14	0	1.14	1.28
764	0	0	0	0.14	0.14
765	2.29	2.14	4.29	2.14	10.86
767	0.29	0	0	0	0.29
768	0.57	0	0	0	0.57
769	5.57	7.43	4	4.71	21.71
770	39.85	71.53	47.35	70.17	228.9
773	14.57	27.03	10.29	22	73.89
774	3.57	3.29	5.14	4.43	16.43
775	13.57	11.14	11.43	11.14	47.28
776	4.57	6.43	4	7.43	22.43
777	1.43	0.29	0.86	0.57	3.15
779	3.29	3.29	6.43	5.71	18.72
780	0.43	0.43	1.29	0.43	2.58
781	0.43	0.57	0.43	1.14	2.57
782	13.43	13.28	15	16.86	58.57
784	3.14	2.86	3.86	4.29	14.15
785	4.14	7.57	3.86	4.14	19.71
786	12.28	18.14	13.43	20.28	64.13
787	48	42.64	41.71	52.57	184.92
788	0	0.14	0.14	0	0.28
789	0	0	0.29	0.14	0.43
790	0	0	0.14	0	0.14
791	4.29	2.29	3.71	2	12.29
794	0.43	0.86	0.57	1.29	3.15
795	0.14	0.43	0.29	0.29	1.15
796	5.89	4.89	0.43	0	11.21
797	4.57	7.57	8	6.57	26.71
799	26.28	31.28	27.43	27.57	112.56

TOTAL	332.7	384.7	326.66	409.67	1,453.73

STATE: UTAH

ZIP CODE	QUARTER 1	QUARTER 2	QUARTER 3	QUARTER 4	TOTAL GRAMS
840	26.86	23.43	28.28	24.86	103.43
841	19.71	27.03	16	25.5	88.24
843	7.29	7.14	8.86	14.14	37.43
844	44.14	35.57	33.07	28.71	141.49
845	0.71	0.14	0	0.14	0.99
846	3	2.71	1.57	3.71	10.99
847	1.57	2.57	2.14	2.14	8.42
TOTAL	103.28	98.59	89.92	99.2	390.99

STATE: VERMONT

ZIP CODE	QUARTER 1	QUARTER 2	QUARTER 3	QUARTER 4	TOTAL GRAMS
50	0.43	0.86	0.57	0	1.86
52	0	0.57	0.57	0.29	1.43
53	0.29	0.29	0	0.14	0.72
54	8.21	10.18	3	7.07	28.46
56	1.43	1.29	2.14	0.86	5.72
58	0	0.29	0	0	0.29
TOTAL	10.36	13.48	6.28	8.36	38.48

STATE: VIRGINIA

ZIP CODE	QUARTER 1	QUARTER 2	QUARTER 3	QUARTER 4	TOTAL GRAMS
201	11.43	14.14	14.71	13.14	53.42
220	31.43	32.57	29.57	26	119.57
221	6.14	11.71	8.36	8.64	34.85
222	0.86	1.14	0	1.29	3.29
223	5.43	2.14	2.86	3.14	13.57
224	8.57	5.57	6.29	7	27.43
225	0.29	0.71	0.57	1	2.57
226	0.43	0.86	0.71	2	4
227	0	0	0.57	0	0.57

	228	1.79	0	1	0	2.79
	229	2.71	2	2.57	0.86	8.14
	230	0.57	0	0	0.29	0.86
	231	10.71	10.57	9.14	11.43	41.85
	232	23.71	19.46	17.28	18.86	79.31
	233	1.43	0	2.43	1.29	5.15
	234	8.86	8.29	8.86	7.14	33.15
	235	8.14	9.29	8.71	5	31.14
	236	19.57	16.86	14	22.43	72.86
	237	0.86	0.43	3	0.86	5.15
	238	2.14	2.14	2.14	0.71	7.13
	239	1.71	2.29	1.86	0.71	6.57
	240	1	4.14	2	4.86	12
	241	9.21	2	13.53	1.71	26.45
	242	2	3.43	1.29	2	8.72
	243	0.57	0	0	1.71	2.28
	244	0.29	0.86	2.14	0.57	3.86
	245	2	2.57	1.29	1.57	7.43
	246	0	0	0	0.43	0.43
TOTAL		161.85	153.17	154.88	144.64	614.54

STATE: WASHINGTON

ZIP CODE		QUARTER 1	QUARTER 2	QUARTER 3	QUARTER 4	TOTAL GRAMS
	980	30.43	12.14	31.86	23.14	97.57
	981	43.75	48.32	49.71	42	183.78
	982	3.57	4.14	5.71	2.86	16.28
	983	3.57	3.57	4.71	4.86	16.71
	984	9.57	8	6.43	6.29	30.29
	985	1	4.29	1.43	1.86	8.58
	986	3	3.43	4.14	4.89	15.46
	988	0.57	1	1.29	0	2.86
	989	1.14	0.29	2	1	4.43
	991	0	0.14	0.57	0.29	1
	992	1.29	0.86	1.43	3.71	7.29
	993	15.86	16.43	14.71	19.57	66.57
	994	0.71	1.57	0.43	0.57	3.28

TOTAL	114.46	104.18	124.42	111.04	454.1

STATE: WEST VIRGINIA

ZIP CODE	QUARTER 1	QUARTER 2	QUARTER 3	QUARTER 4	TOTAL GRAMS
247	4.29	0.57	6.43	6.57	17.86
249	0.29	0.29	0.29	0.14	1.01
251	0.89	0	0.89	0.89	2.67
252	0.14	0.14	0.29	0	0.57
253	7.36	9.79	11.71	10.21	39.07
254	4.14	3.71	1.71	1.71	11.27
255	0.14	0.14	0.43	0.57	1.28
256	0.57	1.14	1.57	1.14	4.42
257	2.71	3.57	3.14	2.43	11.85
258	2	3.43	2.86	4.57	12.86
260	5	6.14	5.14	3.43	19.71
261	0.86	0.29	0.57	0.57	2.29
262	0.43	0	0.29	0.29	1.01
263	10.86	10	8.14	10.29	39.29
264	1.71	0	1.29	1.14	4.14
265	11.14	9.14	5.14	7.07	32.49
TOTAL	52.53	48.35	49.89	51.02	201.79

STATE: WISCONSIN

ZIP CODE	QUARTER 1	QUARTER 2	QUARTER 3	QUARTER 4	TOTAL GRAMS
530	14.14	13	11.57	16.43	55.14
531	15.28	15.43	11.57	14.71	56.99
532	29.86	33.57	27.86	24.71	116
534	5.14	4.86	4.29	3.57	17.86
535	30.64	28.93	22	27.43	109
537	24.71	21.86	17.71	18.71	82.99
538	0.29	1.79	1.07	0.29	3.44
539	3.29	3.29	2.21	1.71	10.5
540	1.14	0.57	1.71	0.43	3.85
541	0.71	2.43	1	0.43	4.57

	542	0.71	2.71	0.86	0.29	4.57
	543	5.29	6	3.14	5.14	19.57
	544	2.21	1.07	2.07	3.07	8.42
	545	0.57	3	1	0.29	4.86
	546	8	3.29	2.29	3.79	17.37
	547	0.57	0.57	1	0.86	3
	548	3.43	2.86	3.43	4	13.72
	549	18.43	13	15.86	25.71	73
TOTAL		164.41	158.23	130.64	151.57	604.85

STATE: WYOMING

ZIP CODE		QUARTER 1	QUARTER 2	QUARTER 3	QUARTER 4	TOTAL GRAMS
	820	2.71	0.71	1	1	5.42
	822	1	0.43	0.71	0.71	2.85
	823	0	0.71	0	0	0.71
	824	1	1	2.57	1.14	5.71
	825	1	0.29	0.57	0.57	2.43
	826	1.71	2.86	2.43	2	9
	827	0.57	0.29	1.14	1	3
	829	9.71	5.71	9.71	10.43	35.56
	830	0	0.71	0.29	0.29	1.29
	831	0.29	1.14	1.86	0	3.29
TOTAL		17.99	13.85	20.28	17.14	69.26

DRUG CODE: 9050 DRUG NAME: CODEINE

STATE: ALABAMA

ZIP CODE		QUARTER 1	QUARTER 2	QUARTER 3	QUARTER 4	TOTAL GRAMS
	350	5,975.76	6,003.04	6,076.96	6,075.81	24,131.57
	351	3,877.13	4,024.98	3,676.91	3,565.01	15,144.03
	352	5,756.60	5,523.13	5,030.43	5,490.17	21,800.33
	354	1,570.92	1,741.17	1,883.04	1,650.39	6,845.52
	355	1,720.35	1,820.70	1,807.32	1,967.35	7,315.72
	356	4,999.03	4,919.23	4,855.32	5,287.13	20,060.71
	357	908.4	824.4	732.81	654.16	3,119.77

	358	1,112.08	1,022.20	1,020.80	961.24	4,116.32
	359	2,416.57	2,135.72	2,138.62	2,222.78	8,913.69
	360	2,924.28	3,235.34	3,663.03	3,253.05	13,075.70
	361	2,636.54	2,650.83	2,772.04	2,570.84	10,630.25
	362	1,447.57	1,311.93	1,308	1,325.52	5,393.02
	363	1,907.49	1,742.39	1,727.66	1,607.06	6,984.60
	364	2,046.63	2,023.63	2,034.67	1,769.46	7,874.39
	365	3,851.73	4,096.88	3,910.02	4,126.01	15,984.64
	366	3,946.26	4,146.93	3,978.03	4,642.92	16,714.14
	367	2,382.47	2,465.96	2,498.47	2,578.22	9,925.12
	368	2,269.32	2,349.16	2,162.22	2,251.43	9,032.13
TOTAL		52,097.22	52,426.60	51,821.14	52,546.65	208,891.61

STATE: ALASKA

ZIP CODE		QUARTER 1	QUARTER 2	QUARTER 3	QUARTER 4	TOTAL GRAMS
	995	3,134.65	2,932.57	2,701.47	2,562.46	11,331.15
	996	1,713.78	1,410.44	1,388.38	1,249.32	5,761.92
	997	1,720.54	1,841.48	1,999.09	1,699.89	7,261
	998	561.98	577.47	596.83	460.43	2,196.71
	999	285.47	310.32	354.72	296.15	1,246.66
TOTAL		7,416.42	7,072.28	7,040.49	6,268.25	27,797.44

STATE: AMERICAN SAMOA

ZIP CODE		QUARTER 1	QUARTER 2	QUARTER 3	QUARTER 4	TOTAL GRAMS
	967	353.62	340.36	353.62	313.83	1,361.43
TOTAL		353.62	340.36	353.62	313.83	1,361.43

STATE: ARIZONA

ZIP CODE		QUARTER 1	QUARTER 2	QUARTER 3	QUARTER 4	TOTAL GRAMS
	850	19,008.98	19,381.91	17,940.22	19,124.91	75,456.02
	851	3,162.34	3,093.95	2,903.30	2,860.94	12,020.53
	852	22,323.96	21,462.29	20,792.20	21,321.80	85,900.25
	853	11,431.36	9,911.33	9,636.58	10,321.75	41,301.02

	855	1,088.18	949.94	822.69	940.55	3,801.36
	856	2,682.89	2,341.01	2,051.99	2,204.85	9,280.74
	857	9,916.04	9,806.41	8,810.65	9,121.90	37,655
	859	722.61	708.25	777.62	544.12	2,752.60
	860	1,689.62	1,290.28	1,186.89	1,326.14	5,492.93
	863	2,011.20	2,197.91	1,976.38	1,929.31	8,114.80
	864	2,762.71	2,671.56	2,443.66	2,403.57	10,281.50
	865	1,596.42	1,239.66	1,257.13	998.58	5,091.79
TOTAL		78,396.31	75,054.50	70,599.31	73,098.42	297,148.54

STATE: ARKANSAS

ZIP CODE		QUARTER 1	QUARTER 2	QUARTER 3	QUARTER 4	TOTAL GRAMS
	716	3,902.25	3,806.18	3,723.69	3,616.62	15,048.74
	717	3,642.76	3,808.18	3,533.46	3,322.25	14,306.65
	718	5,824.34	6,179.61	5,636.11	5,699.21	23,339.27
	719	5,692.48	5,379.38	5,036.35	5,298.39	21,406.60
	720	7,222.83	7,255.82	7,094.29	6,501.98	28,074.92
	721	7,657.61	7,632.73	7,200.12	7,171.16	29,661.62
	722	3,185.99	3,137.37	3,013.70	2,935.61	12,272.67
	723	9,387.58	9,809.48	9,445.86	8,674.73	37,317.65
	724	8,212.81	8,749.40	8,350.94	8,239.97	33,553.12
	725	4,421.96	4,704.25	4,410.94	4,900.53	18,437.68
	726	2,671.03	2,667.57	2,300.33	2,280.43	9,919.36
	727	3,786.49	4,123.72	3,986.87	3,623.23	15,520.31
	728	3,038.45	3,404.84	2,970.56	2,938.90	12,352.75
	729	5,473.68	5,805.74	5,270.61	5,047.71	21,597.74
TOTAL		74,120.26	76,464.27	71,973.83	70,250.72	292,809.08

STATE: CALIFORNIA

ZIP CODE		QUARTER 1	QUARTER 2	QUARTER 3	QUARTER 4	TOTAL GRAMS
	900	30,148.97	28,696.47	27,334.41	26,234.99	112,414.84
	902	25,766.03	25,738.62	23,395.66	22,119.55	97,019.86
	903	4,073.66	3,669.17	2,940.90	2,981.40	13,665.13
	904	1,313.23	1,203.86	1,172.17	1,161.61	4,850.87
	905	2,622.93	2,219.84	2,224.59	2,042.06	9,109.42

906	7,837.51	7,338.25	7,190.78	7,165.73	29,532.27
907	10,168.72	9,495.84	9,228.53	9,060.07	37,953.16
908	7,907.95	7,490.81	7,287.29	7,235.34	29,921.39
910	3,321.37	3,312.24	3,248.78	3,021.34	12,903.73
911	2,321.17	2,352	2,347.10	2,100.96	9,121.23
912	3,093.20	3,276.50	2,917.85	3,105.55	12,393.10
913	14,554.66	14,582.33	13,619.31	13,207.91	55,964.21
914	5,168.25	5,027.59	5,137.24	5,023.38	20,356.46
915	1,683.56	1,597.48	1,393.46	1,634.39	6,308.89
916	1,288.94	1,364.90	1,138.24	1,170.60	4,962.68
917	25,096.23	23,783.46	22,695.06	22,273.04	93,847.79
918	1,110.33	1,010.04	927.84	957.78	4,005.99
919	4,814.05	4,864.45	4,215.41	4,179.69	18,073.60
920	19,324.34	18,618.63	18,320.60	18,348.74	74,612.31
921	12,067.04	12,095.28	11,972.97	11,138.94	47,274.23
922	11,026.58	9,822.50	9,660.26	9,665.96	40,175.30
923	20,355.11	20,127.95	19,367.92	18,299.94	78,150.92
924	3,622.42	3,445.17	3,341.34	3,126.47	13,535.40
925	19,227.82	18,453.56	18,377.10	17,495.72	73,554.20
926	16,448.85	11,695.35	14,199.70	11,352.49	53,696.39
927	3,927.80	3,875.59	3,772.31	3,686.55	15,262.25
928	13,266.11	12,775.49	12,590.30	12,055.55	50,687.45
930	7,890.09	8,180.60	7,613.37	6,991.53	30,675.59
931	1,886.71	1,885.75	1,753.98	1,569.10	7,095.54
932	11,491.74	11,141.65	10,453.39	10,914.04	44,000.82
933	8,534.78	7,905.45	7,256.22	7,403.37	31,099.82
934	5,906.51	6,815.27	6,120.62	5,867.68	24,710.08
935	11,256.11	10,887.73	10,814.40	10,476.22	43,434.46
936	9,488.88	8,553.50	7,803.51	8,361.05	34,206.94
937	10,434.14	9,918.06	10,319.10	10,484.31	41,155.61
939	4,168.94	4,103.58	3,772.48	3,730.63	15,775.63
940	6,193.60	6,059.95	6,356.87	6,588.28	25,198.70
941	9,409.34	8,454.05	8,440.85	8,768.22	35,072.46
943	737.19	658.64	773.73	521.24	2,690.80
944	959.03	827.08	909.96	953.36	3,649.43
945	38,692.95	38,201.08	30,926.13	29,035.79	136,855.95
946	4,213.23	3,783.25	4,238.72	4,226.55	16,461.75

	947	1,074.49	1,036.38	1,060.67	954.59	4,126.13
	948	1,630.63	1,460.45	1,455.01	1,563.55	6,109.64
	949	3,550.91	3,222.91	3,683.80	3,695.98	14,153.60
	950	6,874.31	5,938.49	6,320.74	6,738.95	25,872.49
	951	7,380.29	6,894.71	6,153.15	6,607.01	27,035.16
	952	11,606.54	11,141.63	11,153.50	10,825.02	44,726.69
	953	16,860.59	15,765.01	16,620.04	16,752.54	65,998.18
	954	5,222.15	5,617.78	5,123.28	4,878.89	20,842.10
	955	3,402.32	3,650.15	3,295.16	2,850.31	13,197.94
	956	16,212.25	16,080.79	15,134.54	15,850.34	63,277.92
	957	2,535.98	2,553.30	2,521.11	2,333.11	9,943.50
	958	9,057.49	7,816.57	7,740.04	8,173.90	32,788
	959	7,966.17	8,161.52	7,475.68	7,826.04	31,429.41
	960	3,712.11	3,790.12	3,746.16	3,643.54	14,891.93
	961	1,536.29	1,509.86	1,493.99	1,350.88	5,891.02
TOTAL		501,442.59	479,948.68	460,547.32	449,781.77	1,891,720.36

STATE: COLORADO

ZIP CODE		QUARTER 1	QUARTER 2	QUARTER 3	QUARTER 4	TOTAL GRAMS
	800	4,652.42	4,407.04	4,460.78	4,295.93	17,816.17
	801	2,967.21	3,262.62	2,893.44	3,100.23	12,223.50
	802	4,862.67	4,639.22	4,456.39	4,401.44	18,359.72
	803	444.77	392.12	313.65	404.61	1,555.15
	804	685.09	691.3	708.7	565.8	2,650.89
	805	2,291.46	2,383.52	2,368.05	2,335.82	9,378.85
	806	1,161	984.56	960.71	937.48	4,043.75
	807	431.52	340.54	397.92	380.31	1,550.29
	808	686.33	684.38	764.49	718.45	2,853.65
	809	2,702.68	2,533.25	2,483.24	2,465.28	10,184.45
	810	2,115.64	1,898.10	2,079.40	1,862.59	7,955.73
	811	392.47	504.45	475.16	367.61	1,739.69
	812	799.04	713.45	763.81	764.1	3,040.40
	813	314.94	404.63	279.67	302.97	1,302.21
	814	547.66	593.61	647.29	577.2	2,365.76
	815	1,033.62	1,041.46	952.24	1,019.45	4,046.77
	816	464.67	417.34	419.5	500.35	1,801.86

TOTAL		26,553.19	25,891.59	25,424.44	24,999.62	102,868.84

STATE:	CONNECTICUT					
ZIP CODE		QUARTER 1	QUARTER 2	QUARTER 3	QUARTER 4	TOTAL GRAMS
	060	5,466.79	5,414.37	4,936.10	4,654.62	20,471.88
	061	1,733.74	1,664.91	1,493.23	1,521.94	6,413.82
	062	789.64	792.4	697	722.6	3,001.64
	063	1,575.22	1,795.79	1,653.09	1,663.42	6,687.52
	064	5,416.14	5,695.85	5,613	5,235.61	21,960.60
	065	1,592.85	1,620.79	1,641.50	1,651.45	6,506.59
	066	1,511.07	1,632.54	1,408.33	1,557.76	6,109.70
	067	2,473.49	2,612.77	2,416.54	2,462.17	9,964.97
	068	3,272.84	2,997.87	3,075.85	2,974.36	12,320.92
	069	710.43	752.13	744.23	671.71	2,878.50
TOTAL		24,542.21	24,979.42	23,678.87	23,115.64	96,316.14

STATE:	DELAWARE					
ZIP CODE		QUARTER 1	QUARTER 2	QUARTER 3	QUARTER 4	TOTAL GRAMS
	197	3,789.77	3,538.12	3,173.74	3,161.97	13,663.60
	198	2,595.97	2,548.28	2,222.49	2,242.47	9,609.21
	199	3,520.69	3,437.45	3,325.35	3,430.78	13,714.27
TOTAL		9,906.43	9,523.85	8,721.58	8,835.22	36,987.08

STATE:	DISTRICT OF COLUMBIA					
ZIP CODE		QUARTER 1	QUARTER 2	QUARTER 3	QUARTER 4	TOTAL GRAMS
	200	12,414.44	12,277.52	11,652.91	10,877.95	47,222.82
	203	22.47	7.18	39.78	0	69.43
	204	309.39	292.83	311.62	207.75	1,121.59
	205	0	0	2.21	4.42	6.63
TOTAL		12,746.30	12,577.53	12,006.52	11,090.12	48,420.47

STATE:	FLORIDA					
ZIP CODE		QUARTER 1	QUARTER 2	QUARTER 3	QUARTER 4	TOTAL GRAMS

	320	5,345.94	5,280.65	4,952.03	5,109.42	20,688.04
	321	6,451	6,177.37	5,388.62	5,676.65	23,693.64
	322	6,914.87	6,564.70	6,988.39	6,622.72	27,090.68
	323	3,922.88	3,839.42	3,464.92	3,712.41	14,939.63
	324	3,598.55	3,496.16	3,127.18	3,273.06	13,494.95
	325	4,472.48	4,043.95	3,931.68	4,093.73	16,541.84
	326	2,571.05	2,489.47	2,140.62	2,577.05	9,778.19
	327	7,832.51	7,664.85	7,367.62	7,763.98	30,628.96
	328	6,424.90	5,816.85	6,055.86	5,587.21	23,884.82
	329	5,357.06	5,192.75	5,062.79	5,155.39	20,767.99
	330	14,359.39	13,443.96	12,806.51	12,977.03	53,586.89
	331	16,426.32	16,874.26	15,196.94	15,488.15	63,985.67
	333	6,681.66	6,848.63	6,625.89	7,062.15	27,218.33
	334	13,063.62	12,000.27	11,834.92	12,144.81	49,043.62
	335	8,892.98	8,577.59	7,529.19	8,652.85	33,652.61
	336	9,313.41	8,827.56	8,520.66	8,637.44	35,299.07
	337	9,893.78	9,812.72	9,512.71	9,279.27	38,498.48
	338	9,271.87	8,392.51	7,904.54	8,075.30	33,644.22
	339	8,457.80	7,194.42	6,571.35	7,285.99	29,509.56
	341	2,962.29	2,317.99	1,627.26	2,464.54	9,372.08
	342	6,284.53	5,973.71	5,365.55	5,161.04	22,784.83
	344	6,020.24	5,409.67	5,647.63	5,839.43	22,916.97
	346	7,321.36	6,792.32	6,594.70	7,381.78	28,090.16
	347	5,315.87	4,700.84	4,725.61	4,693.20	19,435.52
	349	3,530.14	3,104.28	2,781.33	3,424.89	12,840.64
TOTAL		180,686.50	170,836.90	161,724.50	168,139.49	681,387.39

STATE: GEORGIA

ZIP CODE		QUARTER 1	QUARTER 2	QUARTER 3	QUARTER 4	TOTAL GRAMS
	300	15,962.23	16,583.80	15,201.65	15,874.79	63,622.47
	301	9,646.30	9,949.21	9,103.91	9,518.33	38,217.75
	302	12,084.63	12,883.20	12,038.11	11,739.74	48,745.68
	303	8,441.01	8,915.81	7,580.27	8,036.22	32,973.31
	304	3,389.95	3,185.22	3,162.60	3,003.94	12,741.71
	305	7,123.35	7,073.04	6,810.80	6,533.38	27,540.57
	306	6,244.81	6,208.75	5,817.44	5,986.09	24,257.09

	307	2,800.40	2,768.39	2,763.83	2,844.03	11,176.65
	308	2,306.82	2,448.46	2,354.82	2,338.70	9,448.80
	309	3,988.76	4,087.09	4,059.80	3,877.09	16,012.74
	310	6,022.29	6,227.66	5,808.39	5,894.29	23,952.63
	312	2,215.63	2,026.08	1,964.11	2,097.06	8,302.88
	313	2,058.04	2,050.53	1,865.12	1,853.74	7,827.43
	314	1,807.39	1,633.35	1,620.23	1,790.74	6,851.71
	315	6,756.97	6,990.69	6,664.59	6,255.02	26,667.27
	316	2,445.80	2,722.42	2,715.67	2,569.21	10,453.10
	317	7,026.53	7,175.62	6,549.76	6,539.71	27,291.62
	318	560.18	562.54	419.92	454.09	1,996.73
	319	3,381.72	3,470.18	3,361.47	2,924.04	13,137.41
	398	2,094.46	2,312.87	2,302.25	2,337.75	9,047.33
TOTAL		106,357.27	109,274.91	102,164.74	102,467.96	420,264.88

STATE: GUAM

ZIP CODE		QUARTER 1	QUARTER 2	QUARTER 3	QUARTER 4	TOTAL GRAMS
	969	1,289.01	1,191.33	1,196.49	1,088.84	4,765.67
TOTAL		1,289.01	1,191.33	1,196.49	1,088.84	4,765.67

STATE: HAWAII

ZIP CODE		QUARTER 1	QUARTER 2	QUARTER 3	QUARTER 4	TOTAL GRAMS
	967	8,744.06	9,259.56	8,840.53	8,456.65	35,300.80
	968	5,063.47	4,920.04	4,925.94	4,352.50	19,261.95
TOTAL		13,807.53	14,179.60	13,766.47	12,809.15	54,562.75

STATE: IDAHO

ZIP CODE		QUARTER 1	QUARTER 2	QUARTER 3	QUARTER 4	TOTAL GRAMS
	832	1,659.68	1,529.84	1,542.77	1,692	6,424.29
	833	1,243.51	1,528.35	1,388.48	1,275.29	5,435.63
	834	1,756.58	1,729.11	1,627.53	1,429.56	6,542.78
	835	1,086.03	1,182.29	946.08	1,103.90	4,318.30
	836	3,495.99	3,609.01	3,790.75	3,582.61	14,478.36
	837	2,055.40	1,897.73	1,842.18	1,912.99	7,708.30

	838	2,482.56	2,512.54	2,457.99	2,282.93	9,736.02
TOTAL		13,779.75	13,988.87	13,595.78	13,279.28	54,643.68

STATE:		ILLINOIS				
ZIP CODE		QUARTER 1	QUARTER 2	QUARTER 3	QUARTER 4	TOTAL GRAMS
	600	21,191.02	21,771.86	20,165.68	21,747.71	84,876.27
	601	14,888.81	14,534.54	14,093.75	13,976.37	57,493.47
	602	530.8	518.28	502.06	498.47	2,049.61
	603	1,176.95	1,264.92	1,203.93	1,343.73	4,989.53
	604	19,862.19	19,549.56	19,223.15	18,583.27	77,218.17
	605	9,891.28	9,695.02	9,542.42	9,820.39	38,949.11
	606	34,165.52	32,410.14	31,870.33	31,041.27	129,487.26
	607	1,676.56	1,620.31	1,547.73	1,549.46	6,394.06
	608	1,971.78	2,232.91	1,945.48	2,214.41	8,364.58
	609	2,685.54	2,677.82	2,586.32	2,325.34	10,275.02
	610	3,201.62	3,299.18	2,942.13	3,216.78	12,659.71
	611	5,250.41	4,977.82	4,638.31	4,069.75	18,936.29
	612	2,003.84	1,998.79	1,982.20	2,082.23	8,067.06
	613	1,714.70	1,761.93	1,579.47	1,752.97	6,809.07
	614	2,130.32	1,845.69	2,069.71	1,979.51	8,025.23
	615	2,393.06	2,398.13	2,579.32	2,148.57	9,519.08
	616	3,088.37	3,056.60	3,131.32	2,884.33	12,160.62
	617	3,597.72	3,318.92	3,298.43	3,359.73	13,574.80
	618	4,122.82	4,080.06	3,642.13	3,868.06	15,713.07
	619	1,520.90	1,304.49	1,282.22	1,039.29	5,146.90
	620	5,134.29	5,377.80	5,119.11	4,667.71	20,298.91
	622	6,743.83	6,249.01	6,399.84	5,942.10	25,334.78
	623	1,531.65	1,371.81	1,311.50	1,399.71	5,614.67
	624	1,698.63	1,459.11	1,601.66	1,442.53	6,201.93
	625	2,674.34	2,728.67	2,621.78	2,447.63	10,472.42
	626	2,193.83	1,976.29	2,073.88	1,969.40	8,213.40
	627	3,180.44	3,190.75	3,230.25	3,082.18	12,683.62
	628	2,560.83	2,663.53	2,347.85	2,267.37	9,839.58
	629	4,513.91	4,647.52	4,532.69	4,215.82	17,909.94
TOTAL		167,295.96	163,981.46	159,064.65	156,936.09	647,278.16

STATE: INDIANA

ZIP CODE	QUARTER 1	QUARTER 2	QUARTER 3	QUARTER 4	TOTAL GRAMS
460	2,739.17	2,536.90	2,133.61	2,137.22	9,546.90
461	3,574.03	3,674.31	3,710.16	3,486.61	14,445.11
462	7,393.83	7,051.29	6,306.52	5,793.56	26,545.20
463	8,951.06	9,254.13	8,184.06	8,994.84	35,384.09
464	2,296.27	2,292.07	2,189.97	2,105.09	8,883.40
465	3,077.89	2,856.62	2,805.47	2,749.53	11,489.51
466	1,122.37	1,119.77	1,009.05	977.37	4,228.56
467	1,909.47	1,878.29	1,604.04	1,916.98	7,308.78
468	2,356.42	2,045.89	1,830	2,037.43	8,269.74
469	2,759.42	2,586.92	2,022.77	2,027.91	9,397.02
470	748.23	596.18	856.77	627.47	2,828.65
471	2,085.65	3,125.57	3,644.73	3,952.03	12,807.98
472	1,419.97	1,306.98	1,219.97	1,183.10	5,130.02
473	1,390.34	1,556.96	1,426.57	1,759.60	6,133.47
474	1,630.88	1,786.57	1,429.96	1,514.13	6,361.54
475	1,163.80	1,104.06	958.31	969.94	4,196.11
476	913.73	877.49	744.9	784.38	3,320.50
477	2,417.64	2,284.44	1,817.23	2,030.35	8,549.66
478	2,296.70	1,979.35	1,758.19	1,927.76	7,962
479	1,891.32	2,103.64	1,832.57	1,849.63	7,677.16
TOTAL	52,138.19	52,017.43	47,484.85	48,824.93	200,465.40

STATE: IOWA

ZIP CODE	QUARTER 1	QUARTER 2	QUARTER 3	QUARTER 4	TOTAL GRAMS
500	1,302.11	1,265.88	1,196.75	1,362.57	5,127.31
501	1,092.07	1,112.94	1,095.99	1,034.85	4,335.85
502	1,145.47	1,072.03	1,063.62	1,141.13	4,422.25
503	2,152.90	2,186.62	2,224.83	2,009.37	8,573.72
504	520.48	512.93	339.43	496.71	1,869.55
505	705.14	701.83	685.42	774.63	2,867.02
506	776.07	772.3	779.42	788.08	3,115.87
507	442.1	546.43	572.03	572.39	2,132.95
508	197.8	182.33	240.9	270	891.03

	510	464.65	443.84	477.93	441.46	1,827.88
	511	686.03	720.84	720.47	746.64	2,873.98
	512	141.45	251.25	122.66	167.97	683.33
	513	368.35	452.69	391.7	350.11	1,562.85
	514	370.73	317.88	366.31	300.37	1,355.29
	515	1,010.61	1,009.50	1,018.62	840.12	3,878.85
	516	265.8	276.63	264.11	197.61	1,004.15
	520	1,156.85	1,225.76	1,209.91	1,081	4,673.52
	521	329.85	321.01	298.36	218.8	1,168.02
	522	1,192.15	1,221.97	1,111.55	1,207.77	4,733.44
	523	618.07	603.9	538.24	476.26	2,236.47
	524	902.63	792.13	730.43	837.43	3,262.62
	525	957.13	987.06	847.64	846.25	3,638.08
	526	784.83	910.82	955.6	909.21	3,560.46
	527	1,262.57	1,374.12	1,436.01	1,536.01	5,608.71
	528	1,216.12	1,141.44	1,037.07	1,098.37	4,493
TOTAL		20,061.96	20,404.13	19,725	19,705.11	79,896.20

STATE: KANSAS

ZIP CODE		QUARTER 1	QUARTER 2	QUARTER 3	QUARTER 4	TOTAL GRAMS
	660	3,108.69	3,002.13	3,030.36	2,741.66	11,882.84
	661	1,040.15	1,144.76	1,109.37	951.02	4,245.30
	662	7,853.72	7,633.27	6,261.70	7,405.88	29,154.57
	664	590.64	608.34	510.05	603.49	2,312.52
	665	895.4	955.35	855.88	785.69	3,492.32
	666	1,244.55	1,050.01	1,132.49	1,014.62	4,441.67
	667	1,080.53	1,262.88	1,158.72	1,104.74	4,606.87
	668	287.35	312.98	398.69	397.4	1,396.42
	669	245.32	309.41	276.63	350.3	1,181.66
	670	1,793.11	1,590.64	1,743.38	1,572.27	6,699.40
	671	1,022.35	1,189.75	1,075.46	1,165.28	4,452.84
	672	3,939.71	3,943.97	3,872.13	4,289.35	16,045.16
	673	984.7	758.97	732.27	693.24	3,169.18
	674	1,599.25	1,522.19	1,421.79	1,539.30	6,082.53
	675	861.94	768.58	825.37	847.66	3,303.55
	676	377.16	338.59	372.6	311.42	1,399.77

	677	151.48	216.59	227.64	269.63	865.34
	678	1,033.71	1,039.91	1,035.03	815.4	3,924.05
	679	887.45	791.22	748.48	712.76	3,139.91
TOTAL		28,997.21	28,439.54	26,788.04	27,571.11	111,795.90

STATE:	KENTUCKY					
ZIP CODE		QUARTER 1	QUARTER 2	QUARTER 3	QUARTER 4	TOTAL GRAMS
	400	1,924.94	2,065.90	1,906.13	1,758.84	7,655.81
	401	861.3	1,144.27	1,079.19	994.34	4,079.10
	402	6,340.10	6,603.84	6,305.52	5,716.66	24,966.12
	403	2,482.44	2,467.89	2,521.47	2,486.39	9,958.19
	404	1,158.11	1,062.12	1,166.52	1,055.67	4,442.42
	405	1,368.86	1,400.97	1,310.24	1,388.78	5,468.85
	406	167.97	164.74	269.63	144.76	747.1
	407	1,931.25	1,929.60	1,830.14	1,759.05	7,450.04
	408	870.78	804.48	766.9	771.32	3,213.48
	409	2,700.74	2,433.50	2,283.58	2,252.27	9,670.09
	410	3,863.59	3,748.74	3,671.56	3,723.15	15,007.04
	411	1,358.45	1,257.85	1,169.82	1,401.41	5,187.53
	412	1,664.21	1,841.01	1,657.58	1,891.85	7,054.65
	413	1,640.62	1,604.53	1,635.47	1,405.62	6,286.24
	414	496.34	421.39	451.84	494.73	1,864.30
	415	2,368.12	2,411.22	2,592.45	2,273.01	9,644.80
	416	1,005.37	1,149.25	1,068.58	1,010.02	4,233.22
	417	614.02	717.87	555.44	683.26	2,570.59
	418	592.31	537.2	651.98	627.67	2,409.16
	420	2,349.97	2,161.57	2,236.99	2,213.69	8,962.22
	421	2,748.49	2,416.51	2,550.04	2,267.88	9,982.92
	422	1,505.17	1,434.45	1,486.10	1,278.63	5,704.35
	423	1,938.16	2,114.27	2,118.30	1,895.71	8,066.44
	424	1,895.92	1,647.54	1,631.30	1,596.18	6,770.94
	425	554.92	645.35	676.29	638.72	2,515.28
	426	1,375.05	1,323.08	1,121.04	1,179.42	4,998.59
	427	2,899.26	2,725.14	2,858.95	2,735	11,218.35
TOTAL		48,676.46	48,234.28	47,573.05	45,644.03	190,127.82

STATE: LOUISIANA

ZIP CODE	QUARTER 1	QUARTER 2	QUARTER 3	QUARTER 4	TOTAL GRAMS
700	5,179.85	5,327.24	4,714.36	4,598.45	19,819.90
701	3,109.93	2,716.33	2,572.81	2,606.77	11,005.84
703	2,127.28	2,155.12	1,879.20	1,889.22	8,050.82
704	3,529.33	3,643.46	3,428.85	3,240	13,841.64
705	8,744.65	8,918.03	8,213.23	8,003.93	33,879.84
706	3,344.70	3,280.18	3,398.77	3,283.10	13,306.75
707	2,037.77	2,295.14	1,866.43	2,056.99	8,256.33
708	2,479.22	2,295.19	2,187.66	2,045.64	9,007.71
710	1,404.95	1,724.40	1,370.09	1,341.56	5,841
711	3,319.79	3,534.40	3,237.69	2,816	12,907.88
712	4,728.96	4,573.94	4,435.80	4,255.58	17,994.28
713	3,220.26	3,261.29	2,937.88	2,766.80	12,186.23
714	2,047.07	1,868.57	1,842.04	1,939.76	7,697.44
TOTAL	45,273.76	45,593.29	42,084.81	40,843.80	173,795.66

STATE: MAINE

ZIP CODE	QUARTER 1	QUARTER 2	QUARTER 3	QUARTER 4	TOTAL GRAMS
39	247.53	143.66	121.74	130.58	643.51
40	2,136.48	2,055.36	1,906.17	1,921.95	8,019.96
41	445.56	496.78	417.22	370.71	1,730.27
42	1,771.62	1,655.05	1,374.09	1,644.81	6,445.57
43	877.78	926.42	883.28	916.69	3,604.17
44	1,558.23	1,633.88	1,394.06	1,563.34	6,149.51
45	283.44	306.74	323.22	116.55	1,029.95
46	699.65	870.74	752.02	817.13	3,139.54
47	1,260.28	1,400.06	1,122.98	1,148.40	4,931.72
48	268.24	278.08	256.15	240.14	1,042.61
49	1,281.09	1,241.02	1,224.54	1,072.67	4,819.32
TOTAL	10,829.90	11,007.79	9,775.47	9,942.97	41,556.13

STATE: MARYLAND

ZIP CODE	QUARTER 1	QUARTER 2	QUARTER 3	QUARTER 4	TOTAL GRAMS

	206	4,491.58	4,371.72	4,075.03	4,235.10	17,173.43
	207	15,659.40	14,382.17	13,811.62	12,926.23	56,779.42
	208	6,243.04	5,920.59	6,022.69	5,678.36	23,864.68
	209	2,614.14	2,548.08	2,280.93	2,150.08	9,593.23
	210	6,652.94	6,458.44	5,669.74	5,971.24	24,752.36
	211	3,367.17	3,446.25	2,822.72	3,044.81	12,680.95
	212	11,429.56	11,227.11	10,336.40	9,794.88	42,787.95
	214	1,174.75	1,149.89	1,062.75	1,155.62	4,543.01
	215	1,014.66	990.66	961.95	903.12	3,870.39
	216	1,546.19	1,590.08	1,502	1,325.87	5,964.14
	217	5,252.35	4,874.35	4,436.23	5,129.23	19,692.16
	218	1,619.97	1,524	1,294.13	1,431.43	5,869.53
	219	1,095.18	1,107.77	1,079.14	1,074.16	4,356.25
TOTAL		62,160.93	59,591.11	55,355.33	54,820.13	231,927.50

STATE: MASSACHUSETTS

ZIP CODE		QUARTER 1	QUARTER 2	QUARTER 3	QUARTER 4	TOTAL GRAMS
	010	3,316.18	3,078.84	2,988.53	3,044.63	12,428.18
	011	1,197.72	1,258.30	1,169.52	1,142.63	4,768.17
	012	474.95	617.57	482.95	628.44	2,203.91
	013	628.5	562.19	629.17	523.48	2,343.34
	014	2,017.63	1,703.16	1,777.94	1,698.65	7,197.38
	015	2,962.44	2,925.39	2,947.90	2,841.28	11,677.01
	016	2,104.31	2,183.74	1,905.45	2,027.65	8,221.15
	017	2,829.77	2,653.27	2,341.77	2,572.11	10,396.92
	018	8,881.03	8,370.17	7,925.13	7,504.41	32,680.74
	019	3,285.01	2,895.21	3,146.43	2,938.05	12,264.70
	020	2,385.99	2,258.59	2,073.21	2,108.45	8,826.24
	021	8,128.77	7,582.01	7,316.55	7,408.80	30,436.13
	022	212.85	193.32	160.3	250.07	816.54
	023	2,692.09	2,737.51	2,661.52	2,862.75	10,953.87
	024	1,777.67	1,553.21	1,583.18	1,540.82	6,454.88
	025	719.7	824	894.4	775.24	3,213.34
	026	1,077.53	1,237.90	1,256.09	1,164.09	4,735.61
	027	3,968.99	4,098.90	3,873.28	3,670.79	15,611.96
TOTAL		48,661.13	46,733.28	45,133.32	44,702.34	185,230.07

STATE: MICHIGAN

ZIP CODE	QUARTER 1	QUARTER 2	QUARTER 3	QUARTER 4	TOTAL GRAMS
480	36,198.17	36,064.11	34,190.63	32,880.12	139,333.03
481	54,923.43	53,274.98	52,388.73	49,324	209,911.14
482	55,634.31	52,423.66	50,127.91	46,584.23	204,770.11
483	17,753.25	18,850.11	18,561.04	18,491.20	73,655.60
484	9,508.69	9,880.58	9,735.13	9,482.42	38,606.82
485	9,588.87	10,460.94	9,639.44	8,120.08	37,809.33
486	13,136.89	13,358.88	12,638.50	12,238.48	51,372.75
487	6,712.88	6,757.66	6,988.39	6,475.27	26,934.20
488	10,476.09	10,980.74	11,218.03	10,345.98	43,020.84
489	3,496.55	3,664.48	3,839.48	3,492.28	14,492.79
490	12,602.41	11,890.26	11,943.26	11,073.94	47,509.87
491	1,090.67	1,072.07	975.1	892.31	4,030.15
492	6,588.58	6,457.53	5,809.07	5,620.99	24,476.17
493	3,073.31	2,819.31	2,673.66	2,752.42	11,318.70
494	8,559.94	8,282.64	8,220.50	7,880.76	32,943.84
495	4,638.66	4,545.02	4,049.81	4,388.92	17,622.41
496	3,717.73	3,926.96	3,583.69	3,388.45	14,616.83
497	4,126.17	4,221.14	4,261.51	4,093.81	16,702.63
498	2,775.92	2,702.79	2,820.60	2,640.77	10,940.08
499	1,273.47	1,330.09	1,264.89	1,339.07	5,207.52
TOTAL	265,875.99	262,963.95	254,929.37	241,505.50	1,025,274.81

STATE: MINNESOTA

ZIP CODE	QUARTER 1	QUARTER 2	QUARTER 3	QUARTER 4	TOTAL GRAMS
550	3,913.32	3,663.07	3,867.32	3,690.28	15,133.99
551	6,456.85	6,113.22	5,907.36	5,872.51	24,349.94
553	5,820.03	5,872.72	5,319.57	5,789.02	22,801.34
554	7,537.42	6,847.31	7,173.64	6,566.23	28,124.60
556	121.28	184.86	142.05	137.75	585.94
557	1,753.96	1,898.05	1,976	1,847.78	7,475.79
558	1,004.66	766.47	736.56	736.3	3,243.99
559	1,570.42	1,373.86	1,341.42	1,270.06	5,555.76

	560	1,587.20	1,601	1,644.79	1,540.70	6,373.69
	561	565.38	504.63	729.1	617.05	2,416.16
	562	1,633.36	1,549.33	1,716.66	1,429.92	6,329.27
	563	2,686.26	2,538.61	2,440.48	2,351.72	10,017.07
	564	1,206.77	1,315.85	1,193.24	1,303.54	5,019.40
	565	1,394.46	1,377.85	1,333.09	1,384.54	5,489.94
	566	823.78	885.93	786.97	852.4	3,349.08
	567	839.84	636.67	692.96	763.15	2,932.62
TOTAL		38,914.99	37,129.43	37,001.21	36,152.95	149,198.58

STATE: MISSISSIPPI

ZIP CODE		QUARTER 1	QUARTER 2	QUARTER 3	QUARTER 4	TOTAL GRAMS
	386	3,407.14	3,110.90	3,138.67	2,910.95	12,567.66
	387	1,571.38	1,529.39	1,421.09	1,600.11	6,121.97
	388	3,137.07	3,169.72	2,626.01	2,995.88	11,928.68
	389	1,256.44	1,196.77	1,326.06	1,364.74	5,144.01
	390	2,367.33	2,450.53	2,587.96	2,288.69	9,694.51
	391	2,934.44	3,084.61	3,205.24	2,753.25	11,977.54
	392	1,578.51	1,580.44	1,631.28	1,472	6,262.23
	393	2,451.65	2,552.07	2,574.79	2,726.24	10,304.75
	394	3,440.50	3,384.19	3,180.63	3,215.25	13,220.57
	395	2,975.02	2,705.84	2,966.04	2,596.73	11,243.63
	396	1,104.61	1,000.85	916.2	798.97	3,820.63
	397	1,843.50	1,804.34	1,742.57	1,936.76	7,327.17
TOTAL		28,067.59	27,569.65	27,316.54	26,659.57	109,613.35

STATE: MISSOURI

ZIP CODE		QUARTER 1	QUARTER 2	QUARTER 3	QUARTER 4	TOTAL GRAMS
	630	7,013.04	7,415.04	7,286.34	7,398.78	29,113.20
	631	19,049.55	19,023.57	17,722.22	17,993.63	73,788.97
	633	3,785.93	3,825.03	3,558.02	3,610.19	14,779.17
	634	623.98	818.91	745.61	678.58	2,867.08
	635	821.05	912.77	844.26	803.37	3,381.45
	636	1,922.85	1,916.59	2,048.53	1,925.01	7,812.98
	637	1,312.32	1,164.62	1,147.89	1,103.91	4,728.74

	638	2,331.19	2,546.04	2,614.08	2,466.62	9,957.93
	639	1,347.42	1,455.44	1,433.34	1,385.91	5,622.11
	640	5,482.72	6,028.61	5,313.70	5,578.80	22,403.83
	641	5,638.29	5,723.65	5,360.75	5,619.47	22,342.16
	644	601.99	632.45	622.51	782.92	2,639.87
	645	664.24	798.28	665.56	609.1	2,737.18
	646	701.1	732.91	755.32	676.5	2,865.83
	647	1,645.51	1,491.91	1,541.53	1,669.52	6,348.47
	648	1,976.29	1,587.93	1,908.23	1,924.97	7,397.42
	650	1,247.41	1,400.22	1,108.74	1,209.29	4,965.66
	651	663.31	829.41	918.13	753.69	3,164.54
	652	2,051.77	2,023.30	1,922.09	2,026.16	8,023.32
	653	1,168.65	1,044.14	970.03	1,118.12	4,300.94
	654	722.7	796.36	797.83	845.17	3,162.06
	655	1,279.75	1,273.72	1,176.37	1,296.11	5,025.95
	656	1,562.61	1,505.14	1,510.29	1,482.60	6,060.64
	657	2,784.95	3,026.42	2,989.29	2,802.93	11,603.59
	658	2,254.39	2,116.57	2,062.86	2,271.49	8,705.31
TOTAL		68,653.01	70,089.03	67,023.52	68,032.84	273,798.40

STATE: MONTANA

ZIP CODE		QUARTER 1	QUARTER 2	QUARTER 3	QUARTER 4	TOTAL GRAMS
	590	672.85	722.86	592.1	665.22	2,653.03
	591	1,133.24	1,177.67	1,075.75	1,069.61	4,456.27
	592	483.39	483	415.27	356.89	1,738.55
	593	442.2	457.67	351.59	426.73	1,678.19
	594	1,284.58	1,179.14	1,299.67	1,124.40	4,887.79
	595	303.32	274.05	209.96	311.62	1,098.95
	596	1,048.92	934.79	826.32	919.81	3,729.84
	597	1,315.44	1,522.55	1,469.20	1,317.09	5,624.28
	598	2,174.07	2,357.77	2,256.96	2,298.64	9,087.44
	599	1,416.36	1,399.85	1,396.99	1,413.39	5,626.59
TOTAL		10,274.37	10,509.35	9,893.81	9,903.40	40,580.93

STATE: NEBRASKA

ZIP CODE	QUARTER 1	QUARTER 2	QUARTER 3	QUARTER 4	TOTAL GRAMS
680	1,160.89	1,257.25	1,097.66	1,132.90	4,648.70
681	4,455.03	4,157.17	4,189.23	4,296.68	17,098.11
683	318.59	231.31	349.9	306.63	1,206.43
684	200.47	302.28	239.57	181.74	924.06
685	1,969.44	2,077.69	1,768.72	1,669.77	7,485.62
686	198.26	159.67	228.19	138.95	725.07
687	775.13	771.91	898.94	840.21	3,286.19
688	1,065.95	954.66	1,023.81	1,119.40	4,163.82
689	426.73	407.95	349.47	400.02	1,584.17
690	152.5	179.02	99.45	123.77	554.74
691	871.67	674.8	850.68	634.83	3,031.98
692	59.67	35.36	53.04	17.68	165.75
693	520.09	555.34	493.82	521.38	2,090.63
TOTAL	12,174.42	11,764.41	11,642.48	11,383.96	46,965.27

STATE: NEVADA

ZIP CODE	QUARTER 1	QUARTER 2	QUARTER 3	QUARTER 4	TOTAL GRAMS
890	6,289.11	6,265.73	6,168.22	6,247.43	24,970.49
891	14,734.96	15,092.23	13,891.57	14,236.35	57,955.11
893	43.1	70.72	101.09	113.82	328.73
894	2,570.95	2,698.96	2,516.43	2,652.06	10,438.40
895	2,297.74	2,462.78	2,148.06	2,286.37	9,194.95
897	741.96	615.67	651.19	840.33	2,849.15
898	348.09	245.32	343.67	275.16	1,212.24
TOTAL	27,025.91	27,451.41	25,820.23	26,651.52	106,949.07

STATE: NEW HAMPSHIRE

ZIP CODE	QUARTER 1	QUARTER 2	QUARTER 3	QUARTER 4	TOTAL GRAMS
30	2,207.62	1,973.64	1,935.31	1,968.27	8,084.84
31	1,387.53	1,389.69	1,221.35	1,400.10	5,398.67
32	935.92	777.71	826.05	962.14	3,501.82
33	502.49	514.38	457.93	560.05	2,034.85
34	519.63	414.65	407.75	446.25	1,788.28
35	380.12	356.46	282.13	342.09	1,360.80

	36	48.62	30.94	26.52	48.62	154.7
	37	521.56	508.12	533.71	538.15	2,101.54
	38	1,377.74	1,578.77	1,636.60	1,328.75	5,921.86
TOTAL		7,881.23	7,544.36	7,327.35	7,594.42	30,347.36

STATE: NEW JERSEY

ZIP CODE		QUARTER 1	QUARTER 2	QUARTER 3	QUARTER 4	TOTAL GRAMS
	70	13,590	12,766.01	12,498.90	11,965.54	50,820.45
	71	2,381.90	2,689.18	2,693.57	2,342.86	10,107.51
	72	1,329.54	1,191.91	1,034.35	897.78	4,453.58
	73	2,271.65	2,295.12	2,379.03	2,330.81	9,276.61
	74	4,606.01	4,464.60	4,348.25	4,336.10	17,754.96
	75	1,626.20	1,695.79	1,696.86	1,649.41	6,668.26
	76	4,026.16	4,045.96	3,565.72	3,604.30	15,242.14
	77	6,298.29	5,698.95	5,823.52	5,664.61	23,485.37
	78	3,147.58	2,963.36	2,546.34	2,746.29	11,403.57
	79	1,456.68	1,329.09	1,355.91	1,441.57	5,583.25
	80	14,716.88	14,422.60	13,359.83	13,522.96	56,022.27
	81	2,468.88	2,443.82	2,194.10	2,221.46	9,328.26
	82	2,696.63	2,454.98	2,901.68	2,579.54	10,632.83
	83	3,357.83	3,117.53	3,019.36	2,419.92	11,914.64
	84	448.92	452.67	531.79	452.14	1,885.52
	85	1,951.31	2,019.60	1,815.46	1,636.08	7,422.45
	86	3,130.49	2,393.99	2,303.76	2,151.83	9,980.07
	87	4,413.63	4,286.85	4,119	4,105.97	16,925.45
	88	7,672.89	7,415.51	6,821.14	7,352.60	29,262.14
	89	601.19	683.5	747.26	612.57	2,644.52
TOTAL		82,192.66	78,831.02	75,755.83	74,034.34	310,813.85

STATE: NEW MEXICO

ZIP CODE		QUARTER 1	QUARTER 2	QUARTER 3	QUARTER 4	TOTAL GRAMS
	870	2,107.80	1,993.95	2,016.63	2,061.46	8,179.84
	871	5,975.27	5,675.43	5,769.59	5,796.10	23,216.39
	873	1,720.83	1,816.99	1,682.70	1,582.90	6,803.42
	874	1,294.38	1,238.94	1,217.95	1,082.21	4,833.48

	875	1,141.47	1,103.44	1,059.55	1,082.05	4,386.51
	877	323.76	418.8	371.83	373.23	1,487.62
	878	72.93	24.31	53.04	77.54	227.82
	879	120.54	119.35	108.29	97.24	445.42
	880	1,962.83	1,998.36	2,230.70	2,518.33	8,710.22
	881	1,054.02	1,092.69	1,085.95	1,151.46	4,384.12
	882	2,936.56	2,759.46	2,881.79	2,663.75	11,241.56
	883	978.83	885.78	948.28	899.42	3,712.31
	884	135.99	120.52	101.66	200.09	558.26
TOTAL		19,825.21	19,248.02	19,527.96	19,585.78	78,186.97

STATE: NEW YORK

ZIP CODE	QUARTER 1	QUARTER 2	QUARTER 3	QUARTER 4	TOTAL GRAMS
063	2.21	0	0	2.21	4.42
100	15,198.52	14,410.61	13,904.56	13,232.12	56,745.81
101	696.23	545.74	496.26	721.35	2,459.58
102	22.19	33.7	30.11	26.52	112.52
103	2,931.70	2,929.95	2,801.76	2,508.06	11,171.47
104	11,645.37	11,830.33	10,454.68	10,346.74	44,277.12
105	3,338.41	3,321.95	3,162.41	3,174.97	12,997.74
106	618.82	603.72	480.14	637.42	2,340.10
107	1,575.04	1,752.87	1,510.86	1,434.16	6,272.93
108	595.98	469.36	442.29	346.41	1,854.04
109	4,070.31	3,931.41	4,045.01	3,938.15	15,984.88
110	1,465.08	1,573.48	1,469.33	1,265.72	5,773.61
111	1,253.68	1,594.21	1,052.66	1,178.54	5,079.09
112	18,536.39	17,496.51	17,328.61	16,632.79	69,994.30
113	7,288.47	6,944.95	6,543.07	6,481.63	27,258.12
114	3,758.07	3,700.71	3,326.65	3,025.84	13,811.27
115	4,713.18	4,515.22	4,220.44	4,261.48	17,710.32
116	913.88	998.13	801.53	790.12	3,503.66
117	8,455.74	8,534.14	7,854.47	7,479.90	32,324.25
118	502.37	572.03	671.92	540.86	2,287.18
119	1,194.35	1,191.55	1,201.10	1,159.14	4,746.14
120	1,579.85	1,633.85	1,613.83	1,643.61	6,471.14
121	912.66	562.46	690.93	770.49	2,936.54

	122	700.53	512.28	660.56	654.76	2,528.13
	123	761.91	796.63	753.81	754.01	3,066.36
	124	961.46	1,074.73	1,206.30	1,072.78	4,315.27
	125	2,716.24	2,703.02	2,853.63	2,664.12	10,937.01
	126	507.86	487.78	512.1	510.07	2,017.81
	127	1,171.15	1,077.03	1,284.78	1,287.18	4,820.14
	128	1,477.22	1,649.31	1,489.09	1,714.42	6,330.04
	129	1,547.96	1,586.16	1,447.31	1,530.38	6,111.81
	130	3,095.77	3,172.97	3,181.60	3,083.15	12,533.49
	131	1,217.45	1,213.40	1,142.49	1,088.71	4,662.05
	132	2,092.45	1,834.27	1,778.16	1,807.57	7,512.45
	133	796.17	790.74	670.38	748.37	3,005.66
	134	909.33	1,066.44	938.06	957.21	3,871.04
	135	577.47	456.84	566.51	521.02	2,121.84
	136	3,257.87	3,428.42	3,605.89	3,585.96	13,878.14
	137	914.45	948.27	825.24	871.54	3,559.50
	138	1,270.77	1,491.43	1,146.08	1,162.58	5,070.86
	139	965.66	935.94	924.36	959.03	3,784.99
	140	4,988.26	5,124.57	4,601.93	4,820.45	19,535.21
	141	2,490.38	2,571.86	2,271.22	2,304.62	9,638.08
	142	10,084.59	9,999.99	9,760.50	9,781.47	39,626.55
	143	817.34	747.63	673.69	765.12	3,003.78
	144	2,485.89	2,763.92	2,632.44	2,492.94	10,375.19
	145	2,101.49	1,954.10	2,181.36	1,704.88	7,941.83
	146	4,361.39	4,172.21	3,868.90	4,100.23	16,502.73
	147	1,183.29	1,204.47	1,088.24	1,198.47	4,674.47
	148	2,202.59	2,236.63	2,006.62	1,985.60	8,431.44
	149	478.84	422.03	451.77	425.25	1,777.89
TOTAL		147,404.28	145,569.95	138,625.64	136,150.12	567,749.99

STATE:		NORTH CAROLINA				
ZIP CODE		QUARTER 1	QUARTER 2	QUARTER 3	QUARTER 4	TOTAL GRAMS
	270	1,127.90	1,353.98	918.62	1,143.79	4,544.29
	271	1,203.97	947.78	1,249.77	1,255.82	4,657.34
	272	2,713.53	2,404.64	2,217.99	2,327.80	9,663.96

273	1,918.36	1,707.72	1,630.12	1,733.03	6,989.23
274	2,042.22	1,760.23	1,905.68	1,948.80	7,656.93
275	5,758.38	5,632.59	5,123.46	5,202.48	21,716.91
276	1,768.64	1,857.17	1,520.57	1,794.48	6,940.86
277	1,562.28	1,444.81	1,285.99	1,381.73	5,674.81
278	3,173.32	2,919.69	2,748.63	3,047.89	11,889.53
279	1,455.86	1,437.90	1,263.87	1,281.23	5,438.86
280	2,799.41	3,054.79	2,471.49	3,093.89	11,419.58
281	2,463.57	2,085.65	2,176.63	2,138.70	8,864.55
282	2,585.52	2,359.61	2,280.72	1,937.06	9,162.91
283	7,871.30	7,492	7,138.18	7,037.69	29,539.17
284	2,414.38	2,210.11	2,322.58	2,228.76	9,175.83
285	3,475.05	3,162.12	3,289.84	3,066.25	12,993.26
286	3,425.27	3,304.69	3,102.86	3,052.55	12,885.37
287	2,125.19	2,303.96	2,053.63	2,022.64	8,505.42
288	867.81	821.98	719.48	699.34	3,108.61
289	760.43	733.74	729.67	565.87	2,789.71
TOTAL	51,512.39	48,995.16	46,149.78	46,959.80	193,617.13

STATE: NORTH DAKOTA

ZIP CODE	QUARTER 1	QUARTER 2	QUARTER 3	QUARTER 4	TOTAL GRAMS
580	446.15	500.76	462.98	410.49	1,820.38
581	987.24	917.45	1,052.79	795.94	3,753.42
582	868.34	889.27	947.3	788.43	3,493.34
583	517.16	499.48	490.64	461.91	1,969.19
584	235.74	268.15	230.36	248.4	982.65
585	1,153.01	1,087.87	1,109.21	1,135.55	4,485.64
586	253.88	296.7	305.81	225.78	1,082.17
587	635.6	582.9	567.97	561.34	2,347.81
588	655.42	775.98	694.86	717.53	2,843.79
TOTAL	5,752.54	5,818.56	5,861.92	5,345.37	22,778.39

STATE: OHIO

ZIP CODE	QUARTER 1	QUARTER 2	QUARTER 3	QUARTER 4	TOTAL GRAMS
430	5,209.81	4,880.36	4,493.28	4,604.19	19,187.64

	431	3,422.74	3,238.31	3,032.04	3,286.78	12,979.87
	432	15,748.78	13,875.46	12,949.03	13,682.15	56,255.42
	433	919.08	1,113.81	978.77	837.5	3,849.16
	434	1,390.73	1,315.21	1,411.53	1,151.25	5,268.72
	435	3,515.92	3,165.43	3,054.70	3,396.04	13,132.09
	436	6,822.65	6,374.10	5,813.38	6,238.69	25,248.82
	437	1,884.64	1,756.99	1,778.35	1,583.89	7,003.87
	438	439.07	495.79	358.22	280.23	1,573.31
	439	2,087.34	1,994.34	1,993.13	1,948.29	8,023.10
	440	5,845.97	5,138.25	5,433.32	4,919.72	21,337.26
	441	11,236.97	10,843.86	10,135.68	9,512.74	41,729.25
	442	2,932.42	2,630.82	2,794.03	2,546.33	10,903.60
	443	1,617.49	1,563.75	1,365.99	1,298.07	5,845.30
	444	2,732.60	2,768	2,588.67	2,548.27	10,637.54
	445	1,861.33	1,838.12	1,686.93	1,637.18	7,023.56
	446	2,654.56	2,519.26	2,327.10	2,225.24	9,726.16
	447	2,031.96	1,646.55	1,920.90	1,802.90	7,402.31
	448	2,645.79	2,334.77	2,439.60	2,336.20	9,756.36
	449	1,208	1,292.90	1,120.88	891.42	4,513.20
	450	4,297.01	4,602.57	4,224.61	4,040.38	17,164.57
	451	2,154.42	2,254.31	1,831.41	2,094.80	8,334.94
	452	6,918.75	7,421.54	6,192.78	6,048.95	26,582.02
	453	3,894.22	3,364.05	3,589.46	3,126.51	13,974.24
	454	5,919.52	4,911.97	4,705.80	4,368.57	19,905.86
	455	845.16	886.38	800.67	892.11	3,424.32
	456	4,117.79	3,798.32	3,325.74	3,098.01	14,339.86
	457	1,490.41	1,644.48	1,524.22	1,480.87	6,139.98
	458	3,182.78	3,055.32	2,833.45	2,672.11	11,743.66
TOTAL		109,027.91	102,725.02	96,703.67	94,549.39	403,005.99

STATE: OKLAHOMA

ZIP CODE		QUARTER 1	QUARTER 2	QUARTER 3	QUARTER 4	TOTAL GRAMS
	730	14,040.17	14,482.89	14,144.40	14,172.20	56,839.66
	731	12,337.46	12,966.56	12,313.42	12,941.30	50,558.74
	734	3,671.12	3,655.40	3,752.27	3,907.08	14,985.87
	735	9,351.30	10,001.02	9,938.78	9,351.93	38,643.03

	736	2,841.51	2,306.54	1,961.61	2,228.81	9,338.47
	737	1,871.78	2,115.42	2,035.95	1,851.94	7,875.09
	738	635.4	681.26	485.09	600.02	2,401.77
	739	470.93	395.79	530.61	449.01	1,846.34
	740	13,033.28	13,100.71	12,257.46	11,881.99	50,273.44
	741	5,009.89	4,729.62	4,693.13	4,510.83	18,943.47
	743	3,710.58	4,090.50	3,849.83	3,839.95	15,490.86
	744	6,392.52	6,281.72	6,514.06	6,644.36	25,832.66
	745	1,970	2,057.60	1,945.99	2,075.04	8,048.63
	746	1,768.08	1,591.27	1,673.33	1,811.10	6,843.78
	747	6,699.45	6,185.05	6,344.58	6,156.62	25,385.70
	748	9,206.61	8,603.52	8,847.02	8,427.79	35,084.94
	749	3,642.41	3,617.93	3,346.09	2,919.54	13,525.97
TOTAL		96,652.49	96,862.80	94,633.62	93,769.51	381,918.42

STATE: OREGON

ZIP CODE		QUARTER 1	QUARTER 2	QUARTER 3	QUARTER 4	TOTAL GRAMS
	970	6,574.76	6,529.98	6,330.91	6,652.94	26,088.59
	971	2,390.98	2,172.07	2,285.26	1,982.82	8,831.13
	972	8,438.78	7,809.75	7,412.76	7,739.90	31,401.19
	973	4,876.79	4,436.04	4,784.49	4,712.57	18,809.89
	974	5,100.73	5,191.59	5,212.67	4,993.72	20,498.71
	975	3,337.90	3,443.43	3,333.90	3,505.49	13,620.72
	976	500.28	543.39	493.44	519.04	2,056.15
	977	1,793.90	1,602.41	1,650.22	1,598.66	6,645.19
	978	1,661.71	1,701.80	1,634.85	1,500.43	6,498.79
	979	450.86	279.72	340.54	338.77	1,409.89
TOTAL		35,126.69	33,710.18	33,479.04	33,544.34	135,860.25

STATE: PENNSYLVANIA

ZIP CODE		QUARTER 1	QUARTER 2	QUARTER 3	QUARTER 4	TOTAL GRAMS
	150	3,646.82	3,570.22	3,592.82	3,328.87	14,138.73
	151	3,082.74	2,975.23	2,745.46	2,952.76	11,756.19
	152	5,142.40	5,405.54	5,390.28	4,942.20	20,880.42
	153	1,639.24	1,370.46	1,599.25	1,349.56	5,958.51

154	1,108.91	1,159.87		1,161.94	1,138.65	4,569.37
155	2,516.73	2,941.50		3,024.24	2,439.19	10,921.66
156	2,158.67	1,860.76		1,969.94	2,219.93	8,209.30
157	1,621.64	1,460.17		1,444.76	1,657.24	6,183.81
158	712.2	800.97		783.01	699.95	2,996.13
159	1,382.10	1,268.71		1,444.61	1,460.73	5,556.15
160	1,574.27	1,817.19		1,540.97	1,615	6,547.43
161	1,873.16	1,632.47		1,786.98	1,542.10	6,834.71
162	1,331.95	1,493.98		1,302.79	1,040.44	5,169.16
163	1,205.19	1,471.11		1,193.22	1,343.04	5,212.56
164	756.03	618.39		615.38	611.01	2,600.81
165	1,621.20	1,851.09		1,935.90	1,467.41	6,875.60
166	1,555.27	1,622.44		1,637.91	1,636.80	6,452.42
167	585.68	638.72		491.75	624.54	2,340.69
168	821.3	833.81		738.92	675.57	3,069.60
169	400.76	392.29		351.5	374.42	1,518.97
170	5,212.70	4,955.12		4,676.13	4,676.73	19,520.68
171	1,228.37	1,363.75		1,176.49	1,193.34	4,961.95
172	1,487.05	1,524.58		1,456.66	1,480.05	5,948.34
173	2,657.46	2,584.60		2,522.35	2,273.87	10,038.28
174	1,626.69	1,683.27		1,687.03	1,431.44	6,428.43
175	1,822.62	1,582.29		1,428.93	1,460.93	6,294.77
176	1,265.22	1,314.22		1,364.71	1,185.62	5,129.77
177	1,411.95	1,244.24		1,140.97	946.46	4,743.62
178	1,874.64	1,983.98		1,820.66	1,726.74	7,406.02
179	1,803.52	1,690.67		1,665.48	1,526.53	6,686.20
180	3,116.34	3,084.21		3,070.93	3,204.27	12,475.75
181	1,550.38	1,418		1,490.70	1,563.70	6,022.78
182	1,220.13	1,425.36		1,212.48	1,198.03	5,056
183	1,470.83	1,785.28		1,801.60	1,658.96	6,716.67
184	1,984.19	1,839.46		1,988.88	1,955.66	7,768.19
185	1,759.72	1,693.09		1,692.43	1,744.26	6,889.50
186	2,667.94	2,126.83		2,305.44	2,230.58	9,330.79
187	4,328.51	4,513.90		3,801.57	4,934.54	17,578.52
188	824.82	934.96		904.48	674.08	3,338.34
189	3,334.80	5,736.55		5,650.37	4,831.81	19,553.53
190	14,994.89	15,616.63		14,596.06	14,531.29	59,738.87

	191	30,199.52	30,144.69	26,804.23	26,529.11	113,677.55
	193	3,696.62	3,235.49	3,200.40	3,092.83	13,225.34
	194	6,417.29	11,029.68	15,755.26	8,240.19	41,442.42
	195	1,671.79	1,640.48	1,613.41	1,582.82	6,508.50
	196	2,635.80	2,574.92	2,423.73	2,557.41	10,191.86
TOTAL		137,000.05	143,911.17	142,003.01	131,550.66	554,464.89
STATE:	PUERTO RICO					
ZIP CODE		QUARTER 1	QUARTER 2	QUARTER 3	QUARTER 4	TOTAL GRAMS
	6	5,153.95	5,120.80	4,074.32	4,751.72	19,100.79
	7	5,054.50	5,084.34	4,277.65	4,665.52	19,082.01
	9	3,375.93	3,269.84	3,277.58	3,119.56	13,042.91
TOTAL		13,584.38	13,474.98	11,629.55	12,536.80	51,225.71
STATE:	RHODE ISLAND					
ZIP CODE		QUARTER 1	QUARTER 2	QUARTER 3	QUARTER 4	TOTAL GRAMS
	28	4,857.47	4,671.41	4,158.87	4,380.09	18,067.84
	29	3,149.74	2,702.36	2,715.05	2,481.75	11,048.90
TOTAL		8,007.21	7,373.77	6,873.92	6,861.84	29,116.74
STATE:	SOUTH CAROLINA					
ZIP CODE		QUARTER 1	QUARTER 2	QUARTER 3	QUARTER 4	TOTAL GRAMS
	290	2,985.41	2,834.10	2,670.11	2,803.59	11,293.21
	291	3,480.59	3,217.44	3,188.20	3,100.16	12,986.39
	292	3,691.28	3,559.99	3,564.18	3,503.07	14,318.52
	293	3,579.41	3,752.27	3,144.28	3,833.95	14,309.91
	294	42,395.43	33,184.63	38,047.10	49,310.49	162,937.65
	295	8,902.40	9,031.58	9,847.89	9,537.18	37,319.05
	296	7,912.62	7,287.29	6,764.53	7,238.89	29,203.33
	297	1,859.63	1,692.21	1,802.37	1,747.39	7,101.60
	298	2,338.74	2,282.04	2,431.92	2,328.63	9,381.33
	299	1,389.81	1,451.66	1,110.92	1,232.62	5,185.01
TOTAL		78,535.32	68,293.21	72,571.50	84,635.97	304,036

STATE: SOUTH DAKOTA

ZIP CODE	QUARTER 1	QUARTER 2	QUARTER 3	QUARTER 4	TOTAL GRAMS
570	911.7	1,227.33	1,007.82	1,009.05	4,155.90
571	2,215.46	2,092.65	1,831.10	2,253.07	8,392.28
572	529.67	458.04	506.84	499.47	1,994.02
573	550.25	626.11	568.06	527.1	2,271.52
574	642.55	513.85	543.03	608.51	2,307.94
575	377.91	334.09	283.62	303.73	1,299.35
576	273.79	232.42	234.27	243.11	983.59
577	1,748.87	1,592.02	1,760.97	1,378.70	6,480.56
TOTAL	7,250.20	7,076.51	6,735.71	6,822.74	27,885.16

STATE: TENNESSEE

ZIP CODE	QUARTER 1	QUARTER 2	QUARTER 3	QUARTER 4	TOTAL GRAMS
370	5,210.26	5,068.46	4,893.34	4,839.50	20,011.56
371	51,881.42	46,969.21	45,136.53	33,433.89	177,421.05
372	2,620.83	2,486.06	2,574.22	2,193.29	9,874.40
373	3,537.29	3,751.89	3,541.53	3,717.88	14,548.59
374	1,463.07	1,496.69	1,295.30	1,604.17	5,859.23
376	2,266.36	2,715.60	2,531.61	2,636.62	10,150.19
377	2,726.36	2,829.47	2,580.31	2,816.17	10,952.31
378	4,328.99	4,171.50	4,131.63	4,375.97	17,008.09
379	2,487.23	2,611.39	2,329.71	2,572.90	10,001.23
380	2,705.61	2,736.85	2,551.79	2,386.48	10,380.73
381	5,512.49	4,992.02	5,133.52	5,350.35	20,988.38
382	869.92	1,004.10	863.76	866.35	3,604.13
383	3,925.14	3,683.86	3,755.37	3,553.25	14,917.62
384	1,240.69	1,012.68	1,085.34	1,042.61	4,381.32
385	2,745.67	2,872.66	3,004.35	2,762.63	11,385.31
TOTAL	93,521.33	88,402.44	85,408.31	74,152.06	341,484.14

STATE: TEXAS

ZIP CODE	QUARTER 1	QUARTER 2	QUARTER 3	QUARTER 4	TOTAL GRAMS

750	64,478.68	69,663.32	66,539.25	65,850.21	266,531.46
751	32,321.07	33,252.23	31,161.21	30,844.29	127,578.80
752	39,190.30	39,028.19	37,114.83	37,224.18	152,557.50
753	509.89	581.26	515.5	561.73	2,168.38
754	21,681.99	21,187.31	20,838.50	19,568.85	83,276.65
755	8,946.84	8,912.23	9,045.55	8,885.99	35,790.61
756	24,762.61	25,592.98	23,440.42	24,230.82	98,026.83
757	25,579.02	26,665.45	24,861.18	23,329.68	100,435.33
758	4,906.72	5,015.82	4,620.36	4,794.19	19,337.09
759	19,223.39	18,851.07	18,865.92	18,471.44	75,411.82
760	49,200.89	51,867.07	48,410.95	48,977.34	198,456.25
761	40,275.58	39,252.59	38,632.02	38,232.84	156,393.03
762	17,622.08	18,139.59	17,871.09	17,416.31	71,049.07
763	11,621.55	12,165.04	12,088.86	11,817.16	47,692.61
764	6,685.65	7,333.41	7,230.89	7,279.35	28,529.30
765	17,348.50	17,303.69	16,389.26	16,226.88	67,268.33
766	5,840.07	5,680.14	5,207.56	5,300.66	22,028.43
767	9,162.90	9,376.04	8,429.41	8,827.69	35,796.04
768	7,696.67	8,426.01	8,135.38	7,908.82	32,166.88
769	7,376.21	7,768.50	7,066.98	7,002.70	29,214.39
770	91,240.52	91,893.43	85,391.02	87,480	356,004.97
772	2.82	23.4	6.15	0	32.37
773	45,813.57	45,338.67	44,197.74	44,372.11	179,722.09
774	33,842.02	34,438.30	33,524.90	35,218.73	137,023.95
775	48,644.48	50,264.47	47,412.71	48,484.61	194,806.27
776	16,260.67	17,457.66	16,176.90	16,209.19	66,104.42
777	9,277.49	9,153.70	9,200.32	9,031.93	36,663.44
778	12,631.07	12,175.74	12,507.10	11,982.04	49,295.95
779	5,444.36	5,677.73	5,564.81	5,730.60	22,417.50
780	15,777.25	15,864.59	16,065.88	15,535.89	63,243.61
781	13,879.43	13,942.06	13,935.72	13,800.93	55,558.14
782	77,855.15	77,217.98	75,551.18	74,897.30	305,521.61
783	14,059.50	14,454.11	13,194.79	12,748.45	54,456.85
784	17,964.41	18,205.50	17,521.18	17,350.21	71,041.30
785	32,124.97	31,213.64	31,663.61	31,991.50	126,993.72
786	22,975.65	22,697.05	22,464.75	22,221.48	90,358.93
787	20,140.76	18,817.29	17,772.70	17,605.44	74,336.19

	788	5,843.50	5,681.24	5,651.23	5,382.87	22,558.84
	789	1,892.95	1,792.76	1,852.17	1,954.66	7,492.54
	790	8,083.69	8,265.34	8,359.18	8,150.56	32,858.77
	791	14,778.46	15,612.88	15,547.72	15,459.88	61,398.94
	792	1,467.83	1,363.07	1,324.03	1,276.88	5,431.81
	793	6,166.67	5,825.48	6,155.66	5,671.80	23,819.61
	794	13,897.34	14,785.79	13,646.50	14,338.36	56,667.99
	795	3,416.65	3,381.45	3,060.05	3,518.24	13,376.39
	796	9,311.47	9,651.31	9,332.18	9,744.90	38,039.86
	797	23,069.44	23,360.57	23,783.14	24,285.85	94,499
	798	1,238.52	1,268.70	1,186.74	1,353.69	5,047.65
	799	28,403.85	28,857.65	28,557.78	27,258.23	113,077.51
TOTAL		1,009,935.10	1,024,743.50	987,072.96	985,807.46	4,007,559.02

STATE: UTAH

ZIP CODE		QUARTER 1	QUARTER 2	QUARTER 3	QUARTER 4	TOTAL GRAMS
	840	7,191.31	7,205.94	6,836.25	6,944.59	28,178.09
	841	3,358.34	3,471.10	3,621.58	3,577.64	14,028.66
	843	829.49	866.44	773.43	721.21	3,190.57
	844	910.6	918.84	921.72	970.79	3,721.95
	845	569.83	514.86	467.44	478.67	2,030.80
	846	1,930.39	1,810.98	2,038.06	1,787.76	7,567.19
	847	1,665.52	1,794.99	1,719.87	1,474.01	6,654.39
TOTAL		16,455.48	16,583.15	16,378.35	15,954.67	65,371.65

STATE: VERMONT

ZIP CODE		QUARTER 1	QUARTER 2	QUARTER 3	QUARTER 4	TOTAL GRAMS
	50	302.79	289.07	241.74	247.46	1,081.06
	51	150.65	87.1	117.68	44.57	400
	52	238.03	233.61	177.9	214	863.54
	53	184.35	199.82	175.25	181.4	740.82
	54	1,258.42	1,218.47	1,172.44	1,139.99	4,789.32
	56	560.68	551.38	521	475.42	2,108.48
	57	385.19	493.76	342.92	396.88	1,618.75
	58	485.44	385.53	282.11	360.86	1,513.94

TOTAL	3,565.55	3,458.74	3,031.04	3,060.58	13,115.91

STATE: VIRGIN ISLANDS

ZIP CODE	QUARTER 1	QUARTER 2	QUARTER 3	QUARTER 4	TOTAL GRAMS
8	950.85	652.13	565.79	830.26	2,999.03
TOTAL	950.85	652.13	565.79	830.26	2,999.03

STATE: VIRGINIA

ZIP CODE	QUARTER 1	QUARTER 2	QUARTER 3	QUARTER 4	TOTAL GRAMS
201	6,001.67	5,713.16	5,164.22	5,423.98	22,303.03
220	4,002.45	3,962.17	3,741.03	3,598.69	15,304.34
221	3,277.93	3,345.37	2,804.46	2,827.82	12,255.58
222	1,322.43	1,198.66	1,086.10	1,145.71	4,752.90
223	2,211.05	2,142.26	1,844.78	1,980.65	8,178.74
224	2,394.71	2,220.78	2,027.15	2,280.45	8,923.09
225	1,317.48	1,212.98	1,181.65	1,142.45	4,854.56
226	1,203.12	1,276.07	923.49	1,231.93	4,634.61
227	352.31	376.45	309.95	354.81	1,393.52
228	1,034.11	1,078.32	974.73	1,168.59	4,255.75
229	1,599.47	1,471.87	1,245.25	1,327.91	5,644.50
230	1,008.48	1,097.05	1,192.64	1,059.84	4,358.01
231	2,221.30	2,137.11	1,689.45	1,834.42	7,882.28
232	4,871.96	4,410.78	4,237.37	4,615.59	18,135.70
233	1,609.96	1,496.41	1,524.67	1,308.81	5,939.85
234	3,983.35	3,902.43	3,776.90	3,405.72	15,068.40
235	1,503.09	1,551.63	1,524.40	1,467.47	6,046.59
236	2,876.27	3,247.50	2,881.52	2,488.35	11,493.64
237	1,039.37	1,000.29	872.99	798.46	3,711.11
238	2,626.25	2,457.18	2,157.72	1,943.02	9,184.17
239	823.73	682.92	485.85	539.39	2,531.89
240	1,861.41	1,947.30	1,784.33	1,563.97	7,157.01
241	1,727.85	1,626.62	1,373.36	1,320.58	6,048.41
242	1,683.35	1,735.30	1,422.49	1,557.16	6,398.30
243	1,670.07	1,326.33	1,134.26	1,179.24	5,309.90

	244	675.79	638.25	632.39	527.13	2,473.56
	245	2,296.19	2,014.67	1,736.44	1,855.29	7,902.59
	246	2,093.81	1,762.75	1,673.52	1,904.23	7,434.31
TOTAL		59,288.96	57,032.61	51,403.11	51,851.66	219,576.34

STATE: WASHINGTON

ZIP CODE		QUARTER 1	QUARTER 2	QUARTER 3	QUARTER 4	TOTAL GRAMS
	980	12,032.25	11,700.46	11,382.08	11,093.76	46,208.55
	981	7,681.65	7,162.01	6,936.36	6,843.51	28,623.53
	982	8,203.77	8,370.82	8,085.45	7,941.25	32,601.29
	983	6,833.41	6,884.66	6,580.23	6,548.64	26,846.94
	984	4,080.94	4,382.17	3,968.91	4,109.82	16,541.84
	985	4,237.98	3,960.19	3,890.36	3,775.50	15,864.03
	986	4,614.31	4,769.20	4,748.93	4,523.80	18,656.24
	988	1,671.18	1,740.63	1,354.44	1,526.75	6,293
	989	2,380.75	2,309.82	2,288.79	2,128.50	9,107.86
	990	1,194.69	1,310.55	1,110.37	1,144.62	4,760.23
	991	1,200.13	1,155.35	1,216.05	1,209.07	4,780.60
	992	5,960.09	6,068.32	5,340.39	5,488.85	22,857.65
	993	2,708.63	2,603.72	2,443.26	2,441.44	10,197.05
	994	571.68	641.66	640.19	557.13	2,410.66
TOTAL		63,371.46	63,059.56	59,985.81	59,332.64	245,749.47

STATE: WEST VIRGINIA

ZIP CODE		QUARTER 1	QUARTER 2	QUARTER 3	QUARTER 4	TOTAL GRAMS
	247	829.66	922.6	902.21	917.43	3,571.90
	248	1,646.52	1,693.96	1,364.66	2,095.09	6,800.23
	249	426.39	456.5	472.92	344.05	1,699.86
	250	1,071.74	1,169.14	1,159.49	1,269.92	4,670.29
	251	779.02	904.76	679.42	880.17	3,243.37
	252	1,098.26	1,045.76	1,077.80	1,284.53	4,506.35
	253	2,523.46	2,015.75	2,211.54	2,089.48	8,840.23
	254	1,542.23	1,495.62	1,356.66	1,364.33	5,758.84
	255	2,393.92	2,404.81	2,191.11	2,535.80	9,525.64
	256	3,713.41	3,585.16	3,708.55	4,092.71	15,099.83

	257	1,459.17	1,345.22	1,254.64	1,286.32	5,345.35
	258	2,174.51	1,853.53	1,993.11	1,833.20	7,854.35
	259	632.99	702.06	657.96	654.18	2,647.19
	260	1,529.74	1,651.11	1,417.40	1,345.65	5,943.90
	261	1,624.33	1,719.89	1,392.18	1,628.20	6,364.60
	262	1,593.16	1,797.07	1,907.12	1,779.50	7,076.85
	263	1,351.52	1,353.15	1,276.54	1,304.88	5,286.09
	264	436.68	517.35	437.69	451.97	1,843.69
	265	2,375.60	2,547.44	2,816.25	2,917.33	10,656.62
	266	962.59	1,033.22	1,078.53	1,034.33	4,108.67
	267	600.76	632.6	633.55	615.32	2,482.23
	268	319.8	286.21	271.13	257.87	1,135.01
TOTAL		31,085.46	31,132.91	30,260.46	31,982.26	124,461.09

STATE:	WISCONSIN					
ZIP CODE	QUARTER 1		QUARTER 2	QUARTER 3	QUARTER 4	TOTAL GRAMS
	530	5,889.73	4,812.05	4,764.63	4,877.16	20,343.57
	531	5,702.19	5,434.90	4,970.32	5,694.41	21,801.82
	532	9,914.30	9,653.27	9,136.12	8,991.22	37,694.91
	534	1,593.24	1,437.29	1,347.59	1,413.44	5,791.56
	535	3,794.93	3,696.92	3,586.86	3,407.43	14,486.14
	537	2,153.22	2,048.01	2,088.97	2,199.75	8,489.95
	538	434.89	577.51	427.59	467.9	1,907.89
	539	2,065.90	1,699.93	1,566.10	1,572.47	6,904.40
	540	811.53	768.89	863.38	1,011.49	3,455.29
	541	1,565.21	1,497.40	1,397.11	1,392.04	5,851.76
	542	1,134.71	1,005.85	994.66	893.52	4,028.74
	543	2,142.79	2,203.40	1,974.10	1,885.63	8,205.92
	544	3,351.09	3,167.39	3,243.12	3,236.61	12,998.21
	545	1,073.58	1,049.80	1,020.52	899.99	4,043.89
	546	1,400.17	1,406.60	1,505.15	1,326.43	5,638.35
	547	1,774.07	1,605.44	1,414.05	1,430.89	6,224.45
	548	1,827.01	1,832.60	1,773.13	1,740.57	7,173.31
	549	4,071.02	3,868.16	3,845.30	3,729.57	15,514.05
TOTAL		50,699.58	47,765.41	45,918.70	46,170.52	190,554.21

STATE: WYOMING

ZIP CODE	QUARTER 1	QUARTER 2	QUARTER 3	QUARTER 4	TOTAL GRAMS
820	1,139.37	995.42	902.13	916.8	3,953.72
822	137.21	221.01	152.5	181.41	692.13
823	108.52	17.68	55.25	48.62	230.07
824	610.14	590.07	651.02	636.28	2,487.51
825	416.82	400.03	408.87	359.14	1,584.86
826	585.3	587.33	671.78	638.25	2,482.66
827	389.71	272.75	302.77	341.81	1,307.04
828	285.96	248.94	396.1	268.46	1,199.46
829	552.53	459.7	444.41	643.32	2,099.96
830	83.54	139.37	77.35	158.24	458.5
831	57.46	98.8	65.65	48.62	270.53
TOTAL	4,366.56	4,031.10	4,127.83	4,240.95	16,766.44

DRUG CODE: 9056 DRUG NAME: ETORPHINE

STATE: WYOMING

ZIP CODE	QUARTER 1	QUARTER 2	QUARTER 3	QUARTER 4	TOTAL GRAMS
820	28	0	0	29	57
TOTAL	28	0	0	29	57

DRUG CODE: 9064 DRUG NAME: BUPRENORPHINE

STATE: ALABAMA

ZIP CODE	QUARTER 1	QUARTER 2	QUARTER 3	QUARTER 4	TOTAL GRAMS
350	3,590.18	3,842.02	3,726.56	3,801.61	14,960.37
351	2,096.48	2,163.26	2,186.44	2,308.21	8,754.39
352	3,008.61	3,090.22	3,186.79	3,188.10	12,473.72
354	339.53	371.63	408.04	431.07	1,550.27
355	1,598.66	1,711.08	1,757.30	1,846.13	6,913.17
356	2,748.27	2,849.65	2,932.20	2,935.10	11,465.22
357	828.11	851.29	819.45	861.35	3,360.20
358	1,125.79	1,194.48	1,190.28	1,211.59	4,722.14

	359	2,373.75	2,628.52	2,781.55	2,982.75	10,766.57
	360	454.1	531.31	496.02	580.7	2,062.13
	361	321.54	345.49	317.34	356.63	1,341
	362	871.59	907.42	940.08	958.82	3,677.91
	363	666.87	685.02	639	732.59	2,723.48
	364	348.48	373.87	349.8	369.08	1,441.23
	365	1,580.38	1,725.77	1,816.30	1,810.42	6,932.87
	366	1,059.32	1,121.95	1,124.14	1,284.65	4,590.06
	367	189.59	204.02	202.61	229.15	825.37
	368	502.59	533.69	513.42	530.55	2,080.25
	369	31.31	29.86	26.68	26.9	114.75
TOTAL		23,735.15	25,160.55	25,414	26,445.40	100,755.10

STATE: ALASKA

ZIP CODE		QUARTER 1	QUARTER 2	QUARTER 3	QUARTER 4	TOTAL GRAMS
	995	982.76	1,078.78	1,089.94	1,127.90	4,279.38
	996	645.28	664.79	698.87	680.02	2,688.96
	997	174.46	192.36	212.05	236.12	814.99
	998	226.98	260.68	273.64	282.72	1,044.02
	999	148.91	154.95	156.9	170.07	630.83
TOTAL		2,178.39	2,351.56	2,431.40	2,496.83	9,458.18

STATE: AMERICAN SAMOA

ZIP CODE		QUARTER 1	QUARTER 2	QUARTER 3	QUARTER 4	TOTAL GRAMS
	967	0	0	0	0	0
TOTAL		0	0	0	0	0

STATE: ARIZONA

ZIP CODE		QUARTER 1	QUARTER 2	QUARTER 3	QUARTER 4	TOTAL GRAMS
	850	2,082.32	2,180.18	2,338.50	2,505.57	9,106.57
	851	547.64	564.59	610.7	623.33	2,346.26
	852	2,595.04	2,716.44	2,721.24	2,705.33	10,738.05
	853	1,353.54	1,437.59	1,484.45	1,550.12	5,825.70
	855	86	92.35	88.13	104.33	370.81

	856	101	93.16	93.62	110.01	397.79
	857	1,205.72	1,273.08	1,291.99	1,337.67	5,108.46
	859	120.32	140.16	135.96	156.5	552.94
	860	103.55	109.87	116.76	115.11	445.29
	863	544.1	521.07	524.33	559.82	2,149.32
	864	167.02	150.88	145.14	141.62	604.66
	865	11.16	9	16.62	17.64	54.42
TOTAL		8,917.41	9,288.37	9,567.44	9,927.05	37,700.27

STATE: ARKANSAS

ZIP CODE		QUARTER 1	QUARTER 2	QUARTER 3	QUARTER 4	TOTAL GRAMS
	716	93.28	103.97	105.66	114.9	417.81
	717	118.91	107.03	145.45	162.46	533.85
	718	174.81	204.83	201.05	205.21	785.9
	719	887.88	910.48	877.99	923.76	3,600.11
	720	595.46	648.65	688.57	714.07	2,646.75
	721	1,059.22	1,056.68	1,069.42	1,096.39	4,281.71
	722	593.67	614.78	612.88	646.75	2,468.08
	723	141.7	154.46	155.5	153.89	605.55
	724	688.37	703.64	737.47	772.93	2,902.41
	725	172.04	201.45	207.44	215.49	796.42
	726	225.96	233.33	264.74	248.3	972.33
	727	680.61	749.11	826.9	844.22	3,100.84
	728	120.34	117.28	131.71	137.09	506.42
	729	324.6	374.15	346.13	393.35	1,438.23
TOTAL		5,876.85	6,179.84	6,370.91	6,628.81	25,056.41

STATE: CALIFORNIA

ZIP CODE		QUARTER 1	QUARTER 2	QUARTER 3	QUARTER 4	TOTAL GRAMS
	900	888.59	974.69	1,015.17	979.87	3,858.32
	902	797.75	812.14	845.81	861.63	3,317.33
	903	27.52	31.63	25.08	25.42	109.65
	904	199.95	201.34	216.46	231.17	848.92
	905	162.08	172.46	187.53	196.17	718.24
	906	270.61	277.63	279.64	282.46	1,110.34

907	599.29	634.98	690.11	657.4	2,581.78
908	291.3	302.28	322.65	347.22	1,263.45
910	108.66	107.59	110.47	117.92	444.64
911	137.54	161.26	158.89	168.39	626.08
912	223.98	239.17	255.47	265.9	984.52
913	1,098.09	1,150.85	1,185.68	1,270.97	4,705.59
914	456.76	475.47	459.07	494.51	1,885.81
915	189.93	195.66	229.34	218.48	833.41
916	114.89	123.14	118.92	124.82	481.77
917	858.43	860.94	911.8	827.66	3,458.83
918	7.91	8.93	6.67	42.7	66.21
919	223.4	222.63	238.79	230.85	915.67
920	1,319.78	1,357.54	1,409.58	1,390.84	5,477.74
921	1,169.23	1,132.86	1,196.62	1,209.33	4,708.04
922	338.09	327.76	324.16	353	1,343.01
923	397.05	445.42	455.39	483.88	1,781.74
924	51.29	51.9	59.6	55.5	218.29
925	1,302.99	1,363.35	1,406.09	1,376.55	5,448.98
926	1,824.73	1,792.88	1,825.53	1,801.09	7,244.23
927	285.54	268.12	286.76	298.98	1,139.40
928	772.02	816.66	820.15	826.97	3,235.80
930	633.82	639.01	678.8	682.18	2,633.81
931	428.19	448.55	499.6	455.01	1,831.35
932	311.05	332.45	355.86	373.52	1,372.88
933	321.11	291.52	234.61	266.65	1,113.89
934	803.76	959.97	1,038.43	925.06	3,727.22
935	390.18	405.32	452.9	435.72	1,684.12
936	201.84	181.69	177.71	193.26	754.5
937	306.73	315.92	325.98	313.64	1,262.27
939	605.2	637.09	672.94	662.49	2,577.72
940	645.96	691.03	683.11	667.86	2,687.96
941	962.68	1,025.64	1,057.64	1,209.09	4,255.05
943	99.06	106.61	115.48	112.53	433.68
944	116.06	126.34	124.28	138.45	505.13
945	2,502.84	2,585.55	2,778.73	2,796.08	10,663.20
946	330.42	328.59	378.39	395.24	1,432.64
947	106.47	120.3	112.43	115.92	455.12

	948	116.23	112.68	115.17	142.56	486.64
	949	814.79	831.89	896.05	913.68	3,456.41
	950	948.95	948.31	1,092.95	1,080.44	4,070.65
	951	584.17	608.18	643.56	682.27	2,518.18
	952	334.59	323.35	353.79	346.58	1,358.31
	953	983.36	956.29	1,022.94	1,038.77	4,001.36
	954	2,041.69	2,102.16	2,210.73	2,257.73	8,612.31
	955	1,191.25	1,225.70	1,247.48	1,324.02	4,988.45
	956	1,780	1,892.22	2,102.31	2,174.63	7,949.16
	957	268.96	272.54	287	306.03	1,134.53
	958	2,063.31	2,049.25	2,083.84	2,128.46	8,324.86
	959	1,226.82	1,271.57	1,387.07	1,358.43	5,243.89
	960	828.5	839.18	972.12	1,034.72	3,674.52
	961	118.49	136.45	154.38	140.92	550.24
TOTAL		36,183.88	37,274.63	39,297.71	39,811.62	152,567.84

STATE: COLORADO

ZIP CODE		QUARTER 1	QUARTER 2	QUARTER 3	QUARTER 4	TOTAL GRAMS
	800	1,167.88	1,205.95	1,262.95	1,234.04	4,870.82
	801	771.77	793.61	838.22	847.46	3,251.06
	802	1,548.32	1,597.83	1,688.52	1,759.52	6,594.19
	803	130.95	141.42	135.89	148.63	556.89
	804	133.56	120.27	143.26	141.21	538.3
	805	895.4	949.36	998.61	985.48	3,828.85
	806	404.28	440.61	450.95	482.38	1,778.22
	807	50.61	54.08	71.95	62.05	238.69
	808	71.75	86.18	92.13	112.8	362.86
	809	666.85	688.25	731.51	791.07	2,877.68
	810	504.7	470.8	468.27	506.86	1,950.63
	811	80.37	83.27	78.38	75.9	317.92
	812	104.34	101.65	97.97	108.25	412.21
	813	106.08	138.91	147.96	111.98	504.93
	814	47.47	57.89	55.95	49.59	210.9
	815	226.26	193.49	252.05	194.41	866.21
	816	118.99	122.09	103.21	108.64	452.93
TOTAL		7,029.58	7,245.66	7,617.78	7,720.27	29,613.29

STATE: CONNECTICUT

ZIP CODE	QUARTER 1	QUARTER 2	QUARTER 3	QUARTER 4	TOTAL GRAMS
60	2,925.17	3,064.05	3,179.72	3,215.22	12,384.16
61	1,236.08	1,234.83	1,283.57	1,411.31	5,165.79
62	742.87	785.76	781.23	786.5	3,096.36
63	1,326.77	1,361.95	1,388.46	1,424.21	5,501.39
64	3,284.84	3,422.05	3,493.47	3,595.60	13,795.96
65	2,155.96	2,346.40	2,289.13	2,266.26	9,057.75
66	295.3	308.31	302.28	320.33	1,226.22
67	1,377.86	1,427.09	1,453.54	1,440.02	5,698.51
68	619.56	643.83	668.22	717.49	2,649.10
69	180.05	177.93	183.01	176.54	717.53
TOTAL	14,144.46	14,772.20	15,022.63	15,353.48	59,292.77

STATE: DELAWARE

ZIP CODE	QUARTER 1	QUARTER 2	QUARTER 3	QUARTER 4	TOTAL GRAMS
197	1,056.27	1,039.01	1,118.95	1,125.69	4,339.92
198	859.87	976.49	924.51	969	3,729.87
199	1,467.50	1,618.31	1,556.06	1,804.25	6,446.12
TOTAL	3,383.64	3,633.81	3,599.52	3,898.94	14,515.91

STATE: DISTRICT OF COLUMBIA

ZIP CODE	QUARTER 1	QUARTER 2	QUARTER 3	QUARTER 4	TOTAL GRAMS
200	2,102.20	2,244.83	2,439.47	2,581.05	9,367.55
204	24.36	20.22	23.6	18.6	86.78
TOTAL	2,126.56	2,265.05	2,463.07	2,599.65	9,454.33

STATE: FLORIDA

ZIP CODE	QUARTER 1	QUARTER 2	QUARTER 3	QUARTER 4	TOTAL GRAMS
320	2,938.69	2,962.34	3,025.39	3,083.04	12,009.46
321	2,191.94	2,255.64	2,268.02	2,354.40	9,070
322	3,623.26	3,719.08	3,854.84	3,892.53	15,089.71

	323	660.3	682.59	711.28	695.85	2,750.02
	324	787.04	862.35	819.25	863.27	3,331.91
	325	1,761.12	1,835.73	2,009.74	2,123.82	7,730.41
	326	2,264.34	2,421.03	2,557.30	2,679.70	9,922.37
	327	2,510.96	2,566.94	2,383.40	2,634.33	10,095.63
	328	1,072.02	1,067.88	1,139.39	1,044.17	4,323.46
	329	2,811.95	2,809.95	2,783.95	2,881.07	11,286.92
	330	1,455.96	1,518.36	1,516.84	1,577.96	6,069.12
	331	808.19	837.77	846.82	858.68	3,351.46
	333	1,078.52	1,132.48	1,194.80	1,271.79	4,677.59
	334	3,662.76	3,769.31	3,811.03	3,786.07	15,029.17
	335	1,357.27	1,489.20	1,372.42	1,495.99	5,714.88
	336	1,241.45	1,303.20	1,429.48	1,377.56	5,351.69
	337	2,329.36	2,393.44	2,525.01	2,426.90	9,674.71
	338	804.01	856.22	865.63	933.85	3,459.71
	339	2,370.95	2,440.90	1,971.09	2,524.41	9,307.35
	341	873.52	854.79	892.39	924.55	3,545.25
	342	3,333.35	3,422.85	3,155.27	3,497.20	13,408.67
	344	1,256.23	1,281.51	1,031.64	1,422.50	4,991.88
	346	1,815.96	1,868.71	1,957.55	2,038.90	7,681.12
	347	1,054.74	1,093.81	962.55	1,101.32	4,212.42
	349	1,298.53	1,390.52	1,441.70	1,437.05	5,567.80
TOTAL		45,362.42	46,836.60	46,526.78	48,926.91	187,652.71

STATE:	GEORGIA				
ZIP CODE	QUARTER 1	QUARTER 2	QUARTER 3	QUARTER 4	TOTAL GRAMS
300	2,341.68	2,422.71	2,440.27	2,517.73	9,722.39
301	2,124.29	2,198.30	2,256.07	2,234.54	8,813.20
302	1,322.68	1,391.55	1,418.59	1,430.65	5,563.47
303	691.53	703.86	688.11	687.37	2,770.87
304	739.76	759.55	737	769.86	3,006.17
305	867.12	922.66	945.97	954.59	3,690.34
306	742.37	797.53	843.82	865.75	3,249.47
307	456.45	541.25	535.48	571.99	2,105.17
308	126.52	129.46	125.6	123.15	504.73

	309	266.54	281.24	319.26	330.43	1,197.47
	310	994.95	1,061.15	940.57	1,090.71	4,087.38
	312	382.35	413.79	396.32	402.97	1,595.43
	313	744.46	745.7	795.05	748.76	3,033.97
	314	775.63	823.84	847.24	880.42	3,327.13
	315	1,497.87	1,653.83	1,648.88	1,762.60	6,563.18
	316	142.4	160.42	160.76	163.33	626.91
	317	334.62	375.28	432.9	421.88	1,564.68
	318	12.59	14.86	14.87	18.05	60.37
	319	255.17	289.14	271.44	287.73	1,103.48
	398	58.1	62.84	78.4	86.31	285.65
TOTAL		14,877.08	15,748.96	15,896.60	16,348.82	62,871.46

STATE: GUAM

ZIP CODE		QUARTER 1	QUARTER 2	QUARTER 3	QUARTER 4	TOTAL GRAMS
	969	0.82	1.99	0	1.68	4.49
TOTAL		0.82	1.99	0	1.68	4.49

STATE: HAWAII

ZIP CODE		QUARTER 1	QUARTER 2	QUARTER 3	QUARTER 4	TOTAL GRAMS
	967	1,404.90	1,493.29	1,537.86	1,579.39	6,015.44
	968	456.04	493.91	474.99	438.95	1,863.89
TOTAL		1,860.94	1,987.20	2,012.85	2,018.34	7,879.33

STATE: IDAHO

ZIP CODE		QUARTER 1	QUARTER 2	QUARTER 3	QUARTER 4	TOTAL GRAMS
	832	311.54	346.17	359.26	372.14	1,389.11
	833	183.16	189.12	212.5	225.84	810.62
	834	217.7	215.03	241.79	218.37	892.89
	835	271.15	288.49	310.75	357.57	1,227.96
	836	451.79	489.19	497.88	600.8	2,039.66
	837	527	565.53	609.55	659.69	2,361.77
	838	1,291.52	1,361.71	1,400.21	1,440.57	5,494.01
TOTAL		3,253.86	3,455.24	3,631.94	3,874.98	14,216.02

STATE: ILLINOIS

ZIP CODE	QUARTER 1	QUARTER 2	QUARTER 3	QUARTER 4	TOTAL GRAMS
600	2,056.66	2,059.97	2,086.86	2,172.58	8,376.07
601	1,082.04	1,133.27	1,137.76	1,107.65	4,460.72
602	28.96	32.39	34.49	32.59	128.43
603	115.81	166.3	179.36	232.28	693.75
604	1,857.06	1,872.27	1,922.98	1,973.91	7,626.22
605	895.95	926.66	935.19	984.86	3,742.66
606	1,584.50	1,796.30	1,875.74	1,868.72	7,125.26
607	105.92	111.9	94.69	102.98	415.49
608	74.76	79.43	71.46	72.09	297.74
609	147.47	162.68	176.39	179.72	666.26
610	175.19	183.53	186.55	187.66	732.93
611	542.69	579.71	596.21	608.13	2,326.74
612	185.92	173.51	182.16	173.77	715.36
613	243.39	242.5	265.87	273.36	1,025.12
614	54.22	61.03	54.73	62.57	232.55
615	182.05	192.39	191.36	207.13	772.93
616	327.13	382.42	382.38	386.73	1,478.66
617	115.17	139.73	135.62	116.93	507.45
618	727.19	865.14	806.88	864.77	3,263.98
619	81.33	83.78	94.74	88.41	348.26
620	312.54	372.33	379.49	366.49	1,430.85
622	267.27	281.47	290.03	288.58	1,127.35
623	77.8	100.2	134.08	148.54	460.62
624	144.5	165.44	151.54	144.1	605.58
625	274.56	274.14	302.38	325	1,176.08
626	146.74	175.76	161.17	170.85	654.52
627	313.16	357.66	338.12	336.49	1,345.43
628	300.33	323.27	355.38	360.11	1,339.09
629	1,014.92	1,084.76	1,145.32	1,206.79	4,451.79
TOTAL	13,435.23	14,379.94	14,668.93	15,043.79	57,527.89

STATE: INDIANA

RETAIL DRUG DISTRIBUTION BY ZIP CODE WITHIN STATE BY GRAMS WT

ZIP CODE	QUARTER 1	QUARTER 2	QUARTER 3	QUARTER 4	TOTAL GRAMS
460	1,757.70	2,070.04	2,248.45	2,317.27	8,393.46
461	2,025.71	2,184.69	2,170.19	2,129.13	8,509.72
462	3,168.30	3,349.31	3,410.18	3,710.12	13,637.91
463	2,048.64	2,162.23	2,188.26	2,222.63	8,621.76
464	150.52	151.34	147.33	183.29	632.48
465	483.75	511.74	522.6	569.2	2,087.29
466	163.36	187.96	224.08	243.86	819.26
467	109.21	133.77	149.52	193.7	586.2
468	248.13	263.21	368.62	422.19	1,302.15
469	621.48	676.61	710.58	797	2,805.67
470	619.97	699.06	729.57	673.55	2,722.15
471	1,636.25	1,874.52	1,856.67	1,786.90	7,154.34
472	1,083.18	1,293.64	1,284.24	1,394.20	5,055.26
473	1,554.61	1,756.46	1,783.03	1,853.72	6,947.82
474	814.69	957.59	1,136.73	1,267.41	4,176.42
475	188.4	181.59	181.35	193.9	745.24
476	51.55	69.16	78.05	89.23	287.99
477	337.4	442.26	455.66	562.27	1,797.59
478	374.24	418.45	443.98	464.91	1,701.58
479	366.64	391.53	448.04	454.19	1,660.40
TOTAL	17,803.73	19,775.16	20,537.13	21,528.67	79,644.69

STATE: IOWA

ZIP CODE	QUARTER 1	QUARTER 2	QUARTER 3	QUARTER 4	TOTAL GRAMS
500	52.04	66.76	79.56	91.07	289.43
501	64.17	70.37	71.55	68.33	274.42
502	43.09	71.56	71.3	80.91	266.86
503	333.21	288.14	321.81	346.17	1,289.33
504	130.96	125.35	128	159.79	544.1
505	35.72	39.79	42.97	54.2	172.68
506	19.29	25.49	23.48	19.42	87.68
507	31	28.67	31.29	44.01	134.97
508	8.43	9.54	10.58	8.22	36.77
510	91.81	109.81	96.86	81.79	380.27
511	114.92	130.83	136.67	148.8	531.22

	512	25.61	32.82	30.02	37.3	125.75
	513	15.7	16.63	19	23.53	74.86
	514	8.61	7.07	6.48	9.88	32.04
	515	61.85	61.3	63.56	71.3	258.01
	516	8.72	7.72	5.02	4.64	26.1
	520	66.46	79.06	63.29	59.36	268.17
	521	4.63	3.57	5.2	3.04	16.44
	522	98.91	96.01	105.21	107.47	407.6
	523	17.83	11.79	13.76	12.74	56.12
	524	32.53	40.31	51.15	47.59	171.58
	525	37.66	45.71	36.16	39.32	158.85
	526	49.42	42.28	46.08	49.62	187.4
	527	161.77	165.34	172.89	163.67	663.67
	528	71.09	62.28	67.21	75.42	276
TOTAL		1,585.43	1,638.20	1,699.10	1,807.59	6,730.32

STATE:	KANSAS					
ZIP CODE		QUARTER 1	QUARTER 2	QUARTER 3	QUARTER 4	TOTAL GRAMS
	660	526.04	537.35	584.88	539.6	2,187.87
	661	62.5	57.23	76.11	96.57	292.41
	662	642.47	613.23	627.25	618.65	2,501.60
	664	32.88	27.47	43.59	32.62	136.56
	665	45.47	48.31	44.78	47.31	185.87
	666	138.2	149.88	154.02	145.27	587.37
	667	148.07	156.19	148.59	159.3	612.15
	668	10.83	12.31	13.38	10.36	46.88
	669	4.64	3.08	1.64	2.91	12.27
	670	87.11	90.83	99.77	98.36	376.07
	671	53.7	48.04	49.77	56.13	207.64
	672	472.5	492.43	526.05	479.94	1,970.92
	673	22.95	28.95	29.09	30.9	111.89
	674	57.35	63.36	75.87	79.45	276.03
	675	56.49	56.05	58.08	53.19	223.81
	676	57.89	47.97	49.14	35.07	190.07
	677	18.28	16.34	12.1	6.66	53.38
	678	42.99	43.61	46.53	48.29	181.42

	679	13.64	14.51	20.09	21.07	69.31
TOTAL		2,494	2,507.14	2,660.73	2,561.65	10,223.52

STATE:	KENTUCKY					
ZIP CODE		QUARTER 1	QUARTER 2	QUARTER 3	QUARTER 4	TOTAL GRAMS
	400	633.5	676.67	660.04	679.56	2,649.77
	401	150.6	164.21	163.43	204.26	682.5
	402	2,569.75	2,825.60	2,757.71	2,970.40	11,123.46
	403	3,967.59	4,047.72	3,933.18	4,080.91	16,029.40
	404	2,205.57	2,082.21	2,167.10	2,223.18	8,678.06
	405	2,420.38	2,495.06	2,581.75	2,477.94	9,975.13
	406	421.8	490.91	534.05	496.97	1,943.73
	407	1,881.45	1,993.23	1,951.98	1,789.16	7,615.82
	408	343.38	356.34	348.45	350.16	1,398.33
	409	1,497.38	1,529.47	1,553.31	1,474.05	6,054.21
	410	1,943.60	1,952.36	1,992.98	1,988.83	7,877.77
	411	1,720.43	1,727.58	1,683.50	1,633.02	6,764.53
	412	1,128.82	1,016.63	989.11	928.35	4,062.91
	413	801.22	881.43	879.9	847.98	3,410.53
	414	605.43	664.73	635.05	661.52	2,566.73
	415	2,513.85	2,509.59	2,521.09	2,609.73	10,154.26
	416	1,743.96	1,898.41	1,962.99	1,905.59	7,510.95
	417	1,158.63	1,210.07	1,200.07	1,206.15	4,774.92
	418	428.71	426.03	446.37	427.39	1,728.50
	420	948.76	1,094.63	1,158.88	1,245.17	4,447.44
	421	880.69	900.52	876.63	1,041.86	3,699.70
	422	243.13	254.38	242.75	243.8	984.06
	423	633.68	634.34	624.89	603.24	2,496.15
	424	313.02	334.9	338.22	338.34	1,324.48
	425	638.01	659.06	559.11	680.63	2,536.81
	426	887.5	788.75	775.33	757.23	3,208.81
	427	1,067.49	1,135.39	1,107.50	1,073.27	4,383.65
TOTAL		33,748.33	34,750.22	34,645.37	34,938.69	138,082.61

STATE: LOUISIANA

ZIP CODE	QUARTER 1	QUARTER 2	QUARTER 3	QUARTER 4	TOTAL GRAMS
700	3,747.24	3,841.59	3,791.32	3,855.43	15,235.58
701	840.18	912.17	941.37	923.26	3,616.98
703	1,000.15	1,026.18	1,031.13	1,078.13	4,135.59
704	3,161.41	3,367.36	3,392.93	3,437.19	13,358.89
705	2,375.89	2,498.72	2,496.86	2,454.58	9,826.05
706	1,103.66	1,163.91	1,200.92	1,228.32	4,696.81
707	1,695.56	1,710.52	1,704.56	1,743.44	6,854.08
708	790.98	812.35	903.87	928	3,435.20
710	397.68	421.28	453.31	277.29	1,549.56
711	1,170.92	1,254.93	1,756.59	1,619.34	5,801.78
712	1,025.20	1,089.61	1,125.05	1,192.43	4,432.29
713	422.05	494.83	555.3	530.19	2,002.37
714	323.75	360.09	329.78	355.2	1,368.82
TOTAL	18,054.67	18,953.54	19,682.99	19,622.80	76,314

STATE: MAINE

ZIP CODE	QUARTER 1	QUARTER 2	QUARTER 3	QUARTER 4	TOTAL GRAMS
39	60.31	50.43	65.4	64.07	240.21
40	2,080.86	2,254.33	2,336.51	2,369.53	9,041.23
41	954.3	1,038.21	1,019.96	1,010.78	4,023.25
42	1,268.94	1,367.69	1,424.18	1,497.23	5,558.04
43	684.32	771.05	798.29	797.38	3,051.04
44	1,019.93	1,035.60	1,076.11	1,065.52	4,197.16
45	331.7	373.59	352.04	383.19	1,440.52
46	498.37	585.37	557.02	643.78	2,284.54
47	404.79	404.17	353.34	476.24	1,638.54
48	660.96	698.3	748.38	715.17	2,822.81
49	1,007.41	1,094.34	1,142.41	1,178.88	4,423.04
TOTAL	8,971.89	9,673.08	9,873.64	10,201.77	38,720.38

STATE: MARYLAND

ZIP CODE	QUARTER 1	QUARTER 2	QUARTER 3	QUARTER 4	TOTAL GRAMS
206	1,469.67	1,530.06	1,642.12	1,721.58	6,363.43
207	1,012.23	1,001.59	945.43	1,024.04	3,983.29

	208	1,070.75	1,107.72	1,082.61	993.33	4,254.41
	209	371.2	426.75	461.22	425.26	1,684.43
	210	3,112.32	3,254.68	3,242.65	3,368.21	12,977.86
	211	1,541.36	1,643.58	1,458.87	1,607.15	6,250.96
	212	9,853.75	10,290.05	10,700.69	11,278.59	42,123.08
	214	283.29	368.55	445.83	504.87	1,602.54
	215	988.1	1,060.60	1,020.37	1,179.93	4,249
	216	743.62	751.98	747.91	752.22	2,995.73
	217	1,800.43	2,042.64	2,047.48	2,120.25	8,010.80
	218	783.49	666.73	722.2	745.74	2,918.16
	219	1,094.86	1,121.67	1,155.78	1,276.76	4,649.07
TOTAL		24,125.07	25,266.60	25,673.16	26,997.93	102,062.76

STATE:	MASSACHUSETTS					
ZIP CODE		QUARTER 1	QUARTER 2	QUARTER 3	QUARTER 4	TOTAL GRAMS
	10	3,355.85	3,502.12	3,457.57	3,673.98	13,989.52
	11	1,250.64	1,288.46	1,226.45	1,281.12	5,046.67
	12	1,145.41	1,228.69	1,201.43	1,250.32	4,825.85
	13	852.87	918.88	913.18	958.57	3,643.50
	14	1,030.71	1,086.07	1,052.92	1,146.57	4,316.27
	15	1,404.40	1,414.52	1,389.58	1,434.63	5,643.13
	16	2,316.50	2,438.45	2,291.32	2,165.17	9,211.44
	17	1,376.40	1,498.34	1,265.70	1,417.62	5,558.06
	18	3,250.59	3,462.23	3,359.08	3,406.36	13,478.26
	19	2,638.99	2,904.16	2,700.20	2,927.67	11,171.02
	20	1,572.51	1,665.54	1,558.46	1,632.85	6,429.36
	21	8,408.79	9,024.98	8,648.17	8,982.41	35,064.35
	22	89.08	84.99	79.33	87.54	340.94
	23	2,783.86	3,058.29	2,992.61	3,061.08	11,895.84
	24	1,050.49	1,142.48	1,021.21	1,048.30	4,262.48
	25	941.81	1,022.98	1,027.65	1,017.58	4,010.02
	26	1,269.48	1,320.72	1,289.70	1,368.83	5,248.73
	27	5,067.11	5,477.60	5,281.21	5,351.97	21,177.89
TOTAL		39,805.49	42,539.50	40,755.77	42,212.57	165,313.33

RETAIL DRUG DISTRIBUTION BY ZIP CODE WITHIN STATE BY GRAMS WT

STATE: MICHIGAN

ZIP CODE	QUARTER 1	QUARTER 2	QUARTER 3	QUARTER 4	TOTAL GRAMS
480	3,763.09	4,064.16	4,115.63	4,229.83	16,172.71
481	7,314.58	7,522.53	7,297.38	7,857.04	29,991.53
482	866.01	923.44	946.96	995.12	3,731.53
483	2,814.45	2,943.16	2,989.52	3,100.54	11,847.67
484	870.78	869.05	897.4	932.36	3,569.59
485	524.6	554.67	546.36	579.7	2,205.33
486	937.15	959.58	999.49	1,010.86	3,907.08
487	620.14	615.5	633.87	645.29	2,514.80
488	1,002.55	1,086.41	1,106.97	1,192.70	4,388.63
489	212.48	226.39	216.96	214.36	870.19
490	1,245.58	1,339.44	1,410.67	1,443.95	5,439.64
491	95.38	95.95	98.46	94.09	383.88
492	715.75	756.47	781.7	802.93	3,056.85
493	271.93	327.47	388.12	383.07	1,370.59
494	680.42	725.22	752.21	799.98	2,957.83
495	783.79	846.32	842.96	960.43	3,433.50
496	1,124.77	1,148.40	1,182.64	1,245.63	4,701.44
497	1,029.11	1,098.86	1,110.01	1,174.54	4,412.52
498	740.9	877.81	826.93	869.08	3,314.72
499	97.42	83.67	92.11	101.34	374.54
TOTAL	25,710.88	27,064.50	27,236.35	28,632.84	108,644.57

STATE: MINNESOTA

ZIP CODE	QUARTER 1	QUARTER 2	QUARTER 3	QUARTER 4	TOTAL GRAMS
550	869.67	889.52	905.35	948.12	3,612.66
551	1,238.48	1,354.29	1,435.65	1,417.28	5,445.70
553	858.4	933.26	982.85	1,007.94	3,782.45
554	1,828.70	1,929.99	2,036.31	2,054.70	7,849.70
556	11.8	11.53	11.29	17.32	51.94
557	180.3	188.8	177.78	197.3	744.18
558	318.85	330.84	340.5	364.8	1,354.99
559	223.82	227.43	226.59	227.37	905.21
560	182.76	206.73	220.71	238.68	848.88
561	5.63	8.17	10.2	11.08	35.08

562	42.68	56.41	53.73	58.58	211.4
563	296.3	294.17	300.84	320.93	1,212.24
564	126.53	139.99	147.37	145.72	559.61
565	254.66	256.64	282.08	336.42	1,129.80
566	497.17	536.89	525.89	592.58	2,152.53
567	63.69	80.23	90.99	83.45	318.36
TOTAL	6,999.44	7,444.89	7,748.13	8,022.27	30,214.73

STATE: MISSISSIPPI

ZIP CODE	QUARTER 1	QUARTER 2	QUARTER 3	QUARTER 4	TOTAL GRAMS
386	1,199.56	1,232.65	1,272.76	1,269.65	4,974.62
387	72.74	89.75	88.75	95.34	346.58
388	1,572.48	1,663.10	1,602.68	1,548.35	6,386.61
389	133.78	139.74	139.45	131.82	544.79
390	620.92	628.51	623.44	640.26	2,513.13
391	702.92	694.32	727.54	710.61	2,835.39
392	533.79	518.73	559.78	518.37	2,130.67
393	470.53	484.86	476.79	492.74	1,924.92
394	1,706.85	1,720.82	1,701.03	1,706.46	6,835.16
395	2,239.04	2,392.86	2,366.57	2,505.14	9,503.61
396	274.72	267.57	248.61	250	1,040.90
397	162.43	162.94	154.93	155.47	635.77
TOTAL	9,689.76	9,995.85	9,962.33	10,024.21	39,672.15

STATE: MISSOURI

ZIP CODE	QUARTER 1	QUARTER 2	QUARTER 3	QUARTER 4	TOTAL GRAMS
630	971.07	973.65	1,019.08	1,036.50	4,000.30
631	1,493.22	1,520.94	1,606.36	1,907.76	6,528.28
633	512.37	557.1	572.95	626.44	2,268.86
634	60.8	61	65.06	77.14	264
635	19.33	25.95	24.85	30.54	100.67
636	420.61	454.89	527.16	654.52	2,057.18
637	95.55	120.84	100.34	156.2	472.93
638	165.58	164.99	174.01	198.83	703.41
639	121.6	168.58	173.06	201.79	665.03

	640	666	680	717.09	724.52	2,787.61
	641	619.13	597.05	658.25	663.64	2,538.07
	644	56.75	61.72	59.17	57.59	235.23
	645	236.02	242.47	253.65	252.74	984.88
	646	20.97	23.66	31.43	26.43	102.49
	647	82.31	93.63	94.8	107.82	378.56
	648	178.46	182.54	208.45	229.43	798.88
	650	138.78	168.96	181.7	180.43	669.87
	651	109.28	115.16	117.27	117.73	459.44
	652	448.27	486.94	522.08	585.12	2,042.41
	653	78.34	90.95	112.1	139.47	420.86
	654	109.86	124.16	142.86	159.33	536.21
	655	150.76	176.91	183.81	199.92	711.4
	656	211.36	194.95	212.77	218.85	837.93
	657	301.81	348.59	379.48	420.47	1,450.35
	658	1,362.62	1,572.75	1,566.27	1,710.17	6,211.81
TOTAL		8,630.85	9,208.38	9,704.05	10,683.38	38,226.66

STATE:	MONTANA					
ZIP CODE	QUARTER 1		QUARTER 2	QUARTER 3	QUARTER 4	TOTAL GRAMS
	590	45.91	52.39	73.82	86.64	258.76
	591	233.1	274.19	301.87	329.41	1,138.57
	592	66.67	79.12	86.01	92.4	324.2
	593	30.25	29.96	34.77	35.31	130.29
	594	610.89	617.49	663.74	679.32	2,571.44
	595	22.51	28.38	21.95	26.6	99.44
	596	239.54	256.63	281.31	277.81	1,055.29
	597	239.54	252.08	248.65	265.97	1,006.24
	598	517.16	536.88	570.79	603.79	2,228.62
	599	214.79	188.82	213.01	222.69	839.31
TOTAL		2,220.36	2,315.94	2,495.92	2,619.94	9,652.16

STATE:	NEBRASKA					
ZIP CODE	QUARTER 1		QUARTER 2	QUARTER 3	QUARTER 4	TOTAL GRAMS
	680	69.86	68.47	67.84	77.19	283.36

	681	500.32	538.6	565.76	556.4	2,161.08
	683	44.14	40.27	35.6	35.87	155.88
	684	22.12	16.8	24.23	19.28	82.43
	685	282.59	292.41	291.43	285.4	1,151.83
	686	30.07	29.81	28.97	31.96	120.81
	687	57.28	61.92	63.98	65.53	248.71
	688	69.72	74.64	87.54	98.78	330.68
	689	26.78	20.39	23.18	27.22	97.57
	690	1.82	3.54	3.75	5.2	14.31
	691	133.65	138.16	147.6	145.88	565.29
	692	1.32	0.4	0.52	1.18	3.42
	693	24.86	23.1	23.16	22.33	93.45
TOTAL		1,264.53	1,308.51	1,363.56	1,372.22	5,308.82

STATE:		NEVADA				
ZIP CODE		QUARTER 1	QUARTER 2	QUARTER 3	QUARTER 4	TOTAL GRAMS
	890	698.46	718.9	730.33	672.3	2,819.99
	891	1,511.85	1,700.85	1,607.98	1,679.90	6,500.58
	893	12.16	13	15.7	9.8	50.66
	894	276.97	307.25	322.35	324.73	1,231.30
	895	568.74	572.52	599.54	580.73	2,321.53
	897	104.65	100.72	110.37	131.86	447.6
	898	65.96	63.31	74.96	69.32	273.55
TOTAL		3,238.79	3,476.55	3,461.23	3,468.64	13,645.21

STATE:		NEW HAMPSHIRE				
ZIP CODE		QUARTER 1	QUARTER 2	QUARTER 3	QUARTER 4	TOTAL GRAMS
	030	1,225.17	1,262.65	1,307.97	1,293.36	5,089.15
	031	1,171.92	1,214.56	1,233.18	1,224.56	4,844.22
	032	1,100.97	1,182.68	1,258.64	1,275.86	4,818.15
	033	732.74	821.49	884.52	869.76	3,308.51
	034	320.91	325.95	305.01	320.16	1,272.03
	035	329.29	322.69	322.03	290.94	1,264.95
	036	9.9	11.8	10.32	9.6	41.62
	037	1,151.18	1,189.73	1,182.09	1,141.71	4,664.71

	038	1,628.51	1,727.09	1,709.70	1,724.62	6,789.92
TOTAL		7,670.59	8,058.64	8,213.46	8,150.57	32,093.26

STATE: NEW JERSEY

ZIP CODE		QUARTER 1	QUARTER 2	QUARTER 3	QUARTER 4	TOTAL GRAMS
	070	2,748.49	2,841.99	2,833.38	2,929.92	11,353.78
	071	918.78	895.06	898.32	900.99	3,613.15
	072	237.79	241.53	228.85	228.18	936.35
	073	163.96	162.25	166.02	166.28	658.51
	074	1,142.80	1,200.50	1,180.73	1,173.57	4,697.60
	075	285.92	255.78	260.5	266.03	1,068.23
	076	774.7	829.61	833.13	848.84	3,286.28
	077	1,989.24	2,115.79	2,089.15	2,082.89	8,277.07
	078	748.19	852.24	872.25	839.01	3,311.69
	079	355.89	367.03	365.27	352.64	1,440.83
	080	6,913.98	7,289.78	6,757.97	7,260.17	28,221.90
	081	725.13	833.13	849.85	886.58	3,294.69
	082	1,738.13	1,871.58	1,869.34	1,842.11	7,321.16
	083	965.14	1,055.30	996.24	1,040.67	4,057.35
	084	223.26	234.34	237.72	251.48	946.8
	085	544.89	589.02	575.93	594.52	2,304.36
	086	737.23	743.25	709.36	730	2,919.84
	087	2,438.20	2,565.54	2,517.35	2,592.46	10,113.55
	088	1,710.24	1,813.31	1,736.38	1,788.81	7,048.74
	089	185.27	164.77	169.28	161.61	680.93
TOTAL		25,547.23	26,921.80	26,147.02	26,936.76	105,552.81

STATE: NEW MEXICO

ZIP CODE		QUARTER 1	QUARTER 2	QUARTER 3	QUARTER 4	TOTAL GRAMS
	870	352.15	391.12	395.09	405.03	1,543.39
	871	3,307.59	3,483.25	3,593.10	3,693.80	14,077.74
	873	100.93	117.55	136.67	116.24	471.39
	874	298.3	419.2	418.89	330.13	1,466.52
	875	1,706.49	1,715.30	1,909.64	2,086.75	7,418.18
	877	156.02	179.98	199.17	198.93	734.1

	878	50.97	54.5	60.53	66.75	232.75
	879	12.9	16.56	11.34	13	53.8
	880	364.08	362.93	349.76	372.4	1,449.17
	881	22.89	26.88	35.54	47.6	132.91
	882	358.86	365.86	384.53	388.56	1,497.81
	883	89.24	97.28	100.45	113.07	400.04
	884	5.5	3.54	4.02	4.74	17.8
TOTAL		6,825.92	7,233.95	7,598.73	7,837	29,495.60

STATE: NEW YORK

ZIP CODE		QUARTER 1	QUARTER 2	QUARTER 3	QUARTER 4	TOTAL GRAMS
	100	2,591.90	2,494.75	2,612.07	2,725	10,423.72
	101	95.14	93.36	70.85	68.81	328.16
	102	0.7	3.3	3.53	1.74	9.27
	103	1,744.13	1,811.78	1,797.04	1,865	7,217.95
	104	2,295.54	2,311.36	2,491.87	2,493.44	9,592.21
	105	1,030.18	1,078.82	1,074.92	1,113.84	4,297.76
	106	234.68	219.81	229.13	243.8	927.42
	107	348.99	363.92	387.05	389.35	1,489.31
	108	102.41	99.57	98.29	110.52	410.79
	109	1,383.19	1,397.52	1,414.29	1,492.48	5,687.48
	110	480.92	526.07	531.74	567.91	2,106.64
	111	249.9	253.64	277.48	279.47	1,060.49
	112	2,919.72	2,939.97	2,876.76	2,964.02	11,700.47
	113	1,113.91	1,115.39	1,086.89	1,081.46	4,397.65
	114	488.97	515.4	529.32	515.5	2,049.19
	115	1,260.28	1,399.72	1,380.04	1,444.35	5,484.39
	116	98.64	106.64	104.13	118.08	427.49
	117	5,730.90	5,875.66	5,906.63	6,121.60	23,634.79
	118	470.49	519.35	486.5	531.57	2,007.91
	119	1,143.35	1,190.61	1,190.69	1,207.88	4,732.53
	120	1,575.11	1,752.77	1,743.70	1,915.45	6,987.03
	121	828.96	869.37	896.34	930.03	3,524.70
	122	616.47	633.66	675.19	758.58	2,683.90
	123	600.43	649.35	648.67	684.86	2,583.31
	124	741.23	782.99	842.02	891.54	3,257.78

	125	1,268.07	1,355.59	1,400.70	1,545.14	5,569.50
	126	282.09	324.18	318.35	319.8	1,244.42
	127	787.86	827.66	844.21	834.48	3,294.21
	128	1,297.11	1,442.06	1,515.68	1,530.12	5,784.97
	129	964.29	1,097.27	1,251.91	1,335.78	4,649.25
	130	981.74	1,049.82	1,087.46	1,112.31	4,231.33
	131	332.17	346.6	343.04	364.16	1,385.97
	132	876.26	910.81	927.86	1,032.47	3,747.40
	133	313.77	316.84	269.57	325.32	1,225.50
	134	640.29	636.95	620.14	727.9	2,625.28
	135	348.97	355.03	367.14	396.44	1,467.58
	136	824.09	856.29	804.26	941.2	3,425.84
	137	274.6	325.15	335.72	363.4	1,298.87
	138	652.56	744.01	769.87	801.97	2,968.41
	139	332.87	351.16	363.41	412.97	1,460.41
	140	2,142.17	2,244.12	2,262.44	2,455.53	9,104.26
	141	956.52	1,014.60	1,025.88	1,013.12	4,010.12
	142	3,107.29	3,319.83	3,321.17	3,515.09	13,263.38
	143	657.42	687.56	680.05	823.68	2,848.71
	144	853.79	868.34	875.65	914.52	3,512.30
	145	612.11	637.06	662.48	692.17	2,603.82
	146	2,067.97	2,099.29	2,159.70	2,247.84	8,574.80
	147	721.9	790.45	763.28	792.74	3,068.37
	148	832.44	948.04	980.05	1,195.15	3,955.68
	149	213.6	251.13	267.02	283.16	1,014.91
TOTAL		50,488.09	52,804.62	53,572.18	56,492.74	213,357.63

STATE: NORTH CAROLINA

ZIP CODE		QUARTER 1	QUARTER 2	QUARTER 3	QUARTER 4	TOTAL GRAMS
	270	570.24	597.14	614.8	647.98	2,430.16
	271	597.07	645.91	657.33	714.32	2,614.63
	272	1,235.66	1,270.70	1,245.30	1,474.48	5,226.14
	273	912.87	989.92	928.51	1,043.23	3,874.53
	274	664.28	668.94	688.03	680.41	2,701.66
	275	1,947.11	2,078.72	2,182.74	2,325.21	8,533.78
	276	1,076.26	1,048.23	1,077.71	1,135.93	4,338.13

277	1,534.63	1,647.57	1,443.65	1,631.72	6,257.57
278	873.03	983.75	1,080.28	1,215.77	4,152.83
279	472.96	490.54	490.54	486.53	1,940.57
280	2,614.36	2,812.75	2,419.40	2,993.74	10,840.25
281	1,746.46	1,925.11	1,768.78	2,211.83	7,652.18
282	1,445.13	1,638.39	1,540.10	1,573.98	6,197.60
283	2,290.01	2,596.70	2,527.54	2,883.36	10,297.61
284	3,163.23	3,525.43	3,366.97	3,524.62	13,580.25
285	1,184.63	1,177.88	1,112.38	1,208.44	4,683.33
286	4,411.92	4,834.07	4,793.25	4,976.57	19,015.81
287	2,128.76	2,328.73	2,275.51	2,448.30	9,181.30
288	1,320.56	1,629.09	1,684.93	1,916.62	6,551.20
289	176.05	185.06	194.67	216.79	772.57
TOTAL	30,365.22	33,074.63	32,092.42	35,309.83	130,842.10

STATE: NORTH DAKOTA

ZIP CODE	QUARTER 1	QUARTER 2	QUARTER 3	QUARTER 4	TOTAL GRAMS
580	3.48	6.95	12.42	15.03	37.88
581	269.53	268.66	269.24	267.17	1,074.60
582	42.48	49.06	50.84	48.47	190.85
583	41.76	46.56	59.94	59.22	207.48
584	12.72	13.87	14.97	18.43	59.99
585	341.98	343.82	380.1	376.15	1,442.05
586	39.68	44.2	44.04	50.78	178.7
587	35.3	41.9	42.93	44.74	164.87
588	65.02	68.11	69.51	76.28	278.92
TOTAL	851.95	883.13	943.99	956.27	3,635.34

STATE: OHIO

ZIP CODE	QUARTER 1	QUARTER 2	QUARTER 3	QUARTER 4	TOTAL GRAMS
430	3,070.56	3,398.57	3,246.23	3,329.23	13,044.59
431	2,469.57	2,587.06	2,556.69	2,595.27	10,208.59
432	5,064.78	5,419.57	5,552.02	5,444.05	21,480.42
433	895.7	1,032.89	1,059.67	1,104.02	4,092.28
434	249.41	292.03	310.22	326.39	1,178.05

	435	719.05	684.59	670.34	675.45	2,749.43
	436	1,886.33	1,890.31	1,962.89	2,082.99	7,822.52
	437	1,308.55	1,368.39	1,333.28	1,374.08	5,384.30
	438	65.25	74.73	74.33	75.49	289.8
	439	1,758.14	1,777.51	1,662.78	1,832.94	7,031.37
	440	2,209.35	2,324	2,206.10	2,252.94	8,992.39
	441	2,976.97	3,170.22	3,054.01	3,145.46	12,346.66
	442	1,446.99	1,571.72	1,540.37	1,568.91	6,127.99
	443	806.78	775	780.06	812.92	3,174.76
	444	1,305.95	1,270.52	1,301.29	1,347.34	5,225.10
	445	1,442.92	1,553.19	1,443.77	1,478.66	5,918.54
	446	800.69	808.2	808.87	800.1	3,217.86
	447	760.29	770.04	828.24	777.5	3,136.07
	448	1,070.57	1,121.44	1,104.79	1,135.25	4,432.05
	449	866.77	933.09	901.91	934.18	3,635.95
	450	2,833.07	2,953.07	2,857.63	2,964.34	11,608.11
	451	2,128.80	2,333.70	2,213.87	2,356.36	9,032.73
	452	4,811.19	4,883.85	4,836.58	4,804.93	19,336.55
	453	1,051.22	1,113.06	1,074	1,019.14	4,257.42
	454	2,041.08	2,099.37	2,209.13	2,164.88	8,514.46
	455	796.89	793.4	808.69	759.82	3,158.80
	456	4,328.32	4,557.93	4,709.42	4,751.30	18,346.97
	457	1,125.37	1,123.23	1,102.28	1,104.32	4,455.20
	458	467.18	473.54	456.83	502.37	1,899.92
TOTAL		50,757.74	53,154.22	52,666.29	53,520.63	210,098.88

STATE: OKLAHOMA

ZIP CODE		QUARTER 1	QUARTER 2	QUARTER 3	QUARTER 4	TOTAL GRAMS
	730	1,615.82	1,652.38	1,783.55	1,826.46	6,878.21
	731	2,315.92	2,289.22	2,584.22	2,629.68	9,819.04
	734	95.62	97.33	104.44	123.65	421.04
	735	546.3	536.11	615.41	552.25	2,250.07
	736	87.91	99.85	117.71	133.11	438.58
	737	242.03	227.69	221.79	203.2	894.71
	738	58.07	48.04	74.42	75.45	255.98
	739	18.05	15.78	13.53	9.16	56.52

	740	1,792.75	1,875.67	2,108.06	2,136.49	7,912.97
	741	1,368.98	1,451.85	1,550.46	1,544.42	5,915.71
	743	161.81	172.91	177.49	208.47	720.68
	744	622.04	673.69	791.79	836.57	2,924.09
	745	187.96	189.77	201.42	206.77	785.92
	746	177.67	172.15	193.17	181.1	724.09
	747	80.97	84.38	97.58	114.51	377.44
	748	620.86	601.88	669.89	671.64	2,564.27
	749	183.04	173.62	209.51	215.4	781.57
TOTAL		10,175.80	10,362.32	11,514.44	11,668.33	43,720.89

STATE: OREGON

ZIP CODE		QUARTER 1	QUARTER 2	QUARTER 3	QUARTER 4	TOTAL GRAMS
	970	1,643.70	1,782.82	1,870.38	1,900.19	7,197.09
	971	597.89	657.04	712.04	765.07	2,732.04
	972	2,939.62	3,084.58	2,858.62	3,094.96	11,977.78
	973	1,322.93	1,415.18	1,443.87	1,558.09	5,740.07
	974	1,261.64	1,361.76	1,494	1,600.29	5,717.69
	975	970.43	936.89	961.06	1,021	3,889.38
	976	33.88	39.24	37.68	43.51	154.31
	977	365.17	358.51	383.53	391.61	1,498.82
	978	554.23	607.44	628.31	666.48	2,456.46
	979	35.14	35	47.84	54.85	172.83
TOTAL		9,724.63	10,278.46	10,437.33	11,096.05	41,536.47

STATE: PENNSYLVANIA

ZIP CODE		QUARTER 1	QUARTER 2	QUARTER 3	QUARTER 4	TOTAL GRAMS
	150	3,273.95	3,334.42	3,275.91	3,448.23	13,332.51
	151	2,310.30	2,483.84	2,198.21	2,557.78	9,550.13
	152	5,856.44	6,141.96	5,989.90	6,224.55	24,212.85
	153	2,118.19	2,116.92	1,924.19	2,096.19	8,255.49
	154	1,256.61	1,250.12	1,120.36	1,209.14	4,836.23
	155	360.21	377.67	410.69	426.5	1,575.07
	156	2,647.99	2,936.76	3,150.14	3,277.60	12,012.49
	157	681.48	677.44	708.91	700.12	2,767.95

158	638.24	651.74	619.01	685	2,593.99
159	1,065.55	1,108.12	1,094.82	1,166.39	4,434.88
160	1,347.29	1,354.15	1,375.58	1,401.78	5,478.80
161	983.72	1,077.98	961.12	1,227.56	4,250.38
162	747.15	790.32	790.35	824.49	3,152.31
163	641.28	675.47	657.92	678.78	2,653.45
164	263.2	294.34	292.55	286.4	1,136.49
165	1,657.13	1,733.32	1,758.90	1,785.21	6,934.56
166	1,611.61	1,683.76	1,708.23	1,847.96	6,851.56
167	118.49	121.81	137.02	131.84	509.16
168	530.12	560.54	553.32	629	2,272.98
169	125.5	146.92	146.66	129.97	549.05
170	1,549.04	1,626.10	1,626.59	1,762.77	6,564.50
171	339.98	387.24	421.18	500.95	1,649.35
172	796.38	871.98	876.05	857.84	3,402.25
173	987.13	1,009	1,001.93	1,055.26	4,053.32
174	426.07	488.93	506.91	555.99	1,977.90
175	329.2	322.98	345.34	337.22	1,334.74
176	410.39	427.95	445.57	494.1	1,778.01
177	1,381.54	1,460.43	1,581.32	1,525.96	5,949.25
178	715.17	799.89	736.66	840.95	3,092.67
179	415.15	457.97	446.43	477.54	1,797.09
180	800.38	810.34	834.09	861.11	3,305.92
181	391.89	437.18	440.74	444.1	1,713.91
182	343.61	424.72	407.75	460.32	1,636.40
183	447.44	481.85	455.49	490.33	1,875.11
184	825.52	831.54	779.67	1,041.15	3,477.88
185	1,812.77	1,909.81	1,999.99	2,137.48	7,860.05
186	795.39	839.46	840.12	1,006.73	3,481.70
187	1,947.14	1,981.89	1,529.73	2,064.57	7,523.33
188	269.38	307.34	312.53	348.48	1,237.73
189	1,018.29	1,065.04	1,087.58	1,105.39	4,276.30
190	5,167.84	5,287.97	5,200.06	5,388.57	21,044.44
191	7,286.14	7,780.70	7,932.56	8,027.11	31,026.51
193	939.5	947.41	992.1	959.89	3,838.90
194	1,863.71	1,880.86	1,743.32	1,888.21	7,376.10
195	258.95	301.72	293.86	293.42	1,147.95

196	579.35	587.43	480.55	658.8	2,306.13
TOTAL	60,331.80	63,245.33	62,191.91	66,318.73	252,087.77

STATE: PUERTO RICO

ZIP CODE	QUARTER 1	QUARTER 2	QUARTER 3	QUARTER 4	TOTAL GRAMS
6	562.15	602.75	478.21	536.9	2,180.01
7	1,371.81	1,426.03	1,204.97	1,603.31	5,606.12
9	558.29	541.95	536.47	533.47	2,170.18
TOTAL	2,492.25	2,570.73	2,219.65	2,673.68	9,956.31

STATE: RHODE ISLAND

ZIP CODE	QUARTER 1	QUARTER 2	QUARTER 3	QUARTER 4	TOTAL GRAMS
28	3,874.27	4,236.70	4,077.32	4,330.03	16,518.32
29	3,204.45	3,464.66	3,412.42	3,497.44	13,578.97
TOTAL	7,078.72	7,701.36	7,489.74	7,827.47	30,097.29

STATE: SOUTH CAROLINA

ZIP CODE	QUARTER 1	QUARTER 2	QUARTER 3	QUARTER 4	TOTAL GRAMS
290	286.52	322.11	338.73	335.23	1,282.59
291	353.97	384.19	379.25	450.32	1,567.73
292	426.48	462.85	498.03	486.57	1,873.93
293	1,353.48	1,422.68	1,364.99	1,497.74	5,638.89
294	1,701.99	1,774.57	1,803.70	1,876.42	7,156.68
295	1,498.76	1,688.03	1,842.33	2,074.04	7,103.16
296	3,029.11	3,167.19	3,309.04	3,463.44	12,968.78
297	558.41	623.49	643.08	656.64	2,481.62
298	176.31	175.69	160.92	172.37	685.29
299	459.54	479.86	455.28	485.41	1,880.09
TOTAL	9,844.57	10,500.66	10,795.35	11,498.18	42,638.76

STATE: SOUTH DAKOTA

ZIP CODE	QUARTER 1	QUARTER 2	QUARTER 3	QUARTER 4	TOTAL GRAMS
570	33.39	39.13	43.39	48.64	164.55

	571	124.68	152.66	140.2	161.33	578.87
	572	49.45	49.76	46.24	49.92	195.37
	573	17.6	17.36	11.29	16.57	62.82
	574	82.41	93.9	83.28	74.78	334.37
	575	10.68	9.43	11.33	11.19	42.63
	576	6.08	5.94	6.98	9.69	28.69
	577	86.32	96.97	106.85	100.34	390.48
TOTAL		410.61	465.15	449.56	472.46	1,797.78

STATE: TENNESSEE

ZIP CODE		QUARTER 1	QUARTER 2	QUARTER 3	QUARTER 4	TOTAL GRAMS
	370	2,884.86	3,172.89	3,156.95	3,273.82	12,488.52
	371	3,302.32	3,480.67	3,153.46	3,813.40	13,749.85
	372	2,049.44	2,264.30	2,349.99	2,446.73	9,110.46
	373	1,319.10	1,417.45	1,419.52	1,551.45	5,707.52
	374	630.6	662.64	639.58	743.46	2,676.28
	376	7,171.82	7,494.14	7,325.21	7,280.22	29,271.39
	377	2,976.56	3,119.40	3,179.06	3,239.65	12,514.67
	378	4,533.97	4,803.61	4,846.49	4,962.37	19,146.44
	379	2,202.27	2,305.71	2,300.76	2,340	9,148.74
	380	771.23	820.87	857.25	870.38	3,319.73
	381	1,165.33	1,185.50	1,114.79	1,148.74	4,614.36
	382	474.08	490.85	486.48	491.92	1,943.33
	383	1,108.41	1,237.53	1,310.61	1,338.68	4,995.23
	384	841.11	938.73	867.38	986.89	3,634.11
	385	1,640.60	1,759.50	1,731.29	1,833.71	6,965.10
TOTAL		33,071.70	35,153.79	34,738.82	36,321.42	139,285.73

STATE: TEXAS

ZIP CODE		QUARTER 1	QUARTER 2	QUARTER 3	QUARTER 4	TOTAL GRAMS
	750	2,520.21	2,640.63	2,766.52	2,692.39	10,619.75
	751	755.64	795.43	808.37	805.11	3,164.55
	752	1,000.52	1,052.78	1,029.91	1,033.15	4,116.36
	753	1.92	2.8	1.34	3.33	9.39
	754	455.69	457.15	470.25	495.02	1,878.11

755	126.18	128.3	135.2	148.47	538.15
756	516.32	531.73	516.49	512.89	2,077.43
757	441.05	435.42	419.4	411.03	1,706.90
758	62.51	67.11	65.17	57.13	251.92
759	337.04	335.15	342.17	347.11	1,361.47
760	1,906.93	2,004.82	2,012.97	2,015.48	7,940.20
761	979.58	1,034.58	1,079.97	1,022.22	4,116.35
762	550.47	547.67	585.97	569.97	2,254.08
763	277.15	296.73	317.99	310.62	1,202.49
764	97.63	103.07	106.4	119.82	426.92
765	291.11	296.22	328.99	299.89	1,216.21
766	65.91	56.28	67.6	69.4	259.19
767	122.6	132.51	145.09	145.19	545.39
768	39.55	46.65	49.15	43.47	178.82
769	65.87	66.11	67.5	68.9	268.38
770	2,341.22	2,423.47	2,348.38	2,489.70	9,602.77
772	0	0	0	0	0
773	2,169.62	2,196.14	2,242.45	2,311.98	8,920.19
774	954.89	943.46	937.3	954.71	3,790.36
775	2,272.12	2,264.79	2,249.86	2,234.19	9,020.96
776	885.08	897.96	881.85	868.73	3,533.62
777	299.21	298.94	291.16	302.89	1,192.20
778	293.88	300.74	305.76	316.03	1,216.41
779	140.66	138.28	142.01	150.64	571.59
780	367.77	354.24	406.37	403.11	1,531.49
781	310.79	310.45	319.54	317.76	1,258.54
782	950.65	973.97	1,023.77	1,012.99	3,961.38
783	154.13	155.21	154.4	151.49	615.23
784	302.93	363.16	366.87	345.9	1,378.86
785	118.99	112.29	112	99.74	443.02
786	857.54	926.92	992.29	1,012.31	3,789.06
787	1,249.24	1,297.97	1,361.17	1,327.19	5,235.57
788	32.11	27.46	28.59	29.2	117.36
789	30.5	24.42	25.89	27.71	108.52
790	34.35	26.23	25.49	24.82	110.89
791	104.69	91.62	113.87	113.06	423.24
792	5.26	3.64	5.1	3.52	17.52

	793	36.08	42.96	48.16	46.37	173.57
	794	149.39	165.49	198.66	192.52	706.06
	795	29.42	32.76	29.13	31.84	123.15
	796	185.85	201.13	210.84	225.85	823.67
	797	454.12	470.29	505.19	543.44	1,973.04
	798	9.64	8.32	6.4	3.92	28.28
	799	126.76	128.34	146.12	136.48	537.7
TOTAL		25,480.77	26,211.79	26,795.07	26,848.68	105,336.31

STATE: UTAH

ZIP CODE		QUARTER 1	QUARTER 2	QUARTER 3	QUARTER 4	TOTAL GRAMS
	840	4,360.55	4,399.34	4,481.35	4,467.78	17,709.02
	841	2,293.53	2,364.75	2,335.24	2,426.57	9,420.09
	843	370.53	422.33	426.06	392.22	1,611.14
	844	776.28	786.27	825.81	808.77	3,197.13
	845	192.22	219.24	232.3	249.95	893.71
	846	1,006.33	1,053.10	1,081.86	1,166.27	4,307.56
	847	298.53	348.39	380.03	396.83	1,423.78
TOTAL		9,297.97	9,593.42	9,762.65	9,908.39	38,562.43

STATE: VERMONT

ZIP CODE		QUARTER 1	QUARTER 2	QUARTER 3	QUARTER 4	TOTAL GRAMS
	50	657.9	683.03	696.57	716.77	2,754.27
	51	243	267.65	240.48	251.53	1,002.66
	52	362.04	392.04	380.13	389.97	1,524.18
	53	370.08	361.18	361.72	350.89	1,443.87
	54	2,209.37	2,447.99	2,481.21	2,579.33	9,717.90
	56	775.96	800.44	811.66	776.17	3,164.23
	57	937.97	1,046.55	970.29	1,037.81	3,992.62
	58	414.72	402.58	404.09	397.97	1,619.36
TOTAL		5,971.04	6,401.46	6,346.15	6,500.44	25,219.09

RETAIL DRUG DISTRIBUTION BY ZIP CODE WITHIN STATE BY GRAMS WT

STATE: VIRGIN ISLANDS

ZIP CODE	QUARTER 1	QUARTER 2	QUARTER 3	QUARTER 4	TOTAL GRAMS
8	19.68	17.54	12.98	12.51	62.71
TOTAL	19.68	17.54	12.98	12.51	62.71

STATE: VIRGINIA

ZIP CODE	QUARTER 1	QUARTER 2	QUARTER 3	QUARTER 4	TOTAL GRAMS
201	1,466.26	1,323.28	1,318.85	1,319.08	5,427.47
220	819.8	802.88	794	779.97	3,196.65
221	594.13	581.07	552.74	528.52	2,256.46
222	631.52	895.03	892.43	897.54	3,316.52
223	210.13	197.15	199.15	196.61	803.04
224	493.36	504.5	499.14	498.51	1,995.51
225	238.74	242.92	218.71	214.86	915.23
226	1,094.79	1,086.22	1,047.48	1,074.26	4,302.75
227	149.62	167	186.06	216.57	719.25
228	202.96	201.89	187.32	189.68	781.85
229	378.22	411.72	418.13	439.67	1,647.74
230	347.44	308.93	315.14	310.11	1,281.62
231	945.72	996.77	764.39	883.58	3,590.46
232	1,030.43	1,058.64	1,176.82	1,240.62	4,506.51
233	293.25	275.52	182.55	277.64	1,028.96
234	639.47	666.37	696.25	689.53	2,691.62
235	118.64	140.8	145.25	150.75	555.44
236	632.23	705.97	701.77	737.36	2,777.33
237	99.54	109.59	103	103.5	415.63
238	425.9	407.68	387.44	353.74	1,574.76
239	139.86	133.49	125.55	137.81	536.71
240	1,904.04	1,907.62	1,583.15	1,858.23	7,253.04
241	779.3	847.06	807.39	783.67	3,217.42
242	3,485.64	3,377.34	3,271.49	3,368.65	13,503.12
243	1,103.66	850.19	771.9	819.27	3,545.02
244	162	140.42	131.23	140.82	574.47
245	776.54	784.4	719.49	729.72	3,010.15
246	1,049.14	988.73	993.82	1,002.24	4,033.93
TOTAL	20,212.33	20,113.18	19,190.64	19,942.51	79,458.66

STATE: WASHINGTON

ZIP CODE	QUARTER 1	QUARTER 2	QUARTER 3	QUARTER 4	TOTAL GRAMS
980	3,298.56	3,453.70	3,562.07	3,546.25	13,860.58
981	2,049.22	2,210.67	2,262.52	2,384.85	8,907.26
982	3,465.57	3,607.62	3,638.03	3,826.76	14,537.98
983	1,608.61	1,682.57	1,706.29	1,826.01	6,823.48
984	1,169.92	1,207.34	1,165.21	1,192.17	4,734.64
985	1,481.49	1,576.20	1,628.48	1,758.19	6,444.36
986	1,106.09	1,155.05	1,230.90	1,389.34	4,881.38
988	466.68	520.46	568.94	652.64	2,208.72
989	587.1	583.95	651.57	688.69	2,511.31
990	102.84	113.56	126.15	137.61	480.16
991	275.68	285.78	302.79	264.8	1,129.05
992	1,461.97	1,619.71	1,659.31	1,722.71	6,463.70
993	733.84	787.39	879.26	922.88	3,323.37
994	174.86	163.41	172.24	182.98	693.49
TOTAL	17,982.43	18,967.41	19,553.76	20,495.88	76,999.48

STATE: WEST VIRGINIA

ZIP CODE	QUARTER 1	QUARTER 2	QUARTER 3	QUARTER 4	TOTAL GRAMS
247	204.8	237.41	274.15	279.73	996.09
248	473.35	539.9	498.78	453.87	1,965.90
249	127.24	115.85	123.62	119.67	486.38
250	806.91	816.14	803.95	788.88	3,215.88
251	270.19	276.74	253.77	246.18	1,046.88
252	313.04	321.05	269.61	240.35	1,144.05
253	1,357.24	1,383.11	1,411.59	1,367.10	5,519.04
254	1,030.58	1,114.96	1,142.18	1,182.67	4,470.39
255	1,313.74	1,292.51	1,243.56	1,374.95	5,224.76
256	1,323.27	1,426.53	1,324.66	863.84	4,938.30
257	940.68	931.49	1,042.62	950.94	3,865.73
258	1,519.26	1,794.82	1,944.92	2,192.29	7,451.29
259	272.38	276.3	333.41	322.86	1,204.95

	260	1,540.47	1,568.35	1,794	1,732.11	6,634.93
	261	556.55	583.3	543.13	576.1	2,259.08
	262	484.44	480.83	554.99	594.3	2,114.56
	263	776.61	739.78	792.48	804.35	3,113.22
	264	151.72	153.26	145.12	144.89	594.99
	265	1,226.44	1,235.83	1,253.28	1,244.32	4,959.87
	266	102.23	90.99	93.19	94.25	380.66
	267	91.49	111.07	117.72	226.75	547.03
	268	15.43	17.82	11.99	11.41	56.65
TOTAL		14,898.06	15,508.04	15,972.72	15,811.81	62,190.63

STATE: WISCONSIN

ZIP CODE		QUARTER 1	QUARTER 2	QUARTER 3	QUARTER 4	TOTAL GRAMS
	530	1,101.49	1,180.04	1,128.75	1,185.44	4,595.72
	531	1,241.02	1,283.62	1,314.97	1,391.14	5,230.75
	532	2,452.05	2,421.57	2,448.45	2,515.23	9,837.30
	534	199.89	195.97	209.08	217.09	822.03
	535	830.73	888.82	935.34	967.73	3,622.62
	537	726.53	759.81	782.09	866.44	3,134.87
	538	22.21	21.67	20.76	29.57	94.21
	539	325.43	340.42	361.72	410.01	1,437.58
	540	81.62	83.02	93.41	91.07	349.12
	541	255.63	263.14	271.33	276.59	1,066.69
	542	101.92	102.96	114.89	124.21	443.98
	543	213.77	234.84	242.08	242.34	933.03
	544	443.98	455.56	427.4	442.39	1,769.33
	545	213.95	238.26	252.29	286.32	990.82
	546	356.55	346.39	326.56	328.8	1,358.30
	547	181.54	183.6	168.72	179.6	713.46
	548	164.71	179.68	204.92	191.84	741.15
	549	970.09	1,002.66	970.04	1,020.58	3,963.37
TOTAL		9,883.11	10,182.03	10,272.80	10,766.39	41,104.33

STATE: WYOMING

ZIP CODE	QUARTER 1	QUARTER 2	QUARTER 3	QUARTER 4	TOTAL GRAMS

	820	134.05	373.69	336.5	458.03	1,302.27
	822	7.2	8.57	10.36	12.36	38.49
	823	5.64	8.98	13.7	8.87	37.19
	824	111.25	101.48	123.69	110.71	447.13
	825	71.46	67.82	60.71	72.69	272.68
	826	340.34	343.41	417.87	404.18	1,505.80
	827	83.67	83.57	80.17	80.95	328.36
	828	40.07	47.7	56.61	56.34	200.72
	829	155.52	162.84	168.52	200.67	687.55
	830	39.38	41.34	32.94	35.11	148.77
	831	13.96	17.42	15.96	16.6	63.94
TOTAL		1,002.54	1,256.82	1,317.03	1,456.51	5,032.90

DRUG CODE: 9120 DRUG NAME: DIHYDROCODEINE

STATE: ALABAMA

ZIP CODE		QUARTER 1	QUARTER 2	QUARTER 3	QUARTER 4	TOTAL GRAMS
	350	117.02	122.73	136.51	128.71	504.97
	351	24.99	36.85	30.5	29.43	121.77
	352	40.8	56.72	49.78	56.13	203.43
	354	129.51	111.25	99.5	76.91	417.17
	355	45.18	56.02	39.15	41.23	181.58
	356	244.29	252.57	281.94	298.29	1,077.09
	357	18.91	24.78	30.87	16.56	91.12
	358	14.95	5.87	13.03	17.72	51.57
	359	26.28	14.95	9.19	14.31	64.73
	360	8.55	20.3	7.48	7.48	43.81
	361	14.95	21.36	12.82	9.61	58.74
	362	14.21	15.33	19.01	22.7	71.25
	363	10.68	4.27	3.2	3.2	21.35
	364	1.07	2.14	1.07	1.07	5.35
	365	23.63	16.2	18.96	17.53	76.32
	366	37.99	26.44	19.9	29.91	114.24
	367	3.2	2.14	6.41	5.34	17.09
	368	7.48	7.48	6.41	5.34	26.71
	369	0	1.07	0	0	1.07

RETAIL DRUG DISTRIBUTION BY ZIP CODE WITHIN STATE BY GRAMS WT

TOTAL		783.69	798.47	785.73	781.47	3,149.36

STATE: ALASKA

ZIP CODE		QUARTER 1	QUARTER 2	QUARTER 3	QUARTER 4	TOTAL GRAMS
	996	8.55	3.2	0	2.14	13.89
TOTAL		8.55	3.2	0	2.14	13.89

STATE: ARIZONA

ZIP CODE		QUARTER 1	QUARTER 2	QUARTER 3	QUARTER 4	TOTAL GRAMS
	850	14.31	9.61	5.34	0	29.26
	852	53.41	75.84	69.43	81.18	279.86
	853	1.17	0	0	2.14	3.31
	856	0	1.07	1.07	0	2.14
	863	0.21	0	0	0	0.21
	864	4.7	0	0	0	4.7
TOTAL		73.8	86.52	75.84	83.32	319.48

STATE: ARKANSAS

ZIP CODE		QUARTER 1	QUARTER 2	QUARTER 3	QUARTER 4	TOTAL GRAMS
	716	1.07	7.48	5.66	0	14.21
	717	1.07	2.14	3.2	0	6.41
	718	13.03	9.61	13.89	4.27	40.8
	719	8.28	8.28	33.7	35.25	85.51
	720	12.5	26.76	13.65	8.55	61.46
	721	4.27	10.68	1.07	3.2	19.22
	722	11.54	8.6	4.01	2.14	26.29
	723	2.24	19.49	37.12	32.47	91.32
	724	18.16	20.03	25.8	19.28	83.27
	726	0	1.07	0	0	1.07
	727	5.53	11.88	10.68	10.68	38.77
	728	3.2	0	0	2.14	5.34
	729	24.78	26.33	26.7	17.09	94.9
TOTAL		105.67	152.35	175.48	135.07	568.57

RETAIL DRUG DISTRIBUTION BY ZIP CODE WITHIN STATE BY GRAMS WT

STATE: CALIFORNIA

ZIP CODE	QUARTER 1	QUARTER 2	QUARTER 3	QUARTER 4	TOTAL GRAMS
900	7.48	8.55	2.14	0	18.17
902	17.09	0	1.07	0	18.16
903	1.07	0	0	0	1.07
913	6.41	160.86	37.39	274.3	478.96
914	0	0	1.07	0	1.07
917	53.73	14.95	10.68	0	79.36
920	17.09	22.43	3.2	9.61	52.33
921	1.07	0	1.07	0.21	2.35
925	1.07	1.07	0	0	2.14
927	4.27	3.2	0	0	7.47
928	21.26	3.2	2.14	3.2	29.8
930	33.97	0.64	0	3.2	37.81
931	2.14	1.07	0	2.14	5.35
934	21.36	5.34	1.07	2.14	29.91
935	1.07	0	0	0	1.07
936	0	0	1.07	1.07	2.14
937	0	0	0.13	0	0.13
941	12.82	14.95	1.07	3.2	32.04
945	1.07	1.07	2.14	1.07	5.35
949	0	0	2.14	0	2.14
950	2.14	1.07	1.07	0	4.28
951	1.07	4.27	1.07	2.14	8.55
952	0	0	1.07	0	1.07
953	0	1.07	1.07	0	2.14
954	1.07	2.14	0	0	3.21
955	2.14	2.14	3.2	0	7.48
956	2.14	1.07	0	0	3.21
957	0	1.07	0	0	1.07
958	2.14	0	0	0	2.14
959	0	2.14	0	0	2.14
960	1.07	0	0	0	1.07
TOTAL	214.74	252.3	73.86	302.28	843.18

STATE: COLORADO

ZIP CODE	QUARTER 1	QUARTER 2	QUARTER 3	QUARTER 4	TOTAL GRAMS
806	0	2.14	3.2	2.14	7.48
808	0	0	2.14	0	2.14
TOTAL	0	2.14	5.34	2.14	9.62

STATE: CONNECTICUT

ZIP CODE	QUARTER 1	QUARTER 2	QUARTER 3	QUARTER 4	TOTAL GRAMS
64	1.07	0	1.07	0	2.14
68	0.13	0.13	1.09	0	1.35
69	0	0	0.27	0	0.27
TOTAL	1.2	0.13	2.43	0	3.76

STATE: DELAWARE

ZIP CODE	QUARTER 1	QUARTER 2	QUARTER 3	QUARTER 4	TOTAL GRAMS
197	3.67	0.43	3.63	2.94	10.67
198	3.14	4.57	4.64	4.7	17.05
199	6.32	8.25	4.17	8.06	26.8
TOTAL	13.13	13.25	12.44	15.7	54.52

STATE: FLORIDA

ZIP CODE	QUARTER 1	QUARTER 2	QUARTER 3	QUARTER 4	TOTAL GRAMS
321	2.14	1.07	2.14	13.89	19.24
323	3.2	3.2	4.27	0	10.67
324	1.07	1.07	0	0	2.14
325	10.09	7.48	11.47	8.83	37.87
326	0	1.07	0	0	1.07
327	1.17	5.71	7.23	9.63	23.74
328	40.27	47.27	147.92	70.88	306.34
329	0	1.07	0	1.07	2.14
330	19.22	1.76	2.56	3.2	26.74
331	29.91	6.14	2.35	6.2	44.6
333	3.85	3.26	1.5	0.64	9.25
334	3.69	0.96	0	5.13	9.78

	335	14.95	21.36	9.61	18.16	64.08
	336	11.75	1.07	1.17	12.18	26.17
	337	1.07	2.35	5.34	22.38	31.14
	338	3.2	23.01	11.46	9.99	47.66
	339	0	0	0	3.52	3.52
	341	2.14	4.27	1.07	2.67	10.15
	342	1.07	1.07	0	2.35	4.49
	344	0	0	2.14	0	2.14
	346	2.14	3.2	4.01	14.85	24.2
	347	2.56	4.49	22.32	10.34	39.71
	349	5.34	6.41	2.14	3.2	17.09
TOTAL		158.83	147.29	238.7	219.11	763.93

STATE: GEORGIA

ZIP CODE		QUARTER 1	QUARTER 2	QUARTER 3	QUARTER 4	TOTAL GRAMS
	300	36	26.06	25.38	63.19	150.63
	301	5.66	4.27	19.55	111.4	140.88
	302	54.76	98.27	70.56	59.13	282.72
	303	9.99	3.63	6.38	17.85	37.85
	304	94.93	43.53	42.73	30.98	212.17
	305	9.29	11.75	15.87	26.11	63.02
	306	9.61	9.61	5.34	6.41	30.97
	307	2.14	3.2	5.34	3.2	13.88
	310	41.02	21.37	68.74	66.6	197.73
	312	9.99	20.87	22.7	24.35	77.91
	313	1.98	21.2	11.75	10.68	45.61
	314	24.79	37.01	33.11	19.23	114.14
	315	2.14	0	0	0	2.14
	316	0.16	0	1.07	4.27	5.5
	317	121.66	241.51	170.42	224.1	757.69
	318	1.07	1.07	0	1.07	3.21
	319	3.2	3.2	2.14	0	8.54
	398	0	3.2	8.55	41.66	53.41
TOTAL		428.39	549.75	509.63	710.23	2,198

STATE: HAWAII

ZIP CODE	QUARTER 1	QUARTER 2	QUARTER 3	QUARTER 4	TOTAL GRAMS
967	1.07	0	0	0	1.07
TOTAL	1.07	0	0	0	1.07

STATE: IDAHO

ZIP CODE	QUARTER 1	QUARTER 2	QUARTER 3	QUARTER 4	TOTAL GRAMS
836	12.82	10.68	5.34	9.61	38.45
837	10.68	29.91	26.7	3.2	70.49
TOTAL	23.5	40.59	32.04	12.81	108.94

STATE: ILLINOIS

ZIP CODE	QUARTER 1	QUARTER 2	QUARTER 3	QUARTER 4	TOTAL GRAMS
600	27.77	45.29	47.21	38.45	158.72
601	9.61	74.13	60.24	48.71	192.69
603	0	0	0	0.09	0.09
604	12.82	16.02	26.28	11.96	67.08
605	1.07	3.2	0	2.14	6.41
606	49.99	67.29	51.27	20.3	188.85
607	12.82	0	0	0	12.82
613	6.41	4.27	0.64	0	11.32
614	1.07	0	0	0	1.07
619	1.07	0	0	0	1.07
624	0	0	4.27	0	4.27
627	2.14	0	0	12.82	14.96
629	2.14	4.27	2.14	3.2	11.75
TOTAL	126.91	214.47	192.05	137.67	671.1

STATE: INDIANA

ZIP CODE	QUARTER 1	QUARTER 2	QUARTER 3	QUARTER 4	TOTAL GRAMS
460	1.07	0	0	0	1.07
468	0	0	1.07	0	1.07
473	0	0	1.07	0	1.07
476	0	0	1.07	1.07	2.14

	477	2.14	0	0	0	2.14
TOTAL		3.21	0	3.21	1.07	7.49

STATE:	IOWA				
ZIP CODE	QUARTER 1	QUARTER 2	QUARTER 3	QUARTER 4	TOTAL GRAMS
500	0	0	0	1.07	1.07
528	0	1.07	0	0	1.07
TOTAL	0	1.07	0	1.07	2.14

STATE:	KANSAS				
ZIP CODE	QUARTER 1	QUARTER 2	QUARTER 3	QUARTER 4	TOTAL GRAMS
661	0	0	0	2.14	2.14
662	1.07	10.68	0	4.27	16.02
TOTAL	1.07	10.68	0	6.41	18.16

STATE:	KENTUCKY				
ZIP CODE	QUARTER 1	QUARTER 2	QUARTER 3	QUARTER 4	TOTAL GRAMS
401	8.55	6.41	3.2	5.34	23.5
402	5.34	0	0	4.27	9.61
405	1.07	0	1.07	0	2.14
406	0	1.07	0	0	1.07
410	1.07	2.19	0	0	3.26
421	0	0	3.2	2.14	5.34
422	6.41	6.41	6.41	6.41	25.64
427	8.55	3.2	0	3.2	14.95
TOTAL	30.99	19.28	13.88	21.36	85.51

STATE:	LOUISIANA				
ZIP CODE	QUARTER 1	QUARTER 2	QUARTER 3	QUARTER 4	TOTAL GRAMS
700	73.66	85.24	65.37	59.28	283.55
701	7.58	7.05	2.14	5.66	22.43
703	34.61	30.76	35.04	26.81	127.22

	704	24.05	47.31	36.13	49.78	157.27
	705	391.27	539.16	438.99	270.5	1,639.92
	706	7.58	2.14	2.33	4.49	16.54
	707	7.99	21.41	6.05	6.02	41.47
	708	35.23	19.72	18.13	13.12	86.2
	710	3.2	8.71	5.34	2.14	19.39
	711	12.39	15.06	11.34	12.48	51.27
	712	21.26	19.77	23.37	31.07	95.47
	713	0	4.27	0	2.14	6.41
	714	2.14	2.14	0	1.07	5.35
TOTAL		620.96	802.74	644.23	484.56	2,552.49

STATE: MAINE

ZIP CODE		QUARTER 1	QUARTER 2	QUARTER 3	QUARTER 4	TOTAL GRAMS
	043	0	0	1.07	0	1.07
TOTAL		0	0	1.07	0	1.07

STATE: MARYLAND

ZIP CODE		QUARTER 1	QUARTER 2	QUARTER 3	QUARTER 4	TOTAL GRAMS
	208	0	4.81	0	3.2	8.01
	210	2.14	1.07	0	1.71	4.92
	211	3.2	1.07	7.48	2.14	13.89
	212	1.07	2.14	1.07	2.14	6.42
	214	0	0	0.21	0.75	0.96
	217	1.07	1.07	0	0	2.14
	219	0	0	1.55	0.21	1.76
TOTAL		7.48	10.16	10.31	10.15	38.1

STATE: MASSACHUSETTS

ZIP CODE		QUARTER 1	QUARTER 2	QUARTER 3	QUARTER 4	TOTAL GRAMS
	18	21.36	16.02	33.11	55.54	126.03
TOTAL		21.36	16.02	33.11	55.54	126.03

DATE RANGE: 01/01/2017 TO 12/31/2017 | ARCOS 3 - REPORT 1 | Run Date: 07/03/2018

RETAIL DRUG DISTRIBUTION BY ZIP CODE WITHIN STATE BY GRAMS WT

STATE: MICHIGAN

ZIP CODE	QUARTER 1	QUARTER 2	QUARTER 3	QUARTER 4	TOTAL GRAMS
480	0.16	0	2.14	1.07	3.37
481	0	0	1.07	0	1.07
482	1.07	0	0	0	1.07
488	0	0	1.07	0	1.07
489	33.01	0	0	0	33.01
494	0	0	1.07	0	1.07
496	0	0	0	0.21	0.21
TOTAL	34.24	0	5.35	1.28	40.87

STATE: MINNESOTA

ZIP CODE	QUARTER 1	QUARTER 2	QUARTER 3	QUARTER 4	TOTAL GRAMS
551	0	2.14	0	2.14	4.28
565	0	2.14	1.07	0	3.21
566	1.71	0	0.64	0	2.35
TOTAL	1.71	4.28	1.71	2.14	9.84

STATE: MISSISSIPPI

ZIP CODE	QUARTER 1	QUARTER 2	QUARTER 3	QUARTER 4	TOTAL GRAMS
386	39.58	49.78	50.1	29.53	168.99
387	20.03	34.02	21.68	16.02	91.75
388	28.2	34.93	32.15	14.95	110.23
389	34.23	30.5	35.2	11.75	111.68
390	63.34	79.74	69.48	78.94	291.5
391	42.25	48.28	37.65	79.58	207.76
392	72.47	94.16	46.3	41.93	254.86
393	4.27	27.51	18.21	14.69	64.68
394	20.94	34.61	16.29	19.23	91.07
395	12.18	12.82	11.75	8.55	45.3
396	8.55	6.41	8.55	13.89	37.4
397	21.52	38.77	32.95	37.55	130.79
TOTAL	367.56	491.53	380.31	366.61	1,606.01

STATE: MISSOURI

ZIP CODE	QUARTER 1	QUARTER 2	QUARTER 3	QUARTER 4	TOTAL GRAMS
630	34.18	28.84	37.39	25.64	126.05
631	2.14	7.48	8.55	5.34	23.51
633	5.34	4.27	4.27	4.27	18.15
641	4.27	0	4.27	0	8.54
646	0	2.14	0	1.07	3.21
656	0	0	0	5.34	5.34
TOTAL	45.93	42.73	54.48	41.66	184.8

STATE: MONTANA

ZIP CODE	QUARTER 1	QUARTER 2	QUARTER 3	QUARTER 4	TOTAL GRAMS
594	1.07	0	0	0	1.07
TOTAL	1.07	0	0	0	1.07

STATE: NEBRASKA

ZIP CODE	QUARTER 1	QUARTER 2	QUARTER 3	QUARTER 4	TOTAL GRAMS
681	1.07	0	0.64	2.78	4.49
685	1.07	1.07	3.2	0	5.34
686	1.07	1.07	0	0	2.14
TOTAL	3.21	2.14	3.84	2.78	11.97

STATE: NEVADA

ZIP CODE	QUARTER 1	QUARTER 2	QUARTER 3	QUARTER 4	TOTAL GRAMS
890	0	2.14	0	0	2.14
891	43.37	28.84	1.07	0	73.28
TOTAL	43.37	30.98	1.07	0	75.42

STATE: NEW JERSEY

ZIP CODE	QUARTER 1	QUARTER 2	QUARTER 3	QUARTER 4	TOTAL GRAMS
70	9.3	5.68	13.25	18.68	46.91
71	229.65	256.36	149.54	122.84	758.39
72	0	0	2.14	0	2.14
73	0.51	0.38	2.24	7.9	11.03
74	2.86	3.12	2.73	3.39	12.1
76	0	0	3.01	4.44	7.45
77	2.16	1.07	1.6	6.09	10.92
79	4.72	0.85	0.51	3.48	9.56
80	54.36	71.43	14.25	8.91	148.95
81	0	0	0.45	0	0.45
82	30.02	22.88	38.03	94.85	185.78
83	1.07	0	0	2.14	3.21
84	0	0	0	1.07	1.07
85	1.67	2.48	0.98	0.26	5.39
86	3.85	0.45	1.58	3.2	9.08
87	15.7	4.49	7.48	6.11	33.78
88	0	0	0	2.14	2.14
89	0	0.19	0	0.21	0.4
TOTAL	355.87	369.38	237.79	285.71	1,248.75

STATE: NEW MEXICO

ZIP CODE	QUARTER 1	QUARTER 2	QUARTER 3	QUARTER 4	TOTAL GRAMS
870	2.14	1.07	0	0	3.21
871	2.14	5.34	12.82	8.55	28.85
874	1.07	0	0	0	1.07
875	13.89	4.27	10.68	7.48	36.32
877	0	1.07	0	0	1.07
880	116.43	76.91	76.91	1.07	271.32
882	0	2.14	0	0	2.14
TOTAL	135.67	90.8	100.41	17.1	343.98

STATE: NEW YORK

ZIP CODE	QUARTER 1	QUARTER 2	QUARTER 3	QUARTER 4	TOTAL GRAMS
100	9.61	65.16	3.2	0	77.97

	103	0	0	0	0.53	0.53
	104	1.07	3.2	0	1.07	5.34
	109	0	0	0	1.07	1.07
	110	1.07	2.14	2.14	1.07	6.42
	111	0	0	2.14	1.07	3.21
	112	34.18	42.73	21.36	49.14	147.41
	113	22.43	37.39	47	51.27	158.09
	114	2.14	0	1.07	3.2	6.41
	115	1.07	2.14	19.23	6.41	28.85
	117	23.5	9.61	8.55	9.61	51.27
	118	0	0	6.41	6.41	12.82
	125	1.07	0	0	1.07	2.14
	130	5.34	0	0	0	5.34
	136	0	5.34	5.34	3.2	13.88
	141	5.34	0	0	0	5.34
	143	2.14	0	0	1.07	3.21
TOTAL		108.96	167.71	116.44	136.19	529.3

STATE: NORTH CAROLINA

ZIP CODE		QUARTER 1	QUARTER 2	QUARTER 3	QUARTER 4	TOTAL GRAMS
	271	0	0	1.07	1.07	2.14
	272	1.07	2.14	1.07	1.07	5.35
	273	0	1.07	0	0	1.07
	275	2.14	0	1.07	0	3.21
	276	0	2.14	0	0	2.14
	278	3.2	2.14	0	0	5.34
	281	0	0	1.07	3.2	4.27
	282	0	0	0	1.07	1.07
	283	1.07	1.07	0	0	2.14
	284	1.07	0	0	0	1.07
	285	0	0	0	1.07	1.07
	286	5.34	1.07	1.07	1.07	8.55
	289	0	0	1.07	0	1.07
TOTAL		13.89	9.63	6.42	8.55	38.49

STATE: OHIO

ZIP CODE	QUARTER 1	QUARTER 2	QUARTER 3	QUARTER 4	TOTAL GRAMS
430	5.34	9.61	4.27	5.34	24.56
432	33.11	8.55	20.3	8.55	70.51
440	1.07	0	0	0	1.07
441	0	2.14	2.14	0	4.28
450	3.2	2.4	0	1.6	7.2
451	0	0	1.6	0	1.6
452	1.87	8.01	4.01	4.01	17.9
453	1.28	0	3.74	1.07	6.09
454	0	0.96	0.8	0	1.76
455	0	0	1.07	0	1.07
456	2.14	2.14	0	0	4.28
TOTAL	48.01	33.81	37.93	20.57	140.32

STATE: OKLAHOMA

ZIP CODE	QUARTER 1	QUARTER 2	QUARTER 3	QUARTER 4	TOTAL GRAMS
730	55.44	62.97	60.05	49.03	227.49
731	97.95	90.63	103.61	75.2	367.39
734	22	26.7	15.27	19.23	83.2
735	25.42	19.23	11.75	20.3	76.7
736	0	2.14	1.07	0	3.21
737	0	3.2	1.07	2.14	6.41
738	1.07	0	0	0	1.07
740	15.06	15.25	18.69	20.46	69.46
741	7.07	11.39	20.66	26.4	65.52
743	18.16	17.09	16.02	12.82	64.09
744	16.54	20.58	32.78	24.03	93.93
745	0	0	1.07	1.07	2.14
746	0	5.34	1.07	0	6.41
747	2.24	4.27	5.34	1.07	12.92
748	11.75	10.68	5.34	7.48	35.25
749	0.11	9.38	1.5	3.2	14.19
TOTAL	272.81	298.85	295.29	262.43	1,129.38

STATE: PENNSYLVANIA

ZIP CODE	QUARTER 1	QUARTER 2	QUARTER 3	QUARTER 4	TOTAL GRAMS
157	2.14	0	1.07	0	3.21
170	1.07	0	0	0	1.07
180	0	1.28	0.98	5.34	7.6
181	0	0	1.15	0.51	1.66
182	4.27	1.07	5.34	2.14	12.82
184	0	0	0	2.14	2.14
186	2.14	0	0	0	2.14
187	1.07	1.07	0	0	2.14
189	0	1.07	0	0	1.07
190	73.88	25.02	43.04	25.68	167.62
191	115.97	10.87	7.97	6.71	141.52
193	5.04	1.74	3.59	5.34	15.71
194	0	0	1.39	2.48	3.87
TOTAL	205.58	42.12	64.53	50.34	362.57

STATE: RHODE ISLAND

ZIP CODE	QUARTER 1	QUARTER 2	QUARTER 3	QUARTER 4	TOTAL GRAMS
28	0	1.07	0	2.14	3.21
TOTAL	0	1.07	0	2.14	3.21

STATE: SOUTH CAROLINA

ZIP CODE	QUARTER 1	QUARTER 2	QUARTER 3	QUARTER 4	TOTAL GRAMS
290	0	0	0	1.07	1.07
291	1.07	1.07	1.07	1.07	4.28
293	2.14	2.14	1.07	2.14	7.49
294	3.2	0	0	2.14	5.34
295	6.41	7.48	4.27	7.48	25.64
296	0	0	1.07	0	1.07
297	1.07	2.14	2.14	0	5.35
298	0	1.07	1.07	0	2.14
TOTAL	13.89	13.9	10.69	13.9	52.38

STATE: SOUTH DAKOTA

ZIP CODE	QUARTER 1	QUARTER 2	QUARTER 3	QUARTER 4	TOTAL GRAMS
572	0	0	0	1.07	1.07
TOTAL	0	0	0	1.07	1.07

STATE: TENNESSEE

ZIP CODE	QUARTER 1	QUARTER 2	QUARTER 3	QUARTER 4	TOTAL GRAMS
370	258.79	210.13	167.7	96.37	732.99
371	15.89	28.75	18.16	9.82	72.62
372	50.8	42.3	43.79	48.22	185.11
373	3.2	2.14	4.27	3.2	12.81
374	9.61	4.27	0	3.2	17.08
377	15.6	92.08	59.6	11.32	178.6
378	48.87	25.85	32.21	30.55	137.48
379	36.17	23.23	54.12	92.45	205.97
380	21.15	19.97	24.35	14.53	80
381	14.47	26.33	25.69	23.07	89.56
382	0.51	2.95	2.14	2.35	7.95
383	24.67	32.21	72.53	64.62	194.03
384	6.41	5.34	3.2	2.14	17.09
385	1.23	1.07	1.07	2.35	5.72
TOTAL	507.37	516.62	508.83	404.19	1,937.01

STATE: TEXAS

ZIP CODE	QUARTER 1	QUARTER 2	QUARTER 3	QUARTER 4	TOTAL GRAMS
750	353.99	307.77	273.43	256.91	1,192.10
751	83.15	75.43	83.57	68.45	310.6
752	227.08	236.8	197.4	142.5	803.78
754	42.26	39.52	42.94	31.28	156
755	26.17	28.84	32.04	25.32	112.37
756	7.69	12.07	6.73	11.06	37.55
757	8.55	5.34	3.2	2.14	19.23
758	2.14	3.2	7.48	7.48	20.3

759	47.69	52.06	25.85	18.39	143.99
760	134.5	171.15	86.92	97.54	490.11
761	96.74	134.43	156.87	295.26	683.3
762	96.24	82.84	100.78	63.18	343.04
763	116.96	114.1	144.33	135.28	510.67
764	13.89	7.48	6.41	3.2	30.98
765	13.89	20.3	14.95	17.09	66.23
766	1.07	5.34	4.27	2.14	12.82
767	0	0	0	2.14	2.14
768	0	0	1.07	5.34	6.41
769	1.39	0.21	0.21	0	1.81
770	500.63	619.89	436.15	503.27	2,059.94
773	96.13	120.56	268.59	312.91	798.19
774	99.86	79.89	64.89	159.63	404.27
775	119.15	141.29	95.71	116.8	472.95
776	23.71	15.38	7.48	26.06	72.63
777	16.66	14.32	4.7	15.49	51.17
778	5.34	6.41	1.07	3.2	16.02
779	8.94	4.7	4.49	12.82	30.95
780	29	28.84	25.64	18.16	101.64
781	50.42	48.28	72.74	59.25	230.69
782	108.85	102.49	55.9	50.04	317.28
783	84.65	86.52	113.01	89.47	373.65
784	164.98	160.01	182.23	108.03	615.25
785	61.1	51.7	129.46	163.11	405.37
786	11.75	7.48	1.07	4.27	24.57
787	7.48	6.41	4.27	9.61	27.77
788	1.07	1.07	0	0	2.14
789	1.17	0	2.14	0	3.31
790	1.07	0	0	0	1.07
791	0	0	2.14	0	2.14
792	1.07	1.07	0	0	2.14
793	2.14	0	0	0	2.14
794	6.41	3.2	4.27	1.07	14.95
796	1.07	1.07	0	0	2.14
797	6.41	10.68	13.89	13.89	44.87
799	5.34	0	1.07	2.14	8.55

TOTAL		2,687.80	2,808.14	2,679.36	2,853.92	11,029.22

STATE:	UTAH					
ZIP CODE		QUARTER 1	QUARTER 2	QUARTER 3	QUARTER 4	TOTAL GRAMS
	841	0	0	1.07	0	1.07
TOTAL		0	0	1.07	0	1.07

STATE:	VERMONT					
ZIP CODE		QUARTER 1	QUARTER 2	QUARTER 3	QUARTER 4	TOTAL GRAMS
	57	0	1.07	0	0	1.07
TOTAL		0	1.07	0	0	1.07

STATE:	VIRGINIA					
ZIP CODE		QUARTER 1	QUARTER 2	QUARTER 3	QUARTER 4	TOTAL GRAMS
	221	0	0	0	1.07	1.07
	226	3.2	0	0	1.07	4.27
	228	0	2.14	2.14	2.14	6.42
	229	2.14	0	0	0	2.14
	230	0	1.07	0	0	1.07
	232	3.2	1.07	0	0	4.27
	234	0	0	1.07	0	1.07
	235	1.07	0	0	3.2	4.27
	236	1.07	0	0	0	1.07
	245	0	0	0	1.07	1.07
TOTAL		10.68	4.28	3.21	8.55	26.72

STATE:	WEST VIRGINIA					
ZIP CODE		QUARTER 1	QUARTER 2	QUARTER 3	QUARTER 4	TOTAL GRAMS
	260	5.34	2.14	0	0	7.48
	265	0	0.21	0	0	0.21
TOTAL		5.34	2.35	0	0	7.69

STATE: WISCONSIN

ZIP CODE	QUARTER 1	QUARTER 2	QUARTER 3	QUARTER 4	TOTAL GRAMS
531	4.27	4.27	5.34	2.14	16.02
532	0	3.2	0	0	3.2
535	0	0	1.07	1.07	2.14
TOTAL	4.27	7.47	6.41	3.21	21.36

DRUG CODE: 9143 DRUG NAME: OXYCODONE

STATE: ALABAMA

ZIP CODE	QUARTER 1	QUARTER 2	QUARTER 3	QUARTER 4	TOTAL GRAMS
350	29,789.33	28,630.79	27,401.23	26,192.96	112,014.31
351	16,285.31	15,074.68	15,191.16	15,139.27	61,690.42
352	23,345.29	22,157.51	21,566.74	21,852.12	88,921.66
354	8,763.44	8,645.65	8,065.72	7,928.77	33,403.58
355	13,165.55	13,076.66	12,564.58	12,249.29	51,056.08
356	21,930.62	20,682.05	19,727.50	19,458.45	81,798.62
357	11,061.92	10,580.64	9,965.46	10,010.97	41,618.99
358	16,204.15	15,565.50	14,520.18	13,776.42	60,066.25
359	20,925.80	19,394.30	18,500.69	19,613.59	78,434.38
360	8,578.75	8,338.33	8,006.03	7,660.58	32,583.69
361	6,094.88	6,037.76	5,132.88	5,000.56	22,266.08
362	11,114.85	10,171.87	9,759.19	9,684.88	40,730.79
363	9,438.82	9,599.10	8,988.36	9,118.79	37,145.07
364	5,113.84	5,105.06	4,216.31	5,014.52	19,449.73
365	19,848.44	18,335.25	16,846.91	16,820.27	71,850.87
366	13,808.94	13,688.42	13,548.65	12,848.94	53,894.95
367	2,648.15	2,403.07	2,254.35	2,256.19	9,561.76
368	6,287.84	5,984.71	5,914.59	5,987.05	24,174.19
369	628.22	614.77	667.44	669.26	2,579.69
TOTAL	245,034.14	234,086.12	222,837.97	221,282.88	923,241.11

STATE: ALASKA

ZIP CODE	QUARTER 1	QUARTER 2	QUARTER 3	QUARTER 4	TOTAL GRAMS
995	19,826.95	19,533.82	18,778.82	18,691.95	76,831.54
996	10,652.08	10,396.10	9,833.01	9,890.24	40,771.43
997	3,553.02	3,457.86	3,472.52	2,912.13	13,395.53
998	1,269.50	1,201.51	1,168.81	1,100.88	4,740.70
999	608.41	517.5	490.7	439.8	2,056.41
TOTAL	35,909.96	35,106.79	33,743.86	33,035	137,795.61

STATE: AMERICAN SAMOA

ZIP CODE	QUARTER 1	QUARTER 2	QUARTER 3	QUARTER 4	TOTAL GRAMS
967	14.34	12.1	10.76	21.52	58.72
TOTAL	14.34	12.1	10.76	21.52	58.72

STATE: ARIZONA

ZIP CODE	QUARTER 1	QUARTER 2	QUARTER 3	QUARTER 4	TOTAL GRAMS
850	97,820.04	91,456.11	86,068.50	84,010.42	359,355.07
851	22,355.40	21,921.31	20,871.70	21,204.52	86,352.93
852	113,162.34	106,998.55	97,836.47	94,617.24	412,614.60
853	84,090.25	77,518.58	73,761.02	72,757.88	308,127.73
855	8,685.15	8,069.04	7,334.34	7,071.28	31,159.81
856	14,364.38	13,734.35	13,008.98	13,248.12	54,355.83
857	68,536.84	65,986.49	64,002.85	62,804.54	261,330.72
859	6,984.83	6,810.24	6,569.16	6,922.18	27,286.41
860	7,220.14	6,809.27	6,365.51	6,087.90	26,482.82
863	13,960.82	13,913.49	13,292.38	12,756.95	53,923.64
864	21,839.49	19,401.73	18,907.39	18,138.50	78,287.11
865	291.36	300.33	257.74	275.67	1,125.10
TOTAL	459,311.04	432,919.49	408,276.04	399,895.20	1,700,401.77

STATE: ARKANSAS

ZIP CODE	QUARTER 1	QUARTER 2	QUARTER 3	QUARTER 4	TOTAL GRAMS
716	4,639.27	4,369.22	4,408.93	4,342.09	17,759.51
717	3,103.90	3,038	2,871.51	2,701.07	11,714.48
718	3,322.80	3,343.09	3,362.69	3,129.13	13,157.71

	719	6,269.45	5,669.43	5,477.76	5,160.95	22,577.59
	720	17,888.14	17,430.40	16,813.54	16,735.65	68,867.73
	721	16,785.75	16,633.40	16,097.51	15,106.30	64,622.96
	722	12,595.52	13,017.89	12,411.20	12,223.51	50,248.12
	723	4,947.87	5,010.23	5,137.27	4,876.14	19,971.51
	724	6,407.17	6,058.76	5,839.41	5,795.42	24,100.76
	725	5,816.53	5,705.79	5,634.29	5,649.07	22,805.68
	726	8,148.99	7,439.84	7,321.47	7,259.90	30,170.20
	727	33,789.07	32,423.69	31,182.23	29,617.50	127,012.49
	728	5,768.08	5,831.11	5,750.73	5,487.01	22,836.93
	729	21,021.09	21,216.58	20,291.65	19,612.66	82,141.98
TOTAL		150,503.63	147,187.43	142,600.19	137,696.40	577,987.65

STATE: CALIFORNIA

ZIP CODE		QUARTER 1	QUARTER 2	QUARTER 3	QUARTER 4	TOTAL GRAMS
	900	26,993.80	26,621.11	26,204.41	24,265.40	104,084.72
	902	16,748.92	16,630.90	16,084.64	16,141.05	65,605.51
	903	1,239.70	1,215.35	1,427.24	1,289.18	5,171.47
	904	4,921.01	4,891.87	4,920.79	5,013.74	19,747.41
	905	3,907.79	3,989.17	3,730.84	3,652.29	15,280.09
	906	8,684.15	7,956.90	7,794.22	7,567.49	32,002.76
	907	8,977.89	9,030.32	9,139.76	8,468.77	35,616.74
	908	7,058.19	6,469.63	6,226.69	5,797.22	25,551.73
	910	5,140.67	5,262.59	4,997	4,324.78	19,725.04
	911	2,886.67	2,997.48	2,908.41	2,853.87	11,646.43
	912	3,628.52	3,329.35	3,397.49	3,270.24	13,625.60
	913	33,102.07	31,982.62	31,331.52	30,635.12	127,051.33
	914	8,467.49	8,808.70	8,449.28	8,980.74	34,706.21
	915	3,834.17	3,514.81	3,573.46	3,336.86	14,259.30
	916	2,230.08	1,935.75	1,828.81	1,849.84	7,844.48
	917	28,285.33	27,739.72	27,270.29	25,868.10	109,163.44
	918	438.84	498.23	500.02	465.26	1,902.35
	919	12,859.10	13,044.15	12,319.32	11,860.38	50,082.95
	920	55,312.42	53,778.20	52,443.25	50,287.18	211,821.05
	921	37,417.13	35,167.06	34,229.01	32,502.15	139,315.35

922	31,436.40	30,022.88	28,844.25	28,144.60	118,448.13
923	33,315.16	32,816.02	32,349.35	31,074.27	129,554.80
924	5,114.22	5,342.18	5,155.20	5,250.74	20,862.34
925	32,543.54	32,174.82	30,555.56	30,247.56	125,521.48
926	30,259.09	29,399.79	28,648.98	27,962.58	116,270.44
927	6,204.33	6,470.96	5,636.40	5,283.70	23,595.39
928	25,069.36	24,333.50	23,312.08	22,888.31	95,603.25
930	18,642.50	18,232.61	17,398.98	16,609.46	70,883.55
931	3,157.93	3,187.06	2,811.16	2,702.27	11,858.42
932	10,671.63	10,167.02	10,411.43	10,163.43	41,413.51
933	14,513.41	14,103.70	13,872.18	13,607.71	56,097
934	16,433.35	15,534.56	15,137.79	14,297.66	61,403.36
935	23,227.78	22,448.56	21,424.35	21,623.23	88,723.92
936	12,680.05	12,788.21	12,073.27	11,524.62	49,066.15
937	14,594.84	14,564.68	14,324.16	14,084.09	57,567.77
939	7,977.98	7,689.27	7,332.84	6,714.69	29,714.78
940	14,276.82	13,738.46	13,880.60	13,584.93	55,480.81
941	24,862.07	23,735.45	22,912.49	22,875.33	94,385.34
943	3,969.55	3,640.80	3,265.98	3,133.95	14,010.28
944	2,429.69	2,222.52	2,237.07	2,137.19	9,026.47
945	58,725.70	56,371.42	54,772.63	52,056.46	221,926.21
946	7,132.42	6,661.65	5,853.84	5,594.49	25,242.40
947	1,589.05	1,476.90	1,615.77	1,626.81	6,308.53
948	1,936.39	1,887.64	1,927.05	1,808.61	7,559.69
949	10,955.98	11,087.04	10,685.77	10,517.30	43,246.09
950	15,054.45	14,610.97	13,478.07	13,231.12	56,374.61
951	10,308.65	9,738.87	9,707.74	9,359.97	39,115.23
952	11,613.35	11,275.85	10,741.41	9,807.05	43,437.66
953	40,167.97	39,429.06	38,433.67	36,833.37	154,864.07
954	26,075.09	25,407.62	23,536.43	21,771.02	96,790.16
955	6,822.33	6,869.11	6,415.89	6,194.81	26,302.14
956	37,813.87	35,526.49	34,344.40	33,785.19	141,469.95
957	6,866.11	6,481.29	6,115.23	6,040.75	25,503.38
958	23,831.90	23,954.98	22,443.25	22,241.99	92,472.12
959	25,443.40	24,583.66	22,906.14	21,960.28	94,893.48
960	13,807.70	12,680.93	12,282	11,633.83	50,404.46
961	3,660.75	3,378.68	3,181.69	3,009.83	13,230.95

TOTAL	905,318.75	878,899.12	848,801.55	819,812.86	3,452,832.28

STATE: COLORADO

ZIP CODE	QUARTER 1	QUARTER 2	QUARTER 3	QUARTER 4	TOTAL GRAMS
800	38,570.68	37,557.23	35,810.85	34,826.69	146,765.45
801	27,148.98	26,568.62	25,370.52	26,258.44	105,346.56
802	46,177.90	45,987.03	43,966.09	42,189.81	178,320.83
803	4,598.36	4,332	3,799.29	3,594.25	16,323.90
804	6,529.08	6,437.87	6,200.12	6,364.97	25,532.04
805	21,112.47	20,517.25	19,098.91	18,702.60	79,431.23
806	10,740.80	10,522.02	9,931.54	10,006.05	41,200.41
807	2,246.90	2,210.84	2,078.13	2,133.73	8,669.60
808	5,486.77	5,413.05	5,078.76	5,113.01	21,091.59
809	29,035.05	28,312.13	27,690.80	26,687.81	111,725.79
810	20,698.20	20,621.65	19,622.63	19,252.05	80,194.53
811	3,159.88	2,381.55	2,456.04	2,261.31	10,258.78
812	3,693.77	3,567.30	3,371.32	3,117.88	13,750.27
813	1,686.21	1,574.64	1,671.60	1,479.31	6,411.76
814	2,545.96	2,511.92	2,406.60	2,332.44	9,796.92
815	6,025.55	5,953.22	5,234.63	5,167.96	22,381.36
816	4,097.32	3,784.93	3,756.83	3,766.91	15,405.99
TOTAL	233,553.88	228,253.25	217,544.66	213,255.22	892,607.01

STATE: CONNECTICUT

ZIP CODE	QUARTER 1	QUARTER 2	QUARTER 3	QUARTER 4	TOTAL GRAMS
60	32,191.37	31,126.43	29,398.55	27,515.35	120,231.70
61	9,039.01	9,125.32	8,860.27	8,246.86	35,271.46
62	5,263.50	4,846.78	4,689.37	4,366.11	19,165.76
63	14,415.65	14,504.69	14,152.20	12,964.90	56,037.44
64	47,393.93	45,703.96	42,786.99	42,604.68	178,489.56
65	17,426.61	17,028.21	15,973.40	16,801.97	67,230.19
66	10,895.05	10,798	9,996.72	10,031.78	41,721.55
67	19,876.66	18,682.01	17,136.34	17,067.11	72,762.12
68	16,381.99	16,107.02	14,990.50	14,945.10	62,424.61

	69	2,530.28	2,657.73	2,459.04	2,479.25	10,126.30
TOTAL		175,414.05	170,580.15	160,443.38	157,023.11	663,460.69

STATE: DELAWARE

ZIP CODE		QUARTER 1	QUARTER 2	QUARTER 3	QUARTER 4	TOTAL GRAMS
	197	25,785.21	24,403.34	23,442.69	23,666.38	97,297.62
	198	15,735.70	14,870.84	14,636.04	14,135.64	59,378.22
	199	30,538.56	29,348.89	28,676.51	28,567.77	117,131.73
TOTAL		72,059.47	68,623.07	66,755.24	66,369.79	273,807.57

STATE: DISTRICT OF COLUMBIA

ZIP CODE		QUARTER 1	QUARTER 2	QUARTER 3	QUARTER 4	TOTAL GRAMS
	200	13,862.86	13,929.59	13,387.86	12,537.71	53,718.02
	203	63.2	41.24	25.55	8.97	138.96
	204	1,185.80	1,072.12	1,145.64	893.18	4,296.74
	205	1.34	0	0.9	0	2.24
TOTAL		15,113.20	15,042.95	14,559.95	13,439.86	58,155.96

STATE: FLORIDA

ZIP CODE		QUARTER 1	QUARTER 2	QUARTER 3	QUARTER 4	TOTAL GRAMS
	320	35,269.44	34,882.46	33,540.32	33,680.45	137,372.67
	321	31,127.34	30,124.09	28,608.90	30,509.14	120,369.47
	322	64,163.16	63,726.14	62,909.21	63,131.08	253,929.59
	323	13,168.98	13,219.87	12,676.56	12,944	52,009.41
	324	16,257.04	15,952.46	15,148.72	16,083.35	63,441.57
	325	31,504.49	31,077.23	30,160.78	29,796.30	122,538.80
	326	24,188.49	24,080.41	23,411.47	23,542.77	95,223.14
	327	45,952.58	46,277.32	44,703.74	45,627.35	182,560.99
	328	30,000.83	29,648.36	28,773.59	29,418.26	117,841.04
	329	55,015.84	53,381.09	52,434.69	53,606.99	214,438.61
	330	66,921.29	68,438.85	64,967.67	69,053.38	269,381.19
	331	55,407.98	54,837.02	54,580.02	58,444.26	223,269.28
	333	46,263.16	46,158.93	46,793.70	48,575.53	187,791.32

	334	83,210.52	83,015	81,632.12	83,222.19	331,079.83
	335	41,636.02	40,917.26	39,846.72	42,199.85	164,599.85
	336	58,394.15	58,661.86	57,432.21	57,647.53	232,135.75
	337	52,622.73	51,830.13	51,305.33	52,131.76	207,889.95
	338	34,394.80	32,752.11	30,438.30	34,076.06	131,661.27
	339	46,502.15	45,075.71	41,729.06	45,745.04	179,051.96
	341	18,546.75	17,719.56	16,932.75	18,733.41	71,932.47
	342	51,537.84	50,297.84	46,560.36	50,189.95	198,585.99
	344	32,343.35	32,857.87	31,223.73	33,192.30	129,617.25
	346	50,400.99	50,727.21	48,980.95	50,739.24	200,848.39
	347	25,826.64	24,562.46	22,987.68	25,165.82	98,542.60
	349	29,980.31	29,141.47	28,585.19	30,250.12	117,957.09
TOTAL		1,040,636.87	1,029,362.71	996,363.77	1,037,706.13	4,104,069.48

STATE: GEORGIA

ZIP CODE		QUARTER 1	QUARTER 2	QUARTER 3	QUARTER 4	TOTAL GRAMS
	300	80,080.25	79,569.02	78,361.46	79,018.01	317,028.74
	301	69,406.49	69,440.67	68,052.62	67,732.63	274,632.41
	302	41,502.82	40,988.79	40,872.38	40,828.31	164,192.30
	303	28,428.48	28,780.22	27,315.74	26,961.14	111,485.58
	304	10,286.27	10,115.76	9,743.80	9,892.50	40,038.33
	305	39,437.12	39,528.43	38,077.03	38,350.24	155,392.82
	306	27,952.08	27,980.56	27,710.74	28,471.92	112,115.30
	307	19,549.39	18,804.03	18,194.75	17,358.53	73,906.70
	308	8,159.09	7,703.45	7,278.83	7,632.80	30,774.17
	309	17,309.37	16,089.57	15,658.87	15,175.38	64,233.19
	310	26,029.56	26,346.63	24,964.64	25,268.61	102,609.44
	312	12,528.42	12,247	12,360.09	12,005.78	49,141.29
	313	12,640.05	12,204.21	10,834.05	9,128.58	44,806.89
	314	10,812.66	10,448.39	10,158.20	9,976.52	41,395.77
	315	20,312.54	20,516.42	19,901.87	18,427.73	79,158.56
	316	6,727.58	6,486.91	6,330.60	6,966.73	26,511.82
	317	12,958.99	12,804.33	12,130.63	12,773.92	50,667.87
	318	906.79	916.35	943.75	913.35	3,680.24
	319	7,764.38	7,774.27	7,968	6,495.50	30,002.15
	398	2,731.15	2,784.03	2,666.05	2,670.47	10,851.70

TOTAL		455,523.48	451,529.04	439,524.10	436,048.65	1,782,625.27

STATE:	GUAM					
ZIP CODE		QUARTER 1	QUARTER 2	QUARTER 3	QUARTER 4	TOTAL GRAMS
	969	697.48	908.57	980.23	835.09	3,421.37
TOTAL		697.48	908.57	980.23	835.09	3,421.37

STATE:	HAWAII					
ZIP CODE		QUARTER 1	QUARTER 2	QUARTER 3	QUARTER 4	TOTAL GRAMS
	967	32,036.17	30,560.72	29,798.67	28,221.53	120,617.09
	968	13,571.25	13,465.52	13,419.34	12,357.53	52,813.64
TOTAL		45,607.42	44,026.24	43,218.01	40,579.06	173,430.73

STATE:	IDAHO					
ZIP CODE		QUARTER 1	QUARTER 2	QUARTER 3	QUARTER 4	TOTAL GRAMS
	832	6,491.97	6,386.24	6,269.95	6,374.24	25,522.40
	833	7,171.66	7,015.35	6,983.06	6,665.87	27,835.94
	834	5,983.88	5,886.70	5,672.78	5,650.20	23,193.56
	835	2,009.05	1,884.76	1,898.35	1,811.66	7,603.82
	836	18,276.40	18,288.65	18,011.76	17,843.22	72,420.03
	837	13,485.84	13,207.94	13,360.08	12,893.64	52,947.50
	838	8,040.48	7,958.41	8,062.23	7,768.88	31,830
TOTAL		61,459.28	60,628.05	60,258.21	59,007.71	241,353.25

STATE:	ILLINOIS					
ZIP CODE		QUARTER 1	QUARTER 2	QUARTER 3	QUARTER 4	TOTAL GRAMS
	600	26,360.75	25,865.97	24,851.11	24,022.09	101,099.92
	601	16,626.99	15,895.41	15,013.04	14,851.68	62,387.12
	602	571.52	542.41	560.62	489.87	2,164.42
	603	558.12	643.06	529.7	530.84	2,261.72
	604	23,206.59	22,321.68	20,626.17	20,900.79	87,055.23
	605	11,072.87	10,790.88	10,411.04	10,156.61	42,431.40
	606	13,574.04	13,406.78	13,201.32	13,172.86	53,355

	607	1,449.38	1,548.21	1,535.02	1,352.66	5,885.27
	608	928.19	704.52	720.21	660.05	3,012.97
	609	3,004.17	3,071.56	2,839.21	2,827.71	11,742.65
	610	3,279.84	3,135	3,063.84	2,988.92	12,467.60
	611	3,633.97	3,546.97	3,380.87	3,592.14	14,153.95
	612	1,886.04	1,743.77	1,692.92	1,564.62	6,887.35
	613	1,827.81	1,750.30	1,670.05	1,852.98	7,101.14
	614	1,417.37	1,328.93	1,301.05	1,252.16	5,299.51
	615	2,579.64	2,412.10	2,282.22	2,126.37	9,400.33
	616	3,082.88	2,951.32	2,784.23	2,738.38	11,556.81
	617	2,427.10	2,404.80	2,200.31	2,194.68	9,226.89
	618	3,845.95	3,807.11	3,970.23	3,746.82	15,370.11
	619	932.94	887.98	894.89	863.05	3,578.86
	620	9,149.70	8,689.01	8,244.65	8,613.02	34,696.38
	622	10,153.35	9,767.73	9,584.40	9,247.10	38,752.58
	623	1,979.92	2,022.96	1,892.53	1,847.66	7,743.07
	624	2,198.94	2,117.99	2,092.19	2,080.39	8,489.51
	625	1,999.93	2,018.34	1,860.13	2,049.83	7,928.23
	626	2,298.32	2,009.36	2,059.80	2,020.17	8,387.65
	627	1,761.53	1,599.13	1,661.56	1,578.35	6,600.57
	628	4,923.32	4,854.62	4,703.47	4,688.46	19,169.87
	629	8,917.27	8,825.05	8,548.41	8,116.66	34,407.39
TOTAL		165,648.44	160,662.95	154,175.19	152,126.92	632,613.50

STATE: INDIANA

ZIP CODE	QUARTER 1	QUARTER 2	QUARTER 3	QUARTER 4	TOTAL GRAMS
460	13,315.83	13,099.48	12,494.55	12,372.35	51,282.21
461	18,720.37	17,834.81	16,892.37	16,813.58	70,261.13
462	42,099.76	41,528.52	39,828.32	40,819.78	164,276.38
463	19,237.20	18,461.57	17,314.72	16,622.48	71,635.97
464	2,024.39	1,983.79	1,953.72	1,811.60	7,773.50
465	18,557.13	18,358.04	17,015.58	17,174.43	71,105.18
466	7,357.54	7,643.49	7,033.52	6,791.08	28,825.63
467	8,447.81	8,280.04	7,913.89	7,891.43	32,533.17
468	11,366.91	11,045.37	10,738.41	10,284.46	43,435.15
469	10,457.80	10,030.13	9,416.02	9,095.82	38,999.77

	470	7,073.84	7,527.54	7,116.96	7,517.28	29,235.62
	471	17,913.99	18,491.43	17,161.91	15,510.01	69,077.34
	472	7,931.82	7,433.46	7,080.63	7,118.56	29,564.47
	473	13,280.37	12,507.16	12,447.26	12,117.49	50,352.28
	474	8,854.62	8,243.54	7,828.03	7,698.81	32,625
	475	5,720.92	5,480.60	5,106.10	5,115.27	21,422.89
	476	4,435.86	4,164.25	4,135.75	4,084.79	16,820.65
	477	11,913.88	11,917.13	11,270.88	10,939.22	46,041.11
	478	3,483.13	3,231.17	3,122.84	3,139.84	12,976.98
	479	9,384.11	9,204.57	8,908.27	8,566.86	36,063.81
TOTAL		241,577.28	236,466.09	224,779.73	221,485.14	924,308.24

STATE: IOWA

ZIP CODE		QUARTER 1	QUARTER 2	QUARTER 3	QUARTER 4	TOTAL GRAMS
	500	4,770.32	4,623.21	4,406.12	4,458.01	18,257.66
	501	3,412.74	3,233.23	3,118.26	3,158.08	12,922.31
	502	3,723.85	3,555.71	3,396.58	3,368.84	14,044.98
	503	10,967.18	10,505.18	10,434.83	10,417.04	42,324.23
	504	1,973.51	2,017.63	1,671.60	1,797.29	7,460.03
	505	1,636.16	1,671.89	1,517.86	1,553.70	6,379.61
	506	1,602.07	1,557.71	1,467.93	1,537.24	6,164.95
	507	1,672.53	1,572.84	1,412.77	1,428.76	6,086.90
	508	439.24	446.59	448.61	477.26	1,811.70
	510	698.88	791.28	721.97	718.93	2,931.06
	511	1,341.75	1,393.76	1,452.01	1,538.17	5,725.69
	512	466.27	443.77	381.06	378.59	1,669.69
	513	538.02	506.87	529.5	450.75	2,025.14
	514	820.36	805.69	690.32	798.96	3,115.33
	515	3,874.49	3,697.65	3,240.46	3,153.45	13,966.05
	516	448.34	477.7	445.96	485.68	1,857.68
	520	1,461.70	1,272.96	1,223.90	1,210	5,168.56
	521	670.51	572.77	489.98	561.88	2,295.14
	522	2,950.48	2,897.73	2,741.67	2,850.71	11,440.59
	523	1,418.76	1,340.31	1,232.13	1,265.41	5,256.61
	524	2,777.37	2,817.30	2,624.32	2,703.25	10,922.24

525	2,127.35	2,034.37	2,125.55	2,181.47	8,468.74
526	2,138.82	2,179.48	2,077.84	2,077.01	8,473.15
527	1,984.65	1,843.90	1,642.41	1,739.73	7,210.69
528	2,131.40	1,973.20	1,807.62	1,932.74	7,844.96
TOTAL	56,046.75	54,232.73	51,301.26	52,242.95	213,823.69

STATE: KANSAS

ZIP CODE	QUARTER 1	QUARTER 2	QUARTER 3	QUARTER 4	TOTAL GRAMS
660	17,228.91	16,851.95	16,985.87	16,859.87	67,926.60
661	8,673.22	8,280.74	7,956.36	7,912.14	32,822.46
662	15,430.25	15,260.52	14,592.71	14,924.02	60,207.50
664	2,799.12	2,480.30	2,528.76	2,167.01	9,975.19
665	2,861.62	3,100.41	2,967.35	2,957.42	11,886.80
666	6,873.50	6,899.27	6,157.09	6,027.50	25,957.36
667	5,461.66	5,634.48	5,474.84	5,278.83	21,849.81
668	1,962.11	1,961.08	1,864.62	1,665.74	7,453.55
669	710.97	718.5	673.5	683.08	2,786.05
670	8,974.39	9,052.07	8,803.44	8,551.90	35,381.80
671	3,963.87	4,030.08	4,241.60	3,709.89	15,945.44
672	26,000.47	26,366.39	25,877.83	25,731.05	103,975.74
673	2,409.44	2,470.61	2,423.96	2,473.06	9,777.07
674	5,312.98	5,386.19	5,561.29	5,252.69	21,513.15
675	6,159.25	5,719.70	5,472.82	5,381.25	22,733.02
676	2,681.48	2,518.58	2,699.08	2,561.94	10,461.08
677	1,337.07	1,436.78	1,315.84	1,481.32	5,571.01
678	4,459.73	4,454.85	4,495.91	4,386.01	17,796.50
679	1,174.36	1,097.95	1,129.32	1,075.68	4,477.31
TOTAL	124,474.40	123,720.45	121,222.19	119,080.40	488,497.44

STATE: KENTUCKY

ZIP CODE	QUARTER 1	QUARTER 2	QUARTER 3	QUARTER 4	TOTAL GRAMS
400	8,649.42	8,614.20	7,947.11	7,958.64	33,169.37
401	4,439.89	4,446.65	3,894.01	4,027.82	16,808.37
402	37,021.50	35,923.74	34,662.27	34,697.89	142,305.40

	403	14,545.63	14,587.12	13,572.05	13,871.78	56,576.58
	404	7,733.55	7,784.02	7,406.87	7,385.60	30,310.04
	405	12,563.98	11,801.45	11,741.84	11,224.34	47,331.61
	406	1,458.56	1,486.45	1,396.63	1,432.16	5,773.80
	407	6,405.01	6,663.52	6,455.96	6,442.13	25,966.62
	408	1,238.25	1,227.26	1,227.54	1,194.59	4,887.64
	409	3,682.60	3,426.06	3,620.12	3,626.72	14,355.50
	410	30,478.38	29,580.49	28,051.07	28,321.90	116,431.84
	411	5,953.06	5,392.14	4,912.27	4,668.70	20,926.17
	412	2,077.70	2,139.77	2,001.17	2,163.90	8,382.54
	413	1,561.59	1,556.86	1,563.36	1,732.44	6,414.25
	414	830.61	841.59	779.06	814.3	3,265.56
	415	3,556.24	3,820.35	3,619.84	3,704.06	14,700.49
	416	3,097.12	2,984.87	3,052.90	3,105.23	12,240.12
	417	4,419.37	4,375.70	4,262.38	4,233.68	17,291.13
	418	1,798.42	1,809.05	1,719.98	1,760.79	7,088.24
	420	12,616.49	12,449.32	12,104.29	12,281.52	49,451.62
	421	10,380.44	10,070.76	10,282.41	10,115.61	40,849.22
	422	6,382.14	6,331.68	6,508.51	5,941.24	25,163.57
	423	7,385.45	7,311.09	6,934.57	6,847.28	28,478.39
	424	8,606.37	8,442.92	8,028.99	8,014.94	33,093.22
	425	2,578.86	2,743.65	2,550.40	2,965.87	10,838.78
	426	3,655.86	3,432.18	3,719.71	3,505.38	14,313.13
	427	7,574.28	7,494.38	7,368.14	6,831.81	29,268.61
TOTAL		210,690.77	206,737.27	199,383.45	198,870.32	815,681.81

STATE: LOUISIANA

ZIP CODE		QUARTER 1	QUARTER 2	QUARTER 3	QUARTER 4	TOTAL GRAMS
	700	38,533.60	36,003.49	32,969.74	32,039.88	139,546.71
	701	13,990	13,980.22	13,321.14	13,061.44	54,352.80
	703	9,696.27	9,410.14	8,980.66	8,463.08	36,550.15
	704	30,016.34	28,386.23	27,701.56	26,441.66	112,545.79
	705	22,011.24	22,002.08	21,198.66	20,931.98	86,143.96
	706	6,649.97	6,452.10	6,348.63	6,196.58	25,647.28
	707	22,502.06	21,617.31	20,774.82	20,556.03	85,450.22
	708	15,788.16	15,163.43	14,810	14,419.72	60,181.31

	710	3,812.50	3,797.31	3,735.77	3,924.29	15,269.87
	711	14,858.35	14,602.02	14,397.98	14,179.93	58,038.28
	712	7,177.17	6,901.86	6,678.16	6,335.40	27,092.59
	713	12,403.35	11,902.59	11,465.91	11,112.82	46,884.67
	714	6,239.56	5,827.53	5,360.35	5,107.95	22,535.39
TOTAL		203,678.57	196,046.31	187,743.38	182,770.76	770,239.02

STATE: MAINE

ZIP CODE		QUARTER 1	QUARTER 2	QUARTER 3	QUARTER 4	TOTAL GRAMS
	039	985.48	771.39	810.93	748.04	3,315.84
	040	14,683.11	13,702.52	13,602.34	13,284.42	55,272.39
	041	3,859.76	3,459.40	3,316.78	3,288.32	13,924.26
	042	6,133.49	5,935.37	5,886.86	5,972.56	23,928.28
	043	7,122.56	6,913.49	6,843.73	6,752.96	27,632.74
	044	5,695.31	5,295.34	4,909.85	4,918.43	20,818.93
	045	1,475.63	1,514.93	1,537.58	1,387.80	5,915.94
	046	2,941.05	2,593.72	2,453.17	2,331.77	10,319.71
	047	1,896.54	1,868.34	1,635.47	1,643.89	7,044.24
	048	1,182.02	1,197.56	1,206.84	1,184.54	4,770.96
	049	8,186.17	7,787.83	7,854.97	7,535.41	31,364.38
TOTAL		54,161.12	51,039.89	50,058.52	49,048.14	204,307.67

STATE: MARYLAND

ZIP CODE		QUARTER 1	QUARTER 2	QUARTER 3	QUARTER 4	TOTAL GRAMS
	206	25,821.83	25,131.23	23,476.59	22,999.35	97,429
	207	29,375.27	29,545.13	28,383.13	27,183.54	114,487.07
	208	24,323.84	23,986.81	22,431.08	21,199.46	91,941.19
	209	4,718.57	4,738.53	4,186.81	4,345.44	17,989.35
	210	55,543.20	54,174.71	50,978.92	49,398.14	210,094.97
	211	26,669.07	25,241.95	23,251.80	22,255.71	97,418.53
	212	105,152.54	101,300.49	95,119.43	95,180.40	396,752.86
	214	4,750.10	4,473.73	4,076.89	4,144.33	17,445.05
	215	9,038.34	8,522.51	7,944.14	7,508.14	33,013.13
	216	10,523.70	10,395.78	9,910.97	9,797.71	40,628.16
	217	29,801.14	28,455.47	27,305.82	27,091.67	112,654.10

	218	9,715.03	9,040.24	9,087.01	8,504.23	36,346.51
	219	7,961.29	7,407.72	6,788.86	7,019.70	29,177.57
TOTAL		343,393.92	332,414.30	312,941.45	306,627.82	1,295,377.49

STATE:	MASSACHUSETTS				
ZIP CODE	QUARTER 1	QUARTER 2	QUARTER 3	QUARTER 4	TOTAL GRAMS
010	26,189.68	25,598.40	24,110.76	23,949.31	99,848.15
011	10,387.39	10,243.14	9,694.26	9,186.94	39,511.73
012	4,270.86	4,184.70	4,037.03	4,005.21	16,497.80
013	3,961.91	4,075.64	4,012.18	3,819.60	15,869.33
014	8,323.48	7,855.42	7,545.98	7,425.12	31,150
015	11,702.07	11,091.49	11,096.50	10,900.60	44,790.66
016	10,023.36	9,384.89	8,945.29	8,674.25	37,027.79
017	9,706.18	9,423.24	8,795.97	8,711.65	36,637.04
018	33,128.24	31,667.10	29,581.90	28,614.60	122,991.84
019	16,796.53	16,627.35	15,327.50	15,276.29	64,027.67
020	11,428.71	11,212.91	10,773.33	10,427.67	43,842.62
021	37,608.22	35,967.49	34,866.89	34,124.48	142,567.08
022	3,088.19	2,567.84	2,484.22	2,406.88	10,547.13
023	19,686.92	18,674.98	17,620.23	16,919.09	72,901.22
024	5,840.91	5,761.96	5,193.07	5,468.09	22,264.03
025	4,828.31	4,988.12	4,622.47	4,733.18	19,172.08
026	6,794.48	6,674.81	6,577.90	6,401.11	26,448.30
027	29,359.63	28,622.17	27,891.50	27,369.65	113,242.95
TOTAL	253,125.07	244,621.65	233,176.98	228,413.72	959,337.42

STATE:	MICHIGAN				
ZIP CODE	QUARTER 1	QUARTER 2	QUARTER 3	QUARTER 4	TOTAL GRAMS
480	52,416.25	50,719.33	50,184.96	47,948.08	201,268.62
481	58,417.69	57,343.20	54,639.13	51,916.98	222,317
482	43,868.87	41,311.03	39,713.05	39,263.97	164,156.92
483	37,979.52	36,402.70	35,060.27	32,945.43	142,387.92
484	9,029.08	8,132.97	7,922.50	7,920.70	33,005.25
485	4,466.75	4,406.50	4,112.56	3,758.48	16,744.29
486	10,574.83	9,379.09	8,685.53	7,971.73	36,611.18

	487	5,130.57	4,831.10	4,319.07	4,063.28	18,344.02
	488	11,524.10	10,966.65	11,003.61	10,656.94	44,151.30
	489	5,133.75	4,884.56	4,955.39	4,782.45	19,756.15
	490	19,435.95	19,007.19	17,937.46	16,819.03	73,199.63
	491	2,193.24	2,098.76	1,929.89	1,967.45	8,189.34
	492	10,575.87	10,736.14	10,423.95	9,968.72	41,704.68
	493	3,483.09	3,234.70	3,359.27	2,941	13,018.06
	494	7,822.81	7,571.99	7,509.68	6,862.22	29,766.70
	495	7,942.63	8,083.84	7,509.54	7,329.60	30,865.61
	496	9,399.12	9,495.37	9,244.21	8,645.36	36,784.06
	497	8,630.99	8,560.76	7,875.61	7,622.70	32,690.06
	498	4,505.46	4,495.35	4,499.06	4,247.47	17,747.34
	499	1,625.36	1,486.65	1,524.89	1,484.69	6,121.59
TOTAL		314,155.93	303,147.88	292,409.63	279,116.28	1,188,829.72

STATE: MINNESOTA

ZIP CODE		QUARTER 1	QUARTER 2	QUARTER 3	QUARTER 4	TOTAL GRAMS
	550	16,667.86	16,265.54	15,954.72	15,629.56	64,517.68
	551	27,029.38	25,667.01	24,960.50	24,793.80	102,450.69
	553	23,565.45	22,558.32	21,865.78	22,128.72	90,118.27
	554	40,706.29	39,099.53	37,755.15	36,727.89	154,288.86
	556	228.21	221.36	191.54	193.82	834.93
	557	4,982.29	4,862.26	4,634.63	4,448.92	18,928.10
	558	2,778.09	2,842.26	2,618.99	2,563.14	10,802.48
	559	7,704.30	7,357.46	6,925.91	6,838.13	28,825.80
	560	4,540.52	4,489.69	4,425.57	4,348.38	17,804.16
	561	1,271.56	1,216.12	1,228.92	1,149.33	4,865.93
	562	2,562.78	2,384.27	2,304.84	2,205.68	9,457.57
	563	7,953.36	7,517.03	7,219	6,941.15	29,630.54
	564	3,679.94	3,713.70	3,399.84	3,434.30	14,227.78
	565	3,185.66	3,108.63	2,981.83	2,838.27	12,114.39
	566	1,398.86	1,388.52	1,395.21	1,276.85	5,459.44
	567	1,596.48	1,539.18	1,600.57	1,570.82	6,307.05
TOTAL		149,851.03	144,230.88	139,463	137,088.76	570,633.67

STATE: MISSISSIPPI

ZIP CODE	QUARTER 1	QUARTER 2	QUARTER 3	QUARTER 4	TOTAL GRAMS
386	14,208.29	14,261.18	13,976.26	14,032.87	56,478.60
387	1,811.02	1,867.41	1,725.63	1,658.39	7,062.45
388	9,210.33	9,120.89	8,912.44	9,092.61	36,336.27
389	2,091.32	1,908.41	1,784.61	1,648.28	7,432.62
390	6,321.69	6,055.35	5,795.75	5,522.18	23,694.97
391	5,427.66	5,403.04	5,170.79	4,823.58	20,825.07
392	6,455.44	6,521.21	6,388.40	6,348.37	25,713.42
393	6,996.80	6,720.41	6,449.34	6,274.22	26,440.77
394	22,046.67	21,442.10	20,830.99	20,471.75	84,791.51
395	25,294.90	24,584.22	23,162.13	22,638.94	95,680.19
396	5,572.93	5,361.92	4,889.05	4,605.72	20,429.62
397	3,889.32	3,698.54	3,429.91	3,351.27	14,369.04
TOTAL	109,326.37	106,944.68	102,515.30	100,468.18	419,254.53

STATE: MISSOURI

ZIP CODE	QUARTER 1	QUARTER 2	QUARTER 3	QUARTER 4	TOTAL GRAMS
630	36,287.42	35,671.94	33,260.63	33,345.88	138,565.87
631	60,759.28	61,572.24	57,727.85	56,255.09	236,314.46
633	19,261.95	18,624.91	17,146.60	16,786.93	71,820.39
634	1,510.20	1,529.41	1,468.74	1,507.30	6,015.65
635	2,477.34	2,435.49	2,307.16	2,144.52	9,364.51
636	11,110.04	11,730.02	11,099.30	10,727.11	44,666.47
637	5,964.25	5,707.20	5,439.53	5,070.70	22,181.68
638	6,042.73	6,092.27	5,525.70	5,260.27	22,920.97
639	2,932.55	2,695.46	2,618.09	2,453.86	10,699.96
640	26,240.93	25,743.97	24,292.37	23,930.74	100,208.01
641	24,549.59	23,746.44	21,889.70	21,665.54	91,851.27
644	2,409.55	2,361.25	2,198.42	2,205.90	9,175.12
645	5,770.73	5,506.17	5,336.99	5,224.11	21,838
646	2,757.94	2,501.93	2,568.60	2,511.15	10,339.62
647	6,943.85	6,071.86	5,752.66	5,498.21	24,266.58
648	11,961.12	12,018.72	11,716.36	10,912.48	46,608.68
650	4,607.86	4,476.34	4,307.12	4,119.07	17,510.39
651	2,155.38	2,138.05	2,135.59	1,950.92	8,379.94

	652	11,757.79	11,490.69	10,790.22	10,091.48	44,130.18
	653	4,021.94	3,810.20	3,772.37	3,660.53	15,265.04
	654	6,443.26	6,233.24	6,403.12	5,732.16	24,811.78
	655	7,951.19	7,827.92	7,499.60	7,319.77	30,598.48
	656	12,107.07	11,724.25	11,451.36	10,773.13	46,055.81
	657	15,851.87	15,825.72	15,200.28	14,707.70	61,585.57
	658	17,951.99	17,948.33	17,058.52	16,535.82	69,494.66
TOTAL		309,827.82	305,484.02	288,966.88	280,390.37	1,184,669.09

STATE: MONTANA

ZIP CODE		QUARTER 1	QUARTER 2	QUARTER 3	QUARTER 4	TOTAL GRAMS
	590	2,886.99	2,585.77	2,460.84	2,458.30	10,391.90
	591	8,951.09	8,767.71	8,342.23	8,297.62	34,358.65
	592	1,239.39	1,298.54	1,203.73	1,240	4,981.66
	593	1,762.09	1,763.34	1,639.48	1,524.29	6,689.20
	594	6,001.02	4,480.11	4,259.79	3,812.09	18,553.01
	595	779.33	744.99	728.27	658.12	2,910.71
	596	3,418.48	3,242.28	3,130.37	3,085.79	12,876.92
	597	4,913.41	4,624.50	4,464.04	4,246.65	18,248.60
	598	5,992.14	5,921.35	5,822.76	5,805.93	23,542.18
	599	5,065.52	4,947.65	5,143.07	4,642.14	19,798.38
TOTAL		41,009.46	38,376.24	37,194.58	35,770.93	152,351.21

STATE: NEBRASKA

ZIP CODE		QUARTER 1	QUARTER 2	QUARTER 3	QUARTER 4	TOTAL GRAMS
	680	4,165.81	4,351.39	4,283.95	4,316.66	17,117.81
	681	20,041.16	19,773.61	18,673.98	18,279.55	76,768.30
	683	1,227.07	1,148.31	1,148.49	1,137.70	4,661.57
	684	1,058.81	1,023.69	1,055.20	991.95	4,129.65
	685	5,304.44	5,133.43	4,981.63	4,913.86	20,333.36
	686	622.37	535.52	589.14	592.66	2,339.69
	687	1,377.16	1,342.37	1,277.04	1,329.18	5,325.75
	688	3,043.84	2,988.53	2,948.92	3,036.92	12,018.21
	689	1,180.57	1,247.14	1,299.86	1,294.08	5,021.65
	690	655.57	628.22	647.18	576.6	2,507.57

	691	1,978.34	1,950.92	1,915.31	1,838.06	7,682.63
	692	247.12	249.45	196.6	180.24	873.41
	693	1,974.28	1,836.44	1,891.35	1,767.10	7,469.17
TOTAL		42,876.54	42,209.02	40,908.65	40,254.56	166,248.77

STATE: NEVADA

ZIP CODE		QUARTER 1	QUARTER 2	QUARTER 3	QUARTER 4	TOTAL GRAMS
	890	42,370.52	41,910.82	40,430.87	40,381.88	165,094.09
	891	101,461.06	99,119.49	95,610.06	95,544.66	391,735.27
	893	756.24	680.89	672.29	592.14	2,701.56
	894	15,187.45	14,364.02	14,338.63	13,742.67	57,632.77
	895	19,045.36	18,837.44	17,589.26	16,620.12	72,092.18
	897	5,608.37	5,334	5,299.82	5,141.22	21,383.41
	898	1,874.45	1,710.43	1,604.96	1,461.88	6,651.72
TOTAL		186,303.45	181,957.09	175,545.89	173,484.57	717,291

STATE: NEW HAMPSHIRE

ZIP CODE		QUARTER 1	QUARTER 2	QUARTER 3	QUARTER 4	TOTAL GRAMS
	030	13,930.44	13,514.63	13,532.16	13,297.71	54,274.94
	031	8,562.43	8,248.17	8,438.10	7,692.22	32,940.92
	032	7,451.16	7,207.10	7,354.49	6,606.25	28,619
	033	4,916.34	4,521.65	4,468.01	4,281.23	18,187.23
	034	3,004.28	2,873.60	2,750.86	2,642.50	11,271.24
	035	3,016.68	2,905.63	2,671.02	2,298.88	10,892.21
	036	283.74	277.02	244.52	258.64	1,063.92
	037	4,287.29	4,123.68	3,789.20	3,500.98	15,701.15
	038	20,190.60	19,148.54	18,817.93	18,273.19	76,430.26
TOTAL		65,642.96	62,820.02	62,066.29	58,851.60	249,380.87

STATE: NEW JERSEY

ZIP CODE		QUARTER 1	QUARTER 2	QUARTER 3	QUARTER 4	TOTAL GRAMS
	70	78,851.21	74,380.20	69,480.16	68,000.57	290,712.14
	71	19,028.20	17,593.31	16,876.31	16,213.01	69,710.83
	72	6,638.33	5,985.78	5,914.77	5,710.58	24,249.46

73	10,785.60	10,462.77	9,945.12	9,886.16	41,079.65
74	23,754.38	22,525.25	20,248.01	20,437.60	86,965.24
75	9,490.75	8,949.73	8,480.43	8,084.52	35,005.43
76	26,283.55	25,822.77	24,270.89	23,703.10	100,080.31
77	36,250.42	34,643.45	32,556.91	32,029.56	135,480.34
78	15,260.45	14,188.37	13,245.05	13,024.91	55,718.78
79	7,484.91	6,882.86	6,753.75	6,586.21	27,707.73
80	124,104.61	117,228.59	109,325.20	102,258.85	452,917.25
81	16,621.21	15,620.06	15,014.75	14,391.33	61,647.35
82	24,875.10	24,404.33	23,377.82	22,076.67	94,733.92
83	16,828.39	16,076.50	15,015.76	14,507.17	62,427.82
84	5,142.40	4,273.71	3,257.89	2,934.84	15,608.84
85	14,825.16	13,959.99	12,527.90	11,474.71	52,787.76
86	16,425.66	15,879.80	15,112.44	14,002.85	61,420.75
87	30,068.69	27,924.06	26,905.19	25,832.28	110,730.22
88	51,698.42	50,142.92	47,460.61	45,879.29	195,181.24
89	4,680.69	4,362.27	4,251.05	3,853.18	17,147.19
TOTAL	539,098.13	511,306.72	480,020.01	460,887.39	1,991,312.25

STATE: NEW MEXICO

ZIP CODE	QUARTER 1	QUARTER 2	QUARTER 3	QUARTER 4	TOTAL GRAMS
870	9,655.53	9,214.38	8,597.10	8,362.10	35,829.11
871	47,244.76	43,432.47	39,977.81	38,496.37	169,151.41
873	1,245.42	1,167.83	1,203.33	1,123.22	4,739.80
874	2,734.89	2,775.88	2,574.61	2,410.96	10,496.34
875	15,016.79	14,917.84	14,073.96	14,107.82	58,116.41
877	1,803.98	1,815.28	1,821.64	1,821.63	7,262.53
878	621.23	712.76	647.72	669.82	2,651.53
879	513.83	499.98	516.06	485.1	2,014.97
880	7,105.75	6,122.42	5,535.47	5,468.84	24,232.48
881	1,305.84	1,292.53	1,143.58	1,008.92	4,750.87
882	7,924.30	7,313.11	7,003.66	6,759.70	29,000.77
883	3,171.83	3,013.94	2,940.49	2,740.14	11,866.40
884	327.67	339.44	348.99	261.33	1,277.43
TOTAL	98,671.82	92,617.86	86,384.42	83,715.95	361,390.05

RETAIL DRUG DISTRIBUTION BY ZIP CODE WITHIN STATE BY GRAMS WT

STATE: NEW YORK

ZIP CODE	QUARTER 1	QUARTER 2	QUARTER 3	QUARTER 4	TOTAL GRAMS
063	0	0	0	0.45	0.45
100	77,204.18	76,143.45	70,683.21	67,014.58	291,045.42
101	1,829.54	1,831.62	1,539.97	1,488.20	6,689.33
102	98.59	93.22	85.14	72.61	349.56
103	32,355.45	30,662.21	27,694.33	26,528.91	117,240.90
104	62,754.50	57,101.82	55,730.50	51,852.75	227,439.57
105	20,227.89	19,731.19	18,896.03	18,200.26	77,055.37
106	3,697.93	3,640.74	3,293.49	3,310.15	13,942.31
107	7,916.96	7,928.60	7,673.20	7,518.15	31,036.91
108	2,632.12	2,437.06	2,435.75	2,189.50	9,694.43
109	29,196.34	27,796.26	26,397.48	26,195.63	109,585.71
110	12,560.34	12,328.66	11,607.53	11,536.59	48,033.12
111	8,886.17	8,323.99	7,794.28	7,560.92	32,565.36
112	77,825.08	71,466.62	66,816.97	63,758.67	279,867.34
113	29,638.30	28,152.88	26,424.95	25,899.93	110,116.06
114	16,290.41	15,409.91	14,351.97	13,589.34	59,641.63
115	30,756.47	30,719.17	30,366.53	29,192.45	121,034.62
116	3,789.06	3,402.88	3,247.94	3,361.01	13,800.89
117	96,542.96	92,503.94	87,241.37	83,956.01	360,244.28
118	3,284.82	3,397.16	3,404.44	3,154.91	13,241.33
119	9,826.08	9,692.41	9,491.75	9,117.46	38,127.70
120	10,436.71	9,938.94	10,195.89	10,065.46	40,637
121	6,170.22	5,988.12	5,688.19	5,394.28	23,240.81
122	5,949.64	5,638.54	5,391.40	5,546.62	22,526.20
123	3,120.86	2,819.68	2,723.40	2,663.90	11,327.84
124	14,049.28	13,870.29	13,564.78	12,943.28	54,427.63
125	23,446.30	23,129.30	22,313.40	21,981.17	90,870.17
126	6,908.67	6,372.23	6,431.77	6,502.58	26,215.25
127	11,883.33	11,429.82	10,634.96	9,957.69	43,905.80
128	9,337.45	8,860.87	9,129.69	8,808.89	36,136.90
129	6,343.63	6,358.55	6,445.18	6,194.84	25,342.20
130	12,207.75	12,291.85	11,333.47	11,119.02	46,952.09
131	3,669.51	3,690.01	3,262.21	3,249.10	13,870.83
132	8,594.83	8,307.16	7,848.22	7,816.90	32,567.11

	133	4,068.04	4,052.81	3,398.70	3,489.46	15,009.01
	134	5,108.72	5,051.87	4,531.82	4,399.36	19,091.77
	135	2,137.46	2,021.83	1,925.27	1,879.44	7,964
	136	6,927.98	6,986.33	6,944.65	6,704.14	27,563.10
	137	4,789.22	4,701.20	4,405	4,542.96	18,438.38
	138	9,247.50	8,949.97	8,196.66	8,068.45	34,462.58
	139	5,503.17	5,466.41	5,122.32	5,167.71	21,259.61
	140	13,919.51	13,257.77	12,978.31	12,233.72	52,389.31
	141	6,488.68	5,977.69	5,912.30	5,593.30	23,971.97
	142	18,938.94	18,268.71	17,659.26	17,196.61	72,063.52
	143	2,244.06	2,442.89	2,430.76	2,347.86	9,465.57
	144	9,066.35	8,987.92	8,654.65	8,304.02	35,012.94
	145	8,255.74	8,011.42	7,651.54	7,458.45	31,377.15
	146	20,564.93	19,731.45	19,242.94	18,069.49	77,608.81
	147	4,296.73	4,141.70	3,870.57	3,870.63	16,179.63
	148	12,043.57	11,892.80	11,704.78	11,219.73	46,860.88
	149	3,729.68	3,593.13	3,663.54	3,239.40	14,225.75
TOTAL		786,761.65	754,995.05	718,432.46	691,526.94	2,951,716.10

STATE: NORTH CAROLINA

ZIP CODE		QUARTER 1	QUARTER 2	QUARTER 3	QUARTER 4	TOTAL GRAMS
	270	18,168.81	17,605.88	16,093.71	15,563.17	67,431.57
	271	13,544.05	12,081.44	11,932.04	10,956.87	48,514.40
	272	38,269.14	35,223.99	33,148.33	32,934.10	139,575.56
	273	24,449.29	23,245.70	22,118.11	22,077.93	91,891.03
	274	13,194.96	12,875.09	12,619.11	12,550.35	51,239.51
	275	52,405.49	49,997.22	48,367.65	47,897.55	198,667.91
	276	14,869.93	14,219.32	13,401	13,406.40	55,896.65
	277	15,864.05	15,446.46	14,820.18	14,511.73	60,642.42
	278	32,224.58	30,840.63	30,529.41	28,984.89	122,579.51
	279	13,186.56	12,771.76	11,753.61	12,122.95	49,834.88
	280	39,100.50	38,197.05	35,881.72	36,128.66	149,307.93
	281	30,987.46	30,046.27	27,754.71	27,984.39	116,772.83
	282	23,924.83	23,336.77	22,286.78	21,683.89	91,232.27
	283	78,779.50	75,405.11	72,533.51	70,320.87	297,038.99
	284	45,740.23	43,504.09	39,643.50	38,521.68	167,409.50

	285	37,774.26	35,467.49	33,299.42	31,852.13	138,393.30
	286	57,781.99	54,386.38	50,658.17	50,305.07	213,131.61
	287	31,586.89	29,835.37	28,297.56	27,279.33	116,999.15
	288	12,932.77	12,293.70	11,797.11	11,368.98	48,392.56
	289	3,483.38	3,524.59	3,401.61	3,712.31	14,121.89
TOTAL		598,268.67	570,304.31	540,337.24	530,163.25	2,239,073.47

STATE: NORTH DAKOTA

ZIP CODE		QUARTER 1	QUARTER 2	QUARTER 3	QUARTER 4	TOTAL GRAMS
	580	705.68	618.32	597.38	582.73	2,504.11
	581	2,779.59	2,585.66	2,481.94	2,346.40	10,193.59
	582	2,289.79	2,275.32	2,120.97	2,172.02	8,858.10
	583	1,344.12	1,359.86	1,269.58	1,195.53	5,169.09
	584	715.05	703.54	665.44	674.4	2,758.43
	585	4,174.69	4,130.19	4,105.11	4,095.67	16,505.66
	586	902.98	964.1	854.27	880.91	3,602.26
	587	1,333.79	1,370.57	1,241.83	1,232.13	5,178.32
	588	917.75	954.14	948.5	999.78	3,820.17
TOTAL		15,163.44	14,961.70	14,285.02	14,179.57	58,589.73

STATE: OHIO

ZIP CODE		QUARTER 1	QUARTER 2	QUARTER 3	QUARTER 4	TOTAL GRAMS
	430	29,678.50	29,004.58	26,845.58	26,372.21	111,900.87
	431	23,448.15	22,428.50	21,427.52	20,261.10	87,565.27
	432	54,229.77	51,608.22	47,983.98	46,266.83	200,088.80
	433	6,929.56	6,833.81	6,606.64	6,291.18	26,661.19
	434	6,033.21	5,904.90	5,789.48	5,657.71	23,385.30
	435	9,050.29	8,488.03	8,514.05	8,167	34,219.37
	436	23,547.40	22,851.15	21,840.15	21,137.33	89,376.03
	437	10,885.24	10,303.77	10,132.78	9,778.25	41,100.04
	438	1,656.59	1,564.18	1,580.90	1,544.74	6,346.41
	439	10,657.26	10,195.25	10,056.56	9,674.10	40,583.17
	440	27,253.31	25,630.53	24,746.32	22,829.54	100,459.70
	441	40,262.33	38,043.88	36,270.46	34,060.12	148,636.79
	442	19,618.39	18,413.04	17,187.15	16,034.99	71,253.57

	443	13,223.89	12,638.58	11,813	11,405.88	49,081.35
	444	19,176.19	18,078.44	17,389.70	16,356.12	71,000.45
	445	9,688.15	9,349.88	8,439.32	8,135.74	35,613.09
	446	13,324.14	12,728.30	12,310.14	11,669.25	50,031.83
	447	8,205.65	7,718.76	7,298.23	7,119.16	30,341.80
	448	12,877.23	12,536.80	12,012.74	11,765.55	49,192.32
	449	3,730.62	3,550.63	3,176.77	2,961.41	13,419.43
	450	34,339.33	32,841.02	31,532	29,333.70	128,046.05
	451	14,510.76	14,507.16	13,908.45	13,717.79	56,644.16
	452	43,950.39	42,654.03	40,095.31	38,945.84	165,645.57
	453	14,234.73	13,780.22	13,281.25	12,756.44	54,052.64
	454	19,993.23	19,254.10	17,804.75	16,418.10	73,470.18
	455	6,103.41	6,060.18	5,741.64	5,586.06	23,491.29
	456	23,607.33	22,643.85	21,987.60	21,332.49	89,571.27
	457	7,751.60	7,260.95	6,576.95	6,044.83	27,634.33
	458	7,335.08	7,183.54	6,685.61	6,513.70	27,717.93
TOTAL		515,301.73	494,056.28	469,035.03	448,137.16	1,926,530.20

STATE: OKLAHOMA

ZIP CODE	QUARTER 1	QUARTER 2	QUARTER 3	QUARTER 4	TOTAL GRAMS
730	39,146.44	38,792.21	37,123.61	37,134.31	152,196.57
731	46,385.65	45,847.62	44,192.54	43,299.95	179,725.76
734	4,903.76	4,944.54	4,787.20	4,668.02	19,303.52
735	12,796.79	12,280.95	12,972.89	10,570.52	48,621.15
736	4,430.24	3,687.75	3,260.60	2,832.81	14,211.40
737	3,925.40	4,043.51	3,764.04	3,903.58	15,636.53
738	796.7	917.16	800.23	803.52	3,317.61
739	176.94	206.85	165.54	182.39	731.72
740	37,066.05	35,859.43	35,092.16	34,597.18	142,614.82
741	26,458.92	26,294.61	25,343.52	25,187.42	103,284.47
743	6,280.44	5,940.09	5,915.46	5,898.41	24,034.40
744	17,665.50	17,841.47	17,314.60	16,919.26	69,740.83
745	4,672.80	4,891.09	4,423.78	3,996.40	17,984.07
746	2,705.55	2,848.57	2,723.96	2,913.88	11,191.96
747	4,082.26	3,804.58	3,529.35	3,456.56	14,872.75
748	10,425.70	10,430.50	9,955.32	9,743.69	40,555.21

749	9,943.93	9,540.78	9,589.51	9,645.57	38,719.79
TOTAL	231,863.07	228,171.71	220,954.31	215,753.47	896,742.56

STATE: OREGON

ZIP CODE	QUARTER 1	QUARTER 2	QUARTER 3	QUARTER 4	TOTAL GRAMS
970	30,469.79	29,390.80	27,997.52	27,313.68	115,171.79
971	13,835.21	13,189.84	13,149.81	12,401.90	52,576.76
972	37,211.17	36,495.59	34,998.85	33,408.76	142,114.37
973	25,997.34	23,999.33	22,298.88	21,355.99	93,651.54
974	34,283.25	33,445.26	32,062.72	31,217.41	131,008.64
975	21,029.75	20,506.77	19,791.53	19,822.38	81,150.43
976	2,876.24	2,698.51	2,589.32	2,501.27	10,665.34
977	7,988.64	7,960.38	7,753.55	7,515.54	31,218.11
978	7,419.44	7,116.10	6,883.58	6,472.93	27,892.05
979	2,054.26	1,985.94	1,997.63	1,850.74	7,888.57
TOTAL	183,165.09	176,788.52	169,523.39	163,860.60	693,337.60

STATE: PENNSYLVANIA

ZIP CODE	QUARTER 1	QUARTER 2	QUARTER 3	QUARTER 4	TOTAL GRAMS
150	24,916.59	24,867.01	25,582.02	25,196.18	100,561.80
151	13,311.85	13,037.64	12,732.63	12,138.87	51,220.99
152	36,353.98	35,064.94	33,545.27	32,516.34	137,480.53
153	7,079.42	6,677.38	6,386.68	6,339.41	26,482.89
154	10,521.12	10,325.17	10,199.95	9,731.12	40,777.36
155	4,066.14	3,919.91	3,657.40	3,421.74	15,065.19
156	16,002.49	15,439.11	14,752	14,266.45	60,460.05
157	6,270.52	6,070.93	5,896.86	5,516.81	23,755.12
158	4,835.18	4,832.72	4,519.36	4,470.07	18,657.33
159	14,946.72	15,067.83	14,251.10	13,309.81	57,575.46
160	7,955.94	7,791.51	7,620.41	7,747	31,114.86
161	11,311.67	11,210.45	9,670.95	10,610.95	42,804.02
162	4,469.04	3,956.14	3,884.93	3,609.41	15,919.52
163	7,838.68	7,604.45	7,199.98	7,401.70	30,044.81
164	2,828.39	2,673.27	2,660.14	2,453.18	10,614.98
165	5,969.44	5,880.98	5,471.49	5,331.61	22,653.52

	166	11,735.04	11,521.39	11,123.52	10,845.29	45,225.24
	167	1,887.51	1,853.20	1,820.31	1,736.91	7,297.93
	168	4,884.92	4,744.37	4,540.26	4,235.64	18,405.19
	169	2,481.85	2,395.83	2,308.39	2,130.20	9,316.27
	170	32,201.79	31,305.38	30,144	29,208.82	122,859.99
	171	9,652.15	9,176.62	9,050.76	8,502.81	36,382.34
	172	9,545.12	9,550.70	9,238.81	8,844.82	37,179.45
	173	14,535.88	14,208.24	13,129.80	12,714.64	54,588.56
	174	8,327.64	7,765.44	7,648.77	7,522.84	31,264.69
	175	12,260	11,420.62	11,355.32	10,411.43	45,447.37
	176	9,924.35	9,806.10	8,949.45	8,610.02	37,289.92
	177	8,562.25	8,261.21	7,935.41	7,541.59	32,300.46
	178	11,945.96	11,447.32	9,900.49	10,015.71	43,309.48
	179	7,757.32	7,370.16	6,869.92	6,590.28	28,587.68
	180	18,708.13	17,783.29	17,106.92	16,941.81	70,540.15
	181	11,328.21	10,737.90	10,093.71	10,261.32	42,421.14
	182	8,251.92	7,698.71	7,399.37	7,523.35	30,873.35
	183	11,548.34	11,097.79	10,376.62	10,754.90	43,777.65
	184	12,220.91	11,927.73	11,215.88	10,503.36	45,867.88
	185	16,999.60	16,731.73	15,435.18	13,996.33	63,162.84
	186	10,153.30	9,610.34	9,449.62	9,172.02	38,385.28
	187	38,521.95	35,610.28	33,096.47	32,582.21	139,810.91
	188	3,744.05	3,655.39	3,501.41	3,322.13	14,222.98
	189	17,653.42	17,624.21	17,257.87	16,299.66	68,835.16
	190	107,524.97	103,206.59	100,147.18	107,582.87	418,461.61
	191	137,800.49	137,841.83	134,510.17	126,821.12	536,973.61
	193	20,186.52	19,423.79	18,859.36	17,554.22	76,023.89
	194	31,193.12	29,569.81	28,646.13	27,605.95	117,015.01
	195	7,001.55	6,499.84	5,951.78	5,943.80	25,396.97
	196	9,426.70	9,321.31	8,562.53	8,345.58	35,656.12
TOTAL		786,642.13	763,586.56	733,656.58	718,182.28	3,002,067.55

STATE:	PUERTO RICO					
ZIP CODE		QUARTER 1	QUARTER 2	QUARTER 3	QUARTER 4	TOTAL GRAMS
	006	4,725.45	3,492.41	1,980.59	1,404.37	11,602.82
	007	6,550.55	4,160.25	2,626.34	2,020.85	15,357.99

	ZIP CODE	QUARTER 1	QUARTER 2	QUARTER 3	QUARTER 4	TOTAL GRAMS
	009	8,495.46	6,416.25	4,785.29	4,068.71	23,765.71
TOTAL		19,771.46	14,068.91	9,392.22	7,493.93	50,726.52

STATE: RHODE ISLAND

ZIP CODE		QUARTER 1	QUARTER 2	QUARTER 3	QUARTER 4	TOTAL GRAMS
	028	25,473.70	24,222.69	23,219.07	22,857.21	95,772.67
	029	18,176.99	16,482.99	16,386.35	15,703.10	66,749.43
TOTAL		43,650.69	40,705.68	39,605.42	38,560.31	162,522.10

STATE: SOUTH CAROLINA

ZIP CODE		QUARTER 1	QUARTER 2	QUARTER 3	QUARTER 4	TOTAL GRAMS
	290	13,162.54	12,821.07	12,570.79	12,529.60	51,084
	291	16,345.46	15,840.78	15,016.72	15,239.37	62,442.33
	292	10,870.95	10,619.18	10,320.23	10,124.23	41,934.59
	293	26,654.68	26,134.40	25,492.18	25,153.23	103,434.49
	294	42,647.99	42,030.83	39,471.98	39,879.57	164,030.37
	295	59,792.13	57,267.92	52,628.40	51,209.12	220,897.57
	296	47,112.97	45,670.94	42,666.28	42,287.04	177,737.23
	297	11,631.66	11,292.36	10,325.66	9,941.28	43,190.96
	298	10,495.40	10,284.97	9,821.67	9,777.12	40,379.16
	299	7,570.75	7,568.84	7,316.26	7,250.75	29,706.60
TOTAL		246,284.53	239,531.29	225,630.17	223,391.31	934,837.30

STATE: SOUTH DAKOTA

ZIP CODE		QUARTER 1	QUARTER 2	QUARTER 3	QUARTER 4	TOTAL GRAMS
	570	2,998.60	2,790.54	2,704.13	2,708.60	11,201.87
	571	5,516.38	5,768.93	5,725.05	5,270.65	22,281.01
	572	822.02	748.13	734.23	727.15	3,031.53
	573	1,000.14	929.15	905.4	910.08	3,744.77
	574	1,047.96	1,017.44	973.11	910.17	3,948.68
	575	879.53	887.34	831.63	807.66	3,406.16
	576	154.2	152.67	155.23	193.11	655.21
	577	5,591.22	5,232.10	5,110.97	4,741.85	20,676.14
TOTAL		18,010.05	17,526.30	17,139.75	16,269.27	68,945.37

STATE: TENNESSEE

ZIP CODE	QUARTER 1	QUARTER 2	QUARTER 3	QUARTER 4	TOTAL GRAMS
370	63,885.86	63,236.57	62,396.84	60,891.69	250,410.96
371	49,483.97	48,285.85	46,633.58	46,757.12	191,160.52
372	33,703.11	32,072.82	30,803.81	30,765.81	127,345.55
373	45,866.63	44,624.10	41,065.48	42,471.03	174,027.24
374	17,260.83	17,494.28	16,226.37	16,769.22	67,750.70
376	39,728.99	38,859.83	36,700.70	36,430.30	151,719.82
377	35,831.44	35,494.33	34,992.14	35,605.49	141,923.40
378	49,287.50	48,402.14	47,344.62	46,686.71	191,720.97
379	25,531.39	25,227.94	24,159.49	24,849.47	99,768.29
380	15,567.88	15,502.58	15,501.81	14,833.75	61,406.02
381	29,086.64	29,667.69	27,922.10	40,966.77	127,643.20
382	8,652.62	8,321.64	8,184.48	7,887.22	33,045.96
383	15,631.65	15,185.28	14,866.76	14,939.23	60,622.92
384	13,982.26	13,687.40	12,104.64	13,714.64	53,488.94
385	30,725.14	30,545.70	28,735.77	29,101.83	119,108.44
TOTAL	474,225.91	466,608.15	447,638.59	462,670.28	1,851,142.93

STATE: TEXAS

ZIP CODE	QUARTER 1	QUARTER 2	QUARTER 3	QUARTER 4	TOTAL GRAMS
750	40,875.46	38,175.18	36,841.41	34,897.72	150,789.77
751	8,261.45	8,384.37	8,441.16	8,192.91	33,279.89
752	11,811.03	11,842.91	11,086.74	10,813.05	45,553.73
753	151.33	146.8	161.41	198.49	658.03
754	5,998.30	5,717.10	5,002.86	4,992.12	21,710.38
755	1,698.51	1,675.95	1,581.19	1,519.42	6,475.07
756	2,841.62	2,884.66	2,731.55	2,778.95	11,236.78
757	4,546.62	4,862.76	4,704.05	4,723.26	18,836.69
758	789.78	834.51	807.89	810.08	3,242.26
759	1,917.97	1,845.51	1,843.65	1,897.08	7,504.21
760	26,485.03	26,387.92	27,048.24	25,650.65	105,571.84
761	19,248.89	19,719.85	18,489.23	18,103.16	75,561.13
762	8,658.72	8,750.71	8,464.36	8,158.74	34,032.53

	763	3,086.53	2,808.30	2,789.12	2,505.02	11,188.97
	764	1,650.91	1,559.59	1,439.45	1,329.47	5,979.42
	765	6,528.05	6,616.14	6,292.10	5,839.85	25,276.14
	766	1,471.16	1,390.79	1,529.76	1,432.52	5,824.23
	767	1,962.41	2,286	2,329.62	2,236.66	8,814.69
	768	1,120.58	1,123.69	1,144.47	1,097.46	4,486.20
	769	1,327.66	1,132.36	1,135.26	979.4	4,574.68
	770	42,322.23	45,503.17	47,109.99	44,392.21	179,327.60
	773	13,243.92	12,834.87	12,740.81	12,765.14	51,584.74
	774	13,572.93	14,266.76	13,757.26	14,722.37	56,319.32
	775	12,916.09	13,451.87	13,097.25	12,845.42	52,310.63
	776	2,636.47	2,454.76	2,378.40	2,420.61	9,890.24
	777	1,035.10	986.94	1,130.19	1,052.94	4,205.17
	778	1,398.65	1,445.11	1,423.79	1,200.37	5,467.92
	779	3,422.67	3,441.71	3,485.10	3,441.98	13,791.46
	780	3,270.99	3,306.69	3,462.72	3,502.89	13,543.29
	781	3,980.04	3,939.33	3,975.24	4,016.96	15,911.57
	782	22,753.23	22,527.69	23,174.98	22,706.56	91,162.46
	783	1,383.75	1,272.50	1,358.91	1,150.23	5,165.39
	784	2,147.29	1,925.96	1,933.29	1,871.89	7,878.43
	785	3,820.03	3,655.26	3,480.78	3,239.72	14,195.79
	786	19,535.93	18,366.42	18,548.09	17,607.63	74,058.07
	787	18,053.11	18,130.72	15,818.81	14,769.60	66,772.24
	788	587.07	542.02	648.29	563.02	2,340.40
	789	703.04	686.81	720.46	682.09	2,792.40
	790	1,628.64	1,521.08	1,361.80	1,282.89	5,794.41
	791	2,987.36	3,044.83	2,946.17	3,041.65	12,020.01
	792	287.78	339.33	340.22	327	1,294.33
	793	632.7	635.26	677.62	636.92	2,582.50
	794	2,503.47	2,681.18	2,807.79	2,641.16	10,633.60
	795	732.13	692.28	710.07	806.4	2,940.88
	796	1,801.40	1,806.07	1,655.12	1,676.56	6,939.15
	797	4,106.40	3,797.66	3,872.51	3,744.18	15,520.75
	798	158.59	140.97	138.96	148.28	586.8
	799	4,874.85	4,737.96	4,953.52	4,123.76	18,690.09
TOTAL		336,927.87	336,280.31	331,571.66	319,536.44	1,324,316.28

DATE RANGE: 01/01/2017 TO 12/31/2017 — ARCOS 3 - REPORT 1 — Run Date: 07/03/2018

RETAIL DRUG DISTRIBUTION BY ZIP CODE WITHIN STATE BY GRAMS WT

STATE: UTAH

ZIP CODE	QUARTER 1	QUARTER 2	QUARTER 3	QUARTER 4	TOTAL GRAMS
840	67,735.11	67,573.79	65,698.53	65,184.47	266,191.90
841	46,991.41	45,113.68	43,152.80	42,176.80	177,434.69
843	5,747.05	5,655.67	5,508.04	5,282.25	22,193.01
844	14,517.55	14,261.57	13,839.33	13,528.56	56,147.01
845	6,179.66	5,338.43	5,056.67	4,777.65	21,352.41
846	13,779.56	13,315.86	12,422.94	12,456.24	51,974.60
847	19,350.13	18,879.74	19,145.59	18,318.16	75,693.62
TOTAL	174,300.47	170,138.74	164,823.90	161,724.13	670,987.24

STATE: VERMONT

ZIP CODE	QUARTER 1	QUARTER 2	QUARTER 3	QUARTER 4	TOTAL GRAMS
050	2,342.51	2,414.93	1,969.77	1,814.32	8,541.53
051	1,703.80	1,735.89	1,510.42	1,718.72	6,668.83
052	1,263.29	1,224.44	1,178.96	1,238.16	4,904.85
053	1,918.77	1,867.42	1,785.14	1,640.91	7,212.24
054	10,054.72	9,742.22	9,640.24	9,151.69	38,588.87
056	2,315.85	2,257.23	2,134.58	2,007.08	8,714.74
057	2,385.85	2,203.97	2,124.58	1,905.64	8,620.04
058	1,866.09	1,945	1,815.40	1,866.43	7,492.92
TOTAL	23,850.88	23,391.10	22,159.09	21,342.95	90,744.02

STATE: VIRGIN ISLANDS

ZIP CODE	QUARTER 1	QUARTER 2	QUARTER 3	QUARTER 4	TOTAL GRAMS
008	584.77	578.06	432.34	506.93	2,102.10
TOTAL	584.77	578.06	432.34	506.93	2,102.10

STATE: VIRGINIA

ZIP CODE	QUARTER 1	QUARTER 2	QUARTER 3	QUARTER 4	TOTAL GRAMS
201	21,817.77	20,877.44	19,470.45	19,192.28	81,357.94
220	13,367.71	12,117.07	11,522.34	10,679.09	47,686.21
221	11,106.97	10,564.94	10,072.94	10,696.50	42,441.35

	222	2,928.97	3,053.87	2,862.79	2,932.78	11,778.41
	223	7,248.69	6,421.68	5,942.29	5,693.18	25,305.84
	224	10,542.84	9,318.71	9,040.26	8,980.31	37,882.12
	225	3,751.72	3,629.99	3,407.15	3,320.57	14,109.43
	226	10,069.90	9,398.52	9,313.21	9,225.51	38,007.14
	227	2,245.95	2,103.52	1,825.99	1,907.80	8,083.26
	228	6,301.25	5,905.82	4,900.46	5,172.70	22,280.23
	229	9,821.11	8,960.18	8,589.54	8,774.26	36,145.09
	230	7,329.62	6,831.66	6,803.17	6,894.04	27,858.49
	231	13,933.31	12,323.08	10,961.85	11,507.76	48,726
	232	28,631.24	25,701.38	24,491.94	23,937.01	102,761.57
	233	9,499.76	9,263.25	8,754.90	8,568.96	36,086.87
	234	21,283.48	20,341.01	19,342.52	18,318.32	79,285.33
	235	8,471.43	8,062.31	7,046.70	7,298.12	30,878.56
	236	13,573.72	12,473.05	11,714.57	10,649.96	48,411.30
	237	6,410.70	6,181.71	5,487.59	5,521.28	23,601.28
	238	16,809.52	16,179.94	15,955.01	15,894.80	64,839.27
	239	2,899.30	2,726.97	2,679.26	2,624.66	10,930.19
	240	19,303.26	17,989.80	17,441.07	16,416.57	71,150.70
	241	16,903.43	16,434.74	15,316.71	15,240.48	63,895.36
	242	22,391.68	21,400.10	21,165.93	20,613.90	85,571.61
	243	9,507.97	8,351.44	8,096.26	8,009.44	33,965.11
	244	5,035.88	4,540.20	4,353.98	4,407.72	18,337.78
	245	14,866.66	13,719.73	12,315.15	12,333.25	53,234.79
	246	5,409.61	5,491.65	5,517.40	5,128.15	21,546.81
TOTAL		321,463.45	300,363.76	284,391.43	279,939.40	1,186,158.04

STATE:	WASHINGTON					
ZIP CODE		QUARTER 1	QUARTER 2	QUARTER 3	QUARTER 4	TOTAL GRAMS
	980	53,132.24	51,229.98	48,844.58	46,873.82	200,080.62
	981	33,150.84	32,881.72	31,072.18	29,485.17	126,589.91
	982	39,907.59	39,322.24	38,116.92	36,441.43	153,788.18
	983	35,691.52	35,189.09	33,642.29	32,605.45	137,128.35
	984	25,468.66	24,512.63	23,623.71	22,582.64	96,187.64
	985	20,092.93	19,296.15	18,934.52	18,434.39	76,757.99
	986	30,882.21	29,525	28,460.72	27,278.05	116,145.98

988	9,550.40	9,714.72	8,693.03	8,608.05	36,566.20
989	12,464.93	12,103.81	11,621.86	10,528.14	46,718.74
990	3,059.57	3,036.77	3,100.34	2,991.94	12,188.62
991	5,020.76	5,058.13	5,066.22	4,747.35	19,892.46
992	18,645.81	17,841.46	17,474.15	16,641.08	70,602.50
993	17,572.14	16,675.07	15,831.13	14,902.34	64,980.68
994	1,087.14	1,193.24	1,107.29	1,199.26	4,586.93
TOTAL	305,726.74	297,580.01	285,588.94	273,319.11	1,162,214.80

STATE: WEST VIRGINIA

ZIP CODE	QUARTER 1	QUARTER 2	QUARTER 3	QUARTER 4	TOTAL GRAMS
247	2,829.34	2,817.62	2,694.29	2,584.73	10,925.98
248	1,623.82	1,650.71	1,355.65	1,501.30	6,131.48
249	2,225.68	2,165.60	2,169.63	1,992.71	8,553.62
250	2,702.87	2,423.51	2,395.94	2,154.15	9,676.47
251	1,643.87	1,557.88	1,558.57	1,483.59	6,243.91
252	2,049.64	2,004.98	1,961.48	1,804.81	7,820.91
253	7,666.52	7,265.54	7,094.64	6,780.23	28,806.93
254	15,201.43	14,342.61	13,923.83	13,093.44	56,561.31
255	5,584.43	5,129.66	4,996.73	4,840	20,550.82
256	1,545.55	1,451.78	1,397.83	1,389.72	5,784.88
257	4,721.42	4,290.60	3,690.80	3,767.17	16,469.99
258	4,026.54	4,138.99	3,656.31	3,743.22	15,565.06
259	2,506.52	2,187.28	1,927.79	1,602.94	8,224.53
260	9,907.40	9,522.23	8,959.14	8,782.04	37,170.81
261	3,435.90	3,423.39	3,367.58	3,073.25	13,300.12
262	2,510.99	2,463.06	2,280.36	2,388.97	9,643.38
263	4,940.67	4,861.64	4,813.59	4,458.05	19,073.95
264	1,503.39	1,446.51	1,407.44	1,377.14	5,734.48
265	7,376.20	7,040.81	7,074.71	6,951.45	28,443.17
266	1,534.45	1,432.45	1,513.98	1,350.80	5,831.68
267	2,576.72	2,461.03	2,272.74	2,191.27	9,501.76
268	846.08	744.28	692.98	609.69	2,893.03
TOTAL	88,959.43	84,822.16	81,206.01	77,920.67	332,908.27

STATE: WISCONSIN

ZIP CODE	QUARTER 1	QUARTER 2	QUARTER 3	QUARTER 4	TOTAL GRAMS
530	26,162.27	22,936.63	21,219.90	21,582.30	91,901.10
531	29,816.23	27,354.12	25,977.01	25,969.70	109,117.06
532	76,809.30	71,136.87	68,121.45	64,317.94	280,385.56
534	6,297.20	5,276.34	5,367.77	5,268.45	22,209.76
535	18,649.09	17,573.05	16,603.78	15,990.75	68,816.67
537	14,501.84	13,269.81	12,831.89	12,062.96	52,666.50
538	1,154.56	1,057.20	1,066.75	1,084.37	4,362.88
539	7,465.56	6,668.61	6,364.09	6,205.45	26,703.71
540	3,418.96	3,348.03	3,111.93	3,066.80	12,945.72
541	3,451.78	3,347.46	3,257.54	3,093	13,149.78
542	2,423.04	2,209.23	2,116.45	1,989.93	8,738.65
543	4,086.25	3,922.99	3,731.45	3,660.13	15,400.82
544	8,019.65	7,479.05	7,070.05	6,887.42	29,456.17
545	3,059.15	2,961.99	2,927.27	2,508.80	11,457.21
546	7,257.62	6,851.56	6,462.72	6,246.35	26,818.25
547	5,091.97	4,484.38	3,907	3,886.93	17,370.28
548	4,797.55	4,741.31	4,575.11	4,277.96	18,391.93
549	13,225.32	11,933.53	11,445.93	11,223.12	47,827.90
TOTAL	235,687.34	216,552.16	206,158.09	199,322.36	857,719.95

STATE: WYOMING

ZIP CODE	QUARTER 1	QUARTER 2	QUARTER 3	QUARTER 4	TOTAL GRAMS
820	5,526.71	5,358.94	5,121.86	4,890.28	20,897.79
822	614.41	569.48	513.02	654.18	2,351.09
823	641.21	495.47	339.58	313.48	1,789.74
824	3,925.50	3,135.73	3,038.16	3,161.67	13,261.06
825	2,129.22	2,264.81	2,089.14	2,258.36	8,741.53
826	4,620.78	4,460.65	4,692.65	4,497.31	18,271.39
827	1,289.97	1,295.06	1,199.64	1,203.93	4,988.60
828	1,756.60	1,482.81	1,626.21	1,489.99	6,355.61
829	5,158.25	5,438.17	5,390.26	5,556.53	21,543.21
830	321.93	253.4	296.02	273.97	1,145.32
831	801.41	649.96	699.76	672.33	2,823.46

TOTAL	26,785.99	25,404.48	25,006.30	24,972.03	102,168.80

DRUG CODE: 9150 DRUG NAME: HYDROMORPHONE

STATE: ALABAMA

ZIP CODE	QUARTER 1	QUARTER 2	QUARTER 3	QUARTER 4	TOTAL GRAMS
350	260.35	212.42	215.45	198.34	886.56
351	122.63	105.25	124.37	114.09	466.34
352	483.38	449.03	513.06	468.15	1,913.62
354	128.15	128.3	130.16	115.75	502.36
355	55.59	50.52	58.21	55.2	219.52
356	203.65	173.87	189.44	192.63	759.59
357	121.21	117.39	112.12	105.85	456.57
358	267.24	281.07	251.07	277.75	1,077.13
359	140.83	129.39	112.91	134.45	517.58
360	186.84	165.22	139.62	145.92	637.6
361	185.34	172.66	159.06	160.57	677.63
362	105.09	85.37	94.96	83.99	369.41
363	251.81	260.36	269.1	221.05	1,002.32
364	70.43	73.95	53.78	68.61	266.77
365	495.76	306.67	290.21	294.46	1,387.10
366	290.66	309.7	364.29	333.57	1,298.22
367	21.88	25.87	21.46	17.57	86.78
368	125.61	119.5	114.06	120.94	480.11
369	12.32	7.76	7.94	4.08	32.1
TOTAL	3,528.77	3,174.30	3,221.27	3,112.97	13,037.31

STATE: ALASKA

ZIP CODE	QUARTER 1	QUARTER 2	QUARTER 3	QUARTER 4	TOTAL GRAMS
995	644.34	582.77	513.87	530.38	2,271.36
996	325.09	315.47	316.22	322.56	1,279.34
997	47.24	41.49	40.84	50.77	180.34
998	28.65	29.28	29	40.66	127.59
999	19.93	7.88	6.61	4.18	38.6
TOTAL	1,065.25	976.89	906.54	948.55	3,897.23

STATE: AMERICAN SAMOA

ZIP CODE	QUARTER 1	QUARTER 2	QUARTER 3	QUARTER 4	TOTAL GRAMS
967	0.89	0.09	0	0	0.98
TOTAL	0.89	0.09	0	0	0.98

STATE: ARIZONA

ZIP CODE	QUARTER 1	QUARTER 2	QUARTER 3	QUARTER 4	TOTAL GRAMS
850	1,329.80	1,330.03	1,212.49	1,083.72	4,956.04
851	204.71	210.29	210.25	191.71	816.96
852	1,698.32	1,733.01	1,723.09	1,699.02	6,853.44
853	1,228.47	1,139.67	1,085.26	1,085.61	4,539.01
855	103.79	87.46	86.61	82.04	359.9
856	199.05	200.3	168.71	166.41	734.47
857	1,137.55	1,038.75	1,024.55	988.53	4,189.38
859	114.25	141.59	113.77	115.14	484.75
860	152.95	119.82	123.31	117.91	513.99
863	286.46	301.98	293.79	250.1	1,132.33
864	632.32	592.95	550.5	447.19	2,222.96
865	1.42	1.76	3.03	2.09	8.3
TOTAL	7,089.09	6,897.61	6,595.36	6,229.47	26,811.53

STATE: ARKANSAS

ZIP CODE	QUARTER 1	QUARTER 2	QUARTER 3	QUARTER 4	TOTAL GRAMS
716	69.51	71.64	71.84	67.68	280.67
717	52.55	44.11	42.77	26.4	165.83
718	92.47	105.6	91.96	84.99	375.02
719	178.26	150.06	183.52	162.36	674.2
720	283.86	269.66	289.71	260.42	1,103.65
721	283.88	300.27	255.16	268.97	1,108.28
722	533.27	554.98	529.54	577.25	2,195.04
723	74.45	55.64	54.05	43.2	227.34

	724	201.66	233.68	253.02	186.75	875.11
	725	145.07	150.91	142.25	131.89	570.12
	726	157.37	133.19	143.25	149.67	583.48
	727	802.5	756.72	735.83	737.67	3,032.72
	728	97.04	81.42	88.24	77.97	344.67
	729	404.95	379.35	339.39	394.8	1,518.49
TOTAL		3,376.84	3,287.23	3,220.53	3,170.02	13,054.62

STATE: CALIFORNIA

ZIP CODE		QUARTER 1	QUARTER 2	QUARTER 3	QUARTER 4	TOTAL GRAMS
	900	1,559.16	1,579.69	1,526.33	1,527.63	6,192.81
	902	633.8	581.84	557.35	547.96	2,320.95
	903	41.76	36.89	36.08	37.31	152.04
	904	242.32	266.25	271.4	264.49	1,044.46
	905	508.86	572.23	527.46	521.32	2,129.87
	906	416.91	420.35	425.94	340.54	1,603.74
	907	483.09	474.26	464.27	402.62	1,824.24
	908	1,247.23	1,327.37	1,251.67	688.17	4,514.44
	910	485.42	473.92	424.21	433.56	1,817.11
	911	190.28	171.12	162.84	168.98	693.22
	912	188.91	211.38	200.12	172.26	772.67
	913	1,394.70	1,362.90	1,314.74	1,229.25	5,301.59
	914	356.94	425.63	562.05	479.87	1,824.49
	915	241.83	304.88	212.36	227.95	987.02
	916	91.96	83.12	73.27	71.01	319.36
	917	1,728.44	1,782.08	1,720.16	1,719.17	6,949.85
	918	23.87	24.38	13.88	20.55	82.68
	919	424.13	404.32	384.78	443.02	1,656.25
	920	1,972.59	1,899.56	1,753.74	1,918.56	7,544.45
	921	1,493.63	1,384.92	1,401.14	1,398.20	5,677.89
	922	979.94	933.74	886.11	850.95	3,650.74
	923	1,446.19	1,336.23	1,374.46	1,278.24	5,435.12
	924	210.38	188.97	168.31	181.65	749.31
	925	1,110.41	1,334.89	1,139.32	1,206.68	4,791.30
	926	930.25	874.25	831.14	765.76	3,401.40
	927	315.64	270.05	297.05	292.22	1,174.96

	928	1,529.34	1,443.81	1,356.17	1,537.52	5,866.84
	930	696.01	653.99	595.52	543.41	2,488.93
	931	167.2	163.42	139.43	121.16	591.21
	932	610.95	527.33	537.82	529.2	2,205.30
	933	844.71	959.61	881.54	774.94	3,460.80
	934	913.34	820.14	785.9	783.74	3,303.12
	935	1,313.01	1,325.84	1,260.16	1,190.65	5,089.66
	936	298.79	337.52	357.17	353.37	1,346.85
	937	413.15	391.77	405.44	417.52	1,627.88
	939	299.06	262.43	264.94	340.99	1,167.42
	940	591.62	544.45	514.74	510.76	2,161.57
	941	1,015.92	887.65	878.42	870.66	3,652.65
	943	284.92	286.5	258.26	315.94	1,145.62
	944	105.31	65.29	76.22	68.04	314.86
	945	2,900.20	2,912.43	2,793.48	2,623.47	11,229.58
	946	455.88	386.46	346.43	429.23	1,618
	947	166.66	192.98	192.04	186.87	738.55
	948	106.18	82.74	61.7	82.53	333.15
	949	515.56	550.84	537.99	465.82	2,070.21
	950	516.47	490.25	477.66	469.99	1,954.37
	951	393.06	391.31	351.28	383.46	1,519.11
	952	499.44	516.97	482.19	407.41	1,906.01
	953	1,046.53	1,062.50	950.7	913.09	3,972.82
	954	988.4	936.35	788.41	678.13	3,391.29
	955	287.73	284.08	270.4	255.83	1,098.04
	956	1,770.13	1,692.63	1,689.83	1,663.10	6,815.69
	957	207.13	177.98	187.41	172.62	745.14
	958	1,090.77	1,165.27	1,220.14	1,053.27	4,529.45
	959	1,113.08	1,040.89	920.28	930.69	4,004.94
	960	544.62	508.25	499.81	477.77	2,030.45
	961	73.87	82.43	63.37	81.69	301.36
TOTAL		40,477.68	39,869.33	38,125.03	36,820.79	155,292.83

STATE:	COLORADO				
ZIP CODE	QUARTER 1	QUARTER 2	QUARTER 3	QUARTER 4	TOTAL GRAMS
800	1,367.40	1,239.98	1,251.22	1,242.79	5,101.39

	801	1,332.56	1,174.25	1,118.03	1,185.57	4,810.41
	802	1,299.39	1,125.26	1,097.39	1,169.59	4,691.63
	803	128	123.02	101.7	92.66	445.38
	804	267.61	254.76	214.03	241.15	977.55
	805	668.79	641.19	613.62	565.55	2,489.15
	806	383.11	354.8	349.21	341.71	1,428.83
	807	71.34	82.37	71.65	69.5	294.86
	808	144.42	127.9	137.58	117.94	527.84
	809	781.27	771.45	739.53	729.36	3,021.61
	810	383.8	367.56	350.92	343.92	1,446.20
	811	48.98	39.88	32.97	37.08	158.91
	812	100.55	94.07	92.53	79.33	366.48
	813	61.76	49	66.75	67.54	245.05
	814	68.24	60.52	56.18	55.58	240.52
	815	160.78	139.48	129.83	157.66	587.75
	816	102.79	92.16	83.85	86.24	365.04
TOTAL		7,370.79	6,737.65	6,506.99	6,583.17	27,198.60

STATE:	CONNECTICUT					
ZIP CODE		QUARTER 1	QUARTER 2	QUARTER 3	QUARTER 4	TOTAL GRAMS
	60	1,282.91	1,361.76	1,117.67	1,133.15	4,895.49
	61	628.43	538.06	525.46	537.19	2,229.14
	62	171.82	156.99	164.8	166.05	659.66
	63	327.21	330.98	306.43	304.68	1,269.30
	64	1,761.83	1,605.27	1,745.38	1,504.06	6,616.54
	65	517.28	509.36	480.39	569.9	2,076.93
	66	152.16	169.61	173.32	180.44	675.53
	67	449.23	404.06	422.89	423.1	1,699.28
	68	1,276.21	1,260.48	1,161.11	1,321.56	5,019.36
	69	104.33	112.08	107.72	110.39	434.52
TOTAL		6,671.41	6,448.65	6,205.17	6,250.52	25,575.75

STATE:	DELAWARE					
ZIP CODE		QUARTER 1	QUARTER 2	QUARTER 3	QUARTER 4	TOTAL GRAMS
	197	992.32	874.23	908.66	948.74	3,723.95

	198	411.83	351.81	333.36	347.32	1,444.32
	199	890.67	853.5	797.2	813.58	3,354.95
TOTAL		2,294.82	2,079.54	2,039.22	2,109.64	8,523.22

STATE:	DISTRICT OF COLUMBIA					
ZIP CODE		QUARTER 1	QUARTER 2	QUARTER 3	QUARTER 4	TOTAL GRAMS
	200	580.45	523.3	513.86	487.21	2,104.82
	203	0	0	0	0.35	0.35
	204	16.22	16.49	15.38	14.05	62.14
TOTAL		596.67	539.79	529.24	501.61	2,167.31

STATE:	FLORIDA					
ZIP CODE		QUARTER 1	QUARTER 2	QUARTER 3	QUARTER 4	TOTAL GRAMS
	320	882.89	848.4	820.82	802.72	3,354.83
	321	1,119.11	1,276.59	1,269.49	1,155.20	4,820.39
	322	2,189.15	1,913.21	1,795.38	1,780.68	7,678.42
	323	431.56	402.09	381.48	393.41	1,608.54
	324	353.3	333.75	279.57	326.78	1,293.40
	325	934.98	758.36	737.75	749.42	3,180.51
	326	571.01	539	538.9	576.23	2,225.14
	327	3,390.33	3,024.18	2,844.10	2,760.33	12,018.94
	328	1,963.33	2,010.66	1,906.87	1,853.32	7,734.18
	329	2,164.34	2,056.64	2,155.42	1,943.14	8,319.54
	330	2,109.01	2,160.89	1,744.29	1,819.60	7,833.79
	331	1,655.60	1,702.91	1,522.71	1,549.37	6,430.59
	333	1,454.54	1,360.85	1,450.17	1,330.48	5,596.04
	334	2,423.37	2,313.04	2,109.64	2,158.96	9,005.01
	335	2,312.59	2,121.06	2,021.16	2,029.20	8,484.01
	336	8,321.09	6,505.75	6,811.94	7,566.74	29,205.52
	337	3,479.81	3,300.87	3,064.39	3,163.79	13,008.86
	338	1,301.84	1,281.29	1,086.58	1,122.05	4,791.76
	339	2,357.40	2,243.87	1,914.39	2,168.41	8,684.07
	341	711.78	675.97	702.19	719.62	2,809.56
	342	2,683.41	2,589.77	2,358.89	2,521.07	10,153.14
	344	1,116.36	1,140.89	1,075.84	1,042.36	4,375.45

	346	3,481.86	3,414.81	3,287.55	3,407.62	13,591.84
	347	1,487.03	1,496.15	1,380.26	1,434.66	5,798.10
	349	1,262.49	1,184.15	1,134.74	1,143.92	4,725.30
TOTAL		50,158.18	46,655.15	44,394.52	45,519.08	186,726.93

STATE:	GEORGIA					
ZIP CODE		QUARTER 1	QUARTER 2	QUARTER 3	QUARTER 4	TOTAL GRAMS
	300	2,011.78	1,779.86	1,876.30	1,746.26	7,414.20
	301	994.68	933.97	949.29	956.51	3,834.45
	302	669.76	620.41	576.49	580.48	2,447.14
	303	902.95	807.22	827.13	804.73	3,342.03
	304	142.58	156.72	142.29	115.78	557.37
	305	570.17	581.96	583.53	571.96	2,307.62
	306	471.1	481.73	443.98	464.13	1,860.94
	307	210.09	184.88	182.84	180.72	758.53
	308	127.76	121.89	123.39	121.4	494.44
	309	279.94	391.37	350.06	319.33	1,340.70
	310	278.93	263.81	252.64	269.11	1,064.49
	312	156.74	163.98	221.67	191.57	733.96
	313	367.77	345.84	271.84	139.49	1,124.94
	314	262.65	230.66	271.56	206.99	971.86
	315	544.02	513.45	557.52	398.86	2,013.85
	316	146.48	166.94	154.41	136.93	604.76
	317	426.29	394.92	434.02	433.08	1,688.31
	318	6.75	6.14	8.5	6.63	28.02
	319	93.27	88.95	114.16	76.94	373.32
	398	109.25	86.31	69.13	72.85	337.54
TOTAL		8,772.96	8,321.01	8,410.75	7,793.75	33,298.47

STATE:	GUAM					
ZIP CODE		QUARTER 1	QUARTER 2	QUARTER 3	QUARTER 4	TOTAL GRAMS
	969	31.75	26.32	23.41	33.03	114.51
TOTAL		31.75	26.32	23.41	33.03	114.51

STATE: HAWAII

ZIP CODE	QUARTER 1	QUARTER 2	QUARTER 3	QUARTER 4	TOTAL GRAMS
967	367.65	353.54	328.35	329.99	1,379.53
968	324.99	352.68	314.52	299.5	1,291.69
TOTAL	692.64	706.22	642.87	629.49	2,671.22

STATE: IDAHO

ZIP CODE	QUARTER 1	QUARTER 2	QUARTER 3	QUARTER 4	TOTAL GRAMS
832	115.23	101.81	102.12	112.63	431.79
833	196.49	194.28	164.83	190.29	745.89
834	82.98	94.3	91.46	83.97	352.71
835	99.86	94.02	87.2	91.28	372.36
836	610.39	598	614	613.84	2,436.23
837	569.55	484.89	519.03	613.82	2,187.29
838	268.16	247.61	260.75	223.03	999.55
TOTAL	1,942.66	1,814.91	1,839.39	1,928.86	7,525.82

STATE: ILLINOIS

ZIP CODE	QUARTER 1	QUARTER 2	QUARTER 3	QUARTER 4	TOTAL GRAMS
600	1,781.86	1,769.08	1,563.94	1,687.40	6,802.28
601	1,306.38	1,139.24	1,199.82	1,264.79	4,910.23
602	66.45	67.71	78.31	80.41	292.88
603	94.2	93.94	95.51	98.75	382.4
604	1,426.01	1,399.19	1,329.55	1,257.06	5,411.81
605	790.11	773.46	733.52	754.04	3,051.13
606	1,701.99	1,659.04	1,509.14	1,483.21	6,353.38
607	57.57	56.69	62.99	70.88	248.13
608	141.45	138.07	136.55	113.04	529.11
609	179.48	146.04	124.59	115.98	566.09
610	79.76	73.63	72.16	61.7	287.25
611	192.44	176.86	206.32	192.29	767.91
612	81.51	57.18	74.39	76.77	289.85
613	63.59	61.63	62.85	67.33	255.4
614	51.79	48.18	54.54	46.45	200.96
615	99.47	93.02	88.02	88.06	368.57

	616	187.27	215.07	209.37	199.61	811.32
	617	193.83	218.55	232.41	187.96	832.75
	618	300.04	303.55	254.3	247.07	1,104.96
	619	61.93	58.75	98.55	36.73	255.96
	620	199.94	169.07	170.66	176.18	715.85
	622	248.95	247.56	234.57	212.6	943.68
	623	73.73	59.41	65.63	81.26	280.03
	624	101.95	82.99	90.23	85.02	360.19
	625	230.25	248.4	225.78	216.27	920.7
	626	87.07	85.48	88.98	84.11	345.64
	627	230.43	147.9	135.28	168.62	682.23
	628	103.91	91.64	89.27	100.07	384.89
	629	159.39	154.48	123.54	112.16	549.57
TOTAL		10,292.75	9,835.81	9,410.77	9,365.82	38,905.15

STATE:	INDIANA					
ZIP CODE		QUARTER 1	QUARTER 2	QUARTER 3	QUARTER 4	TOTAL GRAMS
	460	300.21	365.72	378.25	404.08	1,448.26
	461	340.24	294.96	294.96	346.71	1,276.87
	462	1,374.39	1,454.17	1,522.89	1,204.27	5,555.72
	463	560.25	495.42	488.26	451.19	1,995.12
	464	48.2	46.51	31.53	32.84	159.08
	465	390.03	386.17	364.53	367.94	1,508.67
	466	156.9	166.04	144.23	161.24	628.41
	467	330.09	276.22	281.17	226.72	1,114.20
	468	442.14	431.57	356.47	415.28	1,645.46
	469	169.45	147.35	133.51	138.89	589.2
	470	40.02	46.94	31.7	40.44	159.1
	471	256.93	293.89	225.32	178.65	954.79
	472	191.74	162.49	172.7	186.28	713.21
	473	302.85	333.38	286.54	329.39	1,252.16
	474	189.55	174.36	166.23	168.73	698.87
	475	241.97	267.27	327.39	323.46	1,160.09
	476	69.97	74.78	80.96	115.29	341
	477	277.27	286.31	272.76	328.65	1,164.99
	478	175.58	151.97	131.61	117.17	576.33

	479	203.92	229.61	228.91	224.93	887.37
TOTAL		6,061.70	6,085.13	5,919.92	5,762.15	23,828.90

STATE:	IOWA					
ZIP CODE		QUARTER 1	QUARTER 2	QUARTER 3	QUARTER 4	TOTAL GRAMS
	500	295.54	304.11	249.16	210.21	1,059.02
	501	201.65	178.19	180.98	171.81	732.63
	502	172.18	169.44	164.98	152.86	659.46
	503	726.23	641.46	641.79	632.58	2,642.06
	504	71.62	65.71	71.19	55.65	264.17
	505	44.97	47.66	50.89	62.46	205.98
	506	96.23	72.95	84.57	84.84	338.59
	507	59.37	61.62	55.86	54.21	231.06
	508	19.96	16.38	20.71	13.93	70.98
	510	15.86	12.83	15.77	16.3	60.76
	511	52.36	59.11	55.58	49.04	216.09
	512	10.84	8.32	6.18	9.57	34.91
	513	40.02	31	31.07	32.9	134.99
	514	30.16	32.57	29.38	27.77	119.88
	515	67.08	62.5	58.39	55.46	243.43
	516	7.57	10.14	7.98	14.57	40.26
	520	75.4	77.45	68.4	55.08	276.33
	521	16.92	10.64	23.39	15.1	66.05
	522	402.34	393.27	361.18	345.55	1,502.34
	523	70.13	70.53	67.89	67.35	275.9
	524	128.35	143.92	155.84	145.63	573.74
	525	216.71	207.52	197.42	299.52	921.17
	526	118.66	114.92	93.8	98.5	425.88
	527	132.3	113.46	96.45	98.39	440.6
	528	104.44	80.43	84.21	86.8	355.88
TOTAL		3,176.89	2,986.13	2,873.06	2,856.08	11,892.16

STATE:	KANSAS					
ZIP CODE		QUARTER 1	QUARTER 2	QUARTER 3	QUARTER 4	TOTAL GRAMS
	660	415.79	419.48	408.37	401.6	1,645.24

	661	228.77	181.89	256.29	156.46	823.41
	662	773.47	1,014.46	706.89	593.13	3,087.95
	664	111.19	88.4	87.1	98.7	385.39
	665	143.97	141.96	136.84	115.08	537.85
	666	110.57	95.17	104.48	104.61	414.83
	667	122.37	113.93	130.9	98.91	466.11
	668	38.65	34.39	35.42	30.51	138.97
	669	14.74	15.84	15.01	18.42	64.01
	670	154.18	158.18	156.41	133.66	602.43
	671	117.94	121.81	119.63	120.08	479.46
	672	694.32	710.57	742.69	629.6	2,777.18
	673	28.21	33.56	34.99	33.11	129.87
	674	175.46	162.37	190.18	148.19	676.2
	675	236.27	216.36	223.17	229.19	904.99
	676	76.42	76.22	84.36	92.12	329.12
	677	28.22	22.98	27.7	19.39	98.29
	678	72.61	77.09	69.12	75.71	294.53
	679	32.36	26.51	32.74	30.35	121.96
TOTAL		3,575.51	3,711.17	3,562.29	3,128.82	13,977.79

STATE:	KENTUCKY					
ZIP CODE		QUARTER 1	QUARTER 2	QUARTER 3	QUARTER 4	TOTAL GRAMS
	400	112.74	104.01	85.54	89.63	391.92
	401	45.55	53.87	47.18	38.86	185.46
	402	833.93	787.39	756.11	934.05	3,311.48
	403	83.54	70.55	86.94	75.98	317.01
	404	33.36	70.85	54.67	33.99	192.87
	405	354.19	220.03	271.61	485.57	1,331.40
	406	29.52	24.23	31.48	23.68	108.91
	407	21.76	15.56	23.71	23.16	84.19
	408	5.15	6.39	4.84	5.78	22.16
	409	17.85	17.99	33.31	12.38	81.53
	410	154.27	130.9	146.58	131.63	563.38
	411	77.84	47.34	71.27	72.84	269.29
	412	7.78	5.87	5.09	11.99	30.73
	413	6.34	5.6	4.45	9.09	25.48

	414	0.4	0.04	0.8	0.68	1.92
	415	58.12	41.89	55.95	145.76	301.72
	416	6.47	9	4.94	4.68	25.09
	417	57.35	39.27	56.97	42.65	196.24
	418	5.57	2.42	2.89	2.13	13.01
	420	266.8	284.63	265.55	243.2	1,060.18
	421	182.95	168.31	211.99	371.62	934.87
	422	141.64	144.34	143.96	136.15	566.09
	423	159.63	129.31	143.31	128.23	560.48
	424	169.45	152.04	160.26	215.04	696.79
	425	30.97	26.18	52.77	16.45	126.37
	426	6.44	3.9	6.31	3.9	20.55
	427	100.5	85.55	93.22	94.97	374.24
TOTAL		2,970.11	2,647.46	2,821.70	3,354.09	11,793.36

STATE:	LOUISIANA				
ZIP CODE	QUARTER 1	QUARTER 2	QUARTER 3	QUARTER 4	TOTAL GRAMS
700	261.11	341.27	260.48	228.83	1,091.69
701	198.22	207.24	211.53	211.19	828.18
703	127.6	125.2	109.67	104.84	467.31
704	504.63	447.75	420.99	418.75	1,792.12
705	989.45	880.12	781.67	928.03	3,579.27
706	189.03	196.12	139.3	144.31	668.76
707	354.84	234.52	314.21	334.96	1,238.53
708	305.08	265.85	286.43	242.88	1,100.24
710	32.81	30.83	33.88	32.21	129.73
711	373.2	169.65	185.81	157.03	885.69
712	128.39	116.7	113.34	90.13	448.56
713	177.73	164.85	169.94	151.27	663.79
714	72.36	75.08	68.73	63.15	279.32
TOTAL	3,714.45	3,255.18	3,095.98	3,107.58	13,173.19

STATE:	MAINE				
ZIP CODE	QUARTER 1	QUARTER 2	QUARTER 3	QUARTER 4	TOTAL GRAMS
39	16.52	12.95	15.74	11.38	56.59

	40	384.74	338.97	323.06	325.24	1,372.01
	41	233.82	316.76	262.28	358.92	1,171.78
	42	115.84	122.3	101.52	141.39	481.05
	43	303.81	639.6	130.88	356.66	1,430.95
	44	224.39	193.88	187.59	237.1	842.96
	45	62.7	56.49	51.74	55.35	226.28
	46	59.23	92.61	83.5	70.07	305.41
	47	32.21	43.84	23.39	21.85	121.29
	48	42.84	31.21	32.9	27.06	134.01
	49	135.72	156.02	109.74	142.59	544.07
TOTAL		1,611.82	2,004.63	1,322.34	1,747.61	6,686.40

STATE: MARYLAND

ZIP CODE		QUARTER 1	QUARTER 2	QUARTER 3	QUARTER 4	TOTAL GRAMS
	206	470.11	433.17	415.4	422.41	1,741.09
	207	785.41	730.91	722.06	634.52	2,872.90
	208	1,224.84	1,129.47	1,032.47	1,049.94	4,436.72
	209	231.9	229.08	212.34	253.89	927.21
	210	1,455.49	1,307.21	1,294.24	1,333.21	5,390.15
	211	578.82	549.51	505.3	496.7	2,130.33
	212	2,320.61	2,288.78	2,128.84	2,018.49	8,756.72
	214	147.44	143.34	125.91	129.13	545.82
	215	100.95	84.43	82.68	86.89	354.95
	216	149.64	130.36	120.11	103.92	504.03
	217	772.2	787.69	719.17	708.13	2,987.19
	218	268.54	260.47	300.89	277.38	1,107.28
	219	428.01	415.12	414.23	402.65	1,660.01
TOTAL		8,933.96	8,489.54	8,073.64	7,917.26	33,414.40

STATE: MASSACHUSETTS

ZIP CODE		QUARTER 1	QUARTER 2	QUARTER 3	QUARTER 4	TOTAL GRAMS
	10	509.51	511.36	508.25	513.79	2,042.91
	11	412.92	406.77	348.47	338.41	1,506.57
	12	113.35	103.12	105.15	121.01	442.63
	13	54.04	49.62	60.16	62.92	226.74

	14	91.85	90.27	88.84	70.54	341.5
	15	174.49	193.47	169.66	189.42	727.04
	16	176.96	196.08	193.2	225.02	791.26
	17	435.88	401.4	324.05	380.11	1,541.44
	18	1,157.77	1,296.18	1,017.24	1,223.12	4,694.31
	19	426.82	414.89	392.7	381.03	1,615.44
	20	634.23	535.18	423.11	475	2,067.52
	21	1,690.06	1,566.64	1,647.80	1,667.75	6,572.25
	22	324.13	345.9	262.97	316.15	1,249.15
	23	502.56	410.08	360.5	370.12	1,643.26
	24	248.79	233.1	223.65	252.37	957.91
	25	110.24	103.49	91.73	82.4	387.86
	26	163.2	151.43	159.33	157.88	631.84
	27	565.98	515.95	490.33	462.28	2,034.54
TOTAL		7,792.78	7,524.93	6,867.14	7,289.32	29,474.17

STATE:	MICHIGAN					
ZIP CODE	QUARTER 1	QUARTER 2	QUARTER 3	QUARTER 4	TOTAL GRAMS	
	480	1,394.70	1,361.35	1,167.81	1,173.37	5,097.23
	481	1,262.06	1,290	1,142.65	1,192.29	4,887
	482	850.6	842.31	875.15	893.11	3,461.17
	483	1,686.54	1,033.86	1,499.93	1,356.37	5,576.70
	484	382.97	396.3	336.17	360.88	1,476.32
	485	211.33	206.12	213.61	279.58	910.64
	486	460.79	415.44	403.47	395.69	1,675.39
	487	136.65	135.25	116.45	119.34	507.69
	488	347.14	341.38	342.37	305.3	1,336.19
	489	191.13	217.05	187.23	194.3	789.71
	490	591.35	546.58	533.9	534.95	2,206.78
	491	48.67	38.87	43.58	37.12	168.24
	492	158.34	173.7	168.86	174.87	675.77
	493	150.8	121.75	121.88	108.87	503.3
	494	334.14	307.27	373.66	363.75	1,378.82
	495	433.9	454.89	463.13	417.71	1,769.63
	496	407.88	424.95	440.5	395.76	1,669.09
	497	250.55	261.1	232.47	234.99	979.11

	498	228.07	226.11	233.57	209.02	896.77
	499	54.69	45.77	41.76	44.26	186.48
TOTAL		9,582.30	8,840.05	8,938.15	8,791.53	36,152.03

STATE:		MINNESOTA				
ZIP CODE		QUARTER 1	QUARTER 2	QUARTER 3	QUARTER 4	TOTAL GRAMS
	550	551.85	518.61	531.02	522.07	2,123.55
	551	1,087.22	1,251.14	1,127.06	1,231.72	4,697.14
	553	672.75	657.66	643.64	701.32	2,675.37
	554	1,917.34	1,953.57	2,035.15	1,847.81	7,753.87
	556	5.92	4.17	15.01	7.08	32.18
	557	153.25	172.04	163.58	166.54	655.41
	558	167.93	176.81	187.17	177.15	709.06
	559	569.03	537.88	545.37	451.31	2,103.59
	560	147.33	130.21	135.57	124.89	538
	561	13.13	15.17	14.51	17.26	60.07
	562	84.17	57.88	53.84	57.55	253.44
	563	207.91	194.07	164.15	197.1	763.23
	564	132.4	113.64	133.75	118.51	498.3
	565	163.79	178.99	151.49	184.27	678.54
	566	43.59	46.98	40.75	38.49	169.81
	567	19.82	26.03	17.56	16.36	79.77
TOTAL		5,937.43	6,034.85	5,959.62	5,859.43	23,791.33

STATE:		MISSISSIPPI				
ZIP CODE		QUARTER 1	QUARTER 2	QUARTER 3	QUARTER 4	TOTAL GRAMS
	386	169.17	178.62	157.17	142.31	647.27
	387	16.16	14.75	33.94	13.28	78.13
	388	168.27	154.43	128.28	171.45	622.43
	389	24.45	31.23	28.82	23.58	108.08
	390	110	104.15	97.8	81.11	393.06
	391	3,129.62	4,550.10	4,149.02	4,605.48	16,434.22
	392	314.93	276.8	291.62	382.25	1,265.60
	393	92.58	76.87	70.33	84.86	324.64
	394	368.55	358.75	302.2	351.61	1,381.11

	395	504.6	362.67	385.85	501.92	1,755.04
	396	90.09	71.33	57.36	33.89	252.67
	397	39.43	41.42	33.93	26.63	141.41
TOTAL		5,027.85	6,221.12	5,736.32	6,418.37	23,403.66

STATE: MISSOURI

ZIP CODE		QUARTER 1	QUARTER 2	QUARTER 3	QUARTER 4	TOTAL GRAMS
	630	841.58	596.1	663.95	771.06	2,872.69
	631	1,205.10	1,089.48	1,039.50	1,006.96	4,341.04
	633	282.93	264.2	260.75	229.37	1,037.25
	634	31.32	27.23	42.5	47.69	148.74
	635	76.16	67.02	70.27	66.41	279.86
	636	183.07	194.19	168.48	159.85	705.59
	637	106.44	109.93	106.98	85.9	409.25
	638	58.72	58.22	54.56	56.66	228.16
	639	49.91	49.97	63.3	52.16	215.34
	640	515.04	461.3	433.38	433.12	1,842.84
	641	472.79	454.74	407.03	436.63	1,771.19
	644	129.41	115.51	115.73	95.33	455.98
	645	532.43	499.68	468.23	437.24	1,937.58
	646	143.51	119.05	126.09	119.19	507.84
	647	123.04	83.42	87.78	68.19	362.43
	648	261.5	270.41	263.24	239.41	1,034.56
	650	93.26	100.2	103.4	104.91	401.77
	651	52.07	41.76	35.88	37.8	167.51
	652	312.75	284.53	321.54	438.88	1,357.70
	653	62.5	55.86	51.83	54.61	224.8
	654	38.3	33.55	40.12	24.21	136.18
	655	88.85	69.02	68.19	58.8	284.86
	656	334.94	311.79	316.55	309.1	1,272.38
	657	537.83	510.05	430.64	466.73	1,945.25
	658	466.96	497.87	447.54	448.98	1,861.35
TOTAL		7,000.41	6,365.08	6,187.46	6,249.19	25,802.14

STATE: MONTANA

ZIP CODE	QUARTER 1	QUARTER 2	QUARTER 3	QUARTER 4	TOTAL GRAMS
590	104.01	75.01	104.68	91.22	374.92
591	538.52	503.24	487.99	462.56	1,992.31
592	42	29.77	38.23	28.79	138.79
593	64.55	62.59	57.85	60.18	245.17
594	153.44	124.91	110.26	116.53	505.14
595	14.81	9.65	10.89	10.64	45.99
596	107.67	122.46	122.46	129.94	482.53
597	171.08	217.78	320.6	223.04	932.5
598	291.37	311.51	334.33	282.63	1,219.84
599	193.7	156.29	145.93	131.38	627.3
TOTAL	1,681.15	1,613.21	1,733.22	1,536.91	6,564.49

STATE: NEBRASKA

ZIP CODE	QUARTER 1	QUARTER 2	QUARTER 3	QUARTER 4	TOTAL GRAMS
680	224.83	226.13	184.5	172.16	807.62
681	595.75	590.08	510.17	550.41	2,246.41
683	25.63	30.89	37.15	33.86	127.53
684	22.71	24.07	19.97	25.32	92.07
685	155.04	172.59	150.46	122.65	600.74
686	22.61	28.55	27.03	30.21	108.4
687	45.94	50.53	44.77	38.64	179.88
688	103.48	117.35	102.36	105.46	428.65
689	44.83	24.39	28.78	48.7	146.7
690	22.7	19.52	13.72	15.06	71
691	58.34	52.58	55.5	42.45	208.87
692	5.52	4.61	1.72	1.29	13.14
693	49.88	34.71	46.87	44.28	175.74
TOTAL	1,377.26	1,376	1,223	1,230.49	5,206.75

STATE: NEVADA

ZIP CODE	QUARTER 1	QUARTER 2	QUARTER 3	QUARTER 4	TOTAL GRAMS
890	712.29	724.86	691.74	667.88	2,796.77
891	1,803.81	1,646.75	1,536.08	1,563.61	6,550.25
893	0.83	2.62	1.17	1.21	5.83

	894	207.46	186.44	197.01	184.22	775.13
	895	297.95	278.4	264.81	238.79	1,079.95
	897	121.3	140.49	110.65	101.98	474.42
	898	30.17	28.11	31.17	24.68	114.13
TOTAL		3,173.81	3,007.67	2,832.63	2,782.37	11,796.48

STATE: NEW HAMPSHIRE

ZIP CODE	QUARTER 1	QUARTER 2	QUARTER 3	QUARTER 4	TOTAL GRAMS
30	397.98	374.82	347.76	350.81	1,471.37
31	341.98	383.39	418.24	250.77	1,394.38
32	196.15	166.64	164.71	154.34	681.84
33	205.2	184.7	189.62	228.63	808.15
34	95.55	102.05	83.07	79.03	359.7
35	97.5	109.71	95.78	88.27	391.26
36	10.6	9.68	6.38	5.66	32.32
37	285.75	248.04	280.34	275.03	1,089.16
38	428.68	418.43	391.58	396.86	1,635.55
TOTAL	2,059.39	1,997.46	1,977.48	1,829.40	7,863.73

STATE: NEW JERSEY

ZIP CODE	QUARTER 1	QUARTER 2	QUARTER 3	QUARTER 4	TOTAL GRAMS
70	1,640.81	1,409.10	1,309.19	1,376.06	5,735.16
71	260.31	225.94	217.08	187.09	890.42
72	86.4	71.24	90.01	83.49	331.14
73	76.1	74.68	68.03	61.14	279.95
74	889.95	763.06	714.67	1,038.55	3,406.23
75	138.66	188.17	183.88	116.55	627.26
76	890.81	886.89	837.01	804.41	3,419.12
77	786	766.85	665.46	705.96	2,924.27
78	403.2	374.21	339.54	325.33	1,442.28
79	407.31	413.19	392.23	345.87	1,558.60
80	1,303.01	1,195.46	1,060.11	1,022.55	4,581.13
81	154.6	170.91	215.82	201.63	742.96
82	268.61	202.77	232.78	151.33	855.49
83	152.72	139.49	135.03	114.66	541.9

	84	25.03	22.78	30.83	21.68	100.32
	85	262.24	231.85	206.33	204.76	905.18
	86	162.06	153.83	138.09	134.33	588.31
	87	381.32	383.26	358.97	384.93	1,508.48
	88	1,099.20	1,197.78	1,093.14	1,014.57	4,404.69
	89	186.77	168.43	146.97	152.57	654.74
TOTAL		9,575.11	9,039.89	8,435.17	8,447.46	35,497.63

STATE: NEW MEXICO

ZIP CODE		QUARTER 1	QUARTER 2	QUARTER 3	QUARTER 4	TOTAL GRAMS
	870	76.66	79.26	67.57	74.62	298.11
	871	685.98	710.28	666.21	625.68	2,688.15
	873	12.28	11.58	6.78	16.26	46.9
	874	86	84.71	65.23	67.53	303.47
	875	137.5	147.62	131.83	141.89	558.84
	877	34.13	24.2	27.93	32.91	119.17
	878	2.54	4.61	2.84	2.68	12.67
	879	6.03	5.69	9.52	13.77	35.01
	880	231.02	182.47	125.52	113.55	652.56
	881	24.11	16.67	17.55	10.3	68.63
	882	130.82	115.92	116.78	109.21	472.73
	883	37.19	33.75	31.64	32.79	135.37
	884	1.1	0.97	0.88	1.07	4.02
TOTAL		1,465.36	1,417.73	1,270.28	1,242.26	5,395.63

STATE: NEW YORK

ZIP CODE		QUARTER 1	QUARTER 2	QUARTER 3	QUARTER 4	TOTAL GRAMS
	100	3,308.21	3,150.09	2,855.72	3,215.91	12,529.93
	101	41.54	49.99	51.6	41	184.13
	102	4.26	1.63	2.31	5.85	14.05
	103	526.9	499.71	757.16	765.5	2,549.27
	104	815.48	719.63	903.91	689.1	3,128.12
	105	517.43	489.69	472.78	620.71	2,100.61
	106	120.92	133.83	132.34	128.59	515.68
	107	149.07	127.87	129.71	115.46	522.11

108	73.6	64.74	51.52	50.09	239.95
109	636.84	568.2	530.46	551.86	2,287.36
110	1,137.63	1,073.47	964.92	1,019.83	4,195.85
111	163.35	168.85	180.02	151.46	663.68
112	1,422.53	1,523.89	1,260.08	1,300.59	5,507.09
113	612.13	586.04	504.16	524.97	2,227.30
114	289.09	275.74	261.62	232.27	1,058.72
115	852.78	925.78	889.68	800.08	3,468.32
116	48.27	66.1	46.63	57.03	218.03
117	2,665.24	2,587.71	2,412.94	2,344.79	10,010.68
118	200.14	223.61	225.45	146.52	795.72
119	169.12	166.65	184.9	196.45	717.12
120	214.4	198.73	256.96	195.62	865.71
121	84.96	68.88	92.04	87.51	333.39
122	362.97	346.1	386.05	267.2	1,362.32
123	73.14	76.05	66.51	60.79	276.49
124	234.02	241.91	233.57	209.62	919.12
125	365.72	354.71	365.84	354.74	1,441.01
126	144.95	133.95	147.47	158.33	584.7
127	189.53	139.88	145.43	134.33	609.17
128	254.86	234.02	231.72	211.01	931.61
129	344.59	336.72	356.77	351.23	1,389.31
130	198.7	177.04	180.28	169.32	725.34
131	93.63	107.95	104.97	104	410.55
132	159.54	164.54	149.91	140.4	614.39
133	105.82	123.69	81.05	84.5	395.06
134	91.99	96.55	74.89	86.34	349.77
135	76	66.88	60.03	63.79	266.7
136	153.62	125.13	127.49	130.34	536.58
137	80.15	75.91	68	65.5	289.56
138	153.77	146.03	137.95	142.93	580.68
139	90.55	64.3	53.98	54.23	263.06
140	305.39	307.63	290.89	284.33	1,188.24
141	154.32	162.18	139.76	122.19	578.45
142	585.48	534.84	544.42	541.6	2,206.34
143	54.02	42.84	30.56	27.8	155.22
144	349.43	330.48	317.88	291.21	1,289

145	283.09	286.3	273.5	213.02	1,055.91
146	918.47	1,003.42	980.03	1,008.71	3,910.63
147	71.15	81.19	79.65	76.88	308.87
148	270.51	281.27	258.39	248.45	1,058.62
149	49.3	55.89	56.3	70.43	231.92
TOTAL	20,268.60	19,768.23	19,110.20	18,914.41	78,061.44

STATE: NORTH CAROLINA

ZIP CODE	QUARTER 1	QUARTER 2	QUARTER 3	QUARTER 4	TOTAL GRAMS
270	338.92	285.32	271.98	281.01	1,177.23
271	652.11	525.66	517.36	452.94	2,148.07
272	693.53	736.76	678.92	622.57	2,731.78
273	399.93	397.34	391.37	394.18	1,582.82
274	310.83	262.98	312.01	307.6	1,193.42
275	1,642.09	1,229.42	1,319	1,266.10	5,456.61
276	447.09	438.75	427.58	407.76	1,721.18
277	501.97	556.43	450.63	609.25	2,118.28
278	631.04	448.33	434.43	379.16	1,892.96
279	280.96	274.83	264.13	281.76	1,101.68
280	743.86	709.08	726.01	678.81	2,857.76
281	481.97	476.96	470.48	457.39	1,886.80
282	1,001.88	784.09	979.41	636.71	3,402.09
283	758.04	715.14	705.46	652.42	2,831.06
284	578.6	549.08	542.3	521.7	2,191.68
285	476.01	479.01	474.86	466.92	1,896.80
286	869.17	1,079.53	866.9	817.85	3,633.45
287	469.6	464.52	432.12	351.34	1,717.58
288	291.4	265.75	304.61	301.99	1,163.75
289	86.08	85.63	84.13	76.66	332.5
TOTAL	11,655.08	10,764.61	10,653.69	9,964.12	43,037.50

STATE: NORTH DAKOTA

ZIP CODE	QUARTER 1	QUARTER 2	QUARTER 3	QUARTER 4	TOTAL GRAMS
580	30.11	34.79	32.04	33.81	130.75
581	155.08	177.89	149.89	144.36	627.22

	582	57.09	49.44	71.49	65.35	243.37
	583	13.44	9.39	10.44	11.13	44.4
	584	16.79	25.25	11.04	10.21	63.29
	585	263.89	270.18	241.02	257.04	1,032.13
	586	27.86	31.37	30.49	28.93	118.65
	587	54.08	55.71	55.79	60.4	225.98
	588	26.07	24.3	28.26	30.91	109.54
TOTAL		644.41	678.32	630.46	642.14	2,595.33

STATE: OHIO

ZIP CODE		QUARTER 1	QUARTER 2	QUARTER 3	QUARTER 4	TOTAL GRAMS
	430	844.28	742.12	633.46	769.69	2,989.55
	431	331.45	299.97	237.31	208.33	1,077.06
	432	1,381.49	1,305.49	1,412.07	1,425.03	5,524.08
	433	111.92	128.73	145.01	173.32	558.98
	434	74.5	80.34	64.41	52.81	272.06
	435	268.87	330.76	262.77	369.7	1,232.10
	436	269.81	237.38	255.4	263.19	1,025.78
	437	152.14	155.91	187.89	180.24	676.18
	438	37.28	20.79	19.73	25.54	103.34
	439	114.17	119.04	97.57	115.47	446.25
	440	379.39	383.08	380.11	335.24	1,477.82
	441	1,120.33	1,091.76	1,114.05	1,042.53	4,368.67
	442	401.29	350.45	392.24	375.32	1,519.30
	443	338.92	342.36	356.15	272.62	1,310.05
	444	128.66	129.12	131.27	122.94	511.99
	445	80.07	103.31	86.08	101.67	371.13
	446	435.92	423.07	396	378.66	1,633.65
	447	354.84	342.21	385.57	378.3	1,460.92
	448	236.1	210.13	227.76	263.09	937.08
	449	32.76	40.94	34.95	56.65	165.3
	450	572.16	504.06	611.54	492.67	2,180.43
	451	164.92	190.15	165.4	188.75	709.22
	452	608.31	617.52	566.49	626.52	2,418.84
	453	194.04	154.37	168.25	154.36	671.02
	454	328.95	344.68	359.71	292.43	1,325.77

	455	68.63	64.82	54.02	63.04	250.51
	456	226.93	206.66	183.25	234.87	851.71
	457	93.04	82.33	82.88	69.71	327.96
	458	246.09	298.97	225.87	215.02	985.95
TOTAL		9,597.26	9,300.52	9,237.21	9,247.71	37,382.70

STATE: OKLAHOMA

ZIP CODE		QUARTER 1	QUARTER 2	QUARTER 3	QUARTER 4	TOTAL GRAMS
	730	701.48	734.33	634.89	586.31	2,657.01
	731	899.09	884.01	877.37	835.3	3,495.77
	734	78.97	95.71	81.66	85.15	341.49
	735	229.85	250.06	249.79	202.69	932.39
	736	17.61	20.47	22.73	21.9	82.71
	737	55.75	55.96	59.92	63.95	235.58
	738	12.03	12.61	15.24	15.2	55.08
	739	13.94	24.22	6.88	6.7	51.74
	740	432.5	454.68	400.29	413.91	1,701.38
	741	519.41	496.8	537.35	528.9	2,082.46
	743	77.19	81.12	94.87	100.7	353.88
	744	154.68	184.87	170.94	173.02	683.51
	745	114.55	101.42	99.96	89.19	405.12
	746	23.48	22.49	17.12	23.45	86.54
	747	127.14	132.19	107.66	102.19	469.18
	748	110.34	117.88	99.59	102.07	429.88
	749	98.09	109.07	159.94	169.09	536.19
TOTAL		3,666.10	3,777.89	3,636.20	3,519.72	14,599.91

STATE: OREGON

ZIP CODE		QUARTER 1	QUARTER 2	QUARTER 3	QUARTER 4	TOTAL GRAMS
	970	812.85	767	758.81	691	3,029.66
	971	340.36	352.32	345.31	327.45	1,365.44
	972	1,292.72	1,281.10	1,266.82	1,274.37	5,115.01
	973	706.93	667.39	725.1	651.53	2,750.95
	974	903.27	910.16	859.06	769.25	3,441.74
	975	561.29	548.91	480.36	438.2	2,028.76

	976	40.72	49.07	43.16	30.63	163.58
	977	199.24	187.59	175.1	149.38	711.31
	978	163.77	157.79	153.11	130.97	605.64
	979	34.98	28.28	34.66	35.82	133.74
TOTAL		5,056.13	4,949.61	4,841.49	4,498.60	19,345.83

STATE:	PENNSYLVANIA				
ZIP CODE	QUARTER 1	QUARTER 2	QUARTER 3	QUARTER 4	TOTAL GRAMS
150	390.21	352.32	356.29	351	1,449.82
151	133.41	148.1	121.21	115.41	518.13
152	887.45	862.3	819.87	797.42	3,367.04
153	122.15	107.01	101.84	106.49	437.49
154	81.49	71.65	76.69	90.14	319.97
155	81.36	76.1	73.28	73.36	304.1
156	232.21	174.68	180.53	172.95	760.37
157	60.93	62.21	68.39	57.34	248.87
158	71.52	79.8	78.21	62.4	291.93
159	96.79	106.13	126.86	119.18	448.96
160	100.32	86.95	80.46	77.65	345.38
161	175.11	179.34	142.23	157.91	654.59
162	39.8	46.28	48.5	36.69	171.27
163	372.15	327	252.27	241.17	1,192.59
164	86.11	79.4	75.76	85.1	326.37
165	119.39	120.5	118.47	114.11	472.47
166	170.87	170.69	160.33	164.1	665.99
167	18.26	24.13	28.66	19.03	90.08
168	91.26	99.5	94.61	76.1	361.47
169	34.74	35.38	24.32	34.68	129.12
170	657.18	658.05	646.18	608.98	2,570.39
171	193.58	201.14	187.55	153.71	735.98
172	155.86	152.15	134.1	144.39	586.5
173	255.24	243.75	217.4	211.52	927.91
174	163.25	146.33	131.84	130.57	571.99
175	225.31	219.65	236.08	216	897.04
176	332.05	298.5	330.28	390.75	1,351.58
177	137.3	127.6	87.36	94.11	446.37

	178	329.19	350.81	292.52	275.46	1,247.98
	179	31.7	26.79	31.87	36.51	126.87
	180	433.52	431.85	421.9	402.33	1,689.60
	181	322.55	309.84	332.06	286.85	1,251.30
	182	52.38	63.96	64.02	61.8	242.16
	183	198.51	164.62	160.63	174.17	697.93
	184	169.85	162.18	180.69	174.06	686.78
	185	165.2	163.49	166.98	230.53	726.2
	186	84.09	78.8	82.33	80.98	326.2
	187	884.01	823.34	733.58	732.09	3,173.02
	188	58.32	57.53	50.57	39.89	206.31
	189	366.91	353.92	377.35	330.75	1,428.93
	190	5,983.58	3,071.16	2,892.68	3,162.96	15,110.38
	191	1,741.44	1,723.58	1,598.22	1,795.38	6,858.62
	193	417.76	587.04	421.56	419.42	1,845.78
	194	769.37	803.32	866.04	777.22	3,215.95
	195	91.27	87.31	91.19	74.34	344.11
	196	171.25	172.09	166.5	169.74	679.58
TOTAL		17,756.20	14,688.27	13,930.26	14,126.74	60,501.47

STATE: PUERTO RICO

ZIP CODE		QUARTER 1	QUARTER 2	QUARTER 3	QUARTER 4	TOTAL GRAMS
	6	15.6	13.83	9.93	8.16	47.52
	7	6.56	6.03	4.61	6.92	24.12
	9	53.53	45.22	66.39	59.86	225
TOTAL		75.69	65.08	80.93	74.94	296.64

STATE: RHODE ISLAND

ZIP CODE		QUARTER 1	QUARTER 2	QUARTER 3	QUARTER 4	TOTAL GRAMS
	28	536.93	583.51	486.75	460	2,067.19
	29	349.93	451.24	324.34	340.26	1,465.77
TOTAL		886.86	1,034.75	811.09	800.26	3,532.96

STATE: SOUTH CAROLINA

ZIP CODE	QUARTER 1	QUARTER 2	QUARTER 3	QUARTER 4	TOTAL GRAMS
290	410.68	386.48	393.16	420.51	1,610.83
291	570.59	598.58	611.77	702.09	2,483.03
292	590.39	646.16	570.24	573.1	2,379.89
293	294.99	273.85	259.75	243.82	1,072.41
294	999.07	938.89	974.01	924.83	3,836.80
295	1,164.13	1,118.95	1,024.25	1,032.75	4,340.08
296	980.69	910.95	855.66	795.57	3,542.87
297	215.17	228.06	230.42	184.16	857.81
298	261.66	243.19	229.68	252.78	987.31
299	514.74	484.86	361.06	504.76	1,865.42
TOTAL	6,002.11	5,829.97	5,510	5,634.37	22,976.45

STATE: SOUTH DAKOTA

ZIP CODE	QUARTER 1	QUARTER 2	QUARTER 3	QUARTER 4	TOTAL GRAMS
570	69.21	59.95	70.77	59.73	259.66
571	253.37	259.71	258.41	393.17	1,164.66
572	21.7	24.22	15.94	19.76	81.62
573	23.37	31.26	21.89	15.57	92.09
574	23.11	20.79	20.12	18.99	83.01
575	19.7	18.59	20.69	20.99	79.97
576	4.33	9.11	5.85	7.97	27.26
577	104.89	101.95	99.17	105.42	411.43
TOTAL	519.68	525.58	512.84	641.6	2,199.70

STATE: TENNESSEE

ZIP CODE	QUARTER 1	QUARTER 2	QUARTER 3	QUARTER 4	TOTAL GRAMS
370	542.39	536.39	528.1	574.97	2,181.85
371	427.81	467.68	479.97	436.67	1,812.13
372	881.3	771.78	906.42	913.86	3,473.36
373	470.87	490.35	472.61	526.11	1,959.94
374	359.19	335.87	300.61	293.08	1,288.75
376	355.06	320.16	314.94	362.43	1,352.59
377	102.21	124.93	114.96	114.67	456.77
378	237.85	234.52	237.33	254.28	963.98

	379	389.91	445.21	518.28	250.77	1,604.17
	380	215.82	260.72	216.9	214.22	907.66
	381	3,745.82	1,764.20	1,926.92	1,509.92	8,946.86
	382	63.2	63.49	59.82	51.49	238
	383	114.11	96.27	120.25	107.13	437.76
	384	104.86	86.38	62.21	62.44	315.89
	385	192.21	184.22	190.08	150.15	716.66
TOTAL		8,202.61	6,182.17	6,449.40	5,822.19	26,656.37

STATE: TEXAS

ZIP CODE	QUARTER 1	QUARTER 2	QUARTER 3	QUARTER 4	TOTAL GRAMS
750	2,790.08	2,630.88	2,559.04	2,294.31	10,274.31
751	473.63	495.77	478.28	489.99	1,937.67
752	1,422.38	1,567.63	1,489.86	1,383.46	5,863.33
753	121.08	112.34	104.73	127.66	465.81
754	532.17	508.36	455.36	504.64	2,000.53
755	85.34	70.3	72.47	74.74	302.85
756	606.42	699.42	596.36	977.79	2,879.99
757	273.68	297.02	399.72	288.82	1,259.24
758	54.54	47.7	50.8	35.66	188.7
759	183.51	179.21	190.23	171.33	724.28
760	1,143.44	1,124.78	1,010.42	950.03	4,228.67
761	1,170.20	1,117.91	1,187.56	1,136	4,611.67
762	647.7	658.57	661.68	579.96	2,547.91
763	282.36	270.47	261.15	253.02	1,067
764	115.44	112.64	104.16	95.35	427.59
765	418.99	438.95	390.6	411.94	1,660.48
766	59.76	46.9	50.33	56.56	213.55
767	90.89	83.48	95.36	92.18	361.91
768	51.93	55.97	82.62	52.99	243.51
769	73.09	75.07	52.27	50.02	250.45
770	2,963.47	2,710.64	2,615.67	2,231.79	10,521.57
773	1,025.19	1,004.30	974.6	989.24	3,993.33
774	834.34	740.28	738.86	716.6	3,030.08
775	1,083.33	1,272.79	1,298.80	1,006.54	4,661.46
776	489.7	502.59	458.1	455.37	1,905.76

	777	244.5	248.52	248.86	247.38	989.26
	778	127.81	129.04	117.43	102.08	476.36
	779	457.63	440.6	444.09	425.62	1,767.94
	780	239.13	252.45	202.35	227.29	921.22
	781	308.71	302.99	305.34	287.48	1,204.52
	782	1,940.07	1,957.60	1,897.03	1,865.52	7,660.22
	783	133.26	127.9	110.54	112.95	484.65
	784	278.83	290.89	257.16	239.53	1,066.41
	785	345.56	292.97	321.78	309.34	1,269.65
	786	969.74	877.32	924.9	855.12	3,627.08
	787	1,063.01	939.21	974.34	819.48	3,796.04
	788	33.65	35.27	37.3	36.98	143.2
	789	47.96	40.88	47.76	38.83	175.43
	790	70.96	72.23	78.97	83.23	305.39
	791	188.36	229.45	206.71	200.39	824.91
	792	5.45	7.89	6.56	6.74	26.64
	793	71.46	85.06	83.24	86.68	326.44
	794	399.62	373.65	345.83	369.29	1,488.39
	795	41.36	39.58	37.71	32.88	151.53
	796	85.52	83.7	87.07	72.67	328.96
	797	227.51	228.22	211.17	201.4	868.3
	798	13.33	11.66	2.71	16.18	43.88
	799	459.82	482.69	460.81	438.45	1,841.77
TOTAL		24,745.91	24,373.74	23,788.69	22,501.50	95,409.84

STATE: UTAH

ZIP CODE		QUARTER 1	QUARTER 2	QUARTER 3	QUARTER 4	TOTAL GRAMS
	840	865.82	963.13	1,083.41	1,017.76	3,930.12
	841	893.37	853.42	895.82	1,110.09	3,752.70
	843	121.45	132.64	136.91	148.79	539.79
	844	192.81	198.05	197.83	210.86	799.55
	845	20.82	20.09	23.21	31.81	95.93
	846	214.33	211.63	213.27	209.71	848.94
	847	249.95	232.06	232.35	224.06	938.42
TOTAL		2,558.55	2,611.02	2,782.80	2,953.08	10,905.45

STATE: VERMONT

ZIP CODE	QUARTER 1	QUARTER 2	QUARTER 3	QUARTER 4	TOTAL GRAMS
50	61.31	63.75	49.15	35.85	210.06
51	53.77	48.21	55.88	50.23	208.09
52	41.21	52.07	42.47	39.8	175.55
53	29.3	34.3	40.7	31.38	135.68
54	697.23	725.84	626.43	575.13	2,624.63
56	128.01	117.49	117.12	115.18	477.8
57	159.03	164.8	206.26	254.88	784.97
58	68.97	63.37	58.74	64.94	256.02
TOTAL	1,238.83	1,269.83	1,196.75	1,167.39	4,872.80

STATE: VIRGIN ISLANDS

ZIP CODE	QUARTER 1	QUARTER 2	QUARTER 3	QUARTER 4	TOTAL GRAMS
8	26.6	18.71	13.94	14.9	74.15
TOTAL	26.6	18.71	13.94	14.9	74.15

STATE: VIRGINIA

ZIP CODE	QUARTER 1	QUARTER 2	QUARTER 3	QUARTER 4	TOTAL GRAMS
201	1,179.88	1,114.83	987.19	1,059.46	4,341.36
220	665.64	628.21	564.35	466.73	2,324.93
221	463.54	396.36	355.68	359.41	1,574.99
222	170.99	161.75	144.73	126.12	603.59
223	239.73	218.81	198.18	204.43	861.15
224	398.47	377.25	344.61	337.83	1,458.16
225	134.93	129.96	109.21	125.92	500.02
226	554.78	507.49	482.29	456.65	2,001.21
227	51.34	64.69	56.42	46.31	218.76
228	338.81	325.08	289.61	289.14	1,242.64
229	693.1	563.96	450.04	503.12	2,210.22
230	349.07	340.23	332.67	277.26	1,299.23
231	648.31	554.58	488.2	560.82	2,251.91
232	1,371.12	1,145.07	1,166.87	1,136.24	4,819.30

	233	536.91	444.66	415.73	430.7	1,828
	234	1,035.16	946.61	869.48	810.85	3,662.10
	235	555.05	575	530.17	516.73	2,176.95
	236	670.31	556.18	508.55	483.28	2,218.32
	237	283.49	233.52	225.96	251.38	994.35
	238	307.91	307.35	253.37	242.89	1,111.52
	239	65.27	74.68	74.77	65.4	280.12
	240	565.04	501.21	504.78	559.43	2,130.46
	241	398.71	352.12	345.3	362.35	1,458.48
	242	186.27	200.02	190.63	175.45	752.37
	243	124.55	112.86	105.55	107.06	450.02
	244	176.23	159.11	139.51	154.04	628.89
	245	326.14	339.57	314.45	319.21	1,299.37
	246	65.51	55.15	70.52	54.73	245.91
TOTAL		12,556.26	11,386.31	10,518.82	10,482.94	44,944.33

STATE: WASHINGTON

ZIP CODE		QUARTER 1	QUARTER 2	QUARTER 3	QUARTER 4	TOTAL GRAMS
	980	1,842.58	1,681.19	1,762.27	1,680.73	6,966.77
	981	1,872.75	2,086.69	1,996.36	1,542.90	7,498.70
	982	1,457.67	1,370.11	1,298.68	1,315.70	5,442.16
	983	945.21	897.78	857.89	887.8	3,588.68
	984	573.93	595.3	497.22	524.45	2,190.90
	985	838.31	785.7	789.03	694.35	3,107.39
	986	1,149.97	1,226.58	1,065.46	1,040.50	4,482.51
	988	192.23	287.6	267.57	171.11	918.51
	989	292.29	319.52	254.99	300.51	1,167.31
	990	91.3	86.67	107.03	93.31	378.31
	991	124.34	124.11	123.41	125.13	496.99
	992	1,004.03	999.88	907.53	926.36	3,837.80
	993	708.83	570.51	572.48	478.97	2,330.79
	994	85.09	80.85	163.52	90.54	420
TOTAL		11,178.53	11,112.49	10,663.44	9,872.36	42,826.82

STATE: WEST VIRGINIA

ZIP CODE	QUARTER 1	QUARTER 2	QUARTER 3	QUARTER 4	TOTAL GRAMS
247	43.51	44.94	56.75	42.88	188.08
248	9.75	4.79	6.21	8.87	29.62
249	23.81	27.62	30.07	24.42	105.92
250	5.39	2.06	5.25	4.04	16.74
251	8.64	96.88	13.8	4.97	124.29
252	9.92	5.03	6.54	5.79	27.28
253	227.17	131.45	113.85	93.09	565.56
254	409.09	382.61	379.35	354.26	1,525.31
255	44.64	43.65	50.37	42.93	181.59
256	11.98	9.3	9.19	7.66	38.13
257	84.85	75.63	116.42	84.07	360.97
258	132.65	99.09	92.9	92.92	417.56
259	17.02	21.14	8.47	12.77	59.4
260	92.57	91	73.95	82.58	340.1
261	59.81	62.3	70.89	49.95	242.95
262	20.78	20.97	20.94	21.53	84.22
263	95.4	81.98	87.48	85.73	350.59
264	14.68	14.89	16.18	13.4	59.15
265	273.49	253.19	229.38	261.13	1,017.19
266	18.34	11.95	10.27	10.44	51
267	50.45	48.43	32.42	39.65	170.95
268	31.87	22.08	22.92	20.67	97.54
TOTAL	1,685.81	1,550.98	1,453.60	1,363.75	6,054.14

STATE: WISCONSIN

ZIP CODE	QUARTER 1	QUARTER 2	QUARTER 3	QUARTER 4	TOTAL GRAMS
530	274.29	267.32	239.11	305.14	1,085.86
531	470.37	356.99	400.71	464.98	1,693.05
532	797.47	719.04	707.53	757	2,981.04
534	79.92	69.22	68.2	60.55	277.89
535	274.31	255.53	226.33	247.19	1,003.36
537	268.28	313.9	242.54	266.49	1,091.21
538	47.19	34.47	39.64	26.02	147.32
539	77.56	85.05	77.2	70.39	310.2
540	88.37	75.2	81.84	82.47	327.88

	541	46.74	61.12	53.31	59.31	220.48
	542	45.28	32.13	39.34	36.8	153.55
	543	139.75	118.87	190.65	177.05	626.32
	544	191.68	158.53	164.4	148.16	662.77
	545	52.19	54.71	57.23	49.69	213.82
	546	261.13	235.27	228.83	251.78	977.01
	547	239.09	136.77	136.17	140.82	652.85
	548	127.68	144.18	140.61	129.36	541.83
	549	373.16	392.98	605.66	523.95	1,895.75
TOTAL		3,854.46	3,511.28	3,699.30	3,797.15	14,862.19

STATE: WYOMING

ZIP CODE		QUARTER 1	QUARTER 2	QUARTER 3	QUARTER 4	TOTAL GRAMS
	820	137.22	137.71	144.85	131.46	551.24
	822	6.53	8.59	7.33	7.94	30.39
	823	4.88	5.54	7.1	5.78	23.3
	824	128.69	103.19	97.3	103.73	432.91
	825	34.79	39.08	30.47	31.05	135.39
	826	82.87	99.16	85.59	75.19	342.81
	827	48.59	32.45	34.39	32.71	148.14
	828	39.4	38.19	35.99	41.86	155.44
	829	131.23	151.66	133.21	91.82	507.92
	830	4.44	7.4	13.05	4.75	29.64
	831	33.52	19.46	15.31	13.86	82.15
TOTAL		652.16	642.43	604.59	540.15	2,439.33

DRUG CODE: 9193 DRUG NAME: HYDROCODONE

STATE: ALABAMA

ZIP CODE		QUARTER 1	QUARTER 2	QUARTER 3	QUARTER 4	TOTAL GRAMS
	350	29,344.31	27,829.50	26,898.56	26,569.78	110,642.15
	351	14,695.22	13,917.38	13,821.14	13,662.31	56,096.05
	352	25,633.79	24,760.75	23,410.37	24,340.35	98,145.26
	354	11,497.65	11,163.93	10,693.11	10,306.43	43,661.12
	355	12,138.34	11,803.67	11,306.35	10,900.13	46,148.49

	356	24,658.52	23,643.96	22,919.27	22,350.84	93,572.59
	357	10,113.79	9,832.34	9,149.85	8,986.52	38,082.50
	358	11,780.59	11,160.83	10,407.75	10,315.37	43,664.54
	359	20,667.83	20,312.67	18,761.27	19,752.69	79,494.46
	360	12,206.03	11,467.85	10,674.47	10,747.34	45,095.69
	361	10,305.45	9,672.11	8,326.79	8,153.56	36,457.91
	362	10,144.90	9,571.85	8,958.21	9,364.30	38,039.26
	363	9,866.70	9,428.59	8,832.58	9,070.86	37,198.73
	364	6,321.70	5,683.23	5,158.94	5,263.09	22,426.96
	365	20,532.22	19,196.48	18,128.04	18,151.79	76,008.53
	366	15,977.46	15,247.07	14,557.72	14,102.03	59,884.28
	367	3,661.87	3,468.80	3,276.98	3,306.86	13,714.51
	368	7,628.04	7,295.50	7,120.14	7,319.83	29,363.51
	369	1,170.69	1,055.88	938.02	1,034.81	4,199.40
TOTAL		258,345.10	246,512.39	233,339.56	233,698.89	971,895.94

STATE: ALASKA

ZIP CODE		QUARTER 1	QUARTER 2	QUARTER 3	QUARTER 4	TOTAL GRAMS
	995	5,551.47	5,430.07	5,015.08	4,810.26	20,806.88
	996	3,904.79	3,680.86	3,525.24	3,498.60	14,609.49
	997	1,257.48	1,231.23	1,192.98	1,030.33	4,712.02
	998	651.8	669.98	555.19	507.26	2,384.23
	999	581.71	480.62	486.78	468.37	2,017.48
TOTAL		11,947.25	11,492.76	10,775.27	10,314.82	44,530.10

STATE: AMERICAN SAMOA

ZIP CODE		QUARTER 1	QUARTER 2	QUARTER 3	QUARTER 4	TOTAL GRAMS
	967	4	0.27	3.63	0	7.9
TOTAL		4	0.27	3.63	0	7.9

STATE: ARIZONA

ZIP CODE		QUARTER 1	QUARTER 2	QUARTER 3	QUARTER 4	TOTAL GRAMS
	850	17,021.07	15,620.90	14,700.79	14,539.48	61,882.24
	851	5,787.11	5,455.15	5,070.71	5,435.75	21,748.72

	852	28,070.11	26,336.32	24,240.36	24,063.61	102,710.40
	853	19,702.89	17,740.07	16,451.85	16,952.48	70,847.29
	855	2,180.12	2,160.36	1,957.86	1,941.09	8,239.43
	856	3,962.37	3,715.85	3,455.43	3,572.48	14,706.13
	857	14,278.23	13,148.04	12,724.28	12,370.04	52,520.59
	859	2,307.71	2,281.46	2,650.44	2,291.21	9,530.82
	860	2,282.09	2,163.90	2,076.41	1,886.69	8,409.09
	863	7,350.04	7,199.94	6,761.37	6,417.34	27,728.69
	864	9,037.17	8,583.58	8,160.19	8,245.16	34,026.10
	865	121.38	116.48	112.72	103.55	454.13
TOTAL		112,100.29	104,522.05	98,362.41	97,818.88	412,803.63

STATE: ARKANSAS

ZIP CODE		QUARTER 1	QUARTER 2	QUARTER 3	QUARTER 4	TOTAL GRAMS
	716	6,493.54	6,262.25	6,005.43	5,838.70	24,599.92
	717	5,492.46	5,460.89	5,317.51	5,294.07	21,564.93
	718	7,303.04	7,783.65	7,676.21	7,526.94	30,289.84
	719	11,287.64	10,359.91	9,964.36	9,636.03	41,247.94
	720	13,216.10	12,832.43	12,322.13	12,216.47	50,587.13
	721	16,287.95	15,909.94	15,339.27	15,064.50	62,601.66
	722	8,251.23	8,197.79	7,767.20	7,580.57	31,796.79
	723	8,269.52	7,981.45	7,794.55	7,740.12	31,785.64
	724	12,925.14	12,161.88	11,679.62	12,031.15	48,797.79
	725	6,931.25	7,033.24	6,513.41	6,683.10	27,161
	726	7,028.41	6,783.18	6,379.04	6,291.81	26,482.44
	727	18,120.04	17,545.19	16,025.13	16,187.05	67,877.41
	728	4,865.99	4,815.17	4,370.08	4,262.03	18,313.27
	729	13,733.40	13,342.73	12,779.12	12,716.07	52,571.32
TOTAL		140,205.71	136,469.70	129,933.06	129,068.61	535,677.08

STATE: CALIFORNIA

ZIP CODE		QUARTER 1	QUARTER 2	QUARTER 3	QUARTER 4	TOTAL GRAMS
	900	21,580.72	20,478.08	20,288.49	19,988.06	82,335.35
	902	17,272.59	17,114.49	16,641.44	16,676.92	67,705.44
	903	1,946.11	1,908.91	1,890.75	1,730.79	7,476.56

904	2,041.65	2,107.74	1,941.92	1,922.62	8,013.93
905	4,481.23	4,328.77	4,086.15	3,974.36	16,870.51
906	13,138.02	12,947.35	12,427.52	12,212.19	50,725.08
907	12,632.74	12,746.89	12,335.58	12,151.75	49,866.96
908	8,672.60	8,269.21	8,193.41	7,988.61	33,123.83
910	5,331.80	5,296.80	4,961.32	4,119.97	19,709.89
911	2,667.88	2,672.83	2,585.16	2,497.19	10,423.06
912	3,399.73	3,410.14	3,242.93	3,365.08	13,417.88
913	25,958.38	24,798.39	24,293.65	24,128.39	99,178.81
914	6,423.80	6,459.39	6,328.04	6,313.88	25,525.11
915	2,257.93	2,206.90	2,064.63	2,045.49	8,574.95
916	1,643.76	1,534.77	1,410.43	1,349.98	5,938.94
917	35,276.80	35,129.81	35,323.98	35,034.17	140,764.76
918	998.49	1,055.56	952.64	944.57	3,951.26
919	9,263.04	9,096.06	8,732.80	8,248.19	35,340.09
920	31,199.64	29,962.99	28,928.61	28,538.99	118,630.23
921	22,421.93	21,506.49	20,368.49	19,436.91	83,733.82
922	22,120.41	20,978.38	19,989.93	19,982.50	83,071.22
923	48,085.79	47,287.38	45,549.25	45,198.10	186,120.52
924	8,192.42	8,184.35	7,766.72	7,888.52	32,032.01
925	41,924.62	41,394.63	39,201	39,134.17	161,654.42
926	25,053.23	24,461.43	23,325.26	23,087.35	95,927.27
927	7,482.87	7,158.26	6,897.73	6,737.34	28,276.20
928	26,129.59	25,088.42	24,461.29	24,036.41	99,715.71
930	17,389.37	17,006.04	16,451.11	15,857.83	66,704.35
931	3,261.87	3,026.77	2,964.89	2,742.81	11,996.34
932	24,789.16	24,315.63	22,909.82	22,918.90	94,933.51
933	20,426.17	20,642.16	19,886.09	19,297	80,251.42
934	15,954.52	15,027.46	14,785.94	14,888.89	60,656.81
935	18,799.39	18,374.08	17,682.73	17,863.27	72,719.47
936	19,093.31	19,117.64	17,679.47	17,740.08	73,630.50
937	20,043.43	20,014.31	19,397.44	19,193.29	78,648.47
939	8,883.85	8,808.31	8,397.65	7,910.91	34,000.72
940	10,269.59	10,070.66	9,598.16	9,582.29	39,520.70
941	9,538.64	9,361.96	8,908.90	8,642.46	36,451.96
943	2,057.70	1,953.03	1,853.68	1,886.07	7,750.48
944	1,656.44	1,665.72	1,620.67	1,514.62	6,457.45

945	64,880.16	63,287.83	60,561.28	59,621.35	248,350.62
946	10,197.64	10,185.98	9,507.46	9,457.56	39,348.64
947	1,539.93	1,485.09	1,507.91	1,554.10	6,087.03
948	3,607.03	3,418.46	3,392.85	3,318.01	13,736.35
949	8,780.21	8,683.42	8,674.04	8,496.81	34,634.48
950	14,408.06	13,871.82	12,790.56	12,713.78	53,784.22
951	9,643.54	9,251.13	8,985.43	8,614.59	36,494.69
952	20,070.11	19,476.85	18,444.55	18,199.75	76,191.26
953	51,419.99	50,607.84	48,561.38	47,837.07	198,426.28
954	18,489.88	18,423.59	17,412.64	16,331.88	70,657.99
955	8,684.31	8,700.51	8,179.74	7,683.91	33,248.47
956	56,251.65	54,163	51,941.69	50,438.90	212,795.24
957	10,165.33	10,059.87	9,359.69	8,992.29	38,577.18
958	34,147.39	33,927.26	31,814.90	31,515.39	131,404.94
959	34,120.01	33,768.86	31,994.75	31,118.89	131,002.51
960	22,604.81	22,363.33	21,371.17	20,374.65	86,713.96
961	3,555.44	3,343.37	3,191.39	2,989.63	13,079.83
TOTAL	952,326.70	931,986.40	894,017.10	878,029.48	3,656,359.68

STATE: COLORADO

ZIP CODE	QUARTER 1	QUARTER 2	QUARTER 3	QUARTER 4	TOTAL GRAMS
800	9,797.46	9,517.71	8,953.15	8,708.99	36,977.31
801	7,999.55	7,403.15	7,143.27	7,255.66	29,801.63
802	11,126.67	10,598.99	10,352.98	9,939.68	42,018.32
803	1,034.45	988.98	934.96	921.09	3,879.48
804	2,398.97	2,096.46	2,037.63	2,081.04	8,614.10
805	7,052.47	6,881.36	6,701.63	6,583.94	27,219.40
806	2,920.80	2,849.99	2,761.60	2,735.73	11,268.12
807	965.12	909.68	913.13	875.01	3,662.94
808	1,839.70	1,765.30	1,769.29	1,595.46	6,969.75
809	8,160.80	7,735.91	7,636	7,524.20	31,056.91
810	6,049.63	5,875.10	5,583.64	5,611.19	23,119.56
811	842.69	751.4	769.63	694.62	3,058.34
812	1,516.39	1,513	1,410.91	1,415.37	5,855.67
813	1,310.25	1,205.88	1,142.49	1,100.17	4,758.79

814	1,272.56	1,239.70	1,145.80	1,144.77	4,802.83
815	2,795.21	2,730.79	2,563.61	2,606.54	10,696.15
816	1,746.82	1,508.73	1,497.10	1,406.65	6,159.30
TOTAL	68,829.54	65,572.13	63,316.82	62,200.11	259,918.60

STATE: CONNECTICUT

ZIP CODE	QUARTER 1	QUARTER 2	QUARTER 3	QUARTER 4	TOTAL GRAMS
60	4,797.48	4,471.66	4,203.80	4,146	17,618.94
61	1,348.01	1,285.53	1,189.92	1,117.10	4,940.56
62	1,066.65	970.42	912.27	921.1	3,870.44
63	2,303.32	2,274.90	2,154.67	2,115.53	8,848.42
64	5,054.55	4,774.77	4,504.97	4,503.51	18,837.80
65	1,370.86	1,300.69	1,146.75	1,222.61	5,040.91
66	948.98	903.91	845.43	810.03	3,508.35
67	2,722.20	2,532.95	2,413.41	2,400.98	10,069.54
68	1,788.05	1,655.40	1,576.43	1,505.33	6,525.21
69	299.26	278.49	261.99	259.48	1,099.22
TOTAL	21,699.36	20,448.72	19,209.64	19,001.67	80,359.39

STATE: DELAWARE

ZIP CODE	QUARTER 1	QUARTER 2	QUARTER 3	QUARTER 4	TOTAL GRAMS
197	1,840	1,721.29	1,710.75	1,650.80	6,922.84
198	1,220.17	1,174.65	1,094.77	1,085.35	4,574.94
199	3,329.53	3,020.86	3,020.59	3,091.27	12,462.25
TOTAL	6,389.70	5,916.80	5,826.11	5,827.42	23,960.03

STATE: DISTRICT OF COLUMBIA

ZIP CODE	QUARTER 1	QUARTER 2	QUARTER 3	QUARTER 4	TOTAL GRAMS
200	1,451.26	1,357.39	1,324.12	1,197.17	5,329.94
203	5.45	11.81	3.03	3.63	23.92
204	115.03	90.81	116.84	88.39	411.07
205	0	0.61	0.91	0	1.52
TOTAL	1,571.74	1,460.62	1,444.90	1,289.19	5,766.45

DATE RANGE: 01/01/2017 TO 12/31/2017 — ARCOS 3 - REPORT 1 — Run Date: 07/03/2018

RETAIL DRUG DISTRIBUTION BY ZIP CODE WITHIN STATE BY GRAMS WT

STATE: FLORIDA

ZIP CODE	QUARTER 1	QUARTER 2	QUARTER 3	QUARTER 4	TOTAL GRAMS
320	20,649.85	19,923.35	18,958.93	19,313.33	78,845.46
321	22,475.36	21,964.07	20,633.25	21,616.78	86,689.46
322	37,175.53	35,211.06	33,668.57	33,520.14	139,575.30
323	8,275.61	7,864.37	7,744.25	7,960.83	31,845.06
324	18,205.54	17,950.60	16,208.94	17,270.74	69,635.82
325	28,324.24	27,196.22	25,910.79	26,089.03	107,520.28
326	7,101.61	6,430.10	6,432.89	6,769.47	26,734.07
327	20,947.10	20,054.27	18,639.37	19,716.82	79,357.56
328	9,516.36	9,350.47	8,907.01	9,033.84	36,807.68
329	17,500.61	16,329.83	15,243.16	16,168.03	65,241.63
330	10,474.58	9,990.95	8,841.68	9,327.10	38,634.31
331	5,375.18	5,002.67	4,703.31	4,683.41	19,764.57
333	7,028.91	6,559.42	6,277.48	6,563.48	26,429.29
334	15,607.02	14,458.94	13,519.02	14,107.70	57,692.68
335	13,744.03	13,117.84	12,126.82	12,711.77	51,700.46
336	14,028.98	13,519.51	12,858.84	12,629.15	53,036.48
337	20,392.27	19,317.05	17,763.27	18,557.44	76,030.03
338	13,966.27	12,953.61	12,021.83	12,604.52	51,546.23
339	16,386.76	14,801.92	13,053.74	14,662.51	58,904.93
341	5,412.10	4,129.46	3,610.27	4,288.56	17,440.39
342	19,813.29	17,712.21	16,295.88	17,616.98	71,438.36
344	14,754.91	14,196.47	12,941.83	14,061.38	55,954.59
346	19,404.10	18,670.32	17,172.61	18,017.66	73,264.69
347	9,637.20	9,171.07	8,398.95	8,897.03	36,104.25
349	7,982.90	7,531.88	6,829.12	7,481.09	29,824.99
TOTAL	384,180.31	363,407.66	338,761.81	353,668.79	1,440,018.57

STATE: GEORGIA

ZIP CODE	QUARTER 1	QUARTER 2	QUARTER 3	QUARTER 4	TOTAL GRAMS
300	33,372.96	31,953.63	30,285.87	30,226.25	125,838.71
301	35,915.68	34,524.22	33,070.40	33,180.35	136,690.65
302	21,057.96	20,336.16	19,805.39	19,174.46	80,373.97

	303	7,499.78	6,922.72	6,516.58	6,277.04	27,216.12
	304	7,027.69	6,908.16	6,570.94	6,802.68	27,309.47
	305	21,785.66	21,315.45	20,436.70	20,165.67	83,703.48
	306	15,862.16	15,172.09	14,633.10	14,809.98	60,477.33
	307	13,468.66	12,984.96	12,499.64	12,162.39	51,115.65
	308	3,273.05	3,083.79	2,821.76	2,871.17	12,049.77
	309	6,591.07	5,977.77	5,764.36	5,711.26	24,044.46
	310	23,568.60	23,077.65	21,568.10	22,218.88	90,433.23
	312	8,552.01	8,103.40	7,841.63	7,927.50	32,424.54
	313	5,541.28	5,379.35	4,916.04	4,580.19	20,416.86
	314	5,352.73	5,268.78	4,916.03	4,921.57	20,459.11
	315	17,552.69	17,053.39	16,111.55	16,328.62	67,046.25
	316	9,177.81	9,014.62	7,948.59	8,513.34	34,654.36
	317	11,728.09	11,482.79	10,770.99	10,945.99	44,927.86
	318	1,293.74	1,203.97	1,179.40	1,230.60	4,907.71
	319	6,665.73	6,803.74	6,555.41	5,881.90	25,906.78
	398	3,751.39	3,579.35	3,354.59	3,376.14	14,061.47
TOTAL		259,038.74	250,145.99	237,567.07	237,305.98	984,057.78

STATE:		GUAM				
ZIP CODE		QUARTER 1	QUARTER 2	QUARTER 3	QUARTER 4	TOTAL GRAMS
	969	148.02	196.51	149.38	136.06	629.97
TOTAL		148.02	196.51	149.38	136.06	629.97

STATE:		HAWAII				
ZIP CODE		QUARTER 1	QUARTER 2	QUARTER 3	QUARTER 4	TOTAL GRAMS
	967	13,770.54	13,278.33	12,607.75	12,196.82	51,853.44
	968	4,842.77	4,451.68	4,491.23	4,246.40	18,032.08
TOTAL		18,613.31	17,730.01	17,098.98	16,443.22	69,885.52

STATE:		IDAHO				
ZIP CODE		QUARTER 1	QUARTER 2	QUARTER 3	QUARTER 4	TOTAL GRAMS
	832	5,794.76	5,738.47	5,484.93	5,535.61	22,553.77
	833	6,851.19	6,888.44	6,591.69	6,741.64	27,072.96

	834	4,773.54	4,808.36	4,700.02	4,677.92	18,959.84
	835	3,664.57	3,604.08	3,535.69	3,336.74	14,141.08
	836	16,783.40	16,530.03	16,022.10	16,100.89	65,436.42
	837	10,576.70	10,581.39	9,866.77	10,004.65	41,029.51
	838	10,102.26	9,718.67	9,611.32	9,433.21	38,865.46
TOTAL		58,546.42	57,869.44	55,812.52	55,830.66	228,059.04

STATE: ILLINOIS

ZIP CODE	QUARTER 1	QUARTER 2	QUARTER 3	QUARTER 4	TOTAL GRAMS
600	37,900.19	37,090.51	35,814.16	35,750.66	146,555.52
601	32,640.73	31,756.10	30,383.44	30,978.71	125,758.98
602	1,091.14	1,138.15	1,002.96	1,016.32	4,248.57
603	1,545.82	1,516.38	1,433.30	1,467.89	5,963.39
604	41,012.33	40,315.63	38,217.68	38,053.30	157,598.94
605	19,887.48	19,149.29	18,389.12	18,640.88	76,066.77
606	34,966.73	33,699.31	32,549.74	31,531.23	132,747.01
607	3,343.47	3,374.05	3,180.65	3,217.50	13,115.67
608	2,498.81	2,519.17	2,445.67	2,445.55	9,909.20
609	6,403.54	6,103.85	5,917.71	5,742.55	24,167.65
610	6,857.51	6,773.20	6,538.94	6,565.73	26,735.38
611	10,615.44	10,481.12	10,121.54	10,061.35	41,279.45
612	3,658.86	3,555.14	3,407.03	3,393.93	14,014.96
613	4,489.99	4,386.57	4,205.84	4,307.35	17,389.75
614	4,422.84	4,365.67	4,153.53	4,235.17	17,177.21
615	5,889.65	5,723.70	5,272.16	5,462.99	22,348.50
616	8,352.17	8,266.06	7,986.48	7,787.78	32,392.49
617	5,168.55	4,995.24	4,784.08	4,829.91	19,777.78
618	8,919.57	8,866.47	8,654.73	8,421.10	34,861.87
619	2,862.94	2,819.48	2,590.50	2,560.53	10,833.45
620	12,817.02	12,567.24	12,120.09	12,006.15	49,510.50
622	11,954.44	11,709.72	11,216.59	11,026.73	45,907.48
623	4,146.39	4,044.72	3,880.87	3,868	15,939.98
624	4,520.33	4,495.57	4,409.68	4,335.94	17,761.52
625	5,539.99	5,566.28	5,276.71	5,496.03	21,879.01
626	5,159.48	4,999.27	4,886.49	4,715.83	19,761.07
627	6,844.36	6,493.27	6,261.48	6,384.35	25,983.46

	628	8,409.17	8,115.44	7,957.85	7,739.87	32,222.33
	629	18,127.12	17,885.69	17,045.21	16,228.41	69,286.43
TOTAL		320,046.06	312,772.29	300,104.23	298,271.74	1,231,194.32
STATE:		INDIANA				
ZIP CODE		QUARTER 1	QUARTER 2	QUARTER 3	QUARTER 4	TOTAL GRAMS
	460	14,741.47	14,288.14	13,669.87	13,418.05	56,117.53
	461	19,487.03	19,279.83	18,019.29	17,680.28	74,466.43
	462	32,380.04	31,590.99	30,817.06	30,984.58	125,772.67
	463	29,135.87	29,122.72	27,474.38	26,640.69	112,373.66
	464	3,989.07	3,831.97	3,355	3,058.47	14,234.51
	465	10,926.15	10,941.04	9,979.94	9,886.14	41,733.27
	466	3,825.44	3,848.01	3,471.22	3,583.51	14,728.18
	467	6,369.07	6,240.12	5,934.47	6,070.80	24,614.46
	468	8,607.72	8,269.67	7,984.43	7,694.70	32,556.52
	469	13,637.14	13,260.08	12,488.09	12,351.38	51,736.69
	470	3,630.98	3,587.91	3,353.24	3,233.05	13,805.18
	471	16,539.84	16,635.78	15,874.03	15,053.93	64,103.58
	472	7,807.75	7,664.63	6,984.50	6,986.28	29,443.16
	473	14,765.83	14,117.28	13,368.52	13,061.62	55,313.25
	474	10,310.07	9,878.60	9,199.79	8,975.35	38,363.81
	475	7,585.39	7,254.21	6,822.03	6,875.70	28,537.33
	476	5,214.38	5,081.09	4,778.01	4,771.17	19,844.65
	477	11,983.78	12,208.34	11,343.88	11,407.45	46,943.45
	478	5,860.06	5,639.99	5,058.17	4,879.20	21,437.42
	479	10,108.21	10,022.30	9,401.42	9,250.13	38,782.06
TOTAL		236,905.29	232,762.70	219,377.34	215,862.48	904,907.81
STATE:		IOWA				
ZIP CODE		QUARTER 1	QUARTER 2	QUARTER 3	QUARTER 4	TOTAL GRAMS
	500	4,421.90	4,405.68	4,207.71	4,251.43	17,286.72
	501	3,835.93	3,897.94	3,810.33	3,665.70	15,209.90
	502	4,061.78	3,957.64	3,860.62	3,874.95	15,754.99
	503	9,720.81	9,465.81	9,148.95	9,000.05	37,335.62
	504	2,108.74	2,125.06	2,026.28	1,857.80	8,117.88

	505	2,104.53	2,058.34	1,983.74	2,036.09	8,182.70
	506	1,815.34	1,738.03	1,747.81	1,741.15	7,042.33
	507	1,654.54	1,627.45	1,552.90	1,508.94	6,343.83
	508	692.07	726.99	694.95	695.72	2,809.73
	510	1,042.50	998.9	966.67	994.2	4,002.27
	511	2,109.05	2,173.38	2,109.24	2,167.89	8,559.56
	512	552.12	441.08	468.97	474.17	1,936.34
	513	763.91	767.45	751.97	761.68	3,045.01
	514	809.59	796	764.55	740.23	3,110.37
	515	2,918.09	2,845.89	2,562.34	2,476.23	10,802.55
	516	527.63	520.77	522.31	484.49	2,055.20
	520	1,961.07	1,862.74	1,764.64	1,814.34	7,402.79
	521	597.89	566.42	578.79	547.21	2,290.31
	522	2,842.39	2,714.45	2,616.95	2,441.26	10,615.05
	523	1,589.74	1,628.95	1,458.16	1,432.55	6,109.40
	524	2,637.83	2,606.57	2,537.04	2,531.94	10,313.38
	525	3,141.74	3,224.14	3,116.46	3,040.20	12,522.54
	526	2,325.70	2,372.13	2,247.64	2,230.69	9,176.16
	527	2,286.38	2,211.73	2,100.33	2,074.54	8,672.98
	528	2,873.26	2,843.03	2,602.25	2,661.18	10,979.72
TOTAL		59,394.53	58,576.57	56,201.60	55,504.63	229,677.33

STATE:	KANSAS					
ZIP CODE		QUARTER 1	QUARTER 2	QUARTER 3	QUARTER 4	TOTAL GRAMS
	660	11,096.83	10,743.54	10,412.69	10,268.35	42,521.41
	661	3,494.39	3,351.26	3,285.21	3,156.38	13,287.24
	662	8,409.98	8,122.74	7,900	7,991.65	32,424.37
	664	1,430.21	1,365.60	1,439.12	1,286.86	5,521.79
	665	1,901.50	1,968.64	1,871.79	1,859.29	7,601.22
	666	4,935.94	4,720.32	4,442.43	4,352.97	18,451.66
	667	5,171.84	5,081.44	4,887.79	4,797.65	19,938.72
	668	1,328.37	1,359.96	1,245.13	1,244.90	5,178.36
	669	700.06	667.71	633.55	629.77	2,631.09
	670	6,503.79	6,496.69	6,223.97	6,140.79	25,365.24
	671	3,597.32	3,591.19	3,476.27	3,434.29	14,099.07
	672	18,991.36	18,545.74	18,073.25	17,967.34	73,577.69

	673	2,715.13	2,784.40	2,562	2,615.68	10,677.21
	674	4,613.78	4,506.69	4,340.62	4,327.27	17,788.36
	675	3,760.94	3,550.57	3,456.73	3,541.71	14,309.95
	676	1,447.21	1,512.72	1,345.13	1,402.15	5,707.21
	677	700.27	666.03	631.47	683.14	2,680.91
	678	2,360.49	2,311.09	2,181.08	2,235.21	9,087.87
	679	1,000	896.16	978.85	946.76	3,821.77
TOTAL		84,159.41	82,242.49	79,387.08	78,882.16	324,671.14

STATE: KENTUCKY

ZIP CODE		QUARTER 1	QUARTER 2	QUARTER 3	QUARTER 4	TOTAL GRAMS
	400	8,351.37	8,031.57	7,762.56	7,559.08	31,704.58
	401	4,644.27	4,779.75	4,378.23	4,271.67	18,073.92
	402	37,502.32	36,424.01	34,345.92	34,259.33	142,531.58
	403	11,585.29	11,370.04	10,884.07	10,972.03	44,811.43
	404	7,413.70	7,284.19	7,076.17	7,048.29	28,822.35
	405	8,217.53	7,710.55	7,616.50	7,404	30,948.58
	406	2,062.41	1,982.94	2,036.58	1,904.04	7,985.97
	407	6,449.63	6,334.40	5,988.20	6,177.44	24,949.67
	408	1,834.91	1,811.53	1,689.23	1,738.27	7,073.94
	409	8,473.60	7,874.82	7,768.18	7,638.70	31,755.30
	410	10,595.25	10,335.12	9,739.31	9,753.40	40,423.08
	411	4,028.28	3,991.80	3,704.90	3,684.80	15,409.78
	412	3,019.61	3,139.34	3,059.16	3,007.91	12,226.02
	413	3,008.07	2,970.24	2,860.47	2,873.75	11,712.53
	414	1,335.07	1,345.17	1,233.64	1,256.67	5,170.55
	415	6,987.40	6,905.67	6,622.04	6,168.27	26,683.38
	416	4,684.41	4,577.86	4,357.54	4,246.01	17,865.82
	417	4,922.05	4,967.18	4,674.57	4,477.64	19,041.44
	418	2,077.49	2,100.97	2,014.84	2,009.66	8,202.96
	420	14,948.36	14,958.03	13,706.43	13,900.14	57,512.96
	421	13,270.72	12,758.67	12,378.97	12,548.66	50,957.02
	422	6,643.05	6,481.36	6,570.17	6,406.05	26,100.63
	423	10,112.97	9,779.19	9,399.33	9,324.34	38,615.83
	424	10,233.91	10,182.88	9,485.36	9,577.46	39,479.61
	425	2,953.44	2,896.86	2,476.29	2,805.82	11,132.41

	426	3,840.56	3,702.96	3,707.56	3,539.19	14,790.27
	427	9,252.66	8,899.87	8,827.36	8,607.30	35,587.19
TOTAL		208,448.33	203,596.97	194,363.58	193,159.92	799,568.80

STATE:	LOUISIANA					
ZIP CODE		QUARTER 1	QUARTER 2	QUARTER 3	QUARTER 4	TOTAL GRAMS
	700	18,544.42	17,428.49	16,264.09	15,448.49	67,685.49
	701	8,214.66	7,521.16	7,066.42	6,650.72	29,452.96
	703	11,962.20	11,223.48	10,747.81	10,291.33	44,224.82
	704	17,373.97	16,943.32	16,151.73	15,345.52	65,814.54
	705	28,543.57	28,223.46	27,074.87	27,011.58	110,853.48
	706	8,030.29	7,709.25	7,356.24	7,518.69	30,614.47
	707	14,989.79	14,436.07	13,598.75	13,478.08	56,502.69
	708	12,826.86	12,223.38	11,375.20	11,188.28	47,613.72
	710	4,891.87	4,748.42	4,396.50	4,663.25	18,700.04
	711	15,459.03	14,668.60	14,472.57	13,784.04	58,384.24
	712	12,404.31	11,791.04	11,238.62	11,387.67	46,821.64
	713	14,310.70	13,794.98	13,096.53	12,980.05	54,182.26
	714	5,683.59	5,354.70	5,122.95	5,068.57	21,229.81
TOTAL		173,235.26	166,066.35	157,962.28	154,816.27	652,080.16

STATE:	MAINE					
ZIP CODE		QUARTER 1	QUARTER 2	QUARTER 3	QUARTER 4	TOTAL GRAMS
	039	206.01	176.16	187.7	168.98	738.85
	040	4,458.16	4,340.63	4,238.10	4,217.05	17,253.94
	041	933.08	896.07	944.24	888.95	3,662.34
	042	2,318.57	2,140.15	2,126.46	2,096.30	8,681.48
	043	2,056.34	1,949.49	1,947.26	1,918.33	7,871.42
	044	2,178.78	2,112.83	1,964.59	1,904.81	8,161.01
	045	535.5	547.48	546.81	527.09	2,156.88
	046	1,134.32	1,111.83	1,123.21	1,074.11	4,443.47
	047	1,159.03	1,216.75	1,057.06	1,102.86	4,535.70
	048	506.29	506.68	470.31	460.86	1,944.14
	049	2,290.38	2,298.41	2,233.27	2,188.52	9,010.58
TOTAL		17,776.46	17,296.48	16,839.01	16,547.86	68,459.81

STATE:	MARYLAND				
ZIP CODE	QUARTER 1	QUARTER 2	QUARTER 3	QUARTER 4	TOTAL GRAMS
206	2,830.68	2,704.79	2,622.43	2,612.61	10,770.51
207	3,448.50	3,176.04	2,965.91	2,952.57	12,543.02
208	2,770.76	2,660.79	2,412.13	2,362.52	10,206.20
209	631.64	621.91	522.38	573.83	2,349.76
210	6,249.38	5,771.44	5,505.30	5,337.94	22,864.06
211	3,172.10	2,951.50	2,735.13	2,711.01	11,569.74
212	6,899.30	6,584.51	6,203.10	6,085.62	25,772.53
214	765.41	666.17	656.45	645.81	2,733.84
215	2,738.43	2,606.34	2,319.51	2,222.01	9,886.29
216	1,544.72	1,416.56	1,381.62	1,395.26	5,738.16
217	4,185.26	3,841.69	3,667.83	3,772.03	15,466.81
218	1,594.96	1,462.40	1,418.40	1,364.71	5,840.47
219	737.95	667.33	622.05	699.28	2,726.61
TOTAL	37,569.09	35,131.47	33,032.24	32,735.20	138,468

STATE:	MASSACHUSETTS				
ZIP CODE	QUARTER 1	QUARTER 2	QUARTER 3	QUARTER 4	TOTAL GRAMS
10	2,376.37	2,275.55	2,167.47	2,133.28	8,952.67
11	511.22	492.76	434.9	445.18	1,884.06
12	662.01	618.1	609.19	620.36	2,509.66
13	347.1	376.6	326.93	360.96	1,411.59
14	1,354.33	1,319.28	1,234.50	1,191.26	5,099.37
15	2,127.24	1,997.57	1,929.01	1,958.85	8,012.67
16	1,383.50	1,257.86	1,157.11	1,176.61	4,975.08
17	1,482.51	1,507.47	1,407.08	1,465.24	5,862.30
18	5,873.91	5,724.77	5,389.40	5,195.75	22,183.83
19	2,806.97	2,683.04	2,581.58	2,637.80	10,709.39
20	1,503.05	1,422.37	1,289.39	1,368.31	5,583.12
21	3,459.66	3,343.40	3,142.23	3,169.78	13,115.07
22	76.03	79.76	66.31	47.05	269.15
23	2,340.12	2,222.81	2,089.71	2,015.21	8,667.85
24	839.19	816.24	734.61	804.55	3,194.59

	25	556.8	554.97	542.97	502.45	2,157.19
	26	918.48	927.7	867.04	880.35	3,593.57
	27	3,187.97	3,178.45	3,067.80	3,055.81	12,490.03
TOTAL		31,806.46	30,798.70	29,037.23	29,028.80	120,671.19

STATE: MICHIGAN

ZIP CODE		QUARTER 1	QUARTER 2	QUARTER 3	QUARTER 4	TOTAL GRAMS
	480	56,093.96	55,010.89	52,498.72	50,420.03	214,023.60
	481	70,871.60	70,217.16	66,229.98	63,235.94	270,554.68
	482	33,235.82	31,645.41	29,010.32	28,430.71	122,322.26
	483	36,008.97	35,133.24	33,460.12	32,189.87	136,792.20
	484	19,743.36	19,211.93	18,893.52	18,089.68	75,938.49
	485	12,003.08	12,363.10	11,638.44	11,343.53	47,348.15
	486	21,822.11	21,602.97	20,416.14	19,377.99	83,219.21
	487	11,617.51	11,394.02	10,735.93	10,247.15	43,994.61
	488	21,321.29	21,295.74	20,422.94	19,568.11	82,608.08
	489	8,698.19	8,413.71	8,152.08	7,730.22	32,994.20
	490	30,459.37	29,218.03	28,076.98	26,397.79	114,152.17
	491	2,807.12	2,730.15	2,538.53	2,547.45	10,623.25
	492	12,896.87	12,646.08	12,307.41	11,800.04	49,650.40
	493	9,066.96	9,007.97	8,627.32	8,229.54	34,931.79
	494	23,016.32	22,767.61	21,878.30	21,347.02	89,009.25
	495	16,837.35	17,116.10	16,181.81	14,984.45	65,119.71
	496	11,327.44	11,211.63	11,086.23	10,465.64	44,090.94
	497	9,557.11	9,606	9,212.32	8,647.78	37,023.21
	498	8,336.75	7,782.80	7,763.71	7,245.98	31,129.24
	499	2,543.92	2,448.25	2,424.95	2,241.64	9,658.76
TOTAL		418,265.10	410,822.79	391,555.75	374,540.56	1,595,184.20

STATE: MINNESOTA

ZIP CODE		QUARTER 1	QUARTER 2	QUARTER 3	QUARTER 4	TOTAL GRAMS
	550	4,915.67	4,615.60	4,551.39	4,351.63	18,434.29
	551	5,254.95	4,973.58	4,940.46	4,696.29	19,865.28
	553	6,475.11	6,150.15	6,069.36	6,018.10	24,712.72
	554	8,444.13	7,992.94	7,840.39	7,509.79	31,787.25

556	251.01	221.72	231.9	226.79	931.42
557	3,491.56	3,441.81	3,251.74	3,208.29	13,393.40
558	2,483.59	2,390.16	2,281.30	2,322.08	9,477.13
559	2,246.37	2,119.37	2,028.23	1,947.29	8,341.26
560	2,008.87	1,941.30	1,889.79	1,784.40	7,624.36
561	865.75	855.26	793.99	774.69	3,289.69
562	1,670.30	1,592.72	1,516.07	1,453.65	6,232.74
563	3,995.42	3,797.31	3,595.25	3,418.38	14,806.36
564	1,859.98	1,783.66	1,760.07	1,642.92	7,046.63
565	2,132.47	2,118.10	2,010.43	1,913.20	8,174.20
566	1,255.91	1,264.14	1,170.73	1,114.40	4,805.18
567	979.49	911.75	886.59	882.02	3,659.85
TOTAL	48,330.58	46,169.57	44,817.69	43,263.92	182,581.76

STATE: MISSISSIPPI

ZIP CODE	QUARTER 1	QUARTER 2	QUARTER 3	QUARTER 4	TOTAL GRAMS
386	11,128.94	10,809.02	9,995.51	9,770.03	41,703.50
387	3,432.66	3,411.10	3,091.30	2,760.28	12,695.34
388	14,433.56	13,391.42	12,642	12,325.72	52,792.70
389	3,841.81	3,740.99	3,386.15	3,131.03	14,099.98
390	8,641.47	8,134.69	7,386.96	6,835.79	30,998.91
391	9,096	8,653.34	7,673.54	7,226.58	32,649.46
392	9,105.27	8,483.68	7,915.20	7,208.25	32,712.40
393	12,567.11	12,128.84	10,904.71	10,418.54	46,019.20
394	21,722.44	20,899.51	20,000.89	18,756.32	81,379.16
395	20,822.15	20,209.44	18,998.61	18,445.80	78,476
396	5,615.41	5,652.33	5,197.67	4,866.39	21,331.80
397	5,929.40	5,665.97	5,168.65	4,924.72	21,688.74
TOTAL	126,336.22	121,180.33	112,361.19	106,669.45	466,547.19

STATE: MISSOURI

ZIP CODE	QUARTER 1	QUARTER 2	QUARTER 3	QUARTER 4	TOTAL GRAMS
630	17,359.94	17,091.28	16,032.73	16,129.65	66,613.60
631	23,122.31	22,945.94	21,832.41	21,109.06	89,009.72
633	10,168.16	9,962.32	9,369.39	9,555.07	39,054.94

	634	2,022.42	2,035.47	1,942.23	1,989.94	7,990.06
	635	1,812.09	1,922.58	1,694.75	1,761.15	7,190.57
	636	7,138.90	7,431.98	7,084.29	6,673.83	28,329
	637	5,630.49	5,486.28	5,165.56	4,836.75	21,119.08
	638	10,157.33	10,158.72	9,725.19	8,965.29	39,006.53
	639	7,011.09	6,895.69	6,161.85	5,724.65	25,793.28
	640	17,359.72	16,891.33	16,463.96	16,430.83	67,145.84
	641	13,832.45	13,278.93	12,565.66	12,268.28	51,945.32
	644	2,207.23	2,156.58	2,034.43	2,112.07	8,510.31
	645	4,603.96	4,285.94	4,102.60	3,989.40	16,981.90
	646	1,600.33	1,569.42	1,463.74	1,475.14	6,108.63
	647	4,762.61	4,649.25	4,295.49	4,226.62	17,933.97
	648	7,596.89	7,513.25	6,975.42	6,783.51	28,869.07
	650	3,384.67	3,421.32	3,200.86	3,236.03	13,242.88
	651	1,678.47	1,689.12	1,553.15	1,582.43	6,503.17
	652	4,938.45	4,837.03	4,480.79	4,612.69	18,868.96
	653	2,381.22	2,421.17	2,338.72	2,238.97	9,380.08
	654	2,301.24	2,259.96	2,217.35	2,088.36	8,866.91
	655	3,852.24	3,860.55	3,709.59	3,622.63	15,045.01
	656	7,484.38	7,564.45	7,225.30	6,885.87	29,160
	657	11,156.93	11,401.03	10,621.06	10,680.05	43,859.07
	658	10,826.02	10,451.66	10,212.45	9,930.92	41,421.05
TOTAL		184,389.54	182,181.25	172,468.97	168,909.19	707,948.95

STATE:		MONTANA				
ZIP CODE		QUARTER 1	QUARTER 2	QUARTER 3	QUARTER 4	TOTAL GRAMS
	590	1,181.70	1,099.71	1,172.37	1,179.63	4,633.41
	591	3,589.13	3,502.31	3,317.32	3,351.29	13,760.05
	592	562.04	584.45	529.13	526.54	2,202.16
	593	758.77	743.83	695.63	719.45	2,917.68
	594	3,308.42	3,122.36	3,062.37	2,915.30	12,408.45
	595	703.06	697.75	641.52	647.16	2,689.49
	596	3,073.86	3,132.19	2,993.37	2,881.94	12,081.36
	597	3,161.03	3,037.79	2,974.76	2,859.44	12,033.02
	598	6,310.87	6,240.01	5,979.36	5,898.65	24,428.89
	599	4,140.72	3,929.42	3,923.14	3,730.65	15,723.93

TOTAL	26,789.60	26,089.82	25,288.97	24,710.05	102,878.44

STATE: NEBRASKA

ZIP CODE	QUARTER 1	QUARTER 2	QUARTER 3	QUARTER 4	TOTAL GRAMS
680	3,665.17	3,578.74	3,546.07	3,564.78	14,354.76
681	12,042.97	11,373.09	10,902.91	10,700	45,018.97
683	1,459.05	1,422.41	1,310.90	1,318.59	5,510.95
684	1,002.09	959.5	990.46	935.55	3,887.60
685	5,259.74	4,996.51	5,027.32	5,075.85	20,359.42
686	871.8	859.36	798.69	835.49	3,365.34
687	1,833.90	1,855.08	1,873.67	1,870.44	7,433.09
688	3,066.76	3,000.04	2,872.62	2,922.26	11,861.68
689	1,202.89	1,162.31	1,184.53	1,197.57	4,747.30
690	478.57	371.25	429.54	404.01	1,683.37
691	1,609.40	1,550.54	1,551.54	1,479.17	6,190.65
692	199.97	161.94	201	185.3	748.21
693	1,294.52	1,192.78	1,183.43	1,139.34	4,810.07
TOTAL	33,986.83	32,483.55	31,872.68	31,628.35	129,971.41

STATE: NEVADA

ZIP CODE	QUARTER 1	QUARTER 2	QUARTER 3	QUARTER 4	TOTAL GRAMS
890	25,124.22	24,470.08	23,033.28	23,304.71	95,932.29
891	50,948.63	48,958.70	46,495.43	46,602.11	193,004.87
893	514.61	508.02	523.66	477.75	2,024.04
894	8,730.40	8,685.98	8,386.01	8,387.17	34,189.56
895	9,722.05	9,847.69	8,962.19	8,700.54	37,232.47
897	2,688.96	2,713.02	2,543.41	2,655.69	10,601.08
898	1,183.35	1,167.45	1,088.72	1,081.33	4,520.85
TOTAL	98,912.22	96,350.94	91,032.70	91,209.30	377,505.16

STATE: NEW HAMPSHIRE

ZIP CODE	QUARTER 1	QUARTER 2	QUARTER 3	QUARTER 4	TOTAL GRAMS
030	2,239.69	2,104.99	2,114.37	2,156.70	8,615.75
031	1,364.90	1,271.36	1,222.39	1,251.14	5,109.79

	032	1,159.60	1,132.46	1,088.21	1,054.79	4,435.06
	033	706.84	662.4	656.58	639.77	2,665.59
	034	569.62	539.67	536.01	511.19	2,156.49
	035	371.66	366.18	335.42	334.49	1,407.75
	036	36.62	39.53	32.66	34.01	142.82
	037	489.28	463.5	419.83	434.66	1,807.27
	038	2,553.48	2,452.86	2,347.01	2,429.49	9,782.84
TOTAL		9,491.69	9,032.95	8,752.48	8,846.24	36,123.36

STATE: NEW JERSEY

ZIP CODE		QUARTER 1	QUARTER 2	QUARTER 3	QUARTER 4	TOTAL GRAMS
	070	4,449.83	3,961.61	3,702.72	3,830.34	15,944.50
	071	413.45	345.12	347.26	325.54	1,431.37
	072	252.4	203.28	194.92	200.09	850.69
	073	381.35	396.23	345.55	333.87	1,457
	074	2,312.89	2,023.49	1,960.77	1,979.70	8,276.85
	075	294.46	233.11	276.55	235.25	1,039.37
	076	1,476.23	1,343.83	1,209.21	1,242.55	5,271.82
	077	3,344.62	2,954.21	2,846.86	2,859.34	12,005.03
	078	2,128.38	1,894	1,677.31	1,693.47	7,393.16
	079	878.58	717.29	653.73	667.9	2,917.50
	080	8,911.86	8,250.34	7,758.61	7,483.90	32,404.71
	081	639.79	569.03	566.3	566.05	2,341.17
	082	2,234.85	2,197.01	2,122.53	2,025.84	8,580.23
	083	1,954.15	1,759.69	1,734.80	1,717.60	7,166.24
	084	318.14	306.75	269.22	288.82	1,182.93
	085	1,085.19	922.5	841.25	902.97	3,751.91
	086	1,199.80	1,075.77	1,070.81	1,047.46	4,393.84
	087	2,717.88	2,436.13	2,393.83	2,316.40	9,864.24
	088	3,076.45	2,794.99	2,606.79	2,644.73	11,122.96
	089	211.22	186.04	179.3	155.93	732.49
TOTAL		38,281.52	34,570.42	32,758.32	32,517.75	138,128.01

STATE: NEW MEXICO

ZIP CODE		QUARTER 1	QUARTER 2	QUARTER 3	QUARTER 4	TOTAL GRAMS

	870	2,775.40	2,636.57	2,443.32	2,549.02	10,404.31
	871	13,189.75	12,717.66	11,699.75	11,499.31	49,106.47
	873	656.86	640.61	598.04	622.54	2,518.05
	874	2,018.86	1,998.34	1,693.14	1,713.07	7,423.41
	875	4,213.33	3,944.16	3,891.40	3,858.67	15,907.56
	877	966.78	926.92	915.13	896.48	3,705.31
	878	235.44	215.05	226.56	226.73	903.78
	879	272.4	254.75	250.39	226.81	1,004.35
	880	3,153.94	3,017.12	2,897.98	2,883.10	11,952.14
	881	1,299.96	1,311.60	1,363.67	1,258.96	5,234.19
	882	6,141.67	6,032.85	5,823.73	5,768.02	23,766.27
	883	1,657.80	1,651.92	1,605.11	1,571.27	6,486.10
	884	325.53	277.77	247.42	281	1,131.72
TOTAL		36,907.72	35,625.32	33,655.64	33,354.98	139,543.66

STATE:	NEW YORK				
ZIP CODE	QUARTER 1	QUARTER 2	QUARTER 3	QUARTER 4	TOTAL GRAMS
063	0.3	0	0.24	0	0.54
100	4,602.45	4,398.72	3,935.58	3,972.72	16,909.47
101	263.74	197.74	159.85	184.82	806.15
102	21.19	11.39	18.65	12.89	64.12
103	2,774.50	2,655.08	2,497.62	2,442.32	10,369.52
104	1,744.89	1,663.22	1,545.35	1,386.51	6,339.97
105	2,767.93	2,629.94	2,429.42	2,367.39	10,194.68
106	379.15	325.24	337.46	339.82	1,381.67
107	921.33	916.24	800.7	768.21	3,406.48
108	235.39	261.52	225.61	226.5	949.02
109	2,899.76	2,670.90	2,532.76	2,448.89	10,552.31
110	1,045.39	1,002.83	971.96	934.7	3,954.88
111	577.75	577.19	493.37	471.77	2,120.08
112	4,185.59	4,055.95	3,791.95	3,696.89	15,730.38
113	2,195.62	2,006.83	1,871.55	1,819.28	7,893.28
114	1,088.06	994.6	986.34	903.02	3,972.02
115	3,100.95	2,994.10	2,904.21	2,922.40	11,921.66
116	295.42	315.29	282.23	263.37	1,156.31
117	13,342.46	13,047.80	12,482.72	12,288.39	51,161.37
118	402.67	433.48	412.07	390.46	1,638.68

	119	2,146.24	2,170.31	2,043.74	1,975.69	8,335.98
	120	7,162.78	6,834.95	6,142.49	6,300.20	26,440.42
	121	3,956.37	3,591.10	3,530.21	3,439.05	14,516.73
	122	3,174.55	2,955.57	2,829.72	2,664.06	11,623.90
	123	3,015.35	2,769.36	2,706.46	2,638.91	11,130.08
	124	2,766.53	2,643.16	2,535.36	2,343.68	10,288.73
	125	4,710.48	4,616.56	4,392.65	4,272	17,991.69
	126	769.56	792.6	702.59	645.54	2,910.29
	127	1,078.50	1,043.09	1,019.36	950.26	4,091.21
	128	4,841.85	4,594.74	4,408.47	4,237.53	18,082.59
	129	2,944.77	2,889.35	2,807.53	2,677.56	11,319.21
	130	5,751.61	5,585.06	5,192.55	5,227.97	21,757.19
	131	2,001.43	1,951.90	1,846.33	1,861.38	7,661.04
	132	4,402.65	4,195.91	4,023.27	3,832.46	16,454.29
	133	2,778.51	2,581.27	2,303.23	2,396.23	10,059.24
	134	4,157.52	3,949.13	3,526.76	3,480.04	15,113.45
	135	1,928.08	1,894.33	1,803.48	1,730.43	7,356.32
	136	3,786.01	3,785.61	3,581.09	3,581.49	14,734.20
	137	1,760.60	1,759.51	1,630.18	1,611.32	6,761.61
	138	3,932.03	3,818.66	3,487.41	3,648.51	14,886.61
	139	1,668.56	1,618.68	1,531.62	1,574.25	6,393.11
	140	12,157.98	12,034.55	11,643.75	11,278.17	47,114.45
	141	6,097.82	5,982.77	5,827.30	5,527.15	23,435.04
	142	22,619.75	21,787.09	20,905.22	20,712.62	86,024.68
	143	2,792.38	2,766.89	2,707.92	2,717.62	10,984.81
	144	4,547.97	4,482.29	4,180.67	4,193.66	17,404.59
	145	3,636.45	3,608.06	3,444.64	3,320	14,009.15
	146	6,503.43	6,260.35	5,844.29	5,822.77	24,430.84
	147	4,859.18	4,762.09	4,528	4,384.84	18,534.11
	148	4,420.50	4,319.59	4,219.84	4,049.20	17,009.13
	149	961.59	987.21	923.21	900.56	3,772.57
TOTAL		180,175.57	174,189.80	164,948.98	161,835.50	681,149.85

STATE: NORTH CAROLINA

ZIP CODE		QUARTER 1	QUARTER 2	QUARTER 3	QUARTER 4	TOTAL GRAMS
	270	8,113.48	7,525.78	7,157.36	7,008.86	29,805.48

271	5,844.27	5,249.90	5,032.64	4,853.45	20,980.26
272	14,878.18	13,183.19	12,252.28	12,064.28	52,377.93
273	8,221.94	7,527.60	6,926.06	6,888.90	29,564.50
274	4,968.94	4,500.63	4,169.30	4,226.40	17,865.27
275	15,937.70	14,465.21	13,139.91	13,617.80	57,160.62
276	5,292.75	4,678.24	4,337.59	4,601.56	18,910.14
277	4,073.16	3,637.50	3,265.98	3,518.79	14,495.43
278	10,238.36	9,341.77	8,740.25	8,934.59	37,254.97
279	3,089.40	3,020.34	2,690.95	2,948.94	11,749.63
280	23,075.42	21,619.68	19,816.07	20,198.50	84,709.67
281	16,594.08	15,220.92	14,114.52	14,398.58	60,328.10
282	11,791.57	10,837.14	9,926.61	9,937.74	42,493.06
283	18,721.37	17,222.11	16,214.37	15,908.57	68,066.42
284	12,052.10	11,658.20	10,458.23	10,137.46	44,305.99
285	9,205.81	8,125.78	7,686.33	7,359.40	32,377.32
286	20,740.49	19,410.81	17,862.10	17,787.95	75,801.35
287	11,663.13	11,030.55	10,203.47	9,998.34	42,895.49
288	6,019.59	5,626.55	5,403.81	4,916.64	21,966.59
289	1,545.56	1,601.65	1,464.77	1,600.98	6,212.96
TOTAL	212,067.30	195,483.55	180,862.60	180,907.73	769,321.18

STATE: NORTH DAKOTA

ZIP CODE	QUARTER 1	QUARTER 2	QUARTER 3	QUARTER 4	TOTAL GRAMS
580	613.56	592.52	540.42	548.02	2,294.52
581	2,044.75	1,962.19	1,797.88	1,777.13	7,581.95
582	1,277.49	1,252.97	1,192.96	1,119.43	4,842.85
583	703.28	696.49	640.14	665.95	2,705.86
584	608.45	584.08	588.07	583.31	2,363.91
585	3,630.17	3,394.41	3,260.32	3,248.84	13,533.74
586	648.87	676.99	637.85	656.27	2,619.98
587	803.25	775.49	755.55	724.79	3,059.08
588	626.17	625.87	635.74	603.67	2,491.45
TOTAL	10,955.99	10,561.01	10,048.93	9,927.41	41,493.34

STATE: OHIO

ZIP CODE	QUARTER 1	QUARTER 2	QUARTER 3	QUARTER 4	TOTAL GRAMS
430	10,540.25	10,058.94	9,443.27	9,293.30	39,335.76
431	7,740.24	7,595.81	7,065.10	6,835.41	29,236.56
432	18,045.52	16,908.09	15,977.54	15,472.46	66,403.61
433	2,995.62	2,965.66	2,802.68	2,796.33	11,560.29
434	1,901.17	1,850.88	1,783.38	1,764.44	7,299.87
435	4,049.52	3,868.29	3,823.38	3,688.02	15,429.21
436	7,261.81	6,906.12	6,860.29	6,263.87	27,292.09
437	4,265.02	4,028.52	3,798.21	3,686.30	15,778.05
438	609.61	544.52	567.18	519.24	2,240.55
439	3,679.53	3,701.13	3,416.98	3,257.22	14,054.86
440	9,514.25	8,963.96	8,404.20	8,065.64	34,948.05
441	11,171.03	10,340.80	9,810.41	9,622.33	40,944.57
442	7,155.18	6,821.73	6,472.89	6,033.04	26,482.84
443	3,660.03	3,336.98	3,135.52	3,009.23	13,141.76
444	8,435.62	7,954.89	7,533.53	7,433.39	31,357.43
445	5,249.71	5,056.93	4,672.15	4,559.66	19,538.45
446	6,602.58	6,267.09	6,035.66	5,642.54	24,547.87
447	3,624.92	3,394.51	3,306.69	3,223.84	13,549.96
448	3,851.20	3,689.88	3,470.94	3,312.52	14,324.54
449	997.76	952.58	879.92	817.45	3,647.71
450	16,618.14	15,691.92	14,933.69	14,752.45	61,996.20
451	5,547.45	5,215.36	5,083.49	4,959.92	20,806.22
452	12,979.39	12,418.57	11,880.80	11,131.72	48,410.48
453	9,825.97	9,576.55	9,224.29	9,106.67	37,733.48
454	12,025.73	11,475.35	11,156.42	10,525.59	45,183.09
455	2,686.23	2,601.19	2,536.86	2,471.02	10,295.30
456	9,509.21	9,020.01	8,434.53	8,185.50	35,149.25
457	3,652.44	3,528.98	3,240.88	3,044.10	13,466.40
458	4,930.23	4,748.81	4,541.08	4,526.26	18,746.38
TOTAL	199,125.36	189,484.05	180,291.96	173,999.46	742,900.83

STATE: OKLAHOMA

ZIP CODE	QUARTER 1	QUARTER 2	QUARTER 3	QUARTER 4	TOTAL GRAMS
730	26,792.76	26,400.88	25,420.03	25,429.27	104,042.94
731	37,140.25	36,797.98	34,804.11	35,133.97	143,876.31

	734	5,673.16	5,821.18	5,303.51	5,243.62	22,041.47
	735	10,253.59	10,174.68	9,945.99	9,613.53	39,987.79
	736	1,869.62	1,887.01	1,819.96	1,673.68	7,250.27
	737	4,319.66	4,351.66	4,067.29	3,948.55	16,687.16
	738	743.63	728.84	712.82	688.75	2,874.04
	739	387.22	389.58	375.19	379.36	1,531.35
	740	30,201.09	30,048.99	28,181.92	28,320.64	116,752.64
	741	21,209.69	20,890.74	20,157.82	19,930.73	82,188.98
	743	5,144.20	5,164.26	4,965.11	4,921.49	20,195.06
	744	15,462.39	15,234.24	15,024.80	14,339.73	60,061.16
	745	4,944.42	4,968.66	4,607.52	4,460.17	18,980.77
	746	1,958.32	2,017.70	1,957.90	2,011.27	7,945.19
	747	6,733.63	6,675.37	6,347.39	6,038.04	25,794.43
	748	9,433.24	8,901.25	8,792.22	8,426.12	35,552.83
	749	5,191.90	5,142.45	4,780.98	4,973.19	20,088.52
TOTAL		187,458.77	185,595.47	177,264.56	175,532.11	725,850.91

STATE: OREGON

ZIP CODE		QUARTER 1	QUARTER 2	QUARTER 3	QUARTER 4	TOTAL GRAMS
	970	15,081.98	14,377.68	13,768.78	13,861.38	57,089.82
	971	6,412.78	6,366.98	6,151.53	6,078.08	25,009.37
	972	18,410.92	17,624.12	17,016.35	16,556.51	69,607.90
	973	12,887.21	12,130.36	11,553.47	11,205.85	47,776.89
	974	19,535.40	19,037.15	18,196.47	17,797.57	74,566.59
	975	11,936.11	11,307.62	11,301.07	11,287.21	45,832.01
	976	2,879.89	2,702.76	2,742.63	2,473.81	10,799.09
	977	5,435.04	5,383.50	5,230.18	4,952.14	21,000.86
	978	4,691.58	4,442.60	4,337.52	4,169.58	17,641.28
	979	1,954.63	1,875.05	1,788.03	1,759.57	7,377.28
TOTAL		99,225.54	95,247.82	92,086.03	90,141.70	376,701.09

STATE: PENNSYLVANIA

ZIP CODE		QUARTER 1	QUARTER 2	QUARTER 3	QUARTER 4	TOTAL GRAMS
	150	8,993.72	8,826.88	8,804.69	8,160.14	34,785.43
	151	6,577.79	6,094.37	5,948.01	5,718.48	24,338.65

152	12,672.58	12,150.82	11,409.36	11,011.34	47,244.10
153	3,965.32	3,232.35	3,720.07	3,044.86	13,962.60
154	4,853.08	4,634.73	4,416.30	4,336.13	18,240.24
155	1,698.22	1,617.60	1,586.51	1,269.62	6,171.95
156	6,100.67	5,926.48	5,578.05	5,219.10	22,824.30
157	2,512.76	2,479.88	2,478.24	2,198.70	9,669.58
158	2,143.29	2,130.87	2,035.48	1,969.47	8,279.11
159	2,727.22	2,663.98	2,460.94	2,393.80	10,245.94
160	2,962.81	2,781.20	2,818.78	2,673.83	11,236.62
161	4,232.01	4,127.86	3,918.30	3,883.17	16,161.34
162	1,717.23	1,650.49	1,565.29	1,534.28	6,467.29
163	3,598.14	3,457.19	3,259.23	3,170.34	13,484.90
164	2,269.62	2,300.20	2,159.64	2,140.33	8,869.79
165	7,891.93	7,845.61	7,244.02	7,102.53	30,084.09
166	5,575.51	5,447.91	5,201.41	5,076.88	21,301.71
167	1,361.79	1,396.81	1,321.14	1,317.89	5,397.63
168	1,570.99	1,532.62	1,485.13	1,373.44	5,962.18
169	993.68	931.53	865.66	802.78	3,593.65
170	8,553.44	8,229.80	7,784.32	7,565.29	32,132.85
171	2,055.12	1,998.47	1,843.21	1,741.49	7,638.29
172	1,552.14	1,493.79	1,424.68	1,380.73	5,851.34
173	2,784.76	2,733.33	2,478.29	2,377.71	10,374.09
174	1,644.13	1,571.81	1,424.11	1,448.42	6,088.47
175	2,974.35	2,834.75	2,685.18	2,547.40	11,041.68
176	2,210.89	2,096.12	2,012.80	1,952.27	8,272.08
177	3,157.30	3,084.67	2,931.25	2,743.98	11,917.20
178	2,106.78	2,068.05	1,933.94	1,848.25	7,957.02
179	1,887.55	1,791.20	1,744.12	1,654.81	7,077.68
180	3,309.71	3,099.13	2,982.09	2,965.64	12,356.57
181	1,445.58	1,303.64	1,290.40	1,262.75	5,302.37
182	2,232.15	2,117.55	1,878.36	1,894.65	8,122.71
183	1,696.10	1,628.55	1,553.44	1,514.68	6,392.77
184	3,124.98	3,002.11	2,983.79	2,797.60	11,908.48
185	4,297.65	4,138.49	3,933.27	3,684.45	16,053.86
186	3,223.84	3,137.77	3,040.72	2,820.79	12,223.12
187	8,655.75	7,994.97	7,442.98	7,759.83	31,853.53
188	1,178.25	1,106.35	1,079.11	1,008.77	4,372.48

189	2,574.08	2,477.28	2,379.14	2,305.41	9,735.91
190	12,740.83	9,994.78	14,061.52	8,867.46	45,664.59
191	7,453.96	7,235.37	6,780.14	6,438.20	27,907.67
193	2,731.91	2,653.46	2,575.21	2,398.64	10,359.22
194	4,352.72	4,240.98	3,965.47	3,901.12	16,460.29
195	1,786.32	1,748.89	1,613.25	1,578.67	6,727.13
196	2,056.41	2,088.23	1,952.22	1,879.23	7,976.09
TOTAL	176,205.06	167,098.92	164,049.26	152,735.35	660,088.59

STATE: PUERTO RICO

ZIP CODE	QUARTER 1	QUARTER 2	QUARTER 3	QUARTER 4	TOTAL GRAMS
6	412.28	269.35	151.46	128.61	961.7
7	322.57	194.56	123.65	126.64	767.42
9	362.58	196.86	125	133.12	817.56
TOTAL	1,097.43	660.77	400.11	388.37	2,546.68

STATE: RHODE ISLAND

ZIP CODE	QUARTER 1	QUARTER 2	QUARTER 3	QUARTER 4	TOTAL GRAMS
28	6,660.98	6,195.48	5,926.90	5,882.04	24,665.40
29	4,538.74	4,307.82	4,081.98	3,893.71	16,822.25
TOTAL	11,199.72	10,503.30	10,008.88	9,775.75	41,487.65

STATE: SOUTH CAROLINA

ZIP CODE	QUARTER 1	QUARTER 2	QUARTER 3	QUARTER 4	TOTAL GRAMS
290	8,193.78	7,653.58	7,185.13	7,285.05	30,317.54
291	8,969.60	8,505.71	7,942.34	8,084.60	33,502.25
292	6,511.33	6,059.46	5,606.93	5,600.62	23,778.34
293	23,463.65	22,250.66	20,832.74	21,174	87,721.05
294	21,054.27	20,053.32	18,794.73	18,754.35	78,656.67
295	24,589.63	23,755.82	21,863.42	22,092.52	92,301.39
296	40,235.04	38,481.96	36,233.35	35,927.91	150,878.26
297	7,410.19	6,958.48	6,265.46	6,303.06	26,937.19
298	3,822.91	3,774.80	3,275.55	3,394.38	14,267.64
299	3,232.14	3,053.67	2,856.09	3,017.81	12,159.71

TOTAL		147,482.54	140,547.46	130,855.74	131,634.30	550,520.04

STATE:		SOUTH DAKOTA				
ZIP CODE		QUARTER 1	QUARTER 2	QUARTER 3	QUARTER 4	TOTAL GRAMS
	570	2,430.12	2,320.17	2,264.49	2,306.09	9,320.87
	571	5,237.68	5,021.36	4,739.96	4,679.75	19,678.75
	572	1,114.16	1,010.66	993.19	982.65	4,100.66
	573	1,314.37	1,243.36	1,212.07	1,207.87	4,977.67
	574	967.48	896.4	864.71	846.73	3,575.32
	575	1,157.83	1,088.54	1,074.26	966.87	4,287.50
	576	258.8	232.83	260.4	240.38	992.41
	577	3,320.41	3,104.26	3,009.74	2,839.42	12,273.83
TOTAL		15,800.85	14,917.58	14,418.82	14,069.76	59,207.01

STATE:		TENNESSEE				
ZIP CODE		QUARTER 1	QUARTER 2	QUARTER 3	QUARTER 4	TOTAL GRAMS
	370	30,618.73	29,775.60	28,006.25	27,636.95	116,037.53
	371	27,668.25	28,204.42	28,643.27	27,950.13	112,466.07
	372	16,863.90	14,714.70	12,338.70	11,961.62	55,878.92
	373	28,201.72	27,574.46	24,997.05	25,809.77	106,583
	374	9,884	10,152.62	9,455.03	9,884.62	39,376.27
	376	18,935.99	18,000.37	17,055.08	16,630.09	70,621.53
	377	14,294.32	14,084.50	13,447.26	13,069.84	54,895.92
	378	22,561.43	22,310.21	21,607.34	21,061.91	87,540.89
	379	11,457.40	10,989.35	10,420.91	10,401.05	43,268.71
	380	13,816.38	12,931.84	12,808.76	12,876.34	52,433.32
	381	19,631.05	17,405.53	21,421.25	14,351.03	72,808.86
	382	8,038.66	7,966.36	7,581.36	7,632.53	31,218.91
	383	16,439.02	15,952.75	15,361.70	15,419.93	63,173.40
	384	9,163.44	8,710.18	8,222.89	8,309.60	34,406.11
	385	8,358.12	8,355.28	7,720.53	7,704.67	32,138.60
TOTAL		255,932.41	247,128.17	239,087.38	230,700.08	972,848.04

RETAIL DRUG DISTRIBUTION BY ZIP CODE WITHIN STATE BY GRAMS WT

STATE: TEXAS

ZIP CODE	QUARTER 1	QUARTER 2	QUARTER 3	QUARTER 4	TOTAL GRAMS
750	55,559.04	53,461.62	51,433.46	51,043.94	211,498.06
751	23,398.39	22,971.93	22,310.22	22,003.90	90,684.44
752	29,700.30	28,932.92	27,784.43	27,135.94	113,553.59
753	294.75	289.4	312.66	276.46	1,173.27
754	16,735.83	15,424.19	14,385.50	14,374.01	60,919.53
755	5,656.96	5,434.55	5,039.93	4,778.96	20,910.40
756	11,123.32	10,865.18	10,990.50	10,858.23	43,837.23
757	14,938.15	15,216.44	14,562.42	14,382.62	59,099.63
758	3,001.44	3,004.71	2,789.04	2,817.67	11,612.86
759	9,045.73	9,015.40	8,954.77	8,693.12	35,709.02
760	40,087.65	40,453.55	38,679.82	39,162.24	158,383.26
761	34,689.57	34,880.40	33,092.22	33,624.08	136,286.27
762	14,841.44	14,941.23	14,409.58	14,562.55	58,754.80
763	7,366.78	7,131.29	6,798.11	7,035.85	28,332.03
764	4,747.24	4,549.29	4,539.76	4,609.94	18,446.23
765	14,944.78	14,569.67	13,809.71	13,071.77	56,395.93
766	3,707.41	3,702.66	3,719.16	3,842.88	14,972.11
767	10,534.61	10,455.72	9,836.01	9,717.57	40,543.91
768	3,547.11	3,578.98	3,469.46	3,420.06	14,015.61
769	4,143.08	4,123.64	3,855.62	3,915.78	16,038.12
770	58,043.34	57,885.07	54,654.30	54,025.33	224,608.04
773	31,417.52	31,112	29,859.82	30,380.15	122,769.49
774	19,302.19	18,929.47	18,441.93	18,862.49	75,536.08
775	34,649.20	34,991.46	33,727.20	33,523.62	136,891.48
776	9,765.42	9,130.25	9,217.40	8,931.46	37,044.53
777	4,484.55	4,245.83	4,327.35	4,199.30	17,257.03
778	7,211.62	6,934	6,690.85	6,198.54	27,035.01
779	7,989.75	7,714.04	7,092.70	7,005.66	29,802.15
780	9,816.92	9,792.97	9,520.18	9,578.09	38,708.16
781	11,248.39	10,792.30	10,831.59	10,542.35	43,414.63
782	46,421.96	45,652.80	45,341.01	44,797.30	182,213.07
783	6,659.44	6,494.46	6,156.31	5,855.88	25,166.09
784	12,496.64	11,868.47	11,839.36	11,116.91	47,321.38
785	8,681.35	8,513.34	8,202.97	8,041.77	33,439.43
786	23,913.36	23,513.81	22,633.62	21,927.10	91,987.89

	787	23,066.78	21,755.73	20,304.56	19,186.37	84,313.44
	788	3,054.37	2,973.87	2,823.40	2,765.13	11,616.77
	789	1,696.71	1,693.18	1,598.63	1,668.81	6,657.33
	790	4,562.67	4,500.21	4,142.85	4,271.19	17,476.92
	791	9,304.12	8,682.70	9,059.80	7,775.22	34,821.84
	792	955.4	974.73	898.91	862.4	3,691.44
	793	3,813.29	3,794.61	3,616.74	3,683.77	14,908.41
	794	11,123.90	10,874.54	10,959.75	11,040.32	43,998.51
	795	3,170.61	2,898.66	2,818.53	2,781.22	11,669.02
	796	6,104.75	5,947.16	5,887.28	5,731.21	23,670.40
	797	12,262.44	11,732.08	11,115.57	11,322.98	46,433.07
	798	371.58	367.61	309.84	326.07	1,375.10
	799	9,044.83	8,427.34	8,176.56	7,667.56	33,316.29
TOTAL		688,696.68	675,195.46	651,021.39	643,395.77	2,658,309.30

STATE: UTAH

ZIP CODE		QUARTER 1	QUARTER 2	QUARTER 3	QUARTER 4	TOTAL GRAMS
	840	22,819.99	22,058.92	21,419.53	21,190.29	87,488.73
	841	15,468.33	14,916.11	14,062.54	13,975.70	58,422.68
	843	2,830.70	2,851.76	2,758.98	2,678.62	11,120.06
	844	4,530.34	4,412.86	4,205.19	4,345.59	17,493.98
	845	1,952.37	1,790.57	1,668.05	1,657.57	7,068.56
	846	5,789.31	5,419	5,164.49	5,146.53	21,519.33
	847	7,995.91	7,858.36	7,611.09	7,298.83	30,764.19
TOTAL		61,386.95	59,307.58	56,889.87	56,293.13	233,877.53

STATE: VERMONT

ZIP CODE		QUARTER 1	QUARTER 2	QUARTER 3	QUARTER 4	TOTAL GRAMS
	50	344.84	410.72	324.77	331.41	1,411.74
	51	224.02	198.07	177.49	175.64	775.22
	52	262.84	247.66	247.37	250.54	1,008.41
	53	305.74	292.52	308.26	300.8	1,207.32
	54	2,049.66	1,961.26	1,831.75	1,803.31	7,645.98
	56	844.51	796.68	748.04	723.49	3,112.72

	57	520.63	422.41	387.6	406.58	1,737.22
	58	582.61	580.79	539.96	535.62	2,238.98
TOTAL		5,134.85	4,910.11	4,565.24	4,527.39	19,137.59

STATE: VIRGIN ISLANDS

ZIP CODE		QUARTER 1	QUARTER 2	QUARTER 3	QUARTER 4	TOTAL GRAMS
	8	363.62	340.97	223.69	230.32	1,158.60
TOTAL		363.62	340.97	223.69	230.32	1,158.60

STATE: VIRGINIA

ZIP CODE		QUARTER 1	QUARTER 2	QUARTER 3	QUARTER 4	TOTAL GRAMS
	201	4,850.46	4,297.86	3,964.53	4,081.77	17,194.62
	220	2,140.16	1,896.44	1,779.37	1,840.30	7,656.27
	221	1,906.61	1,657.60	1,514.64	1,714.83	6,793.68
	222	602.35	576.31	515.94	485.26	2,179.86
	223	1,073.81	941.49	881.68	930.24	3,827.22
	224	2,905.74	2,683.07	2,398.97	2,504.55	10,492.33
	225	1,233.24	1,118.62	1,074.48	1,071.05	4,497.39
	226	2,869.59	2,609.63	2,398.17	2,475.51	10,352.90
	227	612.77	506.28	479.1	502.58	2,100.73
	228	2,432.79	2,162.94	1,906.65	2,006.83	8,509.21
	229	2,723.80	2,502.07	2,308.28	2,223.40	9,757.55
	230	2,520.54	2,326.92	2,176.42	2,054.23	9,078.11
	231	4,633.65	4,072.08	3,490.16	3,736.86	15,932.75
	232	7,380.02	6,543.85	5,999.15	5,649.05	25,572.07
	233	3,188.37	2,856.29	2,646.58	2,634.84	11,326.08
	234	6,975.14	6,166.48	5,666.63	5,805.01	24,613.26
	235	2,703.16	2,260.74	2,082.24	2,175.82	9,221.96
	236	4,769.86	4,045.31	3,696.24	3,517.05	16,028.46
	237	2,505.85	2,338.27	1,983.13	1,913.75	8,741
	238	3,734.82	3,258.04	2,905.81	2,924.48	12,823.15
	239	1,352.48	1,216.65	1,109.39	1,107.46	4,785.98
	240	9,522.81	8,823.80	8,445.46	8,088.04	34,880.11
	241	9,661.55	8,921.62	8,259.94	8,145.30	34,988.41
	242	13,820.84	12,993.96	12,508.54	12,069.64	51,392.98

	243	5,182.88	4,580.11	4,393.09	4,283.30	18,439.38
	244	2,901.22	2,599.92	2,461.37	2,492	10,454.51
	245	7,882.63	7,176.87	6,603.81	6,664.62	28,327.93
	246	4,307.69	4,298.55	3,888.83	3,876.47	16,371.54
TOTAL		116,394.83	105,431.77	97,538.60	96,974.24	416,339.44

STATE: WASHINGTON

ZIP CODE	QUARTER 1	QUARTER 2	QUARTER 3	QUARTER 4	TOTAL GRAMS
980	17,936.93	16,886.77	16,150.02	16,047.80	67,021.52
981	8,673.97	8,417.28	7,918.72	7,699.30	32,709.27
982	15,792.98	15,200.57	14,717.36	14,331.62	60,042.53
983	13,054.89	13,026.25	12,406.13	12,083.97	50,571.24
984	7,138.38	6,857.09	6,558.59	6,329.88	26,883.94
985	8,845.69	8,814.84	8,393.77	8,083.82	34,138.12
986	12,980.03	12,771.64	12,537.87	12,051.48	50,341.02
988	4,890.39	4,752.45	4,413.51	4,410.95	18,467.30
989	5,983.87	5,988.35	5,654.84	5,290.25	22,917.31
990	3,457.29	3,520.34	3,453.82	3,506.08	13,937.53
991	4,907.60	4,557.63	4,451.80	4,328.86	18,245.89
992	19,988.26	19,132.18	19,022.28	17,753.64	75,896.36
993	11,578.19	11,278.27	10,564.97	10,334.79	43,756.22
994	2,237.12	2,257.53	1,976.21	2,069.30	8,540.16
TOTAL	137,465.59	133,461.19	128,219.89	124,321.74	523,468.41

STATE: WEST VIRGINIA

ZIP CODE	QUARTER 1	QUARTER 2	QUARTER 3	QUARTER 4	TOTAL GRAMS
247	2,309.02	2,243.84	1,981.78	1,877.48	8,412.12
248	1,510.49	1,441.75	1,321.21	1,236.17	5,509.62
249	1,250.47	1,132.21	1,109.02	1,097.97	4,589.67
250	2,748.54	2,656.92	2,500.23	2,421.36	10,327.05
251	1,883.82	1,812.45	1,857.16	1,821.77	7,375.20
252	1,723.42	1,515.67	1,466.02	1,454.95	6,160.06
253	6,034.27	5,811.22	5,638.47	5,459.56	22,943.52
254	2,393.80	2,369.11	2,263.02	2,186.82	9,212.75
255	5,507.91	5,216.76	5,124.46	4,980.14	20,829.27

	256	3,209.75	3,060.86	2,948.88	2,658.91	11,878.40
	257	4,887.81	4,680.82	4,183.72	3,828.56	17,580.91
	258	4,324.28	4,147.35	3,863.11	3,870.24	16,204.98
	259	1,566.52	1,393.94	1,240.80	1,262.90	5,464.16
	260	3,716.06	3,558.09	3,441.39	3,433.37	14,148.91
	261	3,990.86	3,918.05	3,701.03	3,518.80	15,128.74
	262	2,304.31	2,278.15	2,167.97	2,129.01	8,879.44
	263	5,091.60	5,102.25	5,001.51	4,643.26	19,838.62
	264	1,253.79	1,305.38	1,246.44	1,200.79	5,006.40
	265	4,447.62	4,320.03	4,043.32	3,833.96	16,644.93
	266	1,293.48	1,172.67	1,135.95	1,144.06	4,746.16
	267	868.65	823.05	762.72	771.09	3,225.51
	268	429.89	374.99	354.3	335.98	1,495.16
TOTAL		62,746.36	60,335.56	57,352.51	55,167.15	235,601.58

STATE: WISCONSIN

ZIP CODE		QUARTER 1	QUARTER 2	QUARTER 3	QUARTER 4	TOTAL GRAMS
	530	7,508.67	7,130.27	6,946.78	7,180.39	28,766.11
	531	10,812.57	10,119.37	9,726.25	10,005.97	40,664.16
	532	11,228.78	10,677.71	10,358.64	10,202.34	42,467.47
	534	2,694.32	2,408.72	2,419.73	2,416.92	9,939.69
	535	8,196.46	7,869.04	7,479.48	7,480.68	31,025.66
	537	4,083.30	3,760.12	3,566.23	3,532.01	14,941.66
	538	753.45	750.1	726.33	718.9	2,948.78
	539	4,072.41	3,920.30	3,720.80	3,796.51	15,510.02
	540	887.25	861.66	895.11	828.45	3,472.47
	541	2,639.90	2,533.42	2,516.37	2,460.22	10,149.91
	542	1,476.08	1,357.90	1,373.10	1,327	5,534.08
	543	2,848.41	2,628.79	2,614.77	2,587.07	10,679.04
	544	5,607.78	5,263.89	4,956.55	5,002.69	20,830.91
	545	1,527.70	1,436.53	1,540.24	1,325.96	5,830.43
	546	4,732.97	4,499.88	4,266.56	4,300.39	17,799.80
	547	3,238.96	2,973.83	2,786.85	2,791.75	11,791.39
	548	3,276.96	3,135.93	3,030.41	2,932.55	12,375.85
	549	6,952.63	6,650.10	6,606.64	6,596.08	26,805.45
TOTAL		82,538.60	77,977.56	75,530.84	75,485.88	311,532.88

STATE: WYOMING

ZIP CODE	QUARTER 1	QUARTER 2	QUARTER 3	QUARTER 4	TOTAL GRAMS
820	2,417.31	2,326.17	2,182.18	2,120.60	9,046.26
822	450.03	454.33	433.27	469.19	1,806.82
823	561.41	378.06	205.4	206.21	1,351.08
824	1,264.57	1,153.51	1,186.42	1,186.31	4,790.81
825	783.63	787.44	713.63	740.74	3,025.44
826	2,274.77	2,204.36	2,075.86	2,109.29	8,664.28
827	847.26	802.93	772.29	759.14	3,181.62
828	1,156.13	1,242.40	1,169.19	1,150.49	4,718.21
829	1,745.09	1,689.25	1,712.04	1,677.10	6,823.48
830	234.18	211.55	237.34	205.04	888.11
831	450.04	443.33	424.52	395.44	1,713.33
TOTAL	12,184.42	11,693.33	11,112.14	11,019.55	46,009.44

DRUG CODE: 9220L DRUG NAME: LEVORPHANOL

STATE: ALABAMA

ZIP CODE	QUARTER 1	QUARTER 2	QUARTER 3	QUARTER 4	TOTAL GRAMS
350	1.51	0.58	1.04	0.46	3.59
351	0	0.12	0.12	0.12	0.36
352	1.04	0.93	1.28	1.28	4.53
354	0.93	0.46	0.81	1.04	3.24
357	0.12	0	0	0	0.12
359	0.23	0.35	0.46	0.35	1.39
361	1.04	0.93	0.58	0.35	2.9
364	0.81	0.58	0.46	0.46	2.31
365	0.23	0.35	0.35	0.23	1.16
366	0	0	0.23	0.23	0.46
TOTAL	5.91	4.3	5.33	4.52	20.06

STATE: ARIZONA

ZIP CODE	QUARTER 1	QUARTER 2	QUARTER 3	QUARTER 4	TOTAL GRAMS
850	7.2	11.26	14.28	23.68	56.42
851	0.12	1.16	1.16	0.81	3.25
852	10.68	9.63	13.35	4.29	37.95
853	0.23	0	0.58	1.16	1.97
860	0.23	0	0	0	0.23
863	0	0	0	0.12	0.12
TOTAL	18.46	22.05	29.37	30.06	99.94

STATE: ARKANSAS

ZIP CODE	QUARTER 1	QUARTER 2	QUARTER 3	QUARTER 4	TOTAL GRAMS
716	0	0	0.35	0.12	0.47
719	2.79	1.74	0.58	0.58	5.69
720	0	0.23	0.58	0.12	0.93
727	0	1.39	0.46	0.12	1.97
TOTAL	2.79	3.36	1.97	0.94	9.06

STATE: CALIFORNIA

ZIP CODE	QUARTER 1	QUARTER 2	QUARTER 3	QUARTER 4	TOTAL GRAMS
900	3.02	3.37	3.25	2.32	11.96
902	2.32	2.44	3.13	2.09	9.98
904	14.74	19.85	19.97	23.33	77.89
905	0	0.12	0.12	1.16	1.4
907	1.04	1.04	0.7	1.28	4.06
908	0	0.12	0.46	0.12	0.7
910	0.46	0.12	0.35	0.23	1.16
911	0.7	0.35	0	0	1.05
912	0	0	0	0.12	0.12
913	3.25	2.79	1.86	4.64	12.54
914	0.35	0.35	0.46	0.23	1.39
915	0	0	0	0.35	0.35
917	43.76	38.77	38.31	21.71	142.55
919	1.28	1.04	0.93	0.7	3.95
920	3.02	2.09	3.25	6.04	14.4
921	2.21	1.39	0.58	0.7	4.88

	922	0.46	0.12	0.46	0.35	1.39
	923	2.32	1.28	1.51	1.86	6.97
	924	0.58	1.04	1.63	1.74	4.99
	925	0.12	0.81	1.63	1.63	4.19
	926	5.11	4.29	4.88	3.6	17.88
	927	0	0	0	0.23	0.23
	928	0	0.12	0.58	0.58	1.28
	930	0.12	1.39	0.23	0	1.74
	931	0.12	1.74	0.12	0	1.98
	932	0	0.7	0.7	0.35	1.75
	934	0.35	0	0.35	0	0.7
	935	0.7	1.28	0.81	0.58	3.37
	936	0.12	0.23	0.23	0.7	1.28
	937	0.23	1.16	0.35	0.46	2.2
	939	0.58	0.7	0.81	0.23	2.32
	940	0.93	0.7	0.46	1.28	3.37
	941	1.63	1.86	1.51	3.71	8.71
	943	0	0.12	0	0.23	0.35
	945	2.79	1.86	2.32	1.63	8.6
	949	0.81	0.23	0.12	0.23	1.39
	950	2.32	2.32	3.13	2.9	10.67
	951	0	0	0.46	0.7	1.16
	953	0.35	0.23	0.12	0.12	0.82
	954	1.04	1.63	1.04	1.51	5.22
	956	0.58	0.58	0.23	0.46	1.85
	957	0	0.12	0	0	0.12
	958	12.42	3.6	1.16	1.39	18.57
	959	0	0.23	0.7	0.23	1.16
TOTAL		109.83	102.18	98.91	91.72	402.64

STATE: COLORADO

ZIP CODE		QUARTER 1	QUARTER 2	QUARTER 3	QUARTER 4	TOTAL GRAMS
	800	0.46	0.7	1.63	1.74	4.53
	801	0.23	0.23	1.51	4.06	6.03
	802	2.44	8.01	6.96	8.94	26.35
	803	0	0.23	0	0	0.23

804	0	0.23	0	0	0.23
805	0.7	1.39	1.39	1.28	4.76
806	1.63	1.63	1.74	1.39	6.39
807	0	0	0.12	0.23	0.35
808	0.12	0	0.12	0	0.24
809	0.46	0.46	0.23	0.46	1.61
815	0	0	0.12	0	0.12
TOTAL	6.04	12.88	13.82	18.1	50.84
STATE:	CONNECTICUT				
ZIP CODE	QUARTER 1	QUARTER 2	QUARTER 3	QUARTER 4	TOTAL GRAMS
60	0	0.7	0.7	0.81	2.21
61	0.23	0.23	0.23	0.46	1.15
63	0.23	0	0	0	0.23
64	0.58	1.16	2.21	3.6	7.55
65	0.12	0.58	0.93	2.44	4.07
66	0.35	0.7	0.58	1.51	3.14
67	0.12	0	0	0	0.12
68	0	0	0	0.12	0.12
69	0	0.35	0.46	0.81	1.62
TOTAL	1.63	3.72	5.11	9.75	20.21
STATE:	DELAWARE				
ZIP CODE	QUARTER 1	QUARTER 2	QUARTER 3	QUARTER 4	TOTAL GRAMS
197	0.12	0	0	0	0.12
199	0.81	0	0.58	1.04	2.43
TOTAL	0.93	0	0.58	1.04	2.55
STATE:	FLORIDA				
ZIP CODE	QUARTER 1	QUARTER 2	QUARTER 3	QUARTER 4	TOTAL GRAMS
320	0	0.12	0	0	0.12
321	0	0.23	0.12	0	0.35
322	0	0	0.23	0.35	0.58
324	0	0.23	0.35	0.58	1.16

	325	0.46	0.81	0.93	0.81	3.01
	326	0.23	0	0.12	1.28	1.63
	327	21.01	29.95	27.98	31.34	110.28
	328	0.35	0	0	0	0.35
	330	0.23	0.35	0.23	0	0.81
	331	0	0	0	0.12	0.12
	333	0	0	0	0.23	0.23
	334	0	0	0	0.12	0.12
	335	0	0.23	0.7	1.39	2.32
	336	0	0.12	0	0.35	0.47
	337	0	0.7	0.12	0	0.82
	338	0.12	0.46	0	0	0.58
	339	0.23	0	0.46	0.46	1.15
	341	0.12	0.12	0	0	0.24
	342	3.48	2.55	2.9	2.55	11.48
	344	0	0	0.23	0	0.23
TOTAL		26.23	35.87	34.37	39.58	136.05

STATE: GEORGIA

ZIP CODE		QUARTER 1	QUARTER 2	QUARTER 3	QUARTER 4	TOTAL GRAMS
	300	0	0.23	0.58	0.7	1.51
	301	0.23	0.35	0.46	0.81	1.85
	303	0.46	0.81	0.93	1.51	3.71
	305	0	0.23	0.35	0	0.58
	306	0	0.23	0.46	0.23	0.92
	308	0	0	0.12	0.23	0.35
	309	0	0.23	0	0	0.23
	314	0	0.46	0	0	0.46
TOTAL		0.69	2.54	2.9	3.48	9.61

STATE: IDAHO

ZIP CODE		QUARTER 1	QUARTER 2	QUARTER 3	QUARTER 4	TOTAL GRAMS
	838	0	0	0.12	0.12	0.24
TOTAL		0	0	0.12	0.12	0.24

STATE: ILLINOIS

ZIP CODE	QUARTER 1	QUARTER 2	QUARTER 3	QUARTER 4	TOTAL GRAMS
600	0.7	0.46	1.39	1.16	3.71
601	2.32	5.11	3.6	4.64	15.67
604	0.81	0.93	1.28	1.28	4.3
605	0.93	1.28	1.86	1.74	5.81
606	0.81	0.81	1.63	2.55	5.8
607	0	0.46	0	0	0.46
609	0	0.35	0.23	0.58	1.16
613	0	0	0.12	0.12	0.24
618	0.35	0	0	0	0.35
622	1.86	2.67	3.95	2.79	11.27
627	0.58	0.81	0.58	0.7	2.67
628	0.35	0.23	0.23	0.23	1.04
629	0	0	0	0.23	0.23
TOTAL	8.71	13.11	14.87	16.02	52.71

STATE: INDIANA

ZIP CODE	QUARTER 1	QUARTER 2	QUARTER 3	QUARTER 4	TOTAL GRAMS
460	0.46	0.7	0.7	0.58	2.44
461	0.35	0	0	0	0.35
462	1.16	1.63	1.74	1.39	5.92
465	0.35	0.58	0	0	0.93
467	11.49	8.94	9.52	9.63	39.58
468	1.51	2.21	1.63	0.81	6.16
469	4.53	5.46	3.71	4.88	18.58
470	0	0	0	0.12	0.12
471	1.97	1.16	2.55	0	5.68
472	0.23	0.58	0.93	0.23	1.97
473	2.9	2.32	3.13	3.48	11.83
479	0.93	0.93	0.93	1.04	3.83
TOTAL	25.88	24.51	24.84	22.16	97.39

STATE: IOWA

ZIP CODE	QUARTER 1	QUARTER 2	QUARTER 3	QUARTER 4	TOTAL GRAMS
500	0	0	0.35	0.23	0.58
501	0.12	0	0	0	0.12
503	0.46	0.35	0.46	0.35	1.62
504	0	0	0.12	0	0.12
506	1.74	0.81	0.81	0.7	4.06
507	0.7	1.04	0.35	0	2.09
514	0	0	0	0.35	0.35
515	0.58	1.28	0.81	1.86	4.53
516	0	0.23	0.23	0.23	0.69
520	0.58	0	0	0	0.58
524	0.23	0.23	0.23	0	0.69
TOTAL	4.41	3.94	3.36	3.72	15.43

STATE: KANSAS

ZIP CODE	QUARTER 1	QUARTER 2	QUARTER 3	QUARTER 4	TOTAL GRAMS
660	3.13	3.13	3.13	3.02	12.41
662	8.13	6.85	3.71	4.18	22.87
665	0	0	0.35	0	0.35
677	0	0	0	0.23	0.23
TOTAL	11.26	9.98	7.19	7.43	35.86

STATE: KENTUCKY

ZIP CODE	QUARTER 1	QUARTER 2	QUARTER 3	QUARTER 4	TOTAL GRAMS
410	0	0	0.23	0.12	0.35
TOTAL	0	0	0.23	0.12	0.35

STATE: LOUISIANA

ZIP CODE	QUARTER 1	QUARTER 2	QUARTER 3	QUARTER 4	TOTAL GRAMS
700	0.58	0.12	0.12	0.23	1.05
701	0.35	0	0	0	0.35
703	0.58	1.16	0.93	0.12	2.79
704	0.23	0	0.23	0.35	0.81
705	14.63	17.53	18.69	17.99	68.84

	707	0.12	0.35	0.81	0.81	2.09
	708	0	0.23	0	0	0.23
	711	0	0.12	0	0	0.12
TOTAL		16.49	19.51	20.78	19.5	76.28

STATE: MARYLAND

ZIP CODE		QUARTER 1	QUARTER 2	QUARTER 3	QUARTER 4	TOTAL GRAMS
	206	0	0	0	0.58	0.58
	207	0	0	0	0.58	0.58
	210	0.81	1.97	1.16	1.63	5.57
	211	1.28	0.81	0.93	1.16	4.18
	212	0.7	2.32	0.93	2.21	6.16
	214	0	0.58	0.81	2.21	3.6
	215	0	0.12	0	0	0.12
	217	0.23	0	0	0	0.23
	218	0.12	0.23	0	0	0.35
TOTAL		3.14	6.03	3.83	8.37	21.37

STATE: MASSACHUSETTS

ZIP CODE		QUARTER 1	QUARTER 2	QUARTER 3	QUARTER 4	TOTAL GRAMS
	10	6.27	8.36	7.89	8.01	30.53
	11	3.02	1.74	2.67	1.74	9.17
	13	0.7	0.35	0.35	0.58	1.98
	14	0	0	0.12	0	0.12
	15	0.35	0	0	0	0.35
	16	0.12	0	0	0	0.12
	18	5.8	5.92	6.15	6.73	24.6
	20	0.35	0.35	0.46	0.35	1.51
	21	1.04	1.04	0.7	1.51	4.29
	23	3.02	1.86	1.63	1.16	7.67
	27	0.12	0	0.23	0	0.35
TOTAL		20.79	19.62	20.2	20.08	80.69

DATE RANGE: 01/01/2017 TO 12/31/2017 ARCOS 3 - REPORT 1 Run Date: 07/03/2018

RETAIL DRUG DISTRIBUTION BY ZIP CODE WITHIN STATE BY GRAMS WT

STATE: MICHIGAN

ZIP CODE	QUARTER 1	QUARTER 2	QUARTER 3	QUARTER 4	TOTAL GRAMS
480	0.12	0.23	0	0	0.35
483	0	0.12	0	0	0.12
486	0	0	0.12	0	0.12
496	0	0	0.23	0	0.23
497	0.23	0	0	0	0.23
TOTAL	0.35	0.35	0.35	0	1.05

STATE: MINNESOTA

ZIP CODE	QUARTER 1	QUARTER 2	QUARTER 3	QUARTER 4	TOTAL GRAMS
551	0.35	0.46	0.81	1.16	2.78
TOTAL	0.35	0.46	0.81	1.16	2.78

STATE: MISSISSIPPI

ZIP CODE	QUARTER 1	QUARTER 2	QUARTER 3	QUARTER 4	TOTAL GRAMS
394	0.23	0	0	0	0.23
TOTAL	0.23	0	0	0	0.23

STATE: MISSOURI

ZIP CODE	QUARTER 1	QUARTER 2	QUARTER 3	QUARTER 4	TOTAL GRAMS
630	0.35	0.23	1.74	0.46	2.78
631	11.49	15.55	21.13	23.22	71.39
633	1.51	2.67	2.09	2.09	8.36
636	0	0.23	0.35	0.12	0.7
637	0	0	0.58	0.7	1.28
640	0	0	0	0.35	0.35
641	0	0.46	0	0	0.46
657	0	0	0.35	0.35	0.7
658	0.46	0.46	0	0	0.92
TOTAL	13.81	19.6	26.24	27.29	86.94

STATE:	NEBRASKA				
ZIP CODE	QUARTER 1	QUARTER 2	QUARTER 3	QUARTER 4	TOTAL GRAMS
680	1.04	0.81	1.16	0.81	3.82
681	3.02	4.06	4.06	7.2	18.34
685	0	0.23	0.23	1.16	1.62
TOTAL	4.06	5.1	5.45	9.17	23.78

STATE:	NEVADA				
ZIP CODE	QUARTER 1	QUARTER 2	QUARTER 3	QUARTER 4	TOTAL GRAMS
890	0	0	0	0.12	0.12
891	2.55	2.32	4.29	2.67	11.83
894	0.35	0.23	0	0	0.58
895	0	0.35	0.58	0.7	1.63
TOTAL	2.9	2.9	4.87	3.49	14.16

STATE:	NEW HAMPSHIRE				
ZIP CODE	QUARTER 1	QUARTER 2	QUARTER 3	QUARTER 4	TOTAL GRAMS
30	0	0.12	0	0	0.12
38	1.16	1.74	1.63	0.7	5.23
TOTAL	1.16	1.86	1.63	0.7	5.35

STATE:	NEW JERSEY				
ZIP CODE	QUARTER 1	QUARTER 2	QUARTER 3	QUARTER 4	TOTAL GRAMS
70	311.67	399.55	405.7	496.82	1,613.74
74	0	0.12	0.12	0	0.24
77	0.46	0.7	0.46	0.35	1.97
78	0.46	0.46	0	0	0.92
79	0.23	0	0.12	0	0.35
80	0.46	0	0.35	0.23	1.04
82	0.23	0	0	0.46	0.69
85	0.23	0	0	0.12	0.35
86	0.12	0	0	0	0.12

	87	0	0	0.23	0.35	0.58
	88	0	0	0	0.23	0.23
TOTAL		313.86	400.83	406.98	498.56	1,620.23

STATE:	NEW YORK				
ZIP CODE	QUARTER 1	QUARTER 2	QUARTER 3	QUARTER 4	TOTAL GRAMS
100	1.04	1.39	1.63	0.81	4.87
106	0.23	0.35	0.35	0.7	1.63
110	0.35	0	0.35	0	0.7
111	0	0	0	0.23	0.23
112	0	0.12	0.12	0.12	0.36
113	0.35	0.23	0.12	0.35	1.05
114	0.12	0	0.35	0.23	0.7
115	1.63	1.39	1.86	1.63	6.51
117	8.71	15.32	11.26	11.03	46.32
118	0.58	0	0.46	4.88	5.92
119	0	0.23	0.46	0.93	1.62
120	1.16	1.28	1.28	1.74	5.46
121	2.55	2.09	2.55	2.32	9.51
122	2.9	3.48	2.21	2.21	10.8
123	0.23	0.12	0.35	0.23	0.93
125	0	0	0.12	0.35	0.47
128	0	0	0.7	1.04	1.74
129	0.12	0.23	0.58	0.46	1.39
130	0.58	0.58	0.58	0	1.74
132	0	0	0	0.7	0.7
133	0.46	0.12	0.46	0	1.04
134	1.16	0.58	1.16	1.51	4.41
135	0.23	0.12	0.46	0.23	1.04
136	0.23	0	0	0	0.23
137	0.7	1.04	1.16	3.37	6.27
138	1.97	1.63	4.64	4.64	12.88
139	1.51	2.79	3.37	4.64	12.31
140	0.58	0.93	1.63	1.39	4.53
141	0.46	0.46	0.46	0.93	2.31
142	3.13	2.9	1.97	1.39	9.39

145	0.23	0	0	0	0.23
146	0.58	0	0	0	0.58
148	1.39	1.28	0.35	0.23	3.25
TOTAL	33.18	38.66	40.99	48.29	161.12

STATE: NORTH CAROLINA

ZIP CODE	QUARTER 1	QUARTER 2	QUARTER 3	QUARTER 4	TOTAL GRAMS
270	1.04	0.35	0.7	0.7	2.79
271	0.46	1.04	1.04	1.39	3.93
272	0.35	0.23	0.35	1.63	2.56
273	0	0.35	0.46	0.58	1.39
274	0	0.12	0.12	0.23	0.47
275	15.67	8.59	9.75	10.22	44.23
276	0.93	1.97	2.44	1.39	6.73
277	0.23	1.16	0	0.12	1.51
278	5.69	5.57	6.38	5.57	23.21
279	0.12	0	0.12	0.12	0.36
280	1.97	2.21	2.32	4.29	10.79
281	1.04	1.51	3.37	1.74	7.66
282	2.9	2.09	2.09	0.58	7.66
283	10.33	12.3	24.96	60.59	108.18
284	2.67	2.55	2.09	1.51	8.82
285	4.99	5.92	4.88	6.04	21.83
286	1.63	1.97	3.48	4.64	11.72
287	0.35	0	0	0.23	0.58
288	0.46	0.58	0.12	0.12	1.28
TOTAL	50.83	48.51	64.67	101.69	265.7

STATE: OHIO

ZIP CODE	QUARTER 1	QUARTER 2	QUARTER 3	QUARTER 4	TOTAL GRAMS
430	0.58	0.46	0	0	1.04
432	7.2	12.54	10.45	7.66	37.85
439	0.12	0.35	0.58	0.46	1.51
440	0	0.46	0.12	0.35	0.93

	441	0	0.23	0	0	0.23
	444	0	0.35	0.35	0.58	1.28
	445	0	0.23	0.23	0	0.46
	450	0	0	0	0.12	0.12
	458	0.12	0	0	0	0.12
TOTAL		8.02	14.62	11.73	9.17	43.54

STATE: OREGON

ZIP CODE		QUARTER 1	QUARTER 2	QUARTER 3	QUARTER 4	TOTAL GRAMS
	970	0.35	0.46	0.35	0.35	1.51
	971	0	0	0.12	0.12	0.24
	972	0.12	0.12	0.23	0.35	0.82
	974	2.09	2.21	1.74	2.21	8.25
	977	0.12	0	0	0	0.12
	978	0.81	0.7	0.46	0.7	2.67
TOTAL		3.49	3.49	2.9	3.73	13.61

STATE: PENNSYLVANIA

ZIP CODE		QUARTER 1	QUARTER 2	QUARTER 3	QUARTER 4	TOTAL GRAMS
	150	0	0.35	0.12	0.23	0.7
	151	0	0.58	0	0	0.58
	152	0	0.23	0	0	0.23
	153	0.58	0.12	0	0	0.7
	154	0	0	0.23	0.12	0.35
	156	0	0	0	0.35	0.35
	159	0.12	0	0.12	0	0.24
	160	0	0	0.23	0	0.23
	161	0	1.28	1.39	1.39	4.06
	163	0.46	0	0	0	0.46
	164	0	0	0	0.12	0.12
	170	1.04	0.81	0.81	0.23	2.89
	177	0.35	0.7	0.7	0.58	2.33
	178	2.09	2.44	1.97	1.51	8.01
	179	0.23	0	0	0.12	0.35

180	0.35	0.35	0.35	0.23	1.28
183	0.35	0.81	1.16	0.7	3.02
186	0.12	0	0	0	0.12
187	0.23	0.93	1.74	1.16	4.06
188	0.23	0.23	0.23	0.23	0.92
189	0.7	0	0.46	0.81	1.97
190	1.86	4.06	2.55	1.86	10.33
191	1.74	2.21	1.86	2.21	8.02
193	0.12	0.12	0.35	0.81	1.4
194	0	0	0.7	0.23	0.93
TOTAL	10.57	15.22	14.97	12.89	53.65

STATE: SOUTH CAROLINA

ZIP CODE	QUARTER 1	QUARTER 2	QUARTER 3	QUARTER 4	TOTAL GRAMS
290	0	0.23	0.46	0.23	0.92
291	0	0.35	0	0.12	0.47
292	0.12	0.12	0.46	0.46	1.16
294	0.23	0.35	0.35	0.23	1.16
295	0.12	0	0.23	0.23	0.58
296	0.35	0.7	0.93	1.74	3.72
297	0	0.12	0.35	0.35	0.82
298	0	1.51	0.46	0.23	2.2
TOTAL	0.82	3.38	3.24	3.59	11.03

STATE: TENNESSEE

ZIP CODE	QUARTER 1	QUARTER 2	QUARTER 3	QUARTER 4	TOTAL GRAMS
378	0.12	0.12	0.23	0	0.47
381	0	0.12	0	0	0.12
383	0	0	0.12	0	0.12
TOTAL	0.12	0.24	0.35	0	0.71

STATE: TEXAS

ZIP CODE	QUARTER 1	QUARTER 2	QUARTER 3	QUARTER 4	TOTAL GRAMS
750	25.89	35.06	42.49	39.24	142.68

	751	0.58	0.58	2.55	2.55	6.26
	752	1.51	1.63	1.74	1.74	6.62
	754	0.58	0.35	0	0	0.93
	757	0.35	0.58	0.46	0.23	1.62
	760	4.41	5.46	1.97	4.64	16.48
	761	0.12	0.35	0.81	1.16	2.44
	762	0	0	2.32	3.02	5.34
	763	0	0	0.23	0.35	0.58
	764	0.35	0	0	0	0.35
	765	1.39	1.04	1.16	0.81	4.4
	769	0	0	0.35	0	0.35
	770	1.04	0.93	0.46	0.35	2.78
	773	0.23	0	0.23	0.12	0.58
	774	0.23	0	0.12	0.46	0.81
	775	0.23	0.23	0.23	0	0.69
	776	0.12	0.23	0.12	0.12	0.59
	778	0	0	0.46	0.23	0.69
	780	0	0.23	0	0	0.23
	781	0.46	0.12	0.12	0.12	0.82
	782	0	0.35	0.58	1.86	2.79
	783	0	0.12	0.35	0.46	0.93
	784	0	0	0.12	0.35	0.47
	786	0.58	0.93	0.7	0.35	2.56
	787	0.93	5.22	3.71	7.89	17.75
	791	0	0	0.12	0	0.12
	797	0	0.23	0	0	0.23
	799	0.23	0	0	0	0.23
TOTAL		39.23	53.64	61.4	66.05	220.32

STATE:	VERMONT				
ZIP CODE	QUARTER 1	QUARTER 2	QUARTER 3	QUARTER 4	TOTAL GRAMS
54	0.23	0.46	0.46	0.46	1.61
TOTAL	0.23	0.46	0.46	0.46	1.61

STATE: VIRGINIA

ZIP CODE	QUARTER 1	QUARTER 2	QUARTER 3	QUARTER 4	TOTAL GRAMS
201	0	0	0.12	0	0.12
220	0	0	0.23	1.16	1.39
221	0	0	0.12	1.04	1.16
222	0.12	0.58	1.16	4.18	6.04
223	0	0	0.93	2.44	3.37
224	0	0	0.23	1.16	1.39
229	0	0.12	0	0	0.12
230	0.35	0.12	0	0	0.47
231	1.04	0.46	0.46	0.46	2.42
232	0.81	0.35	0.35	0	1.51
233	0.12	0	0	0.58	0.7
234	0.93	1.63	2.79	0.93	6.28
236	1.28	0.81	0.93	1.04	4.06
237	0.12	0.93	1.39	1.97	4.41
238	0.7	0.58	2.21	1.04	4.53
243	0.35	0.23	0.23	0.23	1.04
TOTAL	5.82	5.81	11.15	16.23	39.01

STATE: WASHINGTON

ZIP CODE	QUARTER 1	QUARTER 2	QUARTER 3	QUARTER 4	TOTAL GRAMS
980	4.18	5.11	8.13	10.33	27.75
981	0.46	0.7	0.46	1.16	2.78
982	8.13	8.13	9.4	8.36	34.02
983	2.09	2.79	3.6	2.9	11.38
984	0.46	0.46	0.93	1.74	3.59
985	6.85	6.85	8.13	5.69	27.52
986	0.35	0.23	0.23	0.35	1.16
988	0.12	0.12	0	0.35	0.59
990	0	0	0	0.23	0.23
992	0	0	0	0.12	0.12
993	0	0.12	0	0	0.12
TOTAL	22.64	24.51	30.88	31.23	109.26

STATE: WISCONSIN

ZIP CODE	QUARTER 1	QUARTER 2	QUARTER 3	QUARTER 4	TOTAL GRAMS
530	0.23	0.23	0.81	0.23	1.5
532	0	0.12	0	0	0.12
535	0.81	1.74	0.35	1.28	4.18
537	0.35	0.81	0.93	1.04	3.13
539	0	0.12	1.63	1.74	3.49
544	0.35	0.7	1.28	0.7	3.03
545	0	0.12	0	0	0.12
TOTAL	1.74	3.84	5	4.99	15.57

DRUG CODE: 9230 DRUG NAME: MEPERIDINE (PETHIDINE)

STATE: ALABAMA

ZIP CODE	QUARTER 1	QUARTER 2	QUARTER 3	QUARTER 4	TOTAL GRAMS
350	480.98	646.94	574.75	568.33	2,271
351	372.78	318.21	323.54	226.05	1,240.58
352	802.98	776.11	690.93	629.16	2,899.18
354	620.94	476.06	468.65	435.64	2,001.29
355	225.17	200.03	220.49	134.97	780.66
356	525.06	500.48	518.67	572.12	2,116.33
357	284.33	270.69	254.04	222.23	1,031.29
358	620.73	537.17	470.39	441.52	2,069.81
359	448.17	463.51	363.89	313.02	1,588.59
360	327.9	266.24	330.89	339.12	1,264.15
361	293.15	277.18	191.51	253.5	1,015.34
362	434.44	392.61	392.07	489.89	1,709.01
363	514.05	419.54	407.6	518.85	1,860.04
364	306.11	238.05	233.34	234.22	1,011.72
365	705.26	533.9	479.33	427.8	2,146.29
366	548.74	608.96	542.79	844.13	2,544.62
367	177.24	138.35	106.32	174.63	596.54
368	311.02	305.83	286.51	316.14	1,219.50
369	10.89	22.44	7.19	8.72	49.24

TOTAL		8,009.94	7,392.30	6,862.90	7,150.04	29,415.18

STATE:	ALASKA					
ZIP CODE		QUARTER 1	QUARTER 2	QUARTER 3	QUARTER 4	TOTAL GRAMS
	995	519.74	468.43	373	423.98	1,785.15
	996	201.75	161.23	194.02	139	696
	997	111.12	70.16	73.42	67.98	322.68
	998	16.12	3.05	20.04	1.74	40.95
	999	1.42	1.31	1.96	1.53	6.22
TOTAL		850.15	704.18	662.44	634.23	2,851

STATE:	ARIZONA					
ZIP CODE		QUARTER 1	QUARTER 2	QUARTER 3	QUARTER 4	TOTAL GRAMS
	850	1,061.92	916.14	933.29	947.91	3,859.26
	851	226.48	117.76	60.02	107.85	512.11
	852	1,671.54	1,652.80	1,559.64	1,689.95	6,573.93
	853	720.51	648.18	563.64	769.32	2,701.65
	855	142.49	164.5	96.52	130.07	533.58
	856	181.27	222.67	175.72	152.51	732.17
	857	1,071.62	783.48	689.57	692.52	3,237.19
	859	13.73	20.7	14.16	14.16	62.75
	860	64.49	61.44	38.56	31.16	195.65
	863	89.98	108.94	86.28	92.71	377.91
	864	200.66	223.32	149.79	131.71	705.48
	865	6.1	1.31	6.54	0	13.95
TOTAL		5,450.79	4,921.24	4,373.73	4,759.87	19,505.63

STATE:	ARKANSAS					
ZIP CODE		QUARTER 1	QUARTER 2	QUARTER 3	QUARTER 4	TOTAL GRAMS
	716	627.22	632.88	497.19	390.87	2,148.16
	717	282.89	307.42	220.93	229.73	1,040.97
	718	75.69	68.7	70.48	45.97	260.84
	719	490.33	436.29	382.7	328.01	1,637.33
	720	1,392.66	1,157.31	1,108.48	1,008.30	4,666.75

	721	1,439.70	1,342.48	1,204.46	1,092.32	5,078.96
	722	1,086.65	812.43	992.2	752.43	3,643.71
	723	447.95	352.09	270.82	283.78	1,354.64
	724	600.25	637.26	492.72	508.67	2,238.90
	725	197.83	229.57	205.13	168.42	800.95
	726	194.13	200.01	216.24	118.31	728.69
	727	1,056.91	1,186.87	933.05	1,011.70	4,188.53
	728	187.92	194.56	208.4	113.84	704.72
	729	268.97	281.39	282.58	273.32	1,106.26
TOTAL		8,349.10	7,839.26	7,085.38	6,325.67	29,599.41

STATE: CALIFORNIA

ZIP CODE	QUARTER 1	QUARTER 2	QUARTER 3	QUARTER 4	TOTAL GRAMS
900	777.81	803.63	749.27	896.01	3,226.72
902	431.07	521.37	446.43	336.83	1,735.70
903	3.81	4.36	13.62	5.99	27.78
904	106.32	120.48	130.51	121.14	478.45
905	275.18	199.25	184.65	229.75	888.83
906	203.06	120.38	138.9	76.8	539.14
907	79.74	84.64	86.5	98.04	348.92
908	147.94	97.5	97.72	72.55	415.71
910	68.3	57.19	103.71	71.9	301.1
911	96.52	93.03	90.42	103.49	383.46
912	153.17	187.26	173.1	183.12	696.65
913	909.85	797.64	706.24	698.94	3,112.67
914	209.7	222.78	280.62	183.34	896.44
915	139.66	159.05	150.33	106.98	556.02
916	8.72	15.25	9.8	7.63	41.4
917	740.01	888.28	917.47	849.49	3,395.25
918	50.11	49.46	49.89	53.16	202.62
919	61.33	87.69	91.07	60.35	300.44
920	883.59	1,022.81	882.61	911.48	3,700.49
921	649.05	661.36	650.36	691.97	2,652.74
922	242.71	277.68	327.68	216.13	1,064.20
923	376.71	322.78	382.26	693.17	1,774.92
924	36.39	23.53	27.89	35.95	123.76

	925	767.36	844.81	568.11	561.14	2,741.42
	926	577.59	403.4	555.25	441.52	1,977.76
	927	261.45	208.72	295.22	268.2	1,033.59
	928	495.56	536.84	543.92	641.32	2,217.64
	930	231.27	190.64	177.13	155.89	754.93
	931	217.88	205.02	159.05	144.89	726.84
	932	380.19	278.92	317.44	211.34	1,187.89
	933	816.16	699.27	599.37	592.29	2,707.09
	934	258.07	272.34	202.95	284.98	1,018.34
	935	114.71	109.92	120.16	145.21	490
	936	158.74	101.79	98.41	143.14	502.08
	937	374.96	495.99	362.11	563.1	1,796.16
	939	169.18	165.69	171.58	195.22	701.67
	940	182.36	193.69	177.57	212.97	766.59
	941	540.77	304.81	487.6	545.23	1,878.41
	943	161.01	187.26	205.13	181.93	735.33
	944	20.26	5.66	14.92	19.06	59.9
	945	885.66	1,001.24	866.16	763.65	3,516.71
	946	17.32	11.22	27.02	17.97	73.53
	947	2.72	4.36	11.77	5.45	24.3
	948	0	10.89	0.22	0	11.11
	949	49.57	52.73	49.24	74.84	226.38
	950	225.94	138.13	150.77	92.27	607.11
	951	119.29	105.78	112.53	90.96	428.56
	952	128.98	122.23	98.81	51.53	401.55
	953	655.15	790.89	547.41	746.22	2,739.67
	954	239.12	166.78	217.11	183.45	806.46
	955	50.55	65.14	64.71	13.07	193.47
	956	494.79	650.57	534.01	583.25	2,262.62
	957	56.65	26.58	18.52	14.16	115.91
	958	308.73	265.15	259.27	327.47	1,160.62
	959	264.65	296.4	240.86	267.22	1,069.13
	960	84.3	76.56	167.11	105.23	433.2
	961	13.07	67.32	36.39	26.15	142.93
TOTAL		15,974.76	15,874.14	15,148.88	15,374.53	62,372.31

RETAIL DRUG DISTRIBUTION BY ZIP CODE WITHIN STATE BY GRAMS WT

STATE: COLORADO

ZIP CODE	QUARTER 1	QUARTER 2	QUARTER 3	QUARTER 4	TOTAL GRAMS
800	237.48	147.39	134.76	93.25	612.88
801	306.77	260.73	290.86	261.23	1,119.59
802	359.49	295.98	204.58	239.66	1,099.71
803	46.3	39.87	40.74	29.41	156.32
804	56.54	65.69	61.77	70.48	254.48
805	191.73	187.59	231.93	126.59	737.84
806	104.36	150.66	108.28	61.01	424.31
807	36.82	20.26	33.77	21.79	112.64
808	83.66	81.27	69.72	71.25	305.9
809	285.09	186.28	175.83	178.55	825.75
810	97.28	126.8	110.46	99.24	433.78
811	18.52	16.34	18.52	13.07	66.45
812	26.04	40.96	37.47	36.71	141.18
813	40.52	22.22	35.95	20.26	118.95
814	100	98.26	57.95	66.45	322.66
815	74.3	64.27	47.93	39.54	226.04
816	106.43	86.39	69.18	58.5	320.5
TOTAL	2,171.33	1,890.96	1,729.70	1,486.99	7,278.98

STATE: CONNECTICUT

ZIP CODE	QUARTER 1	QUARTER 2	QUARTER 3	QUARTER 4	TOTAL GRAMS
60	38.13	68.85	43.47	64.27	214.72
61	82.68	65.25	69.18	64.82	281.93
62	19.61	5.66	36.39	22.88	84.54
63	21.79	44.45	94.99	56.65	217.88
64	382.59	329.43	298.71	359.38	1,370.11
65	78.76	118.52	65.36	76.47	339.11
66	104.58	89.98	113.4	89.33	397.29
67	157.52	98.04	99.35	102.84	457.75
68	209.27	211.34	166.35	220.05	807.01
69	9.15	20.7	49.24	13.07	92.16
TOTAL	1,104.08	1,052.22	1,036.44	1,069.76	4,262.50

RETAIL DRUG DISTRIBUTION BY ZIP CODE WITHIN STATE BY GRAMS WT

STATE: DELAWARE

ZIP CODE	QUARTER 1	QUARTER 2	QUARTER 3	QUARTER 4	TOTAL GRAMS
197	78.44	74.4	86.5	58.83	298.17
198	42.05	26.36	25.82	14.6	108.83
199	127.89	173.76	103.49	58.61	463.75
TOTAL	248.38	274.52	215.81	132.04	870.75

STATE: DISTRICT OF COLUMBIA

ZIP CODE	QUARTER 1	QUARTER 2	QUARTER 3	QUARTER 4	TOTAL GRAMS
200	215.48	220.05	168.96	147.94	752.43
204	2.18	0	0	0	2.18
TOTAL	217.66	220.05	168.96	147.94	754.61

STATE: FLORIDA

ZIP CODE	QUARTER 1	QUARTER 2	QUARTER 3	QUARTER 4	TOTAL GRAMS
320	409.5	447.19	334.44	406.34	1,597.47
321	331.5	306.99	191.4	201.21	1,031.10
322	690.34	730.86	535.65	499.37	2,456.22
323	510.59	407.1	394.68	413.96	1,726.33
324	221.91	185.74	123.1	216.89	747.64
325	754.61	697.31	594.8	586.41	2,633.13
326	194.13	179.64	230.29	134.54	738.6
327	334	288.9	257.2	212.97	1,093.07
328	168.85	129.31	114.17	133.78	546.11
329	568.76	505.69	430.09	398.71	1,903.25
330	624.65	582.05	478.13	479.65	2,164.48
331	641.1	757.99	605.26	623.67	2,628.02
333	493.6	523.66	492.18	727.92	2,237.36
334	495.45	561.9	470.07	636.52	2,163.94
335	386.29	296.42	252.95	361.45	1,297.11
336	482.7	430.96	285.63	414.51	1,613.80
337	196.96	155.02	200.23	131.81	684.02
338	406.01	365.38	354.81	390	1,516.20
339	495.77	408.84	319.4	352.19	1,576.20
341	90.96	99.35	60.13	97.39	347.83

	342	234.76	256.44	263.74	193.8	948.74
	344	347.07	356.33	249.9	241.3	1,194.60
	346	359.93	326.92	167.44	291.84	1,146.13
	347	183.12	181.16	141.73	179.09	685.1
	349	329.32	329.86	261.01	217.88	1,138.07
TOTAL		9,951.88	9,511.01	7,808.43	8,543.20	35,814.52

STATE: GEORGIA

ZIP CODE		QUARTER 1	QUARTER 2	QUARTER 3	QUARTER 4	TOTAL GRAMS
	300	1,078.26	1,011.38	1,107.89	1,031.42	4,228.95
	301	1,124.67	1,060.18	982.73	906.36	4,073.94
	302	878.36	703.24	632.49	552.2	2,766.29
	303	753.63	477.26	552.42	585.1	2,368.41
	304	287.92	233.98	327.25	518.54	1,367.69
	305	679.27	633.69	525.51	501.66	2,340.13
	306	457.1	452.07	404.05	455.9	1,769.12
	307	785.35	705.92	635.65	602.32	2,729.24
	308	183.67	240.97	244.35	250.67	919.66
	309	543.27	489.35	421.37	491.74	1,945.73
	310	814.83	721.91	637.46	720.93	2,895.13
	312	339.56	239.66	269.08	191.29	1,039.59
	313	277.25	259.92	229.31	276.92	1,043.40
	314	287.6	299.03	314.83	361.13	1,262.59
	315	668.88	665.17	595.13	573.58	2,502.76
	316	889.56	869.1	803.85	708.77	3,271.28
	317	784.89	796.68	685.41	690.03	2,957.01
	318	53.38	70.05	47.93	66.45	237.81
	319	401.54	299.45	197.98	339.49	1,238.46
	398	209.62	214.59	100.22	109.63	634.06
TOTAL		11,498.61	10,443.60	9,714.91	9,934.13	41,591.25

STATE: GUAM

ZIP CODE		QUARTER 1	QUARTER 2	QUARTER 3	QUARTER 4	TOTAL GRAMS
	969	30.61	34.64	66.23	52.18	183.66
TOTAL		30.61	34.64	66.23	52.18	183.66

STATE: HAWAII

ZIP CODE	QUARTER 1	QUARTER 2	QUARTER 3	QUARTER 4	TOTAL GRAMS
967	239.77	223.98	157.52	146.52	767.79
968	142.49	170.38	148.37	128.22	589.46
TOTAL	382.26	394.36	305.89	274.74	1,357.25

STATE: IDAHO

ZIP CODE	QUARTER 1	QUARTER 2	QUARTER 3	QUARTER 4	TOTAL GRAMS
832	117.11	132.69	110.57	76.8	437.17
833	98.78	139.7	49.07	116.61	404.16
834	138.35	173.1	120.05	116.02	547.52
835	33.77	36.82	42.16	54.03	166.78
836	136.61	129.64	236.18	153.82	656.25
837	226.59	236.5	124.73	229.53	817.35
838	96.85	76.78	58	69.18	300.81
TOTAL	848.06	925.23	740.76	815.99	3,330.04

STATE: ILLINOIS

ZIP CODE	QUARTER 1	QUARTER 2	QUARTER 3	QUARTER 4	TOTAL GRAMS
600	217.55	243.15	212.75	186.17	859.62
601	317.33	505.47	529.98	306.66	1,659.44
602	7.84	0.44	15.25	16.34	39.87
603	0.44	1.09	15.25	1.09	17.87
604	286.72	259.49	344.35	267.99	1,158.55
605	192.38	175.28	202.51	107.63	677.8
606	169.4	197.83	163.95	92.71	623.89
607	1.53	4.36	0	9.15	15.04
608	44.45	44.01	61.44	35.3	185.2
609	0.22	2.29	1.09	5.45	9.05
610	100.44	130.29	90.85	145.76	467.34
611	34.42	69.5	74.51	65.91	244.34
612	7.41	15.03	29.85	17.97	70.26
613	48.59	66.89	55.34	56.21	227.03

	614	77.35	79.52	51.2	69.72	277.79
	615	58.06	55.12	27.56	55.99	196.73
	616	162.32	177.13	155.56	152.73	647.74
	617	39.44	43.4	19.5	32.46	134.8
	618	152.73	211.12	173.54	142.05	679.44
	619	63.95	47.17	56.54	57.74	225.4
	620	27.89	27.45	33.33	7.41	96.08
	622	337.38	278.01	312.11	215.26	1,142.76
	623	23.42	14.64	37.47	34.86	110.39
	624	16.67	11.16	24.51	30.94	83.28
	625	9.04	24.62	15.03	28.32	77.01
	626	167.48	149.24	178	88.24	582.96
	627	67	104.8	61.66	66.02	299.48
	628	131.6	87.72	66.56	58.61	344.49
	629	227.9	248.03	191.73	189.44	857.1
TOTAL		2,990.95	3,274.25	3,201.42	2,544.13	12,010.75

STATE: INDIANA

ZIP CODE		QUARTER 1	QUARTER 2	QUARTER 3	QUARTER 4	TOTAL GRAMS
	460	283.89	273.43	258.94	308.51	1,124.77
	461	326.16	508.08	367.56	309.16	1,510.96
	462	1,102.23	1,169.99	984.36	729.12	3,985.70
	463	279.42	231.71	269.29	161.66	942.08
	464	23.75	32.03	46.41	17.43	119.62
	465	41.94	41.18	35.19	70.81	189.12
	466	39.22	25.06	30.94	18.52	113.74
	467	105.89	46.84	52.73	46.63	252.09
	468	150.12	142.82	150.55	119.5	562.99
	469	90.74	96.24	146.67	91.29	424.94
	470	121.4	97.39	87.54	77.78	384.11
	471	227.64	165.78	160.01	183.23	736.66
	472	121.57	137.91	137.26	117.65	514.39
	473	140.36	155.78	132.9	109.81	538.85
	474	111.77	72.23	73.86	46.73	304.59
	475	35.62	46.56	53.38	36.6	172.16
	476	66.89	48.7	48.48	187.37	351.44

	477	130.07	71.03	74.84	221.47	497.41
	478	115.47	77.56	80.83	114.38	388.24
	479	75.6	84.64	68.96	82.57	311.77
TOTAL		3,589.75	3,524.96	3,260.70	3,050.22	13,425.63

STATE: IOWA

ZIP CODE		QUARTER 1	QUARTER 2	QUARTER 3	QUARTER 4	TOTAL GRAMS
	500	97.83	74.73	130.94	74.84	378.34
	501	25.93	27.67	34.21	26.15	113.96
	502	216.79	194.45	186.07	160.79	758.1
	503	376.92	395.66	414.62	432.92	1,620.12
	504	4.36	7.84	8.17	4.36	24.73
	505	73.97	76.69	71.46	110.9	333.02
	506	64.93	41.72	48.15	44.12	198.92
	507	282.8	305.9	298.92	378.88	1,266.50
	508	22.88	12.83	11.77	0.54	48.02
	510	12.64	13.51	15.03	27.45	68.63
	511	74.4	55.88	63.62	73.64	267.54
	512	5.66	4.14	6.32	5.45	21.57
	513	13.73	11.77	18.74	25.06	69.3
	514	12.64	11.11	13.07	9.59	46.41
	515	46.52	21.77	38.02	29.3	135.61
	516	5.45	5.45	2.18	0.87	13.95
	520	52.51	41.83	56.65	40.42	191.41
	521	0	0.44	3.7	0	4.14
	522	70.16	111.55	142.93	107.63	432.27
	523	29.63	40.57	31.81	50.55	152.56
	524	236.83	236.18	210.79	197.83	881.63
	525	106.76	104.19	88.57	97.39	396.91
	526	15.47	22.7	24.23	11.98	74.38
	527	42.16	33.77	44.99	23.97	144.89
	528	36.39	41.61	26.25	33.77	138.02
TOTAL		1,927.36	1,893.96	1,991.21	1,968.40	7,780.93

STATE: KANSAS

ZIP CODE	QUARTER 1	QUARTER 2	QUARTER 3	QUARTER 4	TOTAL GRAMS
660	200.77	304.92	220.38	304.48	1,030.55
661	72.33	47.93	58.17	50.66	229.09
662	209.81	171.84	177.68	242.39	801.72
664	47.04	77.02	20.94	28.3	173.3
665	51.85	34.42	45.32	41.4	172.99
666	74.51	63.18	56.65	16.78	211.12
667	78.87	61.24	75.71	53.6	269.42
668	51.75	52.18	46.84	60.68	211.45
669	8.72	11.33	16.56	16.45	53.06
670	157.2	114.8	118.09	92.92	483.01
671	41.51	37.47	66.34	31.59	176.91
672	334.55	306.64	320.43	306.55	1,268.17
673	69.94	63.18	44.66	44.66	222.44
674	89.98	85.67	75.17	79.63	330.45
675	170.16	133.38	167.22	136.72	607.48
676	66.23	70.31	73.1	51.42	261.06
677	16.56	11.33	22.44	31.16	81.49
678	47	87.56	51.85	53.6	240.01
679	71.9	58.43	54.69	62.31	247.33
TOTAL	1,860.68	1,792.83	1,712.24	1,705.30	7,071.05

STATE: KENTUCKY

ZIP CODE	QUARTER 1	QUARTER 2	QUARTER 3	QUARTER 4	TOTAL GRAMS
400	42.27	61.88	52.73	52.12	209
401	47.28	30.5	34.86	5.45	118.09
402	331.37	305.92	411.13	323.54	1,371.96
403	63.73	69.18	72.55	93.47	298.93
404	40.96	46.63	53.82	52.29	193.7
405	516.36	44.56	119.18	46.63	726.73
406	14.16	9.8	0.22	9.04	33.22
407	53.82	55.12	49.24	41.29	199.47
408	41.07	37.47	42.05	46.19	166.78
409	85.19	78.65	69.18	57.74	290.76
410	140.09	155.56	53.27	99.9	448.82
411	54.47	51.2	43.03	56.1	204.8

	412	1.09	0	1.09	0	2.18
	413	18.52	15.25	17.43	23.97	75.17
	414	0	0	0.44	0	0.44
	415	40.74	35.3	27.13	22.33	125.5
	416	4.9	6.75	23.64	13.07	48.36
	417	2.51	5.45	5.01	1.63	14.6
	418	45.75	36.49	50.11	47.39	179.74
	420	286.46	308.99	288.58	310.91	1,194.94
	421	374.92	246.42	311.02	300.12	1,232.48
	422	177.89	140.42	183.67	137.48	639.46
	423	123.32	158.66	148.16	114.71	544.85
	424	176.57	177.55	147.83	139.88	641.83
	425	40.74	39.44	56.32	36.28	172.78
	426	38.35	26.84	54.47	12.31	131.97
	427	117.98	155.93	153.38	83.66	510.95
TOTAL		2,880.51	2,299.96	2,469.54	2,127.50	9,777.51

STATE: LOUISIANA

ZIP CODE		QUARTER 1	QUARTER 2	QUARTER 3	QUARTER 4	TOTAL GRAMS
	700	983.05	821.93	804.83	634.78	3,244.59
	701	156.87	187.81	153.6	131.81	630.09
	703	400.32	407.6	307.42	352.3	1,467.64
	704	839.8	788.77	633.14	456.99	2,718.70
	705	1,609.88	1,586.28	1,483.27	1,522.92	6,202.35
	706	650.23	646.24	524.69	499.06	2,320.22
	707	454.27	399.8	363.31	393.81	1,611.19
	708	549.68	562.47	507.37	562.1	2,181.62
	710	244.02	207.09	233.45	265.26	949.82
	711	995.8	792.96	690.12	650.36	3,129.24
	712	530.96	476.58	424.81	593.93	2,026.28
	713	477.47	446.32	415.38	330.41	1,669.58
	714	218.53	246.55	173.43	229.42	867.93
TOTAL		8,110.88	7,570.40	6,714.82	6,623.15	29,019.25

STATE: MAINE

ZIP CODE	QUARTER 1	QUARTER 2	QUARTER 3	QUARTER 4	TOTAL GRAMS
39	13.51	11.11	6.75	11.11	42.48
40	95.65	115.91	87.26	73.21	372.03
41	19.61	3.92	25.82	15.25	64.6
42	11.33	18.08	15.47	10.24	55.12
43	9.59	10.46	19.83	4.36	44.24
44	146.85	96.52	99.79	85.41	428.57
45	9.15	0	1.31	0	10.46
46	63.84	42.05	55.78	37.47	199.14
47	66.13	66.02	21.68	77.35	231.18
48	0	4.36	4.36	0	8.72
49	29.74	40.96	21.02	29.96	121.68
TOTAL	465.4	409.39	359.07	344.36	1,578.22

STATE: MARYLAND

ZIP CODE	QUARTER 1	QUARTER 2	QUARTER 3	QUARTER 4	TOTAL GRAMS
206	109.16	117.11	99.57	81.7	407.54
207	317.55	267.99	292.39	205.57	1,083.50
208	329.97	322.67	336.51	341.19	1,330.34
209	60.35	39	77.78	57.63	234.76
210	551.44	577.91	261.45	376.16	1,766.96
211	174.08	136.61	110.35	149.68	570.72
212	590.44	454.38	439.02	349.47	1,833.31
214	131.92	119.07	64.6	78.87	394.46
215	65.8	63.62	97.93	107.85	335.2
216	179.42	121.57	97.83	102.62	501.44
217	116.35	126.8	96.95	83.88	423.98
218	100.11	109.16	134.86	90.64	434.77
219	24.08	18.52	6.1	4.36	53.06
TOTAL	2,750.67	2,474.41	2,115.34	2,029.62	9,370.04

STATE: MASSACHUSETTS

ZIP CODE	QUARTER 1	QUARTER 2	QUARTER 3	QUARTER 4	TOTAL GRAMS
010	296.96	293.26	319.19	276.92	1,186.33
011	38.56	93.69	23.31	64.16	219.72

	012	27.02	31.16	1.74	35.95	95.87
	013	17.87	37.04	9.7	17.43	82.04
	014	105.02	118.42	93.25	61.01	377.7
	015	67.54	53.16	86.28	60.35	267.33
	016	33.66	30.94	30.61	11.44	106.65
	017	73.42	126.48	106.32	127.35	433.57
	018	161.66	149.68	131.6	139.88	582.82
	019	198.92	188.68	245.33	116.56	749.49
	020	114.17	116.02	121.36	71.79	423.34
	021	374.85	342.22	367.77	231.82	1,316.66
	022	39.65	40.31	62.75	12.09	154.8
	023	242.49	220.49	192.82	174.74	830.54
	024	102.07	123.97	64.93	168.85	459.82
	025	83.88	100.22	85.84	56.21	326.15
	026	53.6	26.15	46.84	34.86	161.45
	27	237.48	158.83	179.64	104.8	680.75
TOTAL		2,268.82	2,250.72	2,169.28	1,766.21	8,455.03

STATE:	MICHIGAN				
ZIP CODE	QUARTER 1	QUARTER 2	QUARTER 3	QUARTER 4	TOTAL GRAMS
480	414.29	419.3	369.84	280.51	1,483.94
481	301.54	279.21	240.32	213.84	1,034.91
482	70.59	26.8	100.33	53.82	251.54
483	79.42	152.29	102.95	83.45	418.11
484	110.68	98.7	107.52	128.44	445.34
485	29.41	45.43	32.68	72.99	180.51
486	267.81	325.09	270.17	188.94	1,052.01
487	39.87	45.97	103.27	52.29	241.4
488	197.07	170.71	169.62	164.71	702.11
489	218.09	178.55	142.82	181.38	720.84
490	325.29	292.39	267.44	172.88	1,058
491	2.18	8.06	2.18	3.27	15.69
492	158.83	124.62	119.61	118.96	522.02
493	40.09	87.8	64.71	21.79	214.39
494	111.99	63.29	92.92	93.47	361.67
495	265.15	147.07	107.3	117.33	636.85

	496	75.93	74.62	94.12	45.75	290.42
	497	220.93	209.6	172.34	183.02	785.89
	498	76.26	114.82	66.23	57.74	315.05
	499	50.76	41.94	38.78	66.23	197.71
TOTAL		3,056.18	2,906.26	2,665.15	2,300.81	10,928.40

STATE:	MINNESOTA					
ZIP CODE		QUARTER 1	QUARTER 2	QUARTER 3	QUARTER 4	TOTAL GRAMS
	550	50.33	59.92	44.23	60.79	215.27
	551	26.8	28.98	34.75	20.48	111.01
	553	90.53	38.89	61.11	89.22	279.75
	554	81.27	111.66	90.2	57.63	340.76
	556	4.36	0	4.79	0	9.15
	557	67.76	74.3	41.83	42.49	226.38
	558	98.26	68.19	49.68	78.22	294.35
	559	136.39	88.46	117.76	100.55	443.16
	560	16.56	22.44	20.7	18.3	78
	561	5.45	4.36	6.32	2.07	18.2
	562	18.41	22.66	31.92	6.75	79.74
	563	50.76	40.74	31.37	43.47	166.34
	564	59.7	43.62	38.78	25.03	167.13
	565	58.83	25.27	28.19	8.72	121.01
	566	33.55	5.01	11.77	16.89	67.22
	567	36.71	16.56	38.56	24.62	116.45
TOTAL		835.67	651.06	651.96	595.23	2,733.92

STATE:	MISSISSIPPI					
ZIP CODE		QUARTER 1	QUARTER 2	QUARTER 3	QUARTER 4	TOTAL GRAMS
	386	493.6	608.87	468.98	462	2,033.45
	387	292.8	336.18	298.58	300.93	1,228.49
	388	448.6	396.71	280.62	372.89	1,498.82
	389	253.87	236.94	210.84	203.63	905.28
	390	305.46	313.2	281.49	294.68	1,194.83
	391	346.09	322.02	266.46	281.28	1,215.85
	392	436.08	412.33	395.44	309.93	1,553.78

	393	365.4	319.4	366.27	414.62	1,465.69
	394	461.9	482.79	389.17	365.64	1,699.50
	395	907.67	769.69	731.95	781.95	3,191.26
	396	130.73	187.26	78.54	97.93	494.46
	397	228.29	234.78	222.6	147.48	833.15
TOTAL		4,670.49	4,620.17	3,990.94	4,032.96	17,314.56

STATE:	MISSOURI					
ZIP CODE		QUARTER 1	QUARTER 2	QUARTER 3	QUARTER 4	TOTAL GRAMS
	630	214.5	217.96	206.44	265.15	904.05
	631	1,307.18	912.46	1,028.48	511.57	3,759.69
	633	133.45	77.89	72.33	66.45	350.12
	634	7.19	15.47	17.43	16.67	56.76
	635	108.39	117.48	61.22	69.72	356.81
	636	84.86	87.15	94.56	79.74	346.31
	637	59.31	73.86	31.16	25.06	189.39
	638	99.92	130.07	129.16	108.94	468.09
	639	78	54.9	48.26	61.55	242.71
	640	359.47	267.11	230.01	263.08	1,119.67
	641	268.2	235.41	216.89	294.35	1,014.85
	644	34.42	41.4	29.85	20.15	125.82
	645	28.76	32.03	15.9	31.92	108.61
	646	27.89	19.61	7.84	18.52	73.86
	647	76.47	52.29	74.27	61.88	264.91
	648	217.11	272.45	133.67	141.07	764.3
	650	48.15	26.36	40.96	66.78	182.25
	651	89.33	79.74	100.22	73.31	342.6
	652	350.26	303.94	225.06	230.51	1,109.77
	653	68.63	88.11	63.84	100.22	320.8
	654	63.4	88.24	78.22	44.66	274.52
	655	215.91	196.52	124.19	160.57	697.19
	656	108.94	87.15	94.34	101.31	391.74
	657	364.72	237.92	235.2	184.1	1,021.94
	658	213.63	197.39	166.78	145.87	723.67
TOTAL		4,628.09	3,912.91	3,526.28	3,143.15	15,210.43

DATE RANGE: 01/01/2017 TO 12/31/2017

ARCOS 3 - REPORT 1

RETAIL DRUG DISTRIBUTION BY ZIP CODE WITHIN STATE BY GRAMS WT

Run Date: 07/03/2018

STATE: MONTANA

ZIP CODE	QUARTER 1	QUARTER 2	QUARTER 3	QUARTER 4	TOTAL GRAMS
590	10.02	6.75	25.06	8.93	50.76
591	48.8	49.13	57.95	50.98	206.86
592	12.2	28.32	19.61	13.07	73.2
593	27.45	44.99	34.21	5.66	112.31
594	160.84	177.66	137.04	158.18	633.72
595	7.84	1.53	1.53	6.54	17.44
596	95.43	112.42	96.74	86.71	391.3
597	55.34	66.89	58.28	37.26	217.77
598	87.8	90.2	58.28	90.85	327.13
599	151.21	196.74	168.42	139.44	655.81
TOTAL	656.93	774.63	657.12	597.62	2,686.30

STATE: NEBRASKA

ZIP CODE	QUARTER 1	QUARTER 2	QUARTER 3	QUARTER 4	TOTAL GRAMS
680	47.17	57.95	63.62	26.47	195.21
681	220.27	247.07	134.65	156.43	758.42
683	14.38	17.43	12.2	12.31	56.32
684	26.36	11.59	26.36	3.05	67.36
685	164.76	163.91	151.64	128.66	608.97
686	20.26	21.57	23.53	29.2	94.56
687	34.97	52.94	42.92	23.42	154.25
688	114.71	80.85	89.22	43.9	328.68
689	61.22	43.9	39.33	40.63	185.08
690	19.39	14.05	14.27	19.06	66.77
691	31.37	25.27	45.54	39.11	141.29
692	17.56	15.25	2.61	10.89	46.31
693	29.63	21.79	23.97	6.32	81.71
TOTAL	802.05	773.57	669.86	539.45	2,784.93

STATE: NEVADA

ZIP CODE	QUARTER 1	QUARTER 2	QUARTER 3	QUARTER 4	TOTAL GRAMS

	890	290.54	214.28	228.01	222.01	954.84
	891	988.28	700.03	772.58	1,063.67	3,524.56
	893	10.89	19.61	3.27	9.26	43.03
	894	73.97	48.15	49.78	64.27	236.17
	895	194.34	174.08	132.25	130.51	631.18
	897	42.7	57.39	43.4	30.5	173.99
	898	32.14	25.06	23.31	26.58	107.09
TOTAL		1,632.86	1,238.60	1,252.60	1,546.80	5,670.86

STATE: NEW HAMPSHIRE

ZIP CODE		QUARTER 1	QUARTER 2	QUARTER 3	QUARTER 4	TOTAL GRAMS
	030	81.05	41.51	42.05	47.61	212.22
	031	63.4	84.1	62.53	64.71	274.74
	032	115.91	110.46	98.37	106.98	431.72
	033	195	185.19	209.6	114.38	704.17
	034	22.44	31.59	23.97	28.32	106.32
	035	8.72	11.33	15.03	4.36	39.44
	037	29.41	25.82	40.96	20.7	116.89
	038	160.25	120.92	120.05	63.18	464.4
TOTAL		676.18	610.92	612.56	450.24	2,349.90

STATE: NEW JERSEY

ZIP CODE		QUARTER 1	QUARTER 2	QUARTER 3	QUARTER 4	TOTAL GRAMS
	70	297.73	258.51	224.52	210.69	991.45
	71	29.85	35.08	14.05	16.12	95.1
	72	2.18	4.36	1.09	1.09	8.72
	73	119.29	56.21	62.97	45.75	284.22
	74	122.88	163.62	106	30.28	422.78
	75	27.23	30.39	19.39	34.32	111.33
	76	202.62	128.55	148.48	154.69	634.34
	77	203.93	275.5	185.85	202.73	868.01
	78	58.17	45.86	47.39	16.56	167.98
	79	65.04	57.95	84.54	63.95	271.48
	80	328.34	285.85	247.18	154.8	1,016.17
	81	23.09	9.7	0	9.26	42.05

	82	29.63	57.74	35.95	23.42	146.74
	83	26.25	25.27	27.56	2.72	81.8
	84	0	5.45	0	0.87	6.32
	85	49.57	31.16	37.91	51.75	170.39
	86	17.87	41.51	23.09	20.48	102.95
	87	106.43	90.64	102.62	67.65	367.34
	88	423.98	539.35	451.65	387.27	1,802.25
	89	52.51	37.69	12.42	8.72	111.34
TOTAL		2,186.59	2,180.39	1,832.66	1,503.12	7,702.76

STATE: NEW MEXICO

ZIP CODE		QUARTER 1	QUARTER 2	QUARTER 3	QUARTER 4	TOTAL GRAMS
	870	185.63	52.29	75.17	54.9	367.99
	871	709.18	634.67	521.59	503.07	2,368.51
	873	7.41	4.58	7.19	0.44	19.62
	874	52.51	32.57	40.09	44.66	169.83
	875	260.47	175.17	220.27	196.09	852
	877	17.54	23.53	42.7	14.82	98.59
	878	0	9.59	1.74	6.1	17.43
	879	0	0	0.44	5.23	5.67
	880	132.36	260.58	173.21	239.12	805.27
	881	84.75	62.79	88.46	57.08	293.08
	882	183.02	130.83	126.26	98.92	539.03
	883	34.86	23.42	16.89	17.43	92.6
	884	13.07	6.54	0.87	0.44	20.92
TOTAL		1,680.80	1,416.56	1,314.88	1,238.30	5,650.54

STATE: NEW YORK

ZIP CODE		QUARTER 1	QUARTER 2	QUARTER 3	QUARTER 4	TOTAL GRAMS
	100	709.73	665.28	566.58	563.32	2,504.91
	101	24.4	47.28	51.85	39.22	162.75
	103	157.09	132.47	104.14	86.17	479.87
	104	144.45	164.82	150.33	133.34	592.94
	105	240.53	222.67	218.53	179.53	861.26
	106	8.06	26.15	0	0	34.21

107	74.62	62.31	38.67	61.22	236.82
108	9.59	14.38	3.7	12.31	39.98
109	98.26	40.31	33.33	63.07	234.97
110	123.54	154.69	152.95	116.78	547.96
111	11.98	11.98	29.41	8.17	61.54
112	200.99	197.94	205.67	220.82	825.42
113	106.11	63.62	99.68	122.23	391.64
114	35.95	28.98	44.88	23.97	133.78
115	65.04	125.82	104.36	95.65	390.87
116	9.8	22.88	8.72	0	41.4
117	412.98	339.89	276.37	246.63	1,275.87
118	113.84	78.87	79.52	69.72	341.95
119	21.79	11.11	19.72	9.26	61.88
120	129.64	105.02	75.6	91.29	401.55
121	65.8	61.66	29.63	45.97	203.06
122	31.37	52.07	28.21	17.65	129.3
123	82.14	99.79	121.57	68.19	371.69
124	113.95	143.8	106.54	86.61	450.9
125	108.39	58.28	65.47	57.85	289.99
126	60.68	114.93	105.12	125.28	406.01
127	31.7	8.72	29.41	4.36	74.19
128	121.79	79.31	83.88	80.07	365.05
129	118.96	20.59	75.06	120.05	334.66
130	256.11	194.8	178.22	210.25	839.38
131	31.16	33.66	61.01	17.43	143.26
132	89.44	98.59	71.46	87.37	346.86
133	69.2	65.49	82.66	56.12	273.47
134	56.84	39.98	27.78	40.2	164.8
135	40.85	37.37	35.73	39.22	153.17
136	26.47	15.08	6.75	7.34	55.64
137	8.72	12.85	3.92	12.64	38.13
138	51.42	63.88	72.99	54.47	242.76
139	10.78	10.13	12.2	8.72	41.83
140	84.64	42.05	53.71	38.24	218.64
141	97.17	59.7	52.51	70.81	280.19
142	370.61	282.47	254.91	282.47	1,190.46
143	101.31	100.77	119.83	135.08	456.99

	144	31.92	19.5	13.18	35.84	100.44
	145	31.16	66.89	54.9	34.42	187.37
	146	92.49	111.16	84.32	51.2	339.17
	147	53.38	77.65	15.95	59.48	206.46
	148	155.78	148.16	164.71	183.78	652.43
	149	30.72	27.23	26.15	27.23	111.33
TOTAL		5,123.34	4,663.03	4,301.79	4,201.04	18,289.20

STATE: NORTH CAROLINA

ZIP CODE		QUARTER 1	QUARTER 2	QUARTER 3	QUARTER 4	TOTAL GRAMS
	270	273.22	252.95	216.13	161.23	903.53
	271	233.02	188.14	180.62	73.86	675.64
	272	328.12	253.39	274.74	293.7	1,149.95
	273	277.57	209.81	160.79	153.6	801.77
	274	207.96	253.06	151.31	138.02	750.35
	275	749.05	656.89	632.93	478.67	2,517.54
	276	203.93	187.7	178.98	174.41	745.02
	277	49.78	27.45	23.53	62.42	163.18
	278	439.89	378.27	320.71	419.95	1,558.82
	279	277.79	208.51	202.3	198.81	887.41
	280	628.79	571.05	401.54	434.77	2,036.15
	281	723.67	609.83	564.41	644.15	2,542.06
	282	606.04	677.68	565.86	493.97	2,343.55
	283	410.48	378.64	308.08	402.85	1,500.05
	284	366.57	306.77	264.72	251.65	1,189.71
	285	325.72	301.32	218.09	250.23	1,095.36
	286	702.97	587.06	420.17	555.47	2,265.67
	287	314.39	364.29	284.33	215.37	1,178.38
	288	67.65	44.23	35.95	44.12	191.95
	289	4.36	5.45	26.15	9.8	45.76
TOTAL		7,190.97	6,462.49	5,431.34	5,457.05	24,541.85

STATE: NORTH DAKOTA

ZIP CODE		QUARTER 1	QUARTER 2	QUARTER 3	QUARTER 4	TOTAL GRAMS

	580	11.98	14.38	16.56	13.07	55.99
	581	41.83	27.45	35.3	24.4	128.98
	582	48.37	37.26	33.55	9.26	128.44
	583	23.97	19.61	23.09	23.53	90.2
	584	6.97	17.21	24.18	10.13	58.49
	585	50.55	79.31	59.26	100.22	289.34
	586	11.11	12.42	11.98	9.8	45.31
	587	37.47	23.31	43.36	27.78	131.92
	588	5.23	6.65	6.43	4.79	23.1
TOTAL		237.48	237.6	253.71	222.98	951.77

STATE: OHIO

ZIP CODE	QUARTER 1	QUARTER 2	QUARTER 3	QUARTER 4	TOTAL GRAMS
430	166.13	171.47	93.9	159.48	590.98
431	272.78	256.87	267.77	157.31	954.73
432	524.75	381.06	639.14	688.59	2,233.54
433	28.32	28.02	22.66	25.06	104.06
434	136.17	119.4	105.23	94.78	455.58
435	102.62	70.59	61.33	60.35	294.89
436	90.53	128.11	144.02	93.8	456.46
437	211.56	205.02	188.24	171.47	776.29
438	0.44	0.87	2.18	4.36	7.85
439	93.14	132.03	88.46	77.35	390.98
440	293.26	220.16	245.44	176.04	934.9
441	873.03	989.81	970.31	565.6	3,398.75
442	173.86	121.36	86.82	73.1	455.14
443	64.06	67.11	73.64	45.21	250.02
444	96.74	70.37	108.28	33.55	308.94
445	79.85	89	55.34	29.52	253.71
446	107.78	114.38	134.97	83.66	440.79
447	44.01	48.59	76.15	38.35	207.1
448	186.72	177.02	164.28	184.98	713
449	50.55	21.24	49.13	6.43	127.35
450	245.55	298.38	332.26	231.71	1,107.90
451	126.8	113.19	86.71	128.98	455.68
452	671.86	647.79	774.07	563.48	2,657.20

	453	116.89	167.76	136.72	166.67	588.04
	454	256.66	215.48	272.67	173.54	918.35
	455	26.36	27.23	31.59	11.98	97.16
	456	39.11	28.21	43.62	17.97	128.91
	457	37.15	26.8	39.33	23.97	127.25
	458	145.24	153.82	122.03	124.3	545.39
TOTAL		5,261.92	5,091.14	5,416.29	4,211.59	19,980.94

STATE: OKLAHOMA

ZIP CODE		QUARTER 1	QUARTER 2	QUARTER 3	QUARTER 4	TOTAL GRAMS
	730	1,342.11	1,221.82	1,224.57	1,033.53	4,822.03
	731	1,901.94	1,750.02	1,689.82	1,760.32	7,102.10
	734	64.27	49.89	61.88	47.39	223.43
	735	719.34	801.8	575.32	691.08	2,787.54
	736	87.69	52.62	77.45	82.79	300.55
	737	134.04	144.23	128.76	119.18	526.21
	738	20.59	10.46	6.1	15.36	52.51
	739	9.8	28.32	13.29	26.8	78.21
	740	1,203.87	1,132.56	1,097.48	1,197.44	4,631.35
	741	694.15	785.88	591.42	671.6	2,743.05
	743	148.16	125.17	161.12	134.43	568.88
	744	409.04	445.12	359.06	298.49	1,511.71
	745	189.05	211.23	185.85	131.71	717.84
	746	33.33	33.01	49.35	33.23	148.92
	747	181.38	147.28	178.11	186.28	693.05
	748	337.71	271.52	226.81	235.85	1,071.89
	749	178.88	174.13	181.49	141.62	676.12
TOTAL		7,655.35	7,385.06	6,807.88	6,807.10	28,655.39

STATE: OREGON

ZIP CODE		QUARTER 1	QUARTER 2	QUARTER 3	QUARTER 4	TOTAL GRAMS
	970	371.04	313.85	223.32	199.9	1,108.11
	971	146.52	154.36	212.43	110.9	624.21

	972	303.28	213.08	242.49	269.62	1,028.47
	973	330.52	261.34	263.74	194.56	1,050.16
	974	242.82	229.47	238.36	216.13	926.78
	975	168.85	162.53	182.58	165.15	679.11
	976	57.74	33.33	69.07	42.92	203.06
	977	45.54	38.78	51.53	44.45	180.3
	978	81.49	51.68	88.13	62.31	283.61
	979	1.74	5.88	14.16	9.8	31.58
TOTAL		1,749.54	1,464.30	1,585.81	1,315.74	6,115.39

STATE: PENNSYLVANIA

ZIP CODE		QUARTER 1	QUARTER 2	QUARTER 3	QUARTER 4	TOTAL GRAMS
	150	128.55	125.5	96.95	96.95	447.95
	151	96.74	92.6	84.1	63.62	337.06
	152	197.94	163.41	144.45	247.29	753.09
	153	54.03	54.03	43.58	47.17	198.81
	154	7.84	11.98	8.06	13.51	41.39
	155	10.89	10.33	8.72	3.81	33.75
	156	10.24	41.83	40.09	27.23	119.39
	157	11.98	14.86	7.84	24.95	59.63
	158	108.94	69.72	142.71	128.55	449.92
	159	66.34	65.36	59.26	47.93	238.89
	160	20.92	14.16	30.28	28.98	94.34
	161	61.11	51.64	36.6	26.04	175.39
	162	9.15	0.22	5.01	6.1	20.48
	163	46.19	46.19	40.52	23.97	156.87
	164	4.36	39.22	35.4	17.43	96.41
	165	65.04	44.23	42.7	54.8	206.77
	166	109.37	90.85	70.81	70.81	341.84
	167	4.79	4.36	0	4.36	13.51
	168	45.32	22.44	35.73	26.8	130.29
	169	0.87	0.44	3.05	1.53	5.89
	170	155.34	203.71	137.81	176.26	673.12
	171	93.9	30.5	46.63	20.04	191.07
	172	15.25	22.88	35.73	11.11	84.97

	173	186.61	199.68	237.27	218.96	842.52
	174	54.69	86.71	43.14	127.57	312.11
	175	32.03	34.21	28.76	50.22	145.22
	176	64.16	47.5	48.37	57.08	217.11
	177	69.61	79.96	49.13	68.63	267.33
	178	65.47	42.81	35.73	38.78	182.79
	179	25.06	14.16	23.42	22.33	84.97
	180	29.96	31.16	37.37	53.92	152.41
	181	109.7	93.03	125.71	96.19	424.63
	182	61.44	47.28	33.77	13.62	156.11
	183	8.06	18.52	0	4.36	30.94
	184	60.13	41.18	39.22	37.04	177.57
	185	94.12	49.68	82.36	62.31	288.47
	186	71.35	39.44	26.58	36.49	173.86
	187	316.9	274.41	347.29	247.83	1,186.43
	188	99.83	83.32	65.8	124.52	373.47
	189	61.88	69.07	34.53	52.83	218.31
	190	490.11	415.6	409.39	350.02	1,665.12
	191	328.01	259.71	283.24	212.97	1,083.93
	193	223.43	180.84	77.35	116.89	598.51
	194	79.42	100.77	131.6	59.7	371.49
	195	33.12	27.45	34.64	31.81	127.02
	196	53.82	60.79	74.62	58.83	248.06
TOTAL		3,944.01	3,517.74	3,425.32	3,312.14	14,199.21

STATE:	PUERTO RICO				
ZIP CODE	QUARTER 1	QUARTER 2	QUARTER 3	QUARTER 4	TOTAL GRAMS
6	1,536.35	1,894.42	1,320.65	937.84	5,689.26
7	1,905.43	1,213.56	1,072.05	543.49	4,734.53
9	2,211.21	1,823.72	1,726.01	1,187.09	6,948.03
TOTAL	5,652.99	4,931.70	4,118.71	2,668.42	17,371.82

STATE:	RHODE ISLAND				
ZIP CODE	QUARTER 1	QUARTER 2	QUARTER 3	QUARTER 4	TOTAL GRAMS
28	89.76	80.61	76.04	92.38	338.79

	29	163.19	184	180.73	140.09	668.01
TOTAL		252.95	264.61	256.77	232.47	1,006.80

STATE: SOUTH CAROLINA

ZIP CODE		QUARTER 1	QUARTER 2	QUARTER 3	QUARTER 4	TOTAL GRAMS
	290	854.07	732.06	654.71	598.07	2,838.91
	291	602.64	521.48	419.63	456.12	1,999.87
	292	998.41	773.89	887.51	791.98	3,451.79
	293	468.76	571.81	389.34	337.71	1,767.62
	294	991.55	1,075.10	815.18	780.65	3,662.48
	295	933.59	623.45	637.94	652.86	2,847.84
	296	1,047.87	1,009.52	892.85	887.4	3,837.64
	297	306.33	307.86	245.76	192.06	1,052.01
	298	371.91	343.37	367.34	294.13	1,376.75
	299	205.35	160.57	214.5	101.86	682.28
TOTAL		6,780.48	6,119.11	5,524.76	5,092.84	23,517.19

STATE: SOUTH DAKOTA

ZIP CODE		QUARTER 1	QUARTER 2	QUARTER 3	QUARTER 4	TOTAL GRAMS
	570	159.27	142.05	136.39	117.54	555.25
	571	161.23	138.79	153.71	157.31	611.04
	572	44.01	55.88	36.17	37.26	173.32
	573	22.01	32.25	48.37	15.14	117.77
	574	28.43	28.98	26.58	11.11	95.1
	575	16.12	10.24	8.28	0.44	35.08
	576	6.54	0.87	1.74	0	9.15
	577	113.3	149.68	118.52	98.04	479.54
TOTAL		550.91	558.74	529.76	436.84	2,076.25

STATE: TENNESSEE

ZIP CODE		QUARTER 1	QUARTER 2	QUARTER 3	QUARTER 4	TOTAL GRAMS
	370	1,583.41	1,413.68	1,046.56	968.35	5,012
	371	1,263.89	1,202.56	1,090.57	921.94	4,478.96

	372	823.02	704.06	622.69	489.67	2,639.44
	373	885.01	759.43	635.58	713.43	2,993.45
	374	426.16	369.89	259.92	288.03	1,344
	376	318.64	286.94	196.41	255.24	1,057.23
	377	249.14	268.42	187.15	180.29	885
	378	395.51	356.81	313.83	327.79	1,393.94
	379	354.37	244.52	212.36	277.51	1,088.76
	380	381.72	371.52	389.67	261.34	1,404.25
	381	840.45	710.16	668.11	623.23	2,841.95
	382	239.88	185.63	219.73	187.07	832.31
	383	453.51	482.27	416.47	388.36	1,740.61
	384	322.89	288.36	219.4	266.9	1,097.55
	385	212.97	204.19	262.76	202.73	882.65
TOTAL		8,750.57	7,848.44	6,741.21	6,351.88	29,692.10

STATE: TEXAS

ZIP CODE		QUARTER 1	QUARTER 2	QUARTER 3	QUARTER 4	TOTAL GRAMS
	750	808.77	756.53	769.8	684.5	3,019.60
	751	140.68	170.75	164.06	145.43	620.92
	752	401.41	418.78	437.93	378.01	1,636.13
	753	7.63	18.63	13.29	0	39.55
	754	100.03	103.38	119.4	128.98	451.79
	755	75.17	66.45	93.69	47.82	283.13
	756	353.48	214.59	233.37	158.74	960.18
	757	191.51	213.54	188.27	201.58	794.9
	758	21.57	16.25	20.52	35.34	93.68
	759	158.29	165.3	151.53	138.5	613.62
	760	397.01	498.56	508.74	308.95	1,713.26
	761	323	412.87	570.94	447.19	1,754
	762	152.51	180.62	97.17	237.37	667.67
	763	113.84	105.54	81.16	114.38	414.92
	764	52.09	63.88	64.71	51.75	232.43
	765	123.1	114.86	129.31	136.93	504.2
	766	50.33	67.8	56.04	32.46	206.63
	767	57.63	89.55	109.48	86.5	343.16

	768	52.29	46.08	45.75	53.92	198.04
	769	82.25	66.02	106.98	67.43	322.68
	770	2,074	1,701.17	1,846.93	2,476.69	8,098.79
	773	379.09	397.56	396.3	487.6	1,660.55
	774	347.73	264.76	359.1	315.16	1,286.75
	775	616.26	427.58	413.85	437.06	1,894.75
	776	134.1	145.43	151.75	200.45	631.73
	777	136.93	75.6	97.06	192.82	502.41
	778	97.61	148.7	137.26	113.84	497.41
	779	53.49	53.92	53.71	50.66	211.78
	780	152.08	190.53	194.13	186.83	723.57
	781	55.34	60.29	66.13	78.22	259.98
	782	896.77	774.44	832.39	809.1	3,312.70
	783	30.39	37.26	13.51	31.59	112.75
	784	277.88	225.06	258.4	250.34	1,011.68
	785	906.69	918.82	1,012.84	874.33	3,712.68
	786	202.19	168.09	204.58	208.4	783.26
	787	273.87	210.9	332.7	289.12	1,106.59
	788	36.1	52.4	64.16	78.87	231.53
	789	17.43	20.7	6.1	10.46	54.69
	790	38.67	36.25	30.5	34.32	139.74
	791	189.01	209.81	185.19	150.12	734.13
	792	15.8	19.06	20.15	4.9	59.91
	793	83.38	70.83	95.21	147.28	396.7
	794	196.96	156.5	144.56	161.99	660.01
	795	72.01	65.36	24.4	29.74	191.51
	796	103.16	67.65	176.15	63.18	410.14
	797	475.51	357.32	372.02	545.99	1,750.84
	798	2.18	1.31	11.44	0	14.93
	799	252.84	164.06	327.36	264.06	1,008.32
TOTAL		11,780.06	10,811.34	11,790.02	11,948.90	46,330.32

STATE:	UTAH					
ZIP CODE		QUARTER 1	QUARTER 2	QUARTER 3	QUARTER 4	TOTAL GRAMS
	840	772.45	642.23	683.26	697.51	2,795.45
	841	478.78	329.1	416.47	420.5	1,644.85

	843	84.32	71.14	103.6	107.3	366.36
	844	202.62	133.12	91.94	188.68	616.36
	845	127.57	67.21	62.09	78.44	335.31
	846	167.76	183.06	197.83	250.88	799.53
	847	177.24	135.41	233.32	91.73	637.7
TOTAL		2,010.74	1,561.27	1,788.51	1,835.04	7,195.56

STATE: VERMONT

ZIP CODE	QUARTER 1	QUARTER 2	QUARTER 3	QUARTER 4	TOTAL GRAMS
50	7.63	1.09	4.36	0	13.08
51	0.44	0.44	0.44	0	1.32
52	0.22	0.44	1.96	1.09	3.71
53	2.18	0	0	0	2.18
54	231.49	234.22	200.23	319.4	985.34
56	19.61	9.8	11.98	14.49	55.88
57	52.73	61.01	46.19	46.41	206.34
58	9.59	8.06	0.44	1.96	20.05
TOTAL	323.89	315.06	265.6	383.35	1,287.90

STATE: VIRGIN ISLANDS

ZIP CODE	QUARTER 1	QUARTER 2	QUARTER 3	QUARTER 4	TOTAL GRAMS
8	41.94	67.65	41.94	102.4	253.93
TOTAL	41.94	67.65	41.94	102.4	253.93

STATE: VIRGINIA

ZIP CODE	QUARTER 1	QUARTER 2	QUARTER 3	QUARTER 4	TOTAL GRAMS
201	344.24	382.59	273	290.75	1,290.58
220	210.14	230.51	243.58	198.48	882.71
221	113.08	84.1	99.68	112.97	409.83
222	29.85	30.94	60.79	46.08	167.66
223	189.55	159.27	247.94	174.08	770.84
224	280.41	172.34	191.73	174.3	818.78
225	111.12	112.21	31.7	47.06	302.09
226	189.66	161.23	166.46	158.18	675.53

	227	54.47	10.89	12.2	19.17	96.73
	228	127.24	118.74	77.35	75.17	398.5
	229	304.37	270.71	250.45	305.35	1,130.88
	230	252.95	181.71	141.62	194.02	770.3
	231	642.73	606.67	443.48	435.42	2,128.30
	232	884.68	807.16	708.42	662.56	3,062.82
	233	298.92	175.72	206.55	129.42	810.61
	234	810.5	733.91	532.16	432.92	2,509.49
	235	250.99	206.55	168.64	165.26	791.44
	236	290.65	247.72	371.26	238.36	1,147.99
	237	93.25	67.32	92.27	35.51	288.35
	238	297.29	165.8	234.22	264.06	961.37
	239	89.33	72.16	112.25	99.79	373.53
	240	242.17	202.73	164.71	158.4	768.01
	241	187.15	137.48	108.61	66.89	500.13
	242	271.04	245.33	247.51	189.55	953.43
	243	164.71	94.99	109.59	54.25	423.54
	244	72.88	91.73	35.73	77.89	278.23
	245	167.11	157.26	126.37	102.29	553.03
	246	8.72	4.36	1.31	0	14.39
TOTAL		6,979.20	5,932.13	5,459.58	4,908.18	23,279.09

STATE:	WASHINGTON				
ZIP CODE	QUARTER 1	QUARTER 2	QUARTER 3	QUARTER 4	TOTAL GRAMS
980	723.13	582.92	594.25	494.14	2,394.44
981	436.84	450.78	377.14	407.86	1,672.62
982	605.69	518	556.89	597.96	2,278.54
983	373.76	338.58	370.5	308.29	1,391.13
984	194.89	185.52	139.33	90.31	610.05
985	258.4	190.75	167.76	208.29	825.2
986	235.31	173.43	175.61	200.77	785.12
988	163.52	162.1	178.22	151.97	655.81
989	209.27	146.63	119.18	83.45	558.53
990	17.43	26.15	27.23	33.33	104.14
991	166.78	144.12	137.7	163.62	612.22
992	159.59	183.34	210.9	146.96	700.79

	993	194.34	236.15	189.33	214.72	834.54
	994	34.42	32.68	20.92	32.25	120.27
TOTAL		3,773.37	3,371.15	3,264.96	3,133.92	13,543.40

STATE:	WEST VIRGINIA					
ZIP CODE		QUARTER 1	QUARTER 2	QUARTER 3	QUARTER 4	TOTAL GRAMS
	247	40.09	31.59	37.04	39.44	148.16
	248	19.61	13.07	10.89	8.72	52.29
	249	39.54	23.86	20.92	34.86	119.18
	250	4.36	5.45	8.72	4.36	22.89
	251	2.51	1.63	2.94	2.18	9.26
	252	6.32	1.74	1.96	1.31	11.33
	253	90.64	73.64	54.25	43.68	262.21
	254	93.9	54.47	61.44	49.02	258.83
	255	29.2	26.15	34.32	4.79	94.46
	256	14.16	20.7	6.43	37.04	78.33
	257	53.49	71.57	72.23	35.4	232.69
	258	15.47	12.31	13.84	12.2	53.82
	259	9.26	11.98	10.89	15.25	47.38
	260	87.15	62.53	39.33	85.95	274.96
	261	107.74	128.11	110.35	73.21	419.41
	262	3.49	1.74	0.87	0	6.1
	263	48.15	57.08	57.19	38.89	201.31
	264	3.27	6.54	2.18	3.27	15.26
	265	42.05	37.26	27.89	7.52	114.72
	266	14.27	2.18	5.88	1.31	23.64
	267	5.01	11.11	0.44	10.24	26.8
	268	1.96	7.73	6.21	1.53	17.43
TOTAL		731.64	662.44	586.21	510.17	2,490.46

STATE:	WISCONSIN					
ZIP CODE		QUARTER 1	QUARTER 2	QUARTER 3	QUARTER 4	TOTAL GRAMS
	530	230.51	222.34	188.57	207.74	849.16
	531	307.09	287.92	343.15	282.26	1,220.42
	532	140.53	152.29	106.11	121.14	520.07

	534	34.86	32.25	0	24.4	91.51
	535	95.21	115.04	96.3	71.9	378.45
	537	104.58	115.91	85.84	81.92	388.25
	538	4.36	0	2.61	0	6.97
	539	11.44	12.42	11.33	23.42	58.61
	540	6.97	10.89	13.29	0.54	31.69
	541	22.66	17.43	22.01	40.52	102.62
	542	46.3	31.59	46.19	40.31	164.39
	543	173.1	187.81	162.1	167.76	690.77
	544	125.06	69.07	93.03	95.54	382.7
	545	11.77	14.6	11.55	13.07	50.99
	546	37.47	33.99	47.06	18.3	136.82
	547	38.56	21.57	29.52	30.07	119.72
	548	31.37	31.81	46.84	27.02	137.04
	549	136.72	140.53	161.12	84.43	522.8
TOTAL		1,558.56	1,497.46	1,466.62	1,330.34	5,852.98

STATE: WYOMING

ZIP CODE		QUARTER 1	QUARTER 2	QUARTER 3	QUARTER 4	TOTAL GRAMS
	820	33.55	38.65	15.69	28.32	116.21
	822	20.7	42.92	40.31	64.27	168.2
	823	13.07	0	0	17.43	30.5
	824	33.99	18.78	9.37	30.07	92.21
	825	44.66	36.82	24.95	27.23	133.66
	826	81.59	39.44	64.38	58.72	244.13
	827	26.15	28.76	16.34	10.35	81.6
	828	39.65	39.91	35.95	58.83	174.34
	829	17.43	23.09	19.17	32.68	92.37
	830	1.09	2.18	1.09	1.09	5.45
	831	66.45	47.71	27.02	21.79	162.97
TOTAL		378.33	318.26	254.27	350.78	1,301.64

DRUG CODE: 9250B DRUG NAME: METHADONE

STATE: ALABAMA

DATE RANGE: 01/01/2017 TO 12/31/2017 ARCOS 3 - REPORT 1 Run Date: 07/03/2018

RETAIL DRUG DISTRIBUTION BY ZIP CODE WITHIN STATE BY GRAMS WT

ZIP CODE	QUARTER 1	QUARTER 2	QUARTER 3	QUARTER 4	TOTAL GRAMS
350	10,609.82	13,421.81	10,981.26	11,844.09	46,856.98
351	1,380.24	1,339.72	1,291.51	1,274.97	5,286.44
352	13,820.45	11,566.47	13,443.66	12,995.64	51,826.22
354	5,013.74	4,096.73	3,974.27	3,931.51	17,016.25
355	6,822.48	5,457.18	6,274.39	5,687.04	24,241.09
356	994.57	1,680.40	2,380.89	3,059.65	8,115.51
357	988.31	935.54	901.56	880.54	3,705.95
358	11,544.83	13,033.91	11,835.33	13,310.01	49,724.08
359	1,328.01	1,906.41	2,564.16	3,311.96	9,110.54
360	721.24	686.63	550.95	529.75	2,488.57
361	2,372.84	1,491.86	1,450.27	1,356.63	6,671.60
362	3,726.52	4,654.46	3,282.45	4,014.78	15,678.21
363	2,243.16	2,207.83	2,620.59	2,219.01	9,290.59
364	326.46	354.63	226.73	292.92	1,200.74
365	3,874.54	4,020.78	3,338.35	3,022.62	14,256.29
366	8,926.20	12,380.91	8,393.32	11,506.01	41,206.44
367	629.21	592.09	598.8	467.32	2,287.42
368	927.94	875.71	791.37	777.41	3,372.43
369	29.52	23.25	25.94	8.05	86.76
TOTAL	76,280.08	80,726.32	74,925.80	80,489.91	312,422.11

STATE: ALASKA

ZIP CODE	QUARTER 1	QUARTER 2	QUARTER 3	QUARTER 4	TOTAL GRAMS
995	4,423.61	5,561.11	5,290.29	4,757.85	20,032.86
996	2,395.74	1,852.39	1,803.11	1,712.33	7,763.57
997	754.87	789.93	1,081.96	764.26	3,391.02
998	190.95	178.88	180.85	153.39	704.07
999	44.72	52.77	45.17	52.77	195.43
TOTAL	7,809.89	8,435.08	8,401.38	7,440.60	32,086.95

STATE: ARIZONA

ZIP CODE	QUARTER 1	QUARTER 2	QUARTER 3	QUARTER 4	TOTAL GRAMS
850	28,493.80	26,415.34	26,562.29	25,498.23	106,969.66
851	718.02	578.14	588.16	529.84	2,414.16

	852	18,036.38	18,385.02	19,068.52	17,992.64	73,482.56
	853	6,364.22	6,025.39	6,947.97	5,464.16	24,801.74
	855	634.13	609.98	554.08	560.34	2,358.53
	856	579.75	524.12	474.93	443.26	2,022.06
	857	25,579.21	27,596.62	23,946.93	27,061.41	104,184.17
	859	344.25	374.22	352.93	325.83	1,397.23
	860	1,045.11	1,005.31	1,022.84	976.77	4,050.03
	863	1,869.30	2,122.86	1,518.69	1,860.35	7,371.20
	864	1,518.69	1,447.14	1,392.13	2,299.95	6,657.91
	865	7.6	3.58	8.94	4.02	24.14
TOTAL		85,190.46	85,087.72	82,438.41	83,016.80	335,733.39

DRUG CODE:9250BDRUG NAME:METHADONE

STATE: ARKANSAS

ZIP CODE		QUARTER 1	QUARTER 2	QUARTER 3	QUARTER 4	TOTAL GRAMS
	716	264.83	234.33	249.98	197.22	946.36
	717	497.38	435.57	413.21	376.1	1,722.26
	718	1,718.59	1,686.21	1,668.95	1,282.57	6,356.32
	719	634.13	401.23	364.02	373.86	1,773.24
	720	842.08	789.31	696.38	654.52	2,982.29
	721	2,970.75	2,895.71	2,204.70	3,181.20	11,252.36
	722	602.24	1,000.65	1,386.59	490.22	3,479.70
	723	228.97	214.21	219.13	489.68	1,151.99
	724	2,528.47	2,524.89	2,498.95	2,500.30	10,052.61
	725	301.86	306.33	275.92	253.56	1,137.67
	726	560.52	517.41	493.26	453.01	2,024.20
	727	4,165.40	4,126.94	3,690.38	3,602.55	15,585.27
	728	256.69	255.8	227.62	196.77	936.88
	729	1,306.27	1,219.51	1,179.71	1,134.99	4,840.48
TOTAL		16,878.18	16,608.10	15,568.80	15,186.55	64,241.63

STATE: CALIFORNIA

ZIP CODE		QUARTER 1	QUARTER 2	QUARTER 3	QUARTER 4	TOTAL GRAMS
	900	16,395.98	16,881.41	16,238.52	13,911.13	63,427.04
	902	3,219.21	4,213.43	3,267.33	3,421.62	14,121.59

903	1,398.84	1,433.72	1,060.31	1,398.84	5,291.71
904	284.15	271.54	231.74	234.78	1,022.21
905	403.37	376.9	315.99	346.94	1,443.20
906	5,602.34	5,850.27	6,615.88	5,814.49	23,882.98
907	5,154.07	4,259.22	4,730.12	4,909.27	19,052.68
908	2,886.59	1,659.02	2,978.98	1,233.91	8,758.50
910	551.22	629.03	565.98	495.32	2,241.55
911	932.5	587.26	598.8	1,227.39	3,345.95
912	1,316.91	1,651.15	1,288.83	1,656.07	5,912.96
913	6,042.12	6,032.55	6,121.90	5,723.53	23,920.10
914	3,105.36	3,034.34	3,373.95	2,917.35	12,431
915	289.34	272.43	228.34	234.87	1,024.98
916	916.31	700.76	880.54	961.03	3,458.64
917	8,152.72	8,908.49	9,275.29	7,619.04	33,955.54
918	35.33	40.25	36.67	33.54	145.79
919	2,773.98	3,113.41	2,857.79	2,878.63	11,623.81
920	13,497.48	14,296.09	12,552.81	14,259.78	54,606.16
921	11,052.46	12,496.38	11,033.77	9,241.30	43,823.91
922	6,854.41	5,001.40	7,389.35	5,988.46	25,233.62
923	8,831.40	7,579.31	7,592	8,199.63	32,202.34
924	243.9	226.28	189.52	222.26	881.96
925	7,438.46	7,664.29	6,983.83	6,951.46	29,038.04
926	2,292.81	2,272.15	2,136.92	2,116	8,817.88
927	3,443.62	3,510.43	3,532.34	3,232.63	13,719.02
928	5,234.04	5,376.60	5,033.37	4,783.99	20,428
930	10,055.83	10,110.21	10,051.62	9,208.21	39,425.87
931	2,736.15	2,700.82	2,691.25	2,668.62	10,796.84
932	8,628.10	9,885.09	8,316.40	8,784.80	35,614.39
933	9,598.16	9,488.78	9,443.16	9,162.68	37,692.78
934	6,362.49	6,113.76	6,101.24	5,995.61	24,573.10
935	3,352.93	4,601.51	3,847.53	4,129.71	15,931.68
936	2,417.21	2,299.59	2,112.22	2,106.41	8,935.43
937	16,716.51	16,152.15	18,068.76	17,468.97	68,406.39
939	2,514.70	2,424	2,518.99	2,403.25	9,860.94
940	5,173.48	4,646.94	4,561.08	3,968.01	18,349.51
941	20,405.52	24,888.98	20,099.41	22,050.68	87,444.59
943	469.01	484.74	433.34	447.4	1,834.49

	944	333.16	311.79	325.02	322.34	1,292.31
	945	23,719.67	22,914.17	23,232.93	22,388.89	92,255.66
	946	8,429.09	10,536.39	9,023.42	9,273.94	37,262.84
	947	1,476.03	1,076.95	1,427.02	1,109.32	5,089.32
	948	3,940.91	2,732.03	2,742.95	3,861.93	13,277.82
	949	2,513.26	3,318.76	3,249.99	3,269.22	12,351.23
	950	7,918.31	6,828.48	7,453.58	6,484.68	28,685.05
	951	2,859.40	2,822.28	2,815.39	3,603.90	12,100.97
	952	16,733.33	15,186.73	15,576.78	15,121.44	62,618.28
	953	18,343.52	16,506.15	15,841.26	16,814.09	67,505.02
	954	6,735.10	7,803.55	6,592.18	6,506.58	27,637.41
	955	1,365.93	1,384.35	1,252.16	1,200.37	5,202.81
	956	14,877.27	15,412.75	13,860.70	14,658.50	58,809.22
	957	789.93	724.73	684.75	613.83	2,813.24
	958	18,937.54	20,041.04	18,910.18	18,881.52	76,770.28
	959	12,943.22	12,100.43	10,703.20	11,451.54	47,198.39
	960	3,400.60	3,789.84	3,454.17	3,228.43	13,873.04
	961	351.05	368.94	303.38	341.21	1,364.58
TOTAL		352,446.33	355,994.04	342,804.93	337,539.34	1,388,784.64

STATE: COLORADO

ZIP CODE	QUARTER 1	QUARTER 2	QUARTER 3	QUARTER 4	TOTAL GRAMS
800	8,402.27	10,014.20	9,668.39	8,318.02	36,402.88
801	2,157.92	1,983.33	2,411.39	2,968.25	9,520.89
802	19,193.65	17,657.78	18,201.76	18,051.94	73,105.13
803	248.64	448.54	448	762.03	1,907.21
804	314.02	280.84	305.44	338.53	1,238.83
805	3,163.22	3,061.35	3,453.99	3,001.79	12,680.35
806	427.52	468.4	452.3	391.66	1,739.88
807	84.07	63.06	38.46	54.11	239.7
808	290.41	255.8	225.39	184.69	956.29
809	3,319.92	3,961.39	3,911.39	3,699.15	14,891.85
810	1,897.29	2,347.35	2,496.18	2,362.47	9,103.29
811	373.41	367.6	421.71	226.28	1,389
812	186.93	173.96	145.34	164.84	671.07
813	531.72	743.69	517.86	733.86	2,527.13

	814	107.33	105.99	109.56	123.43	446.31
	815	1,261.82	1,234.09	2,055.15	1,174.97	5,726.03
	816	140.42	126.56	107.33	98.38	472.69
TOTAL		42,100.56	43,293.93	44,969.64	42,654.40	173,018.53

STATE: CONNECTICUT

ZIP CODE	QUARTER 1	QUARTER 2	QUARTER 3	QUARTER 4	TOTAL GRAMS
60	16,305.99	10,906.85	14,142.88	17,324.26	58,679.98
61	12,760.58	16,133.23	12,998.49	10,982.83	52,875.13
62	3,741.72	5,074.38	4,888.79	3,939.38	17,644.27
63	7,013.53	6,960.58	10,209.22	10,131.58	34,314.91
64	15,192.55	13,017.10	8,436.79	15,123.77	51,770.21
65	21,776.58	35,617.96	13,649.84	37,163.93	108,208.31
66	23,560.87	17,712.52	19,532.44	12,828.11	73,633.94
67	10,324.69	11,818.60	12,729.73	9,989.82	44,862.84
68	6,658.63	6,702.37	6,650.04	6,655.41	26,666.45
69	1,923.85	1,967.77	1,931.46	1,925.64	7,748.72
TOTAL	119,258.99	125,911.36	105,169.68	126,064.73	476,404.76

STATE: DELAWARE

ZIP CODE	QUARTER 1	QUARTER 2	QUARTER 3	QUARTER 4	TOTAL GRAMS
197	11,191.54	12,796.54	11,738.64	11,544.74	47,271.46
198	6,679.23	6,409.18	6,609.44	5,316.76	25,014.61
199	7,389.26	11,480.97	8,053.18	10,783.87	37,707.28
TOTAL	25,260.03	30,686.69	26,401.26	27,645.37	109,993.35

STATE: DISTRICT OF COLUMBIA

ZIP CODE	QUARTER 1	QUARTER 2	QUARTER 3	QUARTER 4	TOTAL GRAMS
200	8,566.52	10,074.43	8,568.98	10,207.66	37,417.59
204	647.55	607.3	660.96	575.99	2,491.80
TOTAL	9,214.07	10,681.73	9,229.94	10,783.65	39,909.39

STATE: FLORIDA

ZIP CODE	QUARTER 1	QUARTER 2	QUARTER 3	QUARTER 4	TOTAL GRAMS
320	7,731.37	6,395.59	8,383.66	7,324.96	29,835.58
321	7,984.85	8,031.89	10,629.05	7,877.25	34,523.04
322	18,868.44	22,222.44	22,126.54	19,866.39	83,083.81
323	3,937.15	4,044.48	5,588.21	2,951.70	16,521.54
324	875.62	2,712.27	6,284.68	4,457.69	14,330.26
325	11,833.99	15,307.03	14,260.49	11,659.22	53,060.73
326	2,352.18	3,411.60	5,025.01	4,633.35	15,422.14
327	2,518.27	2,517.29	2,217.66	2,364.88	9,618.10
328	8,218.82	12,742.43	10,423.96	14,484.36	45,869.57
329	3,072.26	5,732.66	7,167.27	6,817.12	22,789.31
330	6,420.61	5,419.93	6,221.27	6,173.91	24,235.72
331	2,466.01	2,744.51	2,913.42	2,159.69	10,283.63
333	2,820.76	3,319.39	3,368.13	2,807.70	12,315.98
334	8,570.14	8,863.06	9,442.45	10,138.02	37,013.67
335	2,585.89	2,485.36	2,305.94	2,291.10	9,668.29
336	13,615.99	13,287.85	12,424.70	11,783.01	51,111.55
337	15,571.19	12,719.17	21,129.13	18,913.79	68,333.28
338	2,321.50	2,233.76	2,551.37	2,060.79	9,167.42
339	6,204.01	7,124.07	17,075.80	15,038.89	45,442.77
341	4,280.33	3,332.89	4,182.57	3,301.32	15,097.11
342	10,071.21	8,834.53	15,914.24	16,644.07	51,464.05
344	9,121.45	9,192.55	10,974.91	8,838.28	38,127.19
346	13,017.63	11,353.07	17,077.67	16,234.08	57,682.45
347	4,590.96	4,430.86	4,338.11	4,291.78	17,651.71
349	2,726.67	2,430.62	2,458.26	2,448.51	10,064.06
TOTAL	171,777.30	180,889.30	224,484.50	205,561.86	782,712.96

STATE: GEORGIA

ZIP CODE	QUARTER 1	QUARTER 2	QUARTER 3	QUARTER 4	TOTAL GRAMS
300	15,765.62	17,397.34	15,499.16	15,686.35	64,348.47
301	12,828.02	13,277.90	13,231.14	13,074.90	52,411.96
302	7,416.99	7,951.48	8,675.23	8,221.68	32,265.38
303	5,052.47	4,805.66	4,526.65	5,244.14	19,628.92
304	1,470.84	1,696.23	2,516.66	841.72	6,525.45

	305	14,433.96	12,951.74	12,198.70	14,256.20	53,840.60
	306	5,823.44	5,731.49	5,995.25	5,690.35	23,240.53
	307	23,711.44	29,392.04	23,675.22	25,995.38	102,774.08
	308	437.81	398.9	951.46	1,772.25	3,560.42
	309	2,166.56	3,542.59	2,709.99	3,391.92	11,811.06
	310	5,347.80	5,188.77	5,357.19	3,875.08	19,768.84
	312	2,305.76	2,310.32	2,891.77	2,370.34	9,878.19
	313	496.66	484.59	416.88	396.67	1,794.80
	314	3,786.62	5,111.05	4,067.73	4,345	17,310.40
	315	1,989.15	2,380	3,783.76	3,158.13	11,311.04
	316	457.93	1,006.38	2,141.19	1,586.22	5,191.72
	317	3,141.40	3,293.45	3,126.82	3,129.51	12,691.18
	318	135.5	138.63	132.37	128.61	535.11
	319	3,211.70	3,313.30	3,145.25	2,643.67	12,313.92
	398	988.67	1,119.79	1,351.44	936.88	4,396.78
TOTAL		110,968.34	121,491.65	116,393.86	116,745	465,598.85

STATE: GUAM

ZIP CODE		QUARTER 1	QUARTER 2	QUARTER 3	QUARTER 4	TOTAL GRAMS
	969	58.14	38.46	42.48	32.2	171.28
TOTAL		58.14	38.46	42.48	32.2	171.28

STATE: HAWAII

ZIP CODE		QUARTER 1	QUARTER 2	QUARTER 3	QUARTER 4	TOTAL GRAMS
	967	3,975.34	4,450.18	3,932.95	3,504.08	15,862.55
	968	3,884.11	4,531.03	3,678.49	4,339.54	16,433.17
TOTAL		7,859.45	8,981.21	7,611.44	7,843.62	32,295.72

STATE: IDAHO

ZIP CODE		QUARTER 1	QUARTER 2	QUARTER 3	QUARTER 4	TOTAL GRAMS
	832	955.04	904.78	842.08	828.3	3,530.20
	833	536.64	490.13	484.32	408.74	1,919.83
	834	529.57	515.71	464.91	441.21	1,951.40
	835	413.66	381.46	372.96	343	1,511.08

	836	2,744.20	2,294.49	2,743.04	2,135.38	9,917.11
	837	5,576.67	6,199.89	5,707.26	6,161.34	23,645.16
	838	1,304.04	1,265.67	1,125.60	1,062.73	4,758.04
TOTAL		12,059.82	12,052.13	11,740.17	11,380.70	47,232.82

STATE: ILLINOIS

ZIP CODE	QUARTER 1	QUARTER 2	QUARTER 3	QUARTER 4	TOTAL GRAMS
600	9,701.65	8,971.37	8,586.42	8,529.71	35,789.15
601	8,246.55	7,947.10	6,897.52	7,476.02	30,567.19
602	4,530.31	4,704.54	4,463.15	4,642.03	18,340.03
603	52.77	42.04	52.32	42.48	189.61
604	6,586.54	6,555.06	6,720.25	5,657.80	25,519.65
605	6,743.69	6,418.57	5,601.98	6,377.97	25,142.21
606	52,807.25	56,306.15	54,356.09	53,041.77	216,511.26
607	588.27	1,375.77	1,243.39	1,129.63	4,337.06
608	4,141.07	3,857.10	4,068.18	3,654.07	15,720.42
609	748.79	981.96	927.4	838.05	3,496.20
610	867.12	1,279.44	1,084.55	768.74	3,999.85
611	2,447.44	2,380.45	3,094.18	3,077.99	11,000.06
612	1,321.92	1,487.83	1,109.95	1,097.43	5,017.13
613	589.68	580.29	566.42	571.97	2,308.36
614	200.79	212.87	184.69	156.52	754.87
615	292.02	277.71	237.55	186.93	994.21
616	8,132.86	10,165.42	7,530.85	9,597.72	35,426.85
617	2,270.34	1,368.52	1,325.05	1,825.20	6,789.11
618	2,508.34	2,516.84	1,880.74	2,475.25	9,381.17
619	75.58	69.32	65.38	57.69	267.97
620	2,295.48	2,269.45	2,215.52	2,790.98	9,571.43
622	1,563.86	1,023.55	1,530.14	1,523.16	5,640.71
623	430.12	425.64	404.27	414.55	1,674.58
624	192.3	174.86	167.25	160.54	694.95
625	1,607.95	1,956.95	1,551.78	1,962.76	7,079.44
626	170.83	207.05	220.47	187.38	785.73
627	1,247.69	2,340.20	2,433.22	3,062.87	9,083.98
628	200.79	226.28	210.18	207.5	844.75
629	660.96	673.75	596.74	564.72	2,496.17

TOTAL	121,222.96	126,796.08	119,325.63	122,079.43	489,424.10

STATE: INDIANA

ZIP CODE	QUARTER 1	QUARTER 2	QUARTER 3	QUARTER 4	TOTAL GRAMS
460	703.45	594.95	630.28	652.11	2,580.79
461	1,418.70	1,353.67	1,278.54	1,187.23	5,238.14
462	18,333.80	17,855.23	17,699.38	17,479.62	71,368.03
463	2,195.87	2,226.52	1,768.23	2,132.70	8,323.32
464	5,469.70	6,147.21	5,576.58	6,482.61	23,676.10
465	2,320.88	2,272.13	2,069.82	1,954.26	8,617.09
466	3,204.19	3,676.88	3,701.92	3,701.03	14,284.02
467	1,125.33	1,101.90	1,043.23	985.45	4,255.91
468	6,659.70	6,584.39	6,492	5,692.41	25,428.50
469	7,656.96	7,518.33	7,541.04	7,869.74	30,586.07
470	7,499.28	7,452.14	7,503.21	6,100.17	28,554.80
471	13,610.17	13,571.45	12,310.88	12,233.51	51,726.01
472	553.19	489.68	483.96	505.78	2,032.61
473	7,100.37	6,050.44	5,586.15	6,773.56	25,510.52
474	958.35	845.21	842.97	721.78	3,368.31
475	327.35	279.95	275.92	279.95	1,163.17
476	294.7	272.34	284.87	287.55	1,139.46
477	3,587.98	3,322.87	3,563.29	2,987.12	13,461.26
478	322.88	261.61	180.67	174.41	939.57
479	1,221.48	1,172.29	1,161.83	1,125.78	4,681.38
TOTAL	84,564.33	83,049.19	79,994.77	79,326.77	326,935.06

STATE: IOWA

ZIP CODE	QUARTER 1	QUARTER 2	QUARTER 3	QUARTER 4	TOTAL GRAMS
500	262.51	267.87	216.53	232.1	979.01
501	210.01	188.81	166.36	173.33	738.51
502	240.15	232.45	224.49	220.2	917.29
503	3,961.92	5,381.16	4,639.70	4,938.61	18,921.39
504	157.41	138.18	157.06	147.58	600.23

	505	194.08	216.89	200.79	209.74	821.5
	506	803.89	454.89	775.44	1,132.58	3,166.80
	507	64.84	80.67	60.37	67.88	273.76
	508	92.57	96.15	82.28	80.5	351.5
	510	191.4	186.93	192.3	190.06	760.69
	511	413.93	850.13	398.28	851.02	2,513.36
	512	21.02	15.65	12.07	9.84	58.58
	513	45.17	43.38	51.88	28.17	168.6
	514	69.32	39	49.1	44.99	202.41
	515	381.91	355.52	315.28	266.98	1,319.69
	516	34.43	42.48	35.33	35.78	148.02
	520	122.71	119.04	128.79	104.47	475.01
	521	14.31	29.52	12.52	16.99	73.34
	522	253.47	219.66	231.11	214.75	918.99
	523	1,150.91	845.48	1,188.21	1,028.92	4,213.52
	524	1,241.07	1,338.92	1,268.53	1,575.22	5,423.74
	525	219.58	211.08	199	179.77	809.43
	526	177.72	182.01	154.46	168.5	682.69
	527	162.78	166.81	134.16	144.71	608.46
	528	1,812.50	1,788.53	1,762.42	1,761.16	7,124.61
TOTAL		12,299.61	13,491.21	12,656.46	13,823.85	52,271.13

STATE:	KANSAS					
ZIP CODE		QUARTER 1	QUARTER 2	QUARTER 3	QUARTER 4	TOTAL GRAMS
	660	1,665.73	2,030.38	2,469.89	1,555.72	7,721.72
	661	3,824.45	3,498.18	3,554.79	3,673.75	14,551.17
	662	1,585.50	1,820.64	1,760.27	1,537.38	6,703.79
	664	112.52	118.51	117.61	121.64	470.28
	665	146.5	123.87	120.3	138.36	529.03
	666	1,993.80	2,246.46	1,858.83	1,736.48	7,835.57
	667	402.48	414.55	349.71	382.36	1,549.10
	668	143.55	113.59	85.42	86.76	429.32
	669	98.38	99.28	106.88	92.12	396.66
	670	704.79	697.63	636.81	647.99	2,687.22
	671	324.76	307.94	313.31	304.1	1,250.11
	672	10,567.25	12,019.30	10,145.89	11,495.10	44,227.54

	673	148.02	166.81	114.93	130.14	559.9
	674	370.73	319.3	317.06	276.82	1,283.91
	675	232.99	217.79	190.06	204.37	845.21
	676	101.96	106.88	84.97	98.83	392.64
	677	46.51	41.59	42.48	43.83	174.41
	678	186.48	185.59	167.7	185.59	725.36
	679	31.3	25.94	28.17	27.28	112.69
TOTAL		22,687.70	24,554.23	22,465.08	22,738.62	92,445.63

STATE:	KENTUCKY					
ZIP CODE		QUARTER 1	QUARTER 2	QUARTER 3	QUARTER 4	TOTAL GRAMS
	400	465.98	462.85	451.14	421.26	1,801.23
	401	216.89	200.35	176.2	181.56	775
	402	9,927.66	8,085.38	10,393.46	9,366.87	37,773.37
	403	2,572.47	2,027.16	2,314.44	2,014.64	8,928.71
	404	708.81	666.78	1,002.18	644.68	3,022.45
	405	4,390.39	4,611.98	4,700.23	4,372.73	18,075.33
	406	1,892.10	1,863.48	1,892.10	1,866.17	7,513.85
	407	1,840.68	1,852.75	1,827.71	1,812.95	7,334.09
	408	86.31	100.62	78.71	81.39	347.03
	409	193.64	173.07	165.91	162.33	694.95
	410	9,801.91	9,602.64	11,154.24	9,815.59	40,374.38
	411	3,729.20	4,082.85	4,079.72	4,708.12	16,599.89
	412	1,249.48	1,247.69	1,254.84	1,245	4,997.01
	413	197.66	196.77	193.64	192.74	780.81
	414	99.73	93.02	87.2	86.76	366.71
	415	1,218.89	1,068	1,614.66	1,214.95	5,116.50
	416	170.83	173.07	186.93	151.15	681.98
	417	2,659.95	3,363.84	1,922.51	2,582.13	10,528.43
	418	37.12	39.35	39.8	30.86	147.13
	420	2,211.85	2,079.57	2,275.44	2,258.09	8,824.95
	421	2,266.05	2,332.60	2,859.84	2,511.48	9,969.97
	422	1,759.28	1,728.43	1,749	1,716.35	6,953.06
	423	1,007.45	1,138.57	1,225.78	1,332.66	4,704.46
	424	436.47	455.25	436.91	418.58	1,747.21
	425	148.92	157.41	131.48	430.65	868.46

	426	95.7	84.52	82.28	87.65	350.15
	427	1,723.06	1,713.67	1,700.25	1,706.96	6,843.94
TOTAL		51,108.48	49,601.67	53,996.60	51,414.30	206,121.05

STATE:	LOUISIANA					
ZIP CODE	QUARTER 1		QUARTER 2	QUARTER 3	QUARTER 4	TOTAL GRAMS
	700	6,325.91	9,776.24	7,480.94	5,929.87	29,512.96
	701	4,211.73	4,857.75	5,251.92	3,894.31	18,215.71
	703	193.64	154.73	179.77	182.46	710.6
	704	4,071.76	3,968.27	3,936.70	3,924.72	15,901.45
	705	3,895.56	4,651.77	4,547.85	3,603.90	16,699.08
	706	1,576.38	1,983.15	1,488.28	1,566.09	6,613.90
	707	560.61	475.11	474.3	409.01	1,919.03
	708	4,143.76	3,611.32	4,207.70	3,017.88	14,980.66
	710	495.94	486.11	473.41	461.33	1,916.79
	711	4,220.41	4,079	4,040.24	3,932.03	16,271.68
	712	1,668.95	1,042.69	997.26	976.68	4,685.58
	713	3,941.17	1,772.70	3,732.33	3,868.73	13,314.93
	714	526.8	521.88	425.73	419.92	1,894.33
TOTAL		35,832.62	37,380.72	37,236.43	32,186.93	142,636.70

STATE:	MAINE					
ZIP CODE	QUARTER 1		QUARTER 2	QUARTER 3	QUARTER 4	TOTAL GRAMS
	39	83.18	67.08	76.02	70.66	296.94
	40	5,002.56	4,862.50	3,783.13	4,031.96	17,680.15
	41	5,482.40	5,859.66	5,903.93	6,472.06	23,718.05
	42	2,671.93	2,002.47	2,018.21	2,543.05	9,235.66
	43	1,065.77	783.76	729.92	707.02	3,286.47
	44	8,960.81	10,398.47	9,441.29	10,346.06	39,146.63
	45	124.77	108.67	101.96	100.17	435.57
	46	1,524.50	1,776.73	1,441.77	1,411.18	6,154.18
	47	105.09	96.6	92.12	100.62	394.43
	48	1,580.40	1,360.38	1,467.17	1,420.93	5,828.88
	49	5,745.45	5,486.07	4,437.12	4,379.70	20,048.34
TOTAL		32,346.86	32,802.39	29,492.64	31,583.41	126,225.30

STATE: MARYLAND

ZIP CODE	QUARTER 1	QUARTER 2	QUARTER 3	QUARTER 4	TOTAL GRAMS
206	5,229.29	5,098.17	4,553.21	5,150.67	20,031.34
207	3,619.19	3,715.07	2,741.07	3,265.72	13,341.05
208	3,585.29	2,846.43	3,441.38	2,855.91	12,729.01
209	852.81	857.28	913.63	834.92	3,458.64
210	32,397.23	33,278.48	36,911.62	37,150.42	139,737.75
211	5,313.90	6,369.92	5,091.37	6,980.34	23,755.53
212	107,563.30	105,697.31	99,834.74	99,997.96	413,093.31
214	2,356.57	1,554.11	2,636.15	2,088.33	8,635.16
215	3,606.85	3,594.15	4,073.81	3,627.24	14,902.05
216	3,641.82	3,573.04	4,374.69	3,218.41	14,807.96
217	14,725.31	14,354.32	12,154.81	13,552.13	54,786.57
218	3,191.49	3,990.01	3,516.96	4,362.44	15,060.90
219	6,687.79	8,029.65	7,194.37	8,022.77	29,934.58
TOTAL	192,770.84	192,957.94	187,437.81	191,107.26	764,273.85

STATE: MASSACHUSETTS

ZIP CODE	QUARTER 1	QUARTER 2	QUARTER 3	QUARTER 4	TOTAL GRAMS
10	11,037.79	11,804.83	11,436.87	12,194.61	46,474.10
11	8,142.35	7,396.51	6,865.15	7,790.40	30,194.41
12	4,799.26	5,892.75	4,350.36	5,581.95	20,624.32
13	1,368.16	1,465.12	1,575.75	1,323.09	5,732.12
14	7,034.90	6,232.63	7,398.30	6,552.82	27,218.65
15	3,243.09	3,386.74	4,461.36	4,036.70	15,127.89
16	10,495.19	11,041.44	9,271.66	9,620.07	40,428.36
17	4,123.01	3,824.90	3,982.14	3,983.66	15,913.71
18	18,859.77	17,046.37	18,295.67	15,601.11	69,802.92
19	15,753.69	14,694.63	13,901.48	16,272.89	60,622.69
20	716.15	722.85	572.95	541.83	2,553.78
21	24,936.99	24,458.31	24,038.65	22,727.19	96,161.14
22	295.56	325.35	283.52	307.67	1,212.10

23	9,834.29	7,797.02	10,000.11	8,941.85	36,573.27
24	1,341.06	1,739.88	1,326.57	1,712.95	6,120.46
25	3,144.62	3,764.53	2,754.57	3,575.90	13,239.62
26	3,296.31	3,250.16	2,538.49	3,393.35	12,478.31
27	18,745.82	21,045.95	18,615.86	20,489.18	78,896.81
TOTAL	147,168.01	145,889.97	141,669.46	144,647.22	579,374.66

STATE: MICHIGAN

ZIP CODE	QUARTER 1	QUARTER 2	QUARTER 3	QUARTER 4	TOTAL GRAMS
480	15,733.30	18,922.64	16,045.07	16,499.22	67,200.23
481	15,833.47	16,624.93	13,891.64	13,954.88	60,304.92
482	15,225.66	14,681.99	14,813.63	15,073.25	59,794.53
483	11,465.58	11,538.12	10,348.74	11,766.37	45,118.81
484	1,329.53	1,276.31	1,250.82	1,191.34	5,048
485	10,130.33	6,494.24	11,878.97	8,887.21	37,390.75
486	5,276.33	5,028.59	4,979.93	4,822.60	20,107.45
487	738.77	651.57	591.82	578.86	2,561.02
488	5,623.18	4,212.53	7,308.32	5,832.02	22,976.05
489	7,294.57	9,350.16	7,273.62	8,369.45	32,287.80
490	12,351.04	10,472.89	11,027.24	10,713.12	44,564.29
491	464.1	477.7	441.21	452.12	1,835.13
492	4,798.63	5,527.84	5,418.28	4,675.48	20,420.23
493	1,245.90	1,212.63	1,156.46	1,071.49	4,686.48
494	8,564.60	8,174.10	8,870.12	9,720.61	35,329.43
495	9,944.58	10,594.92	9,661.77	10,189.44	40,390.71
496	2,947.76	3,021.55	2,735.43	2,637.85	11,342.59
497	4,075.69	5,534.90	5,231.79	3,730.27	18,572.65
498	839.39	843.87	826.43	852.36	3,362.05
499	219.58	173.96	160.54	164.93	719.01
TOTAL	134,101.99	134,815.44	133,911.83	131,182.87	534,012.13

STATE: MINNESOTA

ZIP CODE	QUARTER 1	QUARTER 2	QUARTER 3	QUARTER 4	TOTAL GRAMS
550	1,114.42	1,100.11	1,048.33	996.81	4,259.67
551	6,716.06	8,344.84	11,926.15	13,240.61	40,227.66

	553	2,459.69	2,448.87	2,430.53	3,151.24	10,490.33
	554	23,732.28	27,980.88	24,288.63	29,024.60	105,026.39
	556	28.17	17.89	38.01	27.28	111.35
	557	401.23	368.05	368.49	359.82	1,497.59
	558	2,652.52	3,756.12	2,848.04	4,057.45	13,314.13
	559	908.17	1,455.01	1,535.86	1,581.39	5,480.43
	560	195.34	211.35	224.67	245.78	877.14
	561	32.65	31.75	29.52	30.86	124.78
	562	118.96	113.41	110.01	111.62	454
	563	1,743.54	2,360.41	1,907.04	2,366.85	8,377.84
	564	3,973.46	3,952.80	3,917.92	3,882.32	15,726.50
	565	225.57	198.38	200.44	184.25	808.64
	566	132.01	186.84	116.81	126.11	561.77
	567	117.61	124.77	118.06	112.52	472.96
TOTAL		44,551.68	52,651.48	51,108.51	59,499.51	207,811.18

STATE:	MISSISSIPPI					
ZIP CODE		QUARTER 1	QUARTER 2	QUARTER 3	QUARTER 4	TOTAL GRAMS
	386	681.98	552.74	773.66	849.23	2,857.61
	387	379.67	360.44	339.42	327.35	1,406.88
	388	235.94	194.98	199.81	174.94	805.67
	389	103.3	109.56	96.6	106.88	416.34
	390	225.84	208.4	227.62	224.94	886.8
	391	212.87	199.45	217.34	217.79	847.45
	392	1,011.92	1,606.07	1,447.77	1,444.46	5,510.22
	393	911.39	732.07	1,239.64	1,119.34	4,002.44
	394	680.64	598.8	572.42	461.24	2,313.10
	395	1,334	1,189.10	1,915.36	2,011.86	6,450.32
	396	162.33	168.15	147.58	146.68	624.74
	397	145.97	142.66	129.69	103.75	522.07
TOTAL		6,085.85	6,062.42	7,306.91	7,188.46	26,643.64

STATE:	MISSOURI					
ZIP CODE		QUARTER 1	QUARTER 2	QUARTER 3	QUARTER 4	TOTAL GRAMS
	630	5,187.61	4,646.94	4,376.21	4,863.39	19,074.15

	631	13,106.06	9,687.49	12,865.09	13,005.83	48,664.47
	633	1,254.13	1,143.31	1,074.62	1,036.16	4,508.22
	634	278.16	278.16	255.8	259.38	1,071.50
	635	67.97	68.87	61.27	58.58	256.69
	636	330.57	336.03	289.25	270.02	1,225.87
	637	2,069.37	1,128.11	2,096.12	2,059.27	7,352.87
	638	138.72	142.66	145.07	146.68	573.13
	639	1,047.61	839.39	942.25	435.13	3,264.38
	640	1,198.50	1,131.42	1,066.04	993.23	4,389.19
	641	4,834.95	4,894.60	4,965.62	5,179.38	19,874.55
	644	118.96	121.19	104.64	105.99	450.78
	645	2,269.54	1,622.44	2,301.74	2,354.96	8,548.68
	646	175.75	161.44	157.41	137.74	632.34
	647	199.9	186.48	177.54	171.72	735.64
	648	1,627.45	2,160.33	1,619.31	2,773.71	8,180.80
	650	156.52	155.18	153.39	157.41	622.5
	651	146.68	146.68	129.24	125.22	547.82
	652	1,551.34	1,439.36	1,391.51	1,447.59	5,829.80
	653	92.12	93.91	84.97	96.15	367.15
	654	176.2	167.7	172.62	149.81	666.33
	655	262.06	230.76	211.08	212.42	916.32
	656	568.39	559.63	505.25	515.17	2,148.44
	657	1,701.24	1,681.47	1,707.86	1,563.86	6,654.43
	658	5,307.28	4,470.48	4,950.24	4,697.12	19,425.12
TOTAL		43,867.08	37,494.03	41,804.14	42,815.92	165,981.17

STATE: MONTANA

ZIP CODE		QUARTER 1	QUARTER 2	QUARTER 3	QUARTER 4	TOTAL GRAMS
	590	90.33	83.63	75.58	77.81	327.35
	591	1,842.46	818.64	1,389.09	1,396.25	5,446.44
	592	18.34	21.02	22.81	19.23	81.4
	593	51.88	40.25	21.47	28.62	142.22
	594	279.5	269.66	210.63	177.54	937.33
	595	39.35	42.04	42.04	36.22	159.65
	596	355.97	322.43	344.79	267.34	1,290.53
	597	755.59	770.7	1,017.83	968.19	3,512.31

	598	2,275	2,722.73	1,829.32	2,213.82	9,040.87
	599	1,971.97	2,393.86	1,543.73	2,373.29	8,282.85
TOTAL		7,680.39	7,484.96	6,497.29	7,558.31	29,220.95

STATE: NEBRASKA

ZIP CODE		QUARTER 1	QUARTER 2	QUARTER 3	QUARTER 4	TOTAL GRAMS
	680	476.45	394.88	356.42	350.6	1,578.35
	681	5,913.95	4,972.51	5,452.98	5,053.90	21,393.34
	683	78.26	80.94	73.79	55.01	288
	684	41.14	44.27	35.33	41.14	161.88
	685	1,374.55	1,626.02	1,314.64	1,644.94	5,960.15
	686	72.45	59.03	66.63	62.16	260.27
	687	169.94	168.15	140.87	162.33	641.29
	688	275.92	254.9	231.2	253.29	1,015.31
	689	53.84	52.32	46.78	51.07	204.01
	690	46.51	41.59	33.09	29.96	151.15
	691	137.11	127.72	130.58	112.25	507.66
	692	28.62	21.47	22.36	12.07	84.52
	693	131.3	115.02	116.27	116.16	478.75
TOTAL		8,800.04	7,958.82	8,020.94	7,944.88	32,724.68

STATE: NEVADA

ZIP CODE		QUARTER 1	QUARTER 2	QUARTER 3	QUARTER 4	TOTAL GRAMS
	890	11,961.08	10,356.62	10,495.43	9,406.58	42,219.71
	891	15,228.13	16,566.61	14,693.06	16,342.97	62,830.77
	893	35.78	30.41	33.09	33.09	132.37
	894	4,290.53	5,499.58	3,983.75	3,275.74	17,049.60
	895	4,389.36	4,428.17	4,183.91	4,131.86	17,133.30
	897	995.56	1,006.11	1,573.25	1,487.83	5,062.75
	898	77.37	60.37	67.08	62.7	267.52
TOTAL		36,977.81	37,947.87	35,029.57	34,740.77	144,696.02

STATE: NEW HAMPSHIRE

ZIP CODE	QUARTER 1	QUARTER 2	QUARTER 3	QUARTER 4	TOTAL GRAMS
30	4,070.68	3,500.50	4,020.51	4,014.51	15,606.20
31	9,281.10	10,069.33	7,602.31	10,051.45	37,004.19
32	642.36	638.6	591.2	564.37	2,436.53
33	5,270.34	4,805.61	4,134.81	4,187.31	18,398.07
34	2,010.16	2,007.93	3,847.71	2,001.67	9,867.47
35	286.21	294.7	241.94	207.95	1,030.80
36	32.2	34.43	31.75	29.52	127.9
37	2,532.49	3,157.23	3,136.75	2,612.10	11,438.57
38	6,791	6,725.98	6,973.01	6,933.93	27,423.92
TOTAL	30,916.54	31,234.31	30,579.99	30,602.81	123,333.65

STATE: NEW JERSEY

ZIP CODE	QUARTER 1	QUARTER 2	QUARTER 3	QUARTER 4	TOTAL GRAMS
70	9,863.44	8,346.63	7,664.83	8,847.58	34,722.48
71	9,470.18	12,585.43	12,807.92	11,333.55	46,197.08
72	1,851.86	2,744.02	1,839.60	1,886.29	8,321.77
73	3,879.01	5,720.58	5,314.08	4,420.12	19,333.79
74	795.12	762.03	746.82	675.72	2,979.69
75	2,860.29	4,679.59	1,015.32	3,190.77	11,745.97
76	2,698.49	2,625.24	2,177.51	2,593.58	10,094.82
77	8,340.01	8,896.69	8,814.49	9,720.34	35,771.53
78	4,694.71	435.57	1,314.50	2,568.54	9,013.32
79	240.86	232.99	210.36	237.55	921.76
80	3,893.41	3,728.13	5,372.03	3,751.20	16,744.77
81	25,520.36	25,484.23	18,716.48	25,190.06	94,911.13
82	7,352.15	9,324.66	7,959	9,190.94	33,826.75
83	4,554.73	5,543.94	4,272.55	5,576.67	19,947.89
84	135.23	135.14	102.95	103.75	477.07
85	437.99	439.96	437.36	411.42	1,726.73
86	6,334.05	5,615.40	4,460.37	4,822.16	21,231.98
87	8,965.73	10,352.68	11,456.37	10,557.05	41,331.83
88	8,545.37	10,613.40	9,392.63	9,551.83	38,103.23
89	2,255.59	2,953.40	4,353.31	2,595.10	12,157.40
TOTAL	112,688.58	121,219.71	108,428.48	117,224.22	459,560.99

STATE: NEW MEXICO

ZIP CODE	QUARTER 1	QUARTER 2	QUARTER 3	QUARTER 4	TOTAL GRAMS
870	1,284.81	1,121.58	1,834.41	1,261.55	5,502.35
871	31,745.39	31,155.80	31,549.69	33,368.90	127,819.78
873	54.11	74.24	72.89	51.43	252.67
874	644.86	653.81	1,215.49	1,207.44	3,721.60
875	5,800.09	5,958.05	6,111.61	6,562.66	24,432.41
877	56.79	55.01	46.51	61.71	220.02
878	8.94	8.05	9.84	12.52	39.35
879	13.86	17.44	11.18	6.26	48.74
880	2,105.06	2,018.66	1,840.05	2,350.48	8,314.25
881	23.7	26.38	16.99	22.09	89.16
882	1,089.74	1,352.24	1,341.60	1,331.76	5,115.34
883	178.88	206.16	190.06	217.79	792.89
884	16.1	10.29	11.63	9.84	47.86
TOTAL	43,022.33	42,657.71	44,251.95	46,464.43	176,396.42

STATE: NEW YORK

ZIP CODE	QUARTER 1	QUARTER 2	QUARTER 3	QUARTER 4	TOTAL GRAMS
100	82,282.73	87,971.78	87,815.14	83,258.85	341,328.50
101	1,136.25	1,773.24	1,324.16	1,197.60	5,431.25
102	16.1	16.1	17.89	19.23	69.32
103	11,747.32	13,064.68	11,512.45	12,800.74	49,125.19
104	59,137.28	66,725.73	58,511.20	61,302	245,676.21
105	4,903.64	4,910.52	4,881.37	5,366.94	20,062.47
106	135.77	140.87	105.54	140.15	522.33
107	4,836.56	4,850.60	5,689.82	4,824.93	20,201.91
108	1,389	1,659.11	1,294.20	1,826.36	6,168.67
109	2,985.86	3,232.45	3,002.50	3,260	12,480.81
110	3,343.42	3,433.74	3,480.32	3,136.12	13,393.60
111	221.81	213.76	168.15	170.38	774.1
112	33,143.86	36,813.68	32,411.89	34,826.15	137,195.58
113	4,305.28	4,835.48	5,747.68	5,048.71	19,937.15
114	7,467.88	7,301.88	6,884.47	7,634.33	29,288.56

	115	2,774.52	4,866.97	4,646.68	4,840.15	17,128.32
	116	131.92	110.46	134.34	145.34	522.06
	117	10,452.23	10,303.40	9,204.54	10,069.87	40,030.04
	118	146.68	128.35	119.85	116.72	511.6
	119	2,556.82	3,292.73	2,526.68	2,549.04	10,925.27
	120	1,408.23	1,193.58	1,697.66	1,333.10	5,632.57
	121	435.13	548.27	502.65	630.1	2,116.15
	122	5,775.95	5,665.49	6,812.82	6,934.37	25,188.63
	123	433.78	352.84	239.25	372.07	1,397.94
	124	3,436.64	3,174.94	3,673.21	2,259.25	12,544.04
	125	4,387.03	5,295.74	5,608.78	6,331.46	21,623.01
	126	2,093.79	2,123.57	2,404.15	3,087.56	9,709.07
	127	435.13	435.13	434.23	408.92	1,713.41
	128	703.62	710.69	740.12	705.32	2,859.75
	129	838.05	984.73	1,015.86	1,326.93	4,165.57
	130	1,675.21	1,715.46	1,739.16	1,819.48	6,949.31
	131	273.24	270.11	287.1	494.16	1,324.61
	132	6,109.65	6,233.79	8,135.02	7,709.73	28,188.19
	133	227.89	223.6	142.21	214.75	808.45
	134	1,052.98	690.03	1,103.69	1,463.24	4,309.94
	135	176.82	171.28	173.16	149.72	670.98
	136	629.21	722.68	716.41	712.03	2,780.33
	137	219.58	207.05	226.28	229.86	882.77
	138	587.26	525.55	538.88	512.04	2,163.73
	139	1,116.21	1,476.65	1,182.40	1,271.84	5,047.10
	140	1,494.01	1,444.72	1,420.49	1,242.86	5,602.08
	141	631.63	594.42	560.88	490.13	2,277.06
	142	13,062.44	16,605.94	14,265.98	15,300.86	59,235.22
	143	1,456.62	1,481.04	1,465.03	1,477.01	5,879.70
	144	682.87	675.36	663.82	660.87	2,682.92
	145	394.61	391.75	384.77	397.56	1,568.69
	146	6,732.95	4,891.74	6,679.56	6,709.25	25,013.50
	147	604.97	544.78	537.62	529.13	2,216.50
	148	778.49	739.49	761.94	712.03	2,991.95
	149	265.19	284.42	280.39	244.62	1,074.62
TOTAL		291,234.11	316,020.37	303,872.39	308,263.86	1,219,390.73

RETAIL DRUG DISTRIBUTION BY ZIP CODE WITHIN STATE BY GRAMS WT

STATE: NORTH CAROLINA

ZIP CODE	QUARTER 1	QUARTER 2	QUARTER 3	QUARTER 4	TOTAL GRAMS
270	606.4	591.65	571.52	541.11	2,310.68
271	4,072.20	4,095.90	4,352.15	4,113.79	16,634.04
272	6,979.45	7,134.60	7,140.42	7,505.36	28,759.83
273	2,828.09	4,493.02	4,134.36	2,644.74	14,100.21
274	6,268.58	8,416.75	7,823.32	4,967.94	27,476.59
275	6,675.65	7,600.68	7,559.38	7,616.38	29,452.09
276	5,271.06	5,475.96	5,078.31	5,090.12	20,915.45
277	6,643.78	6,878.38	7,777.34	6,768.37	28,067.87
278	5,860.20	6,024.23	5,173.21	6,336.82	23,394.46
279	575.99	854.6	864.44	500.42	2,795.45
280	9,638.05	8,865.38	8,146.46	8,676.22	35,326.11
281	5,853.58	6,846.63	6,371.62	6,552.46	25,624.29
282	7,126.94	6,683.23	7,731.73	7,159.58	28,701.48
283	10,672.52	10,303	11,277.94	10,675.69	42,929.15
284	6,273.67	5,852.41	5,963.77	6,590.35	24,680.20
285	5,494.93	5,188.86	5,445.64	5,363.45	21,492.88
286	14,935.94	13,120.40	13,062.98	14,005.95	55,125.27
287	9,438.25	10,075.86	8,707.88	8,562.18	36,784.17
288	13,967.67	15,416.33	14,575.14	14,849.28	58,808.42
289	251.77	703.27	819.27	1,343.39	3,117.70
TOTAL	129,434.72	134,621.14	132,576.88	129,863.60	526,496.34

STATE: NORTH DAKOTA

ZIP CODE	QUARTER 1	QUARTER 2	QUARTER 3	QUARTER 4	TOTAL GRAMS
580	63.95	77.19	76.83	65.74	283.71
581	246.59	507.84	930.98	1,092.06	2,777.47
582	219.13	249.09	179.15	197.57	844.94
583	36.67	32.2	40.25	31.3	140.42
584	38.91	38.46	28.17	25.49	131.03
585	535.48	232.01	510.7	532.62	1,810.81
586	43.38	47.85	33.99	28.17	153.39
587	341.66	620.53	328.42	691.37	1,981.98
588	64.4	66.19	64.4	53.66	248.65

TOTAL		1,590.17	1,871.36	2,192.89	2,717.98	8,372.40

STATE:		OHIO				
ZIP CODE		QUARTER 1	QUARTER 2	QUARTER 3	QUARTER 4	TOTAL GRAMS
	430	1,663.85	1,616.99	1,520.30	1,424.96	6,226.10
	431	917.65	899.05	862.02	738.33	3,417.05
	432	16,054.08	14,802.60	15,143.41	14,643.11	60,643.20
	433	696.65	670.98	620.45	634.4	2,622.48
	434	304.54	289.34	287.55	270.56	1,151.99
	435	419.03	341.66	279.14	243.9	1,283.73
	436	4,114.60	4,894.87	5,330.80	4,688.53	19,028.80
	437	400.24	372.16	343.63	345.95	1,461.98
	438	113.68	78.26	86.76	96.15	374.85
	439	518.57	497.82	474.93	423.95	1,915.27
	440	1,616	1,561.44	1,549.10	1,412.26	6,138.80
	441	10,599.71	9,463.83	9,100.70	8,217.84	37,382.08
	442	919.89	940.01	764.98	775.36	3,400.24
	443	4,254.30	4,269.24	4,228.19	4,491.59	17,243.32
	444	1,629.60	1,377.38	1,486.94	1,512.88	6,006.80
	445	4,183.29	4,076.94	4,021.67	3,945.74	16,227.64
	446	620.09	607.48	639.59	596.12	2,463.28
	447	1,824.31	1,880.12	1,789.96	1,796.85	7,291.24
	448	777.41	750.94	739.13	684.39	2,951.87
	449	171.99	141.32	144.45	115.38	573.14
	450	3,200.61	3,560.25	3,428.24	3,419.55	13,608.65
	451	852.81	823.65	808.98	834.48	3,319.92
	452	13,487.78	12,911.02	11,859.89	12,214.52	50,473.21
	453	930.53	867.12	840.83	765.16	3,403.64
	454	5,156.48	4,774.49	4,351.43	4,673.24	18,955.64
	455	152.05	140.87	152.05	147.22	592.19
	456	854.87	862.56	799.59	802.19	3,319.21
	457	449.79	436.02	374.31	406.95	1,667.07
	458	1,285.25	868.91	810.24	1,205.47	4,169.87
TOTAL		78,169.65	74,777.32	72,839.26	71,527.03	297,313.26

DATE RANGE: 01/01/2017 TO 12/31/2017 | ARCOS 3 - REPORT 1 | Run Date: 07/03/2018

RETAIL DRUG DISTRIBUTION BY ZIP CODE WITHIN STATE BY GRAMS WT

STATE: OKLAHOMA

ZIP CODE	QUARTER 1	QUARTER 2	QUARTER 3	QUARTER 4	TOTAL GRAMS
730	1,266.92	1,177.12	1,142.69	1,007.72	4,594.45
731	10,146.52	13,001.98	11,001.57	10,561.70	44,711.77
734	2,655.12	2,945.80	2,196.29	1,900.06	9,697.27
735	1,315.66	1,690.42	1,124.71	1,546.86	5,677.65
736	58.58	67.53	66.19	73.79	266.09
737	153.75	150.26	154.28	147.13	605.42
738	81.84	79.6	56.35	53.22	271.01
739	21.91	32.65	14.31	18.34	87.21
740	2,871.02	3,113.05	2,649.66	2,763.70	11,397.43
741	12,567.30	12,631.16	11,594.55	13,129.17	49,922.18
743	1,334.89	1,304.93	766.5	1,303.14	4,709.46
744	758	687.79	656.04	621.61	2,723.44
745	260.27	512.49	233.44	451.22	1,457.42
746	208.57	743.69	738.77	742.35	2,433.38
747	292.47	268.32	191.4	192.56	944.75
748	330.48	321.09	264.74	270.56	1,186.87
749	3,829.37	4,486.76	3,768.55	4,234.98	16,319.66
TOTAL	38,152.67	43,214.64	36,620.04	39,018.11	157,005.46

STATE: OREGON

ZIP CODE	QUARTER 1	QUARTER 2	QUARTER 3	QUARTER 4	TOTAL GRAMS
970	2,637.76	2,422.75	2,278.22	2,215.88	9,554.61
971	1,548.39	1,520.75	1,279.35	1,255.74	5,604.23
972	34,531.08	35,210.05	34,679.53	33,844.31	138,264.97
973	8,679.35	9,242.18	8,896.06	8,956.16	35,773.75
974	7,145.63	7,043.22	7,523.69	6,408.20	28,120.74
975	6,625.72	7,101.09	6,410.79	6,658.54	26,796.14
976	324.67	270.56	296.05	270.56	1,161.84
977	1,522.09	1,359.31	1,323.18	1,624.41	5,828.99
978	570.18	487.98	490.4	470.01	2,018.57
979	148.47	167.16	169.67	146.68	631.98
TOTAL	63,733.34	64,825.05	63,346.94	61,850.49	253,755.82

RETAIL DRUG DISTRIBUTION BY ZIP CODE WITHIN STATE BY GRAMS WT

STATE: PENNSYLVANIA

ZIP CODE	QUARTER 1	QUARTER 2	QUARTER 3	QUARTER 4	TOTAL GRAMS
150	3,229.68	3,785.01	2,958.59	4,212.27	14,185.55
151	623.84	595.31	559.27	461.78	2,240.20
152	22,096.69	22,753.89	20,935.67	22,610.88	88,397.13
153	3,883.66	4,373.88	4,142.86	4,317.27	16,717.67
154	3,144.71	2,585.98	2,632.67	2,974.33	11,337.69
155	101.78	87.2	83.18	71.55	343.71
156	8,081.17	8,734.35	7,789.33	8,529.71	33,134.56
157	1,159.23	820.16	989.21	1,033.03	4,001.63
158	148.2	139.97	156.79	148.83	593.79
159	3,189.16	3,827.23	3,870.07	3,121.37	14,007.83
160	4,408.68	5,105.50	4,208.60	4,697.21	18,419.99
161	5,211.85	7,960.16	4,706.42	7,687.64	25,566.07
162	232.99	224.05	206.61	245.69	909.34
163	2,264.08	1,641.58	2,185.02	2,136.27	8,226.95
164	114.48	113.14	122.53	83.63	433.78
165	3,346.84	3,877.05	3,748.52	3,832.06	14,804.47
166	6,683.31	6,475.01	6,157.14	5,875.85	25,191.31
167	199	207.77	220.29	161.44	788.5
168	3,171.10	3,153.21	2,879.52	3,015.02	12,218.85
169	136.31	124.32	109.56	112.25	482.44
170	6,890.54	5,896.11	8,226.19	6,533.39	27,546.23
171	3,938.49	3,984.10	4,801.14	4,791.30	17,515.03
172	560.79	553.01	520.09	500.86	2,134.75
173	1,630.49	1,535.51	1,421.20	1,934.14	6,521.34
174	3,588.33	3,544.06	3,460.34	3,372.34	13,965.07
175	511.6	470.45	425.38	365.81	1,773.24
176	6,003.03	4,193.66	5,210.51	5,040.26	20,447.46
177	3,771.68	4,607.50	3,812.38	4,538.72	16,730.28
178	1,420.66	1,394.37	1,249.92	1,288.20	5,353.15
179	178.43	183.35	178.43	175.75	715.96
180	5,353.07	5,232.69	5,270.34	950.21	16,806.31
181	2,009.45	2,458.44	2,098.98	2,079.66	8,646.53
182	196.14	195.87	187.38	197.22	776.61
183	3,384.41	3,378.60	3,371.44	3,341.48	13,475.93

	184	355.17	317.42	301.59	287.37	1,261.55
	185	1,903.91	2,743.04	2,733.38	2,265.96	9,646.29
	186	578.77	537.53	531.72	492.46	2,140.48
	187	5,935.83	5,633.83	6,224.17	11,503.86	29,297.69
	188	243.72	213.76	194.53	185.59	837.6
	189	6,099.81	5,467.91	5,927.10	6,208.92	23,703.74
	190	14,773.01	13,989.85	14,426.76	14,453.95	57,643.57
	191	36,717.74	42,567.48	37,857.65	42,779.02	159,921.89
	193	4,723.68	3,885.18	4,701.77	4,708.39	18,019.02
	194	9,209.55	6,797.53	6,108.13	6,681.17	28,796.38
	195	169.49	161.44	146.23	166.27	643.43
	196	2,527.13	2,473.91	4,927.89	3,057.60	12,986.53
TOTAL		194,101.68	199,001.37	192,976.49	203,227.98	789,307.52

STATE: PUERTO RICO

ZIP CODE		QUARTER 1	QUARTER 2	QUARTER 3	QUARTER 4	TOTAL GRAMS
	6	1,404.48	1,510.64	1,620.21	1,082.22	5,617.55
	7	5,194.32	4,495.08	5,393.05	3,605.95	18,688.40
	9	15,791.53	12,306.50	17,053.97	14,570.67	59,722.67
TOTAL		22,390.33	18,312.22	24,067.23	19,258.84	84,028.62

STATE: RHODE ISLAND

ZIP CODE		QUARTER 1	QUARTER 2	QUARTER 3	QUARTER 4	TOTAL GRAMS
	28	19,899.42	20,458.51	23,998.27	21,608.26	85,964.46
	29	20,782.46	22,638.96	21,511.54	26,211.55	91,144.51
TOTAL		40,681.88	43,097.47	45,509.81	47,819.81	177,108.97

STATE: SOUTH CAROLINA

ZIP CODE		QUARTER 1	QUARTER 2	QUARTER 3	QUARTER 4	TOTAL GRAMS
	290	911.39	842.08	855.05	879.64	3,488.16
	291	3,182.72	3,184.69	3,078.79	2,064.54	11,510.74
	292	2,529.81	3,192.38	3,074.77	2,494.57	11,291.53
	293	5,214.17	5,051.03	5,090.21	5,842.85	21,198.26

294	6,824.81	6,779.73	8,225.80	9,373.22	31,203.56
295	18,339.14	16,621.53	14,123.56	16,216.72	65,300.95
296	12,477.60	13,115.39	13,519.84	12,143.36	51,256.19
297	4,573.96	4,558.76	5,574.80	4,300.19	19,007.71
298	2,697.96	1,597.85	1,702.04	1,017.38	7,015.23
299	932.86	1,033.03	1,043.76	1,096.09	4,105.74
TOTAL	57,684.42	55,976.47	56,288.62	55,428.56	225,378.07

STATE: SOUTH DAKOTA

ZIP CODE	QUARTER 1	QUARTER 2	QUARTER 3	QUARTER 4	TOTAL GRAMS
570	433.78	427.17	388.8	395.68	1,645.43
571	1,283.46	1,184.63	1,331.67	1,242.59	5,042.35
572	37.12	34.88	35.78	22.81	130.59
573	105.99	99.73	85.42	85.86	377
574	31.3	30.86	36.67	23.7	122.53
575	30.86	29.07	34.43	21.91	116.27
576	8.94	9.84	12.07	11.63	42.48
577	333.52	330.93	306.87	309.91	1,281.23
TOTAL	2,264.97	2,147.11	2,231.71	2,114.09	8,757.88

STATE: TENNESSEE

ZIP CODE	QUARTER 1	QUARTER 2	QUARTER 3	QUARTER 4	TOTAL GRAMS
370	1,763.22	1,622.26	1,530.94	1,403.85	6,320.27
371	1,403.13	1,291.60	1,232.13	1,129.36	5,056.22
372	4,866.79	4,737.61	5,553.63	4,816.63	19,974.66
373	1,115.41	1,092.33	983.84	891.63	4,083.21
374	8,530.07	8,159.43	7,164.23	7,428.80	31,282.53
376	695.66	722.32	644.42	1,000.83	3,063.23
377	192.74	209.74	190.06	167.25	759.79
378	231.11	249.09	249.98	216.44	946.62
379	10,298.70	11,238.49	9,120.38	10,774.39	41,431.96
380	3,009.66	4,019.61	2,615.85	3,502.47	13,147.59
381	9,782.77	8,704.66	9,697.35	8,917.62	37,102.40
382	3,077.18	2,844.64	3,729.20	2,639.82	12,290.84
383	2,561.29	2,792.32	3,322.16	4,114.51	12,790.28

	384	1,485.78	1,063.26	1,460.02	1,035	5,044.06
	385	312.15	295.15	206.16	196.32	1,009.78
TOTAL		49,325.66	49,042.51	47,700.35	48,234.92	194,303.44

STATE: TEXAS

ZIP CODE		QUARTER 1	QUARTER 2	QUARTER 3	QUARTER 4	TOTAL GRAMS
	750	9,240.23	8,408.25	6,388.43	7,480.05	31,516.96
	751	1,213.70	1,191.34	1,149.30	947.62	4,501.96
	752	9,448.98	9,213.22	9,084.02	8,581.10	36,327.32
	753	11.54	22.99	29.07	12.52	76.12
	754	1,735.58	1,472.18	1,103.51	1,093.85	5,405.12
	755	300.52	321.98	338.98	265.19	1,226.67
	756	2,873.08	4,099.30	3,472.96	3,280.12	13,725.46
	757	5,225	4,975.28	5,563.70	4,446.06	20,210.04
	758	263.04	248.64	267.43	245.07	1,024.18
	759	1,421.83	1,558.67	1,351.98	1,980.20	6,312.68
	760	4,728.96	3,229.95	2,426.15	3,826.96	14,212.02
	761	9,836.43	11,236.17	11,454.58	9,929.09	42,456.27
	762	2,153	2,565.14	2,052.20	2,415.77	9,186.11
	763	711.94	665.17	655.15	691.55	2,723.81
	764	245.16	239.61	241.49	237.02	963.28
	765	994.3	934.65	492.9	874.54	3,296.39
	766	265.19	226.73	198.56	202.58	893.06
	767	2,286.09	2,258.36	2,251.20	1,622.89	8,418.54
	768	319.3	349.8	290.68	302.4	1,262.18
	769	1,439.27	1,045.64	1,047.88	1,055.75	4,588.54
	770	13,625.52	13,699.35	13,062.99	15,412.26	55,800.12
	772	429.31	429.31	429.31	429.31	1,717.24
	773	4,330.15	3,338.96	4,896.69	3,713.49	16,279.29
	774	1,106.46	1,087.14	987.6	905.67	4,086.87
	775	4,705.17	4,938.79	5,779.97	4,425.40	19,849.33
	776	999.49	1,578.17	980.44	2,238.15	5,796.25
	777	228.07	778.58	1,088.75	1,364.68	3,460.08
	778	243.55	233.98	268.32	242.2	988.05
	779	1,646.14	825.26	1,199.84	1,579.06	5,250.30

	780	2,065.53	2,069.91	1,992.72	3,030.50	9,158.66
	781	1,951.40	2,124.74	1,975.10	1,788.35	7,839.59
	782	27,039.05	31,506.04	26,557.87	29,896.48	114,999.44
	783	353.74	348.82	358.65	274.13	1,335.34
	784	5,459.86	7,262.08	6,541.55	6,411.95	25,675.44
	785	1,865.72	1,278.99	1,853.20	1,637.38	6,635.29
	786	2,702.07	2,027.07	1,755.35	1,809.10	8,293.59
	787	7,505.54	8,483.65	6,646.47	8,047.72	30,683.38
	788	107.33	98.38	80.85	68.42	354.98
	789	50.98	47.85	52.77	50.53	202.13
	790	277.26	243.28	267.87	241.04	1,029.45
	791	1,362.53	1,240.09	942.25	1,301.17	4,846.04
	792	46.96	51.88	41.14	38.91	178.89
	793	143.55	145.34	139.53	104.2	532.62
	794	784.75	1,946.04	1,870.55	1,940.67	6,542.01
	795	197.22	177.54	201.24	160.54	736.54
	796	548.71	518.75	462.94	428.86	1,959.26
	797	2,409.07	2,390.28	1,954.35	2,372.75	9,126.45
	798	17.89	6.26	8.94	7.16	40.25
	799	2,520.69	2,753.95	2,393.68	2,626.50	10,294.82
TOTAL		139,436.85	145,893.55	134,651.10	142,036.91	562,018.41

STATE: UTAH

ZIP CODE		QUARTER 1	QUARTER 2	QUARTER 3	QUARTER 4	TOTAL GRAMS
	840	7,583.08	7,605.53	7,248.40	7,816.43	30,253.44
	841	11,550.46	8,802.15	14,723.17	11,021.60	46,097.38
	843	314.38	359.55	331.82	295.15	1,300.90
	844	2,604.94	2,821.38	2,499.40	2,510.40	10,436.12
	845	205.71	229.59	168.59	200.26	804.15
	846	1,977.70	1,409.57	1,346.34	1,838.98	6,572.59
	847	2,647.25	2,640.81	2,218.29	2,715.49	10,221.84
TOTAL		26,883.52	23,868.58	28,536.01	26,398.31	105,686.42

STATE: VERMONT

ZIP CODE		QUARTER 1	QUARTER 2	QUARTER 3	QUARTER 4	TOTAL GRAMS
	050	508.47	534.4	447.65	474.48	1,965
	051	281.29	254.46	242.83	232.99	1,011.57
	052	163.68	144	166.36	152.5	626.54
	053	2,069.64	2,080.37	1,765.10	2,036.55	7,951.66
	054	7,153.86	5,220.17	7,567.52	6,688.32	26,629.87
	056	2,217.22	2,151.03	2,403.25	2,255.23	9,026.73
	057	2,299.59	2,324.99	3,535.38	2,478.92	10,638.88
	058	4,158.60	4,342.49	4,038.04	3,609.89	16,149.02
TOTAL		18,852.35	17,051.91	20,166.13	17,928.88	73,999.27

STATE: VIRGIN ISLANDS

ZIP CODE		QUARTER 1	QUARTER 2	QUARTER 3	QUARTER 4	TOTAL GRAMS
	008	28.17	386.38	23.25	19.68	457.48
TOTAL		28.17	386.38	23.25	19.68	457.48

STATE: VIRGINIA

ZIP CODE		QUARTER 1	QUARTER 2	QUARTER 3	QUARTER 4	TOTAL GRAMS
	201	2,783.28	2,753.05	3,045.34	3,045.97	11,627.64
	220	1,771.81	1,704.37	1,534.07	1,924.39	6,934.64
	221	504.89	502.21	443.98	491.2	1,942.28
	222	114.93	135.5	116.09	94.18	460.7
	223	708.72	661.86	663.38	650.68	2,684.64
	224	4,187.40	4,086.87	4,422.18	3,461.78	16,158.23
	225	217.79	186.48	167.25	164.66	736.18
	226	3,863.45	4,289.27	4,517.97	4,992.72	17,663.41
	227	231.2	222.71	183.8	154.28	791.99
	228	2,416.85	2,367.92	4,059.59	2,311.76	11,156.12
	229	6,379.84	4,146.53	3,964.92	3,969.53	18,460.82
	230	2,377.76	2,006.59	2,907.96	3,089.70	10,382.01
	231	1,665.28	1,462.97	1,179.45	1,229.62	5,537.32
	232	16,984.03	16,757.30	13,568.32	18,476.60	65,786.25
	233	3,587.26	3,137.38	3,578.58	2,749.48	13,052.70
	234	4,746.04	5,230.54	5,397.97	5,930.14	21,304.69

	235	2,087.23	1,485.95	1,462.05	1,972.24	7,007.47
	236	5,386.88	5,212.21	5,778.72	4,899.88	21,277.69
	237	857.01	875.62	882.77	870.88	3,486.28
	238	476.72	656.49	1,510.28	1,720.38	4,363.87
	239	148.47	126.56	134.16	169.49	578.68
	240	4,793.27	4,350.36	3,785.15	4,756.55	17,685.33
	241	1,130.07	1,035.89	989.74	953.79	4,109.49
	242	665.08	625.9	663.29	648.71	2,602.98
	243	4,840.22	4,403.04	4,932.26	5,056.94	19,232.46
	244	500.42	464.19	427.97	611.14	2,003.72
	245	4,025.43	3,680.10	3,999.49	4,157.80	15,862.82
	246	4,214.41	2,834.98	4,288.92	2,844.19	14,182.50
TOTAL		81,665.74	75,402.84	78,605.65	81,398.68	317,072.91

STATE: WASHINGTON

ZIP CODE		QUARTER 1	QUARTER 2	QUARTER 3	QUARTER 4	TOTAL GRAMS
	980	14,454.58	14,739.35	17,563.87	16,352.40	63,110.20
	981	13,608.21	18,574.81	21,134.85	25,951.37	79,269.24
	982	15,833.03	15,361.14	15,253.90	15,147.65	61,595.72
	983	4,037.41	3,907.81	3,645.57	3,422.69	15,013.48
	984	12,160.35	12,338.25	11,868.60	12,559.79	48,926.99
	985	1,308.06	3,920.33	8,466.75	10,398.47	24,093.61
	986	7,238.47	8,043.16	8,188.77	7,375.13	30,845.53
	988	793.69	739.4	687.61	664.18	2,884.88
	989	1,928.86	1,964.55	2,198.79	2,153.72	8,245.92
	990	319.75	316.53	323.33	272.79	1,232.40
	991	624.65	637.71	622.41	563.11	2,447.88
	992	11,189.12	11,513.16	12,848.59	12,161.60	47,712.47
	993	1,353.50	1,121.94	986.88	925.26	4,387.58
	994	286.66	299.62	246.68	241.76	1,074.72
TOTAL		85,136.34	93,477.76	104,036.60	108,189.92	390,840.62

STATE: WEST VIRGINIA

ZIP CODE		QUARTER 1	QUARTER 2	QUARTER 3	QUARTER 4	TOTAL GRAMS
	247	70.21	64.84	57.24	46.06	238.35

	248	21.47	25.49	18.34	18.34	83.64
	249	48.74	50.98	45.61	36.67	182
	250	34.88	29.52	28.62	24.15	117.17
	251	58.58	56.35	67.53	27.28	209.74
	252	50.53	50.53	49.64	51.43	202.13
	253	2,309.25	1,947.20	2,109.89	2,280.72	8,647.06
	254	4,140.45	3,946.54	3,754.24	3,368.31	15,209.54
	255	92.39	97.49	100.17	90.78	380.83
	256	2,697.51	2,797.68	2,692.59	2,701.09	10,888.87
	257	4,512.61	5,300.57	4,775.29	4,794.70	19,383.17
	258	3,257.40	3,448.81	3,259.19	3,042.75	13,008.15
	259	76.02	54.56	46.51	34.43	211.52
	260	1,321.48	1,352.78	819.63	1,694.26	5,188.15
	261	1,177.03	1,222.20	1,321.92	1,116.21	4,837.36
	262	135.5	117.17	109.56	101.96	464.19
	263	1,558.04	1,104.14	1,624.95	1,110.84	5,397.97
	264	84.52	72.89	67.08	70.66	295.15
	265	1,619.94	1,553.93	1,826.01	1,748.64	6,748.52
	266	17.89	17.89	18.78	13.86	68.42
	267	175.75	131.92	139.53	109.56	556.76
	268	75.13	65.11	70.66	40.7	251.6
TOTAL		23,535.32	23,508.59	23,002.98	22,523.40	92,570.29

STATE:	WISCONSIN					
ZIP CODE		QUARTER 1	QUARTER 2	QUARTER 3	QUARTER 4	TOTAL GRAMS
	530	3,757.64	4,025.96	3,600.94	3,579.30	14,963.84
	531	5,003.99	5,144.59	4,745.69	5,253.44	20,147.71
	532	15,835.35	17,381.77	16,239.40	14,491.16	63,947.68
	534	3,442.10	3,307.04	3,386.82	3,288.89	13,424.85
	535	3,763.01	4,627.63	3,428.41	4,242.23	16,061.28
	537	9,730.98	9,821.85	10,061.11	10,199.92	39,813.86
	538	125.22	118.96	124.32	107.78	476.28
	539	726.7	648.89	557.66	480.29	2,413.54
	540	252.49	230.67	228.88	228.07	940.11
	541	260.27	248.2	256.25	212.6	977.32
	542	113.59	105.54	93.46	92.57	405.16

	543	4,549.37	4,412.43	4,456.88	4,197.96	17,616.64
	544	2,599.84	2,876.75	2,470.24	2,508.79	10,455.62
	545	77.81	50.98	54.56	43.2	226.55
	546	3,541.20	4,321.20	3,415.80	3,374.30	14,652.50
	547	2,265.34	2,604.67	2,124.47	2,824.87	9,819.35
	548	375.65	342.11	379.23	354.09	1,451.08
	549	9,293.35	8,601.27	7,907.66	8,551.81	34,354.09
TOTAL		65,713.90	68,870.51	63,531.78	64,031.27	262,147.46

STATE: WYOMING

ZIP CODE		QUARTER 1	QUARTER 2	QUARTER 3	QUARTER 4	TOTAL GRAMS
	820	259.29	225.39	208.84	167.7	861.22
	822	44.72	45.61	45.61	52.77	188.71
	823	16.99	14.31	13.42	21.47	66.19
	824	85.42	55.9	66.63	74.24	282.19
	825	117.7	129.51	116.99	129.69	493.89
	826	267.43	264.3	277.71	257.14	1,066.58
	827	45.17	40.7	41.14	42.04	169.05
	828	88.99	84.07	84.07	80.5	337.63
	829	244.62	229.86	250.43	235.67	960.58
	830	52.77	48.3	50.09	51.43	202.59
	831	54.56	26.83	27.73	18.78	127.9
TOTAL		1,277.66	1,164.78	1,182.66	1,131.43	4,756.53

DRUG CODE: 9300 DRUG NAME: MORPHINE

STATE: ALABAMA

ZIP CODE		QUARTER 1	QUARTER 2	QUARTER 3	QUARTER 4	TOTAL GRAMS
	350	8,176.02	7,756.76	7,988.34	7,393.18	31,314.30
	351	4,093.96	3,918.21	4,133.97	4,200.48	16,346.62
	352	7,176.48	6,685.96	6,572.60	6,260.92	26,695.96
	354	4,504.70	4,581.33	4,271.30	3,951.20	17,308.53
	355	2,582.03	2,463.72	2,303.07	2,134.03	9,482.85
	356	5,221.12	4,801.92	4,664.44	4,519.04	19,206.52
	357	2,754.65	2,885.37	2,435.41	2,431.54	10,506.97

	358	4,408	4,470.34	4,199	4,022.14	17,099.48
	359	6,089.01	5,608.58	5,499.96	5,536.26	22,733.81
	360	4,154.50	3,917.14	3,455.85	3,376.01	14,903.50
	361	3,543.60	3,265.73	2,653.15	2,786.78	12,249.26
	362	2,542.25	2,334.49	2,436.61	2,376.90	9,690.25
	363	2,221.85	2,151.11	2,189.08	2,047.10	8,609.14
	364	1,129.08	1,163.94	952.62	1,168.07	4,413.71
	365	6,325.72	5,819.16	5,452.36	5,102.54	22,699.78
	366	5,092.95	4,961.25	4,416.34	4,490.30	18,960.84
	367	1,107.13	1,000.64	848.24	860.16	3,816.17
	368	4,049.21	3,808.42	3,651.97	3,715.91	15,225.51
	369	213.48	157.74	218.29	214.12	803.63
TOTAL		75,385.74	71,751.81	68,342.60	66,586.68	282,066.83

STATE: ALASKA

ZIP CODE		QUARTER 1	QUARTER 2	QUARTER 3	QUARTER 4	TOTAL GRAMS
	995	5,452.38	5,448.40	5,435.14	4,921.72	21,257.64
	996	4,216.18	4,084.01	3,852.07	4,031.78	16,184.04
	997	1,638.48	1,470.38	1,330.72	1,102.91	5,542.49
	998	466.8	469.13	432.04	410.19	1,778.16
	999	301.24	203.16	228.55	237.34	970.29
TOTAL		12,075.08	11,675.08	11,278.52	10,703.94	45,732.62

STATE: AMERICAN SAMOA

ZIP CODE		QUARTER 1	QUARTER 2	QUARTER 3	QUARTER 4	TOTAL GRAMS
	967	30.08	27.45	3.01	27.07	87.61
TOTAL		30.08	27.45	3.01	27.07	87.61

STATE: ARIZONA

ZIP CODE		QUARTER 1	QUARTER 2	QUARTER 3	QUARTER 4	TOTAL GRAMS
	850	32,152.70	29,936.31	26,364.22	24,195.34	112,648.57
	851	6,430.73	6,351.86	5,692.29	5,742.91	24,217.79
	852	37,644.31	35,032.94	32,958.76	31,202.74	136,838.75
	853	27,318.40	23,762.18	21,809.48	21,181.64	94,071.70

	855	4,663.32	3,968.82	3,518.20	3,244.25	15,394.59
	856	7,176.40	6,614.23	6,239.01	6,134.90	26,164.54
	857	38,277.62	35,929.03	35,670.81	32,631.70	142,509.16
	859	3,596.53	3,935.47	3,648.40	3,176.93	14,357.33
	860	3,573.27	3,320.93	3,396.87	2,750.79	13,041.86
	863	8,522.27	8,342.11	7,986.55	6,685.96	31,536.89
	864	10,116.14	9,236.91	9,035.32	7,900.91	36,289.28
	865	277.74	189.4	303.16	239.2	1,009.50
TOTAL		179,749.43	166,620.19	156,623.07	145,087.27	648,079.96

STATE: ARKANSAS

ZIP CODE		QUARTER 1	QUARTER 2	QUARTER 3	QUARTER 4	TOTAL GRAMS
	716	1,649.65	1,482.77	1,547.40	1,363.99	6,043.81
	717	1,603.24	1,416.31	1,555.89	1,319.54	5,894.98
	718	1,254.79	1,240.75	1,080.49	959.66	4,535.69
	719	2,989.33	2,445.90	2,706.37	2,548.21	10,689.81
	720	5,208.93	5,060.41	4,853.04	4,693.89	19,816.27
	721	6,349.29	5,942.78	5,504.03	5,367.65	23,163.75
	722	3,345.42	3,125.90	3,148.74	2,871.44	12,491.50
	723	1,251.66	1,173.63	1,196.61	1,127.67	4,749.57
	724	3,523.78	3,400.49	3,147.20	2,988.13	13,059.60
	725	2,296.26	1,936.20	1,749.51	1,664.27	7,646.24
	726	3,342.96	3,100.44	3,048.95	3,004.61	12,496.96
	727	9,346.27	8,950.26	8,820.17	8,111.52	35,228.22
	728	2,126.56	1,812.10	1,697.09	1,649.32	7,285.07
	729	4,856.61	4,557.07	4,385.78	3,989.51	17,788.97
TOTAL		49,144.75	45,645.01	44,441.27	41,659.41	180,890.44

STATE: CALIFORNIA

ZIP CODE		QUARTER 1	QUARTER 2	QUARTER 3	QUARTER 4	TOTAL GRAMS
	900	8,064.93	7,456.93	7,578.16	7,260.52	30,360.54
	902	5,463.89	5,167.52	5,231.23	5,014.27	20,876.91
	903	275.38	246.09	303.64	278.86	1,103.97
	904	1,316.64	1,434.96	1,272.36	1,183.86	5,207.82
	905	2,341.02	2,391.42	2,148.24	2,352.42	9,233.10

906	5,682.48	5,781.22	6,391.30	6,234.38	24,089.38
907	4,591.58	4,277.50	4,616.65	4,194.51	17,680.24
908	4,740.51	5,482.04	4,476.01	4,653.61	19,352.17
910	3,112.39	2,937.12	2,953.56	2,286.27	11,289.34
911	1,435.79	1,411.69	1,667.23	1,394.36	5,909.07
912	2,648.48	3,216.07	3,134.86	3,032.32	12,031.73
913	12,750.22	11,768.79	11,681.24	11,613.29	47,813.54
914	4,354.14	4,468.54	4,124.41	4,210.09	17,157.18
915	948.26	961.19	898.31	1,019.52	3,827.28
916	776.42	633.79	384.91	384.12	2,179.24
917	17,250.58	16,094.77	16,075.04	15,358.72	64,779.11
918	206.85	223.4	237.71	221.76	889.72
919	4,980.97	4,967.20	4,457.35	4,624.95	19,030.47
920	23,986.14	22,990.47	22,539.13	21,662.53	91,178.27
921	15,166.29	14,840.81	13,961.43	13,558.64	57,527.17
922	10,764.29	10,210.17	9,889.56	9,321.40	40,185.42
923	20,324.08	18,867.53	18,812.01	18,393.85	76,397.47
924	2,403.70	2,236.07	2,199.20	2,200.91	9,039.88
925	14,657.01	14,860.51	13,282.57	13,586.95	56,387.04
926	11,756.33	12,276.33	11,765.42	11,551.72	47,349.80
927	3,161.94	2,870.99	3,028.22	3,147.86	12,209.01
928	12,322.60	12,211.79	11,824.34	11,341.65	47,700.38
930	8,056.68	7,856.83	7,159.04	6,535.63	29,608.18
931	1,185.11	1,103.14	1,120.51	947.64	4,356.40
932	6,777.29	6,637.05	6,789.52	6,652.05	26,855.91
933	9,861.82	9,053.80	8,460.45	7,885.73	35,261.80
934	9,469.56	8,692.59	8,212.79	7,799.11	34,174.05
935	11,080.55	10,831.63	10,458.13	10,261.80	42,632.11
936	7,192.30	7,114.40	7,044.20	6,247.52	27,598.42
937	9,502	9,395.77	9,157.04	9,026.20	37,081.01
939	4,325.23	3,611.46	3,728.90	3,362.71	15,028.30
940	7,278.79	6,919.52	6,502.32	6,917.82	27,618.45
941	14,051.30	12,715.17	12,727.82	11,969.22	51,463.51
943	1,746.56	1,440.69	1,432.64	1,485.84	6,105.73
944	1,121.93	1,237.75	1,159.69	1,269.20	4,788.57
945	37,730.78	36,404.03	36,057.11	34,998.07	145,189.99
946	4,118.03	3,749.21	3,736.31	3,370.85	14,974.40

	947	847.68	745.71	754.33	651.06	2,998.78
	948	1,305.43	1,212.73	1,187.87	1,262.96	4,968.99
	949	6,336.77	6,467.40	6,207.75	5,667.29	24,679.21
	950	6,724.77	6,403.54	5,986.23	5,982.02	25,096.56
	951	3,460.45	3,494.10	3,585.82	3,217.53	13,757.90
	952	8,754.27	8,573.19	7,684.52	7,562.12	32,574.10
	953	22,261.14	21,614.40	21,000.20	20,131.88	85,007.62
	954	13,966.47	12,918.62	11,757.75	11,423.99	50,066.83
	955	5,524.29	5,573.32	5,082.01	5,063.23	21,242.85
	956	27,246.97	25,427.01	25,422.74	23,464.57	101,561.29
	957	3,610.91	3,029.28	3,139.89	2,880.16	12,660.24
	958	15,076.92	14,483.82	14,119.48	13,854.81	57,535.03
	959	12,776.15	12,277.54	11,209.45	11,000.07	47,263.21
	960	9,490.07	8,683.90	8,455.80	8,700.19	35,329.96
	961	1,949.28	1,841.94	1,689.03	1,752.13	7,232.38
TOTAL		468,312.41	449,794.45	435,963.43	421,426.74	1,775,497.03

STATE:	COLORADO					
ZIP CODE		QUARTER 1	QUARTER 2	QUARTER 3	QUARTER 4	TOTAL GRAMS
	800	13,710.54	13,320.65	12,436.75	12,030.89	51,498.83
	801	9,641.81	9,156.84	9,146.45	9,182.89	37,127.99
	802	16,378.95	15,303.73	14,876.84	14,639.03	61,198.55
	803	1,310.49	1,258.07	1,083.44	959.99	4,611.99
	804	3,171.81	3,001.82	3,138.51	3,056.56	12,368.70
	805	8,276.11	7,741.88	7,882.83	6,988.67	30,889.49
	806	3,524.95	3,277.01	3,239.71	3,193.04	13,234.71
	807	1,342.39	1,204.60	1,112.05	1,043.73	4,702.77
	808	1,865.41	1,467.54	1,577.65	1,522.67	6,433.27
	809	9,275.34	9,345.93	8,681.17	8,707.73	36,010.17
	810	5,040.34	4,848.88	4,684.75	4,400.90	18,974.87
	811	712.03	706.7	684.6	618.61	2,721.94
	812	1,433.25	1,521.35	1,335.37	1,213.39	5,503.36
	813	541.24	783.11	584.74	423.33	2,332.42
	814	694.78	742.08	629.3	660.6	2,726.76
	815	2,664.12	2,493.91	2,373.06	2,355.55	9,886.64
	816	1,367.34	1,297.86	1,321.13	1,251.10	5,237.43

TOTAL		80,950.90	77,471.96	74,788.35	72,248.68	305,459.89

STATE:	CONNECTICUT					
ZIP CODE		QUARTER 1	QUARTER 2	QUARTER 3	QUARTER 4	TOTAL GRAMS
	60	7,175.55	6,946.82	6,399.93	5,830	26,352.30
	61	1,856.28	1,777.63	1,833.40	1,738.59	7,205.90
	62	1,949.64	1,617.45	1,422.20	1,399.17	6,388.46
	63	2,278.86	2,123.39	2,256.59	2,029.13	8,687.97
	64	10,393.61	10,050.17	9,861.29	9,471.36	39,776.43
	65	4,139.66	3,949.74	4,385.82	4,445.68	16,920.90
	66	1,454.50	1,521.78	1,460.60	1,467.26	5,904.14
	67	3,150.09	3,197.39	3,080.14	2,965.88	12,393.50
	68	4,299.77	4,172.73	4,160.37	4,088.44	16,721.31
	69	264.49	294.41	271.35	273.76	1,104.01
TOTAL		36,962.45	35,651.51	35,131.69	33,709.27	141,454.92

STATE:	DELAWARE					
ZIP CODE		QUARTER 1	QUARTER 2	QUARTER 3	QUARTER 4	TOTAL GRAMS
	197	9,115.47	8,719.68	8,430.16	8,474.06	34,739.37
	198	4,704.89	4,847.05	4,570.01	4,371.43	18,493.38
	199	8,666.09	8,326.71	8,333.98	8,127.98	33,454.76
TOTAL		22,486.45	21,893.44	21,334.15	20,973.47	86,687.51

STATE:	DISTRICT OF COLUMBIA					
ZIP CODE		QUARTER 1	QUARTER 2	QUARTER 3	QUARTER 4	TOTAL GRAMS
	200	2,622.88	2,907.60	2,641.99	2,567.63	10,740.10
	203	16.92	32.71	40.67	54.2	144.5
	204	1,073.71	911.91	1,107.58	596.02	3,689.22
	205	0.08	1.28	0.08	0	1.44
TOTAL		3,713.59	3,853.50	3,790.32	3,217.85	14,575.26

STATE: FLORIDA

ZIP CODE	QUARTER 1	QUARTER 2	QUARTER 3	QUARTER 4	TOTAL GRAMS
320	14,035.33	13,355.13	12,834.44	12,948.34	53,173.24
321	15,771.35	15,660.74	14,624.40	15,064.51	61,121
322	23,174.29	22,283.59	22,573.71	22,009.65	90,041.24
323	4,976.97	4,880.32	4,721.91	4,823.84	19,403.04
324	4,303.71	4,168.10	3,952.38	4,314.66	16,738.85
325	9,480.47	8,748.39	8,494.18	8,097.10	34,820.14
326	9,673.65	8,880.23	8,866.09	8,627.71	36,047.68
327	21,176.86	20,303.30	19,160.51	19,829.54	80,470.21
328	11,941.18	11,926.42	11,152.19	10,591.29	45,611.08
329	19,382.72	18,127.10	18,046.06	18,023.90	73,579.78
330	22,853.30	22,171.40	20,325.91	19,233.42	84,584.03
331	8,471.72	8,118.44	7,276.74	7,570.62	31,437.52
333	17,067.60	16,599.88	17,624.11	18,076.62	69,368.21
334	29,241.82	28,773.17	27,936.34	26,532.31	112,483.64
335	16,863.73	16,239.17	16,286.04	16,484.60	65,873.54
336	28,110.58	24,175.56	26,469.11	23,630.39	102,385.64
337	27,571.06	26,453.03	25,296.06	25,465.30	104,785.45
338	9,066.62	8,460.86	7,447.48	8,270.45	33,245.41
339	23,100.09	21,721.25	19,727.18	22,329.68	86,878.20
341	6,624.12	6,431.60	6,076.97	6,366.87	25,499.56
342	22,260.45	21,674.36	20,197.79	20,671.25	84,803.85
344	14,006.83	13,667.17	13,274.87	13,531.12	54,479.99
346	28,166.53	27,711.09	27,074.68	26,272.06	109,224.36
347	10,574.55	9,887.26	9,332.71	9,788.19	39,582.71
349	14,792.57	14,581	14,633.15	14,014.32	58,021.04
TOTAL	412,688.10	394,998.56	383,405.01	382,567.74	1,573,659.41

STATE: GEORGIA

ZIP CODE	QUARTER 1	QUARTER 2	QUARTER 3	QUARTER 4	TOTAL GRAMS
300	22,767.52	22,961.67	22,508.57	22,047.89	90,285.65
301	17,029.36	15,886.85	16,213.56	15,954.40	65,084.17
302	10,890.93	10,692.96	10,806.21	10,201.52	42,591.62
303	6,568.50	6,098.39	6,124.18	6,196.11	24,987.18
304	2,764.53	2,637.19	2,580.37	2,524.73	10,506.82
305	8,627.12	8,449.56	8,179.28	8,639.70	33,895.66

	306	4,742.38	4,513.21	4,235.83	4,373.71	17,865.13
	307	3,998.90	3,615.26	3,460.35	3,561.75	14,636.26
	308	2,326.70	2,109.04	1,957.83	1,963.88	8,357.45
	309	4,792.39	4,461.01	4,477.29	3,745.24	17,475.93
	310	7,338.01	6,906.58	6,789.87	6,462.87	27,497.33
	312	2,628.18	2,816.29	2,996.10	2,652.01	11,092.58
	313	3,083.29	2,974.09	2,886.57	2,654.74	11,598.69
	314	2,636.95	2,555.65	2,548.93	2,527.42	10,268.95
	315	4,438.25	4,042.19	4,012.03	4,163.40	16,655.87
	316	3,070.51	2,950.98	2,642.47	2,886.97	11,550.93
	317	3,661.04	3,077.02	3,028.81	2,904.37	12,671.24
	318	402.49	446.79	427.87	472.45	1,749.60
	319	4,073	4,184.46	4,315.58	3,621.10	16,194.14
	398	991.14	923.35	831.56	843.8	3,589.85
TOTAL		116,831.19	112,302.54	111,023.26	108,398.06	448,555.05

STATE: GUAM

ZIP CODE		QUARTER 1	QUARTER 2	QUARTER 3	QUARTER 4	TOTAL GRAMS
	969	1,270.56	829.71	1,031	866.98	3,998.25
TOTAL		1,270.56	829.71	1,031	866.98	3,998.25

STATE: HAWAII

ZIP CODE		QUARTER 1	QUARTER 2	QUARTER 3	QUARTER 4	TOTAL GRAMS
	967	15,740.49	14,905.20	13,576.07	12,646.22	56,867.98
	968	7,669.92	7,583.10	7,611.82	7,426.18	30,291.02
TOTAL		23,410.41	22,488.30	21,187.89	20,072.40	87,159

STATE: IDAHO

ZIP CODE		QUARTER 1	QUARTER 2	QUARTER 3	QUARTER 4	TOTAL GRAMS
	832	2,995.31	2,850.85	2,656.47	2,703.77	11,206.40
	833	3,316.24	3,411.31	3,092.94	2,975.01	12,795.50
	834	2,047.67	1,883.06	1,863.59	1,695.81	7,490.13
	835	1,917.52	1,613.52	1,554.08	1,566.91	6,652.03
	836	7,807.21	7,976.24	7,502.53	7,455.93	30,741.91

	837	6,583.21	6,276.56	5,844.89	6,136.10	24,840.76
	838	5,109.48	4,544.28	4,676.68	4,469.64	18,800.08
TOTAL		29,776.64	28,555.82	27,191.18	27,003.17	112,526.81

STATE: ILLINOIS

ZIP CODE		QUARTER 1	QUARTER 2	QUARTER 3	QUARTER 4	TOTAL GRAMS
	600	18,871.30	18,261.08	18,066.47	17,949.63	73,148.48
	601	10,465.10	9,739.91	9,293.99	8,878.58	38,377.58
	602	368.79	410.77	371.34	318.77	1,469.67
	603	568.68	580.83	557.42	562.42	2,269.35
	604	11,573.92	11,404.12	10,675.79	10,415.23	44,069.06
	605	6,248.43	5,767.33	5,480.02	5,513.21	23,008.99
	606	11,263.59	10,917.92	10,065.11	10,368.86	42,615.48
	607	660.12	664.61	597.78	625.15	2,547.66
	608	981.06	1,005.11	887.16	678.68	3,552.01
	609	2,041.98	1,853.10	1,624.23	1,590.54	7,109.85
	610	3,074.26	2,906.93	2,976.76	2,998.63	11,956.58
	611	3,953.51	3,949.74	3,652.68	3,604.28	15,160.21
	612	1,441.35	1,125.94	1,192.25	1,329.83	5,089.37
	613	1,367.96	1,401.40	1,267.15	1,206.31	5,242.82
	614	996.48	938.98	1,048.74	1,023.91	4,008.11
	615	1,687.68	1,542.16	1,740.26	1,992.61	6,962.71
	616	3,562.91	3,372.01	3,169.51	2,924.29	13,028.72
	617	1,600	1,533.02	1,792.58	1,636.54	6,562.14
	618	2,737.10	2,701.42	2,775.64	2,705.63	10,919.79
	619	1,028.03	844.76	885.52	995.92	3,754.23
	620	3,894.12	3,598.65	3,573.59	3,458.76	14,525.12
	622	4,398.70	3,973.77	4,164.48	3,875.83	16,412.78
	623	1,254.82	1,284.69	1,136.71	1,033.51	4,709.73
	624	1,334.59	1,379.80	1,364.66	1,173.56	5,252.61
	625	2,273.61	2,220.74	2,036.43	2,092.32	8,623.10
	626	1,806.66	1,678.62	1,630.85	1,430.36	6,546.49
	627	1,688.83	1,613.78	1,623.92	1,592.62	6,519.15
	628	2,104.76	2,040.89	2,037.97	1,850.46	8,034.08
	629	4,763.42	4,508.25	4,395.60	4,211.30	17,878.57
TOTAL		108,011.76	103,220.33	100,084.61	98,037.74	409,354.44

STATE: INDIANA

ZIP CODE	QUARTER 1	QUARTER 2	QUARTER 3	QUARTER 4	TOTAL GRAMS
460	5,273.67	5,046.54	5,047.55	4,970.48	20,338.24
461	7,438.45	7,121.69	6,802.03	6,775.35	28,137.52
462	17,519.90	16,373.78	15,796.61	16,480.37	66,170.66
463	7,647.31	7,123.45	7,308.28	6,653.44	28,732.48
464	718.51	658.44	555.87	643.64	2,576.46
465	6,470.81	6,750.03	5,532.87	5,692	24,445.71
466	2,221.37	2,227.31	1,908.38	1,867.53	8,224.59
467	3,479.30	2,900.21	2,967.08	2,909.05	12,255.64
468	5,105.73	4,475.13	4,057.30	4,278.34	17,916.50
469	4,856.74	4,463.65	4,147.33	4,138.84	17,606.56
470	1,779.50	1,781.16	1,693.86	1,544.94	6,799.46
471	5,647.46	5,589.94	4,788.37	4,359.01	20,384.78
472	2,718.52	2,742.42	2,644.06	2,527.02	10,632.02
473	5,807.78	5,165.65	5,201.36	4,919.72	21,094.51
474	2,051.39	2,005.51	1,791.98	1,634.67	7,483.55
475	2,158.09	2,036.70	1,954.40	1,814.97	7,964.16
476	1,327.74	1,253.82	1,373.92	1,273.14	5,228.62
477	3,866.42	3,902.46	4,100.78	3,745.35	15,615.01
478	2,481.29	2,041.23	1,866.81	1,683.26	8,072.59
479	3,582.37	3,713.75	3,482.90	3,331.86	14,110.88
TOTAL	92,152.35	87,372.87	83,021.74	81,242.98	343,789.94

STATE: IOWA

ZIP CODE	QUARTER 1	QUARTER 2	QUARTER 3	QUARTER 4	TOTAL GRAMS
500	2,421.35	2,488.67	2,318.14	2,342.75	9,570.91
501	2,461.16	2,364.55	2,335.67	2,511.60	9,672.98
502	2,231.40	2,262.40	2,151.80	2,019.93	8,665.53
503	5,631.33	5,308.19	5,035.26	5,060.42	21,035.20
504	1,346.64	1,416.26	1,306.60	1,315.36	5,384.86
505	1,598.35	1,583.56	1,597.77	1,502.87	6,282.55
506	1,175.51	1,060.15	1,076.91	1,044.08	4,356.65
507	879.17	820.73	820.88	813.2	3,333.98

	508	432.11	410.12	517.42	439.57	1,799.22
	510	601.84	569.46	606.05	553.63	2,330.98
	511	1,023.58	958.44	1,043.30	1,030.58	4,055.90
	512	228.01	248.4	270.99	232.45	979.85
	513	414.47	392.25	404.22	372.75	1,583.69
	514	614.59	521.46	525.4	642.37	2,303.82
	515	1,316.59	1,281.42	1,225.26	1,251.77	5,075.04
	516	182.37	219.46	177.89	165.28	745
	520	1,418.47	1,485.87	1,452.30	1,569.88	5,926.52
	521	439.18	376.18	332.69	305.24	1,453.29
	522	2,165.28	2,215.08	2,110.37	2,057.67	8,548.40
	523	865.76	772.06	771.69	662.47	3,071.98
	524	1,651.97	1,600.78	1,487.94	1,419.79	6,160.48
	525	1,740.17	1,652	1,533.43	1,614.53	6,540.13
	526	1,149.15	1,190.11	1,128.21	1,117.20	4,584.67
	527	1,243.33	1,194.46	964.58	1,122.31	4,524.68
	528	1,637.48	1,534.25	1,414.15	1,402.75	5,988.63
TOTAL		34,869.26	33,926.31	32,608.92	32,570.45	133,974.94

STATE: KANSAS

ZIP CODE	QUARTER 1	QUARTER 2	QUARTER 3	QUARTER 4	TOTAL GRAMS
660	6,997.18	6,529.31	6,205.62	6,395.23	26,127.34
661	1,912.58	1,837.69	1,474.09	1,754.82	6,979.18
662	6,527.68	6,427.24	6,219.42	6,129.25	25,303.59
664	978.26	811.56	778.98	608.28	3,177.08
665	1,350.77	1,227.17	1,250.95	1,129.53	4,958.42
666	3,381.01	3,000.60	3,087.61	2,818.89	12,288.11
667	2,982.36	2,728.87	2,890.97	2,566.90	11,169.10
668	788.48	802.88	811.64	718.4	3,121.40
669	685.25	662.02	693.44	610.67	2,651.38
670	2,593.04	2,601.66	2,639.31	2,394.07	10,228.08
671	1,678.11	1,543.28	1,664.50	1,546.40	6,432.29
672	8,635.07	8,270.40	8,539.45	7,894.55	33,339.47
673	1,564.17	1,422.07	1,319.26	1,351.57	5,657.07
674	2,323.97	2,146.79	2,175.90	2,088.42	8,735.08
675	1,342.61	1,250.14	1,380.73	1,343.63	5,317.11

	676	1,018.73	1,057.51	969.86	1,178.92	4,225.02
	677	706.56	684.15	698.47	703.03	2,792.21
	678	1,005.65	938.92	848.35	877.44	3,670.36
	679	371.94	376.82	385.84	367.13	1,501.73
TOTAL		46,843.42	44,319.08	44,034.39	42,477.13	177,674.02

STATE:	KENTUCKY					
ZIP CODE	QUARTER 1	QUARTER 2	QUARTER 3		QUARTER 4	TOTAL GRAMS
400		1,728.65	1,499.23	1,367.80	1,349.19	5,944.87
401		1,296.87	1,197.98	1,062.61	1,093.36	4,650.82
402		8,509.39	8,030.36	7,287.26	7,417.66	31,244.67
403		3,268.79	3,041.99	2,700.02	2,767.60	11,778.40
404		1,554.85	1,485.23	1,433.69	1,454.06	5,927.83
405		4,185.35	3,569.69	3,775.72	3,688.60	15,219.36
406		352.01	313.43	336.32	282.88	1,284.64
407		1,198.67	1,196.24	1,142.36	1,140.53	4,677.80
408		304.58	336.52	349.53	301.75	1,292.38
409		625.57	589.48	648.18	517.93	2,381.16
410		6,059.10	5,852.09	5,756.35	5,886.01	23,553.55
411		1,394.44	1,270.22	1,216.98	1,168.05	5,049.69
412		355.44	273.33	294.25	318.52	1,241.54
413		435.12	394.36	363.19	382.68	1,575.35
414		198.3	208.19	230.92	155.09	792.5
415		579.37	550.47	482.52	448.72	2,061.08
416		311.41	307.85	386.66	313.19	1,319.11
417		1,176.96	1,252.41	898.39	1,066.47	4,394.23
418		198.33	176.12	168.24	171.63	714.32
420		3,014.78	2,674.92	2,692.52	2,880.35	11,262.57
421		3,178.15	3,263.89	2,959.36	3,032.49	12,433.89
422		1,854.51	1,462.82	1,753.74	1,395.35	6,466.42
423		2,169.34	1,977.39	1,970.33	1,806.82	7,923.88
424		2,407.14	2,245.96	2,146.14	2,175.99	8,975.23
425		732.94	753.21	583.77	684.63	2,754.55
426		598.32	543.58	510.75	471.98	2,124.63
427		2,211.09	2,127.50	2,108.81	1,950.87	8,398.27

TOTAL	49,899.47	46,594.46	44,626.41	44,322.40	185,442.74

STATE: LOUISIANA

ZIP CODE	QUARTER 1	QUARTER 2	QUARTER 3	QUARTER 4	TOTAL GRAMS
700	7,751.27	7,287.18	6,702.92	6,304.83	28,046.20
701	4,226.16	3,660.21	3,408.41	3,427.69	14,722.47
703	1,877.47	1,952.14	1,871.95	1,764.30	7,465.86
704	6,828.24	6,337.92	6,177.39	5,814.16	25,157.71
705	5,803.39	6,047.76	5,769.26	5,675.93	23,296.34
706	1,668.29	1,625.88	1,713.76	1,666.55	6,674.48
707	6,108.44	5,758.81	5,476.36	5,454.08	22,797.69
708	5,084.26	5,001.20	4,647.85	4,547.92	19,281.23
710	1,653.56	1,683.56	1,779.62	1,698.83	6,815.57
711	6,044.06	6,182.41	6,087	6,136.33	24,449.80
712	2,119.05	1,863.59	2,069.64	1,931.93	7,984.21
713	4,864.61	4,862.69	4,640.79	4,183.18	18,551.27
714	2,267.07	2,094.65	2,213	1,943.60	8,518.32
TOTAL	56,295.87	54,358	52,557.95	50,549.33	213,761.15

STATE: MAINE

ZIP CODE	QUARTER 1	QUARTER 2	QUARTER 3	QUARTER 4	TOTAL GRAMS
039	407.58	339.24	372.62	377.27	1,496.71
040	4,129.27	3,958.48	3,915.93	3,811.32	15,815
041	883.75	884.16	920.44	838.07	3,526.42
042	2,017.59	1,944.85	1,720.32	1,667.30	7,350.06
043	2,925.81	3,141.26	3,082.67	2,757.42	11,907.16
044	1,646.28	1,626.28	1,505.19	1,386.99	6,164.74
045	329.64	321.16	391.5	315.24	1,357.54
046	756.09	636.18	607.4	614.36	2,614.03
047	576.62	570.53	545.34	564.01	2,256.50
048	572.59	765.84	683.17	627.7	2,649.30
049	2,800.70	2,670.82	2,675.94	2,648.97	10,796.43
TOTAL	17,045.92	16,858.80	16,420.52	15,608.65	65,933.89

RETAIL DRUG DISTRIBUTION BY ZIP CODE WITHIN STATE BY GRAMS WT

STATE: MARYLAND

ZIP CODE	QUARTER 1	QUARTER 2	QUARTER 3	QUARTER 4	TOTAL GRAMS
206	5,538.27	5,293.99	4,840.72	4,611.28	20,284.26
207	6,865.47	6,352.22	6,374.18	5,462.38	25,054.25
208	6,480.01	6,662.92	5,419.03	5,709.06	24,271.02
209	993.89	996.72	976.44	864.68	3,831.73
210	13,778.89	13,126.19	12,152.66	11,480.73	50,538.47
211	6,016.72	5,850	5,404.14	5,417.25	22,688.11
212	20,814.18	19,802.55	18,169.17	17,911.32	76,697.22
214	1,355.99	1,131.71	996.54	910.8	4,395.04
215	2,271.84	2,126.52	1,871.49	1,840.45	8,110.30
216	2,721.37	2,550.70	2,606.07	2,407.84	10,285.98
217	8,855.82	8,117.73	7,646.18	7,615.59	32,235.32
218	2,298.52	2,302.23	2,136.50	2,047.92	8,785.17
219	4,097.89	4,217.25	3,939.16	3,712.15	15,966.45
TOTAL	82,088.86	78,530.73	72,532.28	69,991.45	303,143.32

STATE: MASSACHUSETTS

ZIP CODE	QUARTER 1	QUARTER 2	QUARTER 3	QUARTER 4	TOTAL GRAMS
10	7,995.51	7,470.58	7,113.38	7,042.93	29,622.40
11	3,107.95	3,332.28	2,828.15	2,896.29	12,164.67
12	1,276.98	1,130.84	1,127.70	1,345.17	4,880.69
13	1,561.79	1,548.07	1,391.78	1,263.10	5,764.74
14	2,528.57	2,478.50	2,408.19	2,203.42	9,618.68
15	3,303.17	3,325.90	3,242.70	3,453.36	13,325.13
16	2,324.76	2,205.89	2,070.50	2,134.37	8,735.52
17	3,764.77	3,418.37	3,262.79	3,084.94	13,530.87
18	9,896.63	9,745.45	9,051.93	9,186.31	37,880.32
19	4,309.47	3,881.81	3,887.92	3,883.44	15,962.64
20	2,492.38	2,301.10	2,373.86	2,062.97	9,230.31
21	8,018.84	7,353.47	7,195.18	6,944.31	29,511.80
22	903.69	1,062.44	660.25	1,038.14	3,664.52
23	5,384.77	5,030.93	4,554.35	4,489.87	19,459.92
24	1,318.02	1,243.74	1,221.60	1,279.29	5,062.65
25	1,009.29	1,165.59	1,115.70	1,089.40	4,379.98
26	1,737.57	1,502.01	1,575.20	1,508.39	6,323.17

	27	8,634.53	8,310.78	8,066.35	8,068.61	33,080.27
TOTAL		69,568.69	66,507.75	63,147.53	62,974.31	262,198.28

STATE: MICHIGAN

ZIP CODE	QUARTER 1	QUARTER 2	QUARTER 3	QUARTER 4	TOTAL GRAMS
480	23,053.47	22,165.51	20,520.78	19,122.98	84,862.74
481	23,096.70	22,929.40	22,076.78	20,720.12	88,823
482	13,220.06	12,065.09	11,928.30	11,187.74	48,401.19
483	18,910.41	17,848.03	16,507.76	14,940.67	68,206.87
484	7,518.49	7,071.13	7,406.61	7,054.66	29,050.89
485	4,970.76	4,729.47	4,284.49	4,259.72	18,244.44
486	14,066.62	13,294.49	12,448.68	11,782.70	51,592.49
487	6,812.49	6,944.29	6,194.67	5,851.11	25,802.56
488	10,541.11	9,727.73	9,973.22	9,318.14	39,560.20
489	4,535.84	4,497.10	4,348.07	4,014.89	17,395.90
490	17,698.16	16,601.79	16,038.33	15,084.90	65,423.18
491	1,623.49	1,614.43	1,805.05	1,660.02	6,702.99
492	7,015.92	6,877.85	6,763.37	6,338.75	26,995.89
493	3,993.35	3,795.65	3,638.41	3,541.44	14,968.85
494	10,628.61	9,982.45	9,888.84	9,935.21	40,435.11
495	8,547.98	8,861.18	8,253.62	7,513.52	33,176.30
496	9,953.22	9,488.33	9,414.17	8,981.95	37,837.67
497	8,485.85	8,386.96	8,588.93	7,731.11	33,192.85
498	4,743.67	4,471.02	4,513.80	3,784.16	17,512.65
499	1,432.61	1,336.33	1,384.36	1,231.10	5,384.40
TOTAL	200,848.81	192,688.23	185,978.24	174,054.89	753,570.17

STATE: MINNESOTA

ZIP CODE	QUARTER 1	QUARTER 2	QUARTER 3	QUARTER 4	TOTAL GRAMS
550	5,270.62	4,867.29	4,823.79	4,766.50	19,728.20
551	7,813.69	7,052.19	6,620.09	6,082.58	27,568.55
553	6,596.13	6,177.43	6,057.87	6,078.06	24,909.49
554	11,468.31	11,253.96	10,573.85	10,418.40	43,714.52
556	208.99	180.47	223.43	182.65	795.54
557	1,762.71	1,711.97	1,615.39	1,621.27	6,711.34

	558	1,633.54	1,503.66	1,583.19	1,507.17	6,227.56
	559	2,136.14	2,211.76	2,135.96	1,938.57	8,422.43
	560	2,012.08	1,774.63	1,781.57	1,611.37	7,179.65
	561	789.36	800.8	716.61	660.18	2,966.95
	562	1,145.30	1,117.59	1,089.54	1,059.77	4,412.20
	563	3,882.67	3,715.35	3,452.94	3,332.09	14,383.05
	564	1,728.36	1,672.80	1,533.53	1,513.13	6,447.82
	565	1,529.48	1,373.49	1,470.39	1,394.60	5,767.96
	566	829.05	731.88	859.34	684.26	3,104.53
	567	742.21	799.26	839.67	751.29	3,132.43
TOTAL		49,548.64	46,944.53	45,377.16	43,601.89	185,472.22

STATE: MISSISSIPPI

ZIP CODE		QUARTER 1	QUARTER 2	QUARTER 3	QUARTER 4	TOTAL GRAMS
	386	3,932.82	3,936.91	3,467.78	3,792.70	15,130.21
	387	506	478.92	408.96	376.39	1,770.27
	388	4,376.44	4,474.06	4,079.97	4,091.81	17,022.28
	389	853.69	814.2	710.42	763.59	3,141.90
	390	3,245.38	3,046.41	3,005.78	2,701.44	11,999.01
	391	5,345.69	6,127.83	6,188.03	6,801.66	24,463.21
	392	3,546.66	3,490.03	3,378.70	3,300.55	13,715.94
	393	3,769.77	3,480.25	3,198.93	2,840.69	13,289.64
	394	5,957.48	5,422.07	5,513.65	5,381.93	22,275.13
	395	6,394.19	5,866.08	5,784.95	5,993.68	24,038.90
	396	2,134.43	2,009.51	1,765.59	1,666.89	7,576.42
	397	1,271.21	1,399.25	1,341.68	1,376.32	5,388.46
TOTAL		41,333.76	40,545.52	38,844.44	39,087.65	159,811.37

STATE: MISSOURI

ZIP CODE		QUARTER 1	QUARTER 2	QUARTER 3	QUARTER 4	TOTAL GRAMS
	630	10,797.13	10,030.35	9,656.81	9,744.02	40,228.31
	631	16,737.88	16,075.05	15,566.29	15,491.72	63,870.94
	633	5,935.67	5,768.22	5,520.16	5,125.16	22,349.21
	634	565.99	616.98	660.67	642.31	2,485.95
	635	1,492.97	1,215.57	1,248.69	1,170.09	5,127.32

	636	3,359.81	3,193.73	3,336.25	3,131.54	13,021.33
	637	1,590.37	1,488.78	1,559.27	1,562.74	6,201.16
	638	1,675.25	1,482.40	1,402.89	1,301.06	5,861.60
	639	1,940.90	1,731.36	1,412.12	1,271.25	6,355.63
	640	7,726.61	7,270.13	7,339.49	6,955.04	29,291.27
	641	7,581.28	6,994.76	6,557.63	6,358.16	27,491.83
	644	988.91	841.48	816.81	831.32	3,478.52
	645	1,516.99	1,437.20	1,380.24	1,307.28	5,641.71
	646	952.71	813.12	871.35	796.73	3,433.91
	647	2,781.60	2,605.08	2,336.80	2,341.79	10,065.27
	648	3,883.25	3,708.67	3,698.94	3,408.75	14,699.61
	650	1,821.90	1,793.28	1,579.74	1,708.81	6,903.73
	651	1,215.95	1,282.53	1,120.65	1,011.20	4,630.33
	652	4,430.99	4,495.69	4,635.18	4,486.52	18,048.38
	653	1,183.91	1,149.75	1,062.57	996.48	4,392.71
	654	1,531.72	1,508.33	1,519.19	1,258.36	5,817.60
	655	2,568.88	2,487.33	2,314.27	1,994.70	9,365.18
	656	4,325.66	4,367.79	4,281.52	4,082.01	17,056.98
	657	7,411.28	7,113.90	7,274.42	7,161.95	28,961.55
	658	8,505.57	7,607.24	7,603.84	7,436.90	31,153.55
TOTAL		102,523.18	97,078.72	94,755.79	91,575.89	385,933.58

STATE:	MONTANA					
ZIP CODE		QUARTER 1	QUARTER 2	QUARTER 3	QUARTER 4	TOTAL GRAMS
	590	1,285.03	1,073.50	1,195.62	1,093.99	4,648.14
	591	3,827.06	3,352.43	3,275.68	3,436.95	13,892.12
	592	666.03	564.11	543.46	534.03	2,307.63
	593	725.4	674.94	666.03	538.2	2,604.57
	594	2,702.35	2,022.27	1,904.90	1,888.46	8,517.98
	595	392.12	313.28	281.5	277.25	1,264.15
	596	2,254.05	2,197.20	2,132.72	1,993.51	8,577.48
	597	1,903.21	1,879.28	2,003.36	1,698.64	7,484.49
	598	2,834.32	2,759.30	2,571.61	2,587.41	10,752.64
	599	2,477.17	2,397.75	2,428.90	2,288.93	9,592.75
TOTAL		19,066.74	17,234.06	17,003.78	16,337.37	69,641.95

DATE RANGE: 01/01/2017 TO 12/31/2017 ARCOS 3 - REPORT 1 Run Date: 07/03/2018

RETAIL DRUG DISTRIBUTION BY ZIP CODE WITHIN STATE BY GRAMS WT

STATE: NEBRASKA

ZIP CODE	QUARTER 1	QUARTER 2	QUARTER 3	QUARTER 4	TOTAL GRAMS
680	2,035.45	2,252.57	2,279.04	2,110.03	8,677.09
681	6,799.80	6,531.56	6,561.65	6,304.67	26,197.68
683	664.06	669.78	695.68	667.59	2,697.11
684	664.82	603.01	546.3	532.51	2,346.64
685	2,918.29	2,611.28	2,729.72	2,634.62	10,893.91
686	452.75	464.19	461.03	410.91	1,788.88
687	971.9	978.91	845.42	914.97	3,711.20
688	2,121.21	1,903.50	1,808.18	1,881.88	7,714.77
689	644.38	646.63	634.29	645.71	2,571.01
690	417.41	342.52	414.27	326.53	1,500.73
691	932.52	782.67	827.31	701.62	3,244.12
692	55.68	99.22	43.66	61.18	259.74
693	1,092.51	965.23	1,092.52	939.95	4,090.21
TOTAL	19,770.78	18,851.07	18,939.07	18,132.17	75,693.09

STATE: NEVADA

ZIP CODE	QUARTER 1	QUARTER 2	QUARTER 3	QUARTER 4	TOTAL GRAMS
890	15,732.38	16,074.10	15,291.48	14,266.85	61,364.81
891	32,568.86	31,710.61	30,451.07	29,849.01	124,579.55
893	197.31	152.87	131.3	103.1	584.58
894	4,692.47	4,424.03	4,428.70	4,031.66	17,576.86
895	7,167.54	6,293.31	6,190.44	5,194.55	24,845.84
897	1,810.63	1,674.21	1,667.71	1,666.75	6,819.30
898	465.58	465.2	401.77	410.88	1,743.43
TOTAL	62,634.77	60,794.33	58,562.47	55,522.80	237,514.37

STATE: NEW HAMPSHIRE

ZIP CODE	QUARTER 1	QUARTER 2	QUARTER 3	QUARTER 4	TOTAL GRAMS
30	4,710.48	4,597.81	4,595.49	4,651.82	18,555.60
31	2,753.72	2,694.15	2,598.74	2,782.96	10,829.57
32	1,964.32	1,897.14	1,847.82	1,834.67	7,543.95
33	1,950.08	1,869.13	1,916.42	1,897.06	7,632.69

	34	1,067.14	945	930.64	998.49	3,941.27
	35	731.87	700.34	641.25	632.25	2,705.71
	36	136.86	177.47	155.06	205.85	675.24
	37	1,086.71	1,014.67	1,032.15	1,074.18	4,207.71
	38	4,181.73	3,945.97	4,184.29	4,073.73	16,385.72
TOTAL		18,582.91	17,841.68	17,901.86	18,151.01	72,477.46

STATE: NEW JERSEY

ZIP CODE		QUARTER 1	QUARTER 2	QUARTER 3	QUARTER 4	TOTAL GRAMS
	70	13,467.31	11,266.54	10,555.02	10,186.11	45,474.98
	71	1,842.55	1,612.15	1,524.95	1,624.05	6,603.70
	72	751.66	652.5	621.27	554.98	2,580.41
	73	1,037.49	1,138.23	1,056.88	905.63	4,138.23
	74	5,168.37	4,874.83	4,575.95	4,920.53	19,539.68
	75	935.17	756.21	771.92	710.45	3,173.75
	76	6,606.63	5,712.04	5,573.98	5,487.99	23,380.64
	77	6,274.32	6,072.04	5,566.72	6,047.04	23,960.12
	78	3,376.13	3,084.22	2,709.05	2,620.69	11,790.09
	79	2,424.64	1,902.40	2,028.63	2,144.07	8,499.74
	80	21,015.28	19,916.83	18,519.36	17,280.63	76,732.10
	81	1,716	1,498.08	1,531.19	1,570.48	6,315.75
	82	3,716.60	3,689.32	3,357.87	3,104.93	13,868.72
	83	2,241.25	2,088.27	2,215.21	2,090.68	8,635.41
	84	418.68	452.3	415.7	420.23	1,706.91
	85	2,678.05	2,511.50	2,162.91	1,901.41	9,253.87
	86	2,550.95	2,334.04	2,312.91	2,482.45	9,680.35
	87	5,876.81	5,895.21	5,765.23	5,431.29	22,968.54
	88	10,179.19	10,135.43	9,390.18	9,483.69	39,188.49
	89	813.7	616.19	665.8	654.73	2,750.42
TOTAL		93,090.78	86,208.33	81,320.73	79,622.06	340,241.90

STATE: NEW MEXICO

ZIP CODE		QUARTER 1	QUARTER 2	QUARTER 3	QUARTER 4	TOTAL GRAMS
	870	2,229.12	1,996.98	1,830.10	1,835.45	7,891.65
	871	13,629.86	12,466.40	11,940.91	10,612.92	48,650.09

	873	1,056.03	975.8	858.91	915.4	3,806.14
	874	1,070.26	991	931.12	834.59	3,826.97
	875	3,078.79	2,855.88	2,905.19	2,643.96	11,483.82
	877	381.84	378.75	406.6	381.78	1,548.97
	878	128.44	122.39	113.39	89.1	453.32
	879	231.93	227.59	254.25	266.64	980.41
	880	2,735.27	2,277.94	2,333.43	2,189.04	9,535.68
	881	701.29	576.16	559.9	485.36	2,322.71
	882	2,605.33	2,362.40	2,446.33	2,319.61	9,733.67
	883	630.6	663.94	669.36	555.13	2,519.03
	884	100.21	98.19	107.13	95.04	400.57
TOTAL		28,578.97	25,993.42	25,356.62	23,224.02	103,153.03

STATE:	NEW YORK					
ZIP CODE	QUARTER 1		QUARTER 2	QUARTER 3	QUARTER 4	TOTAL GRAMS
	63	0	0.03	0	0	0.03
	100	13,616.40	12,937.31	12,632.55	12,544.28	51,730.54
	101	188.7	234.46	182.13	179.27	784.56
	102	6.77	6.77	5.64	2.26	21.44
	103	6,813.91	6,466.89	5,808.94	5,624.06	24,713.80
	104	7,043	6,314.03	6,576.06	6,687.82	26,620.91
	105	2,529.11	2,605.13	2,350.82	2,669.36	10,154.42
	106	370.3	328.51	355.91	511.71	1,566.43
	107	920.63	934.45	852.32	888.52	3,595.92
	108	363.58	348.34	364.43	259.65	1,336
	109	3,649.90	3,097.10	3,095.37	3,076.06	12,918.43
	110	3,981.08	4,080.31	3,431.70	3,894.57	15,387.66
	111	853.77	871.75	741.62	722.18	3,189.32
	112	9,054.29	8,755.67	8,233.89	8,015.14	34,058.99
	113	3,631.55	3,108.64	3,105.08	2,858.86	12,704.13
	114	2,053.32	1,810.81	1,884.87	1,637.18	7,386.18
	115	4,947.47	5,152.59	5,157.24	4,971.40	20,228.70
	116	554.13	547.28	551.75	480.91	2,134.07
	117	19,670.12	18,380.83	18,036.93	17,473.38	73,561.26
	118	776.96	624.6	506.75	463.25	2,371.56
	119	1,892.93	1,830.21	1,765.74	2,115.28	7,604.16

	120	3,671.88	3,674.77	3,574.13	3,634.69	14,555.47
	121	1,867.71	1,720.02	1,670.59	1,565.73	6,824.05
	122	3,153.45	3,068.39	2,612.95	2,555.80	11,390.59
	123	1,000.98	853.08	778.08	912.33	3,544.47
	124	2,626.16	2,388.88	2,337.51	2,436.79	9,789.34
	125	3,561.40	3,482.28	3,315.74	3,503.64	13,863.06
	126	790.51	932.78	835.39	737.3	3,295.98
	127	1,841.88	1,727.38	1,587.98	1,248.25	6,405.49
	128	3,105.44	2,746.92	2,896.45	2,810.79	11,559.60
	129	2,313.39	2,297.37	2,263.51	2,280.60	9,154.87
	130	4,159.98	4,263.78	4,178.66	4,219.30	16,821.72
	131	1,445.23	1,433.03	1,309.25	1,435.04	5,622.55
	132	4,285.62	4,282.34	3,777.38	3,840.87	16,186.21
	133	1,537.57	1,571.52	1,259.73	1,260.27	5,629.09
	134	1,899.24	1,934.21	1,620.86	1,911.17	7,365.48
	135	831.49	709.92	732.49	689.3	2,963.20
	136	2,516.45	2,553.39	2,339.77	2,204.75	9,614.36
	137	1,004.89	1,033.17	908.1	1,031.43	3,977.59
	138	3,146.20	3,167.33	3,022.21	3,168.26	12,504
	139	813.71	780.36	750.6	876.08	3,220.75
	140	5,153.34	5,123.84	5,005.90	4,929.81	20,212.89
	141	2,425.34	2,364.81	2,326.08	2,206.95	9,323.18
	142	6,791.82	6,496.12	6,551.67	6,326.72	26,166.33
	143	1,060.37	919.05	862.43	871.28	3,713.13
	144	3,399.24	3,223.28	3,051.83	2,943.22	12,617.57
	145	2,881.93	2,748.70	2,617.61	2,387.12	10,635.36
	146	6,392.88	5,874.73	5,853.08	5,737.43	23,858.12
	147	2,241.06	2,172.21	2,220.39	2,157.16	8,790.82
	148	3,959.14	3,792.69	4,032.58	3,904	15,688.41
	149	866.26	823.01	798.74	907.57	3,395.58
TOTAL		163,662.48	156,595.07	150,731.43	149,768.79	620,757.77

STATE: NORTH CAROLINA

ZIP CODE		QUARTER 1	QUARTER 2	QUARTER 3	QUARTER 4	TOTAL GRAMS
	270	4,445.92	3,580.23	3,718.77	3,584.54	15,329.46
	271	4,239.95	3,675.98	3,390.51	3,205.27	14,511.71

	272	8,309.99	6,955.88	6,938.56	6,439.15	28,643.58
	273	4,665.06	4,466.54	4,227.28	4,412.05	17,770.93
	274	2,996.69	2,800.01	3,035.34	2,973.13	11,805.17
	275	11,087.16	10,742.11	10,447.90	10,586.08	42,863.25
	276	4,091.66	3,751.43	3,711.93	3,471.36	15,026.38
	277	5,664.24	6,023.69	5,302.35	5,400.88	22,391.16
	278	7,201.14	6,795.63	6,941.72	6,711.64	27,650.13
	279	2,463.33	2,432.16	2,228.05	2,110.03	9,233.57
	280	9,639.18	9,367.39	8,787.89	8,664.95	36,459.41
	281	7,938.23	7,813.64	7,435.49	7,526.41	30,713.77
	282	5,949.73	5,494.37	5,443.15	5,281.63	22,168.88
	283	9,221.91	8,468.29	8,759.74	7,673.90	34,123.84
	284	7,565.64	7,512.71	6,876.97	6,814.05	28,769.37
	285	6,953.11	6,635.54	6,517.20	6,260.39	26,366.24
	286	11,329.07	11,200.42	11,058.55	10,549.73	44,137.77
	287	8,264.10	7,784.48	7,428.80	7,127.95	30,605.33
	288	3,870.79	3,712.17	3,439.19	3,240.48	14,262.63
	289	777.78	835.19	776.33	918.15	3,307.45
TOTAL		126,674.68	120,047.86	116,465.72	112,951.77	476,140.03

STATE: NORTH DAKOTA

ZIP CODE		QUARTER 1	QUARTER 2	QUARTER 3	QUARTER 4	TOTAL GRAMS
	580	477.07	358.42	331.28	309.57	1,476.34
	581	1,520.55	1,507.54	1,422.25	1,238.30	5,688.64
	582	1,014.08	921.47	902.6	877.76	3,715.91
	583	370.16	366.55	343.72	303.02	1,383.45
	584	358.85	346.51	303.49	324.88	1,333.73
	585	2,099.74	2,046.90	1,870.14	2,098.58	8,115.36
	586	245.38	232.79	249.69	237.96	965.82
	587	354.34	402.76	284.67	475.93	1,517.70
	588	246.23	238.68	243.72	206.25	934.88
TOTAL		6,686.40	6,421.62	5,951.56	6,072.25	25,131.83

STATE: OHIO

ZIP CODE	QUARTER 1	QUARTER 2	QUARTER 3	QUARTER 4	TOTAL GRAMS
430	7,127.95	7,034.01	6,464.67	6,716.43	27,343.06
431	4,074.31	4,103.57	3,836.28	3,745.74	15,759.90
432	14,366.67	13,559.23	12,860.81	12,308.79	53,095.50
433	1,325.01	1,367.96	1,410.63	1,315.68	5,419.28
434	1,194.30	1,125.83	1,275.84	1,183.27	4,779.24
435	3,329.65	2,889.54	3,299.64	3,304.92	12,823.75
436	5,476.96	5,396.84	5,416.98	5,146.33	21,437.11
437	2,133.52	2,149.85	2,199.35	1,938.84	8,421.56
438	385.02	397.81	340.79	282.41	1,406.03
439	2,351.35	2,261.57	2,320.88	2,188.37	9,122.17
440	5,932.81	5,966.13	5,933.46	5,269.15	23,101.55
441	9,310.26	8,630.81	8,592.26	7,894.42	34,427.75
442	5,665.53	5,441.51	5,222.48	4,956.94	21,286.46
443	2,727.17	2,726.33	2,547.58	2,558.05	10,559.13
444	4,109.54	3,867.39	3,639.05	3,611.28	15,227.26
445	2,702.36	2,632.46	2,472.76	2,260.62	10,068.20
446	3,684.05	3,613.97	3,619.55	3,298.49	14,216.06
447	2,610.40	2,403.22	2,568.32	2,477.47	10,059.41
448	2,771.58	2,587.73	2,683.68	2,652.47	10,695.46
449	728.74	832.17	692	825.1	3,078.01
450	9,982.38	9,401.92	9,226.88	8,337.78	36,948.96
451	2,902.77	2,938.52	3,178.31	3,016.16	12,035.76
452	9,230.48	9,094.45	8,741.22	8,632.98	35,699.13
453	4,591.17	4,660.42	4,515.75	4,396.61	18,163.95
454	6,070.66	5,725.47	5,571.64	5,274.30	22,642.07
455	991.49	937.41	979.93	900.53	3,809.36
456	5,550.28	5,554.64	5,494.90	5,155.63	21,755.45
457	1,242.34	1,251.40	1,185.85	1,264.48	4,944.07
458	2,390.53	2,495.58	2,208.76	2,301.55	9,396.42
TOTAL	124,959.28	121,047.74	118,500.25	113,214.79	477,722.06

STATE: OKLAHOMA

ZIP CODE	QUARTER 1	QUARTER 2	QUARTER 3	QUARTER 4	TOTAL GRAMS
730	17,235.74	16,392.74	15,277.57	14,798.15	63,704.20

	731	20,942.62	20,073.42	19,314.73	18,992.60	79,323.37
	734	2,369.94	2,336.35	2,283.66	2,012.55	9,002.50
	735	6,120.95	6,196.38	5,485.66	4,977.48	22,780.47
	736	1,709.45	1,393.87	1,069.69	952.66	5,125.67
	737	1,563.66	1,536.14	1,333.26	1,320.32	5,753.38
	738	468.07	370.91	326.66	281.05	1,446.69
	739	96.33	76.83	100.8	78.15	352.11
	740	17,481.89	15,879.10	16,375.39	15,779.90	65,516.28
	741	10,575.96	9,669.08	9,872.31	9,462.33	39,579.68
	743	2,386	2,249.23	2,285.01	2,218.89	9,139.13
	744	5,871.74	6,090.54	6,158.58	5,293.87	23,414.73
	745	1,865.04	1,823.99	1,779.29	1,660.24	7,128.56
	746	1,056.75	1,142.20	1,063.91	1,016.76	4,279.62
	747	1,500.61	1,525.65	1,071.18	1,028.67	5,126.11
	748	4,044.07	4,042.30	3,492.71	3,490.35	15,069.43
	749	1,694.16	1,793.66	1,835.82	1,683.31	7,006.95
TOTAL		96,982.98	92,592.39	89,126.23	85,047.28	363,748.88

STATE: OREGON

ZIP CODE		QUARTER 1	QUARTER 2	QUARTER 3	QUARTER 4	TOTAL GRAMS
	970	12,202.73	11,303.25	11,048.23	10,343.37	44,897.58
	971	6,198.12	5,720.45	5,753.47	5,535.70	23,207.74
	972	18,411.24	17,025.82	16,205.71	15,701.90	67,344.67
	973	9,268.99	8,693.51	8,100.97	7,773.70	33,837.17
	974	15,254.40	14,575.34	13,468.40	13,048.58	56,346.72
	975	7,843.50	7,240.49	6,759.54	6,374.90	28,218.43
	976	1,462.17	1,246.94	1,255.37	1,269.92	5,234.40
	977	3,029.32	3,050.64	3,185.02	3,283.20	12,548.18
	978	2,913.91	2,749.76	2,614.22	2,390.59	10,668.48
	979	649.64	471.52	451.91	442.6	2,015.67
TOTAL		77,234.02	72,077.72	68,842.84	66,164.46	284,319.04

STATE: PENNSYLVANIA

ZIP CODE		QUARTER 1	QUARTER 2	QUARTER 3	QUARTER 4	TOTAL GRAMS
	150	12,447.91	12,439.22	12,417.48	11,547	48,851.61

151	3,125.44	3,167.01	3,189.04	3,040.39	12,521.88
152	10,318.72	9,759.32	9,282.72	9,046.01	38,406.77
153	2,456.65	2,350.31	2,516.89	2,312.55	9,636.40
154	2,381.88	2,231.03	2,140.05	2,116.70	8,869.66
155	1,493.26	1,523.54	1,649.93	1,307.77	5,974.50
156	5,557.68	5,267.46	5,533.76	5,000.46	21,359.36
157	2,926.17	2,668.65	2,892.37	2,906.21	11,393.40
158	2,095.86	1,959.68	2,012.04	1,934.76	8,002.34
159	3,035.57	2,733.82	2,871.23	2,635.10	11,275.72
160	3,271.12	3,139.04	3,130.73	3,019.85	12,560.74
161	4,549.22	4,580.96	4,451.07	4,353.97	17,935.22
162	1,519.78	1,429.26	1,309.96	1,409	5,668
163	4,816.17	4,811.19	4,516.53	4,606.46	18,750.35
164	1,517.42	1,526.53	1,554.94	1,489.84	6,088.73
165	4,608.94	4,459.91	4,343.12	3,944.21	17,356.18
166	4,835	4,711.45	4,473.73	4,219.62	18,239.80
167	2,049.26	2,121.42	2,085.60	1,877.37	8,133.65
168	1,299.57	1,269.63	1,378.73	1,175.69	5,123.62
169	816.27	790.5	709.83	740.23	3,056.83
170	12,510.90	11,770.98	11,242.12	11,365.27	46,889.27
171	2,816.79	2,796.03	2,456.36	2,411.98	10,481.16
172	2,677.39	2,510.31	2,385.96	2,556.91	10,130.57
173	4,662.26	4,565.13	4,224.05	4,358.92	17,810.36
174	3,172.78	2,755.17	2,921.02	2,751.49	11,600.46
175	3,945.12	3,754.36	3,608.10	3,559.20	14,866.78
176	3,929.77	3,308.81	3,191.43	3,431.08	13,861.09
177	2,231.90	2,116.76	2,155.51	2,069.80	8,573.97
178	3,349.48	3,513.39	3,132.20	3,130.93	13,126
179	1,593.49	1,513.49	1,410.67	1,307.48	5,825.13
180	4,593.61	4,510.79	4,332.77	4,156.21	17,593.38
181	4,506.10	4,027.10	3,626.15	3,766.94	15,926.29
182	2,409.50	2,196.24	1,848.34	2,025.79	8,479.87
183	2,861.23	2,451.06	2,481.82	2,502.85	10,296.96
184	3,218.77	3,171.34	3,141.68	2,948.85	12,480.64
185	4,056.42	3,937.81	3,635.40	3,199.90	14,829.53
186	2,746.42	2,625.93	2,589.11	2,405.34	10,366.80
187	10,656.35	9,601.92	8,829	9,043.97	38,131.24

	188	1,022.82	939.11	890.11	944.16	3,796.20
	189	4,082.87	4,164.90	4,135.58	4,118.10	16,501.45
	190	70,396.01	44,439.27	42,353.42	43,523.03	200,711.73
	191	17,181.26	16,987.51	15,949.18	14,957.02	65,074.97
	193	6,089.08	5,918.47	5,432.69	5,258.88	22,699.12
	194	9,685.86	9,400.86	9,004.22	9,110.66	37,201.60
	195	1,764.61	1,771	1,631.28	1,618.91	6,785.80
	196	2,335.52	2,210.62	2,249.47	2,189.39	8,985
TOTAL		263,618.20	229,898.29	221,317.39	217,396.25	932,230.13

STATE: PUERTO RICO

ZIP CODE		QUARTER 1	QUARTER 2	QUARTER 3	QUARTER 4	TOTAL GRAMS
	006	600.31	476.31	336.96	211.71	1,625.29
	007	554.52	498.21	379.52	323.53	1,755.78
	009	1,433.64	1,266.76	1,148.94	1,169.34	5,018.68
TOTAL		2,588.47	2,241.28	1,865.42	1,704.58	8,399.75

STATE: RHODE ISLAND

ZIP CODE		QUARTER 1	QUARTER 2	QUARTER 3	QUARTER 4	TOTAL GRAMS
	28	7,506.91	7,560.77	7,617.95	6,893.52	29,579.15
	29	4,563.02	4,129.10	4,404.60	3,952.64	17,049.36
TOTAL		12,069.93	11,689.87	12,022.55	10,846.16	46,628.51

STATE: SOUTH CAROLINA

ZIP CODE		QUARTER 1	QUARTER 2	QUARTER 3	QUARTER 4	TOTAL GRAMS
	290	5,781.25	5,351.58	5,359.14	5,369.54	21,861.51
	291	4,701.48	4,591.87	4,680.92	4,751.28	18,725.55
	292	5,377.13	5,269.33	5,036.19	4,800.47	20,483.12
	293	6,491.23	6,171.77	5,840.98	6,080.76	24,584.74
	294	13,784.97	12,639.51	12,433.86	12,494.56	51,352.90
	295	9,963.45	9,545.18	9,919.70	9,602.27	39,030.60
	296	14,805.11	13,702.48	13,156.17	13,352.99	55,016.75
	297	2,856.81	2,803.12	2,494.53	2,706.85	10,861.31
	298	2,133.54	2,073.86	2,151.44	2,122.38	8,481.22

	299	2,831.83	2,654.44	2,810.18	2,751.89	11,048.34
TOTAL		68,726.80	64,803.14	63,883.11	64,032.99	261,446.04

STATE: SOUTH DAKOTA

ZIP CODE		QUARTER 1	QUARTER 2	QUARTER 3	QUARTER 4	TOTAL GRAMS
	570	1,449.32	1,501.47	1,361.52	1,323	5,635.31
	571	3,126.60	2,988.51	2,899.56	2,880.58	11,895.25
	572	437.31	353.16	414.3	361.81	1,566.58
	573	453.07	465.36	444.03	440.08	1,802.54
	574	443.66	464.62	455.33	452.91	1,816.52
	575	529	525.5	493.96	463.79	2,012.25
	576	63.24	54.99	68.9	114.28	301.41
	577	2,602.91	2,391.35	2,538.40	2,238.54	9,771.20
TOTAL		9,105.11	8,744.96	8,676	8,274.99	34,801.06

STATE: TENNESSEE

ZIP CODE		QUARTER 1	QUARTER 2	QUARTER 3	QUARTER 4	TOTAL GRAMS
	370	19,042.14	18,276.75	17,669.22	16,258.52	71,246.63
	371	18,054.71	17,363.53	15,936.06	15,298.49	66,652.79
	372	10,484.43	9,961.24	9,402.92	9,153.50	39,002.09
	373	13,951.34	12,816.93	12,243.84	11,276.38	50,288.49
	374	4,879.80	4,693.90	4,154.51	4,180.79	17,909
	376	10,657.22	9,783.13	9,101.16	9,086.23	38,627.74
	377	8,368.06	8,063.48	8,145.82	8,135.56	32,712.92
	378	13,558.83	12,409.19	12,148.86	11,440.79	49,557.67
	379	8,748.22	7,954.01	7,607.27	7,943.75	32,253.25
	380	5,009.62	4,656.88	4,527.02	4,392.67	18,586.19
	381	47,822.96	28,703.92	31,943.81	30,975.40	139,446.09
	382	3,160.55	3,061.87	3,037.74	2,920.03	12,180.19
	383	7,963.42	7,553.60	7,305.21	6,629.42	29,451.65
	384	4,859.43	4,686.64	4,208.87	3,983.99	17,738.93
	385	9,163.37	9,097.75	8,262.96	7,845.21	34,369.29
TOTAL		185,724.10	159,082.82	155,695.27	149,520.73	650,022.92

STATE: TEXAS

ZIP CODE	QUARTER 1	QUARTER 2	QUARTER 3	QUARTER 4	TOTAL GRAMS
750	18,438.42	17,632.79	16,894.20	15,880.02	68,845.43
751	5,271.46	5,262.56	5,379.93	5,360.24	21,274.19
752	8,658.89	8,198.85	7,273.91	8,037.59	32,169.24
753	198.81	176.94	213.45	215.31	804.51
754	5,372.34	4,716.14	3,992.07	3,806.91	17,887.46
755	1,239.54	1,273.81	1,254.11	1,165.76	4,933.22
756	3,648.39	3,421.81	3,647.53	3,678.86	14,396.59
757	3,599.52	3,627.09	3,564.03	3,497.91	14,288.55
758	794.19	834.57	900.83	781.04	3,310.63
759	2,337.04	2,598.65	2,416.81	2,501.03	9,853.53
760	12,376.69	11,809.52	11,944.51	11,770.14	47,900.86
761	13,402.45	12,224.12	13,084.61	12,445.30	51,156.48
762	4,357.05	4,559.79	4,459.36	4,106.58	17,482.78
763	3,094.83	2,921.43	2,798.09	2,520.83	11,335.18
764	1,449.35	1,383.95	1,365.26	1,280.61	5,479.17
765	4,442.74	3,875.35	4,003.10	3,589.63	15,910.82
766	969.91	921.17	1,103.68	1,088.84	4,083.60
767	3,165.27	3,467.78	3,184.68	3,275.90	13,093.63
768	1,311.91	1,319.40	1,063.54	1,139.24	4,834.09
769	1,610.36	1,506.23	1,402.57	1,289.82	5,808.98
770	13,028.10	13,321.46	12,527.10	12,169.05	51,045.71
773	8,567.87	8,121.93	8,343.41	8,388.02	33,421.23
774	6,085.44	5,876.38	5,686.82	5,922	23,570.64
775	11,122.52	11,470.50	10,627.64	11,430.16	44,650.82
776	2,358.84	2,548.90	2,438.02	2,397.92	9,743.68
777	1,489.22	1,437.27	1,391.01	1,740.75	6,058.25
778	2,145.91	2,040.09	2,093.93	2,069.15	8,349.08
779	2,455.36	2,504.12	2,233.39	2,198.50	9,391.37
780	2,745.48	2,682.71	2,639.58	2,528.15	10,595.92
781	3,263.53	2,823.53	3,040.12	2,860.98	11,988.16
782	16,672.17	15,903.67	15,981.30	15,531.88	64,089.02
783	1,893.27	1,740.40	1,699.21	1,362.73	6,695.61
784	3,274.29	2,934.40	2,782.92	2,963.56	11,955.17
785	2,720.24	2,580	2,628.88	2,655.81	10,584.93

	786	10,021.53	9,608.55	9,233.39	8,787.84	37,651.31
	787	8,910.53	8,722.53	8,055.20	7,995.02	33,683.28
	788	595.41	557.93	507.19	471.52	2,132.05
	789	545.36	506.26	463.58	510.05	2,025.25
	790	1,670.73	1,552.88	1,586.14	1,348.76	6,158.51
	791	4,620.76	4,228.31	4,400.60	3,607.73	16,857.40
	792	329.86	318.1	334.05	248.48	1,230.49
	793	855.48	730.43	822.5	740.81	3,149.22
	794	3,312.32	3,286.38	3,436.28	3,347.32	13,382.30
	795	845.21	814.31	673.09	681.57	3,014.18
	796	2,214.53	2,252.70	2,073.09	2,019.92	8,560.24
	797	3,099.53	3,075.76	2,960.56	2,838.74	11,974.59
	798	85.2	99.99	86	106.11	377.3
	799	3,964.11	3,472.91	3,254.65	3,261.09	13,952.76
TOTAL		214,631.96	206,944.35	201,945.92	197,615.18	821,137.41

STATE: UTAH

ZIP CODE		QUARTER 1	QUARTER 2	QUARTER 3	QUARTER 4	TOTAL GRAMS
	840	19,924.87	19,305.17	19,044.81	18,045.81	76,320.66
	841	14,382.27	13,846.15	13,912.09	13,429.56	55,570.07
	843	2,104.47	2,072.12	2,026.22	1,700.27	7,903.08
	844	4,461.20	4,337.24	4,437.38	4,231.60	17,467.42
	845	1,494.47	1,177.27	1,123.73	1,247.87	5,043.34
	846	3,409.93	3,203.40	2,923.33	2,960.82	12,497.48
	847	5,445.76	5,458.97	5,578.24	5,267.77	21,750.74
TOTAL		51,222.97	49,400.32	49,045.80	46,883.70	196,552.79

STATE: VERMONT

ZIP CODE		QUARTER 1	QUARTER 2	QUARTER 3	QUARTER 4	TOTAL GRAMS
	50	1,354.24	1,305.89	984.97	1,130.19	4,775.29
	51	549.29	455.19	569.93	564.44	2,138.85
	52	511.19	495.74	484.99	417.59	1,909.51
	53	659.88	659.88	665.52	710.73	2,696.01
	54	4,480	4,106.84	4,204.79	4,024.79	16,816.42
	56	896.44	825.89	853.71	747.99	3,324.03

	57	1,445.13	1,520.48	1,217.72	1,388.21	5,571.54
	58	850.04	821.2	777.17	848.5	3,296.91
TOTAL		10,746.21	10,191.11	9,758.80	9,832.44	40,528.56

STATE:	VIRGIN ISLANDS				
ZIP CODE	QUARTER 1	QUARTER 2	QUARTER 3	QUARTER 4	TOTAL GRAMS
8	139.62	152.01	119.42	113.85	524.9
TOTAL	139.62	152.01	119.42	113.85	524.9

STATE:	VIRGINIA				
ZIP CODE	QUARTER 1	QUARTER 2	QUARTER 3	QUARTER 4	TOTAL GRAMS
201	6,366.14	6,674.81	5,939.50	5,924.56	24,905.01
220	3,310.22	2,659.15	2,706.90	2,658.35	11,334.62
221	3,148.23	2,954.19	2,739.39	2,916.87	11,758.68
222	684.02	715.65	607.32	625.24	2,632.23
223	1,351.47	1,365.09	1,404.24	1,447.90	5,568.70
224	2,985.89	2,647.03	2,689.44	2,639.02	10,961.38
225	799.79	969.72	903.27	862.38	3,535.16
226	3,061.63	2,912.86	2,955.79	3,032.31	11,962.59
227	581.45	489.87	439.98	519	2,030.30
228	2,272.35	2,119.35	1,866.54	1,957.01	8,215.25
229	2,641.46	2,438.74	2,410.38	2,331.03	9,821.61
230	2,272.12	2,049.26	2,002.65	1,972.45	8,296.48
231	3,297.50	3,019.17	2,678.68	3,034.87	12,030.22
232	8,702.01	8,032.56	7,551.38	6,779.42	31,065.37
233	2,417.73	2,403.66	2,407.33	2,408.02	9,636.74
234	5,629.76	5,268.29	5,390.56	5,316.98	21,605.59
235	2,192.86	2,318.76	1,794.30	2,198.99	8,504.91
236	4,390.54	3,490.52	3,882.64	3,358.48	15,122.18
237	2,009.73	1,793.98	1,823.40	1,906.99	7,534.10
238	3,573.51	3,130.19	3,169.23	2,921.56	12,794.49
239	705.26	693.1	640.97	673.84	2,713.17
240	4,259.56	3,931.96	3,945.56	3,884.25	16,021.33
241	5,039.05	4,744.65	4,449.34	4,250.59	18,483.63
242	4,351.80	4,192.70	4,132.62	3,855.86	16,532.98

	243	2,402.21	2,346.05	2,239.68	1,955.22	8,943.16
	244	1,403.44	1,293.12	1,234.84	1,265.37	5,196.77
	245	3,446.34	3,295.36	2,948.07	2,643.05	12,332.82
	246	1,162.40	1,113.33	1,137.61	915.97	4,329.31
TOTAL		84,458.47	79,063.12	76,091.61	74,255.58	313,868.78

STATE: WASHINGTON

ZIP CODE		QUARTER 1	QUARTER 2	QUARTER 3	QUARTER 4	TOTAL GRAMS
	980	16,067.26	15,160.26	15,063.45	13,793.83	60,084.80
	981	12,077.40	11,677.89	11,124.39	10,437.54	45,317.22
	982	13,445.88	12,510.22	12,020.14	11,328.36	49,304.60
	983	12,934	12,671.59	12,059.41	11,653.15	49,318.15
	984	8,316.48	8,009.63	7,557.49	7,034.92	30,918.52
	985	6,857.76	6,557.71	6,685.11	6,249.67	26,350.25
	986	14,874.43	14,272.62	14,067.99	13,143.31	56,358.35
	988	3,163.04	3,131.83	2,970.17	3,116.90	12,381.94
	989	3,976.17	3,659.23	3,537.08	3,329.33	14,501.81
	990	1,559.48	1,603.99	1,623.34	1,585.33	6,372.14
	991	2,622.49	2,719.84	2,469.79	2,506.03	10,318.15
	992	11,458.35	11,046.08	10,885.25	10,342.86	43,732.54
	993	6,835.10	6,250.95	5,707.59	5,588.88	24,382.52
	994	978.16	1,117.06	1,111.65	1,130.82	4,337.69
TOTAL		115,166	110,388.90	106,882.85	101,240.93	433,678.68

STATE: WEST VIRGINIA

ZIP CODE		QUARTER 1	QUARTER 2	QUARTER 3	QUARTER 4	TOTAL GRAMS
	247	914.8	631.72	708.44	609.5	2,864.46
	248	233.24	208.35	223.09	247.96	912.64
	249	547.89	449.66	449.47	382.4	1,829.42
	250	615.81	550.48	640.48	695.52	2,502.29
	251	404.87	730.37	304.16	316.32	1,755.72
	252	511.15	489.58	525.03	550.92	2,076.68
	253	1,576.35	1,449.40	1,452.77	1,326.02	5,804.54
	254	4,142.38	3,899.04	4,024.28	3,442.03	15,507.73
	255	1,000.15	1,062.94	1,090.76	1,062.17	4,216.02

	256	350.25	351.67	394.77	384.03	1,480.72
	257	1,627.77	1,370.29	1,220.52	1,245.70	5,464.28
	258	1,301.62	1,284.80	1,205.37	1,228.08	5,019.87
	259	369.38	356.58	303.04	323.09	1,352.09
	260	2,243.97	2,144.18	2,075.97	2,027.37	8,491.49
	261	917.58	844.69	903.85	983.56	3,649.68
	262	773.6	755.88	818.61	802.67	3,150.76
	263	1,962.09	1,706.73	1,855.34	1,611.54	7,135.70
	264	454.82	457.26	411.7	418.84	1,742.62
	265	1,762.32	1,597.13	1,553.60	1,500.54	6,413.59
	266	445.65	517.49	491.88	452.21	1,907.23
	267	815.05	689.6	691.09	647.06	2,842.80
	268	418.28	328.83	295.28	283.85	1,326.24
TOTAL		23,389.02	21,876.67	21,639.50	20,541.38	87,446.57

STATE: WISCONSIN

ZIP CODE		QUARTER 1	QUARTER 2	QUARTER 3	QUARTER 4	TOTAL GRAMS
	530	9,113.16	8,168.73	8,152.01	8,130.65	33,564.55
	531	9,257.27	8,482.98	7,920.98	8,240.84	33,902.07
	532	15,639.95	14,617.87	13,755.32	13,352.53	57,365.67
	534	1,676.11	1,639.84	1,628.76	1,568.21	6,512.92
	535	8,636.71	8,171.29	8,164.43	7,687.07	32,659.50
	537	7,537.16	7,026.98	6,655.77	6,647.56	27,867.47
	538	770.99	643.98	641.14	625.82	2,681.93
	539	4,372.42	3,976.84	3,690.11	3,693.06	15,732.43
	540	1,139.22	1,056.18	982.04	952.45	4,129.89
	541	1,661.83	1,600.04	1,578.97	1,323.14	6,163.98
	542	1,193.97	1,213.51	1,115.75	1,076.07	4,599.30
	543	2,402.07	2,076.28	2,137.54	1,903.04	8,518.93
	544	3,851.21	3,310.25	3,256.49	3,168.42	13,586.37
	545	1,073.91	1,147.61	1,052.41	848.76	4,122.69
	546	3,590.24	3,492.01	3,537.34	3,449.66	14,069.25
	547	3,264.96	2,721.25	2,680.43	2,441.55	11,108.19
	548	2,374.80	2,436.48	2,095.61	1,982.10	8,888.99
	549	6,135.10	5,766.73	5,600.45	5,446.19	22,948.47
TOTAL		83,691.08	77,548.85	74,645.55	72,537.12	308,422.60

STATE: WYOMING

ZIP CODE	QUARTER 1	QUARTER 2	QUARTER 3	QUARTER 4	TOTAL GRAMS
820	2,043.76	1,899.02	1,565.61	1,498.31	7,006.70
822	255.24	315.21	260.64	256.57	1,087.66
823	139.09	156.81	138.36	138.5	572.76
824	1,039.50	909.25	917.08	848.71	3,714.54
825	828.49	731.24	685.91	678.28	2,923.92
826	1,388.49	1,299.63	1,316.52	1,182.09	5,186.73
827	362.05	439.99	316.98	375.25	1,494.27
828	1,069.10	1,205.53	1,229.55	1,028.59	4,532.77
829	1,786.23	2,053.30	2,176.87	1,979.29	7,995.69
830	52.92	81.1	80.78	85.57	300.37
831	256.45	243.9	311.8	239.93	1,052.08
TOTAL	9,221.32	9,334.98	9,000.10	8,311.09	35,867.49

DRUG CODE: 9333 DRUG NAME: THEBAINE

STATE: TEXAS

ZIP CODE	QUARTER 1	QUARTER 2	QUARTER 3	QUARTER 4	TOTAL GRAMS
782	0	0	25	0	25
TOTAL	0	0	25	0	25

DRUG CODE: 9630 DRUG NAME: OPIUM TINCTURE (LAUDANUM)

STATE: CALIFORNIA

ZIP CODE	QUARTER 1	QUARTER 2	QUARTER 3	QUARTER 4	TOTAL GRAMS
907	1.18	0	0	0	1.18
951	4.73	0	0	0	4.73
TOTAL	5.91	0	0	0	5.91

STATE: CONNECTICUT

ZIP CODE	QUARTER 1	QUARTER 2	QUARTER 3	QUARTER 4	TOTAL GRAMS
64	0	0	4.73	0	4.73
TOTAL	0	0	4.73	0	4.73

STATE: DELAWARE

ZIP CODE	QUARTER 1	QUARTER 2	QUARTER 3	QUARTER 4	TOTAL GRAMS
197	0	1.18	0	0	1.18
TOTAL	0	1.18	0	0	1.18

STATE: FLORIDA

ZIP CODE	QUARTER 1	QUARTER 2	QUARTER 3	QUARTER 4	TOTAL GRAMS
321	9.46	4.73	0	0	14.19
TOTAL	9.46	4.73	0	0	14.19

STATE: ILLINOIS

ZIP CODE	QUARTER 1	QUARTER 2	QUARTER 3	QUARTER 4	TOTAL GRAMS
616	4.73	4.73	0	0	9.46
618	1.18	1.18	0	0	2.36
626	0	4.73	0	0	4.73
TOTAL	5.91	10.64	0	0	16.55

STATE: INDIANA

ZIP CODE	QUARTER 1	QUARTER 2	QUARTER 3	QUARTER 4	TOTAL GRAMS
460	1.18	0	0	0	1.18
461	18.92	0	0	0	18.92
462	1.18	9.46	0	0	10.64
463	0	4.73	0	0	4.73
TOTAL	21.28	14.19	0	0	35.47

STATE: KANSAS

ZIP CODE	QUARTER 1	QUARTER 2	QUARTER 3	QUARTER 4	TOTAL GRAMS

660	4.73	0	0	0	4.73
673	1.18	0	0	0	1.18
TOTAL	5.91	0	0	0	5.91

STATE: KENTUCKY

ZIP CODE	QUARTER 1	QUARTER 2	QUARTER 3	QUARTER 4	TOTAL GRAMS
410	2.36	0	0	0	2.36
TOTAL	2.36	0	0	0	2.36

STATE: MARYLAND

ZIP CODE	QUARTER 1	QUARTER 2	QUARTER 3	QUARTER 4	TOTAL GRAMS
212	0	2.36	0	0	2.36
214	0	1.18	0	0	1.18
217	4.72	4.73	0	0	9.45
TOTAL	4.72	8.27	0	0	12.99

STATE: MICHIGAN

ZIP CODE	QUARTER 1	QUARTER 2	QUARTER 3	QUARTER 4	TOTAL GRAMS
480	9.46	0	0	0	9.46
481	7.08	4.72	0	0	11.8
483	4.73	0	0	0	4.73
489	4.73	0	0	0	4.73
TOTAL	26	4.72	0	0	30.72

STATE: NEW HAMPSHIRE

ZIP CODE	QUARTER 1	QUARTER 2	QUARTER 3	QUARTER 4	TOTAL GRAMS
33	4.73	0	0	0	4.73
TOTAL	4.73	0	0	0	4.73

STATE: NEW JERSEY

ZIP CODE	QUARTER 1	QUARTER 2	QUARTER 3	QUARTER 4	TOTAL GRAMS
74	0	1.18	0	0	1.18
TOTAL	0	1.18	0	0	1.18

STATE: NEW YORK

ZIP CODE	QUARTER 1	QUARTER 2	QUARTER 3	QUARTER 4	TOTAL GRAMS
141	4.73	0	0	0	4.73
TOTAL	4.73	0	0	0	4.73

STATE: OHIO

ZIP CODE	QUARTER 1	QUARTER 2	QUARTER 3	QUARTER 4	TOTAL GRAMS
430	5.9	2.36	0	0	8.26
431	1.18	0	0	0	1.18
432	18.91	1.18	0	0	20.09
440	9.44	1.18	0	0	10.62
442	4.72	0	0	0	4.72
444	4.72	0	0	0	4.72
450	2.36	0	0	0	2.36
452	1.18	0	0	0	1.18
453	13	0	0	0	13
456	9.46	4.73	0	0	14.19
457	1.18	0	0	0	1.18
458	1.18	0	0	0	1.18
TOTAL	73.23	9.45	0	0	82.68

STATE: OREGON

ZIP CODE	QUARTER 1	QUARTER 2	QUARTER 3	QUARTER 4	TOTAL GRAMS
970	1.18	0	0	0	1.18
971	1.18	0	0	0	1.18
TOTAL	2.36	0	0	0	2.36

STATE: PENNSYLVANIA

ZIP CODE	QUARTER 1	QUARTER 2	QUARTER 3	QUARTER 4	TOTAL GRAMS

152	8.26	9.44	0	0	17.7
180	0	1.18	0	0	1.18
190	2.36	0	0	0	2.36
TOTAL	10.62	10.62	0	0	21.24

STATE: TEXAS

ZIP CODE	QUARTER 1	QUARTER 2	QUARTER 3	QUARTER 4	TOTAL GRAMS
750	1.18	2.36	0	0	3.54
760	4.72	1.18	0	0	5.9
761	1.18	1.18	0	0	2.36
790	2.36	0	0	0	2.36
TOTAL	9.44	4.72	0	0	14.16

STATE: VIRGINIA

ZIP CODE	QUARTER 1	QUARTER 2	QUARTER 3	QUARTER 4	TOTAL GRAMS
201	8.26	0	0	0	8.26
223	2.36	0	0	0	2.36
TOTAL	10.62	0	0	0	10.62

STATE: WASHINGTON

ZIP CODE	QUARTER 1	QUARTER 2	QUARTER 3	QUARTER 4	TOTAL GRAMS
982	1.18	0	0	0	1.18
984	2.36	0	0	0	2.36
TOTAL	3.54	0	0	0	3.54

STATE: WEST VIRGINIA

ZIP CODE	QUARTER 1	QUARTER 2	QUARTER 3	QUARTER 4	TOTAL GRAMS
253	2.36	1.18	0	0	3.54
254	1.18	1.18	0	0	2.36
TOTAL	3.54	2.36	0	0	5.9

STATE: WISCONSIN

ZIP CODE	QUARTER 1	QUARTER 2	QUARTER 3	QUARTER 4	TOTAL GRAMS
537	2.36	1.18	0	0	3.54
543	1.18	0	0	0	1.18
546	0	4.73	0	0	4.73
547	0	4.73	0	0	4.73
TOTAL	3.54	10.64	0	0	14.18

DRUG CODE: 9639 DRUG NAME: OPIUM POWDERED

STATE: ALABAMA

ZIP CODE	QUARTER 1	QUARTER 2	QUARTER 3	QUARTER 4	TOTAL GRAMS
350	4.68	6.84	5.4	5.4	22.32
351	1.44	4.32	0	0	5.76
352	37.44	27.36	27.72	15.48	108
354	2.52	7.2	7.56	10.08	27.36
355	0	0.72	0.72	0	1.44
356	5.76	9.72	3.96	4.32	23.76
357	0.72	4.68	1.44	0.72	7.56
358	6.48	11.52	6.84	9.72	34.56
359	1.08	2.88	2.88	3.96	10.8
361	4.68	5.04	10.08	7.2	27
362	0.72	0.36	1.44	1.8	4.32
363	0	0	0.72	2.16	2.88
364	0	0.72	2.52	1.8	5.04
365	14.04	5.4	6.12	9	34.56
366	2.52	7.92	8.28	6.48	25.2
367	0	0	0	0.36	0.36
368	0	0	0.72	0	0.72
TOTAL	82.08	94.68	86.4	78.48	341.64

STATE: ALASKA

ZIP CODE	QUARTER 1	QUARTER 2	QUARTER 3	QUARTER 4	TOTAL GRAMS
995	21.6	11.52	12.6	11.88	57.6
996	5.76	4.32	4.32	8.64	23.04
997	0.72	5.76	1.8	2.52	10.8

998	1.44	3.6	0.72	4.32	10.08
999	0	0	0.36	0.72	1.08
TOTAL	29.52	25.2	19.8	28.08	102.6

STATE: ARIZONA

ZIP CODE	QUARTER 1	QUARTER 2	QUARTER 3	QUARTER 4	TOTAL GRAMS
850	111.24	97.92	106.2	102.24	417.6
851	2.16	2.52	0	4.32	9
852	89.28	84.6	95.4	113.4	382.68
853	17.28	37.44	42.12	45	141.84
855	0.72	0	0.36	7.56	8.64
856	0.72	0.36	3.96	1.44	6.48
857	29.88	26.28	24.12	46.44	126.72
859	0.36	0	0.36	0	0.72
860	1.44	0.72	2.16	2.16	6.48
863	21.96	18.36	17.28	10.8	68.4
864	2.52	5.4	0.36	4.32	12.6
TOTAL	277.56	273.6	292.32	337.68	1,181.16

STATE: ARKANSAS

ZIP CODE	QUARTER 1	QUARTER 2	QUARTER 3	QUARTER 4	TOTAL GRAMS
716	4.32	4.32	4.32	3.6	16.56
717	0.36	0	0	0.72	1.08
719	3.6	1.44	5.4	3.96	14.4
720	1.08	6.48	6.12	3.24	16.92
721	28.44	34.2	23.04	37.8	123.48
722	20.88	15.48	21.24	19.08	76.68
723	1.44	4.68	0	0.36	6.48
724	10.08	15.12	14.76	13.68	53.64
725	2.88	1.8	1.8	1.44	7.92
726	0.36	0.72	1.08	1.44	3.6
727	7.92	11.88	8.28	7.2	35.28
728	0	0	0.36	0.36	0.72
729	2.16	0.72	0.72	0.72	4.32
TOTAL	83.52	96.84	87.12	93.6	361.08

STATE: CALIFORNIA

ZIP CODE	QUARTER 1	QUARTER 2	QUARTER 3	QUARTER 4	TOTAL GRAMS
900	11.16	25.92	13.32	20.16	70.56
902	1.08	3.24	3.24	7.92	15.48
903	0	0.36	0.36	0.36	1.08
904	8.64	1.44	5.04	7.92	23.04
905	2.52	0	1.44	0.36	4.32
906	0.36	1.8	0.36	1.8	4.32
907	8.64	12.24	9.72	9	39.6
908	3.24	6.48	5.4	7.2	22.32
910	1.44	5.4	10.44	4.68	21.96
911	2.88	5.04	2.52	2.88	13.32
912	2.16	5.04	1.44	2.16	10.8
913	5.76	9.36	3.96	11.16	30.24
914	0.72	0.72	0	0.72	2.16
915	0.72	2.16	1.08	0.36	4.32
917	15.84	16.56	8.64	11.16	52.2
918	0	0.72	0	0	0.72
919	2.16	5.04	13.68	0.36	21.24
920	42.48	22.32	41.76	36.36	142.92
921	23.4	43.2	38.88	34.92	140.4
922	26.64	21.24	23.4	17.64	88.92
923	9.36	10.08	6.84	3.24	29.52
925	6.84	7.2	12.6	9	35.64
926	11.88	7.56	17.64	12.96	50.04
927	2.88	5.04	4.68	7.56	20.16
928	8.28	8.28	11.88	9	37.44
930	5.76	7.2	2.88	3.96	19.8
931	0.72	3.24	5.4	0	9.36
932	4.68	5.4	6.84	5.4	22.32
933	5.4	3.24	3.24	1.08	12.96
934	6.12	10.8	7.92	7.92	32.76
935	0.36	0.72	1.8	5.76	8.64
936	0.72	1.08	1.44	1.44	4.68
937	5.4	8.64	22.68	12.6	49.32

939	1.8	1.8	2.16	1.44	7.2
940	12.24	6.84	9.72	4.32	33.12
941	30.96	27.72	31.68	25.92	116.28
943	2.16	9.36	9.72	7.92	29.16
944	0	0.72	0	0	0.72
945	23.76	27.72	23.4	24.12	99
946	6.84	3.96	3.96	6.48	21.24
947	1.08	0	1.08	0.36	2.52
948	0	0	7.2	1.44	8.64
949	2.88	1.8	2.88	3.24	10.8
950	12.6	9.36	8.28	9.72	39.96
951	3.96	4.68	6.12	3.96	18.72
952	11.52	6.12	4.32	11.16	33.12
953	19.08	15.84	10.44	13.68	59.04
954	7.56	6.48	5.76	9.72	29.52
955	2.88	0	0.72	0.72	4.32
956	14.04	21.24	26.64	21.6	83.52
957	5.04	1.08	2.16	0	8.28
958	12.24	29.88	12.96	28.44	83.52
959	11.16	26.28	18.36	16.92	72.72
960	2.52	2.52	4.32	5.4	14.76
961	0.36	0.36	2.16	0	2.88
TOTAL	412.92	470.52	484.56	453.6	1,821.60

STATE: COLORADO

ZIP CODE	QUARTER 1	QUARTER 2	QUARTER 3	QUARTER 4	TOTAL GRAMS
800	37.08	41.4	46.08	28.08	152.64
801	21.24	27.36	36.36	55.08	140.04
802	43.56	59.4	70.92	66.24	240.12
803	1.44	0.72	2.88	2.16	7.2
804	8.64	11.52	19.8	15.84	55.8
805	44.64	53.28	41.4	41.04	180.36
806	0.72	8.28	3.6	3.96	16.56
807	2.16	4.68	0.72	2.52	10.08
808	0	0	0	0.72	0.72
809	48.6	33.48	45.72	32.4	160.2

	810	5.4	3.6	3.6	4.68	17.28
	811	0.36	0	0	0	0.36
	812	0.72	0.36	0.72	1.8	3.6
	813	0	1.44	2.52	3.6	7.56
	814	5.04	7.2	6.84	9.72	28.8
	815	12.24	6.84	6.48	7.92	33.48
	816	0	1.08	0.72	3.6	5.4
TOTAL		231.84	260.64	288.36	279.36	1,060.20

STATE: CONNECTICUT

ZIP CODE		QUARTER 1	QUARTER 2	QUARTER 3	QUARTER 4	TOTAL GRAMS
	60	24.48	11.16	9	12.24	56.88
	61	3.96	18.36	7.92	7.56	37.8
	62	0	0.72	0	0.72	1.44
	63	10.08	11.16	12.96	21.96	56.16
	64	42.48	37.08	30.96	8.64	119.16
	65	11.52	15.48	7.2	9	43.2
	66	6.12	13.68	7.2	2.16	29.16
	67	13.68	11.88	6.12	5.04	36.72
	68	2.16	1.44	0.36	0	3.96
	69	0	0	0	0.72	0.72
TOTAL		114.48	120.96	81.72	68.04	385.2

STATE: DELAWARE

ZIP CODE		QUARTER 1	QUARTER 2	QUARTER 3	QUARTER 4	TOTAL GRAMS
	197	1.08	2.88	6.12	6.12	16.2
	198	7.92	2.52	3.96	3.96	18.36
	199	8.64	9.36	6.12	1.44	25.56
TOTAL		17.64	14.76	16.2	11.52	60.12

STATE: DISTRICT OF COLUMBIA

ZIP CODE		QUARTER 1	QUARTER 2	QUARTER 3	QUARTER 4	TOTAL GRAMS
	200	11.88	10.8	18.36	14.04	55.08
	203	0	1.44	0	0	1.44

	204	0.36	0.72	0	1.44	2.52
TOTAL		12.24	12.96	18.36	15.48	59.04

STATE:	FLORIDA					
ZIP CODE		QUARTER 1	QUARTER 2	QUARTER 3	QUARTER 4	TOTAL GRAMS
	320	6.12	5.76	27.36	17.28	56.52
	321	17.64	24.48	15.48	16.56	74.16
	322	47.88	42.12	26.28	31.32	147.6
	323	0.72	2.16	7.56	14.76	25.2
	324	7.92	7.2	6.48	6.84	28.44
	325	57.96	33.48	37.8	43.92	173.16
	326	10.8	9.72	5.76	6.12	32.4
	327	24.48	27.72	30.24	31.68	114.12
	328	25.92	29.88	28.44	26.28	110.52
	329	46.08	32.04	28.08	36.72	142.92
	330	13.68	12.96	14.4	7.2	48.24
	331	19.8	23.04	20.52	19.44	82.8
	333	8.64	12.96	6.12	16.56	44.28
	334	56.52	44.28	43.92	32.76	177.48
	335	5.76	9.72	7.2	10.8	33.48
	336	26.28	32.76	23.76	40.68	123.48
	337	18.36	21.24	22.32	19.44	81.36
	338	6.48	8.28	7.56	16.92	39.24
	339	28.44	33.84	20.16	32.04	114.48
	341	24.84	15.12	13.68	26.64	80.28
	342	20.16	19.8	10.8	38.16	88.92
	344	0.36	0.36	0.72	1.44	2.88
	346	20.52	10.08	15.12	18.36	64.08
	347	28.8	33.48	27.36	37.44	127.08
	349	2.88	5.76	5.4	4.32	18.36
TOTAL		527.04	498.24	452.52	553.68	2,031.48

STATE:	GEORGIA					
ZIP CODE		QUARTER 1	QUARTER 2	QUARTER 3	QUARTER 4	TOTAL GRAMS

	300	21.96	31.68	23.76	27	104.4
	301	31.68	34.92	42.12	28.08	136.8
	302	17.28	11.52	15.48	21.24	65.52
	303	21.24	28.44	20.52	19.8	90
	304	3.24	3.6	1.08	2.52	10.44
	305	6.12	7.2	10.08	9.72	33.12
	306	9.36	14.4	10.08	13.32	47.16
	307	9	12.24	6.84	7.92	36
	308	0	0.36	0	0	0.36
	309	24.84	15.12	12.6	23.04	75.6
	310	1.8	9.36	2.16	4.32	17.64
	312	5.4	12.96	8.28	3.96	30.6
	313	1.44	0	1.44	0.36	3.24
	314	7.2	11.16	12.24	5.76	36.36
	315	0.72	6.12	5.04	8.64	20.52
	316	5.76	2.16	3.24	1.8	12.96
	317	4.32	9	5.76	6.84	25.92
	319	2.88	0.36	4.32	0	7.56
	398	0	0	0	1.44	1.44
TOTAL		174.24	210.6	185.04	185.76	755.64

STATE: GUAM

ZIP CODE		QUARTER 1	QUARTER 2	QUARTER 3	QUARTER 4	TOTAL GRAMS
	969	0.36	0	0	0	0.36
TOTAL		0.36	0	0	0	0.36

STATE: HAWAII

ZIP CODE		QUARTER 1	QUARTER 2	QUARTER 3	QUARTER 4	TOTAL GRAMS
	967	8.28	11.88	16.2	11.52	47.88
	968	16.56	15.84	18	16.92	67.32
TOTAL		24.84	27.72	34.2	28.44	115.2

STATE: IDAHO

ZIP CODE	QUARTER 1	QUARTER 2	QUARTER 3	QUARTER 4	TOTAL GRAMS
832	3.6	4.32	2.16	4.68	14.76
833	2.16	3.96	1.8	3.24	11.16
834	5.04	5.76	4.32	1.08	16.2
835	0.36	0.36	0	0	0.72
836	18.36	14.4	21.96	23.04	77.76
837	6.48	9.36	9.36	7.2	32.4
838	9.72	12.96	9.36	10.44	42.48
TOTAL	45.72	51.12	48.96	49.68	195.48

STATE: ILLINOIS

ZIP CODE	QUARTER 1	QUARTER 2	QUARTER 3	QUARTER 4	TOTAL GRAMS
600	32.4	31.32	23.04	23.4	110.16
601	35.28	33.84	28.8	32.04	129.96
602	2.52	3.24	0	0.36	6.12
603	1.44	0	2.52	0.36	4.32
604	20.88	23.76	25.92	24.84	95.4
605	10.8	8.28	7.2	5.76	32.04
606	14.4	19.44	9.72	17.28	60.84
608	1.44	0	2.88	1.08	5.4
609	0	0	0	3.96	3.96
610	1.08	2.52	3.24	3.6	10.44
611	11.16	17.64	13.68	16.92	59.4
612	0.36	4.68	3.6	0.72	9.36
613	1.08	2.16	1.44	5.76	10.44
614	2.16	2.16	2.88	6.84	14.04
615	0.36	0	0	1.8	2.16
616	5.4	18	10.08	18	51.48
617	2.52	2.52	3.24	2.88	11.16
618	2.16	3.24	2.16	2.88	10.44
619	0.72	0	0	1.08	1.8
620	0.36	5.76	2.88	4.32	13.32
622	3.96	0.72	3.96	1.08	9.72
623	1.08	3.96	5.04	4.32	14.4
624	1.8	0.72	1.44	0	3.96
625	1.08	0.72	1.8	1.08	4.68

	626	0	1.8	1.08	0.72	3.6
	627	0.72	3.24	10.08	8.28	22.32
	628	0.36	0	0	0	0.36
	629	2.88	4.32	5.4	3.6	16.2
TOTAL		158.4	194.04	172.08	192.96	717.48

STATE:	INDIANA					
ZIP CODE		QUARTER 1	QUARTER 2	QUARTER 3	QUARTER 4	TOTAL GRAMS
	460	21.96	30.24	28.08	33.12	113.4
	461	26.28	25.92	30.6	14.04	96.84
	462	72	82.8	76.68	98.28	329.76
	463	9.36	5.76	3.24	7.2	25.56
	464	0.36	0.36	1.08	1.44	3.24
	465	12.6	14.76	10.44	14.4	52.2
	466	4.32	3.6	3.96	6.48	18.36
	467	0.72	1.44	0.36	6.48	9
	468	8.64	14.4	11.16	17.64	51.84
	469	5.04	3.6	3.6	6.48	18.72
	470	1.44	1.08	1.08	0.72	4.32
	471	13.32	12.96	11.52	13.32	51.12
	472	6.84	7.2	8.64	3.24	25.92
	473	9	15.12	12.6	12.6	49.32
	474	4.68	2.52	9.36	6.48	23.04
	475	7.2	11.52	6.84	7.92	33.48
	476	3.96	2.52	1.44	1.44	9.36
	477	8.64	12.6	10.8	12.6	44.64
	478	2.16	3.24	1.44	2.88	9.72
	479	4.32	2.16	1.8	3.6	11.88
TOTAL		222.84	253.8	234.72	270.36	981.72

STATE:	IOWA					
ZIP CODE		QUARTER 1	QUARTER 2	QUARTER 3	QUARTER 4	TOTAL GRAMS
	500	4.32	5.04	2.16	6.84	18.36
	501	19.44	6.12	3.24	1.8	30.6

	502	1.08	1.8	3.96	2.88	9.72
	503	3.96	3.6	5.04	12.24	24.84
	504	0	4.32	1.44	0.72	6.48
	505	0.36	1.08	2.88	1.44	5.76
	506	2.52	0.72	10.44	1.44	15.12
	507	6.84	12.6	10.8	9	39.24
	508	0	0.36	0	0.36	0.72
	510	2.52	0.72	1.08	1.8	6.12
	511	4.68	7.2	4.32	4.32	20.52
	512	0.36	1.08	0.72	0	2.16
	513	2.52	2.16	1.8	2.88	9.36
	514	3.24	1.08	1.44	0.36	6.12
	515	6.48	15.12	8.64	6.48	36.72
	520	1.08	2.52	1.08	1.08	5.76
	521	0	0.72	1.44	0	2.16
	522	29.88	8.64	19.44	14.4	72.36
	523	0.72	1.08	0.72	1.44	3.96
	524	5.76	6.48	5.4	9.72	27.36
	525	0.36	1.44	0	1.44	3.24
	526	0.72	3.96	0	0	4.68
	527	0.72	0	0.72	0	1.44
	528	14.76	12.96	10.08	11.88	49.68
TOTAL		112.32	100.8	96.84	92.52	402.48

STATE: KANSAS

ZIP CODE		QUARTER 1	QUARTER 2	QUARTER 3	QUARTER 4	TOTAL GRAMS
	660	14.4	30.96	24.84	28.08	98.28
	661	17.28	9.72	6.84	18.72	52.56
	662	60.84	73.08	68.04	69.12	271.08
	664	1.8	0	2.52	0	4.32
	665	13.32	14.76	16.92	16.2	61.2
	666	2.88	5.76	5.04	6.48	20.16
	667	2.88	1.8	0.72	1.8	7.2
	668	0.36	1.08	1.08	0.36	2.88
	669	0	0.36	1.44	0	1.8

	670	3.96	9.36	3.6	2.88	19.8
	671	1.08	0	0.72	0.72	2.52
	672	26.28	33.12	22.32	24.12	105.84
	673	0	0	1.44	2.16	3.6
	674	9.36	6.12	10.08	9.72	35.28
	675	3.6	0.36	3.6	0.72	8.28
	676	6.12	3.6	6.48	1.44	17.64
	677	0	0	0	1.44	1.44
	678	1.44	1.8	1.8	1.08	6.12
TOTAL		165.6	191.88	177.48	185.04	720

STATE: KENTUCKY

ZIP CODE		QUARTER 1	QUARTER 2	QUARTER 3	QUARTER 4	TOTAL GRAMS
	400	2.16	5.4	8.28	1.8	17.64
	401	2.16	0	1.08	0.36	3.6
	402	53.28	39.6	36	40.32	169.2
	403	5.76	1.44	6.48	2.88	16.56
	404	30.6	28.44	7.2	6.12	72.36
	405	43.92	30.96	32.4	37.44	144.72
	406	0	0.72	0	2.16	2.88
	407	0	21.96	4.32	24.12	50.4
	408	2.16	0	0	0	2.16
	409	0	0.72	0	0.72	1.44
	410	6.48	11.52	6.84	11.88	36.72
	411	1.44	2.16	1.44	3.96	9
	412	0	1.44	0	0.72	2.16
	413	0.72	0	1.08	0.36	2.16
	414	0	0.36	0	0.36	0.72
	415	0.36	1.08	0.36	1.44	3.24
	416	0	0.36	0	0.36	0.72
	417	1.44	1.8	1.08	1.08	5.4
	418	0	0	0	0.36	0.36
	420	16.92	9	17.28	16.92	60.12
	421	11.52	12.24	6.84	7.2	37.8
	422	1.44	5.04	4.32	3.6	14.4
	423	1.44	0.72	4.32	2.52	9

	424	8.64	2.52	2.52	2.52	16.2
	425	1.44	1.08	3.6	3.96	10.08
	426	0.72	0	0	0	0.72
	427	4.32	1.08	0.72	0.72	6.84
TOTAL		196.92	179.64	146.16	173.88	696.6

STATE: LOUISIANA

ZIP CODE		QUARTER 1	QUARTER 2	QUARTER 3	QUARTER 4	TOTAL GRAMS
	700	18.72	21.96	17.28	10.44	68.4
	701	15.84	11.52	8.28	14.4	50.04
	703	7.92	10.8	2.16	4.32	25.2
	704	5.76	8.64	7.92	6.48	28.8
	705	20.88	17.28	20.16	28.8	87.12
	706	5.4	8.64	7.56	6.48	28.08
	707	6.48	0	1.44	1.44	9.36
	708	4.68	16.56	21.24	12.6	55.08
	710	7.92	0	0.36	0	8.28
	711	10.8	7.2	9	7.92	34.92
	712	0	4.68	1.44	6.48	12.6
	713	1.08	5.04	1.44	5.04	12.6
	714	0	0.72	0	0	0.72
TOTAL		105.48	113.04	98.28	104.4	421.2

STATE: MAINE

ZIP CODE		QUARTER 1	QUARTER 2	QUARTER 3	QUARTER 4	TOTAL GRAMS
	39	0.36	0.36	0	1.08	1.8
	40	3.24	3.96	14.04	4.68	25.92
	41	10.44	10.44	4.32	10.8	36
	42	9.36	8.28	11.16	9.72	38.52
	43	1.44	3.6	7.2	7.92	20.16
	44	10.44	12.24	7.56	16.2	46.44
	45	1.44	0	0	0	1.44
	46	1.44	4.32	1.44	1.8	9
	47	1.08	1.8	0.72	1.44	5.04
	48	0	0.36	6.84	3.24	10.44

49	0	2.52	0.72	1.08	4.32
TOTAL	39.24	47.88	54	57.96	199.08

STATE: MARYLAND

ZIP CODE	QUARTER 1	QUARTER 2	QUARTER 3	QUARTER 4	TOTAL GRAMS
206	2.88	4.32	2.52	3.6	13.32
207	6.84	4.32	4.32	5.76	21.24
208	18	19.08	14.76	34.56	86.4
209	0	1.08	0.72	0.72	2.52
210	11.88	11.52	10.44	8.28	42.12
211	2.16	6.84	4.32	3.6	16.92
212	11.52	28.8	18	33.48	91.8
214	1.08	0.72	1.44	0.72	3.96
215	1.44	0.72	2.16	2.52	6.84
216	3.6	10.44	5.76	3.96	23.76
217	2.16	6.84	2.88	2.88	14.76
218	1.44	2.16	2.52	3.96	10.08
219	0	0.72	0.72	0	1.44
TOTAL	63	97.56	70.56	104.04	335.16

STATE: MASSACHUSETTS

ZIP CODE	QUARTER 1	QUARTER 2	QUARTER 3	QUARTER 4	TOTAL GRAMS
10	23.04	43.92	28.44	42.48	137.88
11	22.68	26.64	25.56	18.72	93.6
12	1.44	0	0	0	1.44
13	0	0.72	0	0.36	1.08
14	2.52	1.8	5.04	5.04	14.4
15	0	8.28	10.8	6.12	25.2
16	0.72	7.56	6.84	3.6	18.72
17	6.12	1.8	5.76	15.84	29.52
18	14.76	9.72	7.2	1.44	33.12
19	7.92	10.8	10.44	19.8	48.96
20	9.72	16.2	12.96	14.4	53.28
21	42.48	43.92	38.16	54.36	178.92
22	3.96	2.16	1.44	2.52	10.08

23	7.56	10.44	6.48	7.56	32.04
24	16.2	17.28	2.88	16.92	53.28
25	4.68	5.04	6.12	12.24	28.08
26	1.44	0.72	0.72	3.6	6.48
27	7.56	7.56	8.28	13.68	37.08
TOTAL	172.8	214.56	177.12	238.68	803.16

STATE: MICHIGAN

ZIP CODE	QUARTER 1	QUARTER 2	QUARTER 3	QUARTER 4	TOTAL GRAMS
480	44.28	43.92	26.64	46.44	161.28
481	74.52	63	73.44	60.84	271.8
482	18	32.04	30.6	27	107.64
483	37.44	36.72	30.96	48.6	153.72
484	5.04	5.4	8.28	5.76	24.48
485	2.88	5.4	5.04	7.2	20.52
486	10.44	17.28	11.88	16.92	56.52
487	6.48	2.88	6.48	2.88	18.72
488	5.04	13.68	38.16	26.28	83.16
489	9.72	12.24	9.36	13.68	45
490	32.4	31.68	29.16	28.8	122.04
491	0.72	0.72	2.16	5.76	9.36
492	20.16	18.72	13.32	22.32	74.52
493	1.08	0	1.44	1.08	3.6
494	10.08	10.44	10.08	11.52	42.12
495	61.56	50.76	41.04	51.84	205.2
496	17.64	8.28	18.36	20.16	64.44
497	9	10.08	14.04	13.68	46.8
498	7.92	5.76	5.4	11.16	30.24
499	0	0.72	0	0.36	1.08
TOTAL	374.4	369.72	375.84	422.28	1,542.24

STATE: MINNESOTA

ZIP CODE	QUARTER 1	QUARTER 2	QUARTER 3	QUARTER 4	TOTAL GRAMS
550	24.48	31.32	18.72	21.96	96.48
551	34.56	35.64	28.8	54.72	153.72

	553	43.92	43.2	47.16	41.76	176.04
	554	142.2	129.96	132.48	135.36	540
	556	0.72	0	0	0	0.72
	557	5.04	1.08	2.88	1.8	10.8
	558	6.48	7.92	5.76	15.84	36
	559	55.08	61.2	61.92	57.6	235.8
	560	14.4	13.32	10.44	12.24	50.4
	561	0.72	2.16	0	1.44	4.32
	562	19.08	5.76	4.32	2.88	32.04
	563	33.84	27	30.6	21.24	112.68
	564	15.12	16.2	17.64	18.36	67.32
	565	5.04	5.04	2.52	2.88	15.48
	566	0.72	0	0.72	0.72	2.16
	567	0	1.44	0	0.36	1.8
TOTAL		401.4	381.24	363.96	389.16	1,535.76

STATE: MISSISSIPPI

ZIP CODE		QUARTER 1	QUARTER 2	QUARTER 3	QUARTER 4	TOTAL GRAMS
	386	7.56	3.96	7.92	5.4	24.84
	387	0.36	0	2.52	0.72	3.6
	388	3.96	6.84	9	6.84	26.64
	389	0	0	0.72	0	0.72
	391	0	0.36	1.08	0	1.44
	392	2.52	10.44	7.92	2.88	23.76
	393	4.32	1.8	0.72	1.08	7.92
	394	2.16	5.04	0	0.36	7.56
	395	8.28	7.2	4.32	4.32	24.12
	396	0	0.72	0	1.08	1.8
	397	3.96	3.6	2.16	2.52	12.24
TOTAL		33.12	39.96	36.36	25.2	134.64

STATE: MISSOURI

ZIP CODE		QUARTER 1	QUARTER 2	QUARTER 3	QUARTER 4	TOTAL GRAMS
	630	3.6	7.92	3.24	5.76	20.52
	631	24.48	31.32	32.04	42.12	129.96

	633	3.6	4.68	7.2	3.6	19.08
	634	0.72	0	1.08	0.72	2.52
	635	2.52	0	0	0.72	3.24
	636	0	0	0.72	0	0.72
	637	6.12	7.92	4.32	3.6	21.96
	638	0	0	2.52	2.16	4.68
	639	0.72	1.44	0.72	0	2.88
	640	27.36	23.04	24.12	22.68	97.2
	641	35.64	30.24	27	34.56	127.44
	644	0	1.08	0	1.8	2.88
	645	6.48	4.32	8.28	6.48	25.56
	646	0	0	0	1.44	1.44
	647	12.24	11.88	11.16	7.2	42.48
	648	0.72	4.68	4.68	3.96	14.04
	650	0	0.72	0	0.72	1.44
	651	1.08	0.36	0.72	2.52	4.68
	652	11.16	8.28	13.32	4.68	37.44
	653	3.6	1.44	2.88	1.44	9.36
	654	4.32	0.72	4.32	0.72	10.08
	655	3.6	7.92	5.04	15.12	31.68
	656	1.08	0	0	0	1.08
	657	0	0	0	1.8	1.8
	658	20.88	15.48	12.96	10.8	60.12
TOTAL		169.92	163.44	166.32	174.6	674.28

STATE:	MONTANA					
ZIP CODE		QUARTER 1	QUARTER 2	QUARTER 3	QUARTER 4	TOTAL GRAMS
	590	0	0	0	0.36	0.36
	591	16.2	18.36	20.16	21.6	76.32
	592	0	0	0	1.8	1.8
	594	9.72	10.44	7.2	7.56	34.92
	595	0	1.44	2.16	0.72	4.32
	596	6.48	5.76	1.44	0	13.68
	597	3.6	1.8	1.08	2.16	8.64
	598	12.96	11.16	11.52	11.88	47.52
	599	4.32	3.96	4.32	3.96	16.56

TOTAL	53.28	52.92	47.88	50.04	204.12

STATE: NEBRASKA

ZIP CODE	QUARTER 1	QUARTER 2	QUARTER 3	QUARTER 4	TOTAL GRAMS
680	12.24	15.48	13.32	11.52	52.56
681	42.48	54.36	50.04	54	200.88
683	2.52	0	0	0	2.52
684	0	0	0	1.08	1.08
685	9.36	5.76	8.64	11.88	35.64
686	0	0.72	0.72	0.72	2.16
687	1.8	6.48	5.4	1.8	15.48
688	16.2	11.88	6.48	14.4	48.96
689	3.96	6.84	3.6	5.4	19.8
690	0	0	0.72	0	0.72
691	0.36	1.8	1.08	1.8	5.04
692	0.72	0	0	0	0.72
693	2.16	2.88	0.36	2.88	8.28
TOTAL	91.8	106.2	90.36	105.48	393.84

STATE: NEVADA

ZIP CODE	QUARTER 1	QUARTER 2	QUARTER 3	QUARTER 4	TOTAL GRAMS
890	11.52	3.24	3.96	9.36	28.08
891	6.48	11.88	16.92	19.08	54.36
894	2.88	1.08	2.88	2.88	9.72
895	8.28	8.64	9	7.2	33.12
897	0.72	2.88	1.8	2.16	7.56
898	0.72	0.36	0.36	0	1.44
TOTAL	30.6	28.08	34.92	40.68	134.28

STATE: NEW HAMPSHIRE

ZIP CODE	QUARTER 1	QUARTER 2	QUARTER 3	QUARTER 4	TOTAL GRAMS
30	16.92	11.88	14.76	18.72	62.28
31	1.44	3.6	0.72	2.16	7.92

	32	0	0.72	0	0.36	1.08
	33	8.28	10.44	9.36	8.28	36.36
	34	0	4.32	0	1.44	5.76
	35	0.72	0.72	1.44	0.72	3.6
	37	2.52	5.04	8.28	7.92	23.76
	38	0.36	0.72	2.88	1.8	5.76
TOTAL		30.24	37.44	37.44	41.4	146.52

STATE: NEW JERSEY

ZIP CODE		QUARTER 1	QUARTER 2	QUARTER 3	QUARTER 4	TOTAL GRAMS
	70	5.76	6.12	1.08	6.12	19.08
	74	2.52	3.96	5.76	3.96	16.2
	76	1.44	8.28	3.6	6.84	20.16
	77	6.48	4.68	2.88	7.56	21.6
	78	2.52	2.52	4.68	2.16	11.88
	79	0	1.44	0.72	0	2.16
	80	47.52	69.48	57.24	47.16	221.4
	81	1.44	0.72	16.56	3.24	21.96
	82	1.08	5.04	0	0.72	6.84
	83	1.44	2.88	3.96	2.88	11.16
	84	0	0	0.36	0	0.36
	85	4.32	3.24	3.6	2.88	14.04
	86	2.16	4.32	1.08	2.52	10.08
	87	3.24	2.16	3.24	2.52	11.16
	88	4.68	12.24	9	15.12	41.04
	89	2.52	6.48	3.96	1.08	14.04
TOTAL		87.12	133.56	117.72	104.76	443.16

STATE: NEW MEXICO

ZIP CODE		QUARTER 1	QUARTER 2	QUARTER 3	QUARTER 4	TOTAL GRAMS
	871	16.56	22.68	10.08	9.72	59.04
	874	1.44	5.76	0	0	7.2
	875	9.36	7.2	6.12	8.64	31.32
	880	7.56	6.48	7.92	7.2	29.16
	881	0	0.36	0	1.8	2.16

	882	2.16	6.48	5.04	0.72	14.4
	883	0.72	0.36	0	0.36	1.44
TOTAL		37.8	49.32	29.16	28.44	144.72

STATE:	NEW YORK				
ZIP CODE	QUARTER 1	QUARTER 2	QUARTER 3	QUARTER 4	TOTAL GRAMS
100	27	54.36	22.32	35.64	139.32
103	0	0.36	0	0.36	0.72
104	0.72	4.32	0.72	6.12	11.88
105	3.6	1.8	0.72	8.64	14.76
108	0	0	1.08	0	1.08
109	0.72	0	0	0.72	1.44
110	16.2	9	8.64	8.28	42.12
111	0	0	1.8	0	1.8
112	2.52	3.6	3.6	3.96	13.68
113	0	0.36	0	0	0.36
114	0	0.36	0	0	0.36
115	2.52	1.8	0.36	3.24	7.92
117	15.48	10.08	15.48	14.4	55.44
118	0.72	1.44	0	0	2.16
119	0.72	0	0	5.04	5.76
120	7.56	3.6	4.68	6.48	22.32
121	0.72	7.92	1.44	0.72	10.8
122	0.72	2.88	6.12	8.64	18.36
123	15.84	5.4	8.28	8.64	38.16
124	0	2.52	1.44	2.52	6.48
125	2.16	0	0	0	2.16
128	0.36	5.76	7.2	3.6	16.92
129	15.84	7.92	6.84	13.68	44.28
130	1.44	6.84	10.08	4.68	23.04
131	0	0.36	0	0.36	0.72
132	7.2	13.68	4.32	12.6	37.8
133	1.44	0.72	3.6	1.44	7.2
134	5.04	5.76	5.04	7.56	23.4
135	5.4	2.88	4.68	2.16	15.12
136	7.92	3.96	3.96	9.72	25.56

	137	0.72	1.08	0.72	0	2.52
	138	0.72	0	0.72	0.36	1.8
	139	2.88	1.44	2.16	2.52	9
	140	1.8	0.36	9	5.76	16.92
	141	0	0	3.6	1.08	4.68
	142	34.92	37.44	37.8	38.52	148.68
	143	0	0	0.72	0	0.72
	144	3.24	1.44	0	1.44	6.12
	145	3.6	2.16	2.88	3.6	12.24
	146	17.28	12.6	16.2	13.32	59.4
	147	0.36	0	0.72	2.16	3.24
	148	0.72	3.24	4.32	1.8	10.08
	149	0.72	0	0.72	1.44	2.88
TOTAL		208.8	217.44	201.96	241.2	869.4

STATE:	NORTH CAROLINA				
ZIP CODE	QUARTER 1	QUARTER 2	QUARTER 3	QUARTER 4	TOTAL GRAMS
270	0.72	3.24	1.44	0.72	6.12
271	15.12	13.32	20.52	14.4	63.36
272	22.32	22.32	17.28	21.24	83.16
273	6.48	6.12	5.76	6.84	25.2
274	8.64	6.48	7.56	5.4	28.08
275	21.96	20.88	19.8	29.88	92.52
276	24.48	14.76	9	19.8	68.04
277	10.08	9.36	10.8	11.52	41.76
278	9.36	7.92	7.56	7.56	32.4
279	5.76	7.2	3.24	5.4	21.6
280	10.44	12.24	16.2	5.76	44.64
281	19.08	24.84	17.28	18	79.2
282	22.32	14.76	21.6	24.12	82.8
283	10.44	6.84	12.24	16.92	46.44
284	19.8	19.8	19.44	22.32	81.36
285	4.68	3.24	7.2	5.04	20.16
286	17.64	11.16	8.64	10.44	47.88
287	8.28	7.92	8.64	12.6	37.44
288	0.72	3.96	5.76	2.16	12.6

	289	0	2.16	0	0.72	2.88
TOTAL		238.32	218.52	219.96	240.84	917.64

STATE:	NORTH DAKOTA					
ZIP CODE		QUARTER 1	QUARTER 2	QUARTER 3	QUARTER 4	TOTAL GRAMS
	580	0.72	0.72	0.36	0	1.8
	581	5.04	14.76	15.84	8.64	44.28
	582	1.44	2.16	3.6	5.04	12.24
	583	0	0	0	1.44	1.44
	584	1.08	1.44	1.8	2.16	6.48
	585	14.76	21.6	22.32	25.2	83.88
	586	3.6	0.72	0	1.08	5.4
	587	2.88	0.72	1.08	1.8	6.48
	588	1.44	0	0.72	0.72	2.88
TOTAL		30.96	42.12	45.72	46.08	164.88

STATE:	OHIO					
ZIP CODE		QUARTER 1	QUARTER 2	QUARTER 3	QUARTER 4	TOTAL GRAMS
	430	15.48	14.04	19.8	19.8	69.12
	431	3.24	2.52	5.4	6.12	17.28
	432	44.28	55.44	27	31.68	158.4
	433	5.04	2.52	3.6	6.48	17.64
	434	6.84	3.24	1.44	1.8	13.32
	435	18	6.12	6.48	6.48	37.08
	436	22.32	10.44	18.36	24.48	75.6
	437	2.88	0.36	2.52	3.6	9.36
	438	0	0.36	0	0	0.36
	439	0	0	1.44	1.08	2.52
	440	11.16	14.4	22.32	18.36	66.24
	441	62.64	71.28	62.28	66.96	263.16
	442	12.6	14.4	15.48	10.08	52.56
	443	15.12	15.84	12.96	13.32	57.24
	444	1.8	14.4	15.12	16.56	47.88
	445	9.72	12.24	11.52	10.8	44.28

	446	3.96	4.32	3.96	2.16	14.4
	447	8.64	8.64	7.92	7.2	32.4
	448	7.2	1.8	3.24	7.2	19.44
	449	6.84	2.16	2.16	0	11.16
	450	5.76	7.56	10.44	11.88	35.64
	451	19.44	26.28	19.44	22.68	87.84
	452	36.72	35.64	43.2	41.4	156.96
	453	6.84	6.48	10.08	11.16	34.56
	454	26.64	29.52	19.8	29.52	105.48
	455	2.88	0.72	3.96	1.44	9
	456	5.4	6.12	16.92	4.68	33.12
	457	0.72	2.52	0.36	2.52	6.12
	458	9.72	10.8	9	6.84	36.36
TOTAL		371.88	380.16	376.2	386.28	1,514.52

STATE: OKLAHOMA

ZIP CODE		QUARTER 1	QUARTER 2	QUARTER 3	QUARTER 4	TOTAL GRAMS
	730	3.96	3.24	7.92	1.8	16.92
	731	31.68	30.24	19.44	19.08	100.44
	734	0	1.44	0	0	1.44
	735	12.6	3.24	4.68	2.52	23.04
	736	0	0.36	0.36	0.36	1.08
	737	0.72	1.08	2.88	0.72	5.4
	738	1.8	3.6	2.88	0	8.28
	740	0.72	8.28	2.52	3.96	15.48
	741	16.2	19.8	26.64	18.36	81
	743	0	3.6	4.68	2.16	10.44
	744	2.52	3.6	8.64	0.36	15.12
	745	0	0	6.48	1.08	7.56
	746	1.44	0	3.96	0.36	5.76
	747	2.88	2.88	2.16	2.88	10.8
	748	0.72	2.16	2.16	1.44	6.48
	749	1.44	0	0	0	1.44
TOTAL		76.68	83.52	95.4	55.08	310.68

STATE: OREGON

ZIP CODE	QUARTER 1	QUARTER 2	QUARTER 3	QUARTER 4	TOTAL GRAMS
970	19.8	25.56	15.48	18	78.84
971	6.48	12.6	10.08	16.92	46.08
972	38.52	50.4	64.8	60.48	214.2
973	22.32	40.68	15.12	14.04	92.16
974	36.36	32.76	49.32	48.6	167.04
975	37.44	36	39.24	44.28	156.96
976	1.8	3.96	0.72	1.8	8.28
977	3.96	10.8	6.48	4.68	25.92
978	1.44	1.8	1.44	1.8	6.48
979	0.36	0.72	0.36	0.72	2.16
TOTAL	168.48	215.28	203.04	211.32	798.12

STATE: PENNSYLVANIA

ZIP CODE	QUARTER 1	QUARTER 2	QUARTER 3	QUARTER 4	TOTAL GRAMS
150	6.48	2.16	4.68	2.88	16.2
151	1.44	0.36	2.52	2.52	6.84
152	9.72	8.28	15.84	10.08	43.92
153	2.52	7.2	3.96	5.04	18.72
154	0.36	0.72	0	0.36	1.44
155	1.08	1.44	1.44	2.16	6.12
156	1.8	1.44	2.52	11.88	17.64
157	2.88	0	1.8	3.6	8.28
158	2.88	3.24	2.16	3.96	12.24
159	0	0.36	0.36	0	0.72
160	0	3.24	1.08	2.16	6.48
161	1.8	0	0.72	5.4	7.92
162	0.72	2.52	1.44	1.08	5.76
163	1.08	1.08	2.52	1.44	6.12
164	0	4.32	0	0	4.32
165	2.88	2.52	4.32	10.08	19.8
166	0.36	2.16	1.08	1.08	4.68
167	1.08	0.36	0	0.36	1.8
168	2.88	7.2	6.48	8.64	25.2
169	0	0	0	0.36	0.36

	170	15.84	24.84	32.4	19.44	92.52
	171	5.4	6.48	6.12	2.88	20.88
	172	11.16	12.24	12.24	12.24	47.88
	173	5.4	5.4	6.48	4.32	21.6
	174	8.64	5.76	1.44	5.04	20.88
	175	0.72	0	1.8	1.8	4.32
	176	5.04	7.56	2.16	5.4	20.16
	177	1.44	1.44	1.44	16.56	20.88
	178	6.84	13.68	15.12	18	53.64
	179	8.64	8.28	8.28	9.72	34.92
	180	23.04	26.28	20.16	20.88	90.36
	181	5.4	4.32	4.32	6.84	20.88
	182	4.32	1.44	1.44	2.88	10.08
	183	1.44	1.8	0	2.16	5.4
	184	0	0.72	3.24	0	3.96
	185	6.84	5.04	8.64	11.16	31.68
	186	3.24	7.56	5.76	3.96	20.52
	187	39.96	20.16	12.6	42.84	115.56
	188	2.16	1.08	2.16	2.88	8.28
	189	1.8	2.52	10.8	2.16	17.28
	190	26.64	21.96	56.88	47.88	153.36
	191	47.52	43.2	33.84	47.52	172.08
	193	12.24	9.36	19.08	14.76	55.44
	194	10.8	7.56	10.8	3.96	33.12
	195	2.16	0.72	4.68	1.8	9.36
	196	0	0	0	0.72	0.72
TOTAL		296.64	288	334.8	380.88	1,300.32

STATE: RHODE ISLAND

ZIP CODE		QUARTER 1	QUARTER 2	QUARTER 3	QUARTER 4	TOTAL GRAMS
	028	12.24	16.56	17.64	12.24	58.68
	029	20.52	17.64	27.36	13.32	78.84
TOTAL		32.76	34.2	45	25.56	137.52

STATE: SOUTH CAROLINA

ZIP CODE	QUARTER 1	QUARTER 2	QUARTER 3	QUARTER 4	TOTAL GRAMS
290	4.32	3.24	3.24	3.24	14.04
291	6.84	8.64	7.2	8.64	31.32
292	2.88	9.36	9.36	3.24	24.84
293	10.44	9	9	6.48	34.92
294	21.96	25.92	21.96	22.68	92.52
295	20.16	32.4	24.84	32.76	110.16
296	30.96	27.72	21.96	25.2	105.84
297	7.92	9.72	4.68	14.4	36.72
298	8.64	7.92	7.92	11.88	36.36
299	13.68	20.88	17.28	16.56	68.4
TOTAL	127.8	154.8	127.44	145.08	555.12

STATE: SOUTH DAKOTA

ZIP CODE	QUARTER 1	QUARTER 2	QUARTER 3	QUARTER 4	TOTAL GRAMS
570	5.04	3.96	4.68	9.36	23.04
571	48.96	36	17.64	27.72	130.32
572	1.08	1.08	0.72	0.72	3.6
573	2.52	1.08	1.44	3.6	8.64
574	1.8	1.08	0.72	0.72	4.32
575	2.16	1.08	0.72	0.36	4.32
577	2.52	2.52	1.08	0.36	6.48
TOTAL	64.08	46.8	27	42.84	180.72

STATE: TENNESSEE

ZIP CODE	QUARTER 1	QUARTER 2	QUARTER 3	QUARTER 4	TOTAL GRAMS
370	12.24	13.32	20.88	11.88	58.32
371	5.04	3.96	3.96	2.52	15.48
372	28.8	28.8	31.32	58.68	147.6
373	4.32	3.6	7.56	4.68	20.16
374	6.48	6.12	12.6	10.8	36
376	17.64	23.4	22.68	17.64	81.36
377	10.08	9	8.28	11.16	38.52
378	6.84	9.36	11.52	11.52	39.24
379	16.56	18.36	14.76	20.52	70.2

	380	2.52	2.88	1.08	1.8	8.28
	381	42.12	53.64	38.52	35.64	169.92
	382	0.72	2.52	1.8	1.8	6.84
	383	12.96	11.88	6.48	9	40.32
	384	1.44	1.8	2.88	4.32	10.44
	385	3.24	3.96	6.48	3.6	17.28
TOTAL		171	192.6	190.8	205.56	759.96

STATE: TEXAS

ZIP CODE	QUARTER 1	QUARTER 2	QUARTER 3	QUARTER 4	TOTAL GRAMS
750	50.4	41.4	43.92	56.16	191.88
751	1.44	9.72	7.56	3.6	22.32
752	61.2	63.36	68.4	76.68	269.64
753	10.08	20.52	9.36	12.96	52.92
754	3.24	1.8	2.16	3.6	10.8
755	3.96	5.04	5.4	5.76	20.16
756	9.36	6.84	10.8	13.32	40.32
757	24.84	21.6	30.96	18.72	96.12
758	0	0.72	0	0	0.72
759	10.08	12.96	10.44	12.96	46.44
760	32.76	21.24	39.6	34.2	127.8
761	25.92	28.8	24.48	27.72	106.92
762	9.72	8.28	8.28	14.4	40.68
763	6.12	3.6	1.44	3.6	14.76
764	1.44	0	0	0.36	1.8
765	6.84	14.4	11.88	12.24	45.36
766	0	0.72	1.8	2.16	4.68
767	5.04	4.32	3.6	6.48	19.44
768	0.72	0.72	1.44	0	2.88
769	2.52	3.6	1.08	1.8	9
770	55.44	54	59.04	51.12	219.6
773	5.04	6.12	1.8	4.32	17.28
774	6.48	5.04	7.56	10.8	29.88
775	26.64	24.84	27.36	21.96	100.8
776	1.08	1.44	0.72	0	3.24
777	1.08	0.72	1.8	2.16	5.76

	778	4.32	10.44	2.16	5.4	22.32
	779	5.4	1.08	3.6	3.24	13.32
	780	1.44	0	0.36	0.72	2.52
	781	4.32	3.24	2.16	1.44	11.16
	782	40.32	36.72	33.12	47.88	158.04
	784	1.8	3.6	0.36	1.8	7.56
	785	3.24	3.24	1.8	2.16	10.44
	786	17.28	20.88	18.36	21.6	78.12
	787	30.24	34.92	30.6	36	131.76
	788	0.72	0.72	0	1.8	3.24
	789	0	0.36	0	1.08	1.44
	790	2.88	2.16	2.88	2.88	10.8
	791	14.76	10.8	18.72	16.2	60.48
	792	0	0	0	0.72	0.72
	794	9	7.56	5.76	18.36	40.68
	796	1.08	1.08	1.8	2.52	6.48
	797	0	1.44	2.16	3.6	7.2
	799	5.4	4.32	8.64	2.88	21.24
TOTAL		503.64	504.36	513.36	567.36	2,088.72

STATE:	UTAH					
ZIP CODE		QUARTER 1	QUARTER 2	QUARTER 3	QUARTER 4	TOTAL GRAMS
	840	23.04	29.88	28.44	23.4	104.76
	841	29.52	23.4	20.52	28.44	101.88
	843	1.08	0.36	0.36	0.72	2.52
	844	2.16	1.44	2.16	2.16	7.92
	846	4.68	2.88	8.28	5.4	21.24
	847	7.2	6.12	2.88	6.12	22.32
TOTAL		67.68	64.08	62.64	66.24	260.64

STATE:	VERMONT					
ZIP CODE		QUARTER 1	QUARTER 2	QUARTER 3	QUARTER 4	TOTAL GRAMS
	50	5.4	1.8	1.08	1.8	10.08
	51	9	0	0	0	9
	53	0	0.36	0.36	0.36	1.08

	54	14.4	21.6	25.2	30.24	91.44
	56	4.32	9	5.04	2.88	21.24
	57	7.92	7.2	5.04	8.28	28.44
	58	0.36	0	0.72	0.36	1.44
TOTAL		41.4	39.96	37.44	43.92	162.72

STATE: VIRGIN ISLANDS

ZIP CODE		QUARTER 1	QUARTER 2	QUARTER 3	QUARTER 4	TOTAL GRAMS
	8	0	0	1.8	0	1.8
TOTAL		0	0	1.8	0	1.8

STATE: VIRGINIA

ZIP CODE		QUARTER 1	QUARTER 2	QUARTER 3	QUARTER 4	TOTAL GRAMS
	201	14.04	18	28.08	18.72	78.84
	220	21.24	10.08	12.24	20.88	64.44
	221	5.04	9	7.92	6.84	28.8
	222	2.52	2.88	1.44	4.68	11.52
	223	0.72	2.52	3.6	3.24	10.08
	224	1.8	1.8	2.16	1.44	7.2
	225	1.08	0.72	1.44	0	3.24
	226	1.44	1.44	2.16	1.44	6.48
	227	0	0.36	0	0	0.36
	228	3.6	1.8	3.6	7.2	16.2
	229	19.08	26.28	32.4	16.56	94.32
	230	5.04	0	5.04	0	10.08
	231	9	5.4	11.88	8.28	34.56
	232	23.4	30.24	16.92	18.72	89.28
	233	5.04	3.6	10.8	5.4	24.84
	234	9.72	9.36	11.52	11.88	42.48
	235	5.76	3.6	8.28	8.28	25.92
	236	3.6	9.72	4.68	4.68	22.68
	237	9.72	10.8	10.44	9.36	40.32
	238	0	2.16	1.8	0	3.96
	239	0.36	0	0.72	0.72	1.8
	240	4.32	10.44	5.04	11.16	30.96

	241	1.8	6.12	3.96	1.08	12.96
	242	14.76	1.8	1.8	2.52	20.88
	243	0	0.72	0.36	0.36	1.44
	244	0	0	0	0.36	0.36
	245	7.56	6.48	11.16	10.44	35.64
	246	0	2.88	0	0.36	3.24
TOTAL		170.64	178.2	199.44	174.6	722.88

STATE: WASHINGTON

ZIP CODE		QUARTER 1	QUARTER 2	QUARTER 3	QUARTER 4	TOTAL GRAMS
	980	31.68	25.2	34.92	37.08	128.88
	981	77.76	84.24	118.44	111.24	391.68
	982	22.32	33.12	30.24	20.16	105.84
	983	34.56	35.28	31.68	43.92	145.44
	984	21.24	41.76	26.28	26.64	115.92
	985	14.4	16.56	10.8	13.32	55.08
	986	16.2	19.44	23.76	17.64	77.04
	988	33.48	32.04	18.72	28.08	112.32
	989	1.44	0.72	1.8	3.24	7.2
	990	0	0.36	0	0.72	1.08
	991	3.6	2.88	4.32	3.24	14.04
	992	17.28	17.64	10.08	16.56	61.56
	993	7.92	11.16	12.96	7.2	39.24
	994	0	0.72	1.44	0	2.16
TOTAL		281.88	321.12	325.44	329.04	1,257.48

STATE: WEST VIRGINIA

ZIP CODE		QUARTER 1	QUARTER 2	QUARTER 3	QUARTER 4	TOTAL GRAMS
	247	1.44	1.8	1.44	0.72	5.4
	249	7.2	6.48	6.48	6.48	26.64
	250	0	1.08	0.72	0.36	2.16
	252	2.88	2.16	1.44	2.16	8.64
	253	22.32	33.48	27.36	33.84	117
	254	1.08	0.72	0	1.44	3.24

	255	5.76	3.6	2.88	3.24	15.48
	256	0	0	2.52	2.16	4.68
	257	17.28	18	19.08	18.72	73.08
	258	2.16	7.2	4.32	5.04	18.72
	259	11.16	10.8	10.8	10.8	43.56
	260	1.8	3.6	1.08	2.52	9
	261	0.72	1.44	0	2.16	4.32
	262	2.52	1.44	1.08	2.52	7.56
	263	1.44	11.16	3.96	0.72	17.28
	264	0	0	1.44	0	1.44
	265	3.24	7.92	2.16	5.4	18.72
	266	3.6	0	0.36	0	3.96
TOTAL		84.6	110.88	87.12	98.28	380.88

STATE: WISCONSIN

ZIP CODE		QUARTER 1	QUARTER 2	QUARTER 3	QUARTER 4	TOTAL GRAMS
	530	21.96	27.72	15.12	21.24	86.04
	531	6.84	12.6	25.92	13.68	59.04
	532	34.56	47.88	38.52	33.48	154.44
	534	1.44	1.44	2.88	1.44	7.2
	535	15.84	7.92	14.04	17.28	55.08
	537	30.6	50.76	52.92	38.16	172.44
	538	0	0.36	0.36	2.52	3.24
	539	3.96	6.84	12.96	7.56	31.32
	540	2.16	4.32	2.16	2.88	11.52
	541	1.08	0	2.88	9.36	13.32
	542	2.52	2.16	1.44	3.96	10.08
	543	6.84	13.68	10.8	12.24	43.56
	544	7.92	6.12	7.2	10.08	31.32
	545	6.84	5.04	5.04	2.52	19.44
	546	9	13.68	9.72	9.36	41.76
	547	20.16	7.92	11.16	7.2	46.44
	548	0.72	2.52	6.84	3.24	13.32
	549	10.8	12.96	19.08	12.24	55.08
TOTAL		183.24	223.92	239.04	208.44	854.64

STATE: WYOMING

ZIP CODE	QUARTER 1	QUARTER 2	QUARTER 3	QUARTER 4	TOTAL GRAMS
820	11.52	4.32	2.16	5.76	23.76
822	1.8	0	0	0	1.8
824	0	0.36	1.08	0	1.44
825	0	1.44	0	0.72	2.16
826	9.36	15.48	15.12	18.36	58.32
827	0.72	1.44	0	0.36	2.52
828	1.44	0.72	1.44	1.44	5.04
829	0.72	0.36	0	0.72	1.8
830	0	1.44	0	0	1.44
TOTAL	25.56	25.56	19.8	27.36	98.28

DRUG CODE:9652DRUG NAME:OXYMORPHONE

STATE: ALABAMA

ZIP CODE	QUARTER 1	QUARTER 2	QUARTER 3	QUARTER 4	TOTAL GRAMS
350	766.22	643.61	345.64	209.38	1,964.85
351	398.99	381.24	241.13	163.3	1,184.66
352	386.81	329.5	155.23	92.87	964.41
354	195.41	206.48	136.94	107.32	646.15
355	256.43	234.35	175.88	105.13	771.79
356	539.77	458.09	264.24	208.17	1,470.27
357	453.77	398.77	252.69	163.43	1,268.66
358	775.86	644.54	374.64	236.14	2,031.18
359	998.04	887.28	554.66	451.67	2,891.65
360	226.33	162.32	83.9	56.65	529.2
361	120.21	98.76	73.33	36.49	328.79
362	507.87	368.66	221.24	215	1,312.77
363	143.54	148.45	78.24	82.25	452.48
364	30.96	46.84	35.86	15.61	129.27
365	799.95	748.34	416.21	290.2	2,254.70
366	685.89	652.94	410.27	259.69	2,008.79
367	105.36	108.88	84.39	51.74	350.37
368	254.34	233.42	185.47	169.86	843.09

	369	28.9	29.84	18.64	23.37	100.75
TOTAL		7,674.65	6,782.31	4,108.60	2,938.27	21,503.83

STATE:	ALASKA					
ZIP CODE		QUARTER 1	QUARTER 2	QUARTER 3	QUARTER 4	TOTAL GRAMS
	995	104.82	115.66	104.64	68.96	394.08
	996	158.53	152.37	126.5	105.71	543.11
	997	19.8	16.41	11.06	7.14	54.41
	998	0	0.27	0	0	0.27
	999	4.28	2.14	4.28	2.14	12.84
TOTAL		287.43	286.85	246.48	183.95	1,004.71

STATE:	ARIZONA					
ZIP CODE		QUARTER 1	QUARTER 2	QUARTER 3	QUARTER 4	TOTAL GRAMS
	850	1,407.92	1,351.17	918.38	554.62	4,232.09
	851	411.12	359.07	217.4	133.28	1,120.87
	852	2,613.78	2,276.95	1,355.14	778.4	7,024.27
	853	1,484.68	1,308.44	810.43	566.93	4,170.48
	855	182.66	187.43	147.2	75.29	592.58
	856	282.08	220.44	157.68	85.95	746.15
	857	1,284.95	1,100.94	762.28	359.36	3,507.53
	859	106.25	85.78	49.6	13.07	254.7
	860	105.09	63.03	60.44	31.4	259.96
	863	330.57	277.93	192.25	127.39	928.14
	864	393.46	264.46	173.47	82.7	914.09
TOTAL		8,602.56	7,495.64	4,844.27	2,808.39	23,750.86

STATE:	ARKANSAS					
ZIP CODE		QUARTER 1	QUARTER 2	QUARTER 3	QUARTER 4	TOTAL GRAMS
	716	51.12	60.26	50.4	33.99	195.77
	717	52.63	64.37	39.39	19.85	176.24
	718	25.87	30.96	18.2	5	80.03
	719	112.58	86.49	65.3	36.84	301.21
	720	335.25	359.2	270.22	157.81	1,122.48

	721	270.93	252.69	170.3	154.78	848.7
	722	177.17	133.64	102.5	66.37	479.68
	723	56.02	51.03	33.19	26.85	167.09
	724	98.67	83.1	64.5	23.19	269.46
	725	90.33	69.94	60.4	33.1	253.77
	726	56.34	37.56	33.45	12.27	139.62
	727	452.74	494.45	326.95	187.52	1,461.66
	728	62.63	51.25	30.33	18.64	162.85
	729	436.73	410.81	286.72	80.2	1,214.46
TOTAL		2,279.01	2,185.75	1,551.85	856.41	6,873.02

STATE: CALIFORNIA

ZIP CODE		QUARTER 1	QUARTER 2	QUARTER 3	QUARTER 4	TOTAL GRAMS
	900	538.39	594.28	510.47	428.3	2,071.44
	902	425.89	383.69	252.29	199.16	1,261.03
	903	134.89	28.37	41.22	44.07	248.55
	904	57.9	71.1	68.87	63.07	260.94
	905	15.61	25.78	32.83	15.88	90.1
	906	104.78	91.98	69.49	51.03	317.28
	907	175.92	166.2	137.78	78.86	558.76
	908	122.66	83.14	79.13	74.22	359.15
	910	68.25	90.37	48.35	20.7	227.67
	911	132.03	130.43	101.88	114.55	478.89
	912	24.98	26.85	31.58	25.78	109.19
	913	573.34	427.81	356.66	315.18	1,672.99
	914	132.48	134.66	152.46	136.13	555.73
	915	35.06	36.22	20.25	8.47	100
	916	39.07	25.42	29.66	25.07	119.22
	917	608.38	483.61	255.77	234	1,581.76
	918	21.41	9.28	18.91	3.57	53.17
	919	226.95	216.74	170.88	80.96	695.53
	920	1,629.17	1,605.20	1,000.13	996.39	5,230.89
	921	678.71	697.85	712.3	372.36	2,461.22
	922	360.72	324.5	241.85	174.41	1,101.48
	923	419.11	287.48	227.49	142.07	1,076.15
	924	112.44	101.61	63.43	59.41	336.89

	925	282.13	315.89	158.17	98.76	854.95
	926	777.32	705.98	526.53	420.71	2,430.54
	927	113.07	128.6	81.63	44.07	367.37
	928	539.99	483.61	518.89	368.53	1,911.02
	930	383.12	376.73	262.19	241.76	1,263.80
	931	24.27	28.55	21.41	2.23	76.46
	932	91.67	117.8	82.88	54.6	346.95
	933	92.89	113.64	44.96	17.75	269.24
	934	219.55	201.35	189.04	131.67	741.61
	935	364.07	352.29	171.06	92.56	979.98
	936	471.56	409.56	339.53	255.77	1,476.42
	937	446.59	524.06	377.72	311.16	1,659.53
	939	133.06	104.29	60.13	42.64	340.12
	940	415.91	412.72	309.02	190.64	1,328.29
	941	314.65	249.07	185.33	110.98	860.03
	943	21.41	31.22	10.53	7.58	70.74
	944	117.25	74.13	67.44	49.6	308.42
	945	2,027.44	1,719.56	1,268.61	660.38	5,675.99
	946	39.79	24.18	11.69	0	75.66
	947	30.87	16.68	14.5	6.42	68.47
	948	35.77	22.84	16.15	3.48	78.24
	949	148.86	157.9	78.15	74.22	459.13
	950	285.63	307.43	217.98	112.14	923.18
	951	113.3	114.23	59.64	32.96	320.13
	952	199.96	157.37	143.18	70.83	571.34
	953	564.62	520.76	353.36	153.44	1,592.18
	954	301.35	287.69	224.54	164.41	977.99
	955	34.97	36.58	40.95	27.48	139.98
	956	648.24	610.62	362.82	214.24	1,835.92
	957	112.14	100.09	87.78	51.03	351.04
	958	377.53	344.32	177.48	98.22	997.55
	959	390.07	311.74	235.96	188.68	1,126.45
	960	259.82	191.8	69.9	48.44	569.96
	961	73.46	63.96	43.04	17.84	198.3
TOTAL		17,090.47	15,659.81	11,435.87	8,028.86	52,215.01

STATE: COLORADO

ZIP CODE	QUARTER 1	QUARTER 2	QUARTER 3	QUARTER 4	TOTAL GRAMS
800	654.76	573.13	372.18	226.64	1,826.71
801	714.88	663.39	543.51	388.33	2,310.11
802	839.56	776.44	522.41	412.33	2,550.74
803	139.02	102.59	76.72	45.32	363.65
804	130.07	103.93	87.38	21.41	342.79
805	574.22	449.71	408.27	271.42	1,703.62
806	222.77	172.71	101.79	75.11	572.38
807	42.29	39.07	27.74	12.98	122.08
808	165.22	145.37	127.66	56.29	494.54
809	467.28	450.56	355.9	206.61	1,480.35
810	217.53	168.29	119.01	49.33	554.16
811	9.63	8.03	6.78	6.78	31.22
812	40.77	29.17	35.33	27.12	132.39
813	7.4	7.58	1.78	0	16.76
814	1.61	2.41	2.68	2.68	9.38
815	87.52	80.38	54.95	19.36	242.21
816	26.67	21.41	11.69	9.28	69.05
TOTAL	4,341.20	3,794.17	2,855.78	1,830.99	12,822.14

STATE: CONNECTICUT

ZIP CODE	QUARTER 1	QUARTER 2	QUARTER 3	QUARTER 4	TOTAL GRAMS
60	463.59	409.47	285.61	226.64	1,385.31
61	120.61	118.92	72.62	32.92	345.07
62	77.7	63.7	44.52	27.83	213.75
63	256.21	251.57	141.93	78.68	728.39
64	1,167.82	1,086.35	935.9	525.85	3,715.92
65	451.32	386.42	292.81	214.95	1,345.50
66	222.85	219.32	163.08	140.33	745.58
67	275.88	251.97	127.93	100.72	756.5
68	369.55	341.72	218.07	167.18	1,096.52
69	50.31	31.94	37.83	42.29	162.37
TOTAL	3,455.84	3,161.38	2,320.30	1,557.39	10,494.91

STATE: DELAWARE

ZIP CODE	QUARTER 1	QUARTER 2	QUARTER 3	QUARTER 4	TOTAL GRAMS
197	875.86	898.61	838.75	670.86	3,284.08
198	518.89	480.04	329.63	323.3	1,651.86
199	759.99	809.05	675.28	545.39	2,789.71
TOTAL	2,154.74	2,187.70	1,843.66	1,539.55	7,725.65

STATE: DISTRICT OF COLUMBIA

ZIP CODE	QUARTER 1	QUARTER 2	QUARTER 3	QUARTER 4	TOTAL GRAMS
200	120.14	126.32	101.43	130.83	478.72
204	0	0.54	0	0	0.54
TOTAL	120.14	126.86	101.43	130.83	479.26

STATE: FLORIDA

ZIP CODE	QUARTER 1	QUARTER 2	QUARTER 3	QUARTER 4	TOTAL GRAMS
320	1,001.25	907.53	662.25	566.22	3,137.25
321	575.06	557.43	307.28	205.9	1,645.67
322	2,044.56	1,938.40	1,375.75	970.25	6,328.96
323	400.91	393.59	365.81	288.91	1,449.22
324	521.66	482.31	398.28	266.96	1,669.21
325	271.33	235.34	176.46	101.7	784.83
326	588.3	583.83	433.43	340.69	1,946.25
327	1,451.05	1,318.30	947.77	676.43	4,393.55
328	716.62	682.01	518.85	338.51	2,255.99
329	2,066.77	1,905.48	1,470.89	1,009.19	6,452.33
330	1,633.77	1,642.09	1,086.80	553.9	4,916.56
331	672.06	668.32	498.15	247.83	2,086.36
333	883.54	902.31	871.14	660.56	3,317.55
334	2,195.67	2,235.57	1,730.54	1,358.18	7,519.96
335	1,142.78	1,129.89	774.79	404.26	3,451.72
336	1,261.43	1,164.28	805.03	477.54	3,708.28
337	1,631.78	1,505.98	1,133.68	587.89	4,859.33
338	465.54	415.5	260.45	203.22	1,344.71
339	1,206.74	1,132.74	669.48	415	3,423.96
341	519.48	451.18	220.53	107.23	1,298.42

	342	1,783.71	1,655.56	1,047.50	826.17	5,312.94
	344	632.94	560.33	399.17	202.24	1,794.68
	346	2,444.67	2,331.55	1,814.09	1,127.97	7,718.28
	347	701.01	640.35	474.86	340.51	2,156.73
	349	1,225.57	1,077.48	963.11	638.48	3,904.64
TOTAL		28,038.20	26,517.35	19,406.09	12,915.74	86,877.38

STATE: GEORGIA

ZIP CODE		QUARTER 1	QUARTER 2	QUARTER 3	QUARTER 4	TOTAL GRAMS
	300	1,323.54	1,310.15	864.22	649.85	4,147.76
	301	1,379.01	1,353.85	1,073.20	704.8	4,510.86
	302	844.27	724.39	441.63	324.86	2,335.15
	303	364.16	310	246.67	171.73	1,092.56
	304	184.04	187.79	87.96	62.27	522.06
	305	578.71	609.57	402.29	273.35	1,863.92
	306	367.01	330.21	243.28	158.44	1,098.94
	307	327.45	298.59	242.83	139.26	1,008.13
	308	78.86	69.32	65.57	43.89	257.64
	309	126.99	129.93	72.3	63.07	392.29
	310	517.24	452.79	326.95	196.98	1,493.96
	312	178.69	151.66	80.56	53.97	464.88
	313	447.43	473.21	262.9	121.86	1,305.40
	314	350.95	395.2	140.24	53.17	939.56
	315	259.29	249.79	150.63	102.86	762.57
	316	627.37	602.44	236.54	145.68	1,612.03
	317	249.25	240.29	223.87	142.2	855.61
	318	16.86	26.32	6.07	7.32	56.57
	319	285.25	294.08	189.3	162.54	931.17
	398	58.43	40.32	46.08	24.89	169.72
TOTAL		8,564.80	8,249.90	5,403.09	3,602.99	25,820.78

STATE: HAWAII

ZIP CODE		QUARTER 1	QUARTER 2	QUARTER 3	QUARTER 4	TOTAL GRAMS
	967	461.53	481.2	262.81	241.85	1,447.39

	968	216.6	218.56	132.03	131.67	698.86
TOTAL		678.13	699.76	394.84	373.52	2,146.25

STATE: IDAHO

ZIP CODE		QUARTER 1	QUARTER 2	QUARTER 3	QUARTER 4	TOTAL GRAMS
	832	88.5	80.16	62.05	47.91	278.62
	833	186.05	184.26	161.56	115.88	647.75
	834	72.35	84.84	76.19	55.04	288.42
	835	34.3	31.22	22.66	28.73	116.91
	836	647.31	562.74	414.65	326.33	1,951.03
	837	453.91	456.5	341.85	266.11	1,518.37
	838	104.61	92.38	95.9	51.12	344.01
TOTAL		1,587.03	1,492.10	1,174.86	891.12	5,145.11

STATE: ILLINOIS

ZIP CODE		QUARTER 1	QUARTER 2	QUARTER 3	QUARTER 4	TOTAL GRAMS
	600	1,299.79	1,254.87	990.1	655.78	4,200.54
	601	507.78	473.88	403.59	289.66	1,674.91
	602	52.19	43.71	41.35	21.28	158.53
	603	49.96	41.48	38.18	27.21	156.83
	604	789.37	716.8	490.3	323.92	2,320.39
	605	540.61	480.4	319.15	235.29	1,575.45
	606	447.99	447.74	334.98	255.01	1,485.72
	607	33.54	20.34	15.7	29.08	98.66
	608	30.51	19.98	19.54	11.78	81.81
	609	89.03	47.82	33.72	16.95	187.52
	610	46.6	52.14	38.18	30.51	167.43
	611	127.66	128.02	126.86	40.86	423.4
	612	40.1	43.94	46.43	16.28	146.75
	613	67.18	70.12	58.34	46.21	241.85
	614	14.51	13.78	26.23	1.07	55.59
	615	10.19	12.6	9.1	6.6	38.49
	616	31.95	42.5	21.59	19.8	115.84
	617	117.85	95.54	52.54	25.16	291.09
	618	264.15	200.32	107.54	30.33	602.34

	619	57.54	50.27	23.51	4.55	135.87
	620	154.51	185.65	71.9	39.88	451.94
	622	169.41	152.82	108.35	76.27	506.85
	623	30.96	24.71	18.91	18.47	93.05
	624	43.09	35.42	33.01	20.7	132.22
	625	82.61	81.98	32.12	10.35	207.06
	626	55.22	65.75	47.99	21.68	190.64
	627	13.02	26.76	33.81	20.61	94.2
	628	30.51	22.39	7.14	7.32	67.36
	629	106.47	132.92	88.5	67.35	395.24
TOTAL		5,304.30	4,984.65	3,638.66	2,369.96	16,297.57

STATE: INDIANA

ZIP CODE		QUARTER 1	QUARTER 2	QUARTER 3	QUARTER 4	TOTAL GRAMS
	460	385.88	341.92	274.72	137.65	1,140.17
	461	640.88	466.57	337.12	259.51	1,704.08
	462	1,091.20	871.85	654.8	574.11	3,191.96
	463	551.06	507.56	359.61	283.29	1,701.52
	464	2.68	0	0	0	2.68
	465	537.76	528.88	376.29	254.07	1,697
	466	102.5	92.51	44.16	34.17	273.34
	467	181.68	166.87	85.37	43.09	477.01
	468	243.45	196.84	114.23	60.75	615.27
	469	289.65	248	142.78	79.13	759.56
	470	151.57	116.33	94.38	69.94	432.22
	471	446.18	374.77	407.42	121.59	1,349.96
	472	158.88	138.54	93.94	45.68	437.04
	473	286.5	254.43	184.93	115.88	841.74
	474	227.08	191.04	140.51	89.12	647.75
	475	176.9	177.44	114.37	90.37	559.08
	476	144.97	126.05	101.57	75.11	447.7
	477	447.92	392.35	295.95	178.87	1,315.09
	478	408.05	369.86	243.28	215.53	1,236.72
	479	200.39	213.7	149.2	74.85	638.14
TOTAL		6,675.18	5,775.51	4,214.63	2,802.71	19,468.03

STATE:	IOWA				
ZIP CODE	QUARTER 1	QUARTER 2	QUARTER 3	QUARTER 4	TOTAL GRAMS
500	128.74	125.8	91.53	63.96	410.03
501	100.54	119.59	77.35	51.92	349.4
502	169.37	146.57	120.08	102.06	538.08
503	439.76	359.38	262.41	144.79	1,206.34
504	54.51	43.53	40.14	30.87	169.05
505	21.5	19.98	11.42	13.74	66.64
506	20.07	19.67	11.11	2.01	52.86
507	10.71	4.95	1.07	0	16.73
508	3.39	2.68	3.12	2.68	11.87
510	21.41	25.96	29.17	16.24	92.78
511	19.98	21.9	7.85	9.86	59.59
512	1.07	0.54	0.8	0.27	2.68
513	3.48	3.39	2.14	1.96	10.97
514	6.96	7.49	2.14	0.27	16.86
515	110.13	128.91	66.19	44.43	349.66
516	6.51	2.5	2.59	0	11.6
520	32.03	28.46	46.03	3.21	109.73
521	0	1.07	0	0	1.07
522	13.56	14.32	7.14	2.85	37.87
523	23.73	25.16	27.57	23.46	99.92
524	21.23	27.92	27.97	12.31	89.43
525	50.63	35.15	21.86	19.09	126.73
526	15.52	12.58	2.41	3.21	33.72
527	36.95	29.8	14.63	1.69	83.07
528	64.05	43	34.52	19	160.57
TOTAL	1,375.83	1,250.30	911.24	569.88	4,107.25

STATE:	KANSAS				
ZIP CODE	QUARTER 1	QUARTER 2	QUARTER 3	QUARTER 4	TOTAL GRAMS
660	708.15	570.94	347.38	293.59	1,920.06
661	107.68	94.65	88.59	103.93	394.85
662	490.93	437.58	314.96	195.33	1,438.80

	664	49.15	40.14	44.78	32.03	166.1
	665	18.02	18.38	20.16	14.1	70.66
	666	140.51	131.18	90.1	39.07	400.86
	667	44.07	39.97	38.09	21.05	143.18
	668	25.78	23.82	21.95	13.38	84.93
	669	18.56	17.75	9.9	6.87	53.08
	670	147.64	195.82	129.18	88.14	560.78
	671	52.9	57.36	37.91	37.47	185.64
	672	384	407.29	292.61	114.95	1,198.85
	673	41.66	34.7	23.55	16.5	116.41
	674	50.05	35.42	27.74	19.45	132.66
	675	89.21	97.51	72.71	69.49	328.92
	676	32.38	29.17	19.27	20.16	100.98
	677	9.55	12.67	10.35	2.5	35.07
	678	29.8	39.43	33.45	24.53	127.21
	679	18.2	23.64	8.3	1.87	52.01
TOTAL		2,458.24	2,307.42	1,630.98	1,114.41	7,511.05

STATE:	KENTUCKY					
ZIP CODE		QUARTER 1	QUARTER 2	QUARTER 3	QUARTER 4	TOTAL GRAMS
	400	107.59	93.63	49.02	26.36	276.6
	401	94.74	111.16	77.43	74.94	358.27
	402	610.06	530.93	355.64	244.3	1,740.93
	403	319.28	339.58	279.99	254.83	1,193.68
	404	240.47	181.77	145.01	108.43	675.68
	405	217	203.53	173.65	164.64	758.82
	406	31.76	15.79	13.02	6.82	67.39
	407	363.49	359.65	315.27	259.42	1,297.83
	408	51.79	47.64	20.79	14.27	134.49
	409	180.96	166.06	110.89	95.72	553.63
	410	562.47	513.54	356.57	189.71	1,622.29
	411	140.06	146.71	95.01	79.49	461.27
	412	13.2	13.56	7.49	7.49	41.74
	413	19.36	25.34	18.29	6.42	69.41
	414	6.96	7.49	1.61	0.54	16.6
	415	229.05	258.89	121.95	88.41	698.3

	416	74.31	78.15	64.63	79.04	296.13
	417	58.12	62.58	29.71	13.78	164.19
	418	7.49	2.85	2.85	1.78	14.97
	420	197.42	185.65	124.27	54.51	561.85
	421	241.67	240.02	187.79	130.96	800.44
	422	207.9	251.17	197.82	154.33	811.22
	423	120.83	136.22	77.61	38.27	372.93
	424	125.96	115.97	69.32	52.28	363.53
	425	75.52	83.46	59.19	55.31	273.48
	426	34.17	40.77	28.37	14.81	118.12
	427	222.49	207.59	150.14	139.12	719.34
TOTAL		4,554.12	4,419.70	3,133.33	2,355.98	14,463.13

STATE: LOUISIANA

ZIP CODE		QUARTER 1	QUARTER 2	QUARTER 3	QUARTER 4	TOTAL GRAMS
	700	1,110.98	978.41	690.57	633.39	3,413.35
	701	154.15	163.34	188.86	164.68	671.03
	703	350.77	326.82	221.69	209.55	1,108.83
	704	1,352.20	1,186.09	839.24	684.91	4,062.44
	705	649.94	662.34	534.19	445.51	2,291.98
	706	86.09	64.5	47.91	23.02	221.52
	707	1,973.73	1,797.40	1,381.60	1,211.56	6,364.29
	708	1,183.77	1,180.34	950.76	803.2	4,118.07
	710	232.12	227.26	135.42	77.97	672.77
	711	792.54	791.16	551.09	344.13	2,478.92
	712	68.96	59.77	71.19	27.57	227.49
	713	156.07	141.75	98.84	60.57	457.23
	714	94.12	84.88	44.96	23.28	247.24
TOTAL		8,205.44	7,664.06	5,756.32	4,709.34	26,335.16

STATE: MAINE

ZIP CODE		QUARTER 1	QUARTER 2	QUARTER 3	QUARTER 4	TOTAL GRAMS
	39	5.35	1.96	0.89	1.07	9.27
	40	285.25	241.76	190.86	120.88	838.75
	41	12.12	12.04	12.4	12.22	48.78

	42	210.98	196.53	151.17	113.43	672.11
	43	310.45	237.74	262.86	225.43	1,036.48
	44	28.55	28.9	21.68	10.97	90.1
	45	62.18	57.81	64.23	40.59	224.81
	46	3.75	3.48	4.1	3.03	14.36
	47	21.68	14.18	4.55	1.07	41.48
	48	11.51	12.49	5.08	6.42	35.5
	49	238.28	198.98	139.97	109.59	686.82
TOTAL		1,190.10	1,005.87	857.79	644.7	3,698.46

STATE: MARYLAND

ZIP CODE		QUARTER 1	QUARTER 2	QUARTER 3	QUARTER 4	TOTAL GRAMS
	206	1,196.44	1,246.53	954.06	901.24	4,298.27
	207	813.82	757.71	662.92	583.97	2,818.42
	208	874.08	775.73	617.82	465.1	2,732.73
	209	106.43	107.32	85.91	39.88	339.54
	210	2,294.44	2,114.81	1,505.02	1,268.70	7,182.97
	211	961.42	929.57	719.52	647.62	3,258.13
	212	1,347.29	1,297.96	932.74	770.57	4,348.56
	214	265.22	252.87	189.39	166.29	873.77
	215	419.02	342.39	293.41	249.65	1,304.47
	216	306.44	289.49	238.99	214.59	1,049.51
	217	2,173.91	1,994.47	1,652.75	1,362.50	7,183.63
	218	211.87	183.95	102.41	69.14	567.37
	219	316.07	308.93	242.83	224.94	1,092.77
TOTAL		11,286.45	10,601.73	8,197.77	6,964.19	37,050.14

STATE: MASSACHUSETTS

ZIP CODE		QUARTER 1	QUARTER 2	QUARTER 3	QUARTER 4	TOTAL GRAMS
	10	119.72	110.62	84.21	41.39	355.94
	11	11.6	16.24	10.08	1.78	39.7
	12	31.05	36.93	33.72	19.98	121.68
	13	25.92	36.62	28.37	22.03	112.94
	14	34.44	21.59	12.58	11.24	79.85
	15	131.7	110.44	104.73	78.77	425.64

16	35.15	31.4	41.13	12.04	119.72
17	91.57	103.57	64.23	10.71	270.08
18	1,214.15	1,114.01	658.24	275.44	3,261.84
19	77.97	91.57	92.29	80.65	342.48
20	68.44	62.54	82.56	59.59	273.13
21	119.43	99.99	111.29	109.1	439.81
22	106.52	103.57	21.59	36.75	268.43
23	71.33	98.09	78.77	47.82	296.01
24	94.29	117.13	77.08	30.6	319.1
25	22.21	30.26	19.89	22.21	94.57
26	42.73	40.23	36.75	28.64	148.35
27	564.85	505.64	411.08	250.32	1,731.89
TOTAL	2,863.07	2,730.44	1,968.59	1,139.06	8,701.16

STATE: MICHIGAN

ZIP CODE	QUARTER 1	QUARTER 2	QUARTER 3	QUARTER 4	TOTAL GRAMS
480	4,027.62	4,084.88	3,818.99	3,537.09	15,468.58
481	2,854.81	3,454.70	2,964.81	2,769.35	12,043.67
482	8,564.16	8,751.14	7,608.54	7,069.62	31,993.46
483	2,168.25	1,834.47	1,612.87	1,351.89	6,967.48
484	275.93	236.94	181.81	150.5	845.18
485	292.92	243.59	202.46	125.47	864.44
486	197.87	176.9	126.86	91.62	593.25
487	128.28	120.17	64.19	44.84	357.48
488	214.36	220.3	179.4	151.48	765.54
489	130.87	116.29	87.43	71.19	405.78
490	438.56	441.86	369.6	263.08	1,513.10
491	54.6	52.99	50.49	47.28	205.36
492	177.71	198.14	152.46	108.75	637.06
493	45.27	64.77	51.65	35.24	196.93
494	208.93	224.54	189.08	106.61	729.16
495	164.15	118.74	81.54	61.02	425.45
496	192.65	175.16	146.48	122.04	636.33
497	227.22	291.4	216.16	170.52	905.3
498	17.71	24.35	22.44	5.44	69.94
499	15.52	33.45	2.32	21.23	72.52

TOTAL		20,397.39	20,864.78	18,129.58	16,304.26	75,696.01

STATE:	MINNESOTA					
ZIP CODE		QUARTER 1	QUARTER 2	QUARTER 3	QUARTER 4	TOTAL GRAMS
	550	76.02	85.11	60.22	40.77	262.12
	551	112	110.95	77.7	65.3	365.95
	553	86.49	104.02	87.25	70.83	348.59
	554	62.49	63.7	61.55	28.32	216.06
	557	29.35	27.3	33.01	14.99	104.65
	558	14.45	20.34	14.63	6.42	55.84
	559	8.94	12.77	12.4	6.96	41.07
	560	16.5	16.33	15.52	8.3	56.65
	561	2.68	6.42	0	0	9.1
	562	10.17	8.39	5.71	3.21	27.48
	563	44.25	28.55	14.81	8.56	96.17
	564	14.76	12.31	3.35	0.67	31.09
	565	7.14	8.92	7.14	6.24	29.44
	566	3.57	1.07	0	0	4.64
	567	12.49	13.2	10.88	12.67	49.24
TOTAL		501.3	519.38	404.17	273.24	1,698.09

STATE:	MISSISSIPPI					
ZIP CODE		QUARTER 1	QUARTER 2	QUARTER 3	QUARTER 4	TOTAL GRAMS
	386	224.27	236.27	161.96	127.57	750.07
	387	20.12	28.32	22.57	15.7	86.71
	388	495.76	524.02	414.65	291.58	1,726.01
	389	61.55	43.62	39.52	21.95	166.64
	390	209.51	198.27	160.04	76.63	644.45
	391	171.02	107.41	65.57	46.39	390.39
	392	260.23	220.57	142.87	93.76	717.43
	393	174	172.04	113.43	54.28	513.75
	394	374.77	358.4	224.85	147.82	1,105.84
	395	1,720.68	1,527.23	1,245.68	1,046.61	5,540.20
	396	98.4	118.56	76.32	55.67	348.95
	397	76.63	54.64	71.41	55.13	257.81

TOTAL	3,886.94	3,589.35	2,738.87	2,033.09	12,248.25

STATE: MISSOURI

ZIP CODE	QUARTER 1	QUARTER 2	QUARTER 3	QUARTER 4	TOTAL GRAMS
630	705.38	666.18	442.93	253.71	2,068.20
631	1,274.55	1,061.96	811.1	611.18	3,758.79
633	461.48	437.22	285.47	143.98	1,328.15
634	87.92	80.56	67.09	41.66	277.23
635	12.94	20.61	14.99	6.69	55.23
636	202.6	195.77	132.57	71.01	601.95
637	133.1	91.4	55.71	25.69	305.9
638	27.3	24.44	15.34	4.91	71.99
639	3.66	5.35	0.45	1.34	10.8
640	526.34	496.1	300.19	197.24	1,519.87
641	523.66	428.83	249.61	197.69	1,399.79
644	22.12	16.33	9.63	3.21	51.29
645	216.51	231.32	143.09	88.59	679.51
646	94.56	84.84	85.02	28.19	292.61
647	133.46	157.37	107.68	50.31	448.82
648	155.31	151.92	105.49	28.81	441.53
650	103.26	108.12	85.46	59.68	356.52
651	96.84	89.25	89.17	53.79	329.05
652	121.59	98.62	55.13	49.78	325.12
653	43.98	37.16	23.91	17.66	122.71
654	62.27	58.74	52.77	17.75	191.53
655	99.56	101.21	66.46	38.32	305.55
656	113.07	120.17	108.88	30.06	372.18
657	306.08	304.92	206.21	166.33	983.54
658	326.11	262.41	207.37	134.35	930.24
TOTAL	5,853.65	5,330.80	3,721.72	2,321.93	17,228.10

STATE: MONTANA

ZIP CODE	QUARTER 1	QUARTER 2	QUARTER 3	QUARTER 4	TOTAL GRAMS
590	6.24	4.73	1.61	0	12.58
591	48.82	66.28	42.82	31.22	189.14
592	0.89	0	0	0	0.89

	593	3.03	1.34	0	0	4.37
	594	111.42	39.74	40.55	24.09	215.8
	596	17.8	4.28	6.42	2.14	30.64
	597	35.83	27.12	19.98	5.53	88.46
	598	42.46	32.47	42.37	24	141.3
	599	121.5	120.57	136.22	71.28	449.57
TOTAL		387.99	296.53	289.97	158.26	1,132.75

STATE: NEBRASKA

ZIP CODE		QUARTER 1	QUARTER 2	QUARTER 3	QUARTER 4	TOTAL GRAMS
	680	92.42	108.39	63.07	24.44	288.32
	681	816.9	753.78	542.22	293.37	2,406.27
	683	12.04	9.28	9.99	15.25	46.56
	684	9.1	21.68	19.54	18.47	68.79
	685	277.09	252.06	172.93	120.52	822.6
	686	47.1	53.84	47.24	45.99	194.17
	687	132.25	135.64	88.36	73.2	429.45
	688	43.53	55.09	31.45	7.36	137.43
	689	25.92	29.53	5.17	3.39	64.01
	690	4.64	0	0	0	4.64
	691	37.91	24.18	19.8	8.03	89.92
	692	2.68	5.26	4.46	0	12.4
	693	65.61	63.16	53.84	20.16	202.77
TOTAL		1,567.19	1,511.89	1,058.07	630.18	4,767.33

STATE: NEVADA

ZIP CODE		QUARTER 1	QUARTER 2	QUARTER 3	QUARTER 4	TOTAL GRAMS
	890	1,360.41	1,243.50	983.14	590.48	4,177.53
	891	3,349.13	2,936.47	2,063.25	1,253.27	9,602.12
	893	2.99	0	0	1.78	4.77
	894	257.24	226.99	193.9	113.97	792.1
	895	344.48	319.91	192.34	82.74	939.47
	897	114.55	108.21	78.5	95.45	396.71

	898	5.35	4.28	3.75	4.28	17.66
TOTAL		5,434.15	4,839.36	3,514.88	2,141.97	15,930.36

STATE:	NEW HAMPSHIRE					
ZIP CODE		QUARTER 1	QUARTER 2	QUARTER 3	QUARTER 4	TOTAL GRAMS
	30	246.26	206.88	202.77	137.65	793.56
	31	106.35	108.48	68.33	34.61	317.77
	32	145.01	142.25	113.52	85.55	486.33
	33	149.38	142.74	110.35	101.92	504.39
	34	20.16	17.93	13.65	9.72	61.46
	35	19.72	25.25	22.3	11.78	79.05
	37	33.01	13.2	20.96	8.03	75.2
	38	375.87	336.1	274.32	187.52	1,173.81
TOTAL		1,095.76	992.83	826.2	576.78	3,491.57

STATE:	NEW JERSEY					
ZIP CODE		QUARTER 1	QUARTER 2	QUARTER 3	QUARTER 4	TOTAL GRAMS
	70	1,258.71	1,185.69	842.41	741.07	4,027.88
	71	89.57	107.85	80.02	44.16	321.6
	72	47.91	48.98	40.41	23.55	160.85
	73	55.67	58.61	38.18	31.58	184.04
	74	389.04	335.22	245.42	216.25	1,185.93
	75	87.25	87.96	83.05	61.73	319.99
	76	417.45	470.63	320.4	226.06	1,434.54
	77	732.55	648.2	488.83	412.95	2,282.53
	78	271.15	247.69	167.09	126.41	812.34
	79	173.34	136.31	127.84	70.74	508.23
	80	2,088.02	1,928.01	1,183.24	826.31	6,025.58
	81	133.15	101.74	102.5	79.04	416.43
	82	189.08	183.15	97.68	62.63	532.54
	83	212.99	193.99	113.92	57.81	578.71
	84	20.96	24.18	21.95	16.68	83.77
	85	304.56	236.23	198.31	179.94	919.04
	86	167.71	135.15	96.35	45.76	444.97
	87	632.72	593.16	373.25	315.27	1,914.40

	88	1,060.57	1,031.09	778.71	497.79	3,368.16
	89	59.86	55.85	23.37	33.45	172.53
TOTAL		8,392.26	7,809.69	5,422.93	4,069.18	25,694.06

STATE: NEW MEXICO

ZIP CODE		QUARTER 1	QUARTER 2	QUARTER 3	QUARTER 4	TOTAL GRAMS
	870	101.34	91.75	94.52	74.8	362.41
	871	671.8	518.82	435.43	347.07	1,973.12
	873	45.1	30.24	33.72	33.86	142.92
	874	12.76	13.56	14.81	14.81	55.94
	875	165.66	175.57	89.66	71.1	501.99
	877	5.44	12.22	4.19	9.37	31.22
	879	15.61	23.11	8.03	4.46	51.21
	880	137.12	118.47	68.83	22.21	346.63
	881	1.28	1.47	0	0	2.75
	882	57.94	55	36.53	50.85	200.32
	883	34.7	36.13	23.91	24.09	118.83
	884	3.21	3.21	4.28	0	10.7
TOTAL		1,251.96	1,079.55	813.91	652.62	3,798.04

STATE: NEW YORK

ZIP CODE		QUARTER 1	QUARTER 2	QUARTER 3	QUARTER 4	TOTAL GRAMS
	100	1,718.54	1,695.18	1,458.58	1,246.53	6,118.83
	101	64.5	59.41	46.57	36.58	207.06
	102	1.61	4.55	3.48	0	9.64
	103	1,155.54	1,136.22	797.94	664.26	3,753.96
	104	783	742.32	575.36	407.82	2,508.50
	105	453.72	456.67	338.91	272.71	1,522.01
	106	106.78	97.42	50.85	32.56	287.61
	107	215.75	194.03	108.3	67.44	585.52
	108	71.19	50.14	47.73	36.31	205.37
	109	926.71	882.96	720.46	555.24	3,085.37
	110	237.39	238.77	222	169.1	867.26
	111	216.91	196.35	158.17	191.09	762.52
	112	2,134.72	2,105.98	1,823.41	1,433.16	7,497.27

113	547.04	442.39	335.12	299.3	1,623.85
114	222.67	223.65	194.3	178.87	819.49
115	634.77	618.85	468.49	358.94	2,081.05
116	91.26	77.88	76.63	62.45	308.22
117	2,400.46	2,287.08	1,467.19	1,173.42	7,328.15
118	39.61	47.01	26.32	22.66	135.6
119	195.01	223.92	151.66	111.24	681.83
120	213.21	233.73	154.69	125.7	727.33
121	134.26	120.7	97.82	38.63	391.41
122	55.13	66.46	55.04	21.14	197.77
123	47.73	55.49	34.35	21.14	158.71
124	374.73	386.37	318.21	269.95	1,349.26
125	633.97	662.96	529.24	390.74	2,216.91
126	182.43	179.49	172.18	176.19	710.29
127	338.15	269.59	268.03	152.86	1,028.63
128	261.57	227.84	197.78	127.04	814.23
129	483.56	488.96	410.81	292.21	1,675.54
130	605.33	545.56	466.12	332.22	1,949.23
131	153.13	134.13	88.85	81.09	457.2
132	217.49	198.58	110	68.25	594.32
133	152.46	166.06	122.49	95.37	536.38
134	209.78	194.79	119.18	47.64	571.39
135	59.73	47.91	33.63	17.08	158.35
136	270.13	219.46	204.51	137.43	831.53
137	146.44	177.17	135.06	108.93	567.6
138	345.6	370.94	277.8	210.98	1,205.32
139	232.39	214.59	202.69	179.04	828.71
140	1,145.46	995.76	812.69	593.29	3,547.20
141	661.36	621.35	483.52	301.35	2,067.58
142	1,249.79	1,117.62	854.45	676.93	3,898.79
143	338.2	275.61	225.97	239.04	1,078.82
144	183.77	146.62	141.22	113.48	585.09
145	146.46	131.41	118.11	74.67	470.65
146	143.18	131.58	76.85	96.66	448.27
147	182.79	169.77	143.98	95.32	591.86
148	451.67	424.1	392.7	281.28	1,549.75
149	66.33	70.39	52.46	27.48	216.66

TOTAL		21,903.41	20,825.77	16,371.90	12,712.81	71,813.89

STATE: NORTH CAROLINA

ZIP CODE		QUARTER 1	QUARTER 2	QUARTER 3	QUARTER 4	TOTAL GRAMS
	270	1,313.04	1,167.31	650.61	327.94	3,458.90
	271	946.19	619.61	506.45	303.05	2,375.30
	272	2,240.37	1,982.16	1,178.95	685.49	6,086.97
	273	1,042.33	850.22	637.36	306.61	2,836.52
	274	543.78	517.86	428.61	300.41	1,790.66
	275	2,269.77	2,044.47	1,384.54	851.15	6,549.93
	276	574.02	524.06	353.67	281.37	1,733.12
	277	400.51	439.93	267.99	200.9	1,309.33
	278	2,305.81	2,331.55	1,957.18	1,703.42	8,297.96
	279	414.38	334.36	304.92	264.24	1,317.90
	280	2,603.90	2,390.59	1,718.45	1,426.60	8,139.54
	281	1,732.02	1,602.27	1,156.34	852.62	5,343.25
	282	948.15	921.58	781.79	633.3	3,284.82
	283	3,004.55	2,604.31	1,439.63	803.43	7,851.92
	284	1,774.16	1,590.88	940.45	606.32	4,911.81
	285	1,469.24	1,394.71	1,094.70	944.47	4,903.12
	286	3,207.80	2,965.88	2,013.78	1,320.66	9,508.12
	287	671.89	614.34	375.62	206.34	1,868.19
	288	139.57	142.6	73.69	29.71	385.57
	289	292.07	298.59	231.23	181.54	1,003.43
TOTAL		27,893.55	25,337.28	17,495.96	12,229.57	82,956.36

STATE: NORTH DAKOTA

ZIP CODE		QUARTER 1	QUARTER 2	QUARTER 3	QUARTER 4	TOTAL GRAMS
	580	0.89	0.89	1.78	0.89	4.45
	581	11.6	6.42	5.44	4.55	28.01
	582	6.6	7.76	5.71	0.89	20.96
	583	2.68	3.48	14.45	8.03	28.64
	584	8.56	6.24	8.56	3.12	26.48
	585	83.32	85.73	57.01	42.82	268.88
	586	4.28	3.21	6.96	4.64	19.09

	587	32.65	33.81	34.61	32.92	133.99
	588	23.64	33.19	25.16	17.13	99.12
TOTAL		174.22	180.73	159.68	114.99	629.62

STATE:	OHIO					
ZIP CODE		QUARTER 1	QUARTER 2	QUARTER 3	QUARTER 4	TOTAL GRAMS
	430	575.63	592.59	465.05	303.31	1,936.58
	431	433.78	387.04	302.42	157.81	1,281.05
	432	1,315.21	1,167.94	780.72	520.86	3,784.73
	433	152.82	107.19	76.63	54.37	391.01
	434	270.48	257.42	198.8	196.17	922.87
	435	326.39	270.71	253.62	156.47	1,007.19
	436	632.28	572.55	436.33	320.17	1,961.33
	437	120.52	125.25	77.52	66.64	389.93
	438	42.73	42.91	44.07	39.7	169.41
	439	473.93	454.52	389.94	263.08	1,581.47
	440	479.1	516.21	354.43	194.25	1,543.99
	441	390.12	388.2	301.93	130.43	1,210.68
	442	401.4	382.18	294.17	173.65	1,251.40
	443	152.5	126.32	99.65	54.15	432.62
	444	371.6	373.66	287.57	172.89	1,205.72
	445	216.47	196.08	180.83	157.5	750.88
	446	353.18	311.66	279.67	200.77	1,145.28
	447	263.44	269.24	215.67	135.24	883.59
	448	477.72	509.52	437.04	363.8	1,788.08
	449	60.13	14.9	44.16	24.44	143.63
	450	1,093.67	969.31	708.91	527.05	3,298.94
	451	467.86	394.32	320.31	223.56	1,406.05
	452	686.18	610.51	436.37	264.78	1,997.84
	453	364.33	320.71	224.54	188.95	1,098.53
	454	408.32	394	286.27	162.45	1,251.04
	455	124.05	104.55	69.41	22.66	320.67
	456	1,319.51	1,349.12	1,045.10	910.7	4,624.43
	457	167.67	184.49	118.74	55.67	526.57
	458	79.08	67.93	64.37	32.47	243.85

TOTAL	12,220.10	11,461.03	8,794.24	6,073.99	38,549.36

STATE: OKLAHOMA

ZIP CODE	QUARTER 1	QUARTER 2	QUARTER 3	QUARTER 4	TOTAL GRAMS
730	1,859.54	1,711.05	1,429.05	1,205.41	6,205.05
731	1,517.21	1,380.53	959.59	607.07	4,464.40
734	86.71	102.41	83.77	49.07	321.96
735	286.45	277.67	257.68	117.18	938.98
736	88.76	79.26	59.28	31.4	258.7
737	60.04	52.81	26.94	24.53	164.32
738	20.88	41.66	26.05	15.52	104.11
739	0	0.27	0	0	0.27
740	916.94	882.2	673.76	369.73	2,842.63
741	785	744.28	475.94	256.88	2,262.10
743	224.68	171.73	114.55	64.05	575.01
744	295.29	279.94	199.3	124.27	898.8
745	185.47	203.71	149.43	131.58	670.19
746	62.8	51.92	71.23	50.67	236.62
747	69.14	78.33	35.55	41.04	224.06
748	355.28	349.44	213.93	161.74	1,080.39
749	169.68	180.16	111.51	51.92	513.27
TOTAL	6,983.87	6,587.37	4,887.56	3,302.06	21,760.86

STATE: OREGON

ZIP CODE	QUARTER 1	QUARTER 2	QUARTER 3	QUARTER 4	TOTAL GRAMS
970	369.92	362.12	292.52	178.78	1,203.34
971	66.25	81.27	59.41	47.82	254.75
972	375.04	368.91	287.79	164.32	1,196.06
973	332.95	245.42	198.4	129.35	906.12
974	489.19	451.63	453.37	339.44	1,733.63
975	133.01	137.29	90.5	63.79	424.59
976	11.06	12.76	8.39	13.11	45.32
977	123.67	111.82	105	76.27	416.76
978	75.68	72.08	52.63	68.16	268.55
979	55.85	33.54	28.01	14.18	131.58

TOTAL	2,032.62	1,876.84	1,576.02	1,095.22	6,580.70

STATE: PENNSYLVANIA

ZIP CODE	QUARTER 1	QUARTER 2	QUARTER 3	QUARTER 4	TOTAL GRAMS
150	1,986.13	1,952.27	1,323.61	1,003.97	6,265.98
151	1,012.93	960.52	835.14	672.64	3,481.23
152	2,743.83	2,629.46	1,911.06	1,616.35	8,900.70
153	701.1	658.64	545.52	464.92	2,370.18
154	1,373.39	1,356.39	1,127.57	991.79	4,849.14
155	447.74	444.53	407.87	268.61	1,568.75
156	1,326.24	1,417.46	1,186.54	951.83	4,882.07
157	663.54	562.34	473.93	359.61	2,059.42
158	499.93	537.58	464.87	403.5	1,905.88
159	1,591.06	1,574.02	1,366.88	1,100.41	5,632.37
160	631.79	635.49	502.39	472.55	2,242.22
161	1,587.27	1,568.67	1,381.64	1,206.07	5,743.65
162	463.31	494.31	418.8	375.13	1,751.55
163	606.27	605.96	566.53	495.43	2,274.19
164	163.88	149.87	142.78	106.78	563.31
165	325.39	319.68	254.07	198.8	1,097.94
166	859.58	829.25	662.65	615.73	2,967.21
167	163.79	234.58	210.45	193.23	802.05
168	153.42	128.33	99.42	66.06	447.23
169	20.34	19.27	14.45	18.73	72.79
170	895.36	927.11	781.3	601.41	3,205.18
171	232.26	236.41	214.33	163.61	846.61
172	480.44	497.21	362.5	294.97	1,635.12
173	587.76	562.42	410.1	364.16	1,924.44
174	340.83	355.1	326.91	242.87	1,265.71
175	298.5	301.26	229.72	167.36	996.84
176	168.12	189.3	153.84	146.48	657.74
177	64.68	64.77	57.63	35.59	222.67
178	98.04	100.58	57.09	23.46	279.17
179	40.77	40.77	25.6	18.56	125.7
180	223.43	250.19	155.05	103.31	731.98
181	140.28	139.21	87.07	81.76	448.32

	182	32.78	31.8	38.49	104.96	208.03
	183	112.81	116.29	109.55	35.55	374.2
	184	184.58	191.27	153.26	77.79	606.9
	185	146.08	148.71	85.02	57.27	437.08
	186	143.72	141.09	146.39	109.55	540.75
	187	1,447.57	1,289.40	862.71	585.31	4,184.99
	188	35.37	33.81	39.88	24.8	133.86
	189	219.99	256.12	214.95	157.14	848.2
	190	1,874.88	1,710.24	1,362.46	929.66	5,877.24
	191	657.75	593.42	441.01	414.17	2,106.35
	193	509.39	420.4	337.79	228.73	1,496.31
	194	386.41	353	265.31	208.39	1,213.11
	195	109.01	86.4	77.52	48.53	321.46
	196	175.03	116.2	116.06	93.67	500.96
TOTAL		26,926.77	26,231.10	21,007.71	16,901.20	91,066.78

STATE: RHODE ISLAND

ZIP CODE		QUARTER 1	QUARTER 2	QUARTER 3	QUARTER 4	TOTAL GRAMS
	28	306.86	280.57	185.65	70.03	843.11
	29	270	206.01	178.73	104.15	758.89
TOTAL		576.86	486.58	364.38	174.18	1,602

STATE: SOUTH CAROLINA

ZIP CODE		QUARTER 1	QUARTER 2	QUARTER 3	QUARTER 4	TOTAL GRAMS
	290	927.92	842.63	517.64	393.42	2,681.61
	291	710.51	732.99	512.6	379.32	2,335.42
	292	910.57	958.16	805.25	656.81	3,330.79
	293	363.4	306.75	136.67	68.42	875.24
	294	1,687.14	1,642.71	1,091.17	751.82	5,172.84
	295	1,004.01	939.69	735.58	479.15	3,158.43
	296	1,175.69	1,187.39	703.38	487.98	3,554.44
	297	495.34	405.28	264.24	161.87	1,326.73
	298	178.69	199.92	101.07	94.74	574.42
	299	313.8	287.84	239.17	151.92	992.73
TOTAL		7,767.07	7,503.36	5,106.77	3,625.45	24,002.65

STATE: SOUTH DAKOTA

ZIP CODE	QUARTER 1	QUARTER 2	QUARTER 3	QUARTER 4	TOTAL GRAMS
570	78.46	62.45	43.09	43	227
571	135.85	133.37	111.87	63.43	444.52
572	6.42	7.49	4.28	4.46	22.65
573	5.62	5.62	1.34	1.78	14.36
574	24.44	7.85	9.28	6.96	48.53
575	11.15	7.32	13.47	6.24	38.18
576	6.42	3.57	0	1.43	11.42
577	363.38	305.9	235.78	200.45	1,105.51
TOTAL	631.74	533.57	419.11	327.75	1,912.17

STATE: TENNESSEE

ZIP CODE	QUARTER 1	QUARTER 2	QUARTER 3	QUARTER 4	TOTAL GRAMS
370	3,408.09	3,262.05	2,451.01	2,019.09	11,140.24
371	2,166.20	2,011.46	1,496.27	1,179.13	6,853.06
372	1,636.95	1,348.50	1,013.65	933.76	4,932.86
373	1,688.66	1,649.27	1,165.31	856.59	5,359.83
374	849.86	865.6	706.19	490.92	2,912.57
376	1,539.10	1,375.80	1,065.57	651.59	4,632.06
377	4,124.89	4,025.29	3,516.03	3,304.43	14,970.64
378	4,791.38	4,384.49	3,651.32	3,202.24	16,029.43
379	1,798.16	1,612.60	1,328.20	1,064.05	5,803.01
380	203.84	169.9	161.2	89.52	624.46
381	197.73	191.62	107.1	134.17	630.62
382	264.33	229.49	178.2	123.87	795.89
383	534.41	464.47	353.54	292.88	1,645.30
384	464.52	393.19	285.47	177.44	1,320.62
385	1,521.79	1,362.77	974.22	724.79	4,583.57
TOTAL	25,189.91	23,346.50	18,453.28	15,244.47	82,234.16

STATE: TEXAS

ZIP CODE	QUARTER 1	QUARTER 2	QUARTER 3	QUARTER 4	TOTAL GRAMS

750	2,068.56	1,811.72	1,232.57	884.96	5,997.81
751	295.96	298.9	182.61	105.98	883.45
752	448.52	423.21	329.15	206.3	1,407.18
754	245.51	207.86	83.59	49.42	586.38
755	9.37	11.46	8.43	0	29.26
756	29.97	33.81	25.42	5.08	94.28
757	59.86	72.04	63.25	39.16	234.31
758	62.27	50.49	49.51	22.03	184.3
759	88.81	76.05	54.06	34.52	253.44
760	928.23	927.74	704.8	432.49	2,993.26
761	752.62	738.17	543.07	238.28	2,272.14
762	274.41	353.67	170.17	119.45	917.7
763	276.77	277.76	210.09	194.88	959.5
764	74	46.17	36.93	27.48	184.58
765	153.89	147.46	85.24	49.42	436.01
766	22.08	24.35	5.8	1.61	53.84
767	18.69	36.04	24	21.5	100.23
768	48.89	34.35	14.45	22.75	120.44
769	93.22	72.08	57.63	57.09	280.02
770	816.76	823.32	560.06	372.45	2,572.59
773	601.41	550.11	338.15	219.55	1,709.22
774	805.57	691.07	406.26	265.18	2,168.08
775	849.73	757.66	589.68	394.98	2,592.05
776	328.96	285.83	168.83	135.42	919.04
777	125.12	162.45	93.67	85.64	466.88
778	142.91	123.96	98.98	45.85	411.7
779	145.01	124.85	81.14	55.18	406.18
780	231.59	206.79	154.15	77.43	669.96
781	268.88	268.34	184.84	83.05	805.11
782	1,745.63	1,592.28	1,149.34	664.48	5,151.73
783	73.82	64.77	31.58	20.07	190.24
784	225.52	212.72	135.24	80.11	653.59
785	42.29	57.9	27.03	5.53	132.75
786	1,208.55	1,097.91	779.7	612.29	3,698.45
787	1,083.81	957.45	678.84	438.33	3,158.43
788	48.84	37.11	31.49	13.56	131
789	33.63	39.07	21.81	14.45	108.96

	790	84.93	64.81	62.76	16.15	228.65
	791	182.17	248.05	162.99	105.45	698.66
	792	1.16	1.87	0	0	3.03
	793	33.63	26.05	18.02	4.73	82.43
	794	67.44	59.9	37.6	24.58	189.52
	795	0.54	3.66	1.61	1.25	7.06
	796	43.94	33.19	30.06	12.13	119.32
	797	152.64	156.83	80.38	32.12	421.97
	798	12.85	0	0	0	12.85
	799	173.78	140.33	90.19	66.37	470.67
TOTAL		15,482.74	14,431.61	9,895.17	6,358.73	46,168.25

STATE: UTAH

ZIP CODE		QUARTER 1	QUARTER 2	QUARTER 3	QUARTER 4	TOTAL GRAMS
	840	2,406.80	2,277.58	1,909.36	1,693.79	8,287.53
	841	873.28	771.94	653.02	523.66	2,821.90
	843	167.54	154.87	117.44	89.34	529.19
	844	515.05	525.13	418.57	368.93	1,827.68
	845	82.12	74.53	73.06	64.01	293.72
	846	380.17	375.31	320.13	261.7	1,337.31
	847	391.36	333.82	345.91	231.01	1,302.10
TOTAL		4,816.32	4,513.18	3,837.49	3,232.44	16,399.43

STATE: VERMONT

ZIP CODE		QUARTER 1	QUARTER 2	QUARTER 3	QUARTER 4	TOTAL GRAMS
	50	8.83	16.06	13.11	10.88	48.88
	51	11.33	4.01	1.07	0	16.41
	52	27.57	17.84	17.84	22.3	85.55
	53	5.89	6.42	5.62	0	17.93
	54	212.5	216.42	174.14	115.17	718.23
	56	28.37	25.59	18.2	9.46	81.62
	57	65.08	35.51	42.73	20.43	163.75
	58	3.12	3.57	2.68	3.12	12.49
TOTAL		362.69	325.42	275.39	181.36	1,144.86

STATE: VIRGINIA

ZIP CODE	QUARTER 1	QUARTER 2	QUARTER 3	QUARTER 4	TOTAL GRAMS
201	952.9	902.14	732.99	618.58	3,206.61
220	744.74	704.76	537.13	494.13	2,480.76
221	742.6	714.3	623.71	585.89	2,666.50
222	168.92	155.76	140.51	148.89	614.08
223	219.77	193.99	138.05	185.33	737.14
224	1,145.90	948.17	711.54	674.43	3,480.04
225	150.32	188.59	135.02	108.48	582.41
226	472.01	475.62	411.61	338.28	1,697.52
227	58.03	51.34	49.11	46.03	204.51
228	63.79	71.37	40.99	29.35	205.5
229	169.23	177.8	128.15	112.58	587.76
230	266.96	300.24	312.64	333.07	1,212.91
231	377.09	353.27	272.05	135.06	1,137.47
232	557.7	455.64	276.96	205	1,495.30
233	381.32	429.37	302.29	250.59	1,363.57
234	872.91	798.93	624.02	498.15	2,794.01
235	251.24	313.26	188.63	162.36	915.49
236	266.29	251.75	189.75	123.56	831.35
237	173.07	216.51	129.35	102.15	621.08
238	428.88	381.11	293.33	264.11	1,367.43
239	76.1	86.89	79.17	59.41	301.57
240	587	457.25	379.77	259.65	1,683.67
241	279.81	280.07	165.26	149.61	874.75
242	504.57	451.4	387.71	271.24	1,614.92
243	667.16	233.28	155.58	124	1,180.02
244	90.28	63.96	22.57	31.4	208.21
245	454.61	457.78	327.13	242.65	1,482.17
246	263.44	260.58	144.16	97.42	765.6
TOTAL	11,386.64	10,375.13	7,899.18	6,651.40	36,312.35

STATE: WASHINGTON

ZIP CODE	QUARTER 1	QUARTER 2	QUARTER 3	QUARTER 4	TOTAL GRAMS
980	835.1	714.68	572.77	399.08	2,521.63

	981	217.93	215.52	190.64	126.23	750.32
	982	439.06	403.45	329.54	306.88	1,478.93
	983	287.15	306.66	229.58	164.77	988.16
	984	184.97	171.02	125.65	95.19	576.83
	985	303.89	263.3	179.89	143	890.08
	986	501.83	483.58	400.2	310.09	1,695.70
	988	57.72	42.73	30.87	20.96	152.28
	989	112.05	94.29	70.3	52.63	329.27
	990	42.15	21.01	25.2	20.16	108.52
	991	31.6	19.89	18.91	4.64	75.04
	992	345.78	298.59	236.5	145.01	1,025.88
	993	258.17	214.91	171.91	113.65	758.64
	994	16.59	17.13	12.31	8.03	54.06
TOTAL		3,633.99	3,266.76	2,594.27	1,910.32	11,405.34

STATE: WEST VIRGINIA

ZIP CODE	QUARTER 1	QUARTER 2	QUARTER 3	QUARTER 4	TOTAL GRAMS
247	58.88	83.77	55.13	37.33	235.11
248	137.34	129.18	119.05	72.35	457.92
249	63.07	66.73	42.64	28.73	201.17
250	121.91	123.47	98.49	16.24	360.11
251	107.68	114.28	35.95	18.91	276.82
252	43.27	31.58	18.2	6.87	99.92
253	206.57	225.12	209.24	112.58	753.51
254	915.61	901.2	873.46	726.66	3,416.93
255	242.87	241.71	206.92	131.45	822.95
256	78.01	86.8	47.28	49.42	261.51
257	138.68	85.15	68.78	58.88	351.49
258	88.41	126.68	59.68	16.59	291.36
259	46.75	49.78	38.18	20.79	155.5
260	366.3	356.97	297.78	194.7	1,215.75
261	42.73	34.17	36.58	9.19	122.67
262	39.21	41.04	38.14	15.52	133.91
263	39.21	28.77	21.54	20.88	110.4
264	18.56	17.49	14.27	6.07	56.39
265	58.61	79.49	46.3	39.79	224.19

	266	23.51	20.88	10.17	8.39	62.95
	267	88.85	67.8	61.11	57.9	275.66
	268	3.48	17.4	11.24	5.35	37.47
TOTAL		2,929.51	2,929.46	2,410.13	1,654.59	9,923.69

STATE: WISCONSIN

ZIP CODE		QUARTER 1	QUARTER 2	QUARTER 3	QUARTER 4	TOTAL GRAMS
	530	423.22	385.58	255.14	156.92	1,220.86
	531	685.72	575.78	433.29	288.15	1,982.94
	532	522.4	431.52	302.33	155.76	1,412.01
	534	124.89	100.81	93.14	91.53	410.37
	535	132.83	122.04	81.63	59.86	396.36
	537	70.85	54.87	34.88	15.88	176.48
	538	8.13	3.48	1.61	3.21	16.43
	539	66.73	43.71	30.55	18.29	159.28
	540	26.85	26.23	19.63	14.27	86.98
	541	97.48	77.26	40.01	24.09	238.84
	542	39.21	37.96	19.98	14.81	111.96
	543	69.99	89.03	72.48	38	269.5
	544	132.66	126.14	122.98	95.41	477.19
	545	25.25	34.44	18.73	10.44	88.86
	546	1.87	0	0.54	4.01	6.42
	547	31.05	20.34	9.9	11.33	72.62
	548	24	35.06	20.79	18.64	98.49
	549	441.42	375.44	286.23	182.7	1,285.79
TOTAL		2,924.55	2,539.69	1,843.84	1,203.30	8,511.38

STATE: WYOMING

ZIP CODE		QUARTER 1	QUARTER 2	QUARTER 3	QUARTER 4	TOTAL GRAMS
	820	40.14	38.72	26.23	25.78	130.87
	822	12.31	18.02	5.89	0	36.22
	823	0.89	0	1.78	2.68	5.35
	824	6.16	3.21	5.89	2.68	17.94
	825	30.96	51.3	43.31	44.2	169.77

	826	45.68	47.28	55.76	47.55	196.27
	827	24.8	26.67	23.11	29.44	104.02
	828	56.92	53.35	17.57	12.67	140.51
	829	73.06	68.87	47.1	20.96	209.99
	830	4.28	6.78	10.71	0	21.77
	831	27.3	28.64	18.02	13.38	87.34
TOTAL		322.5	342.84	255.37	199.34	1,120.05

DRUG CODE:9668DRUG NAME:NOROXYMORPHONE

STATE: ARIZONA

ZIP CODE	QUARTER 1	QUARTER 2	QUARTER 3	QUARTER 4	TOTAL GRAMS
857	0	0	0.1	0	0.1
TOTAL	0	0	0.1	0	0.1

STATE: ARKANSAS

ZIP CODE	QUARTER 1	QUARTER 2	QUARTER 3	QUARTER 4	TOTAL GRAMS
722	7.22	7.13	11.64	7.34	33.33
727	7.41	10.08	15.21	12.04	44.74
TOTAL	14.63	17.21	26.85	19.38	78.07

STATE: CALIFORNIA

ZIP CODE	QUARTER 1	QUARTER 2	QUARTER 3	QUARTER 4	TOTAL GRAMS
902	6.65	10.23	3.17	15.49	35.54
905	5.68	4.37	3.49	2.37	15.91
906	4.23	6.22	5.84	7.83	24.12
907	2.71	4.78	5.23	3.79	16.51
908	1.06	0	4	4.91	9.97
912	2.25	2.25	7.61	0.62	12.73
914	0	0	0.1	0	0.1
919	3.74	4.01	5.2	2.59	15.54
920	3.73	4.07	4.84	6.74	19.38
921	2.04	2.36	2.78	1.12	8.3
923	2.69	7.11	0.1	4.87	14.77
925	0	1.84	5.84	0	7.68

927	0	0	0	0.1	0.1
928	17.07	20.41	15.85	13.24	66.57
943	0	0	1.56	0	1.56
946	0.75	0.87	4.23	4.97	10.82
TOTAL	52.6	68.52	69.84	68.64	259.6

STATE: FLORIDA

ZIP CODE	QUARTER 1	QUARTER 2	QUARTER 3	QUARTER 4	TOTAL GRAMS
322	0	0.1	1.56	0.08	1.74
327	0.04	0	0	0	0.04
328	0	0.29	1.06	0.06	1.41
329	1.45	1.81	0	0	3.26
330	0	0.25	0	0	0.25
331	2.42	2.67	1.93	4.91	11.93
333	8.7	7.15	8.96	10.9	35.71
334	0	1.56	0	1.06	2.62
336	0	0	0	1.06	1.06
TOTAL	12.61	13.83	13.51	18.07	58.02

STATE: GEORGIA

ZIP CODE	QUARTER 1	QUARTER 2	QUARTER 3	QUARTER 4	TOTAL GRAMS
300	3.06	0.37	2.59	0.12	6.14
303	0.12	1.75	4.95	3.63	10.45
309	0	1.06	1.76	1.05	3.87
TOTAL	3.18	3.18	9.3	4.8	20.46

STATE: ILLINOIS

ZIP CODE	QUARTER 1	QUARTER 2	QUARTER 3	QUARTER 4	TOTAL GRAMS
601	0.32	0	0.1	0	0.42
606	4.1	9.76	14.99	8.75	37.6
TOTAL	4.42	9.76	15.09	8.75	38.02

DATE RANGE: 01/01/2017 TO 12/31/2017 ARCOS 3 - REPORT 1 Run Date: 07/03/2018

RETAIL DRUG DISTRIBUTION BY ZIP CODE WITHIN STATE BY GRAMS WT

STATE:	KANSAS				
ZIP CODE	QUARTER 1	QUARTER 2	QUARTER 3	QUARTER 4	TOTAL GRAMS
662	2.75	0	0	0	2.75
TOTAL	2.75	0	0	0	2.75

STATE:	MARYLAND				
ZIP CODE	QUARTER 1	QUARTER 2	QUARTER 3	QUARTER 4	TOTAL GRAMS
212	0	0.1	0	0.1	0.2
TOTAL	0	0.1	0	0.1	0.2

STATE:	MASSACHUSETTS				
ZIP CODE	QUARTER 1	QUARTER 2	QUARTER 3	QUARTER 4	TOTAL GRAMS
24	1.14	1.9	0	0	3.04
TOTAL	1.14	1.9	0	0	3.04

STATE:	MICHIGAN				
ZIP CODE	QUARTER 1	QUARTER 2	QUARTER 3	QUARTER 4	TOTAL GRAMS
490	0	0	1.56	0	1.56
494	0	0	1.56	0	1.56
495	0	0	0	1.17	1.17
TOTAL	0	0	3.12	1.17	4.29

STATE:	MINNESOTA				
ZIP CODE	QUARTER 1	QUARTER 2	QUARTER 3	QUARTER 4	TOTAL GRAMS
TOTAL	0	0.5	0	0	0.5

STATE:	MISSISSIPPI				
ZIP CODE	QUARTER 1	QUARTER 2	QUARTER 3	QUARTER 4	TOTAL GRAMS
392	2.4	1.57	2.22	0	6.19
TOTAL	2.4	1.57	2.22	0	6.19

STATE:	MISSOURI				
ZIP CODE	QUARTER 1	QUARTER 2	QUARTER 3	QUARTER 4	TOTAL GRAMS
631	7.33	13	9.03	5.8	35.16
633	0.37	0.1	0	0	0.47
641	0	0	1.56	0	1.56
TOTAL	7.7	13.1	10.59	5.8	37.19

STATE:	NEVADA				
ZIP CODE	QUARTER 1	QUARTER 2	QUARTER 3	QUARTER 4	TOTAL GRAMS
891	6.64	2.87	7.45	8.31	25.27
TOTAL	6.64	2.87	7.45	8.31	25.27

STATE:	NEW JERSEY				
ZIP CODE	QUARTER 1	QUARTER 2	QUARTER 3	QUARTER 4	TOTAL GRAMS
80	7.12	12.03	1.56	3	23.71
TOTAL	7.12	12.03	1.56	3	23.71

STATE:	NEW YORK				
ZIP CODE	QUARTER 1	QUARTER 2	QUARTER 3	QUARTER 4	TOTAL GRAMS
101	0.41	0	0	0	0.41
105	0	0	0.1	0.14	0.24
112	0.87	2	1.75	2.87	7.49
114	0.25	1.54	1.86	0	3.65
146	1.12	2.15	5.59	1.47	10.33
TOTAL	2.65	5.69	9.3	4.48	22.12

STATE:	NORTH CAROLINA				
ZIP CODE	QUARTER 1	QUARTER 2	QUARTER 3	QUARTER 4	TOTAL GRAMS
277	0	0	0	1.56	1.56
282	0	0	2.12	0	2.12
TOTAL	0	0	2.12	1.56	3.68

STATE: OHIO

ZIP CODE	QUARTER 1	QUARTER 2	QUARTER 3	QUARTER 4	TOTAL GRAMS
447	0.42	0.46	1.82	2.88	5.58
452	0	2.22	0	0	2.22
454	5.47	6.68	4.37	0	16.52
TOTAL	5.89	9.36	6.19	2.88	24.32

STATE: OKLAHOMA

ZIP CODE	QUARTER 1	QUARTER 2	QUARTER 3	QUARTER 4	TOTAL GRAMS
731	0	0	0.1	0.55	0.65
TOTAL	0	0	0.1	0.55	0.65

STATE: OREGON

ZIP CODE	QUARTER 1	QUARTER 2	QUARTER 3	QUARTER 4	TOTAL GRAMS
972	0.15	0.04	0	0	0.19
974	0	0	1.81	0	1.81
TOTAL	0.15	0.04	1.81	0	2

STATE: PENNSYLVANIA

ZIP CODE	QUARTER 1	QUARTER 2	QUARTER 3	QUARTER 4	TOTAL GRAMS
181	0	0.51	0.47	0	0.98
TOTAL	0	0.51	0.47	0	0.98

STATE: PUERTO RICO

ZIP CODE	QUARTER 1	QUARTER 2	QUARTER 3	QUARTER 4	TOTAL GRAMS
9	1.86	4.44	11.94	6.83	25.07
TOTAL	1.86	4.44	11.94	6.83	25.07

STATE: RHODE ISLAND

ZIP CODE	QUARTER 1	QUARTER 2	QUARTER 3	QUARTER 4	TOTAL GRAMS
28	0	0	0.1	0	0.1
TOTAL	0	0	0.1	0	0.1

STATE: TENNESSEE

ZIP CODE	QUARTER 1	QUARTER 2	QUARTER 3	QUARTER 4	TOTAL GRAMS
381	0.13	0	0	0	0.13
TOTAL	0.13	0	0	0	0.13

STATE: TEXAS

ZIP CODE	QUARTER 1	QUARTER 2	QUARTER 3	QUARTER 4	TOTAL GRAMS
750	10.17	8.49	14.83	11.11	44.6
751	5.58	11.06	5.59	3.78	26.01
753	0	0	0	0.01	0.01
761	0	0	3.12	0	3.12
763	0.15	0.24	0	0	0.39
770	0	0	2.22	2.46	4.68
782	0	0	1.56	1.06	2.62
787	5.87	9.8	3.97	7.77	27.41
TOTAL	21.77	29.59	31.29	26.19	108.84

STATE: VERMONT

ZIP CODE	QUARTER 1	QUARTER 2	QUARTER 3	QUARTER 4	TOTAL GRAMS
50	0	0	0.1	0	0.1
TOTAL	0	0	0.1	0	0.1

STATE: WASHINGTON

ZIP CODE	QUARTER 1	QUARTER 2	QUARTER 3	QUARTER 4	TOTAL GRAMS
980	0.41	0.58	3.11	3.94	8.04
992	0.24	0.1	0.1	0	0.44
TOTAL	0.65	0.68	3.21	3.94	8.48

RETAIL DRUG DISTRIBUTION BY ZIP CODE WITHIN STATE BY GRAMS WT

DRUG CODE:9737DRUG NAME:ALFENTANIL

STATE: ALABAMA

ZIP CODE	QUARTER 1	QUARTER 2	QUARTER 3	QUARTER 4	TOTAL GRAMS
357	0	0.13	0	0	0.13
363	0.03	0	0.03	0.03	0.09
368	0.01	0	0.02	0	0.03
TOTAL	0.04	0.13	0.05	0.03	0.25

STATE: ALASKA

ZIP CODE	QUARTER 1	QUARTER 2	QUARTER 3	QUARTER 4	TOTAL GRAMS
995	0.74	0.74	0.5	0.86	2.84
996	0.01	0.01	0	0.03	0.05
TOTAL	0.75	0.75	0.5	0.89	2.89

STATE: ARIZONA

ZIP CODE	QUARTER 1	QUARTER 2	QUARTER 3	QUARTER 4	TOTAL GRAMS
850	0.08	0.08	0.12	0.11	0.39
851	0.02	0.06	0	0.05	0.13
852	0.18	0.09	0.04	0.21	0.52
853	0	0	0.06	0.06	0.12
855	0	0.04	0.03	0	0.07
857	0.84	0.59	0.44	0.55	2.42
860	0	0.1	0	0	0.1
TOTAL	1.12	0.96	0.69	0.98	3.75

STATE: ARKANSAS

ZIP CODE	QUARTER 1	QUARTER 2	QUARTER 3	QUARTER 4	TOTAL GRAMS
718	0.01	0	0.02	0.01	0.04
726	0	0	0.02	0.02	0.04
TOTAL	0.01	0	0.04	0.03	0.08

STATE: CALIFORNIA

ZIP CODE	QUARTER 1	QUARTER 2	QUARTER 3	QUARTER 4	TOTAL GRAMS
900	1.84	1.9	1.39	2.83	7.96
902	0.39	0.65	0.08	0.35	1.47
904	0.1	0.29	0.05	0.1	0.54
905	0	0.05	0	0	0.05
906	0.15	0.25	0.32	0.28	1
910	0	0	0	0.01	0.01
912	0	0	0.01	0	0.01
913	0.44	0.28	0.18	0.4	1.3
914	0.08	0.1	0.05	0.15	0.38
915	0.05	0.06	0.02	0	0.13
917	1.11	0.6	1.46	0.75	3.92
919	0.26	0.13	0.15	0.12	0.66
920	0.94	0.37	0.59	1.31	3.21
921	1.38	0.71	0.9	0.99	3.98
922	0.52	0.76	0.6	0.49	2.37
923	1.02	1.47	1.79	1.95	6.23
925	0.26	0.22	0.21	0.24	0.93
926	0.5	0.43	0.4	0.25	1.58
927	0.04	0	0.18	0.04	0.26

DRUG CODE:9737DRUG NAME:ALFENTANIL

STATE:CALIFORNIA

ZIP CODE	QUARTER 1	QUARTER 2	QUARTER 3	QUARTER 4	TOTAL GRAMS
928	0.55	0.41	0	0	0.96
930	0.18	0.24	0.21	0.21	0.84
931	0.26	0.36	0.2	0.29	1.11
932	0.11	0	0.1	0.03	0.24
934	0.05	0.09	0	0.04	0.18
935	0.03	0.05	0.13	0.21	0.42
936	0.03	0	0.07	0.07	0.17
937	1.41	1.34	0.96	0.9	4.61
939	0.03	0.04	0	0.02	0.09
940	0.46	0.28	0.58	0.44	1.76

	941	0.08	0.32	0.01	0.16	0.57
	943	1.07	0.56	0.47	0.76	2.86
	944	0.02	0.05	0.07	0.1	0.24
	945	0.9	1.27	1.28	0.84	4.29
	946	0.21	0.23	0.49	0.15	1.08
	948	0.03	0	0.02	0	0.05
	949	0	0	0.03	0.08	0.11
	950	0.01	0.02	0.03	0.05	0.11
	951	0.58	0.55	0.45	0.5	2.08
	952	0.29	0.04	0.13	0.38	0.84
	953	0.62	0.65	0.63	0.53	2.43
	954	0.19	0.2	0.15	0.34	0.88
	956	0.06	0	0.04	0.04	0.14
	958	0.86	0.13	1.6	0.3	2.89
	959	0.24	0.22	0.24	0.22	0.92
	960	0.03	0.01	0.07	0.14	0.25
TOTAL		17.38	15.33	16.34	17.06	66.11

STATE: COLORADO

ZIP CODE		QUARTER 1	QUARTER 2	QUARTER 3	QUARTER 4	TOTAL GRAMS
	800	0.89	0.4	0.47	0.8	2.56
	801	0.03	0.09	0.04	0.07	0.23
	805	0.09	0.06	0	0.01	0.16
	808	0.06	0.08	0.13	0	0.27
	811	0	0	0.03	0	0.03
	812	0	0	0	0.01	0.01
	813	0.08	0.08	0.12	0.1	0.38
	814	0.08	0.01	0	0	0.09
	815	0	0	0	0.07	0.07
	816	0.04	0	0	0.02	0.06
TOTAL		1.27	0.72	0.79	1.08	3.86

STATE: CONNECTICUT

ZIP CODE	QUARTER 1	QUARTER 2	QUARTER 3	QUARTER 4	TOTAL GRAMS
64	0.1	0	0	0	0.1
65	0.98	0.77	0.79	0.95	3.49
68	0.44	0.43	0.25	0.6	1.72
TOTAL	1.52	1.2	1.04	1.55	5.31

STATE: FLORIDA

ZIP CODE	QUARTER 1	QUARTER 2	QUARTER 3	QUARTER 4	TOTAL GRAMS
321	0.03	0.02	0	0	0.05
322	0.1	0.21	0.37	0.37	1.05
324	0.59	0.38	0.72	0.07	1.76
325	0	0.33	0	0.03	0.36
326	2.5	2.4	2.35	4.1	11.35
327	0	0.09	0	0	0.09
328	0.15	0.16	0.15	0.24	0.7
329	0.4	0.57	0.48	0.52	1.97
330	0	0.18	0	0.1	0.28
331	0.1	0.2	0.13	0.1	0.53
333	0	0	0	0.01	0.01
334	0.64	0.53	0.55	0.43	2.15
336	0.03	0.02	0	0	0.05
337	0	0.2	0	0	0.2
339	0	0	0.01	0.1	0.11
342	0.01	0.03	0.01	0.03	0.08
344	0	0.09	0.02	0	0.11
346	0	0.05	0	0	0.05
349	0	0.02	0	0.01	0.03
TOTAL	4.55	5.48	4.79	6.11	20.93

STATE: GEORGIA

ZIP CODE	QUARTER 1	QUARTER 2	QUARTER 3	QUARTER 4	TOTAL GRAMS
301	0.02	0.01	0.03	0.02	0.08
303	0.62	0.73	2.13	0.4	3.88
305	0	0	0.01	0.01	0.02

	308	0.06	0.04	0.06	0.08	0.24
	309	1.34	1.19	1.69	1.21	5.43
	312	0.16	0.22	0.12	0.24	0.74
	314	0	0	0	0.05	0.05
	315	0.3	0.15	0.2	0.07	0.72
	317	0.42	0.41	0.4	0.49	1.72
	319	0	0.04	0.02	0.1	0.16
	398	0	0	0	0.02	0.02
TOTAL		2.92	2.79	4.66	2.69	13.06

STATE: GUAM

ZIP CODE		QUARTER 1	QUARTER 2	QUARTER 3	QUARTER 4	TOTAL GRAMS
	969	0	0	0.05	0.03	0.08
TOTAL		0	0	0.05	0.03	0.08

STATE: HAWAII

ZIP CODE		QUARTER 1	QUARTER 2	QUARTER 3	QUARTER 4	TOTAL GRAMS
	967	0.23	0.28	0.25	0.38	1.14
	968	0.22	0.22	0.37	0.46	1.27
TOTAL		0.45	0.5	0.62	0.84	2.41

STATE: IDAHO

ZIP CODE		QUARTER 1	QUARTER 2	QUARTER 3	QUARTER 4	TOTAL GRAMS
	833	0.1	0.15	0.13	0.08	0.46
	836	0	0	0.05	0.08	0.13
	837	1.36	1.24	0.97	1.31	4.88
TOTAL		1.46	1.39	1.15	1.47	5.47

STATE: ILLINOIS

ZIP CODE		QUARTER 1	QUARTER 2	QUARTER 3	QUARTER 4	TOTAL GRAMS
	600	0.16	0.18	0.19	0.21	0.74
	601	1.03	0.88	1.05	1.69	4.65
	604	0	0.63	0.03	0.37	1.03

	605	0.05	0	0.1	0.08	0.23
	606	0.5	0.8	0.77	0.81	2.88
	608	0.06	0.05	0.04	0.05	0.2
	610	0.52	0.44	0.32	0.27	1.55
	611	0.97	0.97	0.65	0.96	3.55
	612	0.06	0	0.03	0.05	0.14
	622	0	0.01	0	0.01	0.02
	624	0.04	0.06	0.04	0.05	0.19
	627	0.11	0.06	0.08	0.37	0.62
	628	0.23	0.12	0.07	0.18	0.6
	629	0.05	0.04	0.01	0.06	0.16
TOTAL		3.78	4.24	3.38	5.16	16.56

STATE: INDIANA

ZIP CODE		QUARTER 1	QUARTER 2	QUARTER 3	QUARTER 4	TOTAL GRAMS
	460	0.75	1.08	1	0.96	3.79
	461	0.03	0	0	0	0.03
	462	0.47	0.59	0.63	0.68	2.37
	463	0.04	0	0.02	0	0.06
	464	0.01	0	0.01	0.01	0.03
	465	0	0.03	0	0.02	0.05
	466	0.67	0.67	0.52	0.5	2.36
	467	0	0	0.01	0.01	0.02
	469	0.02	0.02	0.02	0.01	0.07
	470	0	0	0.02	0.01	0.03
	471	0	0.45	0.01	0.01	0.47
	472	0.08	0.1	0.05	0	0.23
	473	0.03	0.05	0.13	0.1	0.31
	475	0.02	0.01	0	0	0.03
	477	0.05	0.1	0.1	0.03	0.28
	479	0.07	0.08	0.02	0.01	0.18
TOTAL		2.24	3.18	2.54	2.35	10.31

STATE: IOWA

ZIP CODE		QUARTER 1	QUARTER 2	QUARTER 3	QUARTER 4	TOTAL GRAMS

500	0	0.03	0.04	0.03	0.1
503	0.02	0	0	0	0.02
505	0	0.04	0	0.02	0.06
506	0	0	0.01	0	0.01
507	0.01	0.02	0.04	0.01	0.08
520	0	0	0	0.09	0.09
522	1.41	0.85	0.83	0.86	3.95
523	0	0	0.01	0.01	0.02
524	0.21	0.15	0.13	0.21	0.7
526	0	0.2	0	0	0.2
527	0.13	0.02	0.06	0.11	0.32
528	0.27	0.19	0.28	0.41	1.15
TOTAL	2.05	1.5	1.4	1.75	6.7

STATE: KANSAS

ZIP CODE	QUARTER 1	QUARTER 2	QUARTER 3	QUARTER 4	TOTAL GRAMS
660	0	0	0	0.02	0.02
662	0	0.02	0.01	0.02	0.05
667	0	0	0	0.04	0.04
669	0.1	0.05	0	0	0.15
671	0.02	0.02	0.02	0.02	0.08
674	0.99	0.88	0.69	0.98	3.54
675	0.27	0.2	0.24	0.18	0.89
676	0	0	0.01	0.01	0.02
678	0.24	0.34	0.29	0.33	1.2
TOTAL	1.62	1.51	1.26	1.6	5.99

STATE: KENTUCKY

ZIP CODE	QUARTER 1	QUARTER 2	QUARTER 3	QUARTER 4	TOTAL GRAMS
402	0.15	0.44	0.2	0.3	1.09
404	0.03	0.03	0.04	0.06	0.16
408	0.01	0	0.01	0	0.02
410	4.75	2.5	2.5	2.5	12.25
411	0.04	0.13	0.04	0.06	0.27
423	0.02	0.81	0.88	0.93	2.64

TOTAL		5	3.91	3.67	3.85	16.43

STATE:		LOUISIANA				
ZIP CODE		QUARTER 1	QUARTER 2	QUARTER 3	QUARTER 4	TOTAL GRAMS
	700	0	0	0	0.02	0.02
	701	0.2	0.28	0.15	0.28	0.91
	704	0.69	0.8	0.65	0.87	3.01
	705	0.3	0.13	0.3	0.21	0.94
	708	0.1	0.18	0.1	0.17	0.55
	710	0.03	0	0.04	0	0.07
	711	0.95	0.84	1.36	0.93	4.08
	712	0.04	0.05	0.02	0.01	0.12
	713	0.13	0.01	0.18	0.02	0.34
	714	0	0	0.04	0	0.04
TOTAL		2.44	2.29	2.84	2.51	10.08

STATE:		MAINE				
ZIP CODE		QUARTER 1	QUARTER 2	QUARTER 3	QUARTER 4	TOTAL GRAMS
	39	0.13	0.16	0.08	0.14	0.51
	40	0.12	0.21	0.22	0.22	0.77
	41	0.26	0.16	0.23	0.06	0.71
	42	0.01	0	0	0	0.01
	44	0.06	0.23	0.09	0.12	0.5
	46	0.02	0.03	0.01	0.03	0.09
	48	0.02	0	0	0.08	0.1
TOTAL		0.62	0.79	0.63	0.65	2.69

STATE:		MARYLAND				
ZIP CODE		QUARTER 1	QUARTER 2	QUARTER 3	QUARTER 4	TOTAL GRAMS
	207	0.02	0	0	0.02	0.04
	210	0.22	0.19	0.22	0.07	0.7
	212	0.66	0.05	0.63	0.82	2.16
	217	0	0	0	0.01	0.01
TOTAL		0.9	0.24	0.85	0.92	2.91

STATE: MASSACHUSETTS

ZIP CODE	QUARTER 1	QUARTER 2	QUARTER 3	QUARTER 4	TOTAL GRAMS
18	0	0.01	0	0	0.01
21	0.2	0.13	0.25	0.18	0.76
24	1.15	0.59	0.4	0.55	2.69
TOTAL	1.35	0.73	0.65	0.73	3.46

STATE: MICHIGAN

ZIP CODE	QUARTER 1	QUARTER 2	QUARTER 3	QUARTER 4	TOTAL GRAMS
481	0.23	0.24	0.2	0.15	0.82
483	0.18	0.16	0.2	0.28	0.82
485	0.04	0	0.02	0.02	0.08
486	0.03	0	0.01	0	0.04
488	0.26	0.67	0.34	0.52	1.79
489	0.05	0.05	0	0.05	0.15
490	0.08	0.1	0.18	0.1	0.46
492	0.04	0.03	0	0.04	0.11
494	0	0	0	0.02	0.02
497	0	0.03	0	0.03	0.06
498	0	0.04	0.1	0	0.14
TOTAL	0.91	1.32	1.05	1.21	4.49

STATE: MINNESOTA

ZIP CODE	QUARTER 1	QUARTER 2	QUARTER 3	QUARTER 4	TOTAL GRAMS
550	0.08	0.09	0.07	0.12	0.36
551	1.03	0.53	0.66	0.93	3.15
553	0.01	0.17	0	0.02	0.2
554	1.92	2.36	2.11	2.49	8.88
557	0.01	0	0	0	0.01
558	0	0.01	0	0	0.01
559	0.1	0.2	0.23	0.33	0.86
560	0	0	0	0.01	0.01
561	0	0	0.05	0	0.05

	562	0.1	0.05	0.11	0.12	0.38
	563	0	0	0	0.03	0.03
	565	0	0	0.01	0	0.01
TOTAL		3.25	3.41	3.24	4.05	13.95

STATE:	MISSISSIPPI				
ZIP CODE	QUARTER 1	QUARTER 2	QUARTER 3	QUARTER 4	TOTAL GRAMS
387	0	0.01	0	0	0.01
389	0	0.01	0	0	0.01
390	0.02	0.03	0.02	0	0.07
393	0.02	0.04	0.03	0.02	0.11
394	0	0.02	0.06	0.03	0.11
395	0	0.01	0	0	0.01
396	0	0	0.04	0.02	0.06
397	0.03	0	0.02	0	0.05
TOTAL	0.07	0.12	0.17	0.07	0.43

STATE:	MISSOURI				
ZIP CODE	QUARTER 1	QUARTER 2	QUARTER 3	QUARTER 4	TOTAL GRAMS
631	0	0.06	0.04	0.04	0.14
636	0.38	0.4	0.33	0.41	1.52
640	0.07	0.08	0.01	0.09	0.25
641	0.05	0.09	0	0.1	0.24
646	0.09	0.05	0	0	0.14
652	0.03	0.01	0.03	0.03	0.1
654	0.24	0.17	0.22	0.2	0.83
657	0.35	0.14	0.27	0.35	1.11
TOTAL	1.21	1	0.9	1.22	4.33

STATE:	MONTANA				
ZIP CODE	QUARTER 1	QUARTER 2	QUARTER 3	QUARTER 4	TOTAL GRAMS
590	0.12	0.06	0.09	0.1	0.37
591	0	0.02	0	0.01	0.03
592	0.01	0	0.03	0	0.04

	596	0.05	0	0.02	0	0.07
	597	0.12	0.15	0.14	0.17	0.58
	598	0.06	0.04	0.05	0.06	0.21
TOTAL		0.36	0.27	0.33	0.34	1.3

STATE: NEBRASKA

ZIP CODE		QUARTER 1	QUARTER 2	QUARTER 3	QUARTER 4	TOTAL GRAMS
	680	0	0	0	0.01	0.01
	681	0.08	0.1	0.23	0	0.41
	683	0.12	0.02	0.03	0.09	0.26
	684	0.03	0.04	0.05	0.03	0.15
	685	0.01	0	0	0	0.01
	686	0	0.01	0.01	0	0.02
	687	0.1	0.01	0.33	0.19	0.63
	688	0.09	0.13	0.12	0.19	0.53
	689	0.35	0.35	0.24	0.38	1.32
	691	0.04	0.04	0.05	0.04	0.17
	693	0.03	0	0.02	0.01	0.06
TOTAL		0.85	0.7	1.08	0.94	3.57

STATE: NEVADA

ZIP CODE		QUARTER 1	QUARTER 2	QUARTER 3	QUARTER 4	TOTAL GRAMS
	890	0.01	0	0.03	0.01	0.05
	891	0.81	1.05	1.16	1.61	4.63
	894	0.05	0.05	0.07	0.1	0.27
	895	0.38	0.44	0.46	0.36	1.64
	898	0.03	0.05	0.03	0.28	0.39
TOTAL		1.28	1.59	1.75	2.36	6.98

STATE: NEW HAMPSHIRE

ZIP CODE		QUARTER 1	QUARTER 2	QUARTER 3	QUARTER 4	TOTAL GRAMS
	30	0.09	0	0	0.03	0.12
	32	0.03	0.02	0.01	0.02	0.08
	37	0	0	0	0.01	0.01

	38	0.36	0.4	0.43	0.55	1.74
TOTAL		0.48	0.42	0.44	0.61	1.95

STATE: NEW JERSEY

ZIP CODE	QUARTER 1	QUARTER 2	QUARTER 3	QUARTER 4	TOTAL GRAMS
70	0.02	0.03	0.02	0.13	0.2
74	0.02	0.01	0.06	0.02	0.11
75	0	0.05	0	0.02	0.07
77	0.07	0.02	0.04	0.07	0.2
88	0.08	0.16	0.11	0.11	0.46
89	0.01	0	0	0	0.01
TOTAL	0.2	0.27	0.23	0.35	1.05

STATE: NEW MEXICO

ZIP CODE	QUARTER 1	QUARTER 2	QUARTER 3	QUARTER 4	TOTAL GRAMS
871	0.87	0.83	1.14	1.13	3.97
874	0	0.05	0	0.02	0.07
875	0	0	0	0.06	0.06
TOTAL	0.87	0.88	1.14	1.21	4.1

STATE: NEW YORK

ZIP CODE	QUARTER 1	QUARTER 2	QUARTER 3	QUARTER 4	TOTAL GRAMS
100	0.1	0.1	0.29	2.35	2.84
104	0	0	0.08	0.01	0.09
105	0.03	0.05	0.14	0.1	0.32
106	0.03	0	0	0	0.03
112	0.62	0.73	0.78	1.09	3.22
113	0	0	0	0.01	0.01
117	0.1	0	0.05	0	0.15
122	0.15	0.1	0.06	0.16	0.47
123	0.05	0.02	0.04	0.05	0.16
129	0.02	0	0	0.03	0.05
135	0	0.02	0	0.03	0.05
136	0.02	0	0	0	0.02

	138	0.02	0	0.04	0.01	0.07
	142	0.07	0.1	0.06	0.14	0.37
	146	0.73	0	0	0.07	0.8
	148	0.19	0.1	0.12	0.12	0.53
TOTAL		2.13	1.22	1.66	4.17	9.18

STATE: NORTH CAROLINA

ZIP CODE		QUARTER 1	QUARTER 2	QUARTER 3	QUARTER 4	TOTAL GRAMS
	270	0.14	0.15	0.13	0.21	0.63
	271	0.57	0.8	0.58	0.65	2.6
	272	0.1	0.07	0.13	0.05	0.35
	273	0.02	0.01	0.04	0.05	0.12
	274	0.43	0.8	0.4	0.67	2.3
	276	0.14	0.08	0.14	0.11	0.47
	277	0	0.02	0	0.04	0.06
	278	0	0	0	0.03	0.03
	280	0.02	0	0.01	0.04	0.07
	281	0.05	0.08	0.06	0.12	0.31
	282	0.87	0.87	0.99	1.07	3.8
	283	0.11	0.1	0.07	0.2	0.48
	286	0.33	0.4	0.37	0.26	1.36
TOTAL		2.78	3.38	2.92	3.5	12.58

STATE: NORTH DAKOTA

ZIP CODE		QUARTER 1	QUARTER 2	QUARTER 3	QUARTER 4	TOTAL GRAMS
	582	0.03	0.06	0.05	0.06	0.2
	584	0.08	0.07	0.04	0.05	0.24
	585	0	0.14	0	0	0.14
	586	0	0.01	0	0	0.01
TOTAL		0.11	0.28	0.09	0.11	0.59

STATE: OHIO

ZIP CODE		QUARTER 1	QUARTER 2	QUARTER 3	QUARTER 4	TOTAL GRAMS
	430	0	0	0.13	0.05	0.18

432	0	0	0	0.24	0.24
433	0.01	0.02	0.03	0.04	0.1
435	0.02	0.02	0.03	0.05	0.12
436	0	0	0	0.15	0.15
437	0	0	0.01	0	0.01
440	0.15	0.15	0.18	0.25	0.73
441	2.67	4.37	1.28	2.59	10.91
442	0.05	0.09	0.1	0.04	0.28
443	0	0.04	0.06	0.04	0.14
444	0.28	0.29	0.29	0.12	0.98
445	0.78	0.46	0.76	0.66	2.66
448	0.04	0.05	0	0.06	0.15
449	0.03	0	0	0	0.03
452	0	0	0	0.05	0.05
454	0.46	0.65	0.64	0.64	2.39
TOTAL	4.49	6.14	3.51	4.98	19.12

STATE: OKLAHOMA

ZIP CODE	QUARTER 1	QUARTER 2	QUARTER 3	QUARTER 4	TOTAL GRAMS
731	0	0.04	0	0.03	0.07
740	0	0.04	0	0.01	0.05
741	0.22	0.12	0.14	0	0.48
744	0.31	0.48	0.4	0.43	1.62
748	0.3	0.33	0.3	0.37	1.3
TOTAL	0.83	1.01	0.84	0.84	3.52

STATE: OREGON

ZIP CODE	QUARTER 1	QUARTER 2	QUARTER 3	QUARTER 4	TOTAL GRAMS
970	0.66	0.58	0.77	0.98	2.99
971	0.05	0.05	0.09	0.11	0.3
972	2.53	2.53	4.08	2.77	11.91
973	0	0	0.03	0.01	0.04
974	0	0.01	0	0.01	0.02
975	0	0.2	0	0	0.2

	976	0.57	0.93	0.67	0.96	3.13
	977	0.03	0.02	0.03	0.03	0.11
	978	0.01	0	0	0	0.01
TOTAL		3.85	4.32	5.67	4.87	18.71

STATE: PENNSYLVANIA

ZIP CODE		QUARTER 1	QUARTER 2	QUARTER 3	QUARTER 4	TOTAL GRAMS
	152	0.02	0	0	0	0.02
	161	0.03	0	0	0	0.03
	163	0.09	0.04	0.03	0.05	0.21
	164	0.02	0	0	0	0.02
	165	0.11	0.11	0.05	0.04	0.31
	170	0.1	0.09	0.1	0.22	0.51
	171	0.25	0	0.1	0.1	0.45
	173	0	0.01	0	0	0.01
	174	0.08	0.05	0.07	0.05	0.25
	181	0	0.12	0	0	0.12
	185	0	0.01	0	0	0.01
	190	0	0	0.02	0	0.02
	191	0	0.05	0	0	0.05
TOTAL		0.7	0.48	0.37	0.46	2.01

STATE: RHODE ISLAND

ZIP CODE		QUARTER 1	QUARTER 2	QUARTER 3	QUARTER 4	TOTAL GRAMS
	28	0.05	0.08	0.13	0	0.26
TOTAL		0.05	0.08	0.13	0	0.26

STATE: SOUTH CAROLINA

ZIP CODE		QUARTER 1	QUARTER 2	QUARTER 3	QUARTER 4	TOTAL GRAMS
	293	0.12	0.04	0.05	0.13	0.34
	294	0.01	0.01	0	0	0.02
TOTAL		0.13	0.05	0.05	0.13	0.36

STATE: SOUTH DAKOTA

ZIP CODE	QUARTER 1	QUARTER 2	QUARTER 3	QUARTER 4	TOTAL GRAMS
570	0.05	0	0.02	0.04	0.11
572	0.06	0.06	0.08	0.02	0.22
TOTAL	0.11	0.06	0.1	0.06	0.33

STATE: TENNESSEE

ZIP CODE	QUARTER 1	QUARTER 2	QUARTER 3	QUARTER 4	TOTAL GRAMS
371	0.92	1.01	1.08	1.11	4.12
372	0	0	0	0.35	0.35
376	0	0.02	0.01	0	0.03
379	0	0	0	0.05	0.05
382	0.03	0.05	0.07	0.02	0.17
384	0.02	0	0	0.03	0.05
TOTAL	0.97	1.08	1.16	1.56	4.77

STATE: TEXAS

ZIP CODE	QUARTER 1	QUARTER 2	QUARTER 3	QUARTER 4	TOTAL GRAMS
750	0	0.09	0.31	0	0.4
751	0.12	0.09	0.12	0.18	0.51
752	0.25	0.14	0	0.18	0.57
755	0	0.01	0	0	0.01
756	0	0	0.02	0	0.02
757	0	0	0.01	0	0.01
760	0	0.25	0	0	0.25
761	0	0.01	0	0.01	0.02
770	0.36	0.5	0.44	0.76	2.06
773	0.05	0	0	0.04	0.09
774	0.04	0.05	0	0	0.09
775	0.09	0.15	0.2	0.2	0.64
777	0.02	0	0.03	0.01	0.06
779	0	0	0.02	0	0.02

	780	0.02	0.06	0.07	0.05	0.2
	782	1.66	1.65	1.65	1.82	6.78
	785	0.02	0	0	0	0.02
	787	0.01	0.03	0	0.02	0.06
	788	0.01	0.04	0.02	0	0.07
	794	0	0	0.03	0.09	0.12
	797	0.01	0.01	0.01	0	0.03
	799	2.4	2.99	2.29	2.36	10.04
TOTAL		5.06	6.07	5.22	5.72	22.07

STATE: UTAH

ZIP CODE		QUARTER 1	QUARTER 2	QUARTER 3	QUARTER 4	TOTAL GRAMS
	841	0	0.03	0.15	1.18	1.36
	844	0.44	0.31	0.31	0.58	1.64
	845	0	0	0.02	0	0.02
	847	0.03	0.01	0.04	0.01	0.09
TOTAL		0.47	0.35	0.52	1.77	3.11

STATE: VERMONT

ZIP CODE		QUARTER 1	QUARTER 2	QUARTER 3	QUARTER 4	TOTAL GRAMS
	56	0.01	0	0.01	0	0.02
	57	0	0	0	0.02	0.02
TOTAL		0.01	0	0.01	0.02	0.04

STATE: VIRGINIA

ZIP CODE		QUARTER 1	QUARTER 2	QUARTER 3	QUARTER 4	TOTAL GRAMS
	201	0.02	0	0.04	0	0.06
	229	0.06	0.03	0.03	0.03	0.15
	232	0.43	0.41	0.35	0.46	1.65
	234	0.13	0.16	0.17	0.15	0.61
	236	0.03	0.02	0	0.01	0.06
	237	0	0	0	0.01	0.01
	240	0.08	0.06	0.09	0.08	0.31
	241	0.02	0	0	0	0.02

	242	0.1	0.45	0	0.2	0.75
TOTAL		0.87	1.13	0.68	0.94	3.62

STATE: WASHINGTON

ZIP CODE	QUARTER 1	QUARTER 2	QUARTER 3	QUARTER 4	TOTAL GRAMS
980	0.06	0.07	0.1	0.12	0.35
981	1.95	2.37	2.64	2.27	9.23
982	0.2	0.2	0.12	0.3	0.82
983	0.17	0.43	0.21	0.19	1
984	0.09	0.14	0.18	0.19	0.6
985	0.01	0.01	0	0	0.02
986	0	0.01	0.01	0	0.02
988	0	0	0.04	0	0.04
989	0.01	0.03	0.06	0	0.1
991	0	0.01	0.01	0.06	0.08
992	0.03	0.05	0.04	0.08	0.2
993	0.01	0.05	0.07	0.07	0.2
TOTAL	2.53	3.37	3.48	3.28	12.66

STATE: WISCONSIN

ZIP CODE	QUARTER 1	QUARTER 2	QUARTER 3	QUARTER 4	TOTAL GRAMS
530	0.55	0.53	0.55	0.51	2.14
531	0.01	0.02	0.04	0.01	0.08
532	2.4	2.96	2.89	3.15	11.4
535	0.3	0.26	0.26	0.35	1.17
537	0.62	0.68	0.66	0.89	2.85
538	0.1	0.23	0.15	0.28	0.76
539	0.01	0.01	0	0.02	0.04
542	0	0	0	0.06	0.06
544	0.01	0	0.01	0.02	0.04
546	0.05	0.06	0.03	0.07	0.21
547	0.08	0.07	0.23	0.16	0.54
TOTAL	4.13	4.82	4.82	5.52	19.29

STATE: WYOMING

ZIP CODE	QUARTER 1	QUARTER 2	QUARTER 3	QUARTER 4	TOTAL GRAMS
823	0	0	0	0.06	0.06
826	0.01	0	0	0.03	0.04
828	0	0	0.02	0	0.02
TOTAL	0.01	0	0.02	0.09	0.12

DRUG CODE: 9739 DRUG NAME: REMIFENTANIL

STATE: ALABAMA

ZIP CODE	QUARTER 1	QUARTER 2	QUARTER 3	QUARTER 4	TOTAL GRAMS
350	0	0.01	0	0	0.01
352	1.86	2.36	2.2	1.87	8.29
354	0.02	0	0.03	0.01	0.06
356	0.04	0.06	0.04	0.1	0.24
357	0	0	0.01	0	0.01
358	0.05	0.04	0.06	0	0.15
361	0.54	0.51	0.5	0.45	2
363	0.06	0.08	0.06	0.08	0.28
366	0.59	0.34	0.54	0.63	2.1
368	0	0.01	0	0	0.01
TOTAL	3.16	3.41	3.44	3.14	13.15

STATE: ALASKA

ZIP CODE	QUARTER 1	QUARTER 2	QUARTER 3	QUARTER 4	TOTAL GRAMS
995	2.34	2.68	2.37	2.7	10.09
996	0.16	0.07	0.22	0.27	0.72
997	0.23	0.26	0.19	0.17	0.85
999	0	0	0.02	0	0.02
TOTAL	2.73	3.01	2.8	3.14	11.68

RETAIL DRUG DISTRIBUTION BY ZIP CODE WITHIN STATE BY GRAMS WT

STATE: ARIZONA

ZIP CODE	QUARTER 1	QUARTER 2	QUARTER 3	QUARTER 4	TOTAL GRAMS
850	1.82	1.59	1.59	1.88	6.88
851	0	0	0	0.03	0.03
852	0.84	0.69	0.6	0.77	2.9
853	0.16	0.1	0.24	0.4	0.9
855	0.03	0	0.01	0.05	0.09
857	1.03	1.15	0.83	0.89	3.9
859	0.04	0.03	0.06	0	0.13
860	0.22	0.18	0.3	0.14	0.84
863	0.01	0	0	0	0.01
864	0.01	0	0	0	0.01
TOTAL	4.16	3.74	3.63	4.16	15.69

STATE: ARKANSAS

ZIP CODE	QUARTER 1	QUARTER 2	QUARTER 3	QUARTER 4	TOTAL GRAMS
720	0.06	0.06	0.13	0.05	0.3
722	1.43	1.45	1.59	1.37	5.84
724	0.04	0	0	0.02	0.06
725	0	0	0	0.01	0.01
727	0.01	0	0.04	0.01	0.06
728	0	0	0.06	0	0.06
729	0.03	0.01	0	0.02	0.06
TOTAL	1.57	1.52	1.82	1.48	6.39

STATE: CALIFORNIA

ZIP CODE	QUARTER 1	QUARTER 2	QUARTER 3	QUARTER 4	TOTAL GRAMS
900	4.41	4.45	4.03	4.21	17.1
902	0.53	0.22	0.35	0.28	1.38
904	0.68	0.68	0.83	0.96	3.15
905	0.45	0.33	0.38	0.34	1.5
906	0	0	0.01	0	0.01
907	0.11	0.09	0.26	0.14	0.6
908	0.28	0.3	0.32	0.4	1.3
910	0.04	0.04	0.03	0.02	0.13

911	0.28	0.26	0.14	0.24	0.92
912	0	0	0.06	0.04	0.1
913	0.15	0.04	0.15	0.15	0.49
917	0.07	0.03	0.11	0.11	0.32
919	0.19	0.21	0.21	0.15	0.76
920	0.78	0.91	0.97	1.11	3.77
921	0.92	0.89	1.27	0.89	3.97
922	0.24	0.25	0.19	0.27	0.95
923	0.96	1.31	0.94	0.96	4.17
924	0.26	0.24	0.3	0.12	0.92
925	0.26	0.19	0.28	0.2	0.93
926	1.6	1.94	1.46	2.13	7.13
927	0.21	0.21	0.21	0.23	0.86
928	1.84	1.87	1.97	1.78	7.46
930	0.33	0.49	0.3	0.44	1.56
931	0.45	0.64	0.37	0.43	1.89
932	0.32	0.08	0.15	0.21	0.76
933	0.1	0.15	0.05	0.1	0.4
934	0.05	0.01	0.05	0.08	0.19
935	0.02	0.01	0	0.01	0.04
936	0.31	0.43	0.27	0.24	1.25
937	1.13	1.56	0.61	0.99	4.29
939	0.04	0.04	0.01	0.04	0.13
940	1.82	1.44	1.5	1.45	6.21
941	3.11	4.85	1.56	3.03	12.55
943	5.32	4.32	5.43	4.89	19.96
944	0	0.03	0.02	0.07	0.12
945	0.77	0.81	1.49	0.54	3.61
946	0.1	0.12	0.09	0.18	0.49
948	0	0	0.02	0	0.02
949	0.1	0.05	0.09	0.1	0.34
950	0.35	0.31	0.22	0.24	1.12
951	1.51	1.2	1.27	1.26	5.24
952	0.09	0.09	0.1	0.14	0.42
953	0.06	0.08	0.03	0.05	0.22
954	0.09	0.1	0.14	0.19	0.52
955	0.09	0.09	0.05	0	0.23

	956	0.76	0.59	0.91	1.08	3.34
	957	0.01	0	0.01	0	0.02
	958	1.84	2.31	1.82	3.65	9.62
	959	0	0.05	0.02	0.02	0.09
	960	0.18	0.25	0.2	0.18	0.81
TOTAL		33.21	34.56	31.25	34.34	133.36

STATE: COLORADO

ZIP CODE		QUARTER 1	QUARTER 2	QUARTER 3	QUARTER 4	TOTAL GRAMS
	800	2.53	2.6	3.03	3.56	11.72
	801	0.44	0.53	0.31	0.51	1.79
	802	1.05	1.03	0.99	0.95	4.02
	803	0.27	0.3	0.26	0.17	1
	804	0.23	0.15	0.04	0.04	0.46
	805	0.03	0.04	0.2	0.11	0.38
	806	0.1	0.12	0.1	0.04	0.36
	807	0.01	0.01	0.01	0	0.03
	808	0	0.21	0	0	0.21
	809	0.18	0.27	0.19	0.23	0.87
	810	0.38	0.44	0.28	0.63	1.73
	812	0	0	0	0.01	0.01
	813	0.39	0.33	0.29	0.4	1.41
	814	0.01	0.01	0.03	0.02	0.07
	815	0.26	0.42	0.36	0.4	1.44
	816	0.08	0.07	0.1	0.09	0.34
TOTAL		5.96	6.53	6.19	7.16	25.84

STATE: CONNECTICUT

ZIP CODE		QUARTER 1	QUARTER 2	QUARTER 3	QUARTER 4	TOTAL GRAMS
	60	0.12	0.11	0.1	0.17	0.5
	61	1.01	0.38	0.46	0.56	2.41
	62	0	0.02	0.04	0.08	0.14
	63	0.23	0.2	0.18	0.25	0.86
	64	0.43	0.56	0.6	0.76	2.35

65	5.93	5.01	5.19	5.32	21.45
66	1.31	0.71	0.5	0.69	3.21
67	0.14	0.09	0.1	0.06	0.39
68	0.36	0.31	0.38	0.52	1.57
69	0.28	0.33	0.27	0.3	1.18
TOTAL	9.81	7.72	7.82	8.71	34.06

STATE: DELAWARE

ZIP CODE	QUARTER 1	QUARTER 2	QUARTER 3	QUARTER 4	TOTAL GRAMS
197	0.6	0.72	0.74	0.9	2.96
198	1.75	3.01	1.83	1.44	8.03
199	0.32	0.29	0.24	0.2	1.05
TOTAL	2.67	4.02	2.81	2.54	12.04

STATE: DISTRICT OF COLUMBIA

ZIP CODE	QUARTER 1	QUARTER 2	QUARTER 3	QUARTER 4	TOTAL GRAMS
200	0.83	0.86	0.52	0.56	2.77
204	0.05	0	0	0.02	0.07
TOTAL	0.88	0.86	0.52	0.58	2.84

STATE: FLORIDA

ZIP CODE	QUARTER 1	QUARTER 2	QUARTER 3	QUARTER 4	TOTAL GRAMS
320	0.01	0	0.02	0.02	0.05
321	0.11	0.05	0.03	0.04	0.23
322	1.59	2	1.68	1.9	7.17
323	0.08	0	0.04	0.02	0.14
325	0.92	0.92	0.69	1	3.53
326	1.32	1.87	1.41	1.97	6.57
327	0.83	0.75	0.83	0.87	3.28
328	2.4	2.77	2.47	2.9	10.54
329	0.11	0.2	0.21	0.08	0.6
330	0.45	0.48	0.53	0.62	2.08
331	4.96	4.61	4.85	4.31	18.73

	333	0.78	1.06	0.9	1.07	3.81
	334	3.63	3.77	3.35	4.41	15.16
	335	0.19	0.15	0.16	0.52	1.02
	336	4.68	4.56	4.63	6.1	19.97
	337	0.82	0.77	0.79	0.83	3.21
	338	0.27	0.44	0.35	0.26	1.32
	339	0.29	0.24	0.23	0.16	0.92
	341	0.13	0.08	0.14	0.03	0.38
	342	0.4	0.35	0.41	0.49	1.65
	344	0.1	0.14	0.06	0.06	0.36
	346	0.5	0.38	0.33	0.39	1.6
	347	0.79	0.77	0.68	0.94	3.18
	349	0.39	0.56	0.44	0.56	1.95
TOTAL		25.75	26.92	25.23	29.55	107.45

STATE: GEORGIA

ZIP CODE		QUARTER 1	QUARTER 2	QUARTER 3	QUARTER 4	TOTAL GRAMS
	300	0.92	0.67	0.97	1.02	3.58
	301	0.1	0.18	0.1	0.13	0.51
	302	0.4	0.22	0.31	0.18	1.11
	303	0.77	1.06	0.9	0.69	3.42
	305	0.05	0.06	0.04	0.06	0.21
	306	0.08	0.15	0.06	0.09	0.38
	307	0.06	0.04	0.05	0.06	0.21
	309	1.38	1.52	1.32	1.85	6.07
	310	0.01	0	0	0	0.01
	312	0.57	0.51	0.58	0.51	2.17
	313	0	0.18	0	0	0.18
	314	0.09	0.16	0.11	0.05	0.41
	315	0.06	0.04	0.06	0	0.16
	316	0	0.03	0.04	0.02	0.09
	317	0.15	0.2	0.08	0.13	0.56
	319	0	0.04	0.03	0	0.07
	398	0.01	0.03	0.02	0	0.06
TOTAL		4.65	5.09	4.67	4.79	19.2

STATE: GUAM

ZIP CODE	QUARTER 1	QUARTER 2	QUARTER 3	QUARTER 4	TOTAL GRAMS
969	0.02	0.04	0	0.25	0.31
TOTAL	0.02	0.04	0	0.25	0.31

STATE: HAWAII

ZIP CODE	QUARTER 1	QUARTER 2	QUARTER 3	QUARTER 4	TOTAL GRAMS
967	0.01	0.06	0.02	0.06	0.15
968	1.28	1.07	1.32	1.32	4.99
TOTAL	1.29	1.13	1.34	1.38	5.14

STATE: IDAHO

ZIP CODE	QUARTER 1	QUARTER 2	QUARTER 3	QUARTER 4	TOTAL GRAMS
832	0.07	0.09	0.11	0.06	0.33
833	0.49	0.29	0.23	0.13	1.14
834	0.67	0.66	0.53	0.78	2.64
836	0.14	0.18	0.11	0.23	0.66
837	0.82	0.9	1.12	0.83	3.67
838	0.06	0.07	0.02	0.11	0.26
TOTAL	2.25	2.19	2.12	2.14	8.7

STATE: ILLINOIS

ZIP CODE	QUARTER 1	QUARTER 2	QUARTER 3	QUARTER 4	TOTAL GRAMS
600	1.29	0.93	0.94	1	4.16
601	2.78	2.26	2.62	2.63	10.29
602	0.46	0.55	0.36	0.68	2.05
603	0	0	0.05	0.2	0.25
604	1.13	1.4	1.29	1.54	5.36
605	0.7	0.87	0.88	1.27	3.72
606	6.17	5.68	5.9	5.97	23.72
607	0.14	0.14	0.12	0.14	0.54

	610	0	0	0.02	0.01	0.03
	611	0.79	1.07	0.71	1.13	3.7
	612	0.13	0.14	0.18	0.12	0.57
	614	0.22	0.2	0.22	0.29	0.93
	616	0.34	0.39	0.41	0.55	1.69
	617	0.07	0.14	0.11	0.16	0.48
	618	0.02	0.08	0.04	0.1	0.24
	619	0	0.04	0	0.02	0.06
	620	0	0	0.01	0.03	0.04
	622	0.21	0.24	0.2	0.21	0.86
	625	0	0.02	0.02	0.06	0.1
	627	0.24	0.21	0.39	0.66	1.5
	629	0.12	0.12	0.11	0.12	0.47
TOTAL		14.81	14.48	14.58	16.89	60.76

STATE:	INDIANA					
ZIP CODE	QUARTER 1		QUARTER 2	QUARTER 3	QUARTER 4	TOTAL GRAMS
	460	1.1	0.98	0.93	1.16	4.17
	461	0.08	0.12	0.08	0.08	0.36
	462	3.55	3.48	3.38	3.24	13.65
	463	0.49	0.45	0.48	0.52	1.94
	464	0.1	0.09	0.06	0.12	0.37
	465	0.52	0.43	0.57	0.76	2.28
	466	0.08	0.14	0.14	0.15	0.51
	468	0.38	0.34	0.5	0.36	1.58
	469	0.04	0.04	0.02	0.01	0.11
	471	0.1	0.15	0.1	0.05	0.4
	472	0.2	0.24	0.25	0.5	1.19
	473	0.69	0.6	0.51	0.55	2.35
	474	0.17	0.16	0.12	0.14	0.59
	475	0.11	0.12	0.16	0.05	0.44
	476	0.06	0.03	0.07	0.06	0.22
	477	0.17	0.12	0.27	0.08	0.64
	478	0.16	0.15	0.21	0.13	0.65
	479	0.1	0.11	0.12	0.15	0.48

TOTAL	8.1	7.75	7.97	8.11	31.93

STATE: IOWA

ZIP CODE	QUARTER 1	QUARTER 2	QUARTER 3	QUARTER 4	TOTAL GRAMS
500	0.22	0.22	0.16	0.18	0.78
501	0	0.06	0	0	0.06
502	0.02	0.02	0	0.02	0.06
503	0.01	0.04	0.02	0.02	0.09
504	0	0.01	0	0	0.01
505	0.05	0.05	0.04	0.03	0.17
507	0.14	0.12	0.2	0.18	0.64
510	0	0	0	0.04	0.04
512	0	0.02	0	0.04	0.06
520	0.15	0.1	0.12	0.13	0.5
522	2.6	2.47	2.19	1.42	8.68
524	0.05	0	0.01	0.06	0.12
527	0.03	0.03	0.04	0.02	0.12
528	0.3	0.1	0.55	0.3	1.25
TOTAL	3.57	3.24	3.33	2.44	12.58

STATE: KANSAS

ZIP CODE	QUARTER 1	QUARTER 2	QUARTER 3	QUARTER 4	TOTAL GRAMS
660	0.5	0.39	0.23	0.57	1.69
661	0.85	0.88	0.74	0.92	3.39
662	0.21	0.45	0.38	0.22	1.26
664	0	0.18	0	0	0.18
665	0.05	0.03	0.03	0.04	0.15
666	0.23	0.31	0.4	0.2	1.14
670	0.04	0.02	0.04	0.06	0.16
672	0.17	0.22	0.21	0.29	0.89
674	0.03	0.05	0.02	0.03	0.13
675	0.02	0	0.06	0	0.08
TOTAL	2.1	2.53	2.11	2.33	9.07

STATE: KENTUCKY

ZIP CODE	QUARTER 1	QUARTER 2	QUARTER 3	QUARTER 4	TOTAL GRAMS
402	1.71	1.21	1.38	1.48	5.78
403	0.02	0	0.07	0.01	0.1
404	0.35	0.15	0.29	0.33	1.12
405	1.32	1.38	1.74	1.72	6.16
407	0.02	0	0	0	0.02
410	0.12	0.1	0.08	0	0.3
411	0.1	0.13	0.18	0.11	0.52
415	0.02	0	0.02	0.02	0.06
420	0.36	0.26	0.36	0.26	1.24
421	0.97	0.9	1.09	0.67	3.63
422	0.03	0.1	0.01	0	0.14
423	0	0.03	0	0.01	0.04
424	0	0	0.02	0	0.02
425	0	0.01	0	0	0.01
427	0	0	0.02	0	0.02
TOTAL	5.02	4.27	5.26	4.61	19.16

STATE: LOUISIANA

ZIP CODE	QUARTER 1	QUARTER 2	QUARTER 3	QUARTER 4	TOTAL GRAMS
700	0.37	0.22	0.25	0.29	1.13
701	2.34	2.88	2.33	2.18	9.73
703	0.08	0.02	0.01	0.08	0.19
704	0.19	0.18	0.31	0.28	0.96
705	0.47	0.41	0.52	0.63	2.03
706	0	0	0.01	0	0.01
708	0.45	0.52	0.76	1.24	2.97
711	0.49	0.33	0.31	0.26	1.39
712	0.02	0	0	0	0.02
713	0	0.06	0	0	0.06
714	0	0.08	0	0	0.08
TOTAL	4.41	4.7	4.5	4.96	18.57

STATE: MAINE

ZIP CODE	QUARTER 1	QUARTER 2	QUARTER 3	QUARTER 4	TOTAL GRAMS
40	0.02	0.06	0.01	0.01	0.1
41	0.66	0.3	0.34	0.33	1.63
42	0.13	0.23	0.14	0.22	0.72
43	0.09	0.03	0.1	0.07	0.29
44	0.41	0.51	0.43	0.26	1.61
46	0.02	0.02	0.04	0.01	0.09
47	0.02	0	0	0.02	0.04
49	0.02	0.05	0.05	0.01	0.13
TOTAL	1.37	1.2	1.11	0.93	4.61

STATE: MARYLAND

ZIP CODE	QUARTER 1	QUARTER 2	QUARTER 3	QUARTER 4	TOTAL GRAMS
207	0.09	0.2	0.04	0.06	0.39
208	1.39	0.88	0.86	1.12	4.25
209	0.04	0	0.02	0.02	0.08
210	0.21	0.19	0.15	0.19	0.74
211	0.05	0	0.06	0	0.11
212	5.05	5.41	4.4	5.48	20.34
214	0.02	0.02	0.02	0.04	0.1
216	0.02	0	0.04	0	0.06
217	0.08	0.05	0.03	0.05	0.21
218	0.24	0.38	0.26	0.24	1.12
TOTAL	7.19	7.13	5.88	7.2	27.4

STATE: MASSACHUSETTS

ZIP CODE	QUARTER 1	QUARTER 2	QUARTER 3	QUARTER 4	TOTAL GRAMS
10	0.18	0.11	0.1	0	0.39
11	0.2	0.21	0.22	0.17	0.8
12	0.08	0.07	0.02	0.06	0.23
14	0.07	0.11	0.08	0.09	0.35
15	0.02	0.03	0.01	0	0.06

16	1.09	1.03	1.06	1.42	4.6
17	0.23	0.26	0.21	0.45	1.15
18	0.96	1.05	0.92	1.21	4.14
19	0.47	0.58	0.41	0.56	2.02
20	0.35	0.6	0.32	0.42	1.69
21	15.32	17.75	16.7	17.31	67.08
22	1.09	1.17	1.02	0.74	4.02
23	0.14	0.28	0.14	0.23	0.79
24	0.33	0.4	0.17	0.28	1.18
25	0.04	0.03	0.05	0.03	0.15
26	0.09	0.13	0.11	0.06	0.39
27	0.02	0.09	0.08	0.04	0.23
TOTAL	20.68	23.9	21.62	23.07	89.27

STATE: MICHIGAN

ZIP CODE	QUARTER 1	QUARTER 2	QUARTER 3	QUARTER 4	TOTAL GRAMS
480	0.79	1.31	0.57	1.09	3.76
481	3.68	4.27	4.24	3.66	15.85
482	2.61	2.91	2.39	2.6	10.51
483	0.97	0.84	1.21	0.81	3.83
485	0.02	0.08	0.04	0.1	0.24
486	0.34	0.33	0.29	0.35	1.31
487	0.29	0.31	0.28	0.18	1.06
489	0.17	0.15	0.16	0.14	0.62
490	0.16	0.25	0.09	0.23	0.73
492	0.12	0.11	0.08	0.09	0.4
494	0.04	0.11	0.05	0.05	0.25
495	0.26	0.23	0.2	0.12	0.81
497	0.03	0.03	0.06	0.05	0.17
498	0.03	0.02	0.02	0.02	0.09
TOTAL	9.51	10.95	9.68	9.49	39.63

STATE: MINNESOTA

ZIP CODE	QUARTER 1	QUARTER 2	QUARTER 3	QUARTER 4	TOTAL GRAMS
550	0	0.01	0.01	0	0.02

551	0.79	0.86	0.87	0.83	3.35
553	0.05	0.06	0.04	0.06	0.21
554	3.37	3.62	3.84	3.85	14.68
558	0.24	0.76	0.15	0.34	1.49
559	2.44	2.45	2.87	4.57	12.33
562	0	0.02	0	0	0.02
563	0.12	0.11	0.13	0.2	0.56
564	0.01	0.01	0.01	0	0.03
TOTAL	7.02	7.9	7.92	9.85	32.69

STATE: MISSISSIPPI

ZIP CODE	QUARTER 1	QUARTER 2	QUARTER 3	QUARTER 4	TOTAL GRAMS
392	0.37	0.41	0.23	0.25	1.26
394	0	0.12	0	0	0.12
395	0.18	0.07	0.04	0.06	0.35
396	0.01	0	0	0.01	0.02
397	0	0.03	0	0	0.03
TOTAL	0.56	0.63	0.27	0.32	1.78

STATE: MISSOURI

ZIP CODE	QUARTER 1	QUARTER 2	QUARTER 3	QUARTER 4	TOTAL GRAMS
630	0.07	0.06	0.09	0.06	0.28
631	2.09	2.25	2.36	2.62	9.32
633	0	0.02	0.01	0	0.03
637	0.26	0.17	0.25	0.28	0.96
638	0	0	0.02	0.04	0.06
640	0.07	0.06	0.06	0.08	0.27
641	1.44	1.48	1.78	1.34	6.04
645	0.05	0.1	0.04	0.05	0.24
648	0.04	0.08	0.14	0.12	0.38
650	0.17	0.1	0.15	0.17	0.59
651	0.06	0.06	0.04	0.12	0.28
652	0.55	0.65	0.68	0.44	2.32
654	0.06	0.06	0.06	0.06	0.24

	656	0.06	0.06	0.04	0.06	0.22
	658	0.1	0.16	0.14	0.14	0.54
TOTAL		5.02	5.31	5.86	5.58	21.77

STATE: MONTANA

ZIP CODE		QUARTER 1	QUARTER 2	QUARTER 3	QUARTER 4	TOTAL GRAMS
	591	0.03	0.12	0.09	0.05	0.29
	592	0.02	0	0.03	0	0.05
	593	0.01	0	0.01	0	0.02
	594	0.02	0.26	0	0.04	0.32
	596	0.1	0.08	0.08	0.04	0.3
	597	0.04	0.08	0.11	0.14	0.37
	598	1.22	1.25	1.38	1.69	5.54
	599	0.01	0.04	0.02	0.03	0.1
TOTAL		1.45	1.83	1.72	1.99	6.99

STATE: NEBRASKA

ZIP CODE		QUARTER 1	QUARTER 2	QUARTER 3	QUARTER 4	TOTAL GRAMS
	680	0.05	0.05	0.02	0.05	0.17
	681	0.85	0.67	0.47	0.68	2.67
	685	0.01	0.06	0.14	0.04	0.25
	686	0.01	0	0.01	0.01	0.03
	687	0	0	0.01	0	0.01
	688	0.07	0.1	0.11	0.19	0.47
	689	0.01	0.03	0.03	0.14	0.21
	691	0.01	0	0	0.02	0.03
	693	0.13	0.06	0.12	0.14	0.45
TOTAL		1.14	0.97	0.91	1.27	4.29

STATE: NEVADA

ZIP CODE		QUARTER 1	QUARTER 2	QUARTER 3	QUARTER 4	TOTAL GRAMS
	890	0.03	0.04	0.06	0.06	0.19
	891	0.27	0.24	0.43	0.24	1.18
	894	0.02	0.03	0.02	0.02	0.09
	895	0.42	0.43	0.58	0.62	2.05

	897	0.15	0	0	0.02	0.17
	898	0	0	0.02	0	0.02
TOTAL		0.89	0.74	1.11	0.96	3.7

STATE: NEW HAMPSHIRE

ZIP CODE		QUARTER 1	QUARTER 2	QUARTER 3	QUARTER 4	TOTAL GRAMS
	30	0.1	0.14	0.1	0.12	0.46
	31	0.29	0.32	0.43	0.68	1.72
	32	0.02	0	0.01	0.01	0.04
	33	0.08	0.13	0.1	0.16	0.47
	34	0	0.01	0.03	0	0.04
	35	0	0.01	0	0	0.01
	37	0.37	0.54	0.47	0.97	2.35
	38	0.29	0.37	0.29	0.48	1.43
TOTAL		1.15	1.52	1.43	2.42	6.52

STATE: NEW JERSEY

ZIP CODE		QUARTER 1	QUARTER 2	QUARTER 3	QUARTER 4	TOTAL GRAMS
	070	0.76	1.06	0.9	0.76	3.48
	071	1.93	1.12	1.31	1.96	6.32
	072	0.1	0.02	0.08	0.16	0.36
	073	0.1	0.08	0.12	0.12	0.42
	074	0.44	0.42	0.38	0.38	1.62
	075	0.48	0.77	0.64	0.62	2.51
	076	1.03	1.16	1.19	1.33	4.71
	077	0.73	1.11	0.89	1.37	4.1
	078	0	0.02	0.04	0.04	0.1
	079	1.22	0.98	0.88	1.16	4.24
	080	1.51	1.68	1.23	1.53	5.95
	081	1.14	0.81	0.81	0.97	3.73
	082	0.58	0.54	0.73	0.4	2.25
	084	0.2	0.3	0.42	0.18	1.1
	085	0.16	0.26	0.27	0.29	0.98
	086	0.17	0.3	0.26	0.31	1.04
	087	0.02	0.25	0.08	0.1	0.45

	088	0.64	0.54	0.7	0.73	2.61
	089	1.58	1.59	2.01	1.76	6.94
TOTAL		12.79	13.01	12.94	14.17	52.91

STATE:	NEW MEXICO					
ZIP CODE		QUARTER 1	QUARTER 2	QUARTER 3	QUARTER 4	TOTAL GRAMS
	871	0.43	0.69	0.73	0.49	2.34
	874	0.06	0.06	0.05	0.02	0.19
	875	0.14	0.13	0.18	0.24	0.69
	880	0.31	0.2	0.26	0.23	1
	882	0.02	0	0.01	0.01	0.04
	884	0.04	0.06	0.04	0.04	0.18
TOTAL		1	1.14	1.27	1.03	4.44

STATE:	NEW YORK					
ZIP CODE		QUARTER 1	QUARTER 2	QUARTER 3	QUARTER 4	TOTAL GRAMS
	100	19.87	20.51	20.33	21.26	81.97
	101	0.02	0.04	0	0	0.06
	103	0.2	0.31	0.2	0.22	0.93
	104	1.53	1.61	1.5	0.85	5.49
	105	0.96	0.96	1.18	1.57	4.67
	106	0.28	0.3	0.22	0.4	1.2
	107	0	0	0	0.02	0.02
	109	0.08	0.05	0.1	0.03	0.26
	110	3.58	3.71	4.42	3.72	15.43
	112	2.37	2.27	3.18	2.52	10.34
	113	0.16	0.16	0.3	0.29	0.91
	114	0.13	0.21	0.13	0.17	0.64
	115	1.03	0.83	1.09	0.82	3.77
	116	0	0	0.02	0	0.02
	117	0.78	0.76	0.78	0.91	3.23
	119	0.01	0.03	0	0	0.04
	120	0	0	0	0.04	0.04
	121	0.21	0.24	0.19	0.03	0.67
	122	1.06	1.02	1.24	0.76	4.08

	123	0.06	0.11	0.08	0.09	0.34
	124	0.02	0.04	0	0.03	0.09
	125	0.14	0.03	0.09	0.22	0.48
	126	0.19	0.31	0.32	0.52	1.34
	127	0.04	0.02	0.03	0.04	0.13
	128	0.04	0.06	0.06	0.03	0.19
	129	0	0.02	0	0	0.02
	132	1.25	1.35	1.27	1.4	5.27
	133	0.17	0.14	0.14	0.17	0.62
	134	0.01	0	0.02	0.02	0.05
	135	0.13	0.16	0.22	0.03	0.54
	136	0.07	0.05	0.09	0.06	0.27
	137	0.3	0.3	0.34	0.24	1.18
	138	0.02	0.03	0	0.02	0.07
	139	0.04	0.02	0.02	0	0.08
	140	0.02	0.01	0.03	0.01	0.07
	142	1.34	0.95	1.72	2.83	6.84
	144	0.02	0.06	0.05	0.02	0.15
	146	0.52	1	0.88	0.88	3.28
	147	0	0	0.01	0.01	0.02
	148	0.04	0.05	0.06	0.08	0.23
TOTAL		36.69	37.72	40.31	40.31	155.03

STATE:	NORTH CAROLINA				
ZIP CODE	QUARTER 1	QUARTER 2	QUARTER 3	QUARTER 4	TOTAL GRAMS
270	0	0.01	0	0.02	0.03
271	1.38	1.88	1.51	2.1	6.87
272	0.72	0.57	0.45	0.68	2.42
273	0.02	0	0.04	0	0.06
274	0.14	0.31	0.28	0.12	0.85
275	1.73	1.8	1.9	1.47	6.9
276	2.09	2.01	1.3	1.31	6.71
277	2.86	2.91	3.48	3.52	12.77
278	0.44	0.52	0.67	0.74	2.37
280	1.15	1.05	1.01	1.08	4.29
281	0.21	0.18	0.29	0.21	0.89

282	1.13	1.48	1.04	1.85	5.5
283	0.47	0.5	0.43	0.45	1.85
284	0.01	0	0.02	0	0.03
285	0.05	0.09	0.08	0.09	0.31
286	0.91	0.94	0.85	0.97	3.67
287	0.21	0.2	0.16	0.17	0.74
288	0.35	0.4	0.33	0.4	1.48
TOTAL	13.87	14.85	13.84	15.18	57.74

STATE: NORTH DAKOTA

ZIP CODE	QUARTER 1	QUARTER 2	QUARTER 3	QUARTER 4	TOTAL GRAMS
581	0.44	0.18	0.27	0.23	1.12
585	0.13	0.1	0.06	0.12	0.41
587	0	0.02	0.02	0.04	0.08
588	0.01	0.02	0.03	0	0.06
TOTAL	0.58	0.32	0.38	0.39	1.67

STATE: OHIO

ZIP CODE	QUARTER 1	QUARTER 2	QUARTER 3	QUARTER 4	TOTAL GRAMS
430	0.36	0.09	0.22	0.21	0.88
432	2.36	3.06	3.11	4.36	12.89
433	0.05	0.06	0	0.1	0.21
434	0	0	0.01	0	0.01
435	0	0.03	0	0	0.03
436	0.17	0.26	0.31	0.33	1.07
437	0.79	0.82	0.24	0.3	2.15
439	0.02	0	0	0	0.02
440	0.24	0.23	0.24	0.23	0.94
441	6.86	7.59	4.52	4.99	23.96
442	0.08	0.1	0.03	0.03	0.24
443	0.78	0.96	0.65	1.11	3.5
444	0.1	0.09	0.13	0.09	0.41
445	2.38	1.34	1.55	1.92	7.19
446	0.06	0.03	0.03	0.03	0.15

	447	0.07	0.03	0.1	0.09	0.29
	448	0.05	0.01	0.07	0.08	0.21
	449	0.05	0.07	0.04	0.07	0.23
	450	0.39	0.29	0.44	0.5	1.62
	452	2.89	3.37	2.98	3.6	12.84
	453	0	0	0.02	0	0.02
	454	1.03	1.1	0.84	1.55	4.52
	455	0.01	0.01	0	0	0.02
	456	0.24	0.24	0.16	0.54	1.18
	457	0.23	0.18	0.2	0.21	0.82
	458	0.06	0.04	0.04	0.1	0.24
TOTAL		19.27	20	15.93	20.44	75.64

STATE: OKLAHOMA

ZIP CODE		QUARTER 1	QUARTER 2	QUARTER 3	QUARTER 4	TOTAL GRAMS
	730	0.04	0.1	0	0.06	0.2
	731	1.96	1.92	1.98	1.96	7.82
	734	0.02	0	0.08	0.08	0.18
	735	0.05	0.05	0.14	0.02	0.26
	740	0.01	0.04	0.01	0.01	0.07
	741	0.45	0.75	0.39	0.44	2.03
	743	0	0	0.02	0	0.02
	744	0.04	0	0.02	0.03	0.09
	745	0.02	0.02	0.01	0.01	0.06
TOTAL		2.59	2.88	2.65	2.61	10.73

STATE: OREGON

ZIP CODE		QUARTER 1	QUARTER 2	QUARTER 3	QUARTER 4	TOTAL GRAMS
	970	0.4	0.27	0.42	0.34	1.43
	971	0.11	0.1	0.12	0.1	0.43
	972	0.36	0.52	0.56	0.46	1.9
	973	0.12	0.11	0.09	0.03	0.35
	974	0.88	1.12	0.87	1.17	4.04
	975	0.32	0.6	0.08	0.38	1.38
	977	0.5	0.53	0.38	0.43	1.84

	978	0	0.01	0.01	0	0.02
	979	0	0.01	0	0	0.01
TOTAL		2.69	3.27	2.53	2.91	11.4

STATE:	PENNSYLVANIA				
ZIP CODE	QUARTER 1	QUARTER 2	QUARTER 3	QUARTER 4	TOTAL GRAMS
150	0.36	0.51	0.33	0.39	1.59
151	0.5	0.36	0.54	0.55	1.95
152	7.65	8.28	8.12	9.07	33.12
153	0.07	0.07	0.04	0.05	0.23
155	0.02	0.01	0.01	0.02	0.06
156	0.08	0.1	0.06	0.15	0.39
158	0.08	0.07	0.13	0.15	0.43
159	0.12	0.09	0.07	0.09	0.37
160	0.29	0.47	0.31	0.54	1.61
161	0	0.01	0.01	0	0.02
162	0.01	0.03	0.03	0.01	0.08
165	0.46	0.45	0.26	0.55	1.72
166	0.02	0.05	0.06	0.28	0.41
168	0.04	0.12	0.03	0.04	0.23
170	0.98	0.99	1.01	0.54	3.52
171	0.07	0.04	0.15	0.03	0.29
172	0	0	0.02	0	0.02
174	0.04	0.1	0	0.02	0.16
175	0.1	0.1	0.1	0	0.3
176	0.01	0.02	0.01	0.03	0.07
178	0.91	1.36	1.06	1.12	4.45
179	0.04	0	0.02	0.02	0.08
180	1.17	1.38	0.81	1.13	4.49
181	0.92	1.12	0.9	1.07	4.01
182	0	0.01	0.01	0	0.02
183	0	0.31	0.26	0.16	0.73
184	0.08	0.06	0.05	0.09	0.28
185	0.5	0.37	0.5	0.45	1.82
186	0.01	0.03	0.02	0	0.06
187	0.52	0.52	0.56	0.48	2.08

	188	0.05	0	0.05	0.11	0.21
	189	0.06	0	0	0	0.06
	190	1.68	1.62	1.66	1.7	6.66
	191	18.36	16.11	16.97	16.31	67.75
	193	0.59	0.58	0.61	0.86	2.64
	194	0.3	0.19	0.23	0.24	0.96
	196	0.1	0.06	0.09	0.12	0.37
TOTAL		36.19	35.59	35.09	36.37	143.24

STATE:	RHODE ISLAND				
ZIP CODE	QUARTER 1	QUARTER 2	QUARTER 3	QUARTER 4	TOTAL GRAMS
28	0.13	0.01	0.1	0.16	0.4
29	0.55	0.5	0.66	1.06	2.77
TOTAL	0.68	0.51	0.76	1.22	3.17

STATE:	SOUTH CAROLINA				
ZIP CODE	QUARTER 1	QUARTER 2	QUARTER 3	QUARTER 4	TOTAL GRAMS
290	0	0.03	0	0.01	0.04
291	0.93	0.64	0.87	1.09	3.53
292	0.61	0.57	0.61	0.61	2.4
293	0.3	0.18	0.2	0.26	0.94
294	1.68	1.55	1.69	1.48	6.4
295	0.19	0.18	0.14	0.16	0.67
296	1.2	1.12	1.02	0.84	4.18
297	0.16	0.07	0.08	0.09	0.4
299	0.01	0.02	0.03	0.03	0.09
TOTAL	5.08	4.36	4.64	4.57	18.65

STATE:	SOUTH DAKOTA				
ZIP CODE	QUARTER 1	QUARTER 2	QUARTER 3	QUARTER 4	TOTAL GRAMS
571	0.08	0.1	0.11	0.02	0.31
572	0.01	0	0	0.01	0.02
573	0.02	0	0	0.01	0.03
574	0	0.04	0	0	0.04

	575	0	0	0	0.02	0.02
	577	0.36	0.52	0.33	0.26	1.47
TOTAL		0.47	0.66	0.44	0.32	1.89

STATE: TENNESSEE

ZIP CODE		QUARTER 1	QUARTER 2	QUARTER 3	QUARTER 4	TOTAL GRAMS
	370	0.32	0.31	0.31	0.26	1.2
	371	0.07	0.04	0.07	0.05	0.23
	372	3.08	3.61	4.28	4.19	15.16
	373	0	0.01	0.01	0	0.02
	374	0.07	0.23	0.18	0.24	0.72
	376	0.06	0.13	0.01	0.1	0.3
	378	0.18	0.09	0.71	0.07	1.05
	379	0.33	0.37	0.41	0.5	1.61
	380	0	0	0.04	0.02	0.06
	381	0.41	0.46	0.46	0.76	2.09
	382	0.03	0	0.01	0	0.04
	383	0.02	0.04	0.02	0.16	0.24
	384	0.09	0.11	0.16	0.12	0.48
	385	0	0.02	0.02	0	0.04
TOTAL		4.66	5.42	6.69	6.47	23.24

STATE: TEXAS

ZIP CODE		QUARTER 1	QUARTER 2	QUARTER 3	QUARTER 4	TOTAL GRAMS
	750	0.68	0.57	0.76	0.86	2.87
	751	0.15	0.12	0.06	0.15	0.48
	752	2.99	3.06	3.24	2.36	11.65
	753	3.26	3.62	3.33	3.4	13.61
	754	0.08	0.07	0.05	0.07	0.27
	756	0	0.04	0	0.02	0.06
	757	0.28	0.41	0.22	0.5	1.41
	759	0.01	0.01	0	0	0.02
	760	0.53	0.79	0.77	0.44	2.53
	761	3.66	3.63	4.28	4.88	16.45
	762	0.11	0.13	0.2	0.24	0.68

	763	0.02	0	0.03	0	0.05
	765	0.57	0.39	0.48	0.51	1.95
	767	0.05	0.07	0.17	0.15	0.44
	769	0.02	0.04	0	0	0.06
	770	6.25	4.12	5.07	4.94	20.38
	773	0.23	0.69	0.32	0.17	1.41
	774	0.43	0.33	0.59	0.33	1.68
	775	0.99	1.16	1.04	1.82	5.01
	778	0.13	0.15	0.09	0.11	0.48
	779	0.01	0	0.01	0.03	0.05
	781	0.02	0	0.05	0.1	0.17
	782	2.12	2.79	1.64	3.66	10.21
	784	0.7	0.69	0.58	0.63	2.6
	785	0.02	0.04	0.03	0.04	0.13
	786	0.21	0.21	0.26	0.24	0.92
	787	0.86	1.11	1.03	1.1	4.1
	791	0.06	0.02	0.04	0.04	0.16
	794	1.09	1.73	1.39	2.11	6.32
	796	0.09	0.08	0.15	0.08	0.4
	797	0.2	0.2	0.26	0.16	0.82
	798	0	0.01	0	0	0.01
	799	0.97	1.14	1.06	1.31	4.48
TOTAL		26.79	27.42	27.2	30.45	111.86

STATE: UTAH

ZIP CODE		QUARTER 1	QUARTER 2	QUARTER 3	QUARTER 4	TOTAL GRAMS
	840	1.81	2.02	1.77	2.15	7.75
	841	11.67	7.75	9.33	17.54	46.29
	843	0.07	0.08	0.01	0.05	0.21
	844	0.34	0.23	0.39	0.72	1.68
	846	1.99	1.97	2.21	2.61	8.78
	847	1.94	2.03	1.67	1.43	7.07
TOTAL		17.82	14.08	15.38	24.5	71.78

STATE: VERMONT

ZIP CODE	QUARTER 1	QUARTER 2	QUARTER 3	QUARTER 4	TOTAL GRAMS
50	0.18	0.28	0.1	0.18	0.74
54	0.49	0.62	0.41	0.54	2.06
57	0.01	0.01	0.01	0	0.03
TOTAL	0.68	0.91	0.52	0.72	2.83

STATE: VIRGINIA

ZIP CODE	QUARTER 1	QUARTER 2	QUARTER 3	QUARTER 4	TOTAL GRAMS
201	0.08	0.08	0.06	0.12	0.34
220	0.44	0.32	0.4	0.48	1.64
221	0.03	0.02	0.01	0.01	0.07
222	0.43	0.35	0.39	0.48	1.65
224	0	0.02	0	0	0.02
226	0.2	0.24	0.1	0.22	0.76
228	0.01	0.01	0	0.01	0.03
229	0.51	0.34	0.36	0.8	2.01
231	0.01	0	0	0	0.01
232	1.98	2.23	2.05	2.35	8.61
233	0.1	0.1	0.12	0.06	0.38
234	0.14	0.25	0.21	0.24	0.84
235	0.87	1.67	0.73	1.15	4.42
236	0.43	0.51	0.44	0.63	2.01
237	0.12	0.24	0.24	0.12	0.72
238	0.15	0.1	0.05	0.06	0.36
239	0	0	0	0.01	0.01
240	0.44	0.45	0.48	0.28	1.65
241	0.15	0.22	0.26	0.27	0.9
244	0	0.01	0.01	0	0.02
245	0.18	0.23	0.13	0.18	0.72
TOTAL	6.27	7.39	6.04	7.47	27.17

STATE: WASHINGTON

ZIP CODE	QUARTER 1	QUARTER 2	QUARTER 3	QUARTER 4	TOTAL GRAMS
980	0.5	0.49	0.79	0.64	2.42
981	8.53	9.04	10.59	7.74	35.9

	982	0.41	0.27	0.52	0.67	1.87
	983	0.08	0.1	0.12	0.09	0.39
	984	1.11	0.66	1.03	0.48	3.28
	985	0.27	0.41	0.34	0.23	1.25
	986	0.02	0.11	0.09	0.03	0.25
	988	0.08	0.06	0.16	0.09	0.39
	989	0.37	0.27	0.24	0.18	1.06
	991	0	0	0.03	0	0.03
	992	0.32	0.12	0.16	0.13	0.73
	993	1.02	0.55	0.57	0.3	2.44
TOTAL		12.71	12.08	14.64	10.58	50.01

STATE: WEST VIRGINIA

ZIP CODE		QUARTER 1	QUARTER 2	QUARTER 3	QUARTER 4	TOTAL GRAMS
	247	0.01	0.01	0.01	0	0.03
	249	0.01	0	0	0.01	0.02
	253	2.2	2.83	1.81	1.16	8
	254	0.01	0	0.03	0.04	0.08
	255	0.16	0.13	0.11	0.15	0.55
	257	0.67	0.74	0.65	0.93	2.99
	260	0	0	0.03	0.06	0.09
	261	0.17	0.08	0.16	0.14	0.55
	263	0.08	0.13	0.14	0.23	0.58
	265	2.06	2.1	2.01	1.69	7.86
TOTAL		5.37	6.02	4.95	4.41	20.75

STATE: WISCONSIN

ZIP CODE		QUARTER 1	QUARTER 2	QUARTER 3	QUARTER 4	TOTAL GRAMS
	530	0.25	0.16	0.32	0.35	1.08
	531	0.11	0.14	0.13	0.06	0.44
	532	2.68	4.16	1.81	3.02	11.67
	534	0.22	0.19	0.17	0.11	0.69
	535	0.39	0.39	0.28	0.17	1.23
	537	1.28	1.24	1.35	1.75	5.62

	539	0.02	0	0.01	0	0.03
	540	0	0.01	0.01	0	0.02
	543	0.09	0.16	0.14	0.42	0.81
	544	0.45	0.7	0.49	0.66	2.3
	545	0	0	0.02	0.02	0.04
	546	0.12	0.05	0.11	0.46	0.74
	547	0.01	0.03	0	0.02	0.06
	548	0	0	0.08	0.04	0.12
	549	0.43	0.33	0.4	0.72	1.88
TOTAL		6.05	7.56	5.32	7.8	26.73

STATE: WYOMING

ZIP CODE		QUARTER 1	QUARTER 2	QUARTER 3	QUARTER 4	TOTAL GRAMS
	820	0.03	0.16	0.07	0.11	0.37
	822	0	0	0.01	0	0.01
	823	0	0.02	0.02	0	0.04
	824	0.01	0.03	0.03	0.02	0.09
	826	0.24	0.05	0.06	0.1	0.45
	827	0.04	0.02	0.06	0.05	0.17
	829	0	0	0.02	0	0.02
	830	0.08	0.08	0.1	0.12	0.38
	831	0.1	0.03	0.06	0.06	0.25
TOTAL		0.5	0.39	0.43	0.46	1.78

DRUG CODE: 9740 DRUG NAME: SUFENTANIL BASE

STATE: ALABAMA

ZIP CODE		QUARTER 1	QUARTER 2	QUARTER 3	QUARTER 4	TOTAL GRAMS
	352	0.04	0.03	0.03	0.04	0.14
	354	0	0	0	0	0
	356	0.01	0.02	0.01	0.03	0.07
	358	0.71	1.37	1.4	1.64	5.12
	359	0.02	0.02	0.01	0.02	0.07
	360	0.01	0.01	0	0	0.02
	361	0.02	0.02	0.05	0.02	0.11

	363	0.06	0.09	0.09	0.08	0.32
	364	0	0	0	0	0
	365	0.02	0.05	0.01	0.01	0.09
	366	0.14	0.16	0.14	0.16	0.6
	367	0	0	0	0	0
TOTAL		1.03	1.77	1.74	2	6.54

STATE: ALASKA

ZIP CODE		QUARTER 1	QUARTER 2	QUARTER 3	QUARTER 4	TOTAL GRAMS
	995	0	0.01	0.01	0.01	0.03
	996	0	0	0	0	0
	997	0	0	0	0	0
	998	0	0	0	0	0
TOTAL		0	0.01	0.01	0.01	0.03

STATE: ARIZONA

ZIP CODE		QUARTER 1	QUARTER 2	QUARTER 3	QUARTER 4	TOTAL GRAMS
	850	0.04	0.04	0.03	0.07	0.18
	852	0	0.01	0.02	0.02	0.05
	853	0	0	0.01	0.01	0.02
	857	0.02	0.02	0.01	0.03	0.08
	859	0	0	0	0	0
	860	0	0	0	0	0
	863	0	0	0	0	0
TOTAL		0.06	0.07	0.07	0.13	0.33

STATE: ARKANSAS

ZIP CODE		QUARTER 1	QUARTER 2	QUARTER 3	QUARTER 4	TOTAL GRAMS
	716	0	0	0	0	0
	717	0.01	0.01	0.01	0.01	0.04
	720	0	0	0	0	0
	722	0.06	0.06	0.09	0.06	0.27
	723	0	0	0	0	0

	724	0.03	0.02	0.02	0.03	0.1
	725	0	0	0	0	0
	726	0	0	0	0	0
	727	0.04	0.01	0.03	0.04	0.12
	729	0.01	0	0.01	0.01	0.03
TOTAL		0.15	0.1	0.16	0.15	0.56

STATE: CALIFORNIA

ZIP CODE	QUARTER 1	QUARTER 2	QUARTER 3	QUARTER 4	TOTAL GRAMS
900	0.11	0.13	0.21	0.17	0.62
902	0	0.02	0.05	0.01	0.08
904	0.01	0	0	0.01	0.02
905	0.01	0.01	0	0.01	0.03
906	0	0.01	0	0	0.01
907	0.01	0.01	0.01	0	0.03
908	7.35	10.69	9.36	7.36	34.76
910	0	0.02	0	0.01	0.03
911	0	0	0	0	0
913	0.01	0.01	0.01	0.01	0.04
917	0	0.03	0.02	0.03	0.08
919	0	0	0	0	0
920	0.01	0.02	0.01	0.04	0.08
921	0.06	0.01	0.06	0.02	0.15
922	0.01	0.01	0.02	0.01	0.05
923	0.02	0.04	0.01	0.04	0.11
925	1.47	1.2	0.85	1.46	4.98
926	0.01	0.01	0.03	0.03	0.08
927	0	0	0	0.01	0.01
928	0.02	0.01	0.02	0.01	0.06
930	0	0	0.01	0.03	0.04
931	0.01	0.01	0	0.01	0.03
932	0	0.01	0	0.01	0.02
933	0	0.07	0	0	0.07
934	0.03	0.01	0.01	0.02	0.07
935	0	0	0.01	0.01	0.02
936	0	0	0	0	0

	937	0.01	0.01	0.01	0.03	0.06
	939	0.01	0	0	0	0.01
	940	0	0	0.01	0	0.01
	941	0.1	0.12	0.11	0.14	0.47
	943	0.11	0.12	0.14	0.14	0.51
	945	0.02	0.02	0.04	0.03	0.11
	946	0	0	0.01	0.14	0.15
	948	0	0	0	0	0
	949	0	0	0	0	0
	950	0.01	0	0.01	0.02	0.04
	951	0.12	0.03	0.03	0.02	0.2
	952	0.01	0.01	0.01	0.01	0.04
	953	0.01	0.01	0.03	0.02	0.07
	954	0	0	0	0	0
	955	0.03	0.04	0.03	0.03	0.13
	956	0.01	0.01	0.01	0.07	0.1
	958	0.07	0.06	0.09	0.06	0.28
	959	0	0	0	0	0
	960	0.02	0.01	0.01	0.01	0.05
	961	0	0	0	0	0
TOTAL		9.67	12.77	11.23	10.03	43.7

STATE:	COLORADO				
ZIP CODE		QUARTER 1	QUARTER 2	QUARTER 3	QUARTER 4	TOTAL GRAMS

ZIP CODE		QUARTER 1	QUARTER 2	QUARTER 3	QUARTER 4	TOTAL GRAMS
	800	0.06	0.06	0.07	0.06	0.25
	801	0	0.01	0	0.01	0.02
	802	0.02	0.02	0.06	0.11	0.21
	803	0	0	0	0.01	0.01
	805	0	0.01	0.01	0	0.02
	806	0.01	0.01	0.01	0.01	0.04
	807	0	0	0	0	0
	808	0	0	0	0	0
	809	0.07	0.04	0.02	0.03	0.16
	810	0	0	0	0.02	0.02
	811	0	0	0	0	0
	812	0	0	0	0	0

	813	0	0.01	0	0	0.01
	814	0	0	0	0	0
	815	0.02	0.01	0.02	0.02	0.07
	816	0	0	0	0	0
TOTAL		0.18	0.17	0.19	0.27	0.81

STATE:	CONNECTICUT					
ZIP CODE		QUARTER 1	QUARTER 2	QUARTER 3	QUARTER 4	TOTAL GRAMS
	60	0	0	0	0.01	0.01
	61	0.05	0.01	0.07	0.02	0.15
	63	0	0	0	0.01	0.01
	64	0	0	0	0	0
	65	0.08	0.11	0.1	0.1	0.39
	67	0	0	0	0	0
	68	0.27	0.34	0.4	0.8	1.81
TOTAL		0.4	0.46	0.57	0.94	2.37

STATE:	DELAWARE					
ZIP CODE		QUARTER 1	QUARTER 2	QUARTER 3	QUARTER 4	TOTAL GRAMS
	198	0.01	0.01	0.01	0.01	0.04
	199	0	0	0	0	0
TOTAL		0.01	0.01	0.01	0.01	0.04

STATE:	DISTRICT OF COLUMBIA					
ZIP CODE		QUARTER 1	QUARTER 2	QUARTER 3	QUARTER 4	TOTAL GRAMS
	200	0.01	0	0.01	0	0.02
TOTAL		0.01	0	0.01	0	0.02

STATE:	FLORIDA					
ZIP CODE		QUARTER 1	QUARTER 2	QUARTER 3	QUARTER 4	TOTAL GRAMS
	321	0	0.02	0.01	0.01	0.04
	322	0.03	0.05	0.09	0.02	0.19
	323	0.02	0.01	0.02	0.04	0.09

	324	0.05	0.04	0.05	0.04	0.18
	325	0.02	0.01	0.01	0.01	0.05
	326	0.07	0.06	0.04	0.21	0.38
	327	0	0	0	0	0
	328	0.08	0.08	0.08	0.08	0.32
	329	1.35	1.35	1.35	1.35	5.4
	330	0	0	0	0	0
	331	0.08	0.14	0.1	0.11	0.43
	333	0	0	0	0.01	0.01
	334	0.01	0.01	0.01	0.01	0.04
	335	0	0	0	0.01	0.01
	336	2.47	0.15	1.45	0.94	5.01
	337	0.01	0.02	0.01	0.01	0.05
	339	0	0	0	0	0
	341	0	0	0	0	0
	342	0	0	0.03	0.04	0.07
	344	0.03	0.02	0.01	0.04	0.1
	346	0.02	0.02	0.01	0.03	0.08
	347	0.01	0.03	0.05	0.08	0.17
	349	0	0	0	0	0
TOTAL		4.25	2.01	3.32	3.04	12.62

STATE: GEORGIA

ZIP CODE		QUARTER 1	QUARTER 2	QUARTER 3	QUARTER 4	TOTAL GRAMS
	300	0.04	0.03	0.03	0.01	0.11
	301	0.08	0.08	0.02	0.03	0.21
	302	0	0	0.08	0.01	0.09
	303	0.01	0.02	0.19	0.01	0.23
	304	0	0	0	0.04	0.04
	305	0.04	0.04	0.04	0.05	0.17
	306	0.03	0.02	0.02	0.02	0.09
	309	0.04	0.03	0.03	0.05	0.15
	310	0.01	0.02	0.03	0.01	0.07
	312	0.07	0.06	0.07	0.07	0.27
	313	0	0	0	0	0
	314	0	0.02	0	0.3	0.32

	317	0	0	0	0	0
	319	0	0	0	0.01	0.01
TOTAL		0.32	0.32	0.51	0.61	1.76

STATE:	HAWAII				
ZIP CODE	QUARTER 1	QUARTER 2	QUARTER 3	QUARTER 4	TOTAL GRAMS
967	0	0	0	0	0
968	0.01	0	0.01	0.01	0.03
TOTAL	0.01	0	0.01	0.01	0.03

STATE:	IDAHO				
ZIP CODE	QUARTER 1	QUARTER 2	QUARTER 3	QUARTER 4	TOTAL GRAMS
832	0	0	0	0	0
833	0	0	0.01	0	0.01
834	0.01	0.02	0.02	0.01	0.06
836	0	0.01	0.01	0.01	0.03
837	0.02	0.03	0.02	0.02	0.09
838	0	0	0	0	0
TOTAL	0.03	0.06	0.06	0.04	0.19

STATE:	ILLINOIS				
ZIP CODE	QUARTER 1	QUARTER 2	QUARTER 3	QUARTER 4	TOTAL GRAMS
600	0.08	0.04	0.07	0.04	0.23
601	0.03	0.05	0.05	0.06	0.19
602	0.03	0.04	0.01	0.01	0.09
603	0.01	0.01	0	0	0.02
604	0.06	0.07	0.06	0.06	0.25
605	0.02	0.06	0.01	0.01	0.1
606	0.11	0.11	0.11	0.15	0.48
609	0	0	0	0	0
610	0	0	0	0	0
611	0.01	0	0.01	0.01	0.03
612	0.01	0.01	0	0.01	0.03
613	0	0	0	0	0

	614	0	0	0	0	0
	615	0	0	0	0	0
	616	0.01	0.01	0.01	0.02	0.05
	618	0.02	0.02	0.02	0.01	0.07
	619	0	0	0	0	0
	620	0	0	0	0	0
	622	0.14	0.16	0.16	0.12	0.58
	623	0	0.01	0.01	0	0.02
	624	0	0	0	0	0
	625	0	0	0	0.01	0.01
	626	0	0	0	0	0
	627	0.01	0	0.01	0.02	0.04
	628	0	0	0	0	0
	629	0	0.01	0.03	0.01	0.05
TOTAL		0.54	0.6	0.56	0.54	2.24

STATE: INDIANA

ZIP CODE		QUARTER 1	QUARTER 2	QUARTER 3	QUARTER 4	TOTAL GRAMS
	460	0	0	0	0.05	0.05
	461	0	0	0	0	0
	462	0.24	0.3	0.19	0.1	0.83
	463	0.01	0.02	0.01	0.01	0.05
	464	0	0	0	0	0
	465	0.01	0	0	0	0.01
	466	0	0	0.01	0.01	0.02
	467	0	0	0	0	0
	468	0.02	0.01	0	0.02	0.05
	469	0	0	0	0	0
	471	0	0	0	0	0
	472	0	0	0	0.01	0.01
	473	0	0	0	0	0
	474	0	0	0.01	0	0.01
	475	0	0	0	0	0
	476	0	0	0	0	0
	477	0	0.01	0	0.01	0.02

	478	0.02	0.01	0.02	0.01	0.06
	479	0.01	0	0	0	0.01
TOTAL		0.31	0.35	0.24	0.22	1.12

STATE:	IOWA				
ZIP CODE	QUARTER 1	QUARTER 2	QUARTER 3	QUARTER 4	TOTAL GRAMS
500	0	0	0	0	0
501	0	0	0	0	0
502	0	0	0	0	0
503	0.03	0.01	0.01	0.03	0.08
505	0	0	0	0	0
506	0	0	0	0	0
507	0	0	0	0	0
511	0	0	0	0.02	0.02
513	0	0	0	0	0
515	0	0	0	0	0
520	0	0	0	0	0
523	0	0	0	0	0
524	0.01	0.01	0.01	0	0.03
526	0	0	0	0	0
527	0	0	0	0	0
528	0.02	0.01	0.01	0.01	0.05
TOTAL	0.06	0.03	0.03	0.06	0.18

STATE:	KANSAS				
ZIP CODE	QUARTER 1	QUARTER 2	QUARTER 3	QUARTER 4	TOTAL GRAMS
660	0	0.01	0.01	0.04	0.06
661	0	0.01	0.02	0.03	0.06
662	0.09	0.1	0.11	0.05	0.35
664	0	0	0	0.01	0.01
665	0	0	0	0	0
666	0.02	0.04	0.03	0.03	0.12
667	0.01	0.01	0.01	0.01	0.04
668	0	0	0.01	0	0.01

	669	0	0	0	0	0
	670	0.01	0.02	0	0.01	0.04
	671	0.01	0.01	0.01	0.01	0.04
	672	0.08	0.05	0.1	0.07	0.3
	673	0	0	0	0	0
	674	0.01	0.02	0.02	0.03	0.08
	675	0.01	0	0.01	0.01	0.03
	676	0	0	0.01	0	0.01
	678	0	0	0	0	0
	679	0	0	0	0	0
TOTAL		0.24	0.27	0.34	0.3	1.15

STATE: KENTUCKY

ZIP CODE		QUARTER 1	QUARTER 2	QUARTER 3	QUARTER 4	TOTAL GRAMS
	400	0.01	0.01	0.01	0.01	0.04
	402	0.04	0.06	0.04	0.04	0.18
	403	0	0	0	0	0
	404	0	0.01	0	0	0.01
	405	0.08	0.13	0.13	0.13	0.47
	407	0	0	0	0	0
	411	0.01	0.03	0.02	0.01	0.07
	415	0	0	0	0	0
	420	0.14	0.07	0.14	0.14	0.49
	421	0.03	0.03	0.04	0.04	0.14
	422	0	0.04	0.02	0	0.06
	423	0	0.01	0	0	0.01
	424	0	0	0	0.01	0.01
	427	0	0	0	0	0
TOTAL		0.31	0.39	0.4	0.38	1.48

STATE: LOUISIANA

ZIP CODE		QUARTER 1	QUARTER 2	QUARTER 3	QUARTER 4	TOTAL GRAMS
	700	0.01	0.02	0.01	0.05	0.09
	701	0.06	0.06	0.08	0	0.2
	703	0	0	0	0.01	0.01

	704	0.03	0.01	0.01	0.03	0.08
	705	0.03	1.7	0.7	0.69	3.12
	706	0.01	0.01	0.01	0	0.03
	707	0.07	0.07	0.13	0.07	0.34
	708	0.02	0.02	0.02	0.05	0.11
	711	0.09	0.09	0.1	0.09	0.37
	712	0.03	0.05	0.02	0.06	0.16
	713	0	0	0	0.11	0.11
	714	0	0	0	0	0
TOTAL		0.35	2.03	1.08	1.16	4.62

STATE: MAINE

ZIP CODE		QUARTER 1	QUARTER 2	QUARTER 3	QUARTER 4	TOTAL GRAMS
	40	0	0	0	0	0
	41	0	0	0	0	0
	42	0	0	0	0	0
	43	0.01	0	0.01	0.01	0.03
	44	0	0	0	0.01	0.01
	46	0	0	0	0	0
	47	0	0	0	0	0
	48	0	0	0	0	0
TOTAL		0.01	0	0.01	0.02	0.04

STATE: MARYLAND

ZIP CODE		QUARTER 1	QUARTER 2	QUARTER 3	QUARTER 4	TOTAL GRAMS
	207	0	0.01	0	0	0.01
	208	0.02	0.01	0.01	0.01	0.05
	210	0	0.01	0	0.01	0.02
	212	0.01	0.03	0.04	0.02	0.1
	215	0.01	0.01	0.01	0	0.03
	216	0	0	0	0	0
	217	0	0	0	0	0
	218	0	0	0.01	0	0.01
TOTAL		0.04	0.07	0.07	0.04	0.22

STATE: MASSACHUSETTS

ZIP CODE	QUARTER 1	QUARTER 2	QUARTER 3	QUARTER 4	TOTAL GRAMS
10	0.03	0.04	0.03	0.04	0.14
11	0	0	0	0	0
12	0	0	0	0	0
16	0.01	0	0.01	0.01	0.03
18	0	0	0	0.01	0.01
20	0	0	0	0	0
21	0.05	0.06	0.07	0.09	0.27
22	0	0	0	0.03	0.03
25	0	0	0	0	0
27	0	0.02	0.01	0.01	0.04
TOTAL	0.09	0.12	0.12	0.19	0.52

STATE: MICHIGAN

ZIP CODE	QUARTER 1	QUARTER 2	QUARTER 3	QUARTER 4	TOTAL GRAMS
480	0.12	0.12	0.04	0.17	0.45
481	0.06	0.06	0.05	0.12	0.29
482	0.04	0.06	0.05	0.06	0.21
483	0.2	0.01	0.01	0.49	0.71
484	0	0.01	0.02	0.02	0.05
485	0	0	0	0	0
486	0.01	0.01	0	0.01	0.03
487	0	0	0	0.02	0.02
488	0	0.01	0.02	0.02	0.05
489	0.01	0.01	0	0	0.02
490	0	0.01	0	0.01	0.02
492	0.01	0.01	0.01	0.02	0.05
494	0.01	0	0	0.01	0.02
495	0.01	0	0.05	0.01	0.07
496	0.06	0.07	0.06	0.06	0.25
497	0	0	0	0.01	0.01
498	0	0.01	0.01	0	0.02

	499	0	0	0	0	0
TOTAL		0.53	0.39	0.32	1.03	2.27

STATE:	MINNESOTA					
ZIP CODE		QUARTER 1	QUARTER 2	QUARTER 3	QUARTER 4	TOTAL GRAMS
	551	0	0	0	0.09	0.09
	553	0	0	0	0	0
	554	0.01	0.02	0.01	0.01	0.05
	557	0	0	0	0	0
	558	0	0	0	0.05	0.05
	559	0.11	0.09	0.08	0.12	0.4
	561	0	0	0	0	0
	562	0.01	0.01	0.02	0.01	0.05
	563	0.02	0.03	0.03	0.03	0.11
	564	0	0	0	0.01	0.01
	565	0	0	0	0	0
	566	0	0	0	0	0
	567	0	0	0	0	0
TOTAL		0.15	0.15	0.14	0.32	0.76

STATE:	MISSISSIPPI					
ZIP CODE		QUARTER 1	QUARTER 2	QUARTER 3	QUARTER 4	TOTAL GRAMS
	386	0.02	0.02	0.02	0.02	0.08
	390	0	0	0	0	0
	391	14.66	10.36	12.36	14.38	51.76
	392	0.01	0.03	0.07	0.02	0.13
	393	0.03	0.02	0	0.02	0.07
	394	0.01	0	0	0	0.01
	395	0.01	0.01	0.01	0.02	0.05
	396	0	0	0	0	0
	397	0	0	0	0	0
TOTAL		14.74	10.44	12.46	14.46	52.1

STATE: MISSOURI

ZIP CODE	QUARTER 1	QUARTER 2	QUARTER 3	QUARTER 4	TOTAL GRAMS
630	0.01	0.02	0	0	0.03
631	0.09	0.08	0.1	0.08	0.35
633	0	0	0	0	0
635	0	0	0	0	0
636	0.01	0.01	0.01	0.01	0.04
637	0.04	0.05	0.08	0.07	0.24
639	0.03	0.04	0.04	0.03	0.14
640	0	0	0	0	0
641	0.04	0.05	0.03	0.04	0.16
645	0.02	0	0.02	0.01	0.05
647	0	0	0	0	0
648	0.02	0.02	0.02	0.01	0.07
652	0.04	0.04	0.05	0.03	0.16
653	0.01	0	0	0	0.01
654	0	0	0	0	0
657	0	0	0	0	0
658	0	0	0	0	0
TOTAL	0.31	0.31	0.35	0.28	1.25

STATE: MONTANA

ZIP CODE	QUARTER 1	QUARTER 2	QUARTER 3	QUARTER 4	TOTAL GRAMS
590	0	0	0	0	0
591	0.01	0.02	0.01	0.02	0.06
592	0	0	0	0	0
594	0	0	0	0	0
596	0	0	0	0	0
597	0.01	0.01	0.01	0.01	0.04
598	0.01	0.01	0.01	0.02	0.05
599	0.01	0.01	0	0	0.02
TOTAL	0.04	0.05	0.03	0.05	0.17

STATE: NEBRASKA

ZIP CODE	QUARTER 1	QUARTER 2	QUARTER 3	QUARTER 4	TOTAL GRAMS

680	0	0	0	0	0
681	0.06	0.06	0.05	0.11	0.28
683	0	0	0	0	0
684	0	0	0	0	0
685	0.01	0.01	0.02	0.02	0.06
686	0	0	0	0	0
687	0	0.01	0	0	0.01
688	0	0	0	0	0
689	0	0	0	0	0
690	0	0	0	0	0
691	0	0	0	0	0
TOTAL	0.07	0.08	0.07	0.13	0.35

STATE: NEVADA

ZIP CODE	QUARTER 1	QUARTER 2	QUARTER 3	QUARTER 4	TOTAL GRAMS
890	0.01	0.01	0.01	0.02	0.05
891	0.04	0.02	0.09	0.05	0.2
894	0.01	0	0	0.01	0.02
895	0.01	0.02	0.01	0.02	0.06
897	0	0	0	0	0
898	0.01	0.01	0.01	0.02	0.05
TOTAL	0.08	0.06	0.12	0.12	0.38

STATE: NEW HAMPSHIRE

ZIP CODE	QUARTER 1	QUARTER 2	QUARTER 3	QUARTER 4	TOTAL GRAMS
32	0	0	0	0	0
33	0	0	0	0	0
34	0	0	0	0	0
35	0	0	0	0	0
37	0	0	0.01	0	0.01
38	0.01	0.01	0.01	0.01	0.04
TOTAL	0.01	0.01	0.02	0.01	0.05

DATE RANGE: 01/01/2017 TO 12/31/2017 ARCOS 3 - REPORT 1 Run Date: 07/03/2018

RETAIL DRUG DISTRIBUTION BY ZIP CODE WITHIN STATE BY GRAMS WT

STATE: NEW JERSEY

ZIP CODE	QUARTER 1	QUARTER 2	QUARTER 3	QUARTER 4	TOTAL GRAMS
70	0.01	0.01	0.01	0.01	0.04
71	0.01	0.01	0.01	0.01	0.04
73	0	0	0	0	0
74	0.71	0.73	0.04	1.38	2.86
75	0.03	0.04	0.03	0.03	0.13
76	0.04	0.03	0	0	0.07
77	0.03	0.04	0.03	0.03	0.13
79	0	0	0	0	0
80	0	0	0	0	0
82	0.02	0.02	0.01	0.01	0.06
84	0	0	0	0	0
85	0	0	0	0	0
86	0	0	0	0	0
88	0.01	0	0	0	0.01
89	0.1	0.12	0.1	0.08	0.4
TOTAL	0.96	1	0.23	1.55	3.74

STATE: NEW MEXICO

ZIP CODE	QUARTER 1	QUARTER 2	QUARTER 3	QUARTER 4	TOTAL GRAMS
871	0.05	0.03	0.02	0.08	0.18
873	0	0	0	0	0
874	0	0	0	0	0
875	0	0	0	0	0
880	0	0.01	0	0	0.01
881	0	0	0	0	0
882	0	0	0	0	0
TOTAL	0.05	0.04	0.02	0.08	0.19

STATE: NEW YORK

ZIP CODE	QUARTER 1	QUARTER 2	QUARTER 3	QUARTER 4	TOTAL GRAMS
100	1.18	1.19	1.4	0.7	4.47
103	0	0	0	0	0

	104	0.03	0.01	0.01	0.03	0.08
	105	0	0	0	0	0
	110	0.05	0.03	0.05	0.03	0.16
	112	0	0	0.01	0.01	0.02
	113	0.05	0.06	0.06	0.06	0.23
	114	0.13	0	0.13	0	0.26
	117	0.03	0.03	0.01	0.05	0.12
	119	0	0	0	0	0
	122	0.08	0.05	0.1	0.2	0.43
	123	0	0	0	0	0
	124	0	0	0	0	0
	125	0	0	0	0	0
	126	0	0.01	0	0.01	0.02
	127	0	0	0	0	0
	128	0.02	0.02	0.02	0.02	0.08
	129	0	0	0	0	0
	133	0.01	0.01	0.01	0.01	0.04
	135	0	0	0	0	0
	137	0.01	0	0.01	0.01	0.03
	139	0	0	0	0	0
	140	0	0	0	0	0
	142	0.09	0.08	0.1	0.15	0.42
	144	0	0	0	0	0
	146	0.1	0.08	0.11	0.08	0.37
TOTAL		1.78	1.57	2.02	1.36	6.73

STATE:	NORTH CAROLINA				
ZIP CODE	QUARTER 1	QUARTER 2	QUARTER 3	QUARTER 4	TOTAL GRAMS
270	0	0	0.01	0.01	0.02
271	0.07	0.16	0.18	0.07	0.48
272	0.02	0.01	0.02	0.02	0.07
273	0.01	0	0	0	0.01
274	0.01	0.02	0.07	0	0.1
275	0	0	0.01	0.03	0.04
276	0	0.01	0.01	0.03	0.05

	277	0.02	0.05	0.05	0.08	0.2
	278	0.02	0.01	0.01	0.01	0.05
	279	0	0	0	0	0
	280	0	0	0	0.01	0.01
	282	0.01	0.03	0.01	0.01	0.06
	283	0.03	0.03	0.02	0.03	0.11
	284	0.01	0.02	0.04	0.03	0.1
	285	0.01	0.01	0	0.01	0.03
	286	0	0	0.01	0.02	0.03
	287	0.01	0	0	0.02	0.03
	288	0.07	0.06	0.08	0.07	0.28
	289	0	0	0	0	0
TOTAL		0.29	0.41	0.52	0.45	1.67

STATE: NORTH DAKOTA

ZIP CODE		QUARTER 1	QUARTER 2	QUARTER 3	QUARTER 4	TOTAL GRAMS
	581	0	0	0	0.01	0.01
	582	0.02	0.02	0.02	0.02	0.08
	583	0	0	0	0	0
	585	0	0.04	0	0	0.04
	587	0	0	0	0	0
	588	0	0	0	0	0
TOTAL		0.02	0.06	0.02	0.03	0.13

STATE: OHIO

ZIP CODE		QUARTER 1	QUARTER 2	QUARTER 3	QUARTER 4	TOTAL GRAMS
	430	0	0.02	0.01	0.04	0.07
	431	0	0	0	0.01	0.01
	432	0.05	0.04	0.08	0.13	0.3
	433	0	0	0	0	0
	434	0	0	0	0.02	0.02
	435	0.01	0.07	0.01	0.08	0.17
	436	0.07	0.1	0.09	0.1	0.36
	437	0.01	0.03	0.01	0.01	0.06
	440	0	0.07	0.02	0.01	0.1

	441	0.05	0.09	0.07	0.02	0.23
	442	0	0.01	0	0.01	0.02
	443	0	0	0	0	0
	445	0.01	0	0.01	0	0.02
	446	0	0	0	0	0
	448	0.01	0.01	0	0.01	0.03
	449	0.02	0.01	0.01	0.05	0.09
	450	0	0.01	0	0	0.01
	451	0	0	0	0	0
	452	0.03	0.03	0.02	0.1	0.18
	453	0	0	0	0	0
	454	0.02	0.03	0.06	0.07	0.18
	455	0	0	0	0	0
	457	0.01	0.01	0.01	0.01	0.04
	458	0.02	0.01	0.01	0.04	0.08
TOTAL		0.31	0.54	0.41	0.71	1.97

STATE: OKLAHOMA

ZIP CODE		QUARTER 1	QUARTER 2	QUARTER 3	QUARTER 4	TOTAL GRAMS
	730	0.03	0.03	0.03	0.03	0.12
	731	0.11	0.09	0.07	0.07	0.34
	735	0.01	0.01	0.01	0.01	0.04
	737	0	0	0	0	0
	738	0	0	0	0	0
	741	0.04	0.04	0.04	0.05	0.17
	743	0	0	0	0	0
	744	0	0	0	0	0
	748	0	0	0	0	0
TOTAL		0.19	0.17	0.15	0.16	0.67

STATE: OREGON

ZIP CODE		QUARTER 1	QUARTER 2	QUARTER 3	QUARTER 4	TOTAL GRAMS
	970	0.03	0.03	0.03	0.04	0.13
	971	0.04	0	0.01	0.01	0.06
	972	0.2	0.2	0.25	0.29	0.94

	973	0.01	0.01	0.01	0.01	0.04
	974	0	0	0	0	0
	975	0	0	0	0.02	0.02
	977	0	0	0.01	0.01	0.02
	978	0	0.01	0	0	0.01
TOTAL		0.28	0.25	0.31	0.38	1.22

STATE: PENNSYLVANIA

ZIP CODE		QUARTER 1	QUARTER 2	QUARTER 3	QUARTER 4	TOTAL GRAMS
	150	0.01	0.01	0.01	0.02	0.05
	151	0	0	0	0	0
	152	0	0.01	0.01	0.01	0.03
	154	0	0	0	0	0
	158	0	0.01	0	0	0.01
	159	0	0	0.01	0.01	0.02
	163	0	0	0	0	0
	165	0	0.02	0.01	0.01	0.04
	166	0.01	0.01	0	0.01	0.03
	167	0	0	0	0	0
	169	0.01	0	0	0	0.01
	170	0.02	0.01	0	0	0.03
	171	0.01	0	0	0.01	0.02
	174	0	0	0	0.01	0.01
	175	0.01	0.03	0.04	0.03	0.11
	176	0	0	0	0	0
	177	0	0	0	0	0
	178	0.01	0.01	0.01	0.02	0.05
	180	0	0	0	0.01	0.01
	181	0.01	0.01	0.03	0.14	0.19
	185	0.02	0.01	0.02	0.01	0.06
	187	0.02	0.03	0.02	0.04	0.11
	188	0	0	0	0.03	0.03
	190	0.34	1.67	4.02	0.7	6.73
	191	0.1	0.14	0.13	0.2	0.57
	196	0	0.01	0	0	0.01
TOTAL		0.57	1.98	4.31	1.26	8.12

DATE RANGE: 01/01/2017 TO 12/31/2017 ARCOS 3 - REPORT 1 Run Date: 07/03/2018

RETAIL DRUG DISTRIBUTION BY ZIP CODE WITHIN STATE BY GRAMS WT

STATE: PUERTO RICO

ZIP CODE	QUARTER 1	QUARTER 2	QUARTER 3	QUARTER 4	TOTAL GRAMS
6	0	0	0	0.06	0.06
7	0.03	0.01	0.03	0.02	0.09
9	0.18	0.15	0.21	0.08	0.62
TOTAL	0.21	0.16	0.24	0.16	0.77

STATE: RHODE ISLAND

ZIP CODE	QUARTER 1	QUARTER 2	QUARTER 3	QUARTER 4	TOTAL GRAMS
28	0	0.01	0	0	0.01
TOTAL	0	0.01	0	0	0.01

STATE: SOUTH CAROLINA

ZIP CODE	QUARTER 1	QUARTER 2	QUARTER 3	QUARTER 4	TOTAL GRAMS
290	0	0	0	0	0
291	0	0.01	0	0	0.01
292	0.02	0.01	0.04	0.09	0.16
293	0.01	0.01	0.02	0.01	0.05
294	0.04	0.06	0.06	0.07	0.23
295	0.01	0.01	0.01	0.03	0.06
296	0.04	0.04	0.03	0.03	0.14
299	0.67	0.67	0.67	0	2.01
TOTAL	0.79	0.81	0.83	0.23	2.66

STATE: SOUTH DAKOTA

ZIP CODE	QUARTER 1	QUARTER 2	QUARTER 3	QUARTER 4	TOTAL GRAMS
570	0.01	0.01	0.01	0.02	0.05
571	0	0	0	0.05	0.05
575	0	0	0	0	0
577	0.01	0.02	0.02	0.02	0.07
TOTAL	0.02	0.03	0.03	0.09	0.17

STATE: TENNESSEE

ZIP CODE	QUARTER 1	QUARTER 2	QUARTER 3	QUARTER 4	TOTAL GRAMS
370	0.43	0.25	0.33	0.3	1.31
371	0.05	0.05	0.06	0.05	0.21
372	0.04	0.05	0.05	0.1	0.24
373	0	0.01	0.01	0	0.02
376	0.02	0.02	0.01	0.02	0.07
377	0	0.02	0.01	0	0.03
378	0.02	0.01	0.01	0.02	0.06
379	0.02	0.01	0.02	0.03	0.08
380	0	0	0	0	0
381	0.05	0.06	0.09	0.14	0.34
382	0	0	0	0	0
383	0.01	0.03	0.02	0.03	0.09
384	0.02	0.01	0.02	0.02	0.07
385	0.01	0.01	0.01	0.01	0.04
TOTAL	0.67	0.53	0.64	0.72	2.56

STATE: TEXAS

ZIP CODE	QUARTER 1	QUARTER 2	QUARTER 3	QUARTER 4	TOTAL GRAMS
750	0.04	0.1	0.05	0.05	0.24
751	0	0	0	0	0
752	0.05	0.06	0.06	0.08	0.25
753	0.01	0.01	0.02	0.02	0.06
754	0	0.01	0	0.01	0.02
755	0	0	0	0	0
756	0.18	0.19	0.06	0.19	0.62
757	0.02	0.03	0.02	0.04	0.11
759	0.01	0	0	0	0.01
760	0.01	0.03	0.01	0.02	0.07
761	0.13	0.15	0.13	0.18	0.59
762	0.01	0.01	0.01	0.01	0.04
763	0	0.01	0.02	0.02	0.05
765	0.04	0.05	0.05	0.04	0.18

	767	0.01	0	0.01	0	0.02
	769	0	0	0	0	0
	770	1.06	0.28	0.23	1.23	2.8
	773	0	0.03	0	0.03	0.06
	774	0.01	0.01	0.01	0.01	0.04
	775	0	0.01	0.02	0.01	0.04
	777	0	0	0	0	0
	778	0.01	0.01	0.01	0.01	0.04
	779	0	0	0	0	0
	781	0	0	0	0	0
	782	0.06	0.13	0.03	0.06	0.28
	784	0.02	0.03	0.03	0.03	0.11
	785	0.08	0.09	0.1	0.08	0.35
	786	0	0	0	0	0
	787	0.01	0.02	0.01	0.02	0.06
	788	0	0	0	0	0
	789	0	0	0	0	0
	790	0	0	0	0	0
	791	0	0	0.03	0.01	0.04
	794	0.08	0.06	0.07	0.09	0.3
	796	0.03	0.02	0.03	0	0.08
	797	0	0	0	0.01	0.01
	799	0.03	0.03	0.03	0.08	0.17
TOTAL		1.9	1.37	1.04	2.33	6.64

STATE:	UTAH				
ZIP CODE	QUARTER 1	QUARTER 2	QUARTER 3	QUARTER 4	TOTAL GRAMS
840	0.01	0.01	0.02	0.01	0.05
841	0.21	0.17	0.14	0.19	0.71
843	0	0	0	0	0
844	0.01	0.01	0.01	0.03	0.06
846	0	0	0.01	0.01	0.02
847	0.02	0.02	0.02	0.01	0.07
TOTAL	0.25	0.21	0.2	0.25	0.91

RETAIL DRUG DISTRIBUTION BY ZIP CODE WITHIN STATE BY GRAMS WT

STATE: VERMONT

ZIP CODE	QUARTER 1	QUARTER 2	QUARTER 3	QUARTER 4	TOTAL GRAMS
54	0.01	0.01	0	0.01	0.03
57	0	0	0	0	0
TOTAL	0.01	0.01	0	0.01	0.03

STATE: VIRGINIA

ZIP CODE	QUARTER 1	QUARTER 2	QUARTER 3	QUARTER 4	TOTAL GRAMS
201	0	0	0	0	0
220	0.07	0.06	0.05	0.06	0.24
222	0.02	0.02	0.03	0.03	0.1
223	0.01	0.01	0.01	0.01	0.04
224	0.03	0.03	0.03	0.02	0.11
225	0.02	0.02	0.01	0.02	0.07
226	0	0	0	0.01	0.01
228	0	0.01	0	0	0.01
229	0.06	0.05	0.06	0.06	0.23
231	0.02	0.01	0.01	0.02	0.06
232	0.09	0.05	0.06	0.12	0.32
233	0	0	0	0	0
234	0	0	0	0	0
235	0	0	0	0	0
236	0	0	0	0.01	0.01
237	0	0	0.01	0	0.01
240	0.03	0.04	0.04	0.03	0.14
241	0	0	0	0	0
242	0	0.01	0	0	0.01
243	0	0	0	0	0
245	0.05	0.06	0.05	0.06	0.22
TOTAL	0.4	0.37	0.36	0.45	1.58

STATE: WASHINGTON

ZIP CODE	QUARTER 1	QUARTER 2	QUARTER 3	QUARTER 4	TOTAL GRAMS
980	0.02	0.02	0.03	0.02	0.09
981	0.13	0.11	0.2	0.16	0.6

	982	0.02	0	0	0	0.02
	983	0	0	0.01	0	0.01
	984	0.02	0.03	0.03	0.03	0.11
	985	0	0	0	0	0
	986	0	0	0	0	0
	988	0	0	0	0	0
	989	0	0	0	0	0
	991	0	0	0	0	0
	992	0.04	0.04	0.05	0.05	0.18
	993	0	0	0	0	0
TOTAL		0.23	0.2	0.32	0.26	1.01

STATE: WEST VIRGINIA

ZIP CODE		QUARTER 1	QUARTER 2	QUARTER 3	QUARTER 4	TOTAL GRAMS
	253	0.02	0.02	0.03	0.01	0.08
	254	0	0	0	0	0
	256	0	0	0	0	0
	257	0	0	0.03	0.01	0.04
	258	0	0	0	0	0
	261	0	0.01	0.01	0.01	0.03
	265	0.03	0.02	0.02	0.03	0.1
TOTAL		0.05	0.05	0.09	0.06	0.25

STATE: WISCONSIN

ZIP CODE		QUARTER 1	QUARTER 2	QUARTER 3	QUARTER 4	TOTAL GRAMS
	530	0	0.01	0.01	0.01	0.03
	531	0	0	0	0	0
	532	0.72	0.73	0.05	0.74	2.24
	535	0	0	0	0	0
	537	0.02	0.03	0.01	0.06	0.12
	539	0	0	0	0	0
	540	0	0	0	0	0
	541	0	0	0	0	0
	542	0	0	0	0	0
	543	0	0.01	0.01	0.01	0.03

544	0	0	0	0	0
546	0.01	0	0	0.01	0.02
547	0	0	0	0	0
549	0.01	0.01	0.01	0.02	0.05
TOTAL	0.76	0.79	0.09	0.85	2.49

STATE: WYOMING

ZIP CODE	QUARTER 1	QUARTER 2	QUARTER 3	QUARTER 4	TOTAL GRAMS
820	0	0	0.02	0.02	0.04
822	0	0	0	0	0
824	0	0	0	0	0
826	0	0	0	0	0
827	0	0	0	0	0
829	0	0	0	0.01	0.01
831	0	0	0	0	0
TOTAL	0	0	0.02	0.03	0.05

DRUG CODE: 9780 DRUG NAME: TAPENTADOL

STATE: ALABAMA

ZIP CODE	QUARTER 1	QUARTER 2	QUARTER 3	QUARTER 4	TOTAL GRAMS
350	7,809.50	8,395	7,573	7,725.50	31,503
351	4,541.50	4,358.50	4,476	3,995	17,371
352	4,557	4,194.50	3,996	3,764.50	16,512
354	4,533	3,703	3,478.50	3,340	15,054.50
355	3,180.50	3,158.50	2,805.50	2,903.50	12,048
356	4,295.50	4,050	3,746.50	3,941	16,033
357	2,122.50	1,964	1,787	1,654.50	7,528
358	2,662.50	2,480	2,523	2,276	9,941.50
359	2,571	2,574.50	2,664	2,292.50	10,102
360	5,150	4,357	4,398.50	3,933	17,838.50
361	2,585	2,579.50	2,208	2,663	10,035.50
362	1,844.50	1,874	1,778	1,682.50	7,179
363	1,577	1,681	1,191	1,238	5,687
364	633	593.5	454.5	394	2,075
365	4,978.50	4,369.50	4,062.50	3,766	17,176.50

	366	2,880.50	2,805.50	2,918	2,491	11,095
	367	598	684	476	514	2,272
	368	2,288	2,291.50	1,797.50	2,009.50	8,386.50
	369	223	181	141	178	723
TOTAL		59,030.50	56,294.50	52,474.50	50,761.50	218,561

STATE: ALASKA

ZIP CODE		QUARTER 1	QUARTER 2	QUARTER 3	QUARTER 4	TOTAL GRAMS
	995	1,081	1,159.50	1,281	1,312.50	4,834
	996	809.5	809.5	851.5	857	3,327.50
	997	144	122.5	117.5	165.5	549.5
	998	51	61	54	69	235
	999	10	15	15	5	45
TOTAL		2,095.50	2,167.50	2,319	2,409	8,991

STATE: ARIZONA

ZIP CODE		QUARTER 1	QUARTER 2	QUARTER 3	QUARTER 4	TOTAL GRAMS
	850	6,252.50	6,126	4,716.50	7,425	24,520
	851	1,262.50	1,079	971	984.5	4,297
	852	16,105.50	15,137	13,440.50	13,806	58,489
	853	7,090	6,465.50	5,916	5,940.50	25,412
	855	289	436	401	415.5	1,541.50
	856	942.5	660.5	809	877.5	3,289.50
	857	4,136	4,039.50	4,323.50	4,779	17,278
	859	128.5	116.5	53.5	43.5	342
	860	313	192.5	207.5	273.5	986.5
	863	977	1,119	1,040.50	880.5	4,017
	864	689	374.5	359	528	1,950.50
TOTAL		38,185.50	35,746	32,238	35,953.50	142,123

STATE: ARKANSAS

ZIP CODE		QUARTER 1	QUARTER 2	QUARTER 3	QUARTER 4	TOTAL GRAMS
	716	727.5	584.5	645	662.5	2,619.50
	717	1,231.50	1,142.50	944	902.5	4,220.50
	718	334	302.5	298.5	281	1,216

	719	1,110.50	1,146.50	1,193	995.5	4,445.50
	720	4,088	3,769.50	3,434	3,923.50	15,215
	721	2,841.50	2,765	2,320	2,513.50	10,440
	722	1,230	1,403.50	1,511	1,294	5,438.50
	723	450	330.5	285	277.5	1,343
	724	1,618	1,736.50	1,305.50	1,614	6,274
	725	990.5	886	775	754.5	3,406
	726	1,203.50	1,159.50	1,037.50	1,068.50	4,469
	727	4,352	4,204	3,240	3,177.50	14,973.50
	728	581	437	326	418.5	1,762.50
	729	2,535	2,523	1,743.50	1,310	8,111.50
TOTAL		23,293	22,390.50	19,058	19,193	83,934.50

STATE: CALIFORNIA

ZIP CODE		QUARTER 1	QUARTER 2	QUARTER 3	QUARTER 4	TOTAL GRAMS
	900	2,382	2,434	2,524	2,532.50	9,872.50
	902	2,613.50	2,635.50	2,356	2,313	9,918
	903	117.5	82	47.5	79	326
	904	1,429.50	1,831.50	1,507.50	1,770.50	6,539
	905	228.5	270.5	252	353	1,104
	906	1,211	1,234.50	806	880.5	4,132
	907	1,487.50	1,362	1,355	1,219.50	5,424
	908	913	959.5	761	801	3,434.50
	910	647	775.5	617	661.5	2,701
	911	453	549.5	568	598.5	2,169
	912	287	255.5	223.5	266.5	1,032.50
	913	4,384	4,256	4,734.50	4,576.50	17,951
	914	1,241.50	1,265	1,092	1,349	4,947.50
	915	196	158.5	150	213	717.5
	916	153	167.5	95	98	513.5
	917	9,358	9,152	8,375	6,664.50	33,549.50
	918	50	75	72.5	68.5	266
	919	1,236	1,268.50	1,143.50	1,310.50	4,958.50
	920	8,760.50	8,735	8,613.50	9,041	35,150
	921	3,449	3,430	3,090	3,305	13,274
	922	2,856	2,775	2,411	2,274.50	10,316.50

	923	3,837	4,020.50	3,651.50	3,443	14,952
	924	1,222	1,000	926	875	4,023
	925	7,171.50	7,174	5,863	7,242.50	27,451
	926	6,783.50	6,645.50	6,334.95	6,122.90	25,886.85
	927	1,062.50	1,102	989	839	3,992.50
	928	4,010.50	4,328	4,126	4,026	16,490.50
	930	2,651	2,376	2,261	2,670	9,958
	931	498	447.5	376.5	424	1,746
	932	4,046.50	3,330.50	3,675.50	3,138	14,190.50
	933	2,316	2,231	1,929.50	1,780.50	8,257
	934	2,514.50	2,718	2,193	2,388.50	9,814
	935	1,753.50	2,120	1,987	1,895.50	7,756
	936	2,769.50	2,827	2,284	2,255	10,135.50
	937	2,580	2,753.50	2,936	3,147	11,416.50
	939	1,308	1,378.50	1,371.50	1,372	5,430
	940	1,432	1,124.50	1,027	1,283.50	4,867
	941	693.5	504.5	646	572.5	2,416.50
	943	57	84	39	36	216
	944	203.5	170	151	254.5	779
	945	4,368	4,281.50	3,764.50	4,224	16,638
	946	321	358.5	249.5	269.5	1,198.50
	947	223	184	142	134.5	683.5
	948	74	81	94	74.5	323.5
	949	327.5	320.5	406	404.5	1,458.50
	950	1,017	985	915.5	968	3,885.50
	951	415	427.5	570.5	507	1,920
	952	710.5	612	452.5	466.5	2,241.50
	953	2,013.50	2,190	2,036	2,121	8,360.50
	954	920	787	708	805	3,220
	955	113.5	67	21	29	230.5
	956	3,064.50	3,202.50	2,751.50	2,487	11,505.50
	957	1,199	938	972.5	1,024.50	4,134
	958	1,812	1,776.50	1,177	1,156.50	5,922
	959	2,337.50	2,680.50	2,502.50	2,174	9,694.50
	960	1,220	1,497.50	1,262.50	1,207.50	5,187.50
	961	58	35	56	50	199
TOTAL		110,557.50	110,431.50	101,643.45	102,244.40	424,876.85

STATE: COLORADO

ZIP CODE	QUARTER 1	QUARTER 2	QUARTER 3	QUARTER 4	TOTAL GRAMS
800	4,166	3,932.50	3,648	3,642.50	15,389
801	5,037	5,290	5,296.50	5,723	21,346.50
802	3,580.50	4,007.50	3,580.50	3,841.50	15,010
803	631	751.5	718	935	3,035.50
804	688	691.5	582.5	691	2,653
805	3,324.50	3,393	3,273.50	3,223	13,214
806	1,791.50	1,833	1,777	1,499	6,900.50
807	471.5	380.5	313	223	1,388
808	578	585.5	597	633.5	2,394
809	3,853	4,300	3,889	3,800.50	15,842.50
810	1,553.50	1,699.50	2,085.50	2,327	7,665.50
811	76	118.5	74	125.5	394
812	534.5	788	567	646.5	2,536
813	374	323	253	295	1,245
814	81	60	52	103	296
815	526	553	543	578	2,200
816	201	182	223.5	136.5	743
TOTAL	27,467	28,889	27,473	28,423.50	112,252.50

STATE: CONNECTICUT

ZIP CODE	QUARTER 1	QUARTER 2	QUARTER 3	QUARTER 4	TOTAL GRAMS
60	1,872	1,787.50	1,646	1,531.50	6,837
61	358.5	309	316	344.5	1,328
62	649	592.5	497.5	656	2,395
63	1,882.50	1,725.50	1,433.50	1,722	6,763.50
64	3,541	3,338.50	3,172.50	2,964	13,016
65	1,025.50	1,000	874	686	3,585.50
66	659.5	533.5	374	239	1,806
67	997	941.5	934.5	938	3,811
68	1,460.50	1,402.50	1,441	1,316	5,620
69	212	265	249	161	887

TOTAL		12,657.50	11,895.50	10,938	10,558	46,049

STATE:	DELAWARE					
ZIP CODE		QUARTER 1	QUARTER 2	QUARTER 3	QUARTER 4	TOTAL GRAMS
	197	2,605.50	2,595	2,524.50	2,594.50	10,319.50
	198	1,655.50	1,720.50	1,803	1,758.50	6,937.50
	199	3,378	3,116	3,076.50	2,846.50	12,417
TOTAL		7,639	7,431.50	7,404	7,199.50	29,674

STATE:	DISTRICT OF COLUMBIA					
ZIP CODE		QUARTER 1	QUARTER 2	QUARTER 3	QUARTER 4	TOTAL GRAMS
	200	1,209.50	1,086	886	788	3,969.50
	204	0	25	30	72	127
TOTAL		1,209.50	1,111	916	860	4,096.50

STATE:	FLORIDA					
ZIP CODE		QUARTER 1	QUARTER 2	QUARTER 3	QUARTER 4	TOTAL GRAMS
	320	3,779.50	3,972.50	3,438	3,344	14,534
	321	2,795	2,594	2,745	2,560	10,694
	322	6,649.50	6,364.50	5,903	5,787	24,704
	323	3,292.50	3,009	3,065	2,616	11,982.50
	324	2,042	2,018.50	1,638.50	1,688	7,387
	325	5,403	4,308.50	4,169	3,755.50	17,636
	326	1,921	1,947	1,927.50	2,045.50	7,841
	327	5,627.50	5,609.50	5,196	5,130.50	21,563.50
	328	3,020	3,038	2,670.50	2,410	11,138.50
	329	4,048.50	3,896	4,077	3,919	15,940.50
	330	3,525	3,590	3,017.50	2,826	12,958.50
	331	705.5	809.5	549.5	435	2,499.50
	333	2,257	2,182	1,957	1,964	8,360
	334	8,529	7,831	7,094	6,969.50	30,423.50
	335	6,598.50	6,366.50	5,714.50	5,807.50	24,487
	336	5,076	4,424	3,698.50	3,505	16,703.50
	337	4,223	3,591	3,027.50	3,220	14,061.50

	338	3,440.50	3,406	2,965.50	3,307.50	13,119.50
	339	11,730.50	10,822.50	9,221	9,619	41,393
	341	4,764.50	4,260.50	3,752.50	4,181.50	16,959
	342	7,013	6,186.50	5,217.50	5,049.50	23,466.50
	344	2,571.50	2,672.50	2,512	2,486	10,242
	346	4,371.50	4,191.50	3,507	3,764	15,834
	347	3,168	3,267.50	2,768.50	2,713.50	11,917.50
	349	2,872.50	2,664	2,418.50	2,456	10,411
TOTAL		109,424.50	103,022.50	92,250.50	91,559.50	396,257

STATE: GEORGIA

ZIP CODE		QUARTER 1	QUARTER 2	QUARTER 3	QUARTER 4	TOTAL GRAMS
	300	12,561.50	12,113	11,174.50	11,510.50	47,359.50
	301	11,351	11,542.50	9,731	10,329.50	42,954
	302	5,267.50	5,018	4,854	5,016	20,155.50
	303	3,060.50	3,202.50	2,852	3,061	12,176
	304	3,874.50	4,076	3,700.50	3,903.50	15,554.50
	305	4,335	4,161.50	3,270.50	3,396.50	15,163.50
	306	2,207	2,010	1,673.50	1,691.50	7,582
	307	1,413	1,718.50	1,443.50	1,428	6,003
	308	996.5	750.5	652	582.5	2,981.50
	309	880	962.5	1,030.50	1,014.50	3,887.50
	310	3,665	4,018	3,811.50	3,432	14,926.50
	312	1,108.50	1,323.50	1,274.50	1,153.50	4,860
	313	4,835	4,832	4,354.50	3,987	18,008.50
	314	2,576	2,367.50	2,139	2,265.50	9,348
	315	7,692	7,777	6,746.50	6,566.50	28,782
	316	1,471.50	1,462.50	1,643	1,533	6,110
	317	1,765	1,756	1,602.50	1,459	6,582.50
	318	222.5	215	222.5	200.5	860.5
	319	1,355.50	1,176	1,015	1,021.50	4,568
	398	477.5	415.5	443.5	431	1,767.50
TOTAL		71,115	70,898	63,634.50	63,983	269,630.50

STATE: HAWAII

ZIP CODE	QUARTER 1	QUARTER 2	QUARTER 3	QUARTER 4	TOTAL GRAMS
967	1,342	1,322	1,255	1,734	5,653
968	1,523	1,962	1,873.50	1,582.50	6,941
TOTAL	2,865	3,284	3,128.50	3,316.50	12,594

STATE: IDAHO

ZIP CODE	QUARTER 1	QUARTER 2	QUARTER 3	QUARTER 4	TOTAL GRAMS
832	1,172	1,167.50	1,129	1,606.50	5,075
833	345	404	564	462.5	1,775.50
834	946	859.5	705	774	3,284.50
835	274	94	137	92	597
836	1,885	1,569	1,544	1,701.50	6,699.50
837	1,061.50	923.5	949.5	1,042.50	3,977
838	742.5	640	680.5	655	2,718
TOTAL	6,426	5,657.50	5,709	6,334	24,126.50

STATE: ILLINOIS

ZIP CODE	QUARTER 1	QUARTER 2	QUARTER 3	QUARTER 4	TOTAL GRAMS
600	6,719	6,416.50	5,879.50	5,615.50	24,630.50
601	4,228.50	3,674.50	3,104.50	3,057.50	14,065
602	268.5	190.5	239	376	1,074
603	515.5	653.5	388.5	354	1,911.50
604	5,311	4,635.50	4,202	4,293	18,441.50
605	4,186.50	4,054.50	3,943	3,680.50	15,864.50
606	3,274	3,077	2,633	2,785	11,769
607	532	448	415.5	442.5	1,838
608	45.5	35	93.5	136.5	310.5
609	718.5	717	707.5	786.5	2,929.50
610	1,012.50	1,026	914.5	852.5	3,805.50
611	1,665.50	1,290.50	1,295.50	1,301.50	5,553
612	229.5	239	145.5	178.5	792.5
613	301	227.5	167	132	827.5
614	195	134	195	149	673

	615	440.5	390	495	422	1,747.50
	616	339.5	292	324	309.5	1,265
	617	1,089	785.5	1,006	904.5	3,785
	618	1,767	1,859	2,233.50	1,841.50	7,701
	619	456	399.5	373.5	245	1,474
	620	1,743.50	1,641	1,380.50	1,136	5,901
	622	3,626.50	3,506	3,050	3,259	13,441.50
	623	291.5	401	347.5	364	1,404
	624	397	352.5	237.5	342	1,329
	625	698.5	738	937.5	1,093	3,467
	626	216	198	238.5	133	785.5
	627	273	243	237	150	903
	628	444	547	476	464	1,931
	629	465	568.5	508.5	536.5	2,078.50
TOTAL		41,449.50	38,740	36,168.50	35,340.50	151,698.50

STATE:	INDIANA					
ZIP CODE		QUARTER 1	QUARTER 2	QUARTER 3	QUARTER 4	TOTAL GRAMS
	460	1,683.50	1,803.50	1,697	1,366	6,550
	461	2,821.50	2,665	2,303.50	2,775.50	10,565.50
	462	2,754	2,497.50	2,546.50	2,549.50	10,347.50
	463	3,725	4,494	4,499	4,662.50	17,380.50
	464	193	262	368	423	1,246
	465	2,394.50	2,155.50	2,617.50	2,359	9,526.50
	466	661.5	735.5	811.5	636.5	2,845
	467	3,304	3,176	3,096.50	3,038.50	12,615
	468	3,764	3,939	3,965.50	4,081.50	15,750
	469	1,531.50	1,676	1,414	1,536	6,157.50
	470	351	451.5	364.5	334.5	1,501.50
	471	1,667.50	1,418.50	1,088	896	5,070
	472	787	672.5	684	675	2,818.50
	473	1,059.50	929	884.5	680	3,553
	474	670	660.5	727	753	2,810.50
	475	869	728	563	620	2,780
	476	1,065.50	1,031	939	928.5	3,964
	477	1,544	1,410.50	1,260	1,276	5,490.50

	478	6,315	5,986.50	5,323.50	5,881.50	23,506.50
	479	1,261.50	1,207.50	1,395	1,089.50	4,953.50
TOTAL		38,422.50	37,899.50	36,547.50	36,562	149,431.50

STATE:	IOWA					
ZIP CODE		QUARTER 1	QUARTER 2	QUARTER 3	QUARTER 4	TOTAL GRAMS
	500	1,768	1,754	1,308.50	1,614	6,444.50
	501	1,288	1,225.50	943.5	1,115	4,572
	502	1,793.50	1,908.50	1,549.50	1,620.50	6,872
	503	3,509	2,918.50	2,287	2,439	11,153.50
	504	105	86	101	20	312
	505	251	349.5	223	276.5	1,100
	506	467.5	409	417	437.5	1,731
	507	394	346	282.5	325.5	1,348
	508	280.5	254	176	254	964.5
	510	175	240	201	198	814
	511	134	97	203.5	376	810.5
	512	165.5	102.5	73.5	110	451.5
	513	78	55	50	124	307
	514	15	15	26	15	71
	515	646.5	384.5	398	544.5	1,973.50
	516	273	289	221	257.5	1,040.50
	520	430	414	389	384	1,617
	521	21	15	20	62.5	118.5
	522	159	134	122	159	574
	523	484.5	431.5	425	387.5	1,728.50
	524	632	757	599	584	2,572
	525	354	398.5	346	398.5	1,497
	526	65	35	86.5	89.5	276
	527	200.5	111	124.5	125	561
	528	283.5	191	136	181	791.5
TOTAL		13,973	12,921	10,709	12,098	49,701

STATE:	KANSAS					
ZIP CODE		QUARTER 1	QUARTER 2	QUARTER 3	QUARTER 4	TOTAL GRAMS

	660	2,189	2,079	1,955	2,091.50	8,314.50
	661	197	265	326.5	301.5	1,090
	662	2,446	2,188	1,964	2,040.50	8,638.50
	664	277	331	312.5	307.5	1,228
	665	292	256.5	261.5	229	1,039
	666	1,184	951	857.5	976.5	3,969
	667	534.5	470.5	398.5	337.5	1,741
	668	73	107	107	142.5	429.5
	669	24	64.5	21	0	109.5
	670	273	155	171	148	747
	671	56	113	108	120	397
	672	828	689	867.5	608	2,992.50
	673	254	252.5	189.5	209	905
	674	337	321	297.5	384	1,339.50
	675	159.5	185	105	184	633.5
	676	195.5	188.5	206.5	204	794.5
	677	152	102	94	70	418
	678	47	52	46	67	212
	679	10	19	0	15	44
TOTAL		9,528.50	8,789.50	8,288.50	8,435.50	35,042

STATE:	KENTUCKY				
ZIP CODE	QUARTER 1	QUARTER 2	QUARTER 3	QUARTER 4	TOTAL GRAMS
400	625.5	672.5	640	549	2,487
401	378	363.5	251.5	252	1,245
402	2,717	3,000.50	3,121.50	2,624	11,463
403	733.5	702	870	795.5	3,101
404	294	317.5	404	462	1,477.50
405	640	627	750.5	606	2,623.50
406	53	57.5	100	76	286.5
407	158	214	155	114.5	641.5
408	20	15	0	0	35
409	111.5	85	52	40.5	289
410	2,308	2,328.50	2,263.50	2,136.50	9,036.50
411	283.5	295.5	224.5	278.5	1,082
412	106	90	46.5	71	313.5

413	35	15	15	22.5	87.5
415	292	233	171	148	844
416	175	239	254	262	930
417	66.5	65	40.5	63.5	235.5
418	54	46.5	61.5	59	221
420	1,578	1,658	1,665.50	1,832	6,733.50
421	533.5	426.5	443	432	1,835
422	832.5	908	1,132.50	821.5	3,694.50
423	1,577.50	1,368.50	1,327.50	1,417.50	5,691
424	822	752	716.5	689.5	2,980
425	20	20	39	56	135
426	23	34	33	45	135
427	724.5	619.5	552	635	2,531
TOTAL	15,161.50	15,153.50	15,330	14,489	60,134

STATE: LOUISIANA

ZIP CODE	QUARTER 1	QUARTER 2	QUARTER 3	QUARTER 4	TOTAL GRAMS
700	1,605.50	1,811	1,753.50	1,808	6,978
701	152	254	176	152.5	734.5
703	543	547	415	660.5	2,165.50
704	3,250	2,769.50	2,636.50	2,937.50	11,593.50
705	1,811.50	1,677	2,019	1,893.50	7,401
706	332.5	394	409	579.5	1,715
707	2,747	2,664	2,868	3,255.50	11,534.50
708	1,568.50	1,456.50	1,508.50	1,662.50	6,196
710	300.5	298	302.5	338.5	1,239.50
711	1,161.50	1,226	1,003.50	1,093.50	4,484.50
712	411.5	491.5	434.5	287	1,624.50
713	984	931	737	831.5	3,483.50
714	436	504.5	334	366	1,640.50
TOTAL	15,303.50	15,024	14,597	15,866	60,790.50

STATE: MAINE

ZIP CODE	QUARTER 1	QUARTER 2	QUARTER 3	QUARTER 4	TOTAL GRAMS
39	34	20	5	23	82

	40	525.5	492	396	391	1,804.50
	41	10	74	80	33	197
	42	409	360	301	287	1,357
	43	54	66.5	76	69	265.5
	44	55	84	72	47	258
	45	98.5	125	89	100.5	413
	46	0	6	12	7.5	25.5
	47	56.5	37	139	226	458.5
	48	30	44	51	9	134
	49	176	179	121	164.5	640.5
TOTAL		1,448.50	1,487.50	1,342	1,357.50	5,635.50

STATE:		MARYLAND				
ZIP CODE		QUARTER 1	QUARTER 2	QUARTER 3	QUARTER 4	TOTAL GRAMS
	206	2,958.50	2,995	2,773	2,697	11,423.50
	207	3,070.50	2,965	2,681	2,577.50	11,294
	208	3,777.50	3,583.50	3,077	2,915.50	13,353.50
	209	568	435	388	431.5	1,822.50
	210	7,398	7,430.50	6,779	6,361.50	27,969
	211	2,971	2,914	2,696.50	2,750	11,331.50
	212	4,245.50	4,565.50	4,113.50	3,967	16,891.50
	214	996	1,033	934	897.5	3,860.50
	215	598	678	643	598	2,517
	216	1,438.50	1,452	1,383.50	1,613.50	5,887.50
	217	5,132	4,866	4,684	4,465	19,147
	218	681.5	684.5	585	784	2,735
	219	469.5	378.5	435.5	488.5	1,772
TOTAL		34,304.50	33,980.50	31,173	30,546.50	130,004.50

STATE:		MASSACHUSETTS				
ZIP CODE		QUARTER 1	QUARTER 2	QUARTER 3	QUARTER 4	TOTAL GRAMS
	10	1,200.50	1,015	992	1,068.50	4,276
	11	469.5	490.5	439	372	1,771
	12	109	120	92	75	396
	13	129.5	92	161	107	489.5

14	387	446.5	354.5	360	1,548
15	622	571	498	576	2,267
16	169	252	242	278.5	941.5
17	464	528	534.5	565.5	2,092
18	9,896	10,406	10,198.50	9,475	39,975.50
19	931	1,046	978	1,313	4,268
20	372	375	289	462	1,498
21	701	617.5	842	880.5	3,041
22	76	48	39.5	61.5	225
23	925	911	735.5	907.5	3,479
24	396.5	341	317	280	1,334.50
25	172	197	237.5	172	778.5
26	264	253	261.5	137.5	916
27	1,954.50	1,885	2,084	1,856	7,779.50
TOTAL	19,238.50	19,594.50	19,295.50	18,947.50	77,076

STATE: MICHIGAN

ZIP CODE	QUARTER 1	QUARTER 2	QUARTER 3	QUARTER 4	TOTAL GRAMS
480	2,647.50	2,571	2,628.50	2,799	10,646
481	3,505	3,584.50	3,482	3,712.50	14,284
482	331.5	269	310	368.5	1,279
483	2,302	2,279	2,520.50	2,548.50	9,650
484	555.5	537	690	628	2,410.50
485	391.5	384.5	451.5	639	1,866.50
486	762	682	765.5	780.5	2,990
487	307.5	243	359.5	351.5	1,261.50
488	1,752	1,741.50	1,597.50	1,708	6,799
489	579	487	426	388.5	1,880.50
490	2,591	2,331	2,334.50	2,326	9,582.50
491	447	427.5	462.5	428	1,765
492	723	676	646	638	2,683
493	888.5	1,019.50	1,026.50	999.5	3,934
494	1,025.50	977.5	889	729	3,621
495	1,551	1,319.50	1,246.50	1,260.50	5,377.50
496	741	738	780	680.5	2,939.50

	497	442	630.5	700	552	2,324.50
	498	241	253	221	255	970
	499	75	100	95	105	375
TOTAL		21,858.50	21,251	21,632	21,897.50	86,639

STATE: MINNESOTA

ZIP CODE	QUARTER 1	QUARTER 2	QUARTER 3	QUARTER 4	TOTAL GRAMS
550	689	632.5	592	660	2,573.50
551	849	925	853.5	925.5	3,553
553	444.5	480	529.5	571	2,025
554	567.5	579.5	620	876	2,643
556	5	10	0	15	30
557	34	15	43	18	110
558	52	51	47.5	39	189.5
559	199	149.5	130	179.5	658
560	222	171.5	260	168	821.5
561	276.5	210	275	273.5	1,035
562	224.5	138	176	62.5	601
563	207	180.5	178	236	801.5
564	81	119.5	169	250.5	620
565	116	240	215.5	92.5	664
566	89	99.5	75.5	102	366
567	260.5	225.5	240.5	184	910.5
TOTAL	4,316.50	4,227	4,405	4,653	17,601.50

STATE: MISSISSIPPI

ZIP CODE	QUARTER 1	QUARTER 2	QUARTER 3	QUARTER 4	TOTAL GRAMS
386	1,132.50	1,197	1,317	1,284.50	4,931
387	261	264	334	247	1,106
388	1,206	1,296.50	1,085	1,111.50	4,699
389	193	216	201	167	777
390	1,096	1,039.50	1,140	1,036	4,311.50
391	1,048.50	883.5	788	762.5	3,482.50
392	976	1,116.50	1,122.50	907	4,122

393	822	764.5	788	731.5	3,106
394	1,667.50	1,601	1,498	1,563.50	6,330
395	3,792	3,130	2,690	2,184.50	11,796.50
396	345.5	323.5	306.5	230.5	1,206
397	601.5	692.5	564.5	559.5	2,418
TOTAL	13,141.50	12,524.50	11,834.50	10,785	48,285.50

STATE: MISSOURI

ZIP CODE	QUARTER 1	QUARTER 2	QUARTER 3	QUARTER 4	TOTAL GRAMS
630	3,539.50	3,075.50	2,771.50	2,892.50	12,279
631	5,592.50	5,155.50	5,233.50	5,311.50	21,293
633	3,076	2,922.50	3,024.50	2,793	11,816
634	229.5	212.5	265	141.5	848.5
635	191	228.5	316	294	1,029.50
636	337	400	368.5	467.5	1,573
637	1,067	912	1,019	1,089.50	4,087.50
638	428.5	473	465	492.5	1,859
639	324	268.5	125	194	911.5
640	2,304.50	2,185	1,800	1,848.50	8,138
641	1,618	1,845.50	1,328	1,267.50	6,059
644	151	193.5	173.5	121.5	639.5
645	749	810.5	641.5	647.5	2,848.50
646	92	106.5	74	131	403.5
647	504	518.5	400	470	1,892.50
648	988.5	1,021	778.5	737.5	3,525.50
650	288.5	434	497.5	452	1,672
651	660	658	460	533	2,311
652	413.5	422.5	443.5	516	1,795.50
653	487	495	344.5	289.5	1,616
654	257	232.5	287	391	1,167.50
655	251.5	350.5	232.5	344.5	1,179
656	664	589.5	447.5	473	2,174
657	690.5	694	636.5	569	2,590
658	947	1,028	953.5	929	3,857.50
TOTAL	25,851	25,232.50	23,086	23,396.50	97,566

STATE: MONTANA

ZIP CODE	QUARTER 1	QUARTER 2	QUARTER 3	QUARTER 4	TOTAL GRAMS
590	201.5	158	199.5	202	761
591	471.5	380.5	400	336	1,588
592	12	3	20	0	35
593	104	88	98	44.5	334.5
594	481	272	232	236	1,221
595	179.5	76	77.5	66	399
596	93	98	135	130	456
597	385	359	344	437	1,525
598	179	174	146.5	264.5	764
599	95	60	106	90	351
TOTAL	2,201.50	1,668.50	1,758.50	1,806	7,434.50

STATE: NEBRASKA

ZIP CODE	QUARTER 1	QUARTER 2	QUARTER 3	QUARTER 4	TOTAL GRAMS
680	989.5	853	806.5	997	3,646
681	4,385	3,804	4,105	4,103.50	16,397.50
683	259	201	152	194	806
684	179	223	141	142	685
685	1,517.50	1,519	1,705.50	1,597	6,339
686	424	377	374	362	1,537
687	654.5	425.5	388	408	1,876
688	1,334	1,205	967.5	1,283.50	4,790
689	216	177	184	269.5	846.5
690	18	37	41.5	62	158.5
691	385	467	361.5	341	1,554.50
692	0	0	0	20	20
693	84.5	53.5	5	21	164
TOTAL	10,446	9,342	9,231.50	9,800.50	38,820

STATE: NEVADA

ZIP CODE	QUARTER 1	QUARTER 2	QUARTER 3	QUARTER 4	TOTAL GRAMS
890	3,445.50	3,745	3,968	3,206	14,364.50

	891	7,168.50	6,763.50	5,958.50	7,332.50	27,223
	893	36	18	9	9	72
	894	841	587	654.5	630	2,712.50
	895	713	720.5	630.5	726	2,790
	897	239.5	162	249	317	967.5
	898	150	168.5	190.5	167	676
TOTAL		12,593.50	12,164.50	11,660	12,387.50	48,805.50

STATE:	NEW HAMPSHIRE					
ZIP CODE		QUARTER 1	QUARTER 2	QUARTER 3	QUARTER 4	TOTAL GRAMS
	30	2,109.50	2,181	1,905	1,762	7,957.50
	31	567.5	722.5	676.5	812	2,778.50
	32	1,189	1,064.50	1,080.50	823	4,157
	33	675	626.5	572.5	571.5	2,445.50
	34	137	79.5	81.5	57	355
	35	290	296	354	406	1,346
	37	120	147.5	61	195.5	524
	38	2,485.50	2,618	2,333.50	2,415	9,852
TOTAL		7,573.50	7,735.50	7,064.50	7,042	29,415.50

STATE:	NEW JERSEY					
ZIP CODE		QUARTER 1	QUARTER 2	QUARTER 3	QUARTER 4	TOTAL GRAMS
	70	7,081	6,715	6,146.50	7,015.50	26,958
	71	449.5	513	502	529	1,993.50
	72	432.5	441	416	289	1,578.50
	73	343	413.5	393.5	310	1,460
	74	2,285.50	2,303	2,136.50	2,261.50	8,986.50
	75	258.5	272.5	157	278	966
	76	2,404	2,270	2,201.50	2,259.50	9,135
	77	4,607.50	4,323.50	4,572.50	4,271	17,774.50
	78	1,829.50	2,016.50	1,907	1,918.50	7,671.50
	79	1,213	987.5	1,039	1,197.50	4,437
	80	10,429.50	10,605.50	10,337	8,739	40,111
	81	572.5	726.5	657	665.5	2,621.50

	82	1,964	2,151.50	2,052	2,313.50	8,481
	83	1,866	1,877.50	1,978	1,951.50	7,673
	84	45	44	36.5	18	143.5
	85	1,975.50	2,002.50	1,920	1,689.50	7,587.50
	86	1,942.50	1,825.50	1,656.50	1,596.50	7,021
	87	3,574.50	3,420.50	3,022.50	3,279.50	13,297
	88	4,931.50	4,746.50	4,628.50	5,066.50	19,373
	89	564.5	421	489	482.5	1,957
TOTAL		48,769.50	48,076.50	46,248.50	46,131.50	189,226

STATE: NEW MEXICO

ZIP CODE		QUARTER 1	QUARTER 2	QUARTER 3	QUARTER 4	TOTAL GRAMS
	870	360	251.5	245.5	319.5	1,176.50
	871	1,381.50	1,204	1,442	1,402.50	5,430
	873	0	12	0	6	18
	874	227	186	143	153	709
	875	312	254	238.5	257	1,061.50
	877	73.5	64	34	40	211.5
	878	30	80	10	39	159
	879	0	0	24	16	40
	880	632	540	546	525	2,243
	881	59	46	52	72	229
	882	372	370	276	226	1,244
	883	80	86	41	17	224
	884	9	36	24	12	81
TOTAL		3,536	3,129.50	3,076	3,085	12,826.50

STATE: NEW YORK

ZIP CODE		QUARTER 1	QUARTER 2	QUARTER 3	QUARTER 4	TOTAL GRAMS
	100	4,403.50	4,129	3,811	3,728	16,071.50
	101	120.5	78	31.5	43	273
	102	0	10	5	0	15
	103	2,623	2,571	2,715.50	2,472.50	10,382
	104	1,690	1,704	1,677	1,506.50	6,577.50
	105	1,451	1,669	1,416	1,522.50	6,058.50

106	87.5	154.5	181.5	130	553.5
107	451	533	467.5	433	1,884.50
108	289	183	250	160.5	882.5
109	1,871	1,732	1,919	1,779	7,301
110	2,201	1,873.50	1,768	1,656	7,498.50
111	1,284.50	1,105	1,011	1,207.50	4,608
112	4,285.50	4,124.50	4,043.50	4,066	16,519.50
113	3,291	3,082.50	2,834.50	2,562	11,770
114	991	954	930	1,035.50	3,910.50
115	4,159.50	4,258	4,074.50	4,248.50	16,740.50
116	218.5	166	220.5	99	704
117	13,772	13,884.50	13,368.50	13,258	54,283
118	485.5	591.5	757	667.5	2,501.50
119	1,392	1,636.50	1,918	1,586	6,532.50
120	1,057	670.5	843.5	732.5	3,303.50
121	272.5	317	413.5	309	1,312
122	1,069	1,053.50	933	1,155	4,210.50
123	342	373	226	244	1,185
124	588.5	719.5	506	552	2,366
125	1,862	1,873	2,072.50	1,857	7,664.50
126	342	241.5	148	171	902.5
127	511.5	461	501.5	435.5	1,909.50
128	1,364	1,258	1,259.50	1,234.50	5,116
129	826	873.5	740	965	3,404.50
130	5,373.50	5,063	5,086	4,884	20,406.50
131	1,321	1,306.50	1,194	1,349	5,170.50
132	2,131.50	2,251.50	1,930	2,096.50	8,409.50
133	2,111	2,170	1,939	1,891.50	8,111.50
134	2,034	2,071.50	1,768.50	2,010.50	7,884.50
135	1,105	1,173.50	860	833	3,971.50
136	3,567	3,426	3,310.50	3,303.50	13,607
137	639.5	797.5	652	876.5	2,965.50
138	873.5	974	964	996	3,807.50
139	1,066.50	987.5	1,077	1,111	4,242
140	6,302	5,782.50	5,785.50	5,491.50	23,361.50
141	2,907	2,747	2,712	2,260	10,626
142	7,074.50	6,786	6,427	6,052	26,339.50

143	1,471	1,336.50	1,032	1,032.50	4,872
144	1,695	1,766	1,619	1,656.50	6,736.50
145	1,610	1,674.50	1,550	1,677	6,511.50
146	2,578.50	2,528	2,449.50	2,333.50	9,889.50
147	665	621.5	689.5	491.5	2,467.50
148	1,952	1,751	1,777.50	1,732.50	7,213
149	209	202	215.5	224	850.5
TOTAL	99,988	97,696	94,081.50	92,119	383,884.50

STATE: NORTH CAROLINA

ZIP CODE	QUARTER 1	QUARTER 2	QUARTER 3	QUARTER 4	TOTAL GRAMS
270	5,233.50	5,134.50	4,281.50	4,532	19,181.50
271	4,528.50	4,752	4,410	4,712.50	18,403
272	7,529.50	7,364	6,842	7,030.50	28,766
273	4,621	4,362.50	3,884	4,271.50	17,139
274	2,953.50	2,904	2,667	2,669	11,193.50
275	11,443	10,910	10,599	10,444.50	43,396.50
276	3,468	3,877.50	3,854.50	4,273.50	15,473.50
277	2,048.50	1,972.50	2,088	2,025	8,134
278	4,172	4,050	3,644	3,702	15,568
279	1,631	1,778.50	1,570.50	1,498.50	6,478.50
280	7,874.50	7,316	6,743.50	6,558	28,492
281	6,155.50	5,789.50	5,521.50	5,779	23,245.50
282	4,554	4,412.50	4,013	4,025.50	17,005
283	8,298	8,329.50	7,439.50	5,918.50	29,985.50
284	5,925.50	6,336	6,576.50	5,876	24,714
285	8,084.50	7,818	7,643	6,858.50	30,404
286	8,736.50	8,867	7,893	7,938.50	33,435
287	2,478.50	2,674.50	2,630.50	2,888	10,671.50
288	980.5	1,048	1,016	914.5	3,959
289	231	219.5	159.5	272.5	882.5
TOTAL	100,947	99,916	93,476.50	92,188	386,527.50

STATE: NORTH DAKOTA

ZIP CODE	QUARTER 1	QUARTER 2	QUARTER 3	QUARTER 4	TOTAL GRAMS

	580	244	200	156	221.5	821.5
	581	124.5	207	224.5	87.5	643.5
	582	396.5	375.5	266	301.5	1,339.50
	583	20	25	30	0	75
	584	85.5	86.5	115	101.5	388.5
	585	1,169	1,094.50	1,070.50	1,104	4,438
	586	154.5	135.5	179.5	212	681.5
	587	339	363	242	362	1,306
	588	15	24	15	28	82
TOTAL		2,548	2,511	2,298.50	2,418	9,775.50

STATE: OHIO

ZIP CODE		QUARTER 1	QUARTER 2	QUARTER 3	QUARTER 4	TOTAL GRAMS
	430	4,167.50	4,078.50	3,599	3,237	15,082
	431	2,571	2,435	2,488	2,098	9,592
	432	7,283.78	6,236.45	6,015.45	6,307.83	25,843.51
	433	496.5	511	617	595.5	2,220
	434	1,086	1,238	1,158	1,251	4,733
	435	3,316	3,048.50	3,040.50	3,077	12,482
	436	5,741	5,615.50	4,987	5,467.50	21,811
	437	577	472	420.5	345.5	1,815
	438	361	286	246.5	200.5	1,094
	439	1,199	1,274	1,400.50	1,216	5,089.50
	440	6,304	6,008.50	5,029.50	5,216.50	22,558.50
	441	4,576.50	4,399	3,537	3,560	16,072.50
	442	2,680	2,421.50	2,208	2,226.50	9,536
	443	873	646.5	675.5	613	2,808
	444	1,430	1,235.50	1,258.50	1,225.50	5,149.50
	445	986	791.5	770.5	712	3,260
	446	2,789	3,053.50	2,547	2,766.50	11,156
	447	1,301.50	1,297.50	1,268.50	1,284.50	5,152
	448	955	749.5	785.5	821	3,311
	449	292.5	310.5	268	214.5	1,085.50
	450	4,018	4,080	3,590.50	3,438.50	15,127
	451	1,540	1,368	1,477.50	1,133	5,518.50
	452	3,534	3,525	3,053.50	2,876.50	12,989

	453	1,950	1,955	1,770.50	1,566.50	7,242
	454	2,150.50	2,198.50	2,090	1,626.50	8,065.50
	455	426.5	283	341.5	364.5	1,415.50
	456	2,024.50	1,883	1,796	1,604	7,307.50
	457	409	402.5	227	348	1,386.50
	458	1,148	872	898	938.5	3,856.50
TOTAL		66,186.78	62,675.45	57,564.95	56,331.83	242,759.01

STATE: OKLAHOMA

ZIP CODE		QUARTER 1	QUARTER 2	QUARTER 3	QUARTER 4	TOTAL GRAMS
	730	5,304	5,036.50	4,989.50	4,578.50	19,908.50
	731	3,764.50	3,811.50	3,446.50	3,139	14,161.50
	734	1,539	1,373.50	1,022	948.5	4,883
	735	619	745.5	801	901.5	3,067
	736	36.5	68.5	67	175.5	347.5
	737	344.5	413	216.5	272.5	1,246.50
	738	99.5	81.5	55	69	305
	739	6	44	10	23	83
	740	6,300.50	5,871	5,172	5,478	22,821.50
	741	3,189	3,556	3,692.50	3,742.50	14,180
	743	587	636	515.5	514	2,252.50
	744	1,206.50	1,118	990	993.5	4,308
	745	258.5	371.5	275	274.5	1,179.50
	746	183	223	217	308.5	931.5
	747	69.5	87	177	152	485.5
	748	1,112	1,064	996.5	844	4,016.50
	749	191.5	218	197	134	740.5
TOTAL		24,810.50	24,718.50	22,840	22,548.50	94,917.50

STATE: OREGON

ZIP CODE		QUARTER 1	QUARTER 2	QUARTER 3	QUARTER 4	TOTAL GRAMS
	970	1,555	1,416.50	1,665.50	1,693.50	6,330.50
	971	325	340	423.5	242	1,330.50
	972	778.5	671.5	741	853	3,044
	973	1,189	1,062.50	1,193	1,257.50	4,702

	974	1,147	1,504	1,417	1,225	5,293
	975	1,382	1,506.50	1,304.50	1,329	5,522
	976	184	209	157	194	744
	977	471	511	454	524.5	1,960.50
	978	274	154	133.5	141	702.5
	979	28	66	53	39	186
TOTAL		7,333.50	7,441	7,542	7,498.50	29,815

STATE:	PENNSYLVANIA				
ZIP CODE	QUARTER 1	QUARTER 2	QUARTER 3	QUARTER 4	TOTAL GRAMS
150	1,814	1,968	1,990	2,113.50	7,885.50
151	790	770.5	942	916	3,418.50
152	835	640	393	468.5	2,336.50
153	594	570.5	579	675.5	2,419
154	204	203.5	211	239	857.5
155	602	615	510.5	461	2,188.50
156	1,071.50	773	570	525	2,939.50
157	302.5	277	194	225	998.5
158	374	218	184	186.5	962.5
159	938	1,200.50	1,118	901.5	4,158
160	272	327.5	345.5	327.5	1,272.50
161	780	809	653.5	722	2,964.50
162	442.5	432	413.5	399.5	1,687.50
163	640.5	692.5	635.5	574	2,542.50
164	230	242	250	292	1,014
165	641	804.5	848	779	3,072.50
166	1,068	1,004.50	794.5	933.5	3,800.50
167	15	84	107.5	62.5	269
168	908	994.5	770.5	711.5	3,384.50
169	145	105	110	106	466
170	3,615.50	4,087.50	3,046.50	3,184.50	13,934
171	1,042.50	1,025.50	919.5	997	3,984.50
172	925.5	974.5	889.5	936	3,725.50
173	2,764.50	3,272.50	2,925.50	3,035	11,997.50
174	1,350	1,357	1,579	1,678	5,964
175	381.5	360	303.5	349	1,394

176	634	620	472	534.5	2,260.50
177	356.5	211	308	280	1,155.50
178	356	318.5	371.5	189.5	1,235.50
179	398.5	427.5	483	536.5	1,845.50
180	6,051.50	5,891.50	5,422	6,085.50	23,450.50
181	3,189.50	3,226	2,782.50	2,973.50	12,171.50
182	556	812	869	1,613	3,850
183	3,303	3,221.50	3,208.50	2,974.50	12,707.50
184	1,312.50	1,416.50	1,479	1,387.50	5,595.50
185	776	703	723	654.5	2,856.50
186	357	342	344.5	276.5	1,320
187	6,500.50	6,727.50	5,943.50	5,984	25,155.50
188	228.5	161.5	137	122.5	649.5
189	2,101.50	2,080.50	1,824	1,890	7,896
190	8,142.50	8,186	8,302	7,762	32,392.50
191	2,237.50	2,661.50	2,232	2,058	9,189
193	2,125.50	2,239	2,107	2,385.50	8,857
194	3,212	2,881	3,048	2,616	11,757
195	1,544	1,578.50	1,281.50	1,353	5,757
196	1,352.50	1,159.50	1,171.50	1,048.50	4,732
TOTAL	67,481.50	68,673	63,793	64,523.50	264,471

STATE: PUERTO RICO

ZIP CODE	QUARTER 1	QUARTER 2	QUARTER 3	QUARTER 4	TOTAL GRAMS
006	156	124.5	112.5	58.5	451.5
007	133.5	158.5	123.5	113.5	529
009	615	707	593	478.5	2,393.50
TOTAL	904.5	990	829	650.5	3,374

STATE: RHODE ISLAND

ZIP CODE	QUARTER 1	QUARTER 2	QUARTER 3	QUARTER 4	TOTAL GRAMS
028	1,442	1,623	1,481	1,439	5,985
029	625.5	543.5	579	657	2,405
TOTAL	2,067.50	2,166.50	2,060	2,096	8,390

STATE: SOUTH CAROLINA

ZIP CODE	QUARTER 1	QUARTER 2	QUARTER 3	QUARTER 4	TOTAL GRAMS
290	3,348.50	2,850	2,571.50	2,934.50	11,704.50
291	2,520	2,924.50	2,689	2,769	10,902.50
292	3,389.50	3,332.50	3,168.50	3,071.50	12,962
293	3,999.50	3,653.50	3,465.50	3,961.50	15,080
294	5,064	5,414.50	5,151.50	4,808	20,438
295	6,286	6,371	6,189	5,670.50	24,516.50
296	12,128	12,487.50	12,023	12,770	49,408.50
297	2,545.50	2,663.50	2,730	2,288.50	10,227.50
298	2,045	2,105.50	1,960.50	2,263.50	8,374.50
299	2,946	2,753.50	2,503.50	2,193	10,396
TOTAL	44,272	44,556	42,452	42,730	174,010

STATE: SOUTH DAKOTA

ZIP CODE	QUARTER 1	QUARTER 2	QUARTER 3	QUARTER 4	TOTAL GRAMS
570	716.5	620.5	531	702	2,570
571	1,737	1,680	1,367	1,272	6,056
572	300.5	294	256	242.5	1,093
573	344.5	279	327.5	324	1,275
574	598	579	539.5	545	2,261.50
575	135	137.5	115	130	517.5
576	136.5	155	135	125	551.5
577	1,243.50	1,099	1,050	1,143	4,535.50
TOTAL	5,211.50	4,844	4,321	4,483.50	18,860

STATE: TENNESSEE

ZIP CODE	QUARTER 1	QUARTER 2	QUARTER 3	QUARTER 4	TOTAL GRAMS
370	6,622	6,688.50	6,384	6,319.50	26,014
371	5,878.50	5,655.50	5,556	5,212.50	22,302.50
372	1,787.50	1,542	1,661	1,872	6,862.50
373	3,434.50	3,449	3,421	3,928.50	14,233
374	2,098	1,909.50	2,028.50	2,278	8,314
376	2,095.50	2,029	1,852.50	2,115.50	8,092.50

	377	888.5	815.5	742.5	740	3,186.50
	378	1,542.50	1,449.50	1,470.50	1,492	5,954.50
	379	1,327.50	1,233.50	1,153.50	999.5	4,714
	380	1,407	1,139	1,464	1,531	5,541
	381	1,434	1,038	1,270	1,482.50	5,224.50
	382	428.5	351	558.5	753.5	2,091.50
	383	1,658.50	1,312	1,398.50	1,814	6,183
	384	1,150.50	1,082.50	993.5	915.5	4,142
	385	1,130	1,094	1,134	1,001.50	4,359.50
TOTAL		32,883	30,788.50	31,088	32,455.50	127,215

STATE:	TEXAS					
ZIP CODE		QUARTER 1	QUARTER 2	QUARTER 3	QUARTER 4	TOTAL GRAMS
	750	14,377.50	13,407.50	12,285	11,905	51,975
	751	4,062	3,855.50	3,585.50	3,490	14,993
	752	4,757.50	4,634.50	4,361	4,147.50	17,900.50
	754	1,223.50	1,201	936	1,169	4,529.50
	755	131	141	164.5	54	490.5
	756	597.5	784	869.5	697.5	2,948.50
	757	1,038	964.5	1,487	1,112.50	4,602
	758	643	570	509.5	693	2,415.50
	759	299	263.5	152.5	259	974
	760	7,256	7,196.50	6,300	6,623.50	27,376
	761	4,570.50	4,254	3,807	3,791.50	16,423
	762	1,730	2,219	1,964	2,258	8,171
	763	1,023.50	1,137.50	1,050.50	1,121.50	4,333
	764	424.5	515.5	337	323	1,600
	765	811	1,096	1,361.50	959.5	4,228
	766	193	183	111.5	146	633.5
	767	352	470.5	356.5	381	1,560
	768	69	65.5	93	79	306.5
	769	813	657.5	677	707	2,854.50
	770	5,517	5,661	5,163.50	5,496	21,837.50
	773	4,940.50	4,809.50	4,429.50	5,078.50	19,258
	774	4,684.50	4,794.50	4,601	4,727.50	18,807.50

	775	5,849.50	5,893.50	5,622	5,258	22,623
	776	1,006	1,052	785	874.5	3,717.50
	777	614.5	462	333	346.5	1,756
	778	755.5	884	743.5	857	3,240
	779	321.5	319	186.5	213	1,040
	780	1,484	1,584.50	1,387	1,579.50	6,035
	781	2,181	2,341	1,970	2,195.50	8,687.50
	782	11,111	10,877	9,507.50	10,130.50	41,626
	783	785	697	473.5	571	2,526.50
	784	1,992	1,833	1,341	1,603.50	6,769.50
	785	744.5	609.5	486	534	2,374
	786	4,145	4,189	3,512	4,076.50	15,922.50
	787	4,889.50	4,879.50	4,171	5,255.50	19,195.50
	788	262	344	250	176.5	1,032.50
	790	633	473.5	433	419.5	1,959
	791	1,063.50	1,341	1,080	1,399.50	4,884
	792	33	9	0	5	47
	793	134	117	148	90	489
	794	916.5	992	917	1,419	4,244.50
	795	50.5	24	27	28	129.5
	796	107.5	130.5	119	100.5	457.5
	797	310	401	282.5	299	1,292.50
	798	9	6	0	3	18
	799	2,898	2,637.50	2,562.50	2,792.50	10,890.50
TOTAL		102,077.50	101,164	91,158.50	95,703	390,103

STATE:	UTAH				
ZIP CODE	QUARTER 1	QUARTER 2	QUARTER 3	QUARTER 4	TOTAL GRAMS
840	7,117.50	6,217.50	6,253.50	5,415.50	25,004
841	2,503	2,223	1,935	1,852	8,513
843	487.5	590.5	575	557.5	2,210.50
844	1,130.50	834.5	1,075	1,068	4,108
845	787	733	707	732.5	2,959.50
846	1,196	1,139	988.5	1,122.50	4,446
847	2,375.50	1,846.50	1,926	1,501.50	7,649.50
TOTAL	15,597	13,584	13,460	12,249.50	54,890.50

STATE: VERMONT

ZIP CODE	QUARTER 1	QUARTER 2	QUARTER 3	QUARTER 4	TOTAL GRAMS
50	35	25	25	20	105
52	140	129	120	175	564
53	0	0	0	9	9
54	606.5	420	300.5	343.5	1,670.50
56	171	139	197	155	662
57	311	289.5	284	355.5	1,240
58	78	93.5	112	86	369.5
TOTAL	1,341.50	1,096	1,038.50	1,144	4,620

STATE: VIRGIN ISLANDS

ZIP CODE	QUARTER 1	QUARTER 2	QUARTER 3	QUARTER 4	TOTAL GRAMS
8	9	6	3	0	18
TOTAL	9	6	3	0	18

STATE: VIRGINIA

ZIP CODE	QUARTER 1	QUARTER 2	QUARTER 3	QUARTER 4	TOTAL GRAMS
201	5,539	5,244	4,669	4,521	19,973
220	2,477	2,309.50	2,319	1,590	8,695.50
221	2,005.50	2,000	1,757	1,628.50	7,391
222	1,183.50	1,177.50	981.5	1,008	4,350.50
223	1,585.50	1,505.25	1,513.50	1,245	5,849.25
224	2,019	1,793.50	1,489.50	1,503.50	6,805.50
225	701	705.5	663.5	584.5	2,654.50
226	1,585.50	1,456.50	1,491.50	1,280.50	5,814
227	209.5	151	219	140	719.5
228	642.5	530	648.5	668	2,489
229	478	499.5	440	428.5	1,846
230	648	553.5	394	406.5	2,002
231	1,868.50	1,793	1,152.50	1,537	6,351
232	2,126.50	2,008	1,851	1,898.50	7,884
233	2,507.50	2,093	2,089.50	2,089.50	8,779.50

	234	5,754.50	5,702.50	5,108.50	5,564.50	22,130
	235	1,299	1,386	1,090	1,428.50	5,203.50
	236	2,296.50	2,345.50	1,917	1,872	8,431
	237	942	819.5	791	761	3,313.50
	238	1,740.50	1,487.50	1,245	992	5,465
	239	322	339.5	383	325.5	1,370
	240	1,784	1,502.25	1,243.50	1,162.50	5,692.25
	241	1,800	1,473	1,255.50	1,488	6,016.50
	242	736	717	775	793	3,021
	243	1,090.50	1,060.50	992.5	793	3,936.50
	244	468	398.5	396.5	407.5	1,670.50
	245	1,886.50	1,972	1,454	1,280	6,592.50
	246	299	295	274	253	1,121
TOTAL		45,995	43,318.50	38,604.50	37,649.50	165,567.50

STATE: WASHINGTON

ZIP CODE		QUARTER 1	QUARTER 2	QUARTER 3	QUARTER 4	TOTAL GRAMS
	980	3,341.50	3,535	2,659	2,339	11,874.50
	981	1,253.50	1,155.50	1,119.50	971.5	4,500
	982	1,584	1,709.50	1,574	1,176.50	6,044
	983	2,675.50	2,532	2,770	2,933	10,910.50
	984	926.5	1,009	922.5	910	3,768
	985	902	1,020.50	1,031.50	953	3,907
	986	1,574.50	1,588.50	1,690	1,688.50	6,541.50
	988	493.5	539	454.5	511.5	1,998.50
	989	536.5	541	375	467	1,919.50
	990	545	416.5	432.5	415.5	1,809.50
	991	228	233	142	183	786
	992	2,938.50	3,021	2,630.50	2,926	11,516
	993	1,266	1,022.50	1,088.50	1,118.50	4,495.50
	994	56	103	55	66	280
TOTAL		18,321	18,426	16,944.50	16,659	70,350.50

STATE: WEST VIRGINIA

ZIP CODE	QUARTER 1	QUARTER 2	QUARTER 3	QUARTER 4	TOTAL GRAMS

	247	208	203	271.5	224.5	907
	248	3	16	0	5	24
	249	22	6	0	15	43
	250	148.5	88	112	64.5	413
	251	125	157	121	37	440
	252	42	56	55	38	191
	253	354.5	342.5	347.5	280.5	1,325
	254	1,860	1,897.50	1,421	1,314.50	6,493
	255	422.5	583	514	486.5	2,006
	256	71	79	57	83	290
	257	647	774.5	750	784.5	2,956
	258	86	92	98	100.5	376.5
	259	33	48	37	17	135
	260	1,359	1,297	1,258.50	1,215	5,129.50
	261	304.5	304.5	277.5	334.5	1,221
	262	81.5	106	97.5	57	342
	263	109	109.5	93	75	386.5
	264	24	41	34	10	109
	265	220.5	184	229.5	238	872
	266	30	33	34.5	12	109.5
	267	69.5	61.5	114	84	329
	268	62.5	114	135	135	446.5
TOTAL		6,283	6,593	6,057.50	5,611	24,544.50

STATE:	WISCONSIN				
ZIP CODE	QUARTER 1	QUARTER 2	QUARTER 3	QUARTER 4	TOTAL GRAMS
530	2,731	2,884.50	3,090.50	2,613.50	11,319.50
531	2,503.50	2,518	2,405	2,244.50	9,671
532	3,199	3,008.50	2,473.50	2,585	11,266
534	907	853.5	776	859	3,395.50
535	720.5	813	886.5	871.5	3,291.50
537	284	301.5	193.5	207	986
538	134	143	64.5	66	407.5
539	309	244.5	262.5	234	1,050
540	479	652	444	511.5	2,086.50
541	271	483	578.5	334	1,666.50

	542	269	276	288	297.5	1,130.50
	543	448.5	437	469	453.5	1,808
	544	965.5	788	819	797	3,369.50
	545	297	286	249	289	1,121
	546	90	68	67	64	289
	547	197	165	132	230.5	724.5
	548	258.5	286	281.5	186	1,012
	549	1,435	1,205.50	1,085	1,155	4,880.50
TOTAL		15,498.50	15,413	14,565	13,998.50	59,475

STATE: WYOMING

ZIP CODE		QUARTER 1	QUARTER 2	QUARTER 3	QUARTER 4	TOTAL GRAMS
	820	1,147	1,082	916.5	1,048.50	4,194
	822	77	69	56	49	251
	823	25	35	50	30	140
	824	127	70	68	68	333
	825	15	10	0	0	25
	826	210	230	212	217	869
	827	181	454.5	375.5	380	1,391
	828	65	55	65	75	260
	829	83	68	54	74	279
	830	3	0	0	6	9
	831	15	0	0	0	15
TOTAL		1,948	2,073.50	1,797	1,947.50	7,766

DRUG CODE: 9801 DRUG NAME: FENTANYL BASE

STATE: ALABAMA

ZIP CODE		QUARTER 1	QUARTER 2	QUARTER 3	QUARTER 4	TOTAL GRAMS
	350	183.09	179.05	164.93	153.55	680.62
	351	104.62	93.67	87.06	84.21	369.56
	352	151.23	140.34	135.95	136.18	563.7
	354	91.79	84.11	81.66	81	338.56
	355	67.2	67.12	61.21	53.88	249.41
	356	112.54	105.82	97.51	88.92	404.79

	357	39.84	37.84	32.21	32.85	142.74
	358	73.42	78.99	73.7	73.11	299.22
	359	132.22	125.71	113.69	108.61	480.23
	360	94.63	91.15	87.05	79.54	352.37
	361	94.86	93.28	81.86	80.37	350.37
	362	50.44	46.03	43.03	45.52	185.02
	363	91.04	87.83	83.13	82.05	344.05
	364	35.73	37.69	26.45	29.06	128.93
	365	113.02	101.23	88.32	83.77	386.34
	366	83.89	76.17	66.88	70.4	297.34
	367	31.04	28.27	29.98	28.77	118.06
	368	61.98	61.74	60.62	57.56	241.9
	369	7.98	7.26	8.25	6.41	29.9
TOTAL		1,620.56	1,543.30	1,423.49	1,375.76	5,963.11

STATE: ALASKA

ZIP CODE		QUARTER 1	QUARTER 2	QUARTER 3	QUARTER 4	TOTAL GRAMS
	995	102.59	103.2	103.31	95.22	404.32
	996	85.4	88.68	83.89	84.71	342.68
	997	28.67	26.14	26.66	27.55	109.02
	998	12.63	12.26	11.14	13.04	49.07
	999	10.59	9.12	8.05	7.75	35.51
TOTAL		239.88	239.4	233.05	228.27	940.6

STATE: AMERICAN SAMOA

ZIP CODE		QUARTER 1	QUARTER 2	QUARTER 3	QUARTER 4	TOTAL GRAMS
	967	0.24	0.23	0	0	0.47
TOTAL		0.24	0.23	0	0	0.47

STATE: ARIZONA

ZIP CODE		QUARTER 1	QUARTER 2	QUARTER 3	QUARTER 4	TOTAL GRAMS
	850	360.01	323.86	291.63	263.42	1,238.92

	851	63.73	59.21	51.62	57.45	232.01
	852	619.79	584.4	525.34	510.61	2,240.14
	853	320.47	275.23	243.81	253.44	1,092.95
	855	25.74	22.15	19.49	18.93	86.31
	856	71.65	61.72	54.07	58.18	245.62
	857	299.53	275.58	257.61	251.61	1,084.33
	859	18.54	16.08	17.81	14.57	67
	860	27.83	28.31	26.49	24.3	106.93
	863	81.23	75.69	65.58	67.94	290.44
	864	87.08	78.77	71.39	63.3	300.54
	865	2.33	2.41	1.3	2.14	8.18
TOTAL		1,977.93	1,803.41	1,626.14	1,585.89	6,993.37

STATE: ARKANSAS

ZIP CODE		QUARTER 1	QUARTER 2	QUARTER 3	QUARTER 4	TOTAL GRAMS
	716	46.01	46.3	41.12	38.4	171.83
	717	31.77	28.83	28.61	27.45	116.66
	718	25.48	24.33	24.09	21.45	95.35
	719	75.5	71.71	71.53	69.23	287.97
	720	132.52	123.47	106.68	96.34	459.01
	721	148.54	145.26	131.39	121.16	546.35
	722	100.79	95.21	87.49	87.13	370.62
	723	29.81	29.64	24.06	24.56	108.07
	724	60.07	61.92	55.27	52.66	229.92
	725	48.45	52.6	46.09	44.29	191.43
	726	50	51.01	44.84	42.25	188.1
	727	160.88	152.33	139.53	137.85	590.59
	728	40.82	36.97	37.34	32.7	147.83
	729	59.81	56.81	48.85	46.02	211.49
TOTAL		1,010.45	976.39	886.89	841.49	3,715.22

STATE: CALIFORNIA

ZIP CODE		QUARTER 1	QUARTER 2	QUARTER 3	QUARTER 4	TOTAL GRAMS
	900	224.37	247.9	211.91	189.1	873.28

902	91.03	82.54	82.01	77.13	332.71
903	5.88	4.41	5.18	5	20.47
904	59.69	56.83	55.35	66.52	238.39
905	39.52	38.04	35.79	34.5	147.85
906	88.56	89.16	88.28	77.76	343.76
907	71.63	72.59	70.53	67.67	282.42
908	154.55	132.21	164.02	112.48	563.26
910	60.06	60.18	64.12	46.62	230.98
911	25.27	26.6	23.36	23.19	98.42
912	36.69	39.64	38.4	35.18	149.91
913	206.06	206.11	197.35	198.67	808.19
914	84.64	83.18	79.01	76.19	323.02
915	18.55	18.76	14.61	13.67	65.59
916	11.61	11.32	7.07	7.18	37.18
917	297.66	270.2	287.31	274.72	1,129.89
918	6.36	7.45	6.75	5.2	25.76
919	57.26	53.22	49.9	50.56	210.94
920	371.83	361.18	351.73	341.21	1,425.95
921	393.49	413.51	371.79	354.21	1,533
922	217.92	213.6	197.58	197.61	826.71
923	296.42	275.54	250.97	243.34	1,066.27
924	64.79	54.54	50.32	38.46	208.11
925	284.16	272.05	256.97	248.36	1,061.54
926	286.86	273.93	268.41	256.65	1,085.85
927	60.46	53.71	50.75	53.52	218.44
928	242.33	241.13	222.1	210.48	916.04
930	138.57	134.39	124.8	117.29	515.05
931	24.75	25.81	24.94	25.7	101.2
932	116.75	120.8	113.44	107.18	458.17
933	162.66	161.07	137.31	164.9	625.94
934	125.22	132.95	115.05	98.54	471.76
935	105.22	102.05	90.72	86.95	384.94
936	75.51	73.97	70.91	69.5	289.89
937	106.7	104.43	101.31	101.73	414.17
939	36.51	35.93	34.87	34.85	142.16
940	123.67	115.88	111.62	110.56	461.73
941	138.53	130.88	126.35	128.14	523.9

	943	35.7	31.98	29.2	30.94	127.82
	944	19.38	15.59	16.24	15.57	66.78
	945	498.62	464.86	451.83	414.19	1,829.50
	946	49.12	48.82	45.85	44.44	188.23
	947	14.22	14.85	16.1	16.91	62.08
	948	16.3	14.28	14.05	12.43	57.06
	949	64.18	64.95	61.68	56.88	247.69
	950	123.54	122.7	115.87	118.08	480.19
	951	65.33	65.82	61.6	63.03	255.78
	952	128.13	123.93	107.04	107.13	466.23
	953	281	267.87	262.41	253.76	1,065.04
	954	142.83	129.89	116.04	107.07	495.83
	955	54.42	55.69	51.22	47.86	209.19
	956	362.6	336.83	332.77	313.92	1,346.12
	957	58.23	54.55	51.64	47.64	212.06
	958	208.56	198.79	194.04	183.91	785.3
	959	205.1	192.43	173.03	169.1	739.66
	960	136.69	122.26	122.23	112.51	493.69
	961	18.94	19.14	18.9	17.23	74.21
TOTAL		7,394.63	7,142.92	6,794.63	6,483.12	27,815.30

STATE: COLORADO

ZIP CODE	QUARTER 1	QUARTER 2	QUARTER 3	QUARTER 4	TOTAL GRAMS
800	219.67	203.56	194.97	183.67	801.87
801	176.95	153.9	157.14	147.05	635.04
802	214.14	207.77	198.46	192.8	813.17
803	33.56	33.85	29.52	29.92	126.85
804	54.42	51.68	43.78	49.66	199.54
805	158.79	144.97	134.02	126.47	564.25
806	56.71	52.29	49.62	50.98	209.6
807	28.44	27.72	24.42	23.56	104.14
808	32.09	27.69	25.03	24.41	109.22
809	149.79	147.48	144.78	134.73	576.78
810	80.5	78.48	77.77	72	308.75
811	15.97	13.71	13.35	11.1	54.13
812	25.5	23.76	22.72	22.01	93.99

	813	17.05	15.76	16.66	12.62	62.09
	814	18.23	16.12	15.36	12.52	62.23
	815	61.73	53.84	51.4	48.42	215.39
	816	18.7	19.52	17.63	17.69	73.54
TOTAL		1,362.24	1,272.10	1,216.63	1,159.61	5,010.58

STATE: CONNECTICUT

ZIP CODE		QUARTER 1	QUARTER 2	QUARTER 3	QUARTER 4	TOTAL GRAMS
	60	205.2	195.33	181.51	157.68	739.72
	61	49.14	49.73	47.39	48.4	194.66
	62	40.23	36.82	36.95	35.27	149.27
	63	75.45	75.46	72.77	64.9	288.58
	64	252.95	243.68	233.53	232.98	963.14
	65	82.74	79.11	76.54	85.7	324.09
	66	40.59	42.53	36.43	35.27	154.82
	67	91.72	85.49	84.43	75.58	337.22
	68	152.96	164.25	141.34	130.97	589.52
	69	15.21	16.88	13.31	14.19	59.59
TOTAL		1,006.19	989.28	924.2	880.94	3,800.61

STATE: DELAWARE

ZIP CODE		QUARTER 1	QUARTER 2	QUARTER 3	QUARTER 4	TOTAL GRAMS
	197	135.29	130.61	129.89	127.83	523.62
	198	70.56	66.83	62.69	62.36	262.44
	199	152.42	152.77	132.18	137.17	574.54
TOTAL		358.27	350.21	324.76	327.36	1,360.60

STATE: DISTRICT OF COLUMBIA

ZIP CODE		QUARTER 1	QUARTER 2	QUARTER 3	QUARTER 4	TOTAL GRAMS
	200	84.93	83.59	74.69	71.06	314.27
	203	0	0.08	0.04	0.1	0.22
	204	7.48	6.47	5.63	4.68	24.26
	205	0	0.02	0	0	0.02

TOTAL		92.41	90.16	80.36	75.84	338.77

STATE: FLORIDA

ZIP CODE		QUARTER 1	QUARTER 2	QUARTER 3	QUARTER 4	TOTAL GRAMS
	320	237.27	225.69	208.58	207.69	879.23
	321	246.03	218.29	202.7	214.69	881.71
	322	477.67	429.32	411.35	401.18	1,719.52
	323	90.04	90.31	84.78	89.45	354.58
	324	130.74	119.95	114.62	117.25	482.56
	325	160.26	159.85	148.43	134.6	603.14
	326	124.35	120.61	121.94	124.14	491.04
	327	353.14	334.41	308.43	311.79	1,307.77
	328	188.91	174.31	161.86	162.84	687.92
	329	358.79	346.69	310.73	327.09	1,343.30
	330	296.99	286.48	252.92	272.62	1,109.01
	331	226.44	215.64	195.3	205.22	842.6
	333	230.05	210.82	212.65	219.91	873.43
	334	511.29	469.04	449.59	455.44	1,885.36
	335	220.14	198.78	193.99	198.48	811.39
	336	463.13	401.91	387.6	332.09	1,584.73
	337	314.37	291.14	288.08	293.05	1,186.64
	338	172.81	157.77	145.74	159.74	636.06
	339	304.82	265.05	229.04	261.3	1,060.21
	341	98.47	88.85	77.82	85.29	350.43
	342	344.2	313.6	274.22	306.7	1,238.72
	344	169.8	159.31	142.81	152.84	624.76
	346	314.66	296.06	276.57	287.31	1,174.60
	347	151.69	135.67	113.01	131.52	531.89
	349	185.54	174.26	159.99	172.34	692.13
TOTAL		6,371.60	5,883.81	5,472.75	5,624.57	23,352.73

STATE: GEORGIA

ZIP CODE		QUARTER 1	QUARTER 2	QUARTER 3	QUARTER 4	TOTAL GRAMS
	300	564.82	529.47	509.22	516.16	2,119.67
	301	375.86	362.64	341.16	333.97	1,413.63

	302	241.18	234.09	217	210.82	903.09
	303	181.88	171.1	151.32	170.68	674.98
	304	90.98	95.5	87.91	77.43	351.82
	305	228.24	217.26	200.13	197.29	842.92
	306	111.67	107.81	104.99	104.08	428.55
	307	71.69	66.32	61.95	55.17	255.13
	308	60.28	54.27	53.3	52.35	220.2
	309	119.42	108.61	95.95	92.98	416.96
	310	199.92	201.16	186.63	175.43	763.14
	312	77.16	70.42	66.24	65.76	279.58
	313	87.34	79.96	66.84	52.73	286.87
	314	76.1	71.09	70.44	64.45	282.08
	315	141.91	137.17	129.89	117.33	526.3
	316	68.8	69.74	62.69	69.51	270.74
	317	129.92	119.79	120.68	118.75	489.14
	318	8.57	9.01	7.26	7.02	31.86
	319	62.72	56.19	57.27	51.56	227.74
	398	34.56	30.21	28.63	30.11	123.51
TOTAL		2,933.02	2,791.81	2,619.50	2,563.58	10,907.91

STATE: GUAM

ZIP CODE		QUARTER 1	QUARTER 2	QUARTER 3	QUARTER 4	TOTAL GRAMS
TOTAL		12.2	10.02	9.17	9.95	41.34

STATE: HAWAII

ZIP CODE		QUARTER 1	QUARTER 2	QUARTER 3	QUARTER 4	TOTAL GRAMS
	967	120.58	114.18	104.19	99.5	438.45
	968	77.6	79.59	71.01	75.65	303.85
TOTAL		198.18	193.77	175.2	175.15	742.3

STATE: IDAHO

ZIP CODE		QUARTER 1	QUARTER 2	QUARTER 3	QUARTER 4	TOTAL GRAMS
	832	54.32	57.56	56.59	55.36	223.83
	833	60.46	62.31	61.1	55.82	239.69

	834	55.44	62.69	55.01	55.05	228.19
	835	39.37	38.3	39.48	38.05	155.2
	836	169.69	167.1	162.68	154.66	654.13
	837	125.17	121.74	112.07	120.2	479.18
	838	90.1	79.52	78.72	76.34	324.68
TOTAL		594.55	589.22	565.65	555.48	2,304.90
STATE:	ILLINOIS					
ZIP CODE		QUARTER 1	QUARTER 2	QUARTER 3	QUARTER 4	TOTAL GRAMS
	600	431.51	418.01	410.04	394.44	1,654
	601	268.12	254.87	243.67	255.42	1,022.08
	602	13.31	11.22	10.01	11.52	46.06
	603	12.14	11.6	10.53	12.68	46.95
	604	366.07	336.89	328.39	318.02	1,349.37
	605	207.42	197.57	189.15	185.17	779.31
	606	282.69	279.56	258.43	256.44	1,077.12
	607	22.87	22.34	23.32	26.63	95.16
	608	20.19	20.87	17.7	23.82	82.58
	609	47.34	46.46	43.6	44.08	181.48
	610	109.39	106.62	99.03	97.17	412.21
	611	117.88	119.07	114.16	104.03	455.14
	612	36.72	36.82	32.91	31.12	137.57
	613	45.07	41.41	38.49	37.12	162.09
	614	50.23	46.97	46.27	44.18	187.65
	615	66.93	67.72	65.63	65.84	266.12
	616	142.56	136.23	128.83	121.75	529.37
	617	74.61	71.11	70.71	67.34	283.77
	618	73.12	68.77	66.51	65.2	273.6
	619	33.74	34.23	33.5	33.36	134.83
	620	114.4	108.87	103.78	100.76	427.81
	622	116.42	105.74	104.47	92.57	419.2
	623	45.57	46.67	47.07	43.6	182.91
	624	45.01	44.61	43.76	44.51	177.89
	625	93.01	83.02	91.43	82.57	350.03
	626	59.78	58.7	56.63	53.8	228.91
	627	73.89	68.2	66.39	69.44	277.92

	628	54.52	49.99	49.01	48.08	201.6
	629	99.21	96.23	87.5	83.49	366.43
TOTAL		3,123.72	2,990.37	2,880.92	2,814.15	11,809.16

STATE:	INDIANA					
ZIP CODE		QUARTER 1	QUARTER 2	QUARTER 3	QUARTER 4	TOTAL GRAMS
	460	138.04	126.7	118.05	117.89	500.68
	461	151.66	139.09	132.53	130.77	554.05
	462	362.62	344.36	317.92	349.14	1,374.04
	463	193.36	194.14	179.99	175.11	742.6
	464	15.91	14.96	12.77	15.14	58.78
	465	144.27	136.05	126.4	130.52	537.24
	466	49.36	48.32	41.2	41.84	180.72
	467	62.89	60.79	59.31	56.56	239.55
	468	120.63	118.63	113.88	107.97	461.11
	469	106.14	96.13	88.12	82.75	373.14
	470	48.92	47.25	43.63	44.39	184.19
	471	133.7	131.54	114.39	90.54	470.17
	472	57.27	51.55	47.28	48.23	204.33
	473	104.73	98.17	100.97	96	399.87
	474	75.85	72.1	64.25	58.92	271.12
	475	63.23	52.61	51.14	49.32	216.3
	476	32.83	33.07	33.09	21.65	120.64
	477	53.91	52.2	47.94	50.78	204.83
	478	42.78	40.18	36.73	34.35	154.04
	479	82.68	64.23	63.02	66.23	276.16
TOTAL		2,040.78	1,922.07	1,792.61	1,768.10	7,523.56

STATE:	IOWA					
ZIP CODE		QUARTER 1	QUARTER 2	QUARTER 3	QUARTER 4	TOTAL GRAMS
	500	65.07	69.27	66.76	68.94	270.04
	501	81.13	75.41	77.69	77.4	311.63
	502	69.66	70.79	63.56	64.6	268.61

	503	109.25	95.58	94.78	93.42	393.03
	504	43.88	44.45	39.04	39.83	167.2
	505	43.21	40.9	41.52	41.98	167.61
	506	50.72	50.81	45.89	46.06	193.48
	507	20.54	17.54	18.66	18.09	74.83
	508	13.95	12.5	11.75	11.2	49.4
	510	12.55	12.56	12.28	11.43	48.82
	511	24.81	28.39	24.64	23.48	101.32
	512	13.49	11.76	10.33	8.39	43.97
	513	13.07	11.41	12.49	11.33	48.3
	514	26.07	24.11	25.84	24.5	100.52
	515	37.82	36.89	33.99	30.94	139.64
	516	7.3	6.84	6.27	7.47	27.88
	520	39.26	38.83	39.11	37.55	154.75
	521	16.62	16.49	15.33	15	63.44
	522	32.26	32.61	30.88	30.06	125.81
	523	25.71	24.61	24.37	22.89	97.58
	524	29.56	34.52	32.31	30.81	127.2
	525	46.21	42.52	41.47	40.26	170.46
	526	29.02	29.07	26.21	27.1	111.4
	527	23.13	25.36	20.98	22.41	91.88
	528	33.76	33.7	29.75	30.55	127.76
TOTAL		908.05	886.92	845.9	835.69	3,476.56

STATE:	KANSAS					
ZIP CODE		QUARTER 1	QUARTER 2	QUARTER 3	QUARTER 4	TOTAL GRAMS
	660	166.93	158.86	156.72	144.37	626.88
	661	52.53	43	46.27	50.54	192.34
	662	176.47	175.61	158.41	152.26	662.75
	664	27.96	28.35	28.14	25.03	109.48
	665	44.05	44.45	42.16	43.85	174.51
	666	67.02	68.87	64.9	62.24	263.03
	667	65.09	65.8	62.78	143.68	337.35
	668	11.22	12.06	12	10.84	46.12
	669	18.19	18.73	19.85	17.63	74.4
	670	58.92	56.12	57.45	55.02	227.51

671	37.74	39.31	35.12	37.65	149.82
672	148.47	145.19	139.2	131.05	563.91
673	31.31	28.92	26.88	26.47	113.58
674	66.86	72.03	70.85	68.15	277.89
675	38.02	36.2	33.96	29.93	138.11
676	36.28	37.2	36.49	36.05	146.02
677	20.88	22.01	18.77	20.09	81.75
678	51.21	51.56	49.8	49.01	201.58
679	20.88	17.21	18.84	17.99	74.92
TOTAL	1,140.03	1,121.48	1,078.59	1,121.85	4,461.95

STATE: KENTUCKY

ZIP CODE	QUARTER 1	QUARTER 2	QUARTER 3	QUARTER 4	TOTAL GRAMS
400	54	48.32	46.65	45.78	194.75
401	22.14	22.59	18.66	19.42	82.81
402	230.43	212.89	195.78	194.17	833.27
403	79.4	83.83	74.43	74.34	312
404	46.75	45.83	43.44	39.89	175.91
405	104.54	96.79	89.5	90.64	381.47
406	7.49	7.62	7.78	8.05	30.94
407	37.84	33.23	34.34	33.41	138.82
408	7.42	7.39	6.37	5.72	26.9
409	16.59	14.29	13.55	11.76	56.19
410	148.4	135.32	128.42	121.27	533.41
411	33	34.03	30.66	27.52	125.21
412	14.99	13.52	11.05	12.07	51.63
413	13.08	10.93	10.43	9.01	43.45
414	3.06	2.87	2.73	2.56	11.22
415	22.64	21.05	17.16	16.88	77.73
416	13.59	13.5	13.14	11.71	51.94
417	26.58	28.54	31.63	31.19	117.94
418	3.4	3.22	2.59	3.27	12.48
420	56.82	55.33	51.8	52.9	216.85
421	71.36	69.04	66.63	63.73	270.76
422	28.32	27.56	28.57	24.37	108.82

	423	40.17	41.68	36.97	37.43	156.25
	424	55.55	50.22	49.02	45.64	200.43
	425	24.78	26.75	20.47	18.38	90.38
	426	17.5	15.67	15.42	16.01	64.6
	427	52.98	52.92	51.96	43.05	200.91
TOTAL		1,232.82	1,174.93	1,099.15	1,060.17	4,567.07

STATE: LOUISIANA

ZIP CODE		QUARTER 1	QUARTER 2	QUARTER 3	QUARTER 4	TOTAL GRAMS
	700	109.48	105.46	96.79	90.14	401.87
	701	77.43	71.64	67.28	65.66	282.01
	703	87.32	76.91	74.53	66.5	305.26
	704	131.98	116.79	104.33	100.58	453.68
	705	225.83	229.68	226.14	196.43	878.08
	706	45.96	42.08	48.82	44.25	181.11
	707	141.59	119.55	114.61	114.61	490.36
	708	103.09	98.05	89.59	92.03	382.76
	710	32.1	32.93	31.25	30.97	127.25
	711	92.15	88.32	80.39	77.86	338.72
	712	104.66	99.96	96.2	91.01	391.83
	713	105.37	97.24	88.1	79.33	370.04
	714	44.4	37.87	35.8	34.56	152.63
TOTAL		1,301.36	1,216.48	1,153.83	1,083.93	4,755.60

STATE: MAINE

ZIP CODE		QUARTER 1	QUARTER 2	QUARTER 3	QUARTER 4	TOTAL GRAMS
	39	5.46	3.84	3.47	2.85	15.62
	40	97.03	79.55	76.03	77.85	330.46
	41	26.78	31.69	32.34	31.04	121.85
	42	67.6	38.48	34.33	33.9	174.31
	43	46.79	47.9	39.92	41.37	175.98
	44	46.51	42.62	38.9	35.93	163.96
	45	6.13	5.04	5.31	5.56	22.04
	46	14.76	12.48	11.25	12.95	51.44

	47	24.25	22.78	21	19.17	87.2
	48	8.99	8.31	7.09	7.59	31.98
	49	43.97	38.02	34.24	31.74	147.97
TOTAL		388.27	330.71	303.88	299.95	1,322.81

STATE: MARYLAND

ZIP CODE	QUARTER 1	QUARTER 2	QUARTER 3	QUARTER 4	TOTAL GRAMS
206	94.46	86.64	80.06	75.64	336.8
207	133.08	121.36	128.15	101.07	483.66
208	175.25	149.78	152.35	134.74	612.12
209	38.7	39.33	30.68	35.15	143.86
210	222.22	209.85	194.83	180.67	807.57
211	125.24	109.24	98.51	94.79	427.78
212	342.69	313.52	299.63	277.65	1,233.49
214	30.93	26.49	27.32	24.8	109.54
215	57.13	54.06	46.38	37.16	194.73
216	45.06	43.16	39.38	37.17	164.77
217	192.33	180.71	164.63	163.13	700.8
218	56.69	48.44	48.72	43.88	197.73
219	39.02	34.25	34.36	31.93	139.56
TOTAL	1,552.80	1,416.83	1,345	1,237.78	5,552.41

STATE: MASSACHUSETTS

ZIP CODE	QUARTER 1	QUARTER 2	QUARTER 3	QUARTER 4	TOTAL GRAMS
10	144.74	139.05	138.74	132.93	555.46
11	47.36	47.4	42.91	39.12	176.79
12	29.63	26.66	27.26	24.69	108.24
13	14.48	15.01	17.13	15.14	61.76
14	38.46	35.84	34.12	32.01	140.43
15	63.48	63.93	57.27	59.49	244.17
16	51.48	47.15	41.83	40.73	181.19
17	71.26	69.72	61.87	59.64	262.49
18	212.18	208.92	195.04	175.29	791.43
19	86.34	82.18	79.13	85.69	333.34
20	58.74	53.02	48.94	50.54	211.24

	21	181.34	172.93	166.13	174.99	695.39
	22	27.6	26.87	19.91	22.96	97.34
	23	108.33	103.44	92.34	91.17	395.28
	24	45.21	41.2	35.99	37.2	159.6
	25	25.75	24.4	24.43	21.34	95.92
	26	24.81	22.02	24.66	23.63	95.12
	27	158.66	157.6	158.71	148.88	623.85
TOTAL		1,389.85	1,337.34	1,266.41	1,235.44	5,229.04

STATE: MICHIGAN

ZIP CODE		QUARTER 1	QUARTER 2	QUARTER 3	QUARTER 4	TOTAL GRAMS
	480	386.97	363.73	338.98	318.51	1,408.19
	481	443.02	433.49	407.1	396.45	1,680.06
	482	97.97	97.42	92.93	82.83	371.15
	483	331.26	323.66	296.55	278.47	1,229.94
	484	133.23	127.94	121.55	113.77	496.49
	485	69.62	67.06	61.85	67.4	265.93
	486	212.9	194.43	195.1	188.1	790.53
	487	100.52	96.07	85.93	83.17	365.69
	488	175.85	174.56	157.76	149.18	657.35
	489	61.73	60.54	57.46	54.69	234.42
	490	348.21	333.4	316.83	299.96	1,298.40
	491	33.77	34.11	33.04	33.66	134.58
	492	110.89	112.27	100.35	95.2	418.71
	493	68.07	66.64	66.63	58.97	260.31
	494	184.55	175.87	173.56	172.39	706.37
	495	182.83	181.21	176.11	166.09	706.24
	496	145.1	138.8	136.1	130.54	550.54
	497	102.53	104.49	95.46	92.32	394.8
	498	79.68	75.11	79.65	74.58	309.02
	499	26.79	24.83	23.94	21.7	97.26
TOTAL		3,295.49	3,185.63	3,016.88	2,877.98	12,375.98

STATE: MINNESOTA

ZIP CODE		QUARTER 1	QUARTER 2	QUARTER 3	QUARTER 4	TOTAL GRAMS

	550	108.68	103.7	100.47	93.99	406.84
	551	150.84	149.08	144.17	140.68	584.77
	553	137	126.67	118.33	120.16	502.16
	554	225.96	252.74	216.13	212.78	907.61
	556	2.51	2.16	2.48	2.58	9.73
	557	39.89	37.56	36.04	33.27	146.76
	558	29.56	30.23	30.5	24.66	114.95
	559	65.36	62.94	62.12	64.59	255.01
	560	46.07	38.49	41.81	38.18	164.55
	561	20.97	19.55	17.41	19.26	77.19
	562	45.61	41.92	40.04	36.35	163.92
	563	79.63	76.01	72.7	66.72	295.06
	564	39.1	38.32	34.17	34.71	146.3
	565	41.07	41.31	34.25	32.36	148.99
	566	18.74	18.94	18.21	15.74	71.63
	567	29.42	29.58	32.03	29.16	120.19
TOTAL		1,080.41	1,069.20	1,000.86	965.19	4,115.66

STATE: MISSISSIPPI

ZIP CODE		QUARTER 1	QUARTER 2	QUARTER 3	QUARTER 4	TOTAL GRAMS
	386	136.15	127.89	119.44	110.3	493.78
	387	26.71	24.59	21.82	22.14	95.26
	388	106.48	107.46	103.25	103.17	420.36
	389	30.35	30.84	26.11	25.76	113.06
	390	80.26	77.34	75.7	65.42	298.72
	391	275.01	362.24	331.9	316.27	1,285.42
	392	107.11	96.51	98.79	92.62	395.03
	393	105.82	102.26	94.97	86.06	389.11
	394	166.77	165.19	154.39	140.64	626.99
	395	122.41	116.15	101.27	95.45	435.28
	396	61.44	67.11	54.37	52.16	235.08
	397	34.57	34.12	32.14	33.7	134.53
TOTAL		1,253.08	1,311.70	1,214.15	1,143.69	4,922.62

STATE: MISSOURI

ZIP CODE	QUARTER 1	QUARTER 2	QUARTER 3	QUARTER 4	TOTAL GRAMS
630	206	203.98	183.46	186.94	780.38
631	373.02	360.81	352.99	830.57	1,917.39
633	125.9	124.08	113.65	109.79	473.42
634	28.88	29.74	28.61	26.93	114.16
635	34.88	34.05	32.24	31.91	133.08
636	52.67	50.58	48.77	52.48	204.5
637	43.36	44.75	39.55	36.19	163.85
638	46.71	43.16	44.66	38.88	173.41
639	22.24	21.55	15.89	16.08	75.76
640	217.31	210.12	209.16	197.2	833.79
641	198.92	173.61	171.33	169.18	713.04
644	32.79	30.48	30.82	30.54	124.63
645	46.89	50.53	44.42	40.36	182.2
646	29.47	28.1	28.14	27.91	113.62
647	67.21	66.86	60.66	55.83	250.56
648	79.9	77.68	72.2	73.67	303.45
650	45.02	47.15	47.2	45.41	184.78
651	29.52	27.25	28.61	24.7	110.08
652	81.02	82.19	81.26	77.5	321.97
653	33.11	35	30.95	29.9	128.96
654	41.78	39.42	37.62	33.69	152.51
655	43.46	46.34	40.04	36.55	166.39
656	88.56	80.97	83.33	76.24	329.1
657	131.53	122.28	119.49	117.35	490.65
658	158.64	168.98	157.76	158.01	643.39
TOTAL	2,258.79	2,199.66	2,102.81	2,523.81	9,085.07

STATE: MONTANA

ZIP CODE	QUARTER 1	QUARTER 2	QUARTER 3	QUARTER 4	TOTAL GRAMS
590	19.94	18.87	19.61	19.72	78.14
591	71.31	68.4	59.45	61.23	260.39
592	14.23	13.27	12.26	12.67	52.43
593	16.64	15.19	13.66	13.02	58.51
594	52.52	45.09	42.41	42.39	182.41
595	11.65	12.26	11.87	10.78	46.56

	596	35.48	33.43	29.88	29.64	128.43
	597	34.74	33.07	30.68	31.29	129.78
	598	58.95	58.65	58.18	54.24	230.02
	599	38.52	38.39	37.93	36.59	151.43
TOTAL		353.98	336.62	315.93	311.57	1,318.10

STATE:	NEBRASKA				
ZIP CODE	QUARTER 1	QUARTER 2	QUARTER 3	QUARTER 4	TOTAL GRAMS
680	68.26	68.08	67.89	64.42	268.65
681	160.68	143.96	133.93	133.02	571.59
683	25.4	28.11	26.24	25.39	105.14
684	17.35	16.93	16.52	15.84	66.64
685	57.08	55.99	54.25	54.36	221.68
686	14.99	13.39	13.64	13.26	55.28
687	39.89	35.5	34.87	33.19	143.45
688	55.37	61.41	58.34	59.02	234.14
689	35.25	28.77	26.4	25.66	116.08
690	16.84	16.98	18.37	17.25	69.44
691	33.51	33.6	31.42	30.09	128.62
692	2.92	3.44	3.08	2.49	11.93
693	34.18	34.01	35.31	32.9	136.4
TOTAL	561.72	540.17	520.26	506.89	2,129.04

STATE:	NEVADA				
ZIP CODE	QUARTER 1	QUARTER 2	QUARTER 3	QUARTER 4	TOTAL GRAMS
890	168.55	152.54	140.95	136.12	598.16
891	331.16	297.6	281.47	277.66	1,187.89
893	1.53	1.56	1.54	2.01	6.64
894	48.69	43.34	42.13	38.52	172.68
895	76.54	75.27	69.07	59.89	280.77
897	18.52	18.13	14.21	15.05	65.91
898	11.34	11.22	10.16	9.14	41.86
TOTAL	656.33	599.66	559.53	538.39	2,353.91

STATE: NEW HAMPSHIRE

ZIP CODE	QUARTER 1	QUARTER 2	QUARTER 3	QUARTER 4	TOTAL GRAMS
30	101.43	100.7	94.56	95.32	392.01
31	48.73	46.12	43.84	43.65	182.34
32	29.52	25.54	27.39	23.93	106.38
33	45.43	44.74	41.53	39.13	170.83
34	9.9	10.13	10.02	10.11	40.16
35	17.81	15.17	15.05	12.09	60.12
36	1.96	1.88	1.31	1.43	6.58
37	30.1	26.45	26.28	23.86	106.69
38	87.19	80.47	80.46	77.89	326.01
TOTAL	372.07	351.2	340.44	327.41	1,391.12

STATE: NEW JERSEY

ZIP CODE	QUARTER 1	QUARTER 2	QUARTER 3	QUARTER 4	TOTAL GRAMS
70	447.33	453.38	389.93	365.68	1,656.32
71	40.98	36.04	34.88	33.53	145.43
72	13.98	14.43	12.93	11.84	53.18
73	18.12	18.11	14.71	13.16	64.1
74	172.49	141.06	135.43	171.06	620.04
75	30.83	30.67	26.8	25.7	114
76	214.03	204	200.92	191.74	810.69
77	189.47	183.26	168.88	166.52	708.13
78	74.62	74.43	63.96	60.15	273.16
79	75.19	61.89	66.69	67.92	271.69
80	438.07	417.65	362.95	336.23	1,554.90
81	36.41	36.98	32.21	26.46	132.06
82	82.45	74.9	70.71	57.27	285.33
83	39.67	35.35	32.61	31.75	139.38
84	11.84	10.82	10.18	9.88	42.72
85	70.49	71.52	63.01	58.48	263.5
86	74.72	73.13	66.39	65.32	279.56
87	141.2	140.5	129.44	126.44	537.58
88	298.52	293.63	263.58	264.72	1,120.45
89	26.01	27.51	25.19	27.82	106.53

TOTAL		2,496.42	2,399.26	2,171.40	2,111.67	9,178.75

STATE: NEW MEXICO

ZIP CODE		QUARTER 1	QUARTER 2	QUARTER 3	QUARTER 4	TOTAL GRAMS
	870	29.17	28.86	24.93	23.54	106.5
	871	220.32	206.23	182.89	170.12	779.56
	873	8.38	7.17	5.68	6.5	27.73
	874	29.88	26.89	26.69	21.6	105.06
	875	55.74	48.96	47.12	46.34	198.16
	877	7.46	8.34	7.68	7.45	30.93
	878	3.09	1.65	1.41	1.61	7.76
	879	3.72	4.34	4.04	4.54	16.64
	880	33.21	28.73	28.07	29.23	119.24
	881	12.31	12.15	11.4	11.62	47.48
	882	59.85	52.02	49.56	48.99	210.42
	883	18.95	17.17	17.93	17.55	71.6
	884	4.08	3.61	2.66	3.61	13.96
TOTAL		486.16	446.12	410.06	392.7	1,735.04

STATE: NEW YORK

ZIP CODE		QUARTER 1	QUARTER 2	QUARTER 3	QUARTER 4	TOTAL GRAMS
	100	436.67	415.32	376.28	367.11	1,595.38
	101	9.7	10.37	9.66	8.94	38.67
	102	0.28	0.73	0.61	0.41	2.03
	103	149.1	152.17	131.65	136.38	569.3
	104	161.72	164.77	147.28	143.07	616.84
	105	122.88	115.28	107.95	103.03	449.14
	106	18.19	20.66	18.55	22.15	79.55
	107	42.99	36.22	28.61	28.05	135.87
	108	13.44	13.96	11.6	10.91	49.91
	109	152.7	128.23	113.42	109.44	503.79
	110	95.99	94.5	84.31	82.3	357.1
	111	23.5	20.91	17.97	19.51	81.89
	112	260.58	258.06	236.34	220.57	975.55
	113	113.65	115.15	100.04	101.08	429.92

	114	46.6	46.2	50.75	62.21	205.76
	115	164.76	158.61	160.45	152.34	636.16
	116	10.59	11.69	10.24	12.02	44.54
	117	512.34	492.43	454.55	437	1,896.32
	118	20.72	26.03	22.12	17.29	86.16
	119	65.96	65.89	55.32	58.69	245.86
	120	95.41	100.67	98.87	95.88	390.83
	121	43.88	43.14	40.43	38.49	165.94
	122	88.06	74.19	69.84	73.37	305.46
	123	31.46	28.15	27.35	26.25	113.21
	124	52.49	52.29	53.89	47.96	206.63
	125	103.63	95.21	90.89	92.33	382.06
	126	30.76	29.51	25.26	26.88	112.41
	127	44.54	37.41	36.84	34.68	153.47
	128	98.66	90.74	89.07	86.48	364.95
	129	57.28	52.19	54.99	48.53	212.99
	130	103.41	96.67	92.13	89.22	381.43
	131	38.23	32.58	30.53	28.58	129.92
	132	122.28	122.76	106.47	109.58	461.09
	133	48.1	47.52	37.9	38.12	171.64
	134	73.79	71.24	59.71	56.2	260.94
	135	30.19	27.12	28.37	25.2	110.88
	136	73.64	69.54	63.84	63.68	270.7
	137	31.12	32.25	29.63	28.18	121.18
	138	90.98	86.18	81.34	77.66	336.16
	139	34.8	34.24	32.15	32.2	133.39
	140	122.06	112.19	106.71	99.11	440.07
	141	68.59	67.14	59.48	56.62	251.83
	142	200.2	184.18	160.29	159.98	704.65
	143	26.22	24.78	20.01	18.69	89.7
	144	80.05	80.79	76.71	75.81	313.36
	145	78.88	74.42	68.87	61.26	283.43
	146	159.23	151.57	147.21	136.21	594.22
	147	66.42	63.75	56.55	51.64	238.36
	148	115.68	109.35	100.31	98.78	424.12
	149	28.77	30	28.72	25.16	112.65
TOTAL		4,661.17	4,468.95	4,112.06	3,995.23	17,237.41

STATE: NORTH CAROLINA

ZIP CODE	QUARTER 1	QUARTER 2	QUARTER 3	QUARTER 4	TOTAL GRAMS
270	86.49	76.91	69.24	63.73	296.37
271	112.55	100.78	96.95	89.6	399.88
272	195.89	181.49	162.56	169.41	709.35
273	116.11	100.51	97.38	94.36	408.36
274	61.79	59.43	55.68	59.5	236.4
275	272.58	270.76	245.93	248.97	1,038.24
276	130.15	122.65	110.24	115.29	478.33
277	96.92	107.5	80.91	82.08	367.41
278	208.97	191.01	180.53	180.48	760.99
279	66.96	63.36	60.49	63.45	254.26
280	237.68	213.97	199.96	199.38	850.99
281	182.9	172.18	153.52	150.19	658.79
282	147.39	136.47	128.33	122.79	534.98
283	256.31	234.53	201.6	182.84	875.28
284	197.07	181.71	164.92	165.86	709.56
285	195.07	195.53	173.84	167.22	731.66
286	276.79	265.3	244.7	237.15	1,023.94
287	165.54	152.81	139.01	127.51	584.87
288	70.86	69.29	60.45	65.87	266.47
289	20.53	20.37	18.81	20.39	80.1
TOTAL	3,098.55	2,916.56	2,645.05	2,606.07	11,266.23

STATE: NORTH DAKOTA

ZIP CODE	QUARTER 1	QUARTER 2	QUARTER 3	QUARTER 4	TOTAL GRAMS
580	14.38	13.68	12.46	13.03	53.55
581	50.3	50.94	50.25	44.33	195.82
582	43.34	39.56	39.29	40.84	163.03
583	20.64	17.88	16.64	16.32	71.48
584	18.43	16.39	14.92	14.26	64
585	57.25	58.63	54.26	57.8	227.94
586	17.61	17.13	16.38	15.22	66.34
587	16.92	17.13	17.45	17.12	68.62

	588	9.28	9.07	9.09	9.64	37.08
TOTAL		248.15	240.41	230.74	228.56	947.86

STATE:	OHIO					
ZIP CODE		QUARTER 1	QUARTER 2	QUARTER 3	QUARTER 4	TOTAL GRAMS
	430	160.12	157.31	141.19	138.6	597.22
	431	80.83	80.39	76.24	75.19	312.65
	432	357.46	355.27	318.39	305.76	1,336.88
	433	37.23	33.96	30.84	28.91	130.94
	434	32.95	33.51	32.2	29.9	128.56
	435	86.5	87.78	83.79	78.01	336.08
	436	159.65	153.18	152.31	136.88	602.02
	437	39.93	37.87	36.56	33.15	147.51
	438	4.36	3.93	3.77	3.43	15.49
	439	39.94	38.48	39.01	34.71	152.14
	440	128.89	122.69	111.81	102.31	465.7
	441	183.32	180.11	173.17	162.84	699.44
	442	93.91	82.58	75.02	69.08	320.59
	443	39.23	37.46	33.41	34.66	144.76
	444	57.01	53.79	49.45	46.72	206.97
	445	63.87	61.08	61.62	58.29	244.86
	446	94.32	81.02	72.13	64.7	312.17
	447	55.12	52.32	47.81	48.28	203.53
	448	63.02	53.85	53.06	50.91	220.84
	449	23.15	20.14	16.76	16.63	76.68
	450	180.9	174.46	168.47	150.59	674.42
	451	53.84	52.66	51.12	49.17	206.79
	452	182.99	178.21	173.79	167.45	702.44
	453	108	106.23	97.79	93	405.02
	454	119.33	113.75	103.21	106.93	443.22
	455	27.91	25.99	22.65	22.01	98.56
	456	90.58	85.53	86.27	83.97	346.35
	457	29.57	27.64	26.28	26.01	109.5
	458	57.87	53.31	48.82	48.89	208.89
TOTAL		2,651.80	2,544.50	2,386.94	2,266.98	9,850.22

RETAIL DRUG DISTRIBUTION BY ZIP CODE WITHIN STATE BY GRAMS WT

STATE: OKLAHOMA

ZIP CODE	QUARTER 1	QUARTER 2	QUARTER 3	QUARTER 4	TOTAL GRAMS
730	293.33	277.28	265.35	239.87	1,075.83
731	354.13	301.43	278.95	265.6	1,200.11
734	45.65	44.01	43.22	37.7	170.58
735	121.95	115.92	115.34	104.29	457.5
736	45.86	39.73	34.8	28.93	149.32
737	50.42	48.38	43.98	42.05	184.83
738	12.24	13.09	11.21	11.18	47.72
739	3.82	2.35	3.31	2.64	12.12
740	304.14	294.16	285.07	268.76	1,152.13
741	239.33	213.72	213.95	214.87	881.87
743	59.09	56.41	55.36	53.41	224.27
744	107.48	113.9	97.73	90.42	409.53
745	45.51	44.91	37.72	39.4	167.54
746	24.7	26.96	25.24	24.26	101.16
747	41.18	35.49	30.88	29.21	136.76
748	71.77	74.66	65.84	66.8	279.07
749	26.85	27.9	25.62	24.44	104.81
TOTAL	1,847.45	1,730.30	1,633.57	1,543.83	6,755.15

STATE: OREGON

ZIP CODE	QUARTER 1	QUARTER 2	QUARTER 3	QUARTER 4	TOTAL GRAMS
970	153.12	140.76	132.16	122.52	548.56
971	78.97	73.6	74.55	72.48	299.6
972	221.65	218.72	189.72	197.72	827.81
973	135.65	130.97	123.52	116.85	506.99
974	183.49	169.68	160.28	151.66	665.11
975	93.57	83.8	76.53	75.47	329.37
976	15.82	13.94	14.65	12.74	57.15
977	54.04	58.54	54.89	51.83	219.3
978	53.33	48.72	45.32	43.61	190.98
979	16.87	15.97	15.2	12.73	60.77
TOTAL	1,006.51	954.7	886.82	857.61	3,705.64

STATE: PENNSYLVANIA

ZIP CODE	QUARTER 1	QUARTER 2	QUARTER 3	QUARTER 4	TOTAL GRAMS
150	251.59	253.32	235.81	232.61	973.33
151	109.41	114.42	110.31	104	438.14
152	266.37	259.24	245.08	225.74	996.43
153	48.07	48.42	42.31	39.53	178.33
154	54.69	51.24	49.69	44.21	199.83
155	48.75	43.39	42.77	38.72	173.63
156	124.94	124.03	108.77	106.86	464.6
157	88.48	90.43	83.76	74.87	337.54
158	68.81	64.25	61.73	58.27	253.06
159	101.25	96.46	95.21	81.57	374.49
160	79.63	76.91	79.57	76.18	312.29
161	87.98	83.09	78.03	71.8	320.9
162	46	43.77	40.32	34.81	164.9
163	97.44	96.34	92.83	91.73	378.34
164	26.62	25.87	25.3	27.07	104.86
165	79.96	74.59	70.59	65.35	290.49
166	115.02	118.2	114.3	102	449.52
167	19.25	20.31	18.74	16.46	74.76
168	46.01	49.03	45.74	38.29	179.07
169	25.41	24.46	24.47	20.25	94.59
170	235.36	214.16	200.38	193.33	843.23
171	65.55	58.57	55.55	53.14	232.81
172	51.86	51.4	53.19	48.94	205.39
173	114.78	109.48	104.13	95.33	423.72
174	71.77	69.32	67.36	64.89	273.34
175	69.31	61.58	59.09	53.87	243.85
176	58.68	56.19	53.98	55.82	224.67
177	90.38	87.61	77.75	77.75	333.49
178	118.8	111.23	104.79	99.07	433.89
179	38.08	40.57	38.53	35.59	152.77
180	159.42	154.39	138.59	130.74	583.14
181	111.66	100.23	92.39	94.87	399.15
182	69.38	67.75	58.96	59.38	255.47
183	58.21	53.62	54.42	53.8	220.05

	184	73.65	71.72	67.95	63.49	276.81
	185	113.94	106.43	95.83	90.2	406.4
	186	85.69	79.56	81.16	72.35	318.76
	187	323.01	330.8	271.93	282.3	1,208.04
	188	35.82	36.27	32.69	32.76	137.54
	189	123.01	111.68	104.4	100.87	439.96
	190	712.99	690.26	641.1	629.52	2,673.87
	191	383.57	385.12	350.67	328.11	1,447.47
	193	120.35	114.28	98.79	100.79	434.21
	194	200.91	195.81	186.31	172.24	755.27
	195	50.99	45.42	46.51	41.45	184.37
	196	51.51	49.43	49.29	49.58	199.81
TOTAL		5,374.36	5,210.65	4,851.07	4,630.50	20,066.58

STATE: PUERTO RICO

ZIP CODE		QUARTER 1	QUARTER 2	QUARTER 3	QUARTER 4	TOTAL GRAMS
	006	51.42	30.57	20.77	16.64	119.4
	007	66.56	53.6	44.01	30.04	194.21
	009	78.89	66.84	61.79	57.12	264.64
TOTAL		196.87	151.01	126.57	103.8	578.25

STATE: RHODE ISLAND

ZIP CODE		QUARTER 1	QUARTER 2	QUARTER 3	QUARTER 4	TOTAL GRAMS
	028	121.94	110.33	108.55	107.6	448.42
	029	75.86	76.45	69.2	65.04	286.55
TOTAL		197.8	186.78	177.75	172.64	734.97

STATE: SOUTH CAROLINA

ZIP CODE		QUARTER 1	QUARTER 2	QUARTER 3	QUARTER 4	TOTAL GRAMS
	290	90.49	89.98	83.93	79.21	343.61
	291	102.8	99.33	88.82	97.39	388.34
	292	100.55	106.37	94.49	84.77	386.18
	293	125.96	121.88	108.72	109.04	465.6
	294	208.32	195.79	197	191.31	792.42

	295	268.23	245.39	225.57	223.89	963.08
	296	297.73	281.29	255.04	252.68	1,086.74
	297	68.91	64.53	56.43	54.24	244.11
	298	79.91	74.76	72.97	72.26	299.9
	299	81.12	67.09	76.14	67.2	291.55
TOTAL		1,424.02	1,346.41	1,259.11	1,231.99	5,261.53

STATE:		SOUTH DAKOTA				
ZIP CODE		QUARTER 1	QUARTER 2	QUARTER 3	QUARTER 4	TOTAL GRAMS
	570	48.87	47.82	47.43	45.81	189.93
	571	145.72	127.19	125.62	122.05	520.58
	572	19.67	16.45	17.17	17.36	70.65
	573	23.06	21.77	23.2	23.36	91.39
	574	21.45	22.06	19.55	18.98	82.04
	575	19.45	19.04	15.09	13.91	67.49
	576	2.84	3.6	3.84	4.45	14.73
	577	44.26	43.95	43.89	35.32	167.42
TOTAL		325.32	301.88	295.79	281.24	1,204.23

STATE:		TENNESSEE				
ZIP CODE		QUARTER 1	QUARTER 2	QUARTER 3	QUARTER 4	TOTAL GRAMS
	370	247.61	232.23	214.12	209.84	903.8
	371	191.73	189.67	178.3	159.66	719.36
	372	165	154.22	145.57	154.79	619.58
	373	168.32	154.04	129.02	128.67	580.05
	374	69.72	66.22	61.89	54.09	251.92
	376	137.37	130.07	119.53	118.31	505.28
	377	78.44	69.6	67.09	62.19	277.32
	378	143.12	133.46	122.46	115.59	514.63
	379	106.09	98.58	85.9	86.38	376.95
	380	90.94	86.52	84.73	81.25	343.44
	381	423.65	399.61	399.17	390.3	1,612.73
	382	30.8	28.2	28.68	28.28	115.96

	383	85.34	77.17	79.3	72.23	314.04
	384	52.2	50.26	43.06	44.03	189.55
	385	103.59	95.3	86.83	79.08	364.8
TOTAL		2,093.92	1,965.15	1,845.65	1,784.69	7,689.41

STATE: TEXAS

ZIP CODE		QUARTER 1	QUARTER 2	QUARTER 3	QUARTER 4	TOTAL GRAMS
	750	576.37	539.43	491.48	469.62	2,076.90
	751	144.66	145.4	142.25	135.74	568.05
	752	249.37	233.6	210.63	206.09	899.69
	753	11.25	10.54	11.21	12.21	45.21
	754	170.97	159.67	151.96	138.91	621.51
	755	21.84	21.85	22.57	21.6	87.86
	756	105.82	103.32	89.4	112.79	411.33
	757	133.27	137.65	130.31	128.83	530.06
	758	23.69	20.33	21.89	22.29	88.2
	759	61.24	58	56.15	55.39	230.78
	760	359.76	357.89	323.8	311.86	1,353.31
	761	351.14	343.81	315.16	311.26	1,321.37
	762	140.53	137.49	133.96	130.54	542.52
	763	91.76	87	80.5	78.92	338.18
	764	57.12	51.68	52.62	51.76	213.18
	765	101.64	90.77	88.02	89.61	370.04
	766	27.2	27.62	24.71	26.96	106.49
	767	48.93	44.09	44.02	44.6	181.64
	768	43.28	46.25	44.79	44.42	178.74
	769	28.86	29.77	27.53	27.82	113.98
	770	364.8	368.36	318.67	340.65	1,392.48
	772	1.19	0.04	0.02	0.03	1.28
	773	262.62	254.27	244.47	251	1,012.36
	774	192.63	182.74	171.78	167.42	714.57
	775	241.92	229.62	229.64	216.16	917.34
	776	64.96	63.93	57.11	56.97	242.97
	777	30.08	30	26.51	26.22	112.81
	778	67.44	63.27	59.8	56.82	247.33
	779	61.85	60.8	58.69	55.29	236.63

	780	79.32	77.5	76.35	75.93	309.1
	781	89.44	88.39	82.61	81.21	341.65
	782	456.38	435.38	413.03	409.71	1,714.50
	783	53.14	48.72	45.3	41.16	188.32
	784	108.83	103.99	97.75	90.76	401.33
	785	134.84	124.63	129.59	126.74	515.8
	786	256.34	247.26	235.27	223.82	962.69
	787	244.34	232.25	208.19	198.57	883.35
	788	25.96	17.88	16.91	16.42	77.17
	789	12.12	10.79	12.86	11.68	47.45
	790	66.39	64.53	60.68	57	248.6
	791	120.98	117.66	111.34	108.83	458.81
	792	14.1	10.03	10.49	9.51	44.13
	793	38.6	36.91	35.02	33.3	143.83
	794	103.85	102.23	101.71	97.35	405.14
	795	35.24	34.51	33.52	32.41	135.68
	796	61.15	68.63	59.84	62.71	252.33
	797	54.35	58.43	55.79	54.35	222.92
	798	5.58	4.55	4.83	4.22	19.18
	799	131.52	129.84	122.3	113.69	497.35
TOTAL		6,128.66	5,913.30	5,543.03	5,441.15	23,026.14

STATE: UTAH

ZIP CODE		QUARTER 1	QUARTER 2	QUARTER 3	QUARTER 4	TOTAL GRAMS
	840	299.67	286.67	277.62	273.55	1,137.51
	841	220.92	205.9	204.15	199.43	830.4
	843	44.9	45.7	44.69	44.82	180.11
	844	64.82	63.1	61.26	58.68	247.86
	845	34.01	29.87	26.53	26.59	117
	846	81.88	78.67	78.36	72.09	311
	847	116.63	111.09	113.32	110.65	451.69
TOTAL		862.83	821	805.93	785.81	3,275.57

STATE: VERMONT

ZIP CODE		QUARTER 1	QUARTER 2	QUARTER 3	QUARTER 4	TOTAL GRAMS

	050	11.58	11.81	10.25	10.6	44.24
	051	9.72	6.45	6.45	5.44	28.06
	052	12.94	12.03	12	11.9	48.87
	053	5.89	4.67	5.84	3.67	20.07
	054	81.4	85.44	73.96	70.66	311.46
	056	27.93	25.8	24.17	19.88	97.78
	057	34.71	33.34	30.95	29.07	128.07
	058	18.58	17.18	16.53	17.21	69.5
TOTAL		202.75	196.72	180.15	168.43	748.05

STATE: VIRGIN ISLANDS

ZIP CODE		QUARTER 1	QUARTER 2	QUARTER 3	QUARTER 4	TOTAL GRAMS
	008	4.01	3.46	3.02	3.47	13.96
TOTAL		4.01	3.46	3.02	3.47	13.96

STATE: VIRGINIA

ZIP CODE		QUARTER 1	QUARTER 2	QUARTER 3	QUARTER 4	TOTAL GRAMS
	201	133.66	123.51	110.19	110.43	477.79
	220	73.94	60.76	65.1	52.49	252.29
	221	48.44	46.16	37.47	37.08	169.15
	222	15.77	14.94	13.93	9.98	54.62
	223	33.28	34.89	31.17	26.8	126.14
	224	48.85	44.37	41.39	40.47	175.08
	225	17.22	15.09	14.88	14.53	61.72
	226	56.15	59.32	55.49	54.71	225.67
	227	15.52	13	10.37	9.24	48.13
	228	47.2	49.31	43.79	46.28	186.58
	229	77.27	70.98	67.12	60.72	276.09
	230	64.5	65.77	59.34	56.69	246.3
	231	92.85	81.59	65.84	74.79	315.07
	232	179.99	156.32	148.69	133.47	618.47
	233	73.52	65.59	65.21	66.23	270.55
	234	158.37	149.43	139.51	134.04	581.35
	235	56.02	50.92	46.39	46.6	199.93

	236	94.98	82.84	79.22	69.92	326.96
	237	50.14	43.66	41.14	41.64	176.58
	238	69.8	68.58	57.19	55.16	250.73
	239	20.28	17.29	16.59	14.99	69.15
	240	110.39	101.7	99.36	90.58	402.03
	241	88.43	83.55	71.34	67.81	311.13
	242	93.76	83.82	76.19	71.23	325
	243	43.54	41.29	35.45	32.93	153.21
	244	46.79	48.95	43.03	44.93	183.7
	245	86.43	83.55	85.13	82.48	337.59
	246	24.21	24.47	22.81	21.01	92.5
TOTAL		1,921.30	1,781.65	1,643.33	1,567.23	6,913.51

STATE: WASHINGTON

ZIP CODE		QUARTER 1	QUARTER 2	QUARTER 3	QUARTER 4	TOTAL GRAMS
	980	209.69	199.24	185.64	171.11	765.68
	981	170.15	183.94	151.42	155.94	661.45
	982	172.51	166.4	154.99	144.61	638.51
	983	155.33	155.78	150.46	143.13	604.7
	984	98.32	89.05	85.71	91.24	364.32
	985	78.43	71.39	72.02	70.08	291.92
	986	193.24	181.21	167.36	163.72	705.53
	988	35.33	37.15	35.41	36.66	144.55
	989	74.31	73.48	69.88	65.77	283.44
	990	22.88	23.89	24.77	21.79	93.33
	991	37.69	34.95	34.16	32.95	139.75
	992	178.35	175.27	172.79	161.79	688.2
	993	164.34	142.31	133	122.93	562.58
	994	24.02	22.49	21.33	20.6	88.44
TOTAL		1,614.59	1,556.55	1,458.94	1,402.32	6,032.40

STATE: WEST VIRGINIA

ZIP CODE		QUARTER 1	QUARTER 2	QUARTER 3	QUARTER 4	TOTAL GRAMS
	247	14.41	14.96	12.98	14.45	56.8
	248	5.45	4.83	4.24	5.08	19.6

	249	13.41	9.07	8.3	7.6	38.38
	250	14.82	14.57	14.45	14.42	58.26
	251	10	8.48	7.69	9.28	35.45
	252	15.01	15.79	15.27	13.64	59.71
	253	71.92	68.34	67.97	59.32	267.55
	254	73.44	68.75	65.18	62	269.37
	255	29.38	28.98	27.8	25.93	112.09
	256	10.74	9.06	10.1	9.26	39.16
	257	51.38	49	43.16	40.16	183.7
	258	19.3	20.18	20	18.33	77.81
	259	12.42	11.41	9.97	8.7	42.5
	260	39.42	38.99	37.82	35.36	151.59
	261	29.05	29.59	27.97	26.35	112.96
	262	19.83	20.51	21.29	20.46	82.09
	263	35.61	36.27	31.09	29.34	132.31
	264	11.41	11.11	10.54	9.27	42.33
	265	63.25	61.59	57.84	56.41	239.09
	266	8.83	7.7	7.78	7.19	31.5
	267	19.99	20.62	20.18	17.92	78.71
	268	8.56	9.34	9.2	9.39	36.49
TOTAL		577.63	559.14	530.82	499.86	2,167.45

STATE: WISCONSIN

ZIP CODE		QUARTER 1	QUARTER 2	QUARTER 3	QUARTER 4	TOTAL GRAMS
	530	161.07	147.86	142.48	136.22	587.63
	531	155.35	155.87	132.47	126.47	570.16
	532	250.59	226.51	210.82	205.07	892.99
	534	29.09	25.75	22.52	23.28	100.64
	535	159.85	150.08	133.45	127.08	570.46
	537	118.99	100.96	90.54	89.73	400.22
	538	22.37	22.36	22.7	21.28	88.71
	539	74.56	69.83	62.03	54.01	260.43
	540	25.81	24.3	24.02	21.38	95.51
	541	38.93	38.54	34.89	32.63	144.99
	542	24.76	23.2	21.94	22.2	92.1
	543	57.1	59.22	54.02	52.81	223.15

	544	82.56	71.01	66.39	65.49	285.45
	545	16.09	16.27	17.51	14.08	63.95
	546	96.7	98	89.84	87.62	372.16
	547	82.05	77.48	66.67	67.19	293.39
	548	41.87	40.56	33.12	35.44	150.99
	549	199.73	176.56	165.58	163.32	705.19
TOTAL		1,637.47	1,524.36	1,390.99	1,345.30	5,898.12

STATE: WYOMING

ZIP CODE		QUARTER 1	QUARTER 2	QUARTER 3	QUARTER 4	TOTAL GRAMS
	820	36.39	29.53	32.62	29.42	127.96
	822	7.57	7.14	5.7	5.81	26.22
	823	1.36	1.36	1.43	1.68	5.83
	824	17.39	16.99	18.9	17.93	71.21
	825	8.28	6.88	7.66	5.73	28.55
	826	31.74	29.68	27.33	25.68	114.43
	827	6.13	4.53	6.97	5.45	23.08
	828	11.27	9.59	10.36	10.06	41.28
	829	36.07	34.32	36.63	33.35	140.37
	830	6.07	5.04	6.32	5.16	22.59
	831	6	4.45	4.1	4.94	19.49
TOTAL		168.27	149.51	158.02	145.21	621.01

DRUG CODE: 9809 DRUG NAME: OPIUM COMBINATION PRODUCT (C-III)

STATE: ALABAMA

ZIP CODE		QUARTER 1	QUARTER 2	QUARTER 3	QUARTER 4	TOTAL GRAMS
	350	0	0.76	0.76	0.38	1.9
	351	0.38	0.57	2.08	0.19	3.22
	352	3.03	3.59	1.7	3.03	11.35
	354	0.19	0	0.19	0	0.38
	355	0	0	0.19	0	0.19
	356	0	0.19	0	0.19	0.38
	357	1.32	0.95	0.95	0.95	4.17
	358	1.51	2.27	1.7	1.14	6.62

	359	3.78	3.41	3.41	3.22	13.82
	361	0	0.19	0	0	0.19
	362	1.32	0.19	0.19	0.38	2.08
	363	0	0	0	0.19	0.19
	364	0	0	0.38	0.19	0.57
	365	0.38	0.38	0.19	0.38	1.33
	366	0	0.19	0	0.38	0.57
	368	0	0	0	0.19	0.19
TOTAL		11.91	12.69	11.74	10.81	47.15

STATE: ALASKA

ZIP CODE		QUARTER 1	QUARTER 2	QUARTER 3	QUARTER 4	TOTAL GRAMS
	997	0	0	0.19	0	0.19
TOTAL		0	0	0.19	0	0.19

STATE: ARIZONA

ZIP CODE		QUARTER 1	QUARTER 2	QUARTER 3	QUARTER 4	TOTAL GRAMS
	850	1.51	0.95	0.38	1.89	4.73
	851	0.19	0	0	0	0.19
	852	10.03	4.16	6.24	5.49	25.92
	853	0.19	0.19	0	0.19	0.57
	857	0.19	0.19	0.57	0.76	1.71
	864	0	0	0	0.19	0.19
TOTAL		12.11	5.49	7.19	8.52	33.31

STATE: ARKANSAS

ZIP CODE		QUARTER 1	QUARTER 2	QUARTER 3	QUARTER 4	TOTAL GRAMS
	719	0	0	0.57	0	0.57
	720	0.76	0.95	0	0.38	2.09
	721	0	0	0	0.19	0.19
	722	0	0	0.19	0.19	0.38
	727	0.57	1.32	1.89	1.32	5.1
	729	0	0	0.19	0	0.19
TOTAL		1.33	2.27	2.84	2.08	8.52

STATE: CALIFORNIA

ZIP CODE	QUARTER 1	QUARTER 2	QUARTER 3	QUARTER 4	TOTAL GRAMS
900	3.59	1.89	3.03	3.03	11.54
902	0	0	0	0.19	0.19
903	0	0.38	0	0	0.38
904	0	0	0.19	0	0.19
905	0.38	0	0	0	0.38
906	0	0.19	0	0	0.19
907	0	0.19	0	0	0.19
908	0.57	1.14	0.38	0.57	2.66
910	0.19	0	0	0	0.19
913	0.19	0	0	1.32	1.51
914	0.19	0.57	0.19	0	0.95
915	0.38	0.19	0.38	0.38	1.33
917	0	0.19	0.19	0.38	0.76
920	3.41	1.14	1.89	2.84	9.28
921	1.7	0.57	1.7	1.32	5.29
922	0.19	0.19	0	0.19	0.57
925	0.38	0.57	0.76	0.57	2.28
926	0	0.19	0.19	0	0.38
928	0.38	0.38	0.19	0.38	1.33
930	0	0.19	0	0.38	0.57
932	0.57	0	0	0	0.57
933	0.76	0.76	0.76	0.76	3.04
934	0.57	0.76	2.08	2.84	6.25
935	0.38	0.19	0	0	0.57
936	0	0.19	0	0	0.19
939	0.19	0	0	0.19	0.38
940	0	0.19	0.19	1.32	1.7
941	0	0	0.57	0.19	0.76
943	0.19	0	0	0.19	0.38
944	0	0	0	0.19	0.19
945	0	1.32	0.38	0	1.7
947	0	0.19	0	0	0.19
948	0.19	0	0	0	0.19

949	0.19	0.38	0	0.38	0.95
950	0.76	0.38	1.14	0.38	2.66
951	0	0.19	0.38	0.19	0.76
953	1.51	2.46	1.7	2.27	7.94
954	0	0.76	0.95	0.76	2.47
955	0.19	0	0.57	0.76	1.52
956	0.19	0.57	0	0	0.76
957	0.38	0	0	0	0.38
958	0	0.57	0.19	0.38	1.14
959	0.57	1.14	0	0.19	1.9
TOTAL	18.19	18.02	18	22.54	76.75

STATE: COLORADO

ZIP CODE	QUARTER 1	QUARTER 2	QUARTER 3	QUARTER 4	TOTAL GRAMS
800	0	0.19	0	0	0.19
801	0.19	0	0.19	0.38	0.76
802	1.14	1.32	0.95	0.76	4.17
804	0	0.19	0	0	0.19
805	0.57	0.19	0.95	0.57	2.28
809	0.19	0.38	0.19	0.57	1.33
810	1.14	0.76	0	0	1.9
811	0	0	0	0.19	0.19
TOTAL	3.23	3.03	2.28	2.47	11.01

STATE: CONNECTICUT

ZIP CODE	QUARTER 1	QUARTER 2	QUARTER 3	QUARTER 4	TOTAL GRAMS
60	1.7	0.95	1.14	2.27	6.06
61	0.19	0.38	0.38	0.57	1.52
62	0.57	0.19	0.38	0.19	1.33
64	1.89	0.95	0.38	0.38	3.6
66	0	0	0.19	0.19	0.38
68	0.76	1.14	1.14	0.57	3.61
69	0	0.19	2.84	0	3.03
TOTAL	5.11	3.8	6.45	4.17	19.53

DATE RANGE: 01/01/2017 TO 12/31/2017

ARCOS 3 - REPORT 1

RETAIL DRUG DISTRIBUTION BY ZIP CODE WITHIN STATE BY GRAMS WT

Run Date: 07/03/2018

STATE: DELAWARE

ZIP CODE	QUARTER 1	QUARTER 2	QUARTER 3	QUARTER 4	TOTAL GRAMS
197	0	0.57	0	0.19	0.76
199	0.19	0.19	0.19	0.76	1.33
TOTAL	0.19	0.76	0.19	0.95	2.09

STATE: DISTRICT OF COLUMBIA

ZIP CODE	QUARTER 1	QUARTER 2	QUARTER 3	QUARTER 4	TOTAL GRAMS
200	0.19	0.38	0	0.57	1.14
204	0	0	0.38	0	0.38
TOTAL	0.19	0.38	0.38	0.57	1.52

STATE: FLORIDA

ZIP CODE	QUARTER 1	QUARTER 2	QUARTER 3	QUARTER 4	TOTAL GRAMS
320	1.32	0.57	0.76	0.19	2.84
321	0	0.76	0.19	0.76	1.71
322	1.7	0.95	0	0.76	3.41
323	0.38	0.57	0.57	0	1.52
324	0.76	0.57	0.38	0.38	2.09
325	0.38	0.38	1.14	0.76	2.66
326	3.97	4.35	2.46	5.49	16.27
327	0.38	0.95	0.76	0.38	2.47
328	1.7	1.7	0.95	1.14	5.49
329	4.54	2.84	4.73	3.22	15.33
330	2.08	2.08	1.32	2.46	7.94
331	0.95	4.73	2.65	2.84	11.17
333	2.08	4.16	1.89	1.89	10.02
334	6.24	4.35	3.97	3.59	18.15
335	0.95	0.57	0.19	0.19	1.9
336	1.14	1.32	2.27	0.95	5.68
337	1.14	0.38	0.57	0.38	2.47
338	0.76	0.19	0.57	0.57	2.09

	339	1.14	0.19	1.14	1.51	3.98
	341	0	0.38	0	0	0.38
	342	2.65	3.03	2.84	1.51	10.03
	344	0.19	0.19	0.19	0	0.57
	346	0.57	0.76	0.19	0	1.52
	347	0	0.57	0	0.19	0.76
	349	1.51	1.51	2.08	2.27	7.37
TOTAL		36.53	38.05	31.81	31.43	137.82

STATE: GEORGIA

ZIP CODE		QUARTER 1	QUARTER 2	QUARTER 3	QUARTER 4	TOTAL GRAMS
	300	1.51	0.38	0	0.38	2.27
	301	0.57	0.38	0.57	0.95	2.47
	302	0.76	0.95	0.57	0.19	2.47
	303	0.57	0.76	1.32	1.32	3.97
	305	0.76	1.7	1.32	1.51	5.29
	306	0.38	0.38	0.38	0.38	1.52
	307	0.19	0	0	0.19	0.38
	308	0	1.32	0.76	1.14	3.22
	309	0.57	0.57	0.19	0	1.33
	312	0	0.38	0.38	0	0.76
	314	0.19	0.38	0.38	0	0.95
	315	0.19	0.95	0.95	0.76	2.85
	316	0.19	0.19	0.57	0.38	1.33
	317	0.19	0	0	0	0.19
	319	0	0.38	0.19	0	0.57
TOTAL		6.07	8.72	7.58	7.2	29.57

STATE: HAWAII

ZIP CODE		QUARTER 1	QUARTER 2	QUARTER 3	QUARTER 4	TOTAL GRAMS
	968	1.89	1.51	1.32	0	4.72
TOTAL		1.89	1.51	1.32	0	4.72

STATE: IDAHO

ZIP CODE	QUARTER 1	QUARTER 2	QUARTER 3	QUARTER 4	TOTAL GRAMS
832	0.76	1.14	0.38	0.57	2.85
833	0.19	0.19	0.38	0.38	1.14
834	0.19	0	0.19	0	0.38
836	0.19	0	0	0	0.19
838	2.65	2.46	2.65	2.84	10.6
TOTAL	3.98	3.79	3.6	3.79	15.16

STATE: ILLINOIS

ZIP CODE	QUARTER 1	QUARTER 2	QUARTER 3	QUARTER 4	TOTAL GRAMS
600	0.19	1.14	2.08	1.51	4.92
601	0.38	0.19	0.19	0.57	1.33
602	0	0	0	0.19	0.19
603	0.57	0.57	0.38	0.38	1.9
604	0	0.19	0	0	0.19
605	0	0	0.57	0	0.57
606	2.08	0.95	1.14	0.95	5.12
609	0.19	0.95	0.38	0	1.52
610	0	0	0	0.19	0.19
612	0	0.19	0	0	0.19
613	0	0.19	0	0	0.19
617	0.57	0	0.19	0	0.76
618	0.38	0.57	0.57	0.19	1.71
622	0.95	0.76	0.57	0.76	3.04
626	0.19	0	0	0	0.19
627	0.38	0.38	0.57	0.38	1.71
628	0.19	0	0	0	0.19
TOTAL	6.07	6.08	6.64	5.12	23.91

STATE: INDIANA

ZIP CODE	QUARTER 1	QUARTER 2	QUARTER 3	QUARTER 4	TOTAL GRAMS
460	0	0.19	0.19	0	0.38
462	0.76	0.57	0.38	2.65	4.36
463	0	0.57	0	0	0.57

	465	0	0.19	0	0.38	0.57
	467	0.19	0	0	0	0.19
	468	0.19	0	0	0	0.19
	471	0	0	0.19	0	0.19
	472	3.22	1.89	3.78	0.76	9.65
	473	0	0	0	0.19	0.19
	474	2.27	3.03	3.78	2.84	11.92
	475	0.19	0.38	0	0.38	0.95
TOTAL		6.82	6.82	8.32	7.2	29.16

STATE: IOWA

ZIP CODE		QUARTER 1	QUARTER 2	QUARTER 3	QUARTER 4	TOTAL GRAMS
	503	0.38	0	0	0	0.38
	513	0.95	0.76	0.57	0.76	3.04
	522	1.51	1.14	1.14	1.32	5.11
	525	0.19	0	0	0	0.19
	528	0.19	0	0	0	0.19
TOTAL		3.22	1.9	1.71	2.08	8.91

STATE: KANSAS

ZIP CODE		QUARTER 1	QUARTER 2	QUARTER 3	QUARTER 4	TOTAL GRAMS
	662	0.19	0	0.19	0.38	0.76
	666	0	0	0	0.57	0.57
	668	0.19	0	0	0	0.19
	670	0	0.19	0	0	0.19
	671	0	0.19	0	0.19	0.38
	672	0	0.19	0	0.19	0.38
	674	0.19	0	0	0	0.19
	679	0.19	0	0	0	0.19
TOTAL		0.76	0.57	0.19	1.33	2.85

STATE: KENTUCKY

ZIP CODE		QUARTER 1	QUARTER 2	QUARTER 3	QUARTER 4	TOTAL GRAMS

	400	0	0	0	0.38	0.38
	401	0	0	0.19	0	0.19
	402	0.76	0.95	0.57	0.57	2.85
	404	0.57	0.95	0.95	0.57	3.04
	405	2.27	1.51	1.51	0.94	6.23
	406	2.65	3.03	1.7	1.89	9.27
	407	0.76	1.32	0.76	0.76	3.6
	410	0.19	0.19	0.19	0.38	0.95
	413	0	0	0.19	0	0.19
	414	0	0.19	0	0	0.19
	415	0	0	0	0.38	0.38
	418	0.19	0	0.19	0.38	0.76
	420	1.89	2.08	0.76	0.76	5.49
	421	0	0	0	0.19	0.19
	422	0.57	0	0.38	0	0.95
	425	0.38	0.57	0	0.57	1.52
	427	0.38	0.19	0.38	0	0.95
TOTAL		10.61	10.98	7.77	7.77	37.13

STATE: LOUISIANA

ZIP CODE		QUARTER 1	QUARTER 2	QUARTER 3	QUARTER 4	TOTAL GRAMS
	700	4.16	3.97	4.54	3.03	15.7
	701	1.32	0.57	2.27	3.03	7.19
	703	0.38	0.19	0.19	0	0.76
	704	1.14	3.03	1.89	1.14	7.2
	705	0	0	0	0.38	0.38
	706	0	0	0	0.19	0.19
	707	0	0.19	0.19	0	0.38
	708	0	0.19	0.19	0.19	0.57
	711	0.19	0.19	0	0.19	0.57
	712	0	0.38	0	0	0.38
TOTAL		7.19	8.71	9.27	8.15	33.32

STATE: MAINE

ZIP CODE	QUARTER 1	QUARTER 2	QUARTER 3	QUARTER 4	TOTAL GRAMS
40	0.38	0.38	0.57	1.51	2.84
41	0	0	0.38	0	0.38
42	0	0.38	0.57	0.19	1.14
43	0.38	0.38	0.38	0.57	1.71
44	0	0	0	0.57	0.57
48	0	0	0.19	0	0.19
TOTAL	0.76	1.14	2.09	2.84	6.83

STATE: MARYLAND

ZIP CODE	QUARTER 1	QUARTER 2	QUARTER 3	QUARTER 4	TOTAL GRAMS
206	1.14	1.32	0.57	0.57	3.6
208	0.38	0.19	0	0.38	0.95
209	0	0.19	0	0	0.19
210	1.14	0.38	0.19	0	1.71
211	0	0	0	0.19	0.19
212	2.27	3.03	3.97	2.84	12.11
214	0.19	0.19	0	0	0.38
216	3.22	2.46	3.59	3.03	12.3
217	0	0.19	0.38	0.95	1.52
218	0	0.76	0.19	0	0.95
219	0.19	0	0	0	0.19
TOTAL	8.53	8.71	8.89	7.96	34.09

STATE: MASSACHUSETTS

ZIP CODE	QUARTER 1	QUARTER 2	QUARTER 3	QUARTER 4	TOTAL GRAMS
10	0.19	0.57	0	0	0.76
12	0	0.19	0.19	0.57	0.95
15	0.38	0	0	0	0.38
17	0.38	0.38	0	0	0.76
18	0	0	0.19	0	0.19
19	0.76	0.57	0.38	0.38	2.09
21	0	0.57	0.38	0	0.95
24	0.19	0	0.19	0	0.38
27	0.19	0.19	0	0	0.38

TOTAL	2.09	2.47	1.33	0.95	6.84

STATE: MICHIGAN

ZIP CODE	QUARTER 1	QUARTER 2	QUARTER 3	QUARTER 4	TOTAL GRAMS
480	0.38	0.76	1.89	0.75	3.78
481	0.38	0.57	0.38	0	1.33
482	0	0.19	0.57	0	0.76
483	1.32	0.76	2.08	1.7	5.86
484	0	0	0	0.19	0.19
487	0.19	0	0	0	0.19
490	0	0.38	0.76	0	1.14
494	0.19	0.38	0.38	0	0.95
495	0.95	0.38	1.32	0.38	3.03
496	0.57	0.57	0.57	0.57	2.28
497	0	0.19	0	0	0.19
499	0.76	0.76	0.57	0.95	3.04
TOTAL	4.74	4.94	8.52	4.54	22.74

STATE: MINNESOTA

ZIP CODE	QUARTER 1	QUARTER 2	QUARTER 3	QUARTER 4	TOTAL GRAMS
550	0.38	0.57	0.38	0.38	1.71
551	0	0.38	0	0	0.38
553	0	0.19	0	0.76	0.95
554	0.19	0.19	0.38	0	0.76
557	0	0	0	0.19	0.19
558	0	0	0	0.19	0.19
559	0	0.57	0.76	0.95	2.28
560	0	0	0.38	0	0.38
561	0.95	0	0	0.38	1.33
563	0.19	0.38	0.19	0.19	0.95
TOTAL	1.71	2.28	2.09	3.04	9.12

STATE: MISSISSIPPI

ZIP CODE	QUARTER 1	QUARTER 2	QUARTER 3	QUARTER 4	TOTAL GRAMS
386	0.19	0	0	0	0.19
388	0.95	0.38	1.51	0	2.84
389	0.38	0	0	0	0.38
393	0.19	0.19	0	0	0.38
394	2.08	0.76	0.57	0.38	3.79
395	0.38	0.57	0.57	0.57	2.09
396	0.38	0.38	0.38	0	1.14
397	0	0.19	0	0.76	0.95
TOTAL	4.55	2.47	3.03	1.71	11.76

STATE: MISSOURI

ZIP CODE	QUARTER 1	QUARTER 2	QUARTER 3	QUARTER 4	TOTAL GRAMS
630	0.95	0	0	0.19	1.14
631	4.16	1.51	1.89	7.38	14.94
633	0	0	0	0.19	0.19
640	1.14	0.76	0.38	0	2.28
641	0.76	0.19	0	0.19	1.14
645	0.19	0.19	0	0	0.38
646	0	0.19	0	0	0.19
648	0	0	0.19	0	0.19
651	0.95	0.76	0	0	1.71
652	1.7	1.51	1.51	1.32	6.04
658	0.38	0	0	0	0.38
TOTAL	10.23	5.11	3.97	9.27	28.58

STATE: MONTANA

ZIP CODE	QUARTER 1	QUARTER 2	QUARTER 3	QUARTER 4	TOTAL GRAMS
594	0	0	0	0.19	0.19
597	0.19	0	0	0	0.19
598	0.76	0.19	0.95	0.76	2.66
599	0	0	0.38	0	0.38
TOTAL	0.95	0.19	1.33	0.95	3.42

RETAIL DRUG DISTRIBUTION BY ZIP CODE WITHIN STATE BY GRAMS WT

STATE: NEBRASKA

ZIP CODE	QUARTER 1	QUARTER 2	QUARTER 3	QUARTER 4	TOTAL GRAMS
680	0	0	0.19	0	0.19
681	0.95	0.57	0.38	0	1.9
684	0.19	0	0	0.57	0.76
693	0	0	0.19	0	0.19
TOTAL	1.14	0.57	0.76	0.57	3.04

STATE: NEVADA

ZIP CODE	QUARTER 1	QUARTER 2	QUARTER 3	QUARTER 4	TOTAL GRAMS
890	0	0.57	0.19	0	0.76
891	0.57	0.19	1.51	0.38	2.65
895	0.19	0.19	0	0	0.38
897	0	0.38	0	0	0.38
TOTAL	0.76	1.33	1.7	0.38	4.17

STATE: NEW HAMPSHIRE

ZIP CODE	QUARTER 1	QUARTER 2	QUARTER 3	QUARTER 4	TOTAL GRAMS
30	0	0.76	0.57	0.57	1.9
32	0	0	0.38	0	0.38
34	0	0.19	0.19	0	0.38
35	0.76	0.19	0	0.38	1.33
37	0	0	0	0.38	0.38
38	0.95	0.76	0.76	2.08	4.55
TOTAL	1.71	1.9	1.9	3.41	8.92

STATE: NEW JERSEY

ZIP CODE	QUARTER 1	QUARTER 2	QUARTER 3	QUARTER 4	TOTAL GRAMS
70	0.57	0.76	0.76	0.19	2.28
72	0	0.38	0	0	0.38
74	0.19	0.76	0.19	0	1.14
76	0.76	0.57	0	0	1.33
77	0.19	0.38	0.57	0	1.14

	78	0.19	0.19	0	0	0.38
	79	0.19	0	0	0	0.19
	80	1.89	1.51	0.38	1.32	5.1
	81	0	0	0.38	0	0.38
	85	0.95	0	0	0	0.95
	87	0.38	1.14	2.08	1.89	5.49
	88	0.38	0	0.95	0.76	2.09
TOTAL		5.69	5.69	5.31	4.16	20.85

STATE: NEW MEXICO

ZIP CODE	QUARTER 1	QUARTER 2	QUARTER 3	QUARTER 4	TOTAL GRAMS
871	1.32	1.32	0.19	0.19	3.02
875	0.57	0.57	0.76	1.32	3.22
877	0	0	0	0.19	0.19
882	0	0	0	0.19	0.19
TOTAL	1.89	1.89	0.95	1.89	6.62

STATE: NEW YORK

ZIP CODE	QUARTER 1	QUARTER 2	QUARTER 3	QUARTER 4	TOTAL GRAMS
100	0.76	0.57	1.32	1.14	3.79
103	0	0	0.19	0	0.19
104	0	0.38	0.19	0	0.57
105	0.76	0.57	0.19	0.95	2.47
109	0	0	0	0.38	0.38
110	0	0.19	0	0	0.19
112	0.38	0.38	0	0.19	0.95
113	0.76	0.19	0.38	0	1.33
114	0.19	0.19	0	0	0.38
115	3.03	5.11	2.84	2.46	13.44
117	0.19	0	0.38	1.51	2.08
118	0	0.38	0.57	0.76	1.71
119	0.38	0	0	0	0.38
123	0	0	0	0.19	0.19
124	0	0	0	0.19	0.19

	125	0.19	0	0	0.19	0.38
	127	0	0	0.38	0	0.38
	128	0	0	0.19	0	0.19
	129	0	0	0	0.38	0.38
	130	0.19	0	0.19	0	0.38
	132	0	0	0.19	0	0.19
	133	0	0	0.19	0	0.19
	134	0	0	0.19	0.38	0.57
	138	0.19	0	0	0	0.19
	140	0.57	0.19	0.19	0.57	1.52
	141	0	0.19	0	0.38	0.57
	142	0.19	0.76	0.38	0.19	1.52
	144	0.19	0.76	0	0.19	1.14
	145	0	0	0.19	0	0.19
	146	0.19	0.38	0.19	0.19	0.95
	148	0.19	0.19	0.19	0.57	1.14
TOTAL		8.35	10.43	8.53	10.81	38.12

STATE:	NORTH CAROLINA				
ZIP CODE	QUARTER 1	QUARTER 2	QUARTER 3	QUARTER 4	TOTAL GRAMS
270	0.57	0.76	0.38	0.57	2.28
271	0.76	0.19	0.19	0.19	1.33
272	0.57	0.95	1.89	1.7	5.11
273	0.19	0.38	0	0	0.57
274	0.95	1.51	0.95	0.95	4.36
275	0.57	0.76	0.76	0.19	2.28
276	0	0	0	0.38	0.38
278	0.76	0	0.38	0.19	1.33
279	0	0	0	0.19	0.19
280	1.51	1.14	1.32	0.57	4.54
281	0.19	0.38	0.76	0.57	1.9
282	0.57	0.38	0.19	0.19	1.33
283	0.19	0.19	0.19	0.19	0.76
284	0.57	1.32	0.76	1.32	3.97
285	0.95	0.95	0.57	0.76	3.23
286	0.76	1.51	1.7	1.14	5.11

287	0.57	1.32	0.57	0.95	3.41
288	0.95	0.19	0.57	0	1.71
289	1.51	2.84	3.97	1.89	10.21
TOTAL	12.14	14.77	15.15	11.94	54

STATE: NORTH DAKOTA

ZIP CODE	QUARTER 1	QUARTER 2	QUARTER 3	QUARTER 4	TOTAL GRAMS
585	0	0.38	0.57	0.57	1.52
588	0	0	0.19	0.95	1.14
TOTAL	0	0.38	0.76	1.52	2.66

STATE: OHIO

ZIP CODE	QUARTER 1	QUARTER 2	QUARTER 3	QUARTER 4	TOTAL GRAMS
430	0	0.76	0.57	0.19	1.52
431	0	0.38	0	0	0.38
432	8.89	6.62	3.22	10.6	29.33
436	0	0	0.38	0	0.38
440	0.76	0.19	0.19	0	1.14
441	0.76	1.32	2.44	0.76	5.28
442	1.89	0.19	1.14	0.95	4.17
443	0	0	0	0.19	0.19
444	0	0	0	0.19	0.19
445	0	0	0	0.19	0.19
446	0	0.19	0	0.38	0.57
447	1.14	0.38	0.57	0.19	2.28
448	0	0.19	0	0	0.19
449	0.38	0.38	0.38	0.38	1.52
450	0.19	1.89	0.38	1.7	4.16
451	0	0.19	0.19	0	0.38
452	0.19	0.19	0.95	0.76	2.09
453	0.38	0.19	0	0	0.57
454	0	0.76	0.57	0.76	2.09
456	0.19	0	0	0.57	0.76
457	0.19	0	0.19	0.38	0.76
458	0	0	0	0.19	0.19

TOTAL		14.96	13.82	11.17	18.38	58.33

STATE:	OKLAHOMA					
ZIP CODE		QUARTER 1	QUARTER 2	QUARTER 3	QUARTER 4	TOTAL GRAMS
	730	0.19	0.38	0.19	0	0.76
	731	0	0.19	0	0.19	0.38
	734	0.19	0.19	0.38	0	0.76
	735	0	0	0.19	0	0.19
	740	0.76	0	0	1.14	1.9
	741	0.95	1.32	2.46	2.27	7
	744	0.38	0	0	0.76	1.14
	745	0.76	0	0	0	0.76
TOTAL		3.23	2.08	3.22	4.36	12.89

STATE:	OREGON					
ZIP CODE		QUARTER 1	QUARTER 2	QUARTER 3	QUARTER 4	TOTAL GRAMS
	970	0	0.38	0.19	0.57	1.14
	971	0	0	0.19	0	0.19
	972	0	0	0	0.19	0.19
	973	0.19	0	0.38	0	0.57
	975	0	0.19	0.19	1.14	1.52
TOTAL		0.19	0.57	0.95	1.9	3.61

STATE:	PENNSYLVANIA					
ZIP CODE		QUARTER 1	QUARTER 2	QUARTER 3	QUARTER 4	TOTAL GRAMS
	150	0	0.76	0.57	0.76	2.09
	151	0.76	0.57	1.51	0.95	3.79
	152	5.87	4.16	7.38	3.78	21.19
	153	0.19	0.18	0.57	0.38	1.32
	154	0	0	0	0.57	0.57
	155	0	0	0.19	0	0.19
	156	1.32	1.14	1.14	0.76	4.36
	158	0	0	0.19	0	0.19
	160	0	0.19	0.76	0	0.95

	161	0.76	0.38	0.38	0.57	2.09
	162	0.19	0.19	0.19	0.38	0.95
	163	0.19	0.76	0	0	0.95
	165	0.38	0.57	0.38	0.38	1.71
	166	0	0	0	0.38	0.38
	170	0.38	0	0	0.57	0.95
	171	0.19	0	0	0	0.19
	172	0	0.57	0	0	0.57
	173	0.38	0.38	0	0	0.76
	174	0.19	0	0.19	0.38	0.76
	177	0.38	0.19	0.19	0.38	1.14
	178	1.14	1.32	1.14	1.32	4.92
	179	0	0	0	0.38	0.38
	180	0	0	0	0.19	0.19
	181	0.57	0.38	0.19	0	1.14
	182	5.87	4.54	6.43	5.3	22.14
	184	0	0	0.38	0.38	0.76
	186	0.38	0	0.19	0	0.57
	187	8.89	17.6	0.19	16.08	42.76
	189	0.38	0.57	0.57	0	1.52
	190	0.95	1.32	0.38	0.76	3.41
	191	0.76	0.76	1.14	0.76	3.42
	193	0.19	0.19	0.19	0.19	0.76
	194	1.14	1.89	2.84	2.46	8.33
	195	0	0.38	0	0	0.38
TOTAL		31.45	38.99	27.28	38.06	135.78

STATE:	RHODE ISLAND				
ZIP CODE	QUARTER 1	QUARTER 2	QUARTER 3	QUARTER 4	TOTAL GRAMS
28	0	0.19	0.19	0	0.38
TOTAL	0	0.19	0.19	0	0.38

STATE:	SOUTH CAROLINA				
ZIP CODE	QUARTER 1	QUARTER 2	QUARTER 3	QUARTER 4	TOTAL GRAMS
290	0	0.76	0.57	0.95	2.28

	291	0.19	0	0.57	0.19	0.95
	292	0.57	0.95	0.76	0.57	2.85
	293	3.22	4.35	3.97	4.35	15.89
	294	1.32	1.51	1.32	0.19	4.34
	295	0.95	1.32	1.51	1.32	5.1
	296	1.32	1.51	1.32	1.51	5.66
	297	0.38	0.19	0.19	0.38	1.14
	298	0.38	0	0	0	0.38
	299	0	0.19	0.57	0.76	1.52
TOTAL		8.33	10.78	10.78	10.22	40.11

STATE: SOUTH DAKOTA

ZIP CODE		QUARTER 1	QUARTER 2	QUARTER 3	QUARTER 4	TOTAL GRAMS
	571	1.14	0.57	0.57	0	2.28
	577	0.19	0	0	0.19	0.38
TOTAL		1.33	0.57	0.57	0.19	2.66

STATE: TENNESSEE

ZIP CODE		QUARTER 1	QUARTER 2	QUARTER 3	QUARTER 4	TOTAL GRAMS
	370	0.38	0.57	0.19	0.95	2.09
	371	0.76	0.76	1.32	1.14	3.98
	372	0.19	0.19	0.19	0.38	0.95
	373	0	0	0.38	0	0.38
	374	0.95	0.76	0	0	1.71
	376	0.19	0.19	0.38	0.57	1.33
	377	0.38	0.38	0.19	0.38	1.33
	378	0.95	1.14	1.7	1.7	5.49
	379	0.38	0.57	0.38	0.76	2.09
	380	0.38	0	0.38	0.95	1.71
	381	0.57	0.38	0.57	0.76	2.28
	382	0.19	0.19	0	0.19	0.57
	383	0.19	0.38	0	0	0.57
	384	0.19	0	0	0.38	0.57
TOTAL		5.7	5.51	5.68	8.16	25.05

STATE: TEXAS

ZIP CODE	QUARTER 1	QUARTER 2	QUARTER 3	QUARTER 4	TOTAL GRAMS
750	0.76	0.57	0.76	0.38	2.47
752	0.76	0.76	0.76	0.95	3.23
753	0	0	0.19	0	0.19
754	0	0	0	0.19	0.19
756	0	0	0.19	0	0.19
757	0.19	0.76	1.89	1.51	4.35
760	0.38	0.76	0.38	0.19	1.71
761	0.76	0.95	0.19	0.38	2.28
762	0	0	0	0.38	0.38
764	0.19	0	0	0	0.19
765	0.76	0.76	0	0	1.52
766	0.57	0.38	0.38	0.76	2.09
768	0	1.32	0.76	0.76	2.84
769	1.51	3.03	2.46	2.27	9.27
770	0	0.19	0.57	0.38	1.14
773	0.76	0.19	0	0	0.95
774	0.38	0.76	1.14	0.57	2.85
775	0.76	0	0.95	0.57	2.28
777	0	0	0	0.19	0.19
780	0.19	0	0	0	0.19
782	0.38	0.76	0.76	0.57	2.47
786	0.38	0.76	0.38	0.95	2.47
787	2.65	1.51	0.76	1.32	6.24
790	0	0.38	0	0	0.38
793	0	0	0	0.19	0.19
796	0	0.38	0.57	1.7	2.65
799	0	0	0	0.19	0.19
TOTAL	11.38	14.22	13.09	14.4	53.09

STATE: UTAH

ZIP CODE	QUARTER 1	QUARTER 2	QUARTER 3	QUARTER 4	TOTAL GRAMS
840	5.68	4.35	3.97	6.24	20.24
841	2.27	2.08	2.46	1.7	8.51

	843	0.19	0.19	0.19	0.38	0.95
	844	1.7	2.27	2.27	2.84	9.08
	846	2.84	3.59	1.51	0.76	8.7
	847	0.38	0.19	0.57	0.95	2.09
TOTAL		13.06	12.67	10.97	12.87	49.57

STATE: VERMONT

ZIP CODE		QUARTER 1	QUARTER 2	QUARTER 3	QUARTER 4	TOTAL GRAMS
	50	0	0.38	0.19	0.38	0.95
TOTAL		0	0.38	0.19	0.38	0.95

STATE: VIRGINIA

ZIP CODE		QUARTER 1	QUARTER 2	QUARTER 3	QUARTER 4	TOTAL GRAMS
	201	0	0	0.57	0	0.57
	221	0.95	0.76	0.19	0.19	2.09
	224	0.95	0.19	0.38	0	1.52
	226	0	0.19	0	0.19	0.38
	228	0.38	0.57	0	0.19	1.14
	229	0.76	0.19	0.76	0.57	2.28
	230	0.38	0.19	0.19	0	0.76
	231	0.57	1.32	0.38	0.19	2.46
	232	0.19	0.19	0.76	0.95	2.09
	234	0.57	0.38	0.19	0	1.14
	235	1.14	0.19	0	0.19	1.52
	236	0	0	0.19	0	0.19
	238	0	0	0	0.19	0.19
	239	0	0	0	0.19	0.19
	240	0.95	0	0.19	0.19	1.33
	242	0.76	0.57	0.38	0.38	2.09
	243	0	0.38	0.38	0.19	0.95
	244	0.19	0.38	0.76	1.14	2.47
	245	0.19	0	0.19	0	0.38
	246	0	0	0.19	0	0.19
TOTAL		7.98	5.5	5.7	4.75	23.93

DATE RANGE: 01/01/2017 TO 12/31/2017

ARCOS 3 - REPORT 1

RETAIL DRUG DISTRIBUTION BY ZIP CODE WITHIN STATE BY GRAMS WT

Run Date: 07/03/2018

STATE: WASHINGTON

ZIP CODE	QUARTER 1	QUARTER 2	QUARTER 3	QUARTER 4	TOTAL GRAMS
980	0	0	0.38	0.19	0.57
981	0	0.57	0	0	0.57
982	0.38	0.76	1.14	0.19	2.47
983	0.38	0	0.19	0.38	0.95
984	0	0	0.19	0	0.19
985	0	0.19	0	0	0.19
986	0	0	0	0.19	0.19
988	0	0	0.19	0	0.19
991	0.38	0.19	0.38	0.57	1.52
992	0.57	0.19	0.56	0.57	1.89
993	0.38	0.19	1.14	0	1.71
TOTAL	2.09	2.09	4.17	2.09	10.44

STATE: WEST VIRGINIA

ZIP CODE	QUARTER 1	QUARTER 2	QUARTER 3	QUARTER 4	TOTAL GRAMS
247	0.57	0.38	0.95	0.57	2.47
251	0.19	0.38	0	0.19	0.76
253	0.19	0	0.19	0.19	0.57
255	0.95	0.76	0.57	1.14	3.42
257	0.38	0.19	0	0	0.57
258	0.19	0	0	0.19	0.38
260	0	0	0	0.19	0.19
261	0	0	0	0.19	0.19
263	0	0.19	0.19	0	0.38
265	0.19	0	0.57	0	0.76
TOTAL	2.66	1.9	2.47	2.66	9.69

STATE: WISCONSIN

ZIP CODE	QUARTER 1	QUARTER 2	QUARTER 3	QUARTER 4	TOTAL GRAMS
532	0	1.7	1.89	1.7	5.29
535	0	0	0	0.19	0.19
537	2.08	0	0.19	0.57	2.84

	541	0.19	0	0.19	0	0.38
	544	2.84	3.03	2.84	4.54	13.25
	545	0.19	0	0	0	0.19
	546	0.19	0	0	0	0.19
	548	0	0.19	0.19	0	0.38
	549	0.19	0	0.19	0	0.38
TOTAL		5.68	4.92	5.49	7	23.09

STATE: WYOMING

ZIP CODE		QUARTER 1	QUARTER 2	QUARTER 3	QUARTER 4	TOTAL GRAMS
	820	0	0.38	0.57	1.51	2.46
	826	0	0.19	0.19	0	0.38
	829	0	0	0	0.19	0.19
TOTAL		0	0.57	0.76	1.7	3.03

RETAIL DRUG DISTRIBUTION BY STATE WITHIN DRUG CODE BY GRAMS WT

DRUG CODE:1100 DRUG NAME:AMPHETAMINE

STATE	QUARTER 1	QUARTER 2	QUARTER 3	QUARTER 4	TOTAL GRAMS
ALABAMA	129,522.52	129,197.88	129,399.68	133,902.59	522,022.67
ALASKA	8,017.63	8,035.54	8,190.14	8,091.96	32,335.27
AMERICAN SAMOA	1.25	1.25	0	0	2.5
ARIZONA	81,311.77	80,184.72	80,199.21	85,874.24	327,569.94
ARKANSAS	50,604.83	50,551.75	50,903.79	52,311.33	204,371.70
CALIFORNIA	295,548.59	301,541.39	296,156.77	304,886.80	1,198,133.55
COLORADO	77,961.82	78,762.94	80,330.49	82,398.93	319,454.18
CONNECTICUT	51,291.82	52,762.24	52,806.74	54,411.74	211,272.54
DELAWARE	21,634.53	21,501.74	21,199.97	21,865.65	86,201.89
DISTRICT OF COLUMBIA	11,565.87	11,640.51	11,504.66	11,955.82	46,666.86
FLORIDA	296,894.70	298,005.58	291,272.31	315,032.71	1,201,205.30
GEORGIA	186,484.74	184,712.71	186,327.72	192,738.54	750,263.71
GUAM	411.77	559.5	574.21	358.63	1,904.11
HAWAII	9,569.19	9,834.42	9,939.96	9,576.50	38,920.07
IDAHO	24,608.37	25,240.99	25,335.53	26,579.68	101,764.57
ILLINOIS	188,728.87	188,378.21	189,659.18	197,200.90	763,967.16
INDIANA	127,718.22	128,736.61	129,954	133,581.79	519,990.62
IOWA	48,435.44	49,394.50	49,817.41	52,122.76	199,770.11
KANSAS	58,640.79	59,886.78	60,660.15	62,570.96	241,758.68
KENTUCKY	68,115.50	66,555.89	67,282.59	69,993.68	271,947.66
LOUISIANA	152,840.05	150,402.28	152,820.35	161,545.77	617,608.45
MAINE	21,419.93	22,777.34	23,454.54	23,020.49	90,672.30
MARYLAND	90,180.32	91,107.65	91,040.76	93,934.66	366,263.39
MASSACHUSETTS	174,995.37	179,718.58	175,691.40	181,453.66	711,859.01
MICHIGAN	238,888.13	239,018.63	239,220.46	244,814.10	961,941.32
MINNESOTA	106,640.37	107,219.41	106,743.34	111,048.37	431,651.49
MISSISSIPPI	62,603.84	60,446.21	60,437.34	62,550.25	246,037.64
MISSOURI	131,873.12	132,162.08	133,905.97	138,655.85	536,597.02
MONTANA	13,605.53	13,878.40	14,159.86	14,352.84	55,996.63
NEBRASKA	25,323.08	25,225.28	26,017.59	26,750.34	103,316.29
NEVADA	25,495.19	25,984.38	25,970.40	26,697.57	104,147.54
NEW HAMPSHIRE	37,268.50	38,741.66	38,476.01	39,382.95	153,869.12
NEW JERSEY	101,969.53	103,664.89	103,491.02	107,553.26	416,678.70
NEW MEXICO	16,802.06	16,226.35	15,622.66	15,009.26	63,660.33
NEW YORK	228,378.91	235,698.16	233,426.04	244,230.77	941,733.88

NORTH CAROLINA	200,834.82	201,804.95	201,294.03	208,597.67	812,531.47
NORTH DAKOTA	8,808.92	8,886.90	9,070.85	9,197.64	35,964.31
OHIO	207,941.78	208,201.07	209,912.76	220,964.23	847,019.84
OKLAHOMA	52,284.73	52,570.95	52,516.13	54,232.93	211,604.74
OREGON	57,185.60	59,272.39	58,568.95	59,537.23	234,564.17
PENNSYLVANIA	208,503.68	211,247.55	208,839.24	215,744.51	844,334.98
PUERTO RICO	9,076.62	8,442.37	7,845.57	7,750.67	33,115.23
RHODE ISLAND	35,384.78	36,481.19	36,854.42	37,818.74	146,539.13
SOUTH CAROLINA	136,661.36	134,954.13	133,936.64	139,640.78	545,192.91
SOUTH DAKOTA	15,983.46	14,915.82	14,655.77	15,257.17	60,812.22
TENNESSEE	129,020.72	128,485.65	128,719.73	134,212.64	520,438.74
TEXAS	407,471.01	411,025.34	407,717.31	429,646.03	1,655,859.69
UTAH	79,212.88	81,029.06	81,748.72	85,695.73	327,686.39
VERMONT	9,800.02	9,909.35	9,781.02	10,199.01	39,689.40
VIRGIN ISLANDS	368.82	364.19	251.15	233.08	1,217.24
VIRGINIA	177,158.94	178,362.49	176,595.37	181,966.29	714,083.09
WASHINGTON	107,196.40	109,746.86	108,248.01	110,209.82	435,401.09
WEST VIRGINIA	23,855.35	23,267.05	23,223.58	23,626.77	93,972.75
WISCONSIN	129,986.53	126,614.29	125,467.50	128,764.70	510,833.02
WYOMING	6,609.86	6,232.11	6,410.92	6,483.05	25,735.94
TOTAL	5,168,698.43	5,199,570.16	5,183,649.92	5,386,234.04	20,938,152.55

DRUG CODE:1105B DRUG NAME:DL-METHAMPHETAMINE RACEMIC BASE

STATE	QUARTER 1	QUARTER 2	QUARTER 3	QUARTER 4	TOTAL GRAMS
ALABAMA	22.5	24.51	27.73	21.3	96.04
ALASKA	0.8	4.82	4.02	1.21	10.85
ARIZONA	34.16	32.15	28.53	39.38	134.22
ARKANSAS	5.22	7.23	6.83	6.03	25.31
CALIFORNIA	320.68	311.03	271.25	249.95	1,152.91
COLORADO	49.03	54.25	49.83	57.46	210.57
CONNECTICUT	20.49	24.51	20.09	24.51	89.6
DELAWARE	2.41	3.62	4.82	4.42	15.27
DISTRICT OF COLUMBIA	4.02	2.01	0	0.8	6.83
FLORIDA	28.13	39.38	36.57	31.75	135.83
GEORGIA	32.15	24.51	17.68	20.49	94.83
HAWAII	5.22	6.83	4.02	4.42	20.49

IDAHO	7.64	6.83	10.45	10.05	34.97
ILLINOIS	63.89	58.27	63.49	62.69	248.34
INDIANA	29.74	26.12	29.74	18.08	103.68
IOWA	14.87	18.89	18.89	21.3	73.95
KANSAS	10.85	12.46	8.04	10.45	41.8
KENTUCKY	10.85	10.05	9.64	9.24	39.78
LOUISIANA	14.06	14.87	14.06	12.06	55.05
MAINE	3.21	6.83	10.45	12.86	33.35
MARYLAND	41.39	44.61	38.18	40.19	164.37
MASSACHUSETTS	44.61	41.39	43	39.38	168.38
MICHIGAN	22.91	22.5	21.7	24.51	91.62
MINNESOTA	78.36	87.2	63.89	59.88	289.33
MISSISSIPPI	8.44	8.04	8.84	7.64	32.96
MISSOURI	49.03	62.29	16.07	62.69	190.08
MONTANA	1.21	0.8	0.4	0.4	2.81
NEBRASKA	6.03	8.84	8.84	9.24	32.95
NEVADA	23.71	29.34	22.1	22.5	97.65
NEW HAMPSHIRE	8.04	7.64	6.83	3.62	26.13
NEW JERSEY	38.18	30.54	31.75	37.77	138.24
NEW MEXICO	27.33	20.9	12.86	4.82	65.91
NEW YORK	74.34	80.77	76.75	93.63	325.49
NORTH CAROLINA	47.82	56.66	42.19	42.6	189.27
NORTH DAKOTA	2.81	7.64	6.03	7.23	23.71
OHIO	77.56	80.77	48.62	93.23	300.18
OKLAHOMA	23.71	16.48	20.49	16.48	77.16
OREGON	44.2	32.95	19.29	31.34	127.78
PENNSYLVANIA	118.55	134.22	116.13	120.15	489.05
RHODE ISLAND	4.82	5.63	2.81	3.62	16.88
SOUTH CAROLINA	18.49	12.06	16.48	13.26	60.29
SOUTH DAKOTA	15.67	22.1	17.28	14.47	69.52
TENNESSEE	10.45	12.46	12.46	15.27	50.64
TEXAS	116.54	116.13	91.22	102.87	426.76
UTAH	25.32	23.31	26.52	28.93	104.08
VERMONT	0.8	2.01	2.01	2.41	7.23
VIRGINIA	51.03	40.59	39.78	43.8	175.2
WASHINGTON	90.82	95.64	91.22	103.68	381.36
WEST VIRGINIA	3.21	4.42	5.63	3.21	16.47

WISCONSIN	61.48	62.29	42.19	31.34	197.3
WYOMING	6.03	7.23	4.82	3.21	21.29
TOTAL	1,822.81	1,866.62	1,592.51	1,701.82	6,983.76

DRUG CODE:1105D DRUG NAME:D-METHAMPHETAMINE

STATE	QUARTER 1	QUARTER 2	QUARTER 3	QUARTER 4	TOTAL GRAMS
ALABAMA	0	0.8	6.83	7.23	14.86
ALASKA	0	0	0.8	0	0.8
ARIZONA	0	4.42	4.02	11.25	19.69
CALIFORNIA	0	23.06	61.14	58.65	142.85
CONNECTICUT	0	0	3.62	4.42	8.04
DISTRICT OF COLUMBIA	0	0.8	2.81	1.61	5.22
FLORIDA	0	4.82	4.42	3.21	12.45
GEORGIA	0	1.61	4.82	4.82	11.25
HAWAII	0	0	1.61	1.61	3.22
IDAHO	0	1.21	5.22	4.02	10.45
ILLINOIS	0	0	3.62	2.41	6.03
KANSAS	0	0.8	0.8	2.81	4.41
KENTUCKY	0	0.4	1.61	1.61	3.62
LOUISIANA	0	0	2.01	2.41	4.42
MARYLAND	0	0	1.21	3.62	4.83
MASSACHUSETTS	0	0.8	3.62	2.41	6.83
MINNESOTA	0	4.42	25.71	22.9	53.03
MISSISSIPPI	0	0	1.61	0.8	2.41
MISSOURI	0	3.21	12.05	16.87	32.13
NEBRASKA	0	0	0	0.8	0.8
NEVADA	0	0	0.4	0	0.4
NEW JERSEY	0	0.8	2.81	2.01	5.62
NEW YORK	4.02	0	6.83	6.91	17.76
NORTH CAROLINA	0	2.01	9.64	10.04	21.69
NORTH DAKOTA	0	0	5.62	0.4	6.02
OHIO	0	0.4	8.44	12.45	21.29
OKLAHOMA	0	0.4	4.02	2.81	7.23
OREGON	0	9.24	14.06	14.46	37.76
PENNSYLVANIA	0	3.21	11.65	11.25	26.11
RHODE ISLAND	0	0	1.61	2.01	3.62

SOUTH CAROLINA	0	2.01	4.02	2.81	8.84
TENNESSEE	0	0	0	0.4	0.4
TEXAS	0	4.42	18.08	17.27	39.77
WASHINGTON	0	0	0.8	1.61	2.41
WEST VIRGINIA	0	0	2.41	1.21	3.62
WISCONSIN	0	1.61	13.66	12.85	28.12
TOTAL	4.02	70.45	251.58	251.95	578

DRUG CODE:1205 DRUG NAME:LISDEXAMFETAMINE

STATE	QUARTER 1	QUARTER 2	QUARTER 3	QUARTER 4	TOTAL GRAMS
ALABAMA	73,124.45	69,008.38	69,770.43	73,421.78	285,325.04
ALASKA	2,366.74	2,413.57	2,534.39	2,551.16	9,865.86
ARIZONA	35,652.58	34,515.46	35,081.42	38,130.90	143,380.36
ARKANSAS	31,934.82	28,732.73	29,007.32	31,528.74	121,203.61
CALIFORNIA	111,261.70	111,775.06	108,794.14	114,459.16	446,290.06
COLORADO	27,319.27	27,395.58	27,495.59	28,822.33	111,032.77
CONNECTICUT	35,946.26	36,527.83	34,903.37	37,374.17	144,751.63
DELAWARE	14,390.07	14,231.09	13,743.17	14,852.55	57,216.88
DISTRICT OF COLUMBIA	5,127.17	5,166.48	5,261.29	5,454.95	21,009.89
FLORIDA	143,974.07	135,084.05	128,667.31	141,074.99	548,800.42
GEORGIA	105,611.35	98,907.13	101,109.11	103,946.40	409,573.99
GUAM	37.58	27.17	12.14	13.87	90.76
HAWAII	1,348.71	1,304.77	1,331.94	1,372.99	5,358.41
IDAHO	8,492.29	8,748.97	8,782.50	9,373.31	35,397.07
ILLINOIS	71,184.34	69,483	69,469.12	73,303.66	283,440.12
INDIANA	86,359.47	83,479.37	83,966.13	87,564.23	341,369.20
IOWA	35,187.21	34,025.81	33,274.86	35,151.95	137,639.83
KANSAS	31,604.15	31,149.76	31,014.49	33,384.12	127,152.52
KENTUCKY	43,977.79	41,332.99	41,486.74	42,833.74	169,631.26
LOUISIANA	98,011.07	88,695.57	89,750.60	94,902.51	371,359.75
MAINE	16,286.23	16,819.82	16,980.53	17,223.91	67,310.49
MARYLAND	56,770.58	55,794.17	53,445.92	57,445.85	223,456.52
MASSACHUSETTS	35,567.60	36,220.86	34,222.94	36,886.25	142,897.65
MICHIGAN	60,273.80	58,677.73	57,238.14	61,662.92	237,852.59
MINNESOTA	31,804.17	32,248.73	31,807.64	30,819.09	126,679.63
MISSISSIPPI	40,741.02	37,115.18	37,862.08	40,202.81	155,921.09

MISSOURI	67,909.41	64,820.04	65,065.16	68,418.14	266,212.75
MONTANA	5,844.59	5,535.31	5,568.84	5,961.37	22,910.11
NEBRASKA	16,416.88	15,918.56	16,075.92	17,354.48	65,765.84
NEVADA	7,799.73	7,551.72	7,207.06	7,719.95	30,278.46
NEW HAMPSHIRE	13,951.29	14,138.59	13,564.54	13,983.66	55,638.08
NEW JERSEY	49,063.35	49,824.70	47,456.23	49,616.59	195,960.87
NEW MEXICO	4,711.52	4,368.70	4,101.62	4,077.92	17,259.76
NEW YORK	83,860.89	84,888.75	81,943.94	87,724.90	338,418.48
NORTH CAROLINA	104,400.84	101,389.49	97,896.61	106,885.02	410,571.96
NORTH DAKOTA	5,931.88	5,516.23	5,592.54	5,851.53	22,892.18
OHIO	115,341.01	109,211.18	108,377.10	113,584.68	446,513.97
OKLAHOMA	37,115.11	35,305.15	35,866.35	38,390.49	146,677.10
OREGON	14,860.64	15,086.10	14,691.83	15,479.18	60,117.75
PENNSYLVANIA	86,193.55	85,037.93	83,572.45	88,797.32	343,601.25
PUERTO RICO	2,120.47	1,865.53	1,730.25	1,889.23	7,605.48
RHODE ISLAND	7,438.99	7,675.43	7,171.91	7,825.16	30,111.49
SOUTH CAROLINA	62,814.61	59,107.26	58,061.36	64,448.06	244,431.29
SOUTH DAKOTA	11,455.05	10,899.50	10,515.06	10,965.98	43,835.59
TENNESSEE	72,415.12	68,092.09	68,641.40	71,450.56	280,599.17
TEXAS	282,570.08	270,317.25	258,324.68	277,763.06	1,088,975.07
UTAH	21,398.37	21,111.63	21,113.37	22,982.37	86,605.74
VERMONT	6,875.34	6,792.68	6,786.89	7,249.37	27,704.28
VIRGIN ISLANDS	78.62	68.22	47.98	72.84	267.66
VIRGINIA	69,037.86	68,163.77	65,077.30	68,702.91	270,981.84
WASHINGTON	33,478.93	34,401	33,707.74	34,724.65	136,312.32
WEST VIRGINIA	20,520.82	19,364.62	18,863.40	19,838.66	78,587.50
WISCONSIN	65,047.23	65,814.37	65,424.73	71,265.28	267,551.61
WYOMING	2,701.46	2,558.67	2,568.50	2,621.68	10,450.31
TOTAL	2,475,708.13	2,393,705.73	2,352,028.07	2,499,403.38	9,720,845.31

DRUG CODE:1724 DRUG NAME:METHYLPHENIDATE (DL;D;L;ISOMERS)

STATE	QUARTER 1	QUARTER 2	QUARTER 3	QUARTER 4	TOTAL GRAMS
ALABAMA	76,441.86	69,939.23	69,273.14	75,215.07	290,869.30
ALASKA	8,308.25	7,938.66	7,812.94	8,079.10	32,138.95
AMERICAN SAMOA	2.59	6.05	4.32	4.76	17.72
ARIZONA	76,843.41	71,741.10	71,852.92	77,682.89	298,120.32

ARKANSAS	42,738.26	38,885.47	37,985.81	40,491.31	160,100.85
CALIFORNIA	280,017.65	271,790.79	259,368.75	276,806.18	1,087,983.37
COLORADO	62,961.72	60,411.10	59,560.82	63,461.83	246,395.47
CONNECTICUT	55,654.28	55,007.99	50,532.25	55,121.41	216,315.93
DELAWARE	19,422.76	18,908.96	17,653.36	19,110.67	75,095.75
DISTRICT OF COLUMBIA	8,149.44	8,276.15	7,546.97	8,227.44	32,200
FLORIDA	222,746.10	205,780.52	184,896.75	209,350.28	822,773.65
GEORGIA	175,509.10	154,308.03	156,407.40	167,480.79	653,705.32
GUAM	562.07	394.32	633.74	324.62	1,914.75
HAWAII	10,893.55	10,932.98	10,782.82	11,026.95	43,636.30
IDAHO	27,982.04	27,772.05	25,796.99	28,036.03	109,587.11
ILLINOIS	183,062.06	171,136.73	164,334.65	177,772.06	696,305.50
INDIANA	128,505.04	119,020.53	116,461.55	121,311.99	485,299.11
IOWA	85,301.45	81,314.10	77,115.85	82,345.02	326,076.42
KANSAS	52,329.49	49,623.14	47,814.40	50,486.24	200,253.27
KENTUCKY	79,957.38	71,563.96	72,425.05	76,221.48	300,167.87
LOUISIANA	93,211.93	78,871.78	79,234.69	87,401.96	338,720.36
MAINE	35,708.93	35,725.10	34,610.63	35,180.12	141,224.78
MARYLAND	104,696.27	101,052.01	93,114.43	101,335.63	400,198.34
MASSACHUSETTS	149,723.22	149,480.86	136,831.79	148,614.43	584,650.30
MICHIGAN	196,629.98	188,328.66	177,232.93	192,684.13	754,875.70
MINNESOTA	103,381.49	100,563.87	94,501.53	104,893.88	403,340.77
MISSISSIPPI	46,464.57	40,093.82	41,116.20	43,645.13	171,319.72
MISSOURI	135,184.09	126,144.06	128,490.32	136,556.50	526,374.97
MONTANA	18,347.86	17,175.60	16,958.78	17,493.61	69,975.85
NEBRASKA	33,026.53	30,615.36	29,742.71	31,755.77	125,140.37
NEVADA	15,406.98	14,798.82	13,977.47	15,058.02	59,241.29
NEW HAMPSHIRE	34,410.12	34,736.63	32,581.78	33,911.45	135,639.98
NEW JERSEY	96,262.44	93,216.84	84,140.32	93,160.74	366,780.34
NEW MEXICO	20,020.32	18,861.11	18,673.30	18,715.70	76,270.43
NEW YORK	234,957.87	233,012.46	209,800.05	232,310.15	910,080.53
NORTH CAROLINA	205,316.64	189,744.23	175,482.80	191,636.06	762,179.73
NORTH DAKOTA	15,693.46	14,870.97	14,200.42	15,363.74	60,128.59
OHIO	241,069.37	223,831.04	221,728.35	240,266.34	926,895.10
OKLAHOMA	51,387.79	46,391.81	46,687.87	49,045.13	193,512.60
OREGON	62,327.98	63,350.18	58,177.20	62,048.85	245,904.21
PENNSYLVANIA	229,798.99	224,625.98	214,395.79	230,994	899,814.76

PUERTO RICO	22,532.32	17,863.66	14,050.16	13,418.65	67,864.79
RHODE ISLAND	19,040.20	19,549.72	17,663.33	19,046.02	75,299.27
SOUTH CAROLINA	95,241.64	84,944.70	83,046.40	90,539.45	353,772.19
SOUTH DAKOTA	24,985.21	20,180.07	22,968.82	24,851.08	92,985.18
TENNESSEE	88,695.13	80,906.13	80,894.85	85,146.69	335,642.80
TEXAS	359,410.70	333,662.03	305,197.30	338,505.30	1,336,775.33
UTAH	47,750.91	45,267.45	43,816.53	47,326.10	184,160.99
VERMONT	19,251.52	19,375.66	18,661.95	19,638.19	76,927.32
VIRGIN ISLANDS	210	180.55	99.13	107.51	597.19
VIRGINIA	140,383.28	134,905.93	125,005.22	135,489.52	535,783.95
WASHINGTON	113,786.25	114,638.48	105,145.17	113,286.62	446,856.52
WEST VIRGINIA	33,556.50	30,899.27	30,340.53	31,580.48	126,376.78
WISCONSIN	107,110.53	103,041.93	97,436.49	106,075.78	413,664.73
WYOMING	8,742.70	7,947.27	7,794.85	8,091.15	32,575.97
TOTAL	4,801,112.22	4,533,605.90	4,312,060.57	4,663,730	18,310,508.69

DRUG CODE:2012 DRUG NAME:GAMMA HYDROXYBUTYRIC ACID(FDA APPROVED-CSA

STATE	QUARTER 1	QUARTER 2	QUARTER 3	QUARTER 4	TOTAL GRAMS
CALIFORNIA	1,560.38	1,337.47	1,337.47	3,789.50	8,024.82
ILLINOIS	520.13	743.04	668.74	743.04	2,674.95
LOUISIANA	297.22	148.61	0	0	445.83
MASSACHUSETTS	0	1,560.38	297.22	817.34	2,674.94
MICHIGAN	594.43	0	297.22	445.82	1,337.47
MISSOURI	9,221,200.70	5,490,842.69	8,990,338.18	6,709,576.90	30,411,958.47
NEW YORK	0	0	2,972.16	4,978.37	7,950.53
NORTH CAROLINA	222.91	1,411.78	2,006.21	1,337.47	4,978.37
OHIO	1,188.86	2,080.51	1,931.90	2,229.12	7,430.39
SOUTH CAROLINA	965.95	2,749.25	371.52	3,492.29	7,579.01
TENNESSEE	594.43	594.43	594.43	594.43	2,377.72
TEXAS	4,161.02	2,972.16	2,229.12	1,783.30	11,145.60
VIRGINIA	0	2,229.12	1,634.69	2,229.12	6,092.93
WASHINGTON	371.52	817.34	594.43	445.82	2,229.11
TOTAL	9,231,677.55	5,507,486.78	9,005,273.29	6,732,462.52	30,476,900.14

DRUG CODE:2100 DRUG NAME:BARBITURIC ACID DERIVIATIVE OR SALT [PER 21CFR 1

STATE	QUARTER 1	QUARTER 2	QUARTER 3	QUARTER 4	TOTAL GRAMS
ALABAMA	1,865	1,415	1,420	1,445	6,145
ALASKA	225	175	165	150	715
ARIZONA	5,820	5,380	4,530	4,520	20,250
ARKANSAS	1,640	1,335	1,265	1,085	5,325
CALIFORNIA	15,045	14,100	16,110	13,928.50	59,183.50
COLORADO	1,975	1,840	1,440	1,330	6,585
CONNECTICUT	2,160	2,085	2,450	2,015	8,710
DELAWARE	480	380	490	490	1,840
DISTRICT OF COLUMBIA	280	180	285	225	970
FLORIDA	20,135	16,140	17,445	16,125	69,845
GEORGIA	6,285	5,975	4,775	3,990	21,025
GUAM	0	10	0	0	10
HAWAII	770	930	940	740	3,380
IDAHO	640	645	445	435	2,165
ILLINOIS	7,355	7,290	7,975	7,615	30,235
INDIANA	3,425	3,300	3,315	3,120	13,160
IOWA	720	675	650	735	2,780
KANSAS	1,305	1,490	1,030	995	4,820
KENTUCKY	2,060	1,995	1,739	1,465	7,259
LOUISIANA	3,370	3,320	3,785	2,950	13,425
MAINE	480	375	405	430	1,690
MARYLAND	2,610	2,300	2,520	2,110	9,540
MASSACHUSETTS	2,625	2,555	3,185	2,455	10,820
MICHIGAN	6,140	7,125	7,090	6,555	26,910
MINNESOTA	1,390	1,320	1,345	1,260	5,315
MISSISSIPPI	1,200	1,050	1,080	690	4,020
MISSOURI	4,180	4,515	3,910	3,215	15,820
MONTANA	490	540	590	375	1,995
NEBRASKA	715	705	800	750	2,970
NEVADA	5,020	4,835	4,195	4,225	18,275
NEW HAMPSHIRE	485	510	475	525	1,995
NEW JERSEY	4,580	4,205	5,065	4,260	18,110
NEW MEXICO	840	835	860	790	3,325
NEW YORK	4,720	4,390	4,410	4,065	17,585
NORTH CAROLINA	3,875	3,715	4,085	3,590	15,265
NORTH DAKOTA	170	170	125	100	565

OHIO	5,420	5,220	3,425	2,730	16,795
OKLAHOMA	2,665	2,700	3,055	2,835	11,255
OREGON	1,205	1,115	830	715	3,865
PENNSYLVANIA	5,740	5,845	5,560	4,530	21,675
PUERTO RICO	135	150	165	155	605
RHODE ISLAND	635	600	690	595	2,520
SOUTH CAROLINA	2,620	2,205	2,375	2,195	9,395
SOUTH DAKOTA	270	220	225	190	905
TENNESSEE	2,860	2,915	2,880	2,650	11,305
TEXAS	12,555	12,640	13,294.90	10,815	49,304.90
UTAH	1,630	1,710	1,125	1,220	5,685
VERMONT	100	60	85	40	285
VIRGIN ISLANDS	20	45	25	15	105
VIRGINIA	3,670	3,965	3,145	3,120	13,900
WASHINGTON	2,710	2,700	2,130	1,700	9,240
WEST VIRGINIA	665	550	500	405	2,120
WISCONSIN	1,620	1,450	1,750	1,540	6,360
WYOMING	430	395	350	350	1,525
TOTAL	160,025	152,290	152,003.90	134,553.50	598,872.40

DRUG CODE:2125 DRUG NAME:AMOBARBITAL (SCHEDULE 2)

STATE	QUARTER 1	QUARTER 2	QUARTER 3	QUARTER 4	TOTAL GRAMS
ARIZONA	12.76	11.39	12.76	8.2	45.11
ARKANSAS	0	0.46	0.46	0	0.92
CALIFORNIA	27.34	20.96	30.08	22.33	100.71
COLORADO	27.8	15.95	14.58	10.03	68.36
CONNECTICUT	0	2.28	0	1.37	3.65
DISTRICT OF COLUMBIA	0.46	0	1.82	4.56	6.84
FLORIDA	4.1	2.28	8.66	8.2	23.24
GEORGIA	1.82	0.91	10.48	13.22	26.43
ILLINOIS	4.56	0.91	6.84	3.65	15.96
INDIANA	0	0	2.28	1.82	4.1
IOWA	0	0	0.46	0	0.46
KANSAS	1.82	5.01	0.46	6.38	13.67
KENTUCKY	4.56	3.65	3.65	7.29	19.15
MAINE	0	0	0.46	2.28	2.74

MARYLAND	7.29	3.19	6.38	10.48	27.34
MASSACHUSETTS	9.57	1.82	0.91	7.75	20.05
MICHIGAN	5.92	4.1	5.92	2.73	18.67
MINNESOTA	5.01	3.19	7.29	7.75	23.24
MISSISSIPPI	1.82	0	1.37	1.37	4.56
MISSOURI	0.46	0.91	2.73	2.73	6.83
NEBRASKA	2.73	0	0	0.91	3.64
NEW HAMPSHIRE	4.1	1.82	2.73	3.65	12.3
NEW JERSEY	3.65	3.19	7.29	16.41	30.54
NEW YORK	54.68	21.87	26.89	49.22	152.66
NORTH CAROLINA	16.41	5.01	5.47	11.39	38.28
OHIO	3.19	3.65	5.47	5.47	17.78
OREGON	1.37	1.37	1.37	2.73	6.84
PENNSYLVANIA	13.67	2.73	4.1	8.66	29.16
RHODE ISLAND	3.65	0	0	0	3.65
SOUTH CAROLINA	1.37	4.1	5.47	4.1	15.04
TENNESSEE	4.1	5.01	5.01	1.82	15.94
TEXAS	25.52	13.67	27.8	16.41	83.4
UTAH	1.82	13.67	14.58	4.1	34.17
VERMONT	0	0	0.91	0	0.91
VIRGINIA	3.65	2.73	2.73	2.28	11.39
WASHINGTON	4.56	0	11.39	20.96	36.91
WEST VIRGINIA	0	0	0	1.82	1.82
WISCONSIN	6.38	3.19	7.29	10.03	26.89
TOTAL	266.14	159.02	246.09	282.1	953.35

DRUG CODE:2165 DRUG NAME:BUTALBITAL

STATE	QUARTER 1	QUARTER 2	QUARTER 3	QUARTER 4	TOTAL GRAMS
ALABAMA	4,415	4,745	4,615	4,025	17,800
ALASKA	180	265	275	205	925
AMERICAN SAMOA	10	0	0	0	10
ARIZONA	4,710	4,705	5,495	5,625	20,535
ARKANSAS	3,295	3,435	3,520	3,515	13,765
CALIFORNIA	24,630	24,940	25,060	23,680	98,310
COLORADO	1,715	1,835	1,985	2,275	7,810
CONNECTICUT	3,255	3,040	3,350	2,655	12,300

DELAWARE	560	550	495	505	2,110
DISTRICT OF COLUMBIA	315	350	395	340	1,400
FLORIDA	14,835	16,625	17,215	17,045	65,720
GEORGIA	9,170	9,660	10,605	10,365	39,800
GUAM	25	5	5	5	40
HAWAII	1,110	1,075	1,045	930	4,160
IDAHO	1,180	1,240	1,430	1,395	5,245
ILLINOIS	7,300	7,740	7,450	7,170	29,660
INDIANA	3,810	3,680	4,190	3,915	15,595
IOWA	1,230	1,290	1,335	1,245	5,100
KANSAS	2,220	1,875	2,580	2,130	8,805
KENTUCKY	2,385	2,410	2,830	2,740	10,365
LOUISIANA	4,150	4,050	3,835	3,715	15,750
MAINE	750	635	610	525	2,520
MARYLAND	4,850	4,490	4,895	4,755	18,990
MASSACHUSETTS	2,530	2,405	2,350	2,015	9,300
MICHIGAN	9,265	8,495	8,940	8,820	35,520
MINNESOTA	1,535	1,640	1,595	1,935	6,705
MISSISSIPPI	2,130	2,130	2,185	2,100	8,545
MISSOURI	5,405	5,725	6,105	6,515	23,750
MONTANA	735	770	880	695	3,080
NEBRASKA	1,050	1,080	1,105	1,200	4,435
NEVADA	3,435	3,420	4,025	3,935	14,815
NEW HAMPSHIRE	625	615	710	715	2,665
NEW JERSEY	6,570	6,190	6,305	5,605	24,670
NEW MEXICO	820	1,010	995	875	3,700
NEW YORK	5,965	6,075	5,765	4,970	22,775
NORTH CAROLINA	5,140	5,030	5,105	5,115	20,390
NORTH DAKOTA	310	335	305	335	1,285
OHIO	5,915	5,455	7,495	7,765	26,630
OKLAHOMA	5,445	5,275	4,825	4,445	19,990
OREGON	2,370	2,075	2,390	2,550	9,385
PENNSYLVANIA	12,020	11,445	12,105	11,230	46,800
RHODE ISLAND	375	415	360	320	1,470
SOUTH CAROLINA	3,295	3,405	3,430	3,350	13,480
SOUTH DAKOTA	660	635	650	650	2,595
TENNESSEE	3,530	3,625	4,025	4,085	15,265

TEXAS	18,785	18,150	18,445	19,415	74,795
UTAH	3,055	2,735	3,430	3,500	12,720
VERMONT	150	85	95	140	470
VIRGIN ISLANDS	0	0	20	25	45
VIRGINIA	5,545	5,155	5,165	5,005	20,870
WASHINGTON	3,765	4,230	4,445	4,965	17,405
WEST VIRGINIA	1,215	1,240	1,355	1,260	5,070
WISCONSIN	1,670	1,745	1,940	1,640	6,995
WYOMING	340	375	470	445	1,630
TOTAL	209,750	209,605	220,230	214,380	853,965

DRUG CODE:2270 DRUG NAME:PENTOBARBITAL (SCHEDULE 2)

STATE	QUARTER 1	QUARTER 2	QUARTER 3	QUARTER 4	TOTAL GRAMS
ALABAMA	11,145.13	14,500.33	22,368.20	17,579.89	65,593.55
ALASKA	5,687.14	4,996.29	6,220.31	5,887.64	22,791.38
ARIZONA	44,885.44	44,597.77	56,444.25	69,138.71	215,066.17
ARKANSAS	7,552.68	13,890.83	8,916.40	6,067.28	36,427.19
CALIFORNIA	173,418.97	173,257.94	189,476.46	172,640.31	708,793.68
COLORADO	47,273.77	38,582.56	33,020.93	60,578.29	179,455.55
CONNECTICUT	14,888.95	14,529.60	21,462.52	21,570.99	72,452.06
DELAWARE	787.45	1,976.09	1,709.50	2,675.85	7,148.89
DISTRICT OF COLUMBIA	195.95	9.11	871.3	1,476.47	2,552.83
FLORIDA	65,115.01	76,163.21	70,442.04	72,885.89	284,606.15
GEORGIA	37,024.55	33,886.33	38,688.78	44,214.69	153,814.35
GUAM	0	3,215.42	0	3,215.42	6,430.84
HAWAII	4,631.28	1,230.39	17,224.55	4,857.76	27,943.98
IDAHO	16,141.35	20,099.10	28,465.10	25,743.86	90,449.41
ILLINOIS	56,158.15	60,069.08	59,541.08	101,493.52	277,261.83
INDIANA	41,277.70	38,050.43	43,439.08	57,017.45	179,784.66
IOWA	11,284.04	16,575.81	14,194.51	29,448.79	71,503.15
KANSAS	21,721.40	14,447.51	21,334.96	32,986.30	90,490.17
KENTUCKY	20,477	22,887.26	35,235.21	32,743.94	111,343.41
LOUISIANA	24,820.76	21,484.63	27,264.59	26,765.45	100,335.43
MAINE	21,326.21	17,117	19,406.17	23,031.16	80,880.54
MARYLAND	23,025.95	14,663.50	23,838.64	27,402.48	88,930.57
MASSACHUSETTS	21,722.39	26,302.18	31,710.33	33,023.51	112,758.41

MICHIGAN	89,535.30	78,113.67	110,086.02	107,206.29	384,941.28
MINNESOTA	26,227.77	36,097.90	31,190.18	51,363.94	144,879.79
MISSISSIPPI	11,126.08	5,525.17	6,281.72	9,590.44	32,523.41
MISSOURI	36,952.86	26,105.75	47,154.64	50,814.53	161,027.78
MONTANA	12,442.79	10,492.04	21,509.95	29,680.65	74,125.43
NEBRASKA	12,527.65	19,068.77	20,501.94	30,369.58	82,467.94
NEVADA	12,224.69	11,557.92	18,280.86	19,692.25	61,755.72
NEW HAMPSHIRE	11,871.16	16,646.96	16,792.07	16,615.07	61,925.26
NEW JERSEY	18,830.01	15,715.07	26,327.21	21,045.85	81,918.14
NEW MEXICO	13,888.18	18,120.85	26,506.79	21,787.28	80,303.10
NEW YORK	56,153.83	76,688.34	83,875.93	89,677.25	306,395.35
NORTH CAROLINA	51,212.01	55,633.78	60,744.81	74,943.77	242,534.37
NORTH DAKOTA	8,726.66	6,497.83	4,790.77	8,352.98	28,368.24
OHIO	59,844.72	65,744.16	78,226.93	91,305.99	295,121.80
OKLAHOMA	13,683.85	21,513.21	14,100.72	26,814.93	76,112.71
OREGON	28,973.41	35,321.39	43,992.82	46,399.10	154,686.72
PENNSYLVANIA	53,524.62	63,201.28	87,707.74	64,757.20	269,190.84
PUERTO RICO	0	0	0.2	0	0.2
RHODE ISLAND	3,207.67	6,945.87	7,290.47	7,118.40	24,562.41
SOUTH CAROLINA	12,579.37	12,854.39	13,920.90	16,360.09	55,714.75
SOUTH DAKOTA	5,781.01	5,426.02	6,676.01	7,109.83	24,992.87
TENNESSEE	26,638.02	41,338.06	35,204.43	39,413	142,593.51
TEXAS	105,413.74	107,956.51	142,377.96	140,844.93	496,593.14
UTAH	9,644.89	11,271.74	13,304.40	22,693.86	56,914.89
VERMONT	9,795.63	9,364.52	17,431.72	17,770.38	54,362.25
VIRGINIA	42,855.88	47,223.42	47,155	53,755.62	190,989.92
WASHINGTON	55,558.36	53,612.17	57,017.68	74,631.70	240,819.91
WEST VIRGINIA	6,970.84	12,573.12	14,259.29	8,298.29	42,101.54
WISCONSIN	40,971.17	43,561.15	52,120.04	72,768.15	209,420.51
WYOMING	4,531.94	12,623.80	9,096.68	14,217.84	40,470.26
TOTAL	1,512,255.38	1,599,327.23	1,885,200.79	2,107,844.84	7,104,628.24

DRUG CODE:2315 DRUG NAME:SECOBARBITAL (SCHEDULE 2)

STATE	QUARTER 1	QUARTER 2	QUARTER 3	QUARTER 4	TOTAL GRAMS
ARIZONA	9.16	0	0	9.16	18.32
CALIFORNIA	897.19	988.74	1,162.69	1,327.48	4,376.10

COLORADO	109.86	91.55	109.86	64.09	375.36
CONNECTICUT	36.62	27.47	9.16	18.31	91.56
DELAWARE	0	9.16	9.16	9.16	27.48
FLORIDA	100.71	73.24	100.71	91.55	366.21
GEORGIA	36.62	9.16	0	0	45.78
HAWAII	9.16	0	0	0	9.16
ILLINOIS	45.78	54.93	36.62	27.47	164.8
KANSAS	36.62	18.31	18.31	27.47	100.71
MARYLAND	64.09	64.09	45.78	45.78	219.74
MASSACHUSETTS	54.93	45.78	36.62	54.93	192.26
MICHIGAN	0	0	0	18.31	18.31
MINNESOTA	9.16	0	0	9.16	18.32
MISSOURI	27.47	18.31	36.62	18.31	100.71
NEVADA	9.16	0	0	0	9.16
NEW HAMPSHIRE	18.31	9.16	0	0	27.47
NEW JERSEY	73.24	54.93	54.93	45.78	228.88
NEW YORK	466.91	402.82	292.96	338.74	1,501.43
NORTH CAROLINA	0	9.16	0	0	9.16
OHIO	54.93	18.31	27.47	36.62	137.33
OKLAHOMA	0	0	27.47	9.16	36.63
OREGON	256.34	173.95	219.72	302.12	952.13
PENNSYLVANIA	36.62	73.24	82.4	73.24	265.5
RHODE ISLAND	9.16	9.16	0	18.31	36.63
SOUTH CAROLINA	0	0	18.31	18.31	36.62
SOUTH DAKOTA	0	0	0	9.16	9.16
TENNESSEE	0	9.16	0	0	9.16
TEXAS	9.16	0	9.16	27.47	45.79
VERMONT	54.93	27.47	0	54.93	137.33
VIRGINIA	27.47	27.47	27.47	27.47	109.88
WASHINGTON	228.88	173.95	155.64	164.79	723.26
WISCONSIN	9.16	0	0	0	9.16
TOTAL	2,691.64	2,389.52	2,481.06	2,847.28	10,409.50

DRUG CODE:7365 DRUG NAME:DRONABINOL IN AN ORAL SOLUTION IN FDA APPROVE

STATE	QUARTER 1	QUARTER 2	QUARTER 3	QUARTER 4	TOTAL GRAMS
ALABAMA	0	0	1.5	2.7	4.2

ARIZONA	0	0	14.4	7.2	21.6
ARKANSAS	0	0	0.6	0.6	1.2
CALIFORNIA	0	0	15.15	28.65	43.8
COLORADO	0	0	3.6	0.15	3.75
CONNECTICUT	0	0	1.2	0.45	1.65
DELAWARE	0	0	0.15	0	0.15
DISTRICT OF COLUMBIA	0	0	0	0.75	0.75
FLORIDA	0	0	13.95	22.65	36.6
GEORGIA	0	0	1.35	3.3	4.65
ILLINOIS	0	0	0.3	0.75	1.05
INDIANA	0	0	1.65	0	1.65
KANSAS	0	0	0	0.6	0.6
KENTUCKY	0	0	0.75	0.75	1.5
LOUISIANA	0	0	0.15	0.15	0.3
MAINE	0	0	0.3	0	0.3
MARYLAND	0	0	0.15	1.8	1.95
MASSACHUSETTS	0	0	0.75	3	3.75
MICHIGAN	0	0	1.05	1.35	2.4
MINNESOTA	0	0	0	2.85	2.85
MISSISSIPPI	0	0	0.6	0.15	0.75
MISSOURI	0	0	4.35	4.2	8.55
MONTANA	0	0	0	0.3	0.3
NEVADA	0	0	4.95	6.6	11.55
NEW HAMPSHIRE	0	0	0.75	1.05	1.8
NEW JERSEY	0	0	70.8	72.75	143.55
NEW MEXICO	0	0	0.15	0	0.15
NEW YORK	0	0	0.75	1.2	1.95
NORTH CAROLINA	0	0	0.45	0.6	1.05
OHIO	0	0	0.45	8.25	8.7
PENNSYLVANIA	0	0	14.4	1.95	16.35
SOUTH CAROLINA	0	0	0.3	0	0.3
TENNESSEE	0	0	1.5	2.1	3.6
TEXAS	0	0	6.6	5.1	11.7
UTAH	0	0	0.45	0.15	0.6
VIRGINIA	0	0	0	0.75	0.75
WISCONSIN	0	0	0.15	0	0.15
TOTAL	0	0	163.65	182.85	346.5

DRUG CODE:7379 DRUG NAME:NABILONE

STATE	QUARTER 1	QUARTER 2	QUARTER 3	QUARTER 4	TOTAL GRAMS
ARIZONA	0	0	0.15	0.05	0.2
CALIFORNIA	0	0.25	0.05	0.35	0.65
COLORADO	0.15	0.05	0	0.15	0.35
CONNECTICUT	0.1	0.15	0.1	0	0.35
FLORIDA	1.05	0.45	0.45	0.2	2.15
HAWAII	0.45	0	0.15	0	0.6
ILLINOIS	0.25	0.05	0.1	0.1	0.5
IOWA	0.05	0.05	0	0	0.1
KENTUCKY	0.15	0.2	0	0	0.35
MAINE	0.1	0.1	0.1	0	0.3
MARYLAND	1.05	1.1	1.05	0.9	4.1
MINNESOTA	0.1	0	0	0	0.1
MISSOURI	0.2	0.1	0.1	0.15	0.55
NEBRASKA	0.15	0	0	0.15	0.3
NEW HAMPSHIRE	0	0	0	0.05	0.05
NEW JERSEY	0.1	0.15	0.1	0	0.35
NEW MEXICO	0	0	0.1	0	0.1
NEW YORK	0.2	0.15	0.05	0	0.4
NORTH CAROLINA	0.2	0.15	0.15	0.25	0.75
OHIO	0.1	0.1	0.1	0.7	1
OREGON	0	0	0	0.05	0.05
PENNSYLVANIA	0.05	0.05	0.15	0.1	0.35
VIRGINIA	0.2	0.35	0.2	0	0.75
WASHINGTON	0	0	0	0.05	0.05
TOTAL	4.65	3.45	3.1	3.25	14.45

DRUG CODE:9041L DRUG NAME:COCAINE

STATE	QUARTER 1	QUARTER 2	QUARTER 3	QUARTER 4	TOTAL GRAMS
ALABAMA	62.42	65.85	49.85	50.57	228.69
ALASKA	8.57	19.78	9.57	10	47.92
ARIZONA	110.49	109.67	99.57	99.85	419.58
ARKANSAS	56.32	40.93	38.18	40.18	175.61

CALIFORNIA	927.62	879.09	835.77	828.09	3,470.57
COLORADO	82.1	109.17	71.21	80.57	343.05
CONNECTICUT	39.07	32.28	50.14	29.43	150.92
DELAWARE	6.57	18.86	14.86	11.57	51.86
DISTRICT OF COLUMBIA	22.53	21.46	28.14	20.75	92.88
FLORIDA	289.16	306.77	294.66	271.52	1,162.11
GEORGIA	154.45	171.17	147.13	184.13	656.88
GUAM	0	0	0.36	0	0.36
HAWAII	28.28	23.86	24.86	20.43	97.43
IDAHO	64.6	37.43	46.85	67.67	216.55
ILLINOIS	326.62	356.83	272.7	333.98	1,290.13
INDIANA	251.13	195.74	153.13	200.92	800.92
IOWA	105.42	102.85	86.1	98.64	393.01
KANSAS	63.53	84.89	82.32	97.89	328.63
KENTUCKY	109.89	117.89	88.49	118.14	434.41
LOUISIANA	92.32	96.71	86.71	113.71	389.45
MAINE	22.43	24.93	22.86	17.21	87.43
MARYLAND	140.78	146.6	119.53	110.67	517.58
MASSACHUSETTS	228.41	202.7	180.56	203.77	815.44
MICHIGAN	367.55	422.15	336.59	381.58	1,507.87
MINNESOTA	211.52	233.59	199.24	211.17	855.52
MISSISSIPPI	79.67	101.53	75.25	82.53	338.98
MISSOURI	164.2	180.88	159.92	154.56	659.56
MONTANA	48.85	55	41.71	66.03	211.59
NEBRASKA	21.57	31	37.03	29.57	119.17
NEVADA	44.6	52.1	43.93	39.32	179.95
NEW HAMPSHIRE	38.71	54.89	40.57	56.14	190.31
NEW JERSEY	176.02	168.7	148.88	152.13	645.73
NEW MEXICO	76.35	73.71	73.78	66.57	290.41
NEW YORK	361.8	371.01	319.55	327.51	1,379.87
NORTH CAROLINA	135.53	141.67	151.95	142.53	571.68
NORTH DAKOTA	33.25	66.39	37.82	44.53	181.99
OHIO	175.49	202.02	170.02	183.24	730.77
OKLAHOMA	31.28	34	28.14	39.57	132.99
OREGON	103.46	95.92	100.35	84.82	384.55
PENNSYLVANIA	412.69	410.15	356.41	377.48	1,556.73
RHODE ISLAND	25.28	16.43	17.43	16.86	76

SOUTH CAROLINA	30.61	31.57	28	28.14	118.32
SOUTH DAKOTA	44.25	24.78	27.46	52.35	148.84
TENNESSEE	181.04	160.13	159.7	169.13	670
TEXAS	332.69	384.69	326.62	409.69	1,453.69
UTAH	103.28	98.6	89.92	99.21	391.01
VERMONT	10.36	13.46	6.29	8.36	38.47
VIRGINIA	161.85	153.17	154.88	144.63	614.53
WASHINGTON	114.46	104.17	124.42	111.03	454.08
WEST VIRGINIA	52.53	48.35	49.89	51.03	201.8
WISCONSIN	164.42	158.2	130.63	151.56	604.81
WYOMING	18	13.86	20.28	17.14	69.28
TOTAL	6,914.02	7,067.58	6,260.21	6,708.10	26,949.91

DRUG CODE:9050 DRUG NAME:CODEINE

STATE	QUARTER 1	QUARTER 2	QUARTER 3	QUARTER 4	TOTAL GRAMS
ALABAMA	52,097.22	52,426.61	51,821.15	52,546.65	208,891.63
ALASKA	7,416.42	7,072.30	7,040.49	6,268.26	27,797.47
AMERICAN SAMOA	353.62	340.36	353.62	313.83	1,361.43
ARIZONA	78,396.32	75,054.50	70,599.31	73,098.41	297,148.54
ARKANSAS	74,120.24	76,464.28	71,973.79	70,250.71	292,809.02
CALIFORNIA	501,442.60	479,948.70	460,547.34	449,781.78	1,891,720.42
COLORADO	26,553.20	25,891.58	25,424.45	24,999.62	102,868.85
CONNECTICUT	24,542.20	24,979.42	23,678.88	23,115.66	96,316.16
DELAWARE	9,906.43	9,523.85	8,721.58	8,835.22	36,987.08
DISTRICT OF COLUMBIA	12,746.29	12,577.52	12,006.52	11,090.12	48,420.45
FLORIDA	180,686.49	170,836.92	161,724.52	168,139.52	681,387.45
GEORGIA	106,357.27	109,274.90	102,164.73	102,467.93	420,264.83
GUAM	1,289.01	1,191.33	1,196.49	1,088.84	4,765.67
HAWAII	13,807.53	14,179.60	13,766.47	12,809.15	54,562.75
IDAHO	13,779.75	13,988.86	13,595.78	13,279.28	54,643.67
ILLINOIS	167,295.96	163,981.42	159,064.67	156,936.08	647,278.13
INDIANA	52,138.19	52,017.41	47,484.82	48,824.93	200,465.35
IOWA	20,061.94	20,404.12	19,724.99	19,705.10	79,896.15
KANSAS	28,997.24	28,439.53	26,788.02	27,571.11	111,795.90
KENTUCKY	48,676.44	48,234.28	47,573.05	45,644.01	190,127.78
LOUISIANA	45,273.75	45,593.29	42,084.80	40,843.81	173,795.65

MAINE	10,829.90	11,007.78	9,775.47	9,942.97	41,556.12
MARYLAND	62,160.93	59,591.10	55,355.33	54,820.13	231,927.49
MASSACHUSETTS	48,661.15	46,733.29	45,133.33	44,702.33	185,230.10
MICHIGAN	265,875.99	262,963.95	254,929.37	241,505.52	1,025,274.83
MINNESOTA	38,915.01	37,129.43	37,001.21	36,152.95	149,198.60
MISSISSIPPI	28,067.59	27,569.65	27,316.55	26,659.58	109,613.37
MISSOURI	68,652.99	70,089.01	67,023.53	68,032.84	273,798.37
MONTANA	10,274.37	10,509.37	9,893.81	9,903.40	40,580.95
NEBRASKA	12,174.42	11,764.41	11,642.48	11,383.96	46,965.27
NEVADA	27,025.90	27,451.42	25,820.24	26,651.52	106,949.08
NEW HAMPSHIRE	7,881.23	7,544.36	7,327.35	7,594.42	30,347.36
NEW JERSEY	82,192.68	78,830.99	75,755.85	74,034.34	310,813.86
NEW MEXICO	19,825.21	19,248.02	19,527.98	19,585.79	78,187
NEW YORK	147,404.26	145,569.93	138,625.64	136,150.10	567,749.93
NORTH CAROLINA	51,512.39	48,995.17	46,149.79	46,959.82	193,617.17
NORTH DAKOTA	5,752.54	5,818.55	5,861.92	5,345.38	22,778.39
OHIO	109,027.91	102,725.01	96,703.69	94,549.37	403,005.98
OKLAHOMA	96,652.50	96,862.79	94,633.62	93,769.52	381,918.43
OREGON	35,126.69	33,710.18	33,479.03	33,544.33	135,860.23
PENNSYLVANIA	137,000.07	143,911.20	142,003.02	131,550.66	554,464.95
PUERTO RICO	13,584.38	13,474.98	11,629.55	12,536.79	51,225.70
RHODE ISLAND	8,007.21	7,373.78	6,873.92	6,861.83	29,116.74
SOUTH CAROLINA	78,535.31	68,293.22	72,571.49	84,635.97	304,035.99
SOUTH DAKOTA	7,250.21	7,076.52	6,735.71	6,822.74	27,885.18
TENNESSEE	93,521.32	88,402.47	85,408.29	74,152.05	341,484.13
TEXAS	1,009,935.10	1,024,743.49	987,072.94	985,807.44	4,007,558.97
UTAH	16,455.49	16,583.15	16,378.36	15,954.67	65,371.67
VERMONT	3,565.55	3,458.76	3,031.04	3,060.57	13,115.92
VIRGIN ISLANDS	950.85	652.13	565.79	830.26	2,999.03
VIRGINIA	59,288.95	57,032.64	51,403.11	51,851.65	219,576.35
WASHINGTON	63,371.45	63,059.58	59,985.80	59,332.65	245,749.48
WEST VIRGINIA	31,085.46	31,132.91	30,260.45	31,982.24	124,461.06
WISCONSIN	50,699.55	47,765.40	45,918.70	46,170.52	190,554.17
WYOMING	4,366.56	4,031.10	4,127.83	4,240.97	16,766.46
TOTAL	4,171,569.23	4,113,526.52	3,953,257.66	3,914,689.30	16,153,042.71

RETAIL DRUG DISTRIBUTION BY STATE WITHIN DRUG CODE BY GRAMS WT

DRUG CODE:9056 DRUG NAME:ETORPHINE

STATE	QUARTER 1	QUARTER 2	QUARTER 3	QUARTER 4	TOTAL GRAMS
WYOMING	28	0	0	29	57
TOTAL	28	0	0	29	57

DRUG CODE:9064 DRUG NAME:BUPRENORPHINE

STATE	QUARTER 1	QUARTER 2	QUARTER 3	QUARTER 4	TOTAL GRAMS
ALABAMA	23,735.15	25,160.56	25,414	26,445.37	100,755.08
ALASKA	2,178.38	2,351.56	2,431.40	2,496.82	9,458.16
AMERICAN SAMOA	0	0	0	0	0
ARIZONA	8,917.41	9,288.38	9,567.44	9,927.03	37,700.26
ARKANSAS	5,876.84	6,179.83	6,370.91	6,628.80	25,056.38
CALIFORNIA	36,183.89	37,274.64	39,297.69	39,811.60	152,567.82
COLORADO	7,029.58	7,245.65	7,617.76	7,720.27	29,613.26
CONNECTICUT	14,144.45	14,772.20	15,022.64	15,353.47	59,292.76
DELAWARE	3,383.64	3,633.81	3,599.52	3,898.94	14,515.91
DISTRICT OF COLUMBIA	2,126.56	2,265.05	2,463.07	2,599.65	9,454.33
FLORIDA	45,362.39	46,836.59	46,526.79	48,926.92	187,652.69
GEORGIA	14,877.07	15,748.96	15,896.57	16,348.80	62,871.40
GUAM	0.82	1.99	0	1.68	4.49
HAWAII	1,860.94	1,987.21	2,012.85	2,018.34	7,879.34
IDAHO	3,253.87	3,455.24	3,631.94	3,874.99	14,216.04
ILLINOIS	13,435.24	14,379.93	14,668.92	15,043.80	57,527.89
INDIANA	17,803.76	19,775.16	20,537.14	21,528.68	79,644.74
IOWA	1,585.44	1,638.21	1,699.13	1,807.58	6,730.36
KANSAS	2,494.01	2,507.15	2,660.73	2,561.66	10,223.55
KENTUCKY	33,748.36	34,750.20	34,645.38	34,938.66	138,082.60
LOUISIANA	18,054.67	18,953.53	19,682.99	19,622.81	76,314
MAINE	8,971.92	9,673.06	9,873.65	10,201.77	38,720.40
MARYLAND	24,125.10	25,266.60	25,673.14	26,997.92	102,062.76
MASSACHUSETTS	39,805.50	42,539.51	40,755.78	42,212.58	165,313.37
MICHIGAN	25,710.86	27,064.52	27,236.37	28,632.85	108,644.60
MINNESOTA	6,999.44	7,444.88	7,748.16	8,022.26	30,214.74
MISSISSIPPI	9,689.76	9,995.82	9,962.33	10,024.20	39,672.11
MISSOURI	8,630.87	9,208.38	9,704.05	10,683.37	38,226.67
MONTANA	2,220.35	2,315.94	2,495.93	2,619.94	9,652.16

NEBRASKA	1,264.53	1,308.52	1,363.58	1,372.23	5,308.86
NEVADA	3,238.79	3,476.56	3,461.23	3,468.65	13,645.23
NEW HAMPSHIRE	7,670.59	8,058.64	8,213.44	8,150.59	32,093.26
NEW JERSEY	25,547.24	26,921.80	26,147.03	26,936.73	105,552.80
NEW MEXICO	6,825.93	7,233.94	7,598.72	7,837.01	29,495.60
NEW YORK	50,488.07	52,804.61	53,572.20	56,492.76	213,357.64
NORTH CAROLINA	30,365.22	33,074.64	32,092.39	35,309.84	130,842.09
NORTH DAKOTA	851.95	883.13	943.98	956.29	3,635.35
OHIO	50,757.75	53,154.23	52,666.30	53,520.61	210,098.89
OKLAHOMA	10,175.80	10,362.32	11,514.45	11,668.33	43,720.90
OREGON	9,724.64	10,278.46	10,437.33	11,096.05	41,536.48
PENNSYLVANIA	60,331.80	63,245.33	62,191.87	66,318.71	252,087.71
PUERTO RICO	2,492.26	2,570.74	2,219.65	2,673.68	9,956.33
RHODE ISLAND	7,078.72	7,701.37	7,489.74	7,827.47	30,097.30
SOUTH CAROLINA	9,844.56	10,500.67	10,795.35	11,498.19	42,638.77
SOUTH DAKOTA	410.6	465.16	449.56	472.47	1,797.79
TENNESSEE	33,071.70	35,153.80	34,738.83	36,321.42	139,285.75
TEXAS	25,480.77	26,211.78	26,795.05	26,848.72	105,336.32
UTAH	9,297.97	9,593.42	9,762.65	9,908.39	38,562.43
VERMONT	5,971.04	6,401.46	6,346.14	6,500.42	25,219.06
VIRGIN ISLANDS	19.68	17.54	12.98	12.51	62.71
VIRGINIA	20,212.32	20,113.19	19,190.64	19,942.50	79,458.65
WASHINGTON	17,982.44	18,967.40	19,553.76	20,495.88	76,999.48
WEST VIRGINIA	14,898.04	15,508.03	15,972.70	15,811.79	62,190.56
WISCONSIN	9,883.10	10,182.04	10,272.80	10,766.41	41,104.35
WYOMING	1,002.54	1,256.80	1,317.04	1,456.51	5,032.89
TOTAL	797,094.32	837,160.14	842,315.69	874,614.92	3,351,185.07

DRUG CODE:9120 DRUG NAME:DIHYDROCODEINE

STATE	QUARTER 1	QUARTER 2	QUARTER 3	QUARTER 4	TOTAL GRAMS
ALABAMA	783.7	798.47	785.72	781.48	3,149.37
ALASKA	8.55	3.2	0	2.14	13.89
ARIZONA	73.81	86.52	75.84	83.32	319.49
ARKANSAS	105.67	152.34	175.48	135.07	568.56
CALIFORNIA	214.7	252.3	73.83	302.29	843.12
COLORADO	0	2.14	5.34	2.14	9.62

CONNECTICUT	1.2	0.13	2.42	0	3.75
DELAWARE	13.14	13.25	12.43	15.7	54.52
FLORIDA	158.82	147.29	238.7	219.11	763.92
GEORGIA	428.39	549.78	509.62	710.23	2,198.02
HAWAII	1.07	0	0	0	1.07
IDAHO	23.5	40.59	32.04	12.82	108.95
ILLINOIS	126.9	214.49	192.06	137.66	671.11
INDIANA	3.2	0	3.2	1.07	7.47
IOWA	0	1.07	0	1.07	2.14
KANSAS	1.07	10.68	0	6.41	18.16
KENTUCKY	30.98	19.28	13.89	21.36	85.51
LOUISIANA	620.96	802.73	644.22	484.55	2,552.46
MAINE	0	0	1.07	0	1.07
MARYLAND	7.48	10.15	10.31	10.15	38.09
MASSACHUSETTS	21.36	16.02	33.11	55.54	126.03
MICHIGAN	34.23	0	5.34	1.28	40.85
MINNESOTA	1.71	4.27	1.71	2.14	9.83
MISSISSIPPI	367.55	491.51	380.32	366.59	1,605.97
MISSOURI	45.93	42.73	54.48	41.66	184.8
MONTANA	1.07	0	0	0	1.07
NEBRASKA	3.2	2.14	3.85	2.78	11.97
NEVADA	43.37	30.98	1.07	0	75.42
NEW JERSEY	355.87	369.38	237.79	285.71	1,248.75
NEW MEXICO	135.66	90.79	100.41	17.09	343.95
NEW YORK	108.95	167.7	116.43	136.19	529.27
NORTH CAROLINA	13.89	9.61	6.41	8.55	38.46
OHIO	48.01	33.81	37.92	20.56	140.3
OKLAHOMA	272.81	298.86	295.29	262.41	1,129.37
PENNSYLVANIA	205.58	42.12	64.53	50.33	362.56
RHODE ISLAND	0	1.07	0	2.14	3.21
SOUTH CAROLINA	13.89	13.89	10.68	13.89	52.35
SOUTH DAKOTA	0	0	0	1.07	1.07
TENNESSEE	507.39	516.62	508.84	404.19	1,937.04
TEXAS	2,687.78	2,808.14	2,679.34	2,853.92	11,029.18
UTAH	0	0	1.07	0	1.07
VERMONT	0	1.07	0	0	1.07
VIRGINIA	10.68	4.27	3.2	8.55	26.7

WEST VIRGINIA	5.34	2.35	0	0	7.69
WISCONSIN	4.27	7.48	6.41	3.2	21.36
TOTAL	7,491.68	8,059.22	7,324.37	7,464.36	30,339.63

DRUG CODE:9143 DRUG NAME:OXYCODONE

STATE	QUARTER 1	QUARTER 2	QUARTER 3	QUARTER 4	TOTAL GRAMS
ALABAMA	245,034.14	234,086.14	222,837.98	221,282.88	923,241.14
ALASKA	35,909.96	35,106.79	33,743.86	33,035.01	137,795.62
AMERICAN SAMOA	14.34	12.1	10.76	21.52	58.72
ARIZONA	459,311.04	432,919.49	408,276.05	399,895.18	1,700,401.76
ARKANSAS	150,503.65	147,187.43	142,600.16	137,696.38	577,987.62
CALIFORNIA	905,318.77	878,899.08	848,801.57	819,812.84	3,452,832.26
COLORADO	233,553.88	228,253.27	217,544.66	213,255.23	892,607.04
CONNECTICUT	175,414.04	170,580.13	160,443.37	157,023.12	663,460.66
DELAWARE	72,059.47	68,623.07	66,755.23	66,369.80	273,807.57
DISTRICT OF COLUMBIA	15,113.21	15,042.95	14,559.95	13,439.86	58,155.97
FLORIDA	1,040,636.86	1,029,362.72	996,363.76	1,037,706.14	4,104,069.48
GEORGIA	455,523.47	451,529.04	439,524.11	436,048.64	1,782,625.26
GUAM	697.48	908.57	980.23	835.09	3,421.37
HAWAII	45,607.42	44,026.24	43,218.01	40,579.06	173,430.73
IDAHO	61,459.28	60,628.06	60,258.22	59,007.70	241,353.26
ILLINOIS	165,648.45	160,662.93	154,175.18	152,126.92	632,613.48
INDIANA	241,577.26	236,466.08	224,779.73	221,485.13	924,308.20
IOWA	56,046.72	54,232.73	51,301.26	52,242.96	213,823.67
KANSAS	124,474.40	123,720.45	121,222.15	119,080.42	488,497.42
KENTUCKY	210,690.77	206,737.28	199,383.42	198,870.30	815,681.77
LOUISIANA	203,678.58	196,046.31	187,743.37	182,770.76	770,239.02
MAINE	54,161.13	51,039.90	50,058.51	49,048.14	204,307.68
MARYLAND	343,393.91	332,414.30	312,941.46	306,627.83	1,295,377.50
MASSACHUSETTS	253,125.08	244,621.64	233,176.98	228,413.72	959,337.42
MICHIGAN	314,155.93	303,147.88	292,409.66	279,116.29	1,188,829.76
MINNESOTA	149,851.01	144,230.88	139,462.99	137,088.75	570,633.63
MISSISSIPPI	109,326.39	106,944.69	102,515.30	100,468.18	419,254.56
MISSOURI	309,827.79	305,484.02	288,966.90	280,390.36	1,184,669.07
MONTANA	41,009.46	38,376.24	37,194.59	35,770.94	152,351.23
NEBRASKA	42,876.54	42,209.02	40,908.67	40,254.56	166,248.79

NEVADA	186,303.46	181,957.10	175,545.89	173,484.57	717,291.02
NEW HAMPSHIRE	65,642.96	62,820.01	62,066.27	58,851.59	249,380.83
NEW JERSEY	539,098.15	511,306.71	480,020.01	460,887.38	1,991,312.25
NEW MEXICO	98,671.82	92,617.85	86,384.43	83,715.95	361,390.05
NEW YORK	786,761.65	754,995.06	718,432.45	691,526.96	2,951,716.12
NORTH CAROLINA	598,268.67	570,304.32	540,337.25	530,163.25	2,239,073.49
NORTH DAKOTA	15,163.44	14,961.70	14,285.01	14,179.56	58,589.71
OHIO	515,301.72	494,056.29	469,035.01	448,137.18	1,926,530.20
OKLAHOMA	231,863.07	228,171.70	220,954.32	215,753.47	896,742.56
OREGON	183,165.09	176,788.51	169,523.38	163,860.60	693,337.58
PENNSYLVANIA	786,642.17	763,586.57	733,656.61	718,182.30	3,002,067.65
PUERTO RICO	19,771.46	14,068.91	9,392.23	7,493.92	50,726.52
RHODE ISLAND	43,650.69	40,705.67	39,605.42	38,560.31	162,522.09
SOUTH CAROLINA	246,284.54	239,531.29	225,630.18	223,391.32	934,837.33
SOUTH DAKOTA	18,010.05	17,526.31	17,139.74	16,269.27	68,945.37
TENNESSEE	474,225.91	466,608.14	447,638.60	462,670.28	1,851,142.93
TEXAS	336,927.85	336,280.34	331,571.65	319,536.42	1,324,316.26
UTAH	174,300.46	170,138.74	164,823.91	161,724.13	670,987.24
VERMONT	23,850.89	23,391.11	22,159.10	21,342.96	90,744.06
VIRGIN ISLANDS	584.77	578.06	432.34	506.93	2,102.10
VIRGINIA	321,463.45	300,363.76	284,391.42	279,939.38	1,186,158.01
WASHINGTON	305,726.74	297,580.02	285,588.93	273,319.11	1,162,214.80
WEST VIRGINIA	88,959.43	84,822.16	81,205.99	77,920.68	332,908.26
WISCONSIN	235,687.35	216,552.16	206,158.06	199,322.36	857,719.93
WYOMING	26,786	25,404.48	25,006.32	24,972.03	102,168.83
TOTAL	12,839,112.22	12,428,616.40	11,903,142.61	11,685,475.62	48,856,346.85

DRUG CODE:9150 DRUG NAME:HYDROMORPHONE

STATE	QUARTER 1	QUARTER 2	QUARTER 3	QUARTER 4	TOTAL GRAMS
ALABAMA	3,528.78	3,174.30	3,221.27	3,112.98	13,037.33
ALASKA	1,065.25	976.89	906.53	948.56	3,897.23
AMERICAN SAMOA	0.89	0.09	0	0	0.98
ARIZONA	7,089.09	6,897.62	6,595.39	6,229.48	26,811.58
ARKANSAS	3,376.84	3,287.22	3,220.53	3,170.03	13,054.62
CALIFORNIA	40,477.70	39,869.34	38,125	36,820.78	155,292.82
COLORADO	7,370.82	6,737.63	6,506.99	6,583.16	27,198.60

CONNECTICUT	6,671.40	6,448.66	6,205.18	6,250.52	25,575.76
DELAWARE	2,294.82	2,079.53	2,039.22	2,109.63	8,523.20
DISTRICT OF COLUMBIA	596.68	539.79	529.24	501.62	2,167.33
FLORIDA	50,158.18	46,655.17	44,394.53	45,519.06	186,726.94
GEORGIA	8,772.96	8,321.02	8,410.75	7,793.73	33,298.46
GUAM	31.75	26.32	23.41	33.03	114.51
HAWAII	692.64	706.22	642.87	629.49	2,671.22
IDAHO	1,942.65	1,814.90	1,839.38	1,928.86	7,525.79
ILLINOIS	10,292.76	9,835.82	9,410.79	9,365.83	38,905.20
INDIANA	6,061.70	6,085.13	5,919.92	5,762.15	23,828.90
IOWA	3,176.90	2,986.13	2,873.08	2,856.05	11,892.16
KANSAS	3,575.53	3,711.19	3,562.32	3,128.83	13,977.87
KENTUCKY	2,970.10	2,647.44	2,821.70	3,354.10	11,793.34
LOUISIANA	3,714.46	3,255.18	3,095.98	3,107.57	13,173.19
MAINE	1,611.81	2,004.63	1,322.33	1,747.62	6,686.39
MARYLAND	8,933.97	8,489.56	8,073.63	7,917.26	33,414.42
MASSACHUSETTS	7,792.78	7,524.92	6,867.12	7,289.33	29,474.15
MICHIGAN	9,582.29	8,840.05	8,938.15	8,791.55	36,152.04
MINNESOTA	5,937.44	6,034.87	5,959.61	5,859.45	23,791.37
MISSISSIPPI	5,027.87	6,221.14	5,736.32	6,418.37	23,403.70
MISSOURI	7,000.39	6,365.07	6,187.46	6,249.20	25,802.12
MONTANA	1,681.14	1,613.19	1,733.22	1,536.91	6,564.46
NEBRASKA	1,377.27	1,375.99	1,223.01	1,230.50	5,206.77
NEVADA	3,173.81	3,007.67	2,832.63	2,782.37	11,796.48
NEW HAMPSHIRE	2,059.38	1,997.46	1,977.49	1,829.39	7,863.72
NEW JERSEY	9,575.10	9,039.88	8,435.17	8,447.42	35,497.57
NEW MEXICO	1,465.35	1,417.73	1,270.28	1,242.25	5,395.61
NEW YORK	20,268.56	19,768.22	19,110.20	18,914.43	78,061.41
NORTH CAROLINA	11,655.08	10,764.60	10,653.70	9,964.11	43,037.49
NORTH DAKOTA	644.43	678.33	630.46	642.14	2,595.36
OHIO	9,597.27	9,300.50	9,237.22	9,247.73	37,382.72
OKLAHOMA	3,666.11	3,777.89	3,636.19	3,519.73	14,599.92
OREGON	5,056.14	4,949.63	4,841.48	4,498.62	19,345.87
PENNSYLVANIA	17,756.18	14,688.28	13,930.26	14,126.71	60,501.43
PUERTO RICO	75.7	65.08	80.93	74.93	296.64
RHODE ISLAND	886.87	1,034.75	811.1	800.25	3,532.97
SOUTH CAROLINA	6,002.10	5,829.97	5,510	5,634.37	22,976.44

SOUTH DAKOTA	519.68	525.6	512.84	641.6	2,199.72
TENNESSEE	8,202.61	6,182.19	6,449.40	5,822.20	26,656.40
TEXAS	24,745.91	24,373.73	23,788.71	22,501.46	95,409.81
UTAH	2,558.55	2,611.03	2,782.81	2,953.08	10,905.47
VERMONT	1,238.83	1,269.84	1,196.76	1,167.38	4,872.81
VIRGIN ISLANDS	26.6	18.71	13.94	14.9	74.15
VIRGINIA	12,556.24	11,386.29	10,518.79	10,482.93	44,944.25
WASHINGTON	11,178.55	11,112.49	10,663.43	9,872.34	42,826.81
WEST VIRGINIA	1,685.85	1,550.98	1,453.60	1,363.74	6,054.17
WISCONSIN	3,854.46	3,511.27	3,699.29	3,797.14	14,862.16
WYOMING	652.17	642.44	604.6	540.15	2,439.36
TOTAL	371,908.39	354,029.57	341,026.21	337,127.02	1,404,091.19

DRUG CODE:9193 DRUG NAME:HYDROCODONE

STATE	QUARTER 1	QUARTER 2	QUARTER 3	QUARTER 4	TOTAL GRAMS
ALABAMA	258,345.10	246,512.38	233,339.55	233,698.90	971,895.93
ALASKA	11,947.24	11,492.75	10,775.27	10,314.83	44,530.09
AMERICAN SAMOA	4	0.27	3.63	0	7.9
ARIZONA	112,100.29	104,522.05	98,362.40	97,818.88	412,803.62
ARKANSAS	140,205.70	136,469.71	129,933.06	129,068.61	535,677.08
CALIFORNIA	952,326.68	931,986.40	894,017.09	878,029.49	3,656,359.66
COLORADO	68,829.55	65,572.12	63,316.81	62,200.10	259,918.58
CONNECTICUT	21,699.36	20,448.70	19,209.66	19,001.68	80,359.40
DELAWARE	6,389.70	5,916.80	5,826.12	5,827.43	23,960.05
DISTRICT OF COLUMBIA	1,571.73	1,460.61	1,444.90	1,289.19	5,766.43
FLORIDA	384,180.30	363,407.66	338,761.82	353,668.78	1,440,018.56
GEORGIA	259,038.74	250,146	237,567.07	237,305.97	984,057.78
GUAM	148.02	196.51	149.38	136.06	629.97
HAWAII	18,613.32	17,730.01	17,098.98	16,443.22	69,885.53
IDAHO	58,546.42	57,869.44	55,812.53	55,830.66	228,059.05
ILLINOIS	320,046.07	312,772.26	300,104.23	298,271.74	1,231,194.30
INDIANA	236,905.30	232,762.71	219,377.34	215,862.49	904,907.84
IOWA	59,394.52	58,576.56	56,201.60	55,504.63	229,677.31
KANSAS	84,159.41	82,242.50	79,387.08	78,882.16	324,671.15
KENTUCKY	208,448.35	203,596.96	194,363.59	193,159.90	799,568.80
LOUISIANA	173,235.24	166,066.34	157,962.30	154,816.27	652,080.15

MAINE	17,776.45	17,296.48	16,839.01	16,547.86	68,459.80
MARYLAND	37,569.09	35,131.47	33,032.25	32,735.19	138,468
MASSACHUSETTS	31,806.48	30,798.70	29,037.25	29,028.79	120,671.22
MICHIGAN	418,265.12	410,822.78	391,555.73	374,540.57	1,595,184.20
MINNESOTA	48,330.57	46,169.58	44,817.69	43,263.92	182,581.76
MISSISSIPPI	126,336.22	121,180.34	112,361.19	106,669.46	466,547.21
MISSOURI	184,389.53	182,181.22	172,468.98	168,909.19	707,948.92
MONTANA	26,789.61	26,089.84	25,288.98	24,710.06	102,878.49
NEBRASKA	33,986.82	32,483.56	31,872.68	31,628.37	129,971.43
NEVADA	98,912.23	96,350.94	91,032.69	91,209.30	377,505.16
NEW HAMPSHIRE	9,491.69	9,032.94	8,752.47	8,846.23	36,123.33
NEW JERSEY	38,281.54	34,570.40	32,758.32	32,517.77	138,128.03
NEW MEXICO	36,907.72	35,625.33	33,655.64	33,354.99	139,543.68
NEW YORK	180,175.57	174,189.76	164,949	161,835.48	681,149.81
NORTH CAROLINA	212,067.30	195,483.54	180,862.60	180,907.74	769,321.18
NORTH DAKOTA	10,955.98	10,561.01	10,048.93	9,927.41	41,493.33
OHIO	199,125.37	189,484.03	180,291.94	173,999.46	742,900.80
OKLAHOMA	187,458.77	185,595.49	177,264.58	175,532.11	725,850.95
OREGON	99,225.54	95,247.81	92,086.04	90,141.72	376,701.11
PENNSYLVANIA	176,205.06	167,098.93	164,049.26	152,735.32	660,088.57
PUERTO RICO	1,097.43	660.78	400.12	388.37	2,546.70
RHODE ISLAND	11,199.72	10,503.30	10,008.87	9,775.76	41,487.65
SOUTH CAROLINA	147,482.52	140,547.45	130,855.75	131,634.30	550,520.02
SOUTH DAKOTA	15,800.85	14,917.59	14,418.82	14,069.75	59,207.01
TENNESSEE	255,932.40	247,128.17	239,087.40	230,700.09	972,848.06
TEXAS	688,696.66	675,195.44	651,021.39	643,395.78	2,658,309.27
UTAH	61,386.94	59,307.58	56,889.89	56,293.13	233,877.54
VERMONT	5,134.85	4,910.12	4,565.24	4,527.41	19,137.62
VIRGIN ISLANDS	363.62	340.97	223.69	230.32	1,158.60
VIRGINIA	116,394.84	105,431.76	97,538.61	96,974.23	416,339.44
WASHINGTON	137,465.59	133,461.20	128,219.88	124,321.75	523,468.42
WEST VIRGINIA	62,746.35	60,335.58	57,352.51	55,167.17	235,601.61
WISCONSIN	82,538.61	77,977.55	75,530.83	75,485.88	311,532.87
WYOMING	12,184.40	11,693.32	11,112.16	11,019.53	46,009.41
TOTAL	7,148,616.48	6,907,553.70	6,583,264.80	6,490,155.40	27,129,590.38

RETAIL DRUG DISTRIBUTION BY STATE WITHIN DRUG CODE BY GRAMS WT

DRUG CODE:9220L DRUG NAME:LEVORPHANOL

STATE	QUARTER 1	QUARTER 2	QUARTER 3	QUARTER 4	TOTAL GRAMS
ALABAMA	5.92	4.29	5.34	4.53	20.08
ARIZONA	18.46	22.06	29.37	30.06	99.95
ARKANSAS	2.79	3.37	1.97	0.93	9.06
CALIFORNIA	109.81	102.15	98.9	91.7	402.56
COLORADO	6.04	12.88	13.81	18.11	50.84
CONNECTICUT	1.63	3.71	5.11	9.75	20.2
DELAWARE	0.93	0	0.58	1.04	2.55
FLORIDA	26.23	35.87	34.36	39.58	136.04
GEORGIA	0.7	2.55	2.9	3.48	9.63
IDAHO	0	0	0.12	0.12	0.24
ILLINOIS	8.71	13.12	14.86	16.02	52.71
INDIANA	25.89	24.49	24.84	22.17	97.39
IOWA	4.41	3.95	3.37	3.71	15.44
KANSAS	11.26	9.98	7.2	7.43	35.87
KENTUCKY	0	0	0.23	0.12	0.35
LOUISIANA	16.48	19.5	20.78	19.5	76.26
MARYLAND	3.13	6.04	3.83	8.36	21.36
MASSACHUSETTS	20.78	19.62	20.2	20.08	80.68
MICHIGAN	0.35	0.35	0.35	0	1.05
MINNESOTA	0.35	0.46	0.81	1.16	2.78
MISSISSIPPI	0.23	0	0	0	0.23
MISSOURI	13.81	19.62	26.23	27.28	86.94
NEBRASKA	4.06	5.11	5.46	9.17	23.8
NEVADA	2.9	2.9	4.88	3.48	14.16
NEW HAMPSHIRE	1.16	1.86	1.63	0.7	5.35
NEW JERSEY	313.88	400.82	406.98	498.56	1,620.24
NEW YORK	33.2	38.65	40.98	48.29	161.12
NORTH CAROLINA	50.84	48.52	64.66	101.69	265.71
OHIO	8.01	14.63	11.72	9.17	43.53
OREGON	3.48	3.48	2.9	3.71	13.57
PENNSYLVANIA	10.56	15.21	14.97	12.88	53.62
SOUTH CAROLINA	0.81	3.37	3.25	3.6	11.03
TENNESSEE	0.12	0.23	0.35	0	0.7
TEXAS	39.24	53.63	61.41	66.05	220.33
VERMONT	0.23	0.46	0.46	0.46	1.61

VIRGINIA	5.8	5.8	11.14	16.25	38.99
WASHINGTON	22.64	24.49	30.88	31.23	109.24
WISCONSIN	1.74	3.83	4.99	4.99	15.55
TOTAL	776.58	927	981.82	1,135.36	3,820.76

DRUG CODE:9230 DRUG NAME:MEPERIDINE (PETHIDINE)

STATE	QUARTER 1	QUARTER 2	QUARTER 3	QUARTER 4	TOTAL GRAMS
ALABAMA	8,009.96	7,392.30	6,862.91	7,150.03	29,415.20
ALASKA	850.15	704.17	662.45	634.23	2,851
ARIZONA	5,450.80	4,921.23	4,373.73	4,759.85	19,505.61
ARKANSAS	8,349.08	7,839.27	7,085.38	6,325.67	29,599.40
CALIFORNIA	15,974.75	15,874.20	15,148.89	15,374.57	62,372.41
COLORADO	2,171.34	1,890.98	1,729.71	1,487	7,279.03
CONNECTICUT	1,104.08	1,052.23	1,036.43	1,069.77	4,262.51
DELAWARE	248.38	274.52	215.81	132.03	870.74
DISTRICT OF COLUMBIA	217.66	220.05	168.96	147.94	754.61
FLORIDA	9,951.88	9,511.01	7,808.42	8,543.21	35,814.52
GEORGIA	11,498.61	10,443.58	9,714.92	9,934.14	41,591.25
GUAM	30.61	34.64	66.23	52.18	183.66
HAWAII	382.26	394.35	305.9	274.74	1,357.25
IDAHO	848.06	925.23	740.75	815.99	3,330.03
ILLINOIS	2,990.92	3,274.25	3,201.46	2,544.13	12,010.76
INDIANA	3,589.75	3,524.96	3,260.70	3,050.25	13,425.66
IOWA	1,927.32	1,893.97	1,991.20	1,968.39	7,780.88
KANSAS	1,860.67	1,792.85	1,712.24	1,705.29	7,071.05
KENTUCKY	2,880.50	2,299.95	2,469.50	2,127.48	9,777.43
LOUISIANA	8,110.88	7,570.39	6,714.82	6,623.16	29,019.25
MAINE	465.38	409.39	359.06	344.35	1,578.18
MARYLAND	2,750.67	2,474.41	2,115.35	2,029.61	9,370.04
MASSACHUSETTS	2,268.84	2,250.69	2,169.27	1,766.20	8,455
MICHIGAN	3,056.18	2,906.26	2,665.16	2,300.80	10,928.40
MINNESOTA	835.66	651.05	651.97	595.21	2,733.89
MISSISSIPPI	4,670.48	4,620.17	3,990.95	4,032.95	17,314.55
MISSOURI	4,628.10	3,912.93	3,526.29	3,143.17	15,210.49
MONTANA	656.94	774.63	657.11	597.63	2,686.31
NEBRASKA	802.06	773.59	669.86	539.46	2,784.97

NEVADA	1,632.86	1,238.60	1,252.61	1,546.80	5,670.87
NEW HAMPSHIRE	676.18	610.92	612.56	450.24	2,349.90
NEW JERSEY	2,186.59	2,180.38	1,832.66	1,503.12	7,702.75
NEW MEXICO	1,680.80	1,416.56	1,314.88	1,238.29	5,650.53
NEW YORK	5,123.33	4,663	4,301.85	4,201.02	18,289.20
NORTH CAROLINA	7,190.99	6,462.50	5,431.34	5,457.05	24,541.88
NORTH DAKOTA	237.48	237.59	253.72	223	951.79
OHIO	5,261.90	5,091.15	5,416.29	4,211.58	19,980.92
OKLAHOMA	7,655.34	7,385.05	6,807.87	6,807.09	28,655.35
OREGON	1,749.54	1,464.32	1,585.80	1,315.75	6,115.41
PENNSYLVANIA	3,944.02	3,517.70	3,425.32	3,312.14	14,199.18
PUERTO RICO	5,652.98	4,931.71	4,118.71	2,668.42	17,371.82
RHODE ISLAND	252.95	264.61	256.77	232.47	1,006.80
SOUTH CAROLINA	6,780.49	6,119.13	5,524.77	5,092.83	23,517.22
SOUTH DAKOTA	550.9	558.74	529.76	436.84	2,076.24
TENNESSEE	8,750.58	7,848.45	6,741.23	6,351.88	29,692.14
TEXAS	11,780.05	10,811.35	11,790.01	11,948.92	46,330.33
UTAH	2,010.75	1,561.27	1,788.51	1,835.03	7,195.56
VERMONT	323.87	315.05	265.59	383.35	1,287.86
VIRGIN ISLANDS	41.94	67.65	41.94	102.4	253.93
VIRGINIA	6,979.19	5,932.13	5,459.56	4,908.18	23,279.06
WASHINGTON	3,773.38	3,371.16	3,264.97	3,133.91	13,543.42
WEST VIRGINIA	731.62	662.45	586.19	510.15	2,490.41
WISCONSIN	1,558.57	1,497.45	1,466.63	1,330.34	5,852.99
WYOMING	378.34	318.27	254.26	350.78	1,301.65
TOTAL	193,486.61	179,134.44	166,399.23	159,621.01	698,641.29

DRUG CODE:9250B DRUG NAME:METHADONE

STATE	QUARTER 1	QUARTER 2	QUARTER 3	QUARTER 4	TOTAL GRAMS
ALABAMA	76,280.07	80,726.33	74,925.79	80,489.93	312,422.12
ALASKA	7,809.90	8,435.09	8,401.37	7,440.60	32,086.96
ARIZONA	85,190.46	85,087.72	82,438.41	83,016.82	335,733.41
ARKANSAS	16,878.18	16,608.11	15,568.82	15,186.55	64,241.66
CALIFORNIA	352,446.36	355,994.06	342,804.92	337,539.34	1,388,784.68
COLORADO	42,100.58	43,293.93	44,969.64	42,654.39	173,018.54
CONNECTICUT	119,258.98	125,911.35	105,169.68	126,064.74	476,404.75

DELAWARE	25,260.03	30,686.69	26,401.26	27,645.37	109,993.35
DISTRICT OF COLUMBIA	9,214.07	10,681.73	9,229.94	10,783.66	39,909.40
FLORIDA	171,777.30	180,889.29	224,484.51	205,561.84	782,712.94
GEORGIA	110,968.35	121,491.67	116,393.86	116,744.99	465,598.87
GUAM	58.14	38.46	42.48	32.2	171.28
HAWAII	7,859.45	8,981.21	7,611.43	7,843.62	32,295.71
IDAHO	12,059.82	12,052.13	11,740.16	11,380.70	47,232.81
ILLINOIS	121,222.97	126,796.08	119,325.66	122,079.43	489,424.14
INDIANA	84,564.33	83,049.21	79,994.79	79,326.75	326,935.08
IOWA	12,299.61	13,491.22	12,656.48	13,823.85	52,271.16
KANSAS	22,687.71	24,554.23	22,465.09	22,738.60	92,445.63
KENTUCKY	51,108.47	49,601.65	53,996.61	51,414.32	206,121.05
LOUISIANA	35,832.62	37,380.73	37,236.43	32,186.93	142,636.71
MAINE	32,346.87	32,802.39	29,492.66	31,583.41	126,225.33
MARYLAND	192,770.82	192,957.93	187,437.82	191,107.27	764,273.84
MASSACHUSETTS	147,168.01	145,889.95	141,669.46	144,647.23	579,374.65
MICHIGAN	134,102.02	134,815.42	133,911.84	131,182.87	534,012.15
MINNESOTA	44,551.68	52,651.48	51,108.51	59,499.49	207,811.16
MISSISSIPPI	6,085.86	6,062.42	7,306.89	7,188.47	26,643.64
MISSOURI	43,867.07	37,494.03	41,804.11	42,815.91	165,981.12
MONTANA	7,680.39	7,484.97	6,497.28	7,558.31	29,220.95
NEBRASKA	8,800.04	7,958.82	8,020.94	7,944.89	32,724.69
NEVADA	36,977.79	37,947.87	35,029.57	34,740.78	144,696.01
NEW HAMPSHIRE	30,916.55	31,234.33	30,579.98	30,602.79	123,333.65
NEW JERSEY	112,688.59	121,219.70	108,428.50	117,224.25	459,561.04
NEW MEXICO	43,022.34	42,657.69	44,251.96	46,464.44	176,396.43
NEW YORK	291,234.12	316,020.36	303,872.37	308,263.87	1,219,390.72
NORTH CAROLINA	129,434.71	134,621.14	132,576.89	129,863.61	526,496.35
NORTH DAKOTA	1,590.15	1,871.35	2,192.89	2,717.99	8,372.38
OHIO	78,169.68	74,777.31	72,839.25	71,527	297,313.24
OKLAHOMA	38,152.69	43,214.64	36,620.05	39,018.11	157,005.49
OREGON	63,733.33	64,825.05	63,346.93	61,850.48	253,755.79
PENNSYLVANIA	194,101.72	199,001.41	192,976.48	203,227.96	789,307.57
PUERTO RICO	22,390.32	18,312.21	24,067.23	19,258.85	84,028.61
RHODE ISLAND	40,681.87	43,097.47	45,509.81	47,819.81	177,108.96
SOUTH CAROLINA	57,684.42	55,976.47	56,288.62	55,428.56	225,378.07
SOUTH DAKOTA	2,264.98	2,147.10	2,231.71	2,114.09	8,757.88

TENNESSEE	49,325.67	49,042.52	47,700.36	48,234.92	194,303.47
TEXAS	139,436.83	145,893.54	134,651.11	142,036.93	562,018.41
UTAH	26,883.52	23,868.58	28,536.01	26,398.31	105,686.42
VERMONT	18,852.34	17,051.91	20,166.13	17,928.87	73,999.25
VIRGIN ISLANDS	28.17	386.38	23.25	19.68	457.48
VIRGINIA	81,665.76	75,402.83	78,605.66	81,398.67	317,072.92
WASHINGTON	85,136.33	93,477.77	104,036.61	108,189.93	390,840.64
WEST VIRGINIA	23,535.33	23,508.59	23,002.98	22,523.41	92,570.31
WISCONSIN	65,713.89	68,870.50	63,531.78	64,031.26	262,147.43
WYOMING	1,277.65	1,164.78	1,182.67	1,131.42	4,756.52
TOTAL	3,617,148.91	3,719,459.80	3,655,355.64	3,699,498.47	14,691,462.82

DRUG CODE:9300 DRUG NAME:MORPHINE

STATE	QUARTER 1	QUARTER 2	QUARTER 3	QUARTER 4	TOTAL GRAMS
ALABAMA	75,385.73	71,751.81	68,342.60	66,586.68	282,066.82
ALASKA	12,075.08	11,675.08	11,278.53	10,703.94	45,732.63
AMERICAN SAMOA	30.08	27.45	3.01	27.07	87.61
ARIZONA	179,749.43	166,620.20	156,623.07	145,087.27	648,079.97
ARKANSAS	49,144.75	45,645	44,441.27	41,659.42	180,890.44
CALIFORNIA	468,312.40	449,794.43	435,963.44	421,426.73	1,775,497
COLORADO	80,950.89	77,471.94	74,788.34	72,248.67	305,459.84
CONNECTICUT	36,962.44	35,651.52	35,131.70	33,709.26	141,454.92
DELAWARE	22,486.45	21,893.44	21,334.15	20,973.47	86,687.51
DISTRICT OF COLUMBIA	3,713.59	3,853.50	3,790.31	3,217.86	14,575.26
FLORIDA	412,688.12	394,998.55	383,405.02	382,567.73	1,573,659.42
GEORGIA	116,831.19	112,302.56	111,023.27	108,398.04	448,555.06
GUAM	1,270.56	829.71	1,031	866.98	3,998.25
HAWAII	23,410.41	22,488.31	21,187.89	20,072.40	87,159.01
IDAHO	29,776.64	28,555.83	27,191.18	27,003.18	112,526.83
ILLINOIS	108,011.75	103,220.32	100,084.61	98,037.74	409,354.42
INDIANA	92,152.33	87,372.85	83,021.72	81,242.99	343,789.89
IOWA	34,869.28	33,926.30	32,608.91	32,570.43	133,974.92
KANSAS	46,843.42	44,319.08	44,034.39	42,477.13	177,674.02
KENTUCKY	49,899.50	46,594.48	44,626.41	44,322.40	185,442.79
LOUISIANA	56,295.87	54,358.01	52,557.96	50,549.33	213,761.17
MAINE	17,045.94	16,858.81	16,420.51	15,608.65	65,933.91

MARYLAND	82,088.87	78,530.74	72,532.28	69,991.46	303,143.35
MASSACHUSETTS	69,568.66	66,507.75	63,147.53	62,974.31	262,198.25
MICHIGAN	200,848.83	192,688.22	185,978.26	174,054.87	753,570.18
MINNESOTA	49,548.65	46,944.54	45,377.16	43,601.89	185,472.24
MISSISSIPPI	41,333.75	40,545.50	38,844.42	39,087.65	159,811.32
MISSOURI	102,523.17	97,078.72	94,755.80	91,575.87	385,933.56
MONTANA	19,066.75	17,234.06	17,003.77	16,337.37	69,641.95
NEBRASKA	19,770.78	18,851.07	18,939.08	18,132.17	75,693.10
NEVADA	62,634.76	60,794.33	58,562.47	55,522.81	237,514.37
NEW HAMPSHIRE	18,582.91	17,841.69	17,901.86	18,151.01	72,477.47
NEW JERSEY	93,090.78	86,208.33	81,320.72	79,622.09	340,241.92
NEW MEXICO	28,578.98	25,993.41	25,356.63	23,224.01	103,153.03
NEW YORK	163,662.50	156,595.10	150,731.44	149,768.80	620,757.84
NORTH CAROLINA	126,674.67	120,047.86	116,465.73	112,951.75	476,140.01
NORTH DAKOTA	6,686.39	6,421.62	5,951.58	6,072.25	25,131.84
OHIO	124,959.28	121,047.73	118,500.26	113,214.79	477,722.06
OKLAHOMA	96,982.97	92,592.40	89,126.22	85,047.29	363,748.88
OREGON	77,234.02	72,077.72	68,842.86	66,164.46	284,319.06
PENNSYLVANIA	263,618.17	229,898.27	221,317.41	217,396.23	932,230.08
PUERTO RICO	2,588.47	2,241.27	1,865.42	1,704.59	8,399.75
RHODE ISLAND	12,069.92	11,689.87	12,022.55	10,846.17	46,628.51
SOUTH CAROLINA	68,726.78	64,803.14	63,883.11	64,033	261,446.03
SOUTH DAKOTA	9,105.11	8,744.97	8,676	8,274.99	34,801.07
TENNESSEE	185,724.11	159,082.83	155,695.27	149,520.74	650,022.95
TEXAS	214,631.96	206,944.37	201,945.89	197,615.22	821,137.44
UTAH	51,222.98	49,400.32	49,045.82	46,883.70	196,552.82
VERMONT	10,746.22	10,191.11	9,758.79	9,832.44	40,528.56
VIRGIN ISLANDS	139.62	152.01	119.42	113.85	524.9
VIRGINIA	84,458.48	79,063.13	76,091.63	74,255.58	313,868.82
WASHINGTON	115,166.01	110,388.89	106,882.84	101,240.93	433,678.67
WEST VIRGINIA	23,389.02	21,876.66	21,639.48	20,541.36	87,446.52
WISCONSIN	83,691.07	77,548.84	74,645.54	72,537.13	308,422.58
WYOMING	9,221.32	9,334.98	9,000.08	8,311.06	35,867.44
TOTAL	4,436,241.81	4,189,570.63	4,050,816.61	3,927,959.21	16,604,588.26

DRUG CODE:9333 DRUG NAME:THEBAINE

STATE	QUARTER 1	QUARTER 2	QUARTER 3	QUARTER 4	TOTAL GRAMS
TEXAS	0	0	25	0	25
TOTAL	0	0	25	0	25

DRUG CODE:9630 DRUG NAME:OPIUM TINCTURE (LAUDANUM)

STATE	QUARTER 1	QUARTER 2	QUARTER 3	QUARTER 4	TOTAL GRAMS
CALIFORNIA	5.91	0	0	0	5.91
CONNECTICUT	0	0	4.73	0	4.73
DELAWARE	0	1.18	0	0	1.18
FLORIDA	9.46	4.73	0	0	14.19
ILLINOIS	5.91	10.64	0	0	16.55
INDIANA	21.28	14.19	0	0	35.47
KANSAS	5.91	0	0	0	5.91
KENTUCKY	2.36	0	0	0	2.36
MARYLAND	4.72	8.27	0	0	12.99
MICHIGAN	26	4.72	0	0	30.72
NEW HAMPSHIRE	4.73	0	0	0	4.73
NEW JERSEY	0	1.18	0	0	1.18
NEW YORK	4.73	0	0	0	4.73
OHIO	73.23	9.45	0	0	82.68
OREGON	2.36	0	0	0	2.36
PENNSYLVANIA	10.62	10.62	0	0	21.24
TEXAS	9.44	4.72	0	0	14.16
VIRGINIA	10.62	0	0	0	10.62
WASHINGTON	3.54	0	0	0	3.54
WEST VIRGINIA	3.54	2.36	0	0	5.9
WISCONSIN	3.54	10.64	0	0	14.18
TOTAL	207.9	82.7	4.73	0	295.33

DRUG CODE:9639 DRUG NAME:OPIUM POWDERED

STATE	QUARTER 1	QUARTER 2	QUARTER 3	QUARTER 4	TOTAL GRAMS
ALABAMA	82.08	94.68	86.4	78.48	341.64
ALASKA	29.52	25.2	19.8	28.08	102.6
ARIZONA	277.56	273.6	292.32	337.68	1,181.16
ARKANSAS	83.52	96.84	87.12	93.6	361.08

CALIFORNIA	412.92	470.52	484.56	453.6	1,821.60
COLORADO	231.84	260.64	288.36	279.36	1,060.20
CONNECTICUT	114.48	120.96	81.72	68.04	385.2
DELAWARE	17.64	14.76	16.2	11.52	60.12
DISTRICT OF COLUMBIA	12.24	12.96	18.36	15.48	59.04
FLORIDA	527.04	498.24	452.52	553.68	2,031.48
GEORGIA	174.24	210.6	185.04	185.76	755.64
GUAM	0.36	0	0	0	0.36
HAWAII	24.84	27.72	34.2	28.44	115.2
IDAHO	45.72	51.12	48.96	49.68	195.48
ILLINOIS	158.4	194.04	172.08	192.96	717.48
INDIANA	222.84	253.8	234.72	270.36	981.72
IOWA	112.32	100.8	96.84	92.52	402.48
KANSAS	165.6	191.88	177.48	185.04	720
KENTUCKY	196.92	179.64	146.16	173.88	696.6
LOUISIANA	105.48	113.04	98.28	104.4	421.2
MAINE	39.24	47.88	54	57.96	199.08
MARYLAND	63	97.56	70.56	104.04	335.16
MASSACHUSETTS	172.8	214.56	177.12	238.68	803.16
MICHIGAN	374.4	369.72	375.84	422.28	1,542.24
MINNESOTA	401.4	381.24	363.96	389.16	1,535.76
MISSISSIPPI	33.12	39.96	36.36	25.2	134.64
MISSOURI	169.92	163.44	166.32	174.6	674.28
MONTANA	53.28	52.92	47.88	50.04	204.12
NEBRASKA	91.8	106.2	90.36	105.48	393.84
NEVADA	30.6	28.08	34.92	40.68	134.28
NEW HAMPSHIRE	30.24	37.44	37.44	41.4	146.52
NEW JERSEY	87.12	133.56	117.72	104.76	443.16
NEW MEXICO	37.8	49.32	29.16	28.44	144.72
NEW YORK	208.8	217.44	201.96	241.2	869.4
NORTH CAROLINA	238.32	218.52	219.96	240.84	917.64
NORTH DAKOTA	30.96	42.12	45.72	46.08	164.88
OHIO	371.88	380.16	376.2	386.28	1,514.52
OKLAHOMA	76.68	83.52	95.4	55.08	310.68
OREGON	168.48	215.28	203.04	211.32	798.12
PENNSYLVANIA	296.64	288	334.8	380.88	1,300.32
RHODE ISLAND	32.76	34.2	45	25.56	137.52

SOUTH CAROLINA	127.8	154.8	127.44	145.08	555.12
SOUTH DAKOTA	64.08	46.8	27	42.84	180.72
TENNESSEE	171	192.6	190.8	205.56	759.96
TEXAS	503.64	504.36	513.36	567.36	2,088.72
UTAH	67.68	64.08	62.64	66.24	260.64
VERMONT	41.4	39.96	37.44	43.92	162.72
VIRGIN ISLANDS	0	0	1.8	0	1.8
VIRGINIA	170.64	178.2	199.44	174.6	722.88
WASHINGTON	281.88	321.12	325.44	329.04	1,257.48
WEST VIRGINIA	84.6	110.88	87.12	98.28	380.88
WISCONSIN	183.24	223.92	239.04	208.44	854.64
WYOMING	25.56	25.56	19.8	27.36	98.28
TOTAL	7,726.32	8,254.44	7,976.16	8,481.24	32,438.16

DRUG CODE:9652 DRUG NAME:OXYMORPHONE

STATE	QUARTER 1	QUARTER 2	QUARTER 3	QUARTER 4	TOTAL GRAMS
ALABAMA	7,674.65	6,782.29	4,108.61	2,938.27	21,503.82
ALASKA	287.43	286.85	246.49	183.95	1,004.72
ARIZONA	8,602.56	7,495.65	4,844.27	2,808.40	23,750.88
ARKANSAS	2,279	2,185.73	1,551.85	856.42	6,873
CALIFORNIA	17,090.45	15,659.83	11,435.85	8,028.86	52,214.99
COLORADO	4,341.19	3,794.17	2,855.79	1,830.99	12,822.14
CONNECTICUT	3,455.85	3,161.39	2,320.28	1,557.38	10,494.90
DELAWARE	2,154.74	2,187.70	1,843.66	1,539.54	7,725.64
DISTRICT OF COLUMBIA	120.14	126.86	101.43	130.83	479.26
FLORIDA	28,038.19	26,517.35	19,406.08	12,915.73	86,877.35
GEORGIA	8,564.78	8,249.88	5,403.09	3,602.98	25,820.73
HAWAII	678.13	699.76	394.84	373.52	2,146.25
IDAHO	1,587.02	1,492.09	1,174.85	891.12	5,145.08
ILLINOIS	5,304.30	4,984.65	3,638.65	2,369.95	16,297.55
INDIANA	6,675.18	5,775.53	4,214.64	2,802.71	19,468.06
IOWA	1,375.82	1,250.29	911.24	569.87	4,107.22
KANSAS	2,458.24	2,307.42	1,630.98	1,114.41	7,511.05
KENTUCKY	4,554.13	4,419.69	3,133.32	2,355.99	14,463.13
LOUISIANA	8,205.45	7,664.08	5,756.32	4,709.35	26,335.20
MAINE	1,190.10	1,005.89	857.8	644.72	3,698.51

MARYLAND	11,286.45	10,601.73	8,197.77	6,964.20	37,050.15
MASSACHUSETTS	2,863.05	2,730.45	1,968.60	1,139.08	8,701.18
MICHIGAN	20,397.38	20,864.79	18,129.57	16,304.25	75,695.99
MINNESOTA	501.31	519.37	404.17	273.25	1,698.10
MISSISSIPPI	3,886.94	3,589.36	2,738.88	2,033.10	12,248.28
MISSOURI	5,853.66	5,330.79	3,721.71	2,321.96	17,228.12
MONTANA	388	296.53	289.98	158.26	1,132.77
NEBRASKA	1,567.20	1,511.89	1,058.08	630.18	4,767.35
NEVADA	5,434.15	4,839.37	3,514.87	2,141.98	15,930.37
NEW HAMPSHIRE	1,095.76	992.83	826.22	576.79	3,491.60
NEW JERSEY	8,392.25	7,809.68	5,422.94	4,069.18	25,694.05
NEW MEXICO	1,251.97	1,079.55	813.91	652.62	3,798.05
NEW YORK	21,903.43	20,825.78	16,371.89	12,712.78	71,813.88
NORTH CAROLINA	27,893.54	25,337.27	17,495.95	12,229.58	82,956.34
NORTH DAKOTA	174.23	180.74	159.69	114.99	629.65
OHIO	12,220.12	11,461	8,794.23	6,074.01	38,549.36
OKLAHOMA	6,983.86	6,587.36	4,887.55	3,302.06	21,760.83
OREGON	2,032.61	1,876.84	1,576.03	1,095.23	6,580.71
PENNSYLVANIA	26,926.77	26,231.13	21,007.71	16,901.20	91,066.81
RHODE ISLAND	576.86	486.59	364.38	174.18	1,602.01
SOUTH CAROLINA	7,767.06	7,503.36	5,106.78	3,625.45	24,002.65
SOUTH DAKOTA	631.75	533.57	419.11	327.76	1,912.19
TENNESSEE	25,189.91	23,346.52	18,453.28	15,244.47	82,234.18
TEXAS	15,482.71	14,431.61	9,895.18	6,358.75	46,168.25
UTAH	4,816.31	4,513.19	3,837.50	3,232.44	16,399.44
VERMONT	362.68	325.42	275.39	181.36	1,144.85
VIRGINIA	11,386.63	10,375.13	7,899.21	6,651.41	36,312.38
WASHINGTON	3,634.01	3,266.76	2,594.27	1,910.34	11,405.38
WEST VIRGINIA	2,929.48	2,929.43	2,410.14	1,654.58	9,923.63
WISCONSIN	2,924.54	2,539.68	1,843.84	1,203.31	8,511.37
WYOMING	322.49	342.83	255.36	199.34	1,120.02
TOTAL	351,714.46	329,307.65	246,564.23	182,683.08	1,110,269.42

DRUG CODE:9668 DRUG NAME:NOROXYMORPHONE

STATE	QUARTER 1	QUARTER 2	QUARTER 3	QUARTER 4	TOTAL GRAMS
ARIZONA	0	0	0.1	0	0.1

ARKANSAS	14.63	17.21	26.85	19.38	78.07
CALIFORNIA	52.59	68.53	69.85	68.65	259.62
FLORIDA	12.61	13.83	13.51	18.08	58.03
GEORGIA	3.18	3.18	9.3	4.8	20.46
ILLINOIS	4.42	9.76	15.09	8.75	38.02
KANSAS	2.75	0	0	0	2.75
MARYLAND	0	0.1	0	0.1	0.2
MASSACHUSETTS	1.14	1.9	0	0	3.04
MICHIGAN	0	0	3.12	1.17	4.29
MINNESOTA	0	0.5	0	0	0.5
MISSISSIPPI	2.4	1.57	2.22	0	6.19
MISSOURI	7.71	13.1	10.59	5.8	37.2
NEVADA	6.64	2.87	7.45	8.31	25.27
NEW JERSEY	7.12	12.03	1.56	3	23.71
NEW YORK	2.65	5.69	9.3	4.49	22.13
NORTH CAROLINA	0	0	2.12	1.56	3.68
OHIO	5.89	9.36	6.19	2.88	24.32
OKLAHOMA	0	0	0.1	0.55	0.65
OREGON	0.15	0.04	1.81	0	2
PENNSYLVANIA	0	0.51	0.47	0	0.98
PUERTO RICO	1.86	4.44	11.94	6.83	25.07
RHODE ISLAND	0	0	0.1	0	0.1
TENNESSEE	0.13	0	0	0	0.13
TEXAS	21.77	29.59	31.28	26.19	108.83
VERMONT	0	0	0.1	0	0.1
WASHINGTON	0.65	0.68	3.21	3.94	8.48
TOTAL	148.29	194.89	226.26	184.48	753.92

DRUG CODE:9737 DRUG NAME:ALFENTANIL

STATE	QUARTER 1	QUARTER 2	QUARTER 3	QUARTER 4	TOTAL GRAMS
ALABAMA	0.04	0.13	0.05	0.03	0.25
ALASKA	0.75	0.75	0.5	0.89	2.89
ARIZONA	1.12	0.96	0.69	0.97	3.74
ARKANSAS	0.01	0	0.04	0.03	0.08
CALIFORNIA	17.35	15.3	16.28	17	65.93
COLORADO	1.27	0.72	0.79	1.08	3.86

CONNECTICUT	1.52	1.2	1.03	1.55	5.3
FLORIDA	4.55	5.48	4.78	6.1	20.91
GEORGIA	2.92	2.79	4.66	2.69	13.06
GUAM	0	0	0.05	0.03	0.08
HAWAII	0.45	0.5	0.62	0.84	2.41
IDAHO	1.46	1.39	1.15	1.46	5.46
ILLINOIS	3.76	4.23	3.37	5.14	16.5
INDIANA	2.24	3.18	2.54	2.34	10.3
IOWA	2.05	1.5	1.4	1.74	6.69
KANSAS	1.62	1.51	1.26	1.6	5.99
KENTUCKY	5	3.91	3.67	3.85	16.43
LOUISIANA	2.44	2.28	2.84	2.5	10.06
MAINE	0.62	0.79	0.63	0.65	2.69
MARYLAND	0.89	0.24	0.85	0.92	2.9
MASSACHUSETTS	1.35	0.73	0.65	0.73	3.46
MICHIGAN	0.91	1.32	1.05	1.21	4.49
MINNESOTA	3.24	3.41	3.23	4.04	13.92
MISSISSIPPI	0.07	0.12	0.17	0.07	0.43
MISSOURI	1.21	1	0.9	1.22	4.33
MONTANA	0.36	0.27	0.33	0.34	1.3
NEBRASKA	0.84	0.7	1.08	0.94	3.56
NEVADA	1.27	1.59	1.74	2.36	6.96
NEW HAMPSHIRE	0.48	0.42	0.44	0.61	1.95
NEW JERSEY	0.2	0.27	0.23	0.35	1.05
NEW MEXICO	0.87	0.88	1.14	1.21	4.1
NEW YORK	2.12	1.22	1.66	4.16	9.16
NORTH CAROLINA	2.78	3.38	2.92	3.5	12.58
NORTH DAKOTA	0.11	0.28	0.09	0.11	0.59
OHIO	4.49	6.14	3.5	4.97	19.1
OKLAHOMA	0.83	1.01	0.84	0.84	3.52
OREGON	3.84	4.32	5.66	4.86	18.68
PENNSYLVANIA	0.7	0.48	0.37	0.46	2.01
RHODE ISLAND	0.05	0.08	0.13	0	0.26
SOUTH CAROLINA	0.13	0.05	0.05	0.13	0.36
SOUTH DAKOTA	0.11	0.06	0.1	0.06	0.33
TENNESSEE	0.97	1.08	1.16	1.56	4.77
TEXAS	5.06	6.06	5.21	5.72	22.05

UTAH	0.47	0.35	0.52	1.76	3.1
VERMONT	0.01	0	0.01	0.02	0.04
VIRGINIA	0.87	1.13	0.68	0.94	3.62
WASHINGTON	2.53	3.37	3.48	3.28	12.66
WISCONSIN	4.13	4.82	4.81	5.52	19.28
WYOMING	0.01	0	0.02	0.09	0.12
TOTAL	90.07	91.4	89.37	102.47	373.31

DRUG CODE:9739 DRUG NAME:REMIFENTANIL

STATE	QUARTER 1	QUARTER 2	QUARTER 3	QUARTER 4	TOTAL GRAMS
ALABAMA	3.16	3.41	3.44	3.14	13.15
ALASKA	2.73	3.01	2.8	3.14	11.68
ARIZONA	4.16	3.74	3.63	4.16	15.69
ARKANSAS	1.57	1.52	1.82	1.48	6.39
CALIFORNIA	33.2	34.56	31.25	34.34	133.35
COLORADO	5.96	6.53	6.18	7.17	25.84
CONNECTICUT	9.81	7.72	7.82	8.71	34.06
DELAWARE	2.67	4.02	2.81	2.54	12.04
DISTRICT OF COLUMBIA	0.88	0.86	0.52	0.58	2.84
FLORIDA	25.75	26.92	25.23	29.55	107.45
GEORGIA	4.65	5.09	4.67	4.79	19.2
GUAM	0.02	0.04	0	0.25	0.31
HAWAII	1.29	1.13	1.34	1.38	5.14
IDAHO	2.25	2.19	2.12	2.14	8.7
ILLINOIS	14.81	14.48	14.58	16.89	60.76
INDIANA	8.1	7.75	7.97	8.11	31.93
IOWA	3.57	3.24	3.33	2.44	12.58
KANSAS	2.1	2.53	2.11	2.33	9.07
KENTUCKY	5.02	4.27	5.26	4.61	19.16
LOUISIANA	4.41	4.7	4.5	4.96	18.57
MAINE	1.37	1.2	1.11	0.93	4.61
MARYLAND	7.19	7.13	5.88	7.2	27.4
MASSACHUSETTS	20.68	23.9	21.61	23.06	89.25
MICHIGAN	9.51	10.95	9.68	9.49	39.63
MINNESOTA	7.02	7.9	7.92	9.85	32.69
MISSISSIPPI	0.56	0.63	0.27	0.32	1.78

MISSOURI	5.02	5.31	5.86	5.58	21.77
MONTANA	1.45	1.83	1.72	1.99	6.99
NEBRASKA	1.14	0.97	0.91	1.27	4.29
NEVADA	0.89	0.74	1.11	0.96	3.7
NEW HAMPSHIRE	1.15	1.52	1.43	2.42	6.52
NEW JERSEY	12.79	13.01	12.94	14.17	52.91
NEW MEXICO	1	1.14	1.27	1.03	4.44
NEW YORK	36.7	37.72	40.31	40.31	155.04
NORTH CAROLINA	13.88	14.85	13.84	15.18	57.75
NORTH DAKOTA	0.58	0.32	0.38	0.39	1.67
OHIO	19.27	20	15.93	20.44	75.64
OKLAHOMA	2.59	2.88	2.65	2.61	10.73
OREGON	2.69	3.27	2.53	2.91	11.4
PENNSYLVANIA	36.19	35.59	35.09	36.37	143.24
RHODE ISLAND	0.68	0.51	0.76	1.22	3.17
SOUTH CAROLINA	5.08	4.36	4.64	4.57	18.65
SOUTH DAKOTA	0.47	0.66	0.44	0.32	1.89
TENNESSEE	4.66	5.42	6.69	6.47	23.24
TEXAS	26.79	27.42	27.2	30.45	111.86
UTAH	17.82	14.08	15.38	24.5	71.78
VERMONT	0.68	0.91	0.52	0.72	2.83
VIRGINIA	6.27	7.39	6.04	7.47	27.17
WASHINGTON	12.71	12.08	14.64	10.58	50.01
WEST VIRGINIA	5.37	6.02	4.95	4.41	20.75
WISCONSIN	6.05	7.56	5.32	7.8	26.73
WYOMING	0.5	0.39	0.43	0.46	1.78
TOTAL	404.86	415.37	400.83	438.16	1,659.22

DRUG CODE:9740 DRUG NAME:SUFENTANIL BASE

STATE	QUARTER 1	QUARTER 2	QUARTER 3	QUARTER 4	TOTAL GRAMS
ALABAMA	1.04	1.76	1.76	2	6.56
ALASKA	0.01	0.01	0.01	0.01	0.04
ARIZONA	0.07	0.07	0.06	0.14	0.34
ARKANSAS	0.14	0.1	0.17	0.15	0.56
CALIFORNIA	9.68	12.78	11.23	10.05	43.74
COLORADO	0.19	0.16	0.21	0.29	0.85

CONNECTICUT	0.41	0.46	0.58	0.94	2.39
DELAWARE	0.01	0.01	0.01	0.01	0.04
DISTRICT OF COLUMBIA	0.01	0	0.01	0	0.02
FLORIDA	4.25	2.01	3.33	3.06	12.65
GEORGIA	0.32	0.34	0.52	0.61	1.79
HAWAII	0.01	0	0.01	0.02	0.04
IDAHO	0.04	0.05	0.04	0.04	0.17
ILLINOIS	0.55	0.61	0.58	0.54	2.28
INDIANA	0.31	0.37	0.25	0.22	1.15
IOWA	0.06	0.04	0.04	0.07	0.21
KANSAS	0.23	0.28	0.35	0.29	1.15
KENTUCKY	0.32	0.4	0.4	0.39	1.51
LOUISIANA	0.35	2.02	1.09	1.17	4.63
MAINE	0.01	0.01	0.02	0.03	0.07
MARYLAND	0.04	0.06	0.07	0.05	0.22
MASSACHUSETTS	0.09	0.12	0.12	0.19	0.52
MICHIGAN	0.54	0.37	0.32	1.02	2.25
MINNESOTA	0.16	0.16	0.15	0.31	0.78
MISSISSIPPI	14.74	10.44	12.48	14.46	52.12
MISSOURI	0.29	0.32	0.36	0.29	1.26
MONTANA	0.04	0.04	0.03	0.06	0.17
NEBRASKA	0.08	0.09	0.08	0.15	0.4
NEVADA	0.07	0.05	0.13	0.12	0.37
NEW HAMPSHIRE	0.01	0.02	0.02	0.02	0.07
NEW JERSEY	0.95	0.99	0.23	1.56	3.73
NEW MEXICO	0.05	0.04	0.02	0.08	0.19
NEW YORK	1.78	1.57	2.02	1.37	6.74
NORTH CAROLINA	0.27	0.42	0.52	0.45	1.66
NORTH DAKOTA	0.02	0.06	0.02	0.03	0.13
OHIO	0.31	0.54	0.41	0.7	1.96
OKLAHOMA	0.18	0.17	0.16	0.17	0.68
OREGON	0.28	0.26	0.31	0.39	1.24
PENNSYLVANIA	0.59	1.97	4.31	1.25	8.12
PUERTO RICO	0.21	0.16	0.23	0.15	0.75
RHODE ISLAND	0	0.01	0	0	0.01
SOUTH CAROLINA	0.79	0.81	0.82	0.23	2.65
SOUTH DAKOTA	0.03	0.03	0.04	0.09	0.19

TENNESSEE	0.67	0.54	0.64	0.71	2.56
TEXAS	1.91	1.38	1.06	2.31	6.66
UTAH	0.26	0.21	0.21	0.24	0.92
VERMONT	0.01	0.01	0	0.01	0.03
VIRGINIA	0.41	0.36	0.34	0.44	1.55
WASHINGTON	0.24	0.21	0.32	0.27	1.04
WEST VIRGINIA	0.04	0.06	0.09	0.06	0.25
WISCONSIN	0.77	0.8	0.09	0.87	2.53
WYOMING	0	0	0.03	0.03	0.06
TOTAL	43.84	43.75	46.3	48.11	182

DRUG CODE:9780 DRUG NAME:TAPENTADOL

STATE	QUARTER 1	QUARTER 2	QUARTER 3	QUARTER 4	TOTAL GRAMS
ALABAMA	59,030.50	56,294.50	52,474.50	50,761.50	218,561
ALASKA	2,095.50	2,167.50	2,319	2,409	8,991
ARIZONA	38,185.50	35,746	32,238	35,953.50	142,123
ARKANSAS	23,293	22,390.50	19,058	19,193	83,934.50
CALIFORNIA	110,557.50	110,431.50	101,643.45	102,244.40	424,876.85
COLORADO	27,467	28,889	27,473	28,423.50	112,252.50
CONNECTICUT	12,657.50	11,895.50	10,938	10,558	46,049
DELAWARE	7,639	7,431.50	7,404	7,199.50	29,674
DISTRICT OF COLUMBIA	1,209.50	1,111	916	860	4,096.50
FLORIDA	109,424.50	103,022.50	92,250.50	91,559.50	396,257
GEORGIA	71,115	70,898	63,634.50	63,983	269,630.50
HAWAII	2,865	3,284	3,128.50	3,316.50	12,594
IDAHO	6,426	5,657.50	5,709	6,334	24,126.50
ILLINOIS	41,449.50	38,740	36,168.50	35,340.50	151,698.50
INDIANA	38,422.50	37,899.50	36,547.50	36,562	149,431.50
IOWA	13,973	12,921	10,709	12,098	49,701
KANSAS	9,528.50	8,789.50	8,288.50	8,435.50	35,042
KENTUCKY	15,161.50	15,153.50	15,330	14,489	60,134
LOUISIANA	15,303.50	15,024	14,597	15,866	60,790.50
MAINE	1,448.50	1,487.50	1,342	1,357.50	5,635.50
MARYLAND	34,304.50	33,980.50	31,173	30,546.50	130,004.50
MASSACHUSETTS	19,238.50	19,594.50	19,295.50	18,947.50	77,076
MICHIGAN	21,858.50	21,251	21,632	21,897.50	86,639

MINNESOTA	4,316.50	4,227	4,405	4,653	17,601.50
MISSISSIPPI	13,141.50	12,524.50	11,834.50	10,785	48,285.50
MISSOURI	25,851	25,232.50	23,086	23,396.50	97,566
MONTANA	2,201.50	1,668.50	1,758.50	1,806	7,434.50
NEBRASKA	10,446	9,342	9,231.50	9,800.50	38,820
NEVADA	12,593.50	12,164.50	11,660	12,387.50	48,805.50
NEW HAMPSHIRE	7,573.50	7,735.50	7,064.50	7,042	29,415.50
NEW JERSEY	48,769.50	48,076.50	46,248.50	46,131.50	189,226
NEW MEXICO	3,536	3,129.50	3,076	3,085	12,826.50
NEW YORK	99,988	97,696	94,081.50	92,119	383,884.50
NORTH CAROLINA	100,947	99,916	93,476.50	92,188	386,527.50
NORTH DAKOTA	2,548	2,511	2,298.50	2,418	9,775.50
OHIO	66,186.78	62,675.45	57,564.95	56,331.83	242,759.01
OKLAHOMA	24,810.50	24,718.50	22,840	22,548.50	94,917.50
OREGON	7,333.50	7,441	7,542	7,498.50	29,815
PENNSYLVANIA	67,481.50	68,673	63,793	64,523.50	264,471
PUERTO RICO	904.5	990	829	650.5	3,374
RHODE ISLAND	2,067.50	2,166.50	2,060	2,096	8,390
SOUTH CAROLINA	44,272	44,556	42,452	42,730	174,010
SOUTH DAKOTA	5,211.50	4,844	4,321	4,483.50	18,860
TENNESSEE	32,883	30,788.50	31,088	32,455.50	127,215
TEXAS	102,077.50	101,164	91,158.50	95,703	390,103
UTAH	15,597	13,584	13,460	12,249.50	54,890.50
VERMONT	1,341.50	1,096	1,038.50	1,144	4,620
VIRGIN ISLANDS	9	6	3	0	18
VIRGINIA	45,995	43,318.50	38,604.50	37,649.50	165,567.50
WASHINGTON	18,321	18,426	16,944.50	16,659	70,350.50
WEST VIRGINIA	6,283	6,593	6,057.50	5,611	24,544.50
WISCONSIN	15,498.50	15,413	14,565	13,998.50	59,475
WYOMING	1,948	2,073.50	1,797	1,947.50	7,766
TOTAL	1,472,787.28	1,436,810.45	1,338,608.90	1,342,427.23	5,590,633.86

DRUG CODE:9801 DRUG NAME:FENTANYL BASE

STATE	QUARTER 1	QUARTER 2	QUARTER 3	QUARTER 4	TOTAL GRAMS
ALABAMA	1,620.56	1,543.32	1,423.48	1,375.76	5,963.12
ALASKA	239.88	239.41	233.05	228.27	940.61

AMERICAN SAMOA	0.24	0.23	0	0	0.47
ARIZONA	1,977.94	1,803.41	1,626.13	1,585.90	6,993.38
ARKANSAS	1,010.45	976.38	886.88	841.48	3,715.19
CALIFORNIA	7,394.64	7,142.92	6,794.64	6,483.09	27,815.29
COLORADO	1,362.25	1,272.08	1,216.64	1,159.62	5,010.59
CONNECTICUT	1,006.18	989.28	924.2	880.94	3,800.60
DELAWARE	358.26	350.22	324.76	327.37	1,360.61
DISTRICT OF COLUMBIA	92.41	90.16	80.36	75.83	338.76
FLORIDA	6,371.59	5,883.81	5,472.76	5,624.55	23,352.71
GEORGIA	2,933.02	2,791.81	2,619.49	2,563.56	10,907.88
GUAM	12.2	10.02	9.17	9.95	41.34
HAWAII	198.18	193.78	175.2	175.15	742.31
IDAHO	594.55	589.21	565.65	555.48	2,304.89
ILLINOIS	3,123.72	2,990.38	2,880.90	2,814.15	11,809.15
INDIANA	2,040.79	1,922.07	1,792.60	1,768.11	7,523.57
IOWA	908.05	886.91	845.88	835.7	3,476.54
KANSAS	1,140.04	1,121.48	1,078.58	1,121.86	4,461.96
KENTUCKY	1,232.80	1,174.90	1,099.14	1,060.15	4,566.99
LOUISIANA	1,301.35	1,216.49	1,153.84	1,083.95	4,755.63
MAINE	388.28	330.7	303.9	299.95	1,322.83
MARYLAND	1,552.80	1,416.82	1,344.99	1,237.78	5,552.39
MASSACHUSETTS	1,389.85	1,337.33	1,266.41	1,235.44	5,229.03
MICHIGAN	3,295.49	3,185.63	3,016.89	2,877.98	12,375.99
MINNESOTA	1,080.41	1,069.20	1,000.89	965.18	4,115.68
MISSISSIPPI	1,253.09	1,311.71	1,214.16	1,143.68	4,922.64
MISSOURI	2,258.79	2,199.65	2,102.82	2,523.82	9,085.08
MONTANA	353.98	336.62	315.94	311.55	1,318.09
NEBRASKA	561.72	540.17	520.25	506.88	2,129.02
NEVADA	656.33	599.66	559.53	538.39	2,353.91
NEW HAMPSHIRE	372.07	351.19	340.42	327.42	1,391.10
NEW JERSEY	2,496.43	2,399.25	2,171.39	2,111.68	9,178.75
NEW MEXICO	486.16	446.12	410.05	392.71	1,735.04
NEW YORK	4,661.14	4,468.97	4,112.05	3,995.21	17,237.37
NORTH CAROLINA	3,098.54	2,916.55	2,645.07	2,606.06	11,266.22
NORTH DAKOTA	248.16	240.41	230.75	228.56	947.88
OHIO	2,651.77	2,544.49	2,386.96	2,266.99	9,850.21
OKLAHOMA	1,847.46	1,730.28	1,633.57	1,543.84	6,755.15

OREGON	1,006.51	954.7	886.81	857.63	3,705.65
PENNSYLVANIA	5,374.35	5,210.59	4,851.08	4,630.51	20,066.53
PUERTO RICO	196.87	151.01	126.57	103.8	578.25
RHODE ISLAND	197.8	186.79	177.75	172.64	734.98
SOUTH CAROLINA	1,424.02	1,346.42	1,259.11	1,231.99	5,261.54
SOUTH DAKOTA	325.31	301.89	295.79	281.25	1,204.24
TENNESSEE	2,093.91	1,965.15	1,845.64	1,784.70	7,689.40
TEXAS	6,128.66	5,913.30	5,543.02	5,441.14	23,026.12
UTAH	862.83	820.99	805.92	785.81	3,275.55
VERMONT	202.75	196.73	180.15	168.44	748.07
VIRGIN ISLANDS	4.01	3.46	3.02	3.47	13.96
VIRGINIA	1,921.32	1,781.64	1,643.32	1,567.23	6,913.51
WASHINGTON	1,614.60	1,556.54	1,458.94	1,402.32	6,032.40
WEST VIRGINIA	577.65	559.14	530.84	499.87	2,167.50
WISCONSIN	1,637.47	1,524.37	1,390.97	1,345.32	5,898.13
WYOMING	168.25	149.5	158.02	145.2	620.97
TOTAL	87,307.88	83,235.24	77,936.34	76,105.31	324,584.77

DRUG CODE:9809 DRUG NAME:OPIUM COMBINATION PRODUCT (C-III)

STATE	QUARTER 1	QUARTER 2	QUARTER 3	QUARTER 4	TOTAL GRAMS
ALABAMA	11.92	12.68	11.73	10.78	47.11
ALASKA	0	0	0.19	0	0.19
ARIZONA	12.11	5.49	7.19	8.51	33.3
ARKANSAS	1.32	2.27	2.84	2.08	8.51
CALIFORNIA	18.16	17.97	17.97	22.51	76.61
COLORADO	3.22	3.03	2.27	2.46	10.98
CONNECTICUT	5.11	3.78	6.43	4.16	19.48
DELAWARE	0.19	0.76	0.19	0.95	2.09
DISTRICT OF COLUMBIA	0.19	0.38	0.38	0.57	1.52
FLORIDA	36.52	38.03	31.79	31.41	137.75
GEORGIA	6.05	8.7	7.57	7.19	29.51
HAWAII	1.89	1.51	1.32	0	4.72
IDAHO	3.97	3.78	3.59	3.78	15.12
ILLINOIS	6.05	6.05	6.62	5.11	23.83
INDIANA	6.81	6.81	8.32	7.19	29.13
IOWA	3.22	1.89	1.7	2.08	8.89

KANSAS	0.76	0.57	0.19	1.32	2.84
KENTUCKY	10.59	10.97	7.76	7.75	37.07
LOUISIANA	7.19	8.7	9.27	8.14	33.3
MAINE	0.76	1.14	2.08	2.84	6.82
MARYLAND	8.51	8.7	8.89	7.95	34.05
MASSACHUSETTS	2.08	2.46	1.32	0.95	6.81
MICHIGAN	4.73	4.92	8.51	4.54	22.7
MINNESOTA	1.7	2.27	2.08	3.03	9.08
MISSISSIPPI	4.54	2.46	3.03	1.7	11.73
MISSOURI	10.22	5.11	3.97	9.27	28.57
MONTANA	0.95	0.19	1.32	0.95	3.41
NEBRASKA	1.14	0.57	0.76	0.57	3.04
NEVADA	0.76	1.32	1.7	0.38	4.16
NEW HAMPSHIRE	1.7	1.89	1.89	3.41	8.89
NEW JERSEY	5.68	5.68	5.3	4.16	20.82
NEW MEXICO	1.89	1.89	0.95	1.89	6.62
NEW YORK	8.32	10.41	8.51	10.78	38.02
NORTH CAROLINA	12.11	14.76	15.14	11.92	53.93
NORTH DAKOTA	0	0.38	0.76	1.51	2.65
OHIO	14.95	13.81	11.14	18.35	58.25
OKLAHOMA	3.22	2.08	3.22	4.35	12.87
OREGON	0.19	0.57	0.95	1.89	3.6
PENNSYLVANIA	31.41	38.97	27.24	38.03	135.65
RHODE ISLAND	0	0.19	0.19	0	0.38
SOUTH CAROLINA	8.32	10.78	10.78	10.22	40.1
SOUTH DAKOTA	1.32	0.57	0.57	0.19	2.65
TENNESSEE	5.68	5.49	5.68	8.14	24.99
TEXAS	11.35	14.19	13.06	14.38	52.98
UTAH	13.05	12.68	10.97	12.87	49.57
VERMONT	0	0.38	0.19	0.38	0.95
VIRGINIA	7.95	5.49	5.68	4.73	23.85
WASHINGTON	2.08	2.08	4.16	2.08	10.4
WEST VIRGINIA	2.65	1.89	2.46	2.65	9.65
WISCONSIN	5.68	4.92	5.49	7	23.09
WYOMING	0	0.57	0.76	1.7	3.03
TOTAL	308.21	312.18	296.07	318.8	1,235.26

DRUG CODE:1100 DRUG NAME: AMPHETAMINE

STATE	1ST QUARTER	2ND QUARTER	3RD QUARTER	4TH QUARTER	TOTAL GRAMS
ALABAMA	2,709.83	2,703.03	2,707.26	2,801.46	10,921.58
ALASKA	1,128.88	1,131.40	1,153.17	1,139.34	4,552.78
AMERICAN SAMOA	1.91	1.91	0	0	3.81
ARIZONA	1,272.08	1,254.45	1,254.68	1,343.46	5,124.67
ARKANSAS	1,735.47	1,733.65	1,745.72	1,793.99	7,008.83
CALIFORNIA	793.33	809.42	794.97	818.4	3,216.12
COLORADO	1,550.18	1,566.11	1,597.28	1,638.41	6,351.99
CONNECTICUT	1,435.10	1,476.24	1,477.48	1,522.39	5,911.21
DELAWARE	2,409.37	2,394.58	2,360.97	2,435.11	9,600.03
DISTRICT OF COLUMBIA	1,922.13	1,934.53	1,911.95	1,986.93	7,755.54
FLORIDA	1,579.12	1,585.03	1,549.21	1,675.59	6,388.94
GEORGIA	1,924.97	1,906.68	1,923.35	1,989.53	7,744.54
GUAM	230.78	313.57	321.81	200.99	1,067.15
HAWAII	703.46	722.96	730.72	704	2,861.14
IDAHO	1,569.83	1,610.19	1,616.22	1,695.58	6,491.82
ILLINOIS	1,470.92	1,468.19	1,478.17	1,536.95	5,954.24
INDIANA	1,969.80	1,985.51	2,004.29	2,060.24	8,019.84
IOWA	1,589.95	1,621.43	1,635.31	1,710.99	6,557.68
KANSAS	2,055.32	2,098.99	2,126.10	2,193.07	8,473.49
KENTUCKY	1,569.71	1,533.77	1,550.52	1,612.99	6,266.99
LOUISIANA	3,371.44	3,317.67	3,371.01	3,563.48	13,623.60
MAINE	1,612.51	1,714.70	1,765.68	1,733	6,825.88
MARYLAND	1,561.96	1,578.02	1,576.86	1,626.98	6,343.81
MASSACHUSETTS	2,672.65	2,744.79	2,683.28	2,771.29	10,872.01
MICHIGAN	2,417.01	2,418.33	2,420.37	2,476.96	9,732.66
MINNESOTA	2,010.59	2,021.51	2,012.53	2,093.70	8,138.34
MISSISSIPPI	2,109.79	2,037.08	2,036.78	2,107.99	8,291.64
MISSOURI	2,201.95	2,206.77	2,235.89	2,315.20	8,959.82
MONTANA	1,375.11	1,402.69	1,431.13	1,450.64	5,659.57
NEBRASKA	1,386.55	1,381.19	1,424.57	1,464.70	5,657.01
NEVADA	944.07	962.19	961.67	988.6	3,856.53
NEW HAMPSHIRE	2,830.94	2,942.84	2,922.67	2,991.56	11,688.01
NEW JERSEY	1,159.81	1,179.10	1,177.12	1,223.32	4,739.35
NEW MEXICO	815.96	788	758.68	728.9	3,091.54

DRUG CODE:1100 DRUG NAME: AMPHETAMINE

STATE	1ST QUARTER	2ND QUARTER	3RD QUARTER	4TH QUARTER	TOTAL GRAMS
NEW YORK	1,178.54	1,216.31	1,204.59	1,260.34	4,859.78
NORTH CAROLINA	2,106.18	2,116.36	2,111	2,187.59	8,521.14
NORTH DAKOTA	1,309.70	1,321.29	1,348.64	1,367.49	5,347.13
OHIO	1,802.47	1,804.72	1,819.55	1,915.35	7,342.08
OKLAHOMA	1,393.76	1,401.39	1,399.93	1,445.69	5,640.76
OREGON	1,492.68	1,547.15	1,528.79	1,554.06	6,122.67
PENNSYLVANIA	1,641.45	1,663.06	1,644.10	1,698.46	6,647.06
PUERTO RICO	243.62	226.59	210.57	208.03	888.81
RHODE ISLAND	3,361.76	3,465.93	3,501.38	3,593	13,922.07
SOUTH CAROLINA	2,954.61	2,917.70	2,895.70	3,019.02	11,787.03
SOUTH DAKOTA	1,963.14	1,832	1,800.07	1,873.93	7,469.14
TENNESSEE	2,033.07	2,024.64	2,028.33	2,114.88	8,200.92
TEXAS	1,620.45	1,634.58	1,621.43	1,708.64	6,585.10
UTAH	2,866	2,931.71	2,957.75	3,100.55	11,856.01
VERMONT	1,566.15	1,583.62	1,563.11	1,629.91	6,342.78
VIRGIN ISLANDS	335.82	331.61	228.68	212.23	1,108.34
VIRGINIA	2,214.20	2,229.25	2,207.16	2,274.29	8,924.90
WASHINGTON	1,594.11	1,632.04	1,609.75	1,638.92	6,474.81
WEST VIRGINIA	1,287.39	1,255.65	1,253.30	1,275.06	5,071.40

WISCONSIN	2,285.68	2,226.39	2,206.22	2,264.20	8,982.49
WYOMING	1,172.74	1,105.72	1,137.44	1,150.24	4,566.14
U.S. TOTAL	1,652.28	1,662.14	1,657.05	1,721.82	6,693.29

DRUG CODE:1105B DRUG NAME: DL-METHAMPHETAMINE RACEMIC BASE

STATE	1ST QUARTER	2ND QUARTER	3RD QUARTER	4TH QUARTER	TOTAL GRAMS
ALABAMA	0.47	0.51	0.58	0.45	2.01
ALASKA	0.11	0.68	0.57	0.17	1.53
ARIZONA	0.53	0.5	0.45	0.62	2.1
ARKANSAS	0.18	0.25	0.23	0.21	0.87
CALIFORNIA	0.86	0.83	0.73	0.67	3.09
COLORADO	0.97	1.08	0.99	1.14	4.19
CONNECTICUT	0.57	0.69	0.56	0.69	2.51
DELAWARE	0.27	0.4	0.54	0.49	1.7
DISTRICT OF COLUMBIA	0.67	0.33	0	0.13	1.14
FLORIDA	0.15	0.21	0.19	0.17	0.72
GEORGIA	0.33	0.25	0.18	0.21	0.98
HAWAII	0.38	0.5	0.3	0.32	1.51
IDAHO	0.49	0.44	0.67	0.64	2.23
ILLINOIS	0.5	0.45	0.49	0.49	1.94
INDIANA	0.46	0.4	0.46	0.28	1.6
IOWA	0.49	0.62	0.62	0.7	2.43
KANSAS	0.38	0.44	0.28	0.37	1.46
KENTUCKY	0.25	0.23	0.22	0.21	0.92
LOUISIANA	0.31	0.33	0.31	0.27	1.21
MAINE	0.24	0.51	0.79	0.97	2.51
MARYLAND	0.72	0.77	0.66	0.7	2.85
MASSACHUSETTS	0.68	0.63	0.66	0.6	2.57
MICHIGAN	0.23	0.23	0.22	0.25	0.93
MINNESOTA	1.48	1.64	1.2	1.13	5.46
MISSISSIPPI	0.28	0.27	0.3	0.26	1.11
MISSOURI	0.82	1.04	0.27	1.05	3.17
MONTANA	0.12	0.08	0.04	0.04	0.28
NEBRASKA	0.33	0.48	0.48	0.51	1.8
NEVADA	0.88	1.09	0.82	0.83	3.62
NEW HAMPSHIRE	0.61	0.58	0.52	0.27	1.98
NEW JERSEY	0.43	0.35	0.36	0.43	1.57
NEW MEXICO	1.33	1.01	0.62	0.23	3.2
NEW YORK	0.38	0.42	0.4	0.48	1.68
NORTH CAROLINA	0.5	0.59	0.44	0.45	1.98
NORTH DAKOTA	0.42	1.14	0.9	1.08	3.53
OHIO	0.67	0.7	0.42	0.81	2.6
OKLAHOMA	0.63	0.44	0.55	0.44	2.06
OREGON	1.15	0.86	0.5	0.82	3.34
PENNSYLVANIA	0.93	1.06	0.91	0.95	3.85
RHODE ISLAND	0.46	0.53	0.27	0.34	1.6
SOUTH CAROLINA	0.4	0.26	0.36	0.29	1.3
SOUTH DAKOTA	1.92	2.71	2.12	1.78	8.54
TENNESSEE	0.16	0.2	0.2	0.24	0.8
TEXAS	0.46	0.46	0.36	0.41	1.7
UTAH	0.92	0.84	0.96	1.05	3.77
VERMONT	0.13	0.32	0.32	0.39	1.16
VIRGINIA	0.64	0.51	0.5	0.55	2.19
WASHINGTON	1.35	1.42	1.36	1.54	5.67
WEST VIRGINIA	0.17	0.24	0.3	0.17	0.89
WISCONSIN	1.08	1.1	0.74	0.55	3.47
WYOMING	1.07	1.28	0.86	0.57	3.78
U.S. TOTAL	0.58	0.6	0.51	0.54	2.23

DRUG CODE:1105D DRUG NAME: D-METHAMPHETAMINE

STATE	1ST QUARTER	2ND QUARTER	3RD QUARTER	4TH QUARTER	TOTAL GRAMS
ALABAMA	0	0.02	0.14	0.15	0.31
ALASKA	0	0	0.11	0	0.11
ARIZONA	0	0.07	0.06	0.18	0.31
CALIFORNIA	0	0.06	0.16	0.16	0.38
CONNECTICUT	0	0	0.1	0.12	0.22
DISTRICT OF COLUMBIA	0	0.13	0.47	0.27	0.87
FLORIDA	0	0.03	0.02	0.02	0.07
GEORGIA	0	0.02	0.05	0.05	0.12
HAWAII	0	0	0.12	0.12	0.24
IDAHO	0	0.08	0.33	0.26	0.67
ILLINOIS	0	0	0.03	0.02	0.05
KANSAS	0	0.03	0.03	0.1	0.15
KENTUCKY	0	0.01	0.04	0.04	0.08
LOUISIANA	0	0	0.04	0.05	0.1
MARYLAND	0	0	0.02	0.06	0.08
MASSACHUSETTS	0	0.01	0.06	0.04	0.1
MINNESOTA	0	0.08	0.48	0.43	1
MISSISSIPPI	0	0	0.05	0.03	0.08
MISSOURI	0	0.05	0.2	0.28	0.54
NEBRASKA	0	0	0	0.04	0.04
NEVADA	0	0	0.01	0	0.01
NEW JERSEY	0	0.01	0.03	0.02	0.06
NEW YORK	0.02	0	0.04	0.04	0.09
NORTH CAROLINA	0	0.02	0.1	0.11	0.23
NORTH DAKOTA	0	0	0.84	0.06	0.9
OHIO	0	0	0.07	0.11	0.18
OKLAHOMA	0	0.01	0.11	0.07	0.19
OREGON	0	0.24	0.37	0.38	0.99
PENNSYLVANIA	0	0.03	0.09	0.09	0.21
RHODE ISLAND	0	0	0.15	0.19	0.34
SOUTH CAROLINA	0	0.04	0.09	0.06	0.19
TENNESSEE	0	0	0	0.01	0.01
TEXAS	0	0.02	0.07	0.07	0.16
WASHINGTON	0	0	0.01	0.02	0.04
WEST VIRGINIA	0	0	0.13	0.07	0.2
WISCONSIN	0	0.03	0.24	0.23	0.49
U.S. TOTAL	0	0.02	0.08	0.08	0.18

DRUG CODE:1205 DRUG NAME: LISDEXAMFETAMINE

STATE	1ST QUARTER	2ND QUARTER	3RD QUARTER	4TH QUARTER	TOTAL GRAMS
ALABAMA	1,529.88	1,443.77	1,459.71	1,536.11	5,969.47
ALASKA	333.24	339.83	356.84	359.2	1,389.11
ARIZONA	557.77	539.98	548.83	596.54	2,243.12
ARKANSAS	1,095.19	985.37	994.79	1,081.26	4,156.62
CALIFORNIA	298.66	300.04	292.03	307.24	1,197.97
COLORADO	543.21	544.73	546.72	573.1	2,207.76
CONNECTICUT	1,005.74	1,022.02	976.56	1,045.70	4,050.02
DELAWARE	1,602.57	1,584.87	1,530.53	1,654.08	6,372.06
DISTRICT OF COLUMBIA	852.08	858.61	874.37	906.56	3,491.62
FLORIDA	765.77	718.48	684.35	750.35	2,918.95
GEORGIA	1,090.16	1,020.96	1,043.69	1,072.98	4,227.79
GUAM	21.06	15.23	6.8	7.78	50.87
HAWAII	99.15	95.92	97.92	100.93	393.91

IDAHO	541.74	558.12	560.26	597.95	2,258.07
ILLINOIS	554.8	541.54	541.43	571.32	2,209.09
INDIANA	1,331.93	1,287.51	1,295.01	1,350.51	5,264.95
IOWA	1,155.06	1,116.94	1,092.28	1,153.90	4,518.18
KANSAS	1,107.71	1,091.78	1,087.04	1,170.09	4,456.62
KENTUCKY	1,013.46	952.51	956.06	987.1	3,909.12
LOUISIANA	2,161.99	1,956.50	1,979.78	2,093.42	8,191.69
MAINE	1,226.04	1,266.21	1,278.31	1,296.63	5,067.18
MARYLAND	983.29	966.38	925.7	994.98	3,870.35
MASSACHUSETTS	543.21	553.19	522.68	563.35	2,182.43
MICHIGAN	609.83	593.69	579.12	623.89	2,406.53
MINNESOTA	599.63	608.02	599.7	581.06	2,388.41
MISSISSIPPI	1,373	1,250.81	1,275.98	1,354.86	5,254.65
MISSOURI	1,133.92	1,082.33	1,086.42	1,142.41	4,445.08
MONTANA	590.71	559.45	562.84	602.51	2,315.52
NEBRASKA	898.89	871.61	880.23	950.23	3,600.96
NEVADA	288.82	279.64	266.87	285.87	1,121.20
NEW HAMPSHIRE	1,059.75	1,073.98	1,030.37	1,062.21	4,226.31
NEW JERSEY	558.05	566.71	539.77	564.34	2,228.88
NEW MEXICO	228.81	212.16	199.19	198.04	838.19
NEW YORK	432.76	438.07	422.87	452.7	1,746.40
NORTH CAROLINA	1,094.87	1,063.29	1,026.66	1,120.92	4,305.73
NORTH DAKOTA	881.95	820.15	831.49	870	3,403.58
OHIO	999.79	946.66	939.43	984.57	3,870.44
OKLAHOMA	989.38	941.13	956.09	1,023.38	3,909.98
OREGON	387.9	393.78	383.49	404.04	1,569.21
PENNSYLVANIA	678.56	669.46	657.93	699.06	2,705.01
PUERTO RICO	56.91	50.07	46.44	50.71	204.13
RHODE ISLAND	706.75	729.21	681.37	743.44	2,860.77
SOUTH CAROLINA	1,358.05	1,277.89	1,255.28	1,393.36	5,284.58
SOUTH DAKOTA	1,406.94	1,338.71	1,291.49	1,346.87	5,384.02
TENNESSEE	1,141.10	1,072.97	1,081.63	1,125.90	4,421.60
TEXAS	1,123.74	1,075.01	1,027.32	1,104.62	4,330.69
UTAH	774.21	763.84	763.9	831.52	3,133.48
VERMONT	1,098.75	1,085.54	1,084.62	1,158.53	4,427.44
VIRGIN ISLANDS	71.59	62.11	43.69	66.32	243.72
VIRGINIA	862.86	851.94	813.36	858.68	3,386.84
WASHINGTON	497.86	511.57	501.26	516.39	2,027.09
WEST VIRGINIA	1,107.44	1,045.04	1,018	1,070.63	4,241.11
WISCONSIN	1,143.79	1,157.28	1,150.43	1,253.13	4,704.63
WYOMING	479.3	453.97	455.71	465.15	1,854.12
U.S. TOTAL	791.4	765.19	751.87	798.98	3,107.44

DRUG CODE:1724 DRUG NAME: METHYLPHENIDATE (DL;D;L;ISOMERS)

STATE	1ST QUARTER	2ND QUARTER	3RD QUARTER	4TH QUARTER	TOTAL GRAMS
ALABAMA	1,599.29	1,463.24	1,449.31	1,573.62	6,085.47
ALASKA	1,169.80	1,117.76	1,100.06	1,137.53	4,525.14
AMERICAN SAMOA	3.95	9.22	6.59	7.25	27.01
ARIZONA	1,202.18	1,122.35	1,124.10	1,215.31	4,663.95
ARKANSAS	1,465.69	1,333.56	1,302.70	1,388.63	5,490.58
CALIFORNIA	751.65	729.56	696.22	743.02	2,920.45
COLORADO	1,251.92	1,201.21	1,184.30	1,261.87	4,899.30
CONNECTICUT	1,557.16	1,539.07	1,413.85	1,542.25	6,052.32
DELAWARE	2,163.05	2,105.83	1,966	2,128.29	8,363.17
DISTRICT OF COLUMBIA	1,354.35	1,375.41	1,254.23	1,367.31	5,351.30
FLORIDA	1,184.74	1,094.50	983.42	1,113.49	4,376.15
GEORGIA	1,811.68	1,592.83	1,614.50	1,728.81	6,747.82
GUAM	315.01	221	355.18	181.93	1,073.11
HAWAII	800.82	803.72	792.68	810.63	3,207.84

IDAHO	1,785.04	1,771.65	1,645.65	1,788.49	6,990.84
ILLINOIS	1,426.76	1,333.81	1,280.80	1,385.53	5,426.90
INDIANA	1,981.94	1,835.66	1,796.19	1,871	7,484.79
IOWA	2,800.12	2,669.23	2,531.41	2,703.07	10,703.82
KANSAS	1,834.12	1,739.26	1,675.86	1,769.51	7,018.75
KENTUCKY	1,842.60	1,649.18	1,669.02	1,756.51	6,917.32
LOUISIANA	2,056.13	1,739.80	1,747.81	1,927.97	7,471.71
MAINE	2,688.19	2,689.41	2,605.51	2,648.39	10,631.51
MARYLAND	1,813.38	1,750.26	1,612.78	1,755.17	6,931.58
MASSACHUSETTS	2,286.68	2,282.98	2,089.79	2,269.74	8,929.19
MICHIGAN	1,989.45	1,905.46	1,793.19	1,949.53	7,637.63
MINNESOTA	1,949.15	1,896.03	1,781.73	1,977.67	7,604.57
MISSISSIPPI	1,565.89	1,351.19	1,385.64	1,470.87	5,773.60
MISSOURI	2,257.23	2,106.29	2,145.46	2,280.15	8,789.14
MONTANA	1,854.41	1,735.93	1,714.02	1,768.08	7,072.45
NEBRASKA	1,808.34	1,676.32	1,628.54	1,738.76	6,851.97
NEVADA	570.51	547.99	517.58	557.59	2,193.67
NEW HAMPSHIRE	2,613.82	2,638.62	2,474.94	2,575.94	10,303.31
NEW JERSEY	1,094.90	1,060.26	957.02	1,059.62	4,171.80
NEW MEXICO	972.25	915.95	906.83	908.89	3,703.92
NEW YORK	1,212.49	1,202.45	1,082.67	1,198.83	4,696.44
NORTH CAROLINA	2,153.19	1,989.88	1,840.31	2,009.72	7,993.09
NORTH DAKOTA	2,333.28	2,211	2,111.30	2,284.26	8,939.85
OHIO	2,089.62	1,940.20	1,921.97	2,082.66	8,034.45
OKLAHOMA	1,369.85	1,236.67	1,244.56	1,307.40	5,158.48
OREGON	1,626.91	1,653.59	1,518.56	1,619.62	6,418.68
PENNSYLVANIA	1,809.10	1,768.38	1,687.84	1,818.51	7,083.83
PUERTO RICO	604.77	479.46	377.11	360.16	1,821.49
RHODE ISLAND	1,808.93	1,857.34	1,678.12	1,809.48	7,153.87
SOUTH CAROLINA	2,059.12	1,836.50	1,795.46	1,957.46	7,648.53
SOUTH DAKOTA	3,068.76	2,478.58	2,821.10	3,052.28	11,420.71
TENNESSEE	1,397.63	1,274.89	1,274.72	1,341.72	5,288.96
TEXAS	1,429.32	1,326.92	1,213.72	1,346.18	5,316.15
UTAH	1,727.67	1,637.82	1,585.32	1,712.30	6,663.12
VERMONT	3,076.60	3,096.43	2,982.38	3,138.39	12,293.80
VIRGIN ISLANDS	191.22	164.4	90.26	97.89	543.76
VIRGINIA	1,754.57	1,686.11	1,562.37	1,693.40	6,696.44
WASHINGTON	1,692.10	1,704.78	1,563.60	1,684.67	6,645.16
WEST VIRGINIA	1,810.93	1,667.53	1,637.38	1,704.29	6,820.14
WISCONSIN	1,883.43	1,811.89	1,713.32	1,865.24	7,273.88
WYOMING	1,551.15	1,410.03	1,382.98	1,435.55	5,779.72
U.S. TOTAL	1,534.78	1,449.28	1,378.44	1,490.88	5,853.38

DRUG CODE:2012 DRUG NAME: GAMMA HYDROXYBUTYRIC ACID(FDA APPROVED-CSA III)

STATE	1ST QUARTER	2ND QUARTER	3RD QUARTER	4TH QUARTER	TOTAL GRAMS
CALIFORNIA	4.19	3.59	3.59	10.17	21.54
ILLINOIS	4.05	5.79	5.21	5.79	20.85
LOUISIANA	6.56	3.28	0	0	9.83
MASSACHUSETTS	0	23.83	4.54	12.48	40.85
MICHIGAN	6.01	0	3.01	4.51	13.53
MISSOURI	153,970.83	91,683.25	150,116.01	112,033.04	507,803.13
NEW YORK	0	0	15.34	25.69	41.03
NORTH CAROLINA	2.34	14.81	21.04	14.03	52.21
OHIO	10.31	18.03	16.75	19.32	64.41
SOUTH CAROLINA	20.88	59.44	8.03	75.5	163.86
TENNESSEE	9.37	9.37	9.37	9.37	37.47
TEXAS	16.55	11.82	8.86	7.09	44.32
VIRGINIA	0	27.86	20.43	27.86	76.15
WASHINGTON	5.52	12.15	8.84	6.63	33.15

U.S. TOTAL	2,951.07	1,760.56	2,878.69	2,152.15	9,742.47

DRUG CODE:2100 DRUG NAME: BARBITURIC ACID DERIVIATIVE OR SALT [PER 21CFR 1308.13(C)(3)]

STATE	1ST QUARTER	2ND QUARTER	3RD QUARTER	4TH QUARTER	TOTAL GRAMS
ALABAMA	39.02	29.6	29.71	30.23	128.56
ALASKA	31.68	24.64	23.23	21.12	100.67
ARIZONA	91.05	84.17	70.87	70.71	316.8
ARKANSAS	56.24	45.78	43.38	37.21	182.62
CALIFORNIA	40.38	37.85	43.24	37.39	158.87
COLORADO	39.27	36.59	28.63	26.45	130.94
CONNECTICUT	60.43	58.34	68.55	56.38	243.7
DELAWARE	53.46	42.32	54.57	54.57	204.91
DISTRICT OF COLUMBIA	46.53	29.91	47.36	37.39	161.2
FLORIDA	107.09	85.85	92.79	85.77	371.49
GEORGIA	64.88	61.68	49.29	41.19	217.03
GUAM	0	5.6	0	0	5.6
HAWAII	56.61	68.37	69.1	54.4	248.47
IDAHO	40.83	41.15	28.39	27.75	138.11
ILLINOIS	57.32	56.82	62.16	59.35	235.65
INDIANA	52.82	50.9	51.13	48.12	202.97
IOWA	23.63	22.16	21.34	24.13	91.26
KANSAS	45.74	52.22	36.1	34.87	168.94
KENTUCKY	47.47	45.97	40.07	33.76	167.28
LOUISIANA	74.34	73.23	83.49	65.07	296.14
MAINE	36.13	28.23	30.49	32.37	127.22
MARYLAND	45.21	39.84	43.65	36.55	165.24
MASSACHUSETTS	40.09	39.02	48.64	37.49	165.25
MICHIGAN	62.12	72.09	71.73	66.32	272.27
MINNESOTA	26.21	24.89	25.36	23.76	100.21
MISSISSIPPI	40.44	35.39	36.4	23.25	135.48
MISSOURI	69.8	75.39	65.29	53.68	264.15
MONTANA	49.52	54.58	59.63	37.9	201.63
NEBRASKA	39.15	38.6	43.8	41.07	162.62
NEVADA	185.89	179.04	155.34	156.45	676.71
NEW HAMPSHIRE	36.84	38.74	36.08	39.88	151.54
NEW JERSEY	52.09	47.83	57.61	48.45	205.99
NEW MEXICO	40.79	40.55	41.76	38.36	161.47
NEW YORK	24.36	22.65	22.76	20.98	90.75
NORTH CAROLINA	40.64	38.96	42.84	37.65	160.09
NORTH DAKOTA	25.28	25.28	18.58	14.87	84
OHIO	46.98	45.25	29.69	23.66	145.58
OKLAHOMA	71.04	71.97	81.44	75.57	300.03
OREGON	31.45	29.1	21.66	18.66	100.89
PENNSYLVANIA	45.19	46.02	43.77	35.66	170.64
PUERTO RICO	3.62	4.03	4.43	4.16	16.24
RHODE ISLAND	60.33	57	65.55	56.53	239.41
SOUTH CAROLINA	56.64	47.67	51.35	47.46	203.12
SOUTH DAKOTA	33.16	27.02	27.64	23.34	111.15
TENNESSEE	45.07	45.93	45.38	41.76	178.14
TEXAS	49.93	50.27	52.87	43.01	196.08
UTAH	58.97	61.87	40.7	44.14	205.69
VERMONT	15.98	9.59	13.58	6.39	45.55
VIRGIN ISLANDS	18.21	40.97	22.76	13.66	95.61
VIRGINIA	45.87	49.56	39.31	39	173.73
WASHINGTON	40.3	40.15	31.68	25.28	137.41
WEST VIRGINIA	35.89	29.68	26.98	21.86	114.41
WISCONSIN	28.49	25.5	30.77	27.08	111.83
WYOMING	76.29	70.08	62.1	62.1	270.57

U.S. TOTAL	51.15	48.68	48.59	43.01	191.43

DRUG CODE:2125 DRUG NAME: AMOBARBITAL (SCHEDULE 2)

STATE	1ST QUARTER	2ND QUARTER	3RD QUARTER	4TH QUARTER	TOTAL GRAMS
ARIZONA	0.2	0.18	0.2	0.13	0.71
ARKANSAS	0	0.02	0.02	0	0.03
CALIFORNIA	0.07	0.06	0.08	0.06	0.27
COLORADO	0.55	0.32	0.29	0.2	1.36
CONNECTICUT	0	0.06	0	0.04	0.1
DISTRICT OF COLUMBIA	0.08	0	0.3	0.76	1.14
FLORIDA	0.02	0.01	0.05	0.04	0.12
GEORGIA	0.02	0.01	0.11	0.14	0.27
ILLINOIS	0.04	0.01	0.05	0.03	0.12
INDIANA	0	0	0.04	0.03	0.06
IOWA	0	0	0.01	0	0.01
KANSAS	0.06	0.18	0.02	0.22	0.48
KENTUCKY	0.11	0.08	0.08	0.17	0.44
MAINE	0	0	0.03	0.17	0.21
MARYLAND	0.13	0.06	0.11	0.18	0.47
MASSACHUSETTS	0.15	0.03	0.01	0.12	0.31
MICHIGAN	0.06	0.04	0.06	0.03	0.19
MINNESOTA	0.09	0.06	0.14	0.15	0.44
MISSISSIPPI	0.06	0	0.05	0.05	0.15
MISSOURI	0.01	0.02	0.05	0.05	0.11
NEBRASKA	0.15	0	0	0.05	0.2
NEW HAMPSHIRE	0.31	0.14	0.21	0.28	0.93
NEW JERSEY	0.04	0.04	0.08	0.19	0.35
NEW YORK	0.28	0.11	0.14	0.25	0.79
NORTH CAROLINA	0.17	0.05	0.06	0.12	0.4
OHIO	0.03	0.03	0.05	0.05	0.15
OREGON	0.04	0.04	0.04	0.07	0.18
PENNSYLVANIA	0.11	0.02	0.03	0.07	0.23
RHODE ISLAND	0.35	0	0	0	0.35
SOUTH CAROLINA	0.03	0.09	0.12	0.09	0.33
TENNESSEE	0.06	0.08	0.08	0.03	0.25
TEXAS	0.1	0.05	0.11	0.07	0.33
UTAH	0.07	0.49	0.53	0.15	1.24
VERMONT	0	0	0.15	0	0.15
VIRGINIA	0.05	0.03	0.03	0.03	0.14
WASHINGTON	0.07	0	0.17	0.31	0.55
WEST VIRGINIA	0	0	0	0.1	0.1
WISCONSIN	0.11	0.06	0.13	0.18	0.47
U.S. TOTAL	0.09	0.05	0.08	0.09	0.31

DRUG CODE:2165 DRUG NAME: BUTALBITAL

STATE	1ST QUARTER	2ND QUARTER	3RD QUARTER	4TH QUARTER	TOTAL GRAMS
ALABAMA	92.37	99.27	96.55	84.21	372.41
ALASKA	25.34	37.31	38.72	28.86	130.24
AMERICAN SAMOA	15.24	0	0	0	15.24
ARIZONA	73.69	73.61	85.97	88	321.26
ARKANSAS	113	117.8	120.72	120.55	472.06
CALIFORNIA	66.11	66.95	67.27	63.56	263.89
COLORADO	34.1	36.49	39.47	45.24	155.29
CONNECTICUT	91.07	85.06	93.73	74.28	344.14
DELAWARE	62.37	61.25	55.13	56.24	234.98
DISTRICT OF COLUMBIA	52.35	58.17	65.64	56.5	232.67

FLORIDA	78.9	88.42	91.56	90.66	349.55
GEORGIA	94.66	99.71	109.47	106.99	410.83
GUAM	14.01	2.8	2.8	2.8	22.42
HAWAII	81.6	79.03	76.82	68.37	305.81
IDAHO	75.28	79.1	91.22	88.99	334.59
ILLINOIS	56.9	60.32	58.06	55.88	231.17
INDIANA	58.76	56.76	64.62	60.38	240.52
IOWA	40.38	42.35	43.82	40.87	167.41
KANSAS	77.81	65.72	90.43	74.66	308.61
KENTUCKY	54.96	55.54	65.22	63.14	238.86
LOUISIANA	91.54	89.34	84.59	81.95	347.42
MAINE	56.46	47.8	45.92	39.52	189.71
MARYLAND	84	77.77	84.78	82.36	328.91
MASSACHUSETTS	38.64	36.73	35.89	30.77	142.04
MICHIGAN	93.74	85.95	90.45	89.24	359.38
MINNESOTA	28.94	30.92	30.07	36.48	126.42
MISSISSIPPI	71.78	71.78	73.64	70.77	287.97
MISSOURI	90.25	95.59	101.94	108.78	396.57
MONTANA	74.29	77.82	88.94	70.24	311.3
NEBRASKA	57.49	59.13	60.5	65.71	242.84
NEVADA	127.2	126.64	149.04	145.71	548.59
NEW HAMPSHIRE	47.48	46.72	53.93	54.31	202.44
NEW JERSEY	74.73	70.41	71.71	63.75	280.6
NEW MEXICO	39.82	49.05	48.32	42.49	179.68
NEW YORK	30.78	31.35	29.75	25.65	117.53
NORTH CAROLINA	53.9	52.75	53.54	53.64	213.83
NORTH DAKOTA	46.09	49.81	45.35	49.81	191.05
OHIO	51.27	47.28	64.97	67.31	230.83
OKLAHOMA	145.15	140.62	128.62	118.49	532.87
OREGON	61.86	54.16	62.38	66.56	244.97
PENNSYLVANIA	94.63	90.1	95.3	88.41	368.43
RHODE ISLAND	35.63	39.43	34.2	30.4	139.66
SOUTH CAROLINA	71.24	73.62	74.16	72.43	291.44
SOUTH DAKOTA	81.06	77.99	79.83	79.83	318.73
TENNESSEE	55.62	57.12	63.42	64.37	240.54
TEXAS	74.71	72.18	73.35	77.21	297.45
UTAH	110.53	98.95	124.1	126.63	460.22
VERMONT	23.97	13.58	15.18	22.37	75.11
VIRGIN ISLANDS	0	0	18.21	22.76	40.97
VIRGINIA	69.3	64.43	64.55	62.55	260.84
WASHINGTON	55.99	62.9	66.1	73.83	258.83
WEST VIRGINIA	65.57	66.92	73.12	68	273.61
WISCONSIN	29.37	30.68	34.11	28.84	123
WYOMING	60.32	66.53	83.39	78.95	289.2
U.S. TOTAL	67.05	67	70.4	68.53	272.98

DRUG CODE:2270 DRUG NAME: PENTOBARBITAL (SCHEDULE 2)

STATE	1ST QUARTER	2ND QUARTER	3RD QUARTER	4TH QUARTER	TOTAL GRAMS
ALABAMA	233.17	303.37	467.98	367.8	1,372.33
ALASKA	800.74	703.47	875.81	828.98	3,209.01
ARIZONA	702.21	697.71	883.04	1,081.64	3,364.61
ARKANSAS	259.02	476.38	305.78	208.07	1,249.25
CALIFORNIA	465.5	465.07	508.61	463.41	1,902.60
COLORADO	939.99	767.17	656.58	1,204.53	3,568.28
CONNECTICUT	416.58	406.53	600.5	603.54	2,027.14
DELAWARE	87.7	220.07	190.38	298	796.15
DISTRICT OF COLUMBIA	32.56	1.51	144.8	245.37	424.25
FLORIDA	346.33	405.1	374.67	387.66	1,513.76
GEORGIA	382.18	349.79	399.36	456.4	1,587.74

GUAM	0	1,802.06	0	1,802.06	3,604.12
HAWAII	340.46	90.45	1,266.23	357.11	2,054.25
IDAHO	1,029.70	1,282.17	1,815.86	1,642.27	5,770
ILLINOIS	437.69	468.17	464.05	791.03	2,160.94
INDIANA	636.63	586.85	669.96	879.38	2,772.83
IOWA	370.41	544.12	465.95	966.69	2,347.17
KANSAS	761.32	506.38	747.78	1,156.15	3,171.62
KENTUCKY	471.89	527.43	811.99	754.58	2,565.89
LOUISIANA	547.51	473.92	601.42	590.41	2,213.26
MAINE	1,605.45	1,288.58	1,460.91	1,733.80	6,088.75
MARYLAND	398.82	253.98	412.89	474.62	1,540.31
MASSACHUSETTS	331.76	401.71	484.3	504.36	1,722.13
MICHIGAN	905.89	790.33	1,113.82	1,084.68	3,894.73
MINNESOTA	494.5	680.59	588.06	968.41	2,731.56
MISSISSIPPI	374.96	186.2	211.7	323.2	1,096.06
MISSOURI	617.02	435.9	787.36	848.47	2,688.76
MONTANA	1,257.59	1,060.43	2,174.01	2,999.82	7,491.84
NEBRASKA	685.94	1,044.10	1,122.57	1,662.86	4,515.47
NEVADA	452.67	427.98	676.93	729.19	2,286.78
NEW HAMPSHIRE	901.74	1,264.51	1,275.54	1,262.09	4,703.89
NEW JERSEY	214.17	178.75	299.45	239.38	931.75
NEW MEXICO	674.45	880	1,287.25	1,058.06	3,899.76
NEW YORK	289.78	395.75	432.84	462.78	1,581.14
NORTH CAROLINA	537.07	583.44	637.04	785.95	2,543.49
NORTH DAKOTA	1,297.47	966.09	712.29	1,241.91	4,217.75
OHIO	518.74	569.88	678.08	791.45	2,558.16
OKLAHOMA	364.77	573.48	375.88	714.81	2,028.94
OREGON	756.27	921.97	1,148.32	1,211.13	4,037.69
PENNSYLVANIA	421.37	497.55	690.48	509.8	2,119.22
PUERTO RICO	0	0	0.01	0	0.01
RHODE ISLAND	304.75	659.9	692.64	676.29	2,333.57
SOUTH CAROLINA	271.96	277.91	300.97	353.7	1,204.55
SOUTH DAKOTA	710.04	666.44	819.97	873.25	3,069.70
TENNESSEE	419.75	651.39	554.74	621.06	2,246.95
TEXAS	419.21	429.33	566.22	560.12	1,974.87
UTAH	348.96	407.82	481.37	821.09	2,059.24
VERMONT	1,565.44	1,496.55	2,785.77	2,839.89	8,687.66
VIRGINIA	535.63	590.22	589.36	671.86	2,387.07
WASHINGTON	826.2	797.26	847.9	1,109.84	3,581.21
WEST VIRGINIA	376.19	678.53	769.53	447.83	2,272.08
WISCONSIN	720.44	765.98	916.48	1,279.56	3,682.45
WYOMING	804.07	2,239.75	1,613.96	2,522.57	7,180.34
U.S. TOTAL	483.42	511.25	602.64	673.81	2,271.12

DRUG CODE:2315 DRUG NAME: SECOBARBITAL (SCHEDULE 2)

STATE	1ST QUARTER	2ND QUARTER	3RD QUARTER	4TH QUARTER	TOTAL GRAMS
ARIZONA	0.14	0	0	0.14	0.29
CALIFORNIA	2.41	2.65	3.12	3.56	11.75
COLORADO	2.18	1.82	2.18	1.27	7.46
CONNECTICUT	1.02	0.77	0.26	0.51	2.56
DELAWARE	0	1.02	1.02	1.02	3.06
FLORIDA	0.54	0.39	0.54	0.49	1.95
GEORGIA	0.38	0.09	0	0	0.47
HAWAII	0.67	0	0	0	0.67
ILLINOIS	0.36	0.43	0.29	0.21	1.28
KANSAS	1.28	0.64	0.64	0.96	3.53
MARYLAND	1.11	1.11	0.79	0.79	3.81
MASSACHUSETTS	0.84	0.7	0.56	0.84	2.94
MICHIGAN	0	0	0	0.19	0.19

MINNESOTA	0.17	0	0	0.17	0.35
MISSOURI	0.46	0.31	0.61	0.31	1.68
NEVADA	0.34	0	0	0	0.34
NEW HAMPSHIRE	1.39	0.7	0	0	2.09
NEW JERSEY	0.83	0.62	0.62	0.52	2.6
NEW YORK	2.41	2.08	1.51	1.75	7.75
NORTH CAROLINA	0	0.1	0	0	0.1
OHIO	0.48	0.16	0.24	0.32	1.19
OKLAHOMA	0	0	0.73	0.24	0.98
OREGON	6.69	4.54	5.74	7.89	24.85
PENNSYLVANIA	0.29	0.58	0.65	0.58	2.09
RHODE ISLAND	0.87	0.87	0	1.74	3.48
SOUTH CAROLINA	0	0	0.4	0.4	0.79
SOUTH DAKOTA	0	0	0	1.12	1.12
TENNESSEE	0	0.14	0	0	0.14
TEXAS	0.04	0	0.04	0.11	0.18
VERMONT	8.78	4.39	0	8.78	21.95
VIRGINIA	0.34	0.34	0.34	0.34	1.37
WASHINGTON	3.4	2.59	2.31	2.45	10.76
WISCONSIN	0.16	0	0	0	0.16
U.S. TOTAL	0.86	0.76	0.79	0.91	3.32

DRUG CODE:7365 DRUG NAME: DRONABINOL IN AN ORAL SOLUTION IN FDA APPROVED
DRUG PRODUCT (SYNDROS - CII)

STATE	1ST QUARTER	2ND QUARTER	3RD QUARTER	4TH QUARTER	TOTAL GRAMS
ALABAMA	0	0	0.03	0.06	0.09
ARIZONA	0	0	0.23	0.11	0.34
ARKANSAS	0	0	0.02	0.02	0.04
CALIFORNIA	0	0	0.04	0.08	0.12
COLORADO	0	0	0.07	0	0.07
CONNECTICUT	0	0	0.03	0.01	0.05
DELAWARE	0	0	0.02	0	0.02
DISTRICT OF COLUMBIA	0	0	0	0.12	0.12
FLORIDA	0	0	0.07	0.12	0.19
GEORGIA	0	0	0.01	0.03	0.05
ILLINOIS	0	0	0	0.01	0.01
INDIANA	0	0	0.03	0	0.03
KANSAS	0	0	0	0.02	0.02
KENTUCKY	0	0	0.02	0.02	0.03
LOUISIANA	0	0	0	0	0.01
MAINE	0	0	0.02	0	0.02
MARYLAND	0	0	0	0.03	0.03
MASSACHUSETTS	0	0	0.01	0.05	0.06
MICHIGAN	0	0	0.01	0.01	0.02
MINNESOTA	0	0	0	0.05	0.05
MISSISSIPPI	0	0	0.02	0.01	0.03
MISSOURI	0	0	0.07	0.07	0.14
MONTANA	0	0	0	0.03	0.03
NEVADA	0	0	0.18	0.24	0.43
NEW HAMPSHIRE	0	0	0.06	0.08	0.14
NEW JERSEY	0	0	0.81	0.83	1.63
NEW MEXICO	0	0	0.01	0	0.01
NEW YORK	0	0	0	0.01	0.01
NORTH CAROLINA	0	0	0	0.01	0.01
OHIO	0	0	0	0.07	0.08
PENNSYLVANIA	0	0	0.11	0.02	0.13
SOUTH CAROLINA	0	0	0.01	0	0.01
TENNESSEE	0	0	0.02	0.03	0.06
TEXAS	0	0	0.03	0.02	0.05

UTAH	0	0	0.02	0.01	0.02
VIRGINIA	0	0	0	0.01	0.01
WISCONSIN	0	0	0	0	0
U.S. TOTAL	0	0	0.05	0.06	0.11

DRUG CODE:7379 DRUG NAME: NABILONE

STATE	1ST QUARTER	2ND QUARTER	3RD QUARTER	4TH QUARTER	TOTAL GRAMS
ARIZONA	0	0	0	0	0
CALIFORNIA	0	0	0	0	0
COLORADO	0	0	0	0	0.01
CONNECTICUT	0	0	0	0	0.01
FLORIDA	0.01	0	0	0	0.01
HAWAII	0.03	0	0.01	0	0.04
ILLINOIS	0	0	0	0	0
IOWA	0	0	0	0	0
KENTUCKY	0	0	0	0	0.01
MAINE	0.01	0.01	0.01	0	0.02
MARYLAND	0.02	0.02	0.02	0.02	0.07
MINNESOTA	0	0	0	0	0
MISSOURI	0	0	0	0	0.01
NEBRASKA	0.01	0	0	0.01	0.02
NEW HAMPSHIRE	0	0	0	0	0
NEW JERSEY	0	0	0	0	0
NEW MEXICO	0	0	0	0	0
NEW YORK	0	0	0	0	0
NORTH CAROLINA	0	0	0	0	0.01
OHIO	0	0	0	0.01	0.01
OREGON	0	0	0	0	0
PENNSYLVANIA	0	0	0	0	0
VIRGINIA	0	0	0	0	0.01
WASHINGTON	0	0	0	0	0
U.S. TOTAL	0	0	0	0	0

DRUG CODE:9041L DRUG NAME: COCAINE

STATE	1ST QUARTER	2ND QUARTER	3RD QUARTER	4TH QUARTER	TOTAL GRAMS
ALABAMA	1.31	1.38	1.04	1.06	4.78
ALASKA	1.21	2.79	1.35	1.41	6.75
ARIZONA	1.73	1.72	1.56	1.56	6.56
ARKANSAS	1.93	1.4	1.31	1.38	6.02
CALIFORNIA	2.49	2.36	2.24	2.22	9.32
COLORADO	1.63	2.17	1.42	1.6	6.82
CONNECTICUT	1.09	0.9	1.4	0.82	4.22
DELAWARE	0.73	2.1	1.65	1.29	5.77
DISTRICT OF COLUMBIA	3.74	3.57	4.68	3.45	15.44
FLORIDA	1.54	1.63	1.57	1.44	6.18
GEORGIA	1.59	1.77	1.52	1.9	6.78
GUAM	0	0	0.2	0	0.2
HAWAII	2.08	1.75	1.83	1.5	7.16
IDAHO	4.12	2.39	2.99	4.32	13.81
ILLINOIS	2.55	2.78	2.13	2.6	10.06
INDIANA	3.87	3.02	2.36	3.1	12.35
IOWA	3.46	3.38	2.83	3.24	12.9
KANSAS	2.23	2.98	2.89	3.43	11.52
KENTUCKY	2.53	2.72	2.04	2.72	10.01
LOUISIANA	2.04	2.13	1.91	2.51	8.59
MAINE	1.69	1.88	1.72	1.3	6.58

MARYLAND	2.44	2.54	2.07	1.92	8.96
MASSACHUSETTS	3.49	3.1	2.76	3.11	12.45
MICHIGAN	3.72	4.27	3.41	3.86	15.26
MINNESOTA	3.99	4.4	3.76	3.98	16.13
MISSISSIPPI	2.69	3.42	2.54	2.78	11.42
MISSOURI	2.74	3.02	2.67	2.58	11.01
MONTANA	4.94	5.56	4.22	6.67	21.39
NEBRASKA	1.18	1.7	2.03	1.62	6.53
NEVADA	1.65	1.93	1.63	1.46	6.66
NEW HAMPSHIRE	2.94	4.17	3.08	4.26	14.46
NEW JERSEY	2	1.92	1.69	1.73	7.34
NEW MEXICO	3.71	3.58	3.58	3.23	14.1
NEW YORK	1.87	1.91	1.65	1.69	7.12
NORTH CAROLINA	1.42	1.49	1.59	1.49	6
NORTH DAKOTA	4.94	9.87	5.62	6.62	27.06
OHIO	1.52	1.75	1.47	1.59	6.33
OKLAHOMA	0.83	0.91	0.75	1.05	3.55
OREGON	2.7	2.5	2.62	2.21	10.04
PENNSYLVANIA	3.25	3.23	2.81	2.97	12.26
RHODE ISLAND	2.4	1.56	1.66	1.6	7.22
SOUTH CAROLINA	0.66	0.68	0.61	0.61	2.56
SOUTH DAKOTA	5.43	3.04	3.37	6.43	18.28
TENNESSEE	2.85	2.52	2.52	2.67	10.56
TEXAS	1.32	1.53	1.3	1.63	5.78
UTAH	3.74	3.57	3.25	3.59	14.15
VERMONT	1.66	2.15	1	1.34	6.15
VIRGINIA	2.02	1.91	1.94	1.81	7.68
WASHINGTON	1.7	1.55	1.85	1.65	6.75
WEST VIRGINIA	2.83	2.61	2.69	2.75	10.89
WISCONSIN	2.89	2.78	2.3	2.67	10.64
WYOMING	3.19	2.46	3.6	3.04	12.29
U.S. TOTAL	2.21	2.26	2	2.14	8.61

DRUG CODE:9050 DRUG NAME: CODEINE

STATE	1ST QUARTER	2ND QUARTER	3RD QUARTER	4TH QUARTER	TOTAL GRAMS
ALABAMA	1,089.96	1,096.85	1,084.18	1,099.36	4,370.36
ALASKA	1,044.23	995.77	991.3	882.57	3,913.86
AMERICAN SAMOA	538.82	518.61	538.82	478.2	2,074.45
ARIZONA	1,226.47	1,174.19	1,104.49	1,143.59	4,648.74
ARKANSAS	2,541.92	2,622.31	2,468.31	2,409.21	10,041.74
CALIFORNIA	1,346.01	1,288.32	1,236.24	1,207.34	5,077.90
COLORADO	527.98	514.83	505.54	497.09	2,045.43
CONNECTICUT	686.67	698.9	662.51	646.76	2,694.84
DELAWARE	1,103.25	1,060.64	971.29	983.95	4,119.13
DISTRICT OF COLUMBIA	2,118.30	2,090.25	1,995.36	1,843.06	8,046.97
FLORIDA	961.03	908.64	860.18	894.3	3,624.15
GEORGIA	1,097.86	1,127.98	1,054.59	1,057.72	4,338.15
GUAM	722.42	667.68	670.56	610.23	2,670.89
HAWAII	1,015.03	1,042.39	1,012.02	941.64	4,011.08
IDAHO	879.05	892.38	867.31	847.12	3,485.86
ILLINOIS	1,303.88	1,278.05	1,239.73	1,223.14	5,044.79
INDIANA	804.13	802.27	732.36	753.03	3,091.79
IOWA	658.56	669.79	647.49	646.84	2,622.68
KANSAS	1,016.33	996.79	938.9	966.35	3,918.38
KENTUCKY	1,121.74	1,111.55	1,096.31	1,051.86	4,381.46
LOUISIANA	998.68	1,005.73	928.33	900.96	3,833.69
MAINE	815.28	828.67	735.9	748.51	3,128.38
MARYLAND	1,076.65	1,032.14	958.77	949.5	4,017.07
MASSACHUSETTS	743.19	713.74	689.31	682.73	2,828.96

MICHIGAN	2,690.06	2,660.60	2,579.31	2,443.49	10,373.45
MINNESOTA	733.7	700.04	697.62	681.63	2,812.98
MISSISSIPPI	945.9	929.12	920.59	898.45	3,694.05
MISSOURI	1,146.33	1,170.31	1,119.12	1,135.98	4,571.74
MONTANA	1,038.43	1,062.18	999.97	1,000.93	4,101.51
NEBRASKA	666.6	644.15	637.48	623.32	2,571.55
NEVADA	1,000.76	1,016.51	956.11	986.89	3,960.27
NEW HAMPSHIRE	598.66	573.08	556.59	576.88	2,305.21
NEW JERSEY	934.87	896.63	861.66	842.08	3,535.23
NEW MEXICO	962.77	934.74	948.34	951.15	3,797
NEW YORK	760.67	751.21	715.37	702.6	2,929.85
NORTH CAROLINA	540.22	513.82	483.98	492.47	2,030.49
NORTH DAKOTA	855.28	865.09	871.54	794.74	3,386.66
OHIO	945.07	890.43	838.24	819.57	3,493.31
OKLAHOMA	2,576.47	2,582.08	2,522.65	2,499.62	10,180.82
OREGON	916.89	879.91	873.88	875.59	3,546.27
PENNSYLVANIA	1,078.54	1,132.95	1,117.92	1,035.64	4,365.05
PUERTO RICO	364.6	361.67	312.14	336.49	1,374.90
RHODE ISLAND	760.73	700.55	653.06	651.91	2,766.26
SOUTH CAROLINA	1,697.93	1,476.49	1,568.99	1,829.82	6,573.23
SOUTH DAKOTA	890.49	869.16	827.3	837.99	3,424.94
TENNESSEE	1,473.68	1,393.02	1,345.84	1,168.47	5,381
TEXAS	4,016.36	4,075.25	3,925.44	3,920.40	15,937.44
UTAH	595.38	599.99	592.58	577.26	2,365.21
VERMONT	569.81	552.75	484.39	489.11	2,096.06
VIRGIN ISLANDS	865.79	593.79	515.17	755.99	2,730.73
VIRGINIA	741.02	712.82	642.46	648.06	2,744.35
WASHINGTON	942.39	937.75	892.04	882.33	3,654.52
WEST VIRGINIA	1,677.58	1,680.14	1,633.06	1,725.98	6,716.75
WISCONSIN	891.5	839.91	807.43	811.86	3,350.71
WYOMING	774.73	715.21	732.37	752.44	2,974.75
U.S. TOTAL	1,333.63	1,315.04	1,263.89	1,251.50	5,164.06

DRUG CODE:9056 DRUG NAME: ETORPHINE

STATE	1ST QUARTER	2ND QUARTER	3RD QUARTER	4TH QUARTER	TOTAL GRAMS
WYOMING	4.97	0	0	5.15	10.11
U.S. TOTAL	0.01	0	0	0.01	0.02

DRUG CODE:9064 DRUG NAME: BUPRENORPHINE

STATE	1ST QUARTER	2ND QUARTER	3RD QUARTER	4TH QUARTER	TOTAL GRAMS
ALABAMA	496.58	526.4	531.7	553.28	2,107.96
ALASKA	306.71	331.1	342.34	351.55	1,331.70
AMERICAN SAMOA	0	0	0	0	0.01
ARIZONA	139.51	145.31	149.68	155.3	589.8
ARKANSAS	201.54	211.93	218.49	227.33	859.3
CALIFORNIA	97.13	100.06	105.49	106.87	409.53
COLORADO	139.78	144.07	151.47	153.51	588.83
CONNECTICUT	395.75	413.31	420.32	429.58	1,658.96
DELAWARE	376.82	404.69	400.87	434.21	1,616.59
DISTRICT OF COLUMBIA	353.41	376.43	409.34	432.03	1,571.21
FLORIDA	241.27	249.11	247.47	260.23	998.08
GEORGIA	153.57	162.57	164.09	168.76	648.98
GUAM	0.46	1.11	0	0.94	2.52
HAWAII	136.8	146.09	147.97	148.37	579.23
IDAHO	207.57	220.42	231.69	247.2	906.88
ILLINOIS	104.71	112.07	114.33	117.25	448.36

INDIANA	274.59	304.99	316.75	332.04	1,228.36
IOWA	52.04	53.78	55.78	59.34	220.93
KANSAS	87.41	87.87	93.26	89.78	358.33
KENTUCKY	777.73	800.81	798.4	805.16	3,182.09
LOUISIANA	398.26	418.09	434.18	432.85	1,683.38
MAINE	675.41	728.2	743.3	768	2,914.90
MARYLAND	417.86	437.63	444.67	467.61	1,767.76
MASSACHUSETTS	607.94	649.69	622.45	644.7	2,524.78
MICHIGAN	260.14	273.83	275.57	289.7	1,099.24
MINNESOTA	131.97	140.37	146.08	151.25	569.67
MISSISSIPPI	326.55	336.87	335.74	337.82	1,336.98
MISSOURI	144.11	153.76	162.03	178.39	638.29
MONTANA	224.41	234.07	252.26	264.8	975.54
NEBRASKA	69.24	71.65	74.66	75.14	290.68
NEVADA	119.93	128.74	128.17	128.44	505.28
NEW HAMPSHIRE	582.66	612.14	623.9	619.12	2,437.83
NEW JERSEY	290.58	306.21	297.4	306.38	1,200.57
NEW MEXICO	331.49	351.3	369.02	380.59	1,432.40
NEW YORK	260.54	272.5	276.46	291.53	1,101.02
NORTH CAROLINA	318.44	346.86	336.56	370.3	1,372.16
NORTH DAKOTA	126.67	131.3	140.35	142.18	540.5
OHIO	439.98	460.75	456.52	463.92	1,821.17
OKLAHOMA	271.26	276.23	306.94	311.04	1,165.47
OREGON	253.84	268.29	272.44	289.63	1,084.20
PENNSYLVANIA	474.96	497.9	489.61	522.1	1,984.57
PUERTO RICO	66.89	69	59.58	71.76	267.23
RHODE ISLAND	672.52	731.67	711.57	743.66	2,859.42
SOUTH CAROLINA	212.84	227.02	233.39	248.59	921.85
SOUTH DAKOTA	50.43	57.13	55.22	58.03	220.81
TENNESSEE	521.13	553.94	547.4	572.34	2,194.82
TEXAS	101.33	104.24	106.56	106.77	418.91
UTAH	336.41	347.1	353.22	358.49	1,395.23
VERMONT	954.23	1,023.02	1,014.18	1,038.84	4,030.27
VIRGIN ISLANDS	17.92	15.97	11.82	11.4	57.11
VIRGINIA	252.62	251.38	239.85	249.25	993.11
WASHINGTON	267.42	282.06	290.78	304.79	1,145.05
WEST VIRGINIA	804	836.92	861.99	853.31	3,356.22
WISCONSIN	173.78	179.04	180.64	189.32	722.78
WYOMING	177.87	222.99	233.67	258.42	892.95
U.S. TOTAL	254.81	267.61	269.26	279.59	1,071.27

DRUG CODE:9120 DRUG NAME: DIHYDROCODEINE

STATE	1ST QUARTER	2ND QUARTER	3RD QUARTER	4TH QUARTER	TOTAL GRAMS
ALABAMA	16.4	16.71	16.44	16.35	65.89
ALASKA	1.2	0.45	0	0.3	1.96
ARIZONA	1.15	1.35	1.19	1.3	5
ARKANSAS	3.62	5.22	6.02	4.63	19.5
CALIFORNIA	0.58	0.68	0.2	0.81	2.26
COLORADO	0	0.04	0.11	0.04	0.19
CONNECTICUT	0.03	0	0.07	0	0.1
DELAWARE	1.46	1.48	1.38	1.75	6.07
FLORIDA	0.84	0.78	1.27	1.17	4.06
GEORGIA	4.42	5.68	5.26	7.33	22.69
HAWAII	0.08	0	0	0	0.08
IDAHO	1.5	2.59	2.04	0.82	6.95
ILLINOIS	0.99	1.67	1.5	1.07	5.23
INDIANA	0.05	0	0.05	0.02	0.12
IOWA	0	0.04	0	0.04	0.07
KANSAS	0.04	0.37	0	0.22	0.64

KENTUCKY	0.71	0.44	0.32	0.49	1.97
LOUISIANA	13.7	17.71	14.21	10.69	56.3
MAINE	0	0	0.08	0	0.08
MARYLAND	0.13	0.18	0.18	0.18	0.66
MASSACHUSETTS	0.33	0.24	0.51	0.85	1.93
MICHIGAN	0.35	0	0.05	0.01	0.41
MINNESOTA	0.03	0.08	0.03	0.04	0.19
MISSISSIPPI	12.39	16.56	12.82	12.35	54.12
MISSOURI	0.77	0.71	0.91	0.7	3.09
MONTANA	0.11	0	0	0	0.11
NEBRASKA	0.18	0.12	0.21	0.15	0.66
NEVADA	1.61	1.15	0.04	0	2.79
NEW JERSEY	4.05	4.2	2.7	3.25	14.2
NEW MEXICO	6.59	4.41	4.88	0.83	16.7
NEW YORK	0.56	0.87	0.6	0.7	2.73
NORTH CAROLINA	0.15	0.1	0.07	0.09	0.4
OHIO	0.42	0.29	0.33	0.18	1.22
OKLAHOMA	7.27	7.97	7.87	7	30.11
PENNSYLVANIA	1.62	0.33	0.51	0.4	2.85
RHODE ISLAND	0	0.1	0	0.2	0.3
SOUTH CAROLINA	0.3	0.3	0.23	0.3	1.13
SOUTH DAKOTA	0	0	0	0.13	0.13
TENNESSEE	8	8.14	8.02	6.37	30.52
TEXAS	10.69	11.17	10.66	11.35	43.86
UTAH	0	0	0.04	0	0.04
VERMONT	0	0.17	0	0	0.17
VIRGINIA	0.13	0.05	0.04	0.11	0.33
WEST VIRGINIA	0.29	0.13	0	0	0.42
WISCONSIN	0.08	0.13	0.11	0.06	0.38
U.S. TOTAL	2.39	2.58	2.34	2.39	9.7

DRUG CODE:9143 DRUG NAME: OXYCODONE

STATE	1ST QUARTER	2ND QUARTER	3RD QUARTER	4TH QUARTER	TOTAL GRAMS
ALABAMA	5,126.52	4,897.47	4,662.14	4,629.60	19,315.74
ALASKA	5,056.10	4,943.01	4,751.11	4,651.30	19,401.52
AMERICAN SAMOA	21.86	18.44	16.39	32.78	89.48
ARIZONA	7,185.70	6,772.82	6,387.28	6,256.17	26,601.96
ARKANSAS	5,161.45	5,047.72	4,890.40	4,722.23	19,821.81
CALIFORNIA	2,430.13	2,359.21	2,278.42	2,200.61	9,268.36
COLORADO	4,643.96	4,538.56	4,325.63	4,240.34	17,748.50
CONNECTICUT	4,907.93	4,772.68	4,489.06	4,393.36	18,563.03
DELAWARE	8,025.03	7,642.33	7,434.31	7,391.39	30,493.06
DISTRICT OF COLUMBIA	2,511.66	2,499.98	2,419.71	2,233.56	9,664.91
FLORIDA	5,534.92	5,474.95	5,299.44	5,519.33	21,828.64
GEORGIA	4,702.10	4,660.87	4,536.95	4,501.08	18,401
GUAM	390.9	509.2	549.37	468.02	1,917.49
HAWAII	3,352.74	3,236.51	3,177.09	2,983.09	12,749.44
IDAHO	3,920.64	3,867.62	3,844.02	3,764.25	15,396.53
ILLINOIS	1,291.04	1,252.18	1,201.62	1,185.65	4,930.49
INDIANA	3,725.86	3,647.03	3,466.79	3,415.98	14,255.65
IOWA	1,839.80	1,780.25	1,684.02	1,714.93	7,019
KANSAS	4,362.75	4,336.32	4,248.76	4,173.69	17,121.53
KENTUCKY	4,855.33	4,764.23	4,594.76	4,582.93	18,797.25
LOUISIANA	4,492.87	4,324.51	4,141.36	4,031.67	16,990.42
MAINE	4,077.29	3,842.32	3,768.44	3,692.38	15,380.43
MARYLAND	5,947.71	5,757.54	5,420.26	5,310.90	22,436.40
MASSACHUSETTS	3,865.90	3,736.03	3,561.24	3,488.50	14,651.68
MICHIGAN	3,178.54	3,067.17	2,958.52	2,824.02	12,028.26
MINNESOTA	2,825.29	2,719.32	2,629.43	2,584.67	10,758.70

MISSISSIPPI	3,684.38	3,604.11	3,454.84	3,385.85	14,129.17
MISSOURI	5,173.34	5,100.81	4,825.02	4,681.81	19,780.99
MONTANA	4,144.82	3,878.68	3,759.25	3,615.36	15,398.11
NEBRASKA	2,347.67	2,311.12	2,239.93	2,204.11	9,102.83
NEVADA	6,898.72	6,737.78	6,500.37	6,424.04	26,560.91
NEW HAMPSHIRE	4,986.29	4,771.85	4,714.60	4,470.41	18,943.15
NEW JERSEY	6,131.76	5,815.66	5,459.80	5,242.19	22,649.41
NEW MEXICO	4,791.80	4,497.80	4,195.09	4,065.50	17,550.20
NEW YORK	4,060.06	3,896.12	3,707.44	3,568.60	15,232.23
NORTH CAROLINA	6,274.13	5,980.86	5,666.60	5,559.90	23,481.49
NORTH DAKOTA	2,254.48	2,224.49	2,123.88	2,108.20	8,711.05
OHIO	4,466.71	4,282.55	4,065.66	3,884.51	16,699.43
OKLAHOMA	6,180.79	6,082.39	5,889.99	5,751.35	23,904.52
OREGON	4,781.04	4,614.59	4,424.96	4,277.15	18,097.73
PENNSYLVANIA	6,192.87	6,011.37	5,775.74	5,653.92	23,633.90
PUERTO RICO	530.66	377.61	252.09	201.14	1,361.50
RHODE ISLAND	4,147.07	3,867.28	3,762.75	3,663.45	15,440.55
SOUTH CAROLINA	5,324.65	5,178.65	4,878.11	4,829.70	20,211.11
SOUTH DAKOTA	2,212.05	2,152.63	2,105.15	1,998.24	8,468.07
TENNESSEE	7,472.71	7,352.67	7,053.75	7,290.62	29,169.75
TEXAS	1,339.91	1,337.33	1,318.61	1,270.75	5,266.60
UTAH	6,306.36	6,155.78	5,963.49	5,851.33	24,276.96
VERMONT	3,811.62	3,738.15	3,541.26	3,410.83	14,501.86
VIRGIN ISLANDS	532.46	526.35	393.66	461.58	1,914.04
VIRGINIA	4,017.78	3,754.07	3,554.44	3,498.79	14,825.08
WASHINGTON	4,546.43	4,425.28	4,246.97	4,064.50	17,283.19
WEST VIRGINIA	4,800.85	4,577.57	4,382.42	4,205.12	17,965.97
WISCONSIN	4,144.33	3,807.85	3,625.08	3,504.89	15,082.15
WYOMING	4,752.44	4,507.33	4,436.69	4,430.60	18,127.06
U.S. TOTAL	4,104.53	3,973.31	3,805.28	3,735.78	15,618.90

DRUG CODE:9150 DRUG NAME: HYDROMORPHONE

STATE	1ST QUARTER	2ND QUARTER	3RD QUARTER	4TH QUARTER	TOTAL GRAMS
ALABAMA	73.83	66.41	67.39	65.13	272.76
ALASKA	149.99	137.55	127.64	133.56	548.73
AMERICAN SAMOA	1.35	0.14	0	0	1.49
ARIZONA	110.91	107.91	103.18	97.46	419.45
ARKANSAS	115.81	112.73	110.45	108.71	447.7
CALIFORNIA	108.65	107.02	102.34	98.84	416.85
COLORADO	146.56	133.97	129.38	130.9	540.81
CONNECTICUT	186.66	180.43	173.62	174.88	715.59
DELAWARE	255.57	231.59	227.1	234.94	949.2
DISTRICT OF COLUMBIA	99.16	89.71	87.95	83.36	360.19
FLORIDA	266.78	248.15	236.12	242.11	993.16
GEORGIA	90.56	85.89	86.82	80.45	343.72
GUAM	17.79	14.75	13.12	18.51	64.18
HAWAII	50.92	51.92	47.26	46.28	196.37
IDAHO	123.93	115.78	117.34	123.05	480.09
ILLINOIS	80.22	76.66	73.35	73	303.22
INDIANA	93.49	93.85	91.3	88.87	367.51
IOWA	104.29	98.02	94.31	93.75	390.37
KANSAS	125.32	130.07	124.86	109.66	489.92
KENTUCKY	68.45	61.01	65.03	77.29	271.78
LOUISIANA	81.94	71.8	68.29	68.55	290.58
MAINE	121.34	150.91	99.55	131.56	503.36
MARYLAND	154.74	147.04	139.84	137.13	578.75
MASSACHUSETTS	119.02	114.93	104.88	111.33	450.15
MICHIGAN	96.95	89.44	90.43	88.95	365.78
MINNESOTA	111.94	113.78	112.36	110.47	448.56

MISSISSIPPI	169.44	209.66	193.32	216.3	788.72
MISSOURI	116.89	106.28	103.32	104.35	430.83
MONTANA	169.91	163.04	175.18	155.33	663.47
NEBRASKA	75.41	75.34	66.97	67.37	285.09
NEVADA	117.52	111.37	104.89	103.03	436.82
NEW HAMPSHIRE	156.43	151.73	150.21	138.96	597.33
NEW JERSEY	108.91	102.82	95.94	96.08	403.75
NEW MEXICO	71.16	68.85	61.69	60.33	262.03
NEW YORK	104.6	102.01	98.62	97.61	402.83
NORTH CAROLINA	122.23	112.89	111.73	104.5	451.34
NORTH DAKOTA	95.81	100.85	93.74	95.47	385.87
OHIO	83.19	80.62	80.07	80.16	324.04
OKLAHOMA	97.73	100.71	96.93	93.83	389.19
OREGON	131.98	129.2	126.37	117.42	504.97
PENNSYLVANIA	139.79	115.63	109.67	111.21	476.3
PUERTO RICO	2.03	1.75	2.17	2.01	7.96
RHODE ISLAND	84.26	98.31	77.06	76.03	335.65
SOUTH CAROLINA	129.76	126.04	119.13	121.81	496.75
SOUTH DAKOTA	63.83	64.56	62.99	78.8	270.17
TENNESSEE	129.25	97.42	101.63	91.74	420.04
TEXAS	98.41	96.93	94.6	89.48	379.43
UTAH	92.57	94.47	100.68	106.85	394.57
VERMONT	197.98	202.93	191.25	186.56	778.73
VIRGIN ISLANDS	24.22	17.03	12.69	13.57	67.51
VIRGINIA	156.93	142.31	131.47	131.02	561.73
WASHINGTON	166.24	165.25	158.57	146.81	636.87
WEST VIRGINIA	90.98	83.7	78.45	73.6	326.72
WISCONSIN	67.78	61.74	65.05	66.77	261.34
WYOMING	115.71	113.98	107.27	95.83	432.8
U.S. TOTAL	118.89	113.17	109.02	107.77	448.85

DRUG CODE:9193 DRUG NAME: HYDROCODONE

STATE	1ST QUARTER	2ND QUARTER	3RD QUARTER	4TH QUARTER	TOTAL GRAMS
ALABAMA	5,405.01	5,157.45	4,881.85	4,889.37	20,333.67
ALASKA	1,682.16	1,618.17	1,517.15	1,452.32	6,269.80
AMERICAN SAMOA	6.09	0.42	5.53	0	12.04
ARIZONA	1,753.75	1,635.20	1,538.83	1,530.33	6,458.11
ARKANSAS	4,808.29	4,680.16	4,455.99	4,426.35	18,370.79
CALIFORNIA	2,556.31	2,501.71	2,399.79	2,356.88	9,814.69
COLORADO	1,368.60	1,303.83	1,258.98	1,236.78	5,168.19
CONNECTICUT	607.13	572.14	537.47	531.65	2,248.38
DELAWARE	711.6	658.94	648.84	648.98	2,668.35
DISTRICT OF COLUMBIA	261.21	242.74	240.13	214.25	958.32
FLORIDA	2,043.37	1,932.88	1,801.80	1,881.09	7,659.14
GEORGIA	2,673.91	2,582.11	2,452.27	2,449.57	10,157.86
GUAM	82.96	110.13	83.72	76.26	353.07
HAWAII	1,368.32	1,303.39	1,257	1,208.79	5,137.50
IDAHO	3,734.82	3,691.64	3,560.42	3,561.58	14,548.46
ILLINOIS	2,494.39	2,437.70	2,338.97	2,324.68	9,595.74
INDIANA	3,653.80	3,589.91	3,383.47	3,329.26	13,956.44
IOWA	1,949.69	1,922.84	1,844.88	1,822	7,539.41
KANSAS	2,949.73	2,882.55	2,782.47	2,764.77	11,379.52
KENTUCKY	4,803.66	4,691.86	4,479.08	4,451.34	18,425.93
LOUISIANA	3,821.33	3,663.20	3,484.43	3,415.04	14,384
MAINE	1,338.22	1,302.09	1,267.65	1,245.74	5,153.70
MARYLAND	650.71	608.49	572.13	566.99	2,398.32
MASSACHUSETTS	485.77	470.38	443.48	443.35	1,842.98
MICHIGAN	4,231.89	4,156.59	3,961.66	3,789.50	16,139.64
MINNESOTA	911.22	870.48	844.99	815.7	3,442.39

MISSISSIPPI	4,257.62	4,083.86	3,786.65	3,594.84	15,722.97
MISSOURI	3,078.84	3,041.97	2,879.80	2,820.36	11,820.96
MONTANA	2,707.62	2,636.90	2,555.95	2,497.44	10,397.91
NEBRASKA	1,860.92	1,778.61	1,745.17	1,731.79	7,116.49
NEVADA	3,662.67	3,567.83	3,370.89	3,377.43	13,978.82
NEW HAMPSHIRE	721	686.15	664.84	671.97	2,743.95
NEW JERSEY	435.42	393.21	372.6	369.86	1,571.08
NEW MEXICO	1,792.35	1,730.07	1,634.42	1,619.82	6,776.67
NEW YORK	929.79	898.9	851.21	835.15	3,515.05
NORTH CAROLINA	2,223.98	2,050.06	1,896.73	1,897.21	8,067.98
NORTH DAKOTA	1,628.92	1,570.20	1,494.06	1,475.99	6,169.18
OHIO	1,726.05	1,642.47	1,562.80	1,508.25	6,439.57
OKLAHOMA	4,997.10	4,947.43	4,725.35	4,679.17	19,349.05
OREGON	2,590.02	2,486.19	2,403.66	2,352.91	9,832.78
PENNSYLVANIA	1,387.18	1,315.49	1,291.48	1,202.42	5,196.57
PUERTO RICO	29.45	17.74	10.74	10.42	68.35
RHODE ISLAND	1,064.04	997.87	950.9	928.75	3,941.57
SOUTH CAROLINA	3,188.56	3,038.62	2,829.09	2,845.92	11,902.20
SOUTH DAKOTA	1,940.71	1,832.22	1,770.96	1,728.09	7,271.98
TENNESSEE	4,032.91	3,894.17	3,767.47	3,635.30	15,329.84
TEXAS	2,738.84	2,685.15	2,589.01	2,558.69	10,571.68
UTAH	2,221.04	2,145.81	2,058.33	2,036.74	8,461.91
VERMONT	820.6	784.69	729.57	723.53	3,058.39
VIRGIN ISLANDS	331.09	310.47	203.67	209.71	1,054.95
VIRGINIA	1,454.75	1,317.73	1,219.08	1,212.02	5,203.58
WASHINGTON	2,044.24	1,984.69	1,906.75	1,848.78	7,784.45
WEST VIRGINIA	3,386.21	3,256.11	3,095.13	2,977.19	12,714.64
WISCONSIN	1,451.36	1,371.16	1,328.13	1,327.34	5,478
WYOMING	2,161.79	2,074.66	1,971.55	1,955.11	8,163.11
U.S. TOTAL	2,285.19	2,208.13	2,104.46	2,074.70	8,672.48

DRUG CODE:9220L DRUG NAME: LEVORPHANOL

STATE	1ST QUARTER	2ND QUARTER	3RD QUARTER	4TH QUARTER	TOTAL GRAMS
ALABAMA	0.12	0.09	0.11	0.09	0.42
ARIZONA	0.29	0.35	0.46	0.47	1.56
ARKANSAS	0.1	0.12	0.07	0.03	0.31
CALIFORNIA	0.29	0.27	0.27	0.25	1.08
COLORADO	0.12	0.26	0.27	0.36	1.01
CONNECTICUT	0.05	0.1	0.14	0.27	0.57
DELAWARE	0.1	0	0.06	0.12	0.28
FLORIDA	0.14	0.19	0.18	0.21	0.72
GEORGIA	0.01	0.03	0.03	0.04	0.1
IDAHO	0	0	0.01	0.01	0.01
ILLINOIS	0.07	0.1	0.12	0.12	0.41
INDIANA	0.4	0.38	0.38	0.34	1.5
IOWA	0.14	0.13	0.11	0.12	0.51
KANSAS	0.39	0.35	0.25	0.26	1.26
KENTUCKY	0	0	0.01	0	0.01
LOUISIANA	0.36	0.43	0.46	0.43	1.68
MARYLAND	0.05	0.1	0.07	0.14	0.37
MASSACHUSETTS	0.32	0.3	0.31	0.31	1.23
MICHIGAN	0	0	0	0	0.01
MINNESOTA	0.01	0.01	0.02	0.02	0.05
MISSISSIPPI	0.01	0	0	0	0.01
MISSOURI	0.23	0.33	0.44	0.46	1.45
NEBRASKA	0.22	0.28	0.3	0.5	1.3
NEVADA	0.11	0.11	0.18	0.13	0.52
NEW HAMPSHIRE	0.09	0.14	0.12	0.05	0.41
NEW JERSEY	3.57	4.56	4.63	5.67	18.43

NEW YORK	0.17	0.2	0.21	0.25	0.83
NORTH CAROLINA	0.53	0.51	0.68	1.07	2.79
OHIO	0.07	0.13	0.1	0.08	0.38
OREGON	0.09	0.09	0.08	0.1	0.35
PENNSYLVANIA	0.08	0.12	0.12	0.1	0.42
SOUTH CAROLINA	0.02	0.07	0.07	0.08	0.24
TENNESSEE	0	0	0.01	0	0.01
TEXAS	0.16	0.21	0.24	0.26	0.88
VERMONT	0.04	0.07	0.07	0.07	0.26
VIRGINIA	0.07	0.07	0.14	0.2	0.49
WASHINGTON	0.34	0.36	0.46	0.46	1.62
WISCONSIN	0.03	0.07	0.09	0.09	0.27
U.S. TOTAL	0.25	0.3	0.31	0.36	1.22

DRUG CODE:9230 DRUG NAME: MEPERIDINE (PETHIDINE)

STATE	1ST QUARTER	2ND QUARTER	3RD QUARTER	4TH QUARTER	TOTAL GRAMS
ALABAMA	167.58	154.66	143.58	149.59	615.41
ALASKA	119.7	99.15	93.27	89.3	401.42
ARIZONA	85.28	76.99	68.42	74.47	305.16
ARKANSAS	286.33	268.84	242.99	216.94	1,015.10
CALIFORNIA	42.88	42.61	40.66	41.27	167.42
COLORADO	43.17	37.6	34.39	29.57	144.74
CONNECTICUT	30.89	29.44	29	29.93	119.26
DELAWARE	27.66	30.57	24.03	14.7	96.97
DISTRICT OF COLUMBIA	36.17	36.57	28.08	24.59	125.41
FLORIDA	52.93	50.59	41.53	45.44	190.49
GEORGIA	118.69	107.8	100.28	102.54	429.32
GUAM	17.16	19.41	37.12	29.24	102.94
HAWAII	28.1	28.99	22.49	20.2	99.78
IDAHO	54.1	59.02	47.25	52.05	212.43
ILLINOIS	23.31	25.52	24.95	19.83	93.61
INDIANA	55.36	54.37	50.29	47.04	207.06
IOWA	63.27	62.17	65.36	64.61	255.42
KANSAS	65.22	62.84	60.01	59.77	247.84
KENTUCKY	66.38	53	56.91	49.03	225.32
LOUISIANA	178.91	166.99	148.12	146.1	640.13
MAINE	35.03	30.82	27.03	25.92	118.81
MARYLAND	47.64	42.86	36.64	35.15	162.29
MASSACHUSETTS	34.65	34.37	33.13	26.97	129.13
MICHIGAN	30.92	29.4	26.97	23.28	110.57
MINNESOTA	15.76	12.27	12.29	11.22	51.54
MISSISSIPPI	157.4	155.7	134.5	135.91	583.51
MISSOURI	77.28	65.34	58.88	52.48	253.98
MONTANA	66.4	78.29	66.41	60.4	271.51
NEBRASKA	43.92	42.36	36.68	29.54	152.49
NEVADA	60.46	45.86	46.38	57.28	209.99
NEW HAMPSHIRE	51.36	46.41	46.53	34.2	178.5
NEW JERSEY	24.87	24.8	20.84	17.1	87.61
NEW MEXICO	81.62	68.79	63.85	60.14	274.41
NEW YORK	26.44	24.06	22.2	21.68	94.38
NORTH CAROLINA	75.41	67.77	56.96	57.23	257.37
NORTH DAKOTA	35.31	35.32	37.72	33.15	141.51
OHIO	45.61	44.13	46.95	36.51	173.2
OKLAHOMA	204.07	196.86	181.48	181.46	763.87
OREGON	45.67	38.22	41.39	34.34	159.63
PENNSYLVANIA	31.05	27.69	26.97	26.07	111.78
PUERTO RICO	151.73	132.37	110.55	71.62	466.26
RHODE ISLAND	24.03	25.14	24.39	22.09	95.65
SOUTH CAROLINA	146.59	132.3	119.44	110.11	508.44

SOUTH DAKOTA	67.66	68.63	65.07	53.65	255.01
TENNESSEE	137.89	123.67	106.23	100.09	467.88
TEXAS	46.85	43	46.89	47.52	184.25
UTAH	72.75	56.49	64.71	66.39	260.34
VERMONT	51.76	50.35	42.44	61.26	205.81
VIRGIN ISLANDS	38.19	61.6	38.19	93.24	231.22
VIRGINIA	87.23	74.14	68.24	61.34	290.95
WASHINGTON	56.11	50.13	48.55	46.6	201.4
WEST VIRGINIA	39.48	35.75	31.63	27.53	134.4
WISCONSIN	27.41	26.33	25.79	23.39	102.92
WYOMING	67.13	56.47	45.11	62.24	230.94
U.S. TOTAL	61.86	57.26	53.2	51.03	223.35

DRUG CODE:9250B DRUG NAME: METHADONE

STATE	1ST QUARTER	2ND QUARTER	3RD QUARTER	4TH QUARTER	TOTAL GRAMS
ALABAMA	1,595.91	1,688.93	1,567.57	1,683.98	6,536.39
ALASKA	1,099.63	1,187.65	1,182.91	1,047.63	4,517.82
ARIZONA	1,332.76	1,331.16	1,289.71	1,298.76	5,252.39
ARKANSAS	578.83	569.57	533.93	520.82	2,203.14
CALIFORNIA	946.06	955.59	920.18	906.05	3,727.89
COLORADO	837.12	860.85	894.17	848.14	3,440.28
CONNECTICUT	3,336.76	3,522.89	2,942.55	3,527.18	13,329.37
DELAWARE	2,813.13	3,417.48	2,940.22	3,078.77	12,249.60
DISTRICT OF COLUMBIA	1,531.28	1,775.19	1,533.92	1,792.13	6,632.52
FLORIDA	913.65	962.11	1,193.98	1,093.34	4,163.08
GEORGIA	1,145.46	1,254.09	1,201.47	1,205.09	4,806.11
GUAM	32.58	21.55	23.81	18.05	95.99
HAWAII	577.77	660.24	559.54	576.61	2,374.16
IDAHO	769.33	768.84	748.93	726	3,013.10
ILLINOIS	944.79	988.23	930.01	951.47	3,814.50
INDIANA	1,304.24	1,280.87	1,233.76	1,223.46	5,042.34
IOWA	403.75	442.86	415.46	453.78	1,715.86
KANSAS	795.19	860.61	787.39	796.97	3,240.16
KENTUCKY	1,177.79	1,143.06	1,244.34	1,184.83	4,750.03
LOUISIANA	790.42	824.57	821.38	710	3,146.37
MAINE	2,435.10	2,469.39	2,220.23	2,377.62	9,502.34
MARYLAND	3,338.86	3,342.10	3,246.49	3,310.05	13,237.50
MASSACHUSETTS	2,247.65	2,228.13	2,163.68	2,209.15	8,848.62
MICHIGAN	1,356.81	1,364.03	1,354.88	1,327.27	5,402.99
MINNESOTA	839.98	992.69	963.6	1,121.80	3,918.06
MISSISSIPPI	205.1	204.31	246.25	242.26	897.91
MISSOURI	732.47	626.06	698.02	714.92	2,771.47
MONTANA	776.26	756.5	656.68	763.92	2,953.36
NEBRASKA	481.84	435.78	439.18	435.02	1,791.82
NEVADA	1,369.27	1,405.19	1,297.13	1,286.43	5,358.02
NEW HAMPSHIRE	2,348.44	2,372.58	2,322.88	2,324.61	9,368.51
NEW JERSEY	1,281.73	1,378.77	1,233.28	1,333.32	5,227.10
NEW MEXICO	2,089.30	2,071.59	2,149.01	2,256.45	8,566.35
NEW YORK	1,502.90	1,630.81	1,568.12	1,590.78	6,292.62
NORTH CAROLINA	1,357.40	1,411.79	1,390.35	1,361.90	5,521.44
NORTH DAKOTA	236.42	278.23	326.04	404.11	1,244.80
OHIO	677.59	648.18	631.38	620.01	2,577.15
OKLAHOMA	1,017.04	1,151.98	976.18	1,040.11	4,185.30
OREGON	1,663.59	1,692.09	1,653.50	1,614.44	6,623.62
PENNSYLVANIA	1,528.07	1,566.65	1,519.22	1,599.92	6,213.86
PUERTO RICO	600.96	491.5	645.96	516.91	2,255.32
RHODE ISLAND	3,865.02	4,094.51	4,323.70	4,543.16	16,826.38
SOUTH CAROLINA	1,247.13	1,210.21	1,216.96	1,198.36	4,872.66
SOUTH DAKOTA	278.19	263.71	274.1	259.66	1,075.67

TENNESSEE	777.26	772.8	751.65	760.07	3,061.78
TEXAS	554.52	580.2	535.49	564.86	2,235.06
UTAH	972.67	863.59	1,032.46	955.12	3,823.84
VERMONT	3,012.80	2,725.08	3,222.76	2,865.22	11,825.86
VIRGIN ISLANDS	25.65	351.81	21.17	17.92	416.56
VIRGINIA	1,020.69	942.41	982.45	1,017.35	3,962.90
WASHINGTON	1,266.05	1,390.10	1,547.12	1,608.88	5,812.15
WEST VIRGINIA	1,270.12	1,268.68	1,241.40	1,215.51	4,995.72
WISCONSIN	1,155.51	1,211.02	1,117.14	1,125.93	4,609.60
WYOMING	226.68	206.66	209.83	200.74	843.91
U.S. TOTAL	1,156.30	1,189	1,168.51	1,182.62	4,696.43

DRUG CODE:9300 DRUG NAME: MORPHINE

STATE	1ST QUARTER	2ND QUARTER	3RD QUARTER	4TH QUARTER	TOTAL GRAMS
ALABAMA	1,577.19	1,501.17	1,429.84	1,393.10	5,901.31
ALASKA	1,700.16	1,643.84	1,588.01	1,507.11	6,439.12
AMERICAN SAMOA	45.83	41.82	4.58	41.25	133.49
ARIZONA	2,812.09	2,606.69	2,450.29	2,269.82	10,138.90
ARKANSAS	1,685.40	1,565.37	1,524.09	1,428.69	6,203.55
CALIFORNIA	1,257.08	1,207.37	1,170.25	1,131.23	4,765.93
COLORADO	1,609.62	1,540.44	1,487.08	1,436.58	6,073.73
CONNECTICUT	1,034.18	997.5	982.95	943.15	3,957.78
DELAWARE	2,504.24	2,438.20	2,375.92	2,335.75	9,654.11
DISTRICT OF COLUMBIA	617.16	640.41	629.91	534.77	2,422.25
FLORIDA	2,195	2,100.91	2,039.25	2,034.79	8,369.95
GEORGIA	1,205.98	1,159.23	1,146.03	1,118.93	4,630.17
GUAM	712.08	465.01	577.82	485.89	2,240.80
HAWAII	1,720.97	1,653.19	1,557.59	1,475.59	6,407.33
IDAHO	1,899.53	1,821.65	1,734.59	1,722.60	7,178.37
ILLINOIS	841.83	804.48	780.04	764.09	3,190.45
INDIANA	1,421.27	1,347.56	1,280.45	1,253.01	5,302.29
IOWA	1,144.62	1,113.67	1,070.42	1,069.16	4,397.88
KANSAS	1,641.83	1,553.36	1,543.38	1,488.80	6,227.36
KENTUCKY	1,149.93	1,073.76	1,028.41	1,021.40	4,273.50
LOUISIANA	1,241.81	1,199.06	1,159.36	1,115.05	4,715.28
MAINE	1,283.23	1,269.14	1,236.15	1,175.03	4,963.55
MARYLAND	1,421.81	1,360.18	1,256.29	1,212.28	5,250.55
MASSACHUSETTS	1,062.50	1,015.75	964.43	961.79	4,004.48
MICHIGAN	2,032.13	1,949.57	1,881.68	1,761.04	7,624.42
MINNESOTA	934.19	885.09	855.54	822.07	3,496.89
MISSISSIPPI	1,392.98	1,366.41	1,309.08	1,317.28	5,385.75
MISSOURI	1,711.88	1,620.97	1,582.18	1,529.09	6,444.12
MONTANA	1,927.07	1,741.84	1,718.57	1,651.21	7,038.70
NEBRASKA	1,082.53	1,032.18	1,037	992.81	4,144.52
NEVADA	2,319.33	2,251.18	2,168.54	2,055.98	8,795.03
NEW HAMPSHIRE	1,411.57	1,355.27	1,359.84	1,378.76	5,505.44
NEW JERSEY	1,058.83	980.54	924.95	905.63	3,869.95
NEW MEXICO	1,387.88	1,262.32	1,231.39	1,127.83	5,009.42
NEW YORK	844.57	808.1	777.84	772.88	3,203.40
NORTH CAROLINA	1,328.46	1,258.96	1,221.39	1,184.54	4,993.35
NORTH DAKOTA	994.12	954.76	884.87	902.81	3,736.57
OHIO	1,083.16	1,049.26	1,027.18	981.36	4,140.96
OKLAHOMA	2,585.28	2,468.24	2,375.84	2,267.11	9,696.48
OREGON	2,015.99	1,881.40	1,796.96	1,727.05	7,421.39
PENNSYLVANIA	2,075.34	1,809.88	1,742.33	1,711.46	7,339.02
PUERTO RICO	69.47	60.16	50.07	45.75	225.45
RHODE ISLAND	1,146.71	1,110.61	1,142.21	1,030.45	4,429.98
SOUTH CAROLINA	1,485.87	1,401.04	1,381.15	1,384.39	5,652.44
SOUTH DAKOTA	1,118.32	1,074.08	1,065.61	1,016.36	4,274.37

TENNESSEE	2,926.58	2,506.78	2,453.40	2,356.10	10,242.86
TEXAS	853.56	822.99	803.11	785.89	3,265.54
UTAH	1,853.30	1,787.35	1,774.52	1,696.30	7,111.47
VERMONT	1,717.36	1,628.65	1,559.56	1,571.33	6,476.89
VIRGIN ISLANDS	127.13	138.41	108.73	103.66	477.93
VIRGINIA	1,055.60	988.16	951.02	928.08	3,922.86
WASHINGTON	1,712.62	1,641.58	1,589.44	1,505.54	6,449.19
WEST VIRGINIA	1,262.23	1,180.61	1,167.81	1,108.55	4,719.20
WISCONSIN	1,471.62	1,363.62	1,312.57	1,275.49	5,423.30
WYOMING	1,636.07	1,656.24	1,596.82	1,474.57	6,363.70
U.S. TOTAL	1,418.27	1,339.43	1,295.02	1,255.83	5,308.55

DRUG CODE:9333 DRUG NAME: THEBAINE

STATE	1ST QUARTER	2ND QUARTER	3RD QUARTER	4TH QUARTER	TOTAL GRAMS
TEXAS	0	0	0.1	0	0.1
U.S. TOTAL	0	0	0.01	0	0.01

DRUG CODE:9630 DRUG NAME: OPIUM TINCTURE (LAUDANUM)

STATE	1ST QUARTER	2ND QUARTER	3RD QUARTER	4TH QUARTER	TOTAL GRAMS
CALIFORNIA	0.02	0	0	0	0.02
CONNECTICUT	0	0	0.13	0	0.13
DELAWARE	0	0.13	0	0	0.13
FLORIDA	0.05	0.03	0	0	0.08
ILLINOIS	0.05	0.08	0	0	0.13
INDIANA	0.33	0.22	0	0	0.55
KANSAS	0.21	0	0	0	0.21
KENTUCKY	0.05	0	0	0	0.05
MARYLAND	0.08	0.14	0	0	0.22
MICHIGAN	0.26	0.05	0	0	0.31
NEW HAMPSHIRE	0.36	0	0	0	0.36
NEW JERSEY	0	0.01	0	0	0.01
NEW YORK	0.02	0	0	0	0.02
OHIO	0.63	0.08	0	0	0.72
OREGON	0.06	0	0	0	0.06
PENNSYLVANIA	0.08	0.08	0	0	0.17
TEXAS	0.04	0.02	0	0	0.06
VIRGINIA	0.13	0	0	0	0.13
WASHINGTON	0.05	0	0	0	0.05
WEST VIRGINIA	0.19	0.13	0	0	0.32
WISCONSIN	0.06	0.19	0	0	0.25
U.S. TOTAL	0.07	0.03	0	0	0.1

DRUG CODE:9639 DRUG NAME: OPIUM POWDERED

STATE	1ST QUARTER	2ND QUARTER	3RD QUARTER	4TH QUARTER	TOTAL GRAMS
ALABAMA	1.72	1.98	1.81	1.64	7.15
ALASKA	4.16	3.55	2.79	3.95	14.45
ARIZONA	4.34	4.28	4.57	5.28	18.48
ARKANSAS	2.86	3.32	2.99	3.21	12.38
CALIFORNIA	1.11	1.26	1.3	1.22	4.89
COLORADO	4.61	5.18	5.73	5.55	21.08
CONNECTICUT	3.2	3.38	2.29	1.9	10.78
DELAWARE	1.96	1.64	1.8	1.28	6.7
DISTRICT OF COLUMBIA	2.03	2.15	3.05	2.57	9.81

FLORIDA	2.8	2.65	2.41	2.94	10.8
GEORGIA	1.8	2.17	1.91	1.92	7.8
GUAM	0.2	0	0	0	0.2
HAWAII	1.83	2.04	2.51	2.09	8.47
IDAHO	2.92	3.26	3.12	3.17	12.47
ILLINOIS	1.23	1.51	1.34	1.5	5.59
INDIANA	3.44	3.91	3.62	4.17	15.14
IOWA	3.69	3.31	3.18	3.04	13.21
KANSAS	5.8	6.73	6.22	6.49	25.24
KENTUCKY	4.54	4.14	3.37	4.01	16.05
LOUISIANA	2.33	2.49	2.17	2.3	9.29
MAINE	2.95	3.6	4.07	4.36	14.99
MARYLAND	1.09	1.69	1.22	1.8	5.81
MASSACHUSETTS	2.64	3.28	2.71	3.65	12.27
MICHIGAN	3.79	3.74	3.8	4.27	15.6
MINNESOTA	7.57	7.19	6.86	7.34	28.96
MISSISSIPPI	1.12	1.35	1.23	0.85	4.54
MISSOURI	2.84	2.73	2.78	2.92	11.26
MONTANA	5.39	5.35	4.84	5.06	20.63
NEBRASKA	5.03	5.81	4.95	5.78	21.56
NEVADA	1.13	1.04	1.29	1.51	4.97
NEW HAMPSHIRE	2.3	2.84	2.84	3.14	11.13
NEW JERSEY	0.99	1.52	1.34	1.19	5.04
NEW MEXICO	1.84	2.4	1.42	1.38	7.03
NEW YORK	1.08	1.12	1.04	1.24	4.49
NORTH CAROLINA	2.5	2.29	2.31	2.53	9.62
NORTH DAKOTA	4.6	6.26	6.8	6.85	24.51
OHIO	3.22	3.3	3.26	3.35	13.13
OKLAHOMA	2.04	2.23	2.54	1.47	8.28
OREGON	4.4	5.62	5.3	5.52	20.83
PENNSYLVANIA	2.34	2.27	2.64	3	10.24
RHODE ISLAND	3.11	3.25	4.28	2.43	13.07
SOUTH CAROLINA	2.76	3.35	2.76	3.14	12
SOUTH DAKOTA	7.87	5.75	3.32	5.26	22.2
TENNESSEE	2.69	3.03	3.01	3.24	11.98
TEXAS	2	2.01	2.04	2.26	8.31
UTAH	2.45	2.32	2.27	2.4	9.43
VERMONT	6.62	6.39	5.98	7.02	26
VIRGIN ISLANDS	0	0	1.64	0	1.64
VIRGINIA	2.13	2.23	2.49	2.18	9.03
WASHINGTON	4.19	4.78	4.84	4.89	18.7
WEST VIRGINIA	4.57	5.98	4.7	5.3	20.55
WISCONSIN	3.22	3.94	4.2	3.67	15.03
WYOMING	4.53	4.53	3.51	4.85	17.44
U.S. TOTAL	2.47	2.64	2.55	2.71	10.37

DRUG CODE:9652 DRUG NAME: OXYMORPHONE

STATE	1ST QUARTER	2ND QUARTER	3RD QUARTER	4TH QUARTER	TOTAL GRAMS
ALABAMA	160.57	141.9	85.96	61.47	449.9
ALASKA	40.47	40.39	34.71	25.9	141.46
ARIZONA	134.58	117.27	75.79	43.94	371.57
ARKANSAS	78.16	74.96	53.22	29.37	235.71
CALIFORNIA	45.88	42.04	30.7	21.55	140.16
COLORADO	86.32	75.44	56.78	36.41	254.95
CONNECTICUT	96.69	88.45	64.92	43.57	293.64
DELAWARE	239.97	243.64	205.32	171.45	860.38
DISTRICT OF COLUMBIA	19.97	21.08	16.86	21.74	79.65
FLORIDA	149.13	141.04	103.22	68.7	462.08
GEORGIA	88.41	85.16	55.77	37.19	266.53

HAWAII	49.85	51.44	29.03	27.46	157.78
IDAHO	101.24	95.18	74.95	56.85	328.22
ILLINOIS	41.34	38.85	28.36	18.47	127.02
INDIANA	102.95	89.08	65	43.23	300.26
IOWA	45.16	41.04	29.91	18.71	134.82
KANSAS	86.16	80.87	57.16	39.06	263.26
KENTUCKY	104.95	101.85	72.21	54.29	333.3
LOUISIANA	181	169.06	126.98	103.88	580.92
MAINE	89.59	75.72	64.58	48.54	278.43
MARYLAND	195.49	183.63	141.99	120.62	641.72
MASSACHUSETTS	43.73	41.7	30.07	17.4	132.89
MICHIGAN	206.38	211.1	183.43	164.96	765.87
MINNESOTA	9.45	9.79	7.62	5.15	32.02
MISSISSIPPI	130.99	120.96	92.3	68.52	412.78
MISSOURI	97.74	89.01	62.14	38.77	287.67
MONTANA	39.22	29.97	29.31	16	114.49
NEBRASKA	85.81	82.78	57.93	34.51	261.03
NEVADA	201.22	179.2	130.15	79.32	589.89
NEW HAMPSHIRE	83.23	75.42	62.76	43.81	265.22
NEW JERSEY	95.45	88.83	61.68	46.28	292.25
NEW MEXICO	60.8	52.43	39.53	31.69	184.44
NEW YORK	113.03	107.47	84.49	65.6	370.59
NORTH CAROLINA	292.52	265.72	183.48	128.25	869.98
NORTH DAKOTA	25.9	26.87	23.74	17.1	93.61
OHIO	105.93	99.35	76.23	52.65	334.15
OKLAHOMA	186.17	175.6	130.29	88.02	580.08
OREGON	53.06	48.99	41.14	28.59	171.77
PENNSYLVANIA	211.98	206.51	165.38	133.06	716.93
RHODE ISLAND	54.8	46.23	34.62	16.55	152.2
SOUTH CAROLINA	167.92	162.22	110.41	78.38	518.94
SOUTH DAKOTA	77.59	65.53	51.48	40.26	234.86
TENNESSEE	396.93	367.89	290.78	240.22	1,295.82
TEXAS	61.57	57.39	39.35	25.29	183.6
UTAH	174.26	163.29	138.84	116.95	593.35
VERMONT	57.96	52.01	44.01	28.98	182.96
VIRGINIA	142.31	129.67	98.73	83.13	453.85
WASHINGTON	54.04	48.58	38.58	28.41	169.61
WEST VIRGINIA	158.09	158.09	130.07	89.29	535.55
WISCONSIN	51.43	44.66	32.42	21.16	149.66
WYOMING	57.22	60.83	45.31	35.37	198.72
U.S. TOTAL	112.43	105.27	78.82	58.4	354.92

DRUG CODE:9668 DRUG NAME: NOROXYMORPHONE

STATE	1ST QUARTER	2ND QUARTER	3RD QUARTER	4TH QUARTER	TOTAL GRAMS
ARIZONA	0	0	0	0	0
ARKANSAS	0.5	0.59	0.92	0.66	2.68
CALIFORNIA	0.14	0.18	0.19	0.18	0.7
FLORIDA	0.07	0.07	0.07	0.1	0.31
GEORGIA	0.03	0.03	0.1	0.05	0.21
ILLINOIS	0.03	0.08	0.12	0.07	0.3
KANSAS	0.1	0	0	0	0.1
MARYLAND	0	0	0	0	0
MASSACHUSETTS	0.02	0.03	0	0	0.05
MICHIGAN	0	0	0.03	0.01	0.04
MINNESOTA	0	0.01	0	0	0.01
MISSISSIPPI	0.08	0.05	0.07	0	0.21
MISSOURI	0.13	0.22	0.18	0.1	0.62
NEVADA	0.25	0.11	0.28	0.31	0.94
NEW JERSEY	0.08	0.14	0.02	0.03	0.27

NEW YORK	0.01	0.03	0.05	0.02	0.11
NORTH CAROLINA	0	0	0.02	0.02	0.04
OHIO	0.05	0.08	0.05	0.02	0.21
OKLAHOMA	0	0	0	0.01	0.02
OREGON	0	0	0.05	0	0.05
PENNSYLVANIA	0	0	0	0	0.01
PUERTO RICO	0.05	0.12	0.32	0.18	0.67
RHODE ISLAND	0	0	0.01	0	0.01
TENNESSEE	0	0	0	0	0
TEXAS	0.09	0.12	0.12	0.1	0.43
VERMONT	0	0	0.02	0	0.02
WASHINGTON	0.01	0.01	0.05	0.06	0.13
U.S. TOTAL	0.05	0.06	0.07	0.06	0.24

DRUG CODE:9737 DRUG NAME: ALFENTANIL

STATE	1ST QUARTER	2ND QUARTER	3RD QUARTER	4TH QUARTER	TOTAL GRAMS
ALABAMA	0	0	0	0	0.01
ALASKA	0.11	0.11	0.07	0.13	0.41
ARIZONA	0.02	0.02	0.01	0.02	0.06
ARKANSAS	0	0	0	0	0
CALIFORNIA	0.05	0.04	0.04	0.05	0.18
COLORADO	0.03	0.01	0.02	0.02	0.08
CONNECTICUT	0.04	0.03	0.03	0.04	0.15
FLORIDA	0.02	0.03	0.03	0.03	0.11
GEORGIA	0.03	0.03	0.05	0.03	0.13
GUAM	0	0	0.03	0.02	0.04
HAWAII	0.03	0.04	0.05	0.06	0.18
IDAHO	0.09	0.09	0.07	0.09	0.35
ILLINOIS	0.03	0.03	0.03	0.04	0.13
INDIANA	0.03	0.05	0.04	0.04	0.16
IOWA	0.07	0.05	0.05	0.06	0.22
KANSAS	0.06	0.05	0.04	0.06	0.21
KENTUCKY	0.12	0.09	0.08	0.09	0.38
LOUISIANA	0.05	0.05	0.06	0.06	0.22
MAINE	0.05	0.06	0.05	0.05	0.2
MARYLAND	0.02	0	0.01	0.02	0.05
MASSACHUSETTS	0.02	0.01	0.01	0.01	0.05
MICHIGAN	0.01	0.01	0.01	0.01	0.05
MINNESOTA	0.06	0.06	0.06	0.08	0.26
MISSISSIPPI	0	0	0.01	0	0.01
MISSOURI	0.02	0.02	0.02	0.02	0.07
MONTANA	0.04	0.03	0.03	0.03	0.13
NEBRASKA	0.05	0.04	0.06	0.05	0.19
NEVADA	0.05	0.06	0.06	0.09	0.26
NEW HAMPSHIRE	0.04	0.03	0.03	0.05	0.15
NEW JERSEY	0	0	0	0	0.01
NEW MEXICO	0.04	0.04	0.06	0.06	0.2
NEW YORK	0.01	0.01	0.01	0.02	0.05
NORTH CAROLINA	0.03	0.04	0.03	0.04	0.13
NORTH DAKOTA	0.02	0.04	0.01	0.02	0.09
OHIO	0.04	0.05	0.03	0.04	0.17
OKLAHOMA	0.02	0.03	0.02	0.02	0.09
OREGON	0.1	0.11	0.15	0.13	0.49
PENNSYLVANIA	0.01	0	0	0	0.02
RHODE ISLAND	0	0.01	0.01	0	0.02
SOUTH CAROLINA	0	0	0	0	0.01
SOUTH DAKOTA	0.01	0.01	0.01	0.01	0.04
TENNESSEE	0.02	0.02	0.02	0.02	0.08
TEXAS	0.02	0.02	0.02	0.02	0.09

UTAH	0.02	0.01	0.02	0.06	0.11
VERMONT	0	0	0	0	0.01
VIRGINIA	0.01	0.01	0.01	0.01	0.05
WASHINGTON	0.04	0.05	0.05	0.05	0.19
WISCONSIN	0.07	0.08	0.08	0.1	0.34
WYOMING	0	0	0	0.02	0.02
U.S. TOTAL	0.03	0.03	0.03	0.03	0.12

DRUG CODE:9739 DRUG NAME: REMIFENTANIL

STATE	1ST QUARTER	2ND QUARTER	3RD QUARTER	4TH QUARTER	TOTAL GRAMS
ALABAMA	0.07	0.07	0.07	0.07	0.28
ALASKA	0.38	0.42	0.39	0.44	1.64
ARIZONA	0.07	0.06	0.06	0.07	0.25
ARKANSAS	0.05	0.05	0.06	0.05	0.22
CALIFORNIA	0.09	0.09	0.08	0.09	0.36
COLORADO	0.12	0.13	0.12	0.14	0.51
CONNECTICUT	0.27	0.22	0.22	0.24	0.95
DELAWARE	0.3	0.45	0.31	0.28	1.34
DISTRICT OF COLUMBIA	0.15	0.14	0.09	0.1	0.47
FLORIDA	0.14	0.14	0.13	0.16	0.57
GEORGIA	0.05	0.05	0.05	0.05	0.2
GUAM	0.01	0.02	0	0.14	0.17
HAWAII	0.09	0.08	0.1	0.1	0.38
IDAHO	0.14	0.14	0.14	0.14	0.55
ILLINOIS	0.12	0.11	0.11	0.13	0.47
INDIANA	0.12	0.12	0.12	0.13	0.49
IOWA	0.12	0.11	0.11	0.08	0.41
KANSAS	0.07	0.09	0.07	0.08	0.32
KENTUCKY	0.12	0.1	0.12	0.11	0.44
LOUISIANA	0.1	0.1	0.1	0.11	0.41
MAINE	0.1	0.09	0.08	0.07	0.35
MARYLAND	0.12	0.12	0.1	0.12	0.47
MASSACHUSETTS	0.32	0.37	0.33	0.35	1.36
MICHIGAN	0.1	0.11	0.1	0.1	0.4
MINNESOTA	0.13	0.15	0.15	0.19	0.62
MISSISSIPPI	0.02	0.02	0.01	0.01	0.06
MISSOURI	0.08	0.09	0.1	0.09	0.36
MONTANA	0.15	0.18	0.17	0.2	0.71
NEBRASKA	0.06	0.05	0.05	0.07	0.23
NEVADA	0.03	0.03	0.04	0.04	0.14
NEW HAMPSHIRE	0.09	0.12	0.11	0.18	0.5
NEW JERSEY	0.15	0.15	0.15	0.16	0.6
NEW MEXICO	0.05	0.06	0.06	0.05	0.22
NEW YORK	0.19	0.19	0.21	0.21	0.8
NORTH CAROLINA	0.15	0.16	0.15	0.16	0.61
NORTH DAKOTA	0.09	0.05	0.06	0.06	0.25
OHIO	0.17	0.17	0.14	0.18	0.66
OKLAHOMA	0.07	0.08	0.07	0.07	0.29
OREGON	0.07	0.09	0.07	0.08	0.3
PENNSYLVANIA	0.28	0.28	0.28	0.29	1.13
RHODE ISLAND	0.06	0.05	0.07	0.12	0.3
SOUTH CAROLINA	0.11	0.09	0.1	0.1	0.4
SOUTH DAKOTA	0.06	0.08	0.05	0.04	0.23
TENNESSEE	0.07	0.09	0.11	0.1	0.37
TEXAS	0.11	0.11	0.11	0.12	0.44
UTAH	0.64	0.51	0.56	0.89	2.6
VERMONT	0.11	0.15	0.08	0.12	0.45
VIRGINIA	0.08	0.09	0.08	0.09	0.34
WASHINGTON	0.19	0.18	0.22	0.16	0.74

WEST VIRGINIA	0.29	0.32	0.27	0.24	1.12
WISCONSIN	0.11	0.13	0.09	0.14	0.47
WYOMING	0.09	0.07	0.08	0.08	0.32
U.S. TOTAL	0.13	0.13	0.13	0.14	0.53

DRUG CODE:9740 DRUG NAME: SUFENTANIL BASE

STATE	1ST QUARTER	2ND QUARTER	3RD QUARTER	4TH QUARTER	TOTAL GRAMS
ALABAMA	0.02	0.04	0.04	0.04	0.14
ALASKA	0	0	0	0	0.01
ARIZONA	0	0	0	0	0.01
ARKANSAS	0	0	0.01	0	0.02
CALIFORNIA	0.03	0.03	0.03	0.03	0.12
COLORADO	0	0	0	0.01	0.02
CONNECTICUT	0.01	0.01	0.02	0.03	0.07
DELAWARE	0	0	0	0	0
DISTRICT OF COLUMBIA	0	0	0	0	0
FLORIDA	0.02	0.01	0.02	0.02	0.07
GEORGIA	0	0	0.01	0.01	0.02
HAWAII	0	0	0	0	0
IDAHO	0	0	0	0	0.01
ILLINOIS	0	0	0	0	0.02
INDIANA	0	0.01	0	0	0.02
IOWA	0	0	0	0	0.01
KANSAS	0.01	0.01	0.01	0.01	0.04
KENTUCKY	0.01	0.01	0.01	0.01	0.03
LOUISIANA	0.01	0.04	0.02	0.03	0.1
MAINE	0	0	0	0	0.01
MARYLAND	0	0	0	0	0
MASSACHUSETTS	0	0	0	0	0.01
MICHIGAN	0.01	0	0	0.01	0.02
MINNESOTA	0	0	0	0.01	0.01
MISSISSIPPI	0.5	0.35	0.42	0.49	1.76
MISSOURI	0	0.01	0.01	0	0.02
MONTANA	0	0	0	0.01	0.02
NEBRASKA	0	0	0	0.01	0.02
NEVADA	0	0	0	0	0.01
NEW HAMPSHIRE	0	0	0	0	0
NEW JERSEY	0.01	0.01	0	0.02	0.04
NEW MEXICO	0	0	0	0	0.01
NEW YORK	0.01	0.01	0.01	0.01	0.03
NORTH CAROLINA	0	0	0.01	0	0.02
NORTH DAKOTA	0	0.01	0	0	0.02
OHIO	0	0	0	0.01	0.02
OKLAHOMA	0	0	0	0	0.02
OREGON	0.01	0.01	0.01	0.01	0.03
PENNSYLVANIA	0	0.02	0.03	0.01	0.06
PUERTO RICO	0.01	0	0.01	0	0.02
RHODE ISLAND	0	0	0	0	0
SOUTH CAROLINA	0.02	0.02	0.02	0	0.06
SOUTH DAKOTA	0	0	0	0.01	0.02
TENNESSEE	0.01	0.01	0.01	0.01	0.04
TEXAS	0.01	0.01	0	0.01	0.03
UTAH	0.01	0.01	0.01	0.01	0.03
VERMONT	0	0	0	0	0
VIRGINIA	0.01	0	0	0.01	0.02
WASHINGTON	0	0	0	0	0.02
WEST VIRGINIA	0	0	0.01	0	0.01
WISCONSIN	0.01	0.01	0	0.02	0.04
WYOMING	0	0	0	0.01	0.01

U.S. TOTAL	0.01	0.01	0.01	0.02	0.05

DRUG CODE:9780 DRUG NAME: TAPENTADOL

STATE	1ST QUARTER	2ND QUARTER	3RD QUARTER	4TH QUARTER	TOTAL GRAMS
ALABAMA	1,235.02	1,177.77	1,097.85	1,062.01	4,572.66
ALASKA	295.04	305.18	326.51	339.19	1,265.93
ARIZONA	597.39	559.23	504.35	562.48	2,223.45
ARKANSAS	798.82	767.87	653.58	658.21	2,878.49
CALIFORNIA	296.77	296.43	272.84	274.45	1,140.49
COLORADO	546.15	574.43	546.27	565.17	2,232.02
CONNECTICUT	354.15	332.83	306.04	295.4	1,288.41
DELAWARE	850.73	827.62	824.56	801.78	3,304.70
DISTRICT OF COLUMBIA	201.01	184.64	152.23	142.92	680.79
FLORIDA	582	547.95	490.66	486.98	2,107.60
GEORGIA	734.08	731.84	656.86	660.46	2,783.24
HAWAII	210.62	241.42	229.99	243.81	925.82
IDAHO	409.93	360.91	364.19	404.06	1,539.09
ILLINOIS	323.05	301.93	281.89	275.44	1,182.32
INDIANA	592.59	584.53	563.67	563.9	2,304.69
IOWA	458.68	424.15	351.53	397.13	1,631.49
KANSAS	333.97	308.07	290.51	295.66	1,228.20
KENTUCKY	349.39	349.21	353.28	333.9	1,385.78
LOUISIANA	337.57	331.41	321.99	349.98	1,340.96
MAINE	109.04	111.98	101.03	102.19	424.24
MARYLAND	594.17	588.55	539.93	529.08	2,251.72
MASSACHUSETTS	293.82	299.26	294.69	289.38	1,177.16
MICHIGAN	221.16	215.01	218.87	221.55	876.59
MINNESOTA	81.38	79.7	83.05	87.73	331.86
MISSISSIPPI	442.88	422.08	398.83	363.46	1,627.26
MISSOURI	431.65	421.32	385.48	390.66	1,629.11
MONTANA	222.51	168.64	177.73	182.53	751.4
NEBRASKA	571.96	511.51	505.46	536.62	2,125.56
NEVADA	466.33	450.45	431.76	458.7	1,807.24
NEW HAMPSHIRE	575.29	587.59	536.62	534.92	2,234.42
NEW JERSEY	554.71	546.83	526.04	524.7	2,152.28
NEW MEXICO	171.72	151.98	149.38	149.82	622.89
NEW YORK	515.98	504.16	485.5	475.38	1,981.02
NORTH CAROLINA	1,058.65	1,047.83	980.3	966.79	4,053.57
NORTH DAKOTA	378.83	373.33	341.74	359.51	1,453.41
OHIO	573.72	543.28	498.98	488.29	2,104.27
OKLAHOMA	661.38	658.92	608.85	601.08	2,530.22
OREGON	191.42	194.23	196.86	195.73	778.24
PENNSYLVANIA	531.25	540.63	502.21	507.96	2,082.06
PUERTO RICO	24.28	26.57	22.25	17.46	90.56
RHODE ISLAND	196.42	205.83	195.71	199.13	797.1
SOUTH CAROLINA	957.16	963.3	917.81	923.82	3,762.08
SOUTH DAKOTA	640.09	594.95	530.72	550.68	2,316.44
TENNESSEE	518.16	485.16	489.88	511.42	2,004.62
TEXAS	405.95	402.31	362.52	380.6	1,551.38
UTAH	564.31	491.48	487	443.2	1,985.99
VERMONT	214.39	175.15	165.96	182.82	738.32
VIRGIN ISLANDS	8.19	5.46	2.73	0	16.39
VIRGINIA	574.86	541.41	482.49	470.56	2,069.33
WASHINGTON	272.45	274.01	251.98	247.73	1,046.18
WEST VIRGINIA	339.07	355.8	326.9	302.81	1,324.59
WISCONSIN	272.53	271.02	256.11	246.15	1,045.81
WYOMING	345.62	367.89	318.83	345.53	1,377.86
U.S. TOTAL	470.8	459.3	427.91	429.13	1,787.14

DRUG CODE:9801 DRUG NAME: FENTANYL BASE

STATE	1ST QUARTER	2ND QUARTER	3RD QUARTER	4TH QUARTER	TOTAL GRAMS
ALABAMA	33.9	32.29	29.78	28.78	124.76
ALASKA	33.78	33.71	32.81	32.14	132.44
AMERICAN SAMOA	0.36	0.35	0	0	0.71
ARIZONA	30.94	28.21	25.44	24.81	109.41
ARKANSAS	34.65	33.48	30.42	28.86	127.41
CALIFORNIA	19.85	19.17	18.24	17.4	74.66
COLORADO	27.09	25.29	24.19	23.06	99.63
CONNECTICUT	28.15	27.68	25.86	24.65	106.34
DELAWARE	39.9	39	36.17	36.46	151.53
DISTRICT OF COLUMBIA	15.36	14.98	13.35	12.6	56.3
FLORIDA	33.89	31.29	29.11	29.92	124.21
GEORGIA	30.28	28.82	27.04	26.46	112.6
GUAM	6.84	5.61	5.14	5.57	23.16
HAWAII	14.57	14.25	12.88	12.88	54.57
IDAHO	37.93	37.59	36.08	35.44	147.04
ILLINOIS	24.35	23.31	22.45	21.93	92.04
INDIANA	31.48	29.64	27.65	27.27	116.04
IOWA	29.81	29.11	27.77	27.43	114.12
KANSAS	39.96	39.31	37.8	39.32	156.39
KENTUCKY	28.41	27.08	25.33	24.43	105.25
LOUISIANA	28.71	26.83	25.45	23.91	104.9
MAINE	29.23	24.9	22.88	22.58	99.58
MARYLAND	26.9	24.54	23.3	21.44	96.17
MASSACHUSETTS	21.23	20.42	19.34	18.87	79.86
MICHIGAN	33.34	32.23	30.52	29.12	125.22
MINNESOTA	20.37	20.16	18.87	18.2	77.6
MISSISSIPPI	42.23	44.21	40.92	38.54	165.9
MISSOURI	37.72	36.73	35.11	42.14	151.7
MONTANA	35.78	34.02	31.93	31.49	133.22
NEBRASKA	30.76	29.58	28.49	27.75	116.57
NEVADA	24.3	22.21	20.72	19.94	87.16
NEW HAMPSHIRE	28.26	26.68	25.86	24.87	105.67
NEW JERSEY	28.39	27.29	24.7	24.02	104.4
NEW MEXICO	23.61	21.66	19.91	19.07	84.26
NEW YORK	24.05	23.06	21.22	20.62	88.95
NORTH CAROLINA	32.49	30.59	27.74	27.33	118.15
NORTH DAKOTA	36.9	35.74	34.31	33.98	140.93
OHIO	22.99	22.06	20.69	19.65	85.38
OKLAHOMA	49.25	46.12	43.55	41.15	180.07
OREGON	26.27	24.92	23.15	22.39	96.73
PENNSYLVANIA	42.31	41.02	38.19	36.45	157.97
PUERTO RICO	5.28	4.05	3.4	2.79	15.52
RHODE ISLAND	18.79	17.75	16.89	16.4	69.83
SOUTH CAROLINA	30.79	29.11	27.22	26.64	113.75
SOUTH DAKOTA	39.96	37.08	36.33	34.54	147.91
TENNESSEE	33	30.97	29.08	28.12	121.17
TEXAS	24.37	23.52	22.04	21.64	91.57
UTAH	31.22	29.7	29.16	28.43	118.51
VERMONT	32.4	31.44	28.79	26.92	119.55
VIRGIN ISLANDS	3.65	3.15	2.75	3.16	12.71
VIRGINIA	24.01	22.27	20.54	19.59	86.41
WASHINGTON	24.01	23.15	21.7	20.85	89.71
WEST VIRGINIA	31.17	30.17	28.65	26.98	116.97
WISCONSIN	28.79	26.8	24.46	23.66	103.71
WYOMING	29.85	26.53	28.04	25.76	110.18
U.S. TOTAL	27.91	26.61	24.92	24.33	103.77

DRUG CODE:9809 DRUG NAME: OPIUM COMBINATION PRODUCT (C-III)

STATE	1ST QUARTER	2ND QUARTER	3RD QUARTER	4TH QUARTER	TOTAL GRAMS
ALABAMA	0.25	0.27	0.25	0.23	0.99
ALASKA	0	0	0.03	0	0.03
ARIZONA	0.19	0.09	0.11	0.13	0.52
ARKANSAS	0.05	0.08	0.1	0.07	0.29
CALIFORNIA	0.05	0.05	0.05	0.06	0.21
COLORADO	0.06	0.06	0.05	0.05	0.22
CONNECTICUT	0.14	0.11	0.18	0.12	0.55
DELAWARE	0.02	0.08	0.02	0.11	0.23
DISTRICT OF COLUMBIA	0.03	0.06	0.06	0.09	0.25
FLORIDA	0.19	0.2	0.17	0.17	0.73
GEORGIA	0.06	0.09	0.08	0.07	0.3
HAWAII	0.14	0.11	0.1	0	0.35
IDAHO	0.25	0.24	0.23	0.24	0.97
ILLINOIS	0.05	0.05	0.05	0.04	0.19
INDIANA	0.11	0.11	0.13	0.11	0.45
IOWA	0.11	0.06	0.06	0.07	0.29
KANSAS	0.03	0.02	0.01	0.05	0.1
KENTUCKY	0.24	0.25	0.18	0.18	0.85
LOUISIANA	0.16	0.19	0.2	0.18	0.73
MAINE	0.06	0.09	0.16	0.21	0.51
MARYLAND	0.15	0.15	0.15	0.14	0.59
MASSACHUSETTS	0.03	0.04	0.02	0.01	0.1
MICHIGAN	0.05	0.05	0.09	0.05	0.23
MINNESOTA	0.03	0.04	0.04	0.06	0.17
MISSISSIPPI	0.15	0.08	0.1	0.06	0.4
MISSOURI	0.17	0.09	0.07	0.15	0.48
MONTANA	0.1	0.02	0.13	0.1	0.34
NEBRASKA	0.06	0.03	0.04	0.03	0.17
NEVADA	0.03	0.05	0.06	0.01	0.15
NEW HAMPSHIRE	0.13	0.14	0.14	0.26	0.68
NEW JERSEY	0.06	0.06	0.06	0.05	0.24
NEW MEXICO	0.09	0.09	0.05	0.09	0.32
NEW YORK	0.04	0.05	0.04	0.06	0.2
NORTH CAROLINA	0.13	0.15	0.16	0.13	0.57
NORTH DAKOTA	0	0.06	0.11	0.23	0.39
OHIO	0.13	0.12	0.1	0.16	0.5
OKLAHOMA	0.09	0.06	0.09	0.12	0.34
OREGON	0	0.01	0.02	0.05	0.09
PENNSYLVANIA	0.25	0.31	0.21	0.3	1.07
RHODE ISLAND	0	0.02	0.02	0	0.04
SOUTH CAROLINA	0.18	0.23	0.23	0.22	0.87
SOUTH DAKOTA	0.16	0.07	0.07	0.02	0.33
TENNESSEE	0.09	0.09	0.09	0.13	0.39
TEXAS	0.05	0.06	0.05	0.06	0.21
UTAH	0.47	0.46	0.4	0.47	1.79
VERMONT	0	0.06	0.03	0.06	0.15
VIRGINIA	0.1	0.07	0.07	0.06	0.3
WASHINGTON	0.03	0.03	0.06	0.03	0.15
WEST VIRGINIA	0.14	0.1	0.13	0.14	0.52
WISCONSIN	0.1	0.09	0.1	0.12	0.41
WYOMING	0	0.1	0.13	0.3	0.54
U.S. TOTAL	0.1	0.1	0.09	0.1	0.39

CUMULATIVE DISTRIBUTION BY STATE IN GRAMS PER 100,000 POPULATION

DRUG CODE:1100 **DRUG NAME:AMPHETAMINE**

RANK	STATE	2010 POP	TOTAL GRAMS	GRAMS/100K POP
1	RHODE ISLAND	1,052,567	146,539.12	13,922.07
2	LOUISIANA	4,533,372	617,608.44	13,623.60
3	UTAH	2,763,885	327,686.38	11,856.01
4	SOUTH CAROLINA	4,625,364	545,192.92	11,787.03
5	NEW HAMPSHIRE	1,316,470	153,869.12	11,688.01
6	ALABAMA	4,779,736	522,022.67	10,921.58
7	MASSACHUSETTS	6,547,629	711,859.01	10,872.01
8	MICHIGAN	9,883,640	961,941.32	9,732.66
9	DELAWARE	897,934	86,201.90	9,600.03
10	WISCONSIN	5,686,986	510,833.02	8,982.49
11	MISSOURI	5,988,927	536,597.02	8,959.82
12	VIRGINIA	8,001,024	714,083.09	8,924.90
13	NORTH CAROLINA	9,535,483	812,531.47	8,521.14
14	KANSAS	2,853,118	241,758.68	8,473.49
15	MISSISSIPPI	2,967,297	246,037.64	8,291.64
16	TENNESSEE	6,346,105	520,438.74	8,200.92
17	MINNESOTA	5,303,925	431,651.49	8,138.34
18	INDIANA	6,483,802	519,990.62	8,019.84
19	DISTRICT OF COLUMBIA	601,723	46,666.86	7,755.54
20	GEORGIA	9,687,653	750,263.70	7,744.54
21	SOUTH DAKOTA	814,180	60,812.21	7,469.14
22	OHIO	11,536,504	847,019.84	7,342.08
23	ARKANSAS	2,915,918	204,371.71	7,008.83
24	MAINE	1,328,361	90,672.30	6,825.88
25	PENNSYLVANIA	12,702,379	844,334.98	6,647.06
26	TEXAS	25,145,561	1,655,859.69	6,585.10
27	IOWA	3,046,355	199,770.10	6,557.68
28	IDAHO	1,567,582	101,764.56	6,491.82
29	WASHINGTON	6,724,540	435,401.09	6,474.81
30	FLORIDA	18,801,310	1,201,205.30	6,388.94
31	COLORADO	5,029,196	319,454.18	6,351.99
32	MARYLAND	5,773,552	366,263.40	6,343.81
33	VERMONT	625,741	39,689.40	6,342.78
34	KENTUCKY	4,339,367	271,947.67	6,266.99
35	OREGON	3,831,074	234,564.17	6,122.67
36	ILLINOIS	12,830,632	763,967.16	5,954.24
37	CONNECTICUT	3,574,097	211,272.54	5,911.21
38	MONTANA	989,415	55,996.63	5,659.57
39	NEBRASKA	1,826,341	103,316.29	5,657.01
40	OKLAHOMA	3,751,351	211,604.73	5,640.76
41	NORTH DAKOTA	672,591	35,964.31	5,347.13
42	ARIZONA	6,392,017	327,569.95	5,124.67
43	WEST VIRGINIA	1,852,994	93,972.75	5,071.40
44	NEW YORK	19,378,102	941,733.87	4,859.78
45	NEW JERSEY	8,791,894	416,678.70	4,739.35
46	WYOMING	563,626	25,735.95	4,566.14
47	ALASKA	710,231	32,335.27	4,552.78
48	NEVADA	2,700,551	104,147.55	3,856.53
49	CALIFORNIA	37,253,956	1,198,133.55	3,216.12
50	NEW MEXICO	2,059,179	63,660.33	3,091.54
51	HAWAII	1,360,301	38,920.07	2,861.14
52	VIRGIN ISLANDS	109,825	1,217.23	1,108.34
53	GUAM	178,430	1,904.11	1,067.15
54	PUERTO RICO	3,725,789	33,115.23	888.81
55	AMERICAN SAMOA	65,628	2.5	3.81
**U.S. GRAMS / PER 100K:**		312,825,210	20,938,152.53	6,693.24

DRUG CODE:1105B **DRUG NAME:DL-METHAMPHETAMINE RACEMIC BASE**

CUMULATIVE DISTRIBUTION BY STATE IN GRAMS PER 100,000 POPULATION

RANK	STATE	2010 POP	TOTAL GRAMS	GRAMS/100K POP
1	SOUTH DAKOTA	814,180	69.52	8.54
2	WASHINGTON	6,724,540	381.36	5.67
3	MINNESOTA	5,303,925	289.33	5.46
4	COLORADO	5,029,196	210.57	4.19
5	PENNSYLVANIA	12,702,379	489.05	3.85
6	WYOMING	563,626	21.3	3.78
7	UTAH	2,763,885	104.08	3.77
8	NEVADA	2,700,551	97.65	3.62
9	NORTH DAKOTA	672,591	23.71	3.53
10	WISCONSIN	5,686,986	197.31	3.47
11	OREGON	3,831,074	127.79	3.34
12	NEW MEXICO	2,059,179	65.9	3.2
13	MISSOURI	5,988,927	190.08	3.17
14	CALIFORNIA	37,253,956	1,152.91	3.09
15	MARYLAND	5,773,552	164.36	2.85
16	OHIO	11,536,504	300.18	2.6
17	MASSACHUSETTS	6,547,629	168.38	2.57
18	MAINE	1,328,361	33.35	2.51
19	CONNECTICUT	3,574,097	89.61	2.51
20	IOWA	3,046,355	73.94	2.43
21	IDAHO	1,567,582	34.96	2.23
22	VIRGINIA	8,001,024	175.21	2.19
23	ARIZONA	6,392,017	134.22	2.1
24	OKLAHOMA	3,751,351	77.16	2.06
25	ALABAMA	4,779,736	96.04	2.01
26	NEW HAMPSHIRE	1,316,470	26.12	1.98
27	NORTH CAROLINA	9,535,483	189.27	1.98
28	ILLINOIS	12,830,632	248.34	1.94
29	NEBRASKA	1,826,341	32.95	1.8
30	DELAWARE	897,934	15.27	1.7
31	TEXAS	25,145,561	426.76	1.7
32	NEW YORK	19,378,102	325.5	1.68
33	RHODE ISLAND	1,052,567	16.88	1.6
34	INDIANA	6,483,802	103.68	1.6
35	NEW JERSEY	8,791,894	138.24	1.57
36	ALASKA	710,231	10.85	1.53
37	HAWAII	1,360,301	20.49	1.51
38	KANSAS	2,853,118	41.79	1.46
39	SOUTH CAROLINA	4,625,364	60.28	1.3
40	LOUISIANA	4,533,372	55.05	1.21
41	VERMONT	625,741	7.23	1.16
42	DISTRICT OF COLUMBIA	601,723	6.83	1.14
43	MISSISSIPPI	2,967,297	32.95	1.11
44	GEORGIA	9,687,653	94.84	0.98
45	MICHIGAN	9,883,640	91.62	0.93
46	KENTUCKY	4,339,367	39.78	0.92
47	WEST VIRGINIA	1,852,994	16.48	0.89
48	ARKANSAS	2,915,918	25.32	0.87
49	TENNESSEE	6,346,105	50.63	0.8
50	FLORIDA	18,801,310	135.83	0.72
51	MONTANA	989,415	2.81	0.28
**U.S. GRAMS / PER 100K:**		312,825,210	6,983.75	2.23

DRUG CODE:1105D **DRUG NAME:D-METHAMPHETAMINE**

RANK	STATE	2010 POP	TOTAL GRAMS	GRAMS/100K POP
1	MINNESOTA	5,303,925	53.02	1
2	OREGON	3,831,074	37.76	0.99
3	NORTH DAKOTA	672,591	6.03	0.9
4	DISTRICT OF COLUMBIA	601,723	5.22	0.87
5	IDAHO	1,567,582	10.44	0.67

6	MISSOURI	5,988,927	32.14	0.54
7	WISCONSIN	5,686,986	28.12	0.49
8	CALIFORNIA	37,253,956	142.84	0.38
9	RHODE ISLAND	1,052,567	3.62	0.34
10	ARIZONA	6,392,017	19.68	0.31
11	ALABAMA	4,779,736	14.86	0.31
12	HAWAII	1,360,301	3.21	0.24
13	NORTH CAROLINA	9,535,483	21.69	0.23
14	CONNECTICUT	3,574,097	8.03	0.22
15	PENNSYLVANIA	12,702,379	26.11	0.21
16	WEST VIRGINIA	1,852,994	3.62	0.2
17	SOUTH CAROLINA	4,625,364	8.84	0.19
18	OKLAHOMA	3,751,351	7.23	0.19
19	OHIO	11,536,504	21.29	0.18
20	TEXAS	25,145,561	39.77	0.16
21	KANSAS	2,853,118	4.42	0.15
22	GEORGIA	9,687,653	11.25	0.12
23	ALASKA	710,231	0.8	0.11
24	LOUISIANA	4,533,372	4.42	0.1
25	MASSACHUSETTS	6,547,629	6.83	0.1
26	NEW YORK	19,378,102	17.76	0.09
27	MISSISSIPPI	2,967,297	2.41	0.08
28	KENTUCKY	4,339,367	3.62	0.08
29	MARYLAND	5,773,552	4.82	0.08
30	FLORIDA	18,801,310	12.45	0.07
31	NEW JERSEY	8,791,894	5.62	0.06
32	ILLINOIS	12,830,632	6.03	0.05
33	WASHINGTON	6,724,540	2.41	0.04
34	NEBRASKA	1,826,341	0.8	0.04
35	NEVADA	2,700,551	0.4	0.01
36	TENNESSEE	6,346,105	0.4	0.01
**U.S. GRAMS / PER 100K:**		312,825,210	577.97	0.18

DRUG CODE:1205 **DRUG NAME:LISDEXAMFETAMINE**

**RANK**	**STATE**	**2010 POP**	**TOTAL GRAMS**	**GRAMS/100K POP**
1	LOUISIANA	4,533,372	371,359.76	8,191.69
2	DELAWARE	897,934	57,216.87	6,372.06
3	ALABAMA	4,779,736	285,325.03	5,969.47
4	SOUTH DAKOTA	814,180	43,835.59	5,384.02
5	SOUTH CAROLINA	4,625,364	244,431.29	5,284.58
6	INDIANA	6,483,802	341,369.21	5,264.95
7	MISSISSIPPI	2,967,297	155,921.09	5,254.65
8	MAINE	1,328,361	67,310.50	5,067.18
9	WISCONSIN	5,686,986	267,551.62	4,704.63
10	IOWA	3,046,355	137,639.83	4,518.18
11	KANSAS	2,853,118	127,152.52	4,456.62
12	MISSOURI	5,988,927	266,212.74	4,445.08
13	VERMONT	625,741	27,704.29	4,427.44
14	TENNESSEE	6,346,105	280,599.16	4,421.60
15	TEXAS	25,145,561	1,088,975.06	4,330.69
16	NORTH CAROLINA	9,535,483	410,571.97	4,305.73
17	WEST VIRGINIA	1,852,994	78,587.49	4,241.11
18	GEORGIA	9,687,653	409,573.99	4,227.79
19	NEW HAMPSHIRE	1,316,470	55,638.08	4,226.31
20	ARKANSAS	2,915,918	121,203.61	4,156.62
21	CONNECTICUT	3,574,097	144,751.62	4,050.02
22	OKLAHOMA	3,751,351	146,677.10	3,909.98
23	KENTUCKY	4,339,367	169,631.26	3,909.12
24	OHIO	11,536,504	446,513.98	3,870.44
25	MARYLAND	5,773,552	223,456.52	3,870.35
26	NEBRASKA	1,826,341	65,765.84	3,600.96

27	DISTRICT OF COLUMBIA	601,723	21,009.89	3,491.62
28	NORTH DAKOTA	672,591	22,892.18	3,403.58
29	VIRGINIA	8,001,024	270,981.83	3,386.84
30	UTAH	2,763,885	86,605.74	3,133.48
31	FLORIDA	18,801,310	548,800.42	2,918.95
32	RHODE ISLAND	1,052,567	30,111.49	2,860.77
33	PENNSYLVANIA	12,702,379	343,601.25	2,705.01
34	MICHIGAN	9,883,640	237,852.60	2,406.53
35	MINNESOTA	5,303,925	126,679.63	2,388.41
36	MONTANA	989,415	22,910.10	2,315.52
37	IDAHO	1,567,582	35,397.06	2,258.07
38	ARIZONA	6,392,017	143,380.36	2,243.12
39	NEW JERSEY	8,791,894	195,960.87	2,228.88
40	ILLINOIS	12,830,632	283,440.12	2,209.09
41	COLORADO	5,029,196	111,032.78	2,207.76
42	MASSACHUSETTS	6,547,629	142,897.65	2,182.43
43	WASHINGTON	6,724,540	136,312.31	2,027.09
44	WYOMING	563,626	10,450.31	1,854.12
45	NEW YORK	19,378,102	338,418.49	1,746.40
46	OREGON	3,831,074	60,117.75	1,569.21
47	ALASKA	710,231	9,865.85	1,389.11
48	CALIFORNIA	37,253,956	446,290.06	1,197.97
49	NEVADA	2,700,551	30,278.45	1,121.20
50	NEW MEXICO	2,059,179	17,259.75	838.19
51	HAWAII	1,360,301	5,358.41	393.91
52	VIRGIN ISLANDS	109,825	267.66	243.72
53	PUERTO RICO	3,725,789	7,605.48	204.13
54	GUAM	178,430	90.76	50.87
**U.S. GRAMS / PER 100K:**		312,825,210	9,720,845.25	3,107.44

DRUG CODE:1724 **DRUG NAME:METHYLPHENIDATE (DL;D;L;ISOMERS)**

**RANK**	**STATE**	**2010 POP**	**TOTAL GRAMS**	**GRAMS/100K POP**
1	VERMONT	625,741	76,927.33	12,293.80
2	SOUTH DAKOTA	814,180	92,985.17	11,420.71
3	IOWA	3,046,355	326,076.42	10,703.82
4	MAINE	1,328,361	141,224.78	10,631.51
5	NEW HAMPSHIRE	1,316,470	135,639.97	10,303.31
6	NORTH DAKOTA	672,591	60,128.60	8,939.85
7	MASSACHUSETTS	6,547,629	584,650.30	8,929.19
8	MISSOURI	5,988,927	526,374.97	8,789.14
9	DELAWARE	897,934	75,095.75	8,363.17
10	OHIO	11,536,504	926,895.09	8,034.45
11	NORTH CAROLINA	9,535,483	762,179.72	7,993.09
12	SOUTH CAROLINA	4,625,364	353,772.19	7,648.53
13	MICHIGAN	9,883,640	754,875.69	7,637.63
14	MINNESOTA	5,303,925	403,340.78	7,604.57
15	INDIANA	6,483,802	485,299.10	7,484.79
16	LOUISIANA	4,533,372	338,720.36	7,471.71
17	WISCONSIN	5,686,986	413,664.73	7,273.88
18	RHODE ISLAND	1,052,567	75,299.27	7,153.87
19	PENNSYLVANIA	12,702,379	899,814.76	7,083.83
20	MONTANA	989,415	69,975.85	7,072.45
21	KANSAS	2,853,118	200,253.28	7,018.75
22	IDAHO	1,567,582	109,587.11	6,990.84
23	MARYLAND	5,773,552	400,198.33	6,931.58
24	KENTUCKY	4,339,367	300,167.86	6,917.32
25	NEBRASKA	1,826,341	125,140.37	6,851.97
26	WEST VIRGINIA	1,852,994	126,376.79	6,820.14
27	GEORGIA	9,687,653	653,705.32	6,747.82
28	VIRGINIA	8,001,024	535,783.95	6,696.44
29	UTAH	2,763,885	184,160.98	6,663.12

30	WASHINGTON	6,724,540	446,856.52	6,645.16
31	OREGON	3,831,074	245,904.22	6,418.68
32	ALABAMA	4,779,736	290,869.30	6,085.47
33	CONNECTICUT	3,574,097	216,315.94	6,052.32
34	WYOMING	563,626	32,575.98	5,779.72
35	MISSISSIPPI	2,967,297	171,319.72	5,773.60
36	ARKANSAS	2,915,918	160,100.85	5,490.58
37	ILLINOIS	12,830,632	696,305.50	5,426.90
38	DISTRICT OF COLUMBIA	601,723	32,200	5,351.30
39	TEXAS	25,145,561	1,336,775.33	5,316.15
40	TENNESSEE	6,346,105	335,642.81	5,288.96
41	OKLAHOMA	3,751,351	193,512.61	5,158.48
42	COLORADO	5,029,196	246,395.47	4,899.30
43	NEW YORK	19,378,102	910,080.54	4,696.44
44	ARIZONA	6,392,017	298,120.31	4,663.95
45	ALASKA	710,231	32,138.95	4,525.14
46	FLORIDA	18,801,310	822,773.66	4,376.15
47	NEW JERSEY	8,791,894	366,780.33	4,171.80
48	NEW MEXICO	2,059,179	76,270.43	3,703.92
49	HAWAII	1,360,301	43,636.31	3,207.84
50	CALIFORNIA	37,253,956	1,087,983.37	2,920.45
51	NEVADA	2,700,551	59,241.28	2,193.67
52	PUERTO RICO	3,725,789	67,864.79	1,821.49
53	GUAM	178,430	1,914.75	1,073.11
54	VIRGIN ISLANDS	109,825	597.19	543.76
55	AMERICAN SAMOA	65,628	17.73	27.01
**U.S. GRAMS / PER 100K:**		312,825,210	18,310,508.74	5,853.27

DRUG CODE:2100 1308.13(C)(3)] **DRUG NAME:BARBITURIC ACID DERIVIATIVE OR SALT [PER 21CFR**

**RANK**	**STATE**	**2010 POP**	**TOTAL GRAMS**	**GRAMS/100K POP**
1	NEVADA	2,700,551	18,275	676.71
2	FLORIDA	18,801,310	69,845	371.49
3	ARIZONA	6,392,017	20,250	316.8
4	OKLAHOMA	3,751,351	11,255	300.03
5	LOUISIANA	4,533,372	13,425	296.14
6	MICHIGAN	9,883,640	26,910	272.27
7	WYOMING	563,626	1,525	270.57
8	MISSOURI	5,988,927	15,820	264.15
9	HAWAII	1,360,301	3,380	248.47
10	CONNECTICUT	3,574,097	8,710	243.7
11	RHODE ISLAND	1,052,567	2,520	239.41
12	ILLINOIS	12,830,632	30,235	235.65
13	GEORGIA	9,687,653	21,025	217.03
14	NEW JERSEY	8,791,894	18,110	205.99
15	UTAH	2,763,885	5,685	205.69
16	DELAWARE	897,934	1,840	204.91
17	SOUTH CAROLINA	4,625,364	9,395	203.12
18	INDIANA	6,483,802	13,160	202.97
19	MONTANA	989,415	1,995	201.63
20	TEXAS	25,145,561	49,304.90	196.08
21	ARKANSAS	2,915,918	5,325	182.62
22	TENNESSEE	6,346,105	11,305	178.14
23	VIRGINIA	8,001,024	13,900	173.73
24	PENNSYLVANIA	12,702,379	21,675	170.64
25	KANSAS	2,853,118	4,820	168.94
26	KENTUCKY	4,339,367	7,259	167.28
27	MASSACHUSETTS	6,547,629	10,820	165.25
28	MARYLAND	5,773,552	9,540	165.24
29	NEBRASKA	1,826,341	2,970	162.62
30	NEW MEXICO	2,059,179	3,325	161.47
31	DISTRICT OF COLUMBIA	601,723	970	161.2

32	NORTH CAROLINA	9,535,483	15,265	160.09
33	CALIFORNIA	37,253,956	59,183.50	158.87
34	NEW HAMPSHIRE	1,316,470	1,995	151.54
35	OHIO	11,536,504	16,795	145.58
36	IDAHO	1,567,582	2,165	138.11
37	WASHINGTON	6,724,540	9,240	137.41
38	MISSISSIPPI	2,967,297	4,020	135.48
39	COLORADO	5,029,196	6,585	130.94
40	ALABAMA	4,779,736	6,145	128.56
41	MAINE	1,328,361	1,690	127.22
42	WEST VIRGINIA	1,852,994	2,120	114.41
43	WISCONSIN	5,686,986	6,360	111.83
44	SOUTH DAKOTA	814,180	905	111.15
45	OREGON	3,831,074	3,865	100.89
46	ALASKA	710,231	715	100.67
47	MINNESOTA	5,303,925	5,315	100.21
48	VIRGIN ISLANDS	109,825	105	95.61
49	IOWA	3,046,355	2,780	91.26
50	NEW YORK	19,378,102	17,585	90.75
51	NORTH DAKOTA	672,591	565	84
52	VERMONT	625,741	285	45.55
53	PUERTO RICO	3,725,789	605	16.24
54	GUAM	178,430	10	5.6
**U.S. GRAMS / PER 100K:**		312,825,210	598,872.40	191.44

DRUG CODE:2125 **DRUG NAME:AMOBARBITAL (SCHEDULE 2)**

RANK	STATE	2010 POP	TOTAL GRAMS	GRAMS/100K POP
1	COLORADO	5,029,196	68.36	1.36
2	UTAH	2,763,885	34.18	1.24
3	DISTRICT OF COLUMBIA	601,723	6.84	1.14
4	NEW HAMPSHIRE	1,316,470	12.3	0.93
5	NEW YORK	19,378,102	152.66	0.79
6	ARIZONA	6,392,017	45.11	0.71
7	WASHINGTON	6,724,540	36.91	0.55
8	KANSAS	2,853,118	13.67	0.48
9	WISCONSIN	5,686,986	26.89	0.47
10	MARYLAND	5,773,552	27.34	0.47
11	KENTUCKY	4,339,367	19.14	0.44
12	MINNESOTA	5,303,925	23.24	0.44
13	NORTH CAROLINA	9,535,483	38.28	0.4
14	NEW JERSEY	8,791,894	30.53	0.35
15	RHODE ISLAND	1,052,567	3.65	0.35
16	TEXAS	25,145,561	83.39	0.33
17	SOUTH CAROLINA	4,625,364	15.04	0.33
18	MASSACHUSETTS	6,547,629	20.05	0.31
19	CALIFORNIA	37,253,956	100.71	0.27
20	GEORGIA	9,687,653	26.43	0.27
21	TENNESSEE	6,346,105	15.95	0.25
22	PENNSYLVANIA	12,702,379	29.16	0.23
23	MAINE	1,328,361	2.73	0.21
24	NEBRASKA	1,826,341	3.65	0.2
25	MICHIGAN	9,883,640	18.68	0.19
26	OREGON	3,831,074	6.84	0.18
27	MISSISSIPPI	2,967,297	4.56	0.15
28	VERMONT	625,741	0.91	0.15
29	OHIO	11,536,504	17.77	0.15
30	VIRGINIA	8,001,024	11.39	0.14
31	FLORIDA	18,801,310	23.24	0.12
32	ILLINOIS	12,830,632	15.95	0.12
33	MISSOURI	5,988,927	6.84	0.11
34	WEST VIRGINIA	1,852,994	1.82	0.1

35	CONNECTICUT	3,574,097	3.65	0.1
36	INDIANA	6,483,802	4.1	0.06
37	ARKANSAS	2,915,918	0.91	0.03
38	IOWA	3,046,355	0.46	0.01
**U.S. GRAMS / PER 100K:**		312,825,210	953.32	0.3

DRUG CODE:2165 **DRUG NAME:BUTALBITAL**

**RANK**	**STATE**	**2010 POP**	**TOTAL GRAMS**	**GRAMS/100K POP**
1	NEVADA	2,700,551	14,815	548.59
2	OKLAHOMA	3,751,351	19,990	532.87
3	ARKANSAS	2,915,918	13,765	472.06
4	UTAH	2,763,885	12,720	460.22
5	GEORGIA	9,687,653	39,800	410.83
6	MISSOURI	5,988,927	23,750	396.57
7	ALABAMA	4,779,736	17,800	372.41
8	PENNSYLVANIA	12,702,379	46,800	368.43
9	MICHIGAN	9,883,640	35,520	359.38
10	FLORIDA	18,801,310	65,720	349.55
11	LOUISIANA	4,533,372	15,750	347.42
12	CONNECTICUT	3,574,097	12,300	344.14
13	IDAHO	1,567,582	5,245	334.59
14	MARYLAND	5,773,552	18,990	328.91
15	ARIZONA	6,392,017	20,535	321.26
16	SOUTH DAKOTA	814,180	2,595	318.73
17	MONTANA	989,415	3,080	311.3
18	KANSAS	2,853,118	8,805	308.61
19	HAWAII	1,360,301	4,160	305.81
20	TEXAS	25,145,561	74,795	297.45
21	SOUTH CAROLINA	4,625,364	13,480	291.44
22	WYOMING	563,626	1,630	289.2
23	MISSISSIPPI	2,967,297	8,545	287.97
24	NEW JERSEY	8,791,894	24,670	280.6
25	WEST VIRGINIA	1,852,994	5,070	273.61
26	CALIFORNIA	37,253,956	98,310	263.89
27	VIRGINIA	8,001,024	20,870	260.84
28	WASHINGTON	6,724,540	17,405	258.83
29	OREGON	3,831,074	9,385	244.97
30	NEBRASKA	1,826,341	4,435	242.84
31	TENNESSEE	6,346,105	15,265	240.54
32	INDIANA	6,483,802	15,595	240.52
33	KENTUCKY	4,339,367	10,365	238.86
34	DELAWARE	897,934	2,110	234.98
35	DISTRICT OF COLUMBIA	601,723	1,400	232.67
36	ILLINOIS	12,830,632	29,660	231.17
37	OHIO	11,536,504	26,630	230.83
38	NORTH CAROLINA	9,535,483	20,390	213.83
39	NEW HAMPSHIRE	1,316,470	2,665	202.44
40	NORTH DAKOTA	672,591	1,285	191.05
41	MAINE	1,328,361	2,520	189.71
42	NEW MEXICO	2,059,179	3,700	179.68
43	IOWA	3,046,355	5,100	167.41
44	COLORADO	5,029,196	7,810	155.29
45	MASSACHUSETTS	6,547,629	9,300	142.04
46	RHODE ISLAND	1,052,567	1,470	139.66
47	ALASKA	710,231	925	130.24
48	MINNESOTA	5,303,925	6,705	126.42
49	WISCONSIN	5,686,986	6,995	123
50	NEW YORK	19,378,102	22,775	117.53
51	VERMONT	625,741	470	75.11
52	VIRGIN ISLANDS	109,825	45	40.97
53	GUAM	178,430	40	22.42

54	AMERICAN SAMOA	65,628	10	15.24
**U.S. GRAMS / PER 100K:**		312,825,210	853,965	272.98

DRUG CODE:2270 **DRUG NAME:PENTOBARBITAL (SCHEDULE 2)**

RANK	STATE	2010 POP	TOTAL GRAMS	GRAMS/100K POP
1	VERMONT	625,741	54,362.24	8,687.66
2	MONTANA	989,415	74,125.43	7,491.84
3	WYOMING	563,626	40,470.26	7,180.34
4	MAINE	1,328,361	80,880.53	6,088.75
5	IDAHO	1,567,582	90,449.41	5,770
6	NEW HAMPSHIRE	1,316,470	61,925.27	4,703.89
7	NEBRASKA	1,826,341	82,467.93	4,515.47
8	NORTH DAKOTA	672,591	28,368.24	4,217.75
9	OREGON	3,831,074	154,686.71	4,037.69
10	NEW MEXICO	2,059,179	80,303.10	3,899.76
11	MICHIGAN	9,883,640	384,941.28	3,894.73
12	WISCONSIN	5,686,986	209,420.50	3,682.45
13	GUAM	178,430	6,430.84	3,604.12
14	WASHINGTON	6,724,540	240,819.92	3,581.21
15	COLORADO	5,029,196	179,455.55	3,568.28
16	ARIZONA	6,392,017	215,066.18	3,364.61
17	ALASKA	710,231	22,791.38	3,209.01
18	KANSAS	2,853,118	90,490.17	3,171.62
19	SOUTH DAKOTA	814,180	24,992.87	3,069.70
20	INDIANA	6,483,802	179,784.67	2,772.83
21	MINNESOTA	5,303,925	144,879.78	2,731.56
22	MISSOURI	5,988,927	161,027.78	2,688.76
23	KENTUCKY	4,339,367	111,343.42	2,565.89
24	OHIO	11,536,504	295,121.79	2,558.16
25	NORTH CAROLINA	9,535,483	242,534.37	2,543.49
26	VIRGINIA	8,001,024	190,989.91	2,387.07
27	IOWA	3,046,355	71,503.15	2,347.17
28	RHODE ISLAND	1,052,567	24,562.40	2,333.57
29	NEVADA	2,700,551	61,755.73	2,286.78
30	WEST VIRGINIA	1,852,994	42,101.54	2,272.08
31	TENNESSEE	6,346,105	142,593.51	2,246.95
32	LOUISIANA	4,533,372	100,335.42	2,213.26
33	ILLINOIS	12,830,632	277,261.83	2,160.94
34	PENNSYLVANIA	12,702,379	269,190.85	2,119.22
35	UTAH	2,763,885	56,914.89	2,059.24
36	HAWAII	1,360,301	27,943.98	2,054.25
37	OKLAHOMA	3,751,351	76,112.70	2,028.94
38	CONNECTICUT	3,574,097	72,452.06	2,027.14
39	TEXAS	25,145,561	496,593.15	1,974.87
40	CALIFORNIA	37,253,956	708,793.68	1,902.60
41	MASSACHUSETTS	6,547,629	112,758.41	1,722.13
42	GEORGIA	9,687,653	153,814.35	1,587.74
43	NEW YORK	19,378,102	306,395.35	1,581.14
44	MARYLAND	5,773,552	88,930.58	1,540.31
45	FLORIDA	18,801,310	284,606.14	1,513.76
46	ALABAMA	4,779,736	65,593.55	1,372.33
47	ARKANSAS	2,915,918	36,427.18	1,249.25
48	SOUTH CAROLINA	4,625,364	55,714.74	1,204.55
49	MISSISSIPPI	2,967,297	32,523.41	1,096.06
50	NEW JERSEY	8,791,894	81,918.15	931.75
51	DELAWARE	897,934	7,148.89	796.15
52	DISTRICT OF COLUMBIA	601,723	2,552.83	424.25
53	PUERTO RICO	3,725,789	0.2	0.01
U.S. GRAMS / PER 100K:		312,825,210	7,104,628.21	2,271.12

DRUG CODE:2315 **DRUG NAME:SECOBARBITAL (SCHEDULE 2)**

CUMULATIVE DISTRIBUTION BY STATE IN GRAMS PER 100,000 POPULATION

RANK	STATE	2010 POP	TOTAL GRAMS	GRAMS/100K POP
1	OREGON	3,831,074	952.12	24.85
2	VERMONT	625,741	137.33	21.95
3	CALIFORNIA	37,253,956	4,376.09	11.75
4	WASHINGTON	6,724,540	723.25	10.76
5	NEW YORK	19,378,102	1,501.42	7.75
6	COLORADO	5,029,196	375.36	7.46
7	MARYLAND	5,773,552	219.72	3.81
8	KANSAS	2,853,118	100.71	3.53
9	RHODE ISLAND	1,052,567	36.62	3.48
10	DELAWARE	897,934	27.47	3.06
11	MASSACHUSETTS	6,547,629	192.26	2.94
12	NEW JERSEY	8,791,894	228.88	2.6
13	CONNECTICUT	3,574,097	91.55	2.56
14	PENNSYLVANIA	12,702,379	265.5	2.09
15	NEW HAMPSHIRE	1,316,470	27.47	2.09
16	FLORIDA	18,801,310	366.2	1.95
17	MISSOURI	5,988,927	100.71	1.68
18	VIRGINIA	8,001,024	109.86	1.37
19	ILLINOIS	12,830,632	164.79	1.28
20	OHIO	11,536,504	137.33	1.19
21	SOUTH DAKOTA	814,180	9.16	1.12
22	OKLAHOMA	3,751,351	36.62	0.98
23	SOUTH CAROLINA	4,625,364	36.62	0.79
24	HAWAII	1,360,301	9.16	0.67
25	GEORGIA	9,687,653	45.78	0.47
26	MINNESOTA	5,303,925	18.31	0.35
27	NEVADA	2,700,551	9.16	0.34
28	ARIZONA	6,392,017	18.31	0.29
29	MICHIGAN	9,883,640	18.31	0.19
30	TEXAS	25,145,561	45.78	0.18
31	WISCONSIN	5,686,986	9.16	0.16
32	TENNESSEE	6,346,105	9.16	0.14
33	NORTH CAROLINA	9,535,483	9.16	0.1
**U.S. GRAMS / PER 100K:**		312,825,210	10,409.24	3.33

DRUG CODE:7365 DRUG PRODUCT (SYNI DRUG NAME:DRONABINOL IN AN ORAL SOLUTION IN FDA APPROVED

RANK	STATE	2010 POP	TOTAL GRAMS	GRAMS/100K POP
1	NEW JERSEY	8,791,894	143.55	1.63
2	NEVADA	2,700,551	11.55	0.43
3	ARIZONA	6,392,017	21.6	0.34
4	FLORIDA	18,801,310	36.6	0.19
5	MISSOURI	5,988,927	8.55	0.14
6	NEW HAMPSHIRE	1,316,470	1.8	0.14
7	PENNSYLVANIA	12,702,379	16.35	0.13
8	CALIFORNIA	37,253,956	43.8	0.12
9	DISTRICT OF COLUMBIA	601,723	0.75	0.12
10	ALABAMA	4,779,736	4.2	0.09
11	OHIO	11,536,504	8.7	0.08
12	COLORADO	5,029,196	3.75	0.07
13	MASSACHUSETTS	6,547,629	3.75	0.06
14	TENNESSEE	6,346,105	3.6	0.06
15	MINNESOTA	5,303,925	2.85	0.05
16	CONNECTICUT	3,574,097	1.65	0.05
17	TEXAS	25,145,561	11.7	0.05
18	GEORGIA	9,687,653	4.65	0.05
19	ARKANSAS	2,915,918	1.2	0.04
20	MISSISSIPPI	2,967,297	0.75	0.03
21	KENTUCKY	4,339,367	1.5	0.03
22	MARYLAND	5,773,552	1.95	0.03
23	MONTANA	989,415	0.3	0.03

24	INDIANA	6,483,802	1.65	0.03
25	KANSAS	2,853,118	0.6	0.02
26	DELAWARE	897,934	0.15	0.02
27	MAINE	1,328,361	0.3	0.02
28	UTAH	2,763,885	0.6	0.02
29	MICHIGAN	9,883,640	2.4	0.02
30	NEW MEXICO	2,059,179	0.15	0.01
31	SOUTH CAROLINA	4,625,364	0.3	0.01
32	ILLINOIS	12,830,632	1.05	0.01
33	VIRGINIA	8,001,024	0.75	0.01
34	LOUISIANA	4,533,372	0.3	0.01
35	NEW YORK	19,378,102	1.95	0.01
36	NORTH CAROLINA	9,535,483	1.05	0.01
37	WISCONSIN	5,686,986	0.15	0
**U.S. GRAMS / PER 100K:**		312,825,210	346.5	0.11

DRUG CODE:7379 **DRUG NAME:NABILONE**

**RANK**	**STATE**	**2010 POP**	**TOTAL GRAMS**	**GRAMS/100K POP**
1	MARYLAND	5,773,552	4.1	0.07
2	HAWAII	1,360,301	0.6	0.04
3	NEBRASKA	1,826,341	0.3	0.02
4	MAINE	1,328,361	0.3	0.02
5	MISSOURI	5,988,927	0.55	0.01
6	COLORADO	5,029,196	0.35	0.01
7	NORTH CAROLINA	9,535,483	0.75	0.01
8	KENTUCKY	4,339,367	0.35	0.01
9	VIRGINIA	8,001,024	0.75	0.01
10	OHIO	11,536,504	1	0.01
11	CONNECTICUT	3,574,097	0.35	0.01
12	FLORIDA	18,801,310	2.15	0.01
13	PENNSYLVANIA	12,702,379	0.35	0
14	ARIZONA	6,392,017	0.2	0
15	MINNESOTA	5,303,925	0.1	0
16	NEW YORK	19,378,102	0.4	0
17	NEW JERSEY	8,791,894	0.35	0
18	NEW MEXICO	2,059,179	0.1	0
19	IOWA	3,046,355	0.1	0
20	OREGON	3,831,074	0.05	0
21	CALIFORNIA	37,253,956	0.65	0
22	ILLINOIS	12,830,632	0.5	0
23	NEW HAMPSHIRE	1,316,470	0.05	0
24	WASHINGTON	6,724,540	0.05	0
**U.S. GRAMS / PER 100K:**		312,825,210	14.45	0

DRUG CODE:9041L **DRUG NAME:COCAINE**

**RANK**	**STATE**	**2010 POP**	**TOTAL GRAMS**	**GRAMS/100K POP**
1	NORTH DAKOTA	672,591	181.99	27.06
2	MONTANA	989,415	211.59	21.39
3	SOUTH DAKOTA	814,180	148.85	18.28
4	MINNESOTA	5,303,925	855.52	16.13
5	DISTRICT OF COLUMBIA	601,723	92.89	15.44
6	MICHIGAN	9,883,640	1,507.87	15.26
7	NEW HAMPSHIRE	1,316,470	190.31	14.46
8	UTAH	2,763,885	391.01	14.15
9	NEW MEXICO	2,059,179	290.41	14.1
10	IDAHO	1,567,582	216.56	13.81
11	IOWA	3,046,355	393.01	12.9
12	MASSACHUSETTS	6,547,629	815.45	12.45
13	INDIANA	6,483,802	800.91	12.35
14	WYOMING	563,626	69.28	12.29

15	PENNSYLVANIA	12,702,379	1,556.72	12.26
16	KANSAS	2,853,118	328.62	11.52
17	MISSISSIPPI	2,967,297	338.98	11.42
18	MISSOURI	5,988,927	659.56	11.01
19	WEST VIRGINIA	1,852,994	201.81	10.89
20	WISCONSIN	5,686,986	604.82	10.64
21	TENNESSEE	6,346,105	670.01	10.56
22	ILLINOIS	12,830,632	1,290.13	10.06
23	OREGON	3,831,074	384.55	10.04
24	KENTUCKY	4,339,367	434.4	10.01
25	CALIFORNIA	37,253,956	3,470.56	9.32
26	MARYLAND	5,773,552	517.57	8.96
27	LOUISIANA	4,533,372	389.44	8.59
28	VIRGINIA	8,001,024	614.53	7.68
29	NEW JERSEY	8,791,894	645.74	7.34
30	RHODE ISLAND	1,052,567	76	7.22
31	HAWAII	1,360,301	97.42	7.16
32	NEW YORK	19,378,102	1,379.88	7.12
33	COLORADO	5,029,196	343.05	6.82
34	GEORGIA	9,687,653	656.89	6.78
35	WASHINGTON	6,724,540	454.08	6.75
36	ALASKA	710,231	47.93	6.75
37	NEVADA	2,700,551	179.95	6.66
38	MAINE	1,328,361	87.42	6.58
39	ARIZONA	6,392,017	419.58	6.56
40	NEBRASKA	1,826,341	119.17	6.53
41	OHIO	11,536,504	730.77	6.33
42	FLORIDA	18,801,310	1,162.10	6.18
43	VERMONT	625,741	38.46	6.15
44	ARKANSAS	2,915,918	175.6	6.02
45	NORTH CAROLINA	9,535,483	571.68	6
46	TEXAS	25,145,561	1,453.69	5.78
47	DELAWARE	897,934	51.85	5.77
48	ALABAMA	4,779,736	228.7	4.78
49	CONNECTICUT	3,574,097	150.92	4.22
50	OKLAHOMA	3,751,351	132.99	3.55
51	SOUTH CAROLINA	4,625,364	118.31	2.56
52	GUAM	178,430	0.36	0.2
**U.S. GRAMS / PER 100K:**		312,825,210	26,949.90	8.62

**DRUG CODE:9050**	**DRUG NAME:CODEINE**			
**RANK**	**STATE**	**2010 POP**	**TOTAL GRAMS**	**GRAMS/100K POP**
1	TEXAS	25,145,561	4,007,558.98	15,937.44
2	MICHIGAN	9,883,640	1,025,274.84	10,373.45
3	OKLAHOMA	3,751,351	381,918.42	10,180.82
4	ARKANSAS	2,915,918	292,809.02	10,041.74
5	DISTRICT OF COLUMBIA	601,723	48,420.46	8,046.97
6	WEST VIRGINIA	1,852,994	124,461.06	6,716.75
7	SOUTH CAROLINA	4,625,364	304,035.99	6,573.23
8	TENNESSEE	6,346,105	341,484.13	5,381
9	CALIFORNIA	37,253,956	1,891,720.41	5,077.90
10	ILLINOIS	12,830,632	647,278.14	5,044.79
11	ARIZONA	6,392,017	297,148.54	4,648.74
12	MISSOURI	5,988,927	273,798.37	4,571.74
13	KENTUCKY	4,339,367	190,127.79	4,381.46
14	ALABAMA	4,779,736	208,891.64	4,370.36
15	PENNSYLVANIA	12,702,379	554,464.95	4,365.05
16	GEORGIA	9,687,653	420,264.84	4,338.15
17	DELAWARE	897,934	36,987.08	4,119.13
18	MONTANA	989,415	40,580.95	4,101.51
19	MARYLAND	5,773,552	231,927.48	4,017.07

20	HAWAII	1,360,301	54,562.75	4,011.08
21	NEVADA	2,700,551	106,949.08	3,960.27
22	KANSAS	2,853,118	111,795.90	3,918.38
23	ALASKA	710,231	27,797.47	3,913.86
24	LOUISIANA	4,533,372	173,795.65	3,833.69
25	NEW MEXICO	2,059,179	78,187	3,797
26	MISSISSIPPI	2,967,297	109,613.37	3,694.05
27	WASHINGTON	6,724,540	245,749.48	3,654.52
28	FLORIDA	18,801,310	681,387.45	3,624.15
29	OREGON	3,831,074	135,860.23	3,546.27
30	NEW JERSEY	8,791,894	310,813.87	3,535.23
31	OHIO	11,536,504	403,005.98	3,493.31
32	IDAHO	1,567,582	54,643.68	3,485.86
33	SOUTH DAKOTA	814,180	27,885.17	3,424.94
34	NORTH DAKOTA	672,591	22,778.39	3,386.66
35	WISCONSIN	5,686,986	190,554.17	3,350.71
36	MAINE	1,328,361	41,556.12	3,128.38
37	INDIANA	6,483,802	200,465.35	3,091.79
38	WYOMING	563,626	16,766.46	2,974.75
39	NEW YORK	19,378,102	567,749.93	2,929.85
40	MASSACHUSETTS	6,547,629	185,230.09	2,828.96
41	MINNESOTA	5,303,925	149,198.60	2,812.98
42	RHODE ISLAND	1,052,567	29,116.74	2,766.26
43	VIRGINIA	8,001,024	219,576.35	2,744.35
44	VIRGIN ISLANDS	109,825	2,999.03	2,730.73
45	CONNECTICUT	3,574,097	96,316.16	2,694.84
46	GUAM	178,430	4,765.67	2,670.89
47	IOWA	3,046,355	79,896.15	2,622.68
48	NEBRASKA	1,826,341	46,965.27	2,571.55
49	UTAH	2,763,885	65,371.67	2,365.21
50	NEW HAMPSHIRE	1,316,470	30,347.37	2,305.21
51	VERMONT	625,741	13,115.92	2,096.06
52	AMERICAN SAMOA	65,628	1,361.42	2,074.45
53	COLORADO	5,029,196	102,868.85	2,045.43
54	NORTH CAROLINA	9,535,483	193,617.17	2,030.49
55	PUERTO RICO	3,725,789	51,225.70	1,374.90
**U.S. GRAMS / PER 100K:**		312,825,210	16,153,042.74	5,163.60

**DRUG CODE:9056**	**DRUG NAME:ETORPHINE**			
**RANK**	**STATE**	**2010 POP**	**TOTAL GRAMS**	**GRAMS/100K POP**
1	WYOMING	563,626	57	10.11
**U.S. GRAMS / PER 100K:**		312,825,210	57	0.02

**DRUG CODE:9064**	**DRUG NAME:BUPRENORPHINE**			
**RANK**	**STATE**	**2010 POP**	**TOTAL GRAMS**	**GRAMS/100K POP**
1	VERMONT	625,741	25,219.07	4,030.27
2	WEST VIRGINIA	1,852,994	62,190.56	3,356.22
3	KENTUCKY	4,339,367	138,082.60	3,182.09
4	MAINE	1,328,361	38,720.40	2,914.90
5	RHODE ISLAND	1,052,567	30,097.29	2,859.42
6	MASSACHUSETTS	6,547,629	165,313.37	2,524.78
7	NEW HAMPSHIRE	1,316,470	32,093.27	2,437.83
8	TENNESSEE	6,346,105	139,285.76	2,194.82
9	ALABAMA	4,779,736	100,755.08	2,107.96
10	PENNSYLVANIA	12,702,379	252,087.71	1,984.57
11	OHIO	11,536,504	210,098.89	1,821.17
12	MARYLAND	5,773,552	102,062.77	1,767.76
13	LOUISIANA	4,533,372	76,313.99	1,683.38
14	CONNECTICUT	3,574,097	59,292.76	1,658.96
15	DELAWARE	897,934	14,515.91	1,616.59

16	DISTRICT OF COLUMBIA	601,723	9,454.34	1,571.21
17	NEW MEXICO	2,059,179	29,495.60	1,432.40
18	UTAH	2,763,885	38,562.44	1,395.23
19	NORTH CAROLINA	9,535,483	130,842.10	1,372.16
20	MISSISSIPPI	2,967,297	39,672.12	1,336.98
21	ALASKA	710,231	9,458.16	1,331.70
22	INDIANA	6,483,802	79,644.73	1,228.36
23	NEW JERSEY	8,791,894	105,552.79	1,200.57
24	OKLAHOMA	3,751,351	43,720.90	1,165.47
25	WASHINGTON	6,724,540	76,999.48	1,145.05
26	NEW YORK	19,378,102	213,357.64	1,101.02
27	MICHIGAN	9,883,640	108,644.60	1,099.24
28	OREGON	3,831,074	41,536.48	1,084.20
29	FLORIDA	18,801,310	187,652.68	998.08
30	VIRGINIA	8,001,024	79,458.64	993.11
31	MONTANA	989,415	9,652.16	975.54
32	SOUTH CAROLINA	4,625,364	42,638.77	921.85
33	IDAHO	1,567,582	14,216.04	906.88
34	WYOMING	563,626	5,032.89	892.95
35	ARKANSAS	2,915,918	25,056.38	859.3
36	WISCONSIN	5,686,986	41,104.34	722.78
37	GEORGIA	9,687,653	62,871.40	648.98
38	MISSOURI	5,988,927	38,226.66	638.29
39	ARIZONA	6,392,017	37,700.26	589.8
40	COLORADO	5,029,196	29,613.27	588.83
41	HAWAII	1,360,301	7,879.33	579.23
42	MINNESOTA	5,303,925	30,214.73	569.67
43	NORTH DAKOTA	672,591	3,635.35	540.5
44	NEVADA	2,700,551	13,645.22	505.28
45	ILLINOIS	12,830,632	57,527.89	448.36
46	TEXAS	25,145,561	105,336.32	418.91
47	CALIFORNIA	37,253,956	152,567.81	409.53
48	KANSAS	2,853,118	10,223.55	358.33
49	NEBRASKA	1,826,341	5,308.86	290.68
50	PUERTO RICO	3,725,789	9,956.33	267.23
51	IOWA	3,046,355	6,730.35	220.93
52	SOUTH DAKOTA	814,180	1,797.80	220.81
53	VIRGIN ISLANDS	109,825	62.72	57.11
54	GUAM	178,430	4.49	2.52
55	AMERICAN SAMOA	65,628	0.01	0.01
**U.S. GRAMS / PER 100K:**		312,825,210	3,351,185.07	1,071.26

DRUG CODE:9120 **DRUG NAME:DIHYDROCODEINE**

**RANK**	**STATE**	**2010 POP**	**TOTAL GRAMS**	**GRAMS/100K POP**
1	ALABAMA	4,779,736	3,149.36	65.89
2	LOUISIANA	4,533,372	2,552.46	56.3
3	MISSISSIPPI	2,967,297	1,605.98	54.12
4	TEXAS	25,145,561	11,029.18	43.86
5	TENNESSEE	6,346,105	1,937.03	30.52
6	OKLAHOMA	3,751,351	1,129.38	30.11
7	GEORGIA	9,687,653	2,198.02	22.69
8	ARKANSAS	2,915,918	568.56	19.5
9	NEW MEXICO	2,059,179	343.95	16.7
10	NEW JERSEY	8,791,894	1,248.75	14.2
11	IDAHO	1,567,582	108.95	6.95
12	DELAWARE	897,934	54.52	6.07
13	ILLINOIS	12,830,632	671.1	5.23
14	ARIZONA	6,392,017	319.49	5
15	FLORIDA	18,801,310	763.93	4.06
16	MISSOURI	5,988,927	184.79	3.09
17	PENNSYLVANIA	12,702,379	362.55	2.85

18	NEVADA	2,700,551	75.41	2.79
19	NEW YORK	19,378,102	529.27	2.73
20	CALIFORNIA	37,253,956	843.12	2.26
21	KENTUCKY	4,339,367	85.51	1.97
22	ALASKA	710,231	13.89	1.96
23	MASSACHUSETTS	6,547,629	126.04	1.93
24	OHIO	11,536,504	140.3	1.22
25	SOUTH CAROLINA	4,625,364	52.34	1.13
26	MARYLAND	5,773,552	38.08	0.66
27	NEBRASKA	1,826,341	11.96	0.66
28	KANSAS	2,853,118	18.16	0.64
29	WEST VIRGINIA	1,852,994	7.69	0.42
30	MICHIGAN	9,883,640	40.86	0.41
31	NORTH CAROLINA	9,535,483	38.45	0.4
32	WISCONSIN	5,686,986	21.36	0.38
33	VIRGINIA	8,001,024	26.7	0.33
34	RHODE ISLAND	1,052,567	3.2	0.3
35	COLORADO	5,029,196	9.61	0.19
36	MINNESOTA	5,303,925	9.83	0.19
37	VERMONT	625,741	1.07	0.17
38	SOUTH DAKOTA	814,180	1.07	0.13
39	INDIANA	6,483,802	7.48	0.12
40	MONTANA	989,415	1.07	0.11
41	CONNECTICUT	3,574,097	3.75	0.1
42	HAWAII	1,360,301	1.07	0.08
43	MAINE	1,328,361	1.07	0.08
44	IOWA	3,046,355	2.14	0.07
45	UTAH	2,763,885	1.07	0.04
**U.S. GRAMS / PER 100K:**		312,825,210	30,339.58	9.7

DRUG CODE:9143 **DRUG NAME:OXYCODONE**

**RANK**	**STATE**	**2010 POP**	**TOTAL GRAMS**	**GRAMS/100K POP**
1	DELAWARE	897,934	273,807.57	30,493.06
2	TENNESSEE	6,346,105	1,851,142.93	29,169.75
3	ARIZONA	6,392,017	1,700,401.77	26,601.96
4	NEVADA	2,700,551	717,291.03	26,560.91
5	UTAH	2,763,885	670,987.24	24,276.96
6	OKLAHOMA	3,751,351	896,742.56	23,904.52
7	PENNSYLVANIA	12,702,379	3,002,067.64	23,633.90
8	NORTH CAROLINA	9,535,483	2,239,073.49	23,481.49
9	NEW JERSEY	8,791,894	1,991,312.25	22,649.41
10	MARYLAND	5,773,552	1,295,377.50	22,436.40
11	FLORIDA	18,801,310	4,104,069.47	21,828.64
12	SOUTH CAROLINA	4,625,364	934,837.33	20,211.11
13	ARKANSAS	2,915,918	577,987.63	19,821.81
14	MISSOURI	5,988,927	1,184,669.07	19,780.99
15	ALASKA	710,231	137,795.62	19,401.52
16	ALABAMA	4,779,736	923,241.15	19,315.74
17	NEW HAMPSHIRE	1,316,470	249,380.84	18,943.15
18	KENTUCKY	4,339,367	815,681.77	18,797.25
19	CONNECTICUT	3,574,097	663,460.65	18,563.03
20	GEORGIA	9,687,653	1,782,625.27	18,401
21	WYOMING	563,626	102,168.83	18,127.06
22	OREGON	3,831,074	693,337.57	18,097.73
23	WEST VIRGINIA	1,852,994	332,908.26	17,965.97
24	COLORADO	5,029,196	892,607.05	17,748.50
25	NEW MEXICO	2,059,179	361,390.05	17,550.20
26	WASHINGTON	6,724,540	1,162,214.79	17,283.19
27	KANSAS	2,853,118	488,497.43	17,121.53
28	LOUISIANA	4,533,372	770,239.02	16,990.42
29	OHIO	11,536,504	1,926,530.19	16,699.43

30	RHODE ISLAND	1,052,567	162,522.08	15,440.55
31	MONTANA	989,415	152,351.22	15,398.11
32	IDAHO	1,567,582	241,353.25	15,396.53
33	MAINE	1,328,361	204,307.68	15,380.43
34	NEW YORK	19,378,102	2,951,716.13	15,232.23
35	WISCONSIN	5,686,986	857,719.93	15,082.15
36	VIRGINIA	8,001,024	1,186,158.02	14,825.08
37	MASSACHUSETTS	6,547,629	959,337.42	14,651.68
38	VERMONT	625,741	90,744.06	14,501.86
39	INDIANA	6,483,802	924,308.19	14,255.65
40	MISSISSIPPI	2,967,297	419,254.56	14,129.17
41	HAWAII	1,360,301	173,430.73	12,749.44
42	MICHIGAN	9,883,640	1,188,829.75	12,028.26
43	MINNESOTA	5,303,925	570,633.64	10,758.70
44	DISTRICT OF COLUMBIA	601,723	58,155.97	9,664.91
45	CALIFORNIA	37,253,956	3,452,832.26	9,268.36
46	NEBRASKA	1,826,341	166,248.79	9,102.83
47	NORTH DAKOTA	672,591	58,589.72	8,711.05
48	SOUTH DAKOTA	814,180	68,945.36	8,468.07
49	IOWA	3,046,355	213,823.68	7,019
50	TEXAS	25,145,561	1,324,316.26	5,266.60
51	ILLINOIS	12,830,632	632,613.48	4,930.49
52	GUAM	178,430	3,421.37	1,917.49
53	VIRGIN ISLANDS	109,825	2,102.10	1,914.04
54	PUERTO RICO	3,725,789	50,726.52	1,361.50
55	AMERICAN SAMOA	65,628	58.72	89.48
**U.S. GRAMS / PER 100K:**		312,825,210	48,856,346.90	15,617.78

**DRUG CODE:9150**	**DRUG NAME:HYDROMORPHONE**			
**RANK**	**STATE**	**2010 POP**	**TOTAL GRAMS**	**GRAMS/100K POP**
1	FLORIDA	18,801,310	186,726.94	993.16
2	DELAWARE	897,934	8,523.20	949.2
3	MISSISSIPPI	2,967,297	23,403.69	788.72
4	VERMONT	625,741	4,872.81	778.73
5	CONNECTICUT	3,574,097	25,575.75	715.59
6	MONTANA	989,415	6,564.46	663.47
7	WASHINGTON	6,724,540	42,826.80	636.87
8	NEW HAMPSHIRE	1,316,470	7,863.71	597.33
9	MARYLAND	5,773,552	33,414.42	578.75
10	VIRGINIA	8,001,024	44,944.26	561.73
11	ALASKA	710,231	3,897.24	548.73
12	COLORADO	5,029,196	27,198.61	540.81
13	OREGON	3,831,074	19,345.86	504.97
14	MAINE	1,328,361	6,686.40	503.36
15	SOUTH CAROLINA	4,625,364	22,976.43	496.75
16	KANSAS	2,853,118	13,977.87	489.92
17	IDAHO	1,567,582	7,525.79	480.09
18	PENNSYLVANIA	12,702,379	60,501.44	476.3
19	NORTH CAROLINA	9,535,483	43,037.48	451.34
20	MASSACHUSETTS	6,547,629	29,474.16	450.15
21	MINNESOTA	5,303,925	23,791.36	448.56
22	ARKANSAS	2,915,918	13,054.62	447.7
23	NEVADA	2,700,551	11,796.48	436.82
24	WYOMING	563,626	2,439.36	432.8
25	MISSOURI	5,988,927	25,802.13	430.83
26	TENNESSEE	6,346,105	26,656.40	420.04
27	ARIZONA	6,392,017	26,811.58	419.45
28	CALIFORNIA	37,253,956	155,292.82	416.85
29	NEW JERSEY	8,791,894	35,497.58	403.75
30	NEW YORK	19,378,102	78,061.42	402.83
31	UTAH	2,763,885	10,905.47	394.57

32	IOWA	3,046,355	11,892.16	390.37
33	OKLAHOMA	3,751,351	14,599.92	389.19
34	NORTH DAKOTA	672,591	2,595.36	385.87
35	TEXAS	25,145,561	95,409.81	379.43
36	INDIANA	6,483,802	23,828.90	367.51
37	MICHIGAN	9,883,640	36,152.05	365.78
38	DISTRICT OF COLUMBIA	601,723	2,167.32	360.19
39	GEORGIA	9,687,653	33,298.47	343.72
40	RHODE ISLAND	1,052,567	3,532.96	335.65
41	WEST VIRGINIA	1,852,994	6,054.18	326.72
42	OHIO	11,536,504	37,382.73	324.04
43	ILLINOIS	12,830,632	38,905.20	303.22
44	LOUISIANA	4,533,372	13,173.20	290.58
45	NEBRASKA	1,826,341	5,206.77	285.09
46	ALABAMA	4,779,736	13,037.33	272.76
47	KENTUCKY	4,339,367	11,793.33	271.78
48	SOUTH DAKOTA	814,180	2,199.71	270.17
49	NEW MEXICO	2,059,179	5,395.61	262.03
50	WISCONSIN	5,686,986	14,862.16	261.34
51	HAWAII	1,360,301	2,671.22	196.37
52	VIRGIN ISLANDS	109,825	74.15	67.51
53	GUAM	178,430	114.51	64.18
54	PUERTO RICO	3,725,789	296.63	7.96
55	AMERICAN SAMOA	65,628	0.98	1.49
**U.S. GRAMS / PER 100K:**		312,825,210	1,404,091.19	448.84

**DRUG CODE:9193**	**DRUG NAME:HYDROCODONE**			
**RANK**	**STATE**	**2010 POP**	**TOTAL GRAMS**	**GRAMS/100K POP**
1	ALABAMA	4,779,736	971,895.93	20,333.67
2	OKLAHOMA	3,751,351	725,850.95	19,349.05
3	KENTUCKY	4,339,367	799,568.80	18,425.93
4	ARKANSAS	2,915,918	535,677.07	18,370.79
5	MICHIGAN	9,883,640	1,595,184.20	16,139.64
6	MISSISSIPPI	2,967,297	466,547.22	15,722.97
7	TENNESSEE	6,346,105	972,848.06	15,329.84
8	IDAHO	1,567,582	228,059.05	14,548.46
9	LOUISIANA	4,533,372	652,080.15	14,384
10	NEVADA	2,700,551	377,505.15	13,978.82
11	INDIANA	6,483,802	904,907.84	13,956.44
12	WEST VIRGINIA	1,852,994	235,601.60	12,714.64
13	SOUTH CAROLINA	4,625,364	550,520.03	11,902.20
14	MISSOURI	5,988,927	707,948.92	11,820.96
15	KANSAS	2,853,118	324,671.16	11,379.52
16	TEXAS	25,145,561	2,658,309.26	10,571.68
17	MONTANA	989,415	102,878.49	10,397.91
18	GEORGIA	9,687,653	984,057.78	10,157.86
19	OREGON	3,831,074	376,701.12	9,832.78
20	CALIFORNIA	37,253,956	3,656,359.66	9,814.69
21	ILLINOIS	12,830,632	1,231,194.30	9,595.74
22	UTAH	2,763,885	233,877.54	8,461.91
23	WYOMING	563,626	46,009.42	8,163.11
24	NORTH CAROLINA	9,535,483	769,321.19	8,067.98
25	WASHINGTON	6,724,540	523,468.42	7,784.45
26	FLORIDA	18,801,310	1,440,018.56	7,659.14
27	IOWA	3,046,355	229,677.31	7,539.41
28	SOUTH DAKOTA	814,180	59,207.01	7,271.98
29	NEBRASKA	1,826,341	129,971.43	7,116.49
30	NEW MEXICO	2,059,179	139,543.68	6,776.67
31	ARIZONA	6,392,017	412,803.61	6,458.11
32	OHIO	11,536,504	742,900.80	6,439.57
33	ALASKA	710,231	44,530.10	6,269.80

34	NORTH DAKOTA	672,591	41,493.33	6,169.18
35	WISCONSIN	5,686,986	311,532.88	5,478
36	VIRGINIA	8,001,024	416,339.44	5,203.58
37	PENNSYLVANIA	12,702,379	660,088.57	5,196.57
38	COLORADO	5,029,196	259,918.58	5,168.19
39	MAINE	1,328,361	68,459.79	5,153.70
40	HAWAII	1,360,301	69,885.53	5,137.50
41	RHODE ISLAND	1,052,567	41,487.65	3,941.57
42	NEW YORK	19,378,102	681,149.81	3,515.05
43	MINNESOTA	5,303,925	182,581.75	3,442.39
44	VERMONT	625,741	19,137.62	3,058.39
45	NEW HAMPSHIRE	1,316,470	36,123.33	2,743.95
46	DELAWARE	897,934	23,960.06	2,668.35
47	MARYLAND	5,773,552	138,467.99	2,398.32
48	CONNECTICUT	3,574,097	80,359.40	2,248.38
49	MASSACHUSETTS	6,547,629	120,671.22	1,842.98
50	NEW JERSEY	8,791,894	138,128.04	1,571.08
51	VIRGIN ISLANDS	109,825	1,158.60	1,054.95
52	DISTRICT OF COLUMBIA	601,723	5,766.43	958.32
53	GUAM	178,430	629.98	353.07
54	PUERTO RICO	3,725,789	2,546.70	68.35
55	AMERICAN SAMOA	65,628	7.9	12.04
**U.S. GRAMS / PER 100K:**		312,825,210	27,129,590.40	8,672.44

**DRUG CODE:9220L**	**DRUG NAME:LEVORPHANOL**			
**RANK**	**STATE**	**2010 POP**	**TOTAL GRAMS**	**GRAMS/100K POP**
1	NEW JERSEY	8,791,894	1,620.24	18.43
2	NORTH CAROLINA	9,535,483	265.71	2.79
3	LOUISIANA	4,533,372	76.26	1.68
4	WASHINGTON	6,724,540	109.23	1.62
5	ARIZONA	6,392,017	99.94	1.56
6	INDIANA	6,483,802	97.39	1.5
7	MISSOURI	5,988,927	86.94	1.45
8	NEBRASKA	1,826,341	23.8	1.3
9	KANSAS	2,853,118	35.87	1.26
10	MASSACHUSETTS	6,547,629	80.68	1.23
11	CALIFORNIA	37,253,956	402.57	1.08
12	COLORADO	5,029,196	50.84	1.01
13	TEXAS	25,145,561	220.32	0.88
14	NEW YORK	19,378,102	161.12	0.83
15	FLORIDA	18,801,310	136.05	0.72
16	CONNECTICUT	3,574,097	20.2	0.57
17	NEVADA	2,700,551	14.16	0.52
18	IOWA	3,046,355	15.44	0.51
19	VIRGINIA	8,001,024	39	0.49
20	PENNSYLVANIA	12,702,379	53.63	0.42
21	ALABAMA	4,779,736	20.08	0.42
22	NEW HAMPSHIRE	1,316,470	5.34	0.41
23	ILLINOIS	12,830,632	52.7	0.41
24	OHIO	11,536,504	43.53	0.38
25	MARYLAND	5,773,552	21.36	0.37
26	OREGON	3,831,074	13.58	0.35
27	ARKANSAS	2,915,918	9.05	0.31
28	DELAWARE	897,934	2.55	0.28
29	WISCONSIN	5,686,986	15.55	0.27
30	VERMONT	625,741	1.63	0.26
31	SOUTH CAROLINA	4,625,364	11.03	0.24
32	GEORGIA	9,687,653	9.63	0.1
33	MINNESOTA	5,303,925	2.79	0.05
34	KENTUCKY	4,339,367	0.35	0.01
35	IDAHO	1,567,582	0.23	0.01

36	MISSISSIPPI	2,967,297	0.23	0.01
37	MICHIGAN	9,883,640	1.04	0.01
38	TENNESSEE	6,346,105	0.7	0.01
**U.S. GRAMS / PER 100K:**		312,825,210	3,820.77	1.22

DRUG CODE:9230 **DRUG NAME:MEPERIDINE (PETHIDINE)**

**RANK**	**STATE**	**2010 POP**	**TOTAL GRAMS**	**GRAMS/100K POP**
1	ARKANSAS	2,915,918	29,599.41	1,015.10
2	OKLAHOMA	3,751,351	28,655.36	763.87
3	LOUISIANA	4,533,372	29,019.25	640.13
4	ALABAMA	4,779,736	29,415.19	615.41
5	MISSISSIPPI	2,967,297	17,314.55	583.51
6	SOUTH CAROLINA	4,625,364	23,517.21	508.44
7	TENNESSEE	6,346,105	29,692.14	467.88
8	PUERTO RICO	3,725,789	17,371.83	466.26
9	GEORGIA	9,687,653	41,591.25	429.32
10	ALASKA	710,231	2,851	401.42
11	ARIZONA	6,392,017	19,505.61	305.16
12	VIRGINIA	8,001,024	23,279.05	290.95
13	NEW MEXICO	2,059,179	5,650.52	274.41
14	MONTANA	989,415	2,686.31	271.51
15	UTAH	2,763,885	7,195.56	260.34
16	NORTH CAROLINA	9,535,483	24,541.88	257.37
17	IOWA	3,046,355	7,780.88	255.42
18	SOUTH DAKOTA	814,180	2,076.24	255.01
19	MISSOURI	5,988,927	15,210.49	253.98
20	KANSAS	2,853,118	7,071.05	247.84
21	VIRGIN ISLANDS	109,825	253.93	231.22
22	WYOMING	563,626	1,301.65	230.94
23	KENTUCKY	4,339,367	9,777.45	225.32
24	IDAHO	1,567,582	3,330.02	212.43
25	NEVADA	2,700,551	5,670.87	209.99
26	INDIANA	6,483,802	13,425.65	207.06
27	VERMONT	625,741	1,287.86	205.81
28	WASHINGTON	6,724,540	13,543.42	201.4
29	FLORIDA	18,801,310	35,814.51	190.49
30	TEXAS	25,145,561	46,330.33	184.25
31	NEW HAMPSHIRE	1,316,470	2,349.89	178.5
32	OHIO	11,536,504	19,980.92	173.2
33	CALIFORNIA	37,253,956	62,372.41	167.42
34	MARYLAND	5,773,552	9,370.04	162.29
35	OREGON	3,831,074	6,115.40	159.63
36	NEBRASKA	1,826,341	2,784.97	152.49
37	COLORADO	5,029,196	7,279.03	144.74
38	NORTH DAKOTA	672,591	951.79	141.51
39	WEST VIRGINIA	1,852,994	2,490.42	134.4
40	MASSACHUSETTS	6,547,629	8,455.01	129.13
41	DISTRICT OF COLUMBIA	601,723	754.61	125.41
42	CONNECTICUT	3,574,097	4,262.51	119.26
43	MAINE	1,328,361	1,578.18	118.81
44	PENNSYLVANIA	12,702,379	14,199.18	111.78
45	MICHIGAN	9,883,640	10,928.39	110.57
46	GUAM	178,430	183.67	102.94
47	WISCONSIN	5,686,986	5,852.99	102.92
48	HAWAII	1,360,301	1,357.25	99.78
49	DELAWARE	897,934	870.74	96.97
50	RHODE ISLAND	1,052,567	1,006.80	95.65
51	NEW YORK	19,378,102	18,289.21	94.38
52	ILLINOIS	12,830,632	12,010.75	93.61
53	NEW JERSEY	8,791,894	7,702.75	87.61
54	MINNESOTA	5,303,925	2,733.90	51.54

CUMULATIVE DISTRIBUTION BY STATE IN GRAMS PER 100,000 POPULATION

**U.S. GRAMS / PER 100K:**		312,825,210	698,641.25	223.33

DRUG CODE:9250B **DRUG NAME:METHADONE**

RANK	STATE	2010 POP	TOTAL GRAMS	GRAMS/100K POP
1	RHODE ISLAND	1,052,567	177,108.96	16,826.38
2	CONNECTICUT	3,574,097	476,404.75	13,329.37
3	MARYLAND	5,773,552	764,273.85	13,237.50
4	DELAWARE	897,934	109,993.34	12,249.60
5	VERMONT	625,741	73,999.26	11,825.86
6	MAINE	1,328,361	126,225.33	9,502.34
7	NEW HAMPSHIRE	1,316,470	123,333.65	9,368.51
8	MASSACHUSETTS	6,547,629	579,374.66	8,848.62
9	NEW MEXICO	2,059,179	176,396.43	8,566.35
10	DISTRICT OF COLUMBIA	601,723	39,909.39	6,632.52
11	OREGON	3,831,074	253,755.79	6,623.62
12	ALABAMA	4,779,736	312,422.11	6,536.39
13	NEW YORK	19,378,102	1,219,390.72	6,292.62
14	PENNSYLVANIA	12,702,379	789,307.56	6,213.86
15	WASHINGTON	6,724,540	390,840.64	5,812.15
16	NORTH CAROLINA	9,535,483	526,496.35	5,521.44
17	MICHIGAN	9,883,640	534,012.15	5,402.99
18	NEVADA	2,700,551	144,696.01	5,358.02
19	ARIZONA	6,392,017	335,733.41	5,252.39
20	NEW JERSEY	8,791,894	459,561.04	5,227.10
21	INDIANA	6,483,802	326,935.08	5,042.34
22	WEST VIRGINIA	1,852,994	92,570.31	4,995.72
23	SOUTH CAROLINA	4,625,364	225,378.07	4,872.66
24	GEORGIA	9,687,653	465,598.88	4,806.11
25	KENTUCKY	4,339,367	206,121.05	4,750.03
26	WISCONSIN	5,686,986	262,147.43	4,609.60
27	ALASKA	710,231	32,086.96	4,517.82
28	OKLAHOMA	3,751,351	157,005.48	4,185.30
29	FLORIDA	18,801,310	782,712.94	4,163.08
30	VIRGINIA	8,001,024	317,072.93	3,962.90
31	MINNESOTA	5,303,925	207,811.17	3,918.06
32	UTAH	2,763,885	105,686.42	3,823.84
33	ILLINOIS	12,830,632	489,424.13	3,814.50
34	CALIFORNIA	37,253,956	1,388,784.67	3,727.89
35	COLORADO	5,029,196	173,018.55	3,440.28
36	KANSAS	2,853,118	92,445.63	3,240.16
37	LOUISIANA	4,533,372	142,636.71	3,146.37
38	TENNESSEE	6,346,105	194,303.46	3,061.78
39	IDAHO	1,567,582	47,232.82	3,013.10
40	MONTANA	989,415	29,220.94	2,953.36
41	MISSOURI	5,988,927	165,981.13	2,771.47
42	OHIO	11,536,504	297,313.24	2,577.15
43	HAWAII	1,360,301	32,295.71	2,374.16
44	PUERTO RICO	3,725,789	84,028.61	2,255.32
45	TEXAS	25,145,561	562,018.41	2,235.06
46	ARKANSAS	2,915,918	64,241.67	2,203.14
47	NEBRASKA	1,826,341	32,724.69	1,791.82
48	IOWA	3,046,355	52,271.15	1,715.86
49	NORTH DAKOTA	672,591	8,372.39	1,244.80
50	SOUTH DAKOTA	814,180	8,757.88	1,075.67
51	MISSISSIPPI	2,967,297	26,643.64	897.91
52	WYOMING	563,626	4,756.51	843.91
53	VIRGIN ISLANDS	109,825	457.49	416.56
54	GUAM	178,430	171.28	95.99
**U.S. GRAMS / PER 100K:**		312,825,210	14,691,462.79	4,696.38

CUMULATIVE DISTRIBUTION BY STATE IN GRAMS PER 100,000 POPULATION

DRUG CODE:9300 **DRUG NAME:MORPHINE**

RANK	STATE	2010 POP	TOTAL GRAMS	GRAMS/100K POP
1	TENNESSEE	6,346,105	650,022.95	10,242.86
2	ARIZONA	6,392,017	648,079.96	10,138.90
3	OKLAHOMA	3,751,351	363,748.88	9,696.48
4	DELAWARE	897,934	86,687.52	9,654.11
5	NEVADA	2,700,551	237,514.37	8,795.03
6	FLORIDA	18,801,310	1,573,659.42	8,369.95
7	MICHIGAN	9,883,640	753,570.18	7,624.42
8	OREGON	3,831,074	284,319.06	7,421.39
9	PENNSYLVANIA	12,702,379	932,230.09	7,339.02
10	IDAHO	1,567,582	112,526.83	7,178.37
11	UTAH	2,763,885	196,552.81	7,111.47
12	MONTANA	989,415	69,641.95	7,038.70
13	VERMONT	625,741	40,528.56	6,476.89
14	WASHINGTON	6,724,540	433,678.66	6,449.19
15	MISSOURI	5,988,927	385,933.56	6,444.12
16	ALASKA	710,231	45,732.63	6,439.12
17	HAWAII	1,360,301	87,159	6,407.33
18	WYOMING	563,626	35,867.45	6,363.70
19	KANSAS	2,853,118	177,674.01	6,227.36
20	ARKANSAS	2,915,918	180,890.44	6,203.55
21	COLORADO	5,029,196	305,459.85	6,073.73
22	ALABAMA	4,779,736	282,066.83	5,901.31
23	SOUTH CAROLINA	4,625,364	261,446.04	5,652.44
24	NEW HAMPSHIRE	1,316,470	72,477.48	5,505.44
25	WISCONSIN	5,686,986	308,422.58	5,423.30
26	MISSISSIPPI	2,967,297	159,811.32	5,385.75
27	INDIANA	6,483,802	343,789.89	5,302.29
28	MARYLAND	5,773,552	303,143.35	5,250.55
29	NEW MEXICO	2,059,179	103,153.03	5,009.42
30	NORTH CAROLINA	9,535,483	476,140	4,993.35
31	MAINE	1,328,361	65,933.90	4,963.55
32	CALIFORNIA	37,253,956	1,775,497	4,765.93
33	WEST VIRGINIA	1,852,994	87,446.52	4,719.20
34	LOUISIANA	4,533,372	213,761.16	4,715.28
35	GEORGIA	9,687,653	448,555.06	4,630.17
36	RHODE ISLAND	1,052,567	46,628.51	4,429.98
37	IOWA	3,046,355	133,974.93	4,397.88
38	SOUTH DAKOTA	814,180	34,801.07	4,274.37
39	KENTUCKY	4,339,367	185,442.79	4,273.50
40	NEBRASKA	1,826,341	75,693.09	4,144.52
41	OHIO	11,536,504	477,722.06	4,140.96
42	MASSACHUSETTS	6,547,629	262,198.26	4,004.48
43	CONNECTICUT	3,574,097	141,454.92	3,957.78
44	VIRGINIA	8,001,024	313,868.81	3,922.86
45	NEW JERSEY	8,791,894	340,241.92	3,869.95
46	NORTH DAKOTA	672,591	25,131.85	3,736.57
47	MINNESOTA	5,303,925	185,472.24	3,496.89
48	TEXAS	25,145,561	821,137.44	3,265.54
49	NEW YORK	19,378,102	620,757.85	3,203.40
50	ILLINOIS	12,830,632	409,354.42	3,190.45
51	DISTRICT OF COLUMBIA	601,723	14,575.26	2,422.25
52	GUAM	178,430	3,998.25	2,240.80
53	VIRGIN ISLANDS	109,825	524.89	477.93
54	PUERTO RICO	3,725,789	8,399.74	225.45
55	AMERICAN SAMOA	65,628	87.61	133.49
**U.S. GRAMS / PER 100K:**		312,825,210	16,604,588.25	5,307.94

DRUG CODE:9333 **DRUG NAME:THEBAINE**

RANK	STATE	2010 POP	TOTAL GRAMS	GRAMS/100K POP

1	TEXAS	25,145,561	25	0.1
**U.S. GRAMS / PER 100K:**		312,825,210	25	0.01

DRUG CODE:9630 **DRUG NAME:OPIUM TINCTURE (LAUDANUM)**

RANK	STATE	2010 POP	TOTAL GRAMS	GRAMS/100K POP
1	OHIO	11,536,504	82.68	0.72
2	INDIANA	6,483,802	35.47	0.55
3	NEW HAMPSHIRE	1,316,470	4.73	0.36
4	WEST VIRGINIA	1,852,994	5.9	0.32
5	MICHIGAN	9,883,640	30.72	0.31
6	WISCONSIN	5,686,986	14.18	0.25
7	MARYLAND	5,773,552	12.99	0.22
8	KANSAS	2,853,118	5.91	0.21
9	PENNSYLVANIA	12,702,379	21.24	0.17
10	DELAWARE	897,934	1.18	0.13
11	CONNECTICUT	3,574,097	4.73	0.13
12	VIRGINIA	8,001,024	10.62	0.13
13	ILLINOIS	12,830,632	16.55	0.13
14	FLORIDA	18,801,310	14.19	0.08
15	TEXAS	25,145,561	14.16	0.06
16	OREGON	3,831,074	2.36	0.06
17	KENTUCKY	4,339,367	2.36	0.05
18	WASHINGTON	6,724,540	3.54	0.05
19	CALIFORNIA	37,253,956	5.91	0.02
20	NEW YORK	19,378,102	4.73	0.02
21	NEW JERSEY	8,791,894	1.18	0.01
**U.S. GRAMS / PER 100K:**		312,825,210	295.33	0.09

DRUG CODE:9639 **DRUG NAME:OPIUM POWDERED**

RANK	STATE	2010 POP	TOTAL GRAMS	GRAMS/100K POP
1	MINNESOTA	5,303,925	1,535.76	28.96
2	VERMONT	625,741	162.72	26
3	KANSAS	2,853,118	720	25.24
4	NORTH DAKOTA	672,591	164.88	24.51
5	SOUTH DAKOTA	814,180	180.72	22.2
6	NEBRASKA	1,826,341	393.84	21.56
7	COLORADO	5,029,196	1,060.20	21.08
8	OREGON	3,831,074	798.12	20.83
9	MONTANA	989,415	204.12	20.63
10	WEST VIRGINIA	1,852,994	380.88	20.55
11	WASHINGTON	6,724,540	1,257.48	18.7
12	ARIZONA	6,392,017	1,181.16	18.48
13	WYOMING	563,626	98.28	17.44
14	KENTUCKY	4,339,367	696.6	16.05
15	MICHIGAN	9,883,640	1,542.24	15.6
16	INDIANA	6,483,802	981.72	15.14
17	WISCONSIN	5,686,986	854.64	15.03
18	MAINE	1,328,361	199.08	14.99
19	ALASKA	710,231	102.6	14.45
20	IOWA	3,046,355	402.48	13.21
21	OHIO	11,536,504	1,514.52	13.13
22	RHODE ISLAND	1,052,567	137.52	13.07
23	IDAHO	1,567,582	195.48	12.47
24	ARKANSAS	2,915,918	361.08	12.38
25	MASSACHUSETTS	6,547,629	803.16	12.27
26	SOUTH CAROLINA	4,625,364	555.12	12
27	TENNESSEE	6,346,105	759.96	11.98
28	MISSOURI	5,988,927	674.28	11.26
29	NEW HAMPSHIRE	1,316,470	146.52	11.13
30	FLORIDA	18,801,310	2,031.48	10.8

31	CONNECTICUT	3,574,097	385.2	10.78
32	PENNSYLVANIA	12,702,379	1,300.32	10.24
33	DISTRICT OF COLUMBIA	601,723	59.04	9.81
34	NORTH CAROLINA	9,535,483	917.64	9.62
35	UTAH	2,763,885	260.64	9.43
36	LOUISIANA	4,533,372	421.2	9.29
37	VIRGINIA	8,001,024	722.88	9.03
38	HAWAII	1,360,301	115.2	8.47
39	TEXAS	25,145,561	2,088.72	8.31
40	OKLAHOMA	3,751,351	310.68	8.28
41	GEORGIA	9,687,653	755.64	7.8
42	ALABAMA	4,779,736	341.64	7.15
43	NEW MEXICO	2,059,179	144.72	7.03
44	DELAWARE	897,934	60.12	6.7
45	MARYLAND	5,773,552	335.16	5.81
46	ILLINOIS	12,830,632	717.48	5.59
47	NEW JERSEY	8,791,894	443.16	5.04
48	NEVADA	2,700,551	134.28	4.97
49	CALIFORNIA	37,253,956	1,821.60	4.89
50	MISSISSIPPI	2,967,297	134.64	4.54
51	NEW YORK	19,378,102	869.4	4.49
52	VIRGIN ISLANDS	109,825	1.8	1.64
53	GUAM	178,430	0.36	0.2
**U.S. GRAMS / PER 100K:**		312,825,210	32,438.16	10.37

**DRUG CODE:9652**	**DRUG NAME:OXYMORPHONE**			
**RANK**	**STATE**	**2010 POP**	**TOTAL GRAMS**	**GRAMS/100K POP**
1	TENNESSEE	6,346,105	82,234.18	1,295.82
2	NORTH CAROLINA	9,535,483	82,956.34	869.98
3	DELAWARE	897,934	7,725.64	860.38
4	MICHIGAN	9,883,640	75,695.99	765.87
5	PENNSYLVANIA	12,702,379	91,066.81	716.93
6	MARYLAND	5,773,552	37,050.14	641.72
7	UTAH	2,763,885	16,399.44	593.35
8	NEVADA	2,700,551	15,930.36	589.89
9	LOUISIANA	4,533,372	26,335.19	580.92
10	OKLAHOMA	3,751,351	21,760.84	580.08
11	WEST VIRGINIA	1,852,994	9,923.63	535.55
12	SOUTH CAROLINA	4,625,364	24,002.65	518.94
13	FLORIDA	18,801,310	86,877.35	462.08
14	VIRGINIA	8,001,024	36,312.38	453.85
15	ALABAMA	4,779,736	21,503.81	449.9
16	MISSISSIPPI	2,967,297	12,248.28	412.78
17	ARIZONA	6,392,017	23,750.89	371.57
18	NEW YORK	19,378,102	71,813.88	370.59
19	OHIO	11,536,504	38,549.36	334.15
20	KENTUCKY	4,339,367	14,463.13	333.3
21	IDAHO	1,567,582	5,145.08	328.22
22	INDIANA	6,483,802	19,468.06	300.26
23	CONNECTICUT	3,574,097	10,494.91	293.64
24	NEW JERSEY	8,791,894	25,694.05	292.25
25	MISSOURI	5,988,927	17,228.11	287.67
26	MAINE	1,328,361	3,698.50	278.43
27	GEORGIA	9,687,653	25,820.74	266.53
28	NEW HAMPSHIRE	1,316,470	3,491.59	265.22
29	KANSAS	2,853,118	7,511.04	263.26
30	NEBRASKA	1,826,341	4,767.34	261.03
31	COLORADO	5,029,196	12,822.14	254.95
32	ARKANSAS	2,915,918	6,873.01	235.71
33	SOUTH DAKOTA	814,180	1,912.19	234.86
34	WYOMING	563,626	1,120.03	198.72

35	NEW MEXICO	2,059,179	3,798.04	184.44
36	TEXAS	25,145,561	46,168.26	183.6
37	VERMONT	625,741	1,144.86	182.96
38	OREGON	3,831,074	6,580.72	171.77
39	WASHINGTON	6,724,540	11,405.38	169.61
40	HAWAII	1,360,301	2,146.26	157.78
41	RHODE ISLAND	1,052,567	1,602.01	152.2
42	WISCONSIN	5,686,986	8,511.37	149.66
43	ALASKA	710,231	1,004.73	141.46
44	CALIFORNIA	37,253,956	52,214.98	140.16
45	IOWA	3,046,355	4,107.22	134.82
46	MASSACHUSETTS	6,547,629	8,701.18	132.89
47	ILLINOIS	12,830,632	16,297.56	127.02
48	MONTANA	989,415	1,132.77	114.49
49	NORTH DAKOTA	672,591	629.64	93.61
50	DISTRICT OF COLUMBIA	601,723	479.25	79.65
51	MINNESOTA	5,303,925	1,698.09	32.02
**U.S. GRAMS / PER 100K:**		312,825,210	1,110,269.42	354.92

DRUG CODE:9668 **DRUG NAME:NOROXYMORPHONE**

RANK	STATE	2010 POP	TOTAL GRAMS	GRAMS/100K POP
1	ARKANSAS	2,915,918	78.06	2.68
2	NEVADA	2,700,551	25.26	0.94
3	CALIFORNIA	37,253,956	259.61	0.7
4	PUERTO RICO	3,725,789	25.07	0.67
5	MISSOURI	5,988,927	37.2	0.62
6	TEXAS	25,145,561	108.84	0.43
7	FLORIDA	18,801,310	58.03	0.31
8	ILLINOIS	12,830,632	38.02	0.3
9	NEW JERSEY	8,791,894	23.7	0.27
10	MISSISSIPPI	2,967,297	6.19	0.21
11	OHIO	11,536,504	24.31	0.21
12	GEORGIA	9,687,653	20.46	0.21
13	WASHINGTON	6,724,540	8.47	0.13
14	NEW YORK	19,378,102	22.13	0.11
15	KANSAS	2,853,118	2.75	0.1
16	OREGON	3,831,074	2	0.05
17	MASSACHUSETTS	6,547,629	3.03	0.05
18	MICHIGAN	9,883,640	4.29	0.04
19	NORTH CAROLINA	9,535,483	3.68	0.04
20	OKLAHOMA	3,751,351	0.64	0.02
21	VERMONT	625,741	0.1	0.02
22	RHODE ISLAND	1,052,567	0.1	0.01
23	PENNSYLVANIA	12,702,379	0.99	0.01
24	MINNESOTA	5,303,925	0.5	0.01
25	TENNESSEE	6,346,105	0.13	0
26	ARIZONA	6,392,017	0.1	0
27	MARYLAND	5,773,552	0.2	0
**U.S. GRAMS / PER 100K:**		312,825,210	753.86	0.24

DRUG CODE:9737 **DRUG NAME:ALFENTANIL**

RANK	STATE	2010 POP	TOTAL GRAMS	GRAMS/100K POP
1	OREGON	3,831,074	18.67	0.49
2	ALASKA	710,231	2.89	0.41
3	KENTUCKY	4,339,367	16.43	0.38
4	IDAHO	1,567,582	5.45	0.35
5	WISCONSIN	5,686,986	19.27	0.34
6	NEVADA	2,700,551	6.95	0.26
7	MINNESOTA	5,303,925	13.91	0.26
8	IOWA	3,046,355	6.68	0.22

9	LOUISIANA	4,533,372	10.05	0.22
10	KANSAS	2,853,118	5.98	0.21
11	NEW MEXICO	2,059,179	4.1	0.2
12	MAINE	1,328,361	2.69	0.2
13	NEBRASKA	1,826,341	3.56	0.19
14	WASHINGTON	6,724,540	12.65	0.19
15	HAWAII	1,360,301	2.4	0.18
16	CALIFORNIA	37,253,956	65.92	0.18
17	OHIO	11,536,504	19.09	0.17
18	INDIANA	6,483,802	10.29	0.16
19	CONNECTICUT	3,574,097	5.29	0.15
20	NEW HAMPSHIRE	1,316,470	1.95	0.15
21	GEORGIA	9,687,653	13.05	0.13
22	MONTANA	989,415	1.3	0.13
23	NORTH CAROLINA	9,535,483	12.57	0.13
24	ILLINOIS	12,830,632	16.5	0.13
25	UTAH	2,763,885	3.09	0.11
26	FLORIDA	18,801,310	20.91	0.11
27	OKLAHOMA	3,751,351	3.52	0.09
28	NORTH DAKOTA	672,591	0.59	0.09
29	TEXAS	25,145,561	22.04	0.09
30	TENNESSEE	6,346,105	4.77	0.08
31	COLORADO	5,029,196	3.86	0.08
32	MISSOURI	5,988,927	4.33	0.07
33	ARIZONA	6,392,017	3.73	0.06
34	NEW YORK	19,378,102	9.15	0.05
35	MICHIGAN	9,883,640	4.48	0.05
36	VIRGINIA	8,001,024	3.62	0.05
37	MARYLAND	5,773,552	2.89	0.05
38	MASSACHUSETTS	6,547,629	3.45	0.05
39	SOUTH DAKOTA	814,180	0.33	0.04
40	GUAM	178,430	0.08	0.04
41	PENNSYLVANIA	12,702,379	2.01	0.02
42	RHODE ISLAND	1,052,567	0.26	0.02
43	WYOMING	563,626	0.12	0.02
44	VERMONT	625,741	0.04	0.01
45	SOUTH CAROLINA	4,625,364	0.35	0.01
46	ALABAMA	4,779,736	0.25	0.01
47	MISSISSIPPI	2,967,297	0.43	0.01
48	NEW JERSEY	8,791,894	1.05	0.01
49	ARKANSAS	2,915,918	0.08	0
**U.S. GRAMS / PER 100K:**		312,825,210	372.99	0.12

**DRUG CODE:9739**	**DRUG NAME:REMIFENTANIL**			
**RANK**	**STATE**	**2010 POP**	**TOTAL GRAMS**	**GRAMS/100K POP**
1	UTAH	2,763,885	71.78	2.6
2	ALASKA	710,231	11.68	1.64
3	MASSACHUSETTS	6,547,629	89.26	1.36
4	DELAWARE	897,934	12.04	1.34
5	PENNSYLVANIA	12,702,379	143.24	1.13
6	WEST VIRGINIA	1,852,994	20.75	1.12
7	CONNECTICUT	3,574,097	34.06	0.95
8	NEW YORK	19,378,102	155.04	0.8
9	WASHINGTON	6,724,540	50.01	0.74
10	MONTANA	989,415	6.99	0.71
11	OHIO	11,536,504	75.63	0.66
12	MINNESOTA	5,303,925	32.7	0.62
13	NORTH CAROLINA	9,535,483	57.75	0.61
14	NEW JERSEY	8,791,894	52.91	0.6
15	FLORIDA	18,801,310	107.44	0.57
16	IDAHO	1,567,582	8.69	0.55

17	COLORADO	5,029,196	25.83	0.51
18	NEW HAMPSHIRE	1,316,470	6.52	0.5
19	INDIANA	6,483,802	31.93	0.49
20	DISTRICT OF COLUMBIA	601,723	2.84	0.47
21	MARYLAND	5,773,552	27.4	0.47
22	ILLINOIS	12,830,632	60.76	0.47
23	WISCONSIN	5,686,986	26.73	0.47
24	VERMONT	625,741	2.83	0.45
25	TEXAS	25,145,561	111.86	0.44
26	KENTUCKY	4,339,367	19.16	0.44
27	LOUISIANA	4,533,372	18.57	0.41
28	IOWA	3,046,355	12.58	0.41
29	MICHIGAN	9,883,640	39.63	0.4
30	SOUTH CAROLINA	4,625,364	18.65	0.4
31	HAWAII	1,360,301	5.14	0.38
32	TENNESSEE	6,346,105	23.24	0.37
33	MISSOURI	5,988,927	21.77	0.36
34	CALIFORNIA	37,253,956	133.36	0.36
35	MAINE	1,328,361	4.61	0.35
36	VIRGINIA	8,001,024	27.17	0.34
37	WYOMING	563,626	1.78	0.32
38	KANSAS	2,853,118	9.07	0.32
39	OREGON	3,831,074	11.4	0.3
40	RHODE ISLAND	1,052,567	3.17	0.3
41	OKLAHOMA	3,751,351	10.74	0.29
42	ALABAMA	4,779,736	13.15	0.28
43	ARIZONA	6,392,017	15.69	0.25
44	NORTH DAKOTA	672,591	1.67	0.25
45	SOUTH DAKOTA	814,180	1.89	0.23
46	NEBRASKA	1,826,341	4.29	0.23
47	ARKANSAS	2,915,918	6.4	0.22
48	NEW MEXICO	2,059,179	4.44	0.22
49	GEORGIA	9,687,653	19.21	0.2
50	GUAM	178,430	0.31	0.17
51	NEVADA	2,700,551	3.7	0.14
52	MISSISSIPPI	2,967,297	1.78	0.06
U.S. GRAMS / PER 100K:		312,825,210	1,659.21	0.53

DRUG CODE:9740 **DRUG NAME:SUFENTANIL BASE**

**RANK**	**STATE**	**2010 POP**	**TOTAL GRAMS**	**GRAMS/100K POP**
1	MISSISSIPPI	2,967,297	52.13	1.76
2	ALABAMA	4,779,736	6.56	0.14
3	CALIFORNIA	37,253,956	43.74	0.12
4	LOUISIANA	4,533,372	4.63	0.1
5	CONNECTICUT	3,574,097	2.39	0.07
6	FLORIDA	18,801,310	12.66	0.07
7	PENNSYLVANIA	12,702,379	8.12	0.06
8	SOUTH CAROLINA	4,625,364	2.64	0.06
9	WISCONSIN	5,686,986	2.53	0.04
10	NEW JERSEY	8,791,894	3.74	0.04
11	TENNESSEE	6,346,105	2.57	0.04
12	KANSAS	2,853,118	1.16	0.04
13	TEXAS	25,145,561	6.66	0.03
14	KENTUCKY	4,339,367	1.51	0.03
15	NEW YORK	19,378,102	6.74	0.03
16	UTAH	2,763,885	0.92	0.03
17	OREGON	3,831,074	1.23	0.03
18	MICHIGAN	9,883,640	2.25	0.02
19	NEBRASKA	1,826,341	0.4	0.02
20	NORTH DAKOTA	672,591	0.13	0.02
21	WASHINGTON	6,724,540	1.04	0.02

22	OKLAHOMA	3,751,351	0.69	0.02
23	MONTANA	989,415	0.17	0.02
24	PUERTO RICO	3,725,789	0.75	0.02
25	GEORGIA	9,687,653	1.79	0.02
26	MISSOURI	5,988,927	1.26	0.02
27	ILLINOIS	12,830,632	2.29	0.02
28	OHIO	11,536,504	1.95	0.02
29	VIRGINIA	8,001,024	1.54	0.02
30	COLORADO	5,029,196	0.84	0.02
31	INDIANA	6,483,802	1.14	0.02
32	NORTH CAROLINA	9,535,483	1.67	0.02
33	SOUTH DAKOTA	814,180	0.18	0.02
34	ARKANSAS	2,915,918	0.56	0.02
35	MASSACHUSETTS	6,547,629	0.52	0.01
36	IOWA	3,046,355	0.21	0.01
37	WEST VIRGINIA	1,852,994	0.26	0.01
38	ARIZONA	6,392,017	0.34	0.01
39	MINNESOTA	5,303,925	0.77	0.01
40	IDAHO	1,567,582	0.18	0.01
41	NEVADA	2,700,551	0.37	0.01
42	MAINE	1,328,361	0.08	0.01
43	ALASKA	710,231	0.04	0.01
44	NEW MEXICO	2,059,179	0.19	0.01
45	WYOMING	563,626	0.07	0.01
46	RHODE ISLAND	1,052,567	0.01	0
47	HAWAII	1,360,301	0.03	0
48	NEW HAMPSHIRE	1,316,470	0.06	0
49	DISTRICT OF COLUMBIA	601,723	0.03	0
50	DELAWARE	897,934	0.03	0
51	VERMONT	625,741	0.03	0
52	MARYLAND	5,773,552	0.22	0
**U.S. GRAMS / PER 100K:**		312,825,210	182.02	0.06

**DRUG CODE:9780**	**DRUG NAME:TAPENTADOL**			
**RANK**	**STATE**	**2010 POP**	**TOTAL GRAMS**	**GRAMS/100K POP**
1	ALABAMA	4,779,736	218,561	4,572.66
2	NORTH CAROLINA	9,535,483	386,527.50	4,053.57
3	SOUTH CAROLINA	4,625,364	174,010	3,762.08
4	DELAWARE	897,934	29,674	3,304.70
5	ARKANSAS	2,915,918	83,934.50	2,878.49
6	GEORGIA	9,687,653	269,630.50	2,783.24
7	OKLAHOMA	3,751,351	94,917.50	2,530.22
8	SOUTH DAKOTA	814,180	18,860	2,316.44
9	INDIANA	6,483,802	149,431.50	2,304.69
10	MARYLAND	5,773,552	130,004.50	2,251.72
11	NEW HAMPSHIRE	1,316,470	29,415.50	2,234.42
12	COLORADO	5,029,196	112,252.50	2,232.02
13	ARIZONA	6,392,017	142,123	2,223.45
14	NEW JERSEY	8,791,894	189,226	2,152.28
15	NEBRASKA	1,826,341	38,820	2,125.56
16	FLORIDA	18,801,310	396,257	2,107.60
17	OHIO	11,536,504	242,759	2,104.27
18	PENNSYLVANIA	12,702,379	264,471	2,082.06
19	VIRGINIA	8,001,024	165,567.50	2,069.33
20	TENNESSEE	6,346,105	127,215	2,004.62
21	UTAH	2,763,885	54,890.50	1,985.99
22	NEW YORK	19,378,102	383,884.50	1,981.02
23	NEVADA	2,700,551	48,805.50	1,807.24
24	IOWA	3,046,355	49,701	1,631.49
25	MISSOURI	5,988,927	97,566	1,629.11
26	MISSISSIPPI	2,967,297	48,285.50	1,627.26

27	TEXAS	25,145,561	390,103	1,551.38
28	IDAHO	1,567,582	24,126.50	1,539.09
29	NORTH DAKOTA	672,591	9,775.50	1,453.41
30	KENTUCKY	4,339,367	60,134	1,385.78
31	WYOMING	563,626	7,766	1,377.86
32	LOUISIANA	4,533,372	60,790.50	1,340.96
33	WEST VIRGINIA	1,852,994	24,544.50	1,324.59
34	CONNECTICUT	3,574,097	46,049	1,288.41
35	ALASKA	710,231	8,991	1,265.93
36	KANSAS	2,853,118	35,042	1,228.20
37	ILLINOIS	12,830,632	151,698.50	1,182.32
38	MASSACHUSETTS	6,547,629	77,076	1,177.16
39	CALIFORNIA	37,253,956	424,876.85	1,140.49
40	WASHINGTON	6,724,540	70,350.50	1,046.18
41	WISCONSIN	5,686,986	59,475	1,045.81
42	HAWAII	1,360,301	12,594	925.82
43	MICHIGAN	9,883,640	86,639	876.59
44	RHODE ISLAND	1,052,567	8,390	797.1
45	OREGON	3,831,074	29,815	778.24
46	MONTANA	989,415	7,434.50	751.4
47	VERMONT	625,741	4,620	738.32
48	DISTRICT OF COLUMBIA	601,723	4,096.50	680.79
49	NEW MEXICO	2,059,179	12,826.50	622.89
50	MAINE	1,328,361	5,635.50	424.24
51	MINNESOTA	5,303,925	17,601.50	331.86
52	PUERTO RICO	3,725,789	3,374	90.56
53	VIRGIN ISLANDS	109,825	18	16.39
**U.S. GRAMS / PER 100K:**		312,825,210	5,590,633.85	1,787.14

DRUG CODE:9801 **DRUG NAME:FENTANYL BASE**

**RANK**	**STATE**	**2010 POP**	**TOTAL GRAMS**	**GRAMS/100K POP**
1	OKLAHOMA	3,751,351	6,755.14	180.07
2	MISSISSIPPI	2,967,297	4,922.64	165.9
3	PENNSYLVANIA	12,702,379	20,066.53	157.97
4	KANSAS	2,853,118	4,461.96	156.39
5	MISSOURI	5,988,927	9,085.08	151.7
6	DELAWARE	897,934	1,360.60	151.53
7	SOUTH DAKOTA	814,180	1,204.24	147.91
8	IDAHO	1,567,582	2,304.90	147.04
9	NORTH DAKOTA	672,591	947.88	140.93
10	MONTANA	989,415	1,318.09	133.22
11	ALASKA	710,231	940.62	132.44
12	ARKANSAS	2,915,918	3,715.20	127.41
13	MICHIGAN	9,883,640	12,375.98	125.22
14	ALABAMA	4,779,736	5,963.13	124.76
15	FLORIDA	18,801,310	23,352.70	124.21
16	TENNESSEE	6,346,105	7,689.41	121.17
17	VERMONT	625,741	748.07	119.55
18	UTAH	2,763,885	3,275.55	118.51
19	NORTH CAROLINA	9,535,483	11,266.22	118.15
20	WEST VIRGINIA	1,852,994	2,167.50	116.97
21	NEBRASKA	1,826,341	2,129.02	116.57
22	INDIANA	6,483,802	7,523.57	116.04
23	IOWA	3,046,355	3,476.55	114.12
24	SOUTH CAROLINA	4,625,364	5,261.53	113.75
25	GEORGIA	9,687,653	10,907.88	112.6
26	WYOMING	563,626	620.98	110.18
27	ARIZONA	6,392,017	6,993.38	109.41
28	CONNECTICUT	3,574,097	3,800.61	106.34
29	NEW HAMPSHIRE	1,316,470	1,391.09	105.67
30	KENTUCKY	4,339,367	4,567	105.25

31	LOUISIANA	4,533,372	4,755.62	104.9
32	NEW JERSEY	8,791,894	9,178.74	104.4
33	WISCONSIN	5,686,986	5,898.14	103.71
34	COLORADO	5,029,196	5,010.59	99.63
35	MAINE	1,328,361	1,322.82	99.58
36	OREGON	3,831,074	3,705.65	96.73
37	MARYLAND	5,773,552	5,552.39	96.17
38	ILLINOIS	12,830,632	11,809.15	92.04
39	TEXAS	25,145,561	23,026.12	91.57
40	WASHINGTON	6,724,540	6,032.40	89.71
41	NEW YORK	19,378,102	17,237.37	88.95
42	NEVADA	2,700,551	2,353.92	87.16
43	VIRGINIA	8,001,024	6,913.51	86.41
44	OHIO	11,536,504	9,850.21	85.38
45	NEW MEXICO	2,059,179	1,735.05	84.26
46	MASSACHUSETTS	6,547,629	5,229.02	79.86
47	MINNESOTA	5,303,925	4,115.67	77.6
48	CALIFORNIA	37,253,956	27,815.29	74.66
49	RHODE ISLAND	1,052,567	734.98	69.83
50	DISTRICT OF COLUMBIA	601,723	338.77	56.3
51	HAWAII	1,360,301	742.32	54.57
52	GUAM	178,430	41.33	23.16
53	PUERTO RICO	3,725,789	578.26	15.52
54	VIRGIN ISLANDS	109,825	13.96	12.71
55	AMERICAN SAMOA	65,628	0.47	0.71
**U.S. GRAMS / PER 100K:**		312,825,210	324,584.80	103.76

DRUG CODE:9809 **DRUG NAME:OPIUM COMBINATION PRODUCT (C-III)**

**RANK**	**STATE**	**2010 POP**	**TOTAL GRAMS**	**GRAMS/100K POP**
1	UTAH	2,763,885	49.57	1.79
2	PENNSYLVANIA	12,702,379	135.65	1.07
3	ALABAMA	4,779,736	47.11	0.99
4	IDAHO	1,567,582	15.14	0.97
5	SOUTH CAROLINA	4,625,364	40.11	0.87
6	KENTUCKY	4,339,367	37.07	0.85
7	LOUISIANA	4,533,372	33.3	0.73
8	FLORIDA	18,801,310	137.74	0.73
9	NEW HAMPSHIRE	1,316,470	8.89	0.68
10	MARYLAND	5,773,552	34.06	0.59
11	NORTH CAROLINA	9,535,483	53.92	0.57
12	CONNECTICUT	3,574,097	19.49	0.55
13	WYOMING	563,626	3.03	0.54
14	ARIZONA	6,392,017	33.3	0.52
15	WEST VIRGINIA	1,852,994	9.65	0.52
16	MAINE	1,328,361	6.81	0.51
17	OHIO	11,536,504	58.25	0.5
18	MISSOURI	5,988,927	28.57	0.48
19	INDIANA	6,483,802	29.14	0.45
20	WISCONSIN	5,686,986	23.08	0.41
21	MISSISSIPPI	2,967,297	11.73	0.4
22	NORTH DAKOTA	672,591	2.65	0.39
23	TENNESSEE	6,346,105	24.97	0.39
24	HAWAII	1,360,301	4.73	0.35
25	MONTANA	989,415	3.41	0.34
26	OKLAHOMA	3,751,351	12.87	0.34
27	SOUTH DAKOTA	814,180	2.65	0.33
28	NEW MEXICO	2,059,179	6.62	0.32
29	GEORGIA	9,687,653	29.52	0.3
30	VIRGINIA	8,001,024	23.84	0.3
31	ARKANSAS	2,915,918	8.51	0.29
32	IOWA	3,046,355	8.89	0.29

CUMULATIVE DISTRIBUTION BY STATE IN GRAMS PER 100,000 POPULATION

33	DISTRICT OF COLUMBIA	601,723	1.51	0.25
34	NEW JERSEY	8,791,894	20.81	0.24
35	MICHIGAN	9,883,640	22.7	0.23
36	DELAWARE	897,934	2.08	0.23
37	COLORADO	5,029,196	10.97	0.22
38	TEXAS	25,145,561	52.97	0.21
39	CALIFORNIA	37,253,956	76.63	0.21
40	NEW YORK	19,378,102	38.03	0.2
41	ILLINOIS	12,830,632	23.84	0.19
42	NEBRASKA	1,826,341	3.03	0.17
43	MINNESOTA	5,303,925	9.08	0.17
44	NEVADA	2,700,551	4.16	0.15
45	WASHINGTON	6,724,540	10.4	0.15
46	VERMONT	625,741	0.95	0.15
47	MASSACHUSETTS	6,547,629	6.81	0.1
48	KANSAS	2,853,118	2.84	0.1
49	OREGON	3,831,074	3.59	0.09
50	RHODE ISLAND	1,052,567	0.38	0.04
51	ALASKA	710,231	0.19	0.03
**U.S. GRAMS / PER 100K:**		312,825,210	1,235.23	0.39

STATE:ALABAMA BUSINESS ACTIVITY:A - PHARMACIES

DRUG NAME	DRUG CODE	BUYERS	TOTAL GRAMS	AVG GRAMS
AMPHETAMINE	1100	1,371	513,195.41	374.32
DL-METHAMPHETAMINE RACEMIC BASE	1105B	32	96.04	3
D-METHAMPHETAMINE	1105D	4	14.86	3.72
LISDEXAMFETAMINE	1205	1,328	282,505.33	212.73
METHYLPHENIDATE (DL;D;L;ISOMERS)	1724	1,352	283,489.85	209.68
BARBITURIC ACID DERIVIATIVE OR SALT [PER 21C	2100	292	6,130	20.99
BUTALBITAL	2165	610	17,770	29.13
DRONABINOL IN AN ORAL SOLUTION IN FDA APPR	7365	9	4.2	0.47
CODEINE	9050	1,371	201,385.95	146.89
BUPRENORPHINE	9064	1,340	99,420.27	74.19
DIHYDROCODEINE	9120	519	3,017.55	5.81
OXYCODONE	9143	1,379	888,450.18	644.27
HYDROMORPHONE	9150	1,137	9,924.86	8.73
HYDROCODONE	9193	1,394	924,556.65	663.24
LEVORPHANOL	9220L	23	20.08	0.87
MEPERIDINE (PETHIDINE)	9230	832	17,119.96	20.58
METHADONE	9250B	1,145	72,727.60	63.52
MORPHINE	9300	1,341	255,863.42	190.8
OPIUM POWDERED	9639	58	82.44	1.42
OXYMORPHONE	9652	874	21,416.15	24.5
TAPENTADOL	9780	1,134	217,710.50	191.98
FENTANYL BASE	9801	1,308	5,465.95	4.18
OPIUM COMBINATION PRODUCT (C-III)	9809	55	43.71	0.79

STATE:ALABAMA BUSINESS ACTIVITY:B - HOSPITALS

DRUG NAME	DRUG CODE	BUYERS	TOTAL GRAMS	AVG GRAMS
AMPHETAMINE	1100	62	8,827.26	142.38
LISDEXAMFETAMINE	1205	28	2,801.47	100.05
METHYLPHENIDATE (DL;D;L;ISOMERS)	1724	77	7,301.88	94.83
BARBITURIC ACID DERIVIATIVE OR SALT [PER 21C	2100	1	15	15
BUTALBITAL	2165	4	30	7.5
PENTOBARBITAL (SCHEDULE 2)	2270	3	290.28	96.76

COCAINE	9041L	41	137.42	3.35
CODEINE	9050	116	5,082.31	43.81
BUPRENORPHINE	9064	95	1,009.87	10.63
DIHYDROCODEINE	9120	3	4.27	1.42
OXYCODONE	9143	139	33,735.34	242.7
HYDROMORPHONE	9150	130	2,910.62	22.39
HYDROCODONE	9193	144	45,670.59	317.16
MEPERIDINE (PETHIDINE)	9230	119	10,291.54	86.48
METHADONE	9250B	101	3,654.64	36.18
MORPHINE	9300	144	25,226.60	175.18
OPIUM POWDERED	9639	51	254.88	5
OXYMORPHONE	9652	18	86.13	4.79
ALFENTANIL	9737	3	0.25	0.08
REMIFENTANIL	9739	18	11.41	0.63
SUFENTANIL BASE	9740	31	1.55	0.05
TAPENTADOL	9780	31	850.5	27.44
FENTANYL BASE	9801	133	457.5	3.44
OPIUM COMBINATION PRODUCT (C-III)	9809	4	3.41	0.85

STATE:ALABAMA BUSINESS ACTIVITY:C - PRACTITIONERS

DRUG NAME	DRUG CODE	BUYERS	TOTAL GRAMS	AVG GRAMS
LISDEXAMFETAMINE	1205	1	18.22	18.22
METHYLPHENIDATE (DL;D;L;ISOMERS)	1724	1	77.56	77.56
PENTOBARBITAL (SCHEDULE 2)	2270	72	42,999.03	597.21
COCAINE	9041L	8	91.28	11.41
CODEINE	9050	220	2,389.75	10.86
BUPRENORPHINE	9064	257	73.53	0.29
DIHYDROCODEINE	9120	120	124.33	1.04
OXYCODONE	9143	39	1,055.64	27.07
HYDROMORPHONE	9150	119	201.85	1.7
HYDROCODONE	9193	132	1,668.70	12.64
MEPERIDINE (PETHIDINE)	9230	114	2,003.69	17.58
METHADONE	9250B	3	4.13	1.38
MORPHINE	9300	142	976.81	6.88
OPIUM POWDERED	9639	2	4.32	2.16

OXYMORPHONE	9652	1	1.52	1.52
REMIFENTANIL	9739	1	1.74	1.74
SUFENTANIL BASE	9740	7	5.01	0.72
FENTANYL BASE	9801	231	39.68	0.17

STATE:ALABAMA BUSINESS ACTIVITY:D - TEACHING INSTITUTIONS

DRUG NAME	DRUG CODE	BUYERS	TOTAL GRAMS	AVG GRAMS
BUPRENORPHINE	9064	1	0.01	0.01

STATE:ALABAMA BUSINESS ACTIVITY:M - MID-LEVEL PRACTITIONERS

DRUG NAME	DRUG CODE	BUYERS	TOTAL GRAMS	AVG GRAMS
PENTOBARBITAL (SCHEDULE 2)	2270	21	22,304.24	1,062.11
CODEINE	9050	14	33.64	2.4
BUPRENORPHINE	9064	1	0.12	0.12
DIHYDROCODEINE	9120	10	3.2	0.32

STATE:ALABAMA BUSINESS ACTIVITY:N-U NARCOTIC TREATMENT PROGRAMS

DRUG NAME	DRUG CODE	BUYERS	TOTAL GRAMS	AVG GRAMS
BUPRENORPHINE	9064	8	251.28	31.41
METHADONE	9250B	22	236,035.74	10,728.90

STATE:ALASKA BUSINESS ACTIVITY:A - PHARMACIES

DRUG NAME	DRUG CODE	BUYERS	TOTAL GRAMS	AVG GRAMS
AMPHETAMINE	1100	107	25,907.13	242.12
DL-METHAMPHETAMINE RACEMIC BASE	1105B	5	10.85	2.17
D-METHAMPHETAMINE	1105D	1	0.8	0.8
LISDEXAMFETAMINE	1205	96	8,931.65	93.04
METHYLPHENIDATE (DL;D;L;ISOMERS)	1724	106	24,505.89	231.19
BARBITURIC ACID DERIVIATIVE OR SALT [PER 21C	2100	26	715	27.5

BUTALBITAL	2165	36	910	25.28
COCAINE	9041L	1	0.29	0.29
CODEINE	9050	110	19,955.10	181.41
BUPRENORPHINE	9064	104	8,772.51	84.35
DIHYDROCODEINE	9120	1	13.89	13.89
OXYCODONE	9143	111	123,965.53	1,116.81
HYDROMORPHONE	9150	95	3,237.94	34.08
HYDROCODONE	9193	110	39,318.08	357.44
MEPERIDINE (PETHIDINE)	9230	58	2,378.32	41.01
METHADONE	9250B	96	15,470.26	161.15
MORPHINE	9300	109	38,003.31	348.65
OPIUM POWDERED	9639	12	21.96	1.83
OXYMORPHONE	9652	34	991.17	29.15
ALFENTANIL	9737	3	0.14	0.05
REMIFENTANIL	9739	4	0.22	0.06
TAPENTADOL	9780	58	8,259	142.4
FENTANYL BASE	9801	106	750.65	7.08
OPIUM COMBINATION PRODUCT (C-III)	9809	1	0.19	0.19

STATE:ALASKA BUSINESS ACTIVITY:B - HOSPITALS

DRUG NAME	DRUG CODE	BUYERS	TOTAL GRAMS	AVG GRAMS
AMPHETAMINE	1100	27	6,428.14	238.08
LISDEXAMFETAMINE	1205	17	934.21	54.95
METHYLPHENIDATE (DL;D;L;ISOMERS)	1724	31	7,633.06	246.23
BUTALBITAL	2165	1	15	15
PENTOBARBITAL (SCHEDULE 2)	2270	3	2,086.65	695.55
COCAINE	9041L	11	38.07	3.46
CODEINE	9050	38	7,751.98	204
BUPRENORPHINE	9064	33	654.25	19.83
OXYCODONE	9143	47	13,725.79	292.04
HYDROMORPHONE	9150	45	622.59	13.84
HYDROCODONE	9193	50	5,041.10	100.82
MEPERIDINE (PETHIDINE)	9230	37	431.83	11.67
METHADONE	9250B	25	1,254.66	50.19
MORPHINE	9300	51	7,650.67	150.01

OPIUM POWDERED	9639	17	80.28	4.72
OXYMORPHONE	9652	2	5	2.5
ALFENTANIL	9737	4	2.45	0.61
REMIFENTANIL	9739	18	11.03	0.61
SUFENTANIL BASE	9740	6	0.04	0.01
TAPENTADOL	9780	6	732	122
FENTANYL BASE	9801	51	178.82	3.51

STATE:ALASKA BUSINESS ACTIVITY:C - PRACTITIONERS

DRUG NAME	DRUG CODE	BUYERS	TOTAL GRAMS	AVG GRAMS
PENTOBARBITAL (SCHEDULE 2)	2270	32	19,282.95	602.59
COCAINE	9041L	2	9.57	4.79
CODEINE	9050	10	73.82	7.38
BUPRENORPHINE	9064	51	5.01	0.1
OXYCODONE	9143	8	104.31	13.04
HYDROMORPHONE	9150	53	36.69	0.69
HYDROCODONE	9193	43	164.86	3.83
MEPERIDINE (PETHIDINE)	9230	15	40.85	2.72
METHADONE	9250B	3	14.13	4.71
MORPHINE	9300	35	77.71	2.22
OPIUM POWDERED	9639	1	0.36	0.36
OXYMORPHONE	9652	1	8.56	8.56
ALFENTANIL	9737	1	0.3	0.3
REMIFENTANIL	9739	6	0.43	0.07
FENTANYL BASE	9801	72	11.14	0.15

STATE:ALASKA BUSINESS ACTIVITY:M - MID-LEVEL PRACTITIONERS

DRUG NAME	DRUG CODE	BUYERS	TOTAL GRAMS	AVG GRAMS
PENTOBARBITAL (SCHEDULE 2)	2270	4	1,421.78	355.45
CODEINE	9050	3	16.58	5.53
HYDROMORPHONE	9150	1	0.02	0.02
HYDROCODONE	9193	5	6.05	1.21
MORPHINE	9300	5	0.94	0.19

FENTANYL BASE	9801	3	0.01	0

STATE:ALASKA BUSINESS ACTIVITY:N-U NARCOTIC TREATMENT PROGRAMS

DRUG NAME	DRUG CODE	BUYERS	TOTAL GRAMS	AVG GRAMS
BUPRENORPHINE	9064	2	26.4	13.2
METHADONE	9250B	4	15,347.90	3,836.98

STATE:AMERICAN SAMOA BUSINESS ACTIVITY:B - HOSPITALS

DRUG NAME	DRUG CODE	BUYERS	TOTAL GRAMS	AVG GRAMS
AMPHETAMINE	1100	1	2.5	2.5
METHYLPHENIDATE (DL;D;L;ISOMERS)	1724	1	17.73	17.73
BUTALBITAL	2165	1	10	10
CODEINE	9050	1	1,361.42	1,361.42
OXYCODONE	9143	1	58.72	58.72
HYDROMORPHONE	9150	1	0.98	0.98
HYDROCODONE	9193	1	7.9	7.9
MORPHINE	9300	1	87.61	87.61
FENTANYL BASE	9801	1	0.47	0.47

STATE:AMERICAN SAMOA BUSINESS ACTIVITY:C - PRACTITIONERS

DRUG NAME	DRUG CODE	BUYERS	TOTAL GRAMS	AVG GRAMS
BUPRENORPHINE	9064	1	0.01	0.01

STATE:ARIZONA BUSINESS ACTIVITY:A - PHARMACIES

DRUG NAME	DRUG CODE	BUYERS	TOTAL GRAMS	AVG GRAMS
AMPHETAMINE	1100	1,112	319,806.38	287.6
DL-METHAMPHETAMINE RACEMIC BASE	1105B	28	134.22	4.79
D-METHAMPHETAMINE	1105D	5	19.68	3.94
LISDEXAMFETAMINE	1205	1,052	141,762.84	134.76

METHYLPHENIDATE (DL;D;L;ISOMERS)	1724	1,097	282,504.72	257.52
BARBITURIC ACID DERIVIATIVE OR SALT [PER 21C	2100	511	20,245	39.62
BUTALBITAL	2165	486	20,450	42.08
SECOBARBITAL (SCHEDULE 2)	2315	2	18.31	9.16
DRONABINOL IN AN ORAL SOLUTION IN FDA APPR	7365	4	21.6	5.4
NABILONE	7379	1	0.2	0.2
COCAINE	9041L	2	4.61	2.3
CODEINE	9050	1,112	270,203.88	242.99
BUPRENORPHINE	9064	1,059	34,656.01	32.73
DIHYDROCODEINE	9120	25	316.18	12.65
OXYCODONE	9143	1,133	1,631,548.23	1,440.02
HYDROMORPHONE	9150	1,042	23,513.97	22.57
HYDROCODONE	9193	1,139	390,210.36	342.59
LEVORPHANOL	9220L	52	99.94	1.92
MEPERIDINE (PETHIDINE)	9230	520	11,101.71	21.35
METHADONE	9250B	943	50,743.96	53.81
MORPHINE	9300	1,121	594,891.33	530.68
OPIUM POWDERED	9639	110	597.24	5.43
OXYMORPHONE	9652	882	23,704.75	26.88
REMIFENTANIL	9739	1	0.03	0.03
TAPENTADOL	9780	837	141,441.50	168.99
FENTANYL BASE	9801	1,056	6,367.25	6.03
OPIUM COMBINATION PRODUCT (C-III)	9809	26	31.22	1.2

STATE:ARIZONA BUSINESS ACTIVITY:B - HOSPITALS

DRUG NAME	DRUG CODE	BUYERS	TOTAL GRAMS	AVG GRAMS
AMPHETAMINE	1100	96	7,763.57	80.87
LISDEXAMFETAMINE	1205	37	1,617.52	43.72
METHYLPHENIDATE (DL;D;L;ISOMERS)	1724	110	15,615.60	141.96
BARBITURIC ACID DERIVIATIVE OR SALT [PER 21C	2100	1	5	5
AMOBARBITAL (SCHEDULE 2)	2125	5	45.11	9.02
BUTALBITAL	2165	6	85	14.17
PENTOBARBITAL (SCHEDULE 2)	2270	17	1,262.74	74.28
COCAINE	9041L	62	293.98	4.74
CODEINE	9050	129	22,187.70	172

BUPRENORPHINE	9064	68	2,541.31	37.37
OXYCODONE	9143	160	68,431.55	427.7
HYDROMORPHONE	9150	126	3,079.61	24.44
HYDROCODONE	9193	148	21,067.88	142.35
MEPERIDINE (PETHIDINE)	9230	90	5,314.95	59.06
METHADONE	9250B	108	4,880.90	45.19
MORPHINE	9300	155	52,777.89	340.5
OPIUM POWDERED	9639	57	569.16	9.99
OXYMORPHONE	9652	11	44.07	4.01
ALFENTANIL	9737	9	1.65	0.18
REMIFENTANIL	9739	35	13.65	0.39
SUFENTANIL BASE	9740	33	0.31	0.01
TAPENTADOL	9780	13	681.5	52.42
FENTANYL BASE	9801	138	566.69	4.11
OPIUM COMBINATION PRODUCT (C-III)	9809	3	2.08	0.69

STATE:ARIZONA BUSINESS ACTIVITY:C - PRACTITIONERS

DRUG NAME	DRUG CODE	BUYERS	TOTAL GRAMS	AVG GRAMS
PENTOBARBITAL (SCHEDULE 2)	2270	194	213,625.71	1,101.16
COCAINE	9041L	28	120.99	4.32
CODEINE	9050	154	4,483.84	29.12
BUPRENORPHINE	9064	591	84.31	0.14
DIHYDROCODEINE	9120	5	1.6	0.32
OXYCODONE	9143	102	421.55	4.13
HYDROMORPHONE	9150	441	217.96	0.49
HYDROCODONE	9193	320	1,481.80	4.63
MEPERIDINE (PETHIDINE)	9230	284	3,088.94	10.88
METHADONE	9250B	15	69.67	4.64
MORPHINE	9300	288	410.53	1.43
OPIUM POWDERED	9639	8	14.76	1.85
OXYMORPHONE	9652	13	2.07	0.16
NOROXYMORPHONE	9668	1	0.1	0.1
ALFENTANIL	9737	20	2.09	0.1
REMIFENTANIL	9739	27	2.01	0.07
SUFENTANIL BASE	9740	9	0.03	0

FENTANYL BASE	9801	712	59.43	0.08

STATE:ARIZONA BUSINESS ACTIVITY:M - MID-LEVEL PRACTITIONERS

DRUG NAME	DRUG CODE	BUYERS	TOTAL GRAMS	AVG GRAMS
PENTOBARBITAL (SCHEDULE 2)	2270	1	177.72	177.72
CODEINE	9050	73	273.12	3.74
BUPRENORPHINE	9064	1	0.48	0.48
DIHYDROCODEINE	9120	5	1.71	0.34
OXYCODONE	9143	1	0.45	0.45
HYDROMORPHONE	9150	1	0.04	0.04
HYDROCODONE	9193	2	43.57	21.79
MORPHINE	9300	2	0.22	0.11
FENTANYL BASE	9801	1	0	0

STATE:ARIZONA BUSINESS ACTIVITY:N-U NARCOTIC TREATMENT PROGRAMS

DRUG NAME	DRUG CODE	BUYERS	TOTAL GRAMS	AVG GRAMS
BUPRENORPHINE	9064	12	418.16	34.85
METHADONE	9250B	40	280,038.88	7,000.97

STATE:ARKANSAS BUSINESS ACTIVITY:A - PHARMACIES

DRUG NAME	DRUG CODE	BUYERS	TOTAL GRAMS	AVG GRAMS
AMPHETAMINE	1100	787	200,355.92	254.58
DL-METHAMPHETAMINE RACEMIC BASE	1105B	10	25.32	2.53
LISDEXAMFETAMINE	1205	777	120,313.02	154.84
METHYLPHENIDATE (DL;D;L;ISOMERS)	1724	784	156,082.16	199.08
BARBITURIC ACID DERIVIATIVE OR SALT [PER 21C	2100	194	5,320	27.42
BUTALBITAL	2165	355	13,525	38.1
DRONABINOL IN AN ORAL SOLUTION IN FDA APPR	7365	3	1.2	0.4
COCAINE	9041L	1	4.46	4.46
CODEINE	9050	789	287,273.84	364.1
BUPRENORPHINE	9064	737	24,053.25	32.64

DIHYDROCODEINE	9120	128	527.67	4.12
OXYCODONE	9143	795	551,165.83	693.29
HYDROMORPHONE	9150	746	11,018.03	14.77
HYDROCODONE	9193	799	508,544.91	636.48
LEVORPHANOL	9220L	10	9.05	0.91
MEPERIDINE (PETHIDINE)	9230	655	23,073.51	35.23
METHADONE	9250B	713	32,064.87	44.97
MORPHINE	9300	799	163,540.84	204.68
OPIUM POWDERED	9639	26	59.04	2.27
OXYMORPHONE	9652	471	6,819.57	14.48
REMIFENTANIL	9739	1	0.18	0.18
TAPENTADOL	9780	649	82,731	127.47
FENTANYL BASE	9801	772	3,277.14	4.24
OPIUM COMBINATION PRODUCT (C-III)	9809	8	8.14	1.02

STATE:ARKANSAS BUSINESS ACTIVITY:B - HOSPITALS

DRUG NAME	DRUG CODE	BUYERS	TOTAL GRAMS	AVG GRAMS
AMPHETAMINE	1100	50	4,015.79	80.32
LISDEXAMFETAMINE	1205	26	877.56	33.75
METHYLPHENIDATE (DL;D;L;ISOMERS)	1724	70	4,015.23	57.36
BARBITURIC ACID DERIVIATIVE OR SALT [PER 21C	2100	1	5	5
AMOBARBITAL (SCHEDULE 2)	2125	1	0.91	0.91
BUTALBITAL	2165	5	240	48
PENTOBARBITAL (SCHEDULE 2)	2270	7	262.48	37.5
COCAINE	9041L	30	146.99	4.9
CODEINE	9050	91	4,979.88	54.72
BUPRENORPHINE	9064	43	970.56	22.57
DIHYDROCODEINE	9120	1	1.07	1.07
OXYCODONE	9143	122	26,792.67	219.61
HYDROMORPHONE	9150	106	2,012.45	18.99
HYDROCODONE	9193	124	26,950.06	217.34
MEPERIDINE (PETHIDINE)	9230	101	5,933.72	58.75
METHADONE	9250B	76	2,933.50	38.6
MORPHINE	9300	119	17,241.88	144.89
OPIUM POWDERED	9639	37	298.08	8.06

OXYMORPHONE	9652	8	53.44	6.68
ALFENTANIL	9737	2	0.08	0.04
REMIFENTANIL	9739	13	6.08	0.47
SUFENTANIL BASE	9740	21	0.56	0.03
TAPENTADOL	9780	28	1,203.50	42.98
FENTANYL BASE	9801	127	424.22	3.34
OPIUM COMBINATION PRODUCT (C-III)	9809	1	0.38	0.38

STATE:ARKANSAS BUSINESS ACTIVITY:C - PRACTITIONERS

DRUG NAME	DRUG CODE	BUYERS	TOTAL GRAMS	AVG GRAMS
LISDEXAMFETAMINE	1205	1	13.04	13.04
METHYLPHENIDATE (DL;D;L;ISOMERS)	1724	1	3.46	3.46
PENTOBARBITAL (SCHEDULE 2)	2270	47	24,167.03	514.19
COCAINE	9041L	3	24.14	8.05
CODEINE	9050	40	541.65	13.54
BUPRENORPHINE	9064	133	6.65	0.05
DIHYDROCODEINE	9120	25	18.17	0.73
OXYCODONE	9143	6	29.14	4.86
HYDROMORPHONE	9150	58	24.14	0.42
HYDROCODONE	9193	74	182.1	2.46
MEPERIDINE (PETHIDINE)	9230	43	592.18	13.77
MORPHINE	9300	86	107.72	1.25
OPIUM POWDERED	9639	3	3.96	1.32
NOROXYMORPHONE	9668	2	78.06	39.03
REMIFENTANIL	9739	3	0.14	0.05
FENTANYL BASE	9801	144	13.84	0.1

STATE:ARKANSAS BUSINESS ACTIVITY:D - TEACHING INSTITUTIONS

DRUG NAME	DRUG CODE	BUYERS	TOTAL GRAMS	AVG GRAMS
BUPRENORPHINE	9064	1	0	0

STATE:ARKANSAS BUSINESS ACTIVITY:M - MID-LEVEL PRACTITIONERS

DRUG NAME	DRUG CODE	BUYERS	TOTAL GRAMS	AVG GRAMS
PENTOBARBITAL (SCHEDULE 2)	2270	11	11,997.67	1,090.70
CODEINE	9050	7	13.65	1.95
DIHYDROCODEINE	9120	17	21.65	1.27

STATE:ARKANSAS BUSINESS ACTIVITY:N-U NARCOTIC TREATMENT PROGRAMS

DRUG NAME	DRUG CODE	BUYERS	TOTAL GRAMS	AVG GRAMS
BUPRENORPHINE	9064	1	25.92	25.92
METHADONE	9250B	7	29,243.30	4,177.61

STATE:CALIFORNIA BUSINESS ACTIVITY:A - PHARMACIES

DRUG NAME	DRUG CODE	BUYERS	TOTAL GRAMS	AVG GRAMS
AMPHETAMINE	1100	5,552	1,154,151.07	207.88
DL-METHAMPHETAMINE RACEMIC BASE	1105B	301	1,129.20	3.75
D-METHAMPHETAMINE	1105D	30	142.6	4.75
LISDEXAMFETAMINE	1205	4,855	438,739.17	90.37
METHYLPHENIDATE (DL;D;L;ISOMERS)	1724	5,466	1,040,896.98	190.43
BARBITURIC ACID DERIVIATIVE OR SALT [PER 21C	2100	1,616	58,865	36.43
BUTALBITAL	2165	2,342	97,400	41.59
PENTOBARBITAL (SCHEDULE 2)	2270	1	2.73	2.73
SECOBARBITAL (SCHEDULE 2)	2315	64	4,275.39	66.8
DRONABINOL IN AN ORAL SOLUTION IN FDA APPR	7365	25	43.65	1.75
NABILONE	7379	1	0.65	0.65
COCAINE	9041L	38	364.94	9.6
CODEINE	9050	6,208	1,769,207.32	284.99
BUPRENORPHINE	9064	5,225	145,614.02	27.87
DIHYDROCODEINE	9120	53	740.23	13.97
OXYCODONE	9143	5,891	3,289,504.71	558.39
HYDROMORPHONE	9150	5,195	130,572.18	25.13
HYDROCODONE	9193	6,179	3,461,545.80	560.21
LEVORPHANOL	9220L	216	398.04	1.84
MEPERIDINE (PETHIDINE)	9230	1,002	26,944.17	26.89

METHADONE	9250B	4,745	344,813.47	72.67
MORPHINE	9300	5,510	1,613,860.54	292.9
OPIUM TINCTURE (LAUDANUM)	9630	2	5.91	2.96
OPIUM POWDERED	9639	226	410.4	1.82
OXYMORPHONE	9652	2,360	51,574.47	21.85
NOROXYMORPHONE	9668	1	1.49	1.49
ALFENTANIL	9737	15	5.17	0.34
REMIFENTANIL	9739	14	1.27	0.09
SUFENTANIL BASE	9740	9	39.64	4.4
TAPENTADOL	9780	3,440	420,533	122.25
FENTANYL BASE	9801	5,083	24,570.52	4.83
OPIUM COMBINATION PRODUCT (C-III)	9809	99	71.52	0.72

STATE:CALIFORNIA BUSINESS ACTIVITY:B - HOSPITALS

DRUG NAME	DRUG CODE	BUYERS	TOTAL GRAMS	AVG GRAMS
AMPHETAMINE	1100	266	43,132.35	162.15
DL-METHAMPHETAMINE RACEMIC BASE	1105B	2	23.71	11.85
LISDEXAMFETAMINE	1205	98	7,500.27	76.53
METHYLPHENIDATE (DL;D;L;ISOMERS)	1724	401	47,016.35	117.25
GAMMA HYDROXYBUTYRIC ACID(FDA APPROVED	2012	1	2,080.51	2,080.51
BARBITURIC ACID DERIVIATIVE OR SALT [PER 21C	2100	22	240	10.91
AMOBARBITAL (SCHEDULE 2)	2125	21	100.71	4.8
BUTALBITAL	2165	50	910	18.2
PENTOBARBITAL (SCHEDULE 2)	2270	78	13,302.79	170.55
SECOBARBITAL (SCHEDULE 2)	2315	3	100.71	33.57
DRONABINOL IN AN ORAL SOLUTION IN FDA APPR	7365	1	0.15	0.15
COCAINE	9041L	401	2,855.96	7.12
CODEINE	9050	578	85,565.98	148.04
BUPRENORPHINE	9064	322	5,402.04	16.78
OXYCODONE	9143	647	162,276.17	250.81
HYDROMORPHONE	9150	672	23,609.25	35.13
HYDROCODONE	9193	729	182,538.46	250.4
LEVORPHANOL	9220L	5	4.53	0.91
MEPERIDINE (PETHIDINE)	9230	643	26,660.41	41.46
METHADONE	9250B	504	47,308.37	93.87

MORPHINE	9300	750	159,934.38	213.25
OPIUM POWDERED	9639	303	1,401.12	4.62
OXYMORPHONE	9652	61	632.48	10.37
NOROXYMORPHONE	9668	2	7.04	3.52
ALFENTANIL	9737	142	51.43	0.36
REMIFENTANIL	9739	196	128.36	0.65
SUFENTANIL BASE	9740	153	3.96	0.03
TAPENTADOL	9780	73	4,343.85	59.5
FENTANYL BASE	9801	844	2,942.75	3.49
OPIUM COMBINATION PRODUCT (C-III)	9809	10	5.11	0.51

STATE:CALIFORNIA BUSINESS ACTIVITY:C - PRACTITIONERS

DRUG NAME	DRUG CODE	BUYERS	TOTAL GRAMS	AVG GRAMS
AMPHETAMINE	1100	7	850.13	121.45
D-METHAMPHETAMINE	1105D	2	0.24	0.12
LISDEXAMFETAMINE	1205	6	50.63	8.44
METHYLPHENIDATE (DL;D;L;ISOMERS)	1724	3	70.04	23.35
GAMMA HYDROXYBUTYRIC ACID(FDA APPROVED	2012	3	5,944.32	1,981.44
BARBITURIC ACID DERIVIATIVE OR SALT [PER 21C	2100	3	78.5	26.17
PENTOBARBITAL (SCHEDULE 2)	2270	613	689,267.84	1,124.42
COCAINE	9041L	104	249.66	2.4
CODEINE	9050	1,163	36,282.66	31.2
BUPRENORPHINE	9064	2,697	1,066.33	0.4
DIHYDROCODEINE	9120	12	102.89	8.57
OXYCODONE	9143	268	1,051.39	3.92
HYDROMORPHONE	9150	1,743	1,110.82	0.64
HYDROCODONE	9193	2,072	12,275.39	5.92
MEPERIDINE (PETHIDINE)	9230	846	8,767.83	10.36
METHADONE	9250B	141	345.06	2.45
MORPHINE	9300	1,049	1,699.63	1.62
OPIUM POWDERED	9639	10	10.08	1.01
OXYMORPHONE	9652	52	8.03	0.15
NOROXYMORPHONE	9668	22	251.08	11.41
ALFENTANIL	9737	59	9.32	0.16
REMIFENTANIL	9739	63	3.72	0.06

SUFENTANIL BASE	9740	19	0.14	0.01
FENTANYL BASE	9801	2,379	302.02	0.13

STATE:CALIFORNIA BUSINESS ACTIVITY:D - TEACHING INSTITUTIONS

DRUG NAME	DRUG CODE	BUYERS	TOTAL GRAMS	AVG GRAMS
PENTOBARBITAL (SCHEDULE 2)	2270	2	622.03	311.02
BUPRENORPHINE	9064	7	0.04	0.01
HYDROMORPHONE	9150	4	0.57	0.14
MORPHINE	9300	1	2.44	2.44

STATE:CALIFORNIA BUSINESS ACTIVITY:M - MID-LEVEL PRACTITIONERS

DRUG NAME	DRUG CODE	BUYERS	TOTAL GRAMS	AVG GRAMS
PENTOBARBITAL (SCHEDULE 2)	2270	4	5,598.27	1,399.57
CODEINE	9050	133	664.45	5

STATE:CALIFORNIA BUSINESS ACTIVITY:N-U NARCOTIC TREATMENT PROGRAMS

DRUG NAME	DRUG CODE	BUYERS	TOTAL GRAMS	AVG GRAMS
BUPRENORPHINE	9064	52	485.38	9.33
METHADONE	9250B	151	996,317.77	6,598.13

STATE:COLORADO BUSINESS ACTIVITY:A - PHARMACIES

DRUG NAME	DRUG CODE	BUYERS	TOTAL GRAMS	AVG GRAMS
AMPHETAMINE	1100	819	273,180.24	333.55
DL-METHAMPHETAMINE RACEMIC BASE	1105B	83	205.75	2.48
LISDEXAMFETAMINE	1205	785	104,788.14	133.49
METHYLPHENIDATE (DL;D;L;ISOMERS)	1724	823	203,662.76	247.46
BARBITURIC ACID DERIVIATIVE OR SALT [PER 21C	2100	243	6,480	26.67
BUTALBITAL	2165	291	7,390	25.4
SECOBARBITAL (SCHEDULE 2)	2315	14	228.88	16.35

DRONABINOL IN AN ORAL SOLUTION IN FDA APPR	7365	2	3.6	1.8
NABILONE	7379	2	0.35	0.18
COCAINE	9041L	5	4.36	0.87
CODEINE	9050	804	86,742.09	107.89
BUPRENORPHINE	9064	798	25,709.27	32.22
DIHYDROCODEINE	9120	2	9.61	4.81
OXYCODONE	9143	837	766,152.43	915.36
HYDROMORPHONE	9150	809	20,978.29	25.93
HYDROCODONE	9193	840	221,473.23	263.66
LEVORPHANOL	9220L	54	50.73	0.94
MEPERIDINE (PETHIDINE)	9230	263	5,760.18	21.9
METHADONE	9250B	700	31,599.42	45.14
MORPHINE	9300	834	241,902.95	290.05
OPIUM POWDERED	9639	82	298.8	3.64
OXYMORPHONE	9652	532	12,244.12	23.02
SUFENTANIL BASE	9740	1	0.07	0.07
TAPENTADOL	9780	675	107,492	159.25
FENTANYL BASE	9801	802	3,994.35	4.98
OPIUM COMBINATION PRODUCT (C-III)	9809	25	9.46	0.38

STATE:COLORADO BUSINESS ACTIVITY:B - HOSPITALS

DRUG NAME	DRUG CODE	BUYERS	TOTAL GRAMS	AVG GRAMS
AMPHETAMINE	1100	114	46,273.94	405.91
DL-METHAMPHETAMINE RACEMIC BASE	1105B	3	4.82	1.61
LISDEXAMFETAMINE	1205	70	6,244.64	89.21
METHYLPHENIDATE (DL;D;L;ISOMERS)	1724	128	42,732.71	333.85
BARBITURIC ACID DERIVIATIVE OR SALT [PER 21C	2100	5	105	21
AMOBARBITAL (SCHEDULE 2)	2125	4	68.36	17.09
BUTALBITAL	2165	29	420	14.48
PENTOBARBITAL (SCHEDULE 2)	2270	16	1,273.68	79.61
SECOBARBITAL (SCHEDULE 2)	2315	2	146.48	73.24
DRONABINOL IN AN ORAL SOLUTION IN FDA APPR	7365	1	0.15	0.15
COCAINE	9041L	53	230.99	4.36
CODEINE	9050	147	14,835.10	100.92
BUPRENORPHINE	9064	104	3,421.99	32.9

OXYCODONE	9143	200	125,647.41	628.24
HYDROMORPHONE	9150	194	5,888.80	30.35
HYDROCODONE	9193	210	37,304.42	177.64
LEVORPHANOL	9220L	1	0.12	0.12
MEPERIDINE (PETHIDINE)	9230	102	1,099.88	10.78
METHADONE	9250B	126	9,089.61	72.14
MORPHINE	9300	204	62,931.40	308.49
OPIUM POWDERED	9639	73	654.12	8.96
OXYMORPHONE	9652	49	576.74	11.77
ALFENTANIL	9737	10	3.45	0.35
REMIFENTANIL	9739	58	24.68	0.43
SUFENTANIL BASE	9740	41	0.71	0.02
TAPENTADOL	9780	57	4,757.50	83.46
FENTANYL BASE	9801	201	934.83	4.65
OPIUM COMBINATION PRODUCT (C-III)	9809	3	1.51	0.5

STATE:COLORADO BUSINESS ACTIVITY:C - PRACTITIONERS

DRUG NAME	DRUG CODE	BUYERS	TOTAL GRAMS	AVG GRAMS
PENTOBARBITAL (SCHEDULE 2)	2270	203	154,095.84	759.09
COCAINE	9041L	19	107.71	5.67
CODEINE	9050	96	1,198.31	12.48
BUPRENORPHINE	9064	726	55.22	0.08
OXYCODONE	9143	124	806.31	6.5
HYDROMORPHONE	9150	541	330.75	0.61
HYDROCODONE	9193	392	1,140.92	2.91
MEPERIDINE (PETHIDINE)	9230	139	418.97	3.01
METHADONE	9250B	32	38.82	1.21
MORPHINE	9300	352	614.79	1.75
OPIUM POWDERED	9639	18	107.28	5.96
OXYMORPHONE	9652	11	1.28	0.12
ALFENTANIL	9737	5	0.41	0.08
REMIFENTANIL	9739	20	1.15	0.06
SUFENTANIL BASE	9740	10	0.06	0.01
TAPENTADOL	9780	1	3	3
FENTANYL BASE	9801	583	79.92	0.14

STATE:COLORADO BUSINESS ACTIVITY:D - TEACHING INSTITUTIONS

DRUG NAME	DRUG CODE	BUYERS	TOTAL GRAMS	AVG GRAMS
MORPHINE	9300	1	0.19	0.19

STATE:COLORADO BUSINESS ACTIVITY:M - MID-LEVEL PRACTITIONERS

DRUG NAME	DRUG CODE	BUYERS	TOTAL GRAMS	AVG GRAMS
PENTOBARBITAL (SCHEDULE 2)	2270	17	24,086.02	1,416.82
CODEINE	9050	39	93.35	2.39
OXYCODONE	9143	1	0.9	0.9
HYDROMORPHONE	9150	7	0.76	0.11
MORPHINE	9300	30	10.51	0.35
FENTANYL BASE	9801	49	1.49	0.03

STATE:COLORADO BUSINESS ACTIVITY:N-U NARCOTIC TREATMENT PROGRAMS

DRUG NAME	DRUG CODE	BUYERS	TOTAL GRAMS	AVG GRAMS
BUPRENORPHINE	9064	12	426.79	35.57
METHADONE	9250B	23	132,290.70	5,751.77

STATE:CONNECTICUT BUSINESS ACTIVITY:A - PHARMACIES

DRUG NAME	DRUG CODE	BUYERS	TOTAL GRAMS	AVG GRAMS
AMPHETAMINE	1100	728	206,885.22	284.18
DL-METHAMPHETAMINE RACEMIC BASE	1105B	23	84.39	3.67
D-METHAMPHETAMINE	1105D	1	0.4	0.4
LISDEXAMFETAMINE	1205	714	142,992.46	200.27
METHYLPHENIDATE (DL;D;L;ISOMERS)	1724	728	212,034.03	291.26
BARBITURIC ACID DERIVIATIVE OR SALT [PER 21C	2100	244	8,705	35.68
BUTALBITAL	2165	301	12,250	40.7
SECOBARBITAL (SCHEDULE 2)	2315	5	91.55	18.31

DRONABINOL IN AN ORAL SOLUTION IN FDA APPR	7365	3	1.5	0.5
NABILONE	7379	3	0.35	0.12
CODEINE	9050	717	91,857.14	128.11
BUPRENORPHINE	9064	721	56,869.95	78.88
DIHYDROCODEINE	9120	1	2.14	2.14
OXYCODONE	9143	736	642,640.49	873.15
HYDROMORPHONE	9150	704	21,634.27	30.73
HYDROCODONE	9193	736	77,719.32	105.6
LEVORPHANOL	9220L	52	20.08	0.39
MEPERIDINE (PETHIDINE)	9230	115	3,549.73	30.87
METHADONE	9250B	548	22,552.56	41.15
MORPHINE	9300	699	128,704.46	184.13
OPIUM TINCTURE (LAUDANUM)	9630	1	4.73	4.73
OPIUM POWDERED	9639	65	267.84	4.12
OXYMORPHONE	9652	428	10,428.20	24.36
SUFENTANIL BASE	9740	1	1.8	1.8
TAPENTADOL	9780	494	45,647.50	92.4
FENTANYL BASE	9801	684	3,444.83	5.04
OPIUM COMBINATION PRODUCT (C-III)	9809	25	16.27	0.65

STATE:CONNECTICUT BUSINESS ACTIVITY:B - HOSPITALS

DRUG NAME	DRUG CODE	BUYERS	TOTAL GRAMS	AVG GRAMS
AMPHETAMINE	1100	40	4,376.06	109.4
DL-METHAMPHETAMINE RACEMIC BASE	1105B	1	5.22	5.22
D-METHAMPHETAMINE	1105D	1	7.63	7.63
LISDEXAMFETAMINE	1205	20	1,759.16	87.96
METHYLPHENIDATE (DL;D;L;ISOMERS)	1724	53	4,281.91	80.79
BARBITURIC ACID DERIVIATIVE OR SALT [PER 21C	2100	1	5	5
AMOBARBITAL (SCHEDULE 2)	2125	2	3.65	1.82
BUTALBITAL	2165	4	50	12.5
PENTOBARBITAL (SCHEDULE 2)	2270	6	498.99	83.17
DRONABINOL IN AN ORAL SOLUTION IN FDA APPR	7365	1	0.15	0.15
COCAINE	9041L	25	128.35	5.13
CODEINE	9050	49	4,123.28	84.15
BUPRENORPHINE	9064	52	2,306.85	44.36

OXYCODONE	9143	71	19,490.04	274.51
HYDROMORPHONE	9150	62	3,809.77	61.45
HYDROCODONE	9193	55	2,076.54	37.76
LEVORPHANOL	9220L	1	0.12	0.12
MEPERIDINE (PETHIDINE)	9230	47	415.27	8.84
METHADONE	9250B	49	4,550.39	92.87
MORPHINE	9300	62	12,266.95	197.85
OPIUM POWDERED	9639	32	116.64	3.65
OXYMORPHONE	9652	7	66.54	9.51
ALFENTANIL	9737	4	3.93	0.98
REMIFENTANIL	9739	24	33.4	1.39
SUFENTANIL BASE	9740	10	0.59	0.06
TAPENTADOL	9780	6	401.5	66.92
FENTANYL BASE	9801	64	323.04	5.05
OPIUM COMBINATION PRODUCT (C-III)	9809	3	3.22	1.07

STATE:CONNECTICUT BUSINESS ACTIVITY:C - PRACTITIONERS

DRUG NAME	DRUG CODE	BUYERS	TOTAL GRAMS	AVG GRAMS
AMPHETAMINE	1100	1	11.26	11.26
PENTOBARBITAL (SCHEDULE 2)	2270	85	71,953.07	846.51
COCAINE	9041L	3	22.57	7.52
CODEINE	9050	75	325.73	4.34
BUPRENORPHINE	9064	321	23.26	0.07
DIHYDROCODEINE	9120	5	1.61	0.32
OXYCODONE	9143	56	1,330.12	23.75
HYDROMORPHONE	9150	173	131.72	0.76
HYDROCODONE	9193	105	563.54	5.37
MEPERIDINE (PETHIDINE)	9230	31	297.51	9.6
METHADONE	9250B	20	72.85	3.64
MORPHINE	9300	118	483.51	4.1
OPIUM POWDERED	9639	2	0.72	0.36
OXYMORPHONE	9652	3	0.16	0.05
ALFENTANIL	9737	3	1.36	0.45
REMIFENTANIL	9739	8	0.66	0.08
SUFENTANIL BASE	9740	2	0	0

FENTANYL BASE	9801	245	32.6	0.13

STATE:CONNECTICUT BUSINESS ACTIVITY:M - MID-LEVEL PRACTITIONERS

DRUG NAME	DRUG CODE	BUYERS	TOTAL GRAMS	AVG GRAMS
CODEINE	9050	7	10.01	1.43
FENTANYL BASE	9801	1	0.14	0.14

STATE:CONNECTICUT BUSINESS ACTIVITY:N-U NARCOTIC TREATMENT PROGRAMS

DRUG NAME	DRUG CODE	BUYERS	TOTAL GRAMS	AVG GRAMS
BUPRENORPHINE	9064	4	92.7	23.18
METHADONE	9250B	41	449,228.95	10,956.80

STATE:DELAWARE BUSINESS ACTIVITY:A - PHARMACIES

DRUG NAME	DRUG CODE	BUYERS	TOTAL GRAMS	AVG GRAMS
AMPHETAMINE	1100	209	84,389.92	403.78
DL-METHAMPHETAMINE RACEMIC BASE	1105B	3	15.27	5.09
LISDEXAMFETAMINE	1205	206	56,716.81	275.32
METHYLPHENIDATE (DL;D;L;ISOMERS)	1724	207	73,396.46	354.57
BARBITURIC ACID DERIVIATIVE OR SALT [PER 21C	2100	60	1,840	30.67
BUTALBITAL	2165	67	2,095	31.27
SECOBARBITAL (SCHEDULE 2)	2315	1	27.47	27.47
DRONABINOL IN AN ORAL SOLUTION IN FDA APPR	7365	1	0.15	0.15
CODEINE	9050	210	35,516.15	169.12
BUPRENORPHINE	9064	201	11,393.49	56.68
DIHYDROCODEINE	9120	16	29.91	1.87
OXYCODONE	9143	211	264,822.16	1,255.08
HYDROMORPHONE	9150	204	7,852.12	38.49
HYDROCODONE	9193	211	22,889.35	108.48
LEVORPHANOL	9220L	4	2.55	0.64
MEPERIDINE (PETHIDINE)	9230	31	614.41	19.82
METHADONE	9250B	168	11,380.97	67.74

MORPHINE	9300	205	80,773.28	394.02
OPIUM POWDERED	9639	10	27.72	2.77
OXYMORPHONE	9652	151	7,688.79	50.92
TAPENTADOL	9780	175	29,534.50	168.77
FENTANYL BASE	9801	203	1,260.05	6.21
OPIUM COMBINATION PRODUCT (C-III)	9809	4	2.08	0.52

STATE:DELAWARE BUSINESS ACTIVITY:B - HOSPITALS

DRUG NAME	DRUG CODE	BUYERS	TOTAL GRAMS	AVG GRAMS
AMPHETAMINE	1100	14	1,811.98	129.43
LISDEXAMFETAMINE	1205	7	500.06	71.44
METHYLPHENIDATE (DL;D;L;ISOMERS)	1724	15	1,689.96	112.66
BUTALBITAL	2165	1	15	15
PENTOBARBITAL (SCHEDULE 2)	2270	2	402.84	201.42
COCAINE	9041L	11	51.85	4.71
CODEINE	9050	19	1,255.38	66.07
BUPRENORPHINE	9064	18	811.51	45.08
OXYCODONE	9143	27	8,980.44	332.61
HYDROMORPHONE	9150	27	655.98	24.3
HYDROCODONE	9193	24	1,018.62	42.44
MEPERIDINE (PETHIDINE)	9230	16	252.63	15.79
METHADONE	9250B	17	1,555.72	91.51
MORPHINE	9300	27	5,820.77	215.58
OPIUM TINCTURE (LAUDANUM)	9630	1	1.18	1.18
OPIUM POWDERED	9639	7	32.4	4.63
OXYMORPHONE	9652	5	36.84	7.37
REMIFENTANIL	9739	11	12.04	1.09
SUFENTANIL BASE	9740	3	0.03	0.01
TAPENTADOL	9780	4	139.5	34.88
FENTANYL BASE	9801	35	95.4	2.73

STATE:DELAWARE BUSINESS ACTIVITY:C - PRACTITIONERS

DRUG NAME	DRUG CODE	BUYERS	TOTAL GRAMS	AVG GRAMS

METHYLPHENIDATE (DL;D;L;ISOMERS)	1724	1	9.34	9.34
PENTOBARBITAL (SCHEDULE 2)	2270	11	6,746.05	613.28
CODEINE	9050	26	209.22	8.05
BUPRENORPHINE	9064	57	3.79	0.07
DIHYDROCODEINE	9120	55	22.6	0.41
OXYCODONE	9143	5	3.85	0.77
HYDROMORPHONE	9150	32	14.27	0.45
HYDROCODONE	9193	22	52.08	2.37
MEPERIDINE (PETHIDINE)	9230	2	3.7	1.85
METHADONE	9250B	4	1.99	0.5
MORPHINE	9300	12	50.5	4.21
OXYMORPHONE	9652	1	0.01	0.01
FENTANYL BASE	9801	54	4.35	0.08

STATE:DELAWARE BUSINESS ACTIVITY:M - MID-LEVEL PRACTITIONERS

DRUG NAME	DRUG CODE	BUYERS	TOTAL GRAMS	AVG GRAMS
CODEINE	9050	4	6.33	1.58
DIHYDROCODEINE	9120	7	2.01	0.29
OXYCODONE	9143	1	1.12	1.12
HYDROMORPHONE	9150	1	0.83	0.83
METHADONE	9250B	1	0.63	0.63
MORPHINE	9300	1	42.98	42.98
FENTANYL BASE	9801	1	0.8	0.8

STATE:DELAWARE BUSINESS ACTIVITY:N-U NARCOTIC TREATMENT PROGRAMS

DRUG NAME	DRUG CODE	BUYERS	TOTAL GRAMS	AVG GRAMS
BUPRENORPHINE	9064	13	2,307.12	177.47
METHADONE	9250B	12	97,054.03	8,087.84

STATE:DISTRICT OF COLUMBIA BUSINESS ACTIVITY:A - PHARMACIES

DRUG NAME	DRUG CODE	BUYERS	TOTAL GRAMS	AVG GRAMS

AMPHETAMINE	1100	140	43,950.05	313.93
DL-METHAMPHETAMINE RACEMIC BASE	1105B	6	6.83	1.14
D-METHAMPHETAMINE	1105D	1	5.22	5.22
LISDEXAMFETAMINE	1205	129	20,641.06	160.01
METHYLPHENIDATE (DL;D;L;ISOMERS)	1724	141	28,623.51	203
BARBITURIC ACID DERIVIATIVE OR SALT [PER 21C	2100	44	970	22.05
BUTALBITAL	2165	57	1,390	24.39
DRONABINOL IN AN ORAL SOLUTION IN FDA APPR	7365	2	0.75	0.38
CODEINE	9050	150	46,132.75	307.55
BUPRENORPHINE	9064	130	9,305.34	71.58
OXYCODONE	9143	146	49,582.71	339.61
HYDROMORPHONE	9150	119	1,139.38	9.57
HYDROCODONE	9193	140	5,194.16	37.1
MEPERIDINE (PETHIDINE)	9230	16	472.03	29.5
METHADONE	9250B	46	1,225.69	26.65
MORPHINE	9300	108	9,062.96	83.92
OPIUM POWDERED	9639	6	6.48	1.08
OXYMORPHONE	9652	28	458.81	16.39
TAPENTADOL	9780	51	3,937	77.2
FENTANYL BASE	9801	95	193.66	2.04
OPIUM COMBINATION PRODUCT (C-III)	9809	3	0.95	0.32

STATE:DISTRICT OF COLUMBIA BUSINESS ACTIVITY:B - HOSPITALS

DRUG NAME	DRUG CODE	BUYERS	TOTAL GRAMS	AVG GRAMS
AMPHETAMINE	1100	11	2,715.55	246.87
LISDEXAMFETAMINE	1205	4	368.83	92.21
METHYLPHENIDATE (DL;D;L;ISOMERS)	1724	16	3,576.50	223.53
AMOBARBITAL (SCHEDULE 2)	2125	3	6.84	2.28
BUTALBITAL	2165	1	10	10
PENTOBARBITAL (SCHEDULE 2)	2270	6	2,108.52	351.42
COCAINE	9041L	6	92.89	15.48
CODEINE	9050	16	2,193.04	137.06
BUPRENORPHINE	9064	22	142.4	6.47
OXYCODONE	9143	20	8,564.29	428.21
HYDROMORPHONE	9150	24	1,026.06	42.75

HYDROCODONE	9193	21	559.86	26.66
MEPERIDINE (PETHIDINE)	9230	10	252.74	25.27
METHADONE	9250B	16	1,190.46	74.4
MORPHINE	9300	22	5,509.52	250.43
OPIUM POWDERED	9639	7	52.56	7.51
OXYMORPHONE	9652	4	20.45	5.11
REMIFENTANIL	9739	6	2.84	0.47
SUFENTANIL BASE	9740	3	0.03	0.01
TAPENTADOL	9780	3	159.5	53.17
FENTANYL BASE	9801	22	144.75	6.58
OPIUM COMBINATION PRODUCT (C-III)	9809	2	0.57	0.28

STATE:DISTRICT OF COLUMBIA BUSINESS ACTIVITY:C - PRACTITIONERS

DRUG NAME	DRUG CODE	BUYERS	TOTAL GRAMS	AVG GRAMS
AMPHETAMINE	1100	1	1.25	1.25
PENTOBARBITAL (SCHEDULE 2)	2270	3	444.31	148.1
CODEINE	9050	14	92.5	6.61
BUPRENORPHINE	9064	14	0.6	0.04
OXYCODONE	9143	5	8.97	1.79
HYDROMORPHONE	9150	3	1.88	0.63
HYDROCODONE	9193	6	12.41	2.07
MEPERIDINE (PETHIDINE)	9230	4	29.85	7.46
MORPHINE	9300	7	2.78	0.4
FENTANYL BASE	9801	14	0.31	0.02

STATE:DISTRICT OF COLUMBIA BUSINESS ACTIVITY:D - TEACHING INSTITUTIONS

DRUG NAME	DRUG CODE	BUYERS	TOTAL GRAMS	AVG GRAMS
BUPRENORPHINE	9064	1	0	0

STATE:DISTRICT OF COLUMBIA BUSINESS ACTIVITY:M - MID-LEVEL PRACTITIONERS

DRUG NAME	DRUG CODE	BUYERS	TOTAL GRAMS	AVG GRAMS

CODEINE	9050	1	2.17	2.17
FENTANYL BASE	9801	1	0.06	0.06

STATE:DISTRICT OF COLUMBIA BUSINESS ACTIVITY:N-U NARCOTIC TREATMENT PROGRAMS

DRUG NAME	DRUG CODE	BUYERS	TOTAL GRAMS	AVG GRAMS
BUPRENORPHINE	9064	1	6	6
METHADONE	9250B	5	37,493.25	7,498.65

STATE:FLORIDA BUSINESS ACTIVITY:A - PHARMACIES

DRUG NAME	DRUG CODE	BUYERS	TOTAL GRAMS	AVG GRAMS
AMPHETAMINE	1100	4,348	1,171,199.22	269.37
DL-METHAMPHETAMINE RACEMIC BASE	1105B	77	130.2	1.69
D-METHAMPHETAMINE	1105D	3	8.44	2.81
LISDEXAMFETAMINE	1205	3,969	541,680.86	136.48
METHYLPHENIDATE (DL;D;L;ISOMERS)	1724	4,198	791,875.97	188.63
BARBITURIC ACID DERIVIATIVE OR SALT [PER 21C	2100	1,908	69,705	36.53
BUTALBITAL	2165	1,769	65,295	36.91
SECOBARBITAL (SCHEDULE 2)	2315	14	366.2	26.16
DRONABINOL IN AN ORAL SOLUTION IN FDA APPR	7365	24	36.6	1.53
NABILONE	7379	12	2.15	0.18
COCAINE	9041L	11	7.93	0.72
CODEINE	9050	4,405	656,120.95	148.95
BUPRENORPHINE	9064	3,988	178,910.18	44.86
DIHYDROCODEINE	9120	164	711.39	4.34
OXYCODONE	9143	4,526	3,961,522.67	875.28
HYDROMORPHONE	9150	4,091	171,984.53	42.04
HYDROCODONE	9193	4,474	1,376,414.11	307.65
LEVORPHANOL	9220L	54	133.61	2.47
MEPERIDINE (PETHIDINE)	9230	1,353	23,971.15	17.72
METHADONE	9250B	3,351	217,131.70	64.8
MORPHINE	9300	4,238	1,467,377.71	346.24
OPIUM TINCTURE (LAUDANUM)	9630	1	14.19	14.19

OPIUM POWDERED	9639	209	538.56	2.58
OXYMORPHONE	9652	2,672	86,554.40	32.39
NOROXYMORPHONE	9668	1	1.56	1.56
REMIFENTANIL	9739	3	0.16	0.05
SUFENTANIL BASE	9740	5	9.79	1.96
TAPENTADOL	9780	2,883	390,040	135.29
FENTANYL BASE	9801	4,103	21,059.95	5.13
OPIUM COMBINATION PRODUCT (C-III)	9809	181	123.74	0.68

STATE:FLORIDA BUSINESS ACTIVITY:B - HOSPITALS

DRUG NAME	DRUG CODE	BUYERS	TOTAL GRAMS	AVG GRAMS
AMPHETAMINE	1100	189	29,995.22	158.7
DL-METHAMPHETAMINE RACEMIC BASE	1105B	1	5.63	5.63
LISDEXAMFETAMINE	1205	87	7,035.48	80.87
METHYLPHENIDATE (DL;D;L;ISOMERS)	1724	278	30,350.09	109.17
BARBITURIC ACID DERIVIATIVE OR SALT [PER 21C	2100	12	140	11.67
AMOBARBITAL (SCHEDULE 2)	2125	4	23.24	5.81
BUTALBITAL	2165	21	355	16.9
PENTOBARBITAL (SCHEDULE 2)	2270	44	3,986.01	90.59
COCAINE	9041L	241	1,103.64	4.58
CODEINE	9050	396	23,330.35	58.92
BUPRENORPHINE	9064	258	6,440.64	24.96
OXYCODONE	9143	574	139,094.80	242.33
HYDROMORPHONE	9150	591	14,398.63	24.36
HYDROCODONE	9193	576	60,189.11	104.49
LEVORPHANOL	9220L	1	2.44	2.44
MEPERIDINE (PETHIDINE)	9230	499	9,182.78	18.4
METHADONE	9250B	323	10,558.81	32.69
MORPHINE	9300	606	105,102.40	173.44
OPIUM POWDERED	9639	248	1,492.92	6.02
OXYMORPHONE	9652	30	319.06	10.64
NOROXYMORPHONE	9668	1	2.62	2.62
ALFENTANIL	9737	30	19.3	0.64
REMIFENTANIL	9739	173	106.67	0.62
SUFENTANIL BASE	9740	93	2.72	0.03

TAPENTADOL	9780	90	6,217	69.08
FENTANYL BASE	9801	788	2,210.05	2.8
OPIUM COMBINATION PRODUCT (C-III)	9809	17	14	0.82

STATE:FLORIDA BUSINESS ACTIVITY:C - PRACTITIONERS

DRUG NAME	DRUG CODE	BUYERS	TOTAL GRAMS	AVG GRAMS
AMPHETAMINE	1100	7	10.85	1.55
D-METHAMPHETAMINE	1105D	1	4.02	4.02
LISDEXAMFETAMINE	1205	7	84.08	12.01
METHYLPHENIDATE (DL;D;L;ISOMERS)	1724	7	547.59	78.23
BUTALBITAL	2165	1	70	70
PENTOBARBITAL (SCHEDULE 2)	2270	326	178,462.22	547.43
COCAINE	9041L	23	50.53	2.2
CODEINE	9050	285	1,933.98	6.79
BUPRENORPHINE	9064	1,924	612.77	0.32
DIHYDROCODEINE	9120	123	51.4	0.42
OXYCODONE	9143	103	3,452	33.51
HYDROMORPHONE	9150	741	343.79	0.46
HYDROCODONE	9193	898	3,415.34	3.8
MEPERIDINE (PETHIDINE)	9230	291	2,660.58	9.14
METHADONE	9250B	21	54.38	2.59
MORPHINE	9300	484	1,179.30	2.44
OXYMORPHONE	9652	8	3.9	0.49
NOROXYMORPHONE	9668	12	53.85	4.49
ALFENTANIL	9737	6	1.61	0.27
REMIFENTANIL	9739	14	0.61	0.04
SUFENTANIL BASE	9740	12	0.14	0.01
FENTANYL BASE	9801	1,225	82.71	0.07

STATE:FLORIDA BUSINESS ACTIVITY:M - MID-LEVEL PRACTITIONERS

DRUG NAME	DRUG CODE	BUYERS	TOTAL GRAMS	AVG GRAMS
PENTOBARBITAL (SCHEDULE 2)	2270	51	102,157.91	2,003.10
CODEINE	9050	2	2.17	1.08

BUPRENORPHINE	9064	1	0.01	0.01
DIHYDROCODEINE	9120	3	1.13	0.38

STATE:FLORIDA BUSINESS ACTIVITY:N-U NARCOTIC TREATMENT PROGRAMS

DRUG NAME	DRUG CODE	BUYERS	TOTAL GRAMS	AVG GRAMS
BUPRENORPHINE	9064	53	1,689.09	31.87
METHADONE	9250B	57	554,968.04	9,736.28

STATE:GEORGIA BUSINESS ACTIVITY:A - PHARMACIES

DRUG NAME	DRUG CODE	BUYERS	TOTAL GRAMS	AVG GRAMS
AMPHETAMINE	1100	2,239	731,893.92	326.88
DL-METHAMPHETAMINE RACEMIC BASE	1105B	35	90.82	2.59
D-METHAMPHETAMINE	1105D	1	4.82	4.82
LISDEXAMFETAMINE	1205	2,159	403,897.08	187.08
METHYLPHENIDATE (DL;D;L;ISOMERS)	1724	2,231	632,076.38	283.32
BARBITURIC ACID DERIVIATIVE OR SALT [PER 21C	2100	756	20,990	27.76
BUTALBITAL	2165	1,167	39,635	33.96
SECOBARBITAL (SCHEDULE 2)	2315	3	45.78	15.26
DRONABINOL IN AN ORAL SOLUTION IN FDA APPR	7365	8	4.35	0.54
COCAINE	9041L	4	13.03	3.26
CODEINE	9050	2,245	402,112.23	179.11
BUPRENORPHINE	9064	2,097	59,649.42	28.45
DIHYDROCODEINE	9120	324	2,046.59	6.32
OXYCODONE	9143	2,262	1,715,676.50	758.48
HYDROMORPHONE	9150	2,016	28,005.87	13.89
HYDROCODONE	9193	2,280	932,795.26	409.12
LEVORPHANOL	9220L	16	9.63	0.6
MEPERIDINE (PETHIDINE)	9230	1,329	31,934.59	24.03
METHADONE	9250B	1,866	97,497.74	52.25
MORPHINE	9300	2,182	410,549.64	188.15
OPIUM POWDERED	9639	136	189.36	1.39
OXYMORPHONE	9652	1,260	25,730.22	20.42
REMIFENTANIL	9739	1	0.02	0.02

SUFENTANIL BASE	9740	6	0.19	0.03
TAPENTADOL	9780	1,728	266,584	154.27
FENTANYL BASE	9801	2,152	9,589.62	4.46
OPIUM COMBINATION PRODUCT (C-III)	9809	62	28.19	0.45

STATE:GEORGIA BUSINESS ACTIVITY:B - HOSPITALS

DRUG NAME	DRUG CODE	BUYERS	TOTAL GRAMS	AVG GRAMS
AMPHETAMINE	1100	100	18,290.65	182.91
DL-METHAMPHETAMINE RACEMIC BASE	1105B	1	4.02	4.02
D-METHAMPHETAMINE	1105D	1	6.43	6.43
LISDEXAMFETAMINE	1205	55	5,669.43	103.08
METHYLPHENIDATE (DL;D;L;ISOMERS)	1724	145	21,622.98	149.12
BARBITURIC ACID DERIVIATIVE OR SALT [PER 21C	2100	5	30	6
AMOBARBITAL (SCHEDULE 2)	2125	5	26.43	5.29
BUTALBITAL	2165	12	165	13.75
PENTOBARBITAL (SCHEDULE 2)	2270	20	1,446.39	72.32
DRONABINOL IN AN ORAL SOLUTION IN FDA APPR	7365	1	0.3	0.3
COCAINE	9041L	83	584.14	7.04
CODEINE	9050	183	13,325.50	72.82
BUPRENORPHINE	9064	107	1,662.74	15.54
DIHYDROCODEINE	9120	2	9.61	4.81
OXYCODONE	9143	228	63,289.58	277.59
HYDROMORPHONE	9150	203	5,072.56	24.99
HYDROCODONE	9193	227	46,932.24	206.75
MEPERIDINE (PETHIDINE)	9230	174	8,144.67	46.81
METHADONE	9250B	162	3,924.45	24.22
MORPHINE	9300	225	36,615.88	162.74
OPIUM POWDERED	9639	90	535.68	5.95
OXYMORPHONE	9652	16	88.85	5.55
NOROXYMORPHONE	9668	2	5.42	2.71
ALFENTANIL	9737	12	9.41	0.78
REMIFENTANIL	9739	52	18.66	0.36
SUFENTANIL BASE	9740	38	1.43	0.04
TAPENTADOL	9780	54	3,041.50	56.32
FENTANYL BASE	9801	210	1,223.01	5.82

OPIUM COMBINATION PRODUCT (C-III)	9809	4	1.32	0.33

STATE:GEORGIA BUSINESS ACTIVITY:C - PRACTITIONERS

DRUG NAME	DRUG CODE	BUYERS	TOTAL GRAMS	AVG GRAMS
AMPHETAMINE	1100	8	79.13	9.89
LISDEXAMFETAMINE	1205	1	7.49	7.49
METHYLPHENIDATE (DL;D;L;ISOMERS)	1724	2	5.96	2.98
BARBITURIC ACID DERIVIATIVE OR SALT [PER 21C	2100	1	5	5
PENTOBARBITAL (SCHEDULE 2)	2270	183	152,367.96	832.61
COCAINE	9041L	26	59.71	2.3
CODEINE	9050	334	4,755.64	14.24
BUPRENORPHINE	9064	791	88.05	0.11
DIHYDROCODEINE	9120	207	107.3	0.52
OXYCODONE	9143	114	3,659.19	32.1
HYDROMORPHONE	9150	380	220.04	0.58
HYDROCODONE	9193	469	4,330.28	9.23
MEPERIDINE (PETHIDINE)	9230	244	1,511.99	6.2
METHADONE	9250B	10	59.3	5.93
MORPHINE	9300	326	1,389.54	4.26
OPIUM POWDERED	9639	11	30.6	2.78
OXYMORPHONE	9652	6	1.67	0.28
NOROXYMORPHONE	9668	4	15.04	3.76
ALFENTANIL	9737	8	3.64	0.46
REMIFENTANIL	9739	6	0.53	0.09
SUFENTANIL BASE	9740	8	0.17	0.02
TAPENTADOL	9780	1	5	5
FENTANYL BASE	9801	807	95.25	0.12

STATE:GEORGIA BUSINESS ACTIVITY:D - TEACHING INSTITUTIONS

DRUG NAME	DRUG CODE	BUYERS	TOTAL GRAMS	AVG GRAMS
BUPRENORPHINE	9064	1	1.95	1.95

STATE:GEORGIA BUSINESS ACTIVITY:M - MID-LEVEL PRACTITIONERS

DRUG NAME	DRUG CODE	BUYERS	TOTAL GRAMS	AVG GRAMS
CODEINE	9050	54	71.47	1.32
DIHYDROCODEINE	9120	48	34.51	0.72

STATE:GEORGIA BUSINESS ACTIVITY:N-U NARCOTIC TREATMENT PROGRAMS

DRUG NAME	DRUG CODE	BUYERS	TOTAL GRAMS	AVG GRAMS
BUPRENORPHINE	9064	32	1,469.25	45.91
METHADONE	9250B	73	364,117.40	4,987.91

STATE:GUAM BUSINESS ACTIVITY:A - PHARMACIES

DRUG NAME	DRUG CODE	BUYERS	TOTAL GRAMS	AVG GRAMS
AMPHETAMINE	1100	9	1,083.51	120.39
LISDEXAMFETAMINE	1205	4	30.06	7.52
METHYLPHENIDATE (DL;D;L;ISOMERS)	1724	9	667.16	74.13
BARBITURIC ACID DERIVIATIVE OR SALT [PER 21C	2100	1	10	10
BUTALBITAL	2165	2	40	20
CODEINE	9050	17	3,972.65	233.69
BUPRENORPHINE	9064	3	3.84	1.28
OXYCODONE	9143	11	2,489.10	226.28
HYDROMORPHONE	9150	6	83.16	13.86
HYDROCODONE	9193	11	385.19	35.02
MEPERIDINE (PETHIDINE)	9230	3	57.52	19.17
METHADONE	9250B	3	150.26	50.09
MORPHINE	9300	8	3,329.71	416.21
FENTANYL BASE	9801	7	22.44	3.21

STATE:GUAM BUSINESS ACTIVITY:B - HOSPITALS

DRUG NAME	DRUG CODE	BUYERS	TOTAL GRAMS	AVG GRAMS
AMPHETAMINE	1100	4	820.6	205.15

LISDEXAMFETAMINE	1205	3	60.7	20.23
METHYLPHENIDATE (DL;D;L;ISOMERS)	1724	6	1,247.59	207.93
COCAINE	9041L	1	0.36	0.36
CODEINE	9050	6	775.34	129.22
BUPRENORPHINE	9064	2	0.48	0.24
OXYCODONE	9143	7	932.27	133.18
HYDROMORPHONE	9150	8	31.28	3.91
HYDROCODONE	9193	5	231.17	46.23
MEPERIDINE (PETHIDINE)	9230	6	77.24	12.87
METHADONE	9250B	3	21.02	7.01
MORPHINE	9300	7	668.1	95.44
OPIUM POWDERED	9639	1	0.36	0.36
REMIFENTANIL	9739	2	0.31	0.16
FENTANYL BASE	9801	7	18.65	2.66

STATE:GUAM BUSINESS ACTIVITY:C - PRACTITIONERS

DRUG NAME	DRUG CODE	BUYERS	TOTAL GRAMS	AVG GRAMS
CODEINE	9050	4	17.68	4.42
BUPRENORPHINE	9064	2	0.17	0.08
HYDROMORPHONE	9150	1	0.07	0.07
HYDROCODONE	9193	4	13.62	3.41
MEPERIDINE (PETHIDINE)	9230	3	48.91	16.3
MORPHINE	9300	3	0.44	0.15
ALFENTANIL	9737	1	0.08	0.08
FENTANYL BASE	9801	4	0.24	0.06

STATE:GUAM BUSINESS ACTIVITY:M - MID-LEVEL PRACTITIONERS

DRUG NAME	DRUG CODE	BUYERS	TOTAL GRAMS	AVG GRAMS
PENTOBARBITAL (SCHEDULE 2)	2270	1	6,430.84	6,430.84

STATE:HAWAII BUSINESS ACTIVITY:A - PHARMACIES

STATISTICAL SUMMARY FOR RETAIL DRUG PURCHASES BY GRAMS WT

DRUG NAME	DRUG CODE	BUYERS	TOTAL GRAMS	AVG GRAMS
AMPHETAMINE	1100	221	30,177.86	136.55
DL-METHAMPHETAMINE RACEMIC BASE	1105B	8	20.49	2.56
D-METHAMPHETAMINE	1105D	1	3.21	3.21
LISDEXAMFETAMINE	1205	156	4,142.66	26.56
METHYLPHENIDATE (DL;D;L;ISOMERS)	1724	222	33,018.17	148.73
BARBITURIC ACID DERIVIATIVE OR SALT [PER 21C	2100	77	3,380	43.9
BUTALBITAL	2165	92	4,160	45.22
SECOBARBITAL (SCHEDULE 2)	2315	1	9.16	9.16
COCAINE	9041L	3	12.43	4.14
CODEINE	9050	229	49,304.87	215.31
BUPRENORPHINE	9064	203	7,598.52	37.43
DIHYDROCODEINE	9120	1	1.07	1.07
OXYCODONE	9143	231	153,634.24	665.08
HYDROMORPHONE	9150	162	2,063.28	12.74
HYDROCODONE	9193	232	62,805.34	270.71
MEPERIDINE (PETHIDINE)	9230	37	897.86	24.27
METHADONE	9250B	156	12,734.74	81.63
MORPHINE	9300	211	78,654.33	372.77
OPIUM POWDERED	9639	8	15.48	1.94
OXYMORPHONE	9652	79	1,679.02	21.25
REMIFENTANIL	9739	1	0.03	0.03
SUFENTANIL BASE	9740	1	0	0
TAPENTADOL	9780	95	11,776	123.96
FENTANYL BASE	9801	188	571.74	3.04
OPIUM COMBINATION PRODUCT (C-III)	9809	1	4.73	4.73

STATE:HAWAII BUSINESS ACTIVITY:B - HOSPITALS

DRUG NAME	DRUG CODE	BUYERS	TOTAL GRAMS	AVG GRAMS
AMPHETAMINE	1100	17	8,742.21	514.25
LISDEXAMFETAMINE	1205	10	1,215.74	121.57
METHYLPHENIDATE (DL;D;L;ISOMERS)	1724	23	10,618.14	461.66
PENTOBARBITAL (SCHEDULE 2)	2270	5	601.52	120.3
NABILONE	7379	2	0.6	0.3
COCAINE	9041L	14	83.28	5.95

CODEINE	9050	37	4,838.22	130.76
BUPRENORPHINE	9064	20	275.83	13.79
OXYCODONE	9143	40	18,191.54	454.79
HYDROMORPHONE	9150	35	580.01	16.57
HYDROCODONE	9193	40	5,322.58	133.06
MEPERIDINE (PETHIDINE)	9230	30	291.41	9.71
METHADONE	9250B	29	1,138.30	39.25
MORPHINE	9300	42	8,119.21	193.31
OPIUM POWDERED	9639	14	97.56	6.97
OXYMORPHONE	9652	6	462.55	77.09
ALFENTANIL	9737	5	2.08	0.42
REMIFENTANIL	9739	10	5.11	0.51
SUFENTANIL BASE	9740	5	0.03	0.01
TAPENTADOL	9780	9	818	90.89
FENTANYL BASE	9801	47	166.95	3.55

STATE:HAWAII BUSINESS ACTIVITY:C - PRACTITIONERS

DRUG NAME	DRUG CODE	BUYERS	TOTAL GRAMS	AVG GRAMS
PENTOBARBITAL (SCHEDULE 2)	2270	17	27,342.46	1,608.38
COCAINE	9041L	1	1.71	1.71
CODEINE	9050	9	419.65	46.63
BUPRENORPHINE	9064	77	4.98	0.06
OXYCODONE	9143	16	1,604.96	100.31
HYDROMORPHONE	9150	48	27.93	0.58
HYDROCODONE	9193	55	1,757.61	31.96
MEPERIDINE (PETHIDINE)	9230	18	167.98	9.33
METHADONE	9250B	2	5.19	2.59
MORPHINE	9300	26	384.52	14.79
OPIUM POWDERED	9639	1	2.16	2.16
OXYMORPHONE	9652	2	4.68	2.34
ALFENTANIL	9737	2	0.32	0.16
FENTANYL BASE	9801	49	3.62	0.07

STATE:HAWAII BUSINESS ACTIVITY:M - MID-LEVEL PRACTITIONERS

DRUG NAME	DRUG CODE	BUYERS	TOTAL GRAMS	AVG GRAMS
MORPHINE	9300	1	0.94	0.94
FENTANYL BASE	9801	1	0.01	0.01

STATE:HAWAII BUSINESS ACTIVITY:N-U NARCOTIC TREATMENT PROGRAMS

DRUG NAME	DRUG CODE	BUYERS	TOTAL GRAMS	AVG GRAMS
METHADONE	9250B	4	18,417.48	4,604.37

STATE:IDAHO BUSINESS ACTIVITY:A - PHARMACIES

DRUG NAME	DRUG CODE	BUYERS	TOTAL GRAMS	AVG GRAMS
AMPHETAMINE	1100	309	100,261.34	324.47
DL-METHAMPHETAMINE RACEMIC BASE	1105B	14	34.96	2.5
D-METHAMPHETAMINE	1105D	1	10.44	10.44
LISDEXAMFETAMINE	1205	297	35,229.99	118.62
METHYLPHENIDATE (DL;D;L;ISOMERS)	1724	309	107,761.78	348.74
BARBITURIC ACID DERIVIATIVE OR SALT [PER 21C	2100	63	2,150	34.13
BUTALBITAL	2165	136	5,245	38.57
COCAINE	9041L	3	64.17	21.39
CODEINE	9050	309	49,961.71	161.69
BUPRENORPHINE	9064	300	13,653.30	45.51
OXYCODONE	9143	311	229,195.06	736.96
HYDROMORPHONE	9150	300	6,605.30	22.02
HYDROCODONE	9193	315	217,517.68	690.53
LEVORPHANOL	9220L	2	0.23	0.12
MEPERIDINE (PETHIDINE)	9230	108	2,233.22	20.68
METHADONE	9250B	299	24,522.39	82.01
MORPHINE	9300	315	102,543.09	325.53
OPIUM POWDERED	9639	26	34.92	1.34
OXYMORPHONE	9652	211	5,131.85	24.32
REMIFENTANIL	9739	1	0.02	0.02
TAPENTADOL	9780	228	23,937.50	104.99
FENTANYL BASE	9801	305	2,056.93	6.74

OPIUM COMBINATION PRODUCT (C-III)	9809	10	14.95	1.49

STATE:IDAHO BUSINESS ACTIVITY:B - HOSPITALS

DRUG NAME	DRUG CODE	BUYERS	TOTAL GRAMS	AVG GRAMS
AMPHETAMINE	1100	25	1,503.22	60.13
LISDEXAMFETAMINE	1205	10	167.07	16.71
METHYLPHENIDATE (DL;D;L;ISOMERS)	1724	32	1,825.33	57.04
BARBITURIC ACID DERIVIATIVE OR SALT [PER 21C	2100	2	15	7.5
PENTOBARBITAL (SCHEDULE 2)	2270	6	201.42	33.57
COCAINE	9041L	23	143.81	6.25
CODEINE	9050	44	1,995.30	45.35
BUPRENORPHINE	9064	35	423.07	12.09
OXYCODONE	9143	64	12,004.85	187.58
HYDROMORPHONE	9150	56	873.17	15.59
HYDROCODONE	9193	66	10,088.70	152.86
MEPERIDINE (PETHIDINE)	9230	44	846.47	19.24
METHADONE	9250B	46	1,411.81	30.69
MORPHINE	9300	62	9,750.10	157.26
OPIUM POWDERED	9639	24	160.56	6.69
OXYMORPHONE	9652	4	12.22	3.06
ALFENTANIL	9737	7	5.34	0.76
REMIFENTANIL	9739	18	8.31	0.46
SUFENTANIL BASE	9740	20	0.17	0.01
TAPENTADOL	9780	8	189	23.63
FENTANYL BASE	9801	61	228.26	3.74
OPIUM COMBINATION PRODUCT (C-III)	9809	1	0.19	0.19

STATE:IDAHO BUSINESS ACTIVITY:C - PRACTITIONERS

DRUG NAME	DRUG CODE	BUYERS	TOTAL GRAMS	AVG GRAMS
PENTOBARBITAL (SCHEDULE 2)	2270	82	77,336.64	943.13
COCAINE	9041L	2	8.57	4.29
CODEINE	9050	40	2,557.93	63.95
BUPRENORPHINE	9064	126	15.12	0.12

DIHYDROCODEINE	9120	5	108.95	21.79
OXYCODONE	9143	17	153.34	9.02
HYDROMORPHONE	9150	91	47.24	0.52
HYDROCODONE	9193	105	449.37	4.28
MEPERIDINE (PETHIDINE)	9230	26	243.04	9.35
METHADONE	9250B	2	11.9	5.95
MORPHINE	9300	80	233.3	2.92
OXYMORPHONE	9652	4	1.01	0.25
ALFENTANIL	9737	1	0.11	0.11
REMIFENTANIL	9739	4	0.36	0.09
SUFENTANIL BASE	9740	1	0	0
FENTANYL BASE	9801	127	19.42	0.15

STATE:IDAHO BUSINESS ACTIVITY:M - MID-LEVEL PRACTITIONERS

DRUG NAME	DRUG CODE	BUYERS	TOTAL GRAMS	AVG GRAMS
PENTOBARBITAL (SCHEDULE 2)	2270	11	12,911.35	1,173.76
CODEINE	9050	4	128.73	32.18
HYDROMORPHONE	9150	1	0.09	0.09
HYDROCODONE	9193	3	3.3	1.1
MEPERIDINE (PETHIDINE)	9230	3	7.3	2.43
MORPHINE	9300	3	0.34	0.11
FENTANYL BASE	9801	5	0.3	0.06

STATE:IDAHO BUSINESS ACTIVITY:N-U NARCOTIC TREATMENT PROGRAMS

DRUG NAME	DRUG CODE	BUYERS	TOTAL GRAMS	AVG GRAMS
BUPRENORPHINE	9064	3	124.55	41.52
METHADONE	9250B	3	21,286.72	7,095.57

STATE:ILLINOIS BUSINESS ACTIVITY:A - PHARMACIES

DRUG NAME	DRUG CODE	BUYERS	TOTAL GRAMS	AVG GRAMS
AMPHETAMINE	1100	2,181	755,498.65	346.4

DL-METHAMPHETAMINE RACEMIC BASE	1105B	81	246.74	3.05
D-METHAMPHETAMINE	1105D	2	5.62	2.81
LISDEXAMFETAMINE	1205	1,958	281,275.71	143.65
METHYLPHENIDATE (DL;D;L;ISOMERS)	1724	2,162	686,506.49	317.53
BARBITURIC ACID DERIVIATIVE OR SALT [PER 21C	2100	823	30,160	36.65
BUTALBITAL	2165	753	29,480	39.15
SECOBARBITAL (SCHEDULE 2)	2315	7	164.79	23.54
DRONABINOL IN AN ORAL SOLUTION IN FDA APPR	7365	6	1.05	0.18
NABILONE	7379	3	0.5	0.17
COCAINE	9041L	5	42.82	8.56
CODEINE	9050	2,244	630,936.51	281.17
BUPRENORPHINE	9064	1,932	54,012.46	27.96
DIHYDROCODEINE	9120	16	121.77	7.61
OXYCODONE	9143	2,137	605,547.95	283.36
HYDROMORPHONE	9150	1,883	31,714.94	16.84
HYDROCODONE	9193	2,252	1,167,638.05	518.49
LEVORPHANOL	9220L	48	52.7	1.1
MEPERIDINE (PETHIDINE)	9230	244	6,430.25	26.35
METHADONE	9250B	1,508	48,612.25	32.24
MORPHINE	9300	2,041	370,205.33	181.38
OPIUM TINCTURE (LAUDANUM)	9630	3	16.55	5.52
OPIUM POWDERED	9639	119	174.96	1.47
OXYMORPHONE	9652	975	15,986.43	16.4
ALFENTANIL	9737	1	0.05	0.05
REMIFENTANIL	9739	1	0.01	0.01
TAPENTADOL	9780	1,342	150,273	111.98
FENTANYL BASE	9801	2,038	10,531.53	5.17
OPIUM COMBINATION PRODUCT (C-III)	9809	37	22.33	0.6

STATE:ILLINOIS BUSINESS ACTIVITY:B - HOSPITALS

DRUG NAME	DRUG CODE	BUYERS	TOTAL GRAMS	AVG GRAMS
AMPHETAMINE	1100	132	8,461.82	64.1
DL-METHAMPHETAMINE RACEMIC BASE	1105B	1	1.61	1.61
D-METHAMPHETAMINE	1105D	1	0.4	0.4
LISDEXAMFETAMINE	1205	53	2,164.41	40.84

METHYLPHENIDATE (DL;D;L;ISOMERS)	1724	188	9,748.73	51.85
GAMMA HYDROXYBUTYRIC ACID(FDA APPROVED	2012	1	2,674.94	2,674.94
BARBITURIC ACID DERIVIATIVE OR SALT [PER 21C	2100	9	75	8.33
AMOBARBITAL (SCHEDULE 2)	2125	9	15.95	1.77
BUTALBITAL	2165	16	180	11.25
PENTOBARBITAL (SCHEDULE 2)	2270	27	2,103.51	77.91
COCAINE	9041L	133	881.76	6.63
CODEINE	9050	233	12,116.88	52
BUPRENORPHINE	9064	138	1,562.62	11.32
OXYCODONE	9143	229	26,695.81	116.58
HYDROMORPHONE	9150	228	6,838.18	29.99
HYDROCODONE	9193	248	59,244.22	238.89
MEPERIDINE (PETHIDINE)	9230	184	4,303.10	23.39
METHADONE	9250B	177	6,321.13	35.71
MORPHINE	9300	235	38,564.19	164.1
OPIUM POWDERED	9639	129	537.12	4.16
OXYMORPHONE	9652	27	311.1	11.52
ALFENTANIL	9737	23	8.2	0.36
REMIFENTANIL	9739	83	60.32	0.73
SUFENTANIL BASE	9740	75	2.28	0.03
TAPENTADOL	9780	47	1,420.50	30.22
FENTANYL BASE	9801	236	1,186.29	5.03
OPIUM COMBINATION PRODUCT (C-III)	9809	6	1.51	0.25

STATE:ILLINOIS BUSINESS ACTIVITY:C - PRACTITIONERS

DRUG NAME	DRUG CODE	BUYERS	TOTAL GRAMS	AVG GRAMS
AMPHETAMINE	1100	2	6.69	3.34
METHYLPHENIDATE (DL;D;L;ISOMERS)	1724	1	50.28	50.28
PENTOBARBITAL (SCHEDULE 2)	2270	384	243,551.43	634.25
COCAINE	9041L	40	365.55	9.14
CODEINE	9050	342	4,194.42	12.26
BUPRENORPHINE	9064	847	62.58	0.07
DIHYDROCODEINE	9120	15	549.33	36.62
OXYCODONE	9143	56	369.72	6.6
HYDROMORPHONE	9150	456	340.08	0.75

HYDROCODONE	9193	588	4,300.34	7.31
MEPERIDINE (PETHIDINE)	9230	148	1,277.40	8.63
METHADONE	9250B	20	39.98	2
MORPHINE	9300	274	564.98	2.06
OPIUM POWDERED	9639	8	5.4	0.68
OXYMORPHONE	9652	1	0.03	0.03
NOROXYMORPHONE	9668	4	38.02	9.5
ALFENTANIL	9737	8	8.25	1.03
REMIFENTANIL	9739	10	0.43	0.04
SUFENTANIL BASE	9740	2	0.01	0
TAPENTADOL	9780	1	5	5
FENTANYL BASE	9801	641	88.23	0.14

STATE:ILLINOIS BUSINESS ACTIVITY:D - TEACHING INSTITUTIONS

DRUG NAME	DRUG CODE	BUYERS	TOTAL GRAMS	AVG GRAMS
PENTOBARBITAL (SCHEDULE 2)	2270	3	10,397.71	3,465.90
CODEINE	9050	1	2.39	2.39
BUPRENORPHINE	9064	2	0.73	0.36
HYDROMORPHONE	9150	1	12	12
HYDROCODONE	9193	1	11.69	11.69
METHADONE	9250B	1	10.73	10.73
MORPHINE	9300	1	19.93	19.93
FENTANYL BASE	9801	2	3.1	1.55

STATE:ILLINOIS BUSINESS ACTIVITY:M - MID-LEVEL PRACTITIONERS

DRUG NAME	DRUG CODE	BUYERS	TOTAL GRAMS	AVG GRAMS
PENTOBARBITAL (SCHEDULE 2)	2270	15	21,209.19	1,413.95
CODEINE	9050	12	27.94	2.33

STATE:ILLINOIS BUSINESS ACTIVITY:N-U NARCOTIC TREATMENT PROGRAMS

DRUG NAME	DRUG CODE	BUYERS	TOTAL GRAMS	AVG GRAMS

BUPRENORPHINE	9064	11	1,889.50	171.77
METHADONE	9250B	78	434,440.04	5,569.74

STATE:INDIANA BUSINESS ACTIVITY:A - PHARMACIES

DRUG NAME	DRUG CODE	BUYERS	TOTAL GRAMS	AVG GRAMS
AMPHETAMINE	1100	1,186	511,273.61	431.09
DL-METHAMPHETAMINE RACEMIC BASE	1105B	27	103.68	3.84
LISDEXAMFETAMINE	1205	1,168	337,130.58	288.64
METHYLPHENIDATE (DL;D;L;ISOMERS)	1724	1,188	477,422.05	401.87
BARBITURIC ACID DERIVIATIVE OR SALT [PER 21C	2100	443	13,115	29.6
BUTALBITAL	2165	460	15,355	33.38
DRONABINOL IN AN ORAL SOLUTION IN FDA APPR	7365	3	1.65	0.55
COCAINE	9041L	1	8.93	8.93
CODEINE	9050	1,162	195,023.17	167.83
BUPRENORPHINE	9064	1,134	75,445.03	66.53
DIHYDROCODEINE	9120	6	7.48	1.25
OXYCODONE	9143	1,194	886,532.77	742.49
HYDROMORPHONE	9150	1,051	18,399.72	17.51
HYDROCODONE	9193	1,204	861,296.73	715.36
LEVORPHANOL	9220L	40	97.28	2.43
MEPERIDINE (PETHIDINE)	9230	334	5,546.23	16.61
METHADONE	9250B	1,012	66,355.89	65.57
MORPHINE	9300	1,168	314,519.94	269.28
OPIUM TINCTURE (LAUDANUM)	9630	6	35.47	5.91
OPIUM POWDERED	9639	99	274.68	2.77
OXYMORPHONE	9652	780	19,363.57	24.83
REMIFENTANIL	9739	1	0.12	0.12
TAPENTADOL	9780	891	148,182	166.31
FENTANYL BASE	9801	1,144	6,586.88	5.76
OPIUM COMBINATION PRODUCT (C-III)	9809	20	20.62	1.03

STATE:INDIANA BUSINESS ACTIVITY:B - HOSPITALS

DRUG NAME	DRUG CODE	BUYERS	TOTAL GRAMS	AVG GRAMS

AMPHETAMINE	1100	126	8,717.01	69.18
LISDEXAMFETAMINE	1205	74	4,238.63	57.28
METHYLPHENIDATE (DL;D;L;ISOMERS)	1724	146	7,870.13	53.91
BARBITURIC ACID DERIVIATIVE OR SALT [PER 21C	2100	6	45	7.5
AMOBARBITAL (SCHEDULE 2)	2125	2	4.1	2.05
BUTALBITAL	2165	21	240	11.43
PENTOBARBITAL (SCHEDULE 2)	2270	18	10,266.37	570.35
COCAINE	9041L	107	790.84	7.39
CODEINE	9050	172	4,943.54	28.74
BUPRENORPHINE	9064	120	1,982.98	16.52
OXYCODONE	9143	241	37,689.78	156.39
HYDROMORPHONE	9150	239	5,318.78	22.25
HYDROCODONE	9193	261	42,959.24	164.59
LEVORPHANOL	9220L	1	0.12	0.12
MEPERIDINE (PETHIDINE)	9230	220	7,708.61	35.04
METHADONE	9250B	138	2,621.52	19
MORPHINE	9300	260	29,019.77	111.61
OPIUM POWDERED	9639	130	707.04	5.44
OXYMORPHONE	9652	22	104.44	4.75
ALFENTANIL	9737	37	9.96	0.27
REMIFENTANIL	9739	82	31.76	0.39
SUFENTANIL BASE	9740	50	1.12	0.02
TAPENTADOL	9780	36	1,249.50	34.71
FENTANYL BASE	9801	292	883.3	3.02
OPIUM COMBINATION PRODUCT (C-III)	9809	2	8.51	4.26

STATE:INDIANA BUSINESS ACTIVITY:C - PRACTITIONERS

DRUG NAME	DRUG CODE	BUYERS	TOTAL GRAMS	AVG GRAMS
METHYLPHENIDATE (DL;D;L;ISOMERS)	1724	1	6.92	6.92
PENTOBARBITAL (SCHEDULE 2)	2270	215	153,774.86	715.23
COCAINE	9041L	3	1.14	0.38
CODEINE	9050	91	456.12	5.01
BUPRENORPHINE	9064	438	42.05	0.1
OXYCODONE	9143	16	85.64	5.35
HYDROMORPHONE	9150	233	110.41	0.47

HYDROCODONE	9193	258	651.26	2.52
MEPERIDINE (PETHIDINE)	9230	60	170.81	2.85
METHADONE	9250B	4	3.76	0.94
MORPHINE	9300	143	250.19	1.75
OXYMORPHONE	9652	2	0.05	0.03
ALFENTANIL	9737	3	0.33	0.11
REMIFENTANIL	9739	1	0.05	0.05
SUFENTANIL BASE	9740	3	0.03	0.01
FENTANYL BASE	9801	257	53.39	0.21

STATE:INDIANA BUSINESS ACTIVITY:M - MID-LEVEL PRACTITIONERS

DRUG NAME	DRUG CODE	BUYERS	TOTAL GRAMS	AVG GRAMS
PENTOBARBITAL (SCHEDULE 2)	2270	24	15,743.43	655.98
CODEINE	9050	18	42.53	2.36
HYDROCODONE	9193	1	0.61	0.61

STATE:INDIANA BUSINESS ACTIVITY:N-U NARCOTIC TREATMENT PROGRAMS

DRUG NAME	DRUG CODE	BUYERS	TOTAL GRAMS	AVG GRAMS
BUPRENORPHINE	9064	9	2,174.67	241.63
METHADONE	9250B	14	257,953.90	18,425.28

STATE:IOWA BUSINESS ACTIVITY:A - PHARMACIES

DRUG NAME	DRUG CODE	BUYERS	TOTAL GRAMS	AVG GRAMS
AMPHETAMINE	1100	688	194,438.69	282.61
DL-METHAMPHETAMINE RACEMIC BASE	1105B	21	73.94	3.52
LISDEXAMFETAMINE	1205	672	135,650.01	201.86
METHYLPHENIDATE (DL;D;L;ISOMERS)	1724	694	318,706.20	459.23
BARBITURIC ACID DERIVIATIVE OR SALT [PER 21C	2100	104	2,780	26.73
BUTALBITAL	2165	192	5,090	26.51
NABILONE	7379	1	0.1	0.1
COCAINE	9041L	2	0.43	0.21

CODEINE	9050	689	76,566.52	111.13
BUPRENORPHINE	9064	541	5,967.20	11.03
DIHYDROCODEINE	9120	2	2.14	1.07
OXYCODONE	9143	696	199,787.63	287.05
HYDROMORPHONE	9150	649	9,520.96	14.67
HYDROCODONE	9193	707	214,597.76	303.53
LEVORPHANOL	9220L	17	15.32	0.9
MEPERIDINE (PETHIDINE)	9230	123	2,008.59	16.33
METHADONE	9250B	543	16,170.48	29.78
MORPHINE	9300	691	119,127.13	172.4
OPIUM POWDERED	9639	75	104.76	1.4
OXYMORPHONE	9652	265	4,048.04	15.28
SUFENTANIL BASE	9740	1	0.01	0.01
TAPENTADOL	9780	436	48,801	111.93
FENTANYL BASE	9801	678	3,141.82	4.63
OPIUM COMBINATION PRODUCT (C-III)	9809	6	3.97	0.66

STATE:IOWA BUSINESS ACTIVITY:B - HOSPITALS

DRUG NAME	DRUG CODE	BUYERS	TOTAL GRAMS	AVG GRAMS
AMPHETAMINE	1100	48	5,331.42	111.07
LISDEXAMFETAMINE	1205	36	1,989.82	55.27
METHYLPHENIDATE (DL;D;L;ISOMERS)	1724	84	7,370.22	87.74
AMOBARBITAL (SCHEDULE 2)	2125	1	0.46	0.46
BUTALBITAL	2165	2	10	5
PENTOBARBITAL (SCHEDULE 2)	2270	8	1,193.48	149.18
COCAINE	9041L	68	354.69	5.22
CODEINE	9050	114	3,015.04	26.45
BUPRENORPHINE	9064	55	303.55	5.52
OXYCODONE	9143	143	13,814.30	96.6
HYDROMORPHONE	9150	143	2,335.97	16.34
HYDROCODONE	9193	149	14,670.64	98.46
LEVORPHANOL	9220L	1	0.12	0.12
MEPERIDINE (PETHIDINE)	9230	125	4,288.46	34.31
METHADONE	9250B	74	1,252.52	16.93
MORPHINE	9300	147	14,705.59	100.04

OPIUM POWDERED	9639	64	296.64	4.64
OXYMORPHONE	9652	12	59.18	4.93
ALFENTANIL	9737	22	6.37	0.29
REMIFENTANIL	9739	23	12.58	0.55
SUFENTANIL BASE	9740	27	0.19	0.01
TAPENTADOL	9780	31	900	29.03
FENTANYL BASE	9801	151	310.3	2.05
OPIUM COMBINATION PRODUCT (C-III)	9809	1	4.92	4.92

STATE:IOWA BUSINESS ACTIVITY:C - PRACTITIONERS

DRUG NAME	DRUG CODE	BUYERS	TOTAL GRAMS	AVG GRAMS
PENTOBARBITAL (SCHEDULE 2)	2270	98	67,910.41	692.96
COCAINE	9041L	5	37.89	7.58
CODEINE	9050	21	314.59	14.98
BUPRENORPHINE	9064	234	11.94	0.05
OXYCODONE	9143	9	221.75	24.64
HYDROMORPHONE	9150	95	35.09	0.37
HYDROCODONE	9193	103	408.91	3.97
MEPERIDINE (PETHIDINE)	9230	30	1,483.84	49.46
METHADONE	9250B	1	0.54	0.54
MORPHINE	9300	72	141.84	1.97
OPIUM POWDERED	9639	2	1.08	0.54
ALFENTANIL	9737	1	0.31	0.31
SUFENTANIL BASE	9740	1	0.01	0.01
FENTANYL BASE	9801	94	24.42	0.26

STATE:IOWA BUSINESS ACTIVITY:D - TEACHING INSTITUTIONS

DRUG NAME	DRUG CODE	BUYERS	TOTAL GRAMS	AVG GRAMS
HYDROMORPHONE	9150	2	0.14	0.07
MORPHINE	9300	1	0.38	0.38

STATE:IOWA BUSINESS ACTIVITY:M - MID-LEVEL PRACTITIONERS

DRUG NAME	DRUG CODE	BUYERS	TOTAL GRAMS	AVG GRAMS
PENTOBARBITAL (SCHEDULE 2)	2270	5	2,399.26	479.85
FENTANYL BASE	9801	1	0.01	0.01

STATE:IOWA BUSINESS ACTIVITY:N-U NARCOTIC TREATMENT PROGRAMS

DRUG NAME	DRUG CODE	BUYERS	TOTAL GRAMS	AVG GRAMS
BUPRENORPHINE	9064	6	447.66	74.61
METHADONE	9250B	8	34,847.61	4,355.95

STATE:KANSAS BUSINESS ACTIVITY:A - PHARMACIES

DRUG NAME	DRUG CODE	BUYERS	TOTAL GRAMS	AVG GRAMS
AMPHETAMINE	1100	623	232,401.60	373.04
DL-METHAMPHETAMINE RACEMIC BASE	1105B	10	39.78	3.98
D-METHAMPHETAMINE	1105D	2	1.61	0.8
LISDEXAMFETAMINE	1205	609	125,099.11	205.42
METHYLPHENIDATE (DL;D;L;ISOMERS)	1724	625	190,841.84	305.35
BARBITURIC ACID DERIVIATIVE OR SALT [PER 21C	2100	142	4,815	33.91
BUTALBITAL	2165	240	8,710	36.29
SECOBARBITAL (SCHEDULE 2)	2315	3	100.71	33.57
DRONABINOL IN AN ORAL SOLUTION IN FDA APPR	7365	1	0.6	0.6
COCAINE	9041L	3	19.43	6.48
CODEINE	9050	623	105,267.54	168.97
BUPRENORPHINE	9064	521	9,622.72	18.47
DIHYDROCODEINE	9120	3	18.16	6.05
OXYCODONE	9143	631	456,787.68	723.91
HYDROMORPHONE	9150	585	11,704.80	20.01
HYDROCODONE	9193	633	305,595.23	482.77
LEVORPHANOL	9220L	11	35.87	3.26
MEPERIDINE (PETHIDINE)	9230	228	4,910.79	21.54
METHADONE	9250B	529	28,409.27	53.7
MORPHINE	9300	626	159,707.27	255.12
OPIUM TINCTURE (LAUDANUM)	9630	2	5.91	2.96

OPIUM POWDERED	9639	85	317.16	3.73
OXYMORPHONE	9652	340	7,318.65	21.53
SUFENTANIL BASE	9740	4	0.34	0.08
TAPENTADOL	9780	369	33,927.50	91.94
FENTANYL BASE	9801	619	3,897.10	6.3
OPIUM COMBINATION PRODUCT (C-III)	9809	11	2.84	0.26

STATE:KANSAS BUSINESS ACTIVITY:B - HOSPITALS

DRUG NAME	DRUG CODE	BUYERS	TOTAL GRAMS	AVG GRAMS
AMPHETAMINE	1100	55	9,356.34	170.12
DL-METHAMPHETAMINE RACEMIC BASE	1105B	2	2.01	1
D-METHAMPHETAMINE	1105D	1	2.81	2.81
LISDEXAMFETAMINE	1205	27	2,053.41	76.05
METHYLPHENIDATE (DL;D;L;ISOMERS)	1724	90	9,402.80	104.48
BARBITURIC ACID DERIVIATIVE OR SALT [PER 21C	2100	1	5	5
AMOBARBITAL (SCHEDULE 2)	2125	3	13.67	4.56
BUTALBITAL	2165	1	95	95
PENTOBARBITAL (SCHEDULE 2)	2270	8	9,444.84	1,180.60
COCAINE	9041L	52	284.55	5.47
CODEINE	9050	137	5,718	41.74
BUPRENORPHINE	9064	40	376.44	9.41
OXYCODONE	9143	190	31,366.19	165.09
HYDROMORPHONE	9150	164	2,218.75	13.53
HYDROCODONE	9193	195	18,835.04	96.59
MEPERIDINE (PETHIDINE)	9230	149	2,073.32	13.91
METHADONE	9250B	85	1,836.20	21.6
MORPHINE	9300	195	17,866.07	91.62
OPIUM POWDERED	9639	76	402.84	5.3
OXYMORPHONE	9652	13	191.58	14.74
ALFENTANIL	9737	15	5.78	0.39
REMIFENTANIL	9739	27	8.67	0.32
SUFENTANIL BASE	9740	51	0.82	0.02
TAPENTADOL	9780	31	1,114.50	35.95
FENTANYL BASE	9801	201	555.23	2.76

STATE:KANSAS BUSINESS ACTIVITY:C - PRACTITIONERS

DRUG NAME	DRUG CODE	BUYERS	TOTAL GRAMS	AVG GRAMS
AMPHETAMINE	1100	1	0.74	0.74
METHYLPHENIDATE (DL;D;L;ISOMERS)	1724	1	8.65	8.65
PENTOBARBITAL (SCHEDULE 2)	2270	124	81,045.33	653.59
COCAINE	9041L	1	24.64	24.64
CODEINE	9050	21	810.36	38.59
BUPRENORPHINE	9064	256	42.53	0.17
OXYCODONE	9143	12	343.56	28.63
HYDROMORPHONE	9150	110	53.59	0.49
HYDROCODONE	9193	95	240.89	2.54
MEPERIDINE (PETHIDINE)	9230	18	86.93	4.83
MORPHINE	9300	60	93.3	1.55
OXYMORPHONE	9652	1	0.81	0.81
NOROXYMORPHONE	9668	1	2.75	2.75
ALFENTANIL	9737	1	0.2	0.2
REMIFENTANIL	9739	1	0.4	0.4
SUFENTANIL BASE	9740	3	0.01	0
FENTANYL BASE	9801	106	9.2	0.09

STATE:KANSAS BUSINESS ACTIVITY:D - TEACHING INSTITUTIONS

DRUG NAME	DRUG CODE	BUYERS	TOTAL GRAMS	AVG GRAMS
BUPRENORPHINE	9064	1	0	0

STATE:KANSAS BUSINESS ACTIVITY:M - MID-LEVEL PRACTITIONERS

DRUG NAME	DRUG CODE	BUYERS	TOTAL GRAMS	AVG GRAMS
HYDROMORPHONE	9150	13	0.73	0.06
MORPHINE	9300	24	7.38	0.31
FENTANYL BASE	9801	35	0.43	0.01

STATE:KANSAS BUSINESS ACTIVITY:N-U NARCOTIC TREATMENT PROGRAMS

DRUG NAME	DRUG CODE	BUYERS	TOTAL GRAMS	AVG GRAMS
BUPRENORPHINE	9064	6	181.86	30.31
METHADONE	9250B	9	62,200.15	6,911.13

STATE:KENTUCKY BUSINESS ACTIVITY:A - PHARMACIES

DRUG NAME	DRUG CODE	BUYERS	TOTAL GRAMS	AVG GRAMS
AMPHETAMINE	1100	1,110	263,851.55	237.7
DL-METHAMPHETAMINE RACEMIC BASE	1105B	22	39.78	1.81
D-METHAMPHETAMINE	1105D	1	3.62	3.62
LISDEXAMFETAMINE	1205	1,049	167,557.66	159.73
METHYLPHENIDATE (DL;D;L;ISOMERS)	1724	1,107	291,149.77	263.01
BARBITURIC ACID DERIVIATIVE OR SALT [PER 21C	2100	224	7,244	32.34
BUTALBITAL	2165	358	10,350	28.91
DRONABINOL IN AN ORAL SOLUTION IN FDA APPR	7365	3	1.5	0.5
NABILONE	7379	1	0.35	0.35
COCAINE	9041L	2	6.18	3.09
CODEINE	9050	1,088	184,573.48	169.64
BUPRENORPHINE	9064	1,023	136,014.80	132.96
DIHYDROCODEINE	9120	21	84.38	4.02
OXYCODONE	9143	1,124	781,156.07	694.98
HYDROMORPHONE	9150	836	8,689.11	10.39
HYDROCODONE	9193	1,138	770,019.86	676.64
LEVORPHANOL	9220L	1	0.35	0.35
MEPERIDINE (PETHIDINE)	9230	293	4,457.29	15.21
METHADONE	9250B	912	48,033.66	52.67
MORPHINE	9300	1,090	164,328.23	150.76
OPIUM TINCTURE (LAUDANUM)	9630	1	2.36	2.36
OPIUM POWDERED	9639	107	305.64	2.86
OXYMORPHONE	9652	655	14,388.81	21.97
REMIFENTANIL	9739	1	0.02	0.02
SUFENTANIL BASE	9740	1	0	0
TAPENTADOL	9780	629	57,822	91.93
FENTANYL BASE	9801	1,051	3,919.27	3.73

OPIUM COMBINATION PRODUCT (C-III)	9809	39	34.43	0.88

STATE:KENTUCKY BUSINESS ACTIVITY:B - HOSPITALS

DRUG NAME	DRUG CODE	BUYERS	TOTAL GRAMS	AVG GRAMS
AMPHETAMINE	1100	51	8,096.12	158.75
LISDEXAMFETAMINE	1205	26	2,073.60	79.75
METHYLPHENIDATE (DL;D;L;ISOMERS)	1724	83	9,018.09	108.65
BARBITURIC ACID DERIVIATIVE OR SALT [PER 21C	2100	3	15	5
AMOBARBITAL (SCHEDULE 2)	2125	3	19.14	6.38
BUTALBITAL	2165	3	15	5
PENTOBARBITAL (SCHEDULE 2)	2270	6	1,496.97	249.5
COCAINE	9041L	71	426.08	6
CODEINE	9050	129	4,860.51	37.68
BUPRENORPHINE	9064	83	1,205.18	14.52
OXYCODONE	9143	142	34,425.58	242.43
HYDROMORPHONE	9150	129	2,830.51	21.94
HYDROCODONE	9193	145	28,967.44	199.78
MEPERIDINE (PETHIDINE)	9230	122	4,403.01	36.09
METHADONE	9250B	92	2,853.98	31.02
MORPHINE	9300	139	20,441.54	147.06
OPIUM POWDERED	9639	66	374.4	5.67
OXYMORPHONE	9652	20	74.31	3.72
ALFENTANIL	9737	10	4.13	0.41
REMIFENTANIL	9739	35	19	0.54
SUFENTANIL BASE	9740	33	1.35	0.04
TAPENTADOL	9780	20	2,312	115.6
FENTANYL BASE	9801	139	610.82	4.39
OPIUM COMBINATION PRODUCT (C-III)	9809	7	2.64	0.38

STATE:KENTUCKY BUSINESS ACTIVITY:C - PRACTITIONERS

DRUG NAME	DRUG CODE	BUYERS	TOTAL GRAMS	AVG GRAMS
PENTOBARBITAL (SCHEDULE 2)	2270	126	90,647.63	719.43
COCAINE	9041L	3	2.14	0.71

CODEINE	9050	14	682.08	48.72
BUPRENORPHINE	9064	282	25.32	0.09
DIHYDROCODEINE	9120	1	1.12	1.12
OXYCODONE	9143	13	100.12	7.7
HYDROMORPHONE	9150	91	273.71	3.01
HYDROCODONE	9193	119	581.51	4.89
MEPERIDINE (PETHIDINE)	9230	43	481.39	11.2
METHADONE	9250B	1	0.89	0.89
MORPHINE	9300	136	673.02	4.95
OPIUM POWDERED	9639	1	16.56	16.56
ALFENTANIL	9737	2	12.3	6.15
REMIFENTANIL	9739	2	0.14	0.07
SUFENTANIL BASE	9740	2	0.16	0.08
FENTANYL BASE	9801	260	36.91	0.14

STATE:KENTUCKY BUSINESS ACTIVITY:D - TEACHING INSTITUTIONS

DRUG NAME	DRUG CODE	BUYERS	TOTAL GRAMS	AVG GRAMS
PENTOBARBITAL (SCHEDULE 2)	2270	1	88.86	88.86
BUPRENORPHINE	9064	1	0	0

STATE:KENTUCKY BUSINESS ACTIVITY:M - MID-LEVEL PRACTITIONERS

DRUG NAME	DRUG CODE	BUYERS	TOTAL GRAMS	AVG GRAMS
PENTOBARBITAL (SCHEDULE 2)	2270	19	19,109.95	1,005.79
CODEINE	9050	2	11.71	5.86

STATE:KENTUCKY BUSINESS ACTIVITY:N-U NARCOTIC TREATMENT PROGRAMS

DRUG NAME	DRUG CODE	BUYERS	TOTAL GRAMS	AVG GRAMS
BUPRENORPHINE	9064	19	837.29	44.07
MEPERIDINE (PETHIDINE)	9230	1	435.75	435.75
METHADONE	9250B	26	155,232.51	5,970.48

STATE:LOUISIANA BUSINESS ACTIVITY:A - PHARMACIES

DRUG NAME	DRUG CODE	BUYERS	TOTAL GRAMS	AVG GRAMS
AMPHETAMINE	1100	1,175	610,985.05	519.99
DL-METHAMPHETAMINE RACEMIC BASE	1105B	19	55.05	2.9
D-METHAMPHETAMINE	1105D	1	4.42	4.42
LISDEXAMFETAMINE	1205	1,155	369,286.81	319.73
METHYLPHENIDATE (DL;D;L;ISOMERS)	1724	1,178	333,860.42	283.41
BARBITURIC ACID DERIVIATIVE OR SALT [PER 21C	2100	416	13,385	32.18
BUTALBITAL	2165	448	15,625	34.88
DRONABINOL IN AN ORAL SOLUTION IN FDA APPR	7365	1	0.3	0.3
COCAINE	9041L	5	91.17	18.23
CODEINE	9050	1,180	169,554.20	143.69
BUPRENORPHINE	9064	1,064	75,317.98	70.79
DIHYDROCODEINE	9120	212	2,426.86	11.45
OXYCODONE	9143	1,196	740,196.42	618.89
HYDROMORPHONE	9150	979	10,401.87	10.62
HYDROCODONE	9193	1,204	614,356.93	510.26
LEVORPHANOL	9220L	19	76.26	4.01
MEPERIDINE (PETHIDINE)	9230	845	20,806.39	24.62
METHADONE	9250B	732	32,861.15	44.89
MORPHINE	9300	1,099	194,000.76	176.52
OPIUM POWDERED	9639	47	136.8	2.91
OXYMORPHONE	9652	586	26,242.24	44.78
ALFENTANIL	9737	4	0.98	0.24
REMIFENTANIL	9739	3	0.18	0.06
SUFENTANIL BASE	9740	3	3.34	1.11
TAPENTADOL	9780	662	60,225	90.97
FENTANYL BASE	9801	1,098	4,292.54	3.91
OPIUM COMBINATION PRODUCT (C-III)	9809	46	31.79	0.69

STATE:LOUISIANA BUSINESS ACTIVITY:B - HOSPITALS

DRUG NAME	DRUG CODE	BUYERS	TOTAL GRAMS	AVG GRAMS
AMPHETAMINE	1100	54	6,623.39	122.66

LISDEXAMFETAMINE	1205	30	2,067.29	68.91
METHYLPHENIDATE (DL;D;L;ISOMERS)	1724	101	4,859.95	48.12
BARBITURIC ACID DERIVIATIVE OR SALT [PER 21C	2100	4	40	10
BUTALBITAL	2165	7	125	17.86
PENTOBARBITAL (SCHEDULE 2)	2270	13	664.87	51.14
COCAINE	9041L	55	265.7	4.83
CODEINE	9050	140	3,325.39	23.75
BUPRENORPHINE	9064	94	674.26	7.17
OXYCODONE	9143	195	29,874.09	153.2
HYDROMORPHONE	9150	185	2,727.42	14.74
HYDROCODONE	9193	203	37,227.27	183.39
MEPERIDINE (PETHIDINE)	9230	173	7,322.95	42.33
METHADONE	9250B	112	2,520.28	22.5
MORPHINE	9300	193	19,603.41	101.57
OPIUM POWDERED	9639	74	261.36	3.53
OXYMORPHONE	9652	18	92.96	5.16
ALFENTANIL	9737	14	6.66	0.48
REMIFENTANIL	9739	37	18.32	0.5
SUFENTANIL BASE	9740	40	1.23	0.03
TAPENTADOL	9780	17	560.5	32.97
FENTANYL BASE	9801	191	435.19	2.28
OPIUM COMBINATION PRODUCT (C-III)	9809	2	1.51	0.76

STATE:LOUISIANA BUSINESS ACTIVITY:C - PRACTITIONERS

DRUG NAME	DRUG CODE	BUYERS	TOTAL GRAMS	AVG GRAMS
LISDEXAMFETAMINE	1205	1	5.67	5.67
GAMMA HYDROXYBUTYRIC ACID(FDA APPROVED	2012	1	445.82	445.82
PENTOBARBITAL (SCHEDULE 2)	2270	96	60,574.80	630.99
COCAINE	9041L	15	32.57	2.17
CODEINE	9050	112	913.41	8.16
BUPRENORPHINE	9064	302	11.86	0.04
DIHYDROCODEINE	9120	103	102.74	1
OXYCODONE	9143	16	168.51	10.53
HYDROMORPHONE	9150	118	43.76	0.37
HYDROCODONE	9193	215	495.95	2.31

MEPERIDINE (PETHIDINE)	9230	123	889.91	7.24
METHADONE	9250B	4	9.57	2.39
MORPHINE	9300	142	156.99	1.11
OPIUM POWDERED	9639	4	23.04	5.76
ALFENTANIL	9737	4	2.42	0.6
REMIFENTANIL	9739	2	0.07	0.04
SUFENTANIL BASE	9740	3	0.05	0.02
TAPENTADOL	9780	1	5	5
FENTANYL BASE	9801	243	27.87	0.11

STATE:LOUISIANA BUSINESS ACTIVITY:D - TEACHING INSTITUTIONS

DRUG NAME	DRUG CODE	BUYERS	TOTAL GRAMS	AVG GRAMS
HYDROMORPHONE	9150	1	0.14	0.14
FENTANYL BASE	9801	1	0.02	0.02

STATE:LOUISIANA BUSINESS ACTIVITY:M - MID-LEVEL PRACTITIONERS

DRUG NAME	DRUG CODE	BUYERS	TOTAL GRAMS	AVG GRAMS
PENTOBARBITAL (SCHEDULE 2)	2270	19	39,095.76	2,057.67
CODEINE	9050	2	2.66	1.33
DIHYDROCODEINE	9120	26	22.87	0.88

STATE:LOUISIANA BUSINESS ACTIVITY:N-U NARCOTIC TREATMENT PROGRAMS

DRUG NAME	DRUG CODE	BUYERS	TOTAL GRAMS	AVG GRAMS
BUPRENORPHINE	9064	7	309.9	44.27
METHADONE	9250B	11	107,245.72	9,749.61

STATE:MAINE BUSINESS ACTIVITY:A - PHARMACIES

DRUG NAME	DRUG CODE	BUYERS	TOTAL GRAMS	AVG GRAMS
AMPHETAMINE	1100	299	89,282.54	298.6

DL-METHAMPHETAMINE RACEMIC BASE	1105B	11	33.35	3.03
LISDEXAMFETAMINE	1205	298	66,857.84	224.36
METHYLPHENIDATE (DL;D;L;ISOMERS)	1724	304	138,784.44	456.53
BARBITURIC ACID DERIVIATIVE OR SALT [PER 21C	2100	66	1,680	25.45
BUTALBITAL	2165	98	2,515	25.66
DRONABINOL IN AN ORAL SOLUTION IN FDA APPR	7365	1	0.3	0.3
NABILONE	7379	2	0.3	0.15
COCAINE	9041L	4	4.14	1.04
CODEINE	9050	301	40,255.14	133.74
BUPRENORPHINE	9064	305	37,705.27	123.62
DIHYDROCODEINE	9120	1	1.07	1.07
OXYCODONE	9143	304	193,989.81	638.12
HYDROMORPHONE	9150	304	5,459.90	17.96
HYDROCODONE	9193	305	64,434.89	211.26
MEPERIDINE (PETHIDINE)	9230	64	1,363.03	21.3
METHADONE	9250B	287	18,667.56	65.04
MORPHINE	9300	306	60,438.30	197.51
OPIUM POWDERED	9639	23	48.6	2.11
OXYMORPHONE	9652	175	3,693.12	21.1
SUFENTANIL BASE	9740	1	0.03	0.03
TAPENTADOL	9780	119	5,572.50	46.83
FENTANYL BASE	9801	301	1,132.04	3.76
OPIUM COMBINATION PRODUCT (C-III)	9809	11	5.11	0.46

STATE:MAINE BUSINESS ACTIVITY:B - HOSPITALS

DRUG NAME	DRUG CODE	BUYERS	TOTAL GRAMS	AVG GRAMS
AMPHETAMINE	1100	24	1,389.76	57.91
LISDEXAMFETAMINE	1205	14	452.65	32.33
METHYLPHENIDATE (DL;D;L;ISOMERS)	1724	39	2,440.33	62.57
BARBITURIC ACID DERIVIATIVE OR SALT [PER 21C	2100	1	10	10
AMOBARBITAL (SCHEDULE 2)	2125	1	2.73	2.73
BUTALBITAL	2165	1	5	5
PENTOBARBITAL (SCHEDULE 2)	2270	2	109.37	54.68
COCAINE	9041L	24	83.28	3.47
CODEINE	9050	44	1,175.44	26.71

BUPRENORPHINE	9064	38	881.61	23.2
OXYCODONE	9143	46	10,305.09	224.02
HYDROMORPHONE	9150	40	1,191.32	29.78
HYDROCODONE	9193	46	3,924.26	85.31
MEPERIDINE (PETHIDINE)	9230	37	195.11	5.27
METHADONE	9250B	35	1,040.10	29.72
MORPHINE	9300	45	5,431.99	120.71
OPIUM POWDERED	9639	27	150.48	5.57
OXYMORPHONE	9652	2	5.35	2.68
ALFENTANIL	9737	9	2.28	0.25
REMIFENTANIL	9739	13	4.59	0.35
SUFENTANIL BASE	9740	13	0.05	0
TAPENTADOL	9780	2	63	31.5
FENTANYL BASE	9801	43	158.88	3.69
OPIUM COMBINATION PRODUCT (C-III)	9809	1	1.7	1.7

STATE:MAINE BUSINESS ACTIVITY:C - PRACTITIONERS

DRUG NAME	DRUG CODE	BUYERS	TOTAL GRAMS	AVG GRAMS
PENTOBARBITAL (SCHEDULE 2)	2270	108	80,060.54	741.3
CODEINE	9050	6	121.95	20.32
BUPRENORPHINE	9064	158	9.72	0.06
OXYCODONE	9143	6	12.78	2.13
HYDROMORPHONE	9150	77	35.18	0.46
HYDROCODONE	9193	57	99.73	1.75
MEPERIDINE (PETHIDINE)	9230	3	20.04	6.68
MORPHINE	9300	38	63.61	1.67
OXYMORPHONE	9652	1	0.04	0.04
ALFENTANIL	9737	2	0.41	0.21
REMIFENTANIL	9739	1	0.02	0.02
FENTANYL BASE	9801	53	31.9	0.6

STATE:MAINE BUSINESS ACTIVITY:D - TEACHING INSTITUTIONS

DRUG NAME	DRUG CODE	BUYERS	TOTAL GRAMS	AVG GRAMS

BUPRENORPHINE	9064	1	0.03	0.03

STATE:MAINE BUSINESS ACTIVITY:M - MID-LEVEL PRACTITIONERS

DRUG NAME	DRUG CODE	BUYERS	TOTAL GRAMS	AVG GRAMS
PENTOBARBITAL (SCHEDULE 2)	2270	3	710.62	236.87
CODEINE	9050	3	3.58	1.19
HYDROCODONE	9193	1	0.91	0.91

STATE:MAINE BUSINESS ACTIVITY:N-U NARCOTIC TREATMENT PROGRAMS

DRUG NAME	DRUG CODE	BUYERS	TOTAL GRAMS	AVG GRAMS
BUPRENORPHINE	9064	3	123.78	41.26
METHADONE	9250B	10	106,517.67	10,651.77

STATE:MARYLAND BUSINESS ACTIVITY:A - PHARMACIES

DRUG NAME	DRUG CODE	BUYERS	TOTAL GRAMS	AVG GRAMS
AMPHETAMINE	1100	1,233	356,309.52	288.98
DL-METHAMPHETAMINE RACEMIC BASE	1105B	42	164.36	3.91
D-METHAMPHETAMINE	1105D	2	4.82	2.41
LISDEXAMFETAMINE	1205	1,194	221,752.80	185.72
METHYLPHENIDATE (DL;D;L;ISOMERS)	1724	1,230	385,280.49	313.24
BARBITURIC ACID DERIVIATIVE OR SALT [PER 21C	2100	325	9,530	29.32
BUTALBITAL	2165	561	18,965	33.81
PENTOBARBITAL (SCHEDULE 2)	2270	1	1.82	1.82
SECOBARBITAL (SCHEDULE 2)	2315	5	219.72	43.94
DRONABINOL IN AN ORAL SOLUTION IN FDA APPR	7365	5	1.95	0.39
NABILONE	7379	10	4.1	0.41
COCAINE	9041L	9	144.28	16.03
CODEINE	9050	1,244	218,426.99	175.58
BUPRENORPHINE	9064	1,180	94,834.75	80.37
DIHYDROCODEINE	9120	12	25.64	2.14
OXYCODONE	9143	1,264	1,241,338.50	982.07

HYDROMORPHONE	9150	1,177	27,570.06	23.42
HYDROCODONE	9193	1,251	132,976.94	106.3
LEVORPHANOL	9220L	39	21.36	0.55
MEPERIDINE (PETHIDINE)	9230	327	7,490.65	22.91
METHADONE	9250B	979	59,545.93	60.82
MORPHINE	9300	1,211	281,301.48	232.29
OPIUM TINCTURE (LAUDANUM)	9630	5	12.99	2.6
OPIUM POWDERED	9639	64	125.64	1.96
OXYMORPHONE	9652	870	36,335.77	41.77
ALFENTANIL	9737	3	0.7	0.23
REMIFENTANIL	9739	4	0.22	0.06
TAPENTADOL	9780	886	127,109.50	143.46
FENTANYL BASE	9801	1,134	4,905.98	4.33
OPIUM COMBINATION PRODUCT (C-III)	9809	41	31.22	0.76

STATE:MARYLAND BUSINESS ACTIVITY:B - HOSPITALS

DRUG NAME	DRUG CODE	BUYERS	TOTAL GRAMS	AVG GRAMS
AMPHETAMINE	1100	55	9,952.40	180.95
LISDEXAMFETAMINE	1205	21	1,703.72	81.13
METHYLPHENIDATE (DL;D;L;ISOMERS)	1724	70	14,917.85	213.11
BARBITURIC ACID DERIVIATIVE OR SALT [PER 21C	2100	2	10	5
AMOBARBITAL (SCHEDULE 2)	2125	6	27.34	4.56
BUTALBITAL	2165	2	25	12.5
PENTOBARBITAL (SCHEDULE 2)	2270	12	3,301.55	275.13
COCAINE	9041L	57	357.3	6.27
CODEINE	9050	84	5,102.59	60.75
BUPRENORPHINE	9064	71	1,898.44	26.74
OXYCODONE	9143	128	50,967.30	398.18
HYDROMORPHONE	9150	125	5,666.44	45.33
HYDROCODONE	9193	107	3,909.45	36.54
MEPERIDINE (PETHIDINE)	9230	95	1,402.24	14.76
METHADONE	9250B	67	5,577.98	83.25
MORPHINE	9300	115	20,682.94	179.85
OPIUM POWDERED	9639	41	208.44	5.08
OXYMORPHONE	9652	9	262.9	29.21

ALFENTANIL	9737	5	2.2	0.44
REMIFENTANIL	9739	30	26.25	0.88
SUFENTANIL BASE	9740	13	0.21	0.02
TAPENTADOL	9780	20	2,430	121.5
FENTANYL BASE	9801	193	574.45	2.98
OPIUM COMBINATION PRODUCT (C-III)	9809	3	2.84	0.95

STATE:MARYLAND BUSINESS ACTIVITY:C - PRACTITIONERS

DRUG NAME	DRUG CODE	BUYERS	TOTAL GRAMS	AVG GRAMS
AMPHETAMINE	1100	2	1.47	0.73
PENTOBARBITAL (SCHEDULE 2)	2270	96	64,319.59	670
COCAINE	9041L	9	16	1.78
CODEINE	9050	281	8,353.46	29.73
BUPRENORPHINE	9064	537	614.76	1.14
DIHYDROCODEINE	9120	12	11.8	0.98
OXYCODONE	9143	69	3,071.25	44.51
HYDROMORPHONE	9150	261	177.92	0.68
HYDROCODONE	9193	268	1,581.30	5.9
MEPERIDINE (PETHIDINE)	9230	71	477.15	6.72
METHADONE	9250B	17	77.05	4.53
MORPHINE	9300	161	1,158.89	7.2
OPIUM POWDERED	9639	1	1.08	1.08
OXYMORPHONE	9652	18	451.47	25.08
NOROXYMORPHONE	9668	1	0.2	0.2
REMIFENTANIL	9739	6	0.93	0.16
SUFENTANIL BASE	9740	3	0.01	0
TAPENTADOL	9780	8	465	58.13
FENTANYL BASE	9801	367	71.95	0.2

STATE:MARYLAND BUSINESS ACTIVITY:D - TEACHING INSTITUTIONS

DRUG NAME	DRUG CODE	BUYERS	TOTAL GRAMS	AVG GRAMS
MORPHINE	9300	1	0.03	0.03
FENTANYL BASE	9801	1	0.01	0.01

STATE:MARYLAND BUSINESS ACTIVITY:M - MID-LEVEL PRACTITIONERS

DRUG NAME	DRUG CODE	BUYERS	TOTAL GRAMS	AVG GRAMS
PENTOBARBITAL (SCHEDULE 2)	2270	14	21,307.62	1,521.97
CODEINE	9050	36	44.45	1.23
BUPRENORPHINE	9064	5	9.53	1.91
DIHYDROCODEINE	9120	2	0.64	0.32
OXYCODONE	9143	1	0.45	0.45
HYDROCODONE	9193	1	0.3	0.3

STATE:MARYLAND BUSINESS ACTIVITY:N-U NARCOTIC TREATMENT PROGRAMS

DRUG NAME	DRUG CODE	BUYERS	TOTAL GRAMS	AVG GRAMS
BUPRENORPHINE	9064	41	4,705.29	114.76
METHADONE	9250B	81	699,072.88	8,630.53

STATE:MASSACHUSETTS BUSINESS ACTIVITY:A - PHARMACIES

DRUG NAME	DRUG CODE	BUYERS	TOTAL GRAMS	AVG GRAMS
AMPHETAMINE	1100	1,142	682,803.50	597.9
DL-METHAMPHETAMINE RACEMIC BASE	1105B	33	168.38	5.1
D-METHAMPHETAMINE	1105D	4	6.83	1.71
LISDEXAMFETAMINE	1205	1,083	139,220.93	128.55
METHYLPHENIDATE (DL;D;L;ISOMERS)	1724	1,139	555,629.86	487.82
BARBITURIC ACID DERIVIATIVE OR SALT [PER 21C	2100	423	10,755	25.43
BUTALBITAL	2165	359	8,995	25.06
SECOBARBITAL (SCHEDULE 2)	2315	8	192.26	24.03
DRONABINOL IN AN ORAL SOLUTION IN FDA APPR	7365	4	1.2	0.3
CODEINE	9050	1,128	175,363.93	155.46
BUPRENORPHINE	9064	1,113	155,993.82	140.16
DIHYDROCODEINE	9120	2	126.04	63.02
OXYCODONE	9143	1,150	890,076.90	773.98
HYDROMORPHONE	9150	1,089	20,855.92	19.15

HYDROCODONE	9193	1,140	116,077.32	101.82
LEVORPHANOL	9220L	37	80.56	2.18
MEPERIDINE (PETHIDINE)	9230	198	6,080.24	30.71
METHADONE	9250B	972	65,587.78	67.48
MORPHINE	9300	1,098	233,599.19	212.75
OPIUM POWDERED	9639	125	571.32	4.57
OXYMORPHONE	9652	282	7,966.23	28.25
TAPENTADOL	9780	515	75,084	145.79
FENTANYL BASE	9801	1,035	4,472.70	4.32
OPIUM COMBINATION PRODUCT (C-III)	9809	18	6.43	0.36

STATE:MASSACHUSETTS BUSINESS ACTIVITY:B - HOSPITALS

DRUG NAME	DRUG CODE	BUYERS	TOTAL GRAMS	AVG GRAMS
AMPHETAMINE	1100	150	29,055.51	193.7
LISDEXAMFETAMINE	1205	69	3,676.72	53.29
METHYLPHENIDATE (DL;D;L;ISOMERS)	1724	165	29,018.28	175.87
GAMMA HYDROXYBUTYRIC ACID(FDA APPROVED	2012	1	2,674.94	2,674.94
BARBITURIC ACID DERIVIATIVE OR SALT [PER 21C	2100	4	65	16.25
AMOBARBITAL (SCHEDULE 2)	2125	7	20.05	2.86
BUTALBITAL	2165	15	305	20.33
PENTOBARBITAL (SCHEDULE 2)	2270	16	10,487.26	655.45
DRONABINOL IN AN ORAL SOLUTION IN FDA APPR	7365	1	2.55	2.55
COCAINE	9041L	77	784.16	10.18
CODEINE	9050	161	8,682.23	53.93
BUPRENORPHINE	9064	158	8,767.77	55.49
OXYCODONE	9143	202	69,224.93	342.7
HYDROMORPHONE	9150	175	8,513.83	48.65
HYDROCODONE	9193	175	4,182.92	23.9
LEVORPHANOL	9220L	1	0.12	0.12
MEPERIDINE (PETHIDINE)	9230	122	1,984.56	16.27
METHADONE	9250B	156	11,573.04	74.19
MORPHINE	9300	195	28,470.48	146
OPIUM POWDERED	9639	58	227.52	3.92
OXYMORPHONE	9652	17	733.89	43.17
ALFENTANIL	9737	3	3.45	1.15

REMIFENTANIL	9739	67	87.02	1.3
SUFENTANIL BASE	9740	18	0.52	0.03
TAPENTADOL	9780	24	1,992	83
FENTANYL BASE	9801	185	721.87	3.9
OPIUM COMBINATION PRODUCT (C-III)	9809	1	0.38	0.38

STATE:MASSACHUSETTS BUSINESS ACTIVITY:C - PRACTITIONERS

DRUG NAME	DRUG CODE	BUYERS	TOTAL GRAMS	AVG GRAMS
METHYLPHENIDATE (DL;D;L;ISOMERS)	1724	2	2.16	1.08
PENTOBARBITAL (SCHEDULE 2)	2270	187	102,271.15	546.9
COCAINE	9041L	4	31.28	7.82
CODEINE	9050	42	1,178.29	28.05
BUPRENORPHINE	9064	529	59.77	0.11
OXYCODONE	9143	20	35.59	1.78
HYDROMORPHONE	9150	241	104.41	0.43
HYDROCODONE	9193	128	410.98	3.21
MEPERIDINE (PETHIDINE)	9230	37	390.21	10.55
METHADONE	9250B	24	93.02	3.88
MORPHINE	9300	109	128.58	1.18
OPIUM POWDERED	9639	2	4.32	2.16
OXYMORPHONE	9652	10	1.05	0.11
NOROXYMORPHONE	9668	1	3.03	3.03
REMIFENTANIL	9739	20	2.24	0.11
SUFENTANIL BASE	9740	1	0	0
FENTANYL BASE	9801	305	34.45	0.11

STATE:MASSACHUSETTS BUSINESS ACTIVITY:M - MID-LEVEL PRACTITIONERS

DRUG NAME	DRUG CODE	BUYERS	TOTAL GRAMS	AVG GRAMS
CODEINE	9050	5	5.63	1.13
BUPRENORPHINE	9064	1	0.6	0.6

STATE:MASSACHUSETTS BUSINESS ACTIVITY:N-U NARCOTIC TREATMENT PROGRAMS

DRUG NAME	DRUG CODE	BUYERS	TOTAL GRAMS	AVG GRAMS
BUPRENORPHINE	9064	33	491.4	14.89
METHADONE	9250B	74	502,120.81	6,785.42

STATE:MICHIGAN BUSINESS ACTIVITY:A - PHARMACIES

DRUG NAME	DRUG CODE	BUYERS	TOTAL GRAMS	AVG GRAMS
AMPHETAMINE	1100	2,409	943,474.67	391.65
DL-METHAMPHETAMINE RACEMIC BASE	1105B	37	91.62	2.48
LISDEXAMFETAMINE	1205	2,169	233,844.86	107.81
METHYLPHENIDATE (DL;D;L;ISOMERS)	1724	2,319	737,432.11	318
BARBITURIC ACID DERIVIATIVE OR SALT [PER 21C	2100	779	26,750	34.34
BUTALBITAL	2165	1,043	35,215	33.76
SECOBARBITAL (SCHEDULE 2)	2315	1	18.31	18.31
DRONABINOL IN AN ORAL SOLUTION IN FDA APPR	7365	8	2.4	0.3
COCAINE	9041L	11	19.57	1.78
CODEINE	9050	2,472	998,622.91	403.97
BUPRENORPHINE	9064	2,209	105,473.70	47.75
DIHYDROCODEINE	9120	5	7.48	1.5
OXYCODONE	9143	2,441	1,127,113.21	461.74
HYDROMORPHONE	9150	1,967	29,477.48	14.99
HYDROCODONE	9193	2,500	1,517,211.61	606.88
LEVORPHANOL	9220L	4	0.7	0.17
MEPERIDINE (PETHIDINE)	9230	262	6,823.30	26.04
METHADONE	9250B	1,911	137,989.55	72.21
MORPHINE	9300	2,325	694,249.52	298.6
OPIUM TINCTURE (LAUDANUM)	9630	6	30.72	5.12
OPIUM POWDERED	9639	156	487.08	3.12
OXYMORPHONE	9652	1,198	75,289.05	62.85
NOROXYMORPHONE	9668	2	3.12	1.56
REMIFENTANIL	9739	4	2.59	0.65
SUFENTANIL BASE	9740	3	0.72	0.24
TAPENTADOL	9780	1,055	85,282	80.84
FENTANYL BASE	9801	2,205	11,127.47	5.05
OPIUM COMBINATION PRODUCT (C-III)	9809	27	18.92	0.7

STATISTICAL SUMMARY FOR RETAIL DRUG PURCHASES BY GRAMS WT

STATE:MICHIGAN BUSINESS ACTIVITY:B - HOSPITALS

DRUG NAME	DRUG CODE	BUYERS	TOTAL GRAMS	AVG GRAMS
AMPHETAMINE	1100	107	18,425.14	172.2
LISDEXAMFETAMINE	1205	58	3,996.41	68.9
METHYLPHENIDATE (DL;D;L;ISOMERS)	1724	159	17,443.59	109.71
GAMMA HYDROXYBUTYRIC ACID(FDA APPROVED	2012	1	1,337.47	1,337.47
BARBITURIC ACID DERIVIATIVE OR SALT [PER 21C	2100	8	160	20
AMOBARBITAL (SCHEDULE 2)	2125	8	18.68	2.34
BUTALBITAL	2165	23	255	11.09
PENTOBARBITAL (SCHEDULE 2)	2270	26	2,524.12	97.08
COCAINE	9041L	109	1,347.66	12.36
CODEINE	9050	185	23,026.56	124.47
BUPRENORPHINE	9064	125	2,660.55	21.28
OXYCODONE	9143	193	60,934.80	315.72
HYDROMORPHONE	9150	183	6,483.74	35.43
HYDROCODONE	9193	198	76,031.56	384
LEVORPHANOL	9220L	1	0.35	0.35
MEPERIDINE (PETHIDINE)	9230	138	2,873.23	20.82
METHADONE	9250B	151	9,337.97	61.84
MORPHINE	9300	194	58,655.75	302.35
OPIUM POWDERED	9639	105	1,046.52	9.97
OXYMORPHONE	9652	29	405.15	13.97
ALFENTANIL	9737	12	2.4	0.2
REMIFENTANIL	9739	54	37.01	0.69
SUFENTANIL BASE	9740	43	1.47	0.03
TAPENTADOL	9780	28	1,357	48.46
FENTANYL BASE	9801	188	1,165.48	6.2
OPIUM COMBINATION PRODUCT (C-III)	9809	5	3.78	0.76

STATE:MICHIGAN BUSINESS ACTIVITY:C - PRACTITIONERS

DRUG NAME	DRUG CODE	BUYERS	TOTAL GRAMS	AVG GRAMS
AMPHETAMINE	1100	2	41.51	20.75

LISDEXAMFETAMINE	1205	2	11.33	5.67
BUTALBITAL	2165	1	50	50
PENTOBARBITAL (SCHEDULE 2)	2270	448	342,339.26	764.15
COCAINE	9041L	29	140.63	4.85
CODEINE	9050	173	2,643.64	15.28
BUPRENORPHINE	9064	688	378.79	0.55
DIHYDROCODEINE	9120	1	33.01	33.01
OXYCODONE	9143	40	781.74	19.54
HYDROMORPHONE	9150	414	188.5	0.46
HYDROCODONE	9193	283	1,916.02	6.77
MEPERIDINE (PETHIDINE)	9230	117	1,230.78	10.52
METHADONE	9250B	8	16.73	2.09
MORPHINE	9300	305	661.53	2.17
OPIUM POWDERED	9639	7	8.64	1.23
OXYMORPHONE	9652	4	1.79	0.45
NOROXYMORPHONE	9668	1	1.17	1.17
ALFENTANIL	9737	7	2.08	0.3
REMIFENTANIL	9739	1	0.03	0.03
SUFENTANIL BASE	9740	3	0.03	0.01
FENTANYL BASE	9801	527	79.7	0.15

STATE:MICHIGAN BUSINESS ACTIVITY:D - TEACHING INSTITUTIONS

DRUG NAME	DRUG CODE	BUYERS	TOTAL GRAMS	AVG GRAMS
PENTOBARBITAL (SCHEDULE 2)	2270	1	13,329.23	13,329.23
CODEINE	9050	1	745.17	745.17
BUPRENORPHINE	9064	1	1.3	1.3
HYDROMORPHONE	9150	1	2.32	2.32
HYDROCODONE	9193	1	25	25
MEPERIDINE (PETHIDINE)	9230	1	1.09	1.09
METHADONE	9250B	1	31.3	31.3
MORPHINE	9300	1	3.38	3.38
SUFENTANIL BASE	9740	1	0.02	0.02
FENTANYL BASE	9801	2	3.33	1.67

STATE:MICHIGAN BUSINESS ACTIVITY:M - MID-LEVEL PRACTITIONERS

DRUG NAME	DRUG CODE	BUYERS	TOTAL GRAMS	AVG GRAMS
PENTOBARBITAL (SCHEDULE 2)	2270	25	26,748.67	1,069.95
CODEINE	9050	10	236.55	23.66
DIHYDROCODEINE	9120	2	0.37	0.19

STATE:MICHIGAN BUSINESS ACTIVITY:N-U NARCOTIC TREATMENT PROGRAMS

DRUG NAME	DRUG CODE	BUYERS	TOTAL GRAMS	AVG GRAMS
BUPRENORPHINE	9064	6	130.26	21.71
METHADONE	9250B	44	386,636.60	8,787.20

STATE:MINNESOTA BUSINESS ACTIVITY:A - PHARMACIES

DRUG NAME	DRUG CODE	BUYERS	TOTAL GRAMS	AVG GRAMS
AMPHETAMINE	1100	1,026	425,084.45	414.31
DL-METHAMPHETAMINE RACEMIC BASE	1105B	47	264.02	5.62
D-METHAMPHETAMINE	1105D	3	10.04	3.35
LISDEXAMFETAMINE	1205	1,010	125,218.77	123.98
METHYLPHENIDATE (DL;D;L;ISOMERS)	1724	1,030	395,570.50	384.05
BARBITURIC ACID DERIVIATIVE OR SALT [PER 21C	2100	221	5,285	23.91
BUTALBITAL	2165	280	6,670	23.82
PENTOBARBITAL (SCHEDULE 2)	2270	1	802.94	802.94
SECOBARBITAL (SCHEDULE 2)	2315	1	18.31	18.31
DRONABINOL IN AN ORAL SOLUTION IN FDA APPR	7365	2	2.55	1.28
NABILONE	7379	2	0.1	0.05
COCAINE	9041L	3	19.39	6.46
CODEINE	9050	1,025	142,618.66	139.14
BUPRENORPHINE	9064	890	27,024.13	30.36
DIHYDROCODEINE	9120	2	7.48	3.74
OXYCODONE	9143	1,041	538,155.51	516.96
HYDROMORPHONE	9150	995	18,867.01	18.96
HYDROCODONE	9193	1,046	169,756.86	162.29
LEVORPHANOL	9220L	4	2.79	0.7

MEPERIDINE (PETHIDINE)	9230	131	1,562.27	11.93
METHADONE	9250B	863	30,269	35.07
MORPHINE	9300	1,016	167,092.25	164.46
OPIUM POWDERED	9639	175	597.96	3.42
OXYMORPHONE	9652	196	1,678.58	8.56
NOROXYMORPHONE	9668	1	0.5	0.5
ALFENTANIL	9737	2	0.98	0.49
REMIFENTANIL	9739	1	0.02	0.02
SUFENTANIL BASE	9740	1	0.09	0.09
TAPENTADOL	9780	340	17,409	51.2
FENTANYL BASE	9801	987	3,563.36	3.61
OPIUM COMBINATION PRODUCT (C-III)	9809	17	8.51	0.5

STATE:MINNESOTA BUSINESS ACTIVITY:B - HOSPITALS

DRUG NAME	DRUG CODE	BUYERS	TOTAL GRAMS	AVG GRAMS
AMPHETAMINE	1100	81	6,557.53	80.96
DL-METHAMPHETAMINE RACEMIC BASE	1105B	3	25.32	8.44
D-METHAMPHETAMINE	1105D	2	42.98	21.49
LISDEXAMFETAMINE	1205	41	1,460.86	35.63
METHYLPHENIDATE (DL;D;L;ISOMERS)	1724	97	7,770.28	80.11
BARBITURIC ACID DERIVIATIVE OR SALT [PER 21C	2100	4	30	7.5
AMOBARBITAL (SCHEDULE 2)	2125	5	23.24	4.65
BUTALBITAL	2165	5	35	7
PENTOBARBITAL (SCHEDULE 2)	2270	14	13,997.74	999.84
DRONABINOL IN AN ORAL SOLUTION IN FDA APPR	7365	1	0.3	0.3
COCAINE	9041L	87	670.92	7.71
CODEINE	9050	138	5,381.46	39
BUPRENORPHINE	9064	74	1,191.12	16.1
OXYCODONE	9143	149	32,200.38	216.11
HYDROMORPHONE	9150	150	4,765.02	31.77
HYDROCODONE	9193	148	12,228.16	82.62
MEPERIDINE (PETHIDINE)	9230	119	911.7	7.66
METHADONE	9250B	97	3,250.62	33.51
MORPHINE	9300	156	18,164.54	116.44
OPIUM POWDERED	9639	82	923.04	11.26

OXYMORPHONE	9652	9	19.46	2.16
ALFENTANIL	9737	21	10.98	0.52
REMIFENTANIL	9739	24	31.85	1.33
SUFENTANIL BASE	9740	23	0.68	0.03
TAPENTADOL	9780	11	192.5	17.5
FENTANYL BASE	9801	151	501.92	3.32
OPIUM COMBINATION PRODUCT (C-III)	9809	3	0.57	0.19

STATE:MINNESOTA BUSINESS ACTIVITY:C - PRACTITIONERS

DRUG NAME	DRUG CODE	BUYERS	TOTAL GRAMS	AVG GRAMS
AMPHETAMINE	1100	4	9.51	2.38
PENTOBARBITAL (SCHEDULE 2)	2270	179	130,079.10	726.7
COCAINE	9041L	22	165.2	7.51
CODEINE	9050	42	1,175.30	27.98
BUPRENORPHINE	9064	521	34.61	0.07
OXYCODONE	9143	62	277.75	4.48
HYDROMORPHONE	9150	360	159.33	0.44
HYDROCODONE	9193	251	596.46	2.38
MEPERIDINE (PETHIDINE)	9230	57	259.92	4.56
METHADONE	9250B	2	3.4	1.7
MORPHINE	9300	194	215.45	1.11
OPIUM POWDERED	9639	10	14.76	1.48
OXYMORPHONE	9652	2	0.06	0.03
ALFENTANIL	9737	7	1.95	0.28
REMIFENTANIL	9739	7	0.83	0.12
SUFENTANIL BASE	9740	2	0	0
FENTANYL BASE	9801	349	50.38	0.14

STATE:MINNESOTA BUSINESS ACTIVITY:M - MID-LEVEL PRACTITIONERS

DRUG NAME	DRUG CODE	BUYERS	TOTAL GRAMS	AVG GRAMS
CODEINE	9050	4	23.19	5.8
DIHYDROCODEINE	9120	1	2.35	2.35
HYDROCODONE	9193	1	0.27	0.27

FENTANYL BASE	9801	2	0.01	0.01

STATE:MINNESOTA BUSINESS ACTIVITY:N-U NARCOTIC TREATMENT PROGRAMS

DRUG NAME	DRUG CODE	BUYERS	TOTAL GRAMS	AVG GRAMS
BUPRENORPHINE	9064	14	1,964.87	140.35
METHADONE	9250B	18	174,288.15	9,682.68

STATE:MISSISSIPPI BUSINESS ACTIVITY:A - PHARMACIES

DRUG NAME	DRUG CODE	BUYERS	TOTAL GRAMS	AVG GRAMS
AMPHETAMINE	1100	773	239,922.92	310.38
DL-METHAMPHETAMINE RACEMIC BASE	1105B	7	32.95	4.71
D-METHAMPHETAMINE	1105D	1	2.41	2.41
LISDEXAMFETAMINE	1205	750	152,360.57	203.15
METHYLPHENIDATE (DL;D;L;ISOMERS)	1724	763	166,639.05	218.4
BARBITURIC ACID DERIVIATIVE OR SALT [PER 21C	2100	171	3,995	23.36
BUTALBITAL	2165	255	8,435	33.08
DRONABINOL IN AN ORAL SOLUTION IN FDA APPR	7365	2	0.75	0.38
COCAINE	9041L	4	68.39	17.1
CODEINE	9050	769	104,788.51	136.27
BUPRENORPHINE	9064	709	38,754.40	54.66
DIHYDROCODEINE	9120	297	1,529.61	5.15
OXYCODONE	9143	780	403,226.73	516.96
HYDROMORPHONE	9150	626	21,501.04	34.35
HYDROCODONE	9193	789	441,908.96	560.09
LEVORPHANOL	9220L	1	0.23	0.23
MEPERIDINE (PETHIDINE)	9230	510	10,989.83	21.55
METHADONE	9250B	474	15,377.06	32.44
MORPHINE	9300	742	147,472.65	198.75
OPIUM POWDERED	9639	33	36.36	1.1
OXYMORPHONE	9652	416	12,163.29	29.24
SUFENTANIL BASE	9740	1	51.75	51.75
TAPENTADOL	9780	515	46,908.50	91.08
FENTANYL BASE	9801	755	4,527.85	6

OPIUM COMBINATION PRODUCT (C-III)	9809	22	11.73	0.53

STATE:MISSISSIPPI BUSINESS ACTIVITY:B - HOSPITALS

DRUG NAME	DRUG CODE	BUYERS	TOTAL GRAMS	AVG GRAMS
AMPHETAMINE	1100	54	6,114.73	113.24
LISDEXAMFETAMINE	1205	34	3,560.52	104.72
METHYLPHENIDATE (DL;D;L;ISOMERS)	1724	65	4,680.68	72.01
BARBITURIC ACID DERIVIATIVE OR SALT [PER 21C	2100	2	25	12.5
AMOBARBITAL (SCHEDULE 2)	2125	1	4.56	4.56
BUTALBITAL	2165	12	110	9.17
PENTOBARBITAL (SCHEDULE 2)	2270	3	185.01	61.67
COCAINE	9041L	26	244.73	9.41
CODEINE	9050	107	4,698.42	43.91
BUPRENORPHINE	9064	64	894.5	13.98
DIHYDROCODEINE	9120	7	32.04	4.58
OXYCODONE	9143	134	15,299.78	114.18
HYDROMORPHONE	9150	124	1,887.45	15.22
HYDROCODONE	9193	146	24,535.44	168.05
MEPERIDINE (PETHIDINE)	9230	118	5,719.35	48.47
METHADONE	9250B	57	766.32	13.44
MORPHINE	9300	140	12,259.86	87.57
OPIUM POWDERED	9639	30	96.84	3.23
OXYMORPHONE	9652	13	84.99	6.54
ALFENTANIL	9737	9	0.43	0.05
REMIFENTANIL	9739	7	1.59	0.23
SUFENTANIL BASE	9740	18	0.35	0.02
TAPENTADOL	9780	15	1,377	91.8
FENTANYL BASE	9801	139	385.67	2.77

STATE:MISSISSIPPI BUSINESS ACTIVITY:C - PRACTITIONERS

DRUG NAME	DRUG CODE	BUYERS	TOTAL GRAMS	AVG GRAMS
PENTOBARBITAL (SCHEDULE 2)	2270	36	32,338.39	898.29
COCAINE	9041L	5	25.86	5.17

CODEINE	9050	15	116.91	7.79
BUPRENORPHINE	9064	111	5.21	0.05
DIHYDROCODEINE	9120	45	30.5	0.68
OXYCODONE	9143	10	728.05	72.81
HYDROMORPHONE	9150	33	15.1	0.46
HYDROCODONE	9193	41	102.82	2.51
MEPERIDINE (PETHIDINE)	9230	40	605.37	15.13
MORPHINE	9300	54	78.81	1.46
OPIUM POWDERED	9639	1	1.44	1.44
NOROXYMORPHONE	9668	1	6.19	6.19
REMIFENTANIL	9739	4	0.19	0.05
SUFENTANIL BASE	9740	3	0.03	0.01
FENTANYL BASE	9801	105	9.12	0.09

STATE:MISSISSIPPI BUSINESS ACTIVITY:D - TEACHING INSTITUTIONS

DRUG NAME	DRUG CODE	BUYERS	TOTAL GRAMS	AVG GRAMS
BUPRENORPHINE	9064	1	0	0
HYDROMORPHONE	9150	1	0.11	0.11

STATE:MISSISSIPPI BUSINESS ACTIVITY:M - MID-LEVEL PRACTITIONERS

DRUG NAME	DRUG CODE	BUYERS	TOTAL GRAMS	AVG GRAMS
CODEINE	9050	9	9.53	1.06
DIHYDROCODEINE	9120	21	13.83	0.66

STATE:MISSISSIPPI BUSINESS ACTIVITY:N-U NARCOTIC TREATMENT PROGRAMS

DRUG NAME	DRUG CODE	BUYERS	TOTAL GRAMS	AVG GRAMS
BUPRENORPHINE	9064	3	18	6
METHADONE	9250B	5	10,500.26	2,100.05

STATE:MISSOURI BUSINESS ACTIVITY:A - PHARMACIES

DRUG NAME	DRUG CODE	BUYERS	TOTAL GRAMS	AVG GRAMS
AMPHETAMINE	1100	1,315	526,983.86	400.75
DL-METHAMPHETAMINE RACEMIC BASE	1105B	28	190.08	6.79
D-METHAMPHETAMINE	1105D	3	32.14	10.71
LISDEXAMFETAMINE	1205	1,281	263,101.40	205.39
METHYLPHENIDATE (DL;D;L;ISOMERS)	1724	1,318	515,769.49	391.33
GAMMA HYDROXYBUTYRIC ACID(FDA APPROVED 30,411,958.46	2012	1	30,411,958.46	
BARBITURIC ACID DERIVIATIVE OR SALT [PER 21C	2100	378	15,805	41.81
BUTALBITAL	2165	530	23,555	44.44
SECOBARBITAL (SCHEDULE 2)	2315	4	100.71	25.18
DRONABINOL IN AN ORAL SOLUTION IN FDA APPR	7365	8	8.55	1.07
NABILONE	7379	4	0.55	0.14
COCAINE	9041L	2	9.14	4.57
CODEINE	9050	1,323	263,296.65	199.01
BUPRENORPHINE	9064	1,234	35,576.31	28.83
DIHYDROCODEINE	9120	12	65.16	5.43
OXYCODONE	9143	1,333	1,129,075.33	847.02
HYDROMORPHONE	9150	1,191	22,113.99	18.57
HYDROCODONE	9193	1,337	678,034.38	507.13
LEVORPHANOL	9220L	36	86.94	2.42
MEPERIDINE (PETHIDINE)	9230	468	10,250.47	21.9
METHADONE	9250B	1,108	50,516.25	45.59
MORPHINE	9300	1,313	351,780.35	267.92
OPIUM POWDERED	9639	101	322.56	3.19
OXYMORPHONE	9652	796	17,158.29	21.56
REMIFENTANIL	9739	1	0.04	0.04
TAPENTADOL	9780	907	95,896	105.73
FENTANYL BASE	9801	1,294	7,821.11	6.04
OPIUM COMBINATION PRODUCT (C-III)	9809	26	27.81	1.07

STATE:MISSOURI BUSINESS ACTIVITY:B - HOSPITALS

DRUG NAME	DRUG CODE	BUYERS	TOTAL GRAMS	AVG GRAMS
AMPHETAMINE	1100	95	9,611.44	101.17

LISDEXAMFETAMINE	1205	50	3,111.33	62.23
METHYLPHENIDATE (DL;D;L;ISOMERS)	1724	127	10,605.48	83.51
BARBITURIC ACID DERIVIATIVE OR SALT [PER 21C	2100	2	15	7.5
AMOBARBITAL (SCHEDULE 2)	2125	4	6.84	1.71
BUTALBITAL	2165	6	195	32.5
PENTOBARBITAL (SCHEDULE 2)	2270	17	1,238.14	72.83
COCAINE	9041L	107	650.28	6.08
CODEINE	9050	156	7,419.22	47.56
BUPRENORPHINE	9064	91	1,138.33	12.51
OXYCODONE	9143	221	55,217.48	249.85
HYDROMORPHONE	9150	182	3,445.01	18.93
HYDROCODONE	9193	234	28,309.72	120.98
MEPERIDINE (PETHIDINE)	9230	168	4,671.96	27.81
METHADONE	9250B	114	3,886.24	34.09
MORPHINE	9300	231	33,689.82	145.84
OPIUM POWDERED	9639	86	351.72	4.09
OXYMORPHONE	9652	18	68.91	3.83
ALFENTANIL	9737	15	4.33	0.29
REMIFENTANIL	9739	43	21.53	0.5
SUFENTANIL BASE	9740	45	1.22	0.03
TAPENTADOL	9780	30	1,670	55.67
FENTANYL BASE	9801	252	1,243.84	4.94
OPIUM COMBINATION PRODUCT (C-III)	9809	2	0.76	0.38

STATE:MISSOURI BUSINESS ACTIVITY:C - PRACTITIONERS

DRUG NAME	DRUG CODE	BUYERS	TOTAL GRAMS	AVG GRAMS
AMPHETAMINE	1100	2	1.72	0.86
PENTOBARBITAL (SCHEDULE 2)	2270	199	150,903.50	758.31
COCAINE	9041L	1	0.14	0.14
CODEINE	9050	89	2,983.04	33.52
BUPRENORPHINE	9064	445	47.65	0.11
DIHYDROCODEINE	9120	2	119.63	59.82
OXYCODONE	9143	12	376.27	31.36
HYDROMORPHONE	9150	164	234.81	1.43
HYDROCODONE	9193	211	1,597.36	7.57

MEPERIDINE (PETHIDINE)	9230	29	287.18	9.9
METHADONE	9250B	1	1.79	1.79
MORPHINE	9300	161	440.26	2.73
OXYMORPHONE	9652	2	0.9	0.45
NOROXYMORPHONE	9668	5	37.2	7.44
REMIFENTANIL	9739	1	0.2	0.2
SUFENTANIL BASE	9740	3	0.04	0.01
FENTANYL BASE	9801	174	17.84	0.1

STATE:MISSOURI BUSINESS ACTIVITY:D - TEACHING INSTITUTIONS

DRUG NAME	DRUG CODE	BUYERS	TOTAL GRAMS	AVG GRAMS
PENTOBARBITAL (SCHEDULE 2)	2270	1	8,886.15	8,886.15
CODEINE	9050	1	99.45	99.45
BUPRENORPHINE	9064	2	0.43	0.22
HYDROMORPHONE	9150	2	8.24	4.12
HYDROCODONE	9193	2	7.46	3.73
MORPHINE	9300	2	5.85	2.93
FENTANYL BASE	9801	4	1.64	0.41

STATE:MISSOURI BUSINESS ACTIVITY:M - MID-LEVEL PRACTITIONERS

DRUG NAME	DRUG CODE	BUYERS	TOTAL GRAMS	AVG GRAMS
HYDROMORPHONE	9150	3	0.08	0.03
MEPERIDINE (PETHIDINE)	9230	1	0.87	0.87
MORPHINE	9300	39	17.28	0.44
FENTANYL BASE	9801	51	0.65	0.01

STATE:MISSOURI BUSINESS ACTIVITY:N-U NARCOTIC TREATMENT PROGRAMS

DRUG NAME	DRUG CODE	BUYERS	TOTAL GRAMS	AVG GRAMS
BUPRENORPHINE	9064	15	1,463.95	97.6
METHADONE	9250B	16	111,576.85	6,973.55

STATISTICAL SUMMARY FOR RETAIL DRUG PURCHASES BY GRAMS WT

STATE:MONTANA BUSINESS ACTIVITY:A - PHARMACIES

DRUG NAME	DRUG CODE	BUYERS	TOTAL GRAMS	AVG GRAMS
AMPHETAMINE	1100	234	52,028.33	222.34
DL-METHAMPHETAMINE RACEMIC BASE	1105B	4	2.81	0.7
LISDEXAMFETAMINE	1205	231	21,587.41	93.45
METHYLPHENIDATE (DL;D;L;ISOMERS)	1724	236	64,115.64	271.68
BARBITURIC ACID DERIVIATIVE OR SALT [PER 21C	2100	53	1,935	36.51
BUTALBITAL	2165	82	2,990	36.46
COCAINE	9041L	4	1.29	0.32
CODEINE	9050	234	35,922.31	153.51
BUPRENORPHINE	9064	222	8,319.59	37.48
DIHYDROCODEINE	9120	1	1.07	1.07
OXYCODONE	9143	238	137,599.01	578.15
HYDROMORPHONE	9150	233	5,451.12	23.4
HYDROCODONE	9193	240	87,270.44	363.63
MEPERIDINE (PETHIDINE)	9230	101	1,757.16	17.4
METHADONE	9250B	202	7,636.39	37.8
MORPHINE	9300	238	57,885.39	243.22
OPIUM POWDERED	9639	18	32.4	1.8
OXYMORPHONE	9652	90	1,123.38	12.48
TAPENTADOL	9780	120	7,185.50	59.88
FENTANYL BASE	9801	238	1,061.84	4.46
OPIUM COMBINATION PRODUCT (C-III)	9809	6	3.41	0.57

STATE:MONTANA BUSINESS ACTIVITY:B - HOSPITALS

DRUG NAME	DRUG CODE	BUYERS	TOTAL GRAMS	AVG GRAMS
AMPHETAMINE	1100	38	3,968.30	104.43
LISDEXAMFETAMINE	1205	25	1,322.69	52.91
METHYLPHENIDATE (DL;D;L;ISOMERS)	1724	45	5,860.21	130.23
BARBITURIC ACID DERIVIATIVE OR SALT [PER 21C	2100	3	60	20
BUTALBITAL	2165	5	90	18
PENTOBARBITAL (SCHEDULE 2)	2270	2	3.65	1.82
DRONABINOL IN AN ORAL SOLUTION IN FDA APPR	7365	1	0.3	0.3

COCAINE	9041L	27	209.81	7.77
CODEINE	9050	60	4,467.96	74.47
BUPRENORPHINE	9064	27	749.11	27.74
OXYCODONE	9143	81	14,746.38	182.05
HYDROMORPHONE	9150	68	1,083.42	15.93
HYDROCODONE	9193	83	15,433.04	185.94
MEPERIDINE (PETHIDINE)	9230	59	859.76	14.57
METHADONE	9250B	34	1,005.31	29.57
MORPHINE	9300	85	11,668.02	137.27
OPIUM POWDERED	9639	24	171.72	7.16
OXYMORPHONE	9652	2	9.37	4.68
ALFENTANIL	9737	12	1.25	0.1
REMIFENTANIL	9739	15	6.99	0.47
SUFENTANIL BASE	9740	18	0.16	0.01
TAPENTADOL	9780	9	249	27.67
FENTANYL BASE	9801	81	249.14	3.08

STATE:MONTANA BUSINESS ACTIVITY:C - PRACTITIONERS

DRUG NAME	DRUG CODE	BUYERS	TOTAL GRAMS	AVG GRAMS
PENTOBARBITAL (SCHEDULE 2)	2270	88	72,254.79	821.08
COCAINE	9041L	1	0.5	0.5
CODEINE	9050	13	190.69	14.67
BUPRENORPHINE	9064	139	5.58	0.04
OXYCODONE	9143	4	5.83	1.46
HYDROMORPHONE	9150	80	29.91	0.37
HYDROCODONE	9193	75	175	2.33
MEPERIDINE (PETHIDINE)	9230	8	69.39	8.67
METHADONE	9250B	1	8.05	8.05
MORPHINE	9300	71	88.25	1.24
OXYMORPHONE	9652	2	0.03	0.01
ALFENTANIL	9737	2	0.05	0.03
SUFENTANIL BASE	9740	1	0.01	0.01
FENTANYL BASE	9801	78	7.1	0.09

STATE:MONTANA BUSINESS ACTIVITY:D - TEACHING INSTITUTIONS

DRUG NAME	DRUG CODE	BUYERS	TOTAL GRAMS	AVG GRAMS
BUPRENORPHINE	9064	2	0.02	0.01

STATE:MONTANA BUSINESS ACTIVITY:M - MID-LEVEL PRACTITIONERS

DRUG NAME	DRUG CODE	BUYERS	TOTAL GRAMS	AVG GRAMS
PENTOBARBITAL (SCHEDULE 2)	2270	4	1,867	466.75
MORPHINE	9300	1	0.3	0.3
FENTANYL BASE	9801	2	0.01	0

STATE:MONTANA BUSINESS ACTIVITY:N-U NARCOTIC TREATMENT PROGRAMS

DRUG NAME	DRUG CODE	BUYERS	TOTAL GRAMS	AVG GRAMS
BUPRENORPHINE	9064	4	577.87	144.47
METHADONE	9250B	4	20,571.20	5,142.80

STATE:NEBRASKA BUSINESS ACTIVITY:A - PHARMACIES

DRUG NAME	DRUG CODE	BUYERS	TOTAL GRAMS	AVG GRAMS
AMPHETAMINE	1100	437	99,599.28	227.92
DL-METHAMPHETAMINE RACEMIC BASE	1105B	12	32.95	2.75
D-METHAMPHETAMINE	1105D	1	0.8	0.8
LISDEXAMFETAMINE	1205	428	65,132.21	152.18
METHYLPHENIDATE (DL;D;L;ISOMERS)	1724	447	120,734.10	270.1
BARBITURIC ACID DERIVIATIVE OR SALT [PER 21C	2100	88	2,970	33.75
BUTALBITAL	2165	143	4,430	30.98
NABILONE	7379	2	0.3	0.15
CODEINE	9050	439	43,284.92	98.6
BUPRENORPHINE	9064	375	4,950.55	13.2
DIHYDROCODEINE	9120	5	9.61	1.92
OXYCODONE	9143	450	155,885.36	346.41
HYDROMORPHONE	9150	404	4,300.13	10.64

HYDROCODONE	9193	455	121,696.44	267.46
LEVORPHANOL	9220L	22	23.45	1.07
MEPERIDINE (PETHIDINE)	9230	108	1,573.17	14.57
METHADONE	9250B	354	11,368.81	32.12
MORPHINE	9300	449	65,820.14	146.59
OPIUM POWDERED	9639	52	89.64	1.72
OXYMORPHONE	9652	238	4,731.21	19.88
ALFENTANIL	9737	2	0.3	0.15
SUFENTANIL BASE	9740	1	0	0
TAPENTADOL	9780	333	37,786.50	113.47
FENTANYL BASE	9801	432	1,877.69	4.35
OPIUM COMBINATION PRODUCT (C-III)	9809	7	3.03	0.43

STATE:NEBRASKA BUSINESS ACTIVITY:B - HOSPITALS

DRUG NAME	DRUG CODE	BUYERS	TOTAL GRAMS	AVG GRAMS
AMPHETAMINE	1100	37	3,715.76	100.43
LISDEXAMFETAMINE	1205	26	622.61	23.95
METHYLPHENIDATE (DL;D;L;ISOMERS)	1724	59	4,405.40	74.67
AMOBARBITAL (SCHEDULE 2)	2125	2	3.65	1.82
BUTALBITAL	2165	1	5	5
PENTOBARBITAL (SCHEDULE 2)	2270	3	349.07	116.36
COCAINE	9041L	48	117.03	2.44
CODEINE	9050	76	2,646.88	34.83
BUPRENORPHINE	9064	43	325.15	7.56
DIHYDROCODEINE	9120	1	1.07	1.07
OXYCODONE	9143	118	10,097.63	85.57
HYDROMORPHONE	9150	105	830.31	7.91
HYDROCODONE	9193	128	8,021.33	62.67
MEPERIDINE (PETHIDINE)	9230	115	1,106.67	9.62
METHADONE	9250B	53	1,316.05	24.83
MORPHINE	9300	128	9,734.93	76.05
OPIUM POWDERED	9639	55	297	5.4
OXYMORPHONE	9652	11	36.13	3.28
ALFENTANIL	9737	21	3.01	0.14
REMIFENTANIL	9739	20	4.29	0.21

SUFENTANIL BASE	9740	26	0.4	0.02
TAPENTADOL	9780	37	943.5	25.5
FENTANYL BASE	9801	132	238.88	1.81

STATE:NEBRASKA BUSINESS ACTIVITY:C - PRACTITIONERS

DRUG NAME	DRUG CODE	BUYERS	TOTAL GRAMS	AVG GRAMS
AMPHETAMINE	1100	1	1.25	1.25
LISDEXAMFETAMINE	1205	1	11.01	11.01
METHYLPHENIDATE (DL;D;L;ISOMERS)	1724	1	0.86	0.86
PENTOBARBITAL (SCHEDULE 2)	2270	120	82,118.87	684.32
COCAINE	9041L	1	2.14	2.14
CODEINE	9050	34	983.7	28.93
BUPRENORPHINE	9064	153	8.91	0.06
DIHYDROCODEINE	9120	1	1.28	1.28
OXYCODONE	9143	8	265.8	33.22
HYDROMORPHONE	9150	85	75.56	0.89
HYDROCODONE	9193	73	253.67	3.47
LEVORPHANOL	9220L	1	0.35	0.35
MEPERIDINE (PETHIDINE)	9230	23	105.12	4.57
METHADONE	9250B	2	5.28	2.64
MORPHINE	9300	45	130.51	2.9
OPIUM POWDERED	9639	2	7.2	3.6
ALFENTANIL	9737	2	0.25	0.13
SUFENTANIL BASE	9740	1	0	0
TAPENTADOL	9780	2	90	45
FENTANYL BASE	9801	88	11.93	0.14

STATE:NEBRASKA BUSINESS ACTIVITY:D - TEACHING INSTITUTIONS

DRUG NAME	DRUG CODE	BUYERS	TOTAL GRAMS	AVG GRAMS
BUPRENORPHINE	9064	1	0	0

STATE:NEBRASKA BUSINESS ACTIVITY:M - MID-LEVEL PRACTITIONERS

DRUG NAME	DRUG CODE	BUYERS	TOTAL GRAMS	AVG GRAMS
CODEINE	9050	14	49.77	3.55
HYDROMORPHONE	9150	4	0.76	0.19
MORPHINE	9300	23	7.52	0.33
FENTANYL BASE	9801	30	0.51	0.02

STATE:NEBRASKA BUSINESS ACTIVITY:N-U NARCOTIC TREATMENT PROGRAMS

DRUG NAME	DRUG CODE	BUYERS	TOTAL GRAMS	AVG GRAMS
BUPRENORPHINE	9064	1	24.24	24.24
METHADONE	9250B	3	20,034.56	6,678.19

STATE:NEVADA BUSINESS ACTIVITY:A - PHARMACIES

DRUG NAME	DRUG CODE	BUYERS	TOTAL GRAMS	AVG GRAMS
AMPHETAMINE	1100	463	100,986.79	218.11
DL-METHAMPHETAMINE RACEMIC BASE	1105B	28	97.65	3.49
D-METHAMPHETAMINE	1105D	1	0.4	0.4
LISDEXAMFETAMINE	1205	423	29,670.98	70.14
METHYLPHENIDATE (DL;D;L;ISOMERS)	1724	444	54,626.24	123.03
BARBITURIC ACID DERIVIATIVE OR SALT [PER 21C	2100	243	18,270	75.19
BUTALBITAL	2165	256	14,230	55.59
SECOBARBITAL (SCHEDULE 2)	2315	1	9.16	9.16
DRONABINOL IN AN ORAL SOLUTION IN FDA APPR	7365	3	11.55	3.85
COCAINE	9041L	2	14.28	7.14
CODEINE	9050	466	102,752.54	220.5
BUPRENORPHINE	9064	440	12,534.87	28.49
DIHYDROCODEINE	9120	6	74.77	12.46
OXYCODONE	9143	476	670,863.26	1,409.38
HYDROMORPHONE	9150	444	9,415.59	21.21
HYDROCODONE	9193	477	350,438.22	734.67
LEVORPHANOL	9220L	13	14.16	1.09
MEPERIDINE (PETHIDINE)	9230	161	2,866.80	17.81
METHADONE	9250B	448	42,900.07	95.76

MORPHINE	9300	467	208,424.09	446.3
OPIUM POWDERED	9639	24	30.24	1.26
OXYMORPHONE	9652	386	15,788.61	40.9
ALFENTANIL	9737	2	0.07	0.03
SUFENTANIL BASE	9740	1	0.01	0.01
TAPENTADOL	9780	350	47,776.50	136.5
FENTANYL BASE	9801	449	1,898.68	4.23
OPIUM COMBINATION PRODUCT (C-III)	9809	14	4.16	0.3

STATE:NEVADA BUSINESS ACTIVITY:B - HOSPITALS

DRUG NAME	DRUG CODE	BUYERS	TOTAL GRAMS	AVG GRAMS
AMPHETAMINE	1100	35	3,135.11	89.57
LISDEXAMFETAMINE	1205	12	601.8	50.15
METHYLPHENIDATE (DL;D;L;ISOMERS)	1724	50	4,397	87.94
BARBITURIC ACID DERIVIATIVE OR SALT [PER 21C	2100	1	5	5
BUTALBITAL	2165	7	185	26.43
PENTOBARBITAL (SCHEDULE 2)	2270	6	437.02	72.84
COCAINE	9041L	31	165.53	5.34
CODEINE	9050	53	3,778.36	71.29
BUPRENORPHINE	9064	30	391.4	13.05
OXYCODONE	9143	86	41,851.91	486.65
HYDROMORPHONE	9150	77	2,173.90	28.23
HYDROCODONE	9193	91	25,392.72	279.04
MEPERIDINE (PETHIDINE)	9230	77	2,055.35	26.69
METHADONE	9250B	46	2,939.69	63.91
MORPHINE	9300	92	27,846.86	302.68
OPIUM POWDERED	9639	29	103.68	3.58
OXYMORPHONE	9652	8	121.59	15.2
ALFENTANIL	9737	21	6.7	0.32
REMIFENTANIL	9739	19	3.41	0.18
SUFENTANIL BASE	9740	23	0.36	0.02
TAPENTADOL	9780	13	879	67.62
FENTANYL BASE	9801	119	420.12	3.53

STATE:NEVADA BUSINESS ACTIVITY:C - PRACTITIONERS

DRUG NAME	DRUG CODE	BUYERS	TOTAL GRAMS	AVG GRAMS
AMPHETAMINE	1100	1	25.65	25.65
LISDEXAMFETAMINE	1205	1	5.67	5.67
METHYLPHENIDATE (DL;D;L;ISOMERS)	1724	1	217.18	217.18
BUTALBITAL	2165	1	135	135
PENTOBARBITAL (SCHEDULE 2)	2270	55	57,764.25	1,050.26
COCAINE	9041L	1	0.14	0.14
CODEINE	9050	39	290.12	7.44
BUPRENORPHINE	9064	187	343.66	1.84
DIHYDROCODEINE	9120	2	0.64	0.32
OXYCODONE	9143	32	2,470.20	77.19
HYDROMORPHONE	9150	140	176.85	1.26
HYDROCODONE	9193	124	961.21	7.75
MEPERIDINE (PETHIDINE)	9230	61	738.71	12.11
METHADONE	9250B	14	209.29	14.95
MORPHINE	9300	93	610.54	6.56
OPIUM POWDERED	9639	1	0.36	0.36
OXYMORPHONE	9652	6	6.24	1.04
NOROXYMORPHONE	9668	1	25.26	25.26
ALFENTANIL	9737	5	0.19	0.04
REMIFENTANIL	9739	5	0.29	0.06
SUFENTANIL BASE	9740	1	0.01	0.01
TAPENTADOL	9780	1	40	40
FENTANYL BASE	9801	183	28.57	0.16

STATE:NEVADA BUSINESS ACTIVITY:D - TEACHING INSTITUTIONS

DRUG NAME	DRUG CODE	BUYERS	TOTAL GRAMS	AVG GRAMS
MEPERIDINE (PETHIDINE)	9230	1	2.18	2.18
FENTANYL BASE	9801	1	0	0

STATE:NEVADA BUSINESS ACTIVITY:M - MID-LEVEL PRACTITIONERS

DRUG NAME	DRUG CODE	BUYERS	TOTAL GRAMS	AVG GRAMS
METHYLPHENIDATE (DL;D;L;ISOMERS)	1724	1	0.86	0.86
BUTALBITAL	2165	4	265	66.25
PENTOBARBITAL (SCHEDULE 2)	2270	9	3,554.46	394.94
CODEINE	9050	10	128.05	12.81
OXYCODONE	9143	4	2,105.65	526.41
HYDROMORPHONE	9150	4	30.14	7.54
HYDROCODONE	9193	6	713.01	118.83
MEPERIDINE (PETHIDINE)	9230	1	7.84	7.84
METHADONE	9250B	4	355.52	88.88
MORPHINE	9300	5	632.88	126.58
OXYMORPHONE	9652	3	13.92	4.64
TAPENTADOL	9780	2	110	55
FENTANYL BASE	9801	4	6.55	1.64

STATE:NEVADA BUSINESS ACTIVITY:N-U NARCOTIC TREATMENT PROGRAMS

DRUG NAME	DRUG CODE	BUYERS	TOTAL GRAMS	AVG GRAMS
BUPRENORPHINE	9064	7	375.29	53.61
METHADONE	9250B	14	98,291.43	7,020.82

STATE:NEW HAMPSHIRE BUSINESS ACTIVITY:A - PHARMACIES

DRUG NAME	DRUG CODE	BUYERS	TOTAL GRAMS	AVG GRAMS
AMPHETAMINE	1100	266	151,006.22	567.69
DL-METHAMPHETAMINE RACEMIC BASE	1105B	7	26.12	3.73
LISDEXAMFETAMINE	1205	265	55,129.35	208.04
METHYLPHENIDATE (DL;D;L;ISOMERS)	1724	266	133,225.04	500.85
BARBITURIC ACID DERIVIATIVE OR SALT [PER 21C	2100	73	1,995	27.33
BUTALBITAL	2165	104	2,665	25.63
SECOBARBITAL (SCHEDULE 2)	2315	3	27.47	9.16
DRONABINOL IN AN ORAL SOLUTION IN FDA APPR	7365	2	1.8	0.9
NABILONE	7379	1	0.05	0.05
COCAINE	9041L	1	0.89	0.89
CODEINE	9050	262	29,873.77	114.02

BUPRENORPHINE	9064	266	30,886.95	116.12
OXYCODONE	9143	265	239,173.80	902.54
HYDROMORPHONE	9150	262	6,852.23	26.15
HYDROCODONE	9193	267	34,627.25	129.69
LEVORPHANOL	9220L	5	5.34	1.07
MEPERIDINE (PETHIDINE)	9230	62	1,915.56	30.9
METHADONE	9250B	249	22,414.65	90.02
MORPHINE	9300	268	67,312.62	251.17
OPIUM TINCTURE (LAUDANUM)	9630	1	4.73	4.73
OPIUM POWDERED	9639	15	51.84	3.46
OXYMORPHONE	9652	160	3,487.98	21.8
TAPENTADOL	9780	205	29,166.50	142.28
FENTANYL BASE	9801	256	1,224.90	4.78
OPIUM COMBINATION PRODUCT (C-III)	9809	11	8.89	0.81

STATE:NEW HAMPSHIRE BUSINESS ACTIVITY:B - HOSPITALS

DRUG NAME	DRUG CODE	BUYERS	TOTAL GRAMS	AVG GRAMS
AMPHETAMINE	1100	23	2,862.90	124.47
LISDEXAMFETAMINE	1205	12	508.73	42.39
METHYLPHENIDATE (DL;D;L;ISOMERS)	1724	35	2,414.93	69
AMOBARBITAL (SCHEDULE 2)	2125	1	12.3	12.3
PENTOBARBITAL (SCHEDULE 2)	2270	3	359.09	119.7
COCAINE	9041L	26	180.42	6.94
CODEINE	9050	33	457.54	13.86
BUPRENORPHINE	9064	26	651.12	25.04
OXYCODONE	9143	47	10,206.15	217.15
HYDROMORPHONE	9150	40	972.52	24.31
HYDROCODONE	9193	45	1,480.23	32.89
MEPERIDINE (PETHIDINE)	9230	36	323.65	8.99
METHADONE	9250B	31	1,379.61	44.5
MORPHINE	9300	47	5,109.35	108.71
OPIUM POWDERED	9639	17	94.68	5.57
OXYMORPHONE	9652	1	3.57	3.57
ALFENTANIL	9737	7	1.95	0.28
REMIFENTANIL	9739	17	6.51	0.38

SUFENTANIL BASE	9740	7	0.06	0.01
TAPENTADOL	9780	1	249	249
FENTANYL BASE	9801	47	150.54	3.2

STATE:NEW HAMPSHIRE BUSINESS ACTIVITY:C - PRACTITIONERS

DRUG NAME	DRUG CODE	BUYERS	TOTAL GRAMS	AVG GRAMS
PENTOBARBITAL (SCHEDULE 2)	2270	81	61,566.17	760.08
COCAINE	9041L	1	9	9
CODEINE	9050	3	11.64	3.88
BUPRENORPHINE	9064	178	11.4	0.06
OXYCODONE	9143	2	0.9	0.45
HYDROMORPHONE	9150	84	38.97	0.46
HYDROCODONE	9193	14	15.55	1.11
MEPERIDINE (PETHIDINE)	9230	10	110.68	11.07
METHADONE	9250B	3	1.61	0.54
MORPHINE	9300	53	55.51	1.05
OXYMORPHONE	9652	4	0.04	0.01
REMIFENTANIL	9739	1	0.01	0.01
SUFENTANIL BASE	9740	1	0	0
FENTANYL BASE	9801	100	15.65	0.16

STATE:NEW HAMPSHIRE BUSINESS ACTIVITY:M - MID-LEVEL PRACTITIONERS

DRUG NAME	DRUG CODE	BUYERS	TOTAL GRAMS	AVG GRAMS
CODEINE	9050	1	4.42	4.42
HYDROCODONE	9193	1	0.3	0.3
FENTANYL BASE	9801	1	0.01	0.01

STATE:NEW HAMPSHIRE BUSINESS ACTIVITY:N-U NARCOTIC TREATMENT PROGRAMS

DRUG NAME	DRUG CODE	BUYERS	TOTAL GRAMS	AVG GRAMS
BUPRENORPHINE	9064	4	543.8	135.95
METHADONE	9250B	8	99,537.78	12,442.22

STATISTICAL SUMMARY FOR RETAIL DRUG PURCHASES BY GRAMS WT

STATE:NEW JERSEY BUSINESS ACTIVITY:A - PHARMACIES

DRUG NAME	DRUG CODE	BUYERS	TOTAL GRAMS	AVG GRAMS
AMPHETAMINE	1100	1,980	413,434.81	208.81
DL-METHAMPHETAMINE RACEMIC BASE	1105B	49	138.24	2.82
D-METHAMPHETAMINE	1105D	4	5.62	1.41
LISDEXAMFETAMINE	1205	1,807	194,671.13	107.73
METHYLPHENIDATE (DL;D;L;ISOMERS)	1724	1,948	363,224.43	186.46
BARBITURIC ACID DERIVIATIVE OR SALT [PER 21C	2100	515	18,025	35
BUTALBITAL	2165	648	24,470	37.76
PENTOBARBITAL (SCHEDULE 2)	2270	1	11	11
SECOBARBITAL (SCHEDULE 2)	2315	11	228.88	20.81
DRONABINOL IN AN ORAL SOLUTION IN FDA APPR	7365	11	143.55	13.05
NABILONE	7379	4	0.35	0.09
COCAINE	9041L	7	118.31	16.9
CODEINE	9050	1,994	298,841.60	149.87
BUPRENORPHINE	9064	1,831	103,668.13	56.62
DIHYDROCODEINE	9120	82	1,154.68	14.08
OXYCODONE	9143	2,030	1,954,808.71	962.96
HYDROMORPHONE	9150	1,779	30,522.62	17.16
HYDROCODONE	9193	1,985	135,813.57	68.42
LEVORPHANOL	9220L	26	1,620.24	62.32
MEPERIDINE (PETHIDINE)	9230	236	6,194.08	26.25
METHADONE	9250B	1,193	42,942.47	36
MORPHINE	9300	1,842	324,762.10	176.31
OPIUM TINCTURE (LAUDANUM)	9630	1	1.18	1.18
OPIUM POWDERED	9639	109	320.04	2.94
OXYMORPHONE	9652	989	25,615.09	25.9
ALFENTANIL	9737	2	0.47	0.24
REMIFENTANIL	9739	5	0.4	0.08
SUFENTANIL BASE	9740	3	2.78	0.93
TAPENTADOL	9780	1,405	187,283	133.3
FENTANYL BASE	9801	1,810	8,428.03	4.66
OPIUM COMBINATION PRODUCT (C-III)	9809	35	20.43	0.58

STATISTICAL SUMMARY FOR RETAIL DRUG PURCHASES BY GRAMS WT

STATE:NEW JERSEY BUSINESS ACTIVITY:B - HOSPITALS

DRUG NAME	DRUG CODE	BUYERS	TOTAL GRAMS	AVG GRAMS
AMPHETAMINE	1100	52	3,242.42	62.35
LISDEXAMFETAMINE	1205	23	1,289.74	56.08
METHYLPHENIDATE (DL;D;L;ISOMERS)	1724	96	3,555.90	37.04
BARBITURIC ACID DERIVIATIVE OR SALT [PER 21C	2100	2	85	42.5
AMOBARBITAL (SCHEDULE 2)	2125	10	30.53	3.05
BUTALBITAL	2165	5	200	40
PENTOBARBITAL (SCHEDULE 2)	2270	17	793.37	46.67
COCAINE	9041L	62	351.73	5.67
CODEINE	9050	107	4,538.21	42.41
BUPRENORPHINE	9064	84	824.04	9.81
DIHYDROCODEINE	9120	2	5.34	2.67
OXYCODONE	9143	120	36,298.47	302.49
HYDROMORPHONE	9150	112	4,832.17	43.14
HYDROCODONE	9193	89	1,402.54	15.76
MEPERIDINE (PETHIDINE)	9230	76	1,166.72	15.35
METHADONE	9250B	99	3,457.55	34.92
MORPHINE	9300	115	15,255.99	132.66
OPIUM POWDERED	9639	42	122.04	2.91
OXYMORPHONE	9652	7	78.19	11.17
ALFENTANIL	9737	5	0.38	0.08
REMIFENTANIL	9739	54	50.87	0.94
SUFENTANIL BASE	9740	22	0.94	0.04
TAPENTADOL	9780	36	1,935.50	53.76
FENTANYL BASE	9801	108	685.01	6.34
OPIUM COMBINATION PRODUCT (C-III)	9809	2	0.38	0.19

STATE:NEW JERSEY BUSINESS ACTIVITY:C - PRACTITIONERS

DRUG NAME	DRUG CODE	BUYERS	TOTAL GRAMS	AVG GRAMS
AMPHETAMINE	1100	1	1.47	1.47
PENTOBARBITAL (SCHEDULE 2)	2270	94	74,270.34	790.11
COCAINE	9041L	38	175.7	4.62

CODEINE	9050	549	6,748.20	12.29
BUPRENORPHINE	9064	550	408.22	0.74
DIHYDROCODEINE	9120	85	79.68	0.94
OXYCODONE	9143	112	205.08	1.83
HYDROMORPHONE	9150	295	142.79	0.48
HYDROCODONE	9193	258	911.93	3.53
MEPERIDINE (PETHIDINE)	9230	126	341.95	2.71
METHADONE	9250B	22	81.48	3.7
MORPHINE	9300	198	223.81	1.13
OPIUM POWDERED	9639	2	1.08	0.54
OXYMORPHONE	9652	6	0.77	0.13
NOROXYMORPHONE	9668	2	23.7	11.85
ALFENTANIL	9737	2	0.2	0.1
REMIFENTANIL	9739	29	1.64	0.06
SUFENTANIL BASE	9740	3	0.01	0
TAPENTADOL	9780	1	7.5	7.5
FENTANYL BASE	9801	498	65.7	0.13

STATE:NEW JERSEY BUSINESS ACTIVITY:D - TEACHING INSTITUTIONS

DRUG NAME	DRUG CODE	BUYERS	TOTAL GRAMS	AVG GRAMS
BUPRENORPHINE	9064	4	0.02	0
MORPHINE	9300	1	0.02	0.02

STATE:NEW JERSEY BUSINESS ACTIVITY:M - MID-LEVEL PRACTITIONERS

DRUG NAME	DRUG CODE	BUYERS	TOTAL GRAMS	AVG GRAMS
PENTOBARBITAL (SCHEDULE 2)	2270	10	6,843.43	684.34
CODEINE	9050	45	685.87	15.24
BUPRENORPHINE	9064	1	0.12	0.12
DIHYDROCODEINE	9120	6	9.05	1.51

STATE:NEW JERSEY BUSINESS ACTIVITY:N-U NARCOTIC TREATMENT PROGRAMS

DRUG NAME	DRUG CODE	BUYERS	TOTAL GRAMS	AVG GRAMS
BUPRENORPHINE	9064	18	652.27	36.24
METHADONE	9250B	31	413,079.53	13,325.15

STATE:NEW MEXICO BUSINESS ACTIVITY:A - PHARMACIES

DRUG NAME	DRUG CODE	BUYERS	TOTAL GRAMS	AVG GRAMS
AMPHETAMINE	1100	345	60,983.73	176.76
DL-METHAMPHETAMINE RACEMIC BASE	1105B	23	65.9	2.87
LISDEXAMFETAMINE	1205	319	16,776.46	52.59
METHYLPHENIDATE (DL;D;L;ISOMERS)	1724	350	69,650.67	199
BARBITURIC ACID DERIVIATIVE OR SALT [PER 21C	2100	114	3,305	28.99
BUTALBITAL	2165	114	3,690	32.37
DRONABINOL IN AN ORAL SOLUTION IN FDA APPR	7365	1	0.15	0.15
NABILONE	7379	1	0.1	0.1
COCAINE	9041L	1	0.71	0.71
CODEINE	9050	349	66,843.22	191.53
BUPRENORPHINE	9064	335	27,314.03	81.53
DIHYDROCODEINE	9120	15	343.95	22.93
OXYCODONE	9143	354	334,781.44	945.71
HYDROMORPHONE	9150	317	4,463.65	14.08
HYDROCODONE	9193	355	126,982.61	357.7
MEPERIDINE (PETHIDINE)	9230	187	4,691.50	25.09
METHADONE	9250B	283	8,166.95	28.86
MORPHINE	9300	350	86,645.42	247.56
OPIUM POWDERED	9639	15	29.16	1.94
OXYMORPHONE	9652	208	3,785.05	18.2
TAPENTADOL	9780	183	12,742	69.63
FENTANYL BASE	9801	334	1,394.40	4.17
OPIUM COMBINATION PRODUCT (C-III)	9809	6	6.62	1.1

STATE:NEW MEXICO BUSINESS ACTIVITY:B - HOSPITALS

DRUG NAME	DRUG CODE	BUYERS	TOTAL GRAMS	AVG GRAMS
AMPHETAMINE	1100	41	2,676.61	65.28

LISDEXAMFETAMINE	1205	9	483.29	53.7
METHYLPHENIDATE (DL;D;L;ISOMERS)	1724	59	6,619.76	112.2
BARBITURIC ACID DERIVIATIVE OR SALT [PER 21C	2100	3	20	6.67
BUTALBITAL	2165	1	10	10
PENTOBARBITAL (SCHEDULE 2)	2270	5	123.95	24.79
COCAINE	9041L	23	289.7	12.6
CODEINE	9050	71	11,150.29	157.05
BUPRENORPHINE	9064	44	1,744.13	39.64
OXYCODONE	9143	87	26,561.48	305.3
HYDROMORPHONE	9150	65	846.69	13.03
HYDROCODONE	9193	94	12,433.96	132.28
MEPERIDINE (PETHIDINE)	9230	58	933.96	16.1
METHADONE	9250B	47	2,442.16	51.96
MORPHINE	9300	96	16,345.95	170.27
OPIUM POWDERED	9639	29	115.56	3.98
OXYMORPHONE	9652	3	12.94	4.31
ALFENTANIL	9737	10	4.1	0.41
REMIFENTANIL	9739	17	4.42	0.26
SUFENTANIL BASE	9740	12	0.19	0.02
TAPENTADOL	9780	4	84.5	21.13
FENTANYL BASE	9801	100	324.63	3.25

STATE:NEW MEXICO BUSINESS ACTIVITY:C - PRACTITIONERS

DRUG NAME	DRUG CODE	BUYERS	TOTAL GRAMS	AVG GRAMS
PENTOBARBITAL (SCHEDULE 2)	2270	77	64,790.68	841.44
CODEINE	9050	20	144.21	7.21
BUPRENORPHINE	9064	133	115.49	0.87
OXYCODONE	9143	5	47.14	9.43
HYDROMORPHONE	9150	75	76.01	1.01
HYDROCODONE	9193	48	122.47	2.55
MEPERIDINE (PETHIDINE)	9230	7	25.06	3.58
METHADONE	9250B	3	1.34	0.45
MORPHINE	9300	51	132.72	2.6
OXYMORPHONE	9652	2	0.06	0.03
REMIFENTANIL	9739	2	0.02	0.01

SUFENTANIL BASE	9740	2	0	0
FENTANYL BASE	9801	95	15.03	0.16

STATE:NEW MEXICO BUSINESS ACTIVITY:D - TEACHING INSTITUTIONS

DRUG NAME	DRUG CODE	BUYERS	TOTAL GRAMS	AVG GRAMS
PENTOBARBITAL (SCHEDULE 2)	2270	1	177.72	177.72
BUPRENORPHINE	9064	1	0.01	0.01
FENTANYL BASE	9801	1	0.02	0.02

STATE:NEW MEXICO BUSINESS ACTIVITY:M - MID-LEVEL PRACTITIONERS

DRUG NAME	DRUG CODE	BUYERS	TOTAL GRAMS	AVG GRAMS
PENTOBARBITAL (SCHEDULE 2)	2270	9	15,210.75	1,690.08
CODEINE	9050	14	49.29	3.52
BUPRENORPHINE	9064	2	0.57	0.29
HYDROMORPHONE	9150	7	9.26	1.32
HYDROCODONE	9193	3	4.64	1.55
MORPHINE	9300	24	28.94	1.21
FENTANYL BASE	9801	38	0.97	0.03

STATE:NEW MEXICO BUSINESS ACTIVITY:N-U NARCOTIC TREATMENT PROGRAMS

DRUG NAME	DRUG CODE	BUYERS	TOTAL GRAMS	AVG GRAMS
BUPRENORPHINE	9064	4	321.37	80.34
METHADONE	9250B	16	165,785.98	10,361.62

STATE:NEW YORK BUSINESS ACTIVITY:A - PHARMACIES

DRUG NAME	DRUG CODE	BUYERS	TOTAL GRAMS	AVG GRAMS
AMPHETAMINE	1100	4,608	933,140.47	202.5
DL-METHAMPHETAMINE RACEMIC BASE	1105B	99	325.5	3.29
D-METHAMPHETAMINE	1105D	3	5.62	1.87

LISDEXAMFETAMINE	1205	3,780	336,801.64	89.1
METHYLPHENIDATE (DL;D;L;ISOMERS)	1724	4,509	897,756.11	199.1
BARBITURIC ACID DERIVIATIVE OR SALT [PER 21C	2100	691	17,585	25.45
BUTALBITAL	2165	844	22,770	26.98
SECOBARBITAL (SCHEDULE 2)	2315	36	1,501.42	41.71
DRONABINOL IN AN ORAL SOLUTION IN FDA APPR	7365	6	1.35	0.23
NABILONE	7379	4	0.3	0.08
COCAINE	9041L	5	43.32	8.66
CODEINE	9050	4,829	548,795.54	113.65
BUPRENORPHINE	9064	3,975	205,972.50	51.82
DIHYDROCODEINE	9120	52	528.74	10.17
OXYCODONE	9143	4,940	2,855,205.02	577.98
HYDROMORPHONE	9150	3,962	62,142.71	15.68
HYDROCODONE	9193	4,691	652,797.84	139.16
LEVORPHANOL	9220L	175	160.07	0.91
MEPERIDINE (PETHIDINE)	9230	376	12,760.50	33.94
METHADONE	9250B	3,143	138,877.51	44.19
MORPHINE	9300	4,107	559,554.36	136.24
OPIUM TINCTURE (LAUDANUM)	9630	1	4.73	4.73
OPIUM POWDERED	9639	134	352.44	2.63
OXYMORPHONE	9652	2,137	71,418.43	33.42
ALFENTANIL	9737	1	0.36	0.36
REMIFENTANIL	9739	2	0.07	0.04
SUFENTANIL BASE	9740	1	0.27	0.27
TAPENTADOL	9780	2,688	376,794	140.18
FENTANYL BASE	9801	4,070	14,662.30	3.6
OPIUM COMBINATION PRODUCT (C-III)	9809	71	34.43	0.48

STATE:NEW YORK BUSINESS ACTIVITY:B - HOSPITALS

DRUG NAME	DRUG CODE	BUYERS	TOTAL GRAMS	AVG GRAMS
AMPHETAMINE	1100	183	8,586.93	46.92
LISDEXAMFETAMINE	1205	84	1,611.64	19.19
METHYLPHENIDATE (DL;D;L;ISOMERS)	1724	250	12,305.75	49.22
AMOBARBITAL (SCHEDULE 2)	2125	24	152.66	6.36
BUTALBITAL	2165	1	5	5

PENTOBARBITAL (SCHEDULE 2)	2270	39	11,239.38	288.19
DRONABINOL IN AN ORAL SOLUTION IN FDA APPR	7365	2	0.6	0.3
NABILONE	7379	1	0.1	0.1
COCAINE	9041L	146	1,156.68	7.92
CODEINE	9050	286	15,225.18	53.23
BUPRENORPHINE	9064	227	4,980.06	21.94
OXYCODONE	9143	331	95,895.83	289.72
HYDROMORPHONE	9150	285	15,628.55	54.84
HYDROCODONE	9193	253	26,950.88	106.53
LEVORPHANOL	9220L	3	1.04	0.35
MEPERIDINE (PETHIDINE)	9230	208	4,064.81	19.54
METHADONE	9250B	261	25,920.08	99.31
MORPHINE	9300	320	60,556.56	189.24
OPIUM POWDERED	9639	84	504	6
OXYMORPHONE	9652	47	394.54	8.39
ALFENTANIL	9737	25	7.89	0.32
REMIFENTANIL	9739	121	152.48	1.26
SUFENTANIL BASE	9740	48	6.43	0.13
TAPENTADOL	9780	86	7,090.50	82.45
FENTANYL BASE	9801	295	2,431.02	8.24
OPIUM COMBINATION PRODUCT (C-III)	9809	8	3.59	0.45

STATE:NEW YORK BUSINESS ACTIVITY:C - PRACTITIONERS

DRUG NAME	DRUG CODE	BUYERS	TOTAL GRAMS	AVG GRAMS
AMPHETAMINE	1100	2	5.74	2.87
D-METHAMPHETAMINE	1105D	2	8.11	4.06
LISDEXAMFETAMINE	1205	1	5.2	5.2
METHYLPHENIDATE (DL;D;L;ISOMERS)	1724	1	18.68	18.68
GAMMA HYDROXYBUTYRIC ACID(FDA APPROVED	2012	1	7,950.53	7,950.53
PENTOBARBITAL (SCHEDULE 2)	2270	327	283,493.89	866.95
COCAINE	9041L	47	179.88	3.83
CODEINE	9050	130	3,662.15	28.17
BUPRENORPHINE	9064	1,069	162.95	0.15
DIHYDROCODEINE	9120	3	0.53	0.18
OXYCODONE	9143	138	615.28	4.46

HYDROMORPHONE	9150	615	290.15	0.47
HYDROCODONE	9193	430	1,401.04	3.26
MEPERIDINE (PETHIDINE)	9230	214	1,462.81	6.84
METHADONE	9250B	28	93.91	3.35
MORPHINE	9300	395	646.37	1.64
OPIUM POWDERED	9639	2	12.96	6.48
OXYMORPHONE	9652	8	0.91	0.11
NOROXYMORPHONE	9668	5	22.13	4.43
ALFENTANIL	9737	5	0.9	0.18
REMIFENTANIL	9739	29	2.48	0.09
SUFENTANIL BASE	9740	8	0.04	0
FENTANYL BASE	9801	1,102	143.75	0.13

STATE:NEW YORK BUSINESS ACTIVITY:D - TEACHING INSTITUTIONS

DRUG NAME	DRUG CODE	BUYERS	TOTAL GRAMS	AVG GRAMS
AMPHETAMINE	1100	1	0.73	0.73
D-METHAMPHETAMINE	1105D	1	4.02	4.02
PENTOBARBITAL (SCHEDULE 2)	2270	2	182.28	91.14
BUPRENORPHINE	9064	7	0.15	0.02
MORPHINE	9300	1	0.56	0.56
FENTANYL BASE	9801	1	0.01	0.01

STATE:NEW YORK BUSINESS ACTIVITY:M - MID-LEVEL PRACTITIONERS

DRUG NAME	DRUG CODE	BUYERS	TOTAL GRAMS	AVG GRAMS
PENTOBARBITAL (SCHEDULE 2)	2270	9	11,479.79	1,275.53
CODEINE	9050	4	67.06	16.76
BUPRENORPHINE	9064	1	0.18	0.18
HYDROCODONE	9193	1	0.06	0.06
MEPERIDINE (PETHIDINE)	9230	1	1.09	1.09
FENTANYL BASE	9801	1	0.28	0.28

STATE:NEW YORK BUSINESS ACTIVITY:N-U NARCOTIC TREATMENT PROGRAMS

DRUG NAME	DRUG CODE	BUYERS	TOTAL GRAMS	AVG GRAMS
BUPRENORPHINE	9064	35	2,241.80	64.05
METHADONE	9250B	107	1,054,499.21	9,855.13

STATE:NORTH CAROLINA BUSINESS ACTIVITY:A - PHARMACIES

DRUG NAME	DRUG CODE	BUYERS	TOTAL GRAMS	AVG GRAMS
AMPHETAMINE	1100	2,182	783,256.30	358.96
DL-METHAMPHETAMINE RACEMIC BASE	1105B	53	189.27	3.57
D-METHAMPHETAMINE	1105D	7	21.69	3.1
LISDEXAMFETAMINE	1205	2,134	400,999.64	187.91
METHYLPHENIDATE (DL;D;L;ISOMERS)	1724	2,173	729,832.98	335.86
BARBITURIC ACID DERIVIATIVE OR SALT [PER 21C	2100	622	15,190	24.42
AMOBARBITAL (SCHEDULE 2)	2125	1	0.46	0.46
BUTALBITAL	2165	768	20,160	26.25
SECOBARBITAL (SCHEDULE 2)	2315	1	9.16	9.16
DRONABINOL IN AN ORAL SOLUTION IN FDA APPR	7365	4	1.05	0.26
NABILONE	7379	1	0.75	0.75
COCAINE	9041L	8	18.21	2.28
CODEINE	9050	2,119	183,727.15	86.7
BUPRENORPHINE	9064	2,065	116,841.36	56.58
DIHYDROCODEINE	9120	20	38.45	1.92
OXYCODONE	9143	2,196	2,127,760.19	968.93
HYDROMORPHONE	9150	2,081	35,580.68	17.1
HYDROCODONE	9193	2,202	721,205.59	327.52
LEVORPHANOL	9220L	241	265.24	1.1
MEPERIDINE (PETHIDINE)	9230	1,072	21,046.62	19.63
METHADONE	9250B	1,938	101,110.13	52.17
MORPHINE	9300	2,150	425,240.22	197.79
OPIUM POWDERED	9639	145	249.12	1.72
OXYMORPHONE	9652	1,733	81,925.14	47.27
ALFENTANIL	9737	2	0.52	0.26
REMIFENTANIL	9739	5	0.15	0.03
TAPENTADOL	9780	1,880	373,342.50	198.59
FENTANYL BASE	9801	2,099	9,921.68	4.73

OPIUM COMBINATION PRODUCT (C-III)	9809	79	46.16	0.58

STATE:NORTH CAROLINA BUSINESS ACTIVITY:B - HOSPITALS

DRUG NAME	DRUG CODE	BUYERS	TOTAL GRAMS	AVG GRAMS
AMPHETAMINE	1100	144	29,274.43	203.29
LISDEXAMFETAMINE	1205	74	9,571.60	129.35
METHYLPHENIDATE (DL;D;L;ISOMERS)	1724	166	32,330.23	194.76
GAMMA HYDROXYBUTYRIC ACID(FDA APPROVED	2012	1	2,303.42	2,303.42
BARBITURIC ACID DERIVIATIVE OR SALT [PER 21C	2100	5	75	15
AMOBARBITAL (SCHEDULE 2)	2125	5	37.82	7.56
BUTALBITAL	2165	8	230	28.75
PENTOBARBITAL (SCHEDULE 2)	2270	58	57,754.21	995.76
COCAINE	9041L	108	545.75	5.05
CODEINE	9050	179	9,394.60	52.48
BUPRENORPHINE	9064	237	3,060.72	12.91
OXYCODONE	9143	259	111,067.62	428.83
HYDROMORPHONE	9150	320	7,288.89	22.78
HYDROCODONE	9193	307	47,459.63	154.59
LEVORPHANOL	9220L	1	0.46	0.46
MEPERIDINE (PETHIDINE)	9230	194	3,049.05	15.72
METHADONE	9250B	170	8,413.99	49.49
MORPHINE	9300	319	50,321.02	157.75
OPIUM POWDERED	9639	99	668.16	6.75
OXYMORPHONE	9652	82	1,030.11	12.56
ALFENTANIL	9737	29	12.05	0.42
REMIFENTANIL	9739	80	53.01	0.66
SUFENTANIL BASE	9740	61	1.64	0.03
TAPENTADOL	9780	87	13,185	151.55
FENTANYL BASE	9801	329	1,307.64	3.97
OPIUM COMBINATION PRODUCT (C-III)	9809	4	7.76	1.94

STATE:NORTH CAROLINA BUSINESS ACTIVITY:C - PRACTITIONERS

DRUG NAME	DRUG CODE	BUYERS	TOTAL GRAMS	AVG GRAMS

AMPHETAMINE	1100	1	0.74	0.74
LISDEXAMFETAMINE	1205	1	0.72	0.72
METHYLPHENIDATE (DL;D;L;ISOMERS)	1724	1	16.52	16.52
GAMMA HYDROXYBUTYRIC ACID(FDA APPROVED	2012	1	2,674.94	2,674.94
PENTOBARBITAL (SCHEDULE 2)	2270	199	132,180.20	664.22
COCAINE	9041L	5	7.71	1.54
CODEINE	9050	119	376.08	3.16
BUPRENORPHINE	9064	898	57.14	0.06
OXYCODONE	9143	24	245.68	10.24
HYDROMORPHONE	9150	393	166.39	0.42
HYDROCODONE	9193	279	655.97	2.35
MEPERIDINE (PETHIDINE)	9230	98	446.21	4.55
METHADONE	9250B	7	14.13	2.02
MORPHINE	9300	212	455.78	2.15
OPIUM POWDERED	9639	1	0.36	0.36
OXYMORPHONE	9652	6	1.09	0.18
NOROXYMORPHONE	9668	1	1.56	1.56
REMIFENTANIL	9739	24	4.59	0.19
SUFENTANIL BASE	9740	2	0.03	0.01
FENTANYL BASE	9801	415	35.33	0.09

STATE:NORTH CAROLINA BUSINESS ACTIVITY:D - TEACHING INSTITUTIONS

DRUG NAME	DRUG CODE	BUYERS	TOTAL GRAMS	AVG GRAMS
PENTOBARBITAL (SCHEDULE 2)	2270	1	266.58	266.58
BUPRENORPHINE	9064	3	0.03	0.01
HYDROMORPHONE	9150	2	0.14	0.07
METHADONE	9250B	1	0.89	0.89
MORPHINE	9300	1	0.19	0.19
FENTANYL BASE	9801	1	0.03	0.03

STATE:NORTH CAROLINA BUSINESS ACTIVITY:M - MID-LEVEL PRACTITIONERS

DRUG NAME	DRUG CODE	BUYERS	TOTAL GRAMS	AVG GRAMS
PENTOBARBITAL (SCHEDULE 2)	2270	37	52,333.38	1,414.42

CODEINE	9050	70	119.34	1.7
BUPRENORPHINE	9064	2	0.04	0.02
HYDROMORPHONE	9150	10	1.38	0.14
MORPHINE	9300	76	122.8	1.62
NOROXYMORPHONE	9668	1	2.12	2.12
FENTANYL BASE	9801	82	1.53	0.02

STATE:NORTH CAROLINA BUSINESS ACTIVITY:N-U NARCOTIC TREATMENT PROGRAMS

DRUG NAME	DRUG CODE	BUYERS	TOTAL GRAMS	AVG GRAMS
BUPRENORPHINE	9064	44	10,882.82	247.34
METHADONE	9250B	62	416,957.21	6,725.12

STATE:NORTH DAKOTA BUSINESS ACTIVITY:A - PHARMACIES

DRUG NAME	DRUG CODE	BUYERS	TOTAL GRAMS	AVG GRAMS
AMPHETAMINE	1100	170	33,433.63	196.67
DL-METHAMPHETAMINE RACEMIC BASE	1105B	3	22.5	7.5
LISDEXAMFETAMINE	1205	165	21,507.05	130.35
METHYLPHENIDATE (DL;D;L;ISOMERS)	1724	170	54,194.44	318.79
BARBITURIC ACID DERIVIATIVE OR SALT [PER 21C	2100	24	505	21.04
BUTALBITAL	2165	36	1,275	35.42
CODEINE	9050	171	18,917.36	110.63
BUPRENORPHINE	9064	131	3,480.74	26.57
OXYCODONE	9143	174	49,037.51	281.82
HYDROMORPHONE	9150	156	1,848.34	11.85
HYDROCODONE	9193	176	33,229.56	188.8
MEPERIDINE (PETHIDINE)	9230	53	649.05	12.25
METHADONE	9250B	122	3,385.04	27.75
MORPHINE	9300	171	19,115.07	111.78
OPIUM POWDERED	9639	24	46.8	1.95
OXYMORPHONE	9652	44	608.06	13.82
ALFENTANIL	9737	1	0.03	0.03
TAPENTADOL	9780	95	8,464.50	89.1
FENTANYL BASE	9801	167	756.58	4.53

OPIUM COMBINATION PRODUCT (C-III)	9809	1	1.14	1.14

STATE:NORTH DAKOTA BUSINESS ACTIVITY:B - HOSPITALS

DRUG NAME	DRUG CODE	BUYERS	TOTAL GRAMS	AVG GRAMS
AMPHETAMINE	1100	21	2,528.18	120.39
DL-METHAMPHETAMINE RACEMIC BASE	1105B	1	1.21	1.21
D-METHAMPHETAMINE	1105D	1	2.01	2.01
LISDEXAMFETAMINE	1205	15	1,385.13	92.34
METHYLPHENIDATE (DL;D;L;ISOMERS)	1724	25	5,932.29	237.29
BARBITURIC ACID DERIVIATIVE OR SALT [PER 21C	2100	2	60	30
BUTALBITAL	2165	2	10	5
PENTOBARBITAL (SCHEDULE 2)	2270	3	110.28	36.76
COCAINE	9041L	13	181.56	13.97
CODEINE	9050	50	3,728.53	74.57
BUPRENORPHINE	9064	16	149.78	9.36
OXYCODONE	9143	53	9,349.43	176.4
HYDROMORPHONE	9150	48	732.86	15.27
HYDROCODONE	9193	54	8,074.94	149.54
MEPERIDINE (PETHIDINE)	9230	35	255.02	7.29
METHADONE	9250B	22	477.97	21.73
MORPHINE	9300	56	5,988.08	106.93
OPIUM POWDERED	9639	17	117.36	6.9
OXYMORPHONE	9652	2	21.59	10.79
ALFENTANIL	9737	6	0.56	0.09
REMIFENTANIL	9739	8	1.67	0.21
SUFENTANIL BASE	9740	6	0.13	0.02
TAPENTADOL	9780	6	1,311	218.5
FENTANYL BASE	9801	53	185.39	3.5
OPIUM COMBINATION PRODUCT (C-III)	9809	1	1.51	1.51

STATE:NORTH DAKOTA BUSINESS ACTIVITY:C - PRACTITIONERS

DRUG NAME	DRUG CODE	BUYERS	TOTAL GRAMS	AVG GRAMS
AMPHETAMINE	1100	2	2.5	1.25

D-METHAMPHETAMINE	1105D	1	4.02	4.02
METHYLPHENIDATE (DL;D;L;ISOMERS)	1724	1	1.87	1.87
PENTOBARBITAL (SCHEDULE 2)	2270	34	28,257.96	831.12
COCAINE	9041L	2	0.43	0.21
CODEINE	9050	9	132.5	14.72
BUPRENORPHINE	9064	58	4.82	0.08
OXYCODONE	9143	10	202.78	20.28
HYDROMORPHONE	9150	37	14.16	0.38
HYDROCODONE	9193	26	188.83	7.26
MEPERIDINE (PETHIDINE)	9230	14	47.71	3.41
METHADONE	9250B	1	1.61	1.61
MORPHINE	9300	30	28.69	0.96
OPIUM POWDERED	9639	1	0.72	0.72
FENTANYL BASE	9801	43	5.91	0.14

STATE:NORTH DAKOTA BUSINESS ACTIVITY:N-U NARCOTIC TREATMENT PROGRAMS

DRUG NAME	DRUG CODE	BUYERS	TOTAL GRAMS	AVG GRAMS
METHADONE	9250B	3	4,507.78	1,502.59

STATE:OHIO BUSINESS ACTIVITY:A - PHARMACIES

DRUG NAME	DRUG CODE	BUYERS	TOTAL GRAMS	AVG GRAMS
AMPHETAMINE	1100	2,239	832,478.90	371.81
DL-METHAMPHETAMINE RACEMIC BASE	1105B	40	296.57	7.41
D-METHAMPHETAMINE	1105D	6	19.28	3.21
LISDEXAMFETAMINE	1205	2,184	443,038.50	202.86
METHYLPHENIDATE (DL;D;L;ISOMERS)	1724	2,237	910,677.43	407.1
BARBITURIC ACID DERIVIATIVE OR SALT [PER 21C	2100	631	16,775	26.58
BUTALBITAL	2165	833	26,475	31.78
SECOBARBITAL (SCHEDULE 2)	2315	3	137.33	45.78
DRONABINOL IN AN ORAL SOLUTION IN FDA APPR	7365	6	8.55	1.43
NABILONE	7379	2	1	0.5
COCAINE	9041L	3	10.71	3.57
CODEINE	9050	2,211	392,141.04	177.36

BUPRENORPHINE	9064	2,101	196,821.05	93.68
DIHYDROCODEINE	9120	12	108.95	9.08
OXYCODONE	9143	2,251	1,830,420.30	813.16
HYDROMORPHONE	9150	1,922	28,209.03	14.68
HYDROCODONE	9193	2,263	716,367.69	316.56
LEVORPHANOL	9220L	18	43.53	2.42
MEPERIDINE (PETHIDINE)	9230	458	9,740.32	21.27
METHADONE	9250B	1,895	85,828.95	45.29
MORPHINE	9300	2,180	430,821.21	197.62
OPIUM TINCTURE (LAUDANUM)	9630	22	82.68	3.76
OPIUM POWDERED	9639	260	671.4	2.58
OXYMORPHONE	9652	1,422	38,057.25	26.76
ALFENTANIL	9737	2	0.26	0.13
REMIFENTANIL	9739	1	0.1	0.1
SUFENTANIL BASE	9740	2	0.14	0.07
TAPENTADOL	9780	1,657	239,097	144.3
FENTANYL BASE	9801	2,083	8,506.36	4.08
OPIUM COMBINATION PRODUCT (C-III)	9809	62	53.35	0.86

STATE:OHIO BUSINESS ACTIVITY:B - HOSPITALS

DRUG NAME	DRUG CODE	BUYERS	TOTAL GRAMS	AVG GRAMS
AMPHETAMINE	1100	160	14,539.95	90.87
DL-METHAMPHETAMINE RACEMIC BASE	1105B	2	3.62	1.81
D-METHAMPHETAMINE	1105D	1	2.01	2.01
LISDEXAMFETAMINE	1205	70	3,455.88	49.37
METHYLPHENIDATE (DL;D;L;ISOMERS)	1724	217	16,217.66	74.74
GAMMA HYDROXYBUTYRIC ACID(FDA APPROVED	2012	2	7,430.40	3,715.20
BARBITURIC ACID DERIVIATIVE OR SALT [PER 21C	2100	4	20	5
AMOBARBITAL (SCHEDULE 2)	2125	5	17.77	3.55
BUTALBITAL	2165	12	155	12.92
PENTOBARBITAL (SCHEDULE 2)	2270	32	24,444.20	763.88
DRONABINOL IN AN ORAL SOLUTION IN FDA APPR	7365	1	0.15	0.15
COCAINE	9041L	165	716.78	4.34
CODEINE	9050	274	10,508.07	38.35
BUPRENORPHINE	9064	244	5,909.78	24.22

DIHYDROCODEINE	9120	8	31.35	3.92
OXYCODONE	9143	388	95,344.85	245.73
HYDROMORPHONE	9150	383	8,997.19	23.49
HYDROCODONE	9193	396	25,747.75	65.02
MEPERIDINE (PETHIDINE)	9230	318	9,768.02	30.72
METHADONE	9250B	244	9,047.40	37.08
MORPHINE	9300	405	46,408.03	114.59
OPIUM POWDERED	9639	185	842.4	4.55
OXYMORPHONE	9652	64	491.17	7.67
ALFENTANIL	9737	29	18.53	0.64
REMIFENTANIL	9739	115	74.21	0.65
SUFENTANIL BASE	9740	76	1.82	0.02
TAPENTADOL	9780	74	3,662	49.49
FENTANYL BASE	9801	471	1,308.75	2.78
OPIUM COMBINATION PRODUCT (C-III)	9809	9	4.9	0.54

STATE:OHIO BUSINESS ACTIVITY:C - PRACTITIONERS

DRUG NAME	DRUG CODE	BUYERS	TOTAL GRAMS	AVG GRAMS
AMPHETAMINE	1100	1	0.99	0.99
LISDEXAMFETAMINE	1205	2	19.6	9.8
PENTOBARBITAL (SCHEDULE 2)	2270	294	237,887.06	809.14
COCAINE	9041L	7	3.29	0.47
CODEINE	9050	64	332.8	5.2
BUPRENORPHINE	9064	861	131.59	0.15
OXYCODONE	9143	26	765.03	29.42
HYDROMORPHONE	9150	339	175.71	0.52
HYDROCODONE	9193	274	785.35	2.87
MEPERIDINE (PETHIDINE)	9230	69	472.35	6.85
METHADONE	9250B	6	7.33	1.22
MORPHINE	9300	184	476.14	2.59
OPIUM POWDERED	9639	1	0.72	0.72
OXYMORPHONE	9652	5	0.94	0.19
NOROXYMORPHONE	9668	4	24.31	6.08
ALFENTANIL	9737	3	0.3	0.1
REMIFENTANIL	9739	13	1.31	0.1

FENTANYL BASE	9801	361	33.83	0.09

STATE:OHIO BUSINESS ACTIVITY:D - TEACHING INSTITUTIONS

DRUG NAME	DRUG CODE	BUYERS	TOTAL GRAMS	AVG GRAMS
PENTOBARBITAL (SCHEDULE 2)	2270	1	0.91	0.91
BUPRENORPHINE	9064	6	0.17	0.03
HYDROMORPHONE	9150	4	0.25	0.06
MORPHINE	9300	1	0.03	0.03
REMIFENTANIL	9739	1	0.01	0.01
FENTANYL BASE	9801	1	0.15	0.15

STATE:OHIO BUSINESS ACTIVITY:M - MID-LEVEL PRACTITIONERS

DRUG NAME	DRUG CODE	BUYERS	TOTAL GRAMS	AVG GRAMS
PENTOBARBITAL (SCHEDULE 2)	2270	29	32,789.62	1,130.68
CODEINE	9050	2	1.98	0.99
BUPRENORPHINE	9064	5	1.75	0.35
HYDROMORPHONE	9150	6	0.55	0.09
MEPERIDINE (PETHIDINE)	9230	1	0.22	0.22
MORPHINE	9300	93	16.64	0.18
FENTANYL BASE	9801	152	1.11	0.01

STATE:OHIO BUSINESS ACTIVITY:N-U NARCOTIC TREATMENT PROGRAMS

DRUG NAME	DRUG CODE	BUYERS	TOTAL GRAMS	AVG GRAMS
CODEINE	9050	1	22.1	22.1
BUPRENORPHINE	9064	19	7,234.55	380.77
METHADONE	9250B	23	202,429.55	8,801.28

STATE:OKLAHOMA BUSINESS ACTIVITY:A - PHARMACIES

DRUG NAME	DRUG CODE	BUYERS	TOTAL GRAMS	AVG GRAMS

AMPHETAMINE	1100	895	205,219.28	229.3
DL-METHAMPHETAMINE RACEMIC BASE	1105B	18	77.16	4.29
D-METHAMPHETAMINE	1105D	1	7.23	7.23
LISDEXAMFETAMINE	1205	869	142,821.92	164.35
METHYLPHENIDATE (DL;D;L;ISOMERS)	1724	894	183,848.76	205.65
BARBITURIC ACID DERIVIATIVE OR SALT [PER 21C	2100	255	11,250	44.12
BUTALBITAL	2165	443	19,900	44.92
SECOBARBITAL (SCHEDULE 2)	2315	1	36.62	36.62
COCAINE	9041L	2	0.86	0.43
CODEINE	9050	930	343,730.75	369.6
BUPRENORPHINE	9064	863	42,293.71	49.01
DIHYDROCODEINE	9120	239	1,064.96	4.46
OXYCODONE	9143	909	860,854.37	947.03
HYDROMORPHONE	9150	801	12,337.59	15.4
HYDROCODONE	9193	926	684,169.84	738.84
MEPERIDINE (PETHIDINE)	9230	621	22,530.67	36.28
METHADONE	9250B	751	32,580.40	43.38
MORPHINE	9300	899	333,136.65	370.56
OPIUM POWDERED	9639	55	139.32	2.53
OXYMORPHONE	9652	621	21,522.63	34.66
REMIFENTANIL	9739	2	0.31	0.16
SUFENTANIL BASE	9740	1	0.06	0.06
TAPENTADOL	9780	664	93,729.50	141.16
FENTANYL BASE	9801	894	6,131.44	6.86
OPIUM COMBINATION PRODUCT (C-III)	9809	19	9.65	0.51

STATE:OKLAHOMA BUSINESS ACTIVITY:B - HOSPITALS

DRUG NAME	DRUG CODE	BUYERS	TOTAL GRAMS	AVG GRAMS
AMPHETAMINE	1100	70	6,385.44	91.22
LISDEXAMFETAMINE	1205	38	3,822.90	100.6
METHYLPHENIDATE (DL;D;L;ISOMERS)	1724	91	9,663.84	106.2
BARBITURIC ACID DERIVIATIVE OR SALT [PER 21C	2100	1	5	5
BUTALBITAL	2165	8	90	11.25
PENTOBARBITAL (SCHEDULE 2)	2270	4	211.9	52.98
COCAINE	9041L	42	131.56	3.13

CODEINE	9050	162	36,000.58	222.23
BUPRENORPHINE	9064	71	1,133.98	15.97
DIHYDROCODEINE	9120	4	9.61	2.4
OXYCODONE	9143	201	35,343.94	175.84
HYDROMORPHONE	9150	173	2,217.39	12.82
HYDROCODONE	9193	206	40,352.32	195.89
MEPERIDINE (PETHIDINE)	9230	160	5,895.37	36.85
METHADONE	9250B	79	1,763.04	22.32
MORPHINE	9300	205	29,755.72	145.15
OPIUM POWDERED	9639	42	168.84	4.02
OXYMORPHONE	9652	18	236.51	13.14
ALFENTANIL	9737	8	3.52	0.44
REMIFENTANIL	9739	28	10.43	0.37
SUFENTANIL BASE	9740	35	0.62	0.02
TAPENTADOL	9780	28	1,188	42.43
FENTANYL BASE	9801	209	561.05	2.68
OPIUM COMBINATION PRODUCT (C-III)	9809	6	3.22	0.54

STATE:OKLAHOMA BUSINESS ACTIVITY:C - PRACTITIONERS

DRUG NAME	DRUG CODE	BUYERS	TOTAL GRAMS	AVG GRAMS
LISDEXAMFETAMINE	1205	4	32.29	8.07
PENTOBARBITAL (SCHEDULE 2)	2270	94	68,608.69	729.88
COCAINE	9041L	1	0.57	0.57
CODEINE	9050	101	2,100.55	20.8
BUPRENORPHINE	9064	266	51.22	0.19
DIHYDROCODEINE	9120	67	30.45	0.45
OXYCODONE	9143	13	544.26	41.87
HYDROMORPHONE	9150	86	38.36	0.45
HYDROCODONE	9193	65	1,320.32	20.31
MEPERIDINE (PETHIDINE)	9230	44	229.31	5.21
METHADONE	9250B	5	44.72	8.94
MORPHINE	9300	72	800.31	11.12
OPIUM POWDERED	9639	2	2.52	1.26
OXYMORPHONE	9652	3	1.7	0.57
NOROXYMORPHONE	9668	2	0.64	0.32

SUFENTANIL BASE	9740	1	0.01	0.01
FENTANYL BASE	9801	147	58.09	0.4

STATE:OKLAHOMA BUSINESS ACTIVITY:D - TEACHING INSTITUTIONS

DRUG NAME	DRUG CODE	BUYERS	TOTAL GRAMS	AVG GRAMS
PENTOBARBITAL (SCHEDULE 2)	2270	1	88.86	88.86
BUPRENORPHINE	9064	1	0.25	0.25
HYDROMORPHONE	9150	1	6.03	6.03
HYDROCODONE	9193	1	8.47	8.47
METHADONE	9250B	1	27.28	27.28
MORPHINE	9300	1	44.1	44.1
FENTANYL BASE	9801	1	0.95	0.95

STATE:OKLAHOMA BUSINESS ACTIVITY:M - MID-LEVEL PRACTITIONERS

DRUG NAME	DRUG CODE	BUYERS	TOTAL GRAMS	AVG GRAMS
PENTOBARBITAL (SCHEDULE 2)	2270	10	7,203.25	720.32
CODEINE	9050	42	86.53	2.06
DIHYDROCODEINE	9120	42	24.35	0.58
HYDROMORPHONE	9150	12	0.55	0.05
MORPHINE	9300	41	12.1	0.3
FENTANYL BASE	9801	65	3.62	0.06

STATE:OKLAHOMA BUSINESS ACTIVITY:N-U NARCOTIC TREATMENT PROGRAMS

DRUG NAME	DRUG CODE	BUYERS	TOTAL GRAMS	AVG GRAMS
BUPRENORPHINE	9064	10	241.74	24.17
METHADONE	9250B	19	122,590.04	6,452.11

STATE:OREGON BUSINESS ACTIVITY:A - PHARMACIES

DRUG NAME	DRUG CODE	BUYERS	TOTAL GRAMS	AVG GRAMS

AMPHETAMINE	1100	657	231,398.37	352.2
DL-METHAMPHETAMINE RACEMIC BASE	1105B	34	126.58	3.72
D-METHAMPHETAMINE	1105D	4	32.94	8.23
LISDEXAMFETAMINE	1205	618	59,688.25	96.58
METHYLPHENIDATE (DL;D;L;ISOMERS)	1724	655	241,148.11	368.17
BARBITURIC ACID DERIVIATIVE OR SALT [PER 21C	2100	157	3,785	24.11
BUTALBITAL	2165	304	9,355	30.77
SECOBARBITAL (SCHEDULE 2)	2315	31	952.12	30.71
NABILONE	7379	1	0.05	0.05
COCAINE	9041L	6	111.42	18.57
CODEINE	9050	661	127,945.55	193.56
BUPRENORPHINE	9064	646	35,282.71	54.62
OXYCODONE	9143	665	657,250.78	988.35
HYDROMORPHONE	9150	646	16,093.84	24.91
HYDROCODONE	9193	671	351,923.20	524.48
LEVORPHANOL	9220L	12	13.58	1.13
MEPERIDINE (PETHIDINE)	9230	219	4,671.24	21.33
METHADONE	9250B	628	60,988.69	97.12
MORPHINE	9300	669	258,694.62	386.69
OPIUM TINCTURE (LAUDANUM)	9630	2	2.36	1.18
OPIUM POWDERED	9639	53	123.48	2.33
OXYMORPHONE	9652	367	6,573.93	17.91
ALFENTANIL	9737	2	1.84	0.92
REMIFENTANIL	9739	3	0.11	0.04
SUFENTANIL BASE	9740	1	0	0
TAPENTADOL	9780	364	29,756.50	81.75
FENTANYL BASE	9801	636	3,314.47	5.21
OPIUM COMBINATION PRODUCT (C-III)	9809	12	3.59	0.3

STATE:OREGON BUSINESS ACTIVITY:B - HOSPITALS

DRUG NAME	DRUG CODE	BUYERS	TOTAL GRAMS	AVG GRAMS
AMPHETAMINE	1100	44	3,165.81	71.95
DL-METHAMPHETAMINE RACEMIC BASE	1105B	1	1.21	1.21
D-METHAMPHETAMINE	1105D	1	0.8	0.8
LISDEXAMFETAMINE	1205	20	424.9	21.25

METHYLPHENIDATE (DL;D;L;ISOMERS)	1724	54	4,756.11	88.08
BARBITURIC ACID DERIVIATIVE OR SALT [PER 21C	2100	3	80	26.67
AMOBARBITAL (SCHEDULE 2)	2125	2	6.84	3.42
BUTALBITAL	2165	6	30	5
PENTOBARBITAL (SCHEDULE 2)	2270	3	2,184.63	728.21
COCAINE	9041L	45	240.95	5.35
CODEINE	9050	89	4,144.42	46.57
BUPRENORPHINE	9064	57	942.4	16.53
OXYCODONE	9143	92	35,929.50	390.54
HYDROMORPHONE	9150	78	3,109.96	39.87
HYDROCODONE	9193	103	24,069.16	233.68
MEPERIDINE (PETHIDINE)	9230	65	1,036.85	15.95
METHADONE	9250B	65	4,171.95	64.18
MORPHINE	9300	94	25,293.63	269.08
OPIUM POWDERED	9639	47	654.84	13.93
OXYMORPHONE	9652	6	6.11	1.02
ALFENTANIL	9737	16	15.14	0.95
REMIFENTANIL	9739	30	6.72	0.22
SUFENTANIL BASE	9740	28	1.21	0.04
TAPENTADOL	9780	7	58.5	8.36
FENTANYL BASE	9801	82	353.66	4.31

STATE:OREGON BUSINESS ACTIVITY:C - PRACTITIONERS

DRUG NAME	DRUG CODE	BUYERS	TOTAL GRAMS	AVG GRAMS
LISDEXAMFETAMINE	1205	1	4.6	4.6
PENTOBARBITAL (SCHEDULE 2)	2270	179	144,855.44	809.25
COCAINE	9041L	16	31.28	1.96
CODEINE	9050	52	3,739.11	71.91
BUPRENORPHINE	9064	576	97.58	0.17
OXYCODONE	9143	60	156.84	2.61
HYDROMORPHONE	9150	379	141.79	0.37
HYDROCODONE	9193	286	690.89	2.42
MEPERIDINE (PETHIDINE)	9230	87	405.14	4.66
METHADONE	9250B	11	35.06	3.19
MORPHINE	9300	200	330.25	1.65

OPIUM POWDERED	9639	6	19.8	3.3
OXYMORPHONE	9652	12	0.68	0.06
NOROXYMORPHONE	9668	2	2	1
ALFENTANIL	9737	4	1.69	0.42
REMIFENTANIL	9739	37	4.57	0.12
SUFENTANIL BASE	9740	5	0.02	0
FENTANYL BASE	9801	362	37.5	0.1

STATE:OREGON BUSINESS ACTIVITY:D - TEACHING INSTITUTIONS

DRUG NAME	DRUG CODE	BUYERS	TOTAL GRAMS	AVG GRAMS
D-METHAMPHETAMINE	1105D	1	4.02	4.02
COCAINE	9041L	1	0.89	0.89
BUPRENORPHINE	9064	1	0.04	0.04
OXYCODONE	9143	1	0.45	0.45
FENTANYL BASE	9801	1	0.03	0.03

STATE:OREGON BUSINESS ACTIVITY:M - MID-LEVEL PRACTITIONERS

DRUG NAME	DRUG CODE	BUYERS	TOTAL GRAMS	AVG GRAMS
PENTOBARBITAL (SCHEDULE 2)	2270	9	7,646.65	849.63
CODEINE	9050	5	31.16	6.23
BUPRENORPHINE	9064	2	2.37	1.19
HYDROMORPHONE	9150	5	0.27	0.05
HYDROCODONE	9193	7	17.86	2.55
MEPERIDINE (PETHIDINE)	9230	1	2.18	2.18
MORPHINE	9300	3	0.56	0.19
FENTANYL BASE	9801	1	0	0

STATE:OREGON BUSINESS ACTIVITY:N-U NARCOTIC TREATMENT PROGRAMS

DRUG NAME	DRUG CODE	BUYERS	TOTAL GRAMS	AVG GRAMS
BUPRENORPHINE	9064	15	5,211.37	347.42
METHADONE	9250B	17	188,560.09	11,091.77

DATE RANGE: 01/01/2017 TO 12/31/2017

ARCOS 3 - REPORT 5

STATISTICAL SUMMARY FOR RETAIL DRUG PURCHASES BY GRAMS WT

Run Date: 07/03/2018

STATE:PENNSYLVANIA

BUSINESS ACTIVITY:A - PHARMACIES

DRUG NAME	DRUG CODE	BUYERS	TOTAL GRAMS	AVG GRAMS
AMPHETAMINE	1100	2,950	832,126.70	282.08
DL-METHAMPHETAMINE RACEMIC BASE	1105B	92	489.05	5.32
D-METHAMPHETAMINE	1105D	3	26.11	8.7
LISDEXAMFETAMINE	1205	2,742	340,381.23	124.14
METHYLPHENIDATE (DL;D;L;ISOMERS)	1724	2,920	886,809.58	303.7
BARBITURIC ACID DERIVIATIVE OR SALT [PER 21C	2100	634	21,590	34.05
BUTALBITAL	2165	1,203	46,575	38.72
PENTOBARBITAL (SCHEDULE 2)	2270	1	53.77	53.77
SECOBARBITAL (SCHEDULE 2)	2315	9	265.5	29.5
DRONABINOL IN AN ORAL SOLUTION IN FDA APPR	7365	10	16.35	1.64
NABILONE	7379	2	0.35	0.18
COCAINE	9041L	8	17.25	2.16
CODEINE	9050	2,974	511,587.23	172.02
BUPRENORPHINE	9064	2,780	242,773.12	87.33
DIHYDROCODEINE	9120	55	201.88	3.67
OXYCODONE	9143	3,024	2,878,775.18	951.98
HYDROMORPHONE	9150	2,620	49,703.35	18.97
HYDROCODONE	9193	3,000	631,977.42	210.66
LEVORPHANOL	9220L	68	49.8	0.73
MEPERIDINE (PETHIDINE)	9230	463	11,654.13	25.17
METHADONE	9250B	2,292	127,217.34	55.5
MORPHINE	9300	2,925	863,020.28	295.05
OPIUM TINCTURE (LAUDANUM)	9630	4	21.24	5.31
OPIUM POWDERED	9639	174	570.96	3.28
OXYMORPHONE	9652	1,908	89,999.06	47.17
ALFENTANIL	9737	1	0.02	0.02
REMIFENTANIL	9739	4	1.55	0.39
SUFENTANIL BASE	9740	2	6.7	3.35
TAPENTADOL	9780	1,815	262,376.50	144.56
FENTANYL BASE	9801	2,862	18,307.32	6.4
OPIUM COMBINATION PRODUCT (C-III)	9809	117	130.74	1.12

STATE:PENNSYLVANIA BUSINESS ACTIVITY:B - HOSPITALS

DRUG NAME	DRUG CODE	BUYERS	TOTAL GRAMS	AVG GRAMS
AMPHETAMINE	1100	166	12,200.01	73.49
LISDEXAMFETAMINE	1205	52	3,201.52	61.57
METHYLPHENIDATE (DL;D;L;ISOMERS)	1724	242	12,974.45	53.61
BARBITURIC ACID DERIVIATIVE OR SALT [PER 21C	2100	4	85	21.25
AMOBARBITAL (SCHEDULE 2)	2125	11	29.16	2.65
BUTALBITAL	2165	13	225	17.31
PENTOBARBITAL (SCHEDULE 2)	2270	30	2,568.33	85.61
COCAINE	9041L	157	1,297.27	8.26
CODEINE	9050	281	15,831.62	56.34
BUPRENORPHINE	9064	198	7,082.32	35.77
OXYCODONE	9143	331	122,487.64	370.05
HYDROMORPHONE	9150	301	10,530.99	34.99
HYDROCODONE	9193	298	26,864.39	90.15
LEVORPHANOL	9220L	1	3.83	3.83
MEPERIDINE (PETHIDINE)	9230	225	2,169.86	9.64
METHADONE	9250B	242	13,768.20	56.89
MORPHINE	9300	327	68,764	210.29
OPIUM POWDERED	9639	159	727.92	4.58
OXYMORPHONE	9652	78	1,067.49	13.69
ALFENTANIL	9737	13	0.97	0.07
REMIFENTANIL	9739	116	138.94	1.2
SUFENTANIL BASE	9740	44	1.37	0.03
TAPENTADOL	9780	63	2,094.50	33.25
FENTANYL BASE	9801	365	1,678.74	4.6
OPIUM COMBINATION PRODUCT (C-III)	9809	12	4.91	0.41

STATE:PENNSYLVANIA BUSINESS ACTIVITY:C - PRACTITIONERS

DRUG NAME	DRUG CODE	BUYERS	TOTAL GRAMS	AVG GRAMS
AMPHETAMINE	1100	1	8.26	8.26
LISDEXAMFETAMINE	1205	1	18.5	18.5
METHYLPHENIDATE (DL;D;L;ISOMERS)	1724	1	30.29	30.29

PENTOBARBITAL (SCHEDULE 2)	2270	301	262,836.57	873.21
COCAINE	9041L	24	242.2	10.09
CODEINE	9050	321	26,017.67	81.05
BUPRENORPHINE	9064	994	1,728.07	1.74
DIHYDROCODEINE	9120	55	157.49	2.86
OXYCODONE	9143	69	804.83	11.66
HYDROMORPHONE	9150	467	263.88	0.57
HYDROCODONE	9193	393	1,185.12	3.02
MEPERIDINE (PETHIDINE)	9230	111	375.18	3.38
METHADONE	9250B	32	191.76	5.99
MORPHINE	9300	255	408.99	1.6
OPIUM POWDERED	9639	1	1.44	1.44
OXYMORPHONE	9652	3	0.08	0.03
NOROXYMORPHONE	9668	1	0.99	0.99
ALFENTANIL	9737	6	1.02	0.17
REMIFENTANIL	9739	26	2.63	0.1
SUFENTANIL BASE	9740	5	0.05	0.01
FENTANYL BASE	9801	611	77.23	0.13

STATE:PENNSYLVANIA BUSINESS ACTIVITY:D - TEACHING INSTITUTIONS

DRUG NAME	DRUG CODE	BUYERS	TOTAL GRAMS	AVG GRAMS
METHYLPHENIDATE (DL;D;L;ISOMERS)	1724	1	0.43	0.43
PENTOBARBITAL (SCHEDULE 2)	2270	1	3,732.18	3,732.18
CODEINE	9050	1	964.06	964.06
BUPRENORPHINE	9064	2	1.41	0.7
HYDROMORPHONE	9150	2	3.22	1.61
HYDROCODONE	9193	1	61.63	61.63
METHADONE	9250B	1	35.06	35.06
MORPHINE	9300	2	36.81	18.41
OXYMORPHONE	9652	1	0.18	0.18
REMIFENTANIL	9739	2	0.12	0.06
FENTANYL BASE	9801	4	3.23	0.81

STATE:PENNSYLVANIA BUSINESS ACTIVITY:M - MID-LEVEL PRACTITIONERS

DRUG NAME	DRUG CODE	BUYERS	TOTAL GRAMS	AVG GRAMS
CODEINE	9050	32	64.37	2.01
DIHYDROCODEINE	9120	10	3.18	0.32

STATE:PENNSYLVANIA BUSINESS ACTIVITY:N-U NARCOTIC TREATMENT PROGRAMS

DRUG NAME	DRUG CODE	BUYERS	TOTAL GRAMS	AVG GRAMS
BUPRENORPHINE	9064	15	502.79	33.52
METHADONE	9250B	85	648,095.21	7,624.65

STATE:PUERTO RICO BUSINESS ACTIVITY:A - PHARMACIES

DRUG NAME	DRUG CODE	BUYERS	TOTAL GRAMS	AVG GRAMS
AMPHETAMINE	1100	675	32,974.30	48.85
LISDEXAMFETAMINE	1205	306	7,605.48	24.85
METHYLPHENIDATE (DL;D;L;ISOMERS)	1724	732	67,579.14	92.32
BARBITURIC ACID DERIVIATIVE OR SALT [PER 21C	2100	29	605	20.86
CODEINE	9050	1,076	46,967.94	43.65
BUPRENORPHINE	9064	336	9,371.98	27.89
OXYCODONE	9143	827	44,087.66	53.31
HYDROMORPHONE	9150	30	187.78	6.26
HYDROCODONE	9193	547	2,349.42	4.3
MEPERIDINE (PETHIDINE)	9230	680	5,967.49	8.78
METHADONE	9250B	15	229.59	15.31
MORPHINE	9300	152	3,974.06	26.15
TAPENTADOL	9780	71	2,733.50	38.5
FENTANYL BASE	9801	665	408.72	0.61

STATE:PUERTO RICO BUSINESS ACTIVITY:B - HOSPITALS

DRUG NAME	DRUG CODE	BUYERS	TOTAL GRAMS	AVG GRAMS
AMPHETAMINE	1100	8	140.93	17.62
METHYLPHENIDATE (DL;D;L;ISOMERS)	1724	17	285.65	16.8

CODEINE	9050	82	4,257.76	51.92
BUPRENORPHINE	9064	16	583.42	36.46
OXYCODONE	9143	67	6,638.85	99.09
HYDROMORPHONE	9150	5	108.85	21.77
HYDROCODONE	9193	26	197.27	7.59
MEPERIDINE (PETHIDINE)	9230	95	11,355.32	119.53
METHADONE	9250B	1	118.96	118.96
MORPHINE	9300	85	4,423.44	52.04
SUFENTANIL BASE	9740	12	0.75	0.06
TAPENTADOL	9780	5	640.5	128.1
FENTANYL BASE	9801	81	169.13	2.09

STATE:PUERTO RICO BUSINESS ACTIVITY:C - PRACTITIONERS

DRUG NAME	DRUG CODE	BUYERS	TOTAL GRAMS	AVG GRAMS
BUPRENORPHINE	9064	14	0.92	0.07
MEPERIDINE (PETHIDINE)	9230	5	49.02	9.8
MORPHINE	9300	1	2.24	2.24
NOROXYMORPHONE	9668	2	25.07	12.53
FENTANYL BASE	9801	10	0.42	0.04

STATE:PUERTO RICO BUSINESS ACTIVITY:D - TEACHING INSTITUTIONS

DRUG NAME	DRUG CODE	BUYERS	TOTAL GRAMS	AVG GRAMS
PENTOBARBITAL (SCHEDULE 2)	2270	1	0.2	0.2

STATE:PUERTO RICO BUSINESS ACTIVITY:N-U NARCOTIC TREATMENT PROGRAMS

DRUG NAME	DRUG CODE	BUYERS	TOTAL GRAMS	AVG GRAMS
METHADONE	9250B	6	83,680.06	13,946.68

STATE:RHODE ISLAND BUSINESS ACTIVITY:A - PHARMACIES

DRUG NAME	DRUG CODE	BUYERS	TOTAL GRAMS	AVG GRAMS
AMPHETAMINE	1100	189	144,014.30	761.98
DL-METHAMPHETAMINE RACEMIC BASE	1105B	5	16.88	3.38
D-METHAMPHETAMINE	1105D	1	3.62	3.62
LISDEXAMFETAMINE	1205	179	29,753.07	166.22
METHYLPHENIDATE (DL;D;L;ISOMERS)	1724	187	72,332.93	386.81
BARBITURIC ACID DERIVIATIVE OR SALT [PER 21C	2100	71	2,515	35.42
BUTALBITAL	2165	72	1,420	19.72
SECOBARBITAL (SCHEDULE 2)	2315	1	36.62	36.62
CODEINE	9050	184	27,925.75	151.77
BUPRENORPHINE	9064	191	28,706.51	150.3
DIHYDROCODEINE	9120	1	3.2	3.2
OXYCODONE	9143	190	152,626.40	803.3
HYDROMORPHONE	9150	167	2,902.61	17.38
HYDROCODONE	9193	190	39,272.47	206.7
MEPERIDINE (PETHIDINE)	9230	20	634.02	31.7
METHADONE	9250B	152	6,364.46	41.87
MORPHINE	9300	187	41,001.53	219.26
OPIUM POWDERED	9639	20	30.24	1.51
OXYMORPHONE	9652	77	1,601.14	20.79
TAPENTADOL	9780	104	8,380	80.58
FENTANYL BASE	9801	176	590.9	3.36
OPIUM COMBINATION PRODUCT (C-III)	9809	2	0.38	0.19

STATE:RHODE ISLAND BUSINESS ACTIVITY:B - HOSPITALS

DRUG NAME	DRUG CODE	BUYERS	TOTAL GRAMS	AVG GRAMS
AMPHETAMINE	1100	16	2,524.82	157.8
LISDEXAMFETAMINE	1205	8	358.42	44.8
METHYLPHENIDATE (DL;D;L;ISOMERS)	1724	18	2,966.34	164.8
BARBITURIC ACID DERIVIATIVE OR SALT [PER 21C	2100	1	5	5
AMOBARBITAL (SCHEDULE 2)	2125	1	3.65	3.65
BUTALBITAL	2165	2	50	25
PENTOBARBITAL (SCHEDULE 2)	2270	2	37.37	18.68
COCAINE	9041L	11	63.57	5.78
CODEINE	9050	18	1,190.99	66.17

BUPRENORPHINE	9064	16	677.79	42.36
OXYCODONE	9143	21	9,891.65	471.03
HYDROMORPHONE	9150	15	614.15	40.94
HYDROCODONE	9193	20	2,129.09	106.45
MEPERIDINE (PETHIDINE)	9230	13	304.37	23.41
METHADONE	9250B	14	1,317.59	94.11
MORPHINE	9300	19	5,552.54	292.24
OPIUM POWDERED	9639	11	100.8	9.16
OXYMORPHONE	9652	1	0.27	0.27
ALFENTANIL	9737	2	0.26	0.13
REMIFENTANIL	9739	6	2.85	0.48
SUFENTANIL BASE	9740	3	0.01	0
TAPENTADOL	9780	1	10	10
FENTANYL BASE	9801	18	135.22	7.51

STATE:RHODE ISLAND BUSINESS ACTIVITY:C - PRACTITIONERS

DRUG NAME	DRUG CODE	BUYERS	TOTAL GRAMS	AVG GRAMS
PENTOBARBITAL (SCHEDULE 2)	2270	35	24,525.04	700.72
COCAINE	9041L	2	12.43	6.21
BUPRENORPHINE	9064	75	5.48	0.07
OXYCODONE	9143	2	4.03	2.02
HYDROMORPHONE	9150	44	16.2	0.37
HYDROCODONE	9193	29	86.09	2.97
MEPERIDINE (PETHIDINE)	9230	14	68.41	4.89
METHADONE	9250B	3	16.28	5.43
MORPHINE	9300	13	74.44	5.73
OPIUM POWDERED	9639	2	6.48	3.24
OXYMORPHONE	9652	4	0.6	0.15
NOROXYMORPHONE	9668	1	0.1	0.1
REMIFENTANIL	9739	1	0.32	0.32
FENTANYL BASE	9801	48	8.86	0.18

STATE:RHODE ISLAND BUSINESS ACTIVITY:N-U NARCOTIC TREATMENT PROGRAMS

DRUG NAME	DRUG CODE	BUYERS	TOTAL GRAMS	AVG GRAMS
BUPRENORPHINE	9064	13	707.51	54.42
METHADONE	9250B	19	169,410.63	8,916.35

STATE:SOUTH CAROLINA BUSINESS ACTIVITY:A - PHARMACIES

DRUG NAME	DRUG CODE	BUYERS	TOTAL GRAMS	AVG GRAMS
AMPHETAMINE	1100	1,107	535,663.09	483.89
DL-METHAMPHETAMINE RACEMIC BASE	1105B	27	56.66	2.1
LISDEXAMFETAMINE	1205	1,079	242,733.78	224.96
METHYLPHENIDATE (DL;D;L;ISOMERS)	1724	1,100	343,576.83	312.34
BARBITURIC ACID DERIVIATIVE OR SALT [PER 21C	2100	324	9,390	28.98
BUTALBITAL	2165	443	13,475	30.42
SECOBARBITAL (SCHEDULE 2)	2315	3	36.62	12.21
COCAINE	9041L	1	0.89	0.89
CODEINE	9050	1,097	298,483.53	272.09
BUPRENORPHINE	9064	1,015	40,272.55	39.68
DIHYDROCODEINE	9120	11	52.34	4.76
OXYCODONE	9143	1,106	904,896.83	818.17
HYDROMORPHONE	9150	1,021	19,550.41	19.15
HYDROCODONE	9193	1,120	534,772.16	477.48
LEVORPHANOL	9220L	23	11.03	0.48
MEPERIDINE (PETHIDINE)	9230	729	19,518.11	26.77
METHADONE	9250B	882	36,890.33	41.83
MORPHINE	9300	1,094	238,347.35	217.87
OPIUM POWDERED	9639	62	158.4	2.55
OXYMORPHONE	9652	813	23,968.81	29.48
SUFENTANIL BASE	9740	1	2	2
TAPENTADOL	9780	943	172,597	183.03
FENTANYL BASE	9801	1,067	4,749.77	4.45
OPIUM COMBINATION PRODUCT (C-III)	9809	43	37.84	0.88

STATE:SOUTH CAROLINA BUSINESS ACTIVITY:B - HOSPITALS

DRUG NAME	DRUG CODE	BUYERS	TOTAL GRAMS	AVG GRAMS

AMPHETAMINE	1100	55	9,528.35	173.24
DL-METHAMPHETAMINE RACEMIC BASE	1105B	1	3.62	3.62
D-METHAMPHETAMINE	1105D	1	8.84	8.84
LISDEXAMFETAMINE	1205	25	1,678.80	67.15
METHYLPHENIDATE (DL;D;L;ISOMERS)	1724	80	10,195.35	127.44
GAMMA HYDROXYBUTYRIC ACID(FDA APPROVED	2012	1	5,126.98	5,126.98
BARBITURIC ACID DERIVIATIVE OR SALT [PER 21C	2100	1	5	5
AMOBARBITAL (SCHEDULE 2)	2125	3	15.04	5.01
BUTALBITAL	2165	1	5	5
PENTOBARBITAL (SCHEDULE 2)	2270	10	3,118.36	311.84
DRONABINOL IN AN ORAL SOLUTION IN FDA APPR	7365	1	0.3	0.3
COCAINE	9041L	47	113.99	2.43
CODEINE	9050	91	5,258.73	57.79
BUPRENORPHINE	9064	67	924.79	13.8
OXYCODONE	9143	132	29,595.87	224.21
HYDROMORPHONE	9150	144	3,315.90	23.03
HYDROCODONE	9193	139	15,502.34	111.53
MEPERIDINE (PETHIDINE)	9230	122	3,443.84	28.23
METHADONE	9250B	78	2,303.62	29.53
MORPHINE	9300	150	22,845.74	152.3
OPIUM POWDERED	9639	59	396.72	6.72
OXYMORPHONE	9652	8	33.81	4.23
ALFENTANIL	9737	2	0.35	0.18
REMIFENTANIL	9739	30	18.43	0.61
SUFENTANIL BASE	9740	26	0.62	0.02
TAPENTADOL	9780	29	1,413	48.72
FENTANYL BASE	9801	166	496.04	2.99
OPIUM COMBINATION PRODUCT (C-III)	9809	4	2.27	0.57

STATE:SOUTH CAROLINA BUSINESS ACTIVITY:C - PRACTITIONERS

DRUG NAME	DRUG CODE	BUYERS	TOTAL GRAMS	AVG GRAMS
AMPHETAMINE	1100	2	1.48	0.74
LISDEXAMFETAMINE	1205	2	18.7	9.35
GAMMA HYDROXYBUTYRIC ACID(FDA APPROVED	2012	1	2,452.03	2,452.03
PENTOBARBITAL (SCHEDULE 2)	2270	78	52,596.39	674.31

COCAINE	9041L	2	3.43	1.71
CODEINE	9050	26	285.94	11
BUPRENORPHINE	9064	378	58.94	0.16
OXYCODONE	9143	9	344.63	38.29
HYDROMORPHONE	9150	149	110.05	0.74
HYDROCODONE	9193	79	245.53	3.11
MEPERIDINE (PETHIDINE)	9230	70	555.25	7.93
METHADONE	9250B	2	0.89	0.45
MORPHINE	9300	112	226.25	2.02
OXYMORPHONE	9652	1	0.03	0.03
REMIFENTANIL	9739	4	0.22	0.06
SUFENTANIL BASE	9740	1	0.02	0.02
FENTANYL BASE	9801	228	15.01	0.07

STATE:SOUTH CAROLINA BUSINESS ACTIVITY:D - TEACHING INSTITUTIONS

DRUG NAME	DRUG CODE	BUYERS	TOTAL GRAMS	AVG GRAMS
BUPRENORPHINE	9064	1	0.02	0.02
HYDROMORPHONE	9150	1	0.07	0.07

STATE:SOUTH CAROLINA BUSINESS ACTIVITY:M - MID-LEVEL PRACTITIONERS

DRUG NAME	DRUG CODE	BUYERS	TOTAL GRAMS	AVG GRAMS
CODEINE	9050	6	7.8	1.3
MORPHINE	9300	62	26.7	0.43
FENTANYL BASE	9801	66	0.71	0.01

STATE:SOUTH CAROLINA BUSINESS ACTIVITY:N-U NARCOTIC TREATMENT PROGRAMS

DRUG NAME	DRUG CODE	BUYERS	TOTAL GRAMS	AVG GRAMS
BUPRENORPHINE	9064	14	1,382.48	98.75
METHADONE	9250B	23	186,183.22	8,094.92

STATISTICAL SUMMARY FOR RETAIL DRUG PURCHASES BY GRAMS WT

STATE:SOUTH DAKOTA BUSINESS ACTIVITY:A - PHARMACIES

DRUG NAME	DRUG CODE	BUYERS	TOTAL GRAMS	AVG GRAMS
AMPHETAMINE	1100	192	57,976.94	301.96
DL-METHAMPHETAMINE RACEMIC BASE	1105B	9	69.52	7.72
LISDEXAMFETAMINE	1205	190	43,450	228.68
METHYLPHENIDATE (DL;D;L;ISOMERS)	1724	193	89,268.30	462.53
BARBITURIC ACID DERIVIATIVE OR SALT [PER 21C	2100	31	900	29.03
BUTALBITAL	2165	77	2,590	33.64
SECOBARBITAL (SCHEDULE 2)	2315	1	9.16	9.16
COCAINE	9041L	1	0.14	0.14
CODEINE	9050	197	24,547.87	124.61
BUPRENORPHINE	9064	140	1,566	11.19
DIHYDROCODEINE	9120	1	1.07	1.07
OXYCODONE	9143	195	61,411.10	314.93
HYDROMORPHONE	9150	167	1,509.62	9.04
HYDROCODONE	9193	197	48,729.17	247.36
MEPERIDINE (PETHIDINE)	9230	49	1,271.41	25.95
METHADONE	9250B	145	4,672.35	32.22
MORPHINE	9300	191	26,222.82	137.29
OPIUM POWDERED	9639	25	111.24	4.45
OXYMORPHONE	9652	84	1,854.90	22.08
SUFENTANIL BASE	9740	1	0	0
TAPENTADOL	9780	140	17,807.50	127.2
FENTANYL BASE	9801	194	1,019.97	5.26
OPIUM COMBINATION PRODUCT (C-III)	9809	4	2.65	0.66

STATE:SOUTH DAKOTA BUSINESS ACTIVITY:B - HOSPITALS

DRUG NAME	DRUG CODE	BUYERS	TOTAL GRAMS	AVG GRAMS
AMPHETAMINE	1100	23	2,833.81	123.21
LISDEXAMFETAMINE	1205	17	385.59	22.68
METHYLPHENIDATE (DL;D;L;ISOMERS)	1724	36	3,716.87	103.25
BARBITURIC ACID DERIVIATIVE OR SALT [PER 21C	2100	1	5	5
BUTALBITAL	2165	1	5	5
PENTOBARBITAL (SCHEDULE 2)	2270	1	11.39	11.39

COCAINE	9041L	21	148.7	7.08
CODEINE	9050	45	3,253.32	72.3
BUPRENORPHINE	9064	19	230.21	12.12
OXYCODONE	9143	73	7,346.96	100.64
HYDROMORPHONE	9150	63	630.87	10.01
HYDROCODONE	9193	76	10,431.18	137.25
MEPERIDINE (PETHIDINE)	9230	56	671.6	11.99
METHADONE	9250B	26	522.24	20.09
MORPHINE	9300	74	8,523.35	115.18
OPIUM POWDERED	9639	21	65.88	3.14
OXYMORPHONE	9652	7	57.25	8.18
ALFENTANIL	9737	5	0.33	0.07
REMIFENTANIL	9739	9	1.8	0.2
SUFENTANIL BASE	9740	7	0.18	0.03
TAPENTADOL	9780	11	1,052.50	95.68
FENTANYL BASE	9801	77	179.88	2.34

STATE:SOUTH DAKOTA BUSINESS ACTIVITY:C - PRACTITIONERS

DRUG NAME	DRUG CODE	BUYERS	TOTAL GRAMS	AVG GRAMS
PENTOBARBITAL (SCHEDULE 2)	2270	13	24,892.61	1,914.82
CODEINE	9050	2	83.98	41.99
BUPRENORPHINE	9064	61	1.57	0.03
OXYCODONE	9143	5	187.31	37.46
HYDROMORPHONE	9150	29	59.18	2.04
HYDROCODONE	9193	21	46.66	2.22
MEPERIDINE (PETHIDINE)	9230	11	133.23	12.11
MORPHINE	9300	25	54.3	2.17
OPIUM POWDERED	9639	1	3.6	3.6
OXYMORPHONE	9652	1	0.03	0.03
REMIFENTANIL	9739	2	0.09	0.05
FENTANYL BASE	9801	41	4.25	0.1

STATE:SOUTH DAKOTA BUSINESS ACTIVITY:D - TEACHING INSTITUTIONS

DRUG NAME	DRUG CODE	BUYERS	TOTAL GRAMS	AVG GRAMS
AMPHETAMINE	1100	1	1.47	1.47
PENTOBARBITAL (SCHEDULE 2)	2270	1	88.86	88.86
BUPRENORPHINE	9064	2	0.01	0

STATE:SOUTH DAKOTA BUSINESS ACTIVITY:M - MID-LEVEL PRACTITIONERS

DRUG NAME	DRUG CODE	BUYERS	TOTAL GRAMS	AVG GRAMS
HYDROMORPHONE	9150	1	0.04	0.04
MORPHINE	9300	4	0.59	0.15
FENTANYL BASE	9801	6	0.13	0.02

STATE:SOUTH DAKOTA BUSINESS ACTIVITY:N-U NARCOTIC TREATMENT PROGRAMS

DRUG NAME	DRUG CODE	BUYERS	TOTAL GRAMS	AVG GRAMS
METHADONE	9250B	1	3,563.29	3,563.29

STATE:TENNESSEE BUSINESS ACTIVITY:A - PHARMACIES

DRUG NAME	DRUG CODE	BUYERS	TOTAL GRAMS	AVG GRAMS
AMPHETAMINE	1100	1,567	514,247.55	328.17
DL-METHAMPHETAMINE RACEMIC BASE	1105B	11	50.63	4.6
D-METHAMPHETAMINE	1105D	1	0.4	0.4
LISDEXAMFETAMINE	1205	1,498	277,271.79	185.09
METHYLPHENIDATE (DL;D;L;ISOMERS)	1724	1,536	329,808.61	214.72
BARBITURIC ACID DERIVIATIVE OR SALT [PER 21C	2100	468	11,265	24.07
AMOBARBITAL (SCHEDULE 2)	2125	1	0.46	0.46
BUTALBITAL	2165	477	15,200	31.87
SECOBARBITAL (SCHEDULE 2)	2315	1	9.16	9.16
DRONABINOL IN AN ORAL SOLUTION IN FDA APPR	7365	6	3.6	0.6
COCAINE	9041L	3	9.82	3.27
CODEINE	9050	1,530	333,893.63	218.23
BUPRENORPHINE	9064	1,472	136,431.14	92.68
DIHYDROCODEINE	9120	306	1,848.98	6.04

OXYCODONE	9143	1,580	1,760,211.38	1,114.06
HYDROMORPHONE	9150	1,334	21,102.49	15.82
HYDROCODONE	9193	1,588	921,316.31	580.17
LEVORPHANOL	9220L	3	0.7	0.23
MEPERIDINE (PETHIDINE)	9230	990	24,803.54	25.05
METHADONE	9250B	1,052	39,775.04	37.81
MORPHINE	9300	1,560	604,791.01	387.69
OPIUM POWDERED	9639	106	184.68	1.74
OXYMORPHONE	9652	1,202	81,770.78	68.03
REMIFENTANIL	9739	1	0.03	0.03
SUFENTANIL BASE	9740	2	1.2	0.6
TAPENTADOL	9780	1,178	125,409.50	106.46
FENTANYL BASE	9801	1,516	6,831.63	4.51
OPIUM COMBINATION PRODUCT (C-III)	9809	53	23.84	0.45

STATE:TENNESSEE BUSINESS ACTIVITY:B - HOSPITALS

DRUG NAME	DRUG CODE	BUYERS	TOTAL GRAMS	AVG GRAMS
AMPHETAMINE	1100	81	5,471.92	67.55
LISDEXAMFETAMINE	1205	39	2,497.97	64.05
METHYLPHENIDATE (DL;D;L;ISOMERS)	1724	117	5,233.94	44.73
GAMMA HYDROXYBUTYRIC ACID(FDA APPROVED	2012	1	2,377.73	2,377.73
BARBITURIC ACID DERIVIATIVE OR SALT [PER 21C	2100	5	40	8
AMOBARBITAL (SCHEDULE 2)	2125	3	15.49	5.16
BUTALBITAL	2165	4	65	16.25
PENTOBARBITAL (SCHEDULE 2)	2270	13	4,271.73	328.59
COCAINE	9041L	82	491.13	5.99
CODEINE	9050	144	4,363.51	30.3
BUPRENORPHINE	9064	123	1,816.09	14.76
DIHYDROCODEINE	9120	2	6.41	3.2
OXYCODONE	9143	178	90,341.63	507.54
HYDROMORPHONE	9150	165	5,344	32.39
HYDROCODONE	9193	193	50,195.15	260.08
MEPERIDINE (PETHIDINE)	9230	137	3,926.67	28.66
METHADONE	9250B	116	2,476.49	21.35
MORPHINE	9300	180	44,701.49	248.34

OPIUM POWDERED	9639	87	514.08	5.91
OXYMORPHONE	9652	37	462.2	12.49
ALFENTANIL	9737	6	2.21	0.37
REMIFENTANIL	9739	46	21.72	0.47
SUFENTANIL BASE	9740	49	1.3	0.03
TAPENTADOL	9780	42	1,800.50	42.87
FENTANYL BASE	9801	171	809.93	4.74
OPIUM COMBINATION PRODUCT (C-III)	9809	3	1.14	0.38

STATE:TENNESSEE BUSINESS ACTIVITY:C - PRACTITIONERS

DRUG NAME	DRUG CODE	BUYERS	TOTAL GRAMS	AVG GRAMS
AMPHETAMINE	1100	8	719.27	89.91
LISDEXAMFETAMINE	1205	8	829.4	103.68
METHYLPHENIDATE (DL;D;L;ISOMERS)	1724	3	600.25	200.08
PENTOBARBITAL (SCHEDULE 2)	2270	168	117,425.20	698.96
COCAINE	9041L	23	169.06	7.35
CODEINE	9050	56	3,121.60	55.74
BUPRENORPHINE	9064	493	41.09	0.08
DIHYDROCODEINE	9120	64	46.11	0.72
OXYCODONE	9143	78	589.92	7.56
HYDROMORPHONE	9150	272	209.91	0.77
HYDROCODONE	9193	267	1,335.39	5
MEPERIDINE (PETHIDINE)	9230	159	939.69	5.91
METHADONE	9250B	10	11.54	1.15
MORPHINE	9300	172	503.89	2.93
OPIUM POWDERED	9639	9	61.2	6.8
OXYMORPHONE	9652	3	1.2	0.4
NOROXYMORPHONE	9668	1	0.13	0.13
ALFENTANIL	9737	2	2.56	1.28
REMIFENTANIL	9739	9	1.49	0.17
SUFENTANIL BASE	9740	4	0.06	0.01
TAPENTADOL	9780	1	5	5
FENTANYL BASE	9801	410	46.54	0.11

STATISTICAL SUMMARY FOR RETAIL DRUG PURCHASES BY GRAMS WT

STATE:TENNESSEE BUSINESS ACTIVITY:D - TEACHING INSTITUTIONS

DRUG NAME	DRUG CODE	BUYERS	TOTAL GRAMS	AVG GRAMS
BUPRENORPHINE	9064	2	0.13	0.06
FENTANYL BASE	9801	1	0	0

STATE:TENNESSEE BUSINESS ACTIVITY:M - MID-LEVEL PRACTITIONERS

DRUG NAME	DRUG CODE	BUYERS	TOTAL GRAMS	AVG GRAMS
PENTOBARBITAL (SCHEDULE 2)	2270	24	20,896.58	870.69
CODEINE	9050	13	105.39	8.11
DIHYDROCODEINE	9120	55	35.53	0.65
HYDROCODONE	9193	1	1.21	1.21
MEPERIDINE (PETHIDINE)	9230	10	22.22	2.22
MORPHINE	9300	62	26.55	0.43
FENTANYL BASE	9801	78	1.3	0.02

STATE:TENNESSEE BUSINESS ACTIVITY:N-U NARCOTIC TREATMENT PROGRAMS

DRUG NAME	DRUG CODE	BUYERS	TOTAL GRAMS	AVG GRAMS
BUPRENORPHINE	9064	10	997.32	99.73
METHADONE	9250B	13	152,040.40	11,695.42

STATE:TEXAS BUSINESS ACTIVITY:A - PHARMACIES

DRUG NAME	DRUG CODE	BUYERS	TOTAL GRAMS	AVG GRAMS
AMPHETAMINE	1100	4,595	1,620,453.17	352.66
DL-METHAMPHETAMINE RACEMIC BASE	1105B	164	423.95	2.59
D-METHAMPHETAMINE	1105D	13	34.14	2.63
LISDEXAMFETAMINE	1205	4,431	1,079,841.66	243.7
METHYLPHENIDATE (DL;D;L;ISOMERS)	1724	4,565	1,287,419.38	282.02
BARBITURIC ACID DERIVIATIVE OR SALT [PER 21C	2100	1,643	49,025	29.84
BUTALBITAL	2165	2,065	74,475	36.07
PENTOBARBITAL (SCHEDULE 2)	2270	3	7.75	2.58

SECOBARBITAL (SCHEDULE 2)	2315	3	36.62	12.21
DRONABINOL IN AN ORAL SOLUTION IN FDA APPR	7365	13	11.7	0.9
COCAINE	9041L	13	34.53	2.66
CODEINE	9050	4,932	3,881,704.12	787.04
BUPRENORPHINE	9064	4,200	99,861.65	23.78
DIHYDROCODEINE	9120	1,565	10,415.63	6.66
OXYCODONE	9143	4,514	1,262,157.71	279.61
HYDROMORPHONE	9150	4,014	79,229.41	19.74
HYDROCODONE	9193	4,956	2,501,324.80	504.71
LEVORPHANOL	9220L	128	220.2	1.72
MEPERIDINE (PETHIDINE)	9230	1,084	18,663.61	17.22
METHADONE	9250B	3,472	141,211.37	40.67
MORPHINE	9300	4,342	723,947.66	166.73
OPIUM TINCTURE (LAUDANUM)	9630	7	14.16	2.02
OPIUM POWDERED	9639	159	280.44	1.76
OXYMORPHONE	9652	2,189	45,543.49	20.81
NOROXYMORPHONE	9668	2	5.74	2.87
REMIFENTANIL	9739	3	0.15	0.05
SUFENTANIL BASE	9740	4	2.7	0.67
TAPENTADOL	9780	3,009	384,643.50	127.83
FENTANYL BASE	9801	4,359	19,817.34	4.55
OPIUM COMBINATION PRODUCT (C-III)	9809	84	50.89	0.61

STATE:TEXAS BUSINESS ACTIVITY:B - HOSPITALS

DRUG NAME	DRUG CODE	BUYERS	TOTAL GRAMS	AVG GRAMS
AMPHETAMINE	1100	295	35,401.34	120
DL-METHAMPHETAMINE RACEMIC BASE	1105B	1	2.81	2.81
D-METHAMPHETAMINE	1105D	1	5.62	5.62
LISDEXAMFETAMINE	1205	123	9,090.62	73.91
METHYLPHENIDATE (DL;D;L;ISOMERS)	1724	423	49,170.99	116.24
GAMMA HYDROXYBUTYRIC ACID(FDA APPROVED	2012	1	3,269.38	3,269.38
BARBITURIC ACID DERIVIATIVE OR SALT [PER 21C	2100	23	274.9	11.95
AMOBARBITAL (SCHEDULE 2)	2125	15	83.39	5.56
BUTALBITAL	2165	23	320	13.91
PENTOBARBITAL (SCHEDULE 2)	2270	51	4,768.44	93.5

SECOBARBITAL (SCHEDULE 2)	2315	1	9.16	9.16
COCAINE	9041L	292	1,397.87	4.79
CODEINE	9050	936	120,087.44	128.3
BUPRENORPHINE	9064	284	3,981.37	14.02
DIHYDROCODEINE	9120	7	28.84	4.12
OXYCODONE	9143	730	61,703.86	84.53
HYDROMORPHONE	9150	1,121	15,755.34	14.05
HYDROCODONE	9193	1,203	155,005.93	128.85
LEVORPHANOL	9220L	1	0.12	0.12
MEPERIDINE (PETHIDINE)	9230	802	24,435.38	30.47
METHADONE	9250B	473	11,906.77	25.17
MORPHINE	9300	1,285	96,275.62	74.92
OPIUM POWDERED	9639	322	1,803.24	5.6
OXYMORPHONE	9652	46	619	13.46
NOROXYMORPHONE	9668	2	4.12	2.06
ALFENTANIL	9737	53	20.67	0.39
REMIFENTANIL	9739	193	110.7	0.57
SUFENTANIL BASE	9740	197	3.9	0.02
TAPENTADOL	9780	110	5,444.50	49.5
FENTANYL BASE	9801	1,283	3,075.08	2.4
OPIUM COMBINATION PRODUCT (C-III)	9809	6	2.08	0.35

STATE:TEXAS BUSINESS ACTIVITY:C - PRACTITIONERS

DRUG NAME	DRUG CODE	BUYERS	TOTAL GRAMS	AVG GRAMS
AMPHETAMINE	1100	3	5.17	1.72
LISDEXAMFETAMINE	1205	6	42.78	7.13
METHYLPHENIDATE (DL;D;L;ISOMERS)	1724	3	184.97	61.66
GAMMA HYDROXYBUTYRIC ACID(FDA APPROVED	2012	3	7,876.22	2,625.41
BARBITURIC ACID DERIVIATIVE OR SALT [PER 21C	2100	1	5	5
PENTOBARBITAL (SCHEDULE 2)	2270	357	233,571.43	654.26
COCAINE	9041L	14	21.28	1.52
CODEINE	9050	768	5,435.66	7.08
BUPRENORPHINE	9064	1,877	629.55	0.34
DIHYDROCODEINE	9120	464	461.45	0.99
OXYCODONE	9143	33	454.69	13.78

HYDROMORPHONE	9150	770	411.98	0.54
HYDROCODONE	9193	841	1,974.04	2.35
MEPERIDINE (PETHIDINE)	9230	415	3,226.77	7.78
METHADONE	9250B	32	56.88	1.78
MORPHINE	9300	514	781.74	1.52
THEBAINE	9333	1	25	25
OPIUM POWDERED	9639	2	5.04	2.52
OXYMORPHONE	9652	7	5.59	0.8
NOROXYMORPHONE	9668	8	98.98	12.37
ALFENTANIL	9737	12	1.37	0.11
REMIFENTANIL	9739	21	0.93	0.04
SUFENTANIL BASE	9740	7	0.06	0.01
TAPENTADOL	9780	1	15	15
FENTANYL BASE	9801	1,341	84.08	0.06

STATE:TEXAS BUSINESS ACTIVITY:D - TEACHING INSTITUTIONS

DRUG NAME	DRUG CODE	BUYERS	TOTAL GRAMS	AVG GRAMS
PENTOBARBITAL (SCHEDULE 2)	2270	2	2,843.57	1,421.78
BUPRENORPHINE	9064	4	0.73	0.18
HYDROMORPHONE	9150	1	8.44	8.44
HYDROCODONE	9193	1	3.28	3.28
METHADONE	9250B	1	13.59	13.59
MORPHINE	9300	2	6.55	3.28
OXYMORPHONE	9652	1	0.18	0.18
REMIFENTANIL	9739	1	0.08	0.08
FENTANYL BASE	9801	3	2.49	0.83

STATE:TEXAS BUSINESS ACTIVITY:M - MID-LEVEL PRACTITIONERS

DRUG NAME	DRUG CODE	BUYERS	TOTAL GRAMS	AVG GRAMS
PENTOBARBITAL (SCHEDULE 2)	2270	157	255,401.97	1,626.76
CODEINE	9050	184	331.76	1.8
DIHYDROCODEINE	9120	174	123.27	0.71
HYDROMORPHONE	9150	33	4.64	0.14

HYDROCODONE	9193	1	1.21	1.21
MEPERIDINE (PETHIDINE)	9230	4	4.58	1.14
MORPHINE	9300	376	125.86	0.33
FENTANYL BASE	9801	634	47.13	0.07

STATE:TEXAS BUSINESS ACTIVITY:N-U NARCOTIC TREATMENT PROGRAMS

DRUG NAME	DRUG CODE	BUYERS	TOTAL GRAMS	AVG GRAMS
BUPRENORPHINE	9064	28	863.02	30.82
METHADONE	9250B	89	408,829.79	4,593.59

STATE:UTAH BUSINESS ACTIVITY:A - PHARMACIES

DRUG NAME	DRUG CODE	BUYERS	TOTAL GRAMS	AVG GRAMS
AMPHETAMINE	1100	549	322,909.40	588.18
DL-METHAMPHETAMINE RACEMIC BASE	1105B	25	104.08	4.16
LISDEXAMFETAMINE	1205	529	85,720.67	162.04
METHYLPHENIDATE (DL;D;L;ISOMERS)	1724	543	179,635.47	330.82
BARBITURIC ACID DERIVIATIVE OR SALT [PER 21C	2100	154	5,675	36.85
BUTALBITAL	2165	303	12,720	41.98
DRONABINOL IN AN ORAL SOLUTION IN FDA APPR	7365	1	0.45	0.45
COCAINE	9041L	4	4.71	1.18
CODEINE	9050	532	63,584.26	119.52
BUPRENORPHINE	9064	547	37,590.94	68.72
DIHYDROCODEINE	9120	1	1.07	1.07
OXYCODONE	9143	549	643,887.89	1,172.84
HYDROMORPHONE	9150	512	9,064.12	17.7
HYDROCODONE	9193	551	221,703.78	402.37
MEPERIDINE (PETHIDINE)	9230	234	5,381.29	23
METHADONE	9250B	503	38,100.55	75.75
MORPHINE	9300	534	181,248.57	339.42
OPIUM POWDERED	9639	25	36.36	1.45
OXYMORPHONE	9652	415	16,319.77	39.32
REMIFENTANIL	9739	5	0.72	0.14
SUFENTANIL BASE	9740	3	0.08	0.03

TAPENTADOL	9780	400	51,783.50	129.46
FENTANYL BASE	9801	530	2,994.95	5.65
OPIUM COMBINATION PRODUCT (C-III)	9809	72	49	0.68

STATE:UTAH BUSINESS ACTIVITY:B - HOSPITALS

DRUG NAME	DRUG CODE	BUYERS	TOTAL GRAMS	AVG GRAMS
AMPHETAMINE	1100	32	4,776.99	149.28
LISDEXAMFETAMINE	1205	18	885.07	49.17
METHYLPHENIDATE (DL;D;L;ISOMERS)	1724	43	4,496.46	104.57
BARBITURIC ACID DERIVIATIVE OR SALT [PER 21C	2100	1	10	10
AMOBARBITAL (SCHEDULE 2)	2125	3	34.18	11.39
PENTOBARBITAL (SCHEDULE 2)	2270	3	844.87	281.62
DRONABINOL IN AN ORAL SOLUTION IN FDA APPR	7365	1	0.15	0.15
COCAINE	9041L	51	386.01	7.57
CODEINE	9050	37	1,538.08	41.57
BUPRENORPHINE	9064	41	628.1	15.32
OXYCODONE	9143	88	26,736.46	303.82
HYDROMORPHONE	9150	81	1,789.16	22.09
HYDROCODONE	9193	108	11,806.68	109.32
MEPERIDINE (PETHIDINE)	9230	77	1,649.12	21.42
METHADONE	9250B	48	1,373.53	28.62
MORPHINE	9300	100	15,208.01	152.08
OPIUM POWDERED	9639	34	224.28	6.6
OXYMORPHONE	9652	8	78.86	9.86
ALFENTANIL	9737	8	3.09	0.39
REMIFENTANIL	9739	37	70.87	1.92
SUFENTANIL BASE	9740	28	0.83	0.03
TAPENTADOL	9780	22	3,107	141.23
FENTANYL BASE	9801	105	269.31	2.56
OPIUM COMBINATION PRODUCT (C-III)	9809	2	0.57	0.28

STATE:UTAH BUSINESS ACTIVITY:C - PRACTITIONERS

DRUG NAME	DRUG CODE	BUYERS	TOTAL GRAMS	AVG GRAMS

METHYLPHENIDATE (DL;D;L;ISOMERS)	1724	1	29.05	29.05
PENTOBARBITAL (SCHEDULE 2)	2270	46	50,733.77	1,102.91
COCAINE	9041L	1	0.29	0.29
CODEINE	9050	7	249.33	35.62
BUPRENORPHINE	9064	164	20.73	0.13
OXYCODONE	9143	12	362.88	30.24
HYDROMORPHONE	9150	89	52.06	0.58
HYDROCODONE	9193	78	367.08	4.71
MEPERIDINE (PETHIDINE)	9230	30	132.25	4.41
METHADONE	9250B	10	8.85	0.89
MORPHINE	9300	95	96.23	1.01
OXYMORPHONE	9652	1	0.81	0.81
REMIFENTANIL	9739	2	0.15	0.08
SUFENTANIL BASE	9740	1	0.01	0.01
FENTANYL BASE	9801	175	10.7	0.06

STATE:UTAH BUSINESS ACTIVITY:D - TEACHING INSTITUTIONS

DRUG NAME	DRUG CODE	BUYERS	TOTAL GRAMS	AVG GRAMS
BUPRENORPHINE	9064	1	0.01	0.01

STATE:UTAH BUSINESS ACTIVITY:M - MID-LEVEL PRACTITIONERS

DRUG NAME	DRUG CODE	BUYERS	TOTAL GRAMS	AVG GRAMS
PENTOBARBITAL (SCHEDULE 2)	2270	12	5,336.25	444.69
BUPRENORPHINE	9064	2	0.25	0.12
HYDROMORPHONE	9150	1	0.12	0.12
MEPERIDINE (PETHIDINE)	9230	3	32.9	10.97
REMIFENTANIL	9739	1	0.04	0.04
FENTANYL BASE	9801	9	0.58	0.06

STATE:UTAH BUSINESS ACTIVITY:N-U NARCOTIC TREATMENT PROGRAMS

DRUG NAME	DRUG CODE	BUYERS	TOTAL GRAMS	AVG GRAMS

BUPRENORPHINE	9064	12	322.42	26.87
METHADONE	9250B	13	66,203.49	5,092.58

STATE:VERMONT BUSINESS ACTIVITY:A - PHARMACIES

DRUG NAME	DRUG CODE	BUYERS	TOTAL GRAMS	AVG GRAMS
AMPHETAMINE	1100	149	38,761.65	260.15
DL-METHAMPHETAMINE RACEMIC BASE	1105B	2	7.23	3.62
LISDEXAMFETAMINE	1205	149	27,324.47	183.39
METHYLPHENIDATE (DL;D;L;ISOMERS)	1724	149	74,807.64	502.06
BARBITURIC ACID DERIVIATIVE OR SALT [PER 21C	2100	14	285	20.36
BUTALBITAL	2165	30	470	15.67
SECOBARBITAL (SCHEDULE 2)	2315	4	137.33	34.33
COCAINE	9041L	1	4.46	4.46
CODEINE	9050	143	12,700.85	88.82
BUPRENORPHINE	9064	147	20,052.57	136.41
DIHYDROCODEINE	9120	1	1.07	1.07
OXYCODONE	9143	148	84,817.39	573.09
HYDROMORPHONE	9150	149	4,183.76	28.08
HYDROCODONE	9193	149	18,183.61	122.04
LEVORPHANOL	9220L	1	0.12	0.12
MEPERIDINE (PETHIDINE)	9230	24	408.52	17.02
METHADONE	9250B	139	13,500.97	97.13
MORPHINE	9300	147	35,798.07	243.52
OPIUM POWDERED	9639	18	77.04	4.28
OXYMORPHONE	9652	54	1,140.55	21.12
TAPENTADOL	9780	56	4,600	82.14
FENTANYL BASE	9801	147	689.75	4.69
OPIUM COMBINATION PRODUCT (C-III)	9809	1	0.57	0.57

STATE:VERMONT BUSINESS ACTIVITY:B - HOSPITALS

DRUG NAME	DRUG CODE	BUYERS	TOTAL GRAMS	AVG GRAMS
AMPHETAMINE	1100	11	927.75	84.34
LISDEXAMFETAMINE	1205	7	379.81	54.26

METHYLPHENIDATE (DL;D;L;ISOMERS)	1724	15	2,119.69	141.31
AMOBARBITAL (SCHEDULE 2)	2125	1	0.91	0.91
PENTOBARBITAL (SCHEDULE 2)	2270	1	84.76	84.76
COCAINE	9041L	8	34	4.25
CODEINE	9050	13	367.69	28.28
BUPRENORPHINE	9064	17	1,148.38	67.55
OXYCODONE	9143	17	5,924.88	348.52
HYDROMORPHONE	9150	16	672.98	42.06
HYDROCODONE	9193	17	921.13	54.18
LEVORPHANOL	9220L	1	1.51	1.51
MEPERIDINE (PETHIDINE)	9230	15	878.25	58.55
METHADONE	9250B	15	704.97	47
MORPHINE	9300	16	4,689.51	293.09
OPIUM POWDERED	9639	13	85.68	6.59
OXYMORPHONE	9652	1	4.28	4.28
ALFENTANIL	9737	2	0.04	0.02
REMIFENTANIL	9739	3	2.79	0.93
SUFENTANIL BASE	9740	2	0.03	0.01
TAPENTADOL	9780	1	20	20
FENTANYL BASE	9801	17	55.49	3.26
OPIUM COMBINATION PRODUCT (C-III)	9809	1	0.38	0.38

STATE:VERMONT BUSINESS ACTIVITY:C - PRACTITIONERS

DRUG NAME	DRUG CODE	BUYERS	TOTAL GRAMS	AVG GRAMS
PENTOBARBITAL (SCHEDULE 2)	2270	74	53,566.58	723.87
CODEINE	9050	2	12.16	6.08
BUPRENORPHINE	9064	83	3.47	0.04
OXYCODONE	9143	1	1.79	1.79
HYDROMORPHONE	9150	51	16.07	0.32
HYDROCODONE	9193	20	32.88	1.64
MEPERIDINE (PETHIDINE)	9230	1	1.09	1.09
MORPHINE	9300	28	40.98	1.46
OXYMORPHONE	9652	1	0.03	0.03
NOROXYMORPHONE	9668	1	0.1	0.1
REMIFENTANIL	9739	1	0.04	0.04

FENTANYL BASE	9801	37	2.82	0.08

STATE:VERMONT BUSINESS ACTIVITY:D - TEACHING INSTITUTIONS

DRUG NAME	DRUG CODE	BUYERS	TOTAL GRAMS	AVG GRAMS
BUPRENORPHINE	9064	2	0	0

STATE:VERMONT BUSINESS ACTIVITY:M - MID-LEVEL PRACTITIONERS

DRUG NAME	DRUG CODE	BUYERS	TOTAL GRAMS	AVG GRAMS
PENTOBARBITAL (SCHEDULE 2)	2270	3	710.89	236.96
CODEINE	9050	5	35.23	7.05

STATE:VERMONT BUSINESS ACTIVITY:N-U NARCOTIC TREATMENT PROGRAMS

DRUG NAME	DRUG CODE	BUYERS	TOTAL GRAMS	AVG GRAMS
BUPRENORPHINE	9064	10	4,014.64	401.46
METHADONE	9250B	10	59,793.32	5,979.33

STATE:VIRGIN ISLANDS BUSINESS ACTIVITY:A - PHARMACIES

DRUG NAME	DRUG CODE	BUYERS	TOTAL GRAMS	AVG GRAMS
AMPHETAMINE	1100	19	1,217.23	64.06
LISDEXAMFETAMINE	1205	14	267.66	19.12
METHYLPHENIDATE (DL;D;L;ISOMERS)	1724	19	595.89	31.36
BARBITURIC ACID DERIVIATIVE OR SALT [PER 21C	2100	7	105	15
BUTALBITAL	2165	3	45	15
CODEINE	9050	21	2,939.36	139.97
BUPRENORPHINE	9064	11	62.59	5.69
OXYCODONE	9143	21	2,066.24	98.39
HYDROMORPHONE	9150	13	57.1	4.39
HYDROCODONE	9193	21	1,126.02	53.62
MEPERIDINE (PETHIDINE)	9230	7	220.05	31.44

METHADONE	9250B	9	99.73	11.08
MORPHINE	9300	13	491.23	37.79
TAPENTADOL	9780	2	18	9
FENTANYL BASE	9801	12	12.01	1

STATE:VIRGIN ISLANDS BUSINESS ACTIVITY:B - HOSPITALS

DRUG NAME	DRUG CODE	BUYERS	TOTAL GRAMS	AVG GRAMS
METHYLPHENIDATE (DL;D;L;ISOMERS)	1724	2	1.3	0.65
CODEINE	9050	2	55.25	27.63
OXYCODONE	9143	2	35.86	17.93
HYDROMORPHONE	9150	2	16.95	8.48
HYDROCODONE	9193	2	28.34	14.17
MEPERIDINE (PETHIDINE)	9230	1	21.24	21.24
MORPHINE	9300	3	32.38	10.79
OPIUM POWDERED	9639	1	1.8	1.8
FENTANYL BASE	9801	3	1.87	0.62

STATE:VIRGIN ISLANDS BUSINESS ACTIVITY:C - PRACTITIONERS

DRUG NAME	DRUG CODE	BUYERS	TOTAL GRAMS	AVG GRAMS
CODEINE	9050	2	4.42	2.21
BUPRENORPHINE	9064	3	0.13	0.04
HYDROMORPHONE	9150	2	0.1	0.05
HYDROCODONE	9193	2	4.24	2.12
MEPERIDINE (PETHIDINE)	9230	2	12.64	6.32
MORPHINE	9300	1	1.28	1.28
FENTANYL BASE	9801	3	0.07	0.02

STATE:VIRGIN ISLANDS BUSINESS ACTIVITY:N-U NARCOTIC TREATMENT PROGRAMS

DRUG NAME	DRUG CODE	BUYERS	TOTAL GRAMS	AVG GRAMS
METHADONE	9250B	1	357.76	357.76

STATISTICAL SUMMARY FOR RETAIL DRUG PURCHASES BY GRAMS WT

STATE:VIRGINIA BUSINESS ACTIVITY:A - PHARMACIES

DRUG NAME	DRUG CODE	BUYERS	TOTAL GRAMS	AVG GRAMS
AMPHETAMINE	1100	1,682	685,423.29	407.5
DL-METHAMPHETAMINE RACEMIC BASE	1105B	59	175.21	2.97
LISDEXAMFETAMINE	1205	1,618	263,719.39	162.99
METHYLPHENIDATE (DL;D;L;ISOMERS)	1724	1,668	501,926.04	300.91
BARBITURIC ACID DERIVIATIVE OR SALT [PER 21C	2100	540	13,850	25.65
BUTALBITAL	2165	725	20,780	28.66
PENTOBARBITAL (SCHEDULE 2)	2270	1	0.91	0.91
SECOBARBITAL (SCHEDULE 2)	2315	3	109.86	36.62
DRONABINOL IN AN ORAL SOLUTION IN FDA APPR	7365	2	0.6	0.3
NABILONE	7379	4	0.75	0.19
COCAINE	9041L	4	47.18	11.79
CODEINE	9050	1,645	205,635.14	125.01
BUPRENORPHINE	9064	1,582	75,607.16	47.79
DIHYDROCODEINE	9120	13	26.7	2.05
OXYCODONE	9143	1,704	1,116,236.21	655.07
HYDROMORPHONE	9150	1,595	38,087.17	23.88
HYDROCODONE	9193	1,707	394,094.50	230.87
LEVORPHANOL	9220L	51	39	0.76
MEPERIDINE (PETHIDINE)	9230	744	18,804.90	25.28
METHADONE	9250B	1,312	62,956.64	47.99
MORPHINE	9300	1,620	280,864.81	173.37
OPIUM TINCTURE (LAUDANUM)	9630	2	10.62	5.31
OPIUM POWDERED	9639	109	263.16	2.41
OXYMORPHONE	9652	1,095	36,210.03	33.07
REMIFENTANIL	9739	2	0.04	0.02
TAPENTADOL	9780	1,204	163,167.50	135.52
FENTANYL BASE	9801	1,556	6,046.85	3.89
OPIUM COMBINATION PRODUCT (C-III)	9809	58	21.19	0.37

STATE:VIRGINIA BUSINESS ACTIVITY:B - HOSPITALS

DRUG NAME	DRUG CODE	BUYERS	TOTAL GRAMS	AVG GRAMS

AMPHETAMINE	1100	87	28,529.87	327.93
LISDEXAMFETAMINE	1205	43	7,256.31	168.75
METHYLPHENIDATE (DL;D;L;ISOMERS)	1724	121	33,798.20	279.32
GAMMA HYDROXYBUTYRIC ACID(FDA APPROVED	2012	1	6,092.93	6,092.93
BARBITURIC ACID DERIVIATIVE OR SALT [PER 21C	2100	4	45	11.25
AMOBARBITAL (SCHEDULE 2)	2125	4	11.39	2.85
BUTALBITAL	2165	5	90	18
PENTOBARBITAL (SCHEDULE 2)	2270	25	10,081	403.24
DRONABINOL IN AN ORAL SOLUTION IN FDA APPR	7365	1	0.15	0.15
COCAINE	9041L	102	481	4.72
CODEINE	9050	131	7,329.26	55.95
BUPRENORPHINE	9064	103	1,653.81	16.06
OXYCODONE	9143	179	69,163.69	386.39
HYDROMORPHONE	9150	180	6,649.60	36.94
HYDROCODONE	9193	177	20,517.96	115.92
MEPERIDINE (PETHIDINE)	9230	138	3,752.22	27.19
METHADONE	9250B	115	3,940.98	34.27
MORPHINE	9300	188	32,605.56	173.43
OPIUM POWDERED	9639	80	452.52	5.66
OXYMORPHONE	9652	18	64.32	3.57
ALFENTANIL	9737	16	3.56	0.22
REMIFENTANIL	9739	51	26.39	0.52
SUFENTANIL BASE	9740	41	1.54	0.04
TAPENTADOL	9780	35	2,133	60.94
FENTANYL BASE	9801	192	818.15	4.26
OPIUM COMBINATION PRODUCT (C-III)	9809	7	2.65	0.38

STATE:VIRGINIA BUSINESS ACTIVITY:C - PRACTITIONERS

DRUG NAME	DRUG CODE	BUYERS	TOTAL GRAMS	AVG GRAMS
AMPHETAMINE	1100	3	129.94	43.31
LISDEXAMFETAMINE	1205	1	6.13	6.13
METHYLPHENIDATE (DL;D;L;ISOMERS)	1724	2	59.71	29.85
BARBITURIC ACID DERIVIATIVE OR SALT [PER 21C	2100	1	5	5
PENTOBARBITAL (SCHEDULE 2)	2270	189	141,986.67	751.25
COCAINE	9041L	10	84.14	8.41

CODEINE	9050	281	6,068.71	21.6
BUPRENORPHINE	9064	803	56.76	0.07
OXYCODONE	9143	38	758.12	19.95
HYDROMORPHONE	9150	382	198.18	0.52
HYDROCODONE	9193	237	1,720.71	7.26
MEPERIDINE (PETHIDINE)	9230	127	720.29	5.67
METHADONE	9250B	15	155.98	10.4
MORPHINE	9300	211	381.66	1.81
OPIUM POWDERED	9639	2	7.2	3.6
OXYMORPHONE	9652	15	37.85	2.52
ALFENTANIL	9737	1	0.06	0.06
REMIFENTANIL	9739	8	0.7	0.09
TAPENTADOL	9780	4	267	66.75
FENTANYL BASE	9801	473	47.52	0.1

STATE:VIRGINIA BUSINESS ACTIVITY:D - TEACHING INSTITUTIONS

DRUG NAME	DRUG CODE	BUYERS	TOTAL GRAMS	AVG GRAMS
PENTOBARBITAL (SCHEDULE 2)	2270	1	7,997.54	7,997.54
COCAINE	9041L	2	2.21	1.11
CODEINE	9050	1	409.01	409.01
BUPRENORPHINE	9064	2	0.49	0.24
HYDROMORPHONE	9150	1	9.22	9.22
HYDROCODONE	9193	1	6.28	6.28
MEPERIDINE (PETHIDINE)	9230	1	0.54	0.54
METHADONE	9250B	2	9.48	4.74
MORPHINE	9300	1	13.35	13.35
OXYMORPHONE	9652	1	0.18	0.18
REMIFENTANIL	9739	1	0.04	0.04
FENTANYL BASE	9801	1	0.79	0.79

STATE:VIRGINIA BUSINESS ACTIVITY:M - MID-LEVEL PRACTITIONERS

DRUG NAME	DRUG CODE	BUYERS	TOTAL GRAMS	AVG GRAMS
PENTOBARBITAL (SCHEDULE 2)	2270	40	30,923.80	773.1

CODEINE	9050	66	134.23	2.03
HYDROMORPHONE	9150	1	0.09	0.09
MEPERIDINE (PETHIDINE)	9230	1	1.09	1.09
MORPHINE	9300	14	3.43	0.24
FENTANYL BASE	9801	14	0.2	0.01

STATE:VIRGINIA BUSINESS ACTIVITY:N-U NARCOTIC TREATMENT PROGRAMS

DRUG NAME	DRUG CODE	BUYERS	TOTAL GRAMS	AVG GRAMS
BUPRENORPHINE	9064	26	2,140.43	82.32
METHADONE	9250B	39	250,009.84	6,410.51

STATE:WASHINGTON BUSINESS ACTIVITY:A - PHARMACIES

DRUG NAME	DRUG CODE	BUYERS	TOTAL GRAMS	AVG GRAMS
AMPHETAMINE	1100	1,203	420,314.25	349.39
DL-METHAMPHETAMINE RACEMIC BASE	1105B	110	381.36	3.47
D-METHAMPHETAMINE	1105D	2	2.41	1.21
LISDEXAMFETAMINE	1205	1,124	132,656.03	118.02
METHYLPHENIDATE (DL;D;L;ISOMERS)	1724	1,205	422,540.95	350.66
BARBITURIC ACID DERIVIATIVE OR SALT [PER 21C	2100	314	9,215	29.35
BUTALBITAL	2165	553	17,135	30.99
SECOBARBITAL (SCHEDULE 2)	2315	21	595.08	28.34
NABILONE	7379	1	0.05	0.05
COCAINE	9041L	7	34.6	4.94
CODEINE	9050	1,219	229,118.50	187.96
BUPRENORPHINE	9064	1,183	72,336.58	61.15
OXYCODONE	9143	1,222	1,073,381.79	878.38
HYDROMORPHONE	9150	1,180	35,099.43	29.75
HYDROCODONE	9193	1,230	487,973.82	396.73
LEVORPHANOL	9220L	104	108.53	1.04
MEPERIDINE (PETHIDINE)	9230	464	10,742.22	23.15
METHADONE	9250B	1,151	102,582.58	89.12
MORPHINE	9300	1,212	386,631.86	319
OPIUM TINCTURE (LAUDANUM)	9630	2	3.54	1.77

OPIUM POWDERED	9639	99	405	4.09
OXYMORPHONE	9652	673	11,285.99	16.77
REMIFENTANIL	9739	1	0.02	0.02
TAPENTADOL	9780	743	68,635.50	92.38
FENTANYL BASE	9801	1,144	5,195.40	4.54
OPIUM COMBINATION PRODUCT (C-III)	9809	19	9.65	0.51

STATE:WASHINGTON BUSINESS ACTIVITY:B - HOSPITALS

DRUG NAME	DRUG CODE	BUYERS	TOTAL GRAMS	AVG GRAMS
AMPHETAMINE	1100	94	15,085.89	160.49
LISDEXAMFETAMINE	1205	36	3,646.08	101.28
METHYLPHENIDATE (DL;D;L;ISOMERS)	1724	113	24,292.75	214.98
GAMMA HYDROXYBUTYRIC ACID(FDA APPROVED	2012	1	2,229.12	2,229.12
BARBITURIC ACID DERIVIATIVE OR SALT [PER 21C	2100	4	25	6.25
AMOBARBITAL (SCHEDULE 2)	2125	8	36.91	4.61
BUTALBITAL	2165	12	270	22.5
PENTOBARBITAL (SCHEDULE 2)	2270	12	1,935.81	161.32
SECOBARBITAL (SCHEDULE 2)	2315	3	128.17	42.72
COCAINE	9041L	62	416.62	6.72
CODEINE	9050	155	9,671.09	62.39
BUPRENORPHINE	9064	105	2,873.24	27.36
OXYCODONE	9143	228	88,740.88	389.21
HYDROMORPHONE	9150	202	7,445.37	36.86
HYDROCODONE	9193	218	34,311.59	157.39
LEVORPHANOL	9220L	3	0.7	0.23
MEPERIDINE (PETHIDINE)	9230	172	2,141.04	12.45
METHADONE	9250B	131	10,148.13	77.47
MORPHINE	9300	224	46,306.44	206.73
OPIUM POWDERED	9639	83	851.04	10.25
OXYMORPHONE	9652	18	117.33	6.52
ALFENTANIL	9737	19	11.27	0.59
REMIFENTANIL	9739	60	48.39	0.81
SUFENTANIL BASE	9740	35	1.02	0.03
TAPENTADOL	9780	18	1,715	95.28
FENTANYL BASE	9801	235	785.7	3.34

OPIUM COMBINATION PRODUCT (C-III)	9809	2	0.75	0.38

STATE:WASHINGTON BUSINESS ACTIVITY:C - PRACTITIONERS

DRUG NAME	DRUG CODE	BUYERS	TOTAL GRAMS	AVG GRAMS
AMPHETAMINE	1100	1	0.95	0.95
LISDEXAMFETAMINE	1205	1	10.2	10.2
METHYLPHENIDATE (DL;D;L;ISOMERS)	1724	1	22.83	22.83
PENTOBARBITAL (SCHEDULE 2)	2270	218	217,366.89	997.1
COCAINE	9041L	2	2.86	1.43
CODEINE	9050	86	6,927.25	80.55
BUPRENORPHINE	9064	770	160.01	0.21
OXYCODONE	9143	38	85.85	2.26
HYDROMORPHONE	9150	536	280.42	0.52
HYDROCODONE	9193	390	1,182.41	3.03
MEPERIDINE (PETHIDINE)	9230	131	652.75	4.98
METHADONE	9250B	40	175.12	4.38
MORPHINE	9300	312	735.61	2.36
OPIUM POWDERED	9639	2	1.44	0.72
OXYMORPHONE	9652	23	2.06	0.09
NOROXYMORPHONE	9668	2	8.47	4.24
ALFENTANIL	9737	8	1.38	0.17
REMIFENTANIL	9739	43	1.6	0.04
SUFENTANIL BASE	9740	6	0.02	0
FENTANYL BASE	9801	642	50.97	0.08

STATE:WASHINGTON BUSINESS ACTIVITY:D - TEACHING INSTITUTIONS

DRUG NAME	DRUG CODE	BUYERS	TOTAL GRAMS	AVG GRAMS
BUPRENORPHINE	9064	1	0.01	0.01

STATE:WASHINGTON BUSINESS ACTIVITY:M - MID-LEVEL PRACTITIONERS

DRUG NAME	DRUG CODE	BUYERS	TOTAL GRAMS	AVG GRAMS

PENTOBARBITAL (SCHEDULE 2)	2270	22	21,514.93	977.95
CODEINE	9050	4	32.63	8.16
BUPRENORPHINE	9064	2	1.08	0.54
OXYCODONE	9143	2	6.28	3.14
HYDROMORPHONE	9150	5	0.34	0.07
HYDROCODONE	9193	1	0.61	0.61
MEPERIDINE (PETHIDINE)	9230	4	7.41	1.85
METHADONE	9250B	2	3.58	1.79
MORPHINE	9300	4	4.75	1.19
FENTANYL BASE	9801	14	0.33	0.02

STATE:WASHINGTON BUSINESS ACTIVITY:N-U NARCOTIC TREATMENT PROGRAMS

DRUG NAME	DRUG CODE	BUYERS	TOTAL GRAMS	AVG GRAMS
PENTOBARBITAL (SCHEDULE 2)	2270	1	2.28	2.28
BUPRENORPHINE	9064	13	1,628.56	125.27
HYDROMORPHONE	9150	1	1.24	1.24
METHADONE	9250B	24	277,931.22	11,580.47

STATE:WEST VIRGINIA BUSINESS ACTIVITY:A - PHARMACIES

DRUG NAME	DRUG CODE	BUYERS	TOTAL GRAMS	AVG GRAMS
AMPHETAMINE	1100	557	92,437.51	165.96
DL-METHAMPHETAMINE RACEMIC BASE	1105B	5	16.48	3.3
D-METHAMPHETAMINE	1105D	1	3.62	3.62
LISDEXAMFETAMINE	1205	547	77,857.93	142.34
METHYLPHENIDATE (DL;D;L;ISOMERS)	1724	562	123,648.70	220.02
BARBITURIC ACID DERIVIATIVE OR SALT [PER 21C	2100	82	2,105	25.67
BUTALBITAL	2165	166	5,055	30.45
COCAINE	9041L	2	3.04	1.52
CODEINE	9050	558	121,528.28	217.79
BUPRENORPHINE	9064	492	58,295.89	118.49
DIHYDROCODEINE	9120	1	7.48	7.48
OXYCODONE	9143	567	314,687.41	555
HYDROMORPHONE	9150	403	4,667	11.58

HYDROCODONE	9193	565	217,071.24	384.2
MEPERIDINE (PETHIDINE)	9230	72	1,399.96	19.44
METHADONE	9250B	363	15,601.11	42.98
MORPHINE	9300	546	75,327.60	137.96
OPIUM TINCTURE (LAUDANUM)	9630	2	5.9	2.95
OPIUM POWDERED	9639	34	98.28	2.89
OXYMORPHONE	9652	281	9,891.02	35.2
TAPENTADOL	9780	260	24,252	93.28
FENTANYL BASE	9801	513	1,888.46	3.68
OPIUM COMBINATION PRODUCT (C-III)	9809	18	9.08	0.5

STATE:WEST VIRGINIA BUSINESS ACTIVITY:B - HOSPITALS

DRUG NAME	DRUG CODE	BUYERS	TOTAL GRAMS	AVG GRAMS
AMPHETAMINE	1100	36	1,535.24	42.65
LISDEXAMFETAMINE	1205	20	729.56	36.48
METHYLPHENIDATE (DL;D;L;ISOMERS)	1724	51	2,579.06	50.57
BARBITURIC ACID DERIVIATIVE OR SALT [PER 21C	2100	2	15	7.5
AMOBARBITAL (SCHEDULE 2)	2125	1	1.82	1.82
BUTALBITAL	2165	1	15	15
PENTOBARBITAL (SCHEDULE 2)	2270	5	166.33	33.27
COCAINE	9041L	39	198.77	5.1
CODEINE	9050	68	2,738.68	40.27
BUPRENORPHINE	9064	51	2,642.30	51.81
OXYCODONE	9143	78	18,219.96	233.59
HYDROMORPHONE	9150	70	1,367.71	19.54
HYDROCODONE	9193	78	18,331.23	235.02
MEPERIDINE (PETHIDINE)	9230	56	841.87	15.03
METHADONE	9250B	47	802.1	17.07
MORPHINE	9300	75	12,067.19	160.9
OPIUM POWDERED	9639	33	282.6	8.56
OXYMORPHONE	9652	10	32.61	3.26
REMIFENTANIL	9739	24	20.53	0.86
SUFENTANIL BASE	9740	10	0.26	0.03
TAPENTADOL	9780	11	292.5	26.59
FENTANYL BASE	9801	74	271.53	3.67

OPIUM COMBINATION PRODUCT (C-III)	9809	2	0.57	0.28

STATE:WEST VIRGINIA BUSINESS ACTIVITY:C - PRACTITIONERS

DRUG NAME	DRUG CODE	BUYERS	TOTAL GRAMS	AVG GRAMS
METHYLPHENIDATE (DL;D;L;ISOMERS)	1724	1	149.03	149.03
PENTOBARBITAL (SCHEDULE 2)	2270	44	34,470.84	783.43
CODEINE	9050	46	175.04	3.81
BUPRENORPHINE	9064	107	7.74	0.07
DIHYDROCODEINE	9120	1	0.21	0.21
OXYCODONE	9143	1	0.9	0.9
HYDROMORPHONE	9150	32	19.46	0.61
HYDROCODONE	9193	50	199.14	3.98
MEPERIDINE (PETHIDINE)	9230	10	248.6	24.86
MORPHINE	9300	28	39.94	1.43
REMIFENTANIL	9739	1	0.22	0.22
FENTANYL BASE	9801	52	6.95	0.13

STATE:WEST VIRGINIA BUSINESS ACTIVITY:M - MID-LEVEL PRACTITIONERS

DRUG NAME	DRUG CODE	BUYERS	TOTAL GRAMS	AVG GRAMS
PENTOBARBITAL (SCHEDULE 2)	2270	12	7,464.37	622.03
CODEINE	9050	8	19.06	2.38
MORPHINE	9300	41	11.8	0.29
FENTANYL BASE	9801	44	0.55	0.01

STATE:WEST VIRGINIA BUSINESS ACTIVITY:N-U NARCOTIC TREATMENT PROGRAMS

DRUG NAME	DRUG CODE	BUYERS	TOTAL GRAMS	AVG GRAMS
BUPRENORPHINE	9064	7	1,244.64	177.81
METHADONE	9250B	10	76,167.10	7,616.71

STATE:WISCONSIN BUSINESS ACTIVITY:A - PHARMACIES

DRUG NAME	DRUG CODE	BUYERS	TOTAL GRAMS	AVG GRAMS
AMPHETAMINE	1100	1,114	499,223.92	448.14
DL-METHAMPHETAMINE RACEMIC BASE	1105B	50	191.68	3.83
D-METHAMPHETAMINE	1105D	6	24.1	4.02
LISDEXAMFETAMINE	1205	1,095	265,229.39	242.22
METHYLPHENIDATE (DL;D;L;ISOMERS)	1724	1,114	404,367.21	362.99
BARBITURIC ACID DERIVIATIVE OR SALT [PER 21C	2100	266	6,325	23.78
BUTALBITAL	2165	304	6,890	22.66
SECOBARBITAL (SCHEDULE 2)	2315	1	9.16	9.16
DRONABINOL IN AN ORAL SOLUTION IN FDA APPR	7365	1	0.15	0.15
COCAINE	9041L	2	0.86	0.43
CODEINE	9050	1,101	184,148	167.26
BUPRENORPHINE	9064	1,047	36,539.03	34.9
DIHYDROCODEINE	9120	3	18.16	6.05
OXYCODONE	9143	1,123	787,727.19	701.45
HYDROMORPHONE	9150	940	10,604.79	11.28
HYDROCODONE	9193	1,130	294,374.34	260.51
LEVORPHANOL	9220L	12	15.55	1.3
MEPERIDINE (PETHIDINE)	9230	122	2,146.07	17.59
METHADONE	9250B	913	36,011.41	39.44
MORPHINE	9300	1,111	276,399.77	248.78
OPIUM TINCTURE (LAUDANUM)	9630	3	10.64	3.55
OPIUM POWDERED	9639	114	219.24	1.92
OXYMORPHONE	9652	552	8,341.81	15.11
REMIFENTANIL	9739	2	0.09	0.05
SUFENTANIL BASE	9740	1	2	2
TAPENTADOL	9780	668	57,648.50	86.3
FENTANYL BASE	9801	1,076	5,173.41	4.81
OPIUM COMBINATION PRODUCT (C-III)	9809	20	22.14	1.11

STATE:WISCONSIN BUSINESS ACTIVITY:B - HOSPITALS

DRUG NAME	DRUG CODE	BUYERS	TOTAL GRAMS	AVG GRAMS
AMPHETAMINE	1100	108	11,607.85	107.48
DL-METHAMPHETAMINE RACEMIC BASE	1105B	1	5.63	5.63

D-METHAMPHETAMINE	1105D	1	4.02	4.02
LISDEXAMFETAMINE	1205	66	2,322.23	35.19
METHYLPHENIDATE (DL;D;L;ISOMERS)	1724	127	9,297.52	73.21
BARBITURIC ACID DERIVIATIVE OR SALT [PER 21C	2100	2	35	17.5
AMOBARBITAL (SCHEDULE 2)	2125	8	26.89	3.36
BUTALBITAL	2165	6	105	17.5
PENTOBARBITAL (SCHEDULE 2)	2270	13	11,369.26	874.56
COCAINE	9041L	91	476.54	5.24
CODEINE	9050	172	5,839.55	33.95
BUPRENORPHINE	9064	103	2,598.74	25.23
OXYCODONE	9143	170	69,203.69	407.08
HYDROMORPHONE	9150	161	4,006.11	24.88
HYDROCODONE	9193	171	16,505.60	96.52
MEPERIDINE (PETHIDINE)	9230	119	3,223.24	27.09
METHADONE	9250B	121	3,115.33	25.75
MORPHINE	9300	172	31,505.05	183.17
OPIUM TINCTURE (LAUDANUM)	9630	1	3.54	3.54
OPIUM POWDERED	9639	110	598.32	5.44
OXYMORPHONE	9652	24	159.53	6.65
ALFENTANIL	9737	34	15.21	0.45
REMIFENTANIL	9739	59	25.76	0.44
SUFENTANIL BASE	9740	41	0.51	0.01
TAPENTADOL	9780	31	1,826.50	58.92
FENTANYL BASE	9801	166	675.94	4.07
OPIUM COMBINATION PRODUCT (C-III)	9809	2	0.95	0.47

STATE:WISCONSIN BUSINESS ACTIVITY:C - PRACTITIONERS

DRUG NAME	DRUG CODE	BUYERS	TOTAL GRAMS	AVG GRAMS
AMPHETAMINE	1100	1	1.25	1.25
PENTOBARBITAL (SCHEDULE 2)	2270	245	185,498.63	757.14
COCAINE	9041L	17	127.42	7.5
CODEINE	9050	56	564.39	10.08
BUPRENORPHINE	9064	497	83.05	0.17
DIHYDROCODEINE	9120	1	3.2	3.2
OXYCODONE	9143	61	789.05	12.94

HYDROMORPHONE	9150	408	251.27	0.62
HYDROCODONE	9193	236	652.94	2.77
MEPERIDINE (PETHIDINE)	9230	81	483.68	5.97
METHADONE	9250B	13	38.73	2.98
MORPHINE	9300	231	511.93	2.22
OPIUM POWDERED	9639	7	37.08	5.3
OXYMORPHONE	9652	10	10.03	1
ALFENTANIL	9737	14	4.06	0.29
REMIFENTANIL	9739	15	0.88	0.06
SUFENTANIL BASE	9740	3	0.01	0
FENTANYL BASE	9801	337	48.78	0.14

STATE:WISCONSIN BUSINESS ACTIVITY:M - MID-LEVEL PRACTITIONERS

DRUG NAME	DRUG CODE	BUYERS	TOTAL GRAMS	AVG GRAMS
PENTOBARBITAL (SCHEDULE 2)	2270	21	12,552.62	597.74
CODEINE	9050	1	2.23	2.23
MORPHINE	9300	2	5.83	2.91
FENTANYL BASE	9801	1	0.01	0.01

STATE:WISCONSIN BUSINESS ACTIVITY:N-U NARCOTIC TREATMENT PROGRAMS

DRUG NAME	DRUG CODE	BUYERS	TOTAL GRAMS	AVG GRAMS
BUPRENORPHINE	9064	8	1,883.52	235.44
METHADONE	9250B	19	222,981.97	11,735.89

STATE:WYOMING BUSINESS ACTIVITY:A - PHARMACIES

DRUG NAME	DRUG CODE	BUYERS	TOTAL GRAMS	AVG GRAMS
AMPHETAMINE	1100	119	24,045.57	202.06
DL-METHAMPHETAMINE RACEMIC BASE	1105B	5	21.3	4.26
LISDEXAMFETAMINE	1205	118	10,068.77	85.33
METHYLPHENIDATE (DL;D;L;ISOMERS)	1724	119	30,396.30	255.43
BARBITURIC ACID DERIVIATIVE OR SALT [PER 21C	2100	30	1,525	50.83

BUTALBITAL	2165	46	1,605	34.89
COCAINE	9041L	2	21.14	10.57
CODEINE	9050	119	14,898.72	125.2
ETORPHINE	9056	1	57	57
BUPRENORPHINE	9064	120	4,718.06	39.32
OXYCODONE	9143	121	95,190.94	786.7
HYDROMORPHONE	9150	120	2,194.78	18.29
HYDROCODONE	9193	121	40,822.05	337.37
MEPERIDINE (PETHIDINE)	9230	50	1,014.86	20.3
METHADONE	9250B	103	4,216.38	40.94
MORPHINE	9300	121	28,432.25	234.98
OPIUM POWDERED	9639	12	9.72	0.81
OXYMORPHONE	9652	64	1,120.03	17.5
REMIFENTANIL	9739	1	0.11	0.11
TAPENTADOL	9780	63	7,696	122.16
FENTANYL BASE	9801	119	534.41	4.49
OPIUM COMBINATION PRODUCT (C-III)	9809	4	3.03	0.76

STATE:WYOMING BUSINESS ACTIVITY:B - HOSPITALS

DRUG NAME	DRUG CODE	BUYERS	TOTAL GRAMS	AVG GRAMS
AMPHETAMINE	1100	15	1,690.38	112.69
LISDEXAMFETAMINE	1205	10	381.55	38.15
METHYLPHENIDATE (DL;D;L;ISOMERS)	1724	24	2,179.68	90.82
BUTALBITAL	2165	2	25	12.5
PENTOBARBITAL (SCHEDULE 2)	2270	2	32.81	16.41
COCAINE	9041L	22	48.14	2.19
CODEINE	9050	33	1,638.60	49.65
BUPRENORPHINE	9064	17	312.79	18.4
OXYCODONE	9143	43	6,967.60	162.04
HYDROMORPHONE	9150	42	226.07	5.38
HYDROCODONE	9193	43	5,000.69	116.3
MEPERIDINE (PETHIDINE)	9230	38	276.77	7.28
METHADONE	9250B	18	540.13	30.01
MORPHINE	9300	43	7,388.49	171.83
OPIUM POWDERED	9639	13	88.56	6.81

ALFENTANIL	9737	5	0.12	0.02
REMIFENTANIL	9739	15	1.67	0.11
SUFENTANIL BASE	9740	8	0.07	0.01
TAPENTADOL	9780	5	70	14
FENTANYL BASE	9801	45	84.7	1.88

STATE:WYOMING BUSINESS ACTIVITY:C - PRACTITIONERS

DRUG NAME	DRUG CODE	BUYERS	TOTAL GRAMS	AVG GRAMS
PENTOBARBITAL (SCHEDULE 2)	2270	38	40,259.73	1,059.47
CODEINE	9050	13	218.35	16.8
BUPRENORPHINE	9064	58	2.04	0.04
OXYCODONE	9143	1	9.75	9.75
HYDROMORPHONE	9150	40	18.51	0.46
HYDROCODONE	9193	26	186.32	7.17
MEPERIDINE (PETHIDINE)	9230	4	10.02	2.51
MORPHINE	9300	35	46.7	1.33
FENTANYL BASE	9801	39	1.87	0.05

STATE:WYOMING BUSINESS ACTIVITY:D - TEACHING INSTITUTIONS

DRUG NAME	DRUG CODE	BUYERS	TOTAL GRAMS	AVG GRAMS
BUPRENORPHINE	9064	1	0	0

STATE:WYOMING BUSINESS ACTIVITY:M - MID-LEVEL PRACTITIONERS

DRUG NAME	DRUG CODE	BUYERS	TOTAL GRAMS	AVG GRAMS
PENTOBARBITAL (SCHEDULE 2)	2270	2	177.72	88.86
CODEINE	9050	1	10.8	10.8
OXYCODONE	9143	1	0.54	0.54
HYDROCODONE	9193	1	0.36	0.36

UNITED STATES SUMMARY FOR RETAIL DRUG PURCHASES BY GRAMS WT

ENTIRE UNITED STATES BUSINESS ACTIVITY: A - PHARMACIES

DRUG NAME	DRUG CODE	BUYERS	TOTAL GRAMS	AVG GRAMS
AMPHETAMINE	1100	64,254	20,407,502.81	317.61
DL-METHAMPHETAMINE RACEMIC BASE	1105B	1,944	6,893.33	3.55
D-METHAMPHETAMINE	1105D	121	469.99	3.88
LISDEXAMFETAMINE	1205	59,765	9,592,434.05	160.5
METHYLPHENIDATE (DL;D;L;ISOMERS)	1724	63,671	17,701,935.44	278.02
GAMMA HYDROXYBUTYRIC ACID(FDA APPROVED-CSA III)	2012	1	30,411,958.46	30,411,958.46
BARBITURIC ACID DERIVIATIVE OR SALT [PER 21CFR 1308.13(C)(3)]	2100	18,623	596,769	32.04
AMOBARBITAL (SCHEDULE 2)	2125	2	0.91	0.46
BUTALBITAL	2165	24,330	847,360	34.83
PENTOBARBITAL (SCHEDULE 2)	2270	9	880.93	97.88
SECOBARBITAL (SCHEDULE 2)	2315	267	10,024.73	37.55
DRONABINOL IN AN ORAL SOLUTION IN FDA APPROVED DRUG PRODU	7365	191	341.25	1.79
NABILONE	7379	65	13.75	0.21
COCAINE	9041L	206	1,408.73	6.84
CODEINE	9050	65,917	15,427,479.78	234.04
ETORPHINE	9056	1	57	57
BUPRENORPHINE	9064	59,274	3,179,903.93	53.65
DIHYDROCODEINE	9120	4,215	27,742.25	6.58
OXYCODONE	9143	65,710	46,519,135.37	707.95
HYDROMORPHONE	9150	57,230	1,170,251.35	20.45
HYDROCODONE	9193	66,146	25,666,888.29	388.03
LEVORPHANOL	9220L	1,642	3,804.87	2.32
MEPERIDINE (PETHIDINE)	9230	19,735	451,854.54	22.9
METHADONE	9250B	50,318	2,740,641.36	54.47
MORPHINE	9300	61,951	15,050,790.76	242.95
OPIUM TINCTURE (LAUDANUM)	9630	74	290.61	3.93
OPIUM POWDERED	9639	4,044	10,634.40	2.63
OXYMORPHONE	9652	35,318	1,099,737.69	31.14
NOROXYMORPHONE	9668	7	12.41	1.77
ALFENTANIL	9737	43	11.88	0.28
REMIFENTANIL	9739	79	8.98	0.11
SUFENTANIL BASE	9740	62	125.72	2.03
TAPENTADOL	9780	41,713	5,496,988	131.78

BUSINESS ACTIVITY:A - PHARMACIES

DRUG NAME	DRUG CODE	BUYERS	TOTAL GRAMS	AVG GRAMS
FENTANYL BASE	9801	60,607	285,984	4.72
OPIUM COMBINATION PRODUCT (C-III)	9809	1,639	1,136.71	0.69

ENTIRE UNITED STATES BUSINESS ACTIVITY: B - HOSPITALS

DRUG NAME	DRUG CODE	BUYERS	TOTAL GRAMS	AVG GRAMS
AMPHETAMINE	1100	3,923	528,728.62	134.78
DL-METHAMPHETAMINE RACEMIC BASE	1105B	21	90.42	4.31
D-METHAMPHETAMINE	1105D	12	83.55	6.96
LISDEXAMFETAMINE	1205	1,877	127,215.95	67.78
METHYLPHENIDATE (DL;D;L;ISOMERS)	1724	5,376	606,458.81	112.81
GAMMA HYDROXYBUTYRIC ACID(FDA APPROVED-CSA III)	2012	12	37,597.82	3133.15
BARBITURIC ACID DERIVIATIVE OR SALT [PER 21CFR 1308.13(C)(3)]	2100	168	2,009.90	11.96
AMOBARBITAL (SCHEDULE 2)	2125	202	952.41	4.71
BUTALBITAL	2165	375	6,085	16.23
PENTOBARBITAL (SCHEDULE 2)	2270	726	222,038.76	305.84
SECOBARBITAL (SCHEDULE 2)	2315	9	384.51	42.72
DRONABINOL IN AN ORAL SOLUTION IN FDA APPROVED DRUG PRODUCT (SYNDROS - CII)	7365	13	5.25	0.4
NABILONE	7379	3	0.7	0.23
COCAINE	9041L	3,696	22,874.80	6.19
CODEINE	9050	7,298	572,528.25	78.45
BUPRENORPHINE	9064	4,596	97,615.88	21.24
DIHYDROCODEINE	9120	37	129.62	3.5
OXYCODONE	9143	8,760	2,304,923.66	263.12
HYDROMORPHONE	9150	8,669	225,982.09	26.07
HYDROCODONE	9193	9,293	1,404,591.66	151.15
LEVORPHANOL	9220L	22	15.55	0.71
MEPERIDINE (PETHIDINE)	9230	7,155	206,659.13	28.88
METHADONE	9250B	5,536	261,684.36	47.27
MORPHINE	9300	9,625	1,532,432.24	159.21
OPIUM TINCTURE (LAUDANUM)	9630	2	4.72	2.36
OPIUM POWDERED	9639	3,625	21,384	5.9
OXYMORPHONE	9652	937	9,957.38	10.63
NOROXYMORPHONE	9668	7	19.2	2.74
ALFENTANIL	9737	777	299.62	0.39

REMIFENTANIL	9739	2,332	1,608.88	0.69
SUFENTANIL BASE	9740	1,771	49.96	0.03
TAPENTADOL	9780	1,466	92,628.35	63.18
FENTANYL BASE	9801	10,114	36,371.18	3.6
OPIUM COMBINATION PRODUCT (C-III)	9809	152	98.52	0.65

ENTIRE UNITED STATES BUSINESS ACTIVITY: C - PRACTITIONERS

DRUG NAME	DRUG CODE	BUYERS	TOTAL GRAMS	AVG GRAMS
AMPHETAMINE	1100	65	1,918.90	29.52
D-METHAMPHETAMINE	1105D	6	16.39	2.73
LISDEXAMFETAMINE	1205	49	1,195.25	24.39
METHYLPHENIDATE (DL;D;L;ISOMERS)	1724	37	2,113.19	57.11
GAMMA HYDROXYBUTYRIC ACID(FDA APPROVED-CSA III)	2012	10	27,343.87	2,734.39
BARBITURIC ACID DERIVIATIVE OR SALT [PER 21CFR 1308.13(C)(3)]	2100	6	93.5	15.58
BUTALBITAL	2165	3	255	85
PENTOBARBITAL (SCHEDULE 2)	2270	7,610	6,005,685.79	789.18
COCAINE	9041L	571	2,663.26	4.66
CODEINE	9050	6,512	147,102.14	22.59
BUPRENORPHINE	9064	24,463	7,584.69	0.31
DIHYDROCODEINE	9120	1,478	2,168.04	1.47
OXYCODONE	9143	1,985	30,172.04	15.2
HYDROMORPHONE	9150	12,946	7,754.93	0.6
HYDROCODONE	9193	11,928	57,192.35	4.79
LEVORPHANOL	9220L	1	0.35	0.35
MEPERIDINE (PETHIDINE)	9230	4,768	39,600.32	8.31
METHADONE	9250B	591	2,084	3.53
MORPHINE	9300	8,490	20,123.58	2.37
THEBAINE	9333	1	25	25
OPIUM POWDERED	9639	136	419.76	3.09
OXYMORPHONE	9652	266	559.89	2.1
NOROXYMORPHONE	9668	88	720.12	8.18
ALFENTANIL	9737	207	61.49	0.3
REMIFENTANIL	9739	481	41.06	0.09
SUFENTANIL BASE	9740	149	6.32	0.04
TAPENTADOL	9780	22	907.5	41.25
FENTANYL BASE	9801	18,026	2,144.37	0.12

ENTIRE UNITED STATES — BUSINESS ACTIVITY: D - TEACHING INSTITUTIONS

DRUG NAME	DRUG CODE	BUYERS	TOTAL GRAMS	AVG GRAMS
AMPHETAMINE	1100	2	2.2	1.1
D-METHAMPHETAMINE	1105D	2	8.03	4.02
METHYLPHENIDATE (DL;D;L;ISOMERS)	1724	1	0.43	0.43
PENTOBARBITAL (SCHEDULE 2)	2270	20	48,702.68	2,435.13
COCAINE	9041L	3	3.11	1.04
CODEINE	9050	5	2,220.08	444.02
BUPRENORPHINE	9064	64	8	0.12
OXYCODONE	9143	1	0.45	0.45
HYDROMORPHONE	9150	24	50.89	2.12
HYDROCODONE	9193	8	123.82	15.48
MEPERIDINE (PETHIDINE)	9230	3	3.81	1.27
METHADONE	9250B	8	128.35	16.04
MORPHINE	9300	18	133.82	7.43
OXYMORPHONE	9652	3	0.54	0.18
REMIFENTANIL	9739	5	0.25	0.05
SUFENTANIL BASE	9740	1	0.02	0.02
FENTANYL BASE	9801	26	15.81	0.61

ENTIRE UNITED STATES — BUSINESS ACTIVITY: M - MID-LEVEL PRACTITIONERS

DRUG NAME	DRUG CODE	BUYERS	TOTAL GRAMS	AVG GRAMS
METHYLPHENIDATE (DL;D;L;ISOMERS)	1724	1	0.86	0.86
BUTALBITAL	2165	4	265	66.25
PENTOBARBITAL (SCHEDULE 2)	2270	664	827,317.76	1,245.96
CODEINE	9050	967	3,690.39	3.82
BUPRENORPHINE	9064	26	17.09	0.66
DIHYDROCODEINE	9120	429	299.66	0.7
OXYCODONE	9143	11	2,115.38	192.31
HYDROMORPHONE	9150	116	50.69	0.44
HYDROCODONE	9193	36	794.28	22.06
MEPERIDINE (PETHIDINE)	9230	30	87.69	2.92
METHADONE	9250B	7	359.73	51.39
MORPHINE	9300	936	1,107.84	1.18

OXYMORPHONE	9652	3	13.92	4.64
NOROXYMORPHONE	9668	1	2.12	2.12
REMIFENTANIL	9739	1	0.04	0.04
TAPENTADOL	9780	2	110	55
FENTANYL BASE	9801	1,393	69.43	0.05

ENTIRE UNITED STATES — BUSINESS ACTIVITY: N-U NARCOTIC TREATMENT PROGRAMS

DRUG NAME	DRUG CODE	BUYERS	TOTAL GRAMS	AVG GRAMS
PENTOBARBITAL (SCHEDULE 2)	2270	1	2.28	2.28
CODEINE	9050	1	22.1	22.1
BUPRENORPHINE	9064	683	66,055.48	96.71
HYDROMORPHONE	9150	1	1.24	1.24
MEPERIDINE (PETHIDINE)	9230	1	435.75	435.75
METHADONE	9250B	1,495	11,686,565	7,817.10
